# Supplementary material for: Proof of concept study to develop a novel connectivity-based electric-field modelling approach for individualized targeting of transcranial magnetic stimulation treatment
Source: Neuropsychopharmacology. 2021 Jul 28;47(2):588–98. doi: 10.1038/s41386-021-01110-6 (PMC8674270; doi:10.1038/s41386-021-01110-6)

Title: Proof of concept study to develop a novel connectivity-based electric-field modelling approach for individualized targeting of transcranial magnetic stimulation treatment

Running title: Novel modelling approach for individualized TMS targeting

Authors: Nicholas L Balderston[1], Joanne C Beer[2], Darsol Seok[1], Walid Makhoul[1], Zhi-De Deng[3], Tommaso Girelli[1], Marta Teferi[1], Nathan Smyk[1], Marc Jaskir[1], Desmond J Oathes[1], & Yvette I Sheline[1]

1. Center for Neuromodulation in Depression and Stress
Department of Psychiatry
University of Pennsylvania
Philadelphia, PA, USA

2. Penn Statistics in Imaging and Visualization Center
Department of Biostatistics, Epidemiology & Informatics
University of Pennsylvania
Philadelphia, PA, USA

3. Noninvasive Neuromodulation Unit
National Institute of Mental Health
Bethesda, MD, USA

Corresponding Author:
Nicholas Balderston
Center for Neuromodulation in Depression and Stress
3700 Hamilton Walk, Richards D302
Philadelphia, PA, 19104
Phone: 215-746-3058
Fax: 215-573-8556
nicholas.balderston@pennmedicine.upenn.edu

Keywords: Transcranial Magnetic Stimulation, resting state, functional connectivity, electric field modelling, depression, anxiety, anxious misery

Supplemental Methods

MRI Scans

MRI data was acquired as part of the Dimensional Connectomes of Anxious Misery project [1], one of the Connectomes Related to Human Diseases (CRHD) studies (https://www.humanconnectome.org/disease-studies). Participants were scanned on a Siemens Prisma 3T using a 64-channel head coil. Structural T1-weighted images were acquired using a magnetization-prepared rapid acquisition with gradient echo (MPRAGE) sequence with TR=2400ms, TE=2.22ms and flip angle of 8 degrees. 208 slices were acquired with a voxel resolution of 0.8mm isometric, resulting in an FOV of 256 x 240 x 167mm. T2-weighted images were acquired using a variable-flip-angle turbo-spin echo (TSE) sequence with TR=3200ms and TE=563ms, with the same voxel resolution and FOV as the T1w acquisition. Resting state fMRI data were acquired with a multi-band acceleration of 8, TR=800ms, TE=37ms and flip angle of 52 degrees. Whole-brain coverage was achieved with 72 slices and a voxel resolution of 2.0mm isometric, resulting in an FOV of 208 x 208 x 144mm. Each resting state scan was paired with another run with the opposite phase encoding direction (AP-PA), and two such pairs were acquired, resulting in 22:24 minutes of data (5:46 min x 4 runs) for each participant. Spin echo field maps were also acquired in opposite phase encoding directions in order to correct susceptibility distortions. During the resting state scan, participants were shown a white screen with a black crosshair in the center and were instructed to remain still with their eyes open and to blink normally.

MRI preprocessing

Computational head modelling for e-field calculations. We used the SimNIBS (Version 2.1) software package to generate 3D head and coil geometries using the finite element

method (FEM) [2]. Individualized head models were created using T1 and T2 structural MRIs that were segmented into scalp (mri2mesh), skull, CSF, gray matter, and white matter volumes, then converted to tetrahedral meshes using a Gmsh subroutine packaged in SimNIBS [3].

E-field calculations. E-field models were conducted for each site/orientation combination (See Below), and E-norm (i.e. magnitude) component of the electric field at each surface node was used to model the potential TMS effects [4].

fMRI preprocessing

Data were preprocessed using the Human Connectome Project Pipelines v4.0.1 (retrieved from https://github.com/Washington-University/HCPpipelines/releases/tag/v4.0.1). A full description of preprocessing steps is provided elsewhere [5]. In brief, anatomical preprocessing steps included gradient nonlinearity distortion correction, co-registration of T1w and T2w images, bias-field correction using spin-echo field maps and spatial normalization to the Montreal Neurological Institute (MNI) template. Functional image preprocessing steps included removal of spatial distortions via gradient nonlinearity corrections, correction of participant motion through volume realignment, susceptibility distortion correction using dual-phase encoded spin-echo corrections, registration of fMRI data to T1 space, subsequent transformation to MNI-space and removal of extra-parenchymal voxels.

Timeseries analyses using volumetric data were further conducted using the eXtensible Connectivity Pipeline (XCP Engine) [6]. The workflow is summarized as follows: (i) removal of the 10 initial volumes (8 seconds) to achieve signal stabilization, (ii) demeaning and removal of quadratic trends using a general linear model to account for scanner drift, (iii) intensity despiking using 3dDespike from AFNI [7], (iv) bandpass temporal filtering of time series between 0.01 Hz and 0.08Hz using a first-order Butterworth filter [8], (v) regression of nine confounding signals

(six motion parameters + global signal + mean white matter signal + mean cerebral spinal fluid signal) and as well as the temporal derivative, quadratic term and temporal derivatives of each quadratic term (resulting in 36 regressors total) [9], and (vi) spatial smoothing with SUSAN from FSL [10] using a 6mm FWHM kernel. Voxelwise timeseries were then downsampled to the 333 parcels in the Gordon atlas [11] and pairwise interparcel connectivity was calculated using Z-transformed Pearson correlations.

Image quality assessment. MRI data was acquired as part of the Dimensional Connectomes of Anxious Misery project. More detailed image quality reports can be obtained in the Seok et al., (2020) [1]. In brief, whole brain tSNR for the resting state data averaged 32.17 ± 5.67 for the AM group and 31.58 ± 4.94 for the HC group. Finally, motion (RMS across TRs) averaged 0.16 ± 0.08 mm for the AM group and 0.14 ± 0.06 for the HC group. Importantly, SNR, tSNR and motion was similar across groups and comparable to what was observed in the human connectome project, which used similar scans.

Appendix: Equations

Connectivity based model: PCA regression

Let $\mathbf{Y}^{mood}$ be the $n \times 1$ vector of behavioral symptom scores (e.g., MADRS scores) for n participants. We assume the behavioral symptom scores can be predicted using a linear combination of connectivity measures, according to the model

$$\boldsymbol{Y}^{mood} = \boldsymbol{X\beta} + \boldsymbol{\epsilon} \quad (Eq. A1)$$

where $\boldsymbol{X}$ is a $n \times p$ matrix such that each row consists of one participant's concatenated unique connectivity values from the Z-transformed Pearson correlation functional connectivity matrix, i.e., the upper or lower triangular portion of the $N \times N$ connectivity matrix ($p = N(N-1)/2$), for the Gordon atlas, $N = 333$); $\boldsymbol{\beta}$ is a $p \times 1$ vector of coefficients, and $\boldsymbol{\epsilon}$ is a $n \times 1$ vector of error terms. We assume that $\boldsymbol{Y}^{mood}$ and columns of $\boldsymbol{X}$ have been centered so as to have zero empirical means.

We perform principal components analysis (PCA) on the predictor matrix $\boldsymbol{X}$. Let $\boldsymbol{X} = \boldsymbol{U\Delta V^T}$ denote the singular value decomposition of $\boldsymbol{X}$, where $\boldsymbol{\Delta}_{p\times p} = \text{diag}(\delta_1, \dots, \delta_p)$ is a diagonal matrix of non-negative singular values, and columns of $\boldsymbol{U}_{n\times p}$ and $\boldsymbol{V}_{p\times p}$ are orthonormal sets of vectors, i.e., the left and right singular vectors of $\boldsymbol{X}$. The spectral decomposition of $\boldsymbol{X}$ is given by $\boldsymbol{V\Lambda V^T}$, where $\boldsymbol{\Lambda}_{p\times p} = \text{diag}(\lambda_1, \dots, \lambda_p) = \text{diag}(\delta_1^2, \dots, \delta_p^2) = \boldsymbol{\Delta}^2$ is a diagonal matrix of the non-negative eigenvalues of $\boldsymbol{X^T X}$ and columns of $\boldsymbol{V}_{p\times p} = [\boldsymbol{v_1}, \dots, \boldsymbol{v_p}]$ are the corresponding eigenvectors. The jth principal component and jth principal component direction (i.e., PCA loading) corresponding to the jth largest eigenvector are $\boldsymbol{Xv_j}$ and $\boldsymbol{v_j}$, respectively, for each $j \in \{1, \dots, p\}$.

For PCA regression (PCR), we use the first $k \leq n$ components as predictors. Let $\boldsymbol{V}_k$ represent the $p \times k$ matrix comprising the first k columns of $\boldsymbol{V}$, and $\boldsymbol{W}_k = \boldsymbol{XV}_k$ be the $n \times k$ matrix with the first k principal components as columns. We estimate regression coefficients $\boldsymbol{\gamma}_k$ in $\boldsymbol{Y}^{mood} = \boldsymbol{W}_k\boldsymbol{\gamma}_k + \boldsymbol{\epsilon}_k \quad (Eq. A2)$ by ordinary least squares, i.e., $\boldsymbol{\gamma}_k = (\boldsymbol{W}_k^T\boldsymbol{W}_k)^{-1}\boldsymbol{W}_k^T\boldsymbol{Y}^{mood} \in \mathbb{R}^k$. Then the PCR estimator of $\boldsymbol{\beta}$ based on the first k principal components is given by $\widehat{\boldsymbol{\beta}}_k = \boldsymbol{V}_k\widehat{\boldsymbol{\gamma}}_k \in \mathbb{R}^p \quad (Eq. A3)$.

E-field augmented model

Once $\widehat{\boldsymbol{\beta}}_k$ is obtained from the connectivity based model, the e-field augmented model will be used to identify the optimal coil orientation and stimulation sites to generate the greatest reduction in behavioral symptom score for each participant. Let $\Delta\boldsymbol{Y}^{mood}$ be the $n \times 1$ vector of changes in score between the pre- and post-treatment assessments for n participants. We model $\Delta\boldsymbol{Y}^{mood}$ as follows:

$$\Delta\boldsymbol{Y}^{mood} = \boldsymbol{Y}^{mood}_{post} - \boldsymbol{Y}^{mood}_{pre} = \left(\boldsymbol{X}_{post} - \boldsymbol{X}_{pre}\right)\boldsymbol{\beta} + \boldsymbol{\epsilon}' \ (Eq. A4),$$

where $\boldsymbol{\epsilon}' = \boldsymbol{\epsilon}_{post} - \boldsymbol{\epsilon}_{pre}$.

For each site and orientation l, let $\boldsymbol{E}_l$ be a $n \times p$ matrix, where rows correspond to participants, columns correspond to pairs of regions in the Gordon atlas, and entries equal the average e-field model values at each pair of regions for the given participant, site, and orientation. We assume that $\boldsymbol{X}^l_{post} = C \cdot \boldsymbol{E}_l \circ \boldsymbol{X}_{pre}$, where C is a positive proportionality constant, and $\circ$ denotes the Hadamard product. Thus, for a given stimulation site and coil orientation combination l,

$$\Delta\boldsymbol{Y}^{mood} = \left(C \cdot \boldsymbol{E}_l \circ \boldsymbol{X}_{pre} - \boldsymbol{X}_{pre}\right)\boldsymbol{\beta} + \boldsymbol{\epsilon}' \ \ (Eq. A5).$$

With these assumptions, and substituting the $\widehat{\boldsymbol{\beta}}_k$ estimated form the PCR described above, it is possible to compare relative differences in the predicted change in behavioral symptom score across sites and orientations l.

Supplemental References

1. Seok, D. *et al.* Dimensional Connectomics of Anxious Misery, a Human Connectome Study Related to Human Disease: Overview of Protocol and Data Quality. *NeuroImage Clin.* **28**, 102489 (2020).
2. Thielscher, A., Antunes, A. & Saturnino, G. B. Field modeling for transcranial magnetic stimulation: A useful tool to understand the physiological effects of TMS? *Proc. Annu. Int. Conf. IEEE Eng. Med. Biol. Soc. EMBS* 222–225 (2015). doi:10.1109/EMBC.2015.7318340
3. Thielscher, A., Opitz, A. & Windhoff, M. Impact of the gyral geometry on the electric field induced by transcranial magnetic stimulation. *Neuroimage* **54**, 234–243 (2011).
4. Balderston, N. L. *et al.* A generalized workflow for conducting electric field–optimized, fMRI-guided, transcranial magnetic stimulation. *Nat. Protoc.* **15**, 3595–3614 (2020).
5. Glasser, M. F. *et al.* The minimal preprocessing pipelines for the Human Connectome Project. *Neuroimage* (2013). doi:10.1016/j.neuroimage.2013.04.127
6. Ciric, R. *et al.* Mitigating head motion artifact in functional connectivity MRI. *Nat. Protoc.* (2018). doi:10.1038/s41596-018-0065-y
7. Cox, R. W. AFNI: software for analysis and visualization of functional magnetic resonance neuroimages. *Comput. Biomed. Res.* **29**, 162–173 (1996).
8. Biswal, B. B. *et al.* Toward discovery science of human brain function. *Proc. Natl. Acad. Sci. U. S. A.* (2010). doi:10.1073/pnas.0911855107
9. Satterthwaite, T. D. *et al.* An improved framework for confound regression and filtering for control of motion artifact in the preprocessing of resting-state functional connectivity data. *Neuroimage* (2013). doi:10.1016/j.neuroimage.2012.08.052
10. Smith, S. M. & Brady, J. M. SUSAN - A new approach to low level image processing. *Int. J. Comput. Vis.* (1997). doi:10.1023/A:1007963824710
11. Gordon, E. M. *et al.* Generation and Evaluation of a Cortical Area Parcellation from Resting-State Correlations. *Cereb. Cortex* **26**, 288–303 (2016).
12. Fox, M. D., Liu, H. & Pascual-Leone, A. Identification of reproducible individualized targets for treatment of depression with TMS based on intrinsic connectivity. *Neuroimage* **66**, 151–160 (2013).
13. Trapp, N. T. *et al.* Reliability of targeting methods in TMS for depression: Beam F3 vs. 5.5 cm. *Brain Stimul.* **13**, 578–581 (2020).

Supplemental Table 1: Sites used to define the P --> A dlPFC axis

Site	x		y	z	Location Details
	Left	Right			
1	-43.5	43.5	4.5	59.5	Most posterior
2	-42.7	42.7	8.3	57.7	
3	-41.8	41.8	12.2	55.8	
4	-41.0	41.0	16.0	54.0	5 cm therapeutic site
5	-40.2	40.2	19.8	52.2	
6	-39.3	39.3	23.7	50.3	
7	-38.5	38.5	27.5	48.5	
8	-37.7	37.7	31.3	46.7	
9	-36.8	36.8	35.2	44.8	
10	-36.0	36.0	39.0	43.0	BA9
11	-38.0	38.0	39.3	39.5	
12	-40.0	40.0	39.5	36.0	Beam-F3
13	-42.0	42.0	39.8	32.5	
14	-44.0	44.0	40.0	29.0	BA46
15	-46.0	46.0	40.3	25.5	
16	-48.0	48.0	40.5	22.0	
17	-50.0	50.0	40.8	18.5	Most Anterior

Note: Coordinates in MNI space.

Supplemental Table 2: Sites used to define the M --> L Hand Knob axis

Site	x		y	z	Location Details
	Left	Right			
1	-20.0	20.0	-30.0	58.0	Upper gyrus
2	-25.0	25.0	-28.0	58.0	
3	-30.0	30.0	-26.0	58.0	
4	-36.0	36.0	-24.0	58.0	Hand Knob
5	-40.0	40.0	-20.0	58.0	
6	-44.0	44.0	-16.0	58.0	
7	-48.0	48.0	-12.0	58.0	Lower Gyrus

Note: Coordinates in MNI space.

Supplemental Table 3: Demographic information

	AM	Control
	Demographic Information	
Age	28.29 (8.2)	28.92 (6.91)
Total N	94	25
Females	64	16
	Clinical measures	
HAMD	13.3 (5.67)	1.28 (1.93)
MADRS	21.08 (8.87)	0.68 (1.18)
SHAPS	41.24 (7.61)	51.28 (5.37)
MASQ	60.35 (9.07)	48.76 (12.21)
CTQ	62.53 (8.79)	61.04 (5.77)
ISI	11.86 (6.12)	3.28 (3.27)
RTS	95.09 (21.51)	48.2 (21.07)
ASI	28.13 (14.71)	6.72 (5.91)

Note: Values represent Mean (SD).

Supplemental Table 4. Pearson correlations between image quality and model predictions.

Site	AM		HC	
	Motion	tSNR	Motion	tSNR
		MADRS		
5cm	-0.096	-0.06	-0.376	-0.065
e-field	-0.085	-0.069	-0.362	-0.065
sgACC	-0.095	-0.069	-0.365	-0.072
BA9	-0.096	-0.065	-0.36	-0.079
Beam/F3	-0.094	-0.066	-0.361	-0.076
BA46	-0.093	-0.067	-0.365	-0.067
		HAMD		
5cm	-0.097	-0.073	-0.387	-0.061
e-field	-0.096	-0.072	-0.366	-0.072
sgACC	-0.094	-0.069	-0.381	-0.067
BA9	-0.093	-0.073	-0.385	-0.059
Beam/F3	-0.093	-0.073	-0.377	-0.068
BA46	-0.092	-0.075	-0.372	-0.071

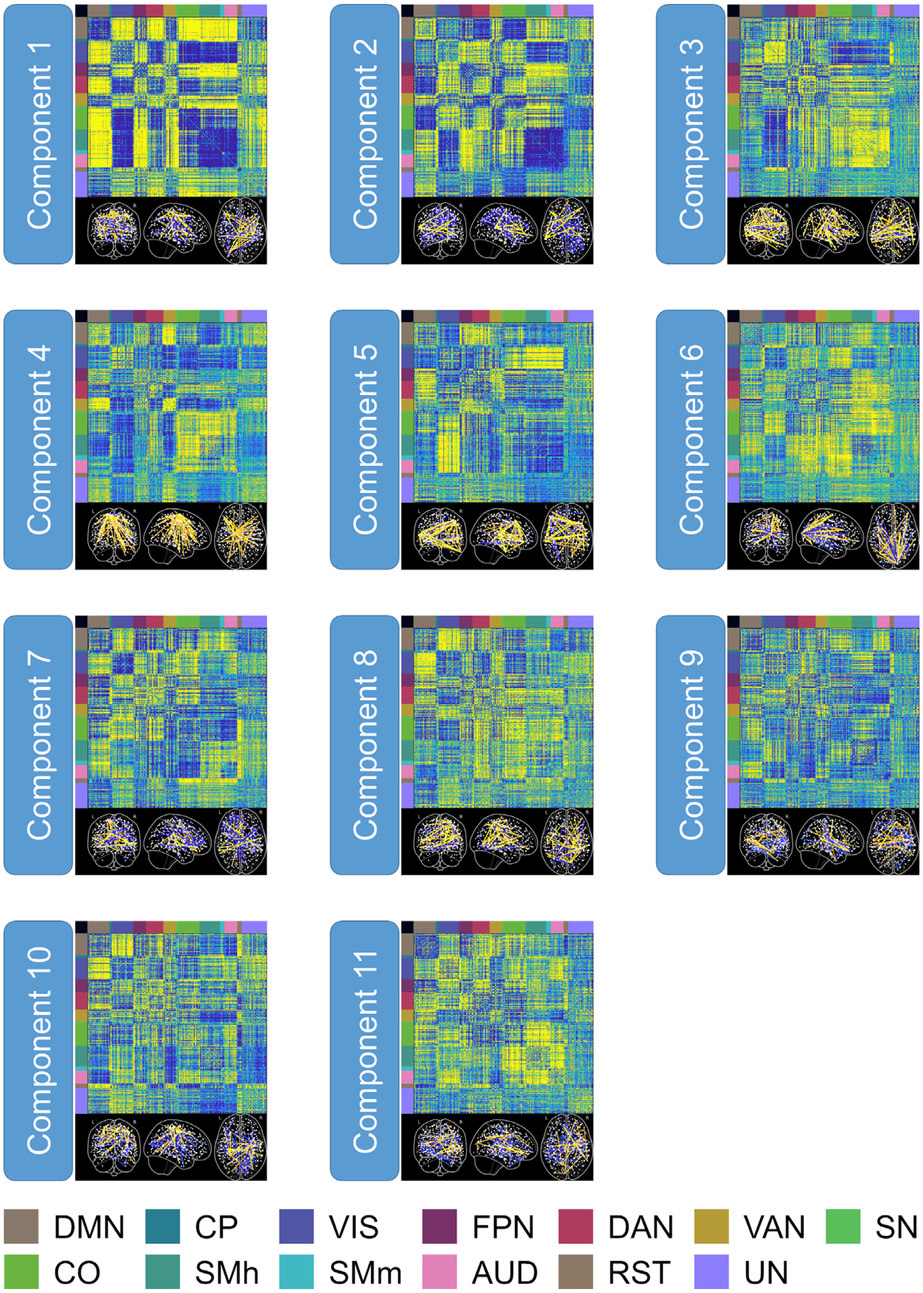

Supplemental Figure 1. Item coefficients for the components selected from the Anxious Misery Principle Components Analysis. Matrices represent the loading of each pairwise connection onto the principle components, sorted by network according to the key at the bottom of the figure. Connectome graphs for each of the principle components show the top 0.1% of connections according to the item weightings. **Network Color key**: **DMN** = Default Mode Network; **CP** = CinguloParietal; **VIS** = Visual; **FPN** = FrontoParietal Network; **DAN** = Dorsal Attention Network; **VAN** = Ventral Attention Network; **SN** = Salience Network; **CO** = CinguloOpercular; **SMh** = SomatoMotor (hand); **SMm** = SomatoMotor (mouth); **AUD** = Auditory; **RST** = RetrosplenialTemporal; **UN** = Unassigned nodes. See full-size images below.

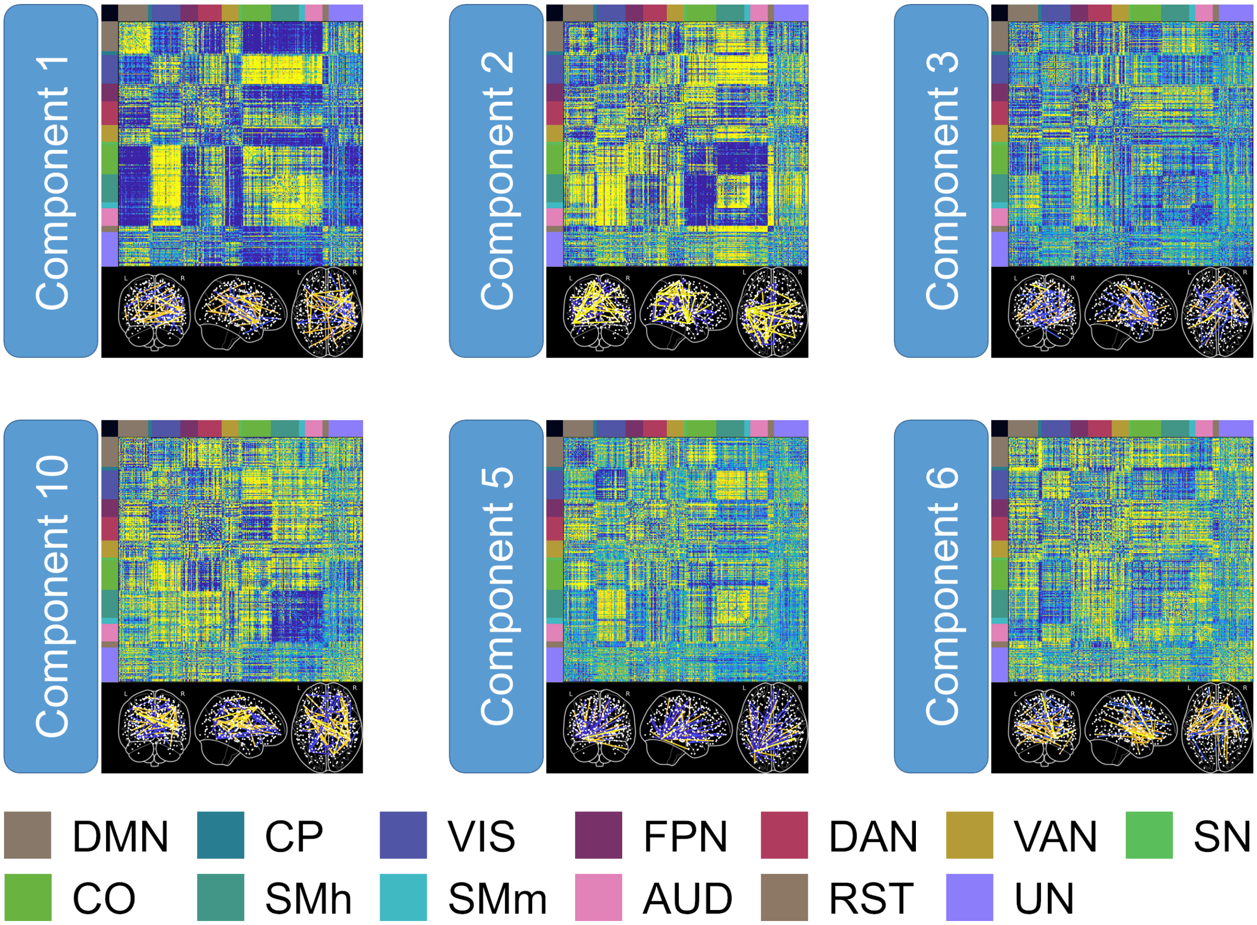

Supplemental Figure 2. Item coefficients for the components selected from the Healthy Control Principle Components Analysis. Matrices represent the loading of each pairwise connection onto the principle components, sorted by network according to the key at the bottom of the figure. Connectome graphs for each of the principle components show the top 0.1% of connections according to the item weightings. **Network Color key**: **DMN** = Default Mode Network; **CP** = CinguloParietal; **VIS** = Visual; **FPN** = FrontoParietal Network; **DAN** = Dorsal Attention Network; **VAN** = Ventral Attention Network; **SN** = Salience Network; **CO** = CinguloOpercular; **SMh** = SomatoMotor (hand); **SMm** = SomatoMotor (mouth); **AUD** = Auditory; **RST** = RetrosplenialTemporal; **UN** = Unassigned nodes. See full-size images below.

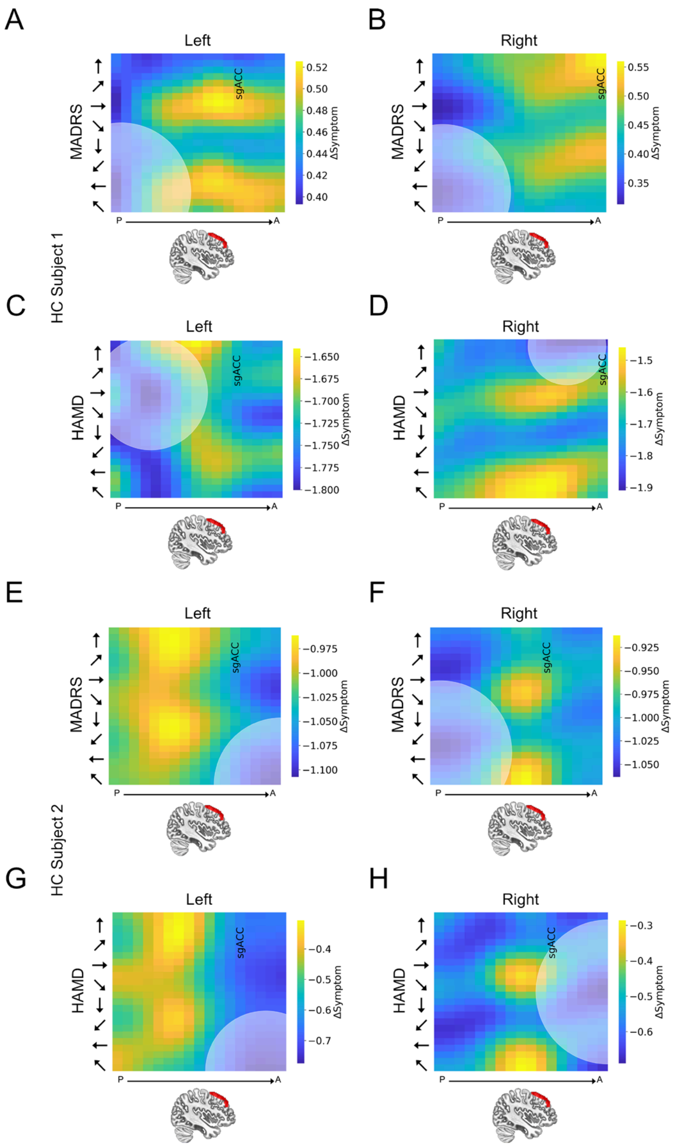

Supplemental Figure 3. Individual subject heatmaps plotting dlPFC predictions for the healthy control group. (**A, C, E, G**) Heatmaps representing the predicted MADRS and HAMD scores following a hypothetical course of TMS treatment to the left dlPFC. (**B, D, F, H**) Heatmaps representing the predicted MADRS/HAMD scores following a hypothetical course of TMS treatment to the right dlPFC. Colors represent the predicted change in MADRS/HAMD scores. Y axis represents coil orientation. X axis represents location along the Z-axis of the middle frontal gyrus. The center point of the shaded circle on the heatmaps represents the site and orientation of stimulation predicted to have the maximal reduction in symptoms for each subject. The area of the shaded circle represents the variability (i.e. Euclidean distance [95% confidence interval]) in this optimal site assessed using bootstrapping. **sGACC** = site along vector with maximal anti-correlation with the subgenual anterior cingulate cortex.

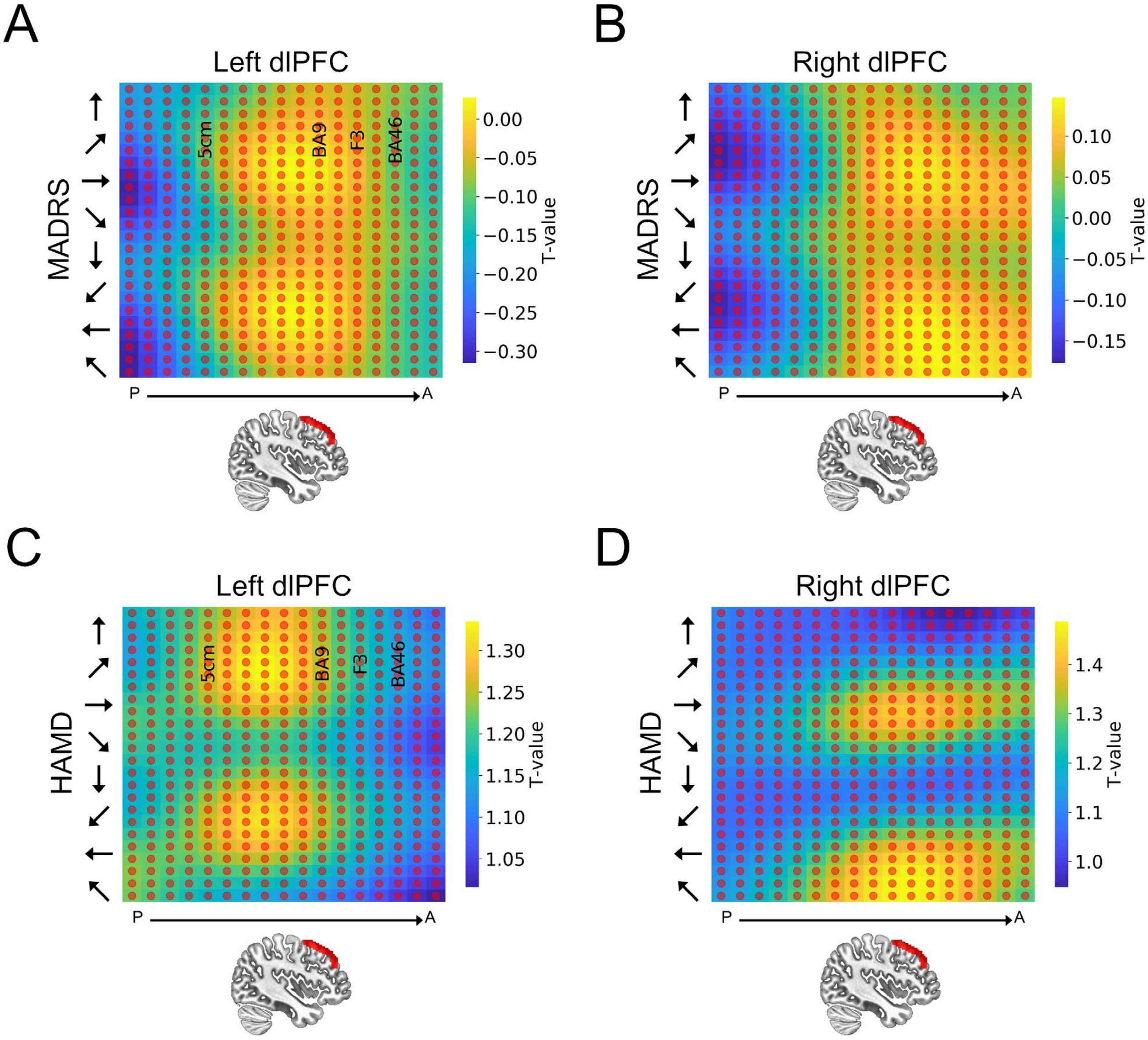

Supplemental Figure 4. Group-level heatmaps plotting dlPFC predictions for the healthy control group. A) Heatmap representing the predicted MADRS scores following a hypothetical course of TMS treatment to the left dlPFC. **B**) Heatmap representing the predicted MADRS scores following a hypothetical course of TMS treatment to the right dlPFC. **C**) Heatmap representing the predicted HAMD scores following a hypothetical course of TMS treatment to the left dlPFC. **D**) Heatmap representing the predicted HAMD scores following a hypothetical course of TMS treatment to the right dlPFC. Y axis represents coil orientation. X axis represents location along the Z-axis of the middle frontal gyrus. Red circles represent sites where the change in MADRS/HAMD scores was not statistically different from 0. **5cm** = Site in vector corresponding to the 5 cm rule target commonly used in therapeutic applications for depression [12]. **BA9** = Site in vector corresponding to Broadmann area 9 [12]. **F3** = Site in vector closest to the BEAM/F3 target commonly used in therapeutic applications for depression [13]. **BA46** = Site in vector corresponding to Broadmann area 46 [12].

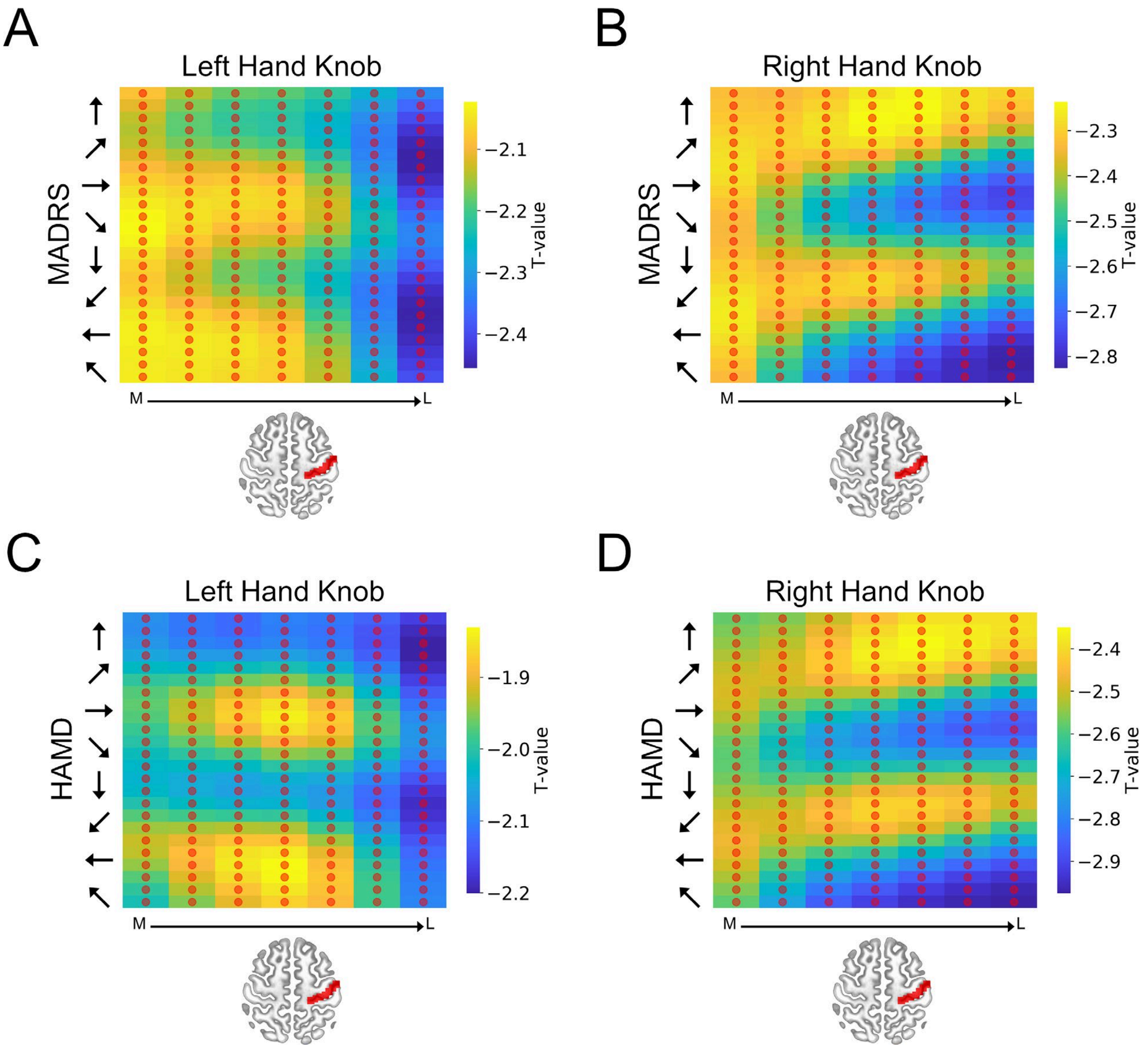

Supplemental Figure 5. Group-level heatmaps plotting hand knob predictions for the anxious misery group. A) Heatmap representing the predicted MADRS scores following a hypothetical course of TMS treatment to the left hand knob. **B**) Heatmap representing the predicted MADRS scores following a hypothetical course of TMS treatment to the right hand knob. **C**) Heatmap representing the predicted HAMD scores following a hypothetical course of TMS treatment to the left hand knob. **D**) Heatmap representing the predicted HAMD scores following a hypothetical course of TMS treatment to the right hand knob. Y axis represents coil orientation. X axis represents location along the medial to lateral axis of the primary motor cortex. Red circles represent sites where the change in MADRS/HAMD scores was not statistically different from 0.

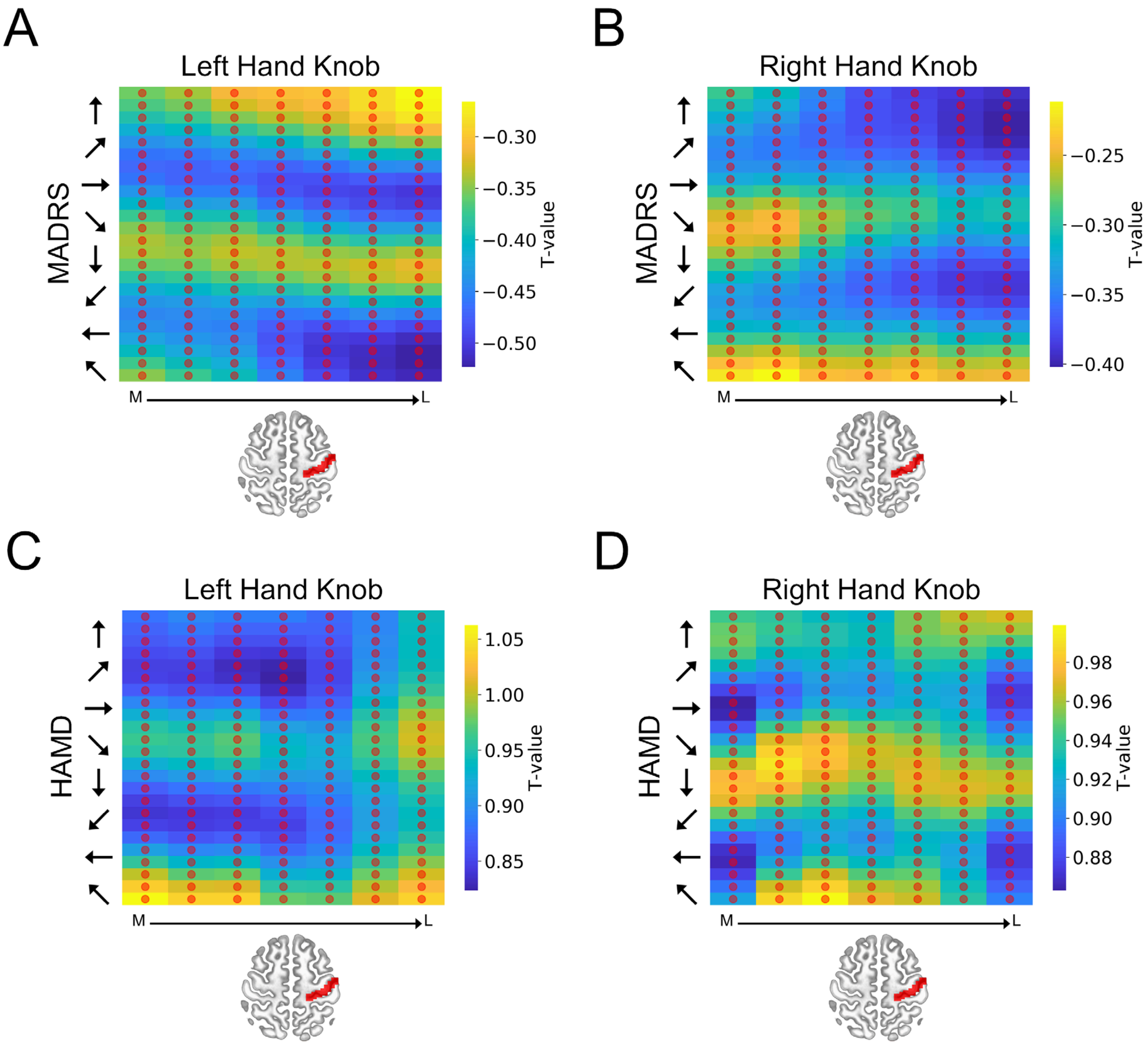

Supplemental Figure 6. Group-level heatmaps plotting hand knob predictions for the healthy control group. A) Heatmap representing the predicted MADRS scores following a hypothetical course of TMS treatment to the left hand knob. **B**) Heatmap representing the predicted MADRS scores following a hypothetical course of TMS treatment to the right hand knob. **C**) Heatmap representing the predicted HAMD scores following a hypothetical course of TMS treatment to the left hand knob. **D**) Heatmap representing the predicted HAMD scores following a hypothetical course of TMS treatment to the right hand knob. Y axis represents coil orientation. X axis represents location along the medial to lateral axis of the primary motor cortex. Red circles represent sites where the change in MADRS/HAMD scores was not statistically different from 0.

Anxious misery group component maps

component_0

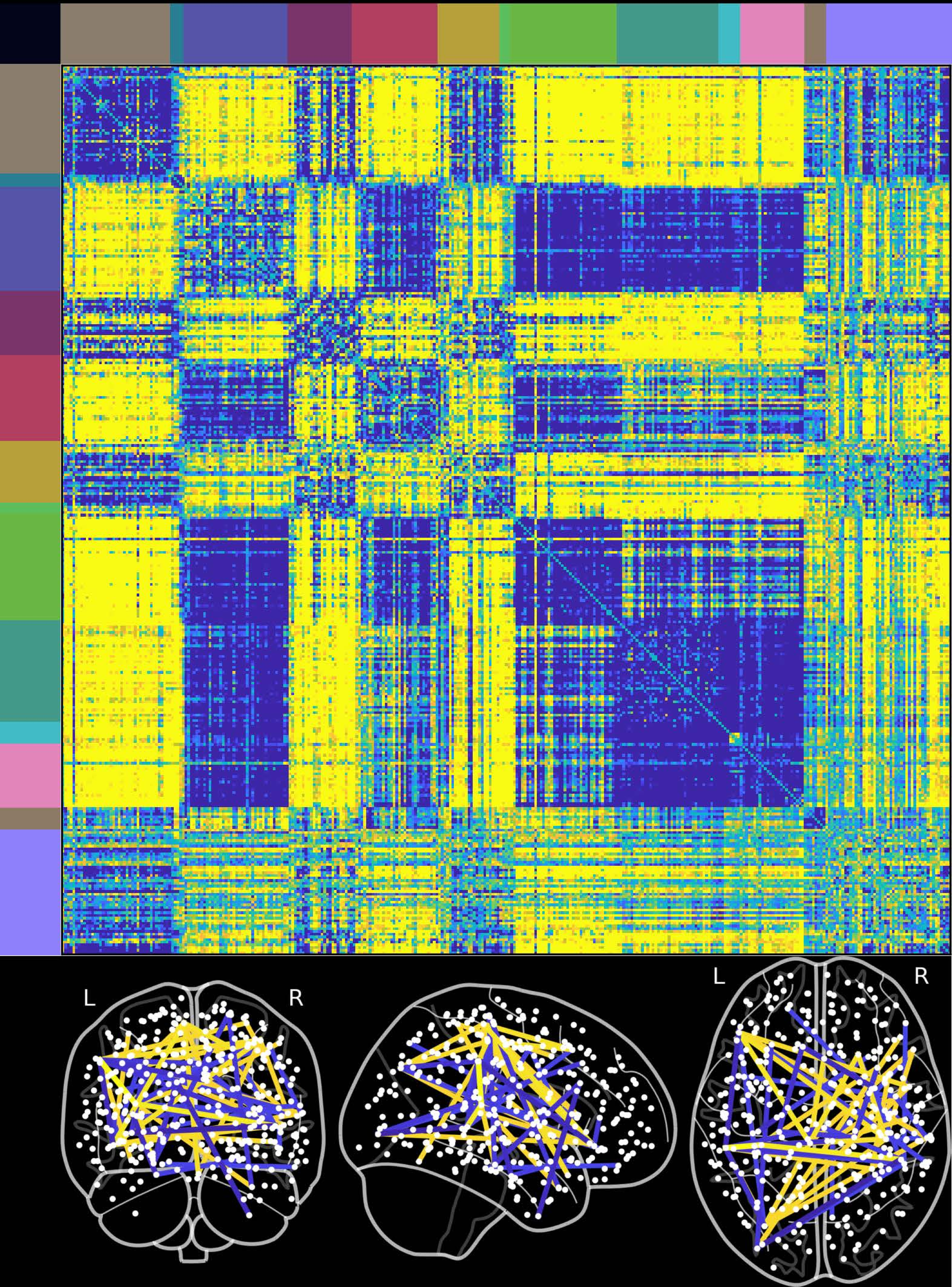

component_1

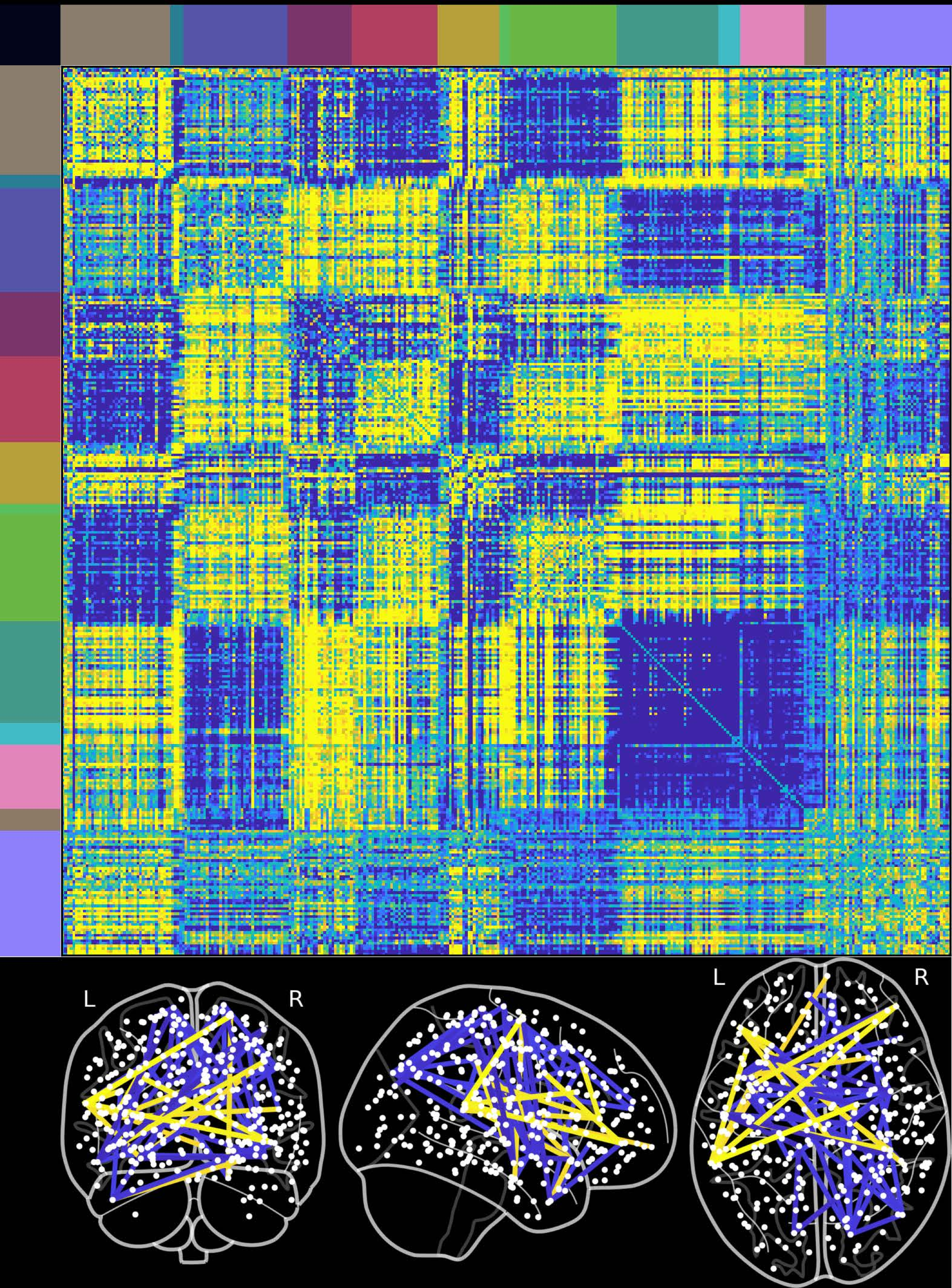

component_2

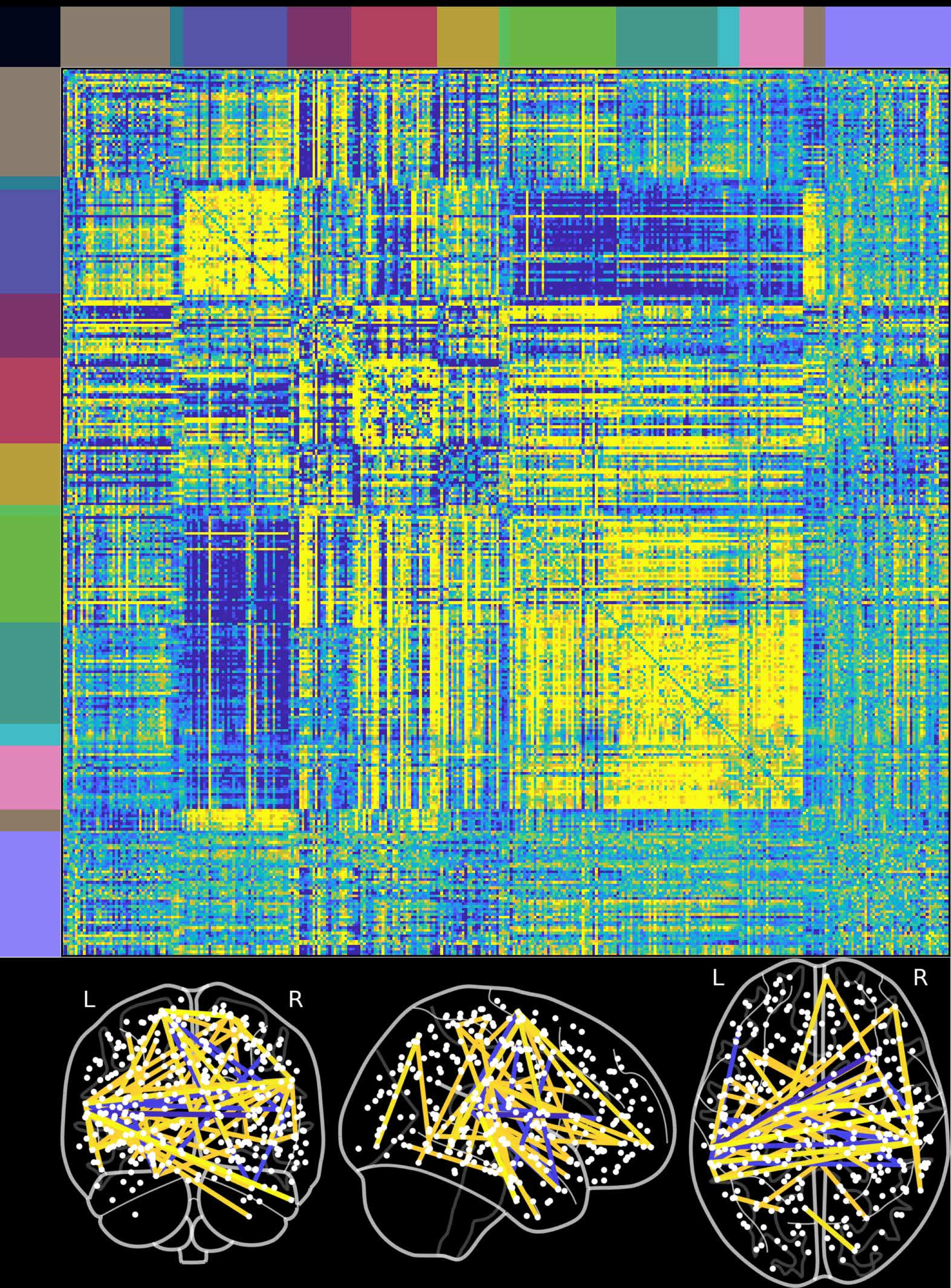

component_3

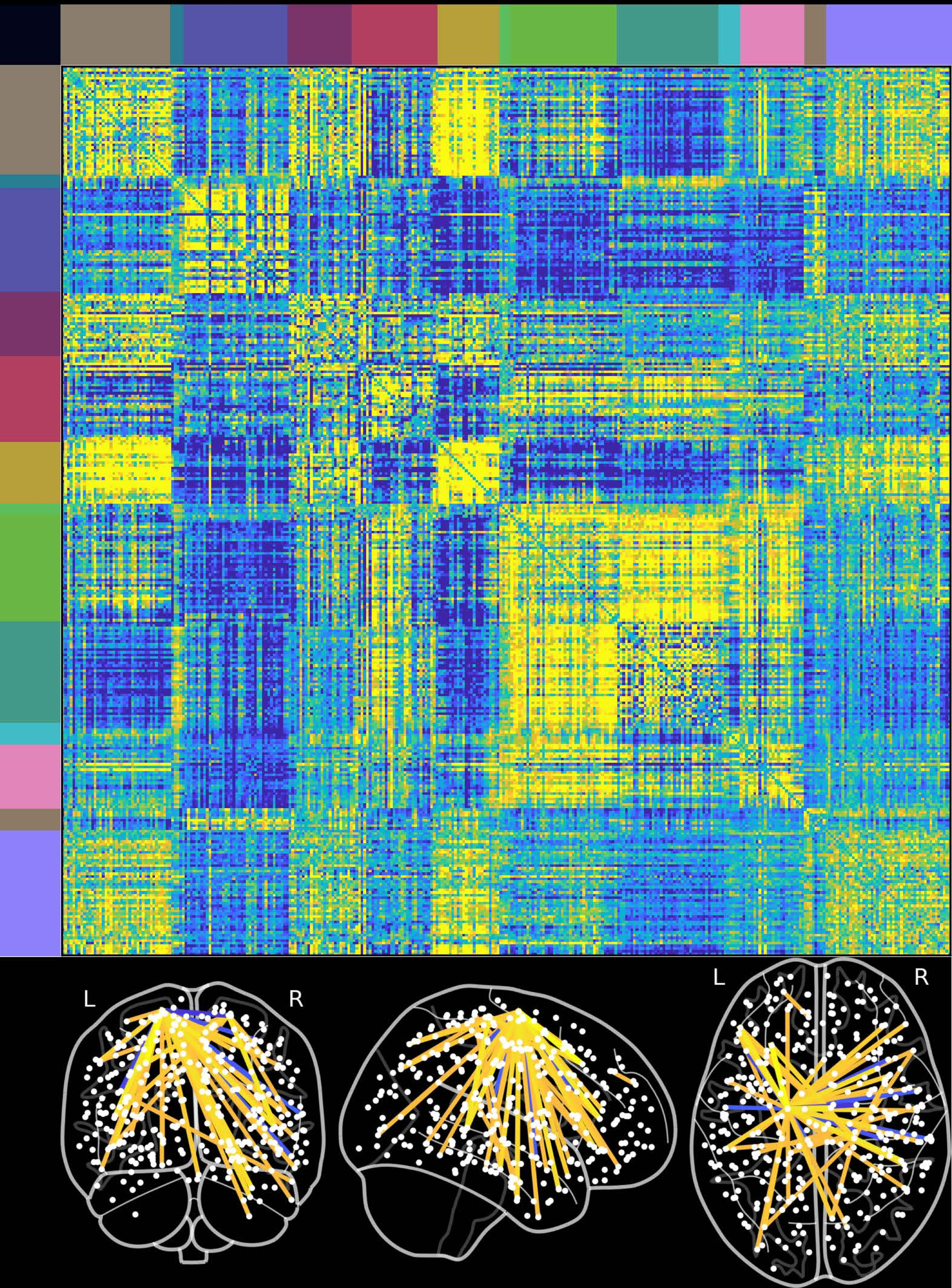

component_4

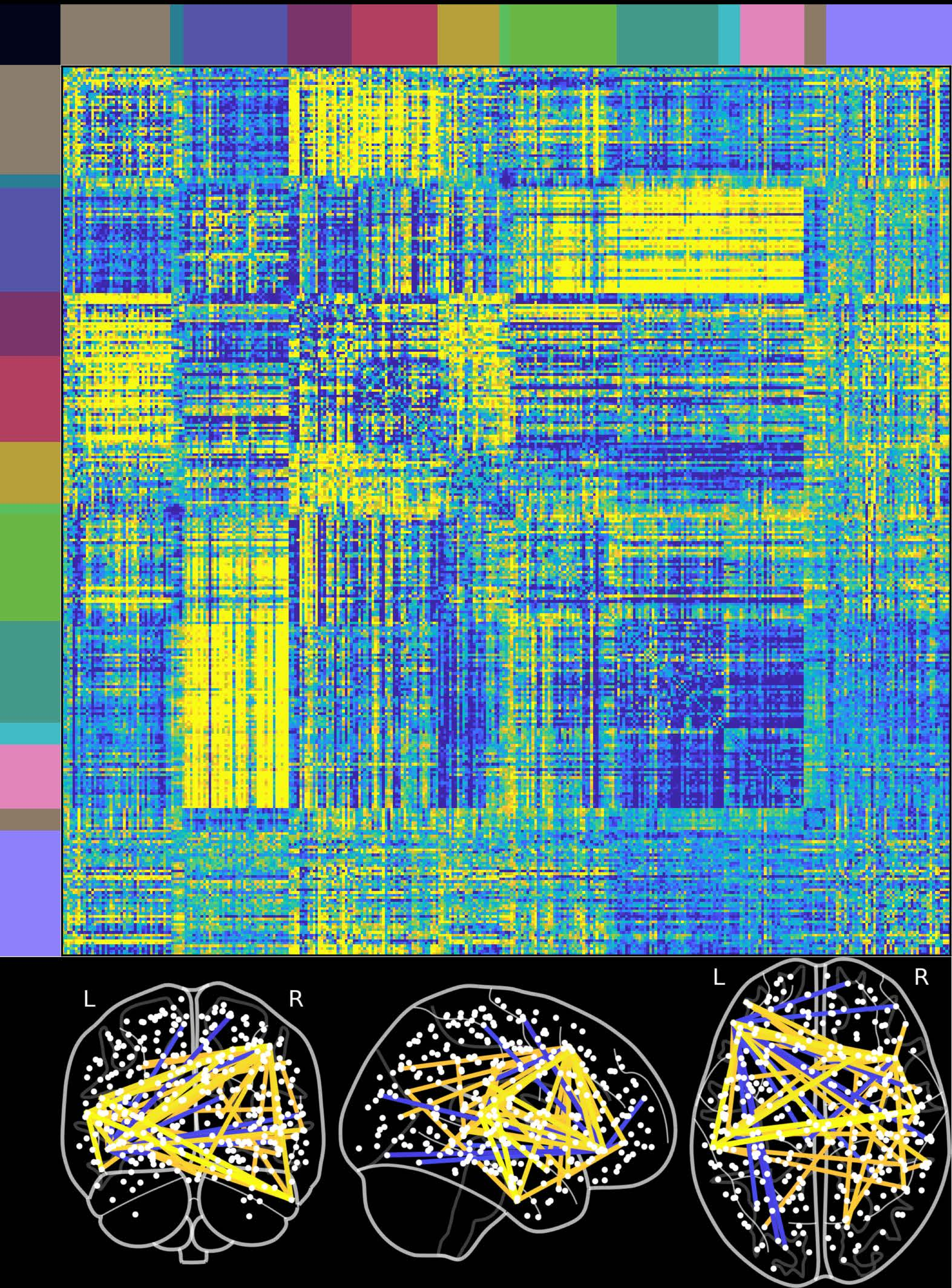

component_5

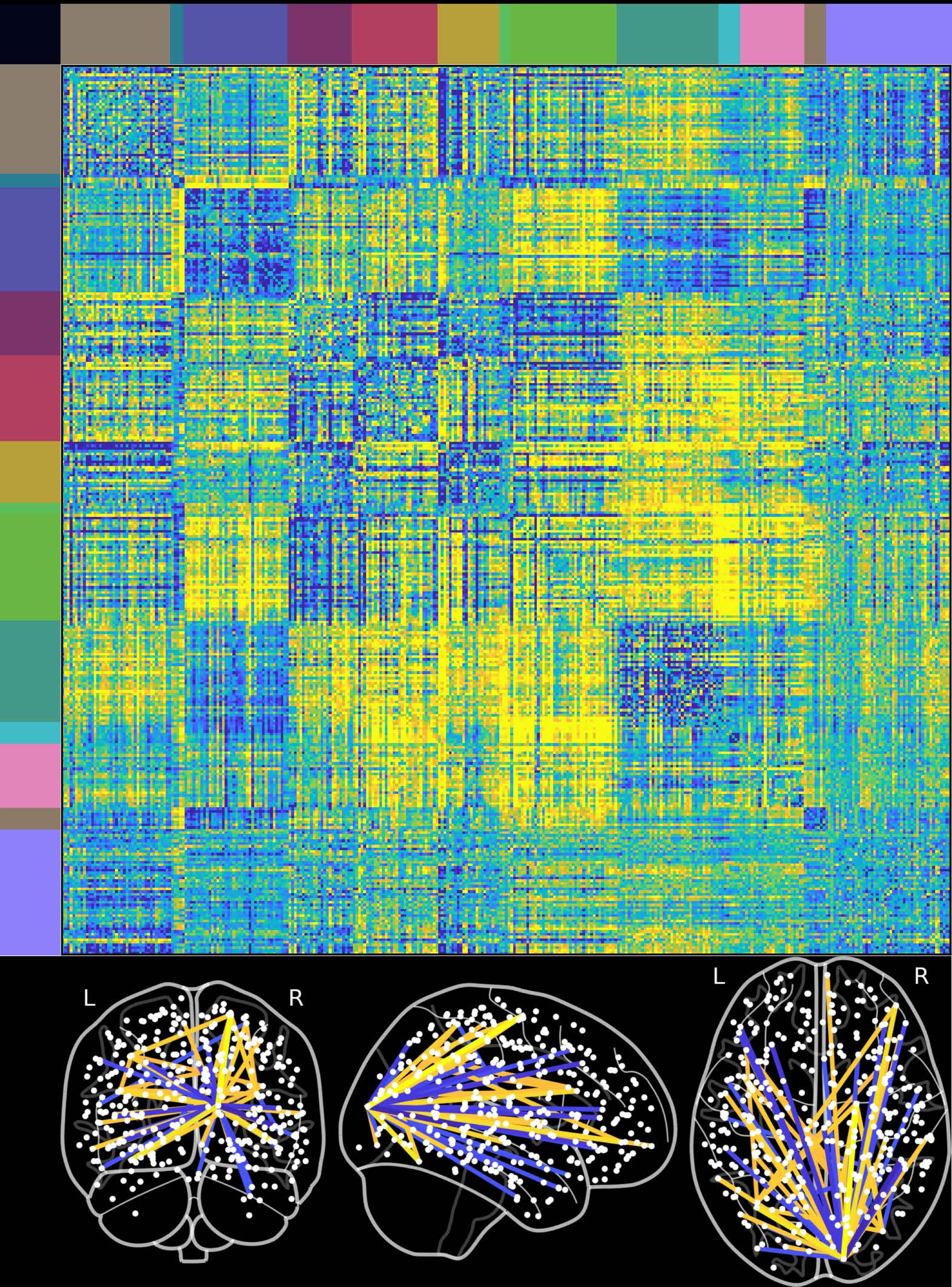

component_6

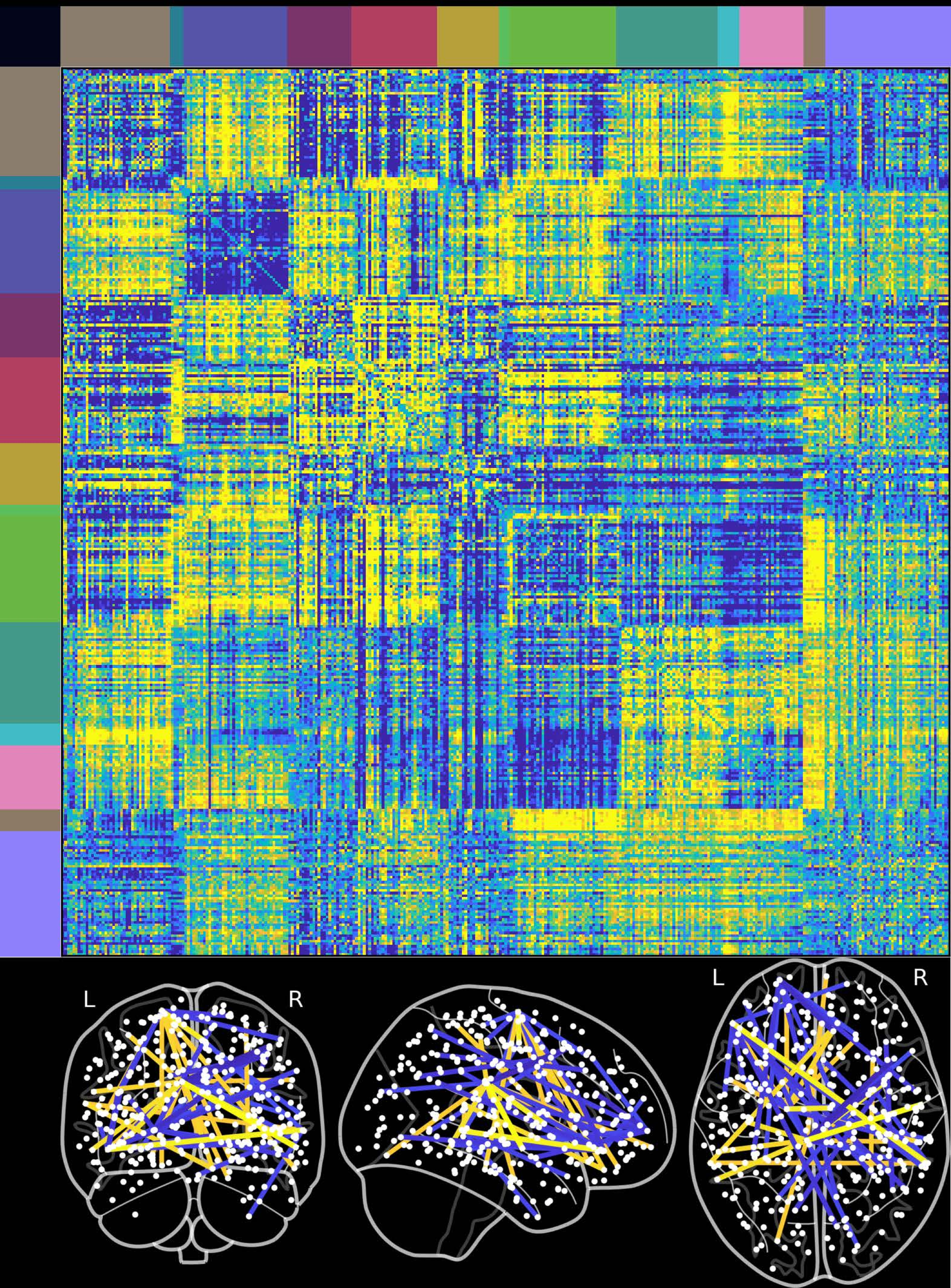

component_7

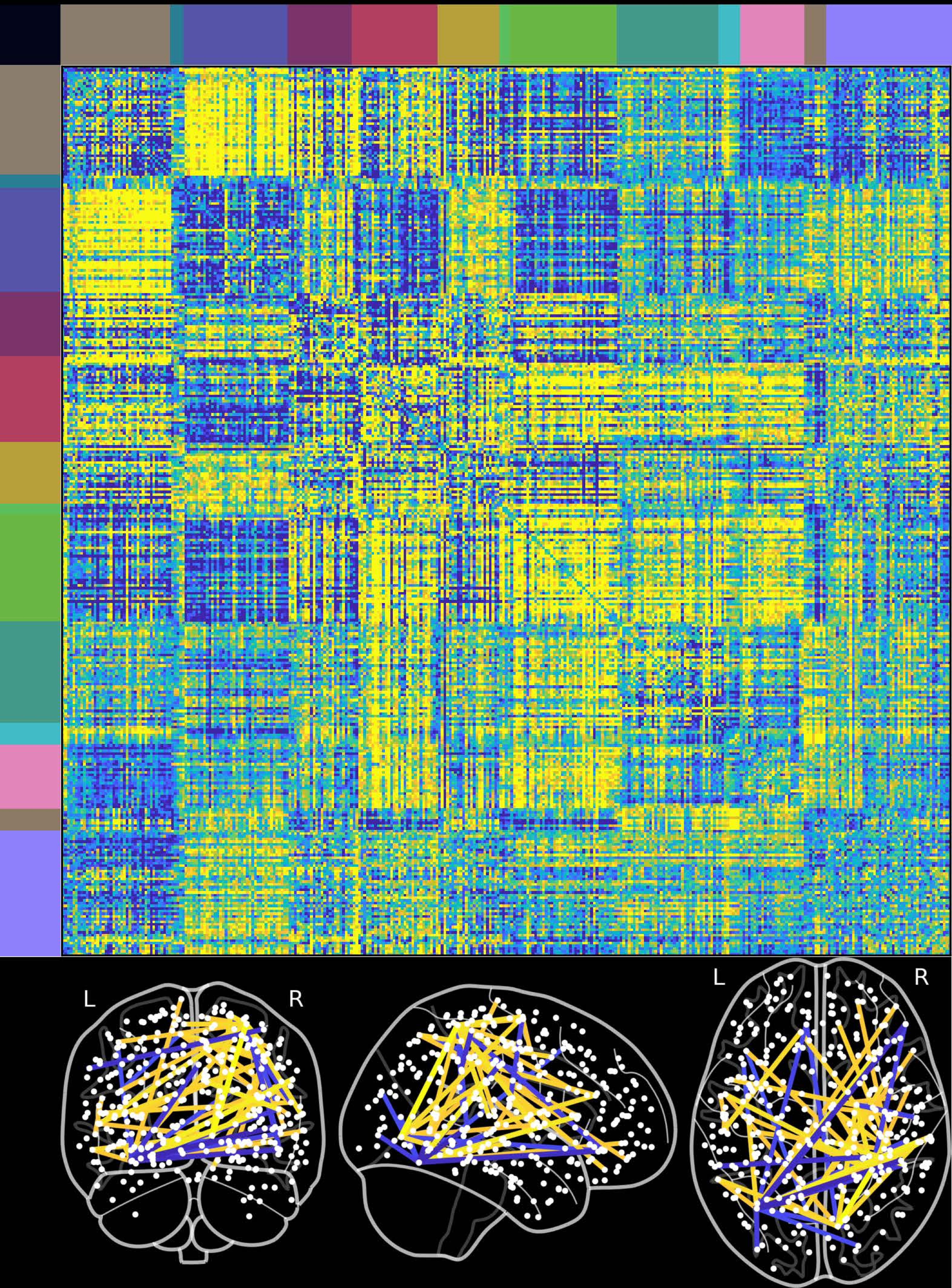

component_8

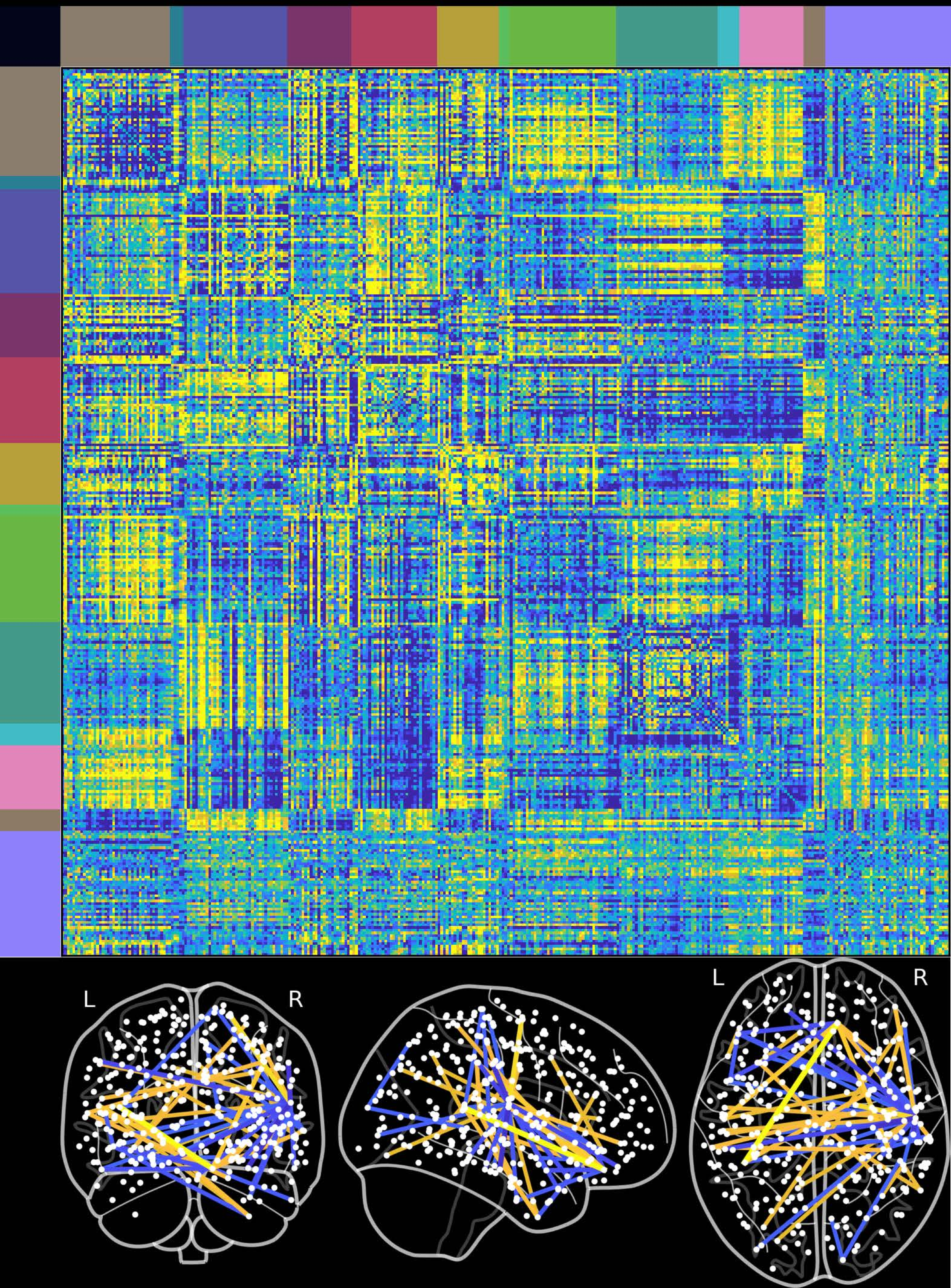

component_9

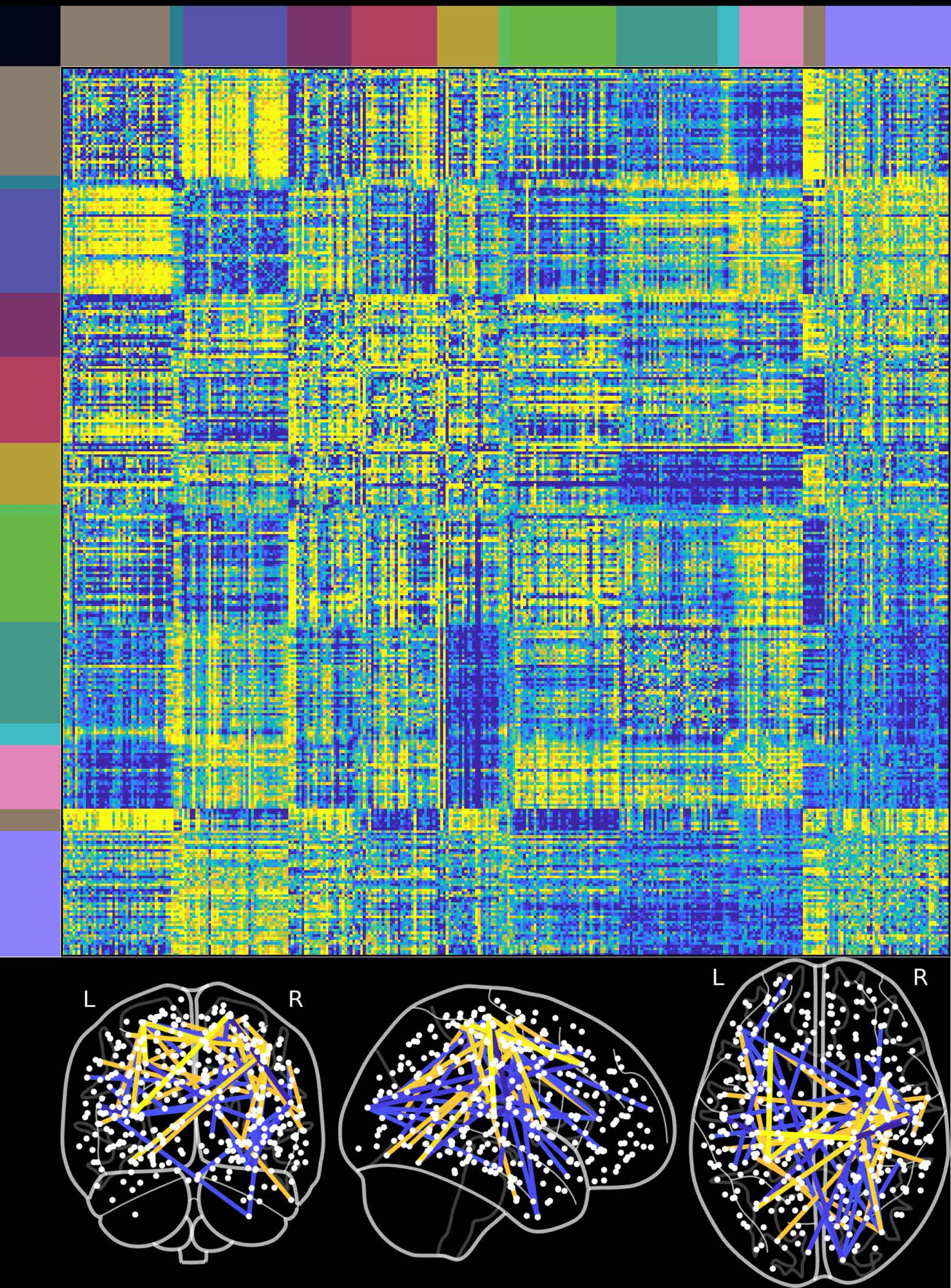

component_10

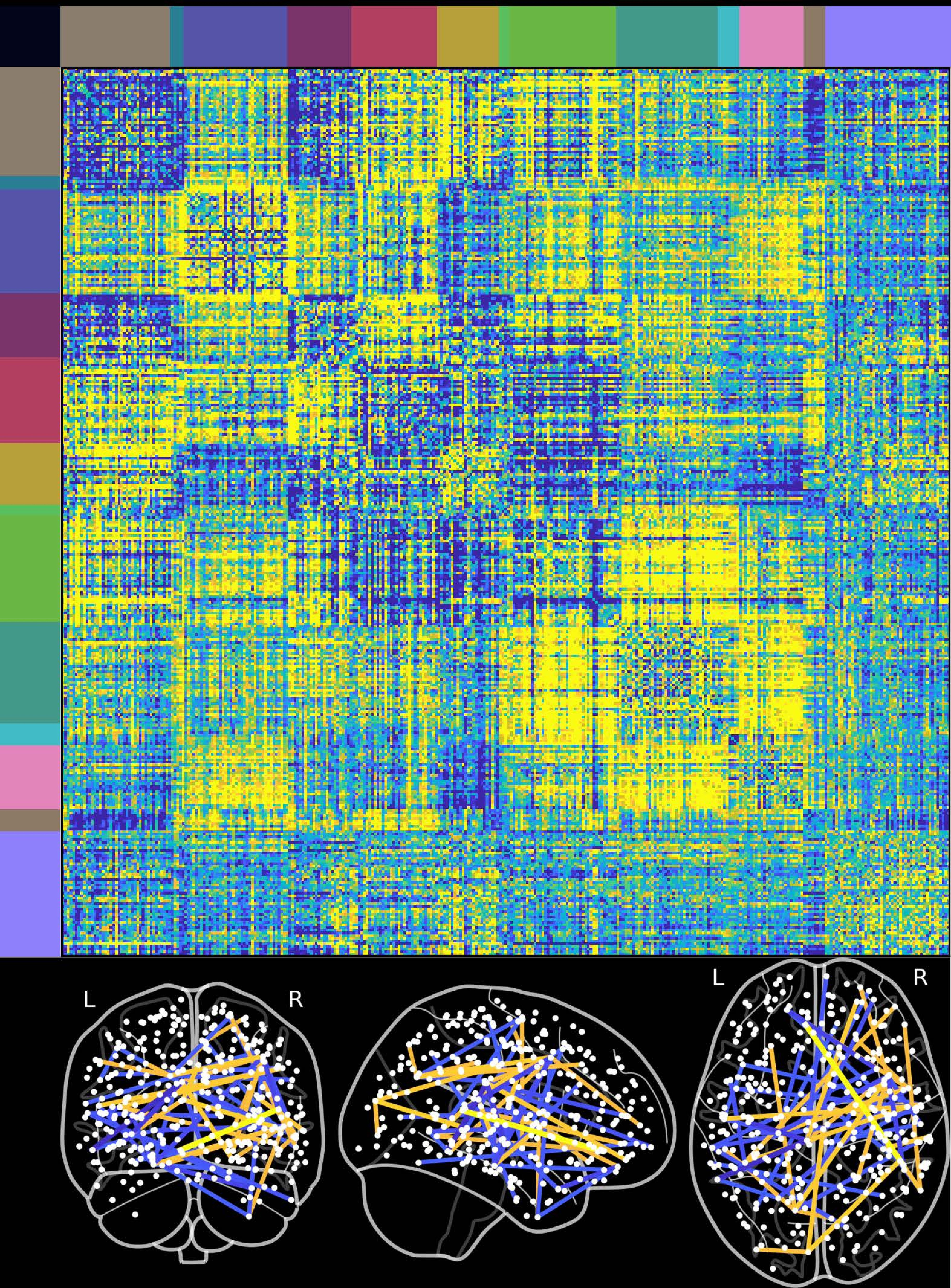

component_11

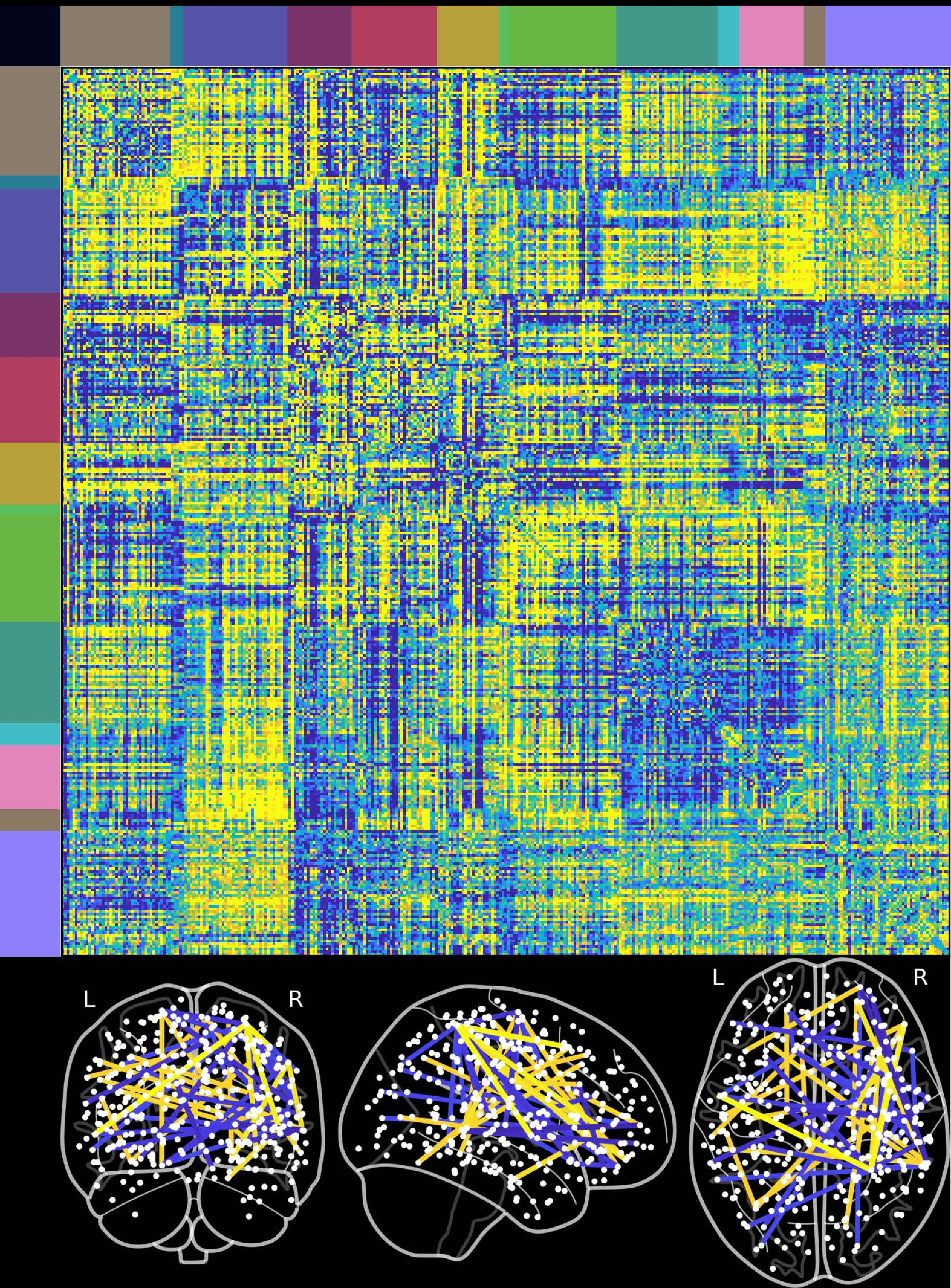

component_12

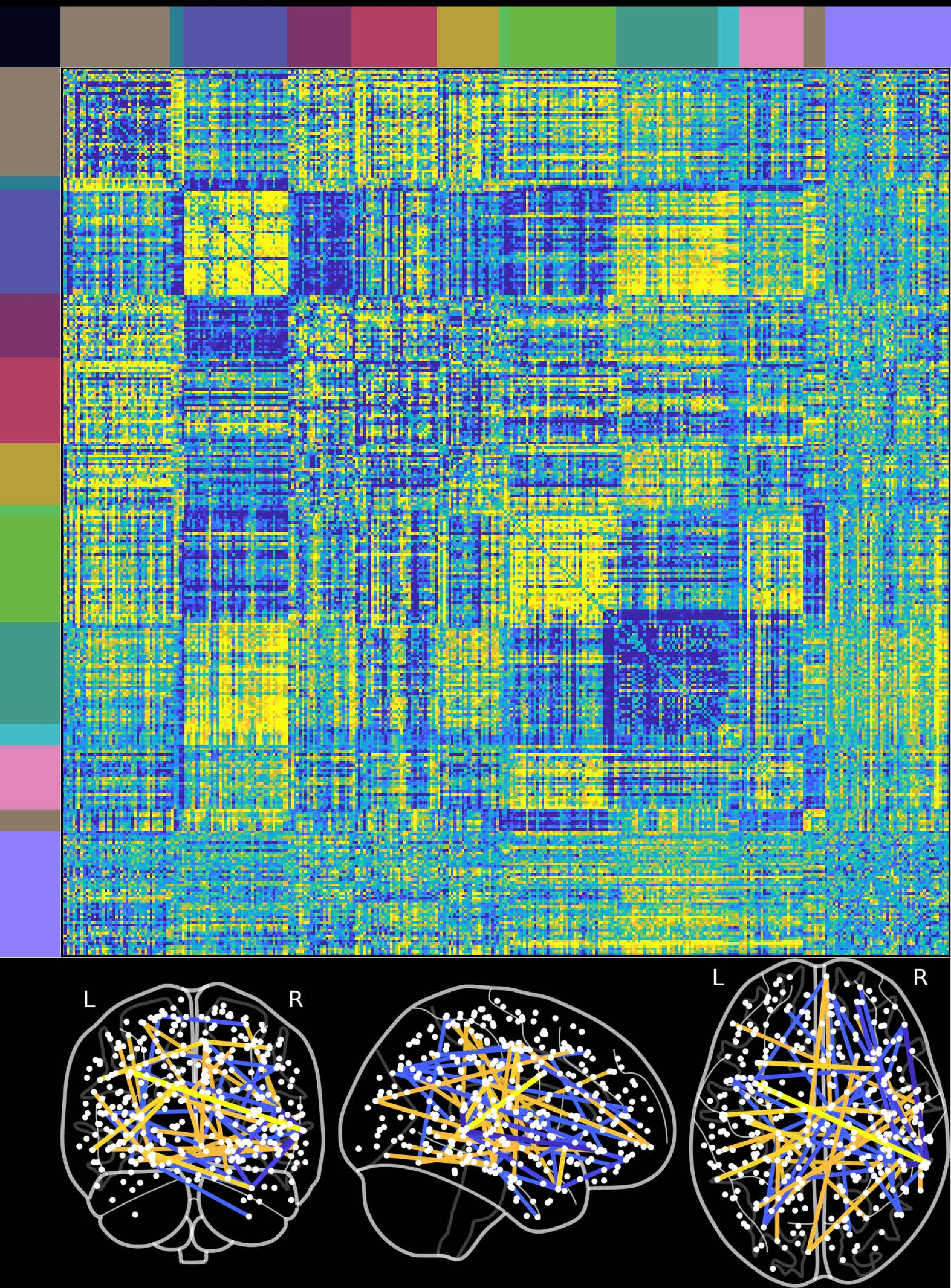

component_13

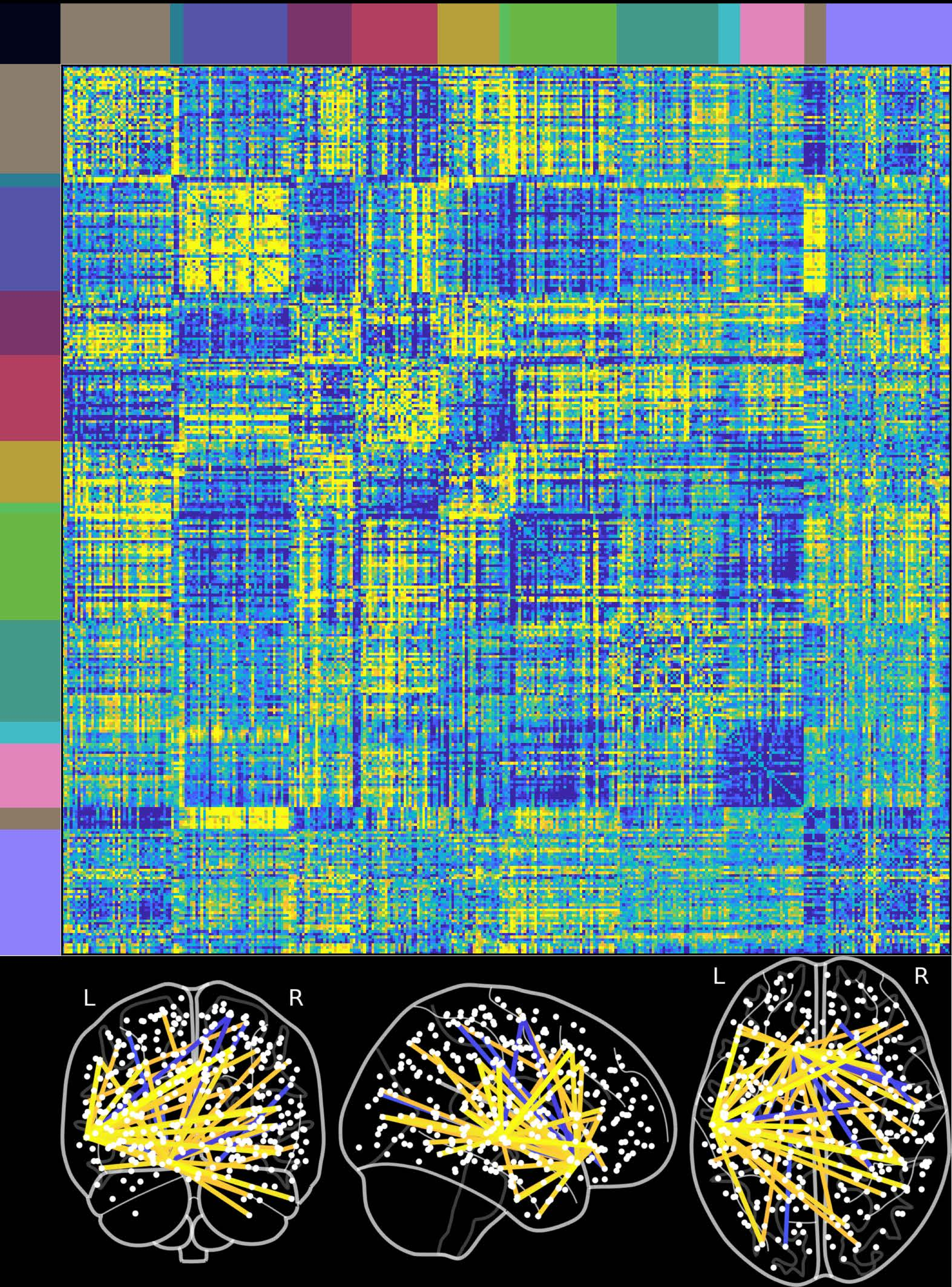

component_14

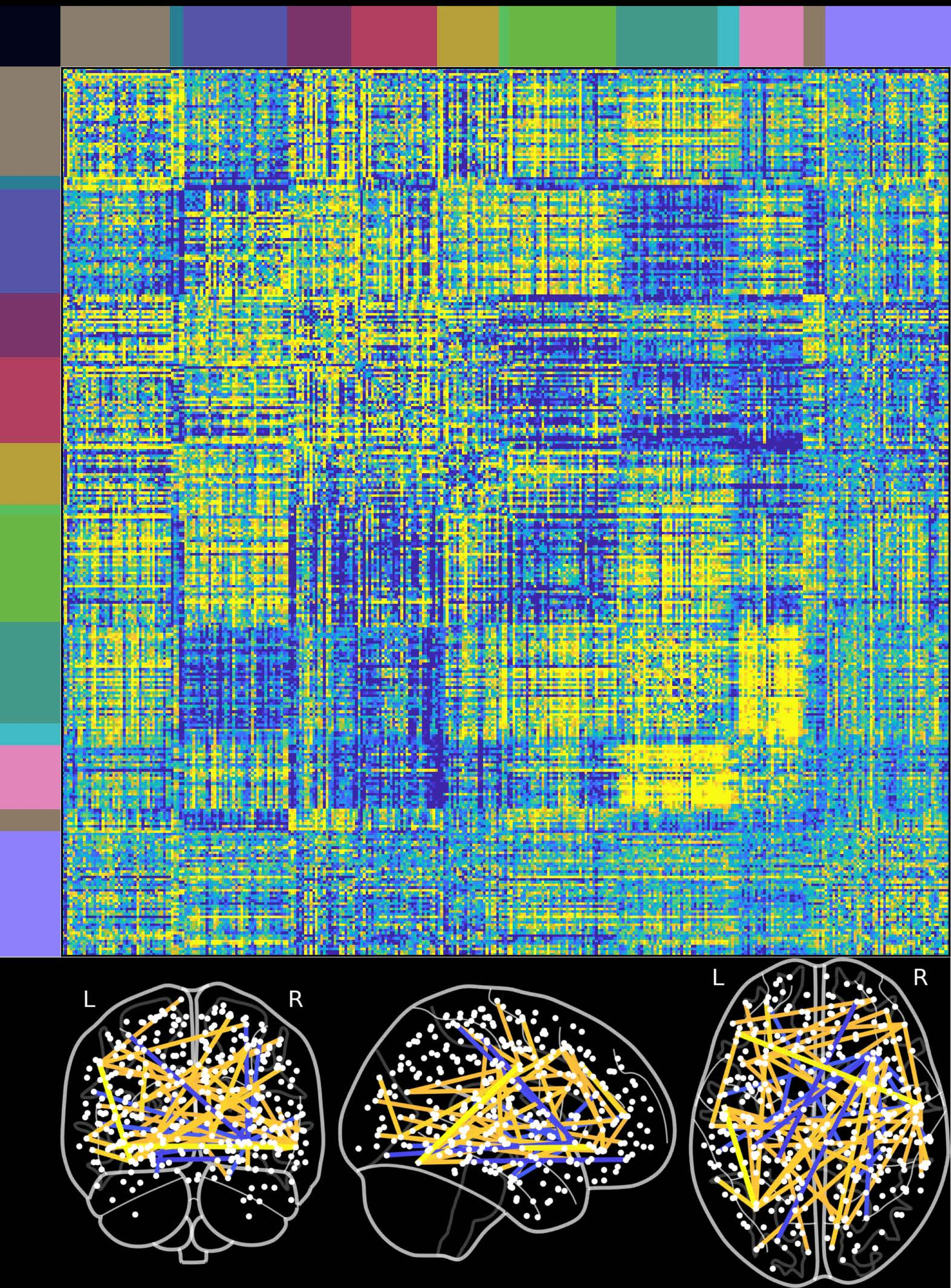

component_15

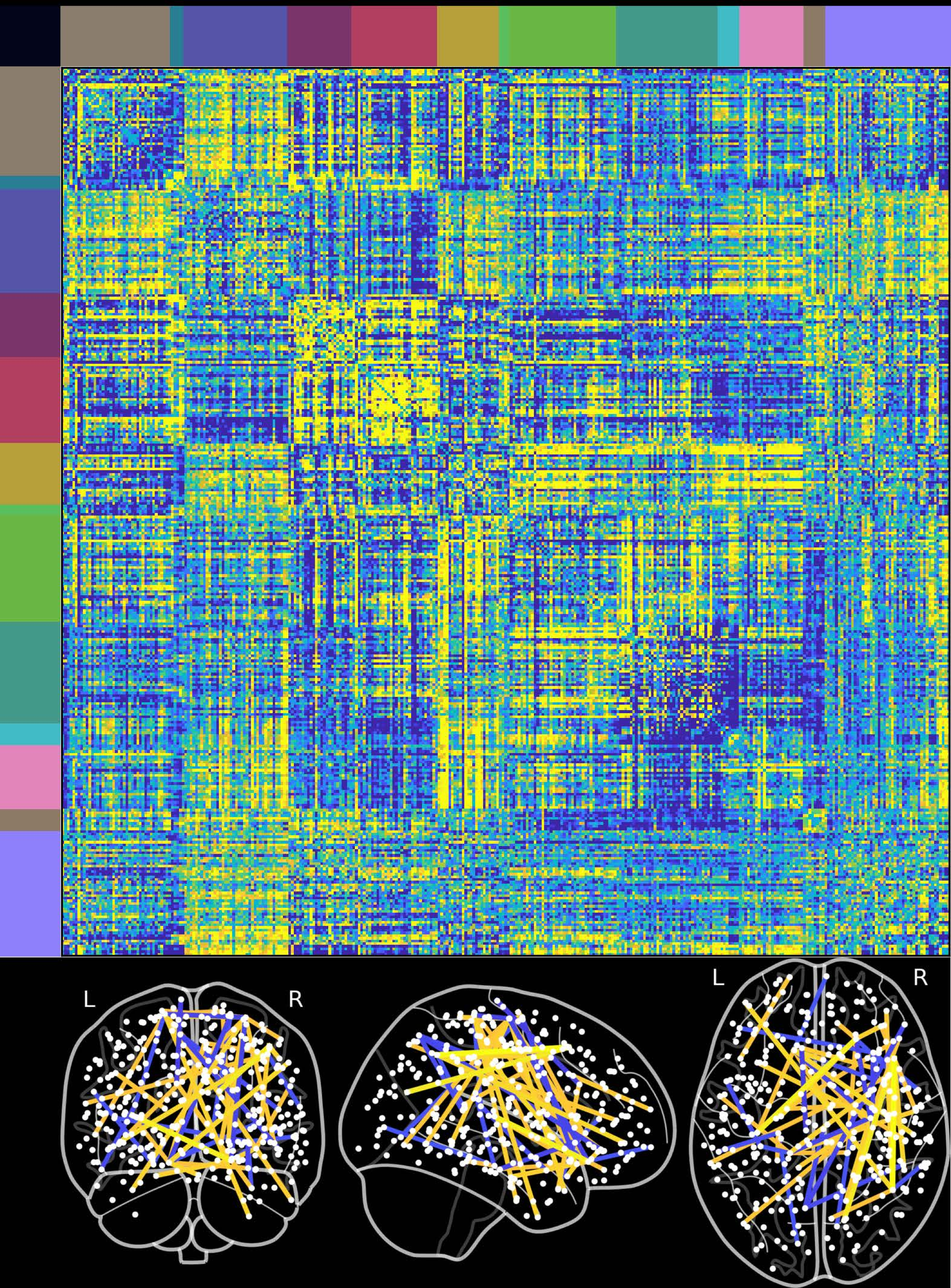

component_16

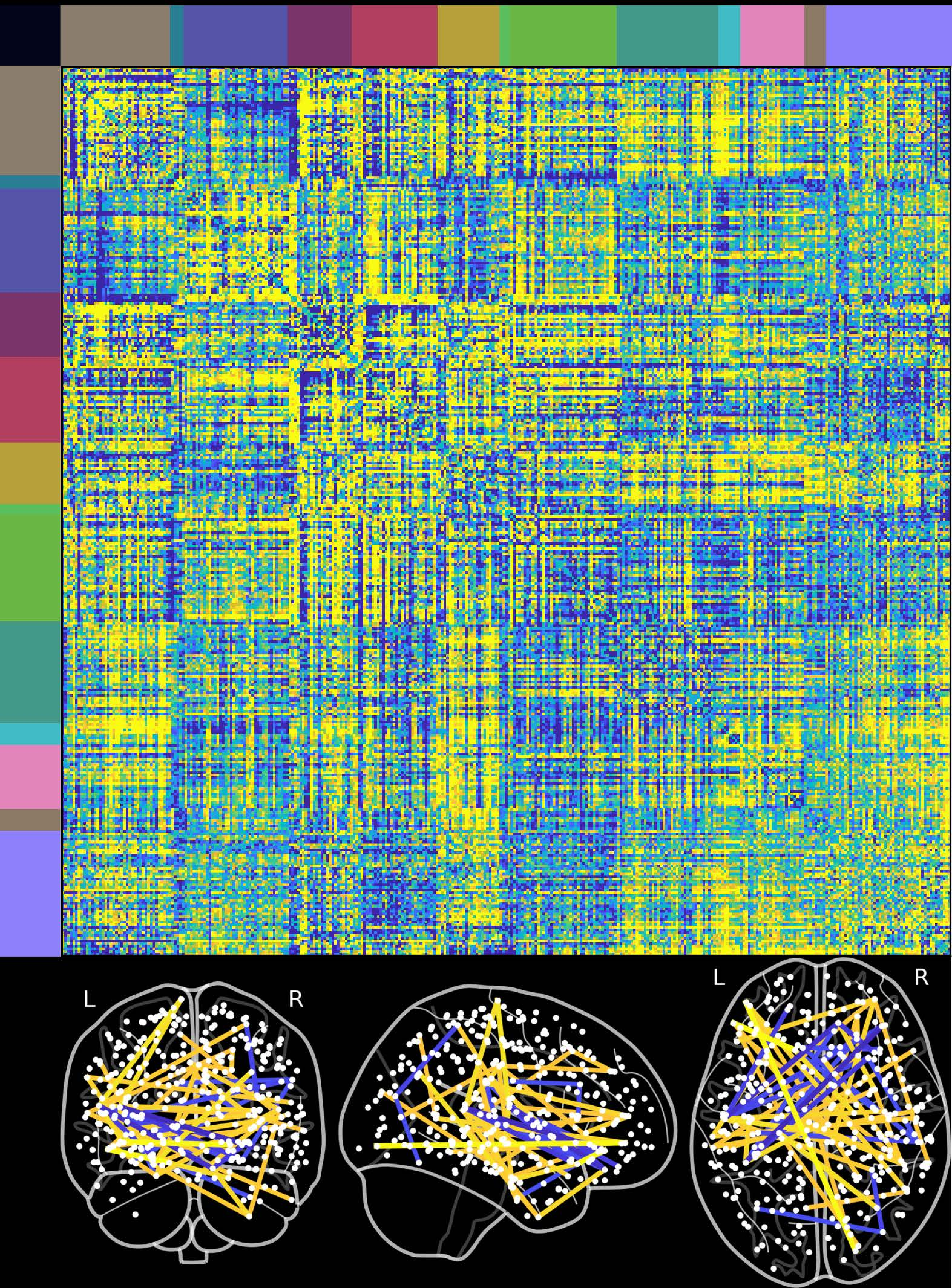

component_17

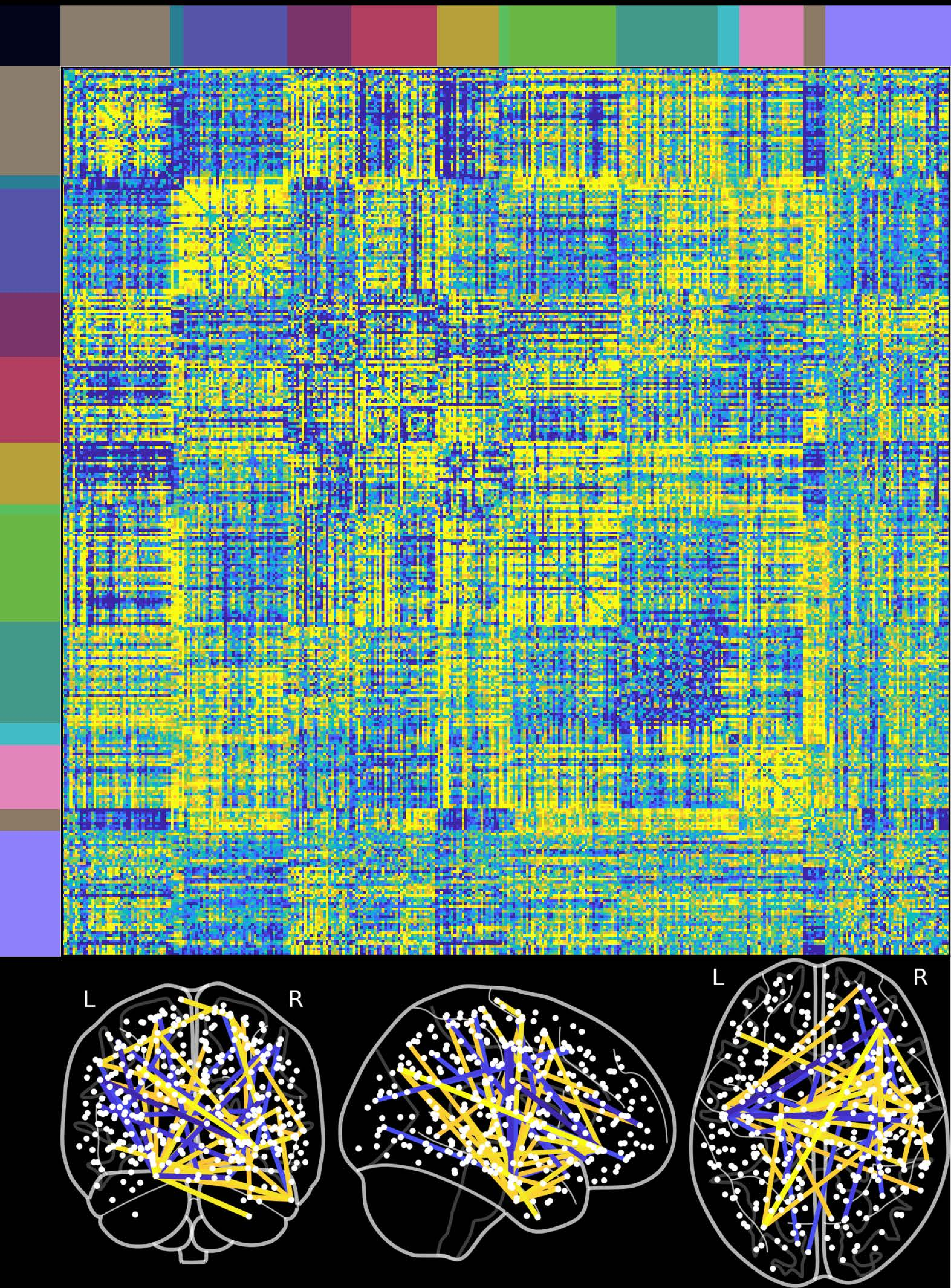

component_18

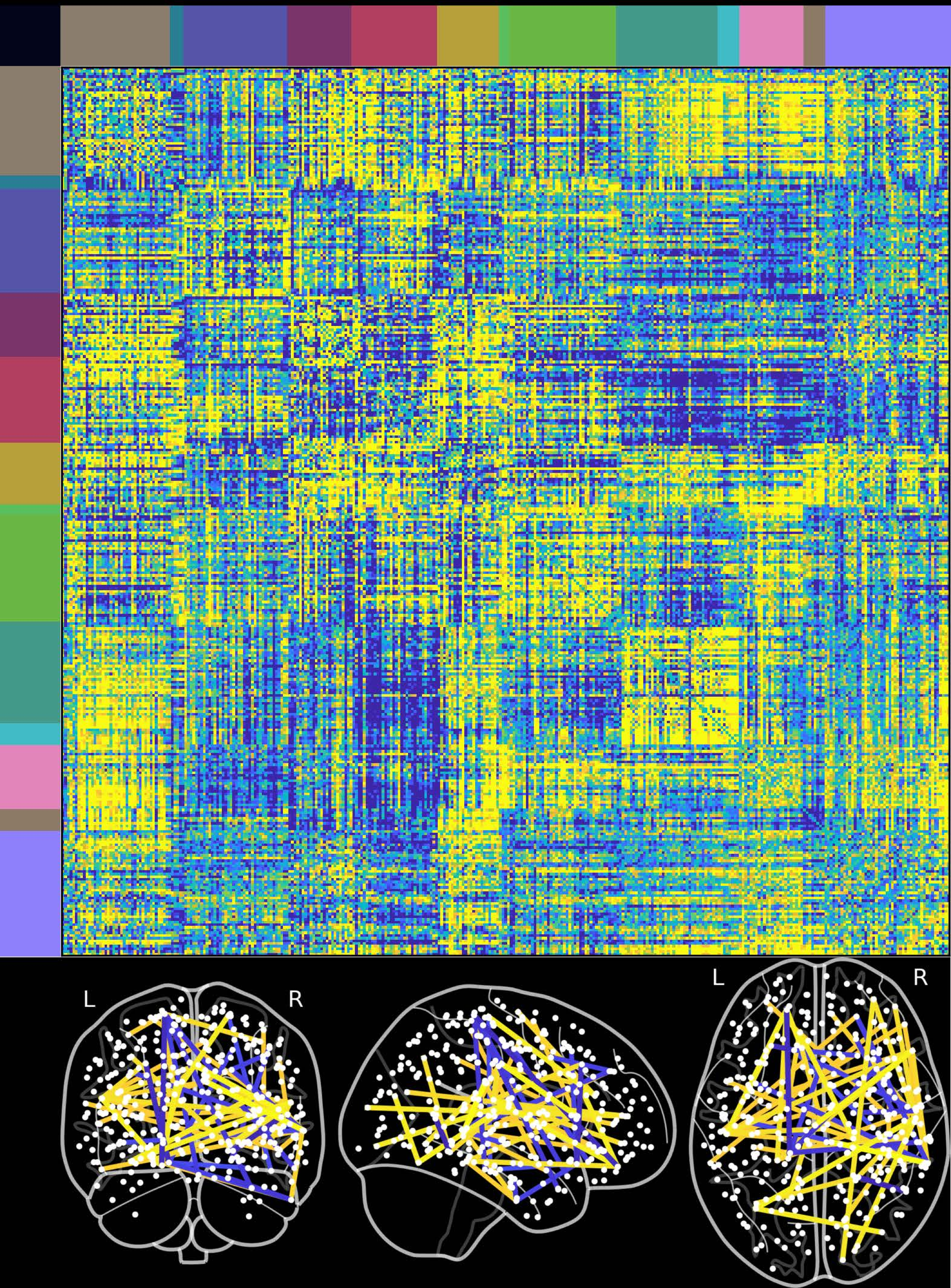

component_19

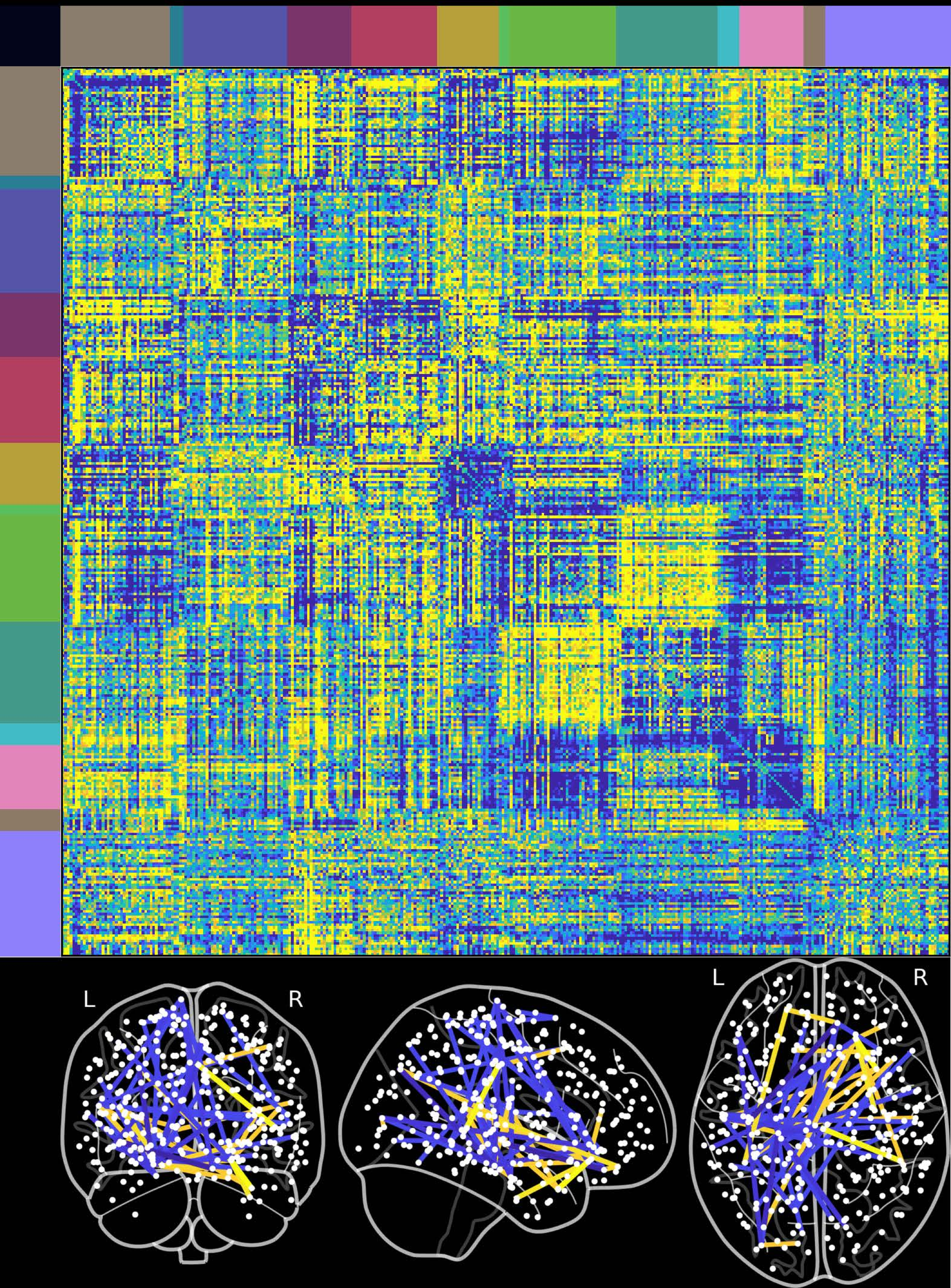

component_20

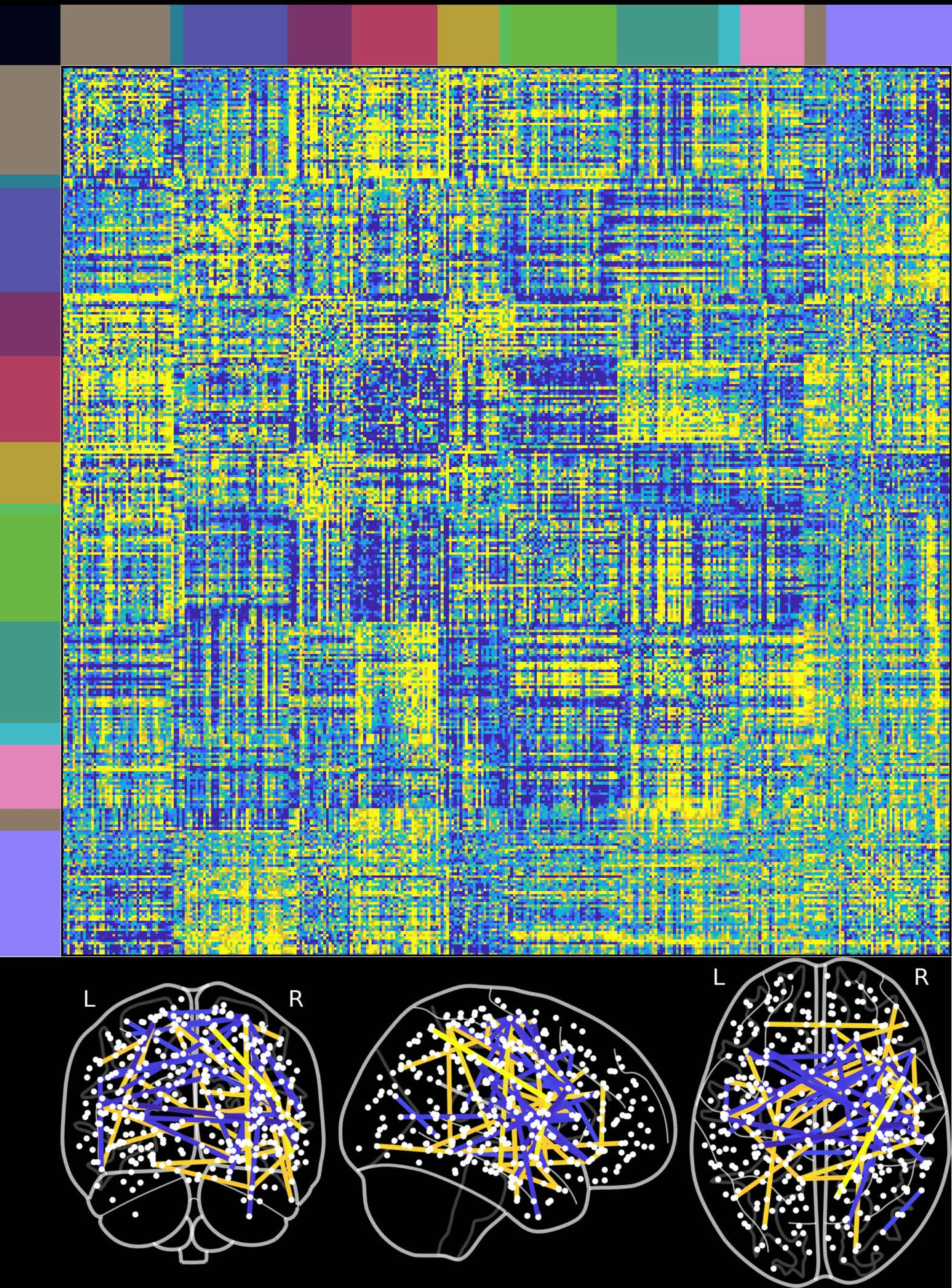

component_21

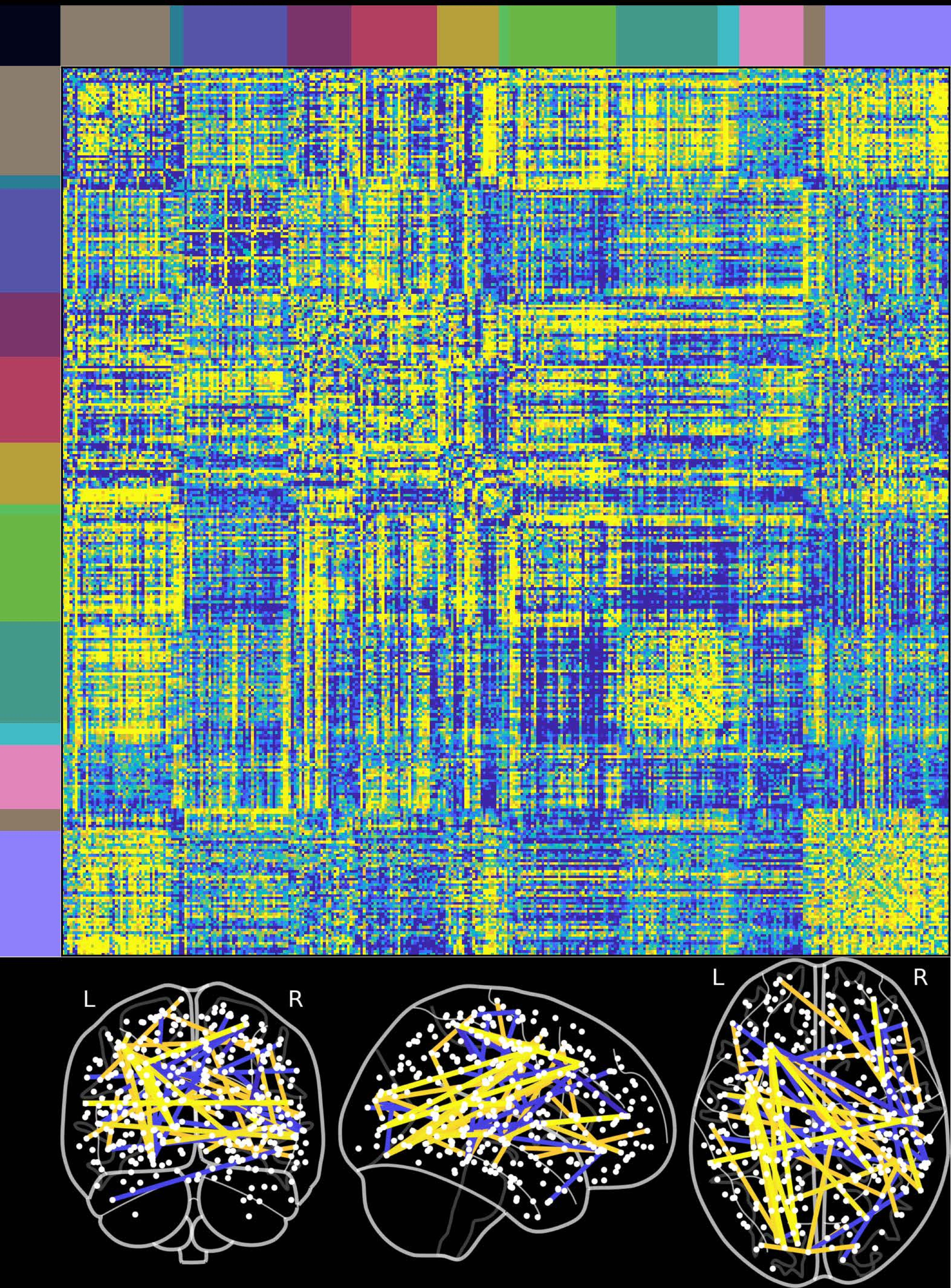

component_22

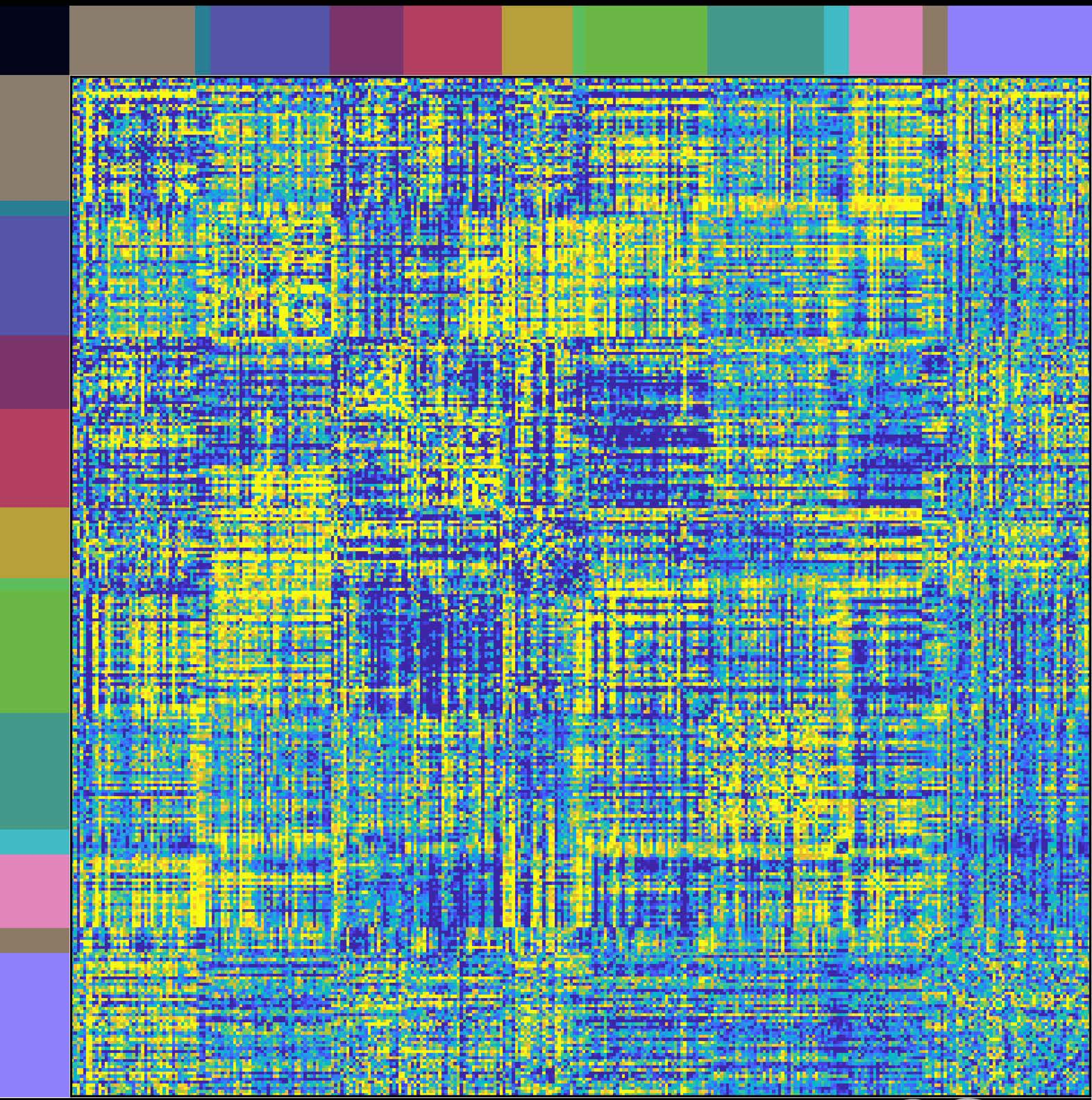

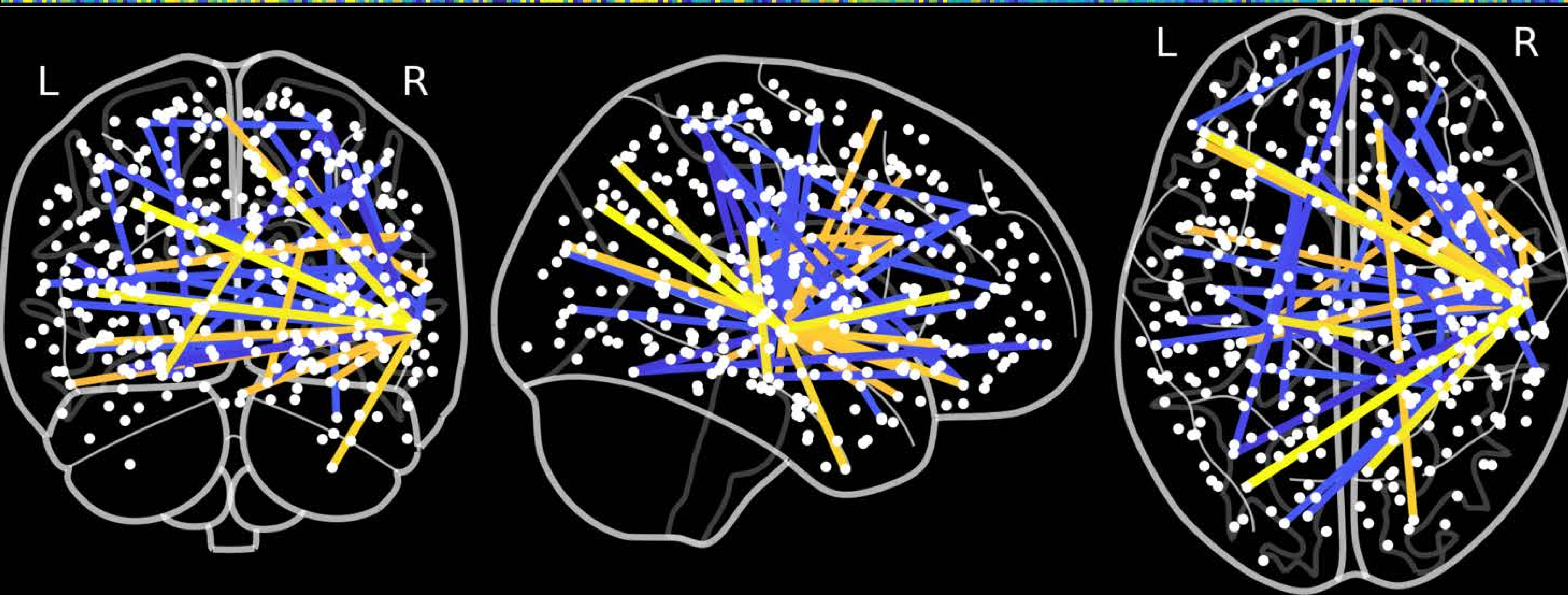

component_23

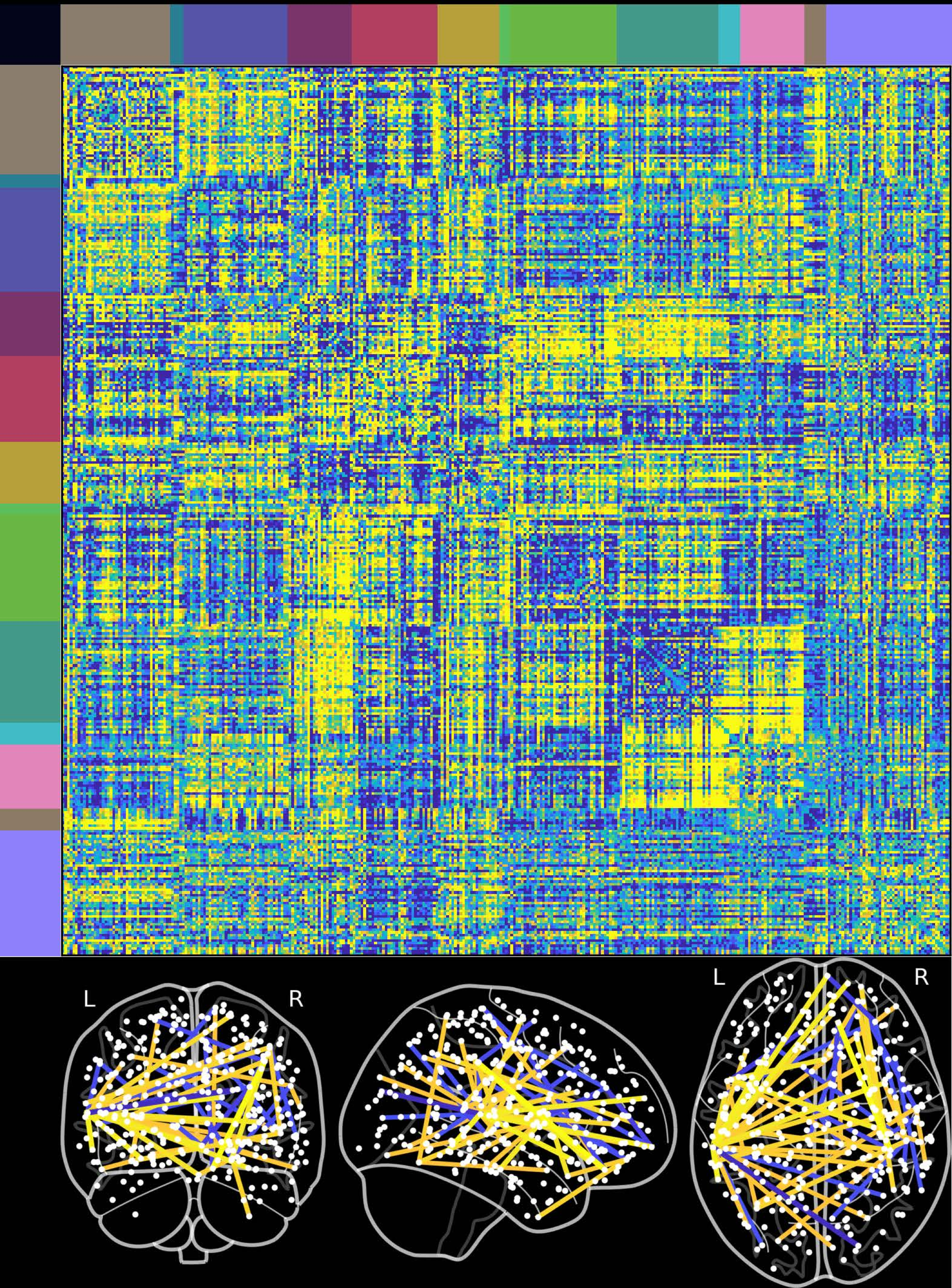

component_24

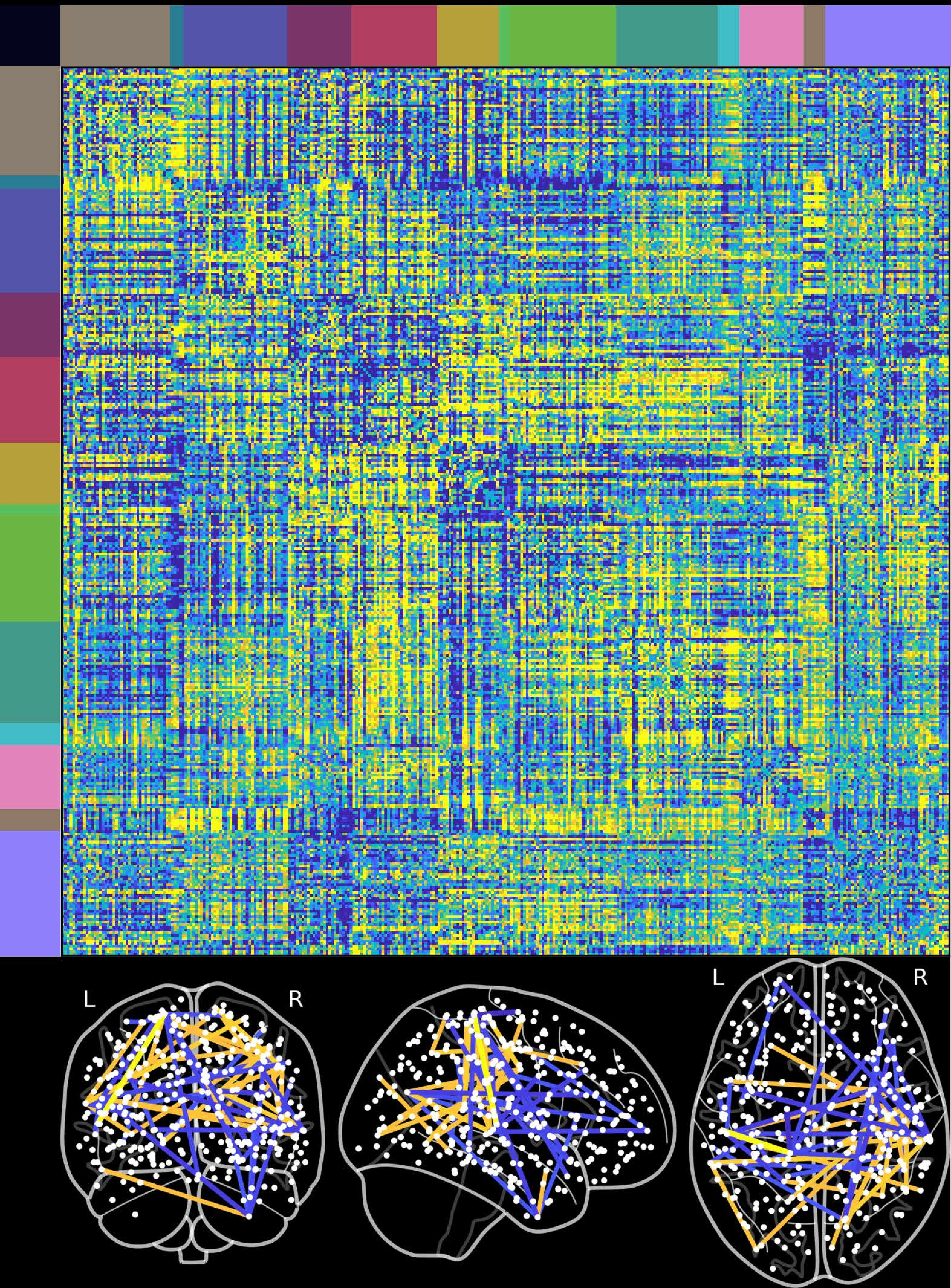

component_25

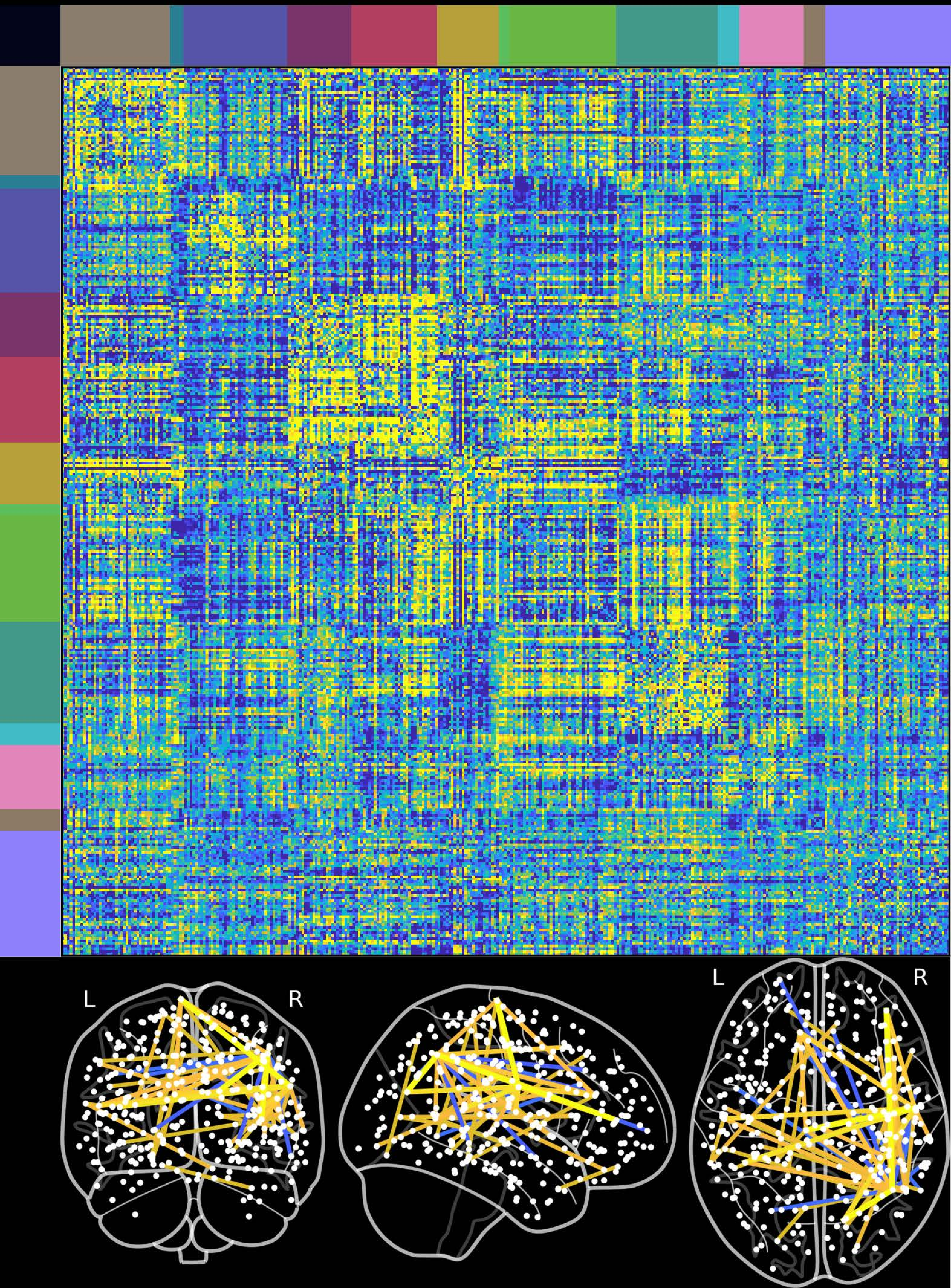

component_26

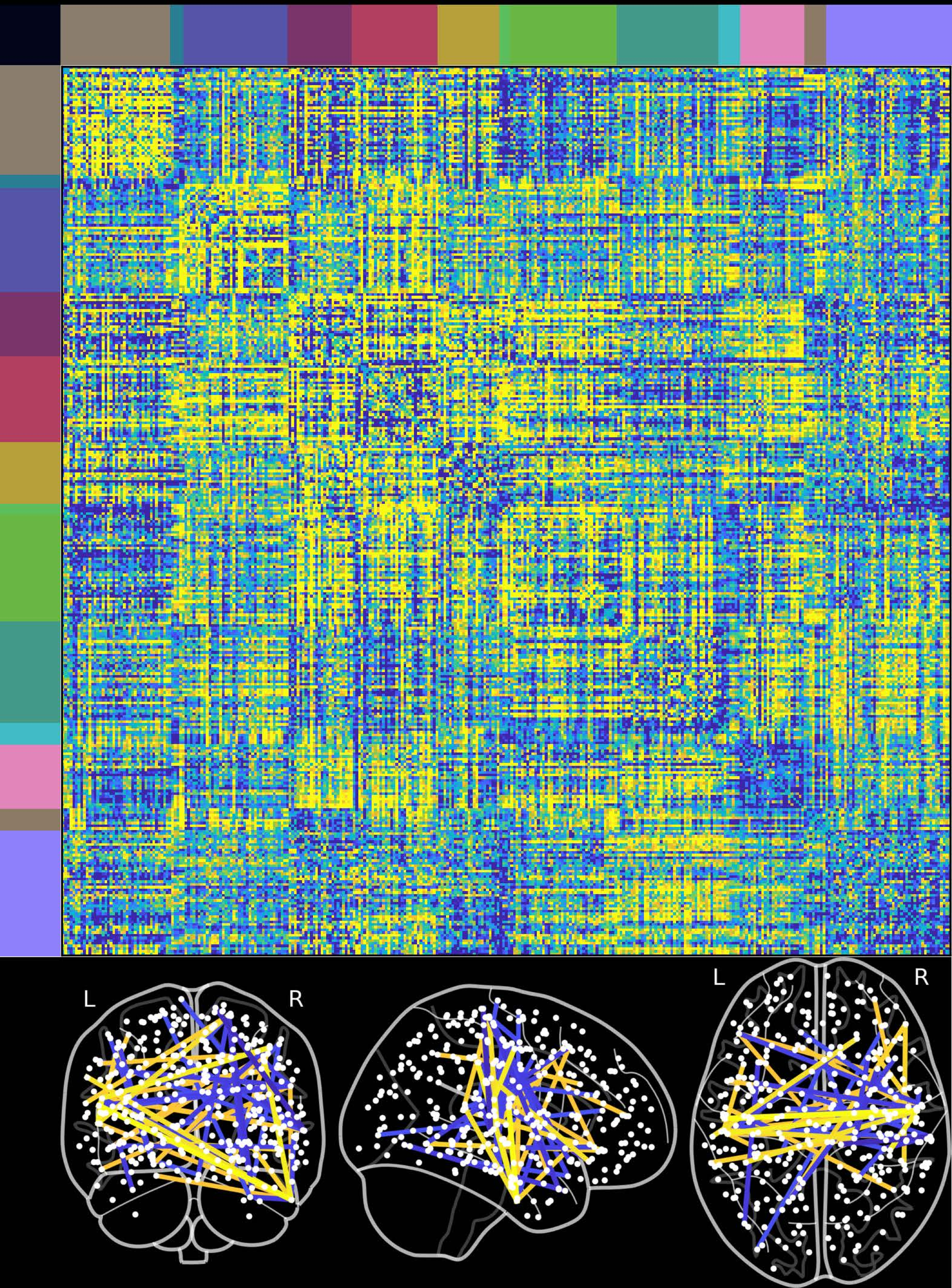

component_27

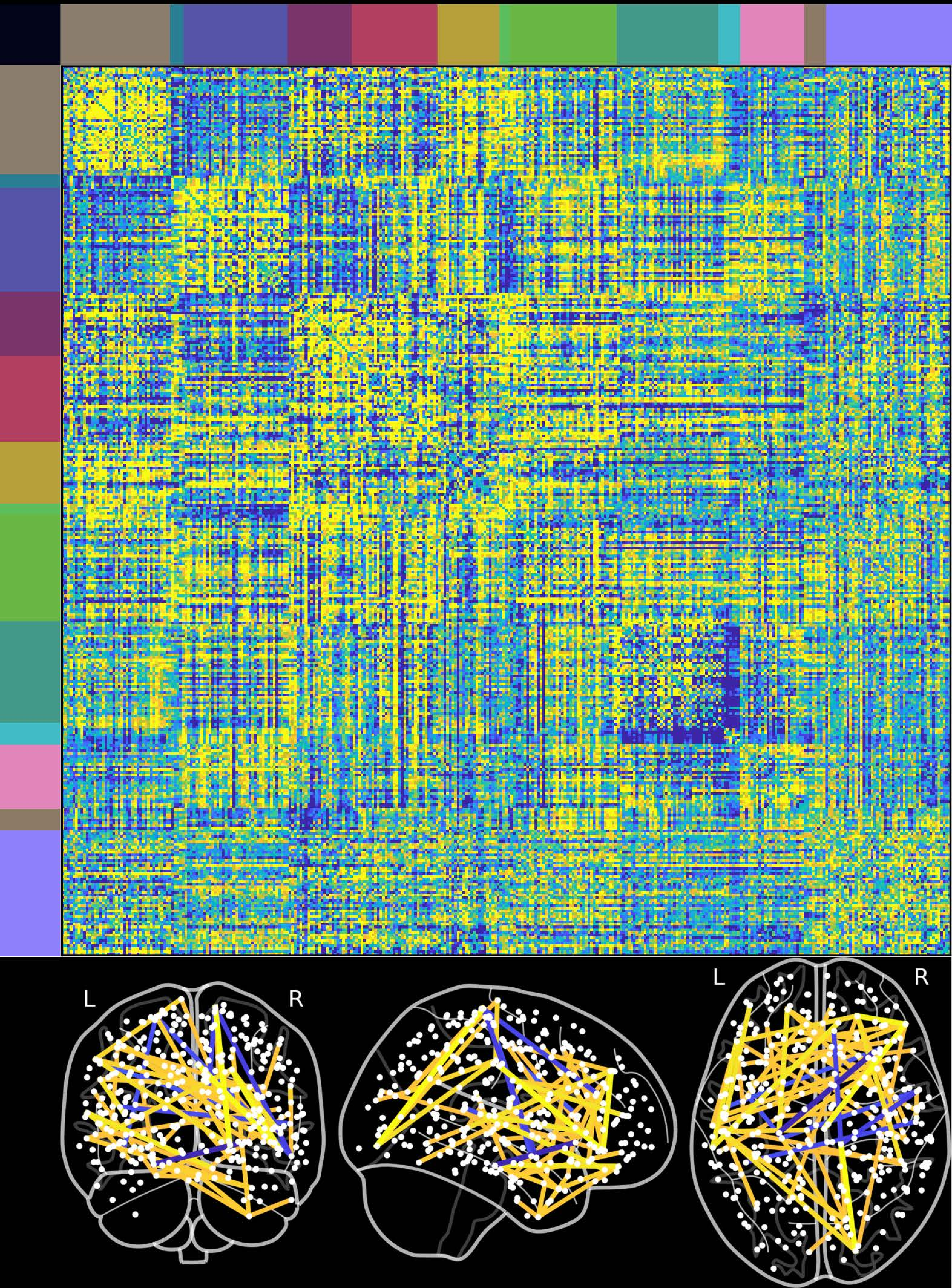

component_28

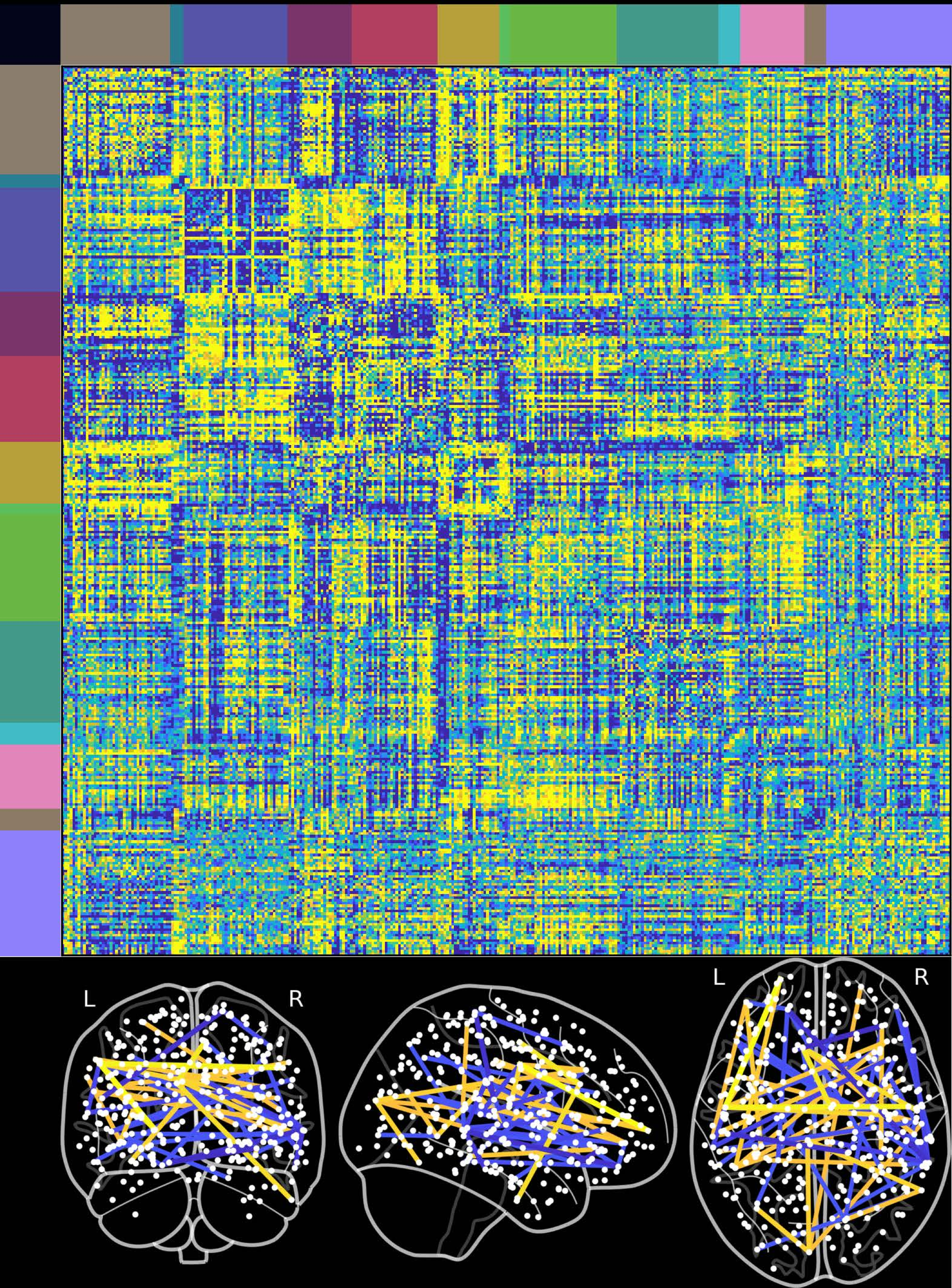

component_29

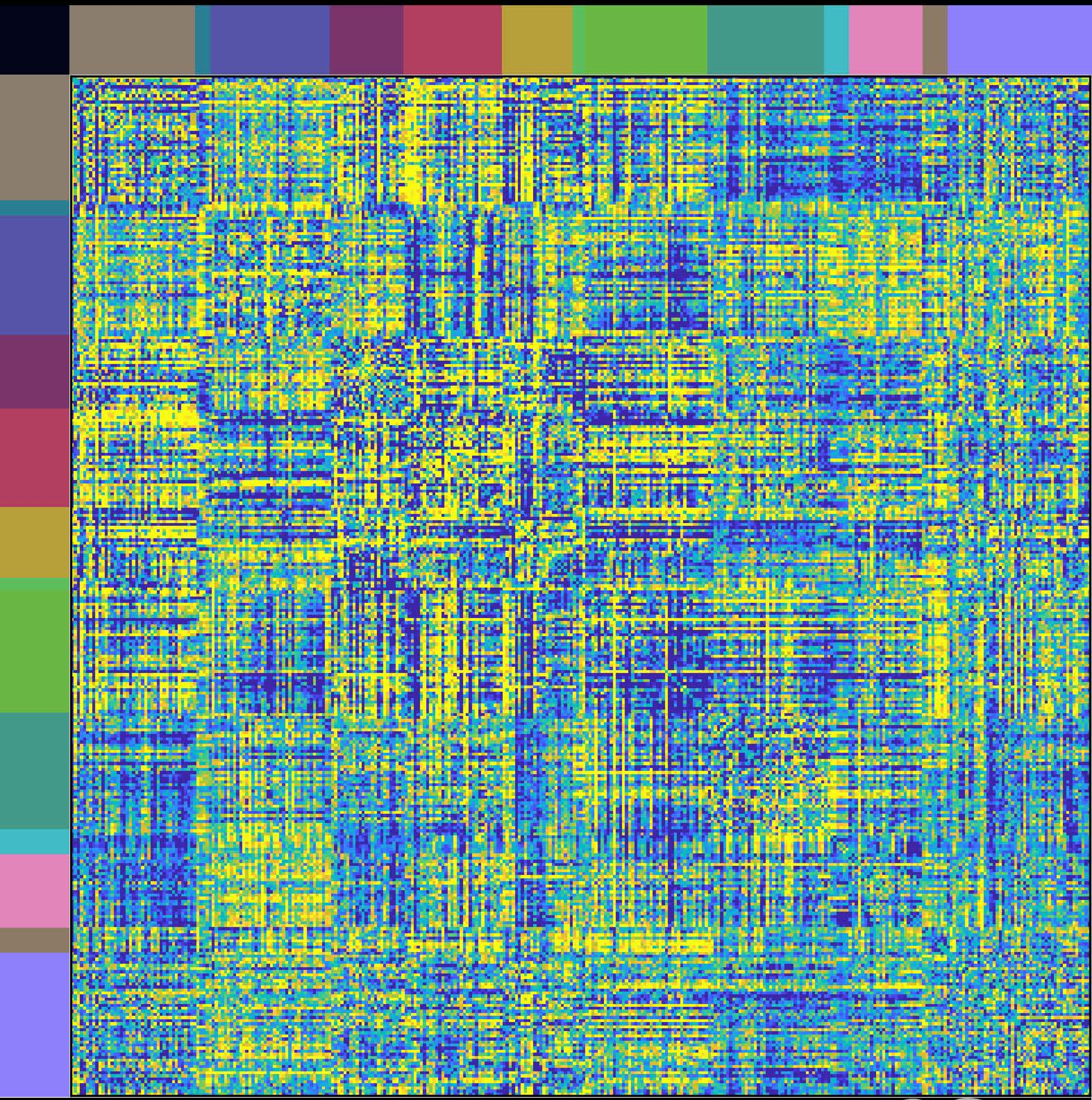

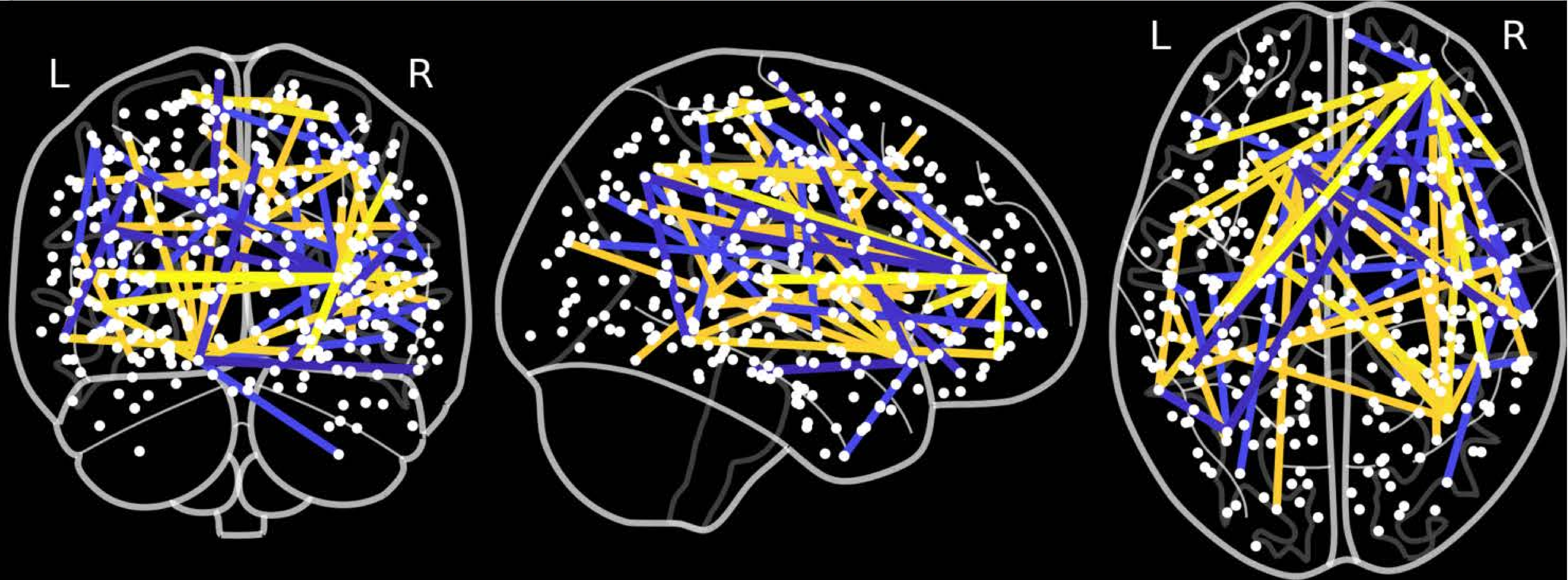

component_30

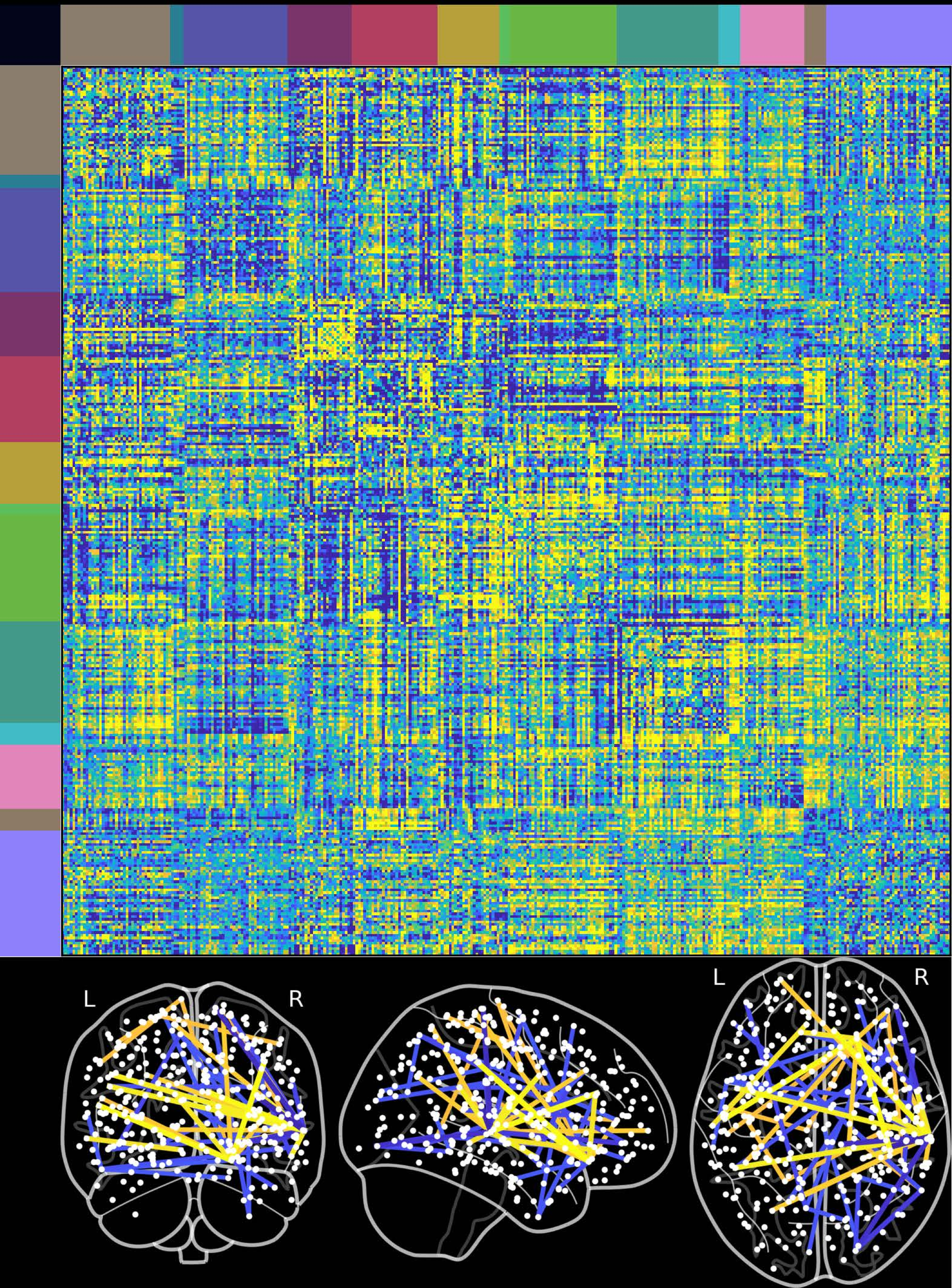

component_31

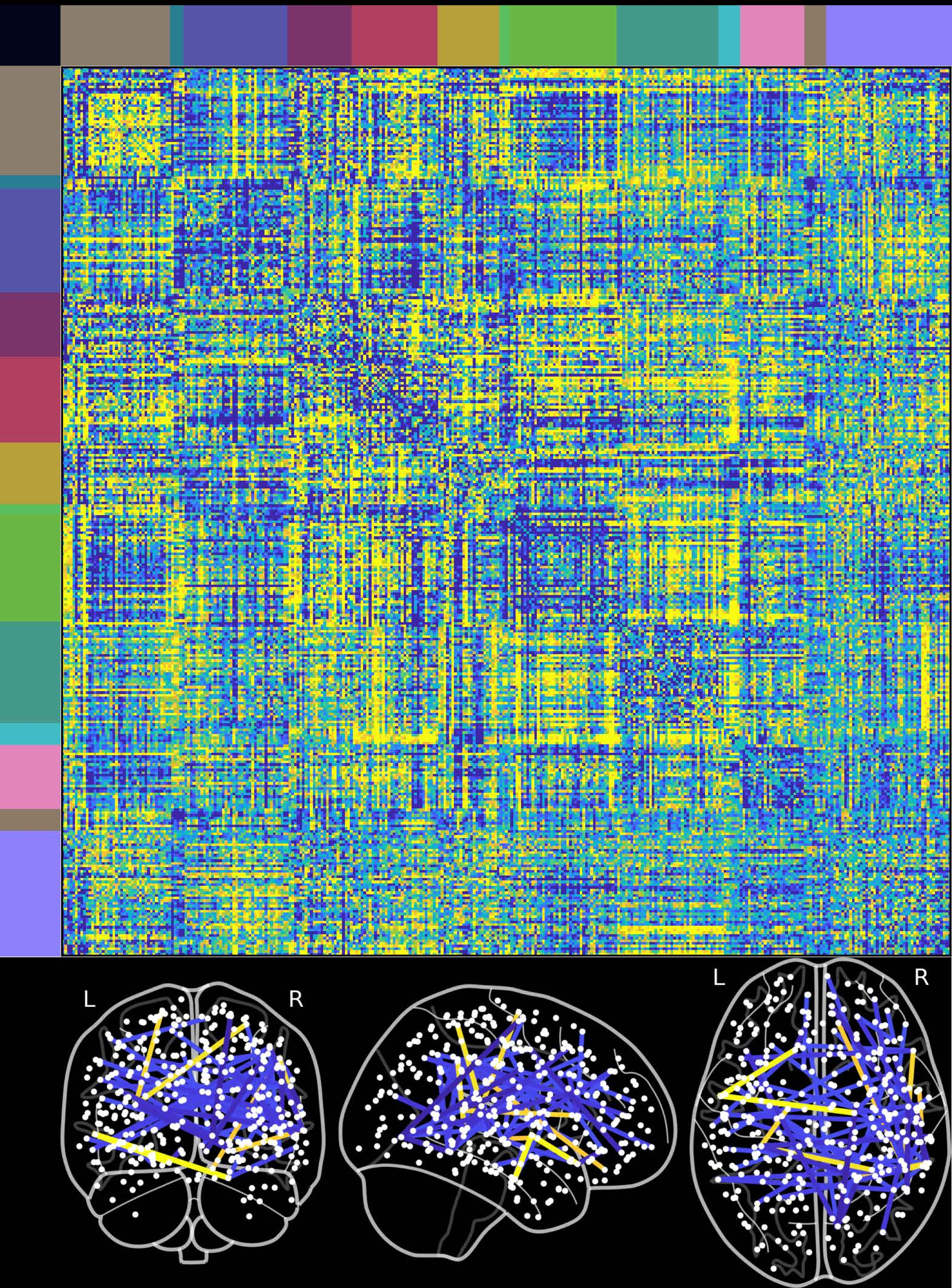

component_32

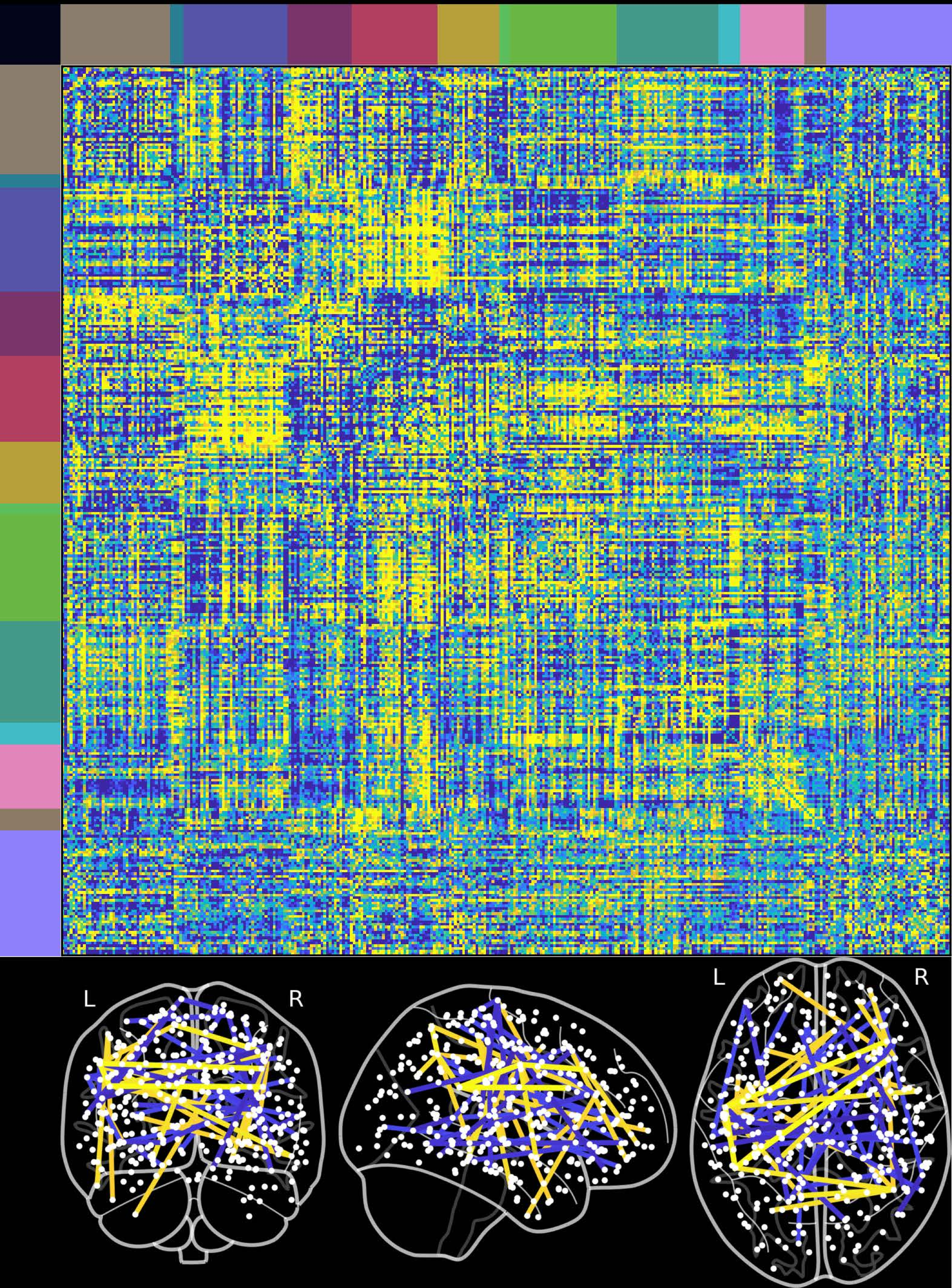

component_33

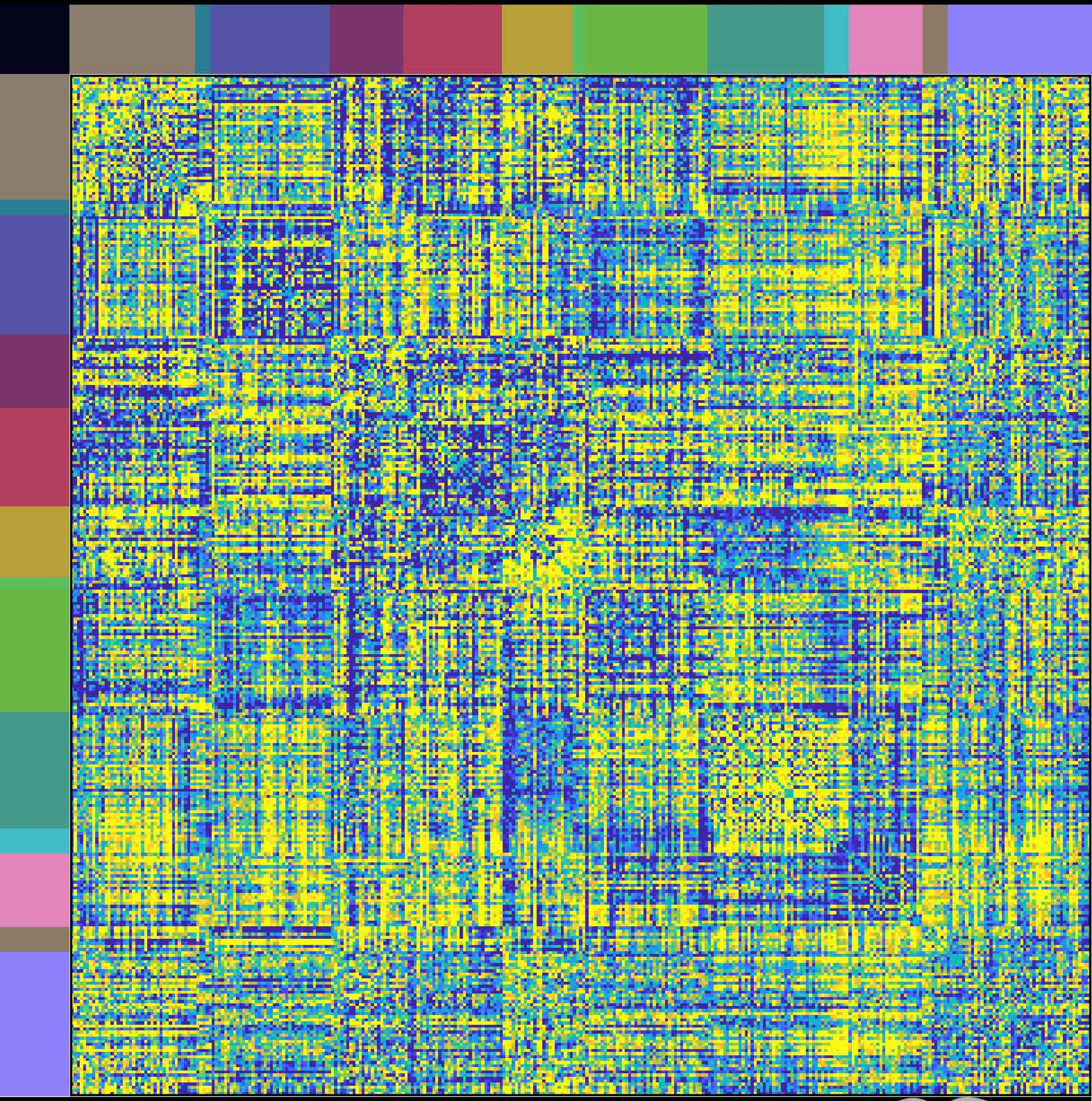

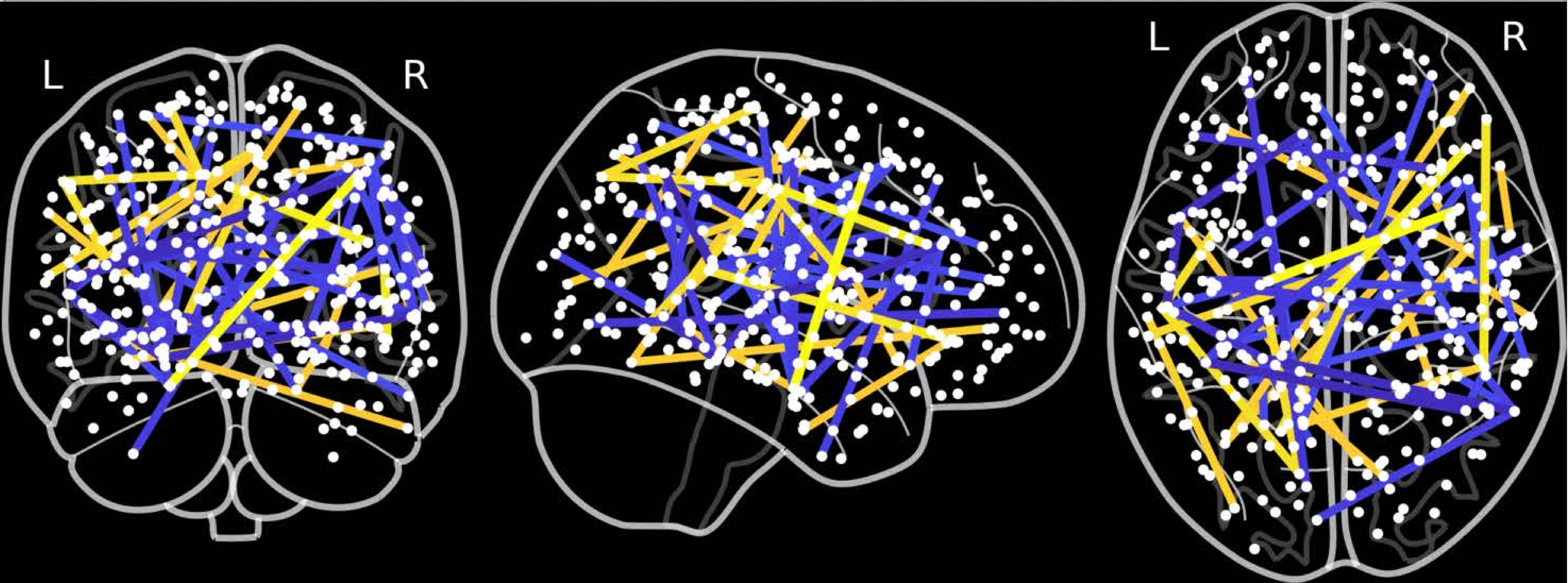

component_34

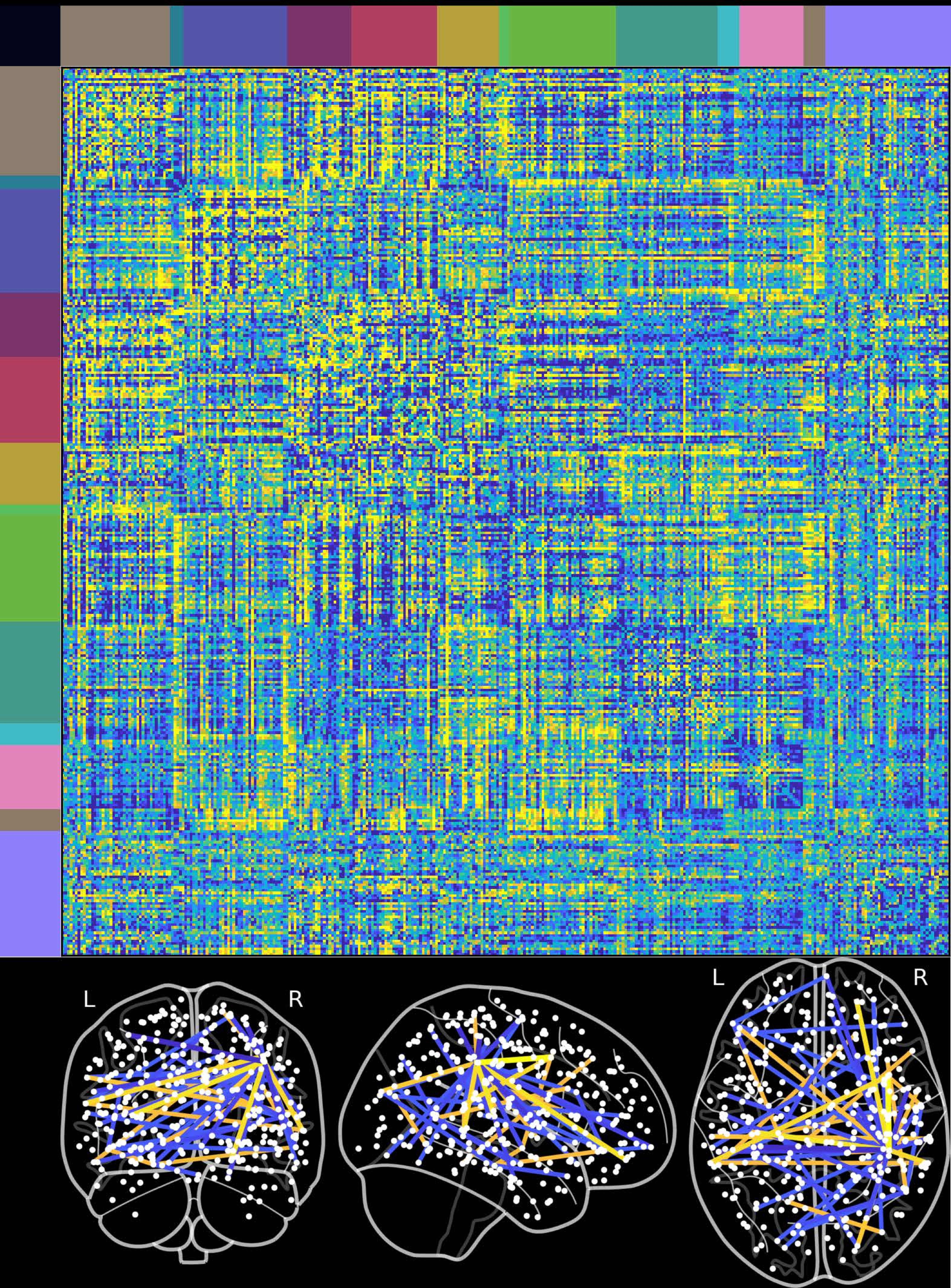

component_35

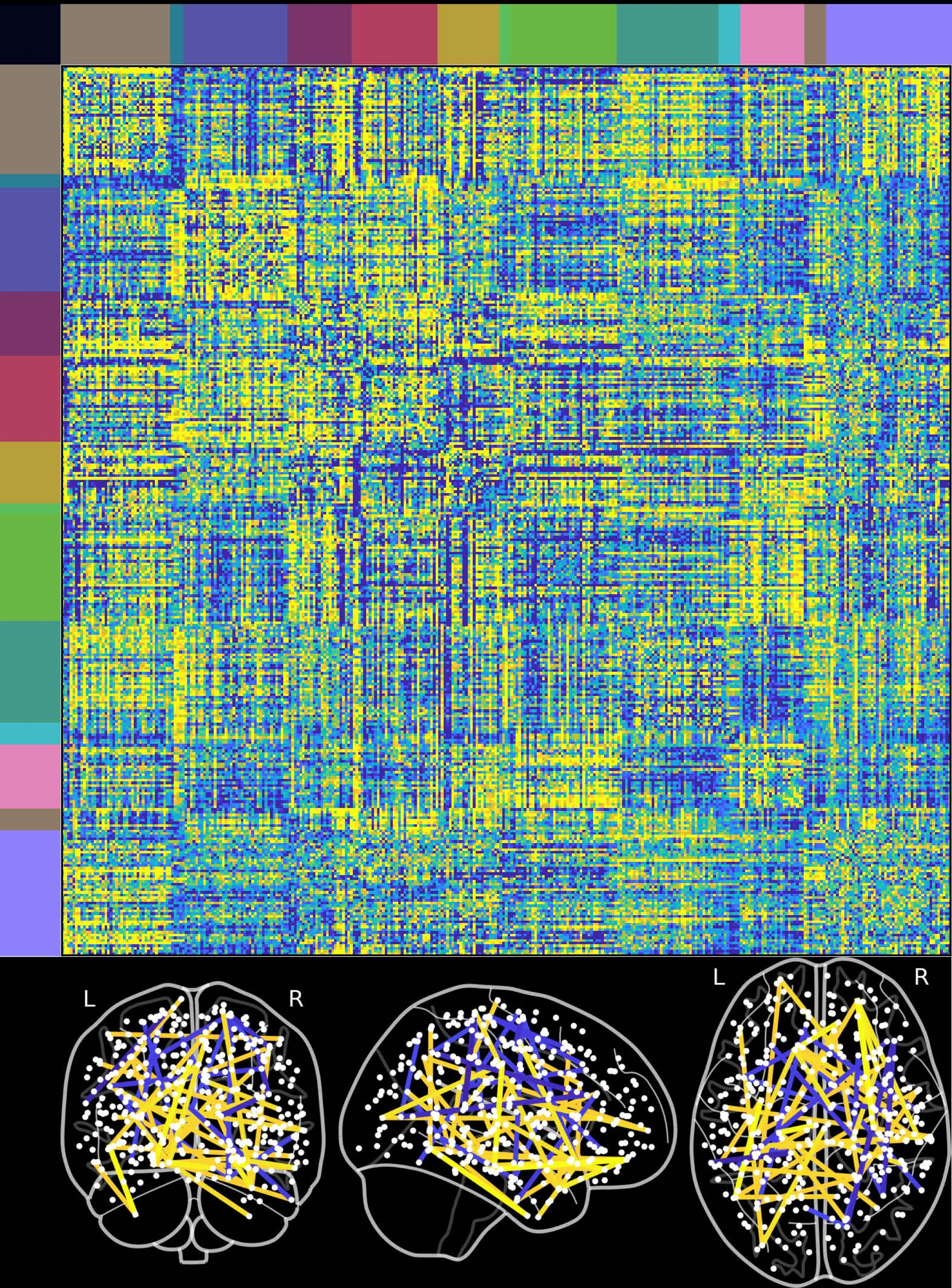

component_36

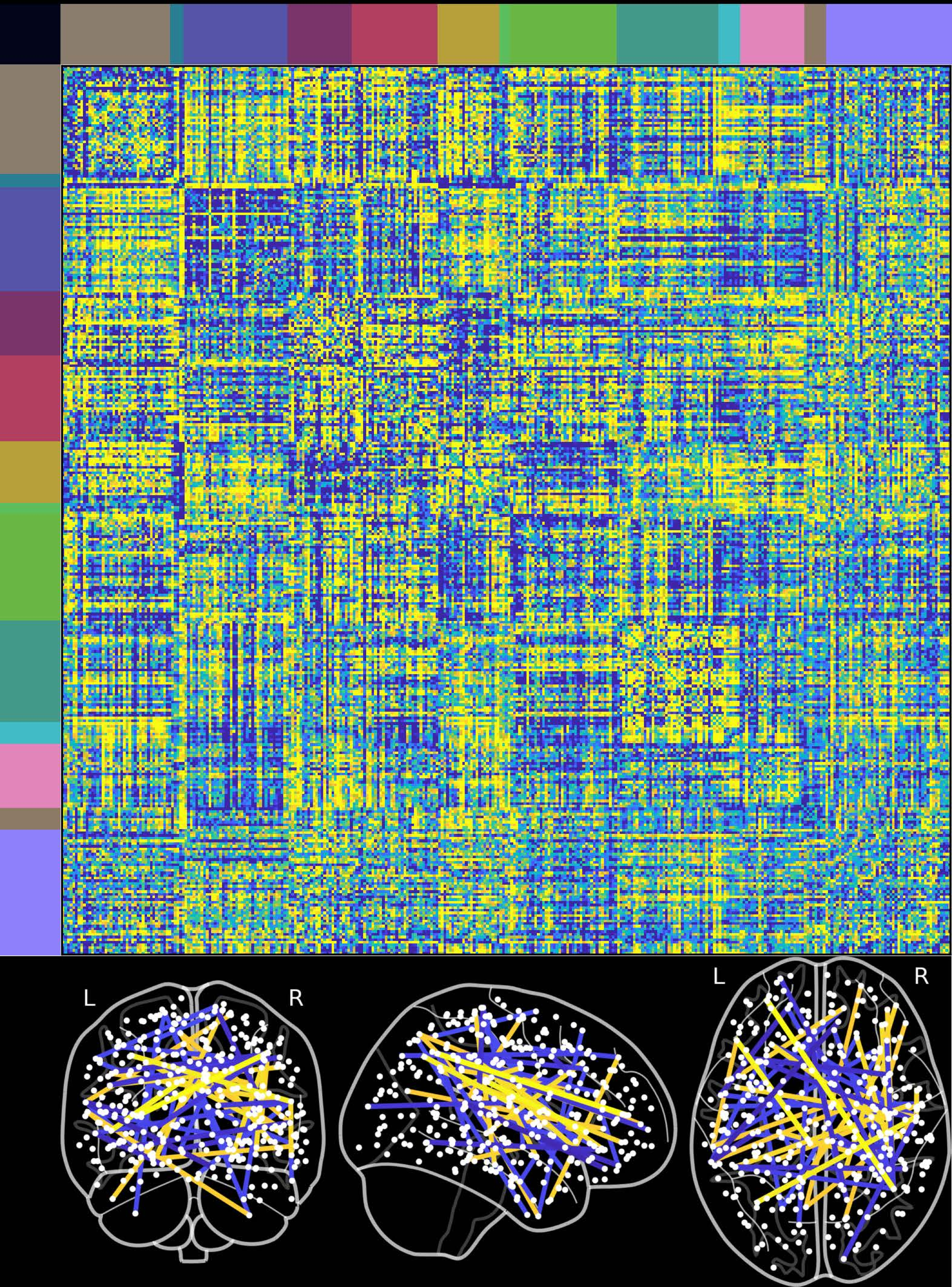

component_37

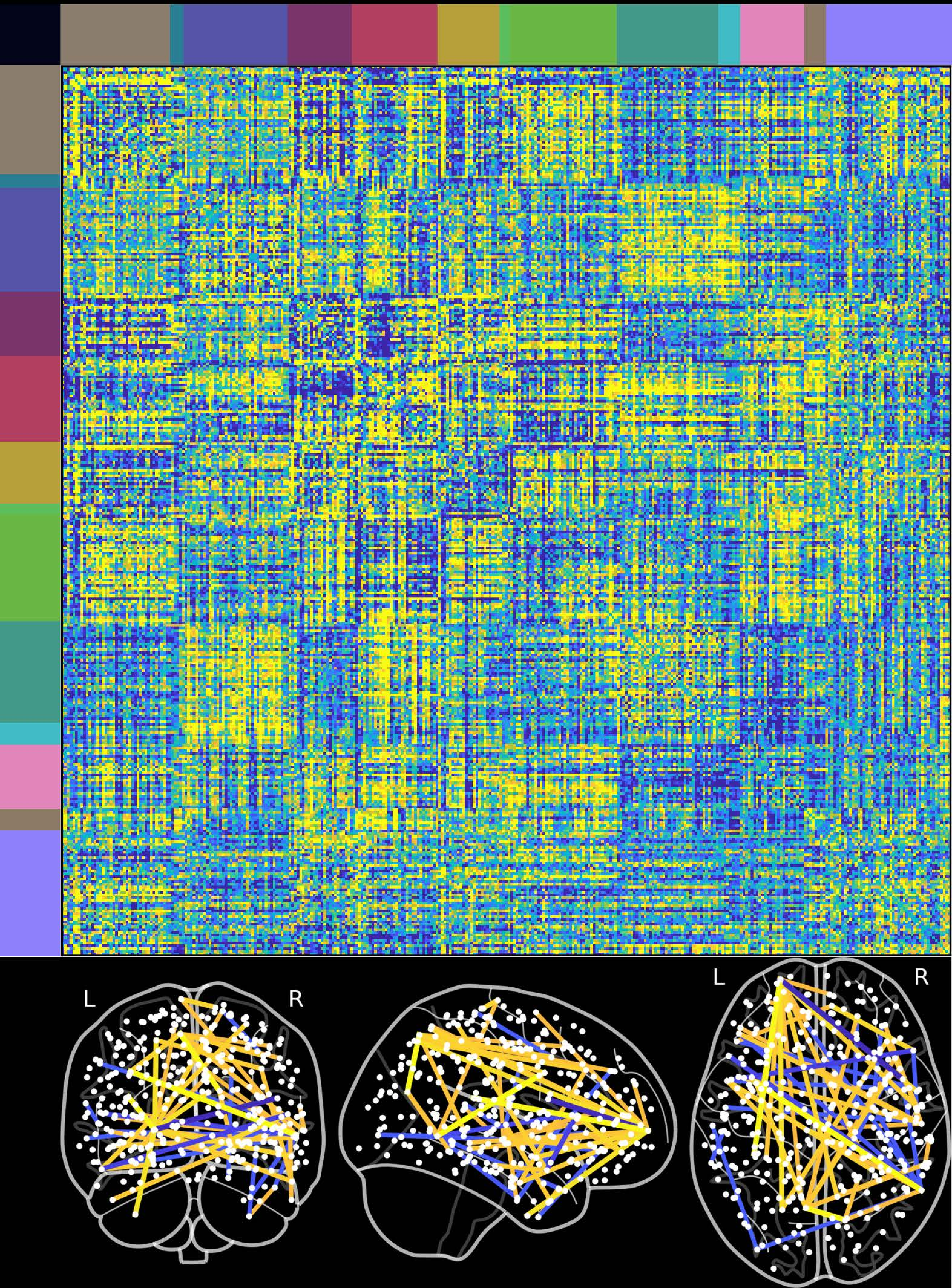

component_38

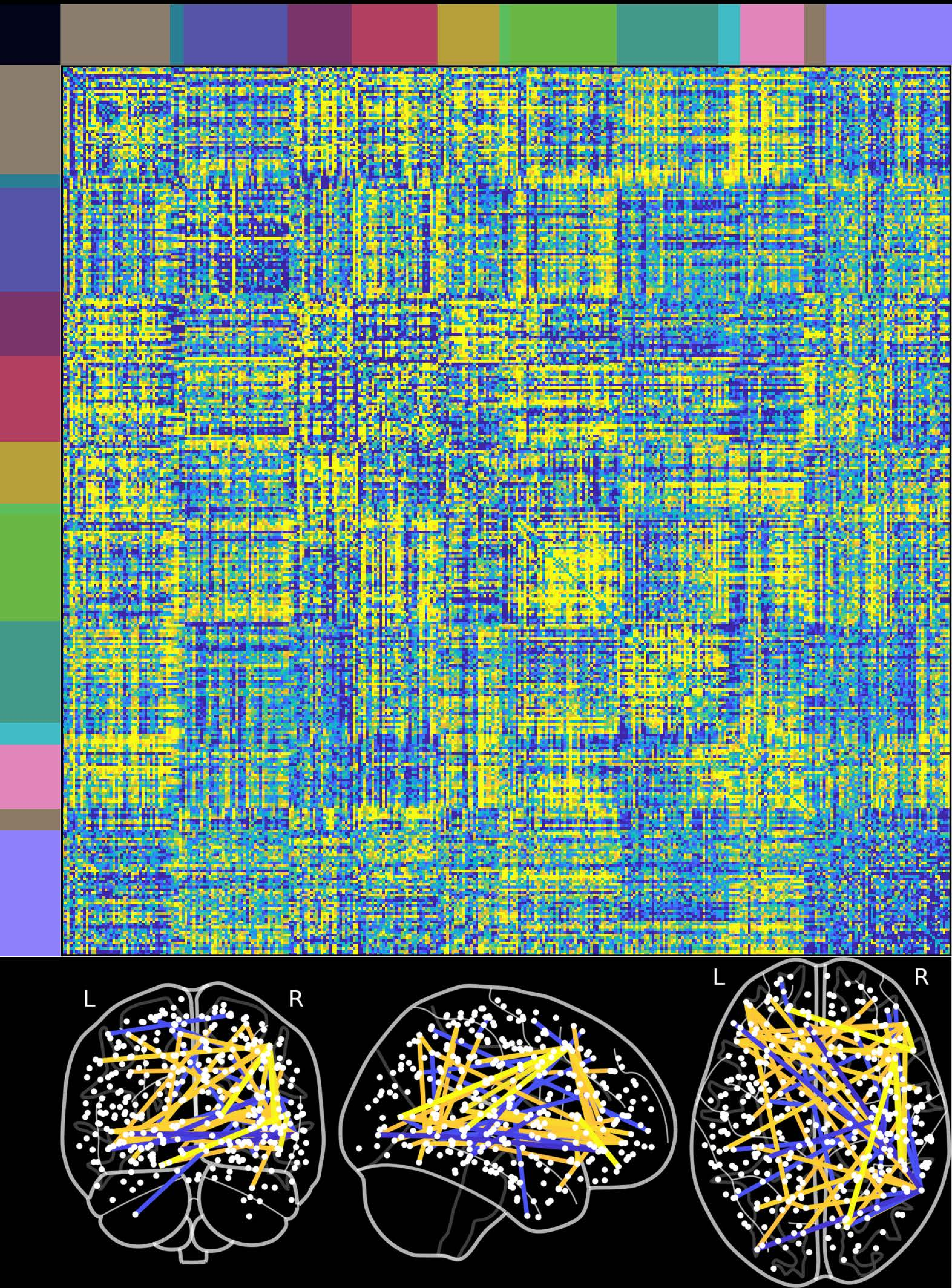

component_39

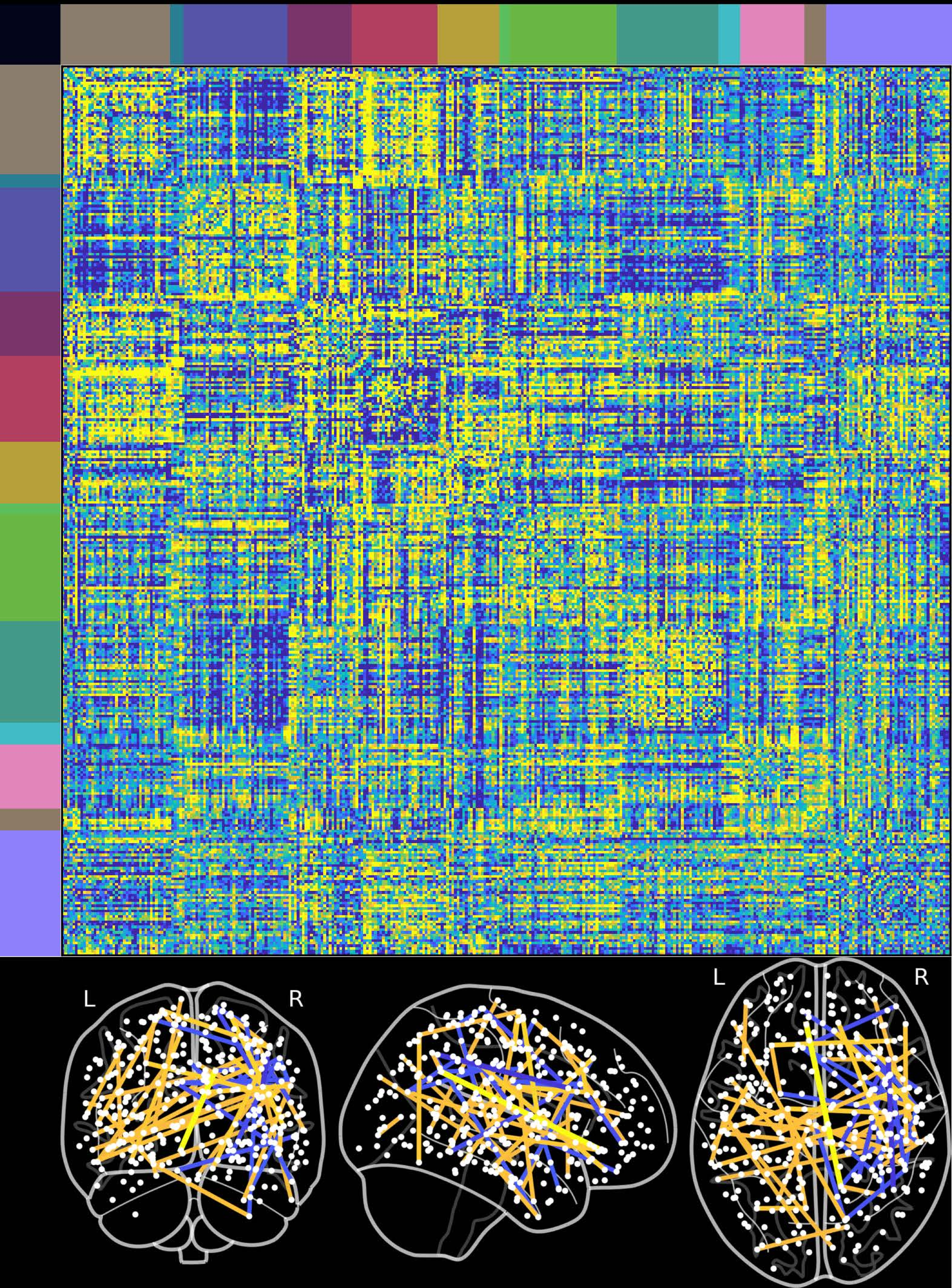

component_40

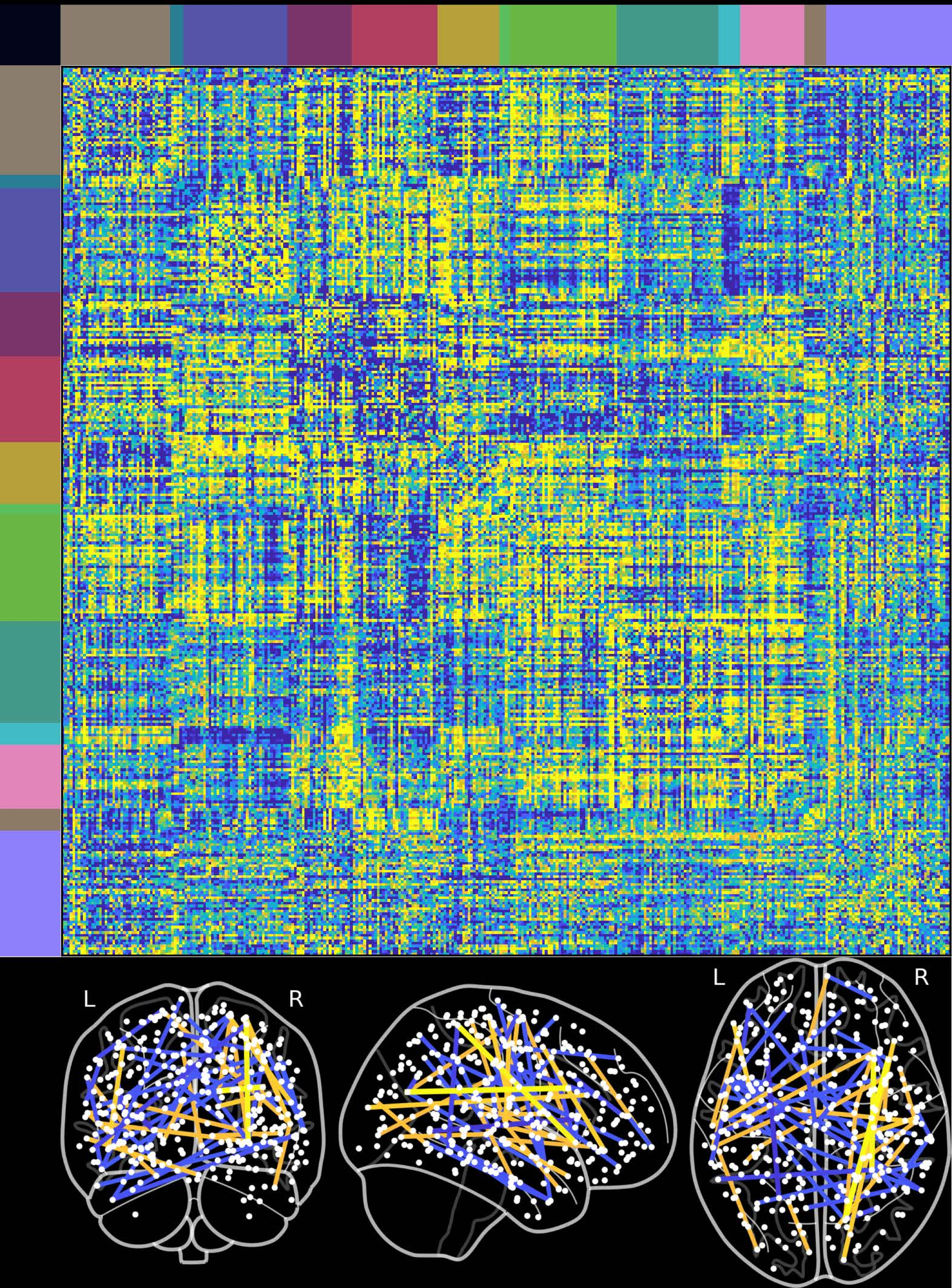

component_41

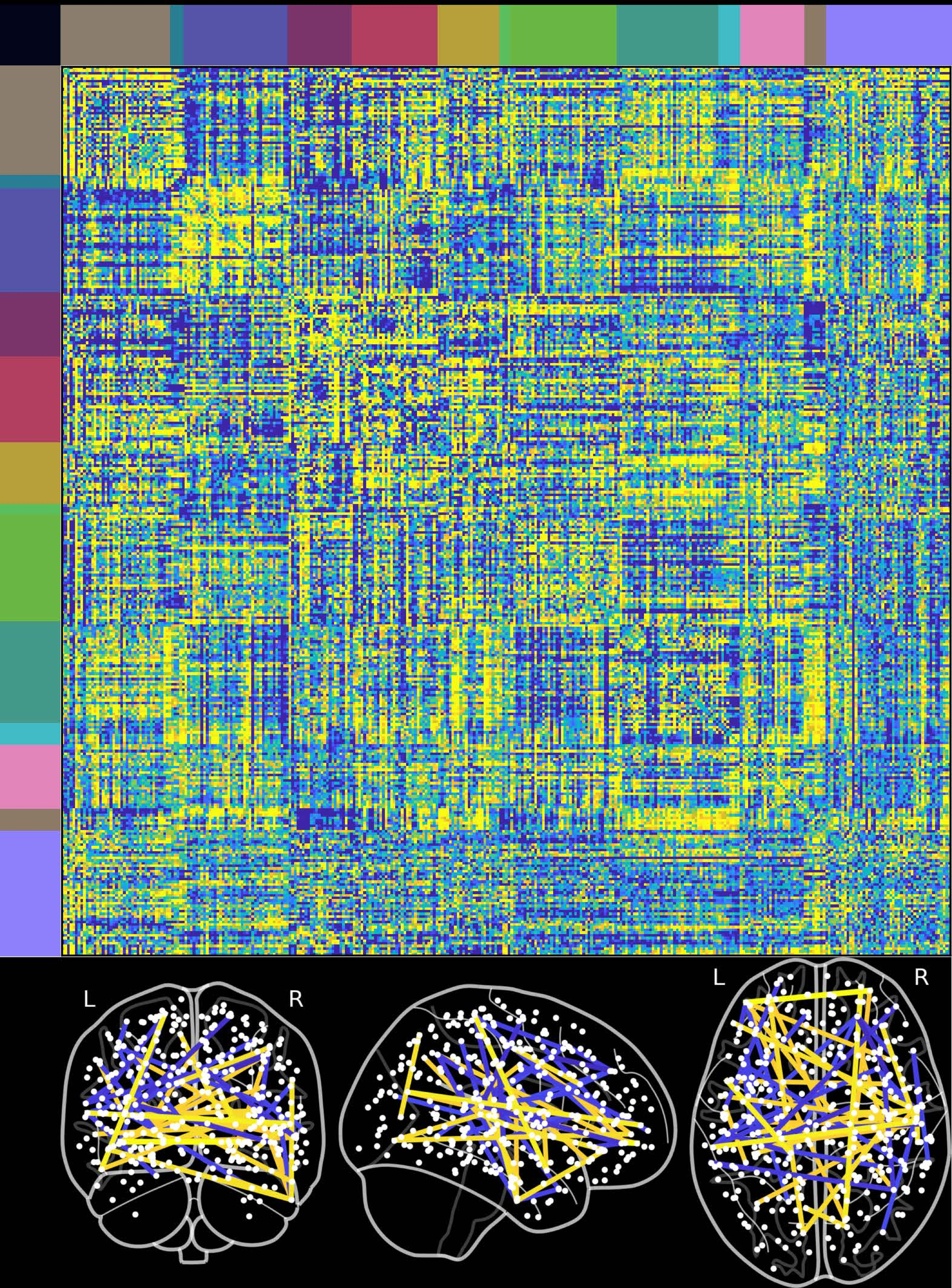

component_42

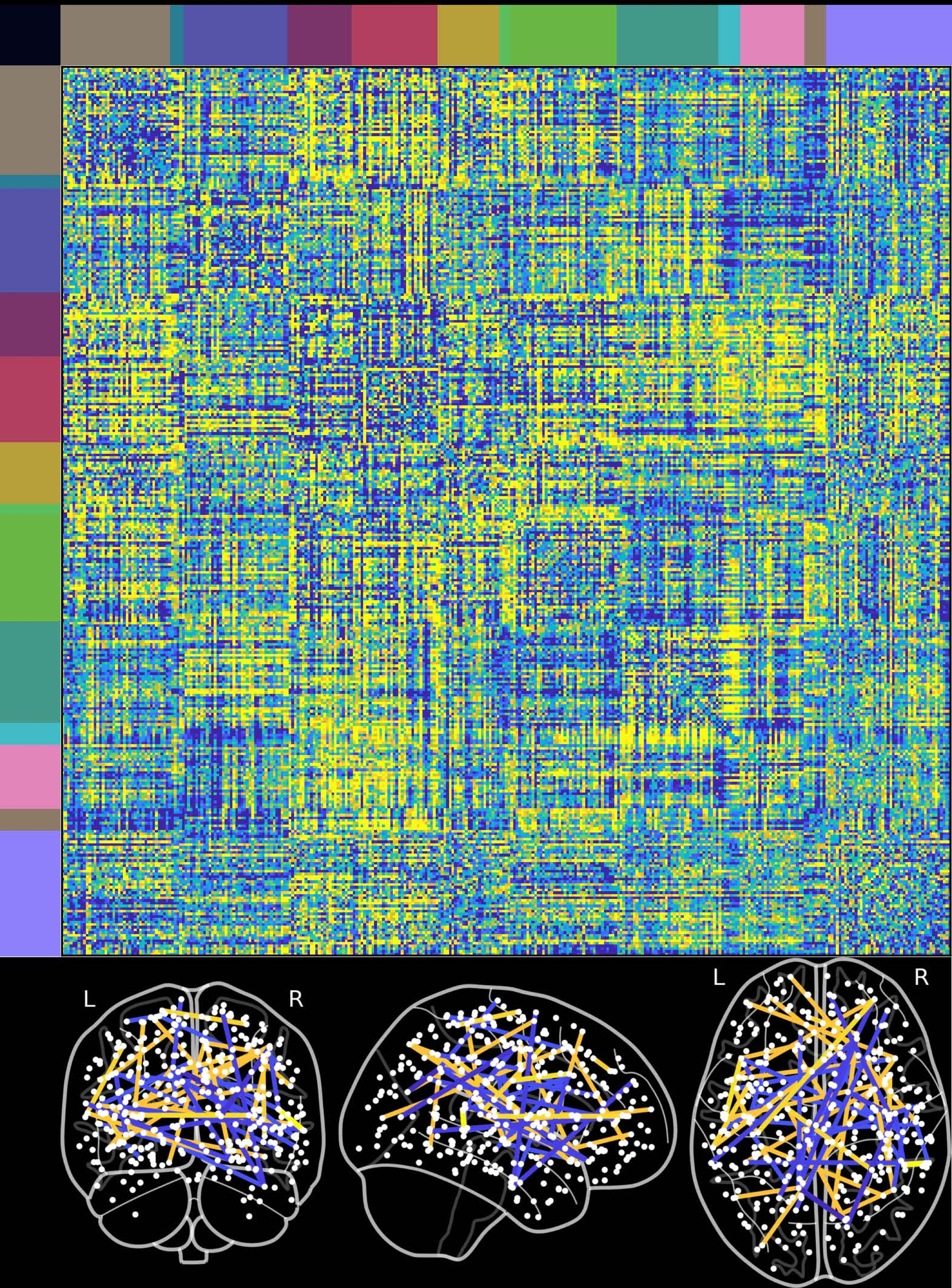

component_43

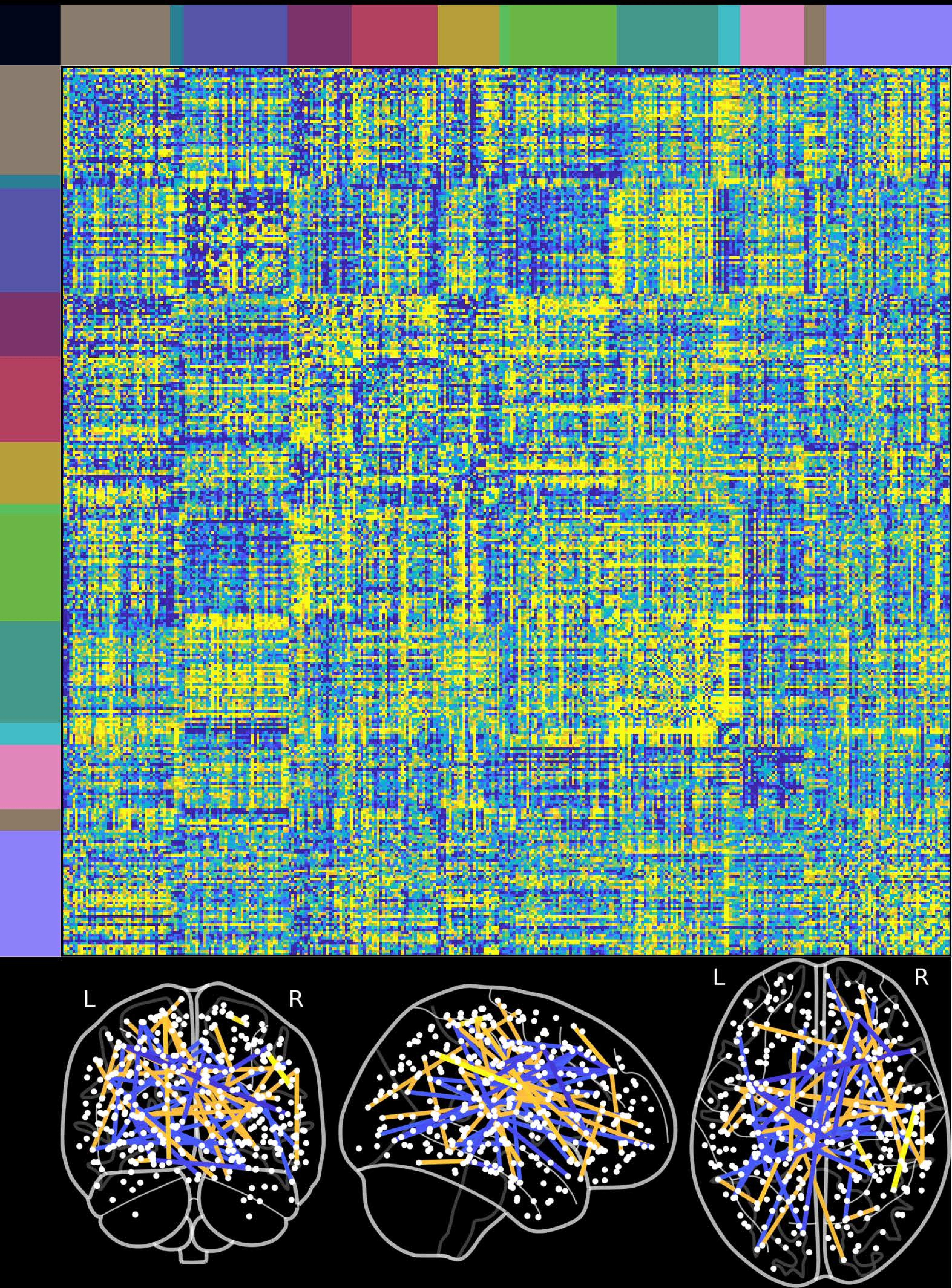

component_44

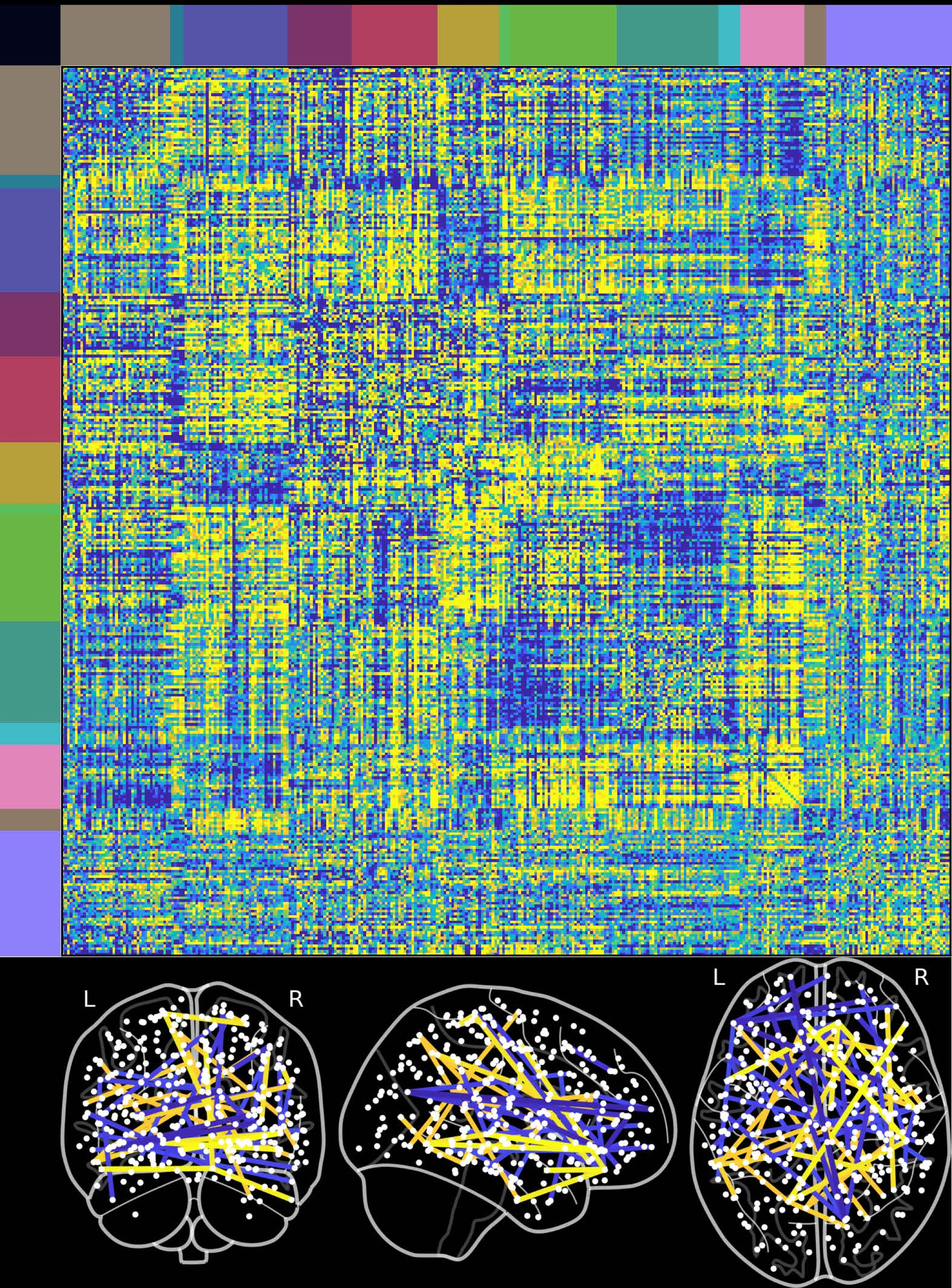

component_45

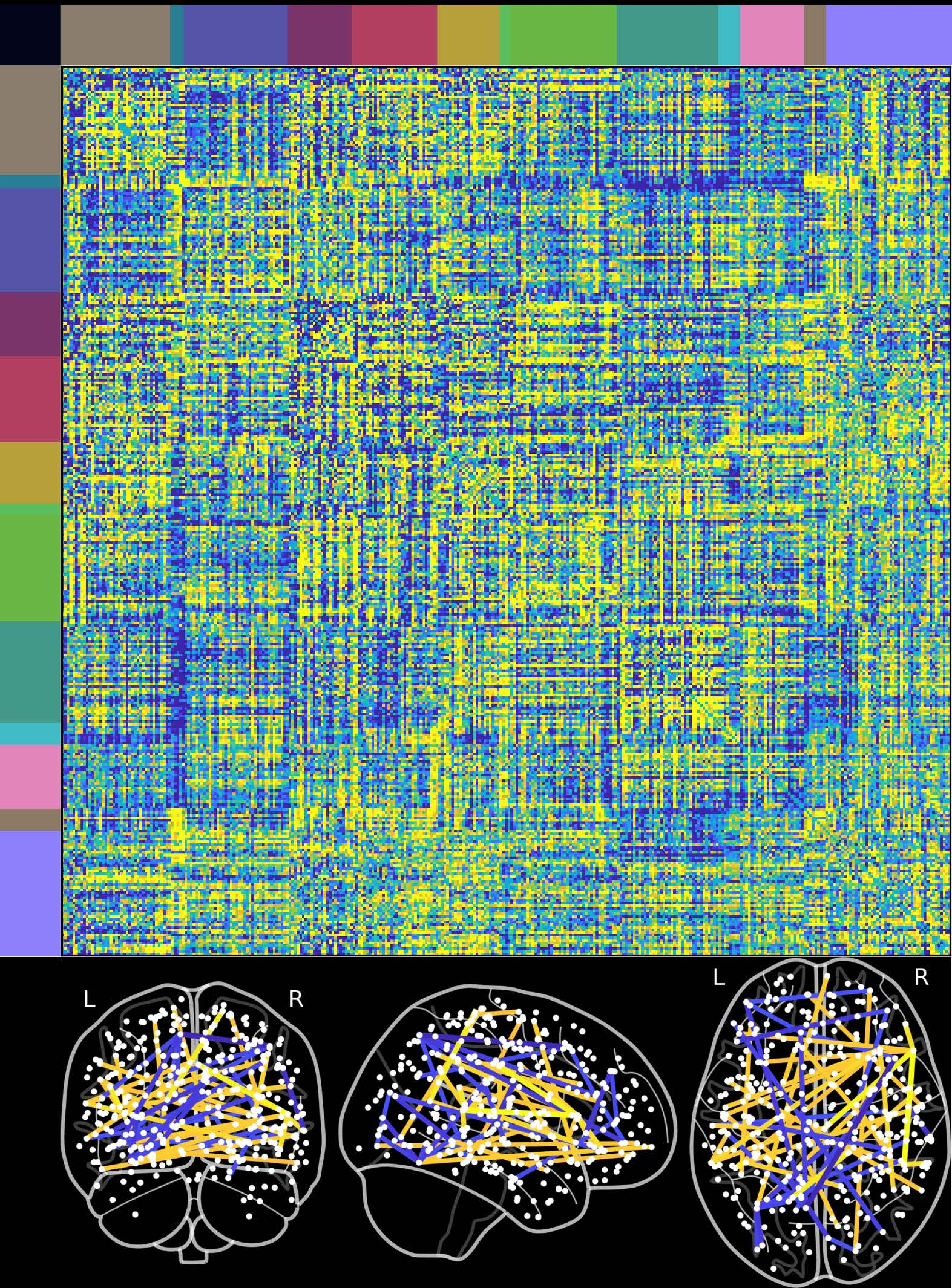

component_46

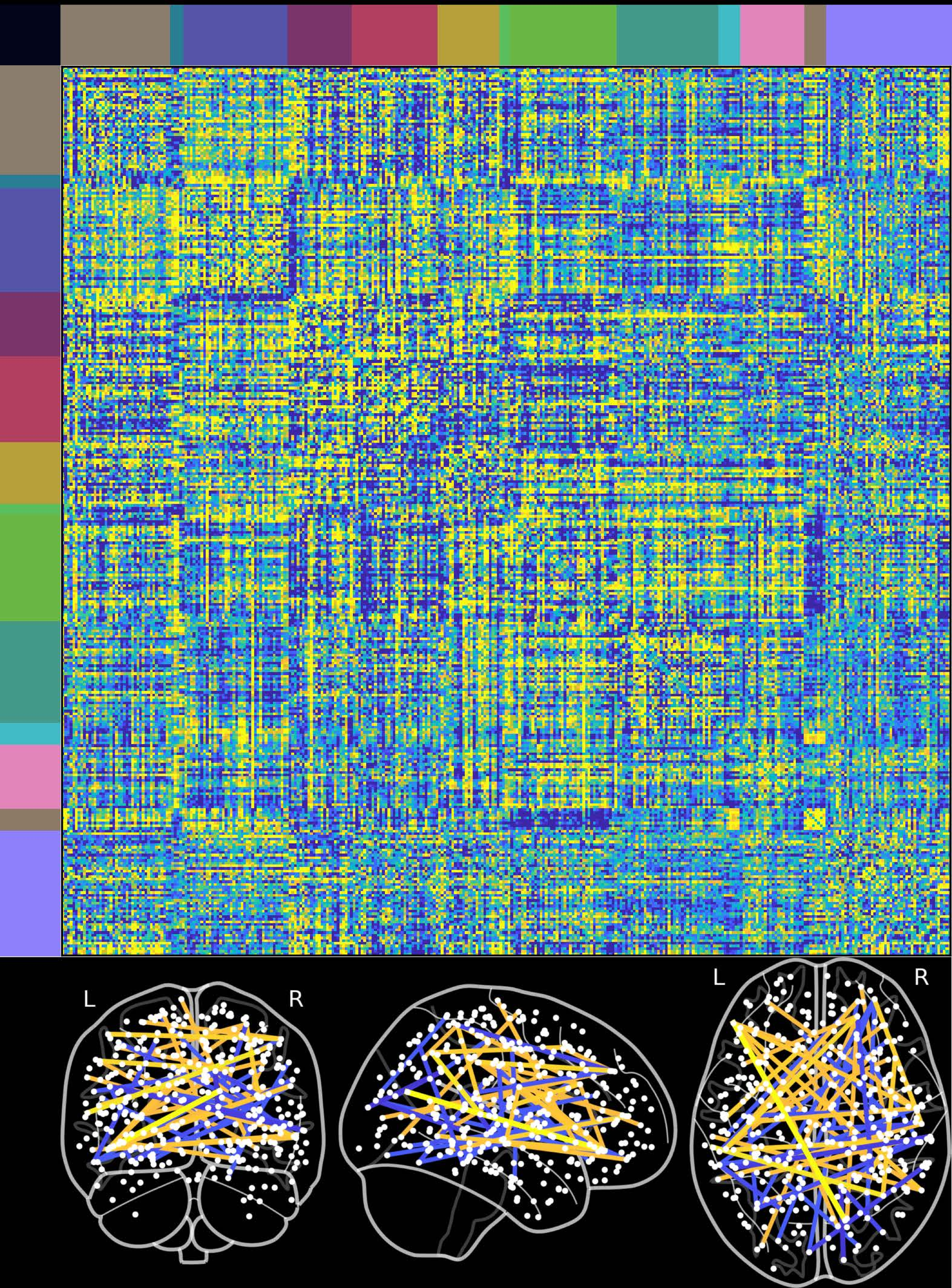

component_47

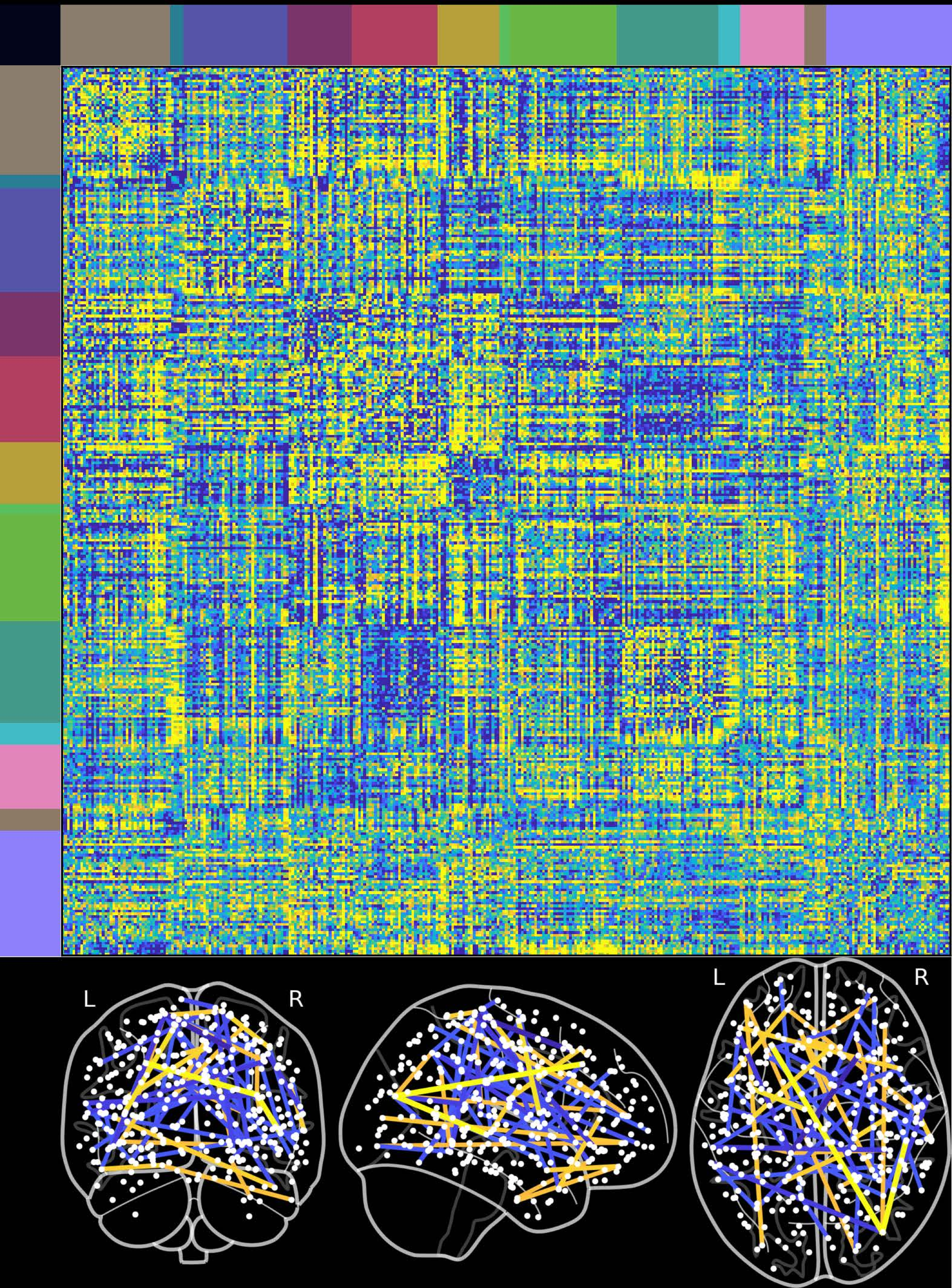

component_48

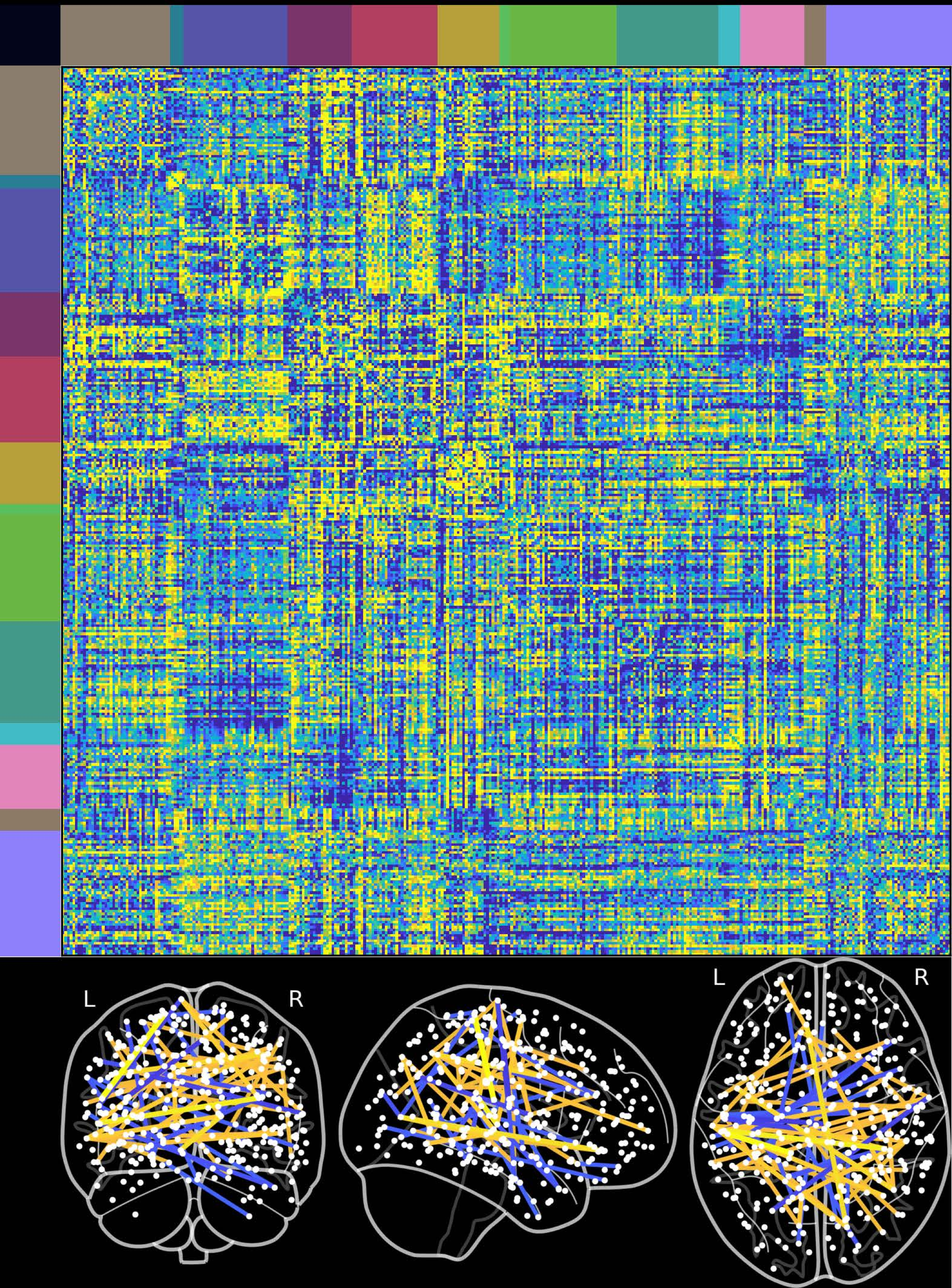

component_49

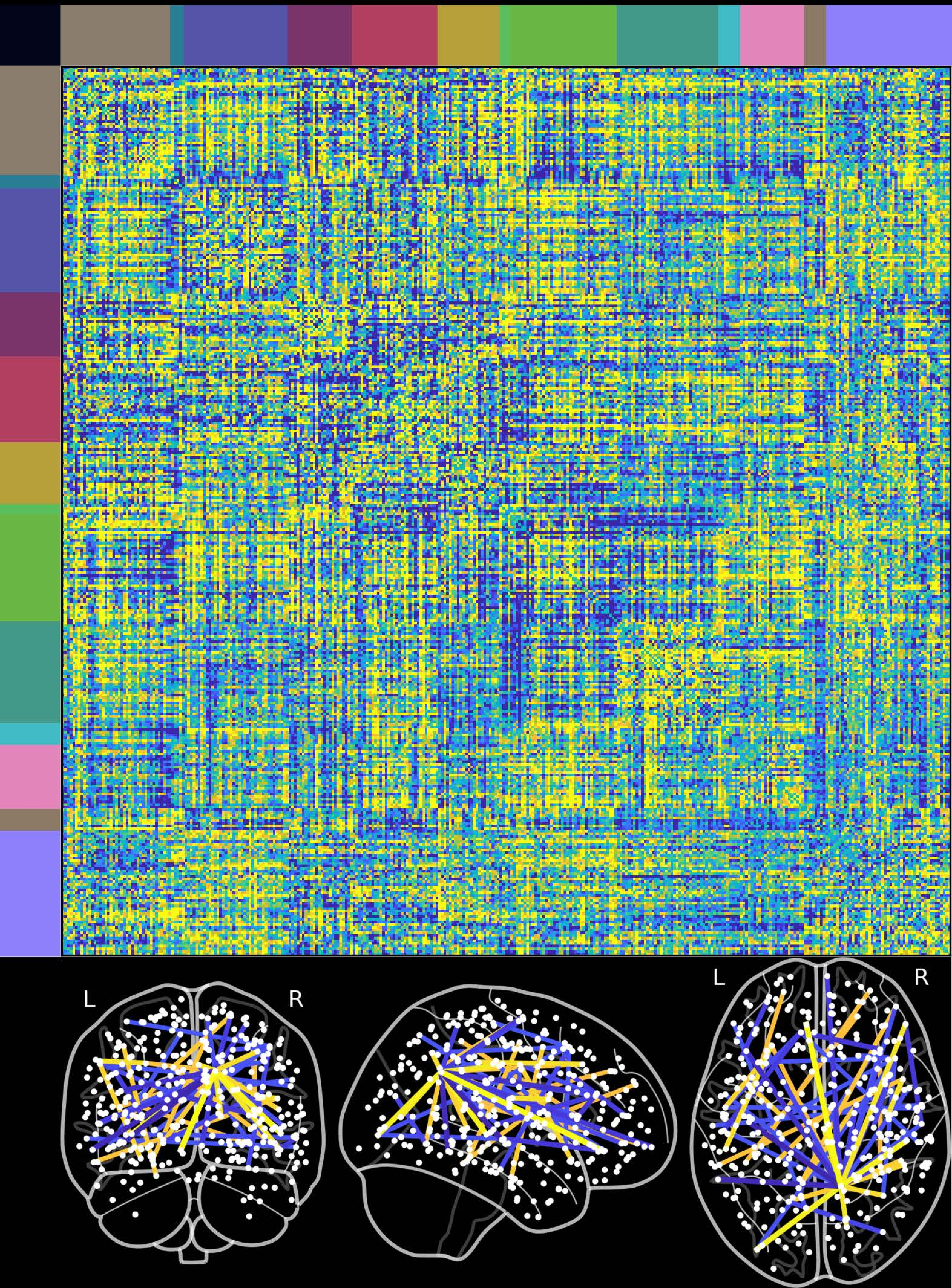

component_50

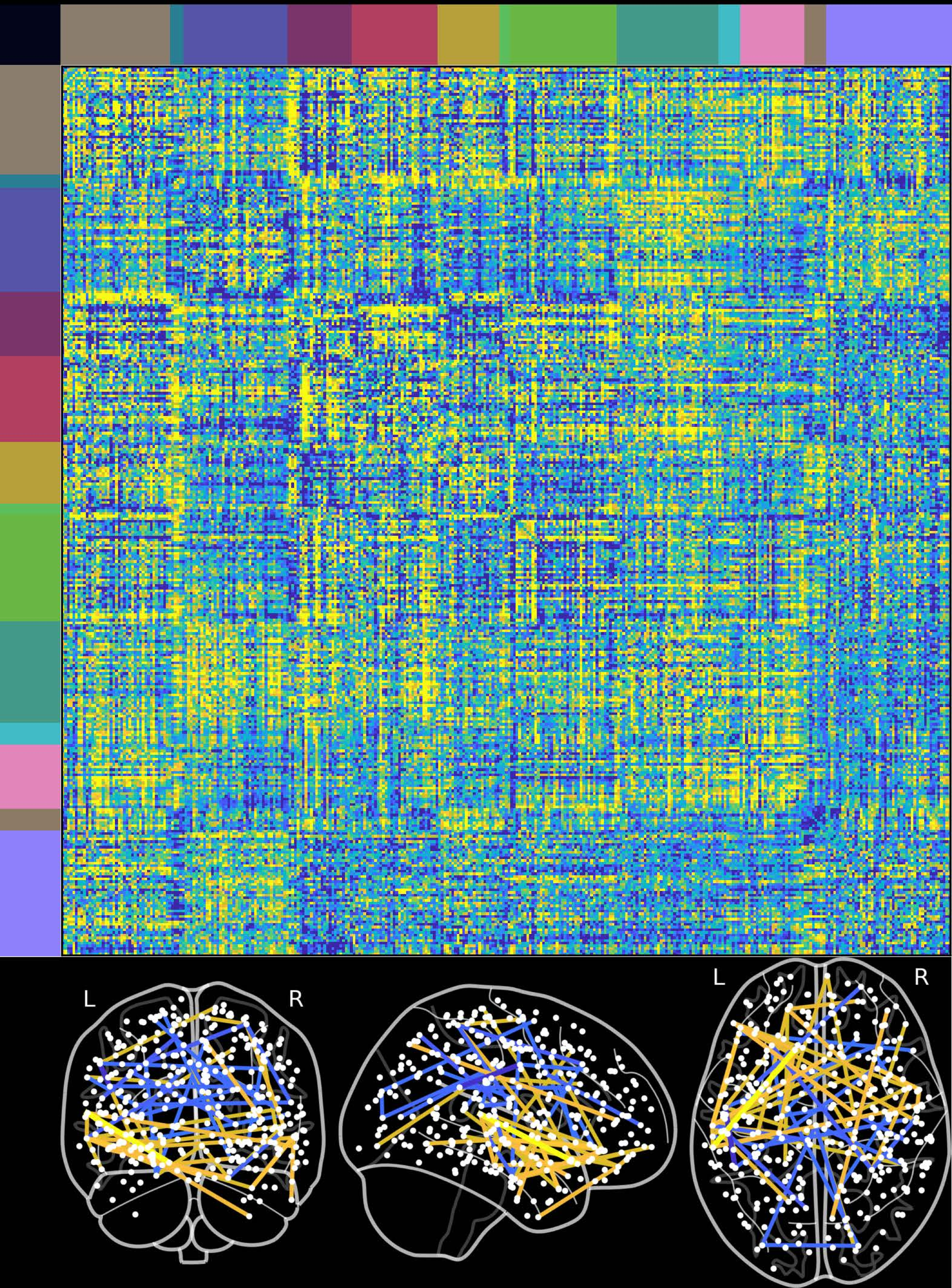

component_51

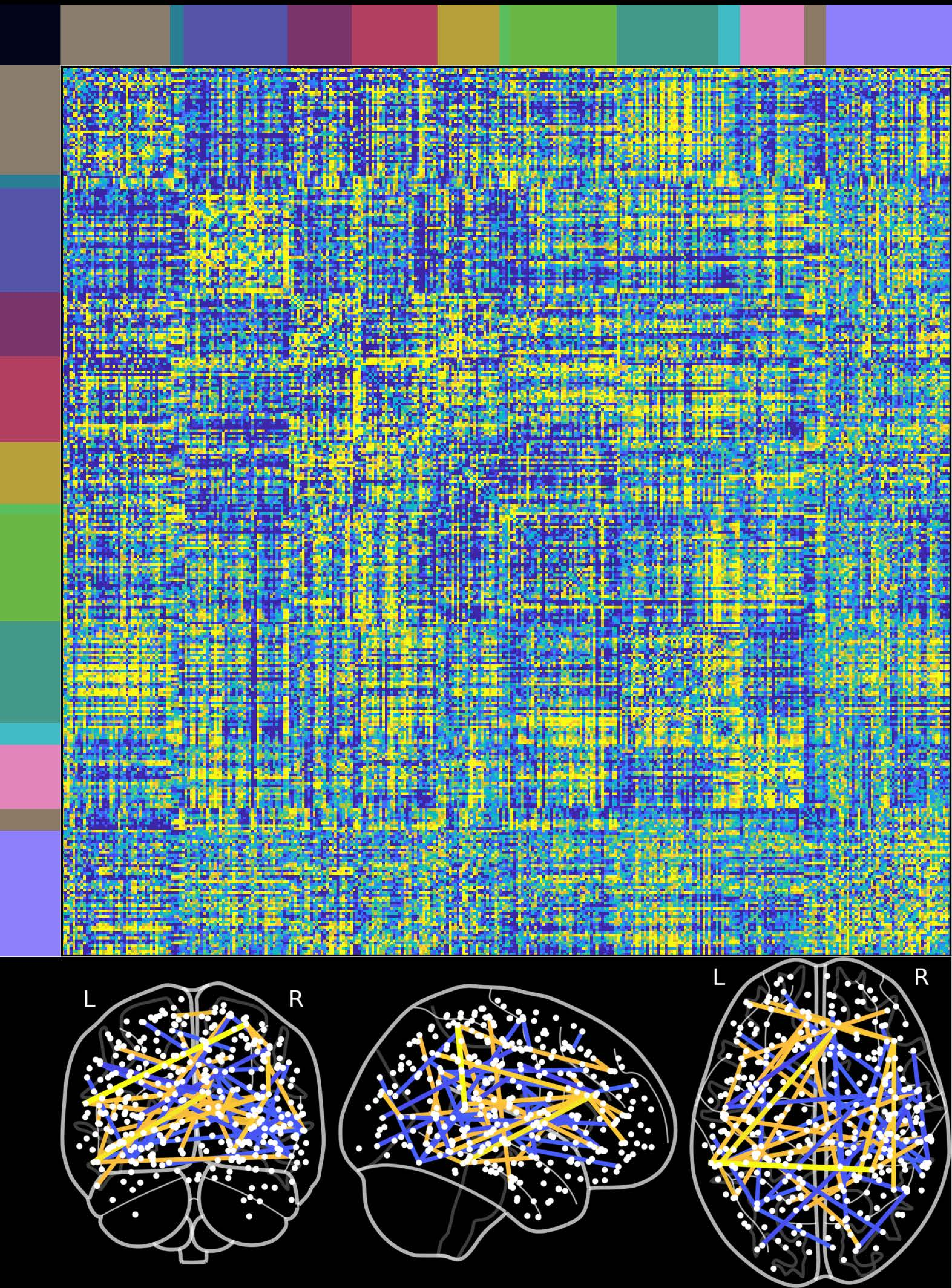

component_52

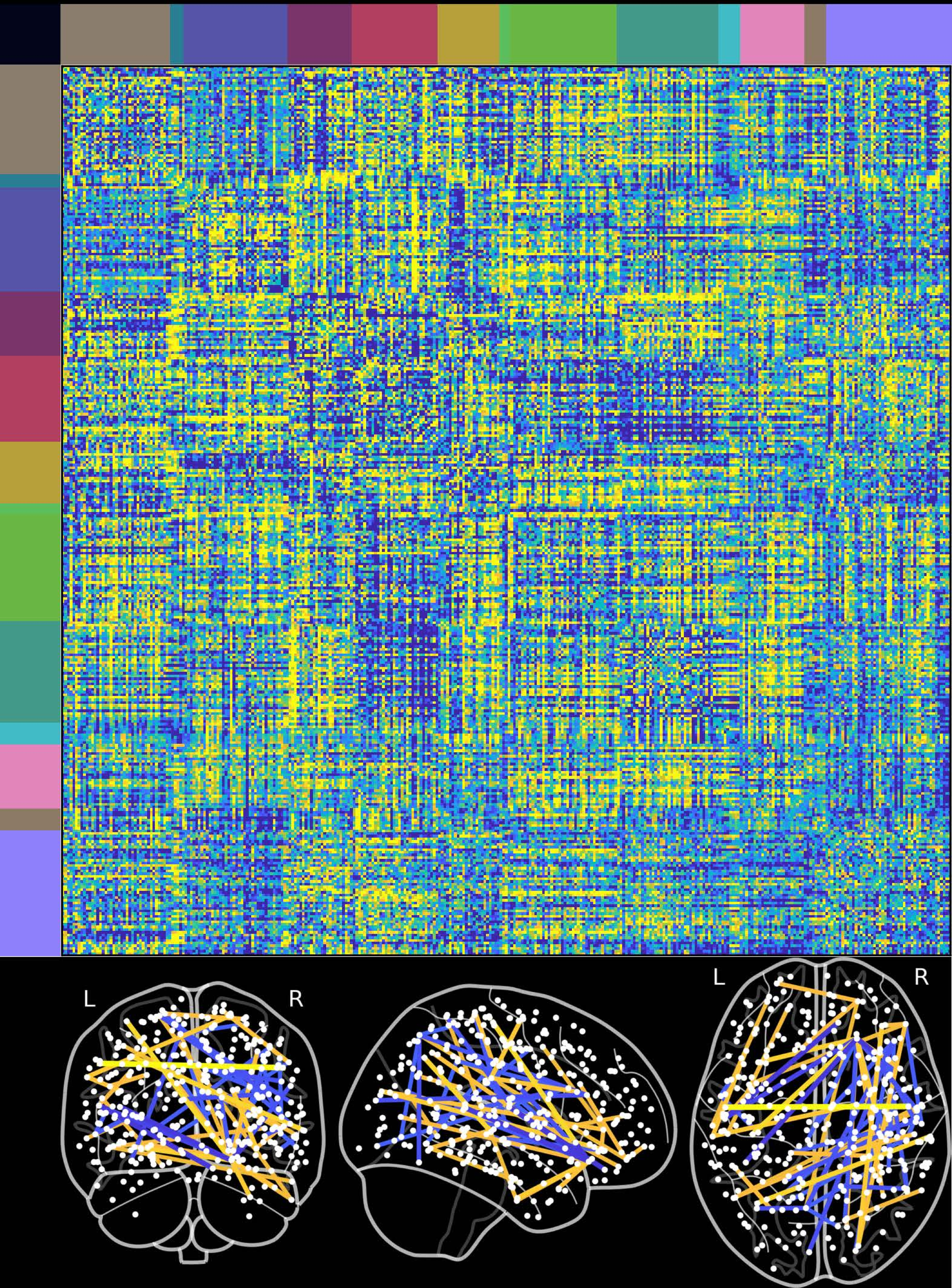

component_53

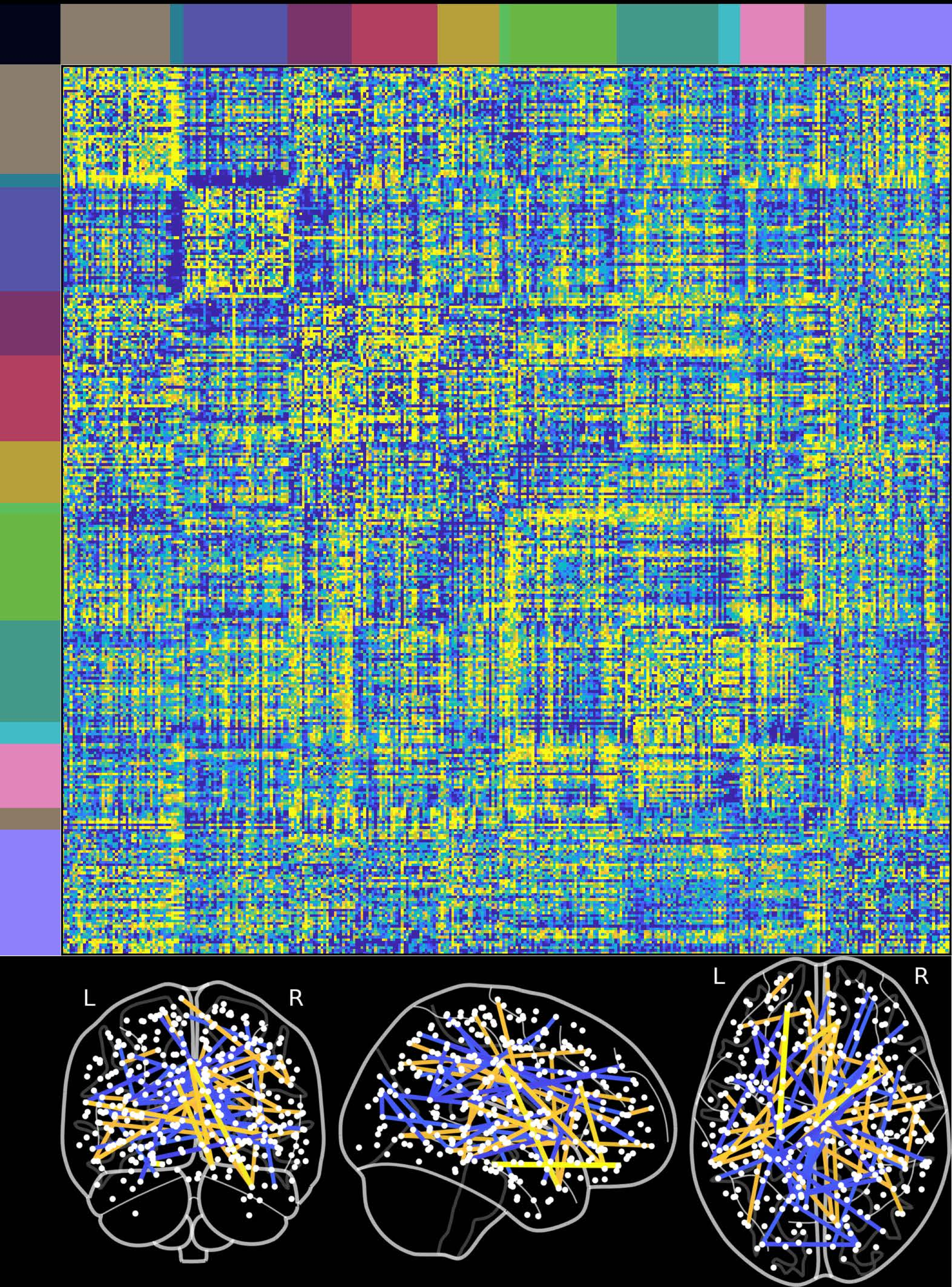

component_54

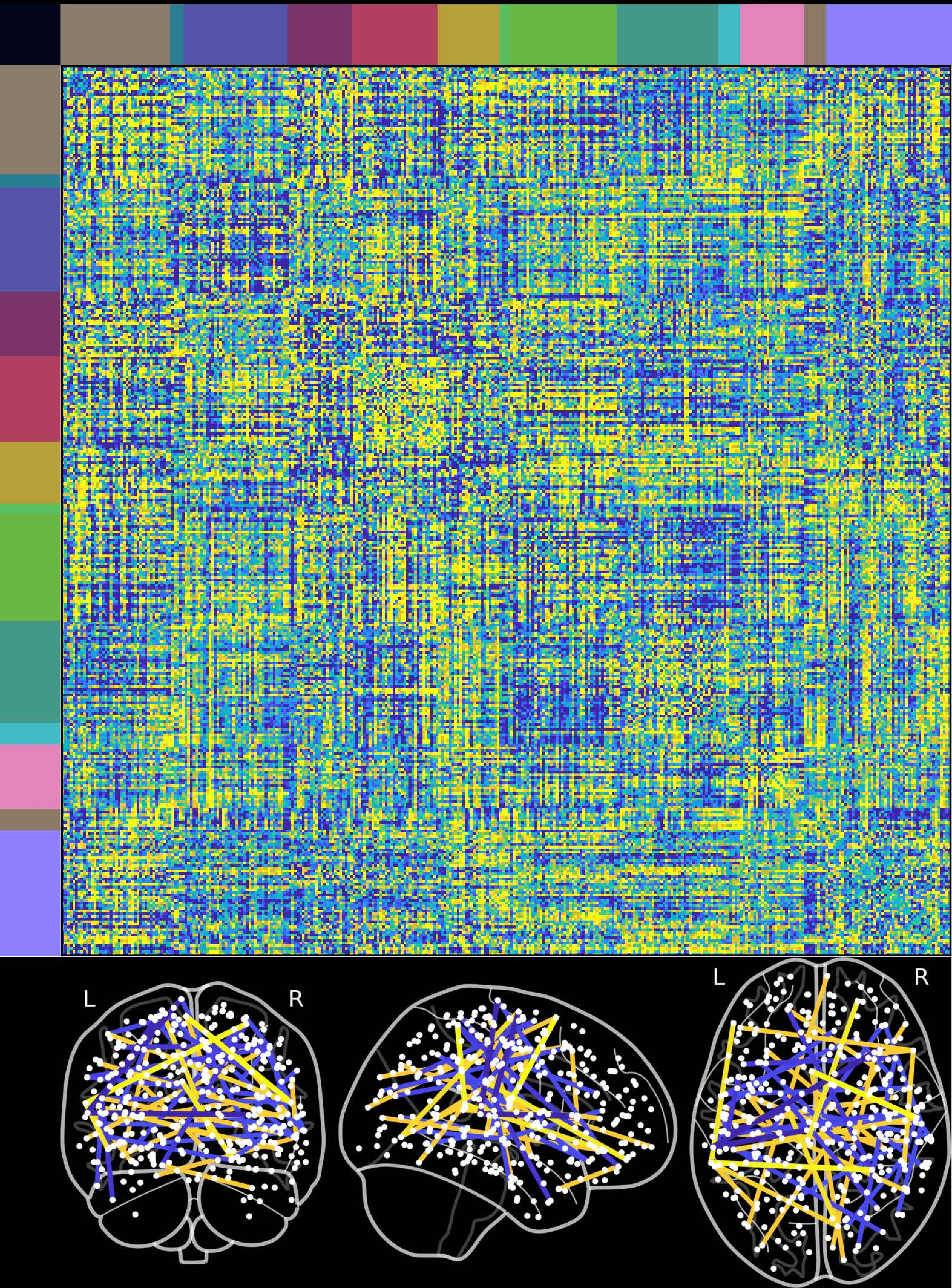

component_55

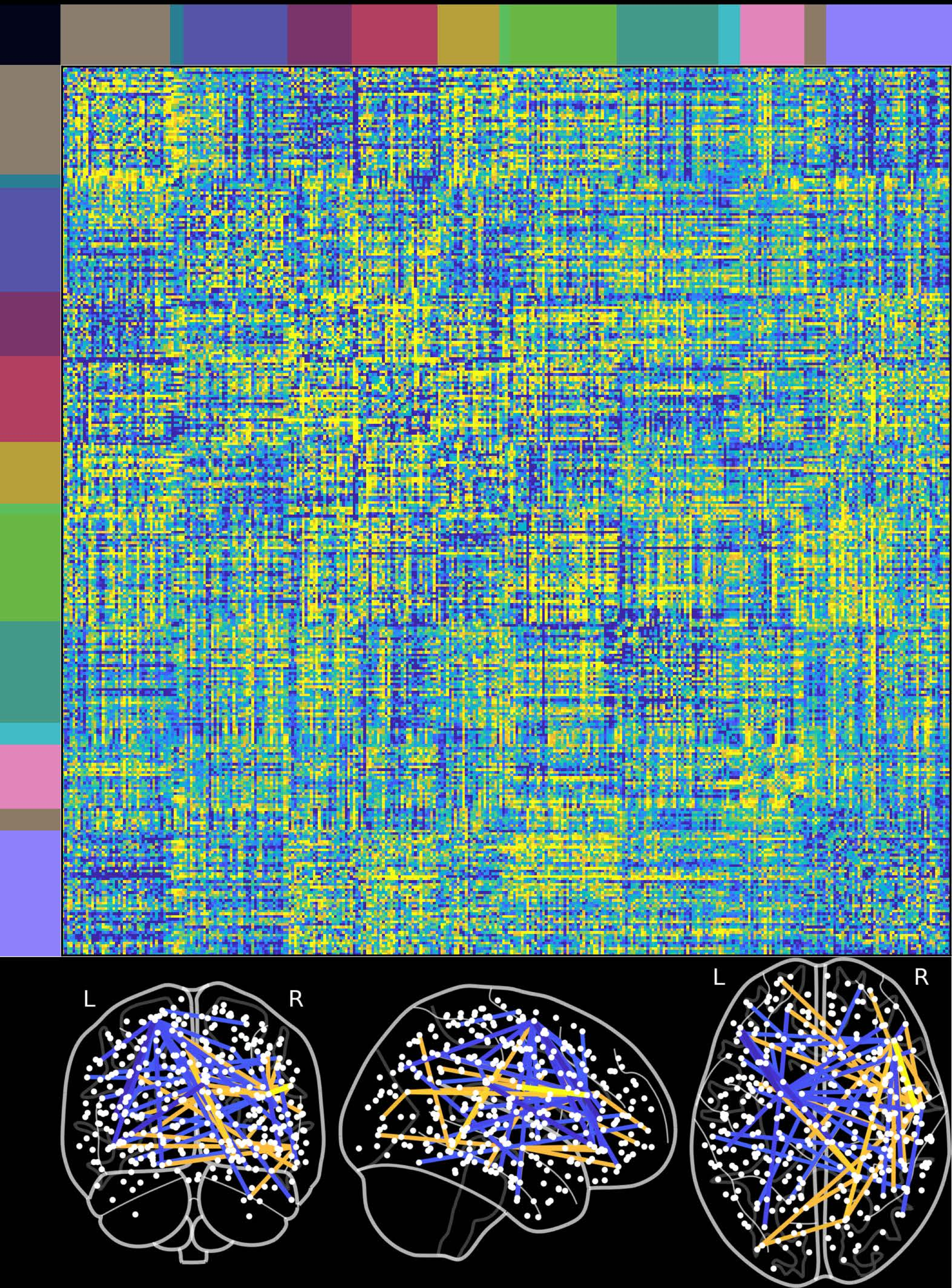

component_56

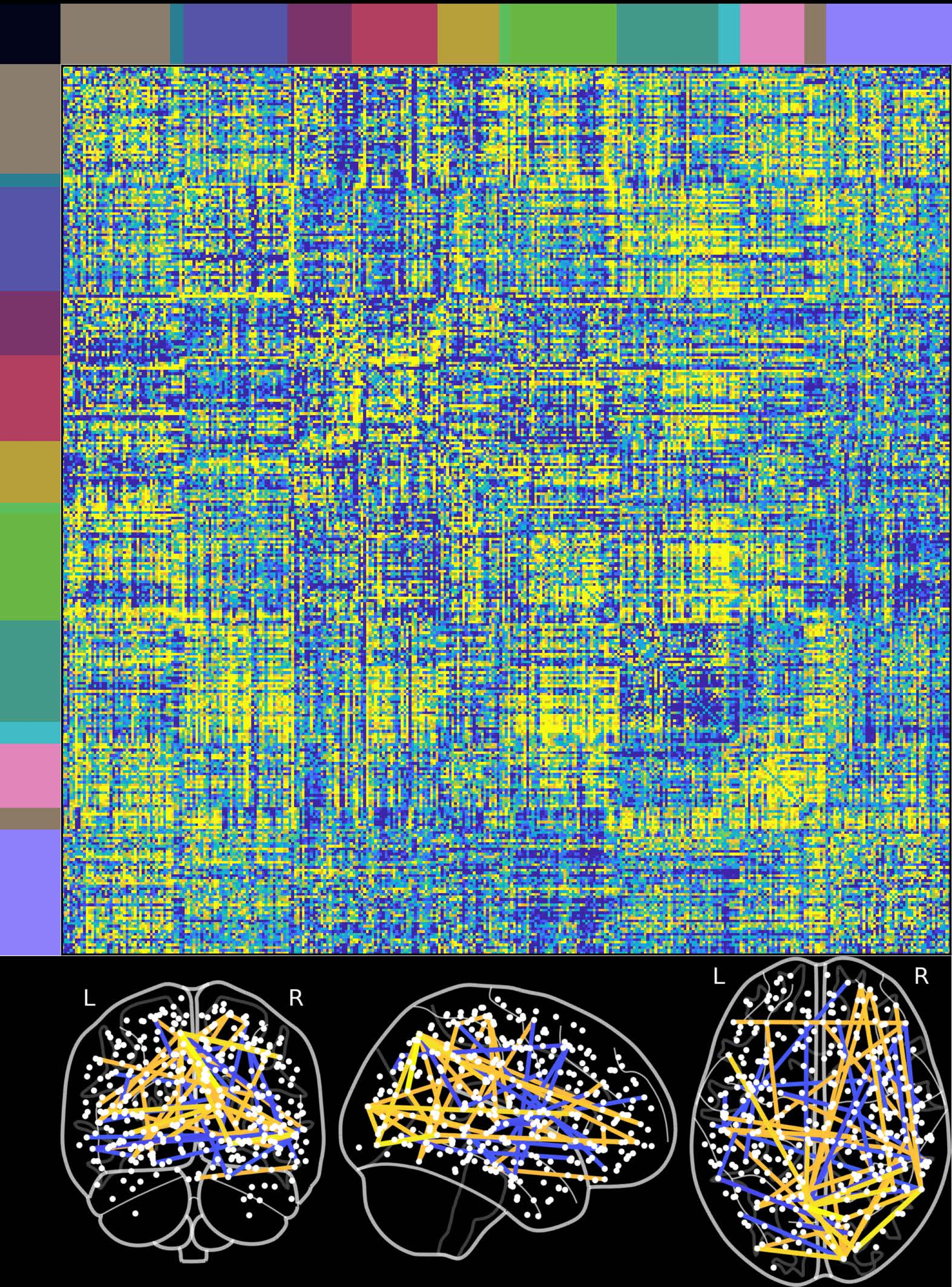

component_57

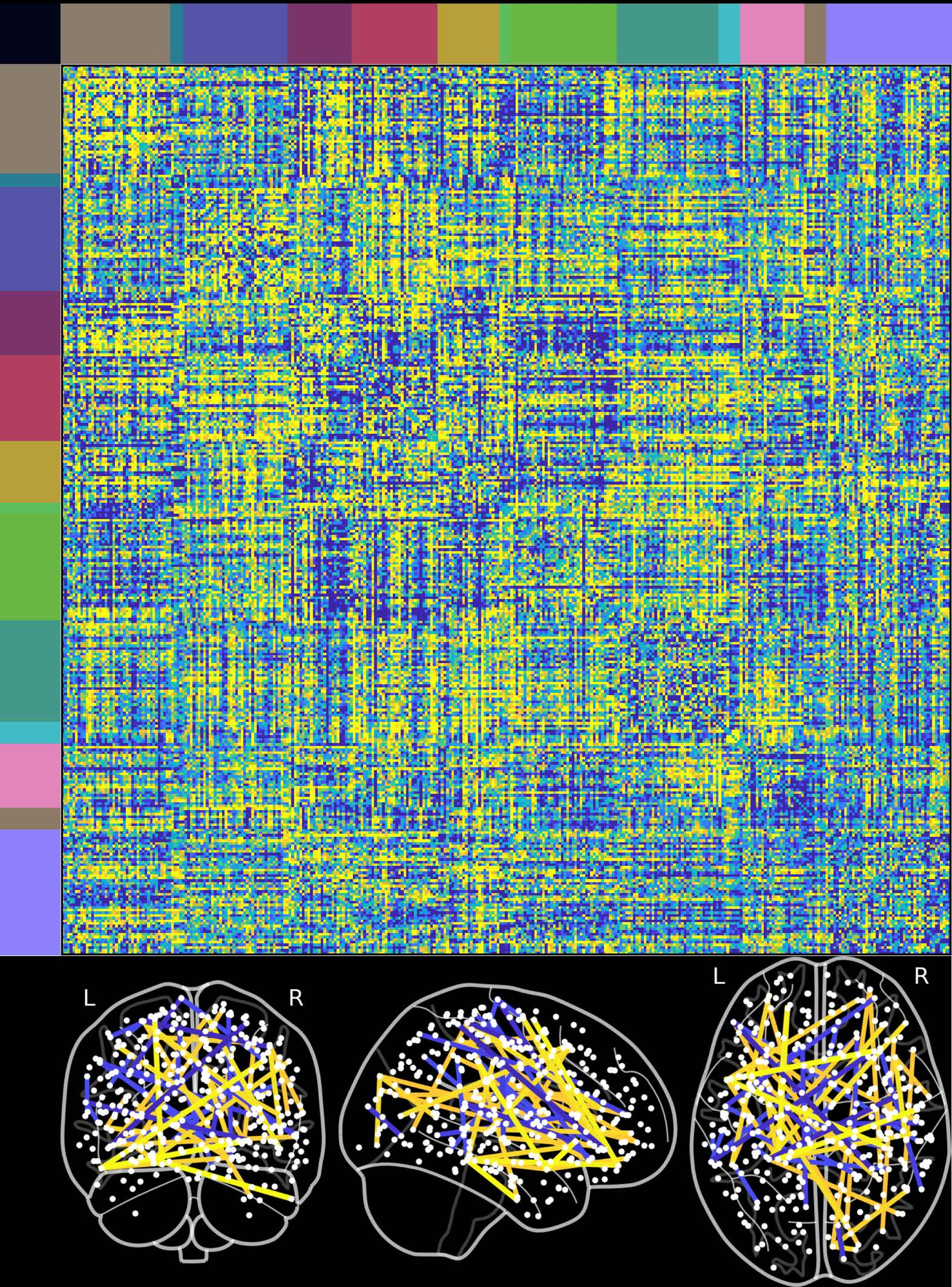

component_58

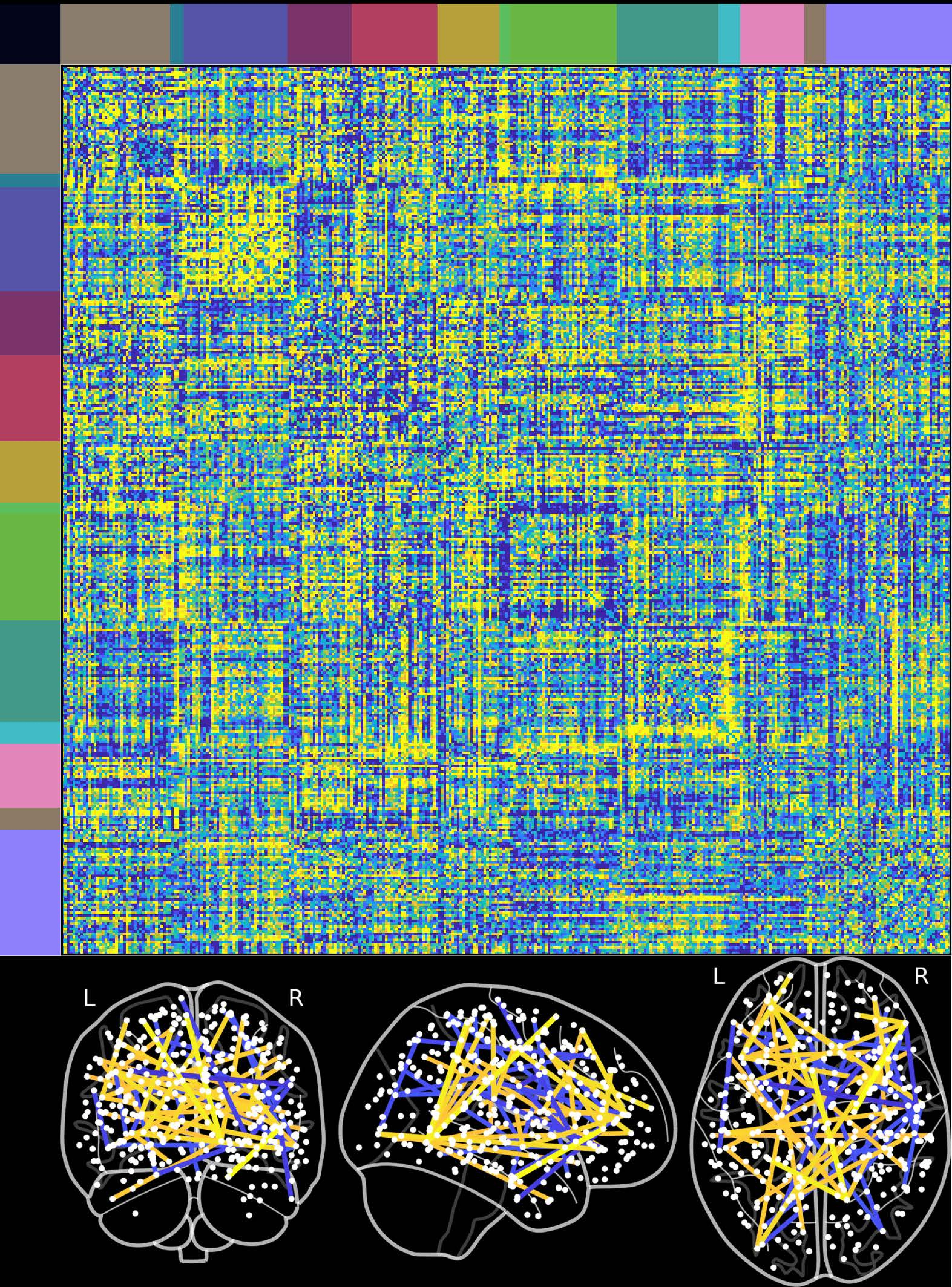

component_59

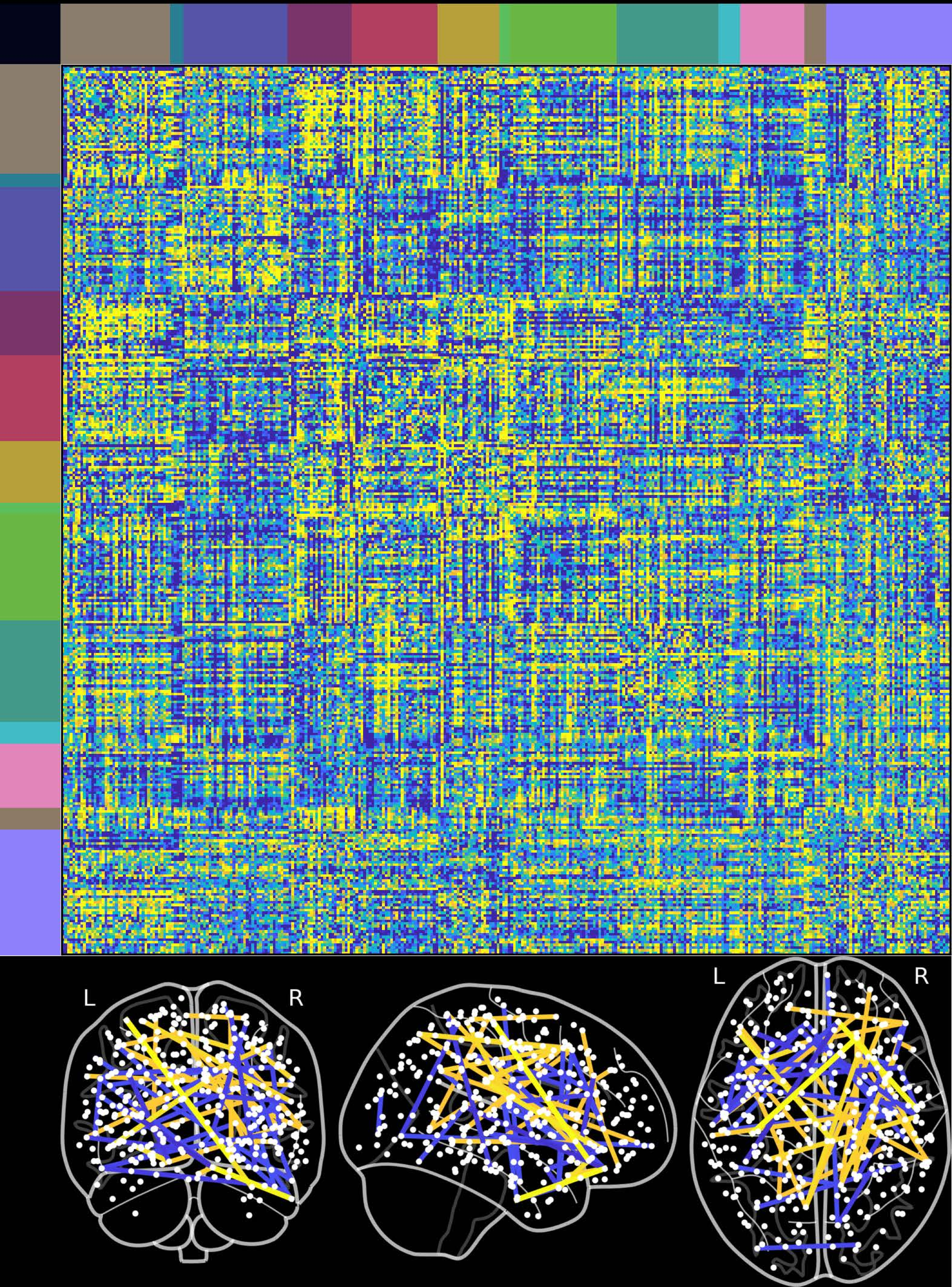

component_60

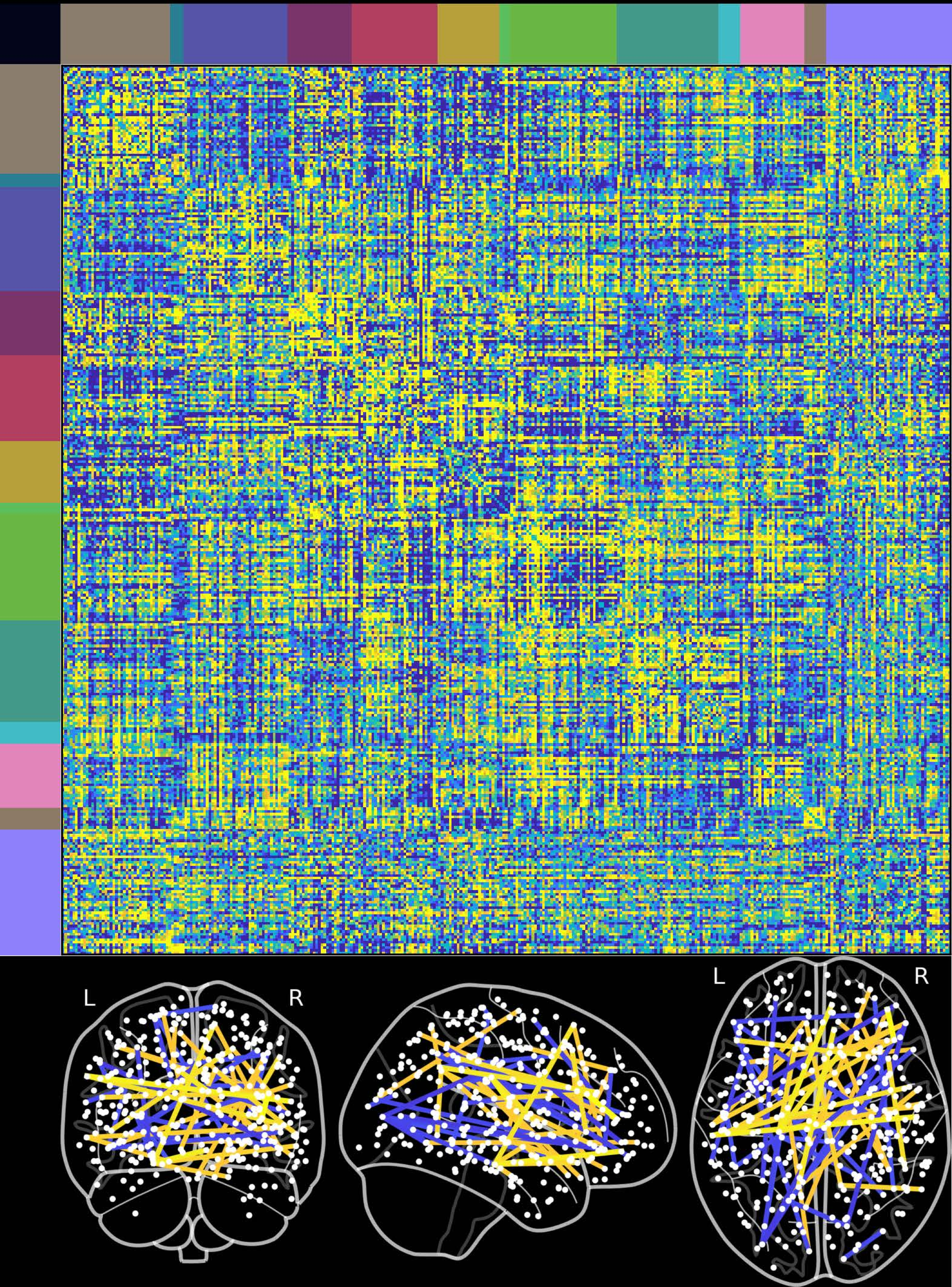

component_61

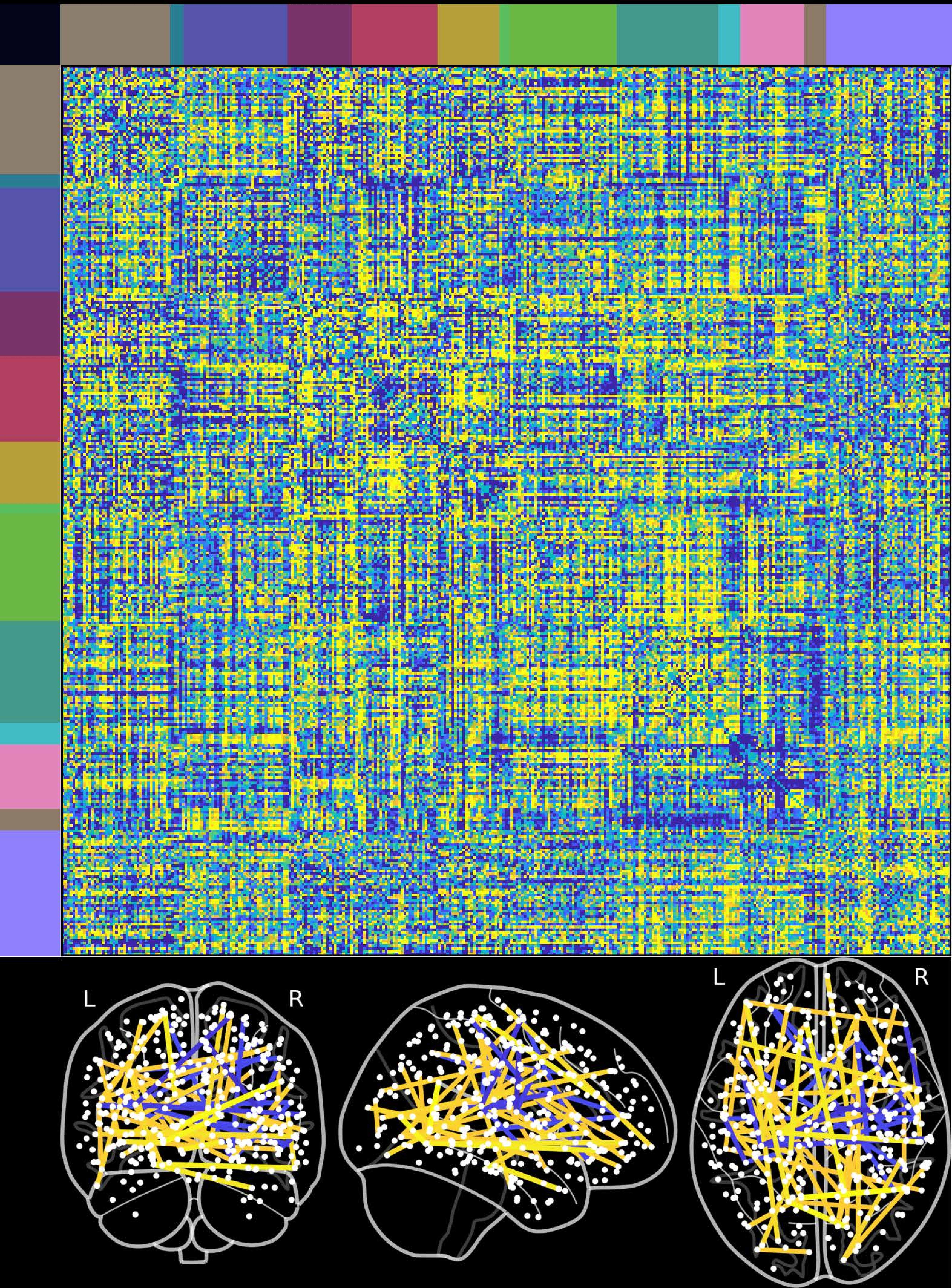

component_62

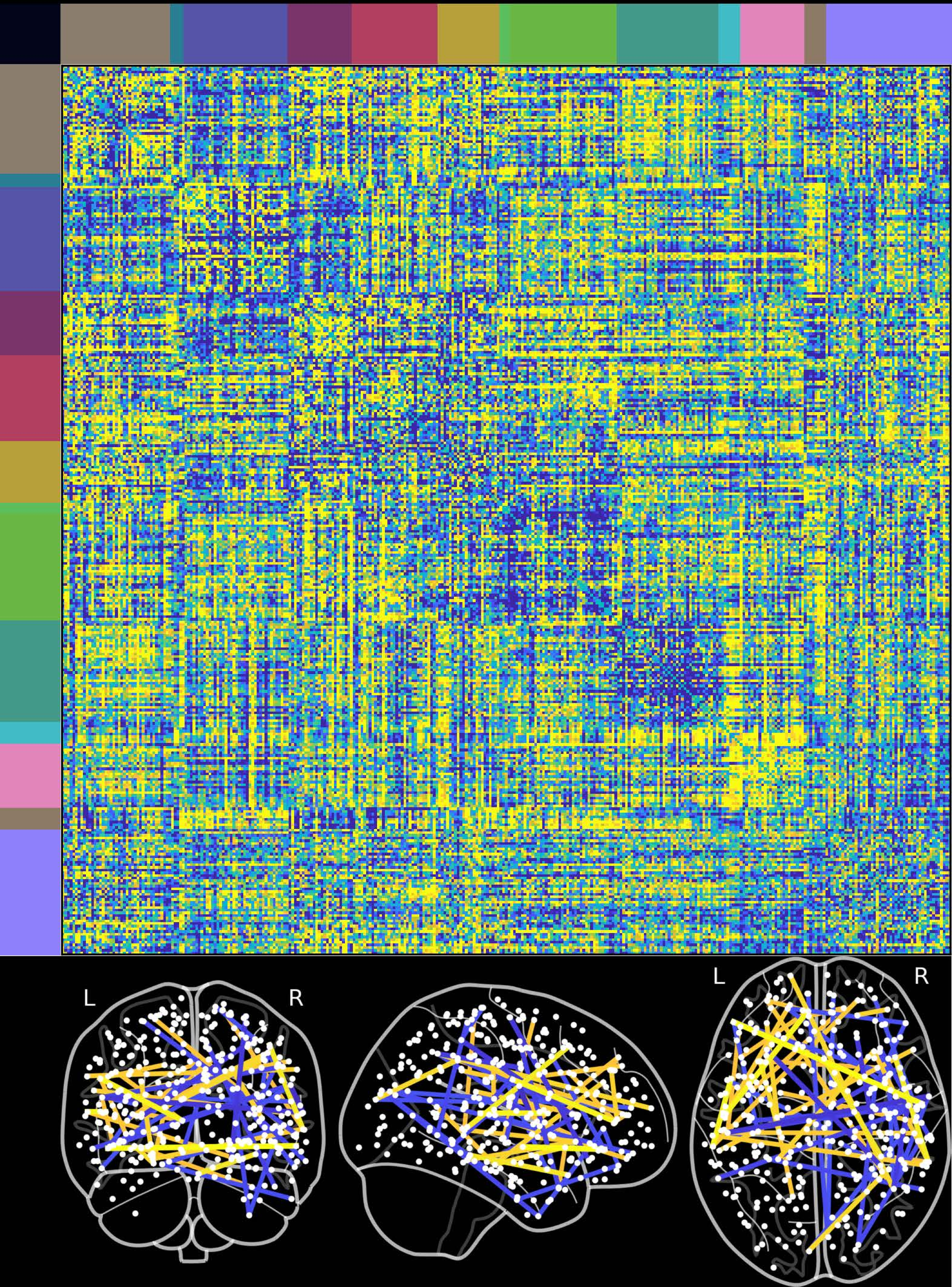

component_63

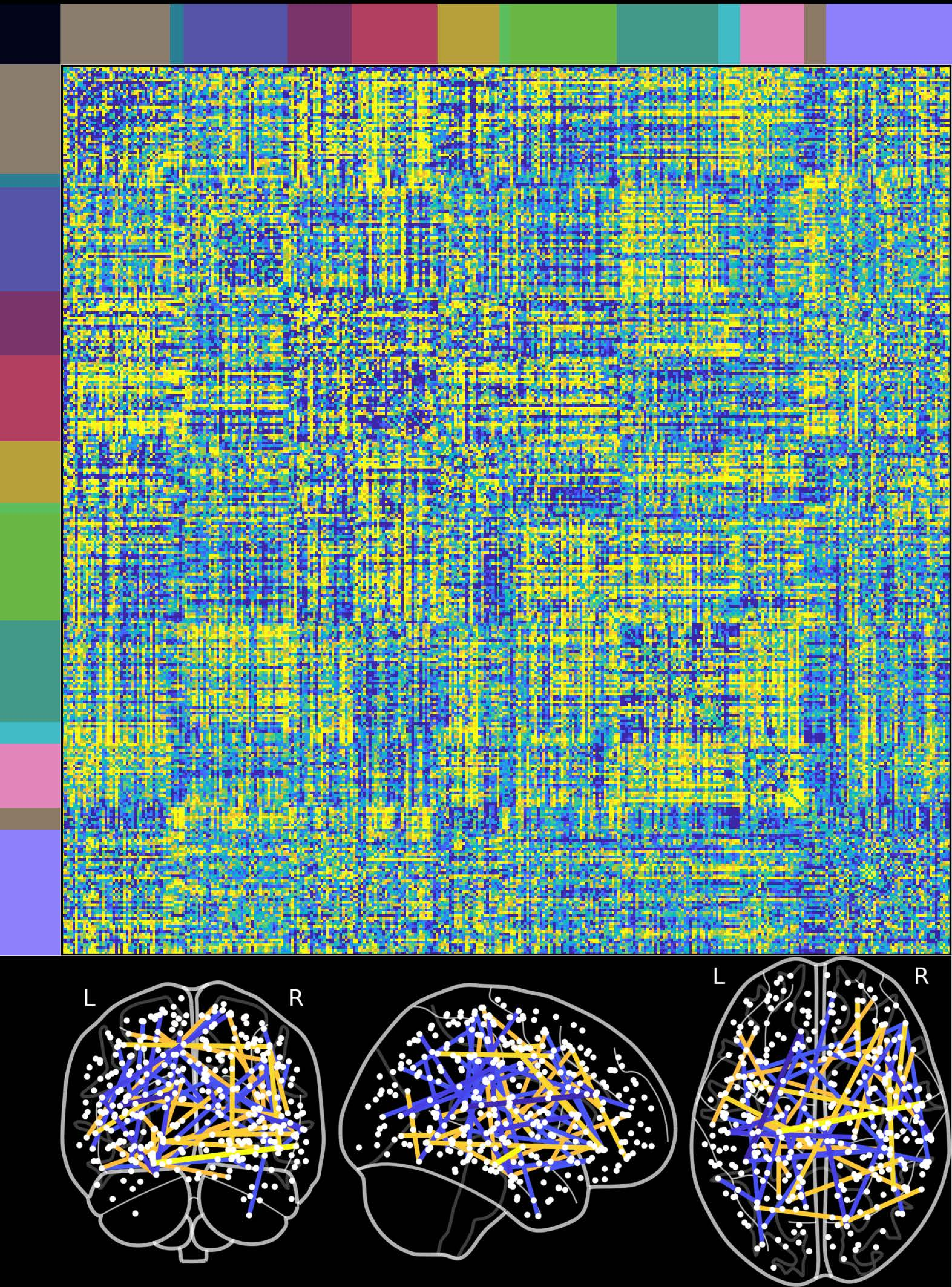

component_64

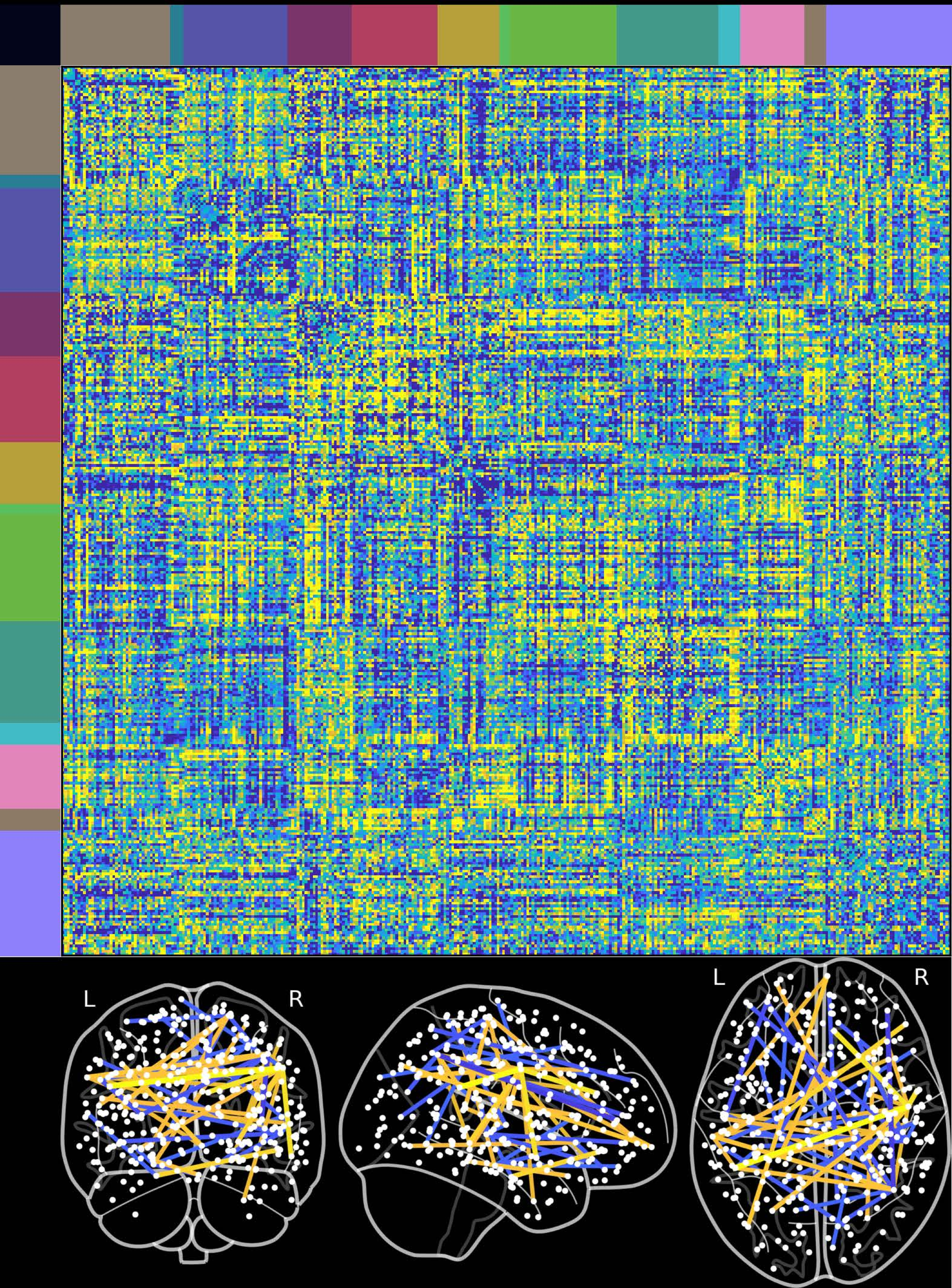

component_65

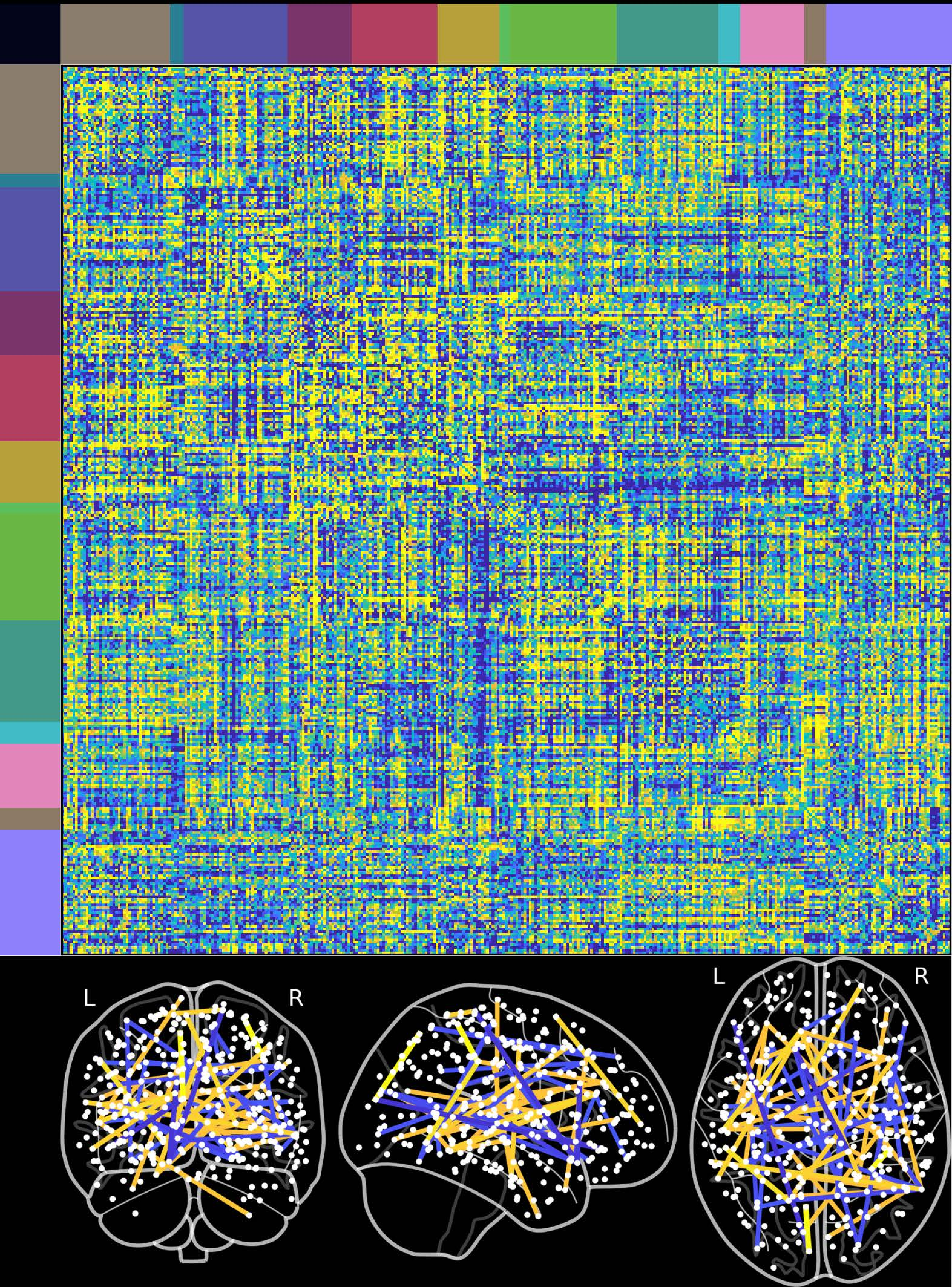

component_66

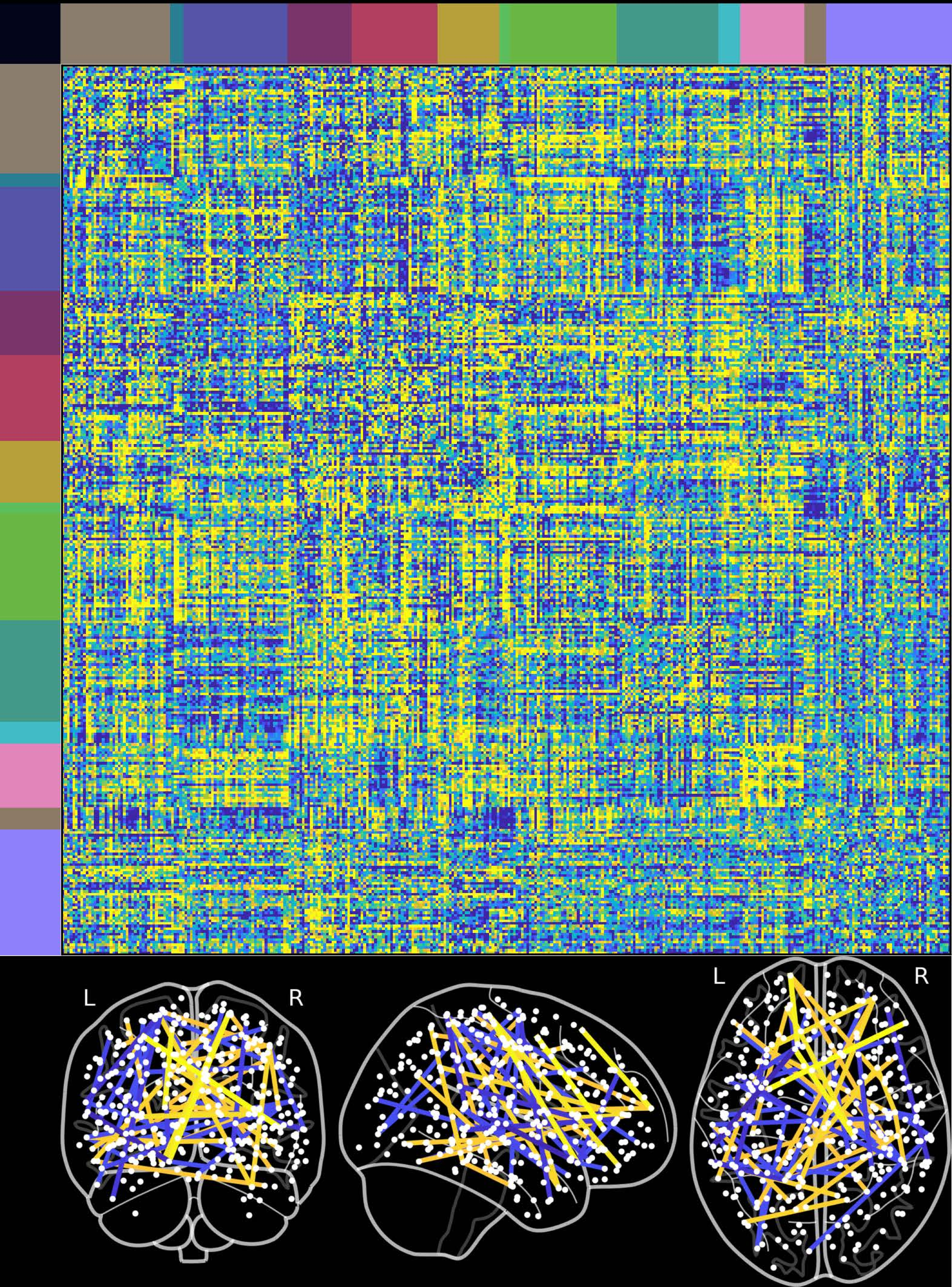

component_67

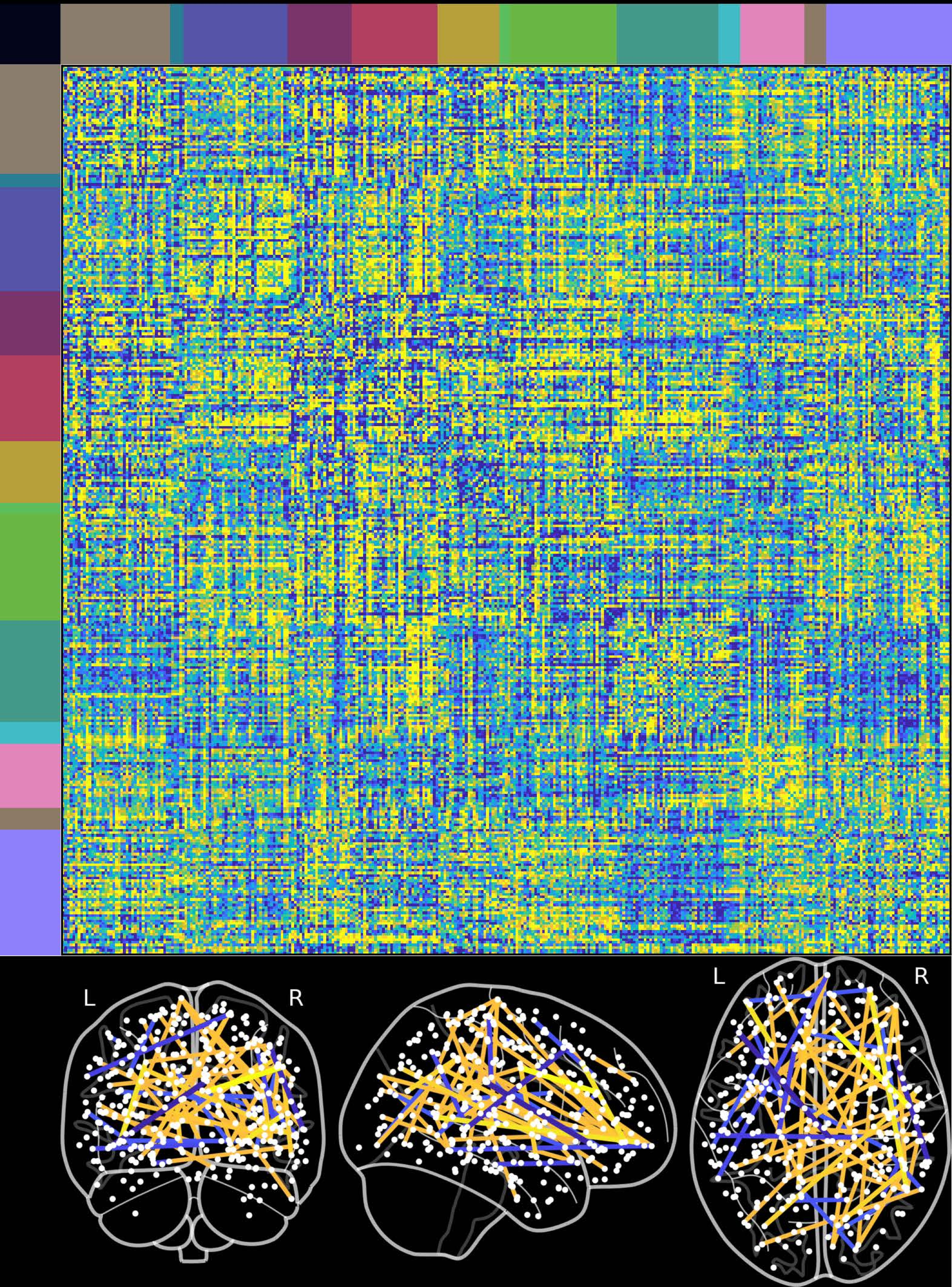

component_68

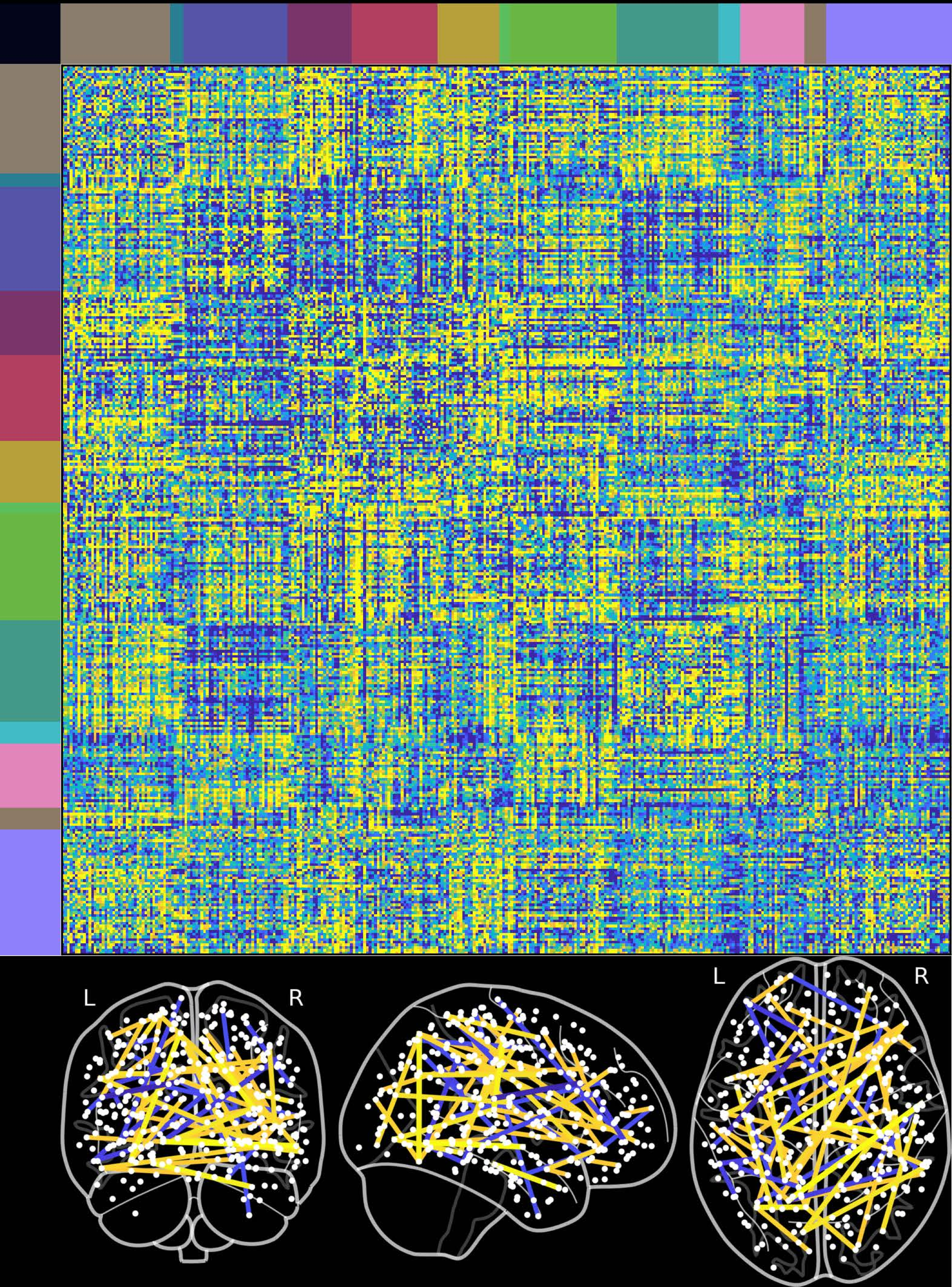

component_69

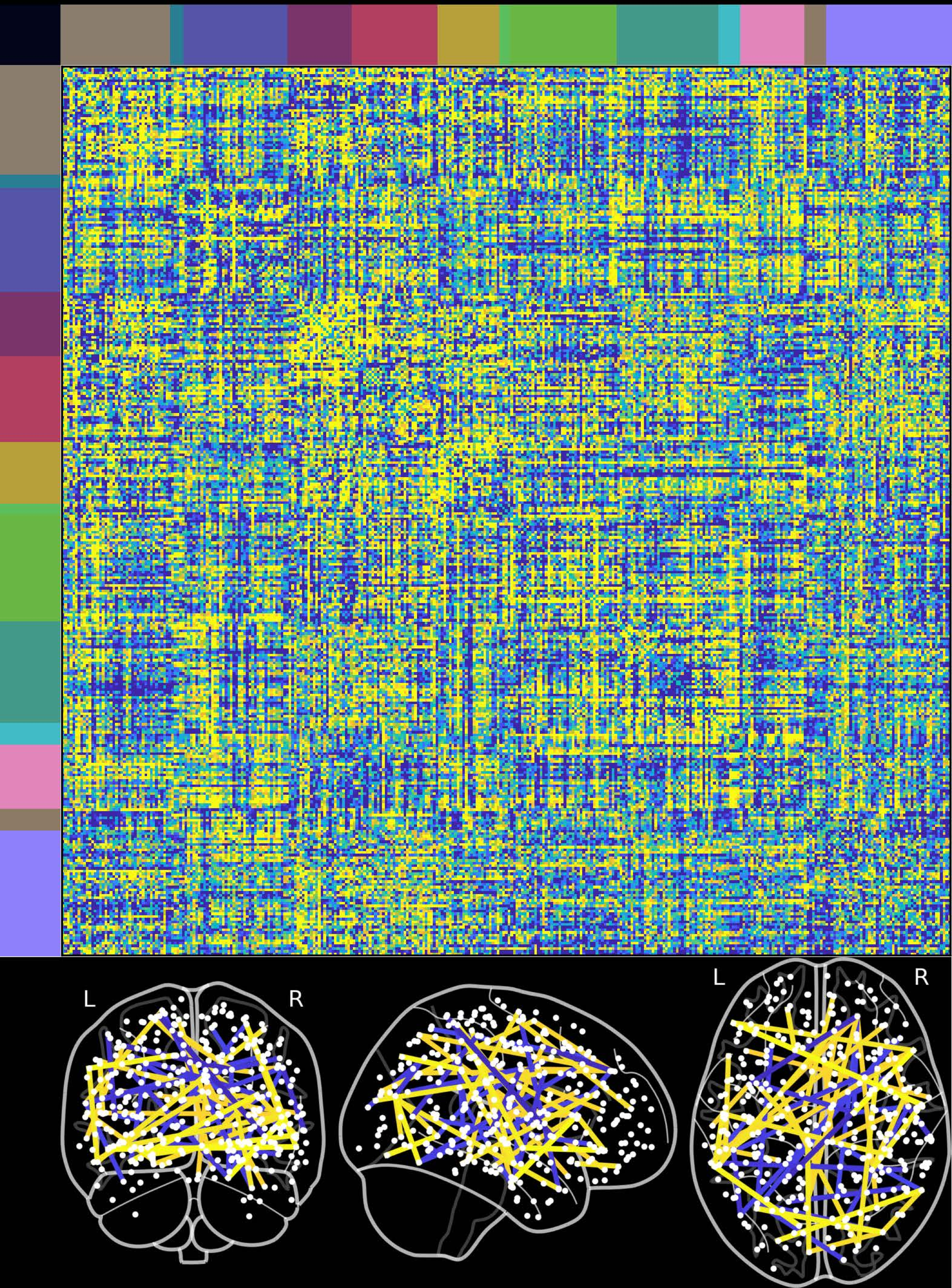

component_70

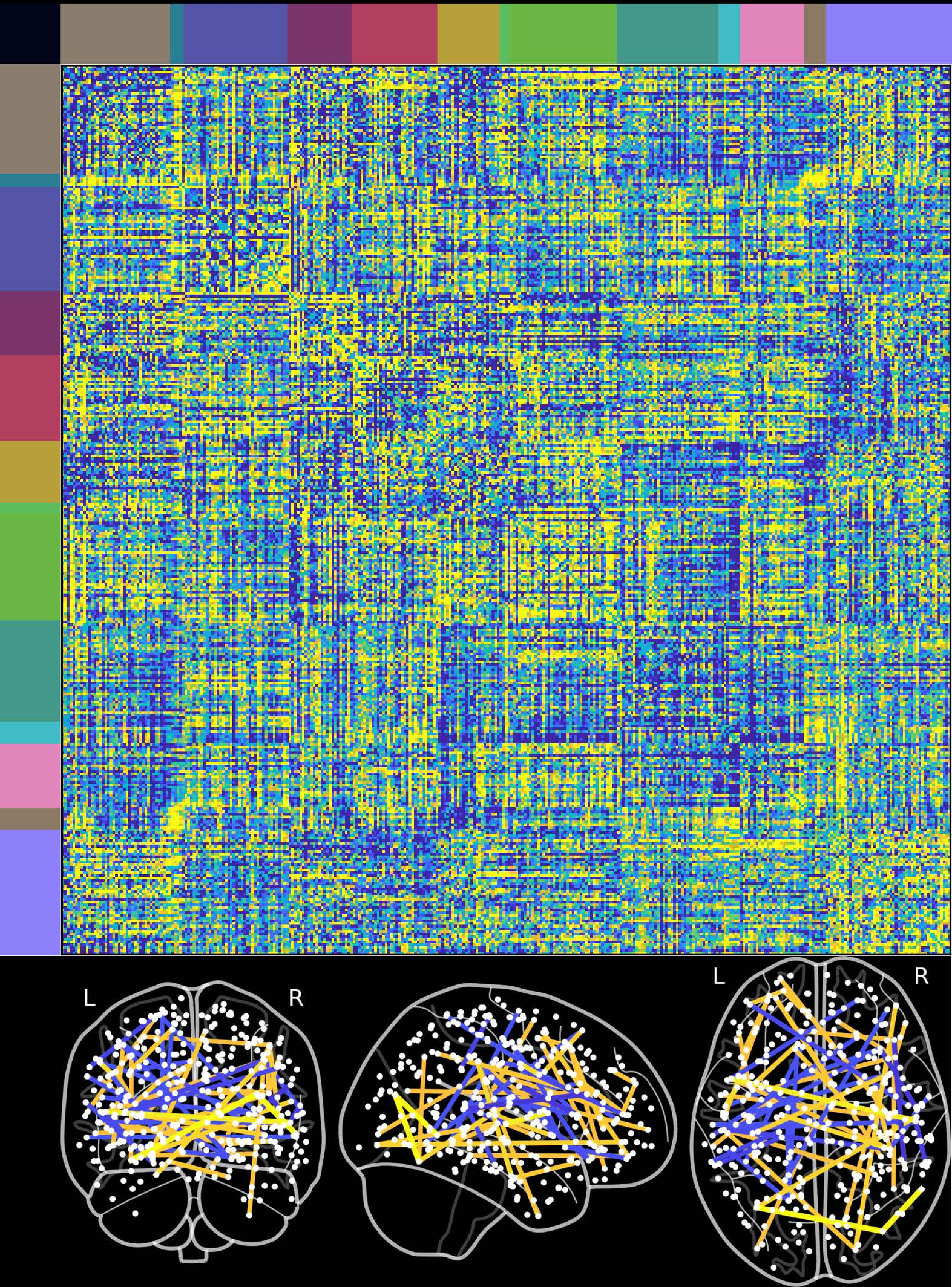

component_71

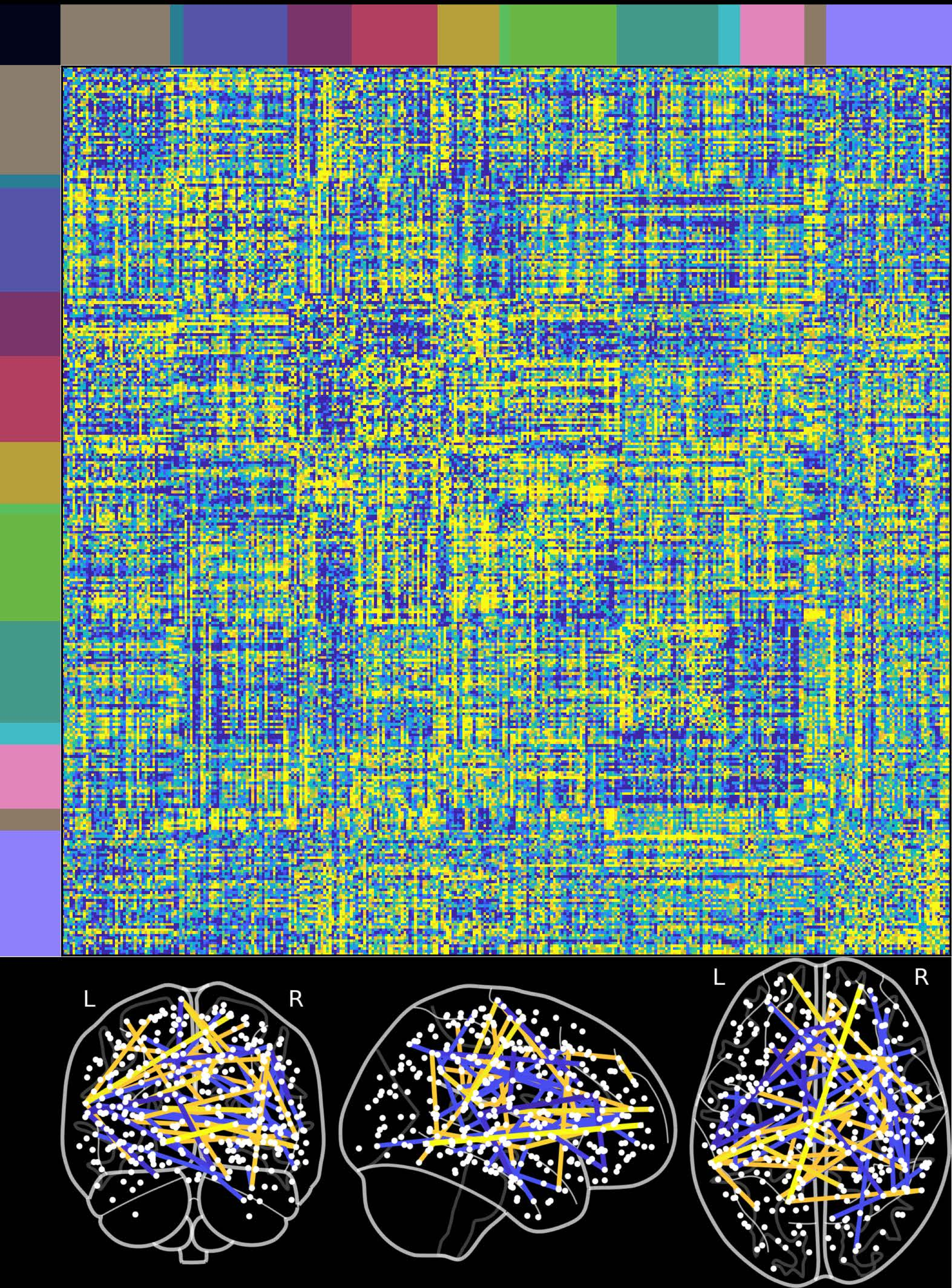

component_72

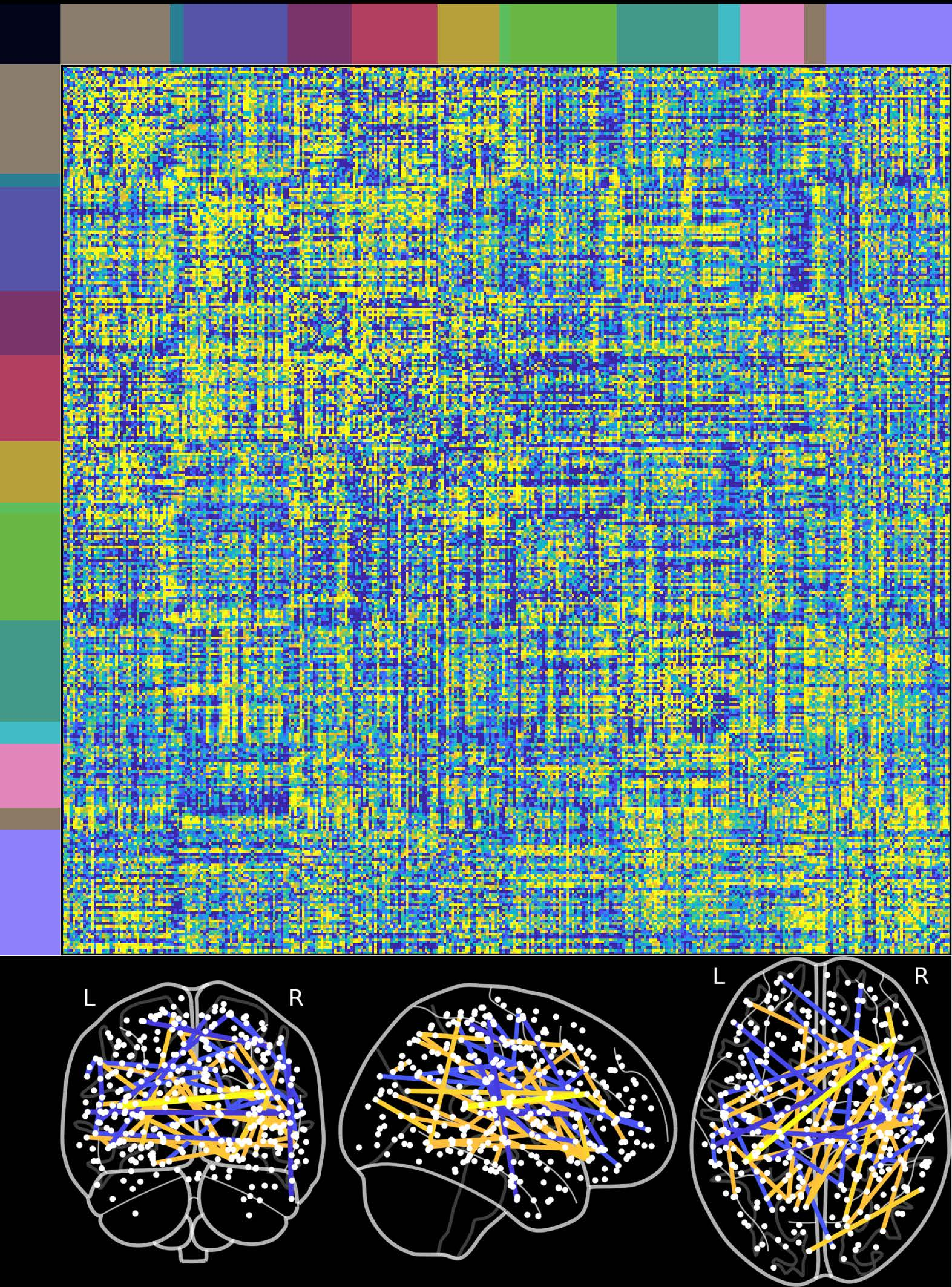

component_73

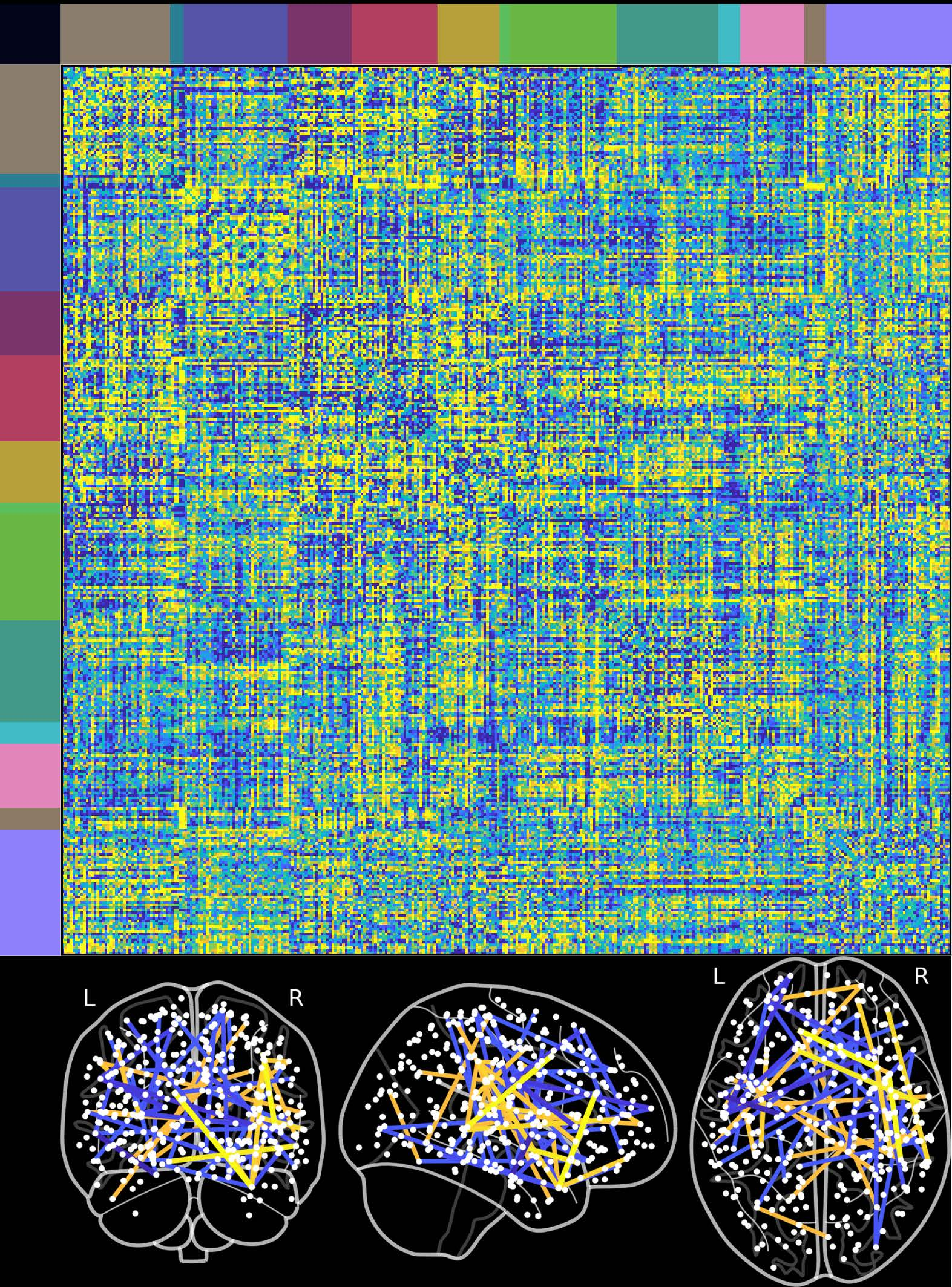

component_74

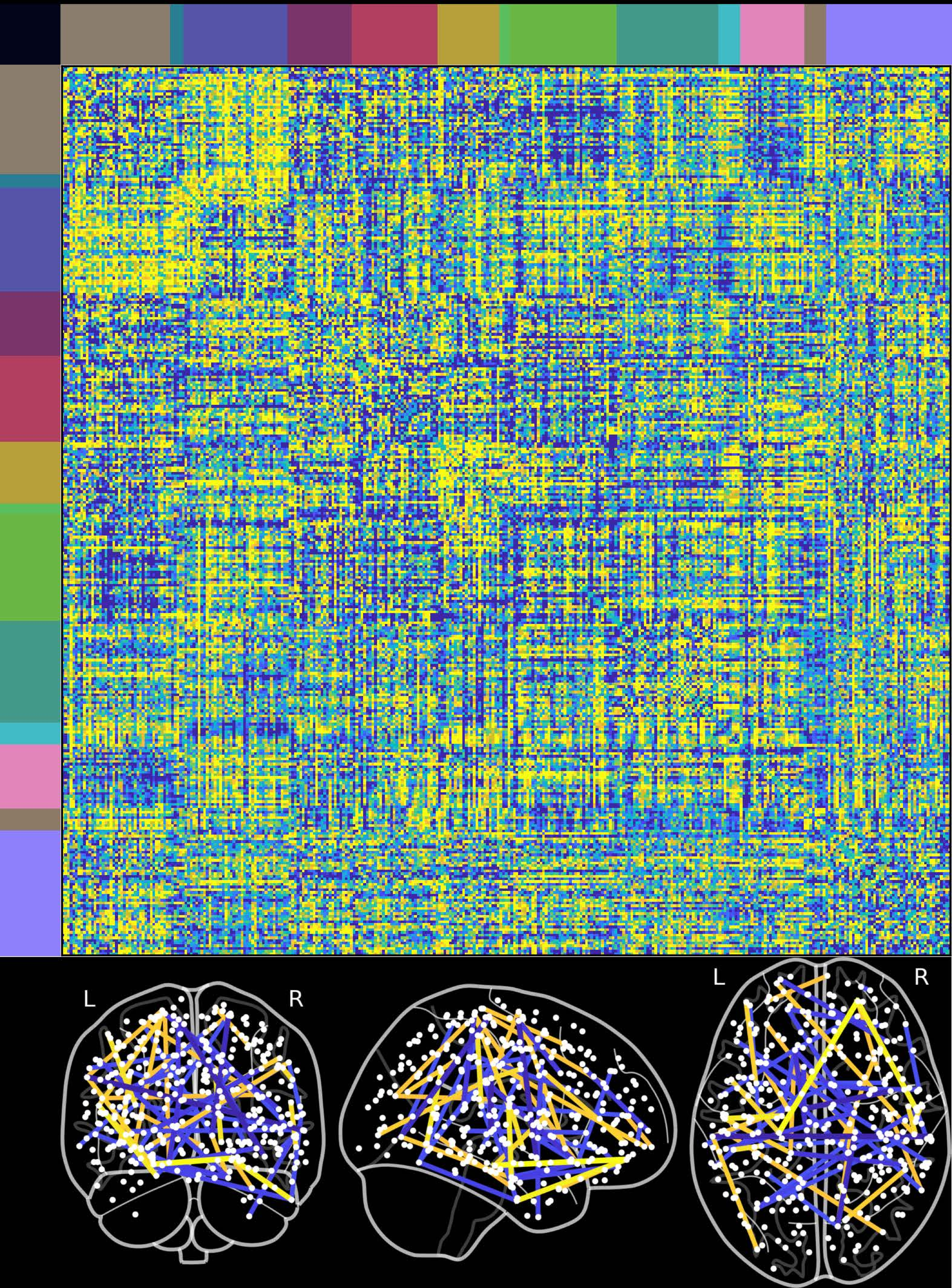

component_75

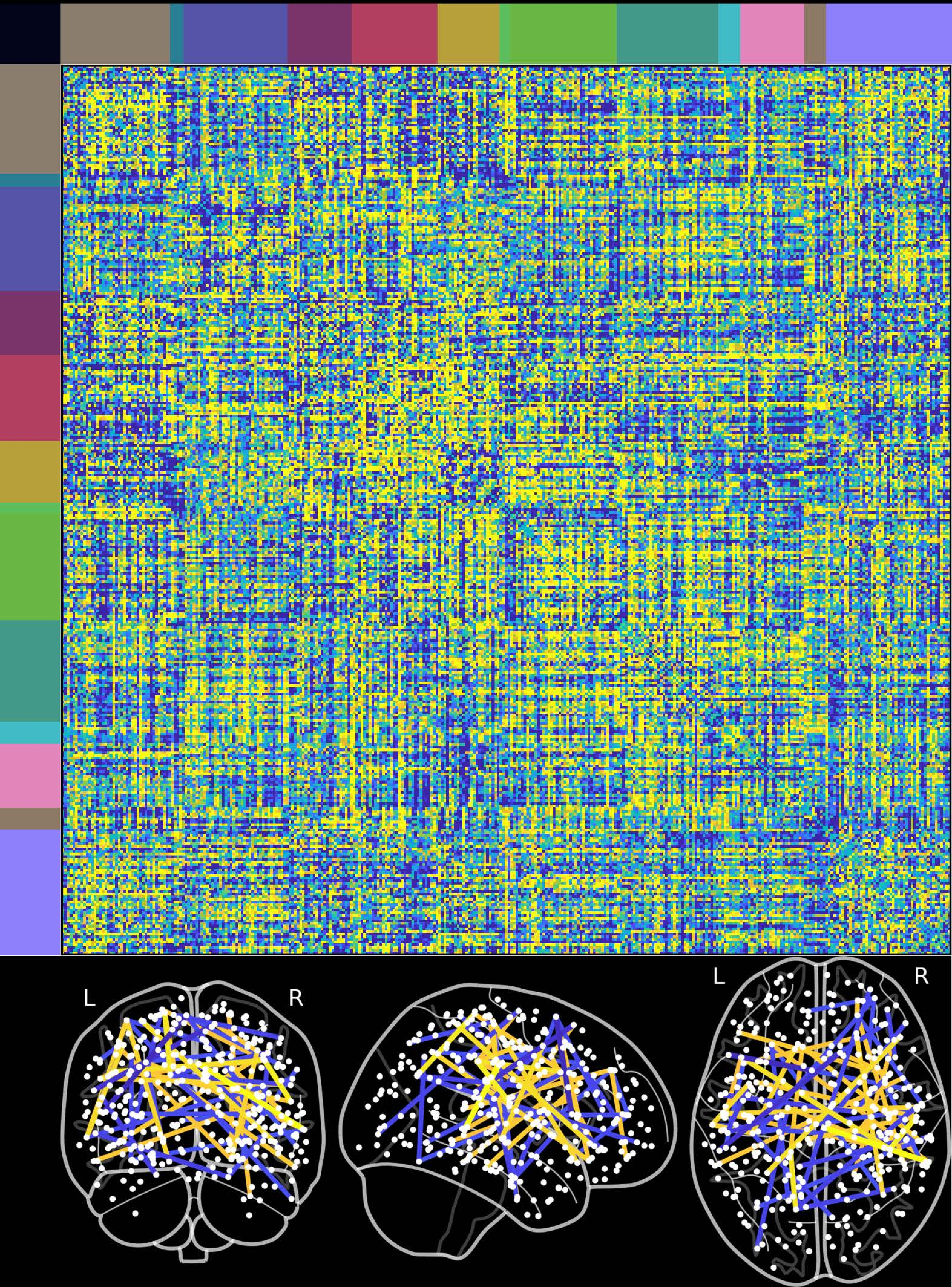

component_76

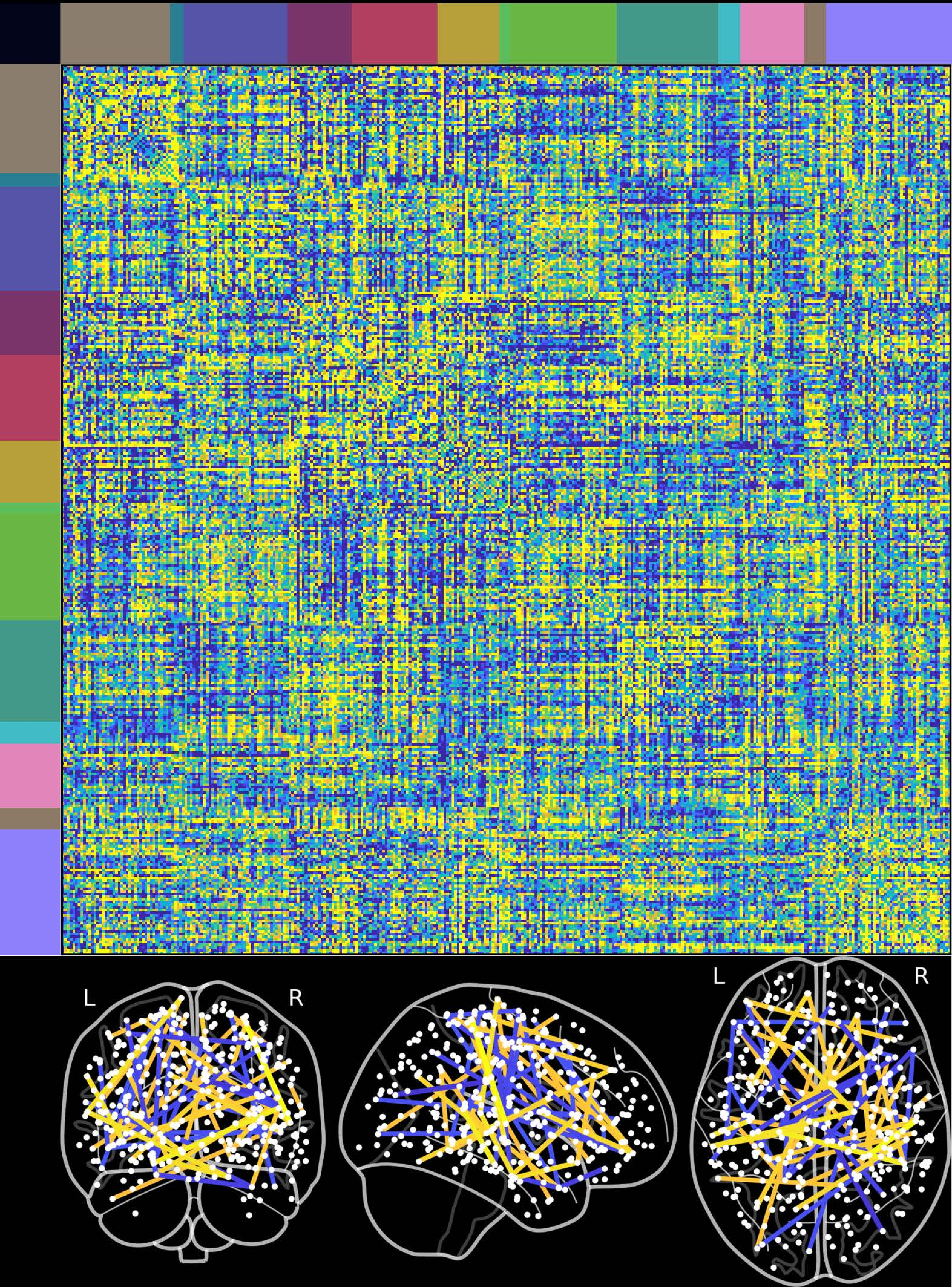

component_77

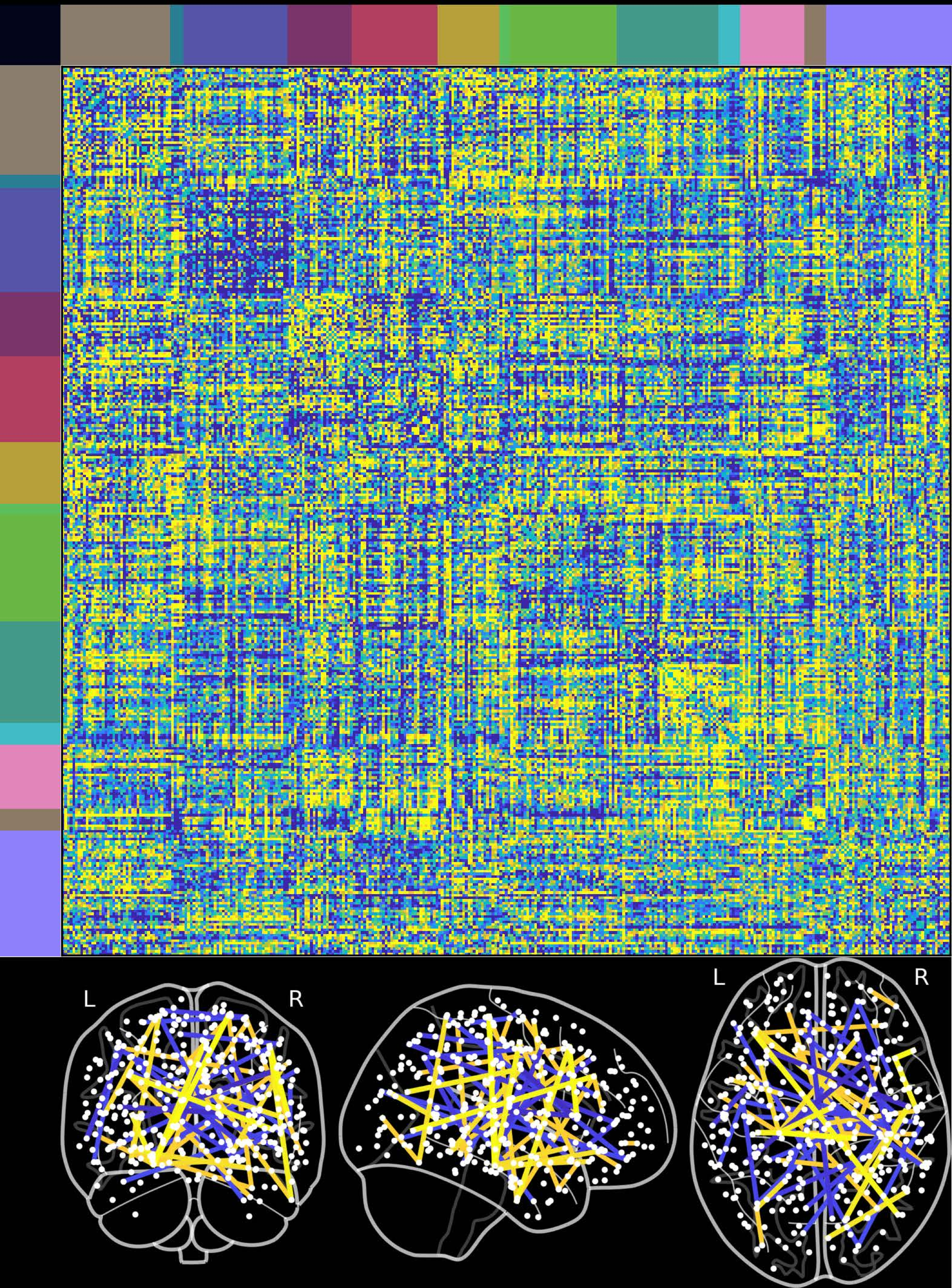

component_78

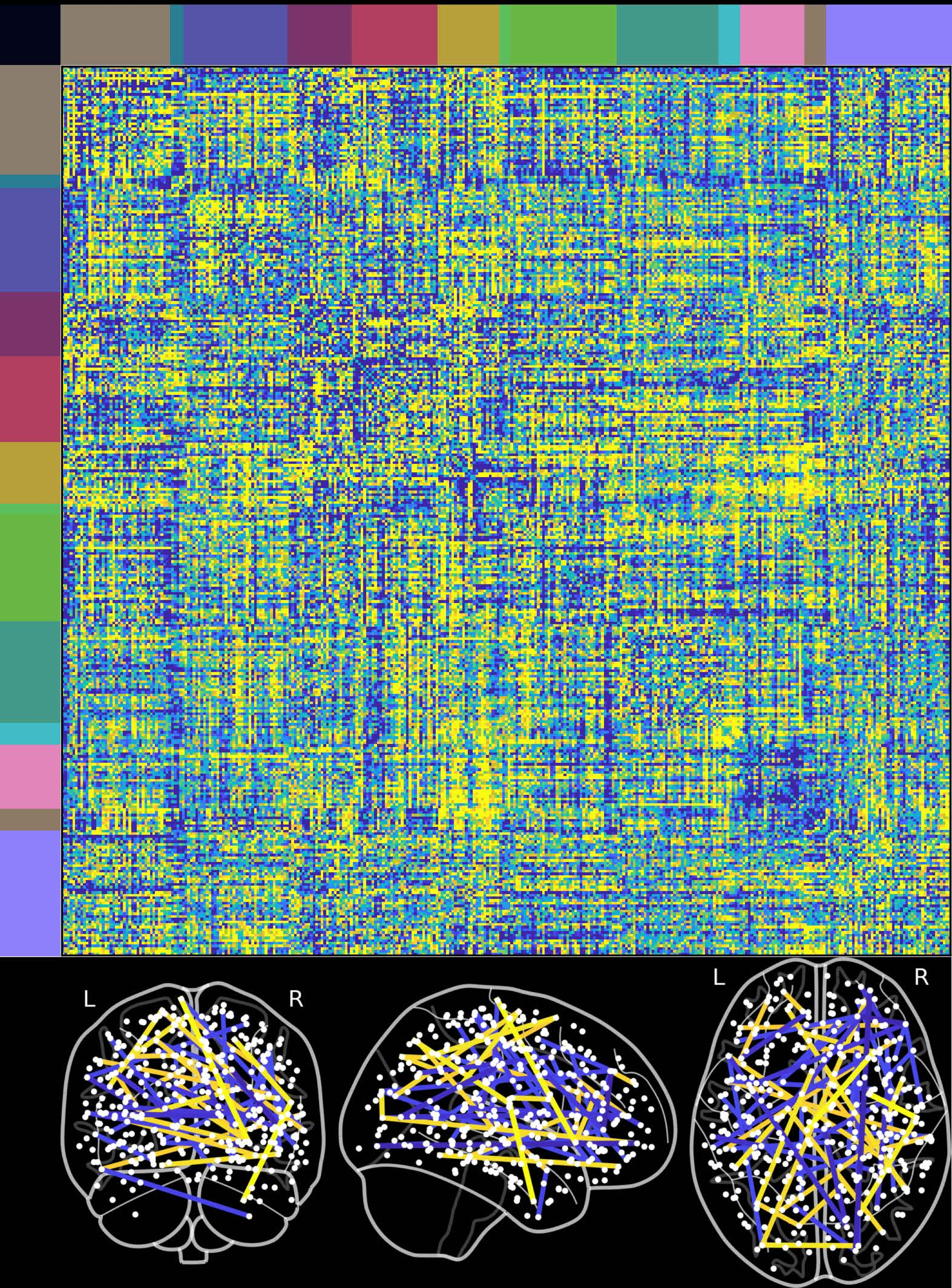

component_79

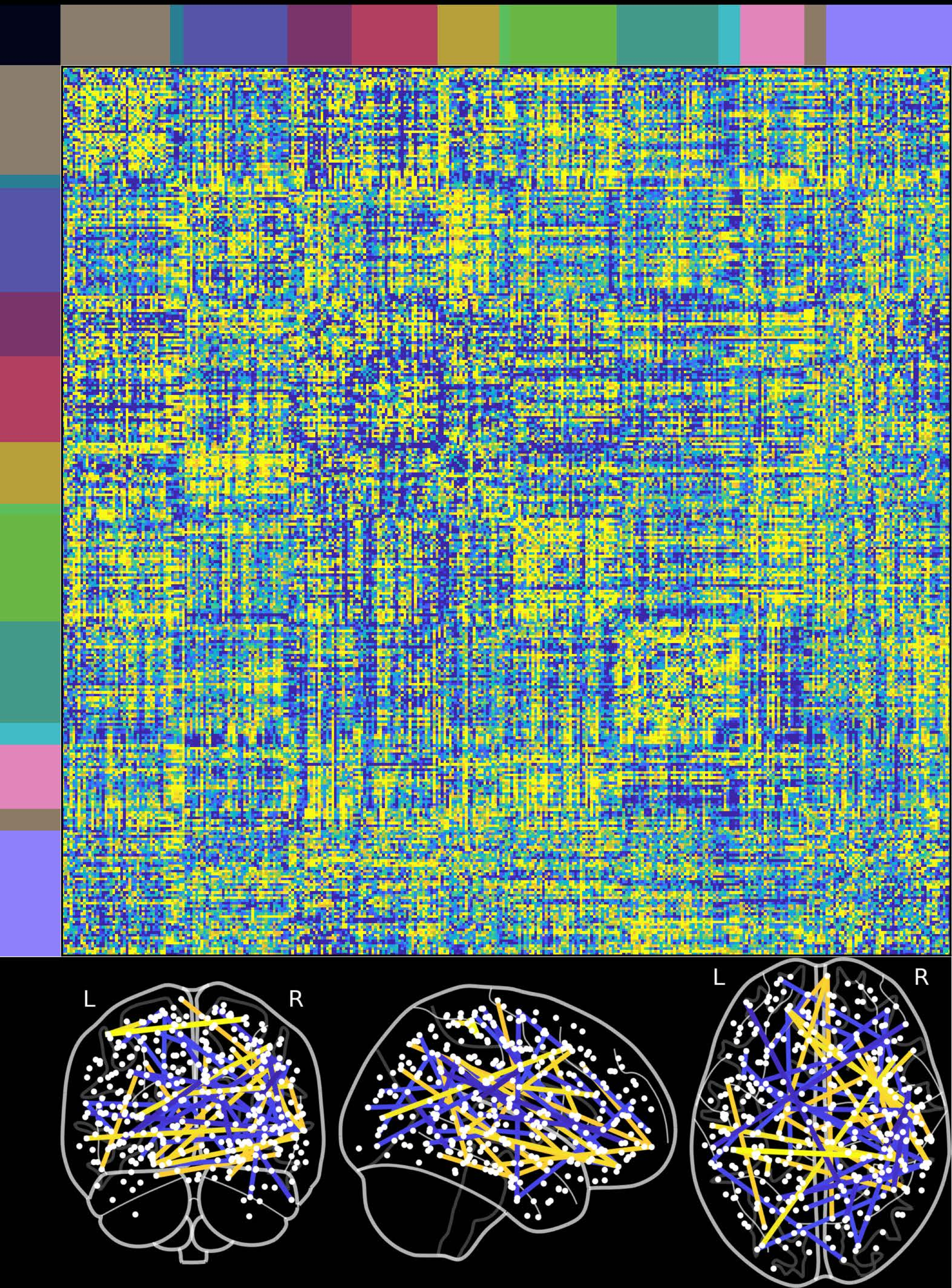

component_80

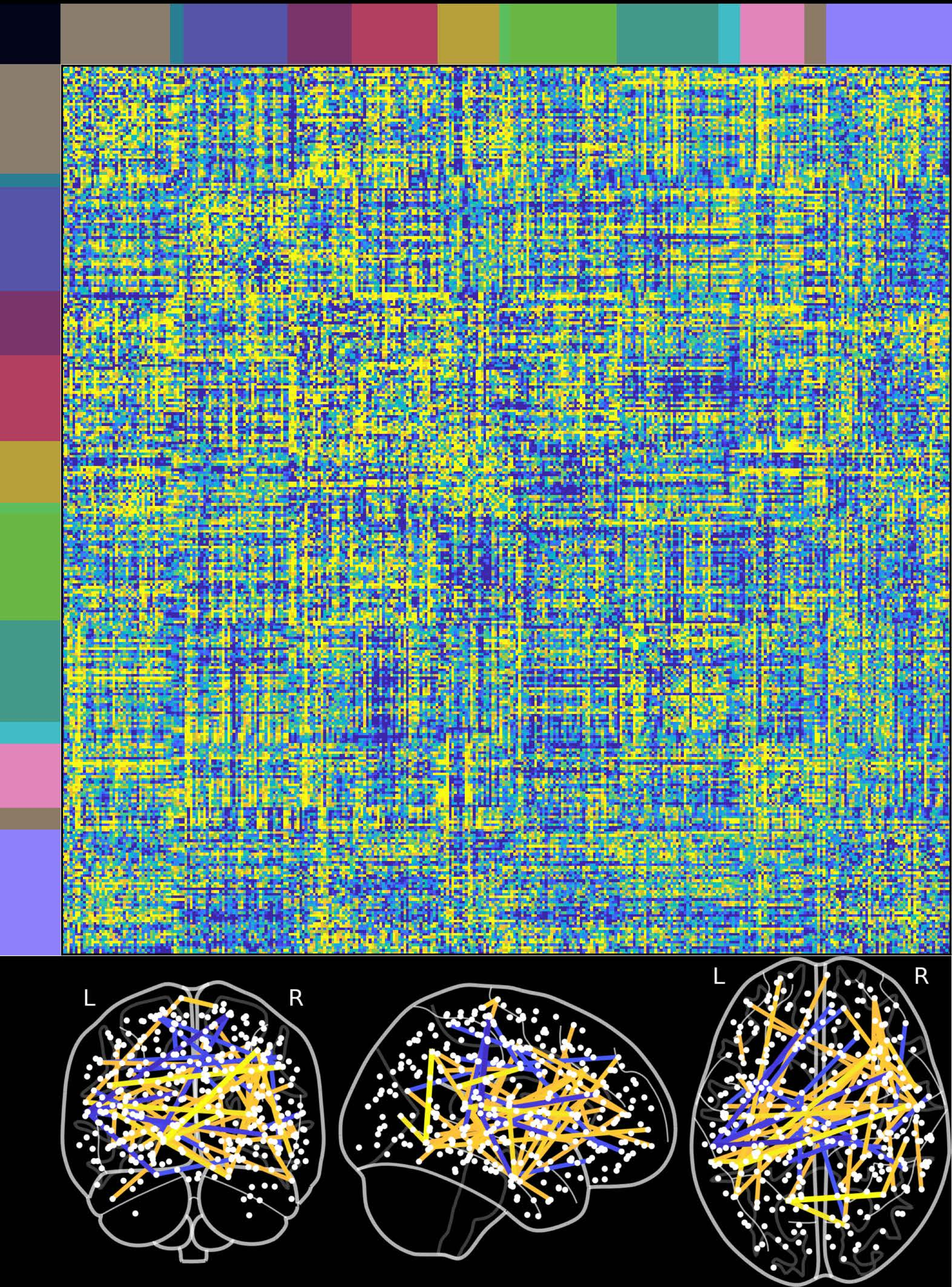

component_81

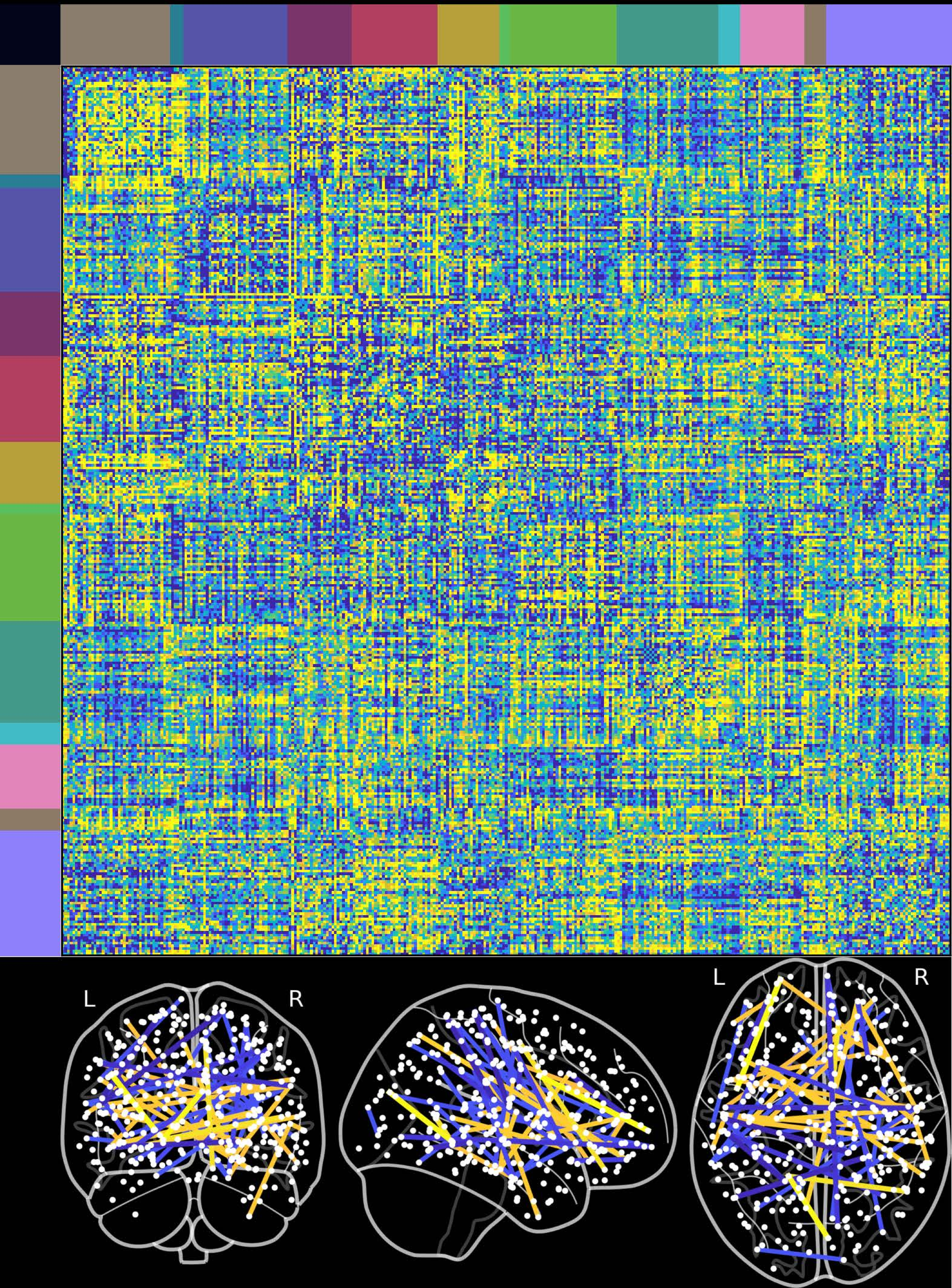

component_82

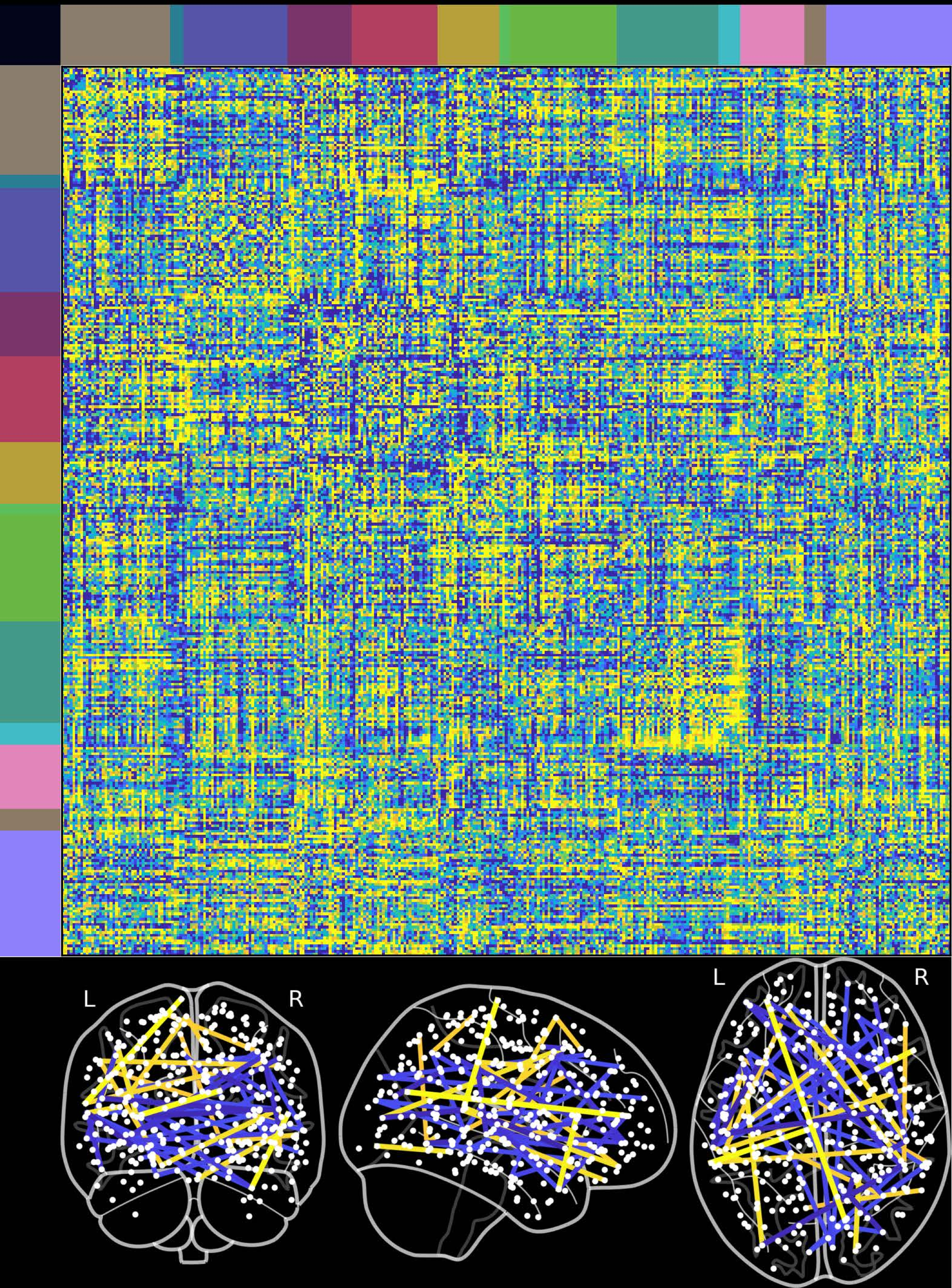

component_83

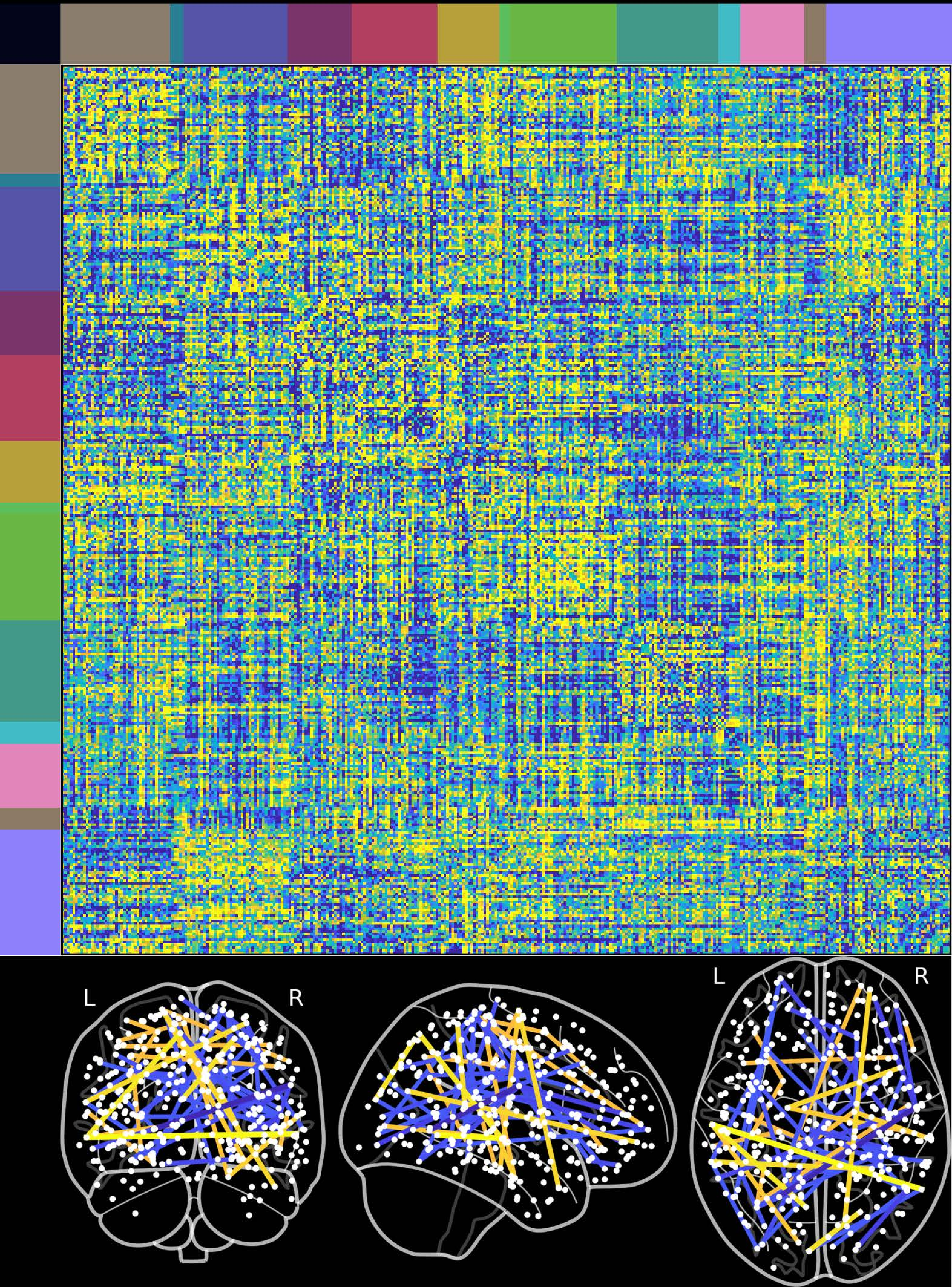

component_84

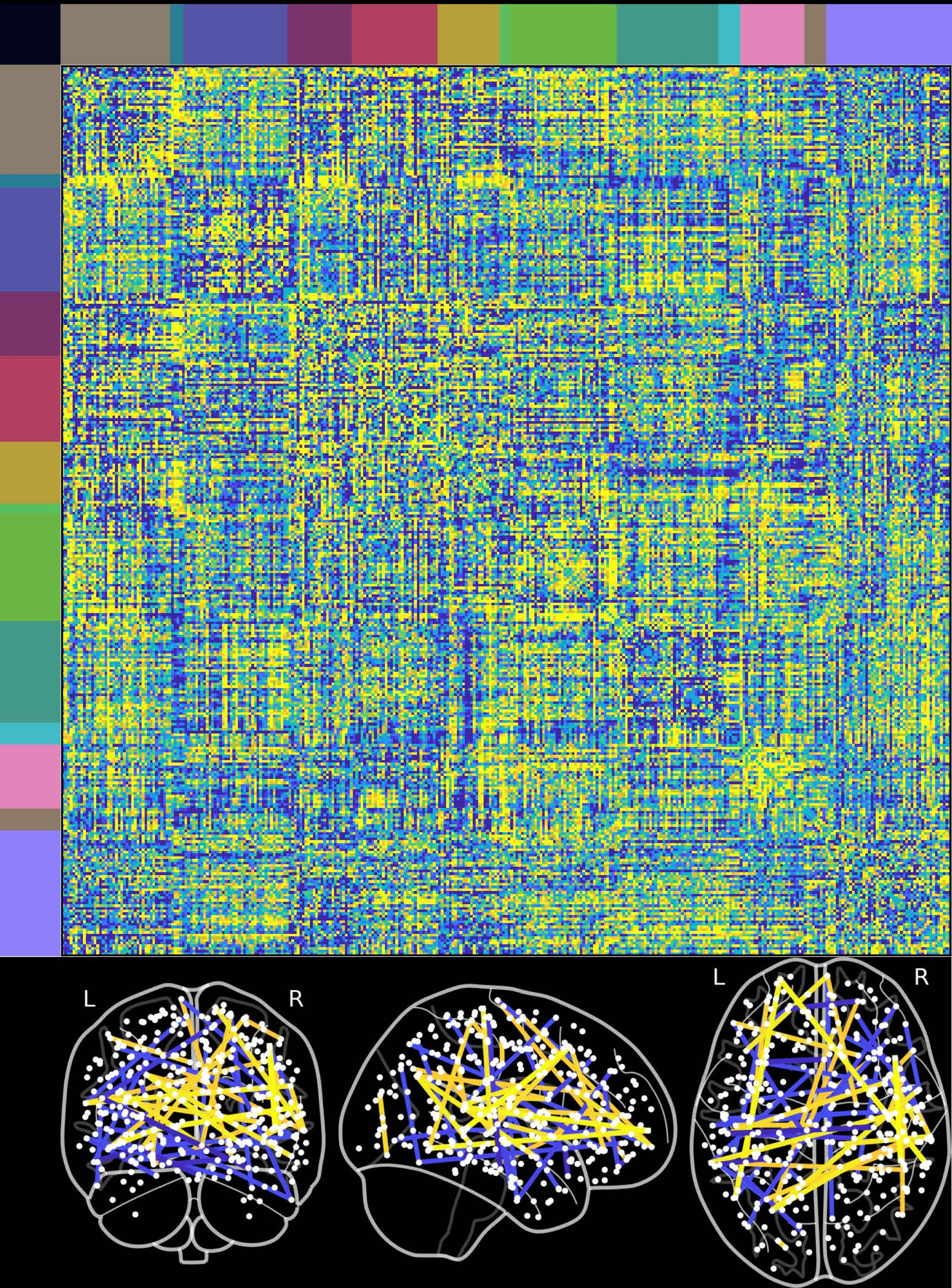

component_85

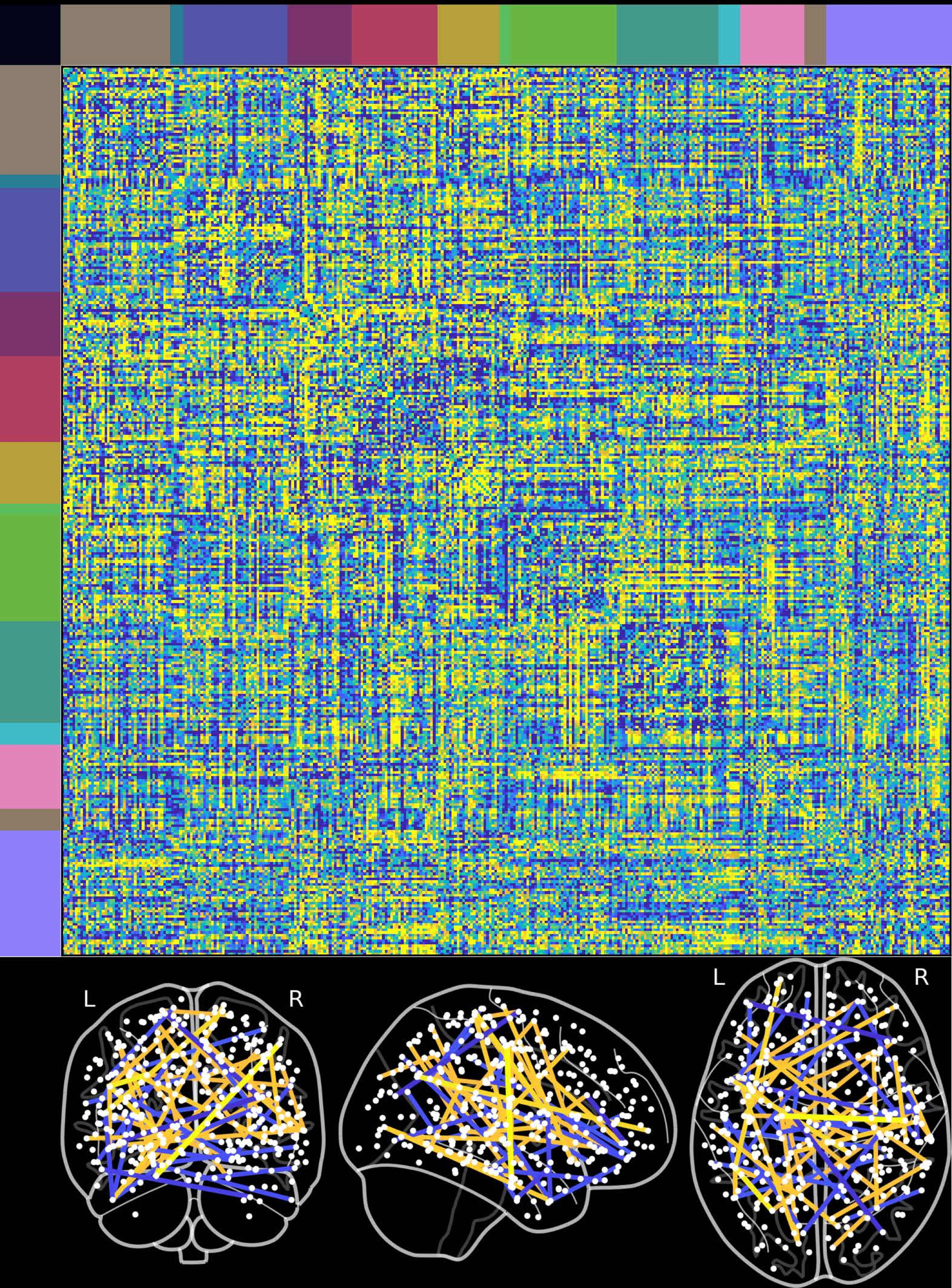

component_86

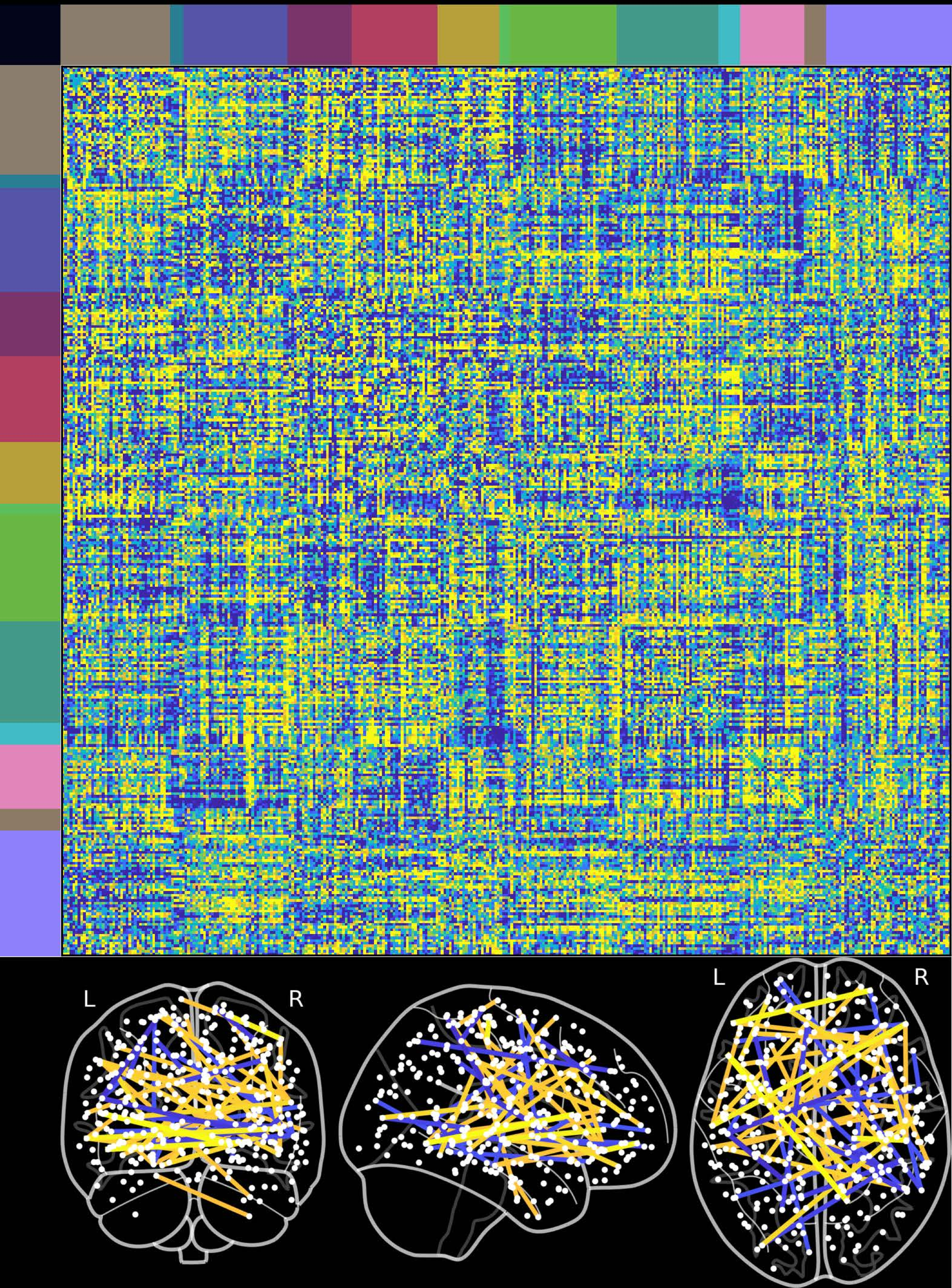

component_87

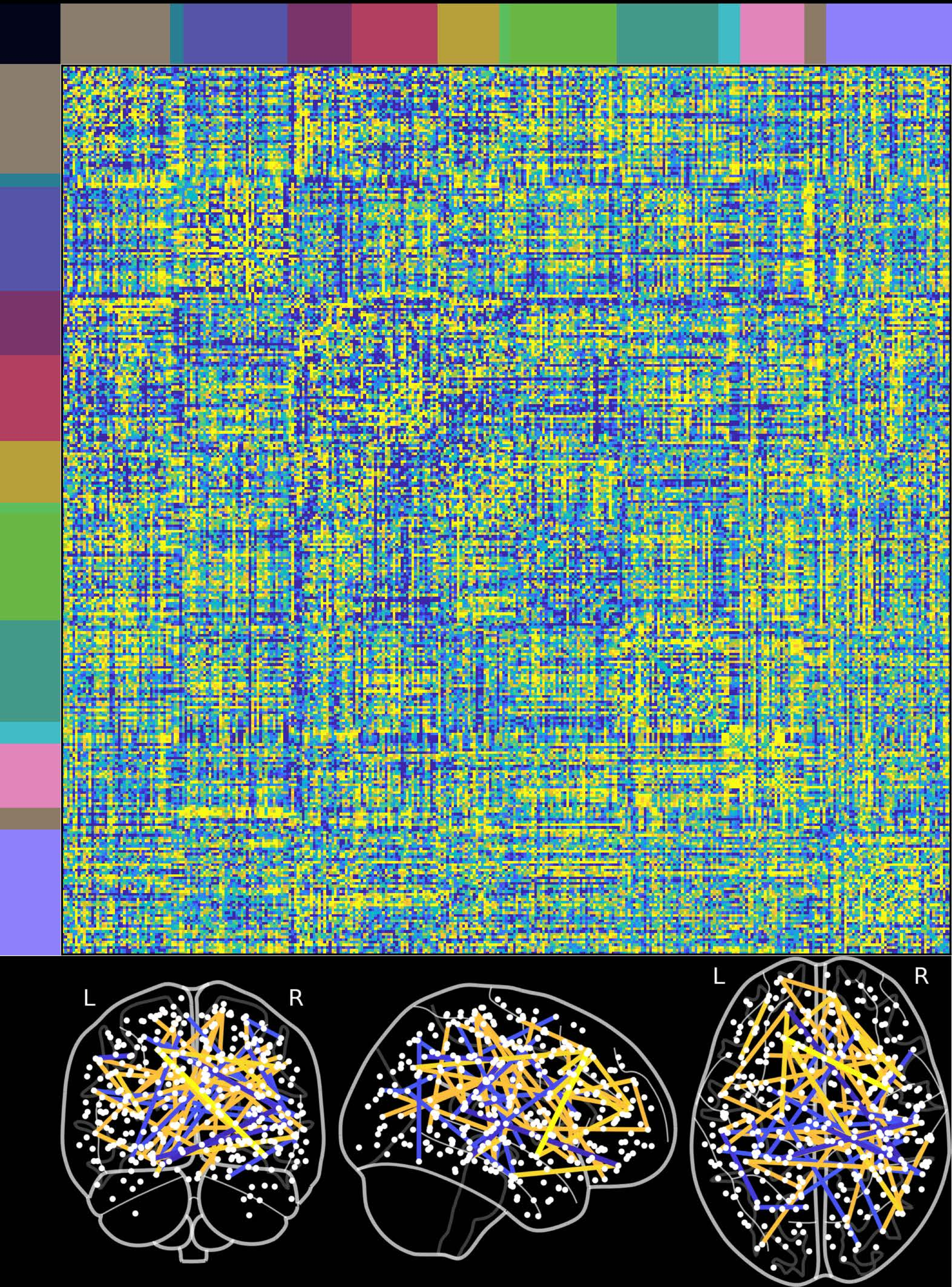

component_88

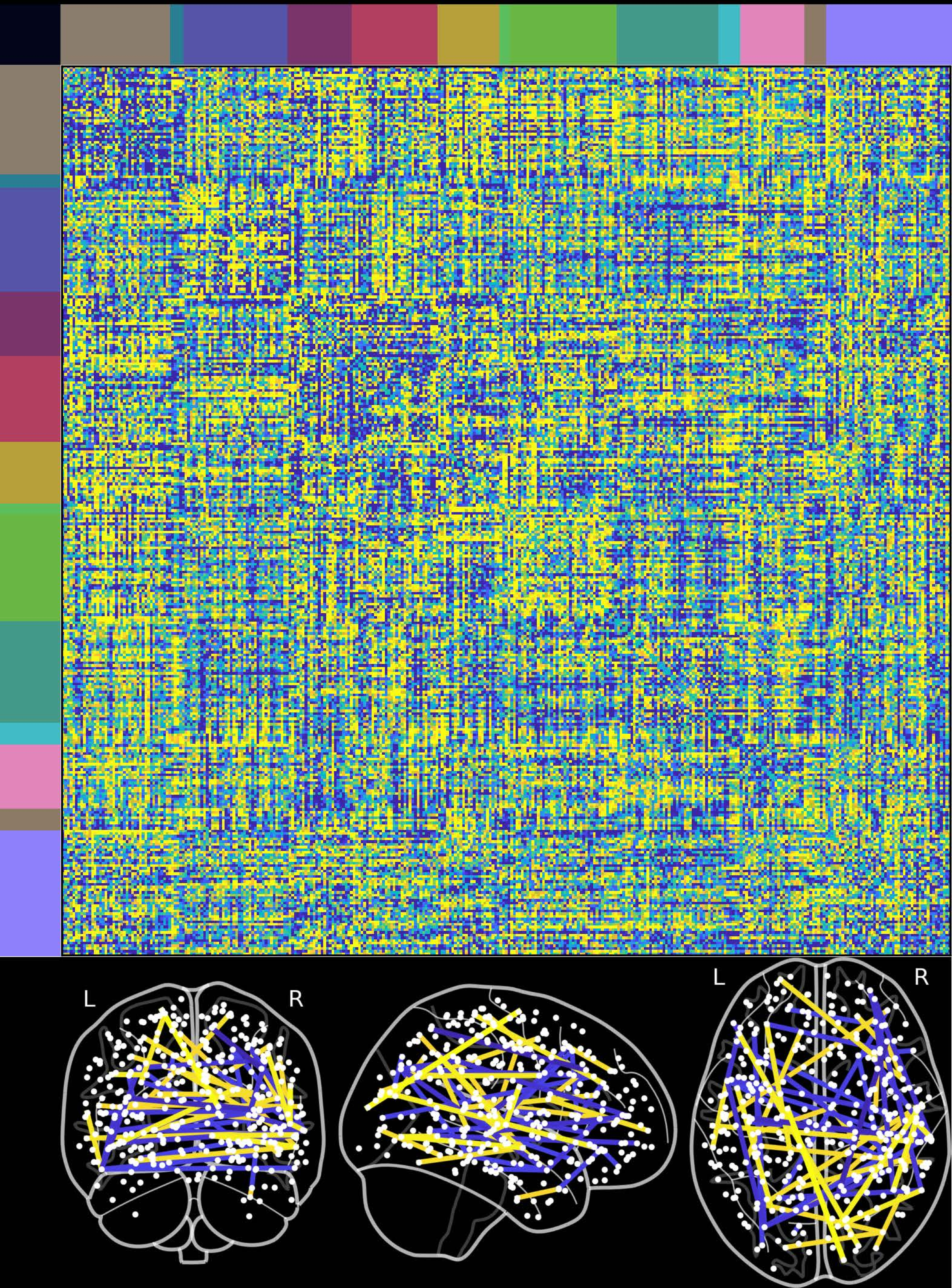

Healthy control group component maps

component_0

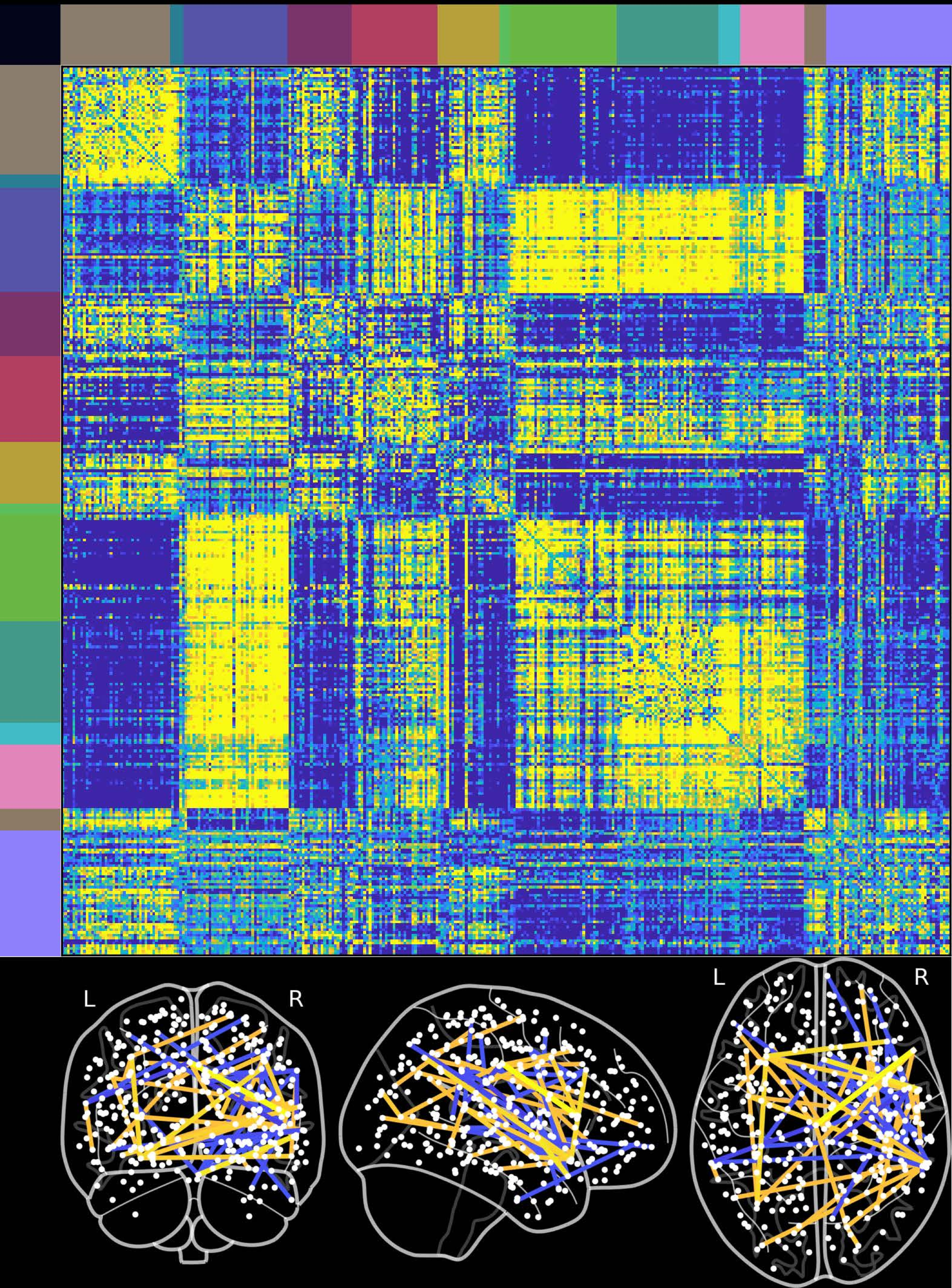

component_1

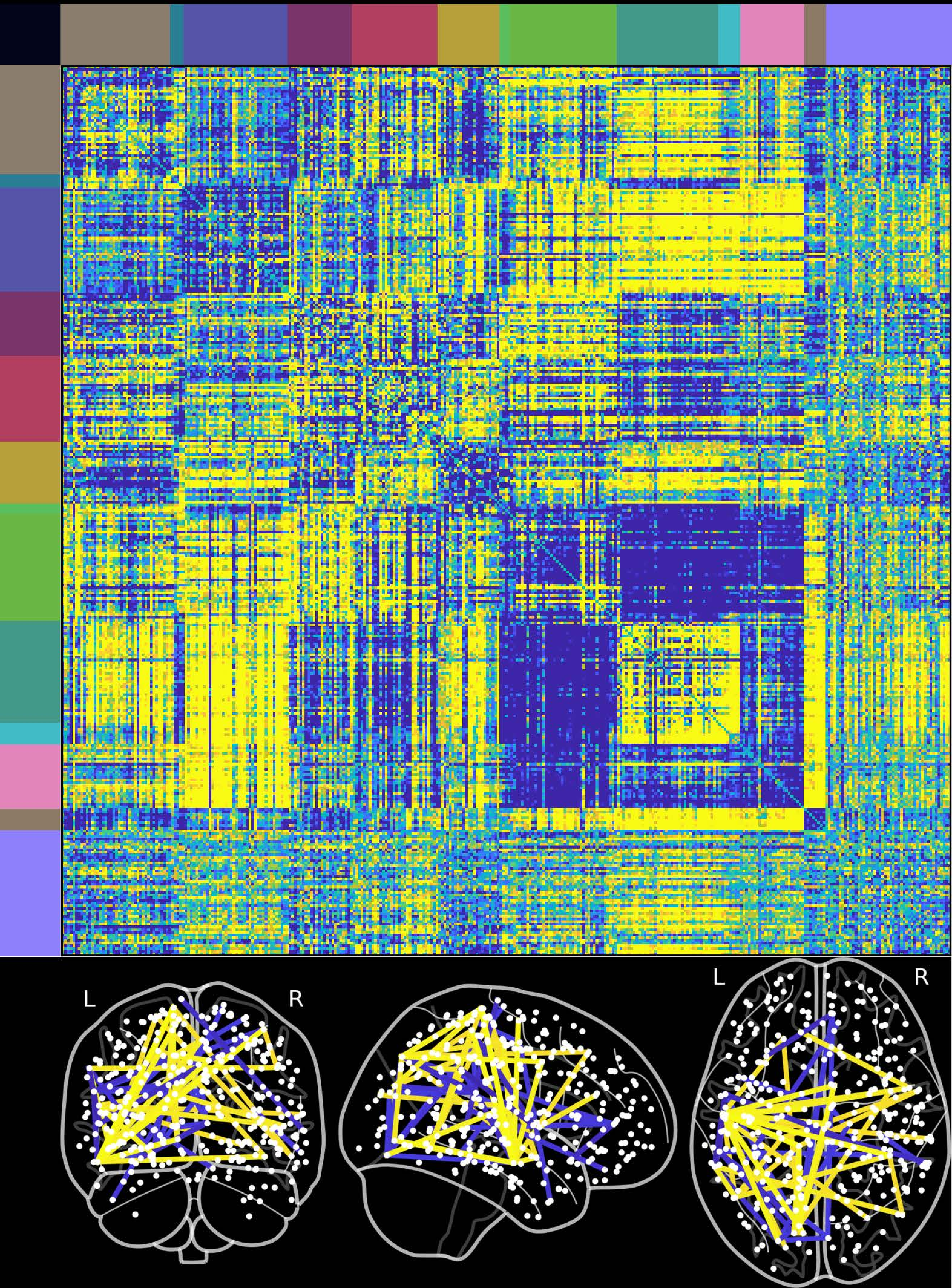

component_2

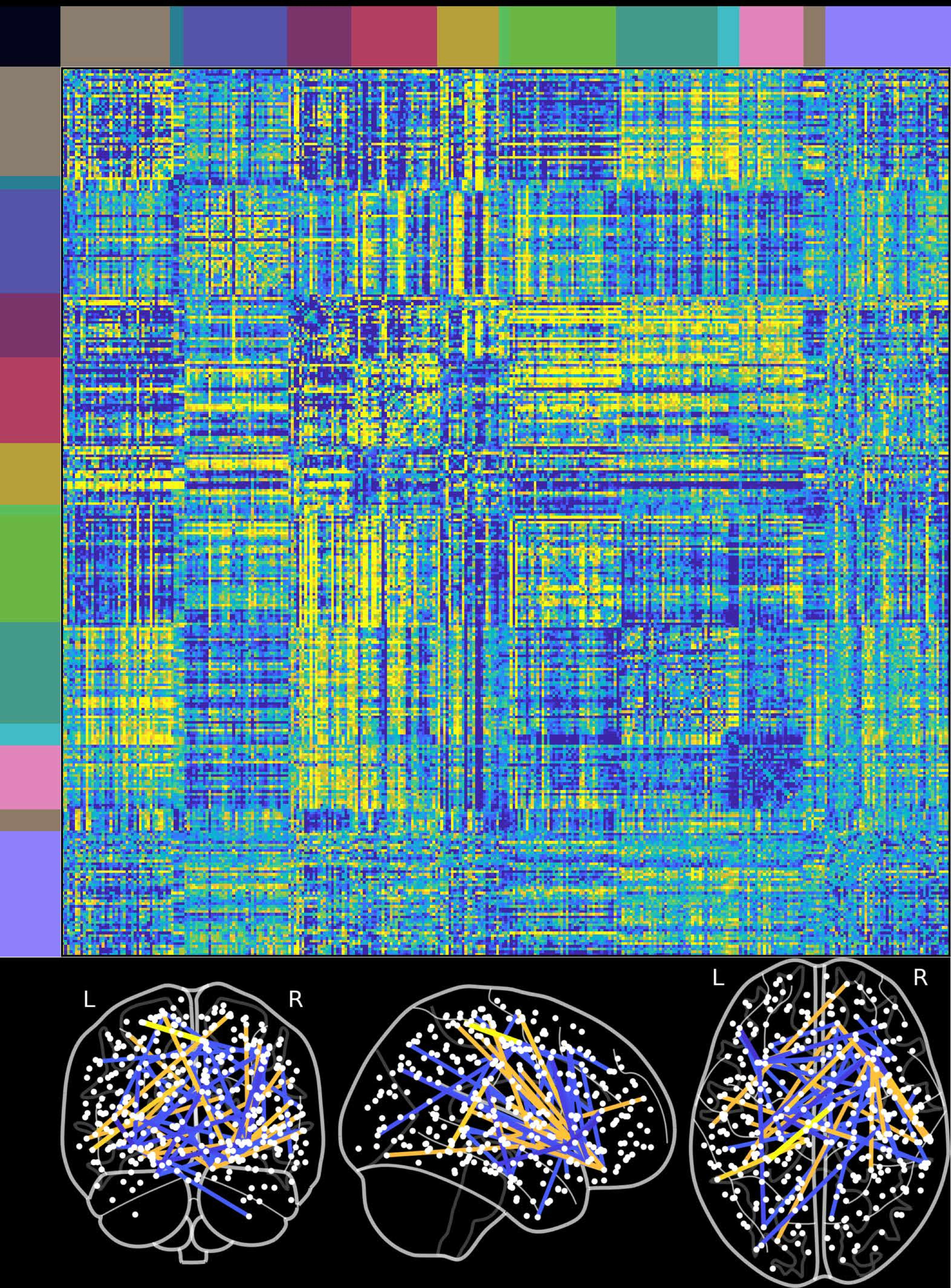

component_3

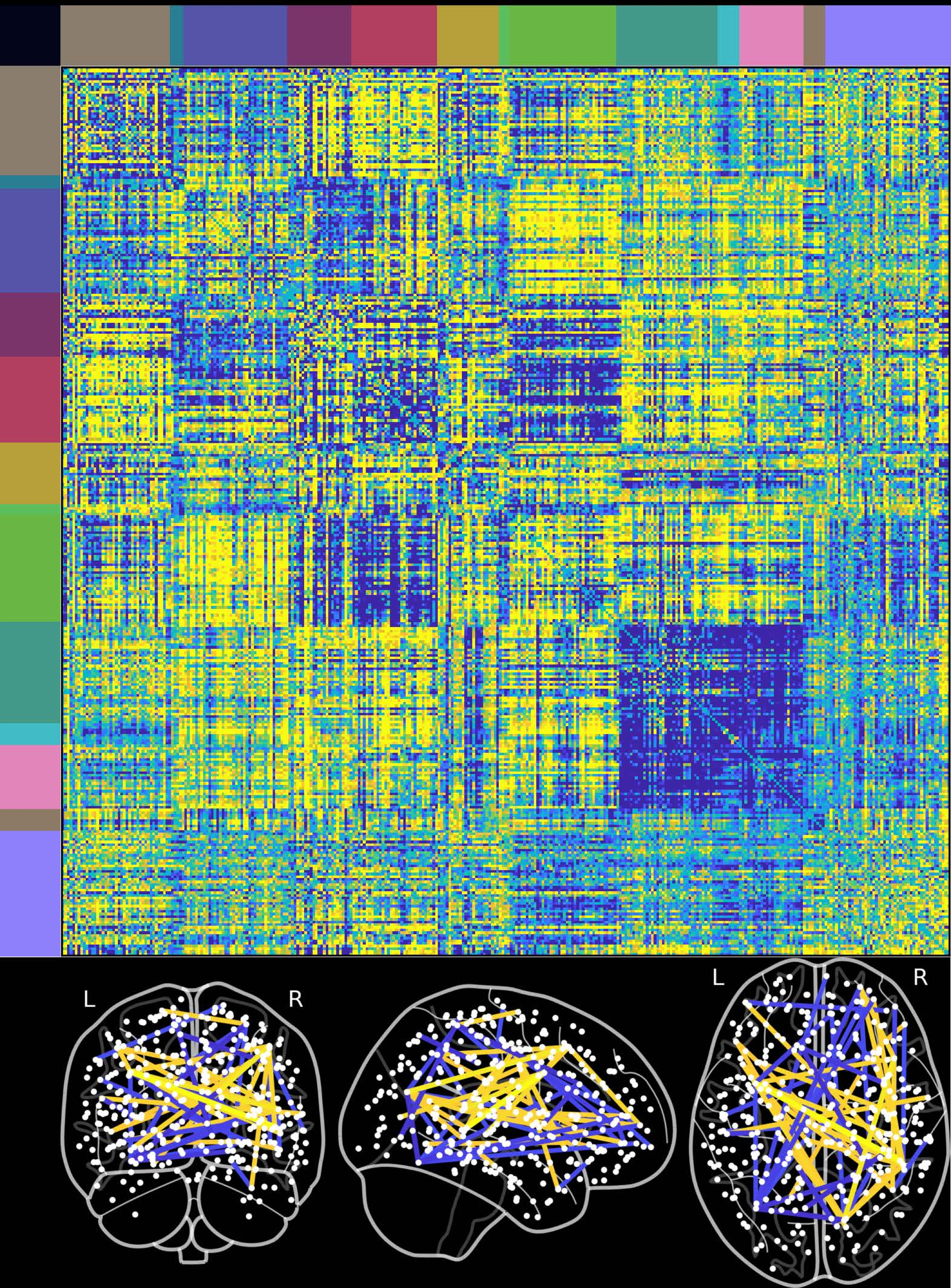

component_4

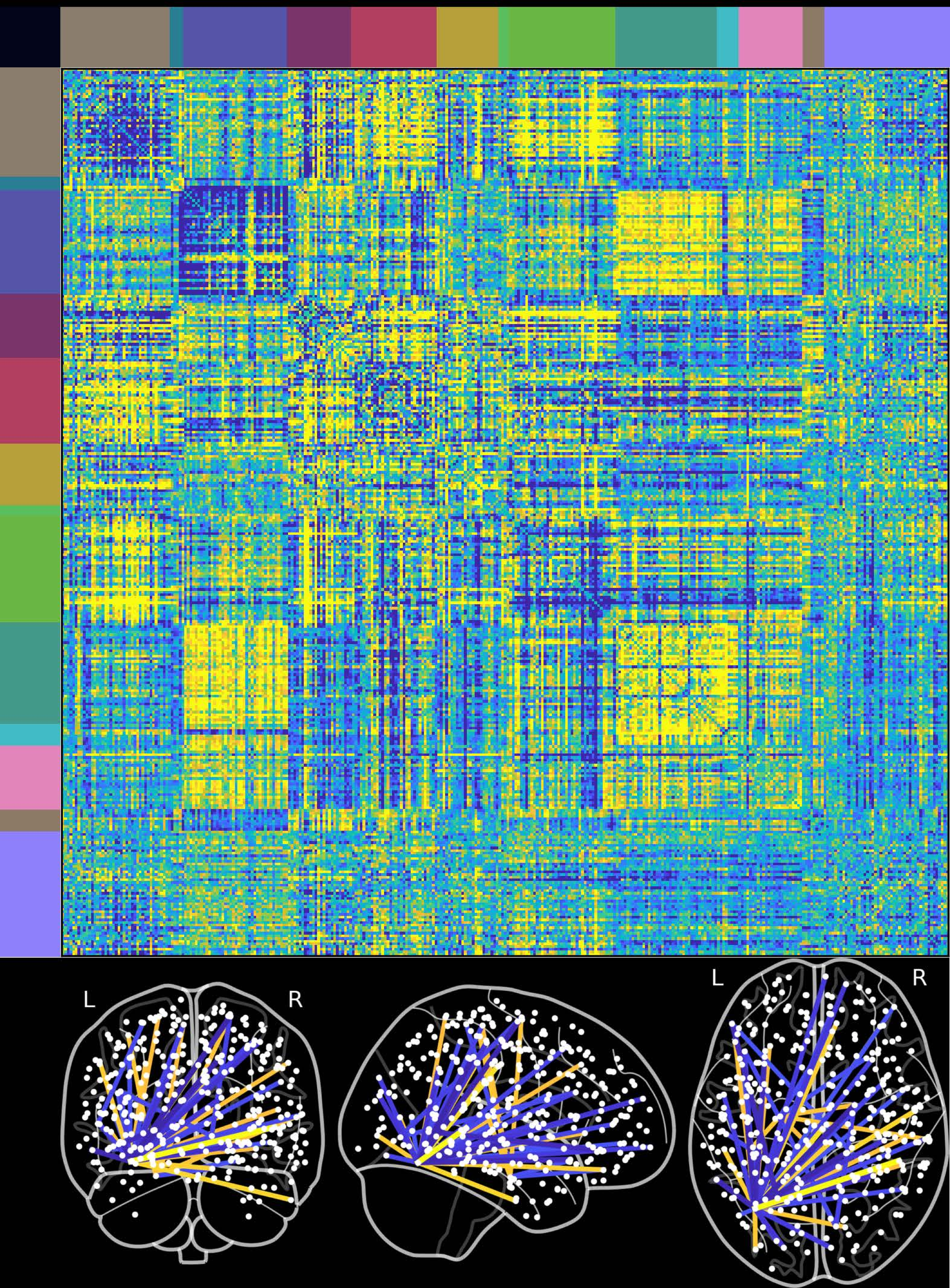

component_5

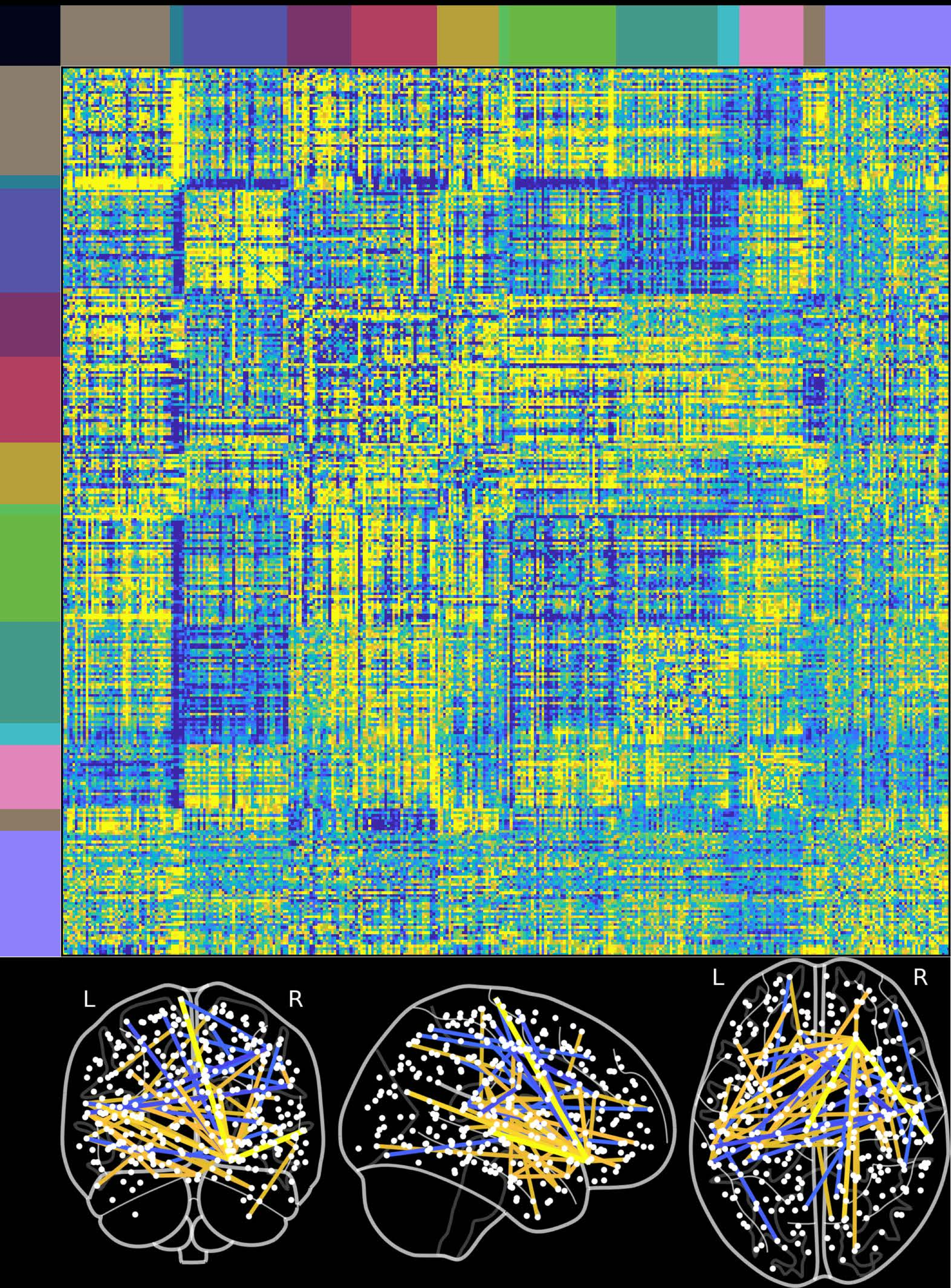

component_6

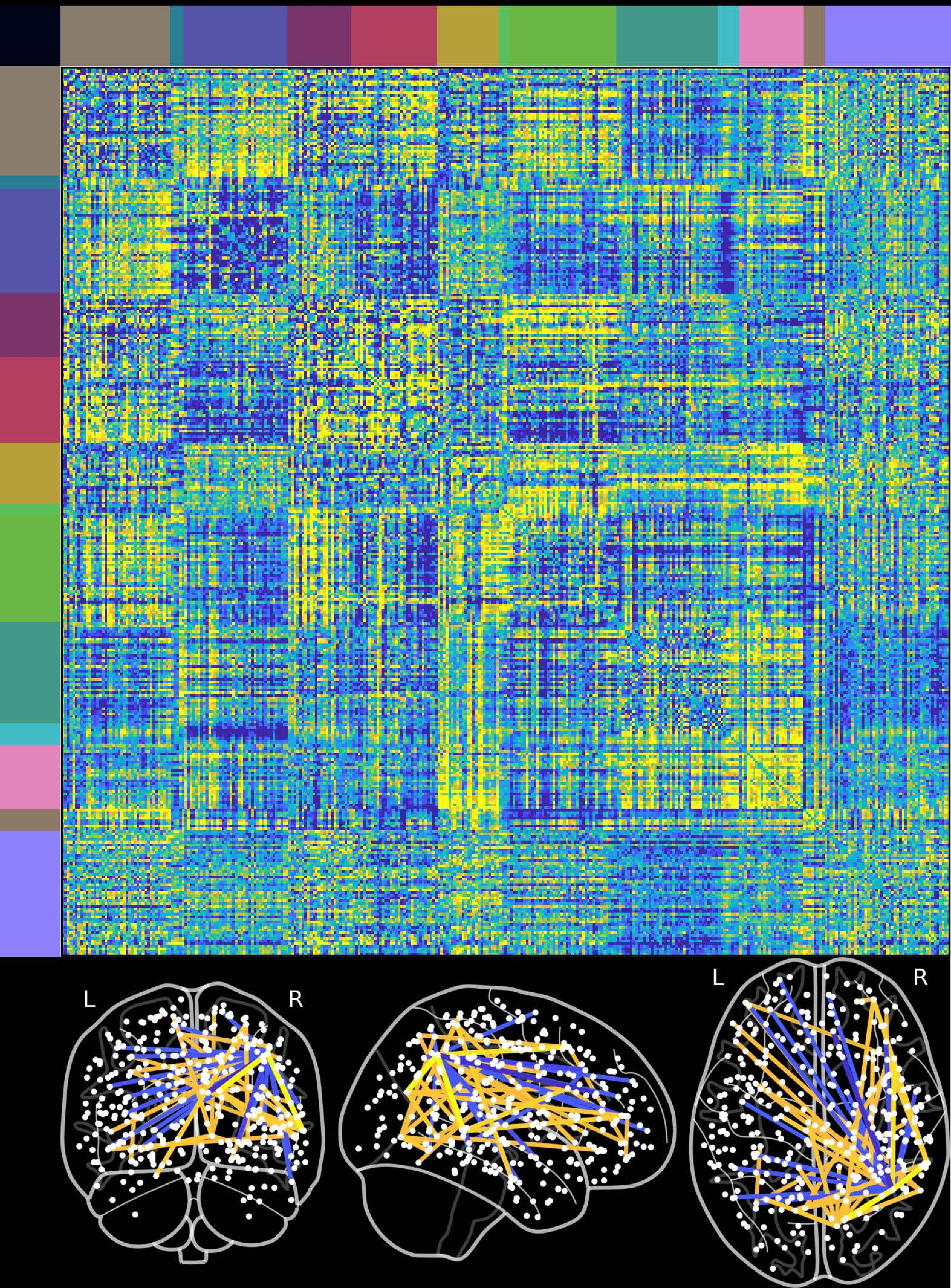

component_7

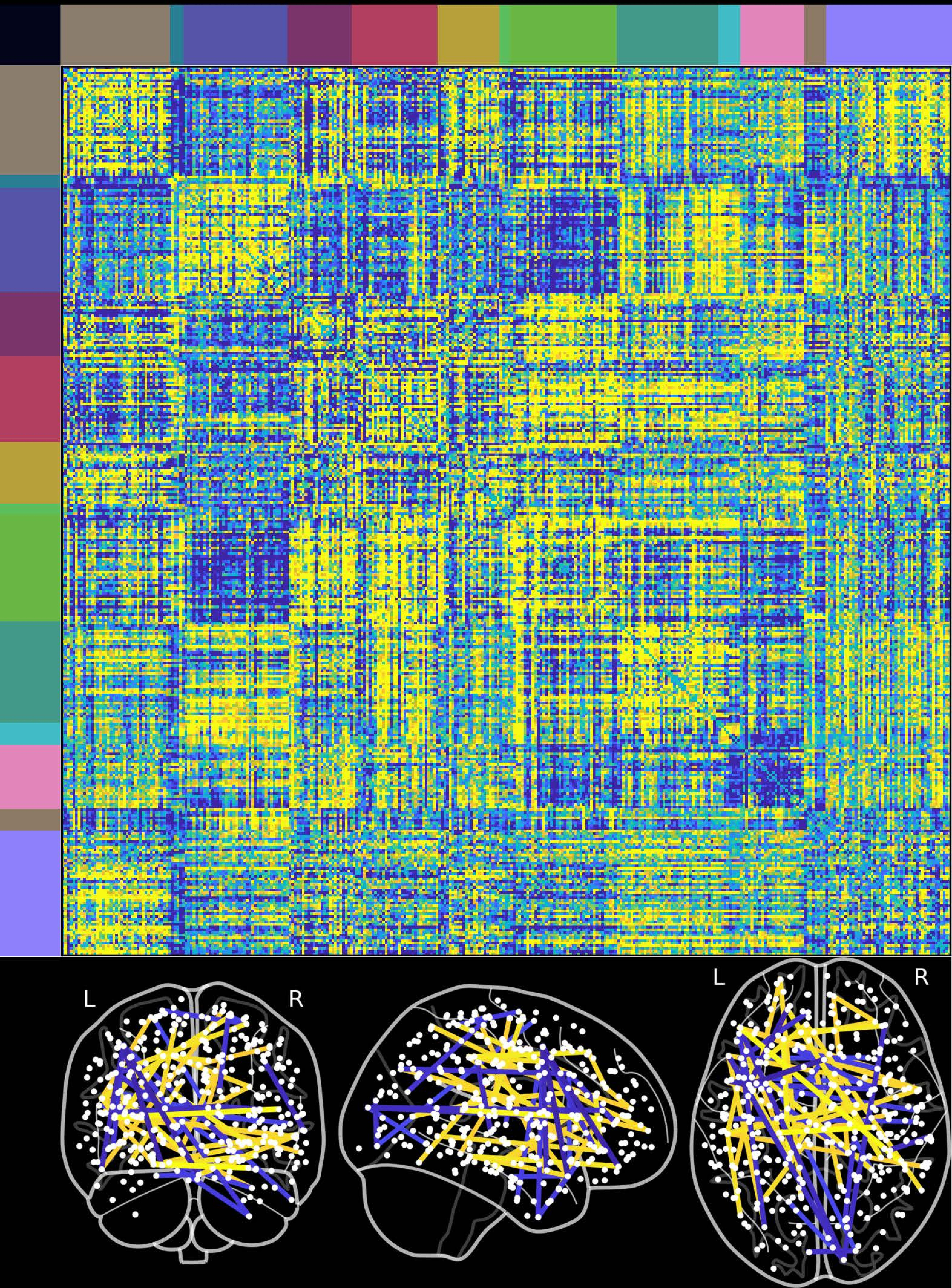

component_8

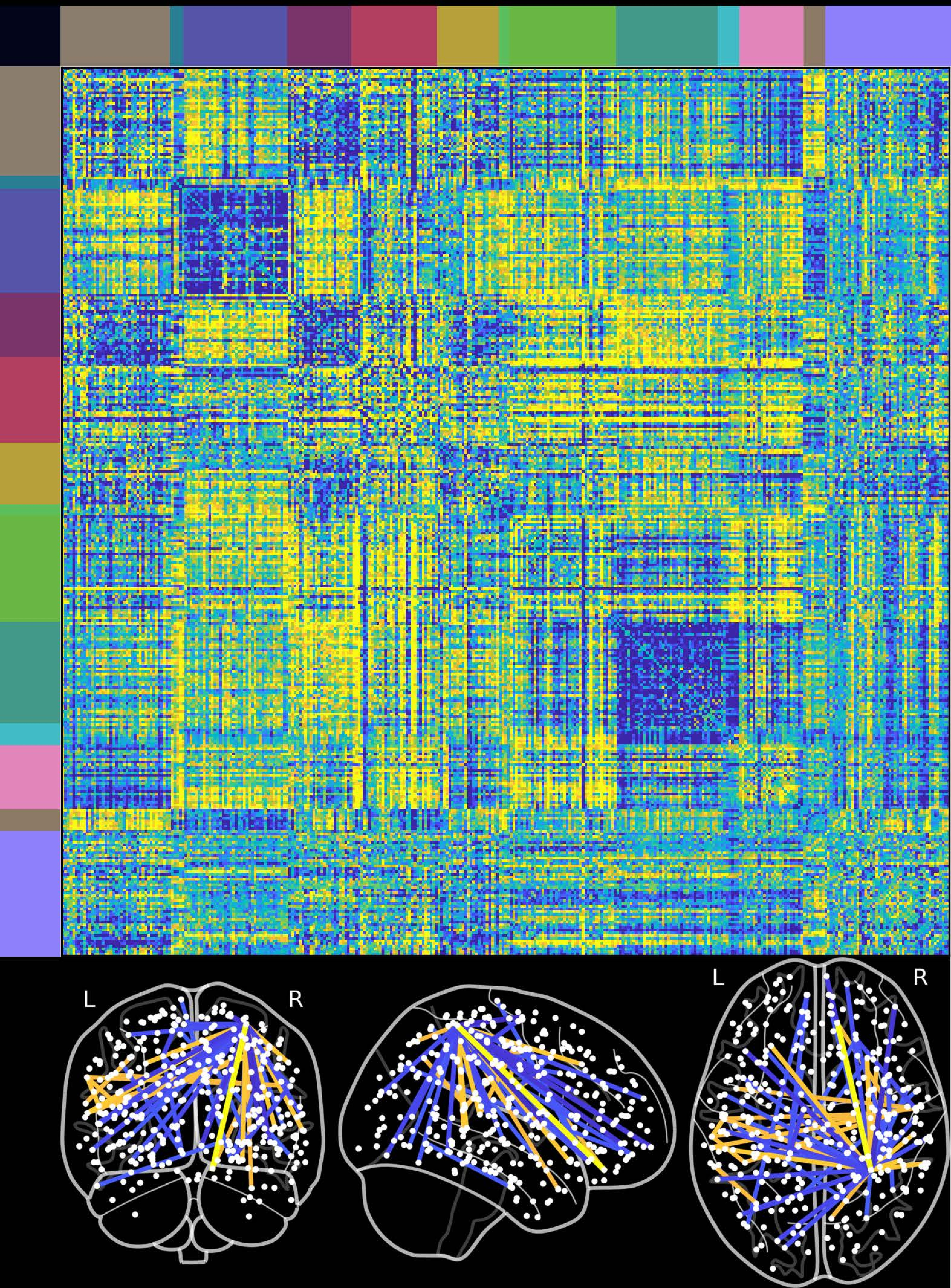

component_9

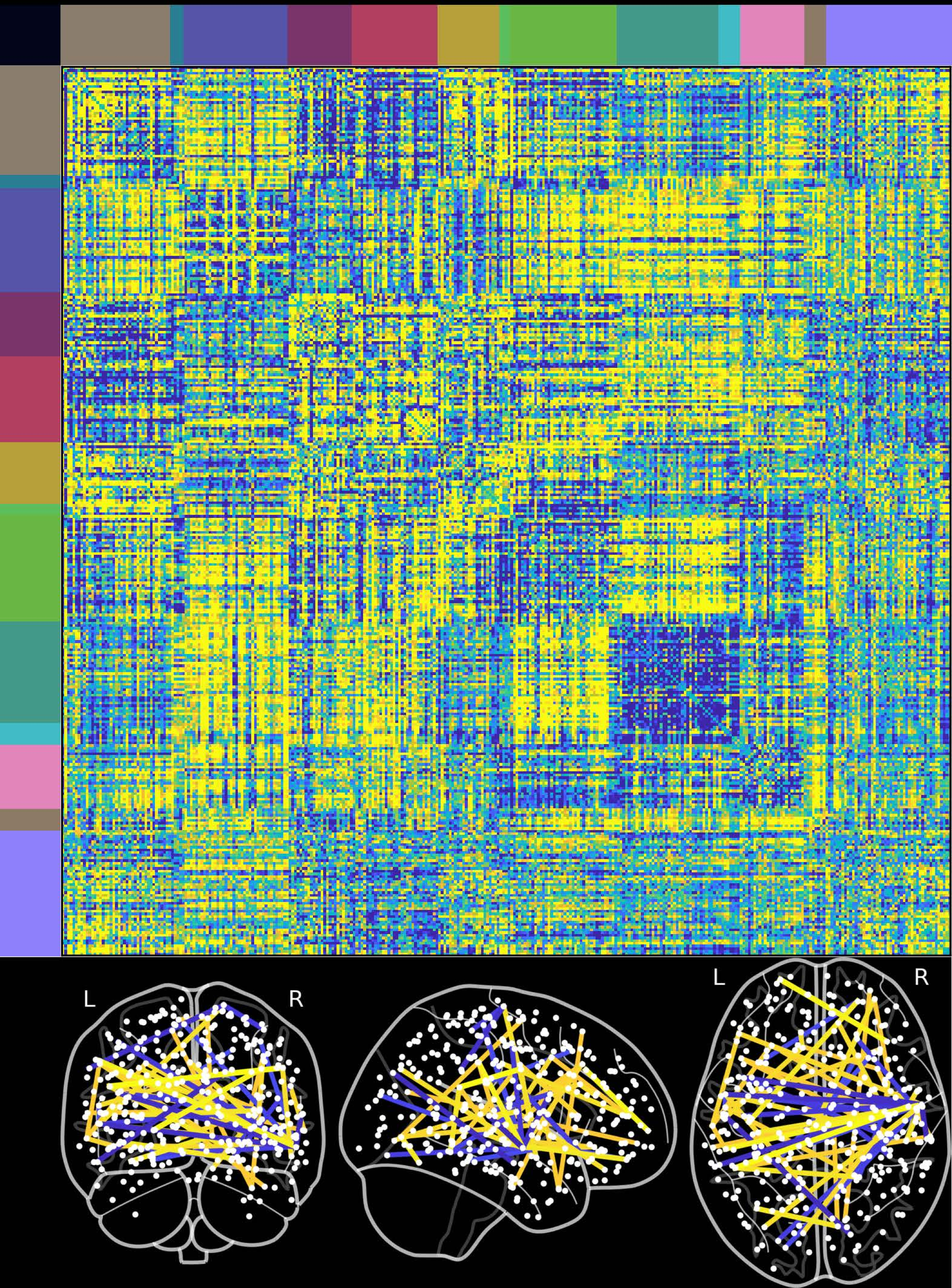

component_10

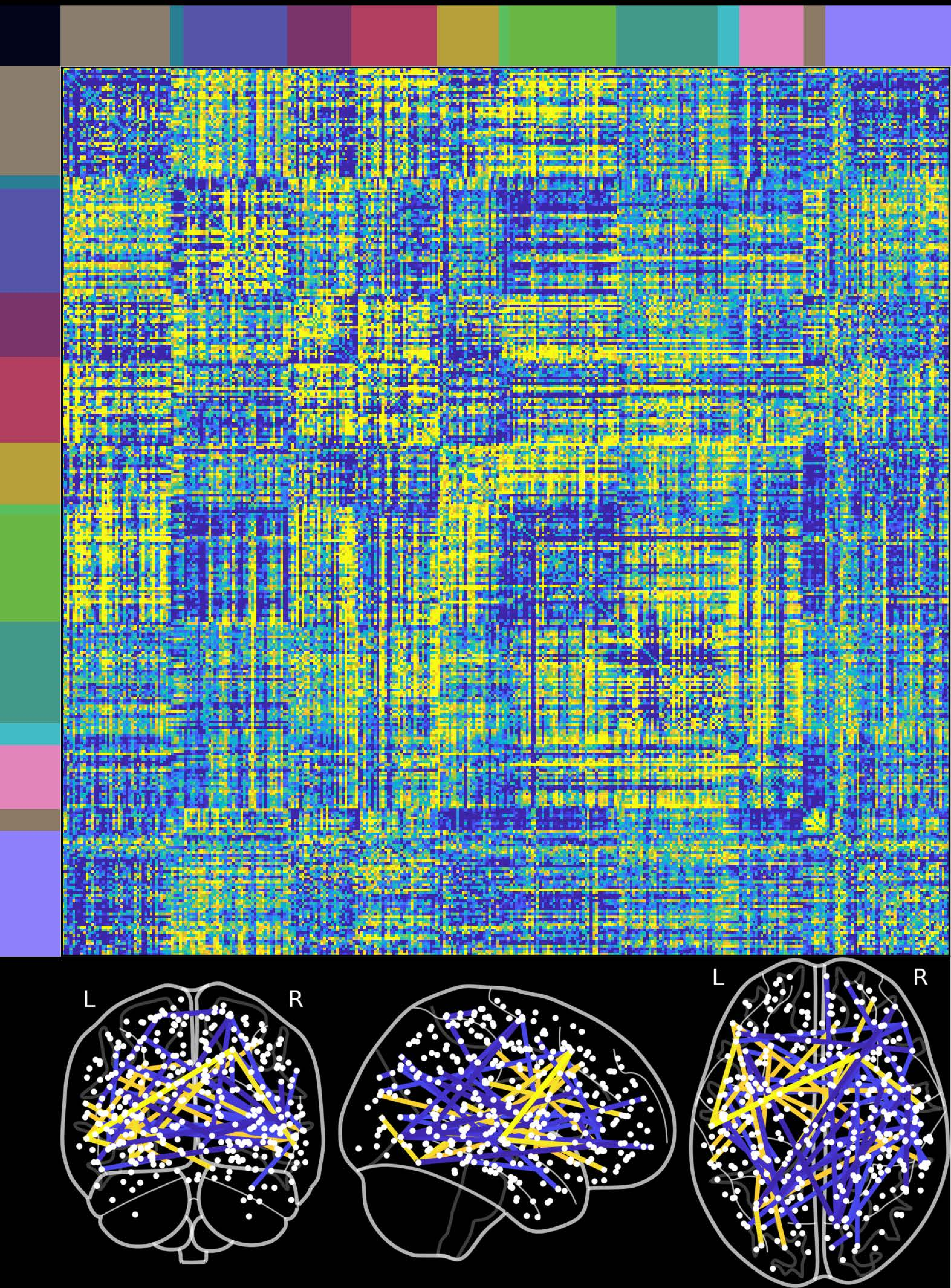

component_11

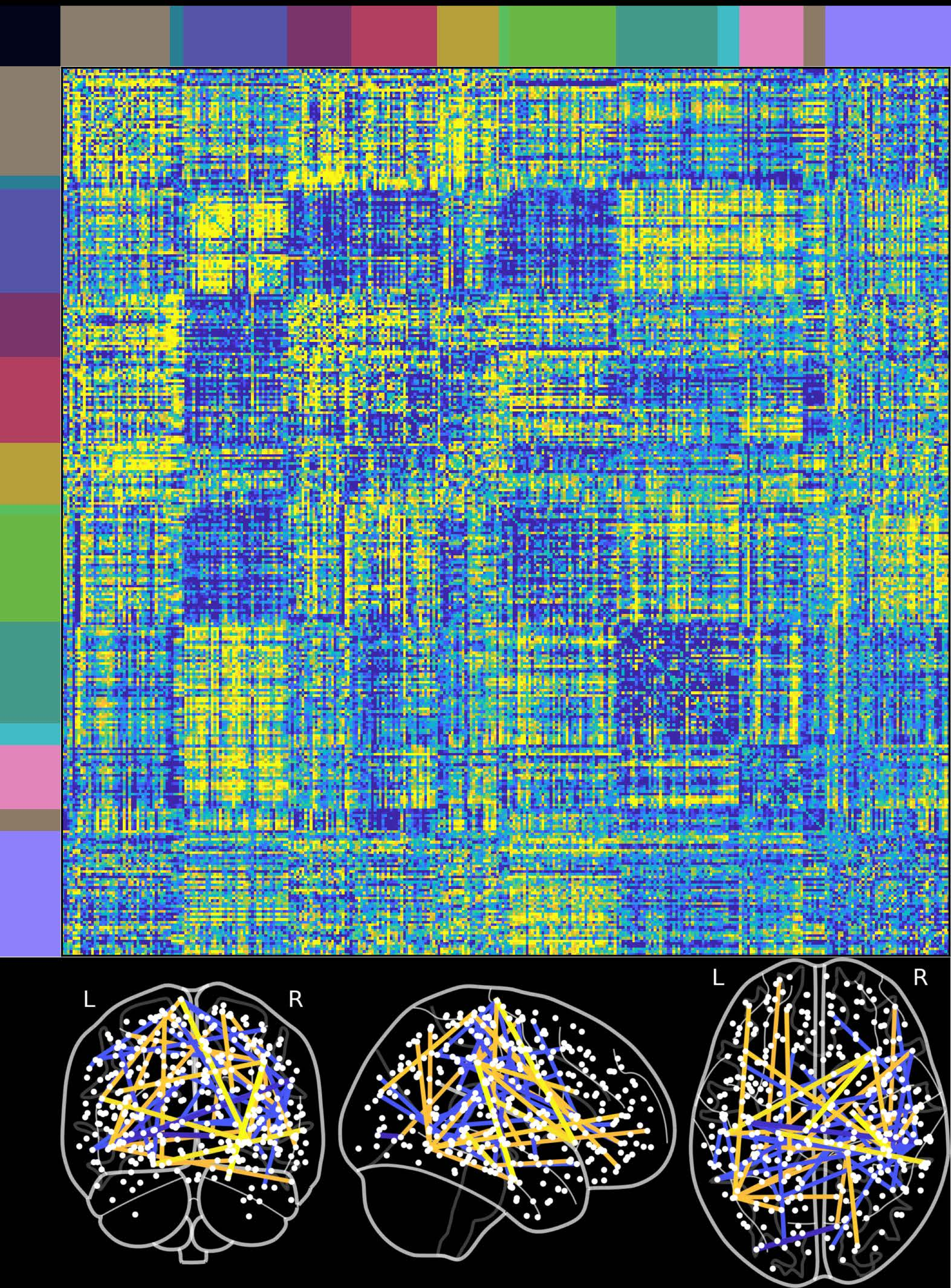

component_12

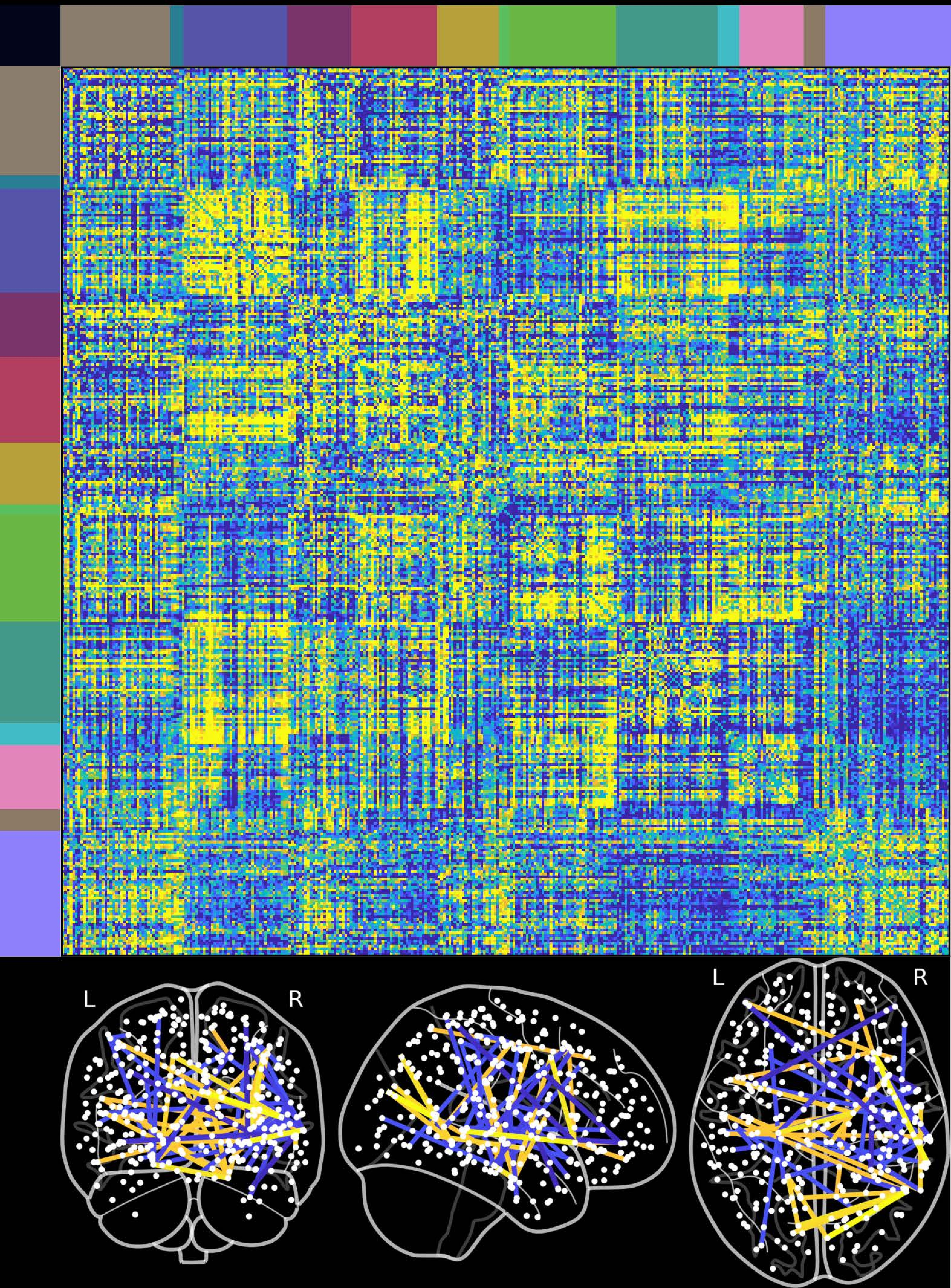

component_13

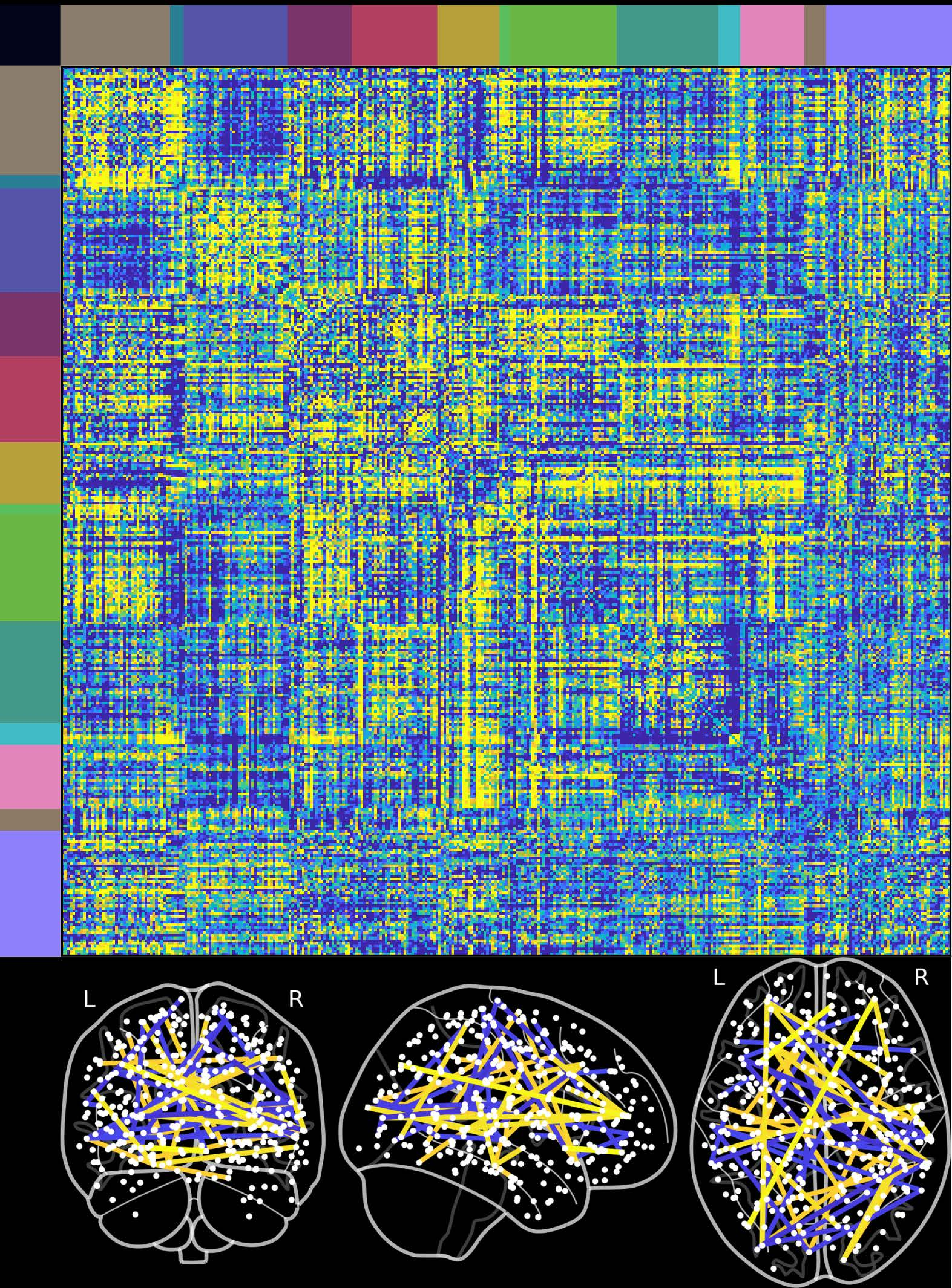

component_14

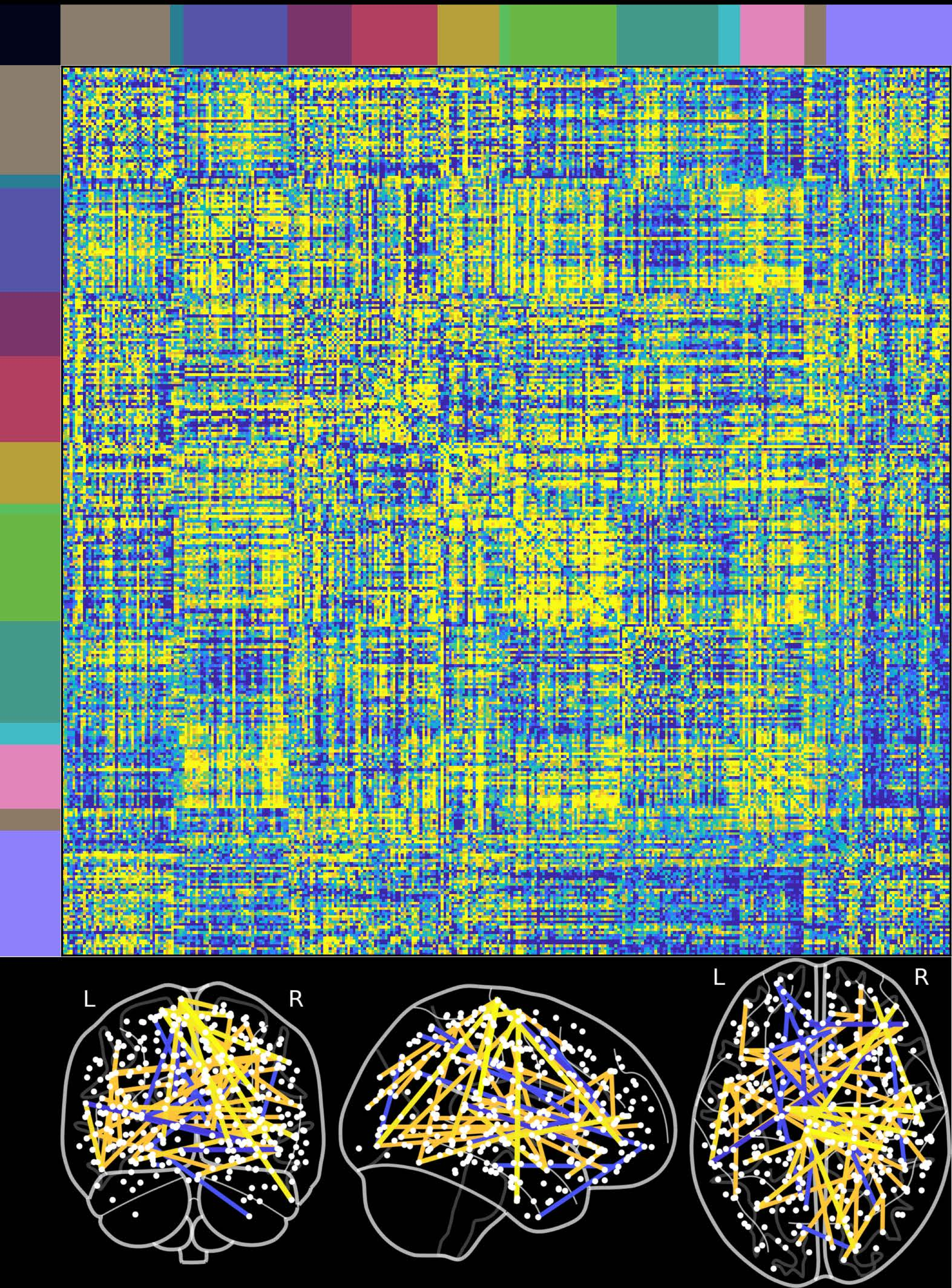

component_15

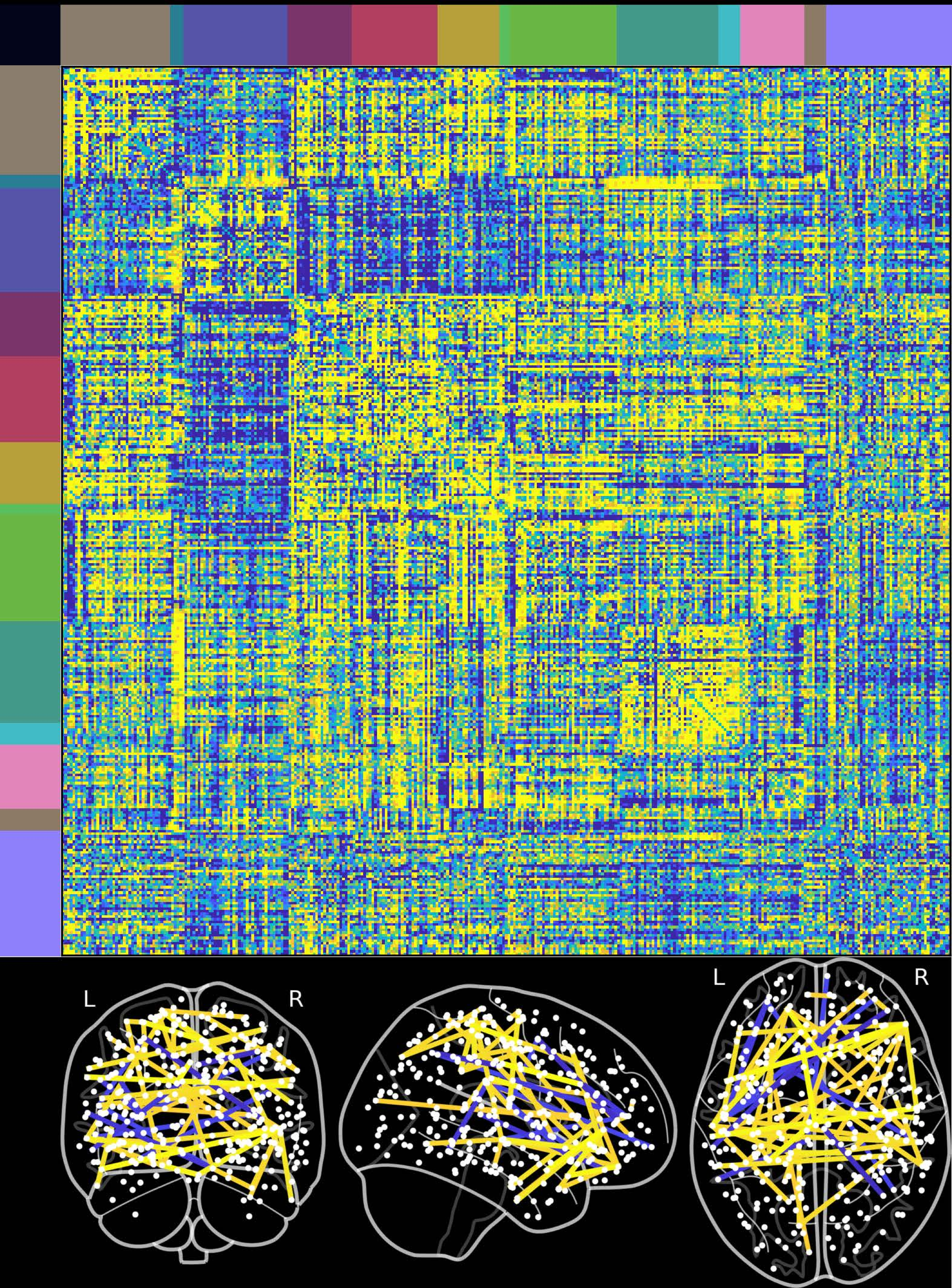

component_16

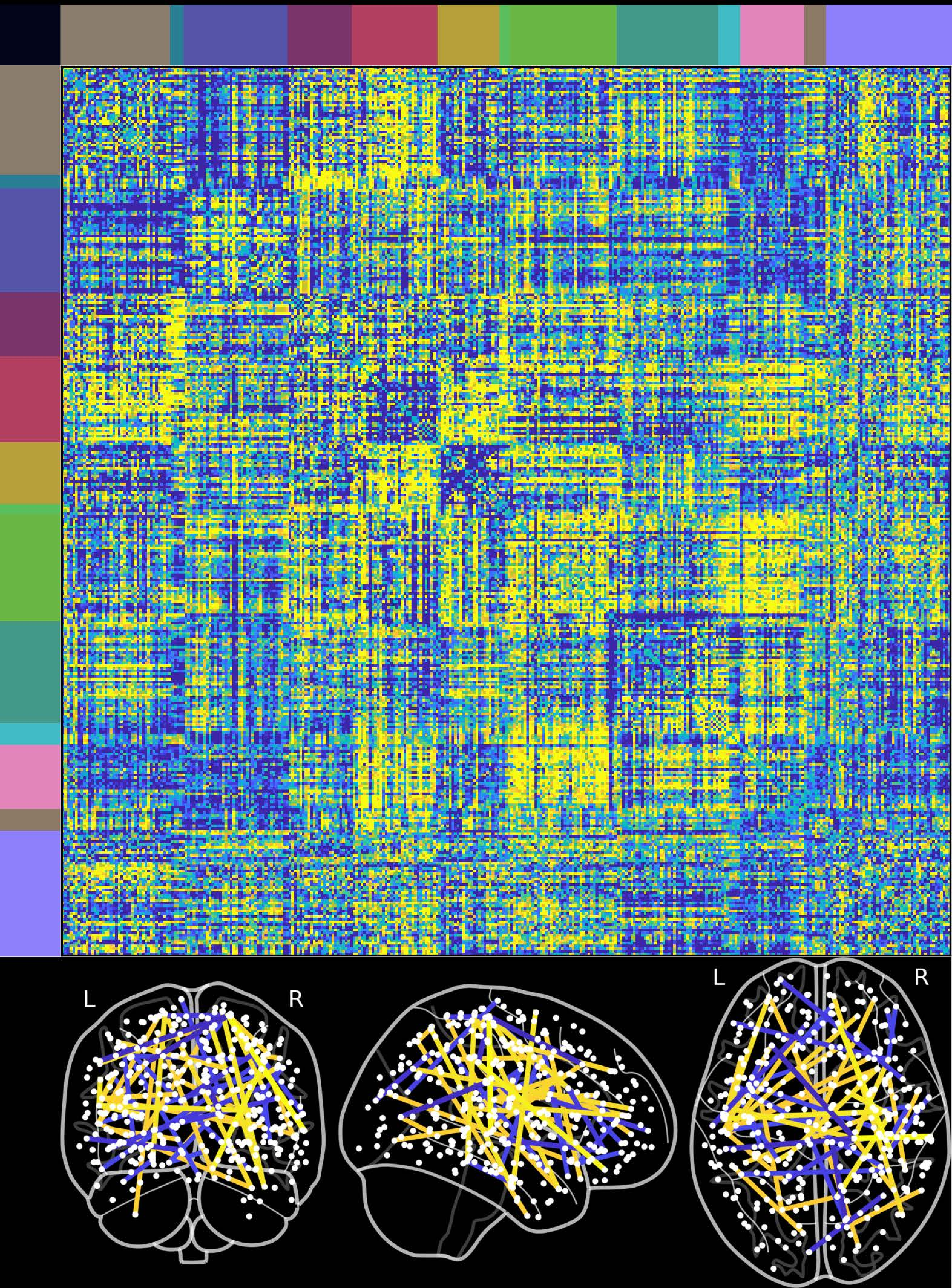

component_17

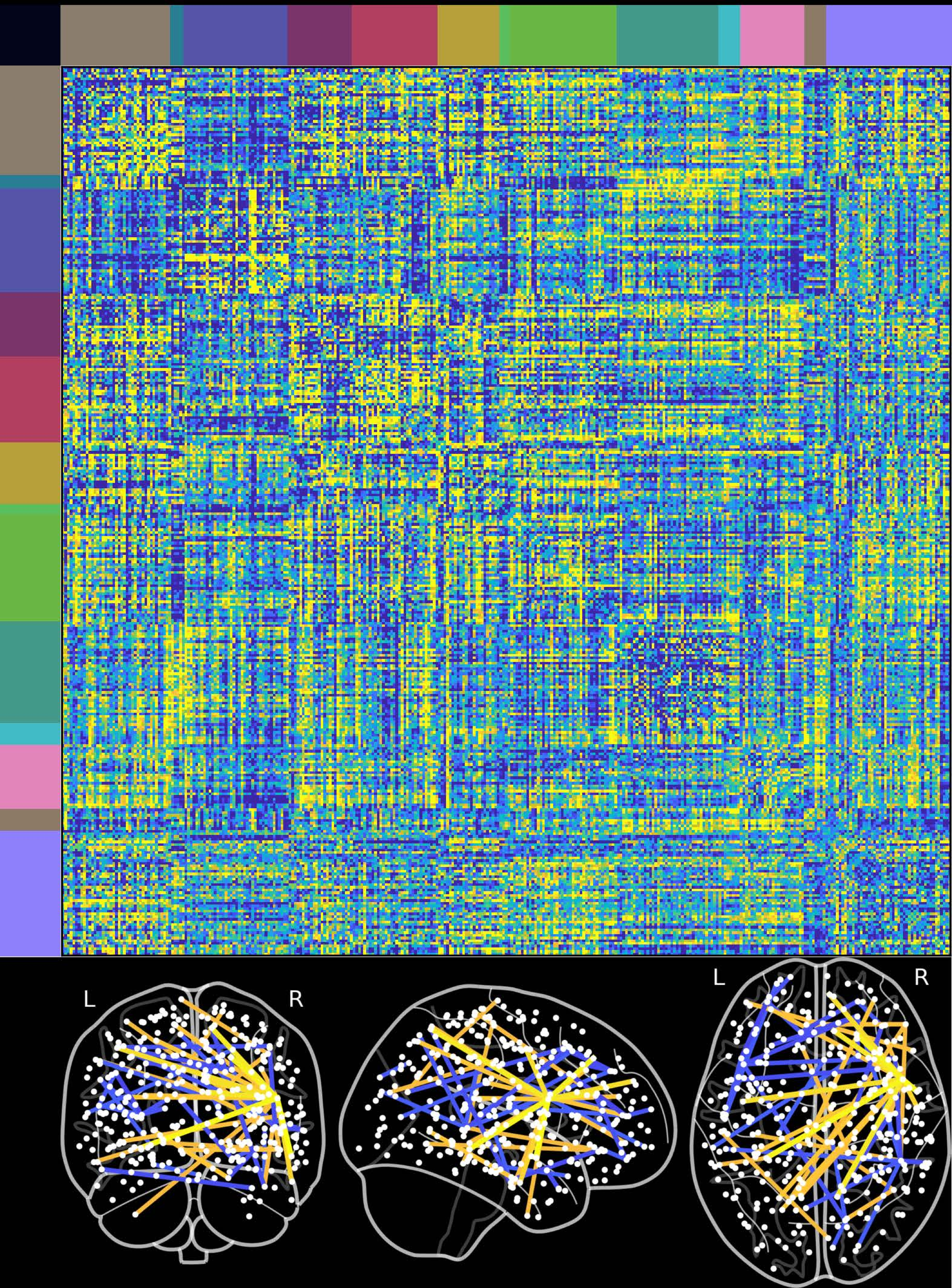

component_18

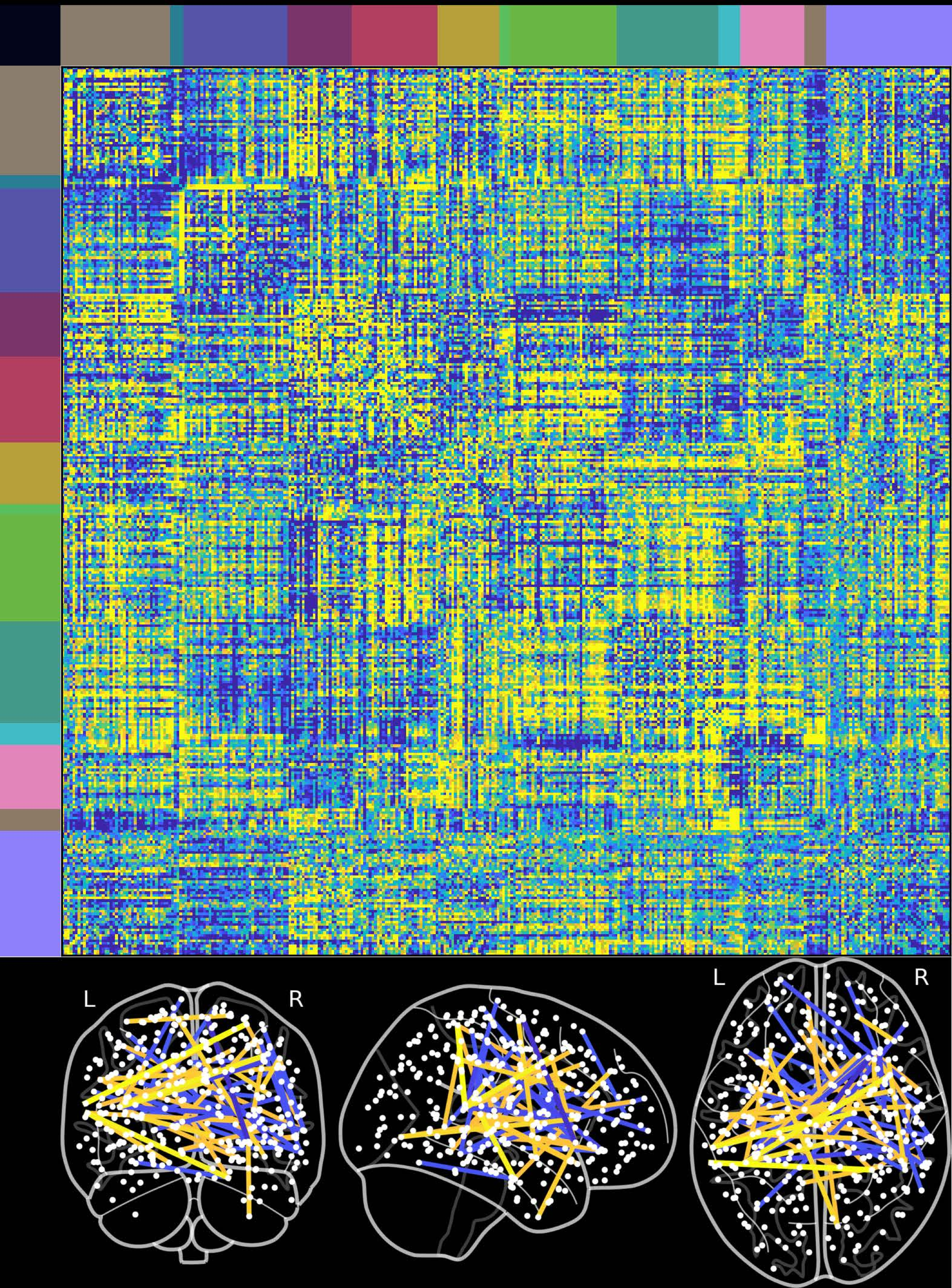

component_19

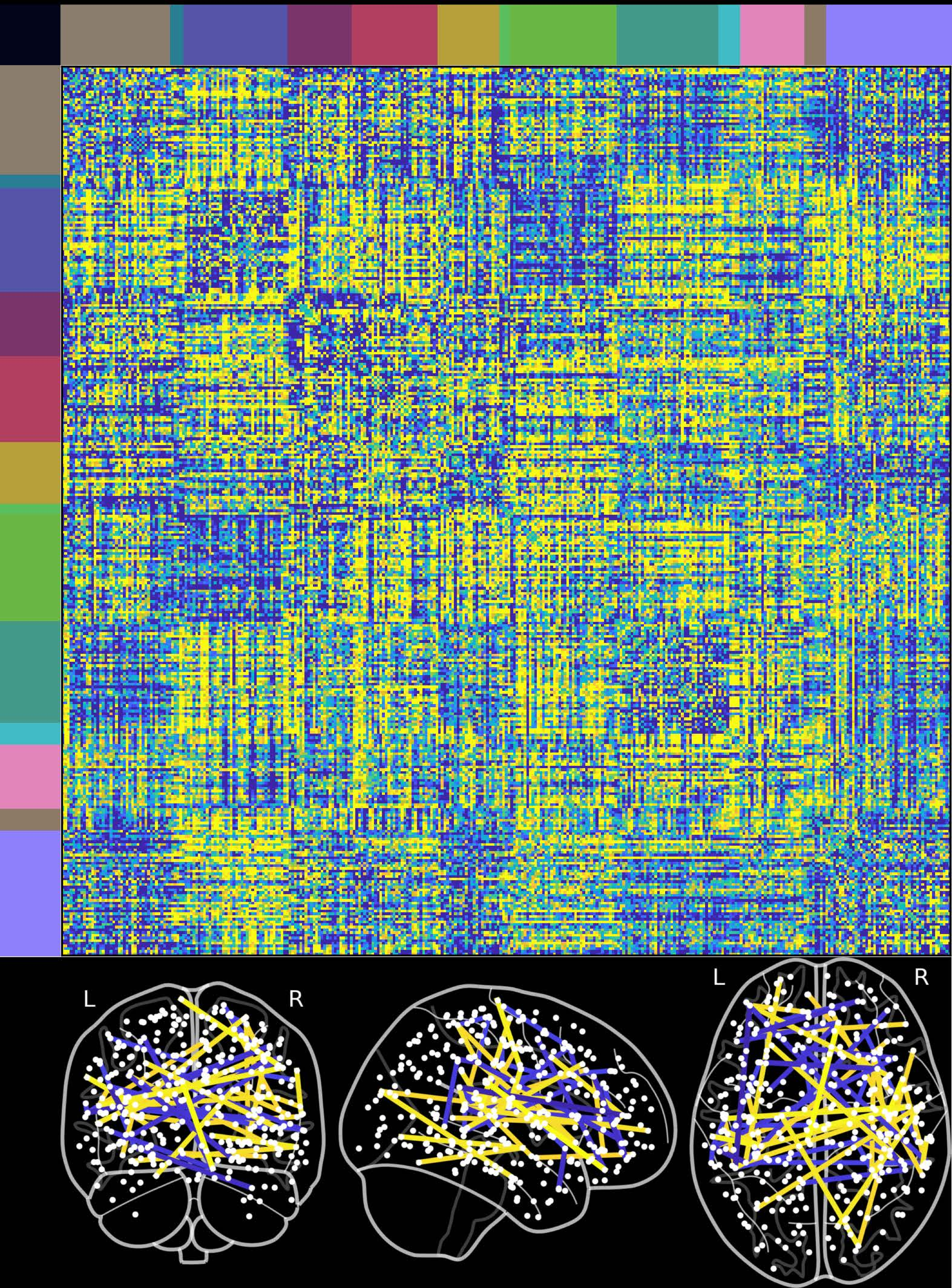

component_20

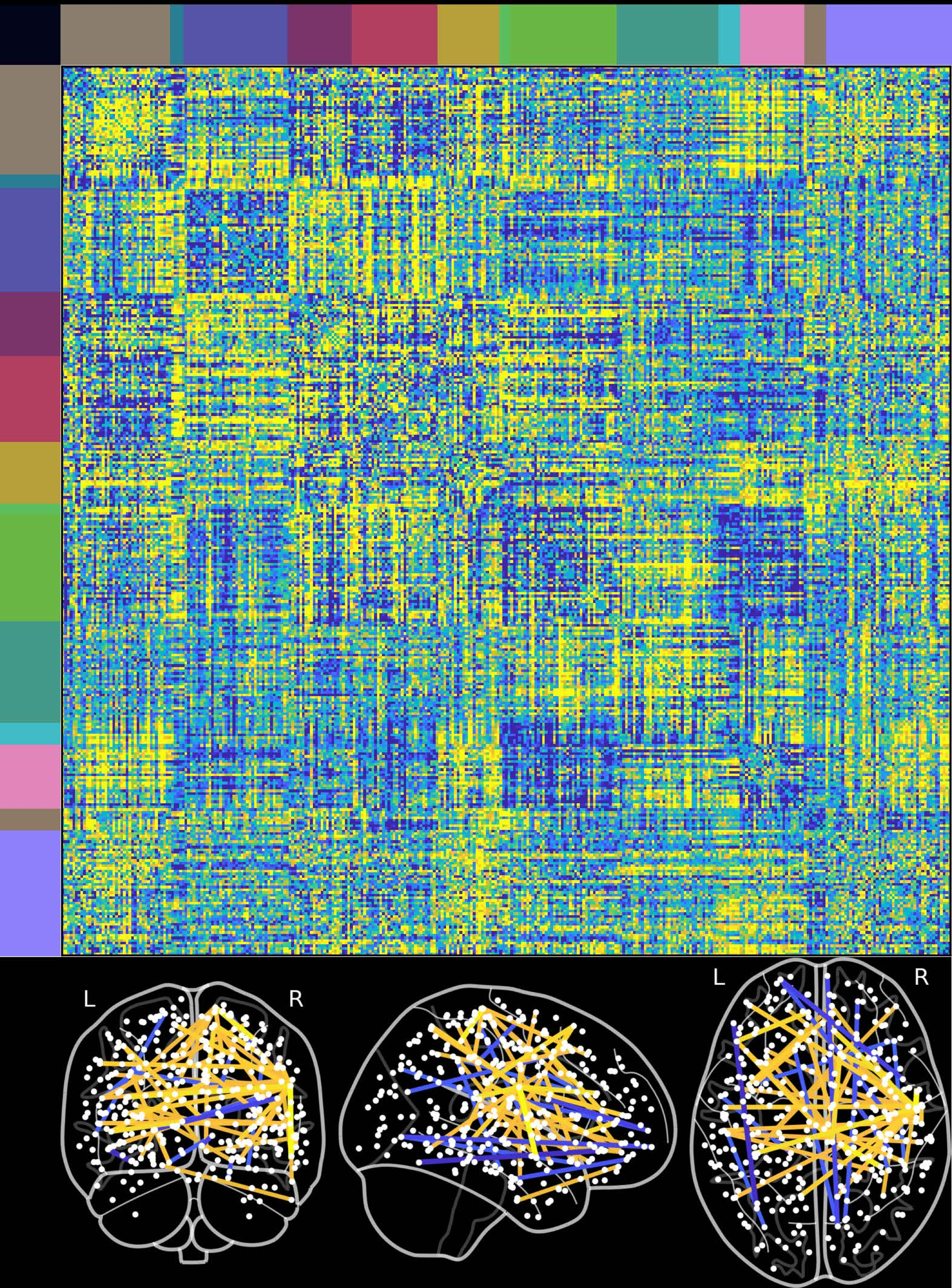

component_21

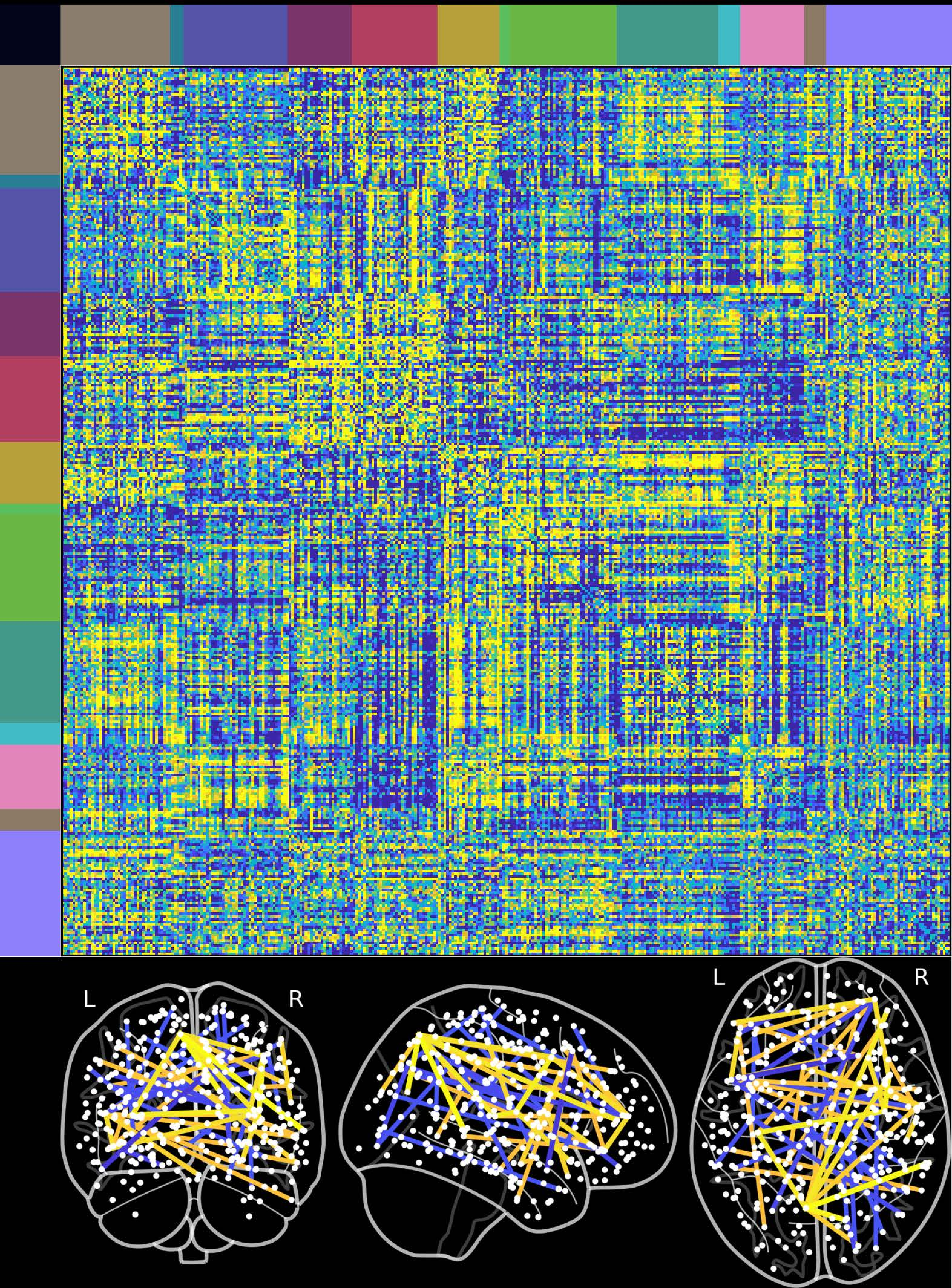

component_22

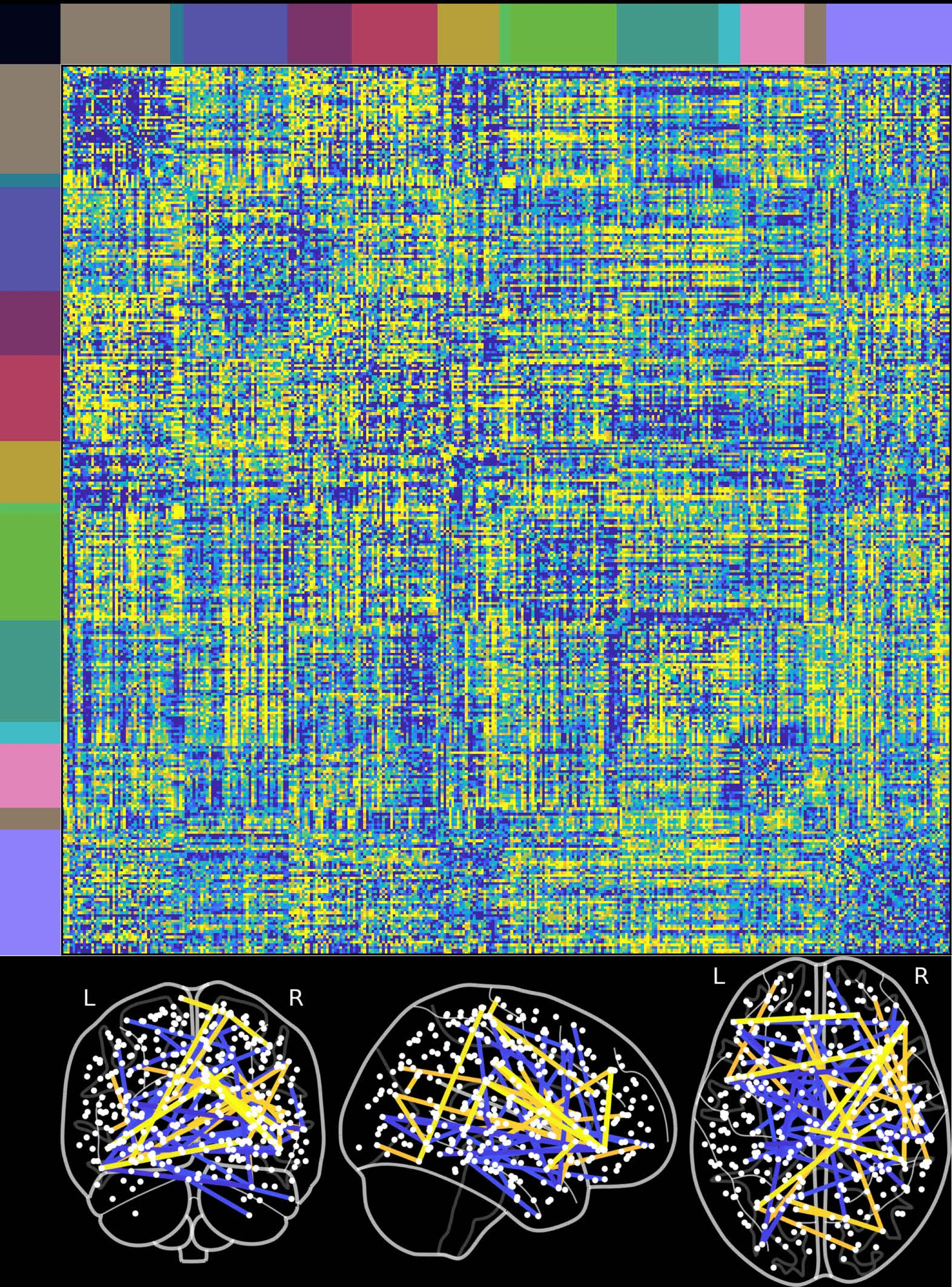

component_23

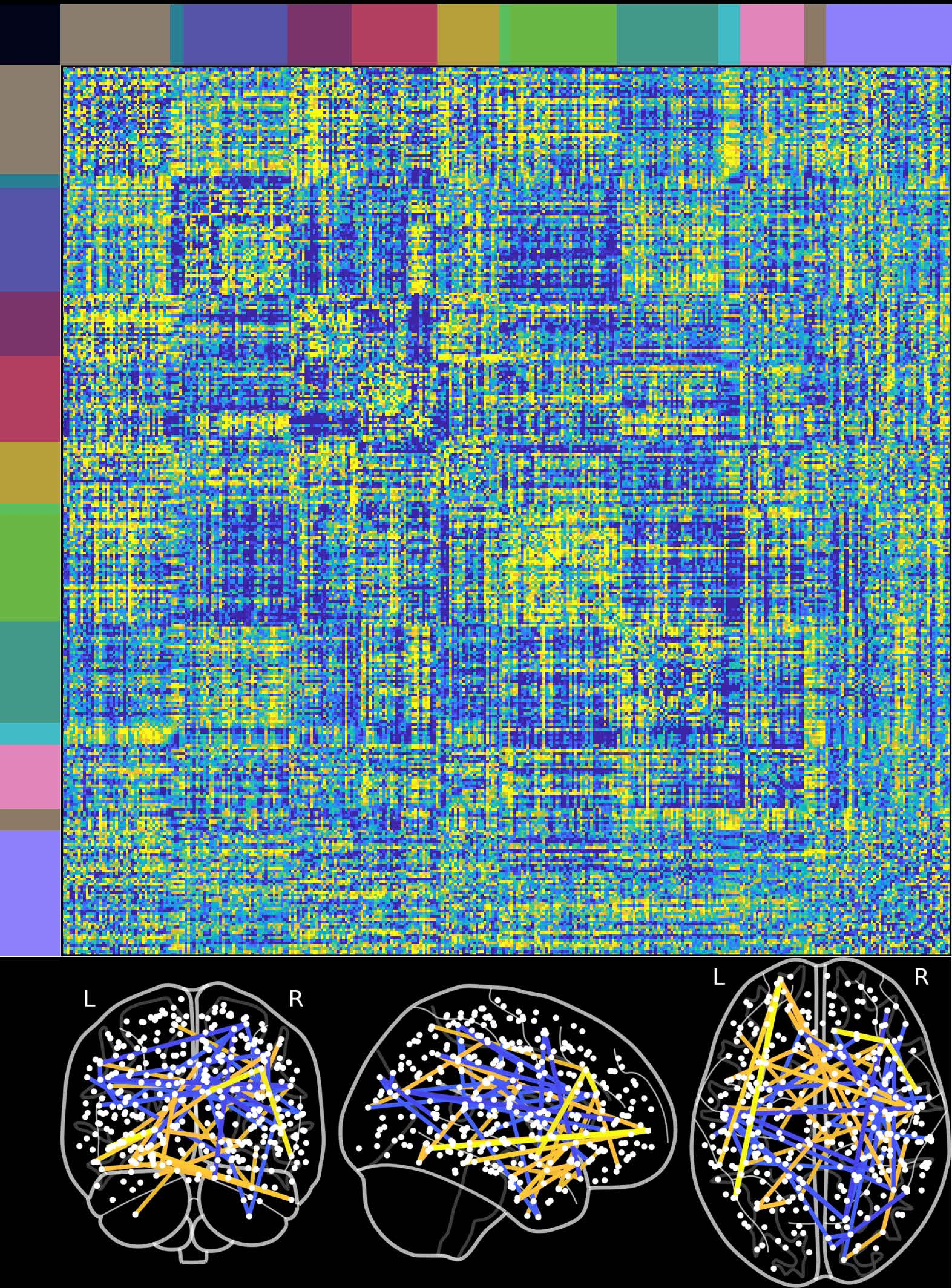

Anxious misery subjects' heatmaps

NDARBB471NAU_madrs_left_dlpfc

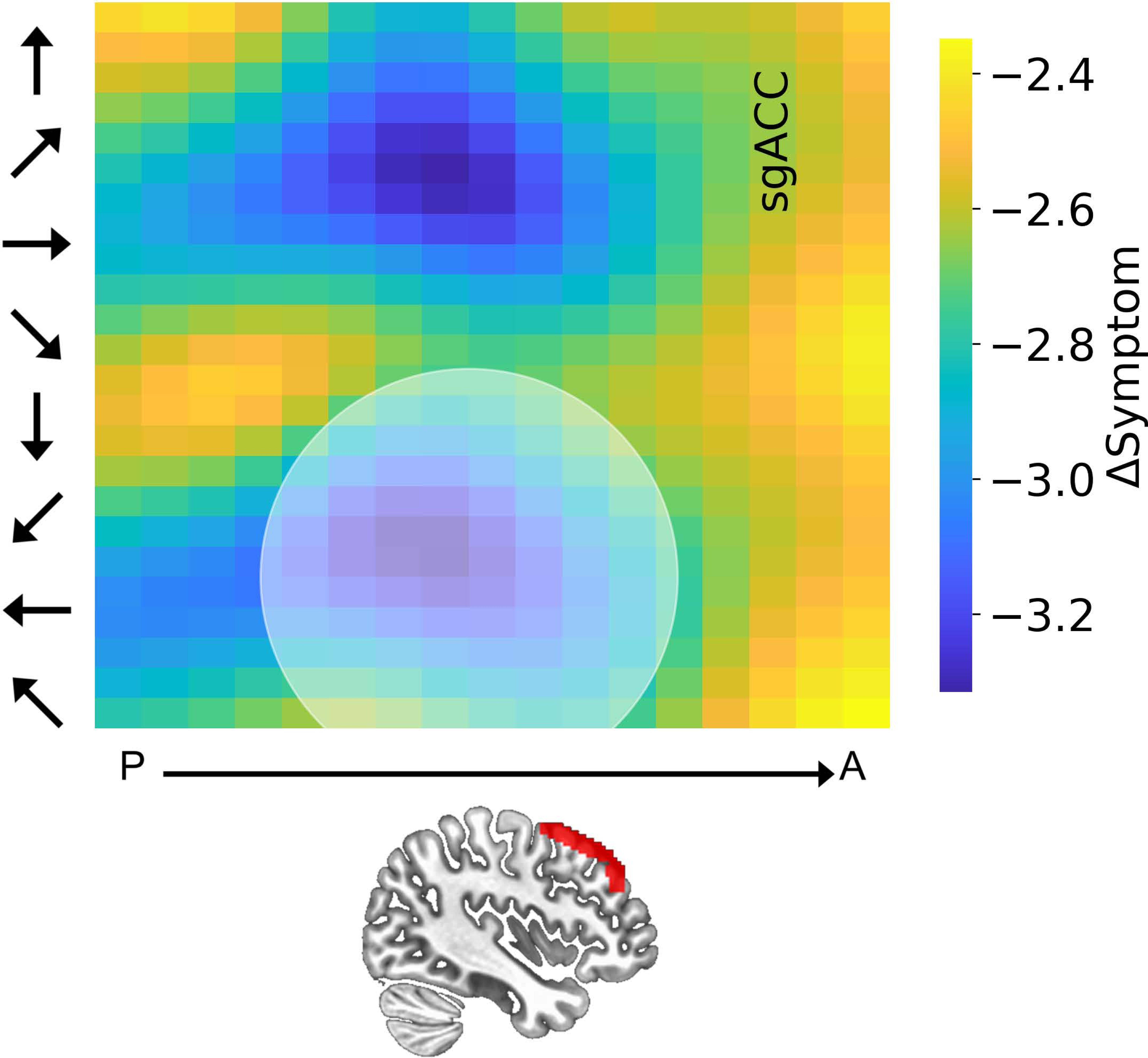

NDARBG497RE1_madrs_left_dlpfc

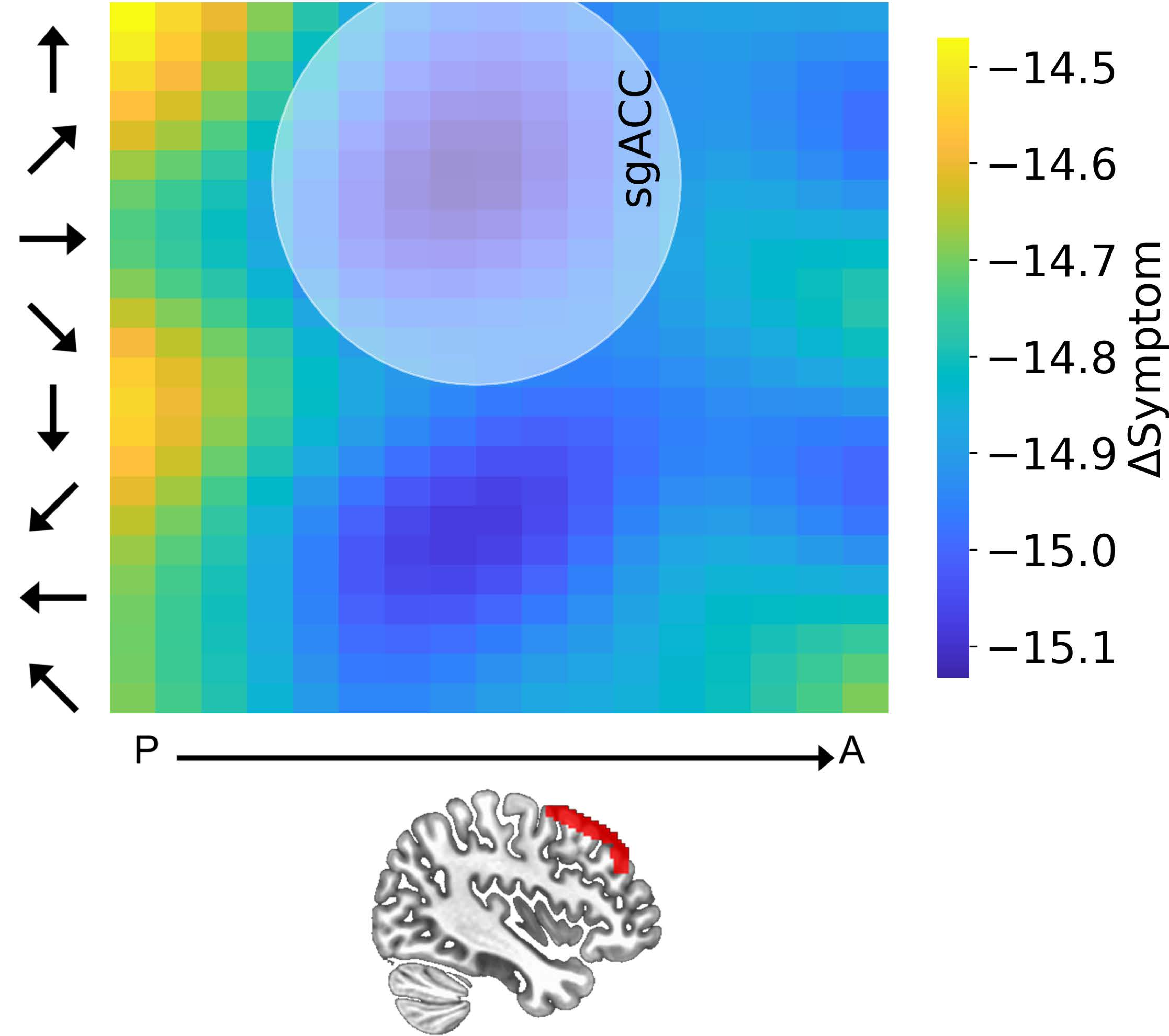

NDARBK027PDF_madrs_left_dlpfc

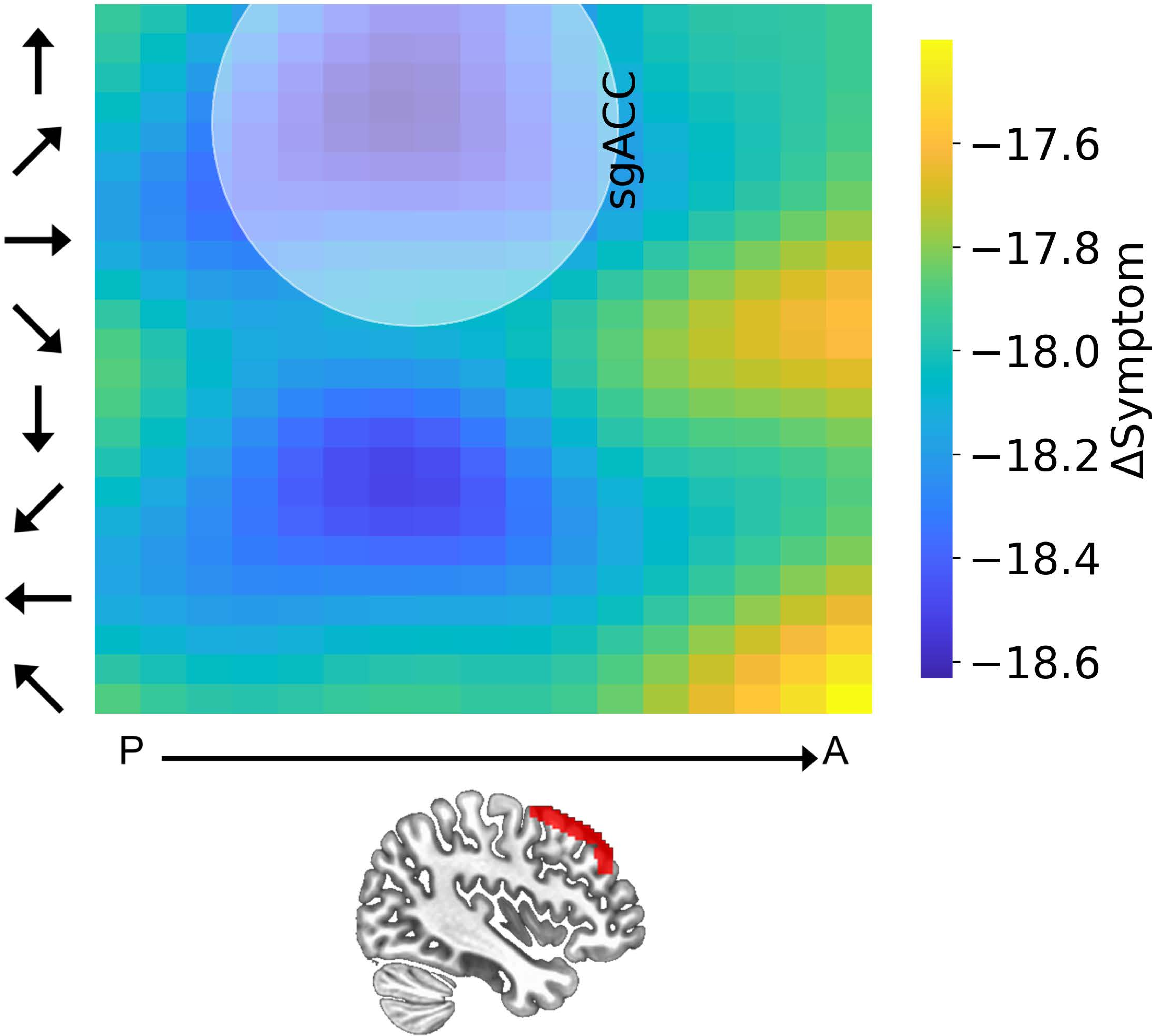

NDARBL814BJK_madrs_left_dlpfc

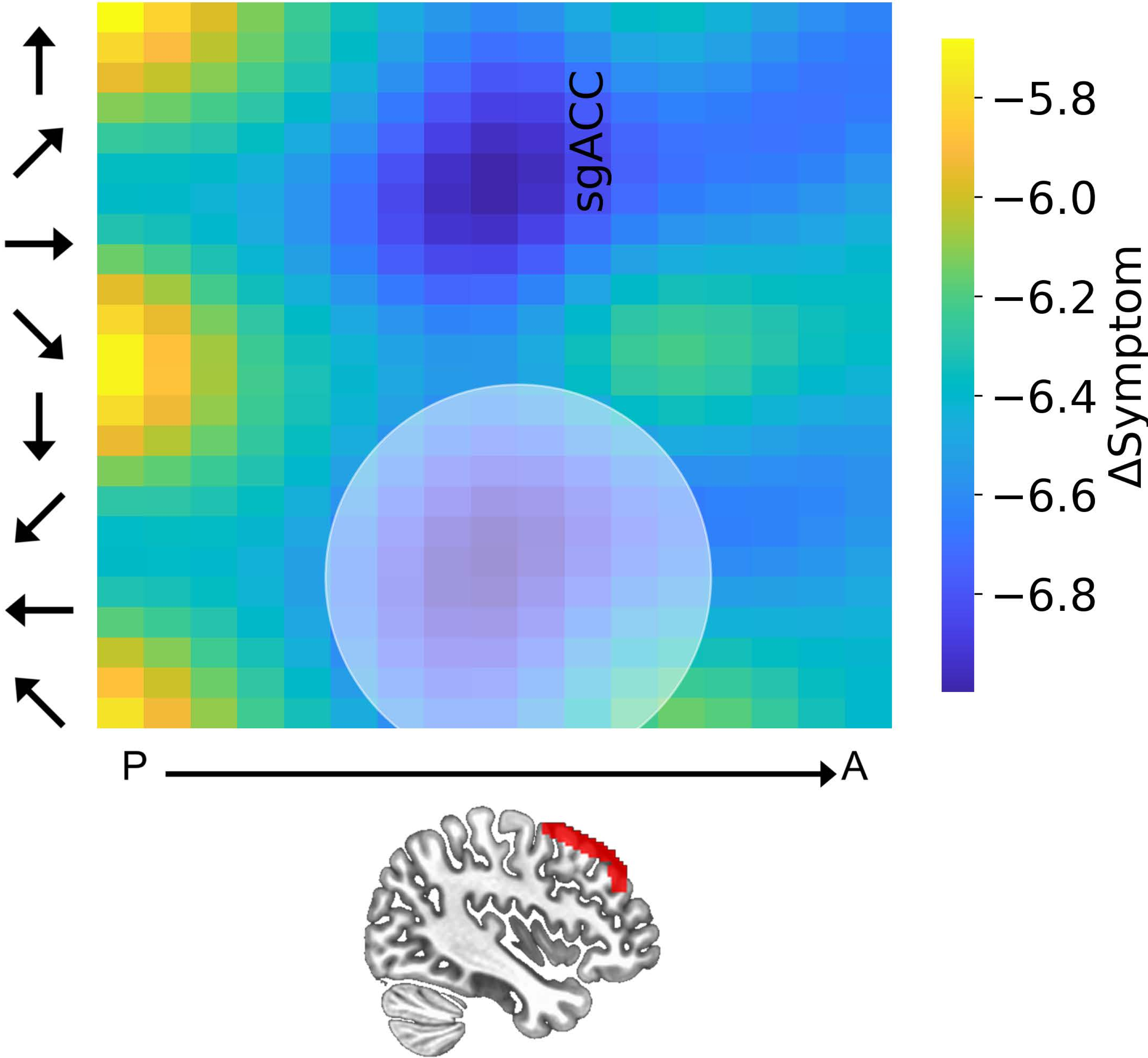

NDARBP353XD2_madrs_left_dlpfc

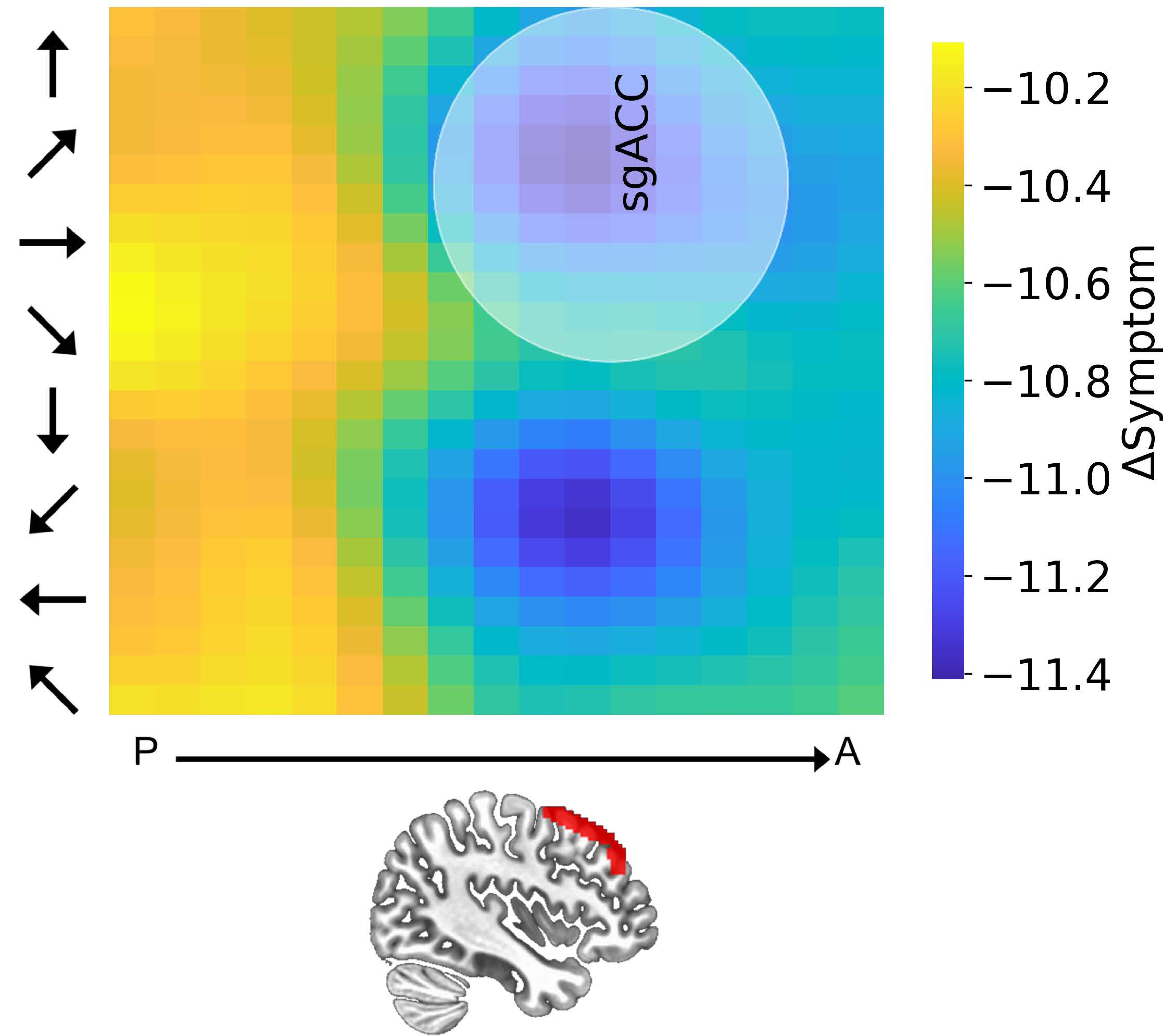

NDARBT583XH6_madrs_left_dlpfc

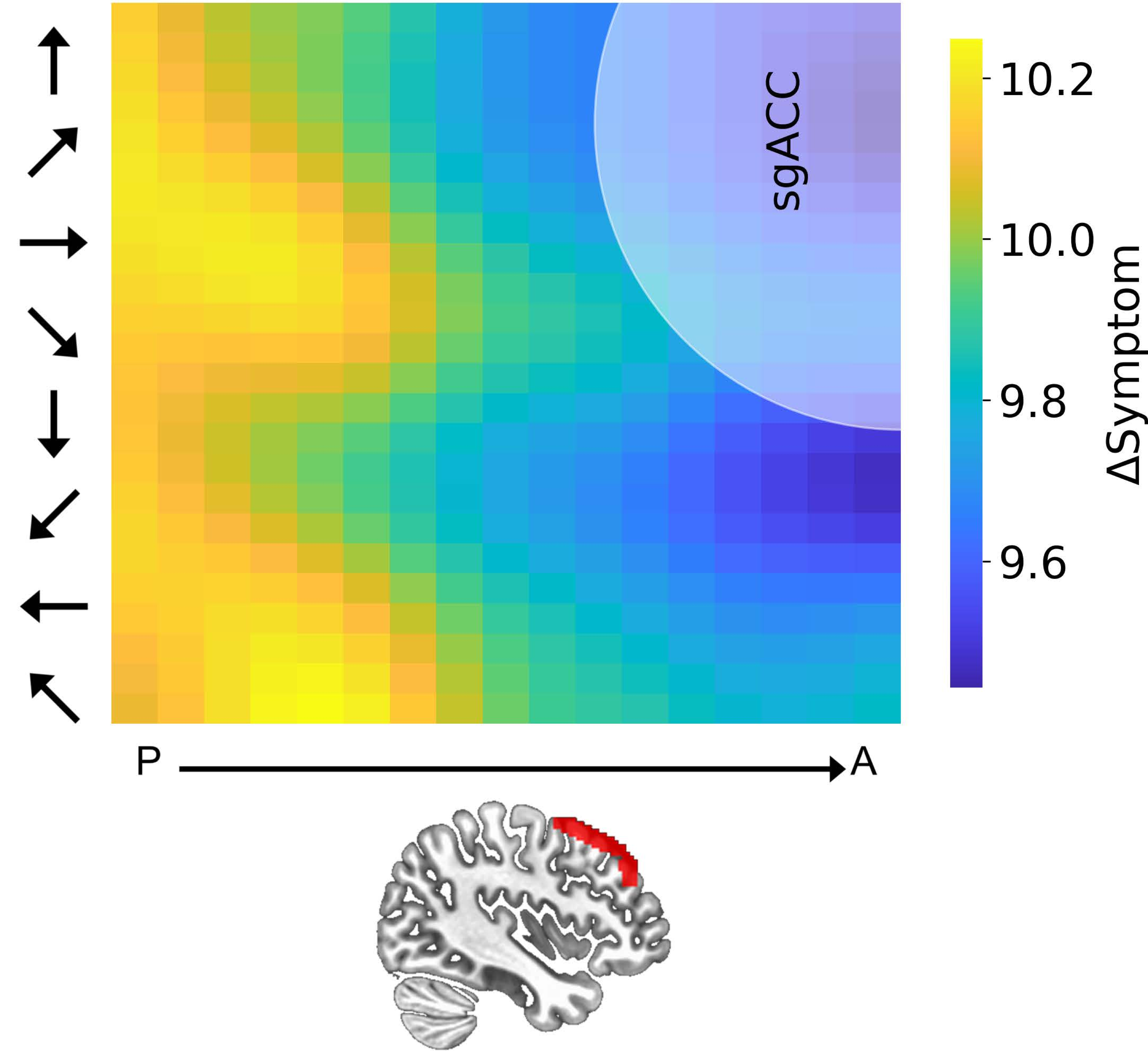

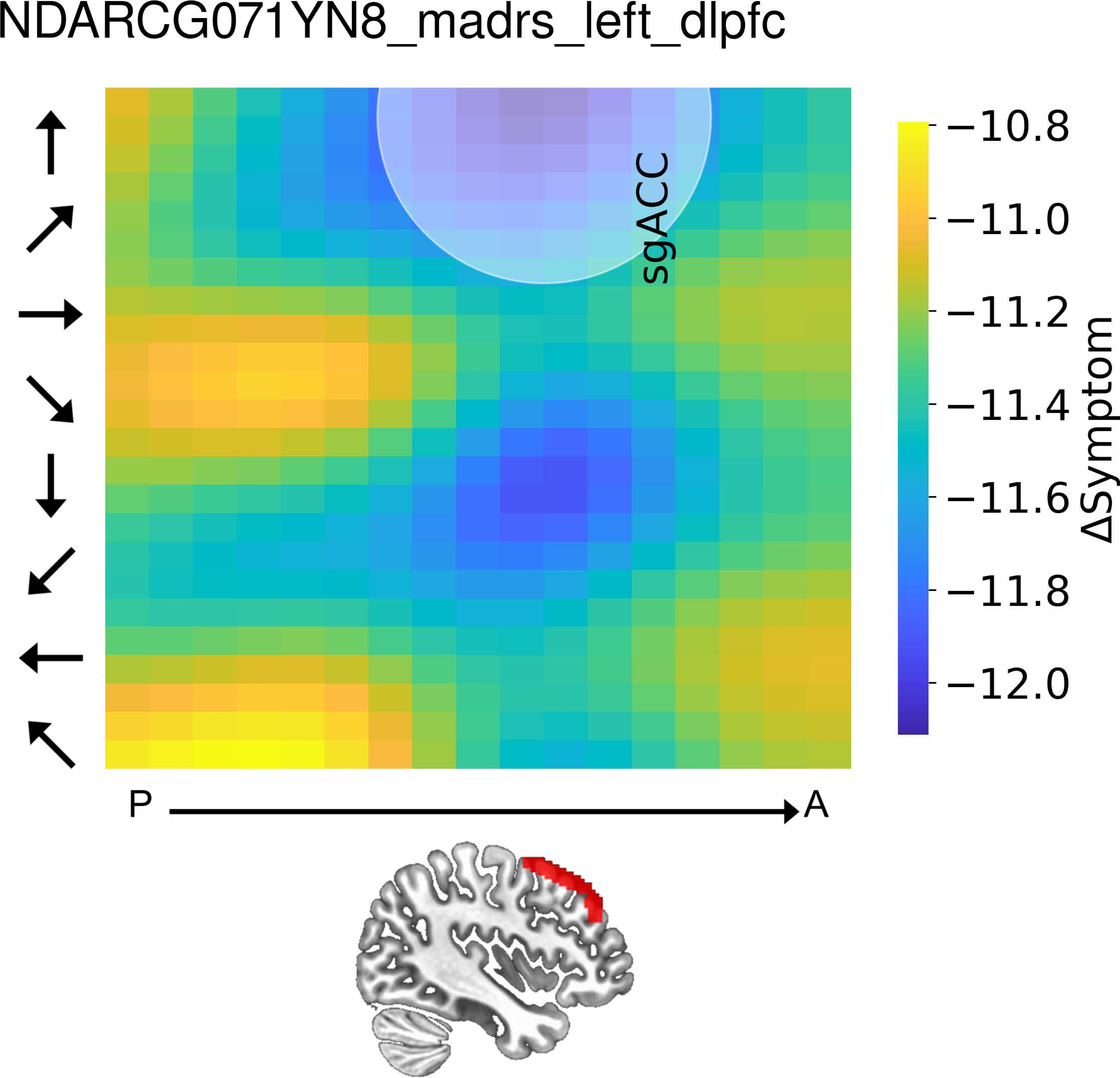
NDARCG071YN8_madrs_left_dlpfc
sgACC
−10.8
−11.0
−11.2
−11.4
−11.6
−11.8
−12.0
ΔSymptom
P
A

NDARCG271VGC_madrs_left_dlpfc

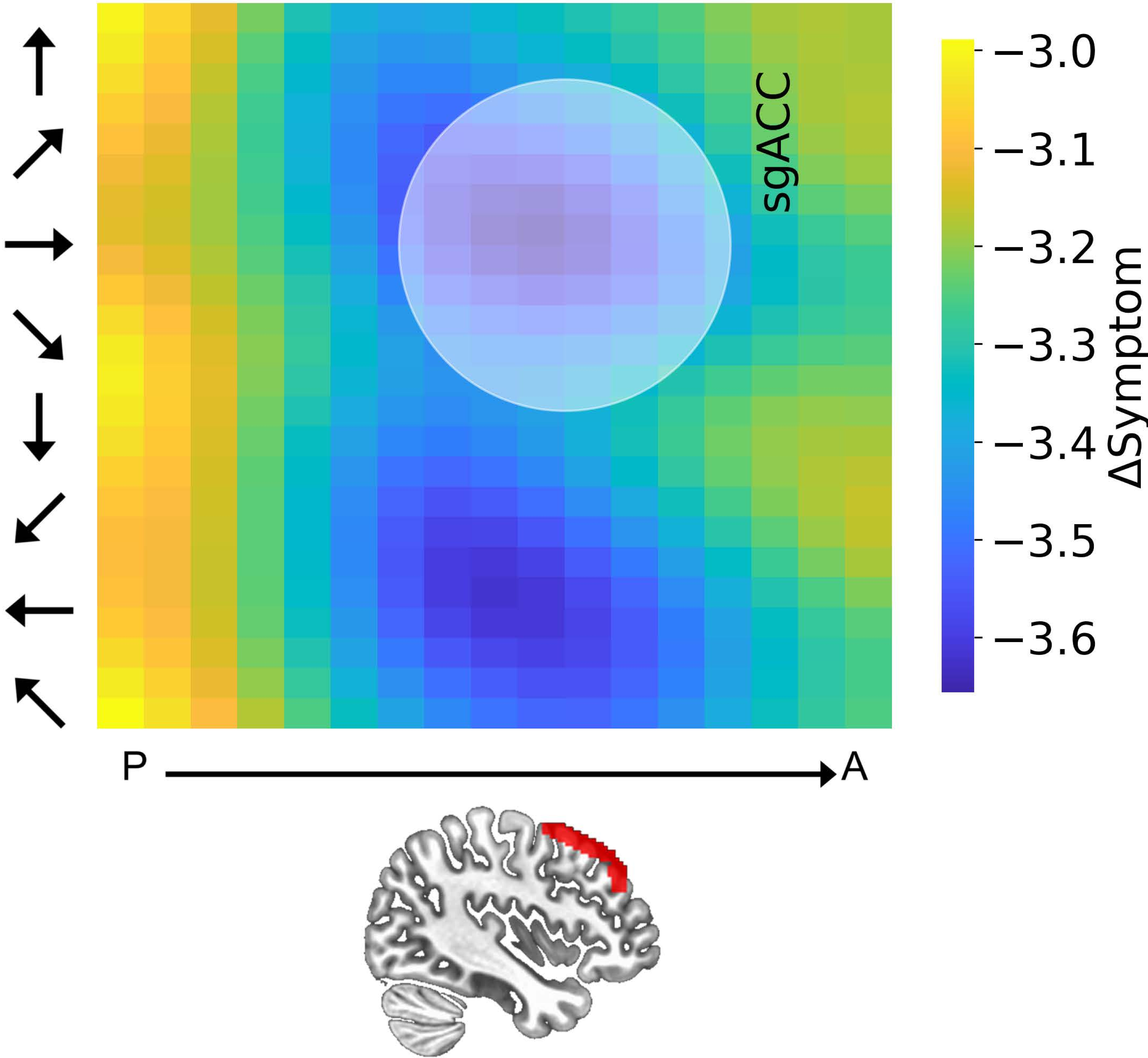

NDARCM769WZL_madrs_left_dlpfc

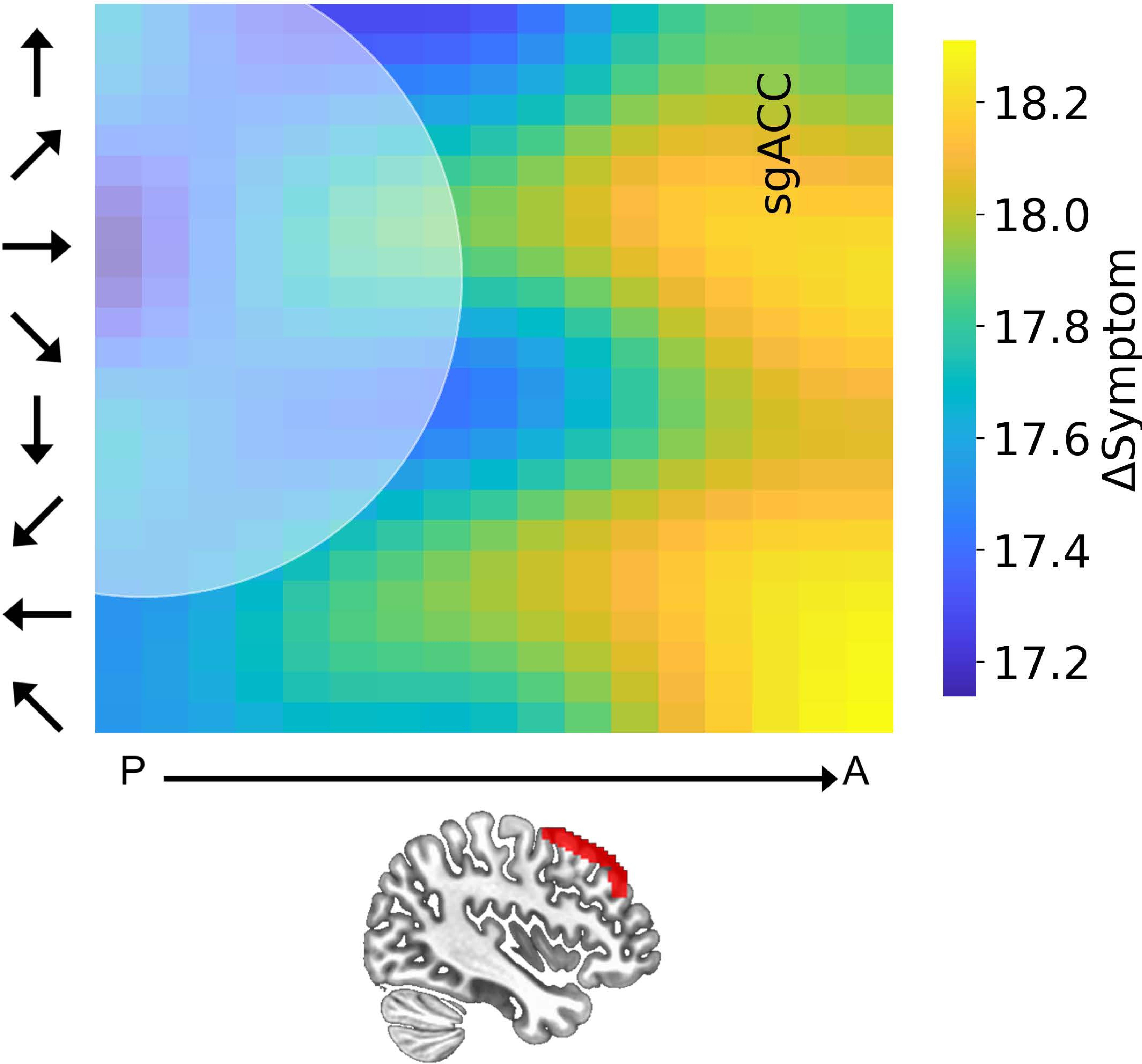

NDARDL291XT0_madrs_left_dlpfc

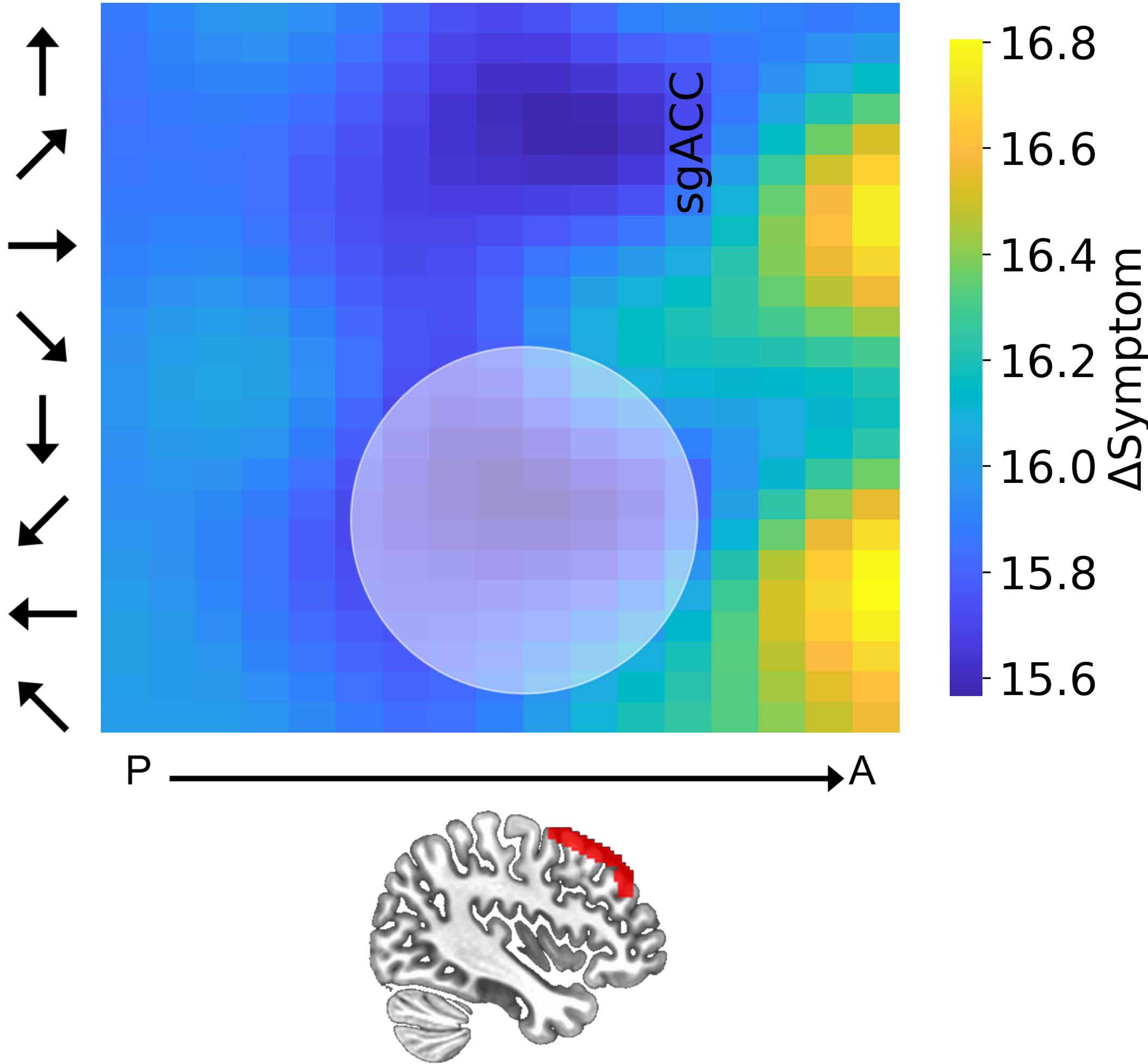

NDARDL667VMW_madrs_left_dlpfc

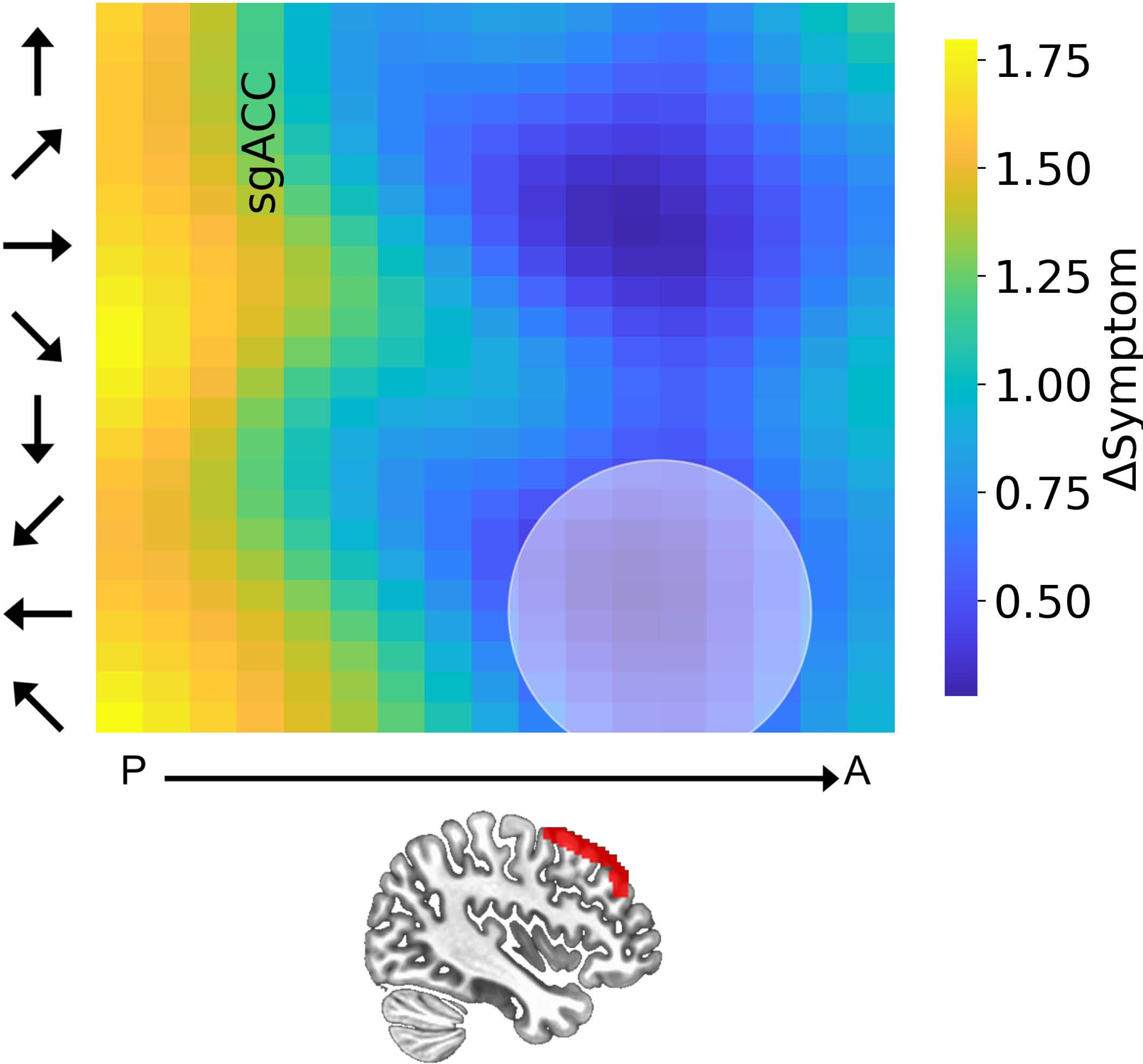

NDARDR017YX2_madrs_left_dlpfc

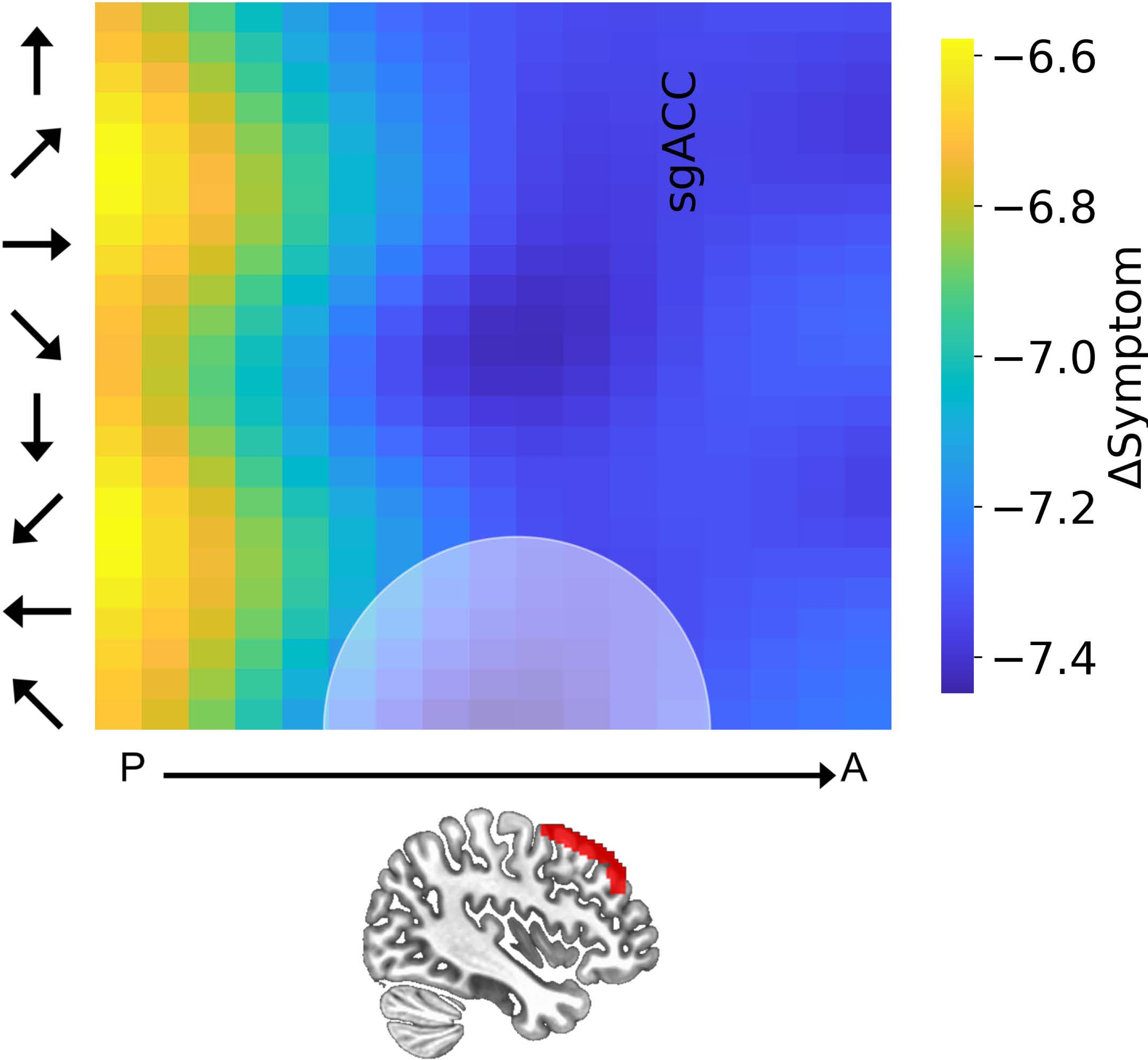

NDARDU203PAW_madrs_left_dlpfc

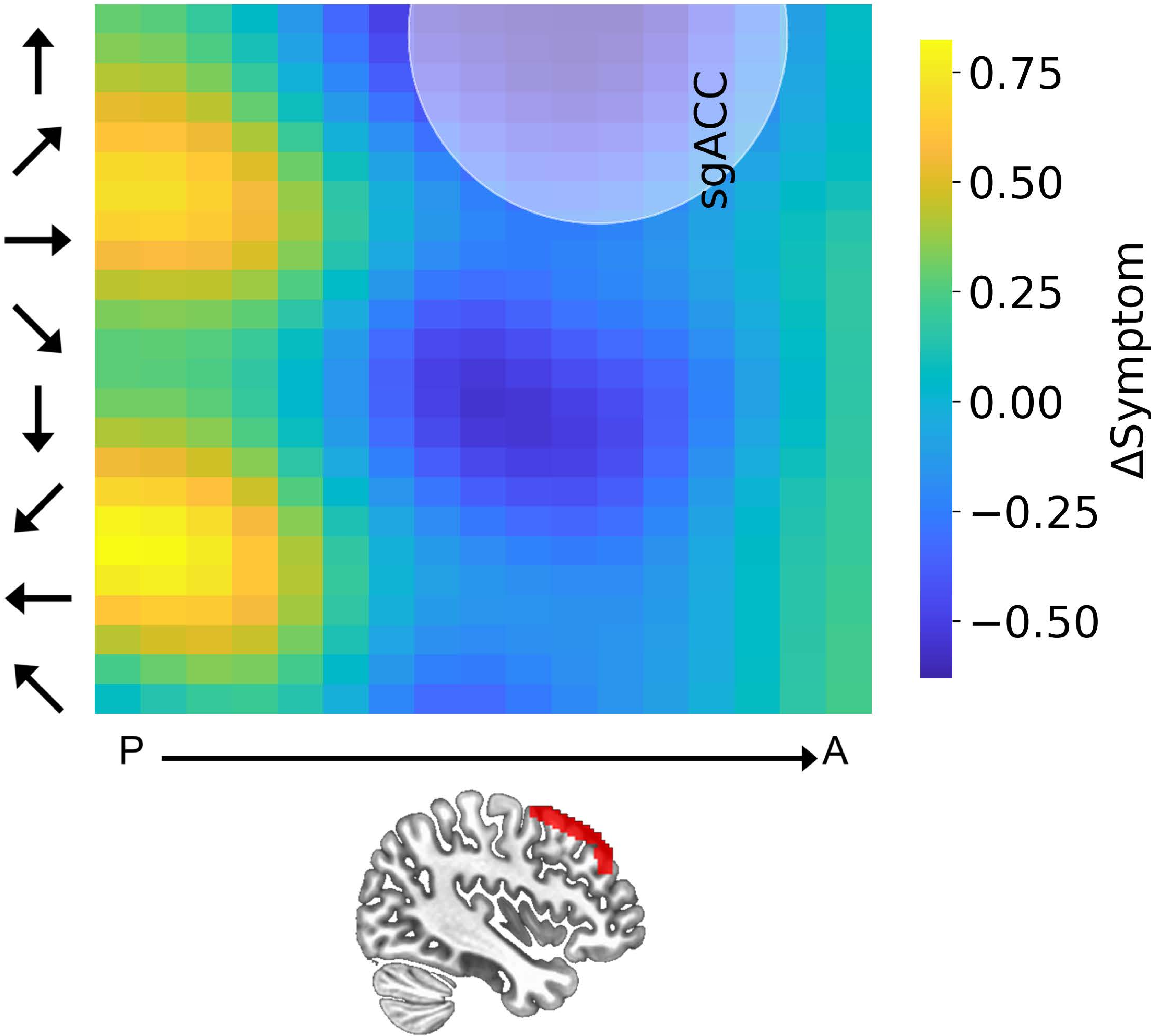

NDARDU282NKX_madrs_left_dlpfc

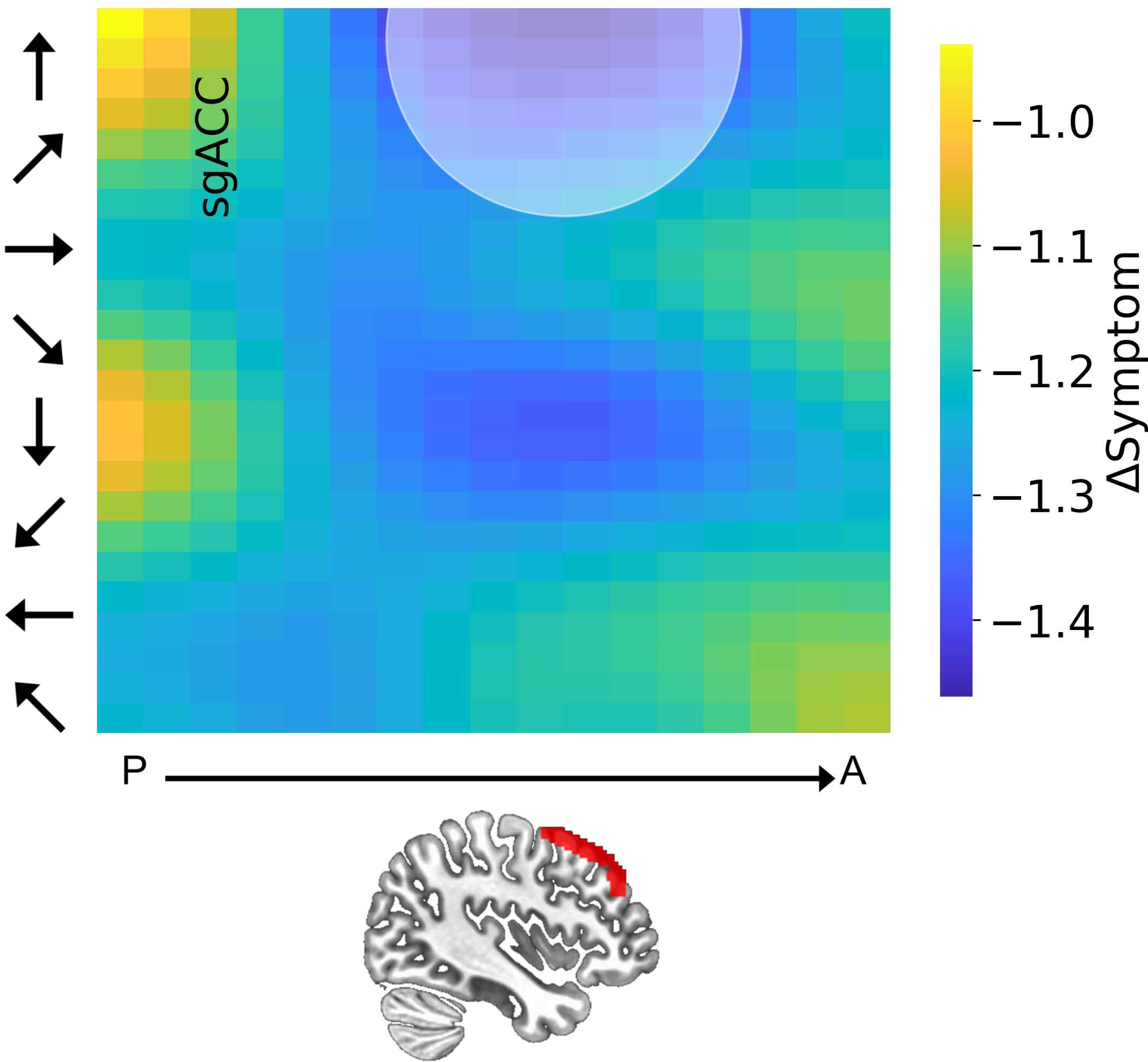

NDARDV056JZW_madrs_left_dlpfc

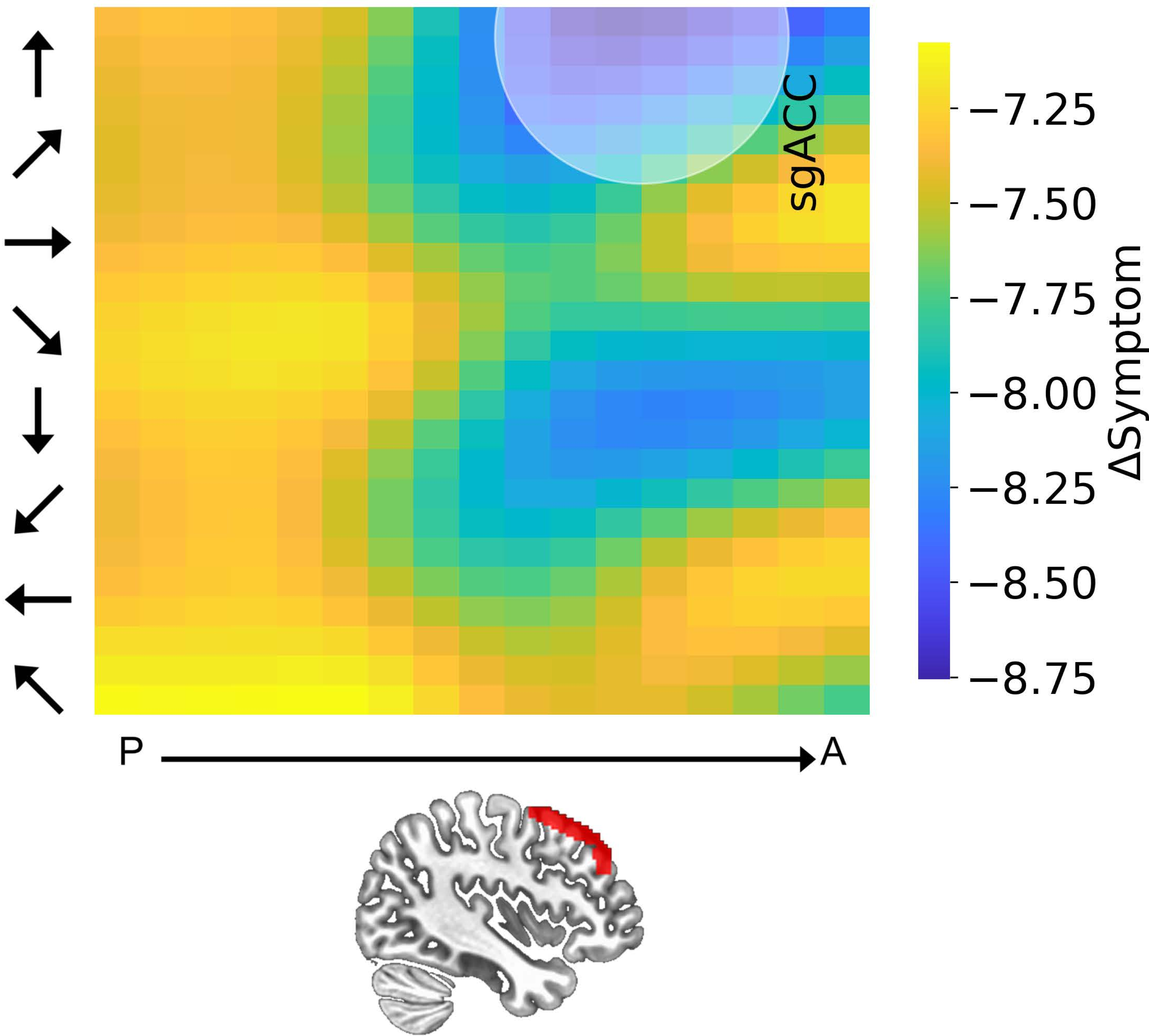

NDAREB933ZEU_madrs_left_dlpfc

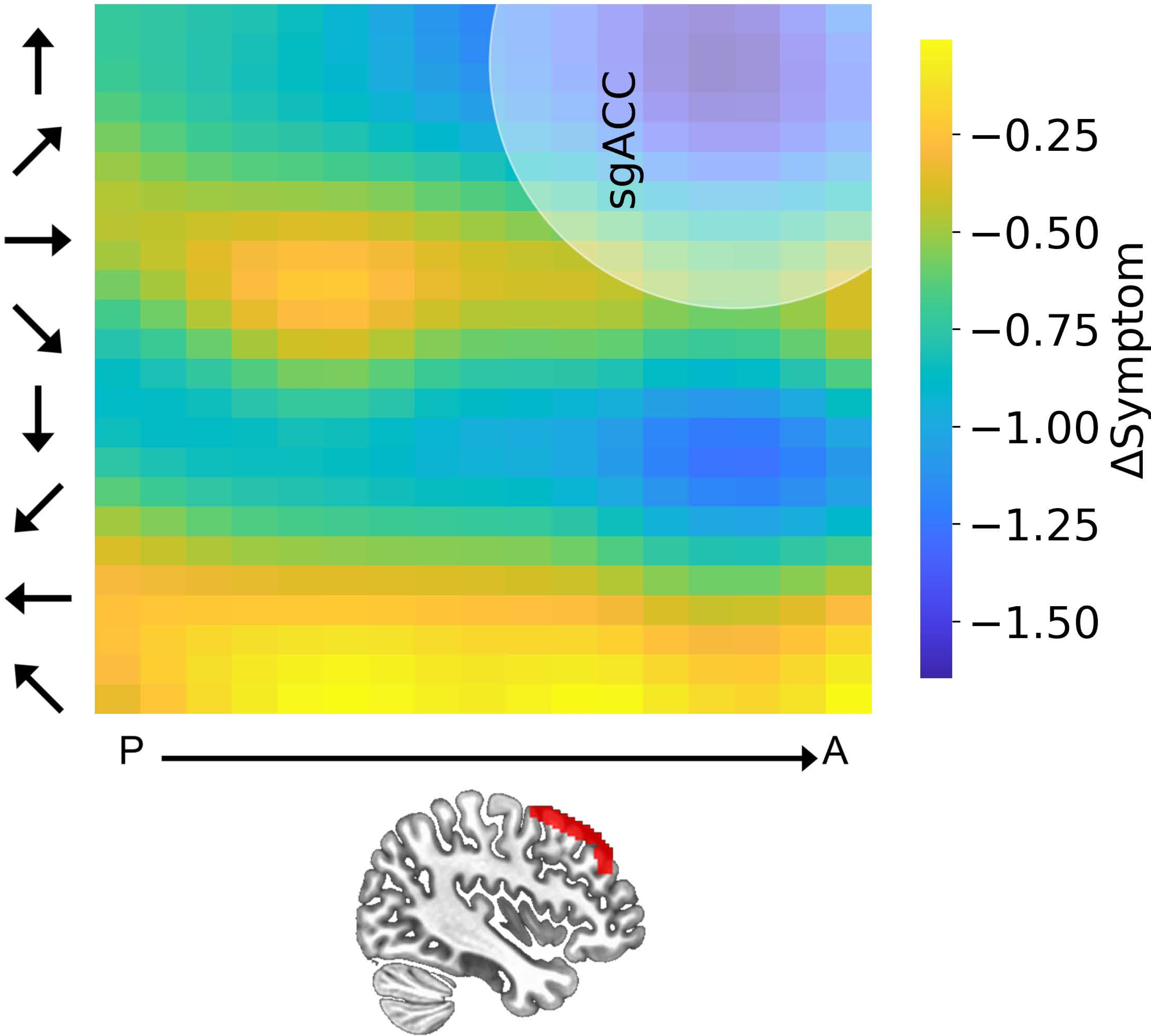

NDAREU727GCY_madrs_left_dlpfc

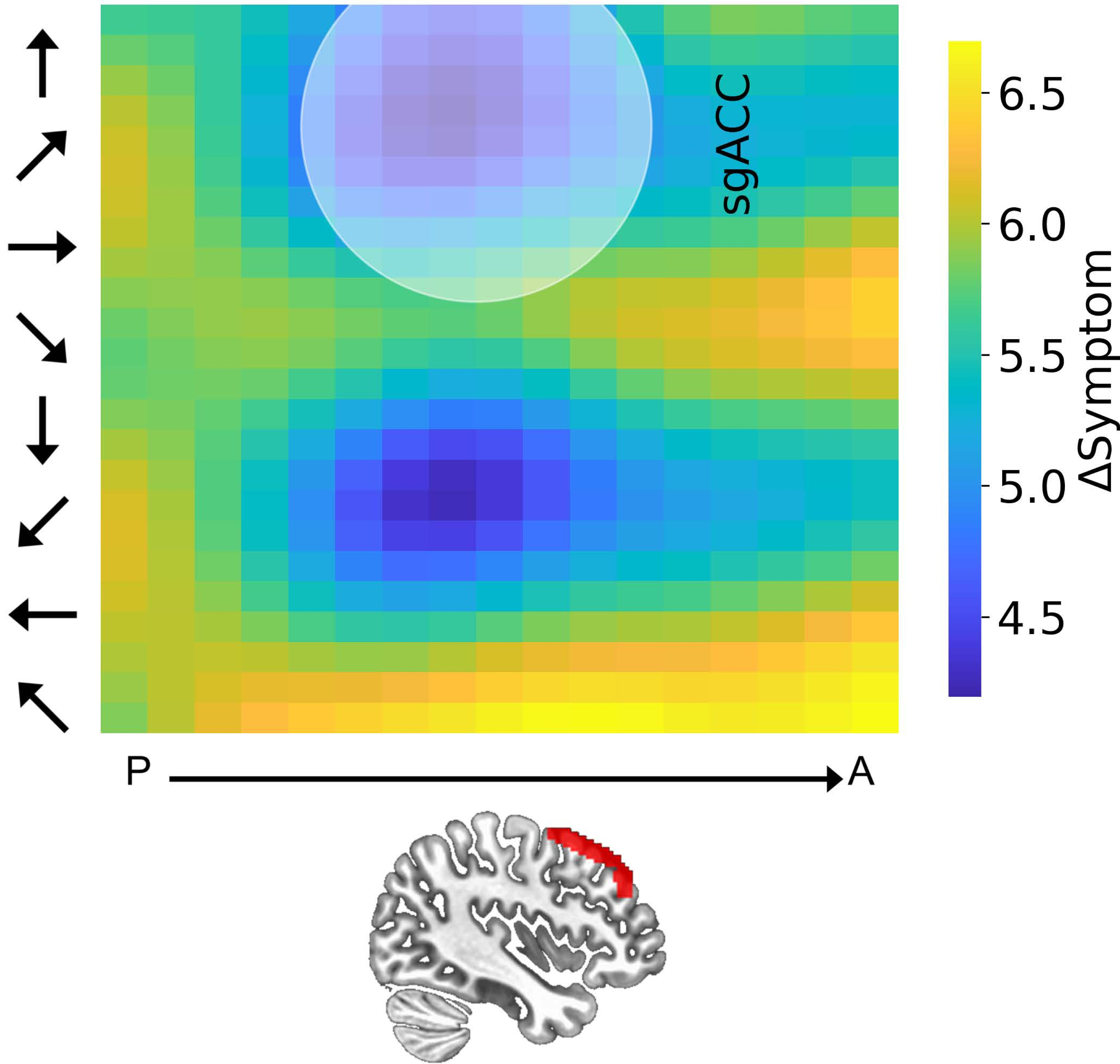

NDAREW484UCJ_madrs_left_dlpfc

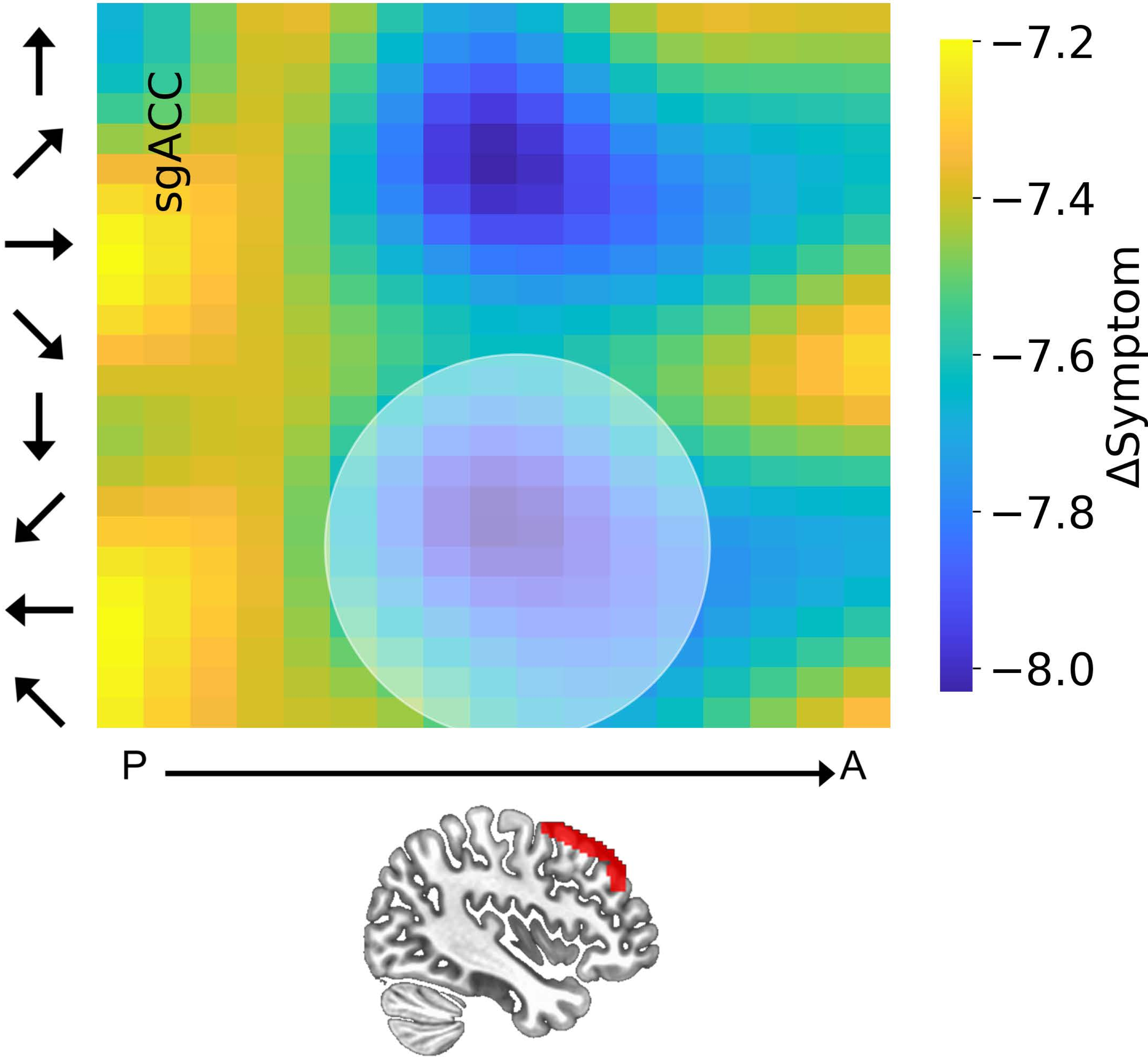

NDAREZ915JK5_madrs_left_dlpfc

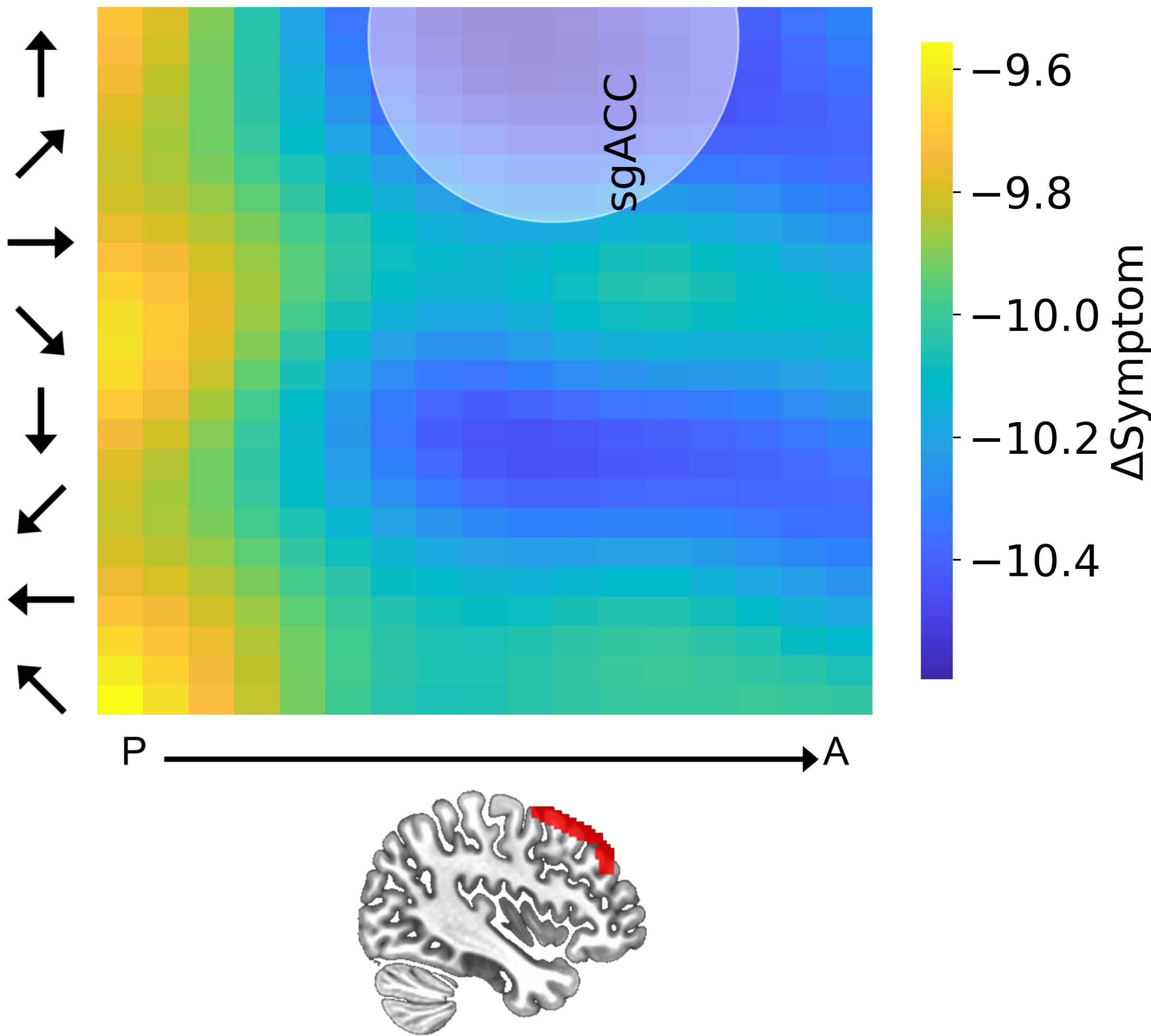

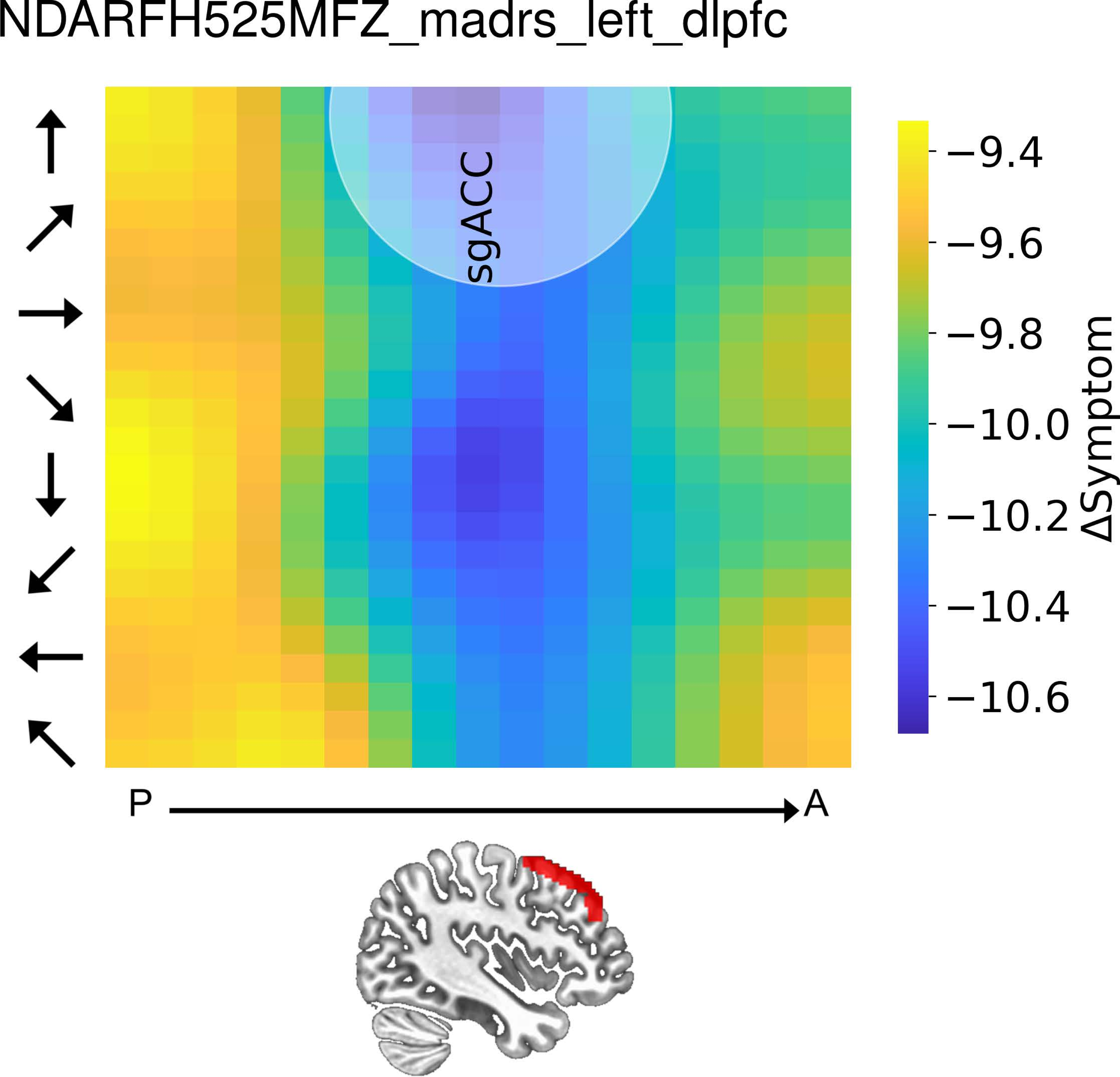

NDARFH525MFZ_madrs_left_dlpfc
sgACC
−9.4
−9.6
−9.8
−10.0
−10.2
−10.4
−10.6
ΔSymptom
P
A

NDARFR746ENQ_madrs_left_dlpfc

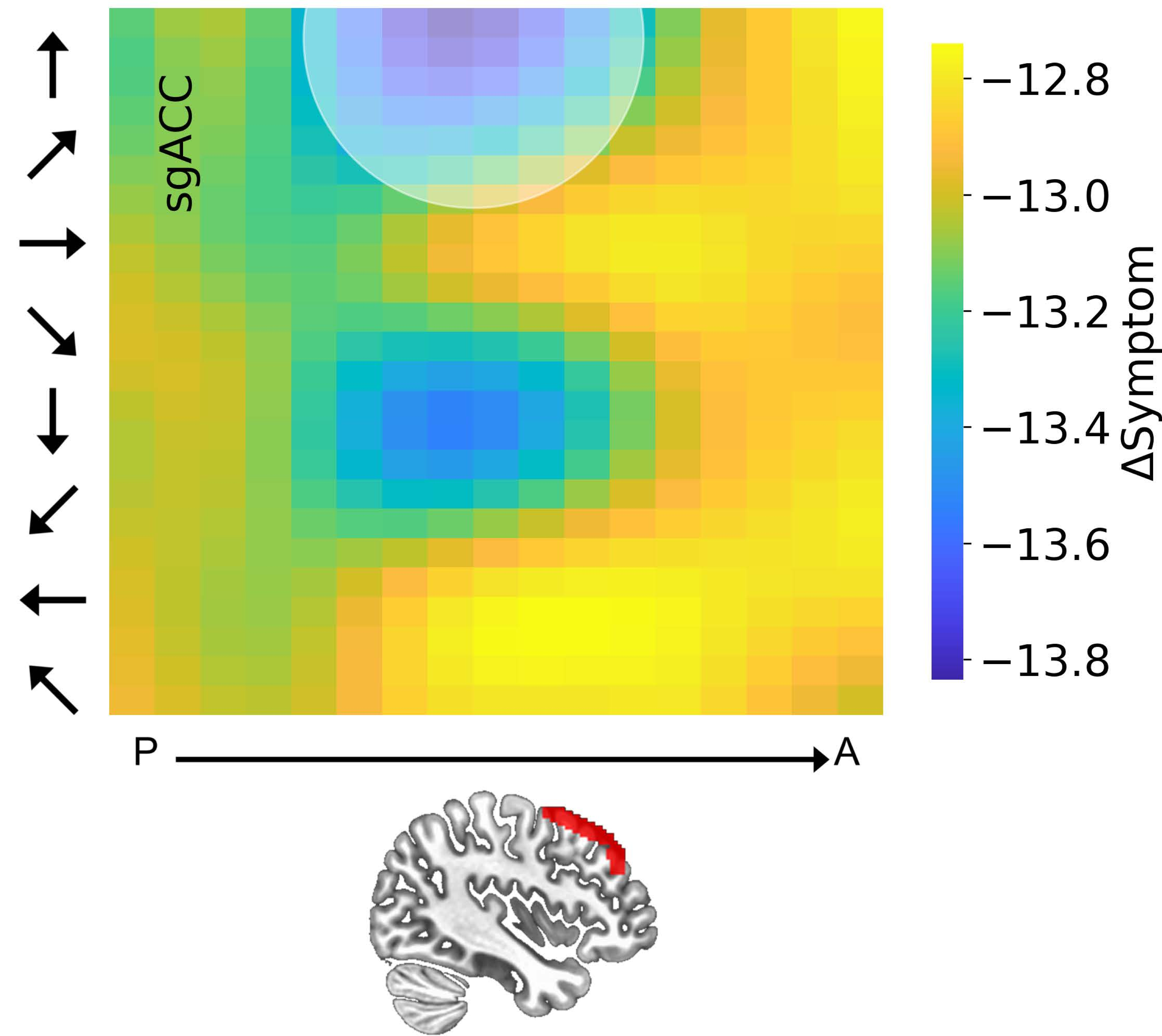

NDARFW096LMD_madrs_left_dlpfc

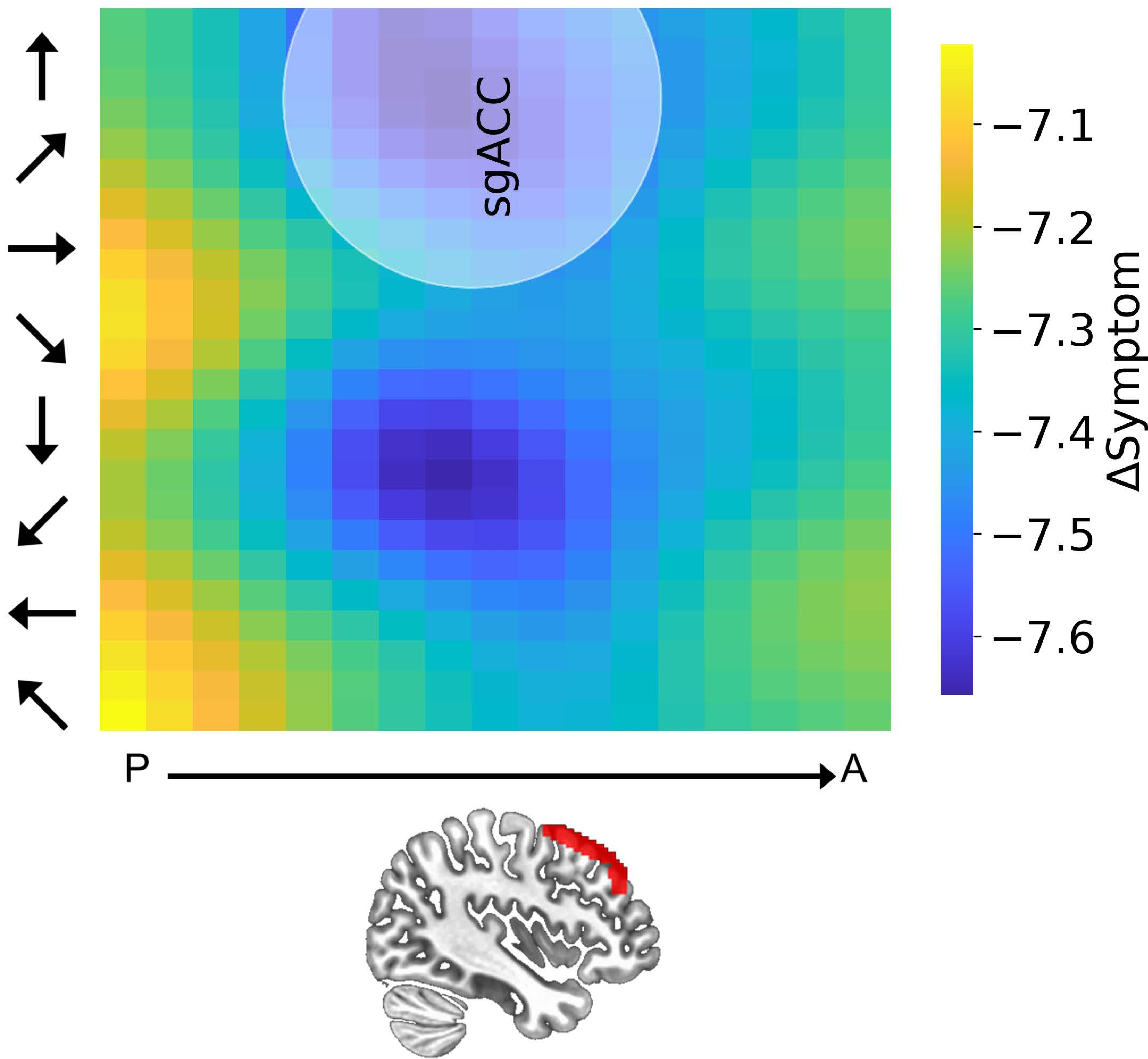

NDARGH895WJJ_madrs_left_dlpfc

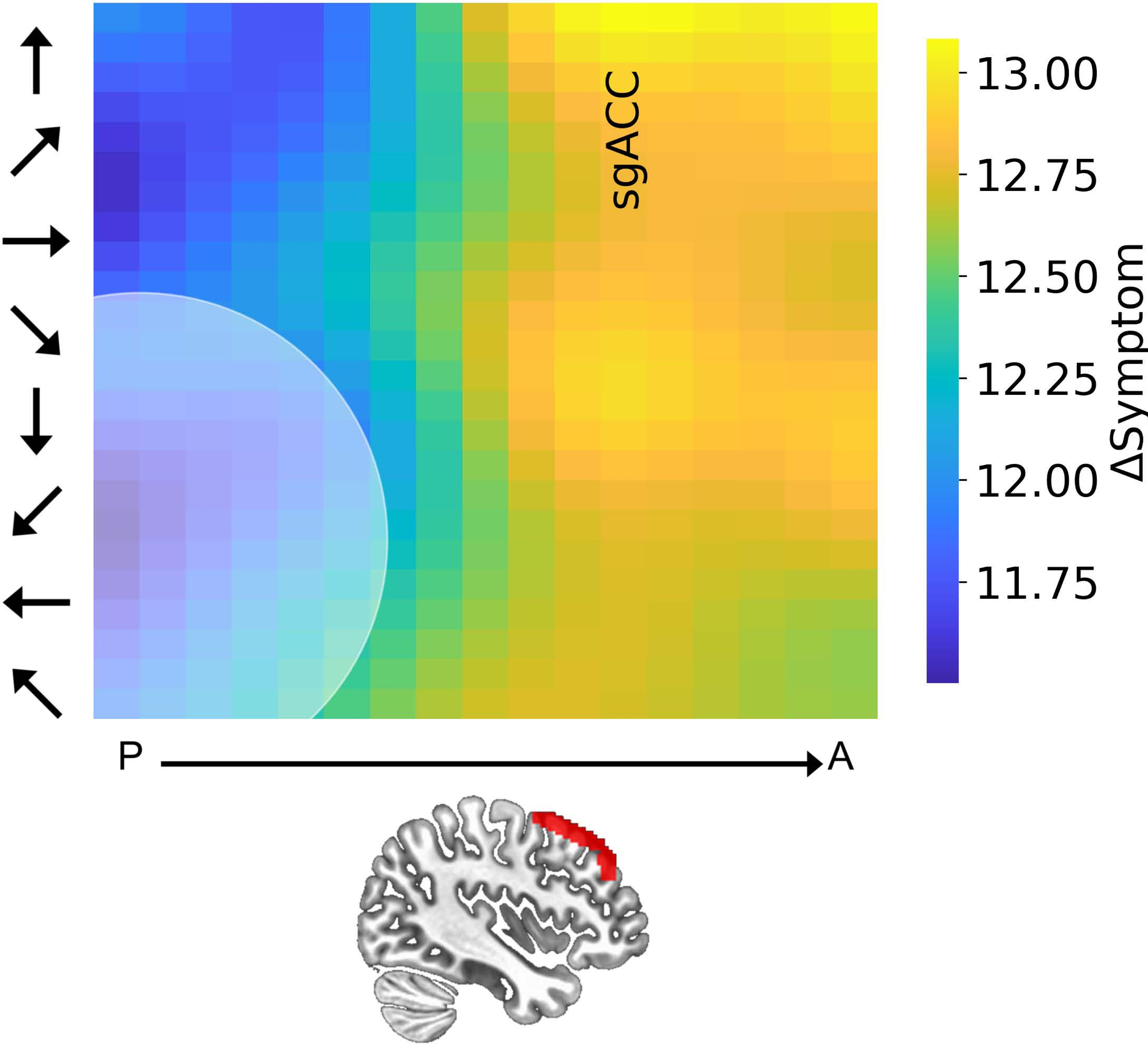

NDARGR637FTT_madrs_left_dlpfc

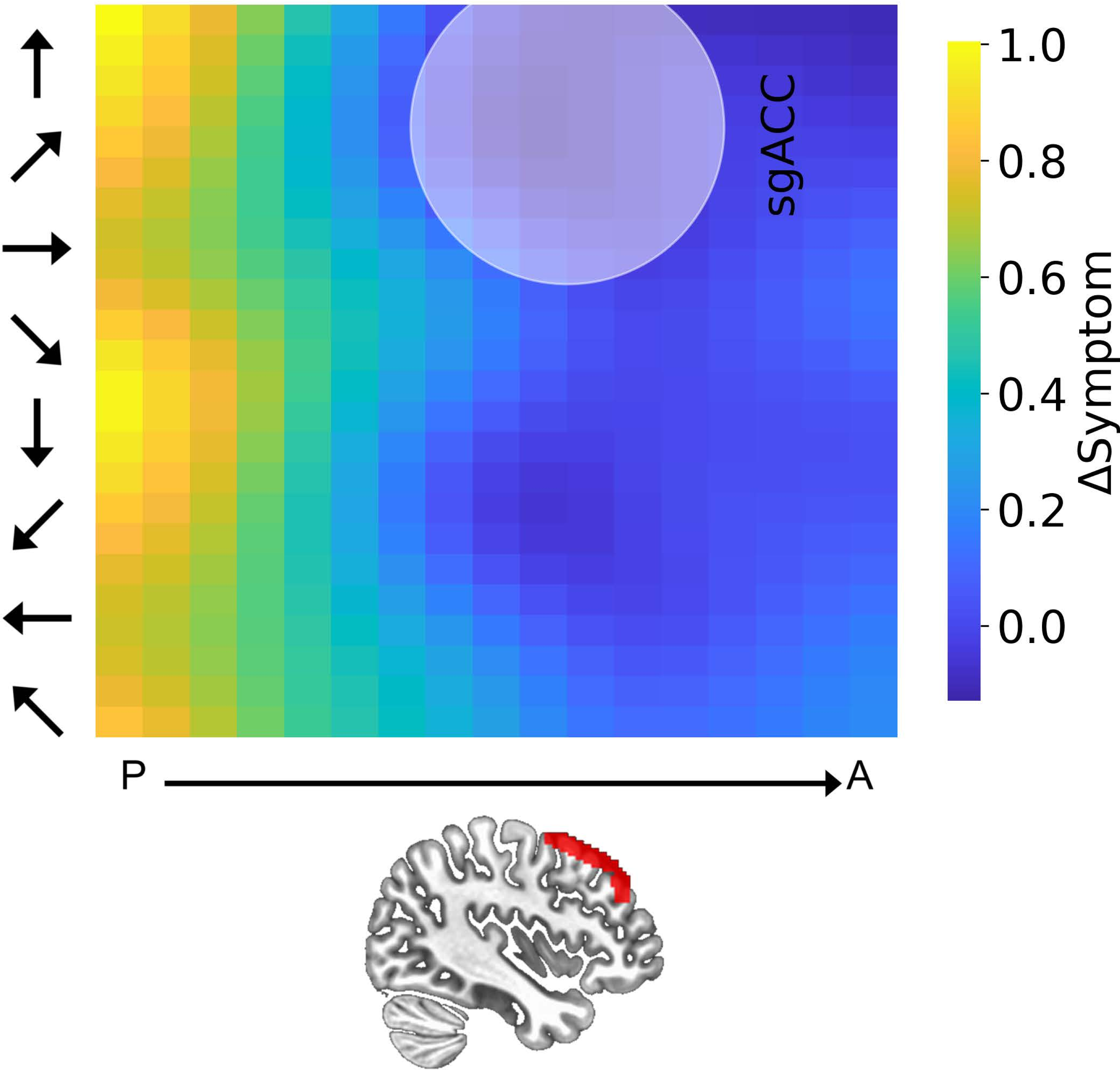

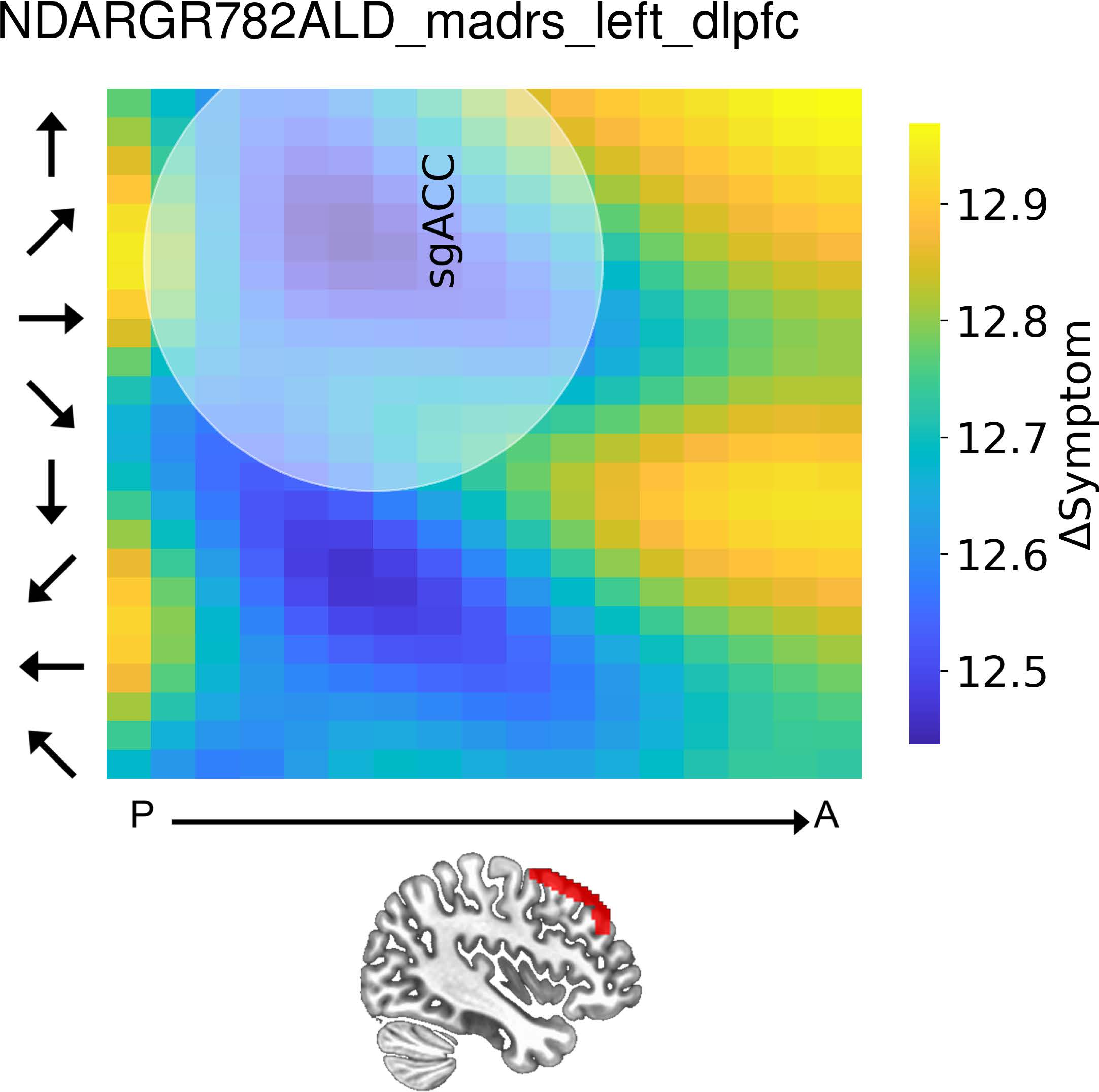
NDARGR782ALD_madrs_left_dlpfc
sgACC
12.9
12.8
12.7
12.6
12.5
ΔSymptom
P
A

NDARHA109LH7_madrs_left_dlpfc

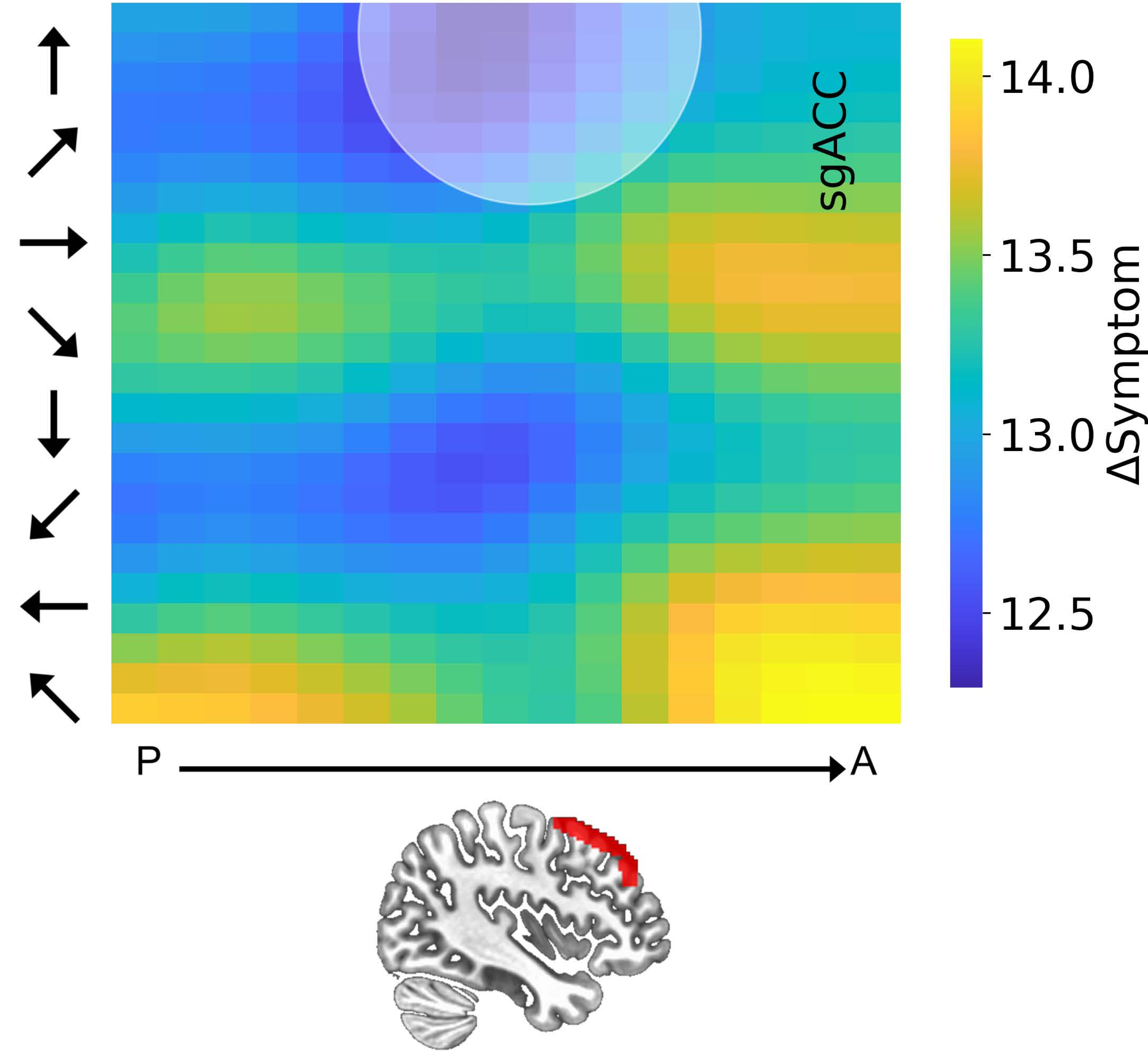

NDARHR981AP1_madrs_left_dlpfc

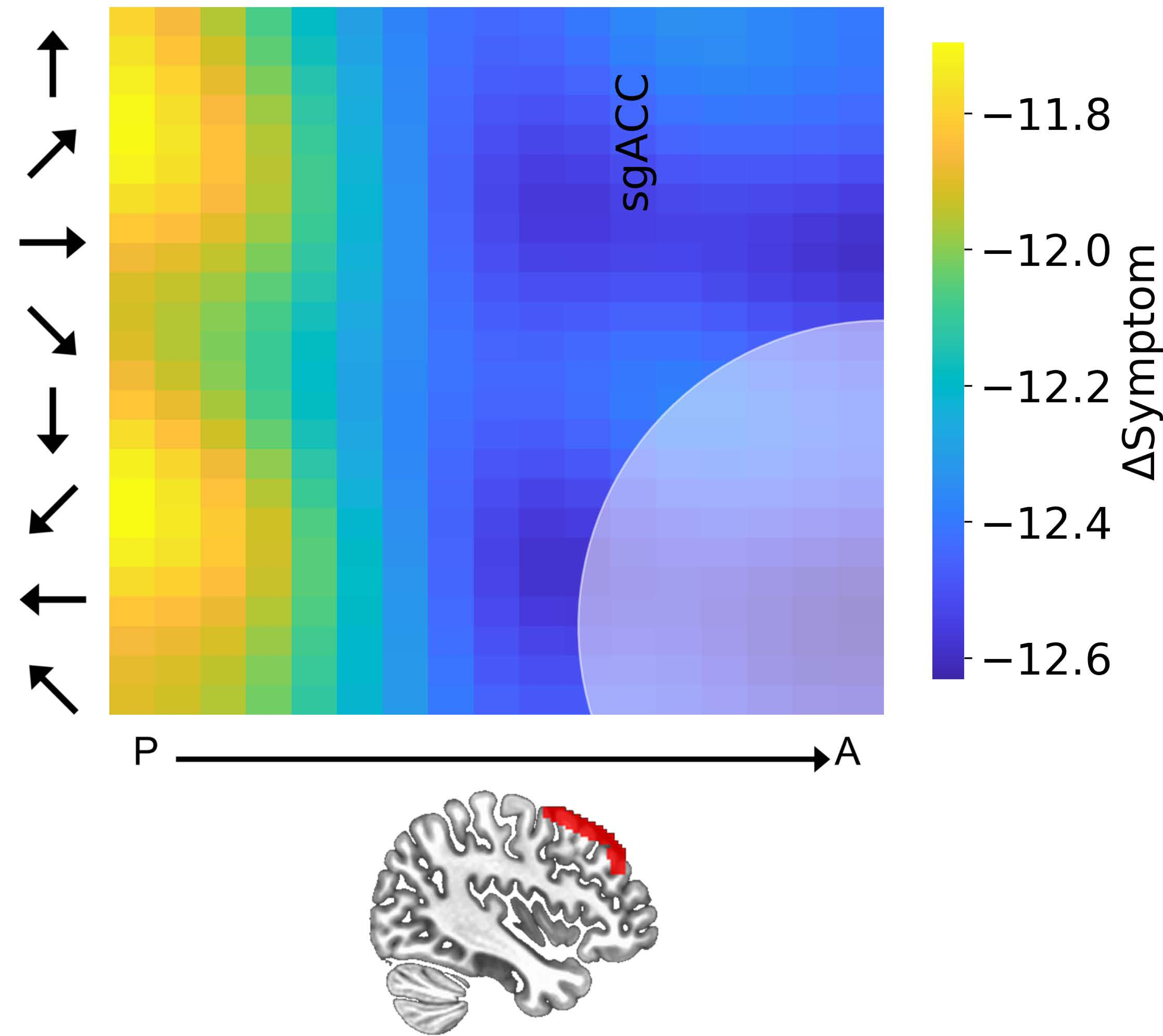

NDARHX487CT9_madrs_left_dlpfc

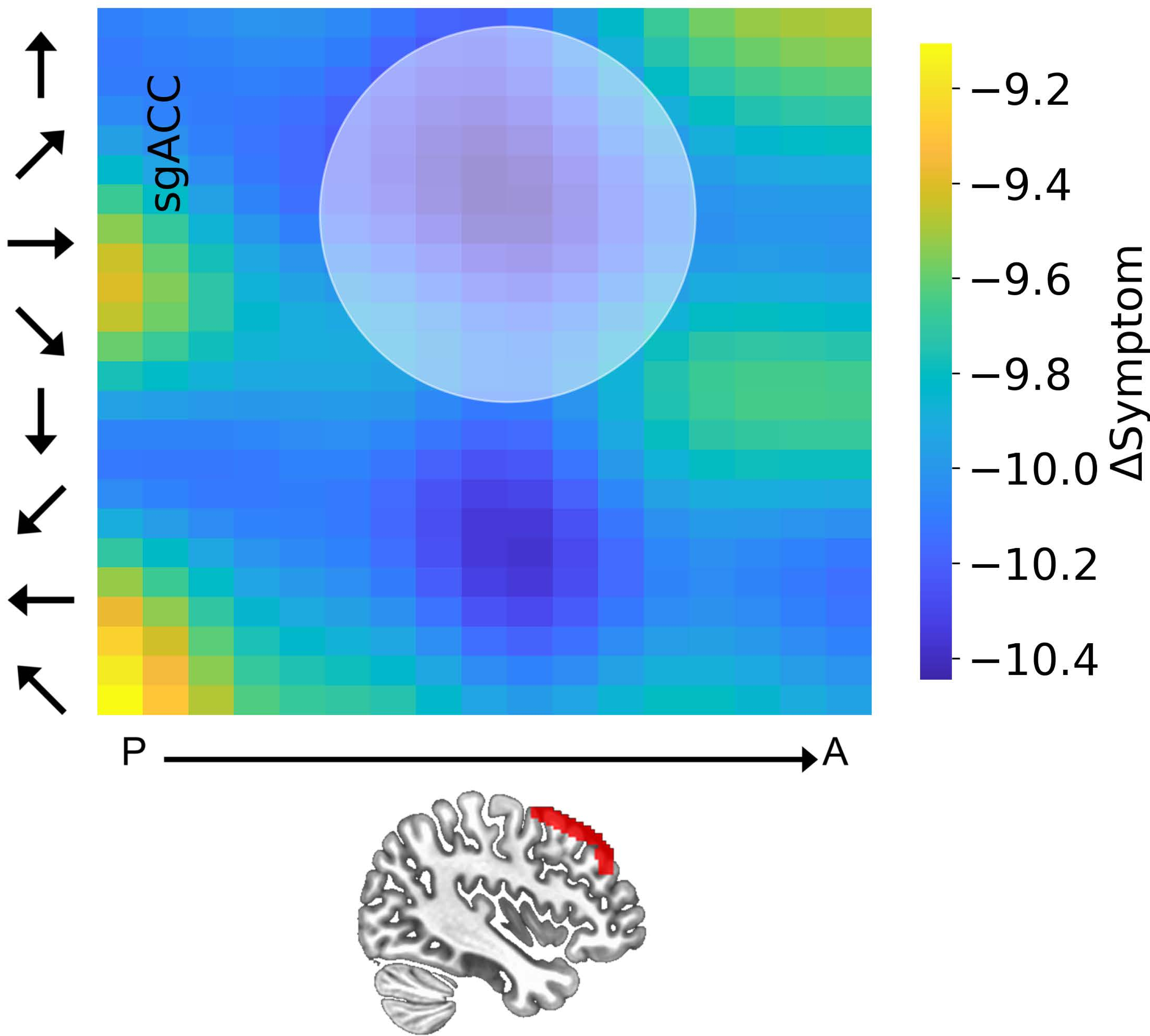

NDARHZ301NUW_madrs_left_dlpfc

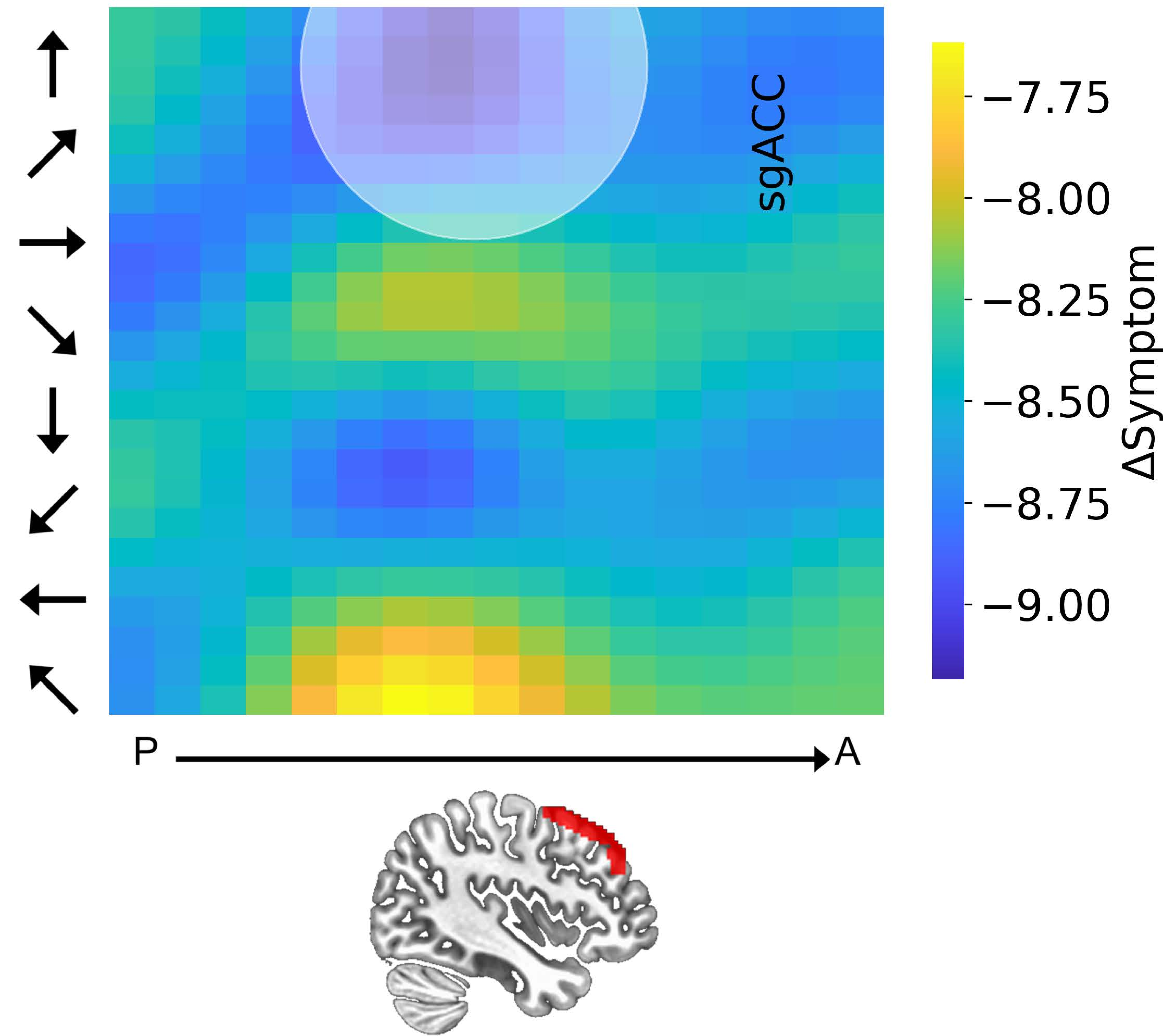

NDARHZ662EPK_madrs_left_dlpfc

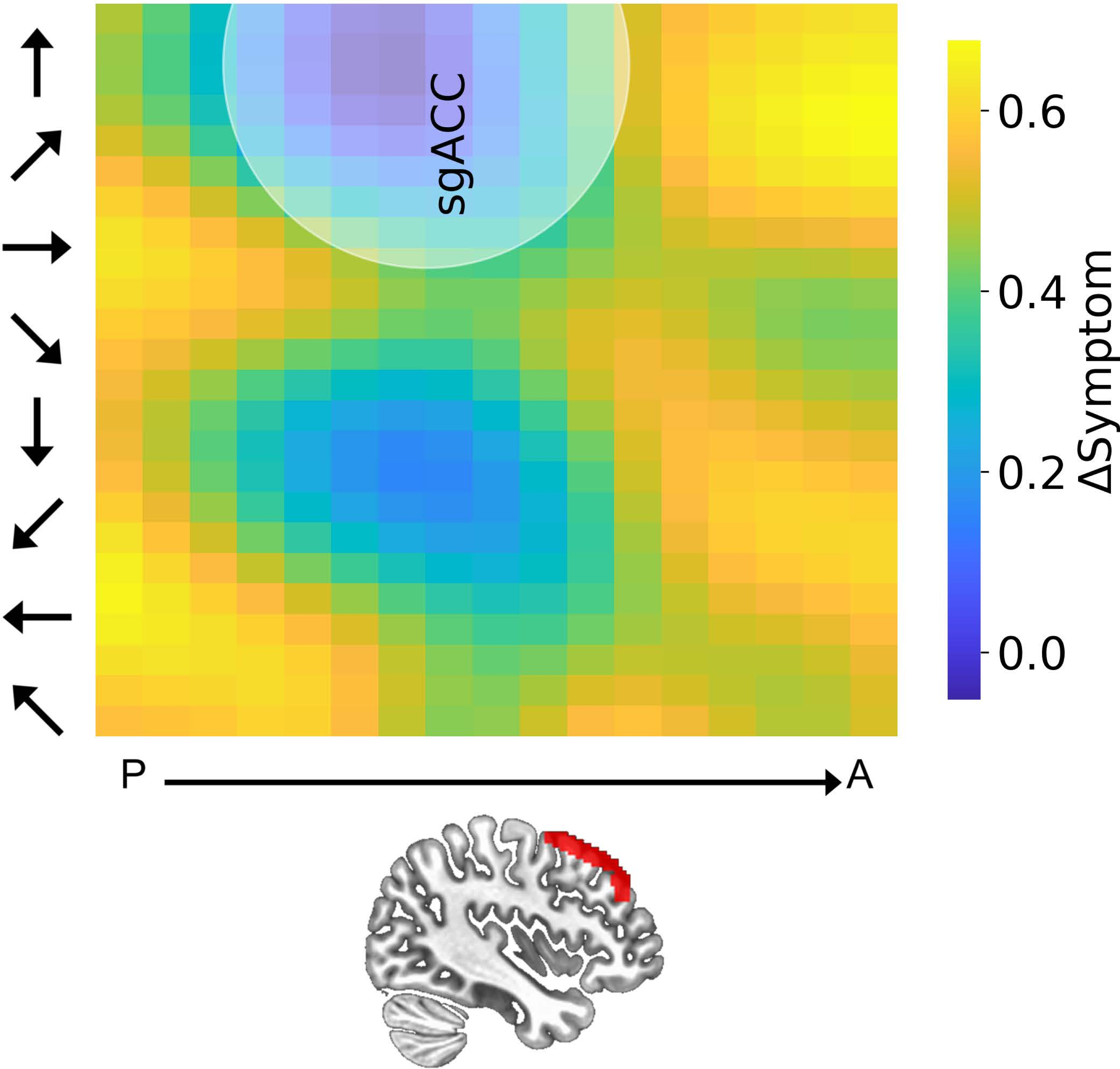

NDARJD271PMF_madrs_left_dlpfc

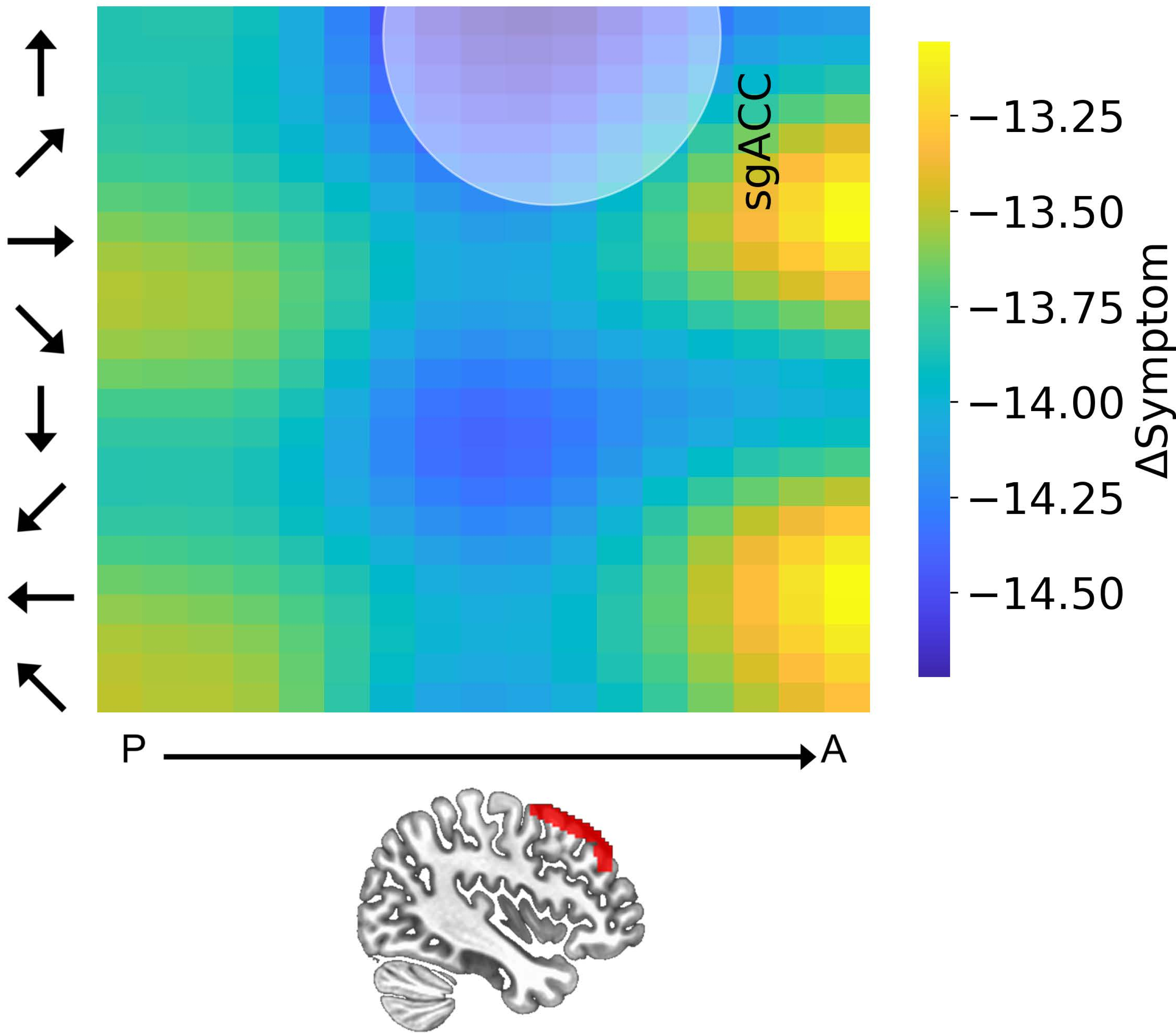

NDARJD548GTC_madrs_left_dlpfc

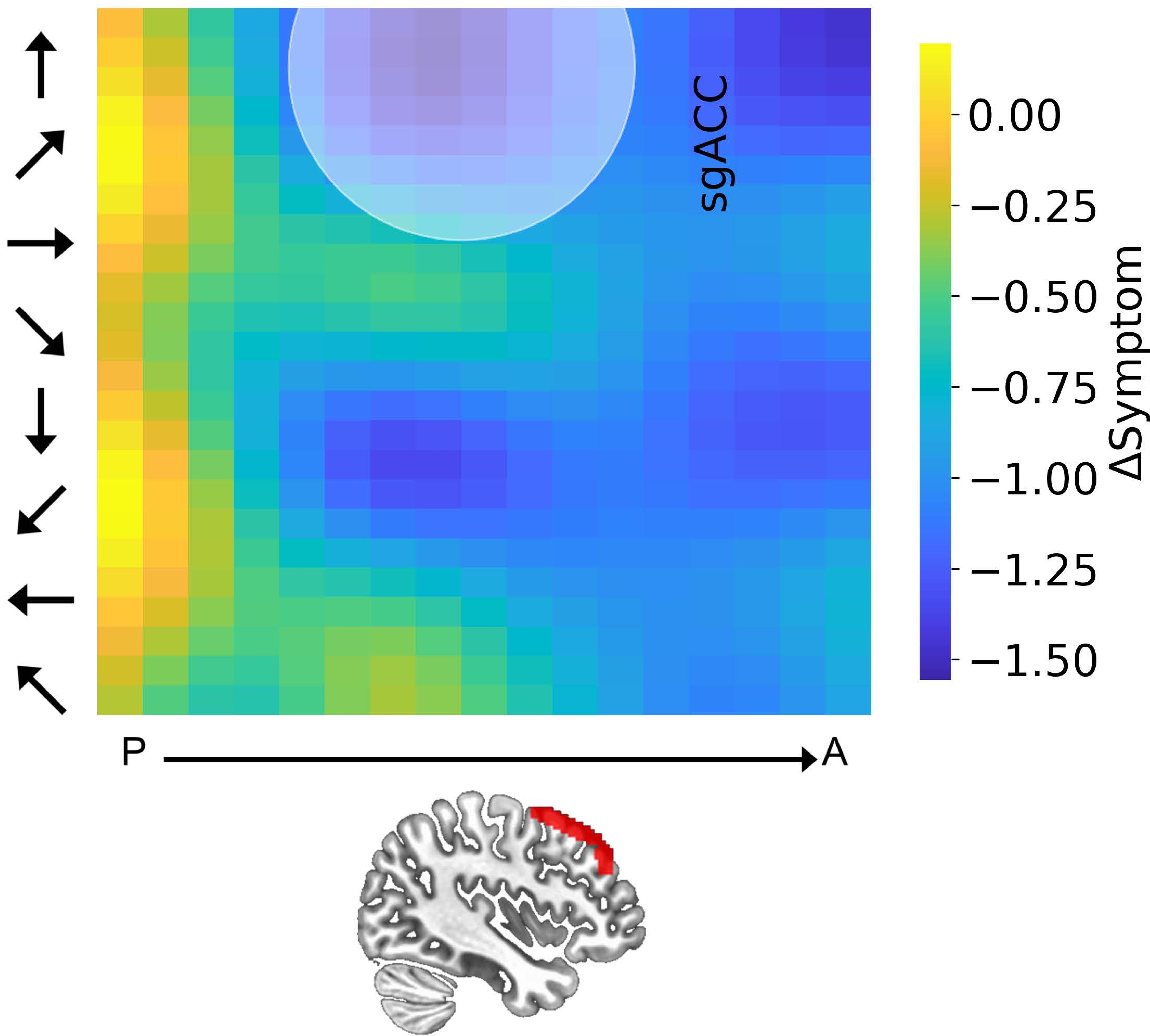

NDARJF993NV1_madrs_left_dlpfc

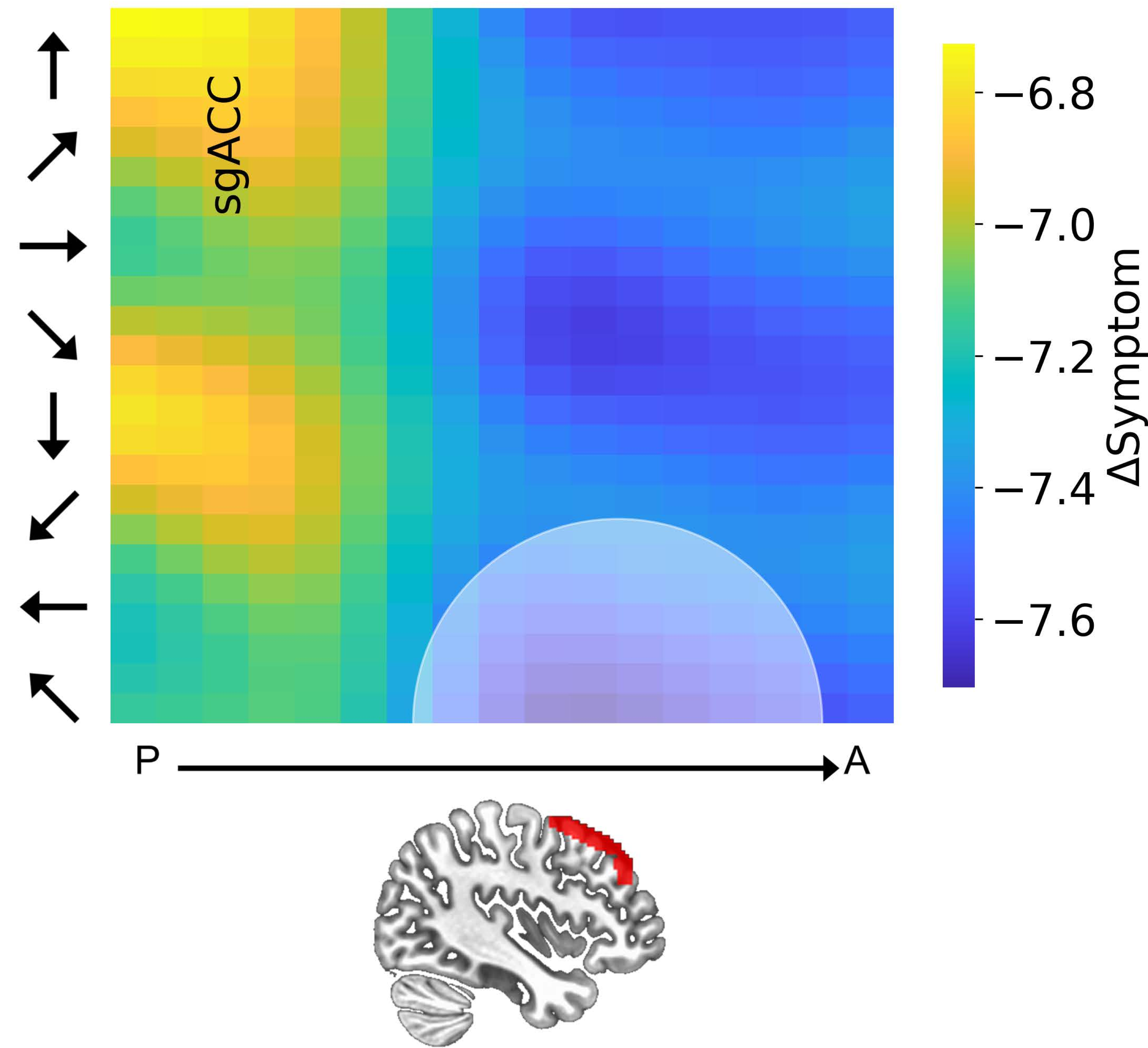

NDARJH406WC9_madrs_left_dlpfc

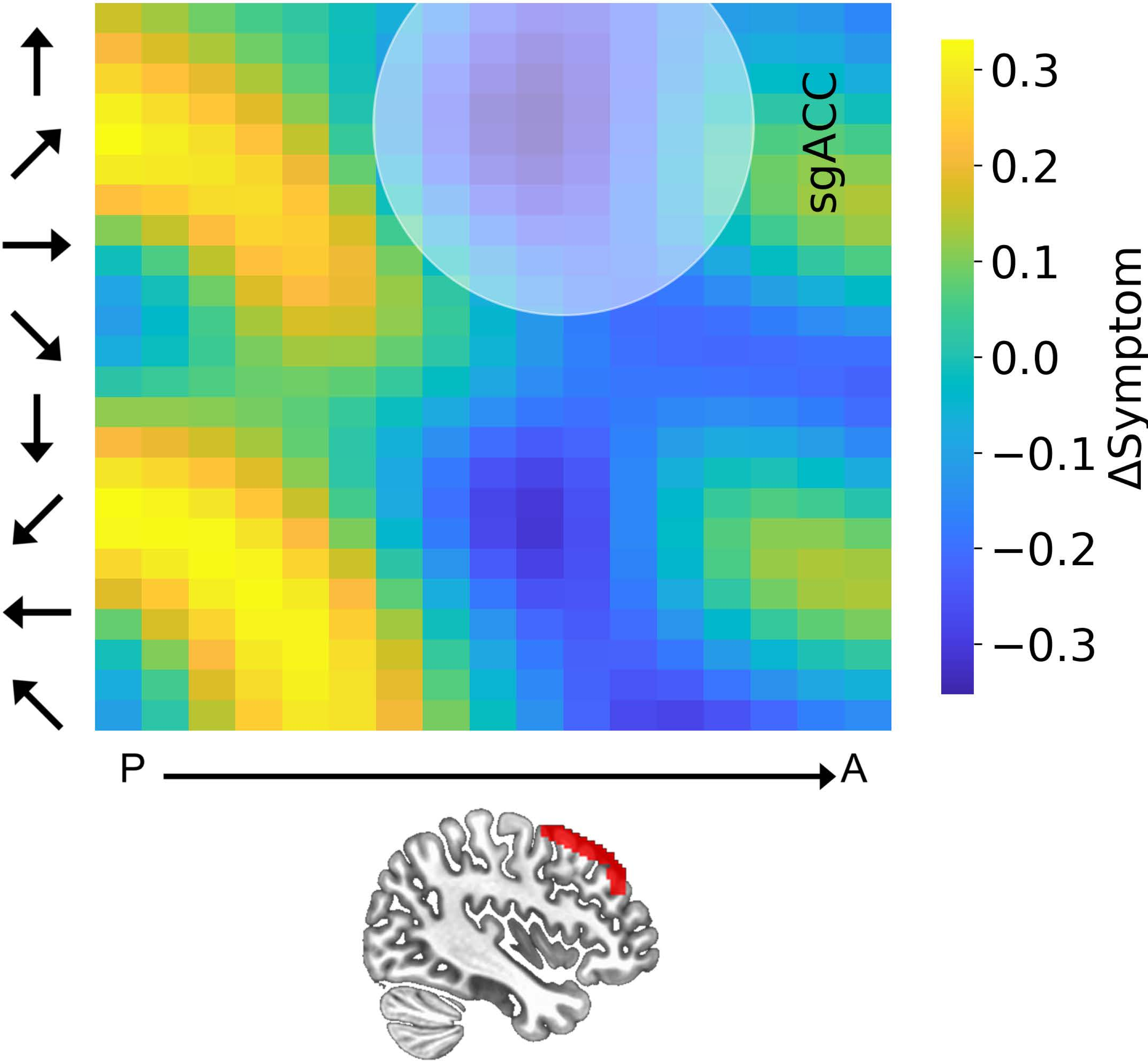

NDARJX575DVK_madrs_left_dlpfc

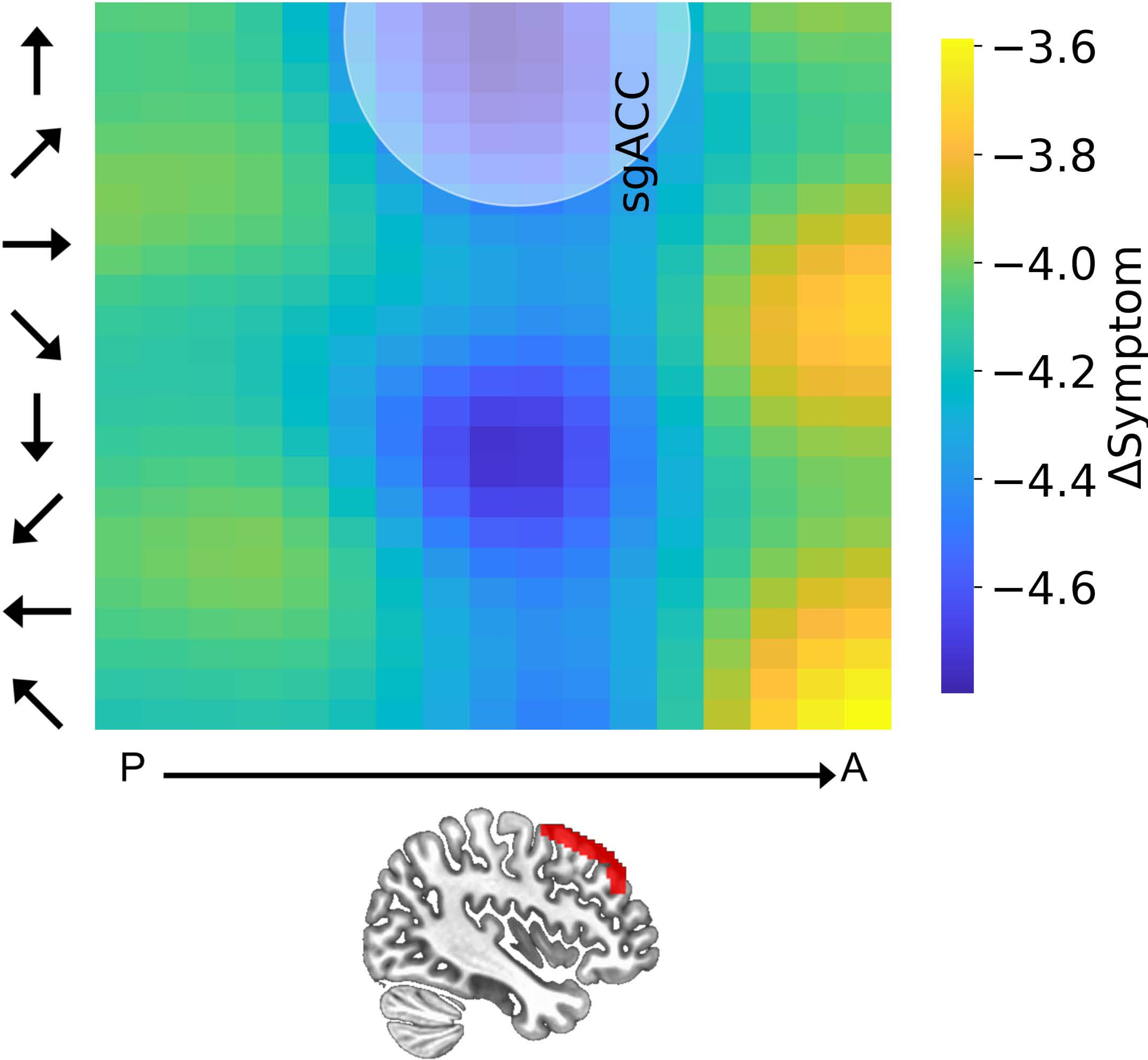

NDARJY042TLY_madrs_left_dlpfc

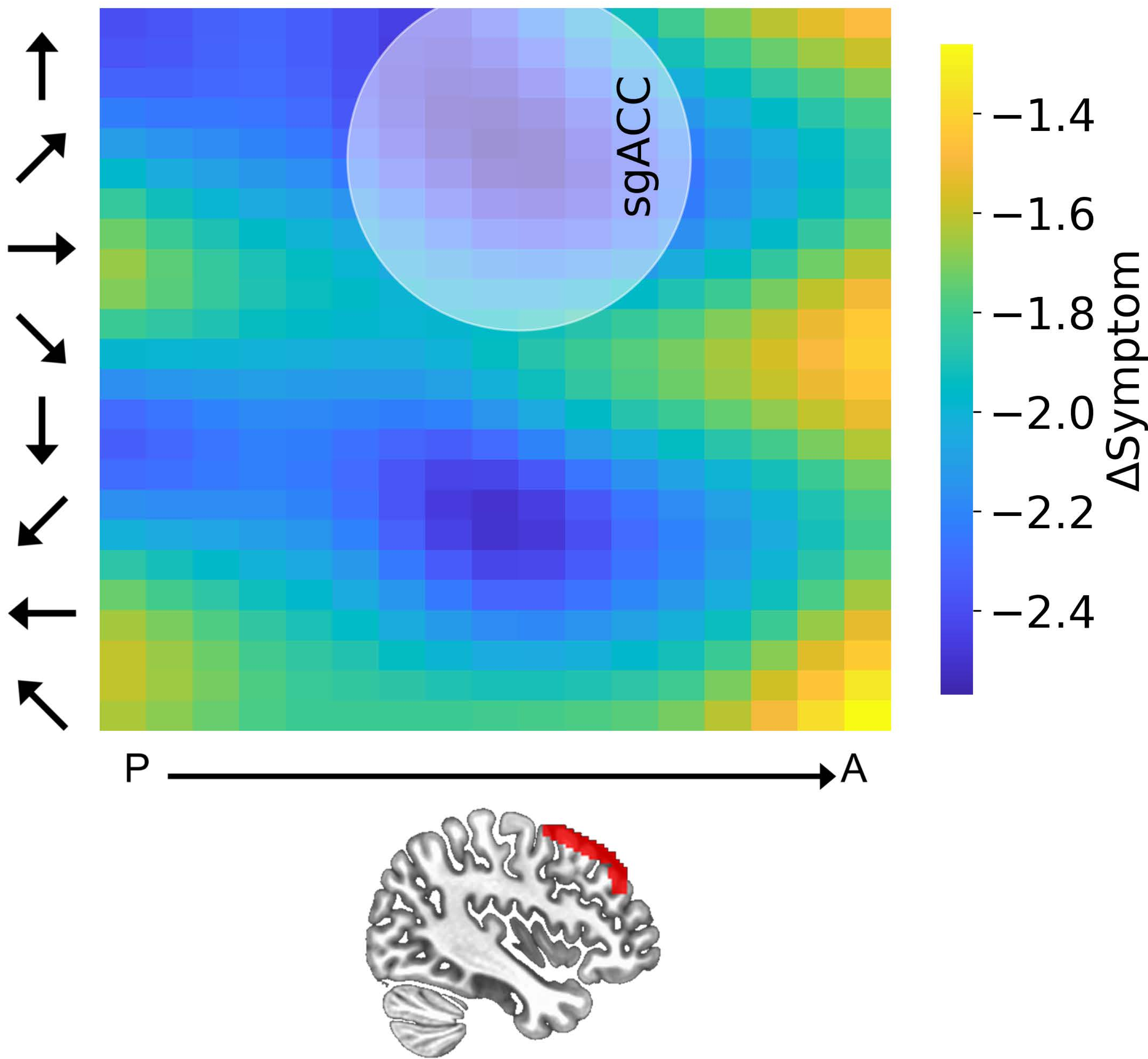

NDARKK212YTX_madrs_left_dlpfc

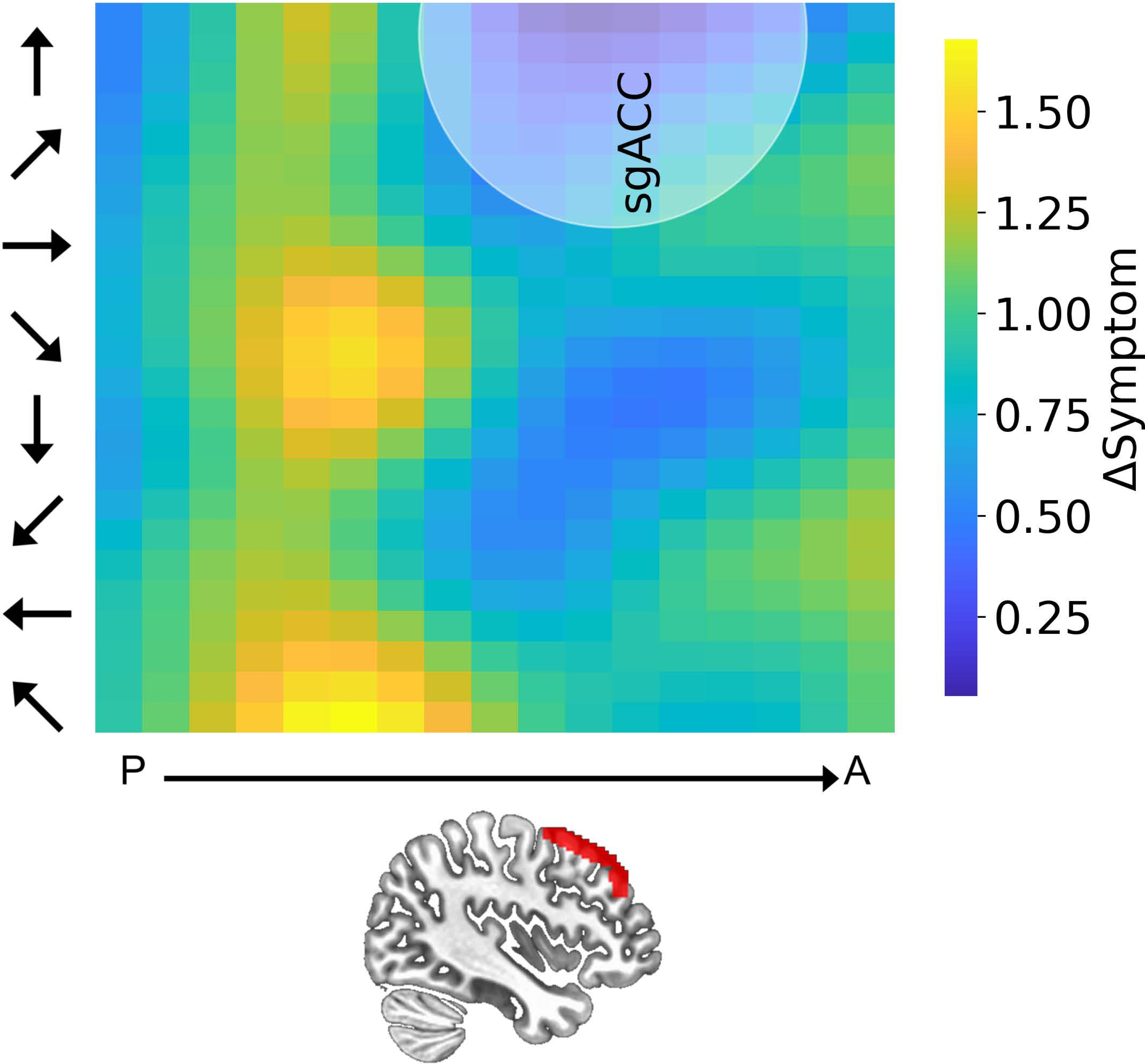

NDARKM333VX2_madrs_left_dlpfc

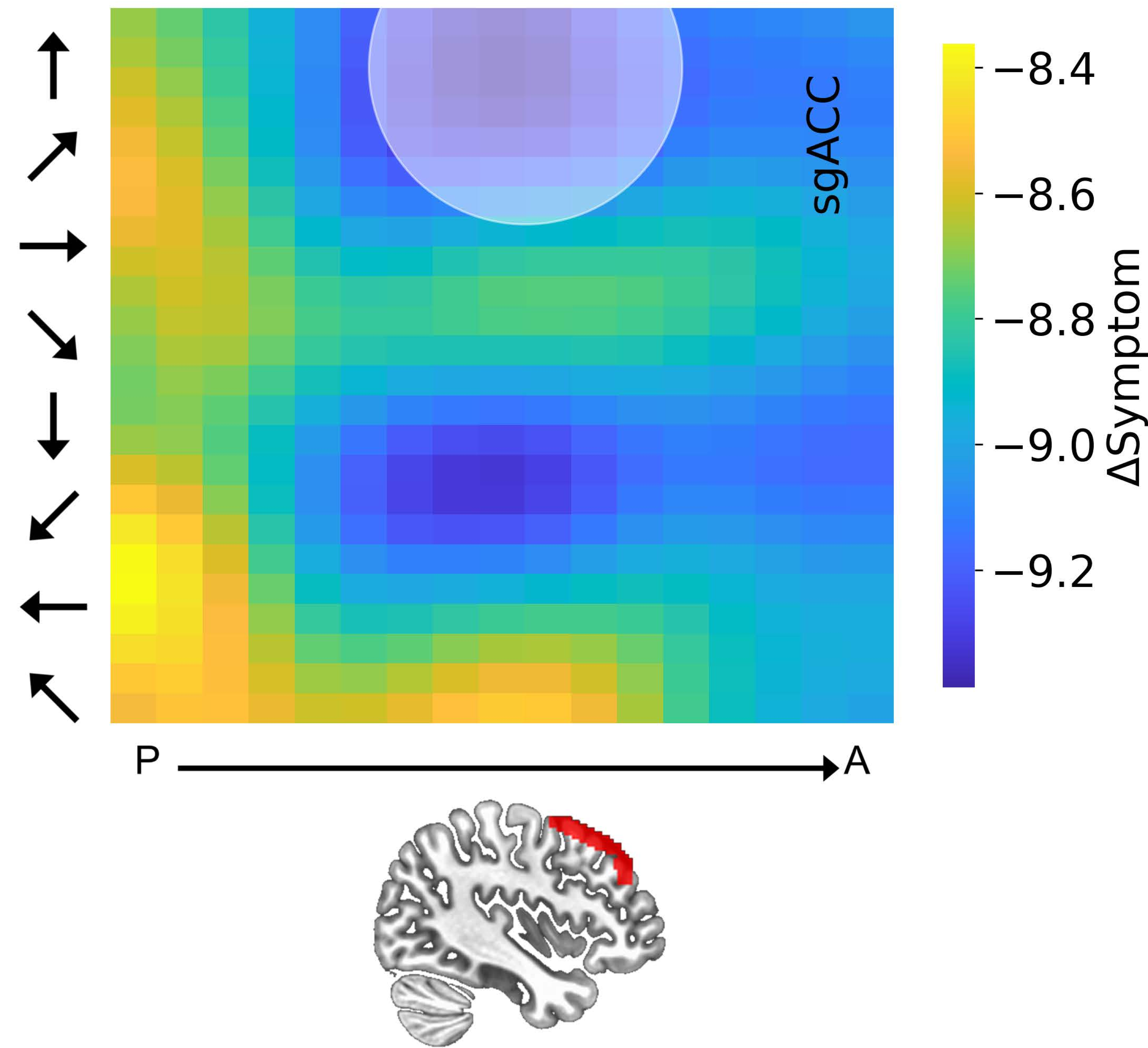

NDARKT991DUC_madrs_left_dlpfc

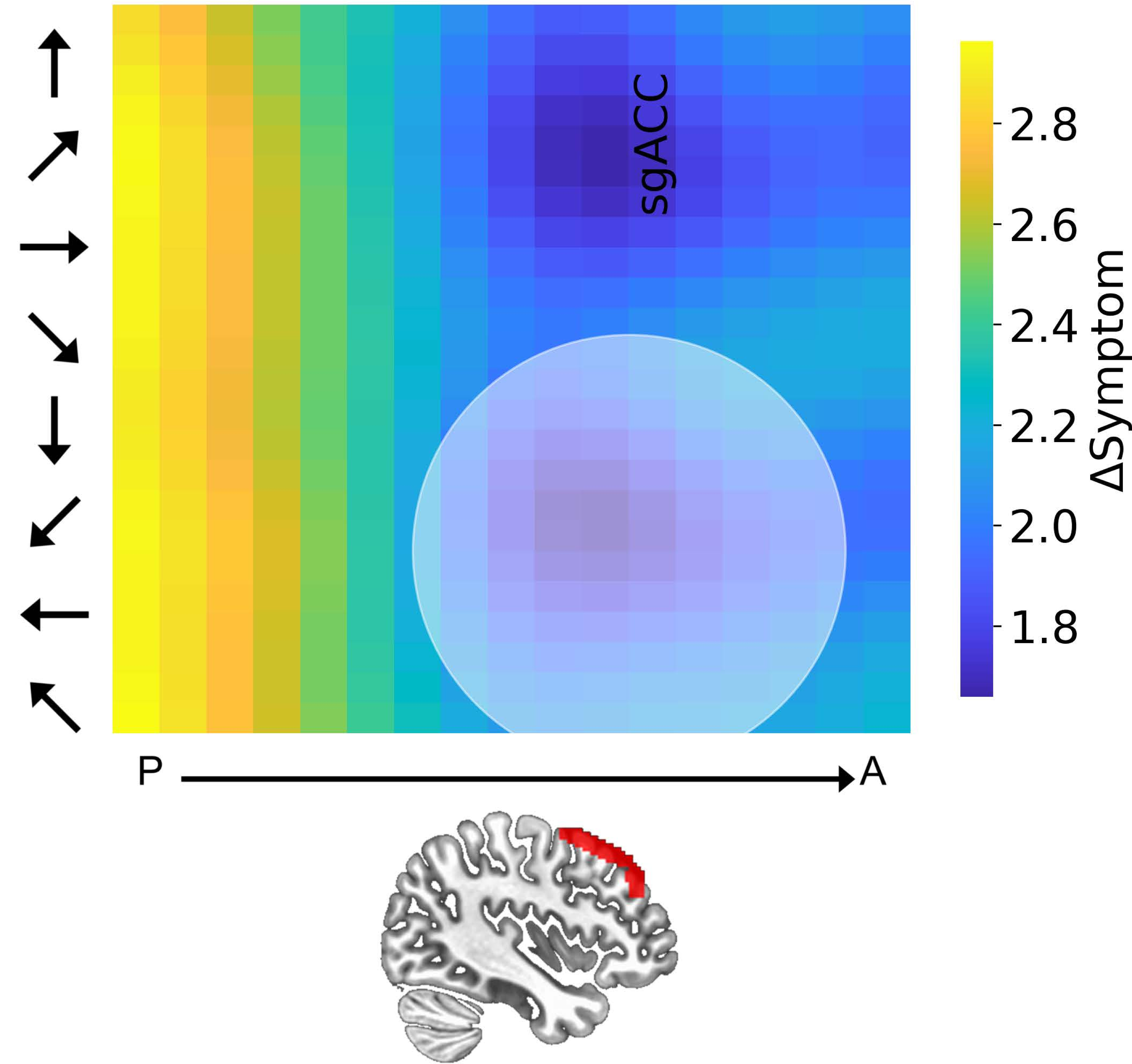

NDARKU015LTM_madrs_left_dlpfc

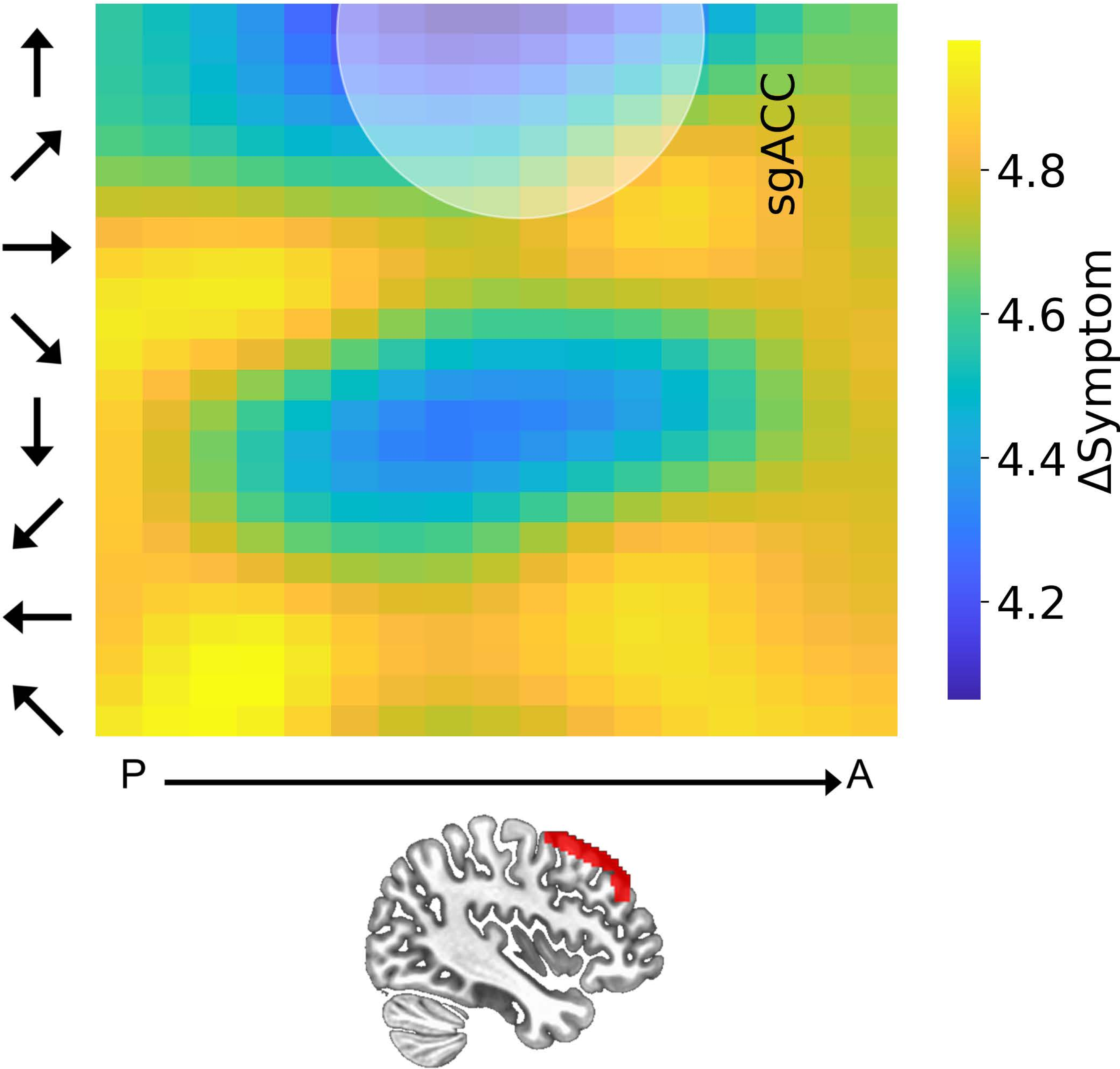

NDARKV126RTP_madrs_left_dlpfc

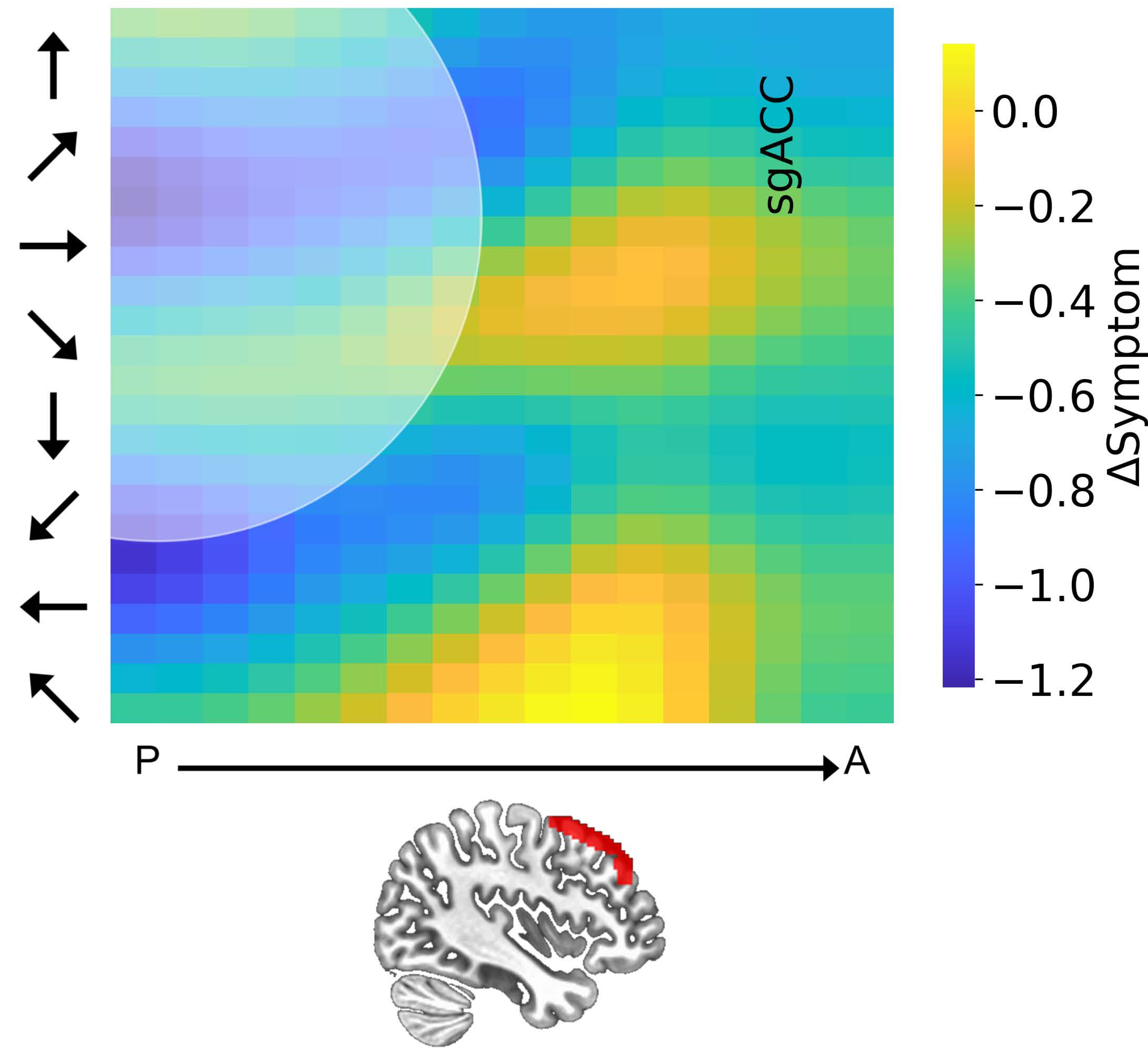

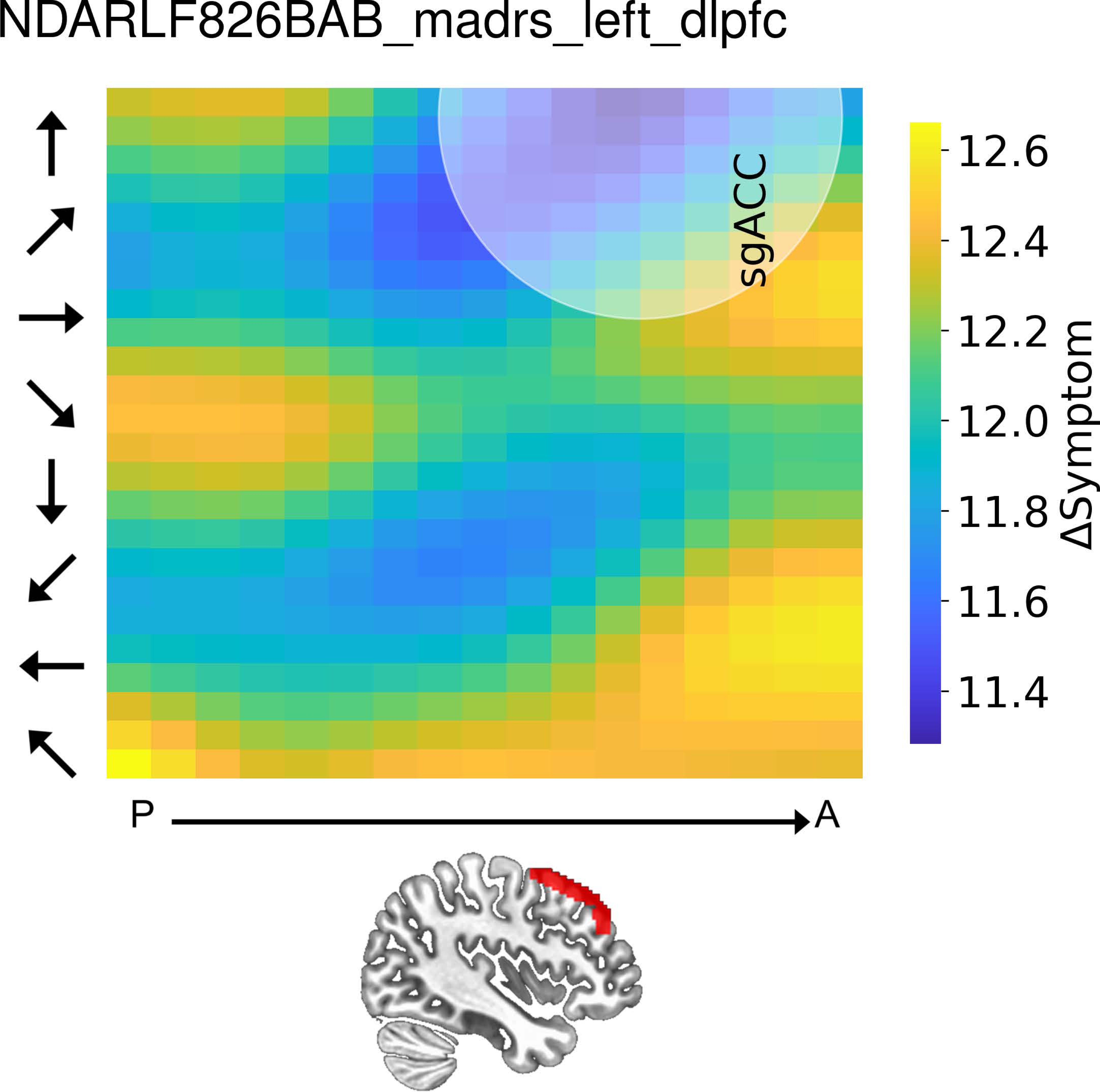
NDARLF826BAB_madrs_left_dlpfc
sgACC
12.6
12.4
12.2
12.0
11.8
11.6
11.4
ΔSymptom
P
A

NDARLM890ELE_madrs_left_dlpfc

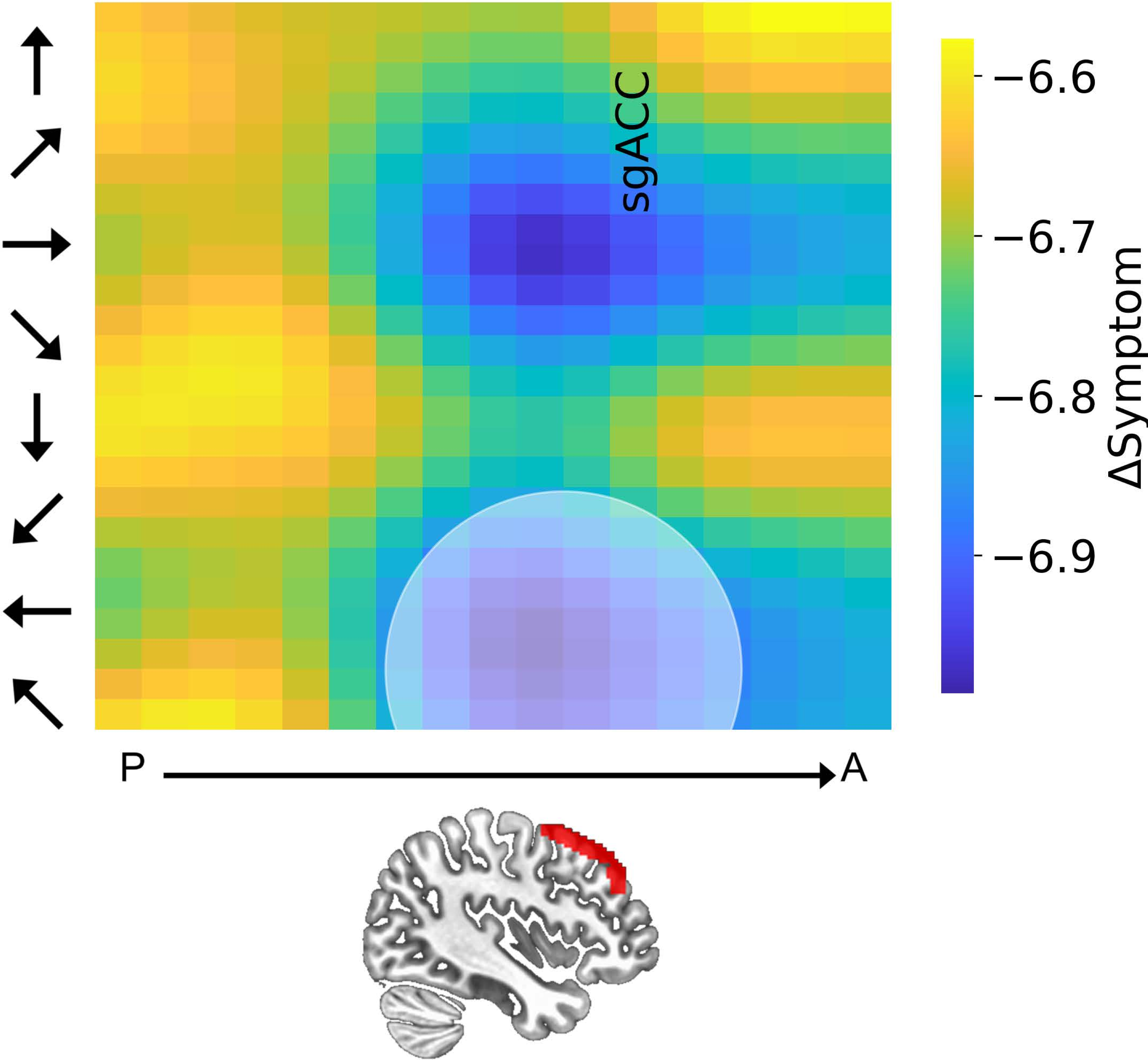

NDARLZ935EEN_madrs_left_dlpfc

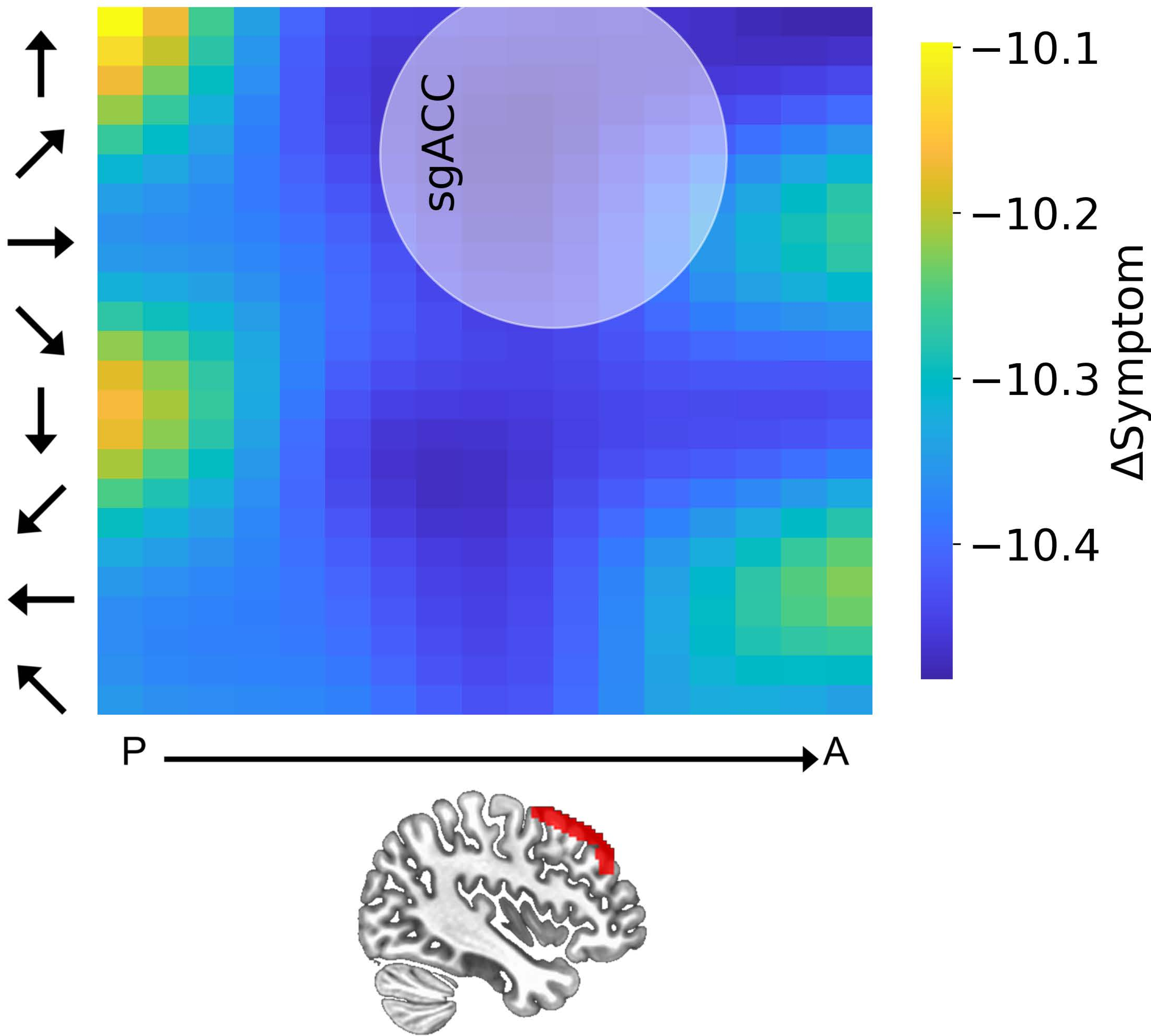

NDARMA155ATG_madrs_left_dlpfc

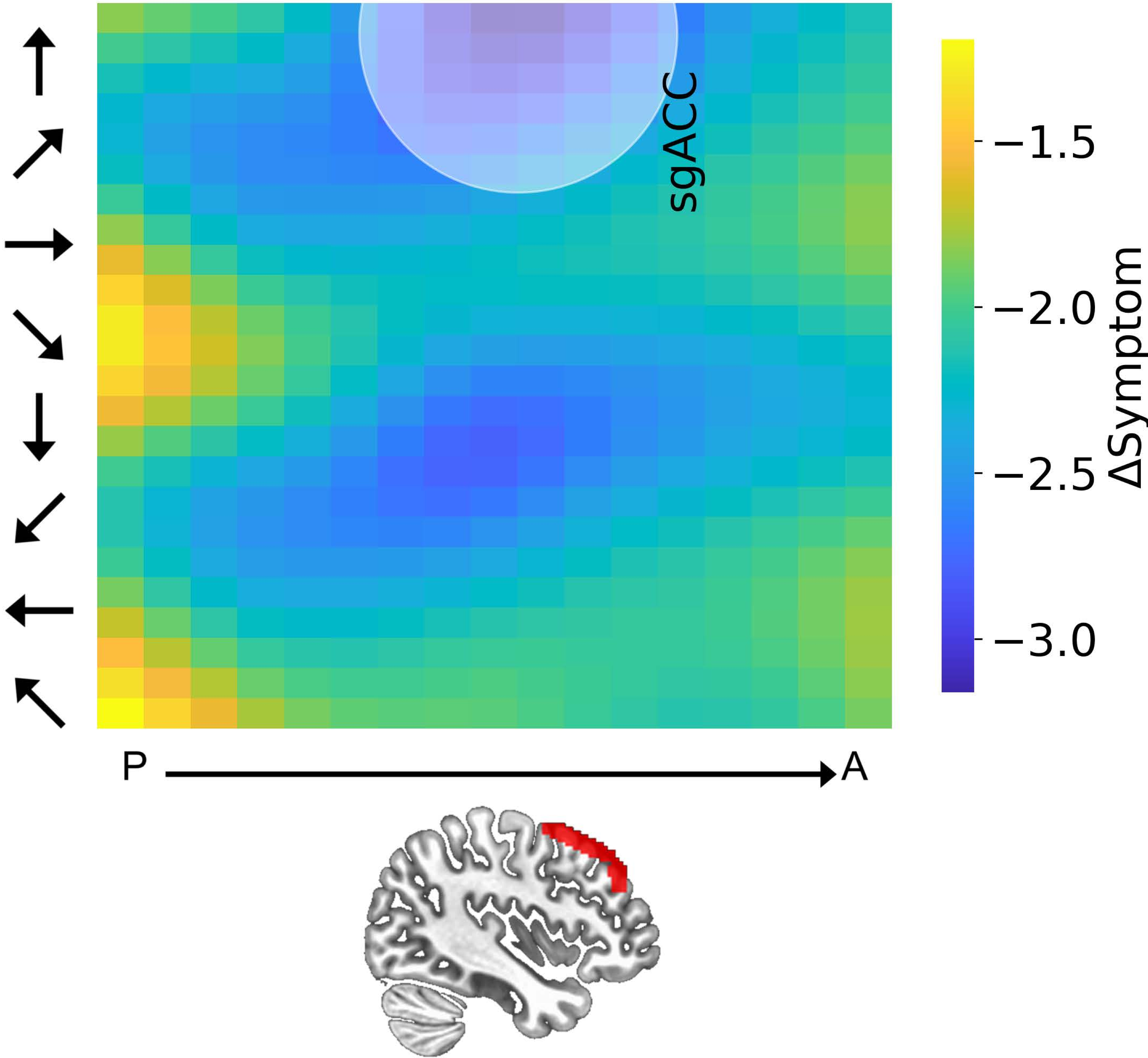

NDARMB943VPJ_madrs_left_dlpfc

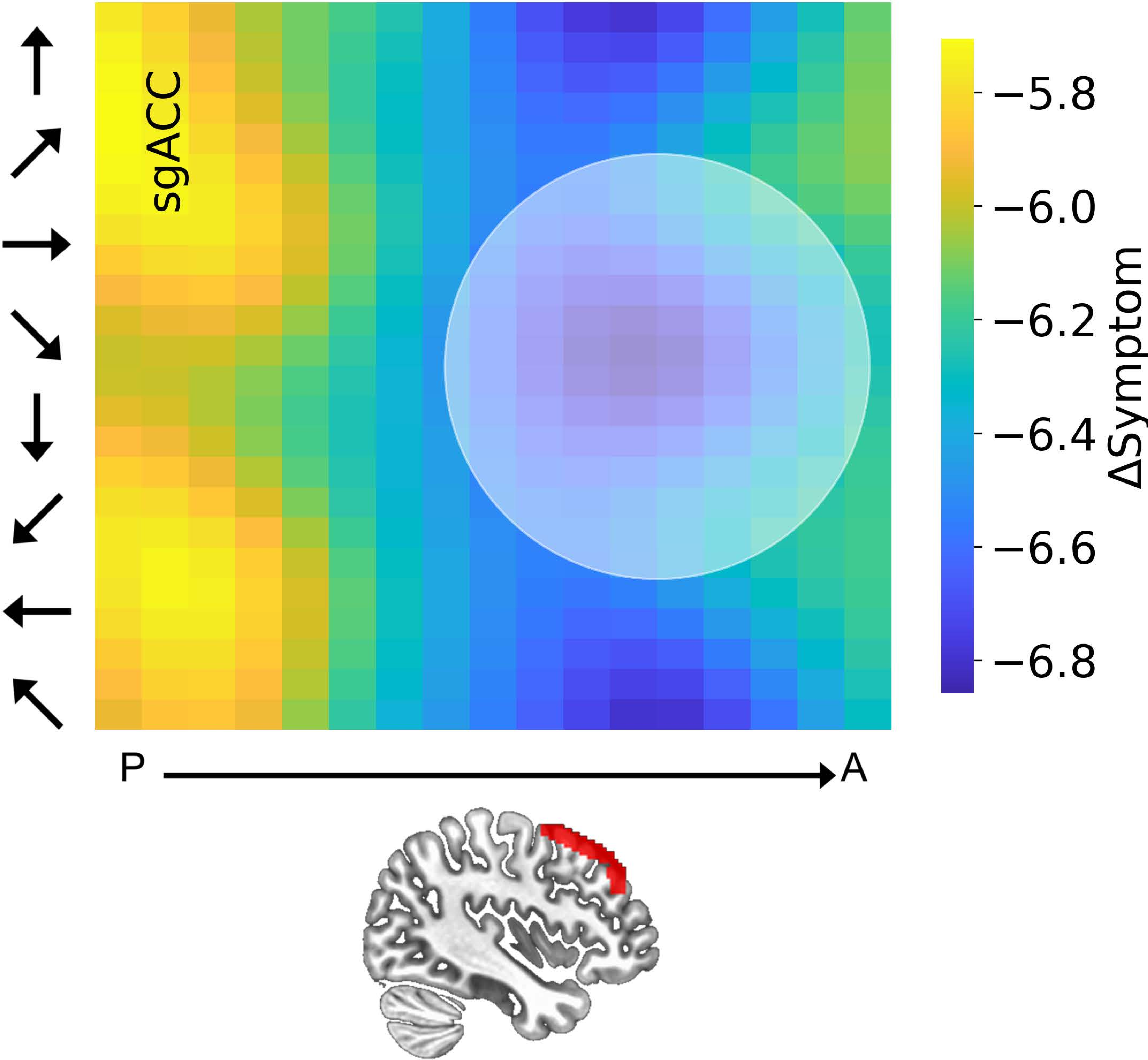

NDARMD016CB8_madrs_left_dlpfc

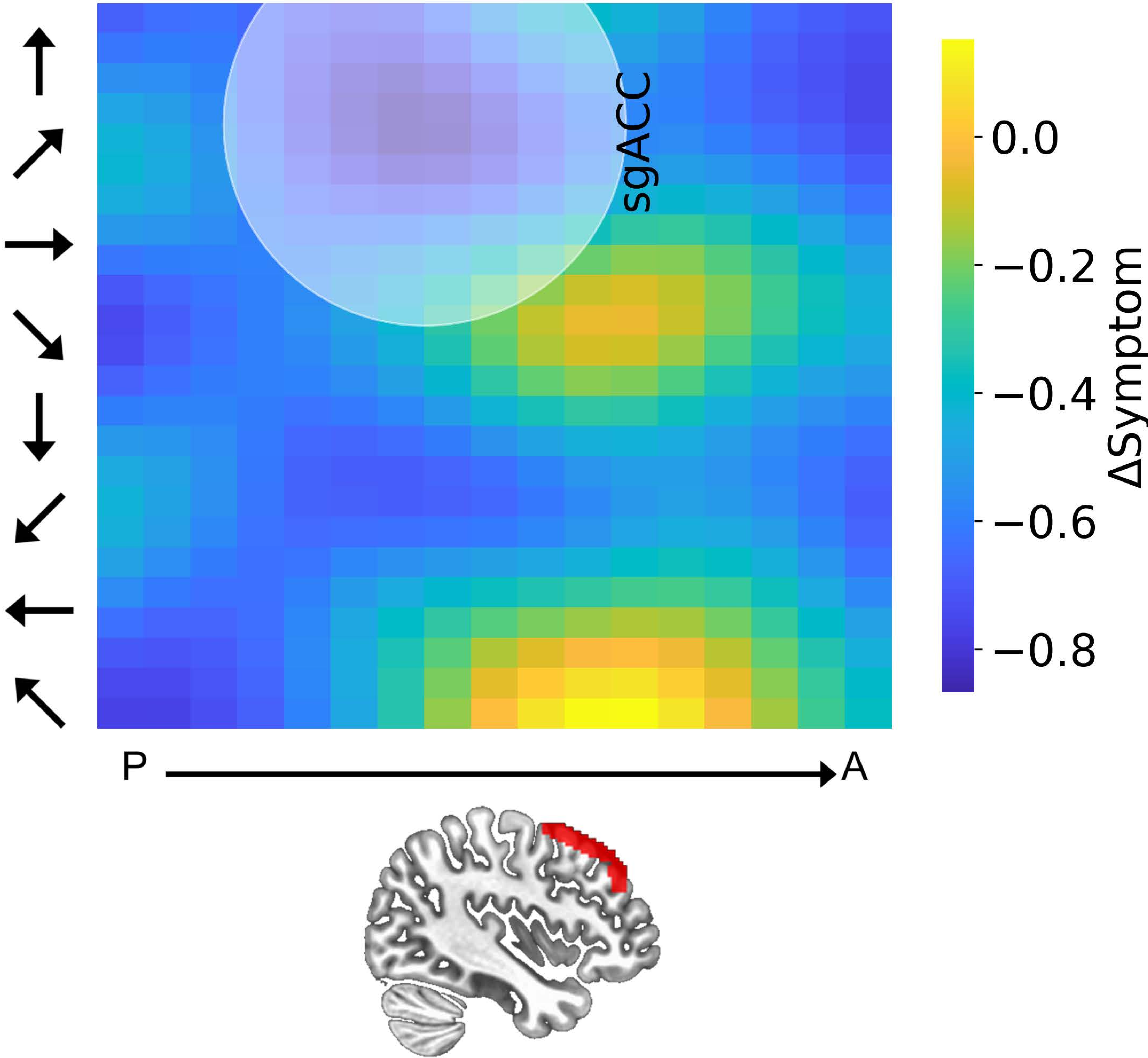

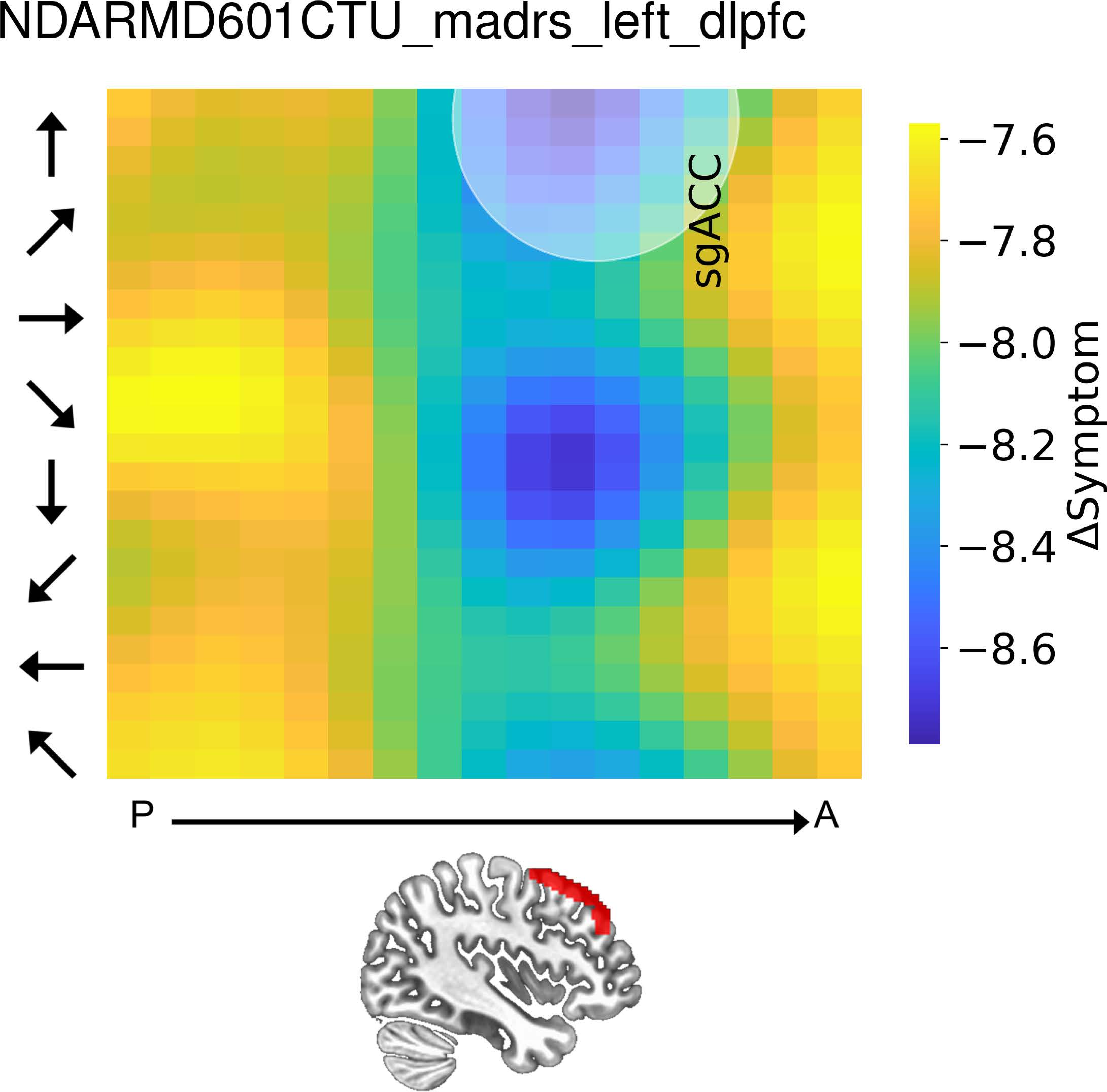
NDARMD601CTU_madrs_left_dlpfc
sgACC
−7.6
−7.8
−8.0
−8.2
−8.4
−8.6
ΔSymptom
P
A

NDARME189AXP_madrs_left_dlpfc

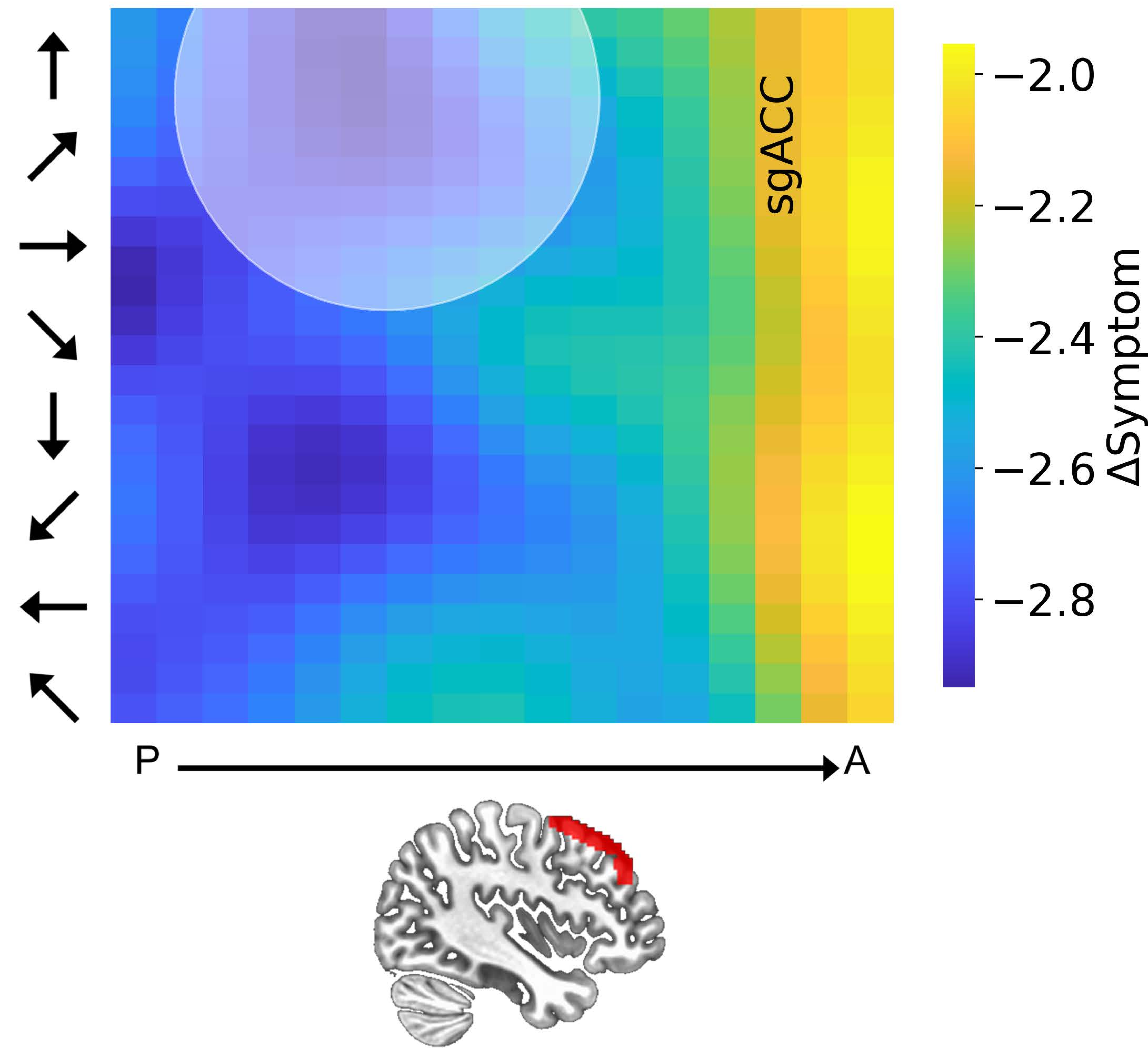

NDARME335LZJ_madrs_left_dlpfc

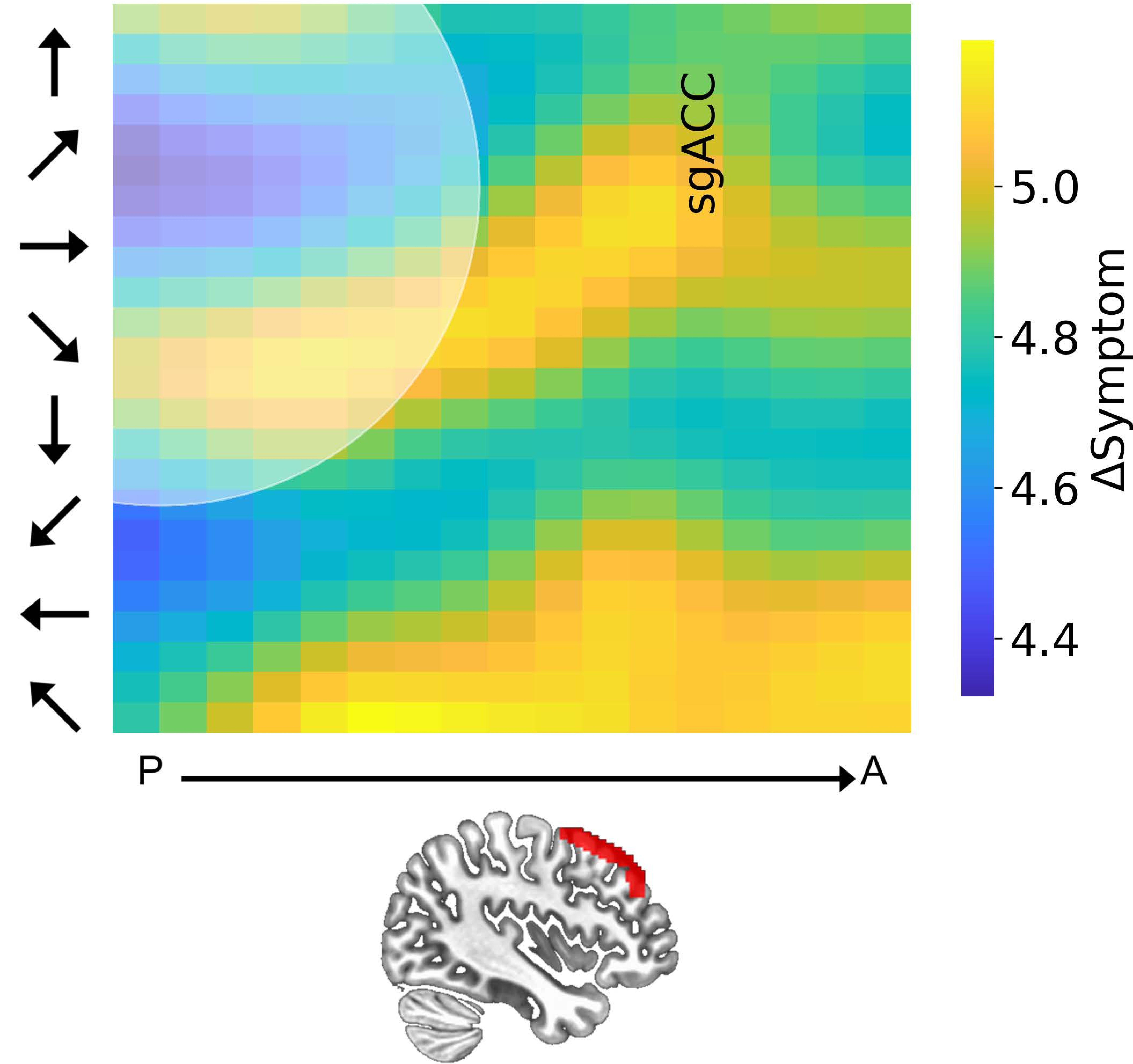

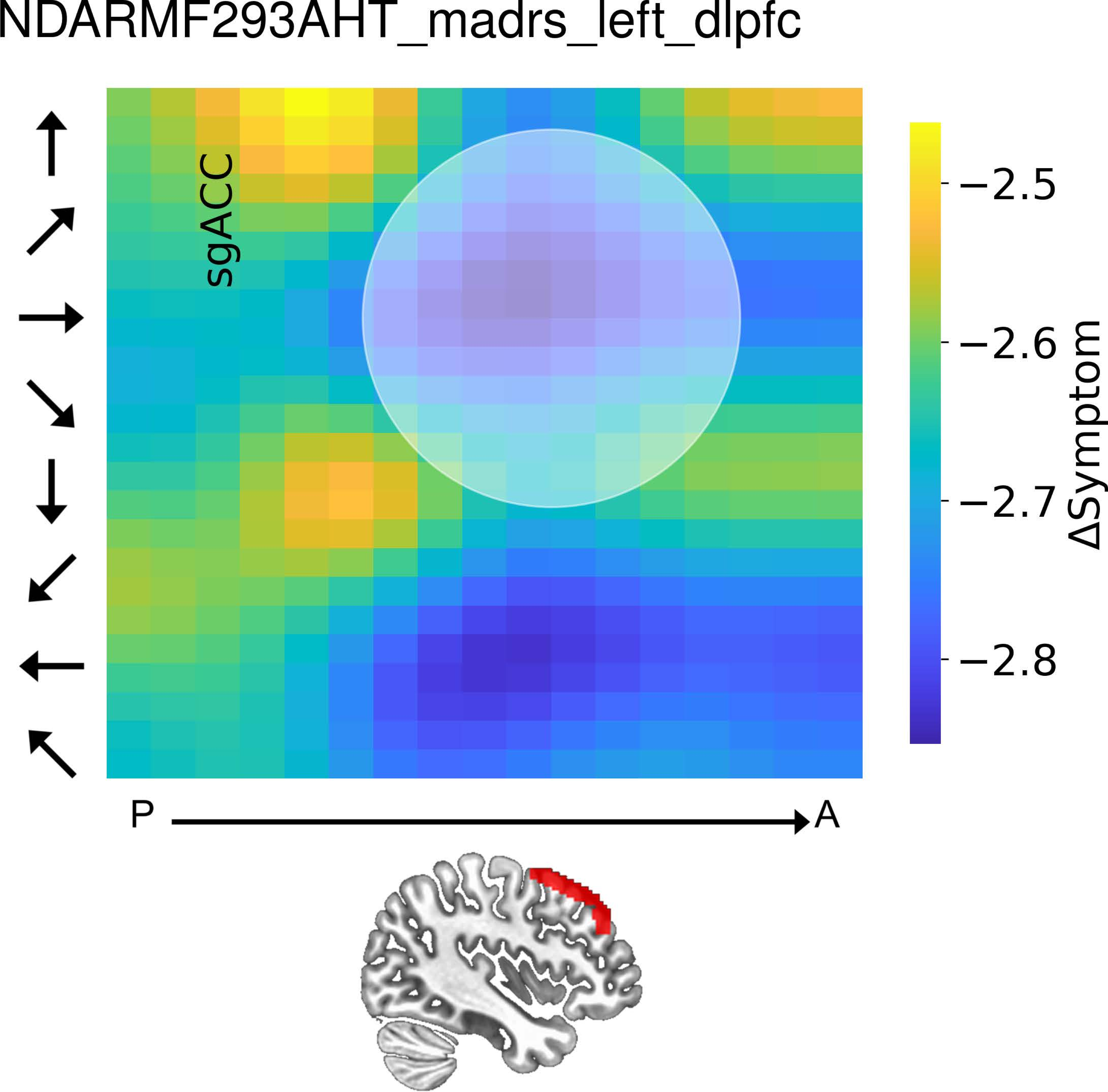
NDARMF293AHT_madrs_left_dlpfc
sgACC
P
A
−2.5
−2.6
−2.7
−2.8
ΔSymptom

NDARMK972BFE_madrs_left_dlpfc

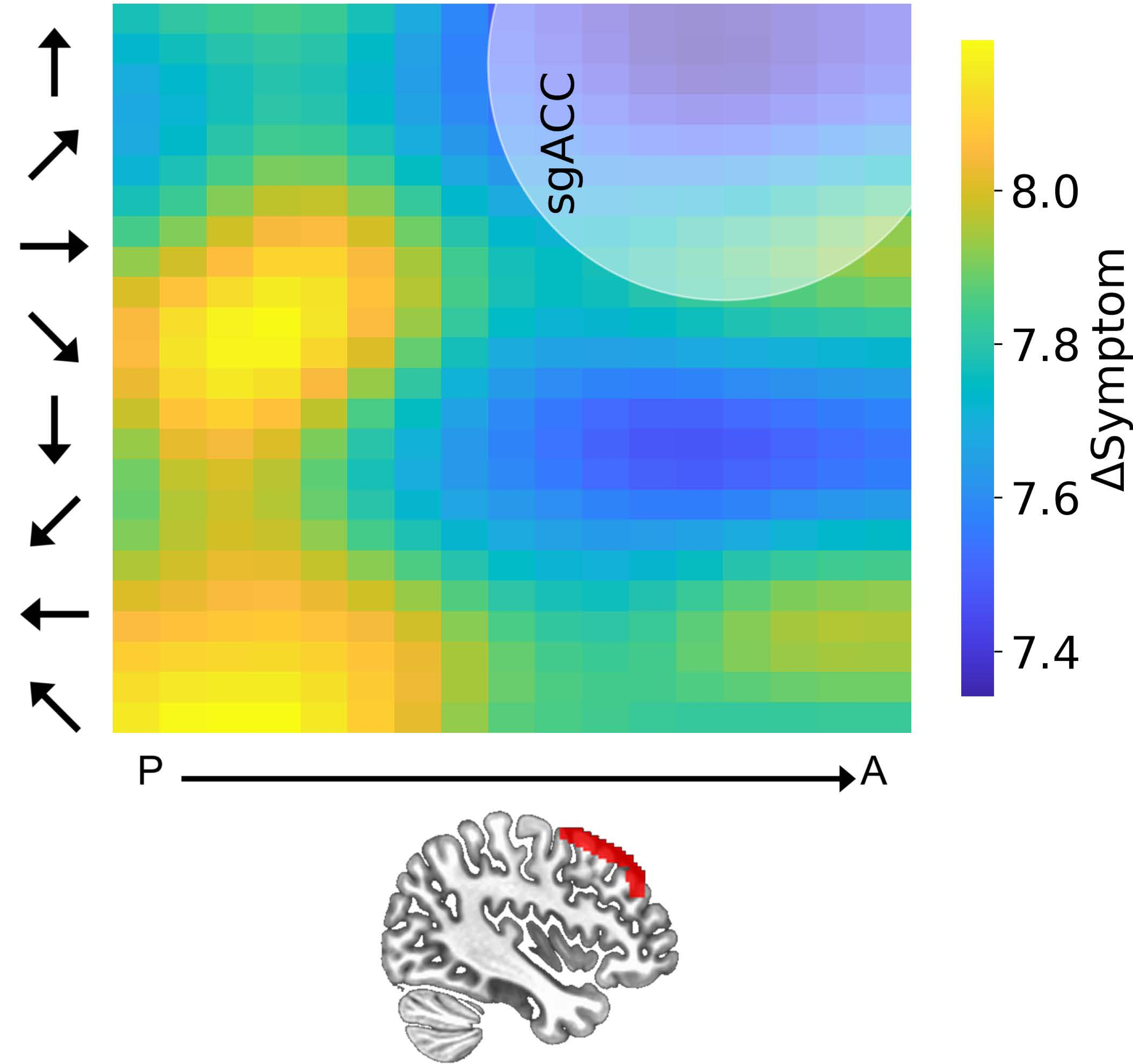

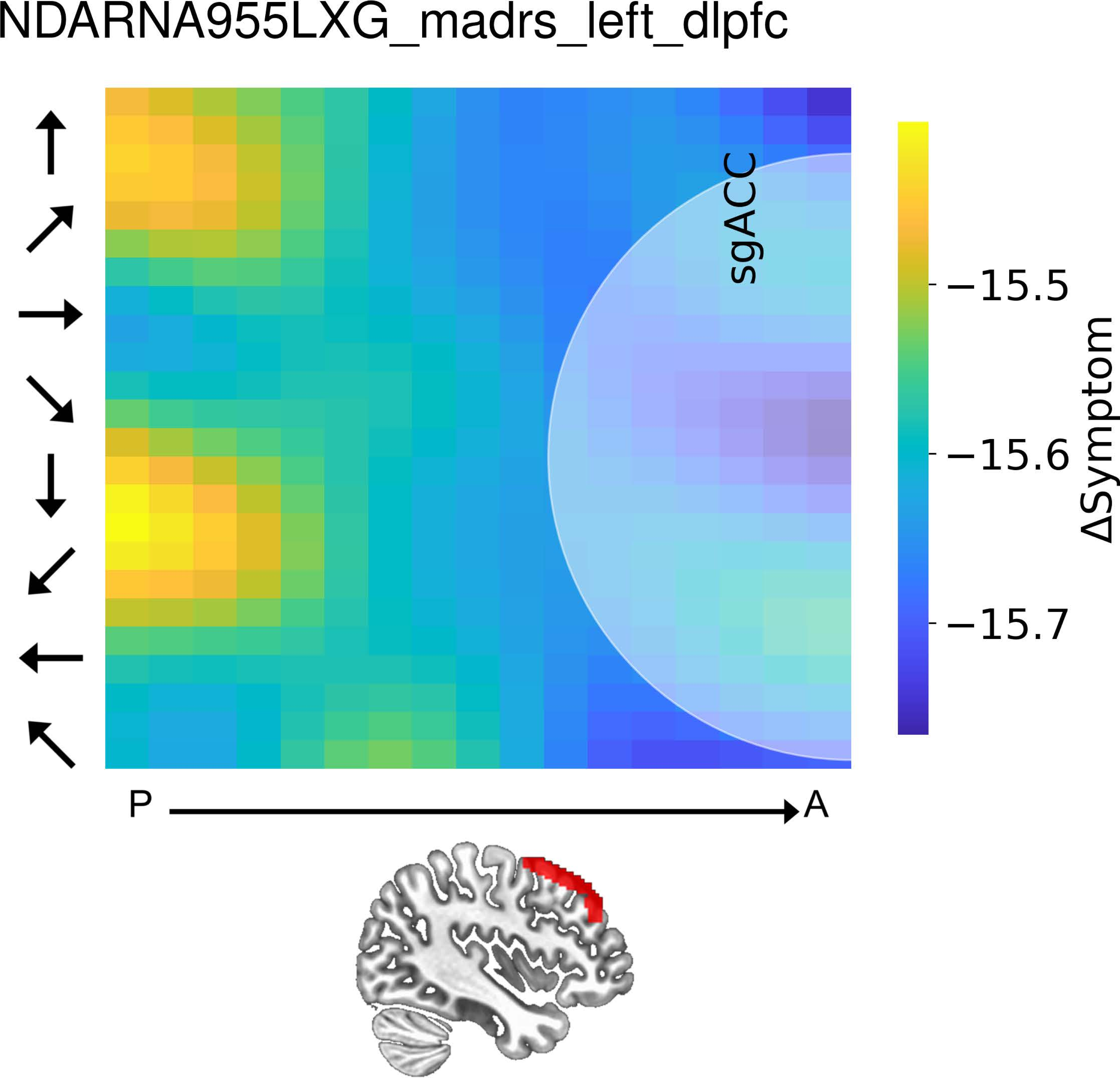
NDARNA955LXG_madrs_left_dlpfc
sgACC
−15.5
−15.6
−15.7
ΔSymptom
P
A

NDARNR764NAK_madrs_left_dlpfc

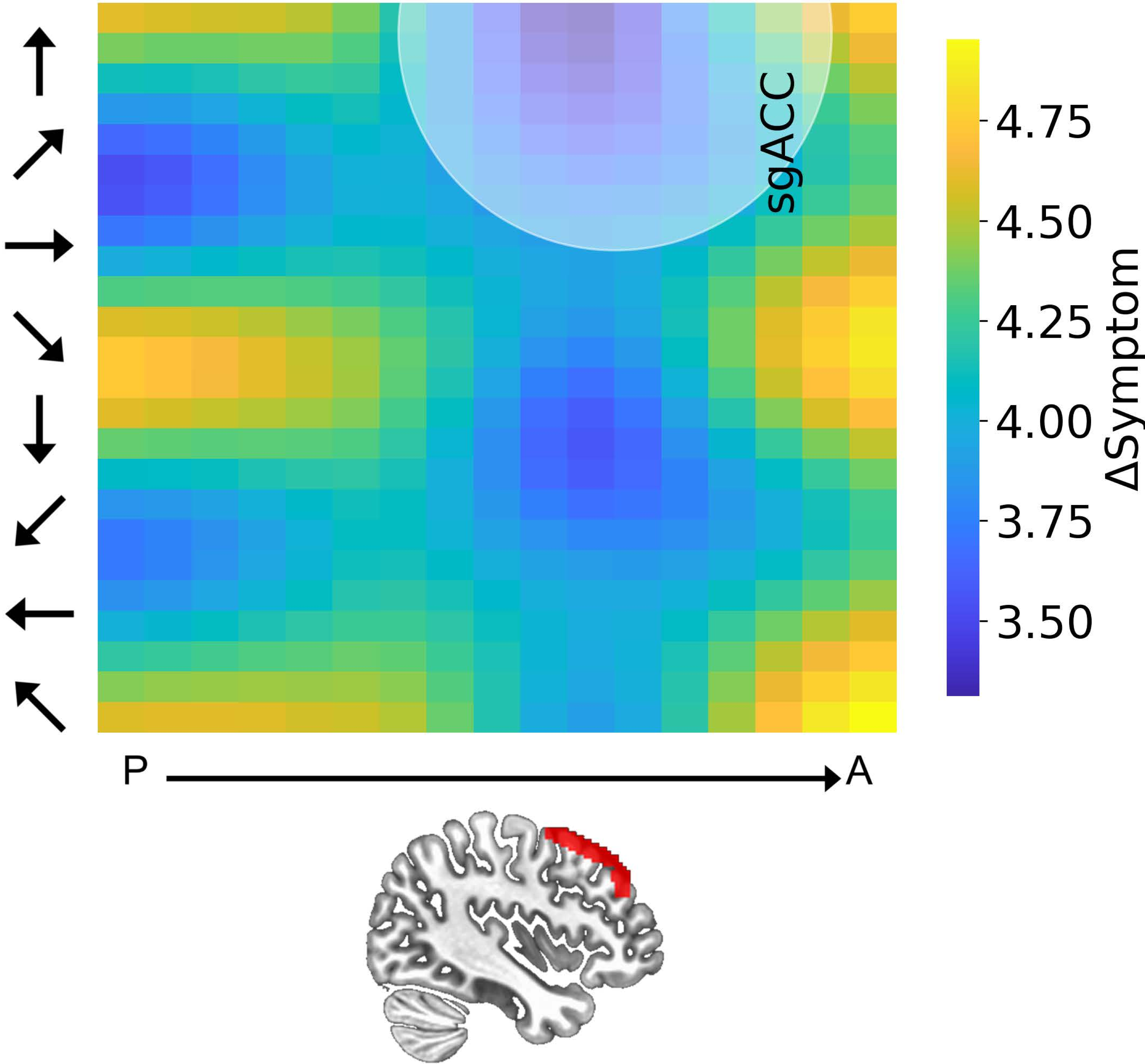

NDARNU180HL3_madrs_left_dlpfc

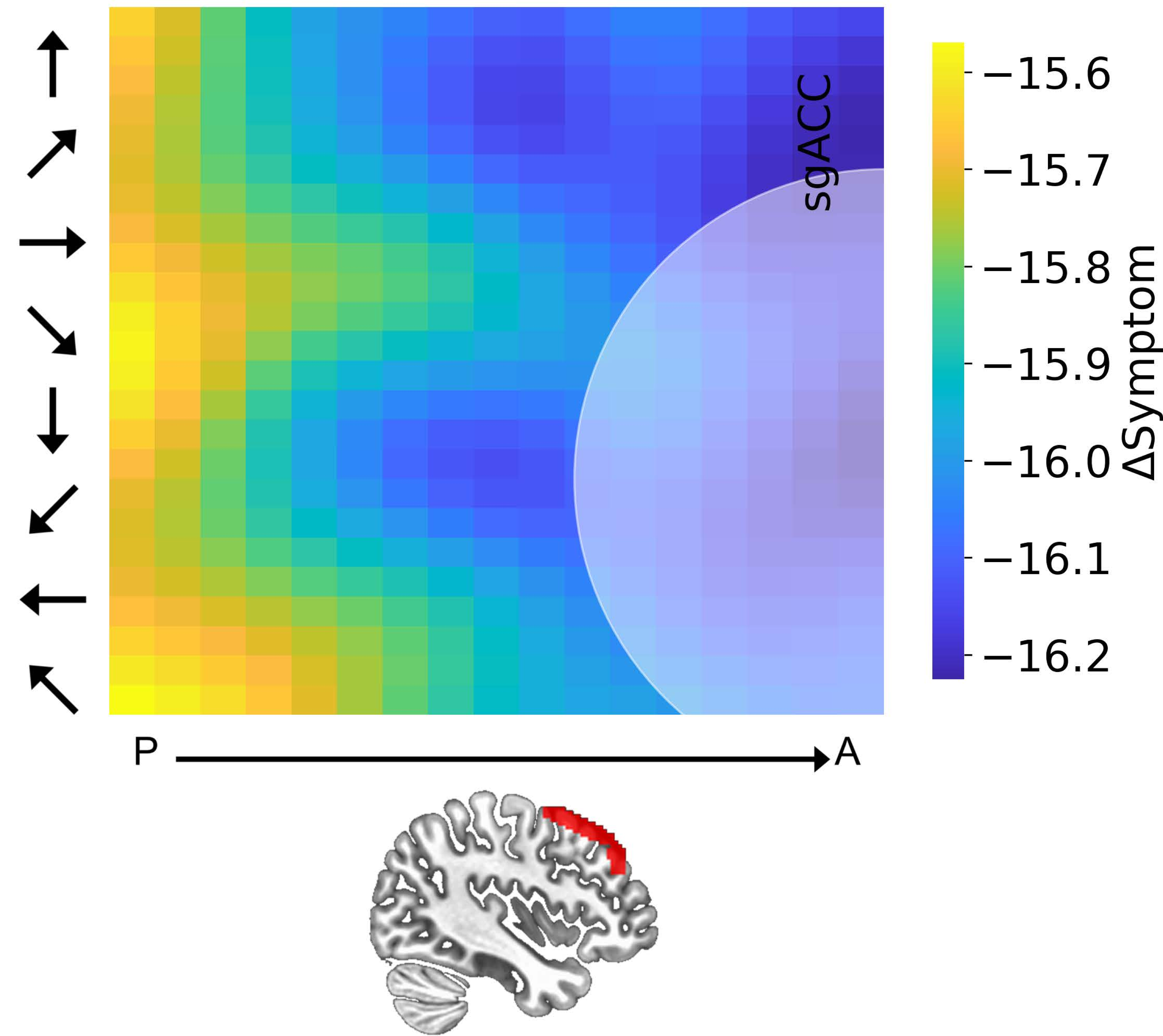

NDARNZ824XHB_madrs_left_dlpfc

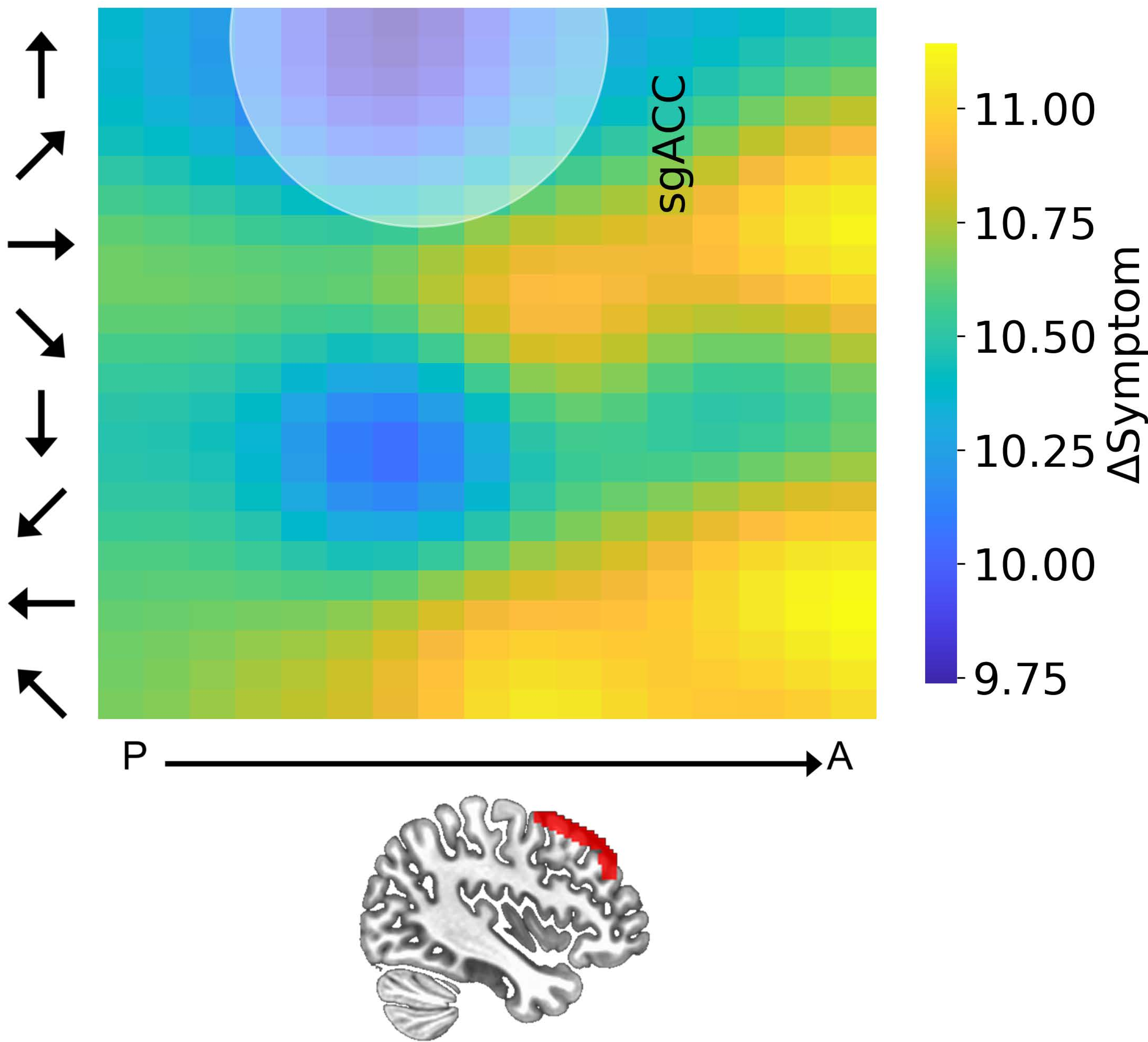

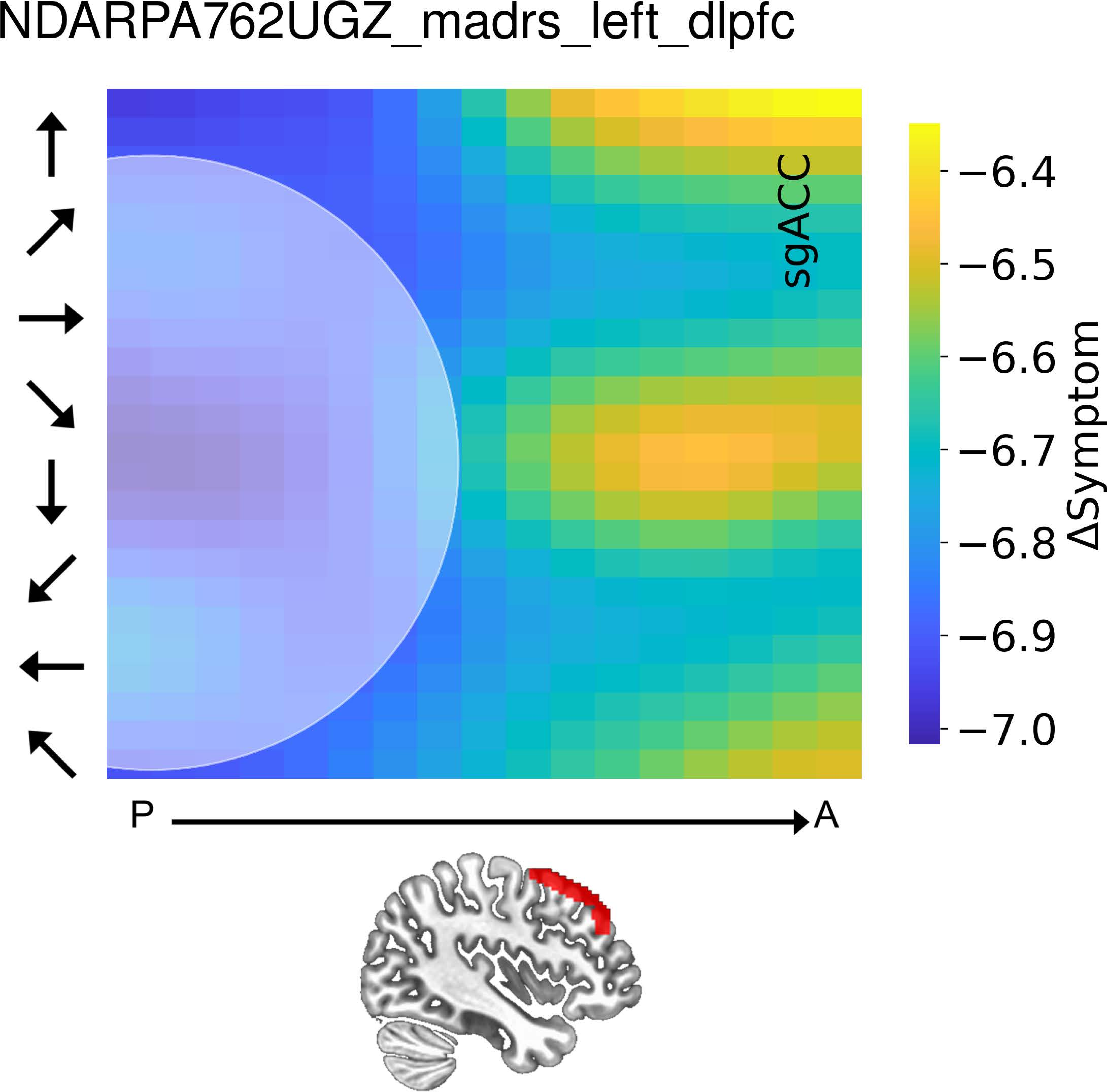

NDARPA762UGZ_madrs_left_dlpfc
sgACC
−6.4
−6.5
−6.6
−6.7
−6.8
−6.9
−7.0
ΔSymptom
P
A

NDARPC197VR3_madrs_left_dlpfc

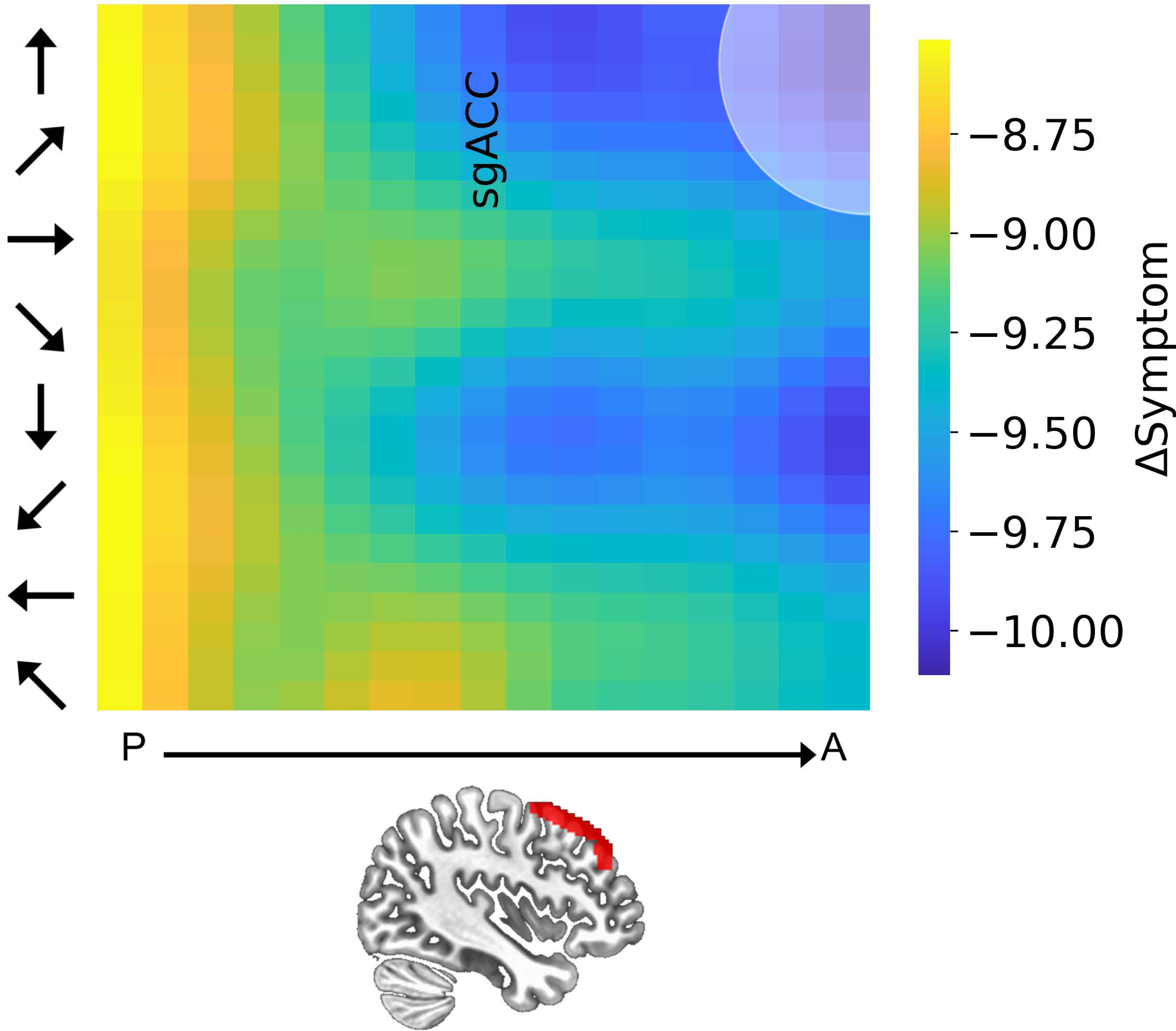

NDARPF499HVH_madrs_left_dlpfc

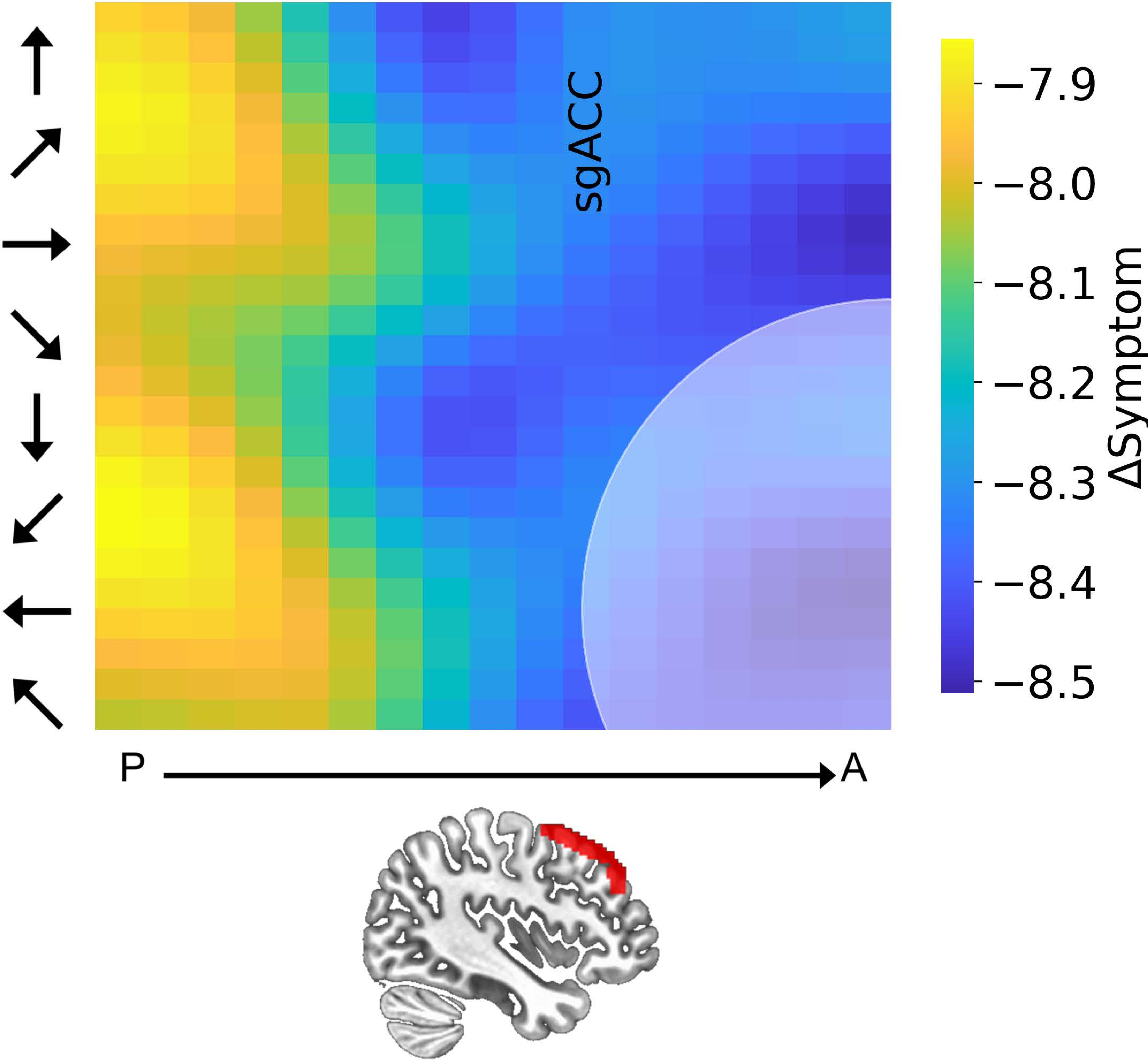

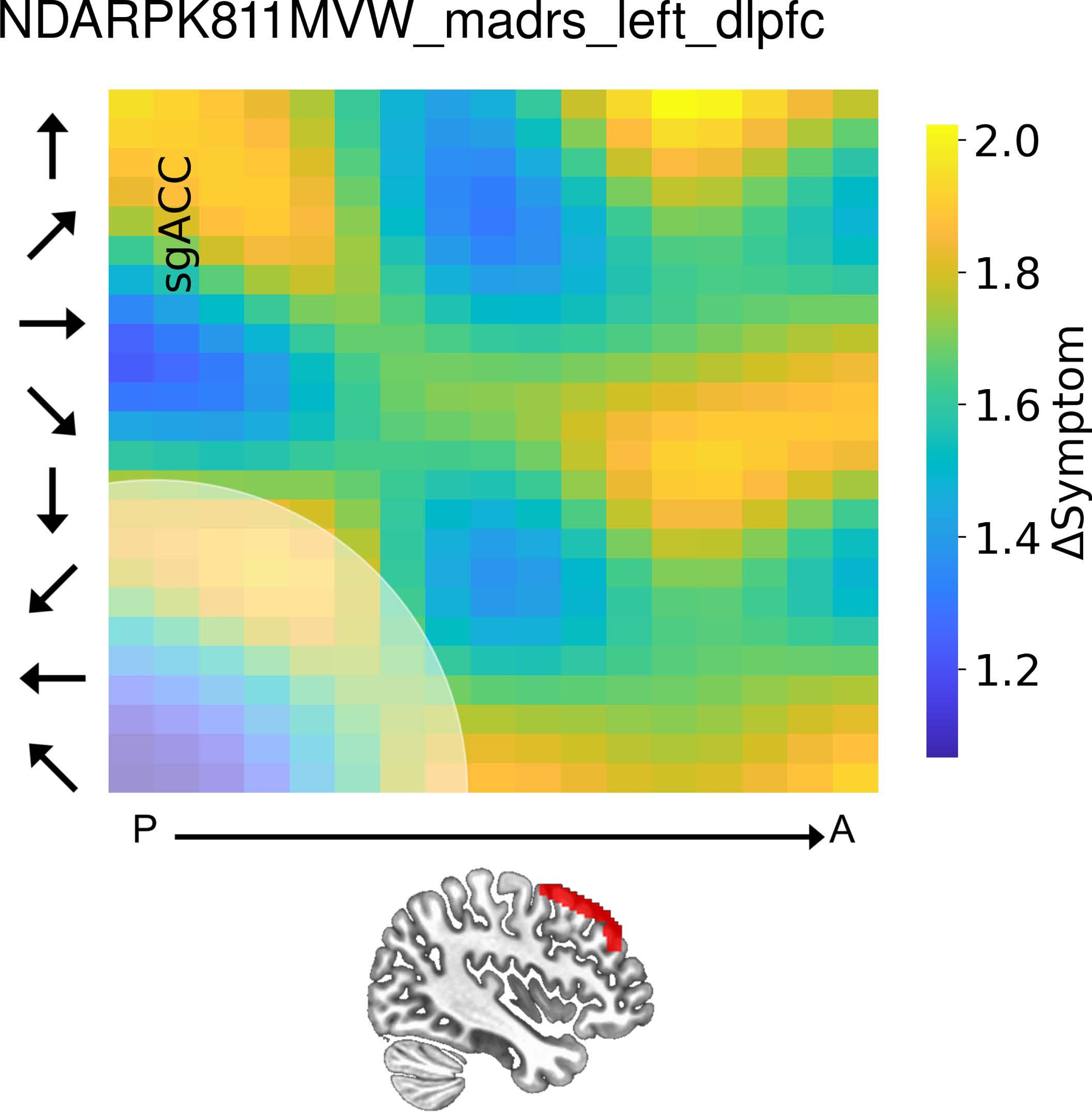
NDARPK811MVW_madrs_left_dlpfc
sgACC
2.0
1.8
1.6
1.4
1.2
ΔSymptom
P
A

NDARPT040EGR_madrs_left_dlpfc

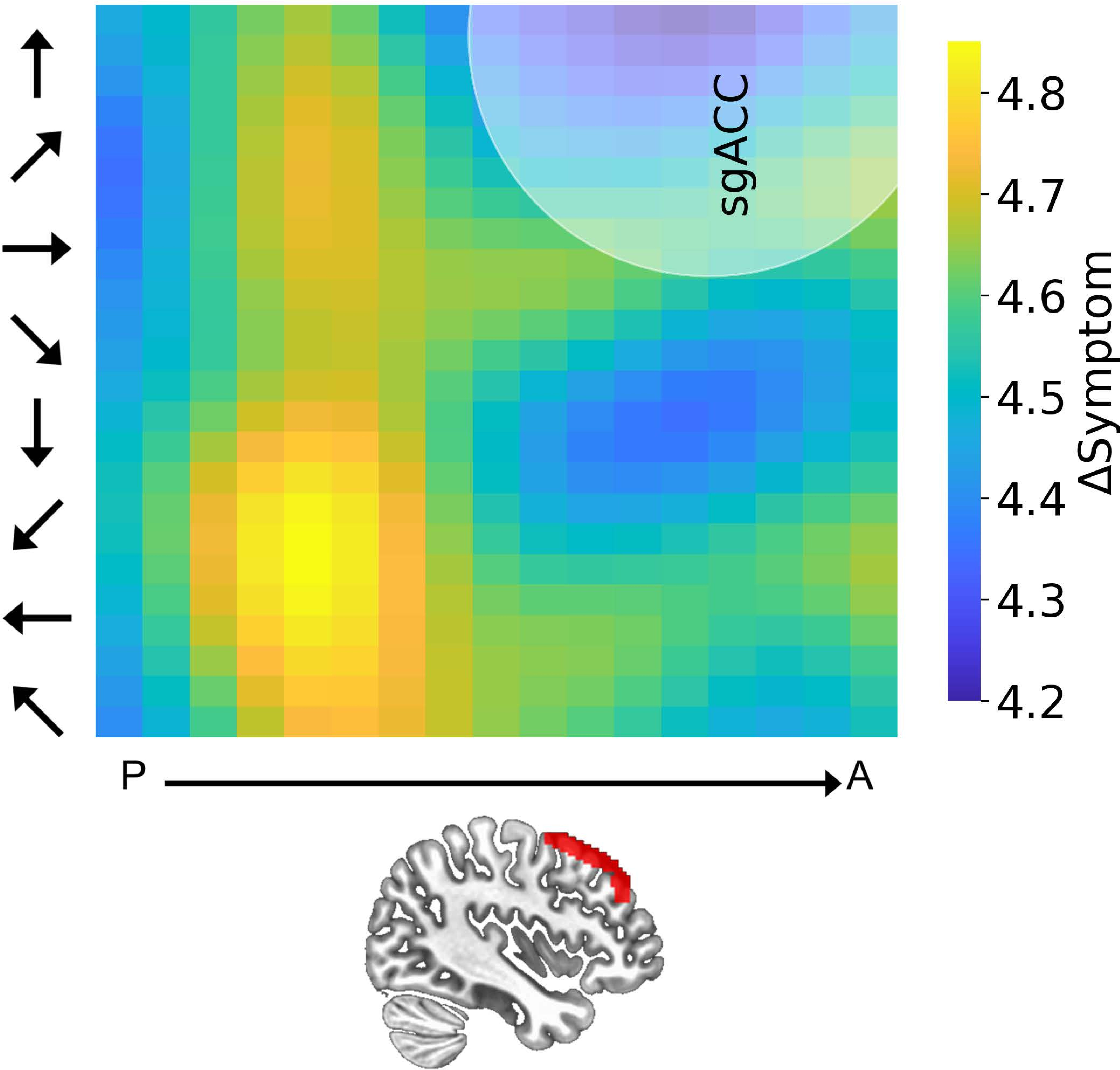

NDARPW706YNA_madrs_left_dlpfc

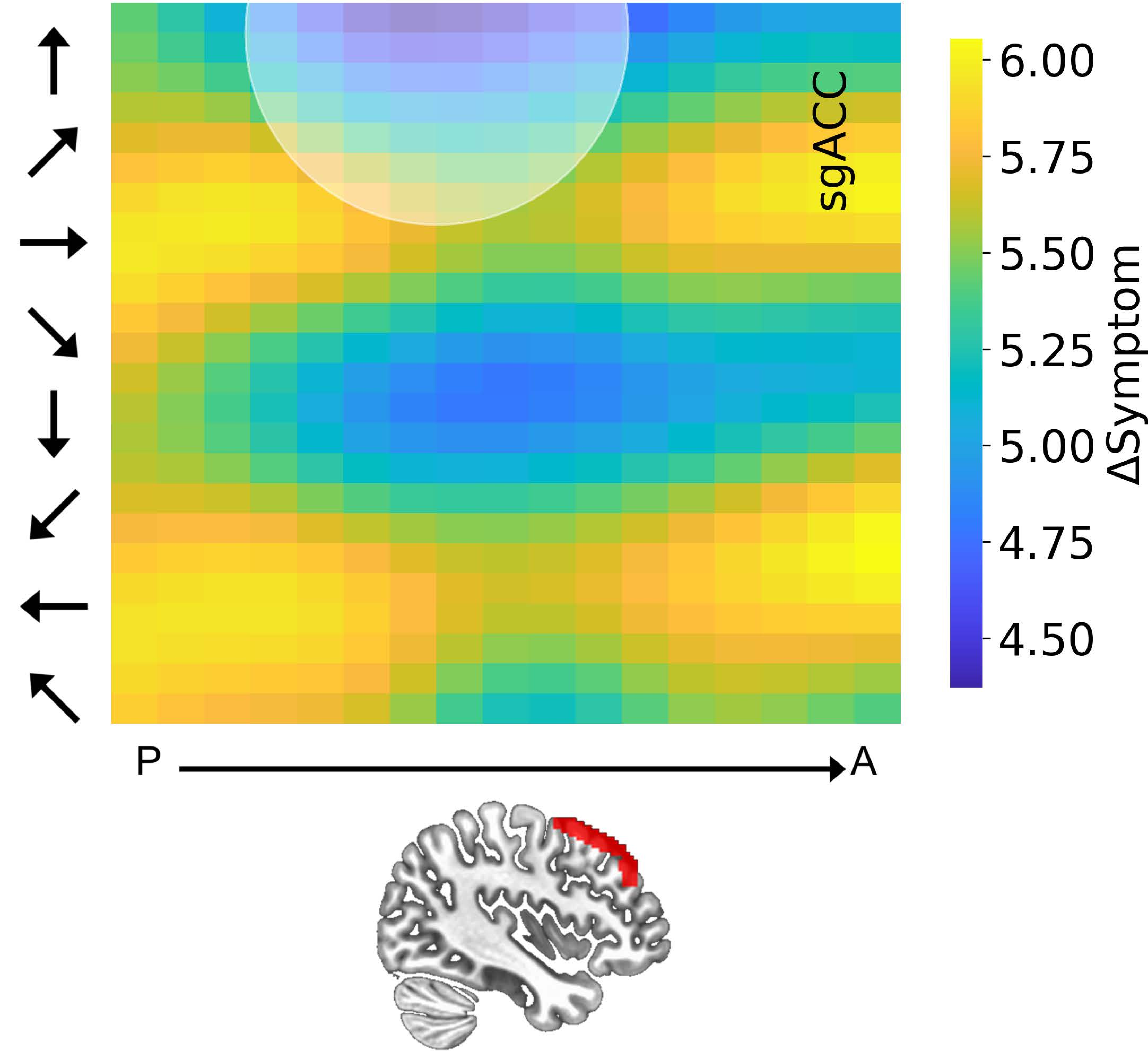

NDARPZ537YY0_madrs_left_dlpfc

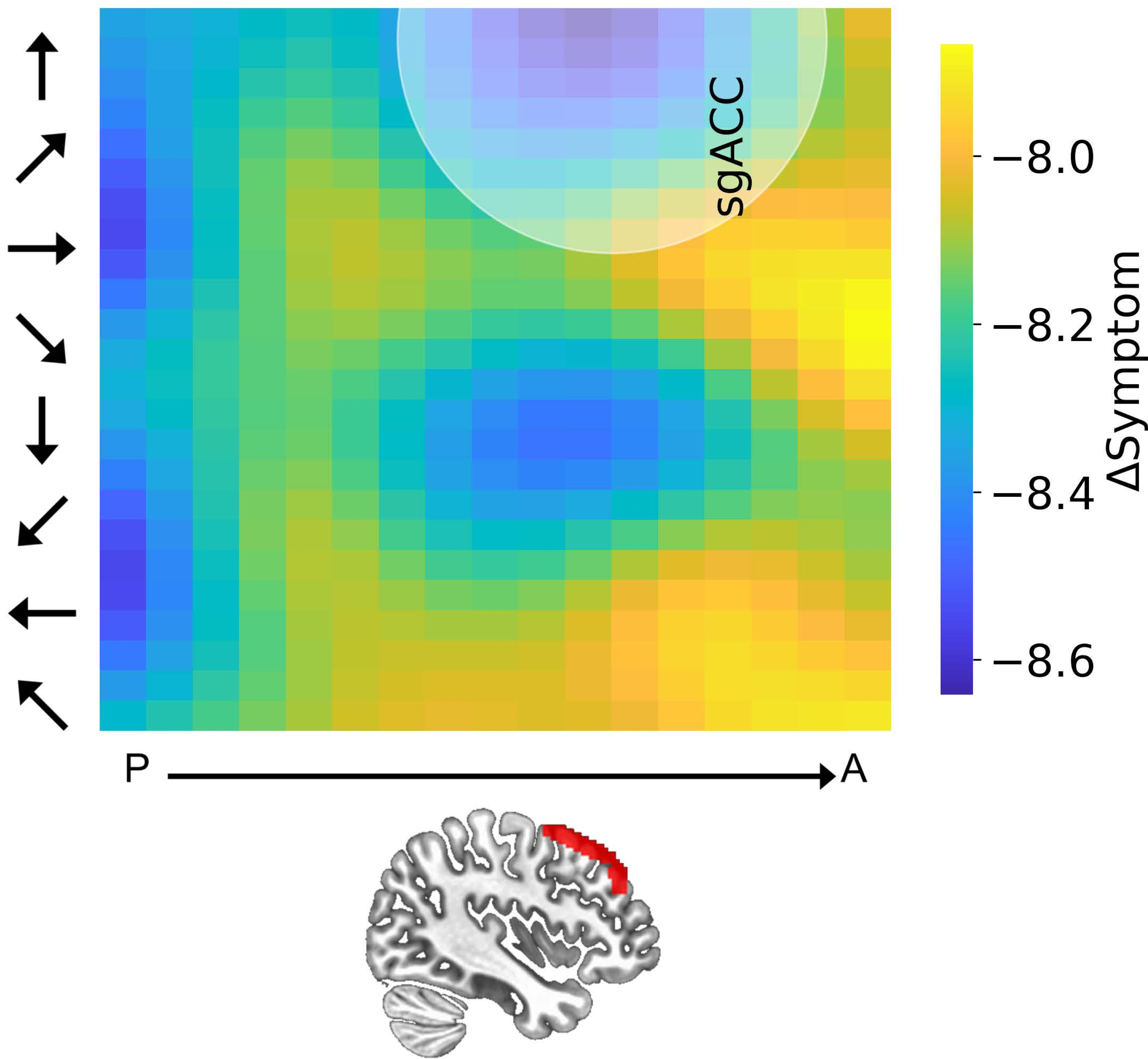

NDARRD555DDH_madrs_left_dlpfc

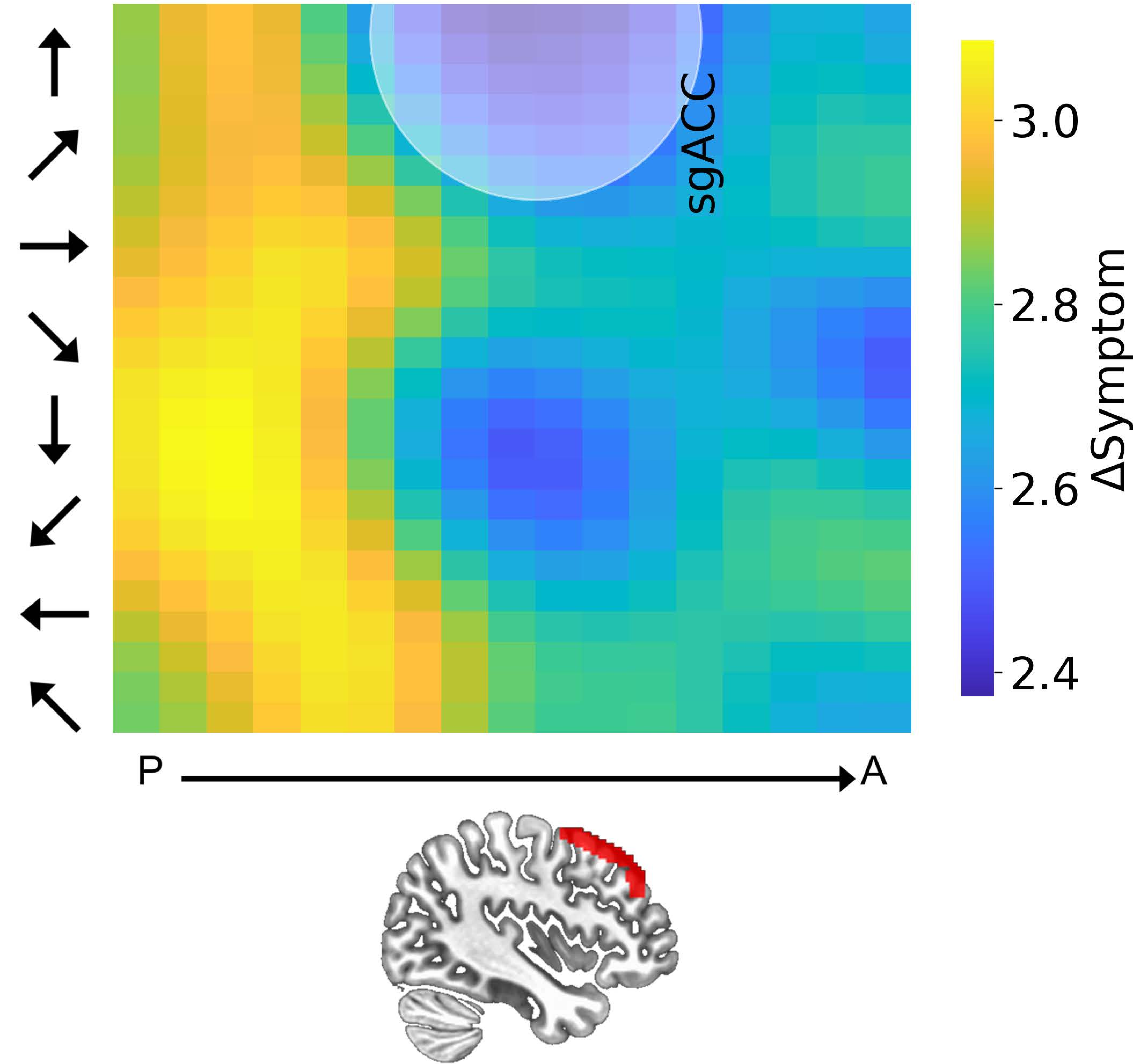

NDARRF879TA3_madrs_left_dlpfc

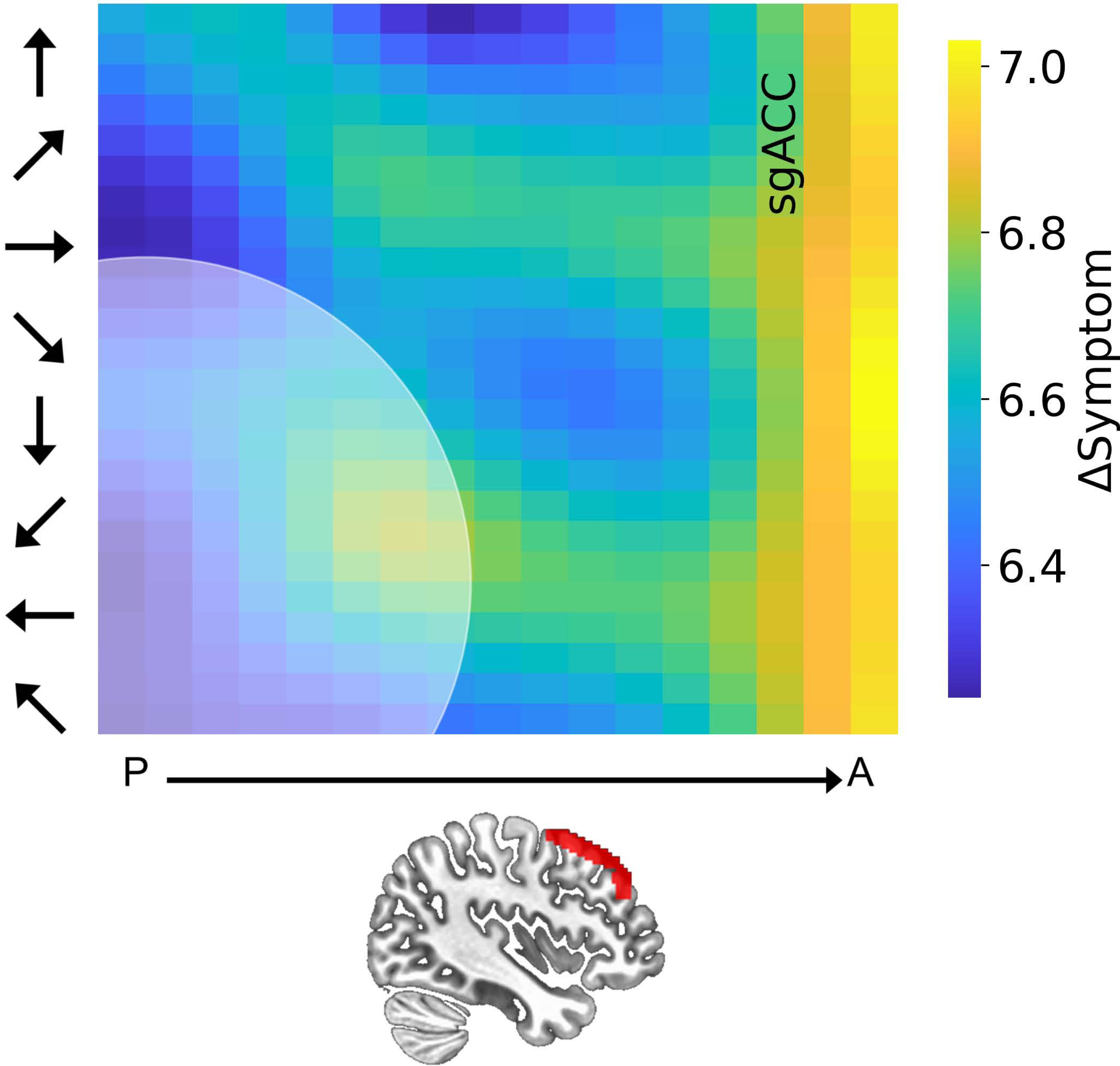

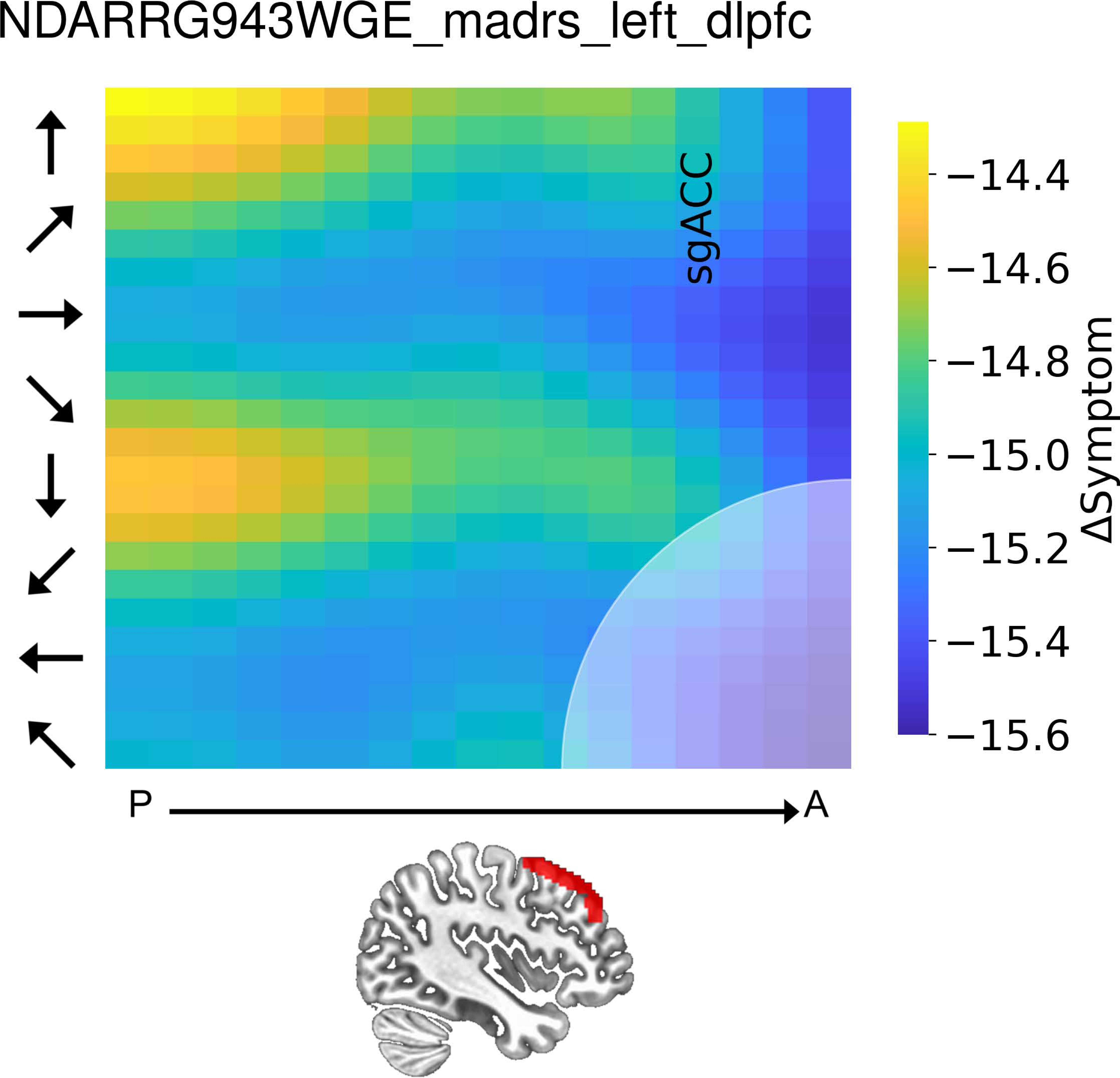
NDARRG943WGE_madrs_left_dlpfc
sgACC
−14.4
−14.6
−14.8
−15.0
−15.2
−15.4
−15.6
ΔSymptom
P
A

NDARRM328EP8_madrs_left_dlpfc

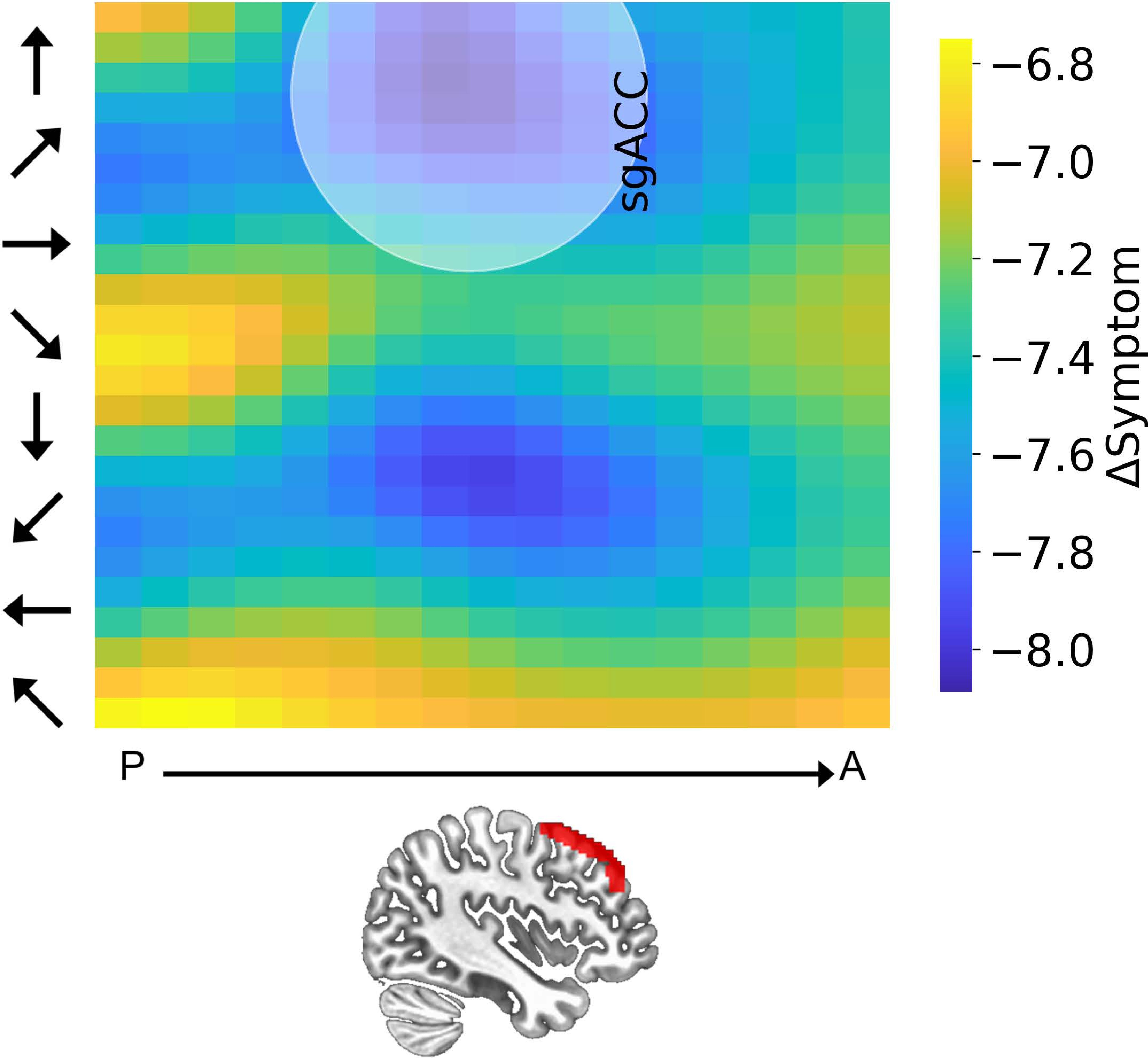

NDARRZ612NKG_madrs_left_dlpfc

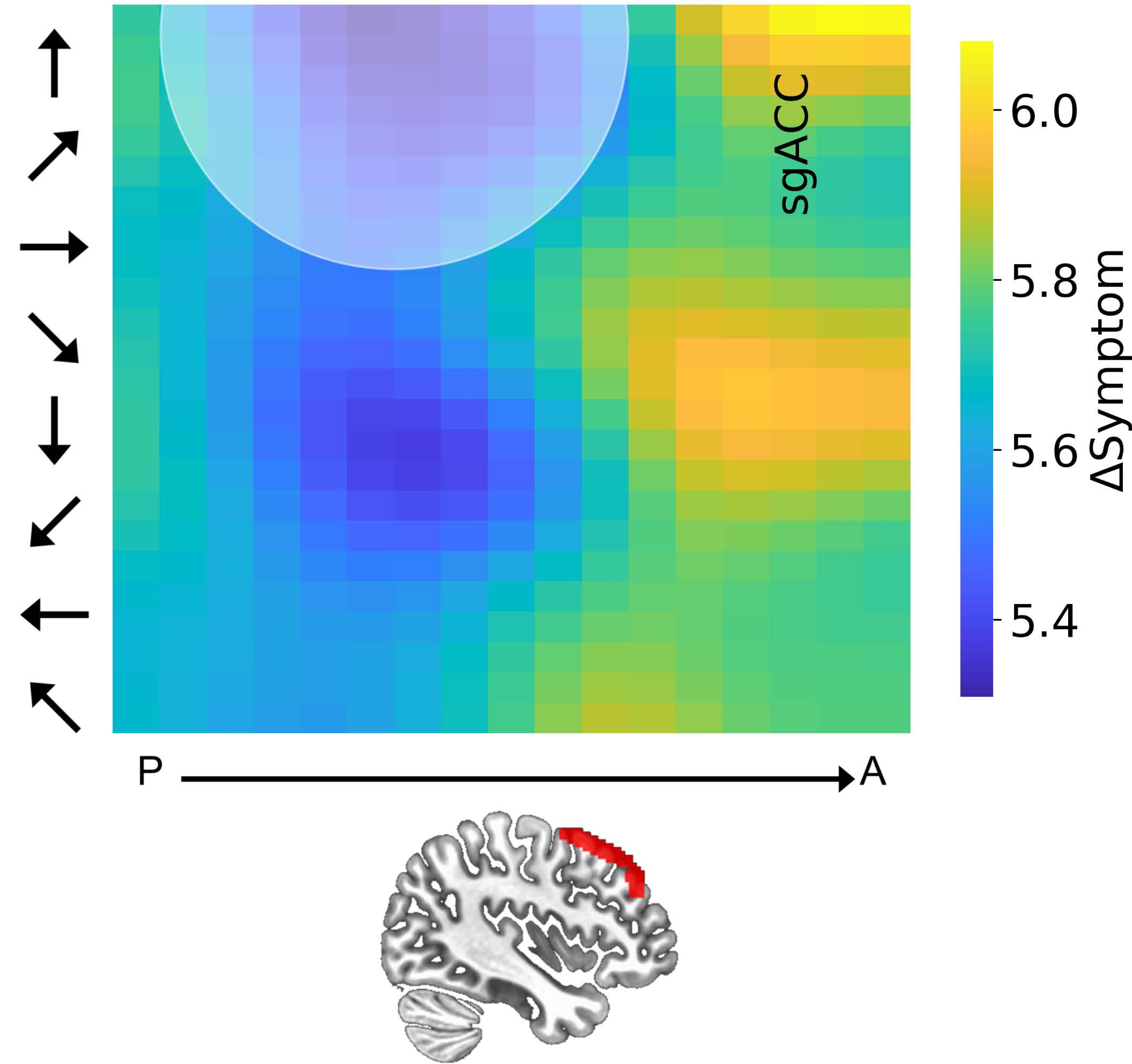

NDARTH509MNV_madrs_left_dlpfc

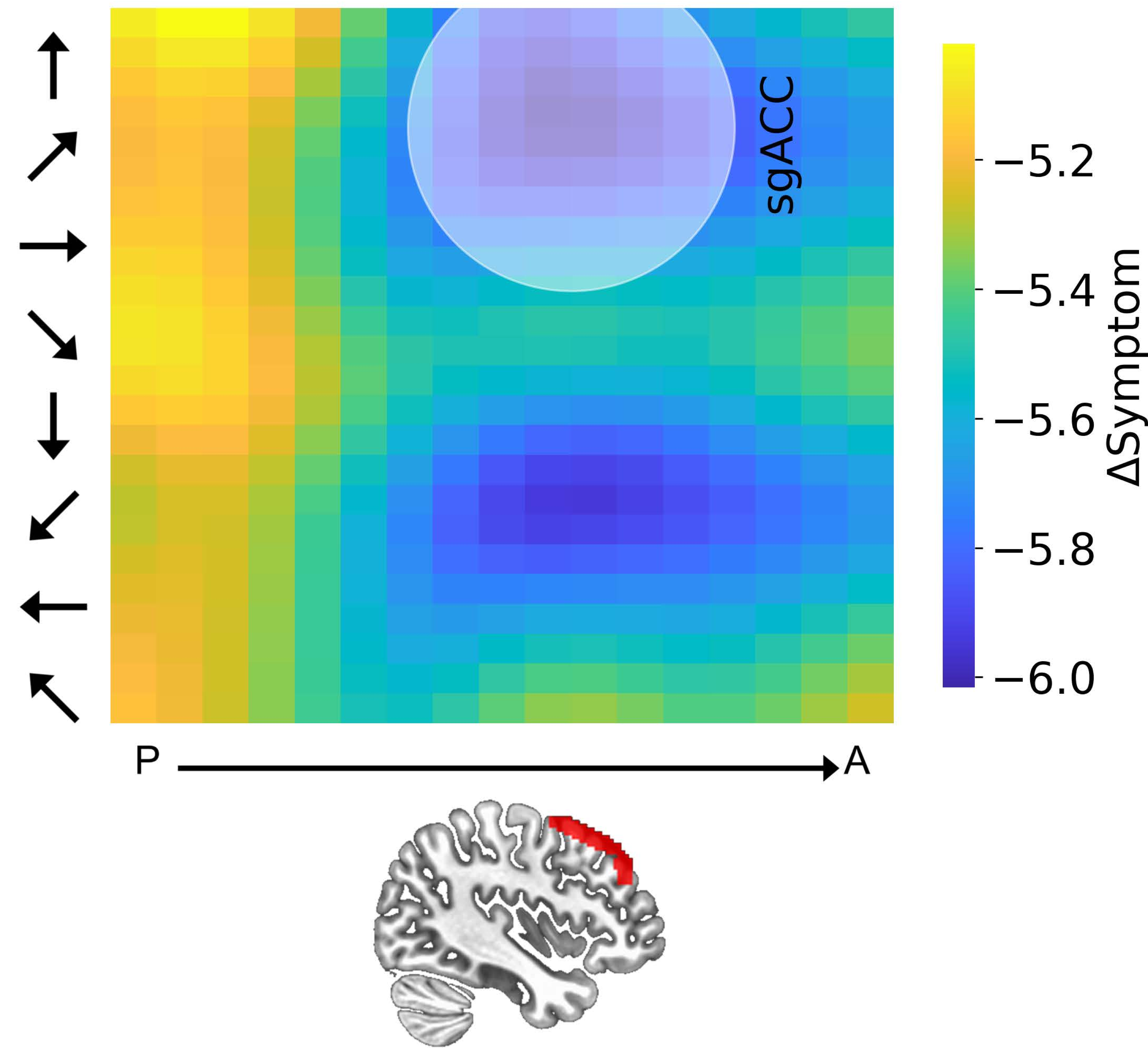

NDARTT796GJK_madrs_left_dlpfc

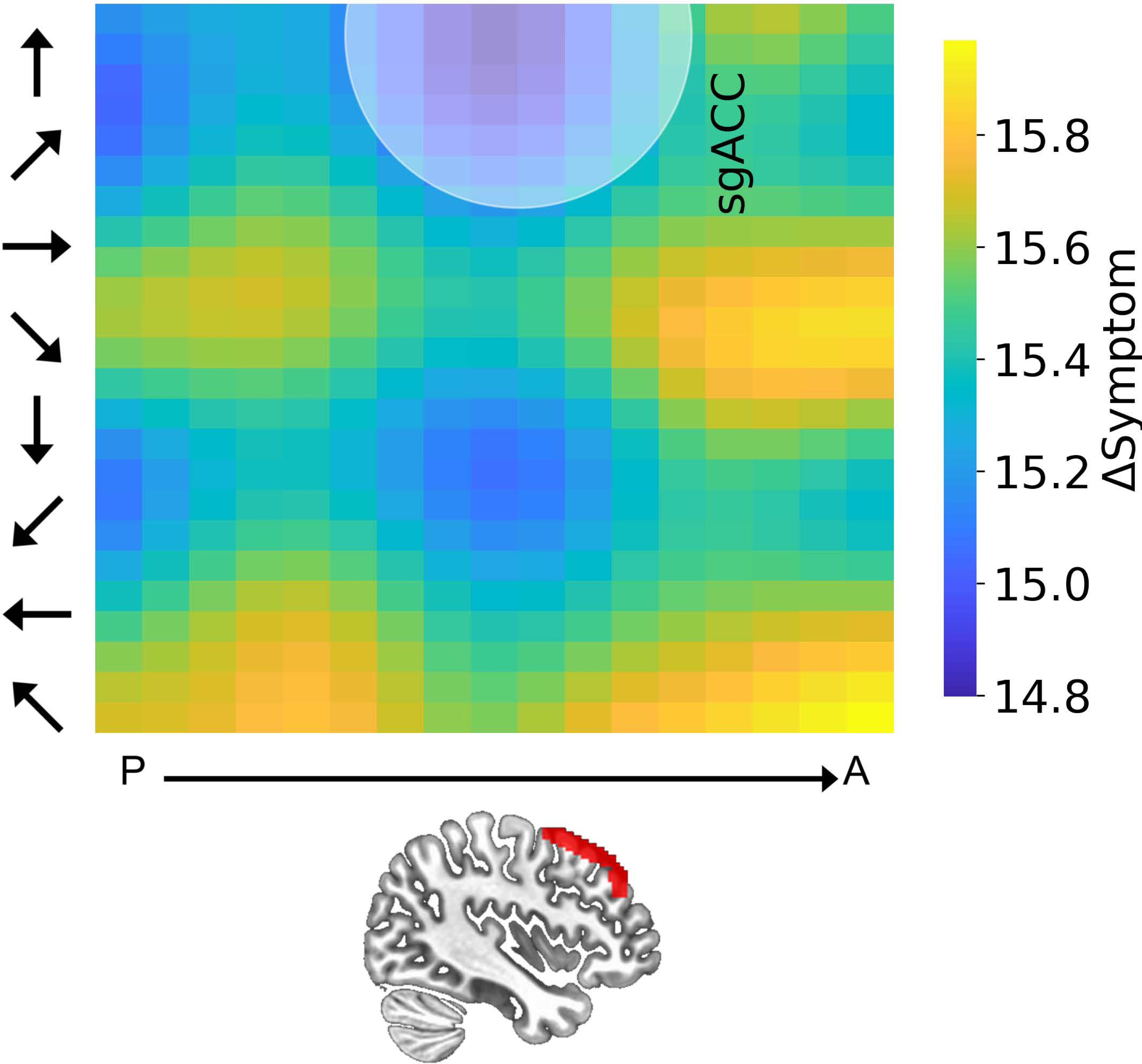

NDARTZ590DYE_madrs_left_dlpfc

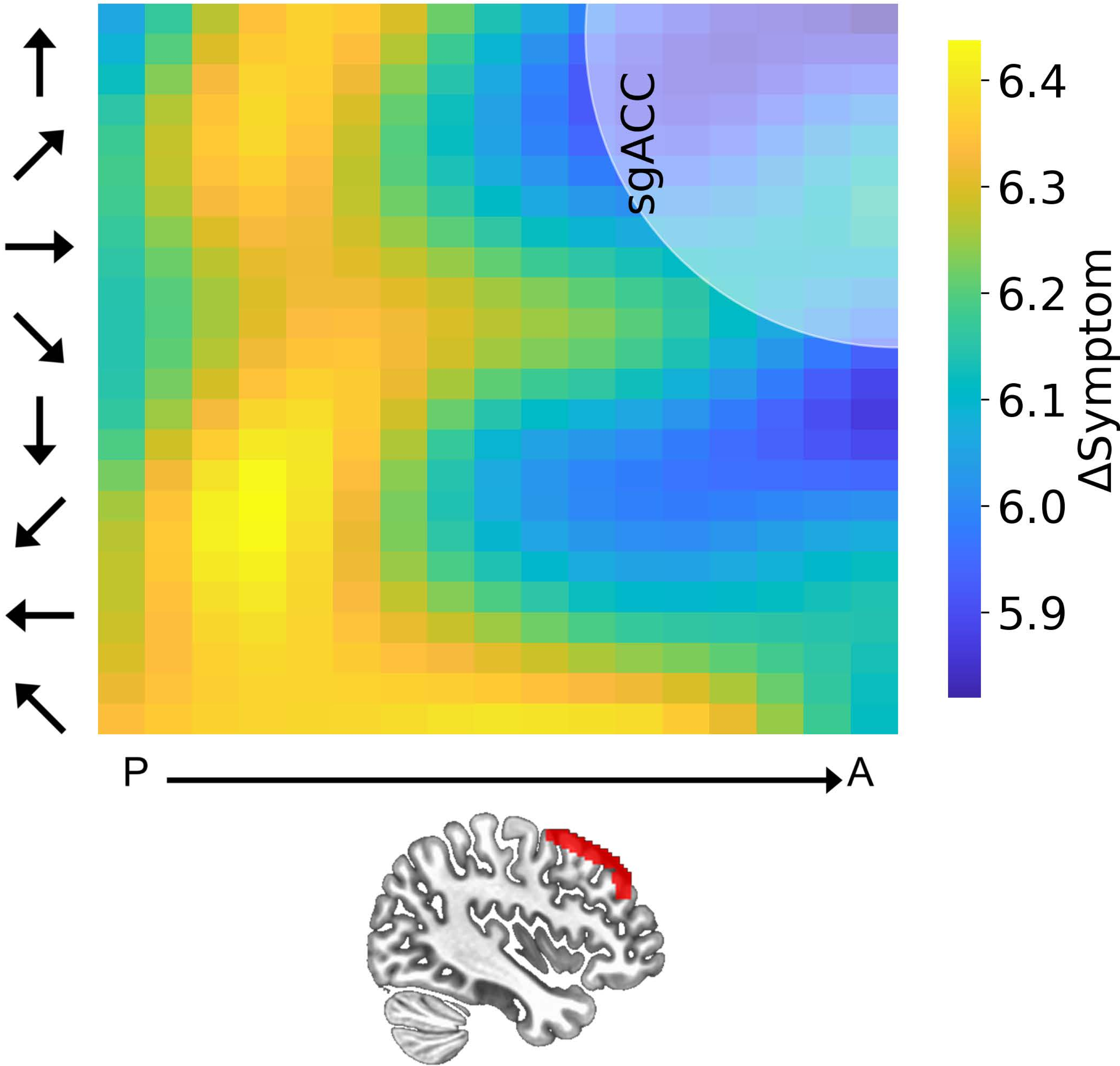

NDARUJ223ZPN_madrs_left_dlpfc

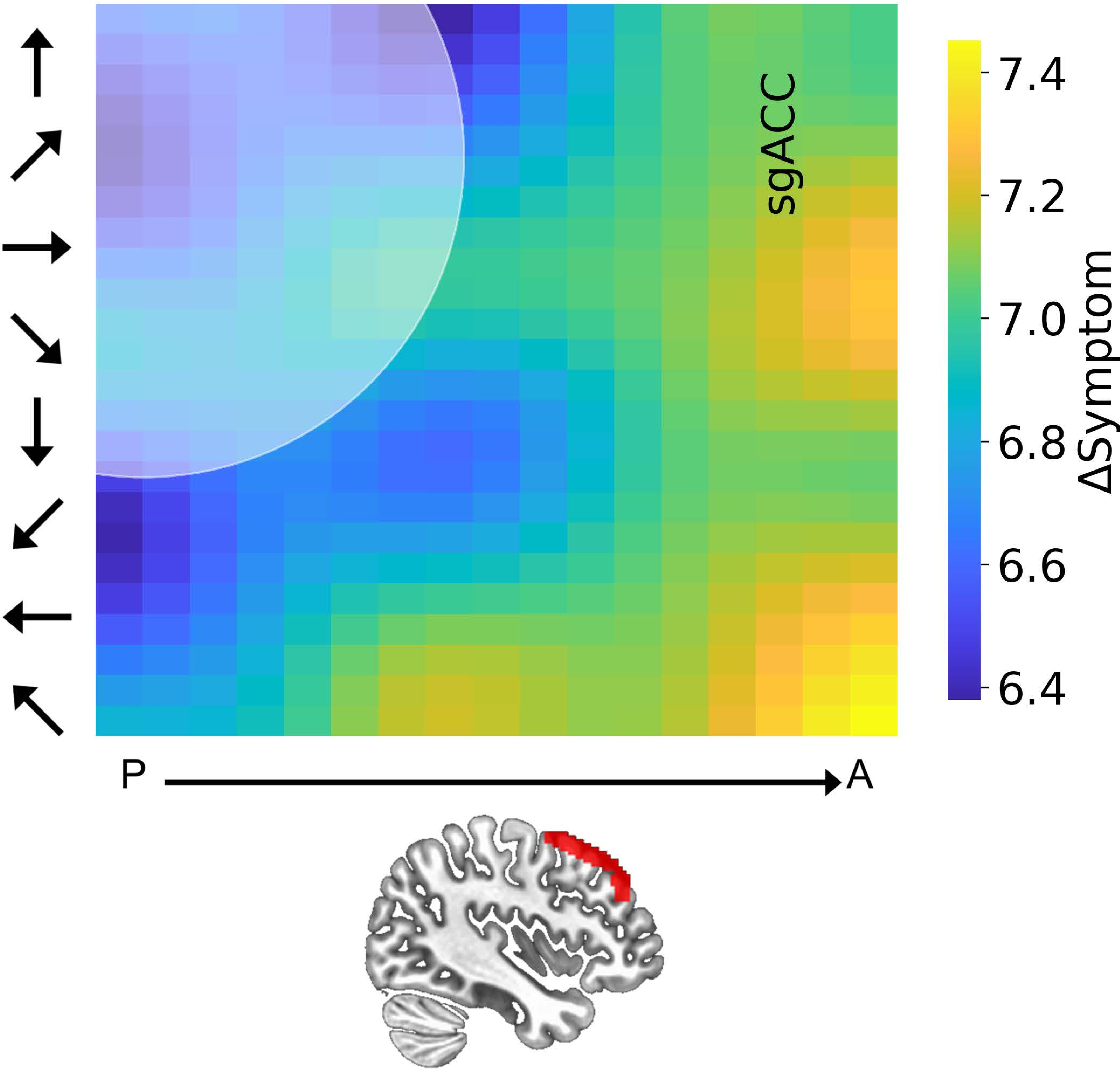

NDARUM818JC1_madrs_left_dlpfc

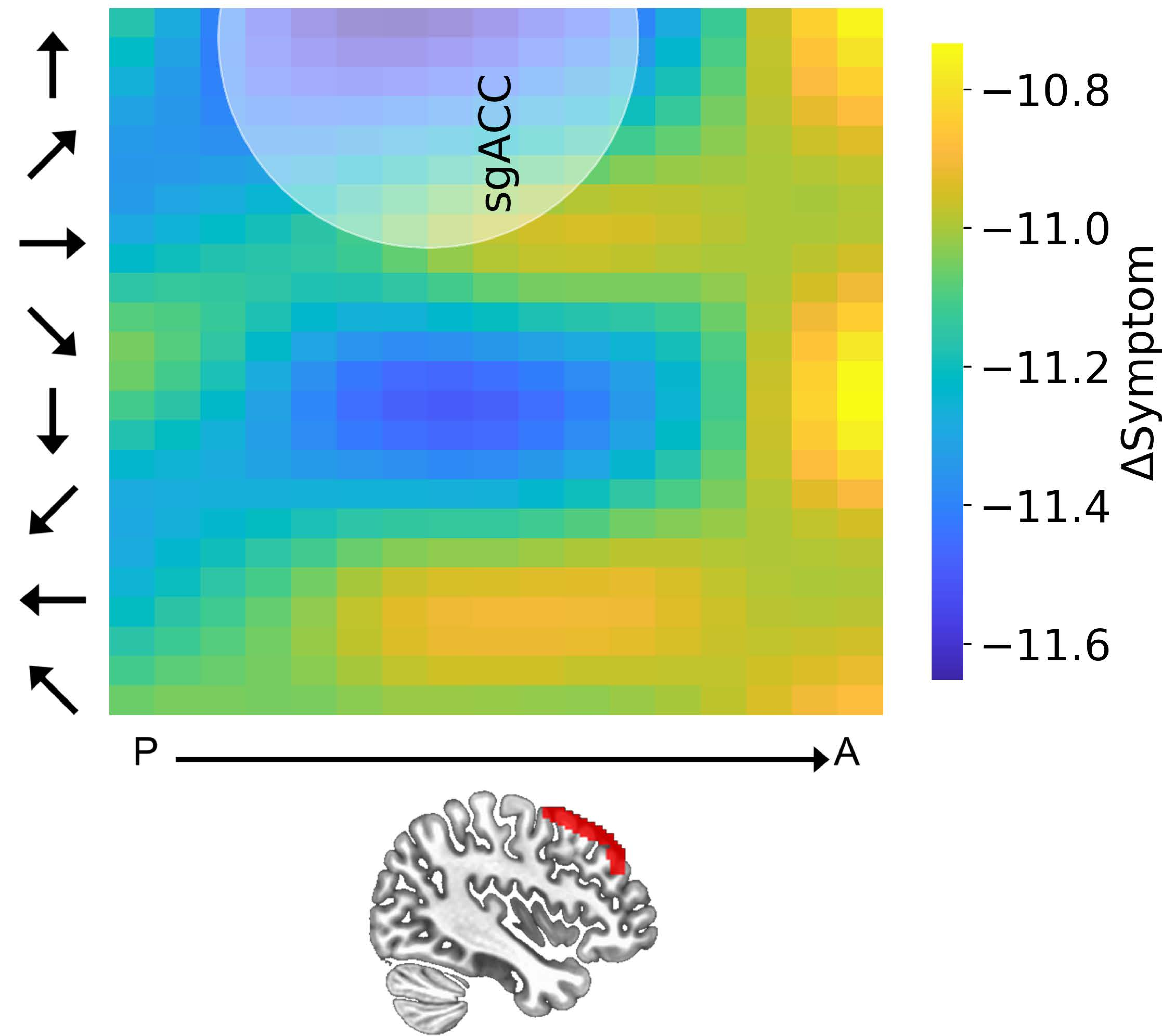

NDARUT852KUX_madrs_left_dlpfc

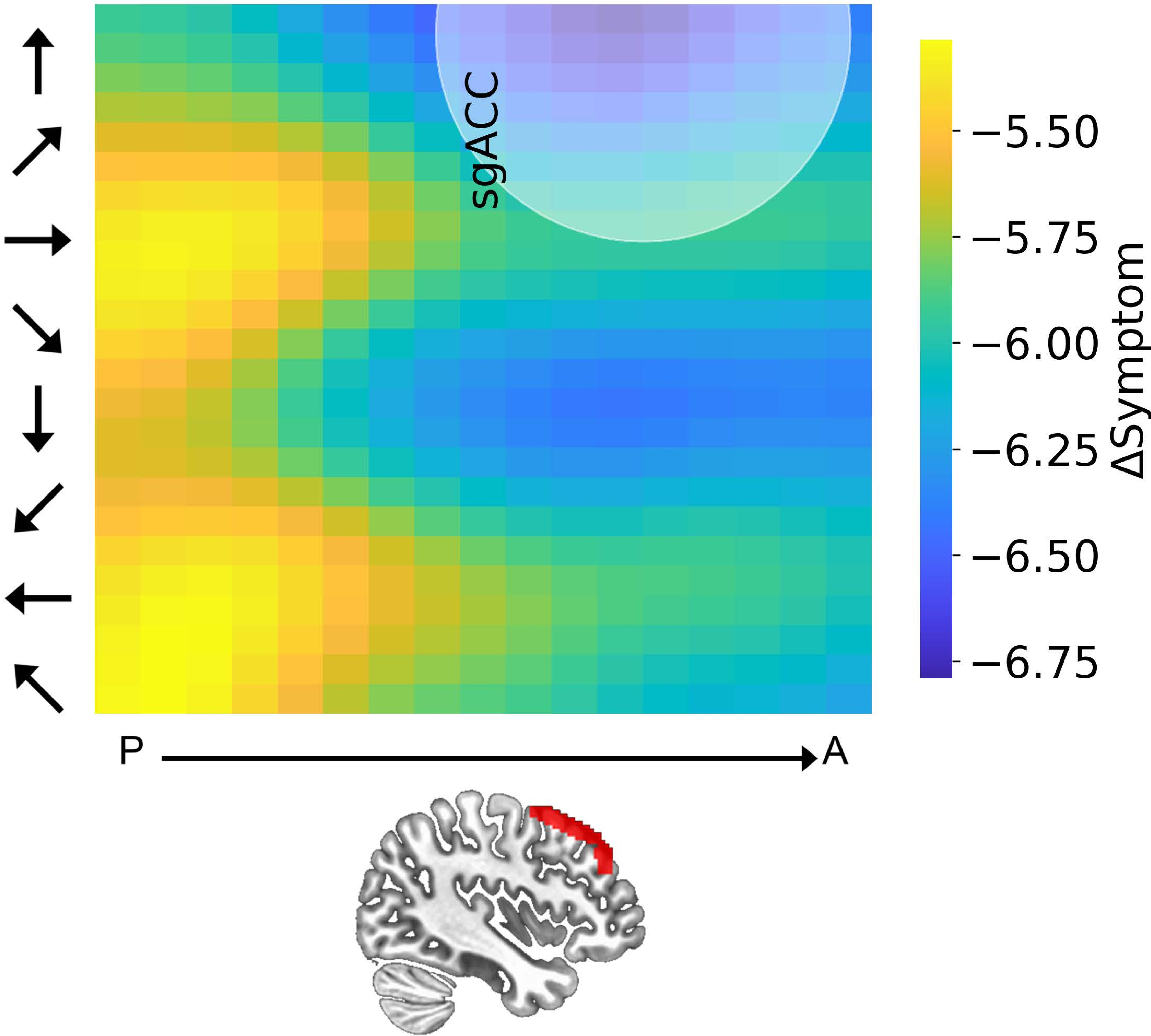

NDARVE642FAN_madrs_left_dlpfc

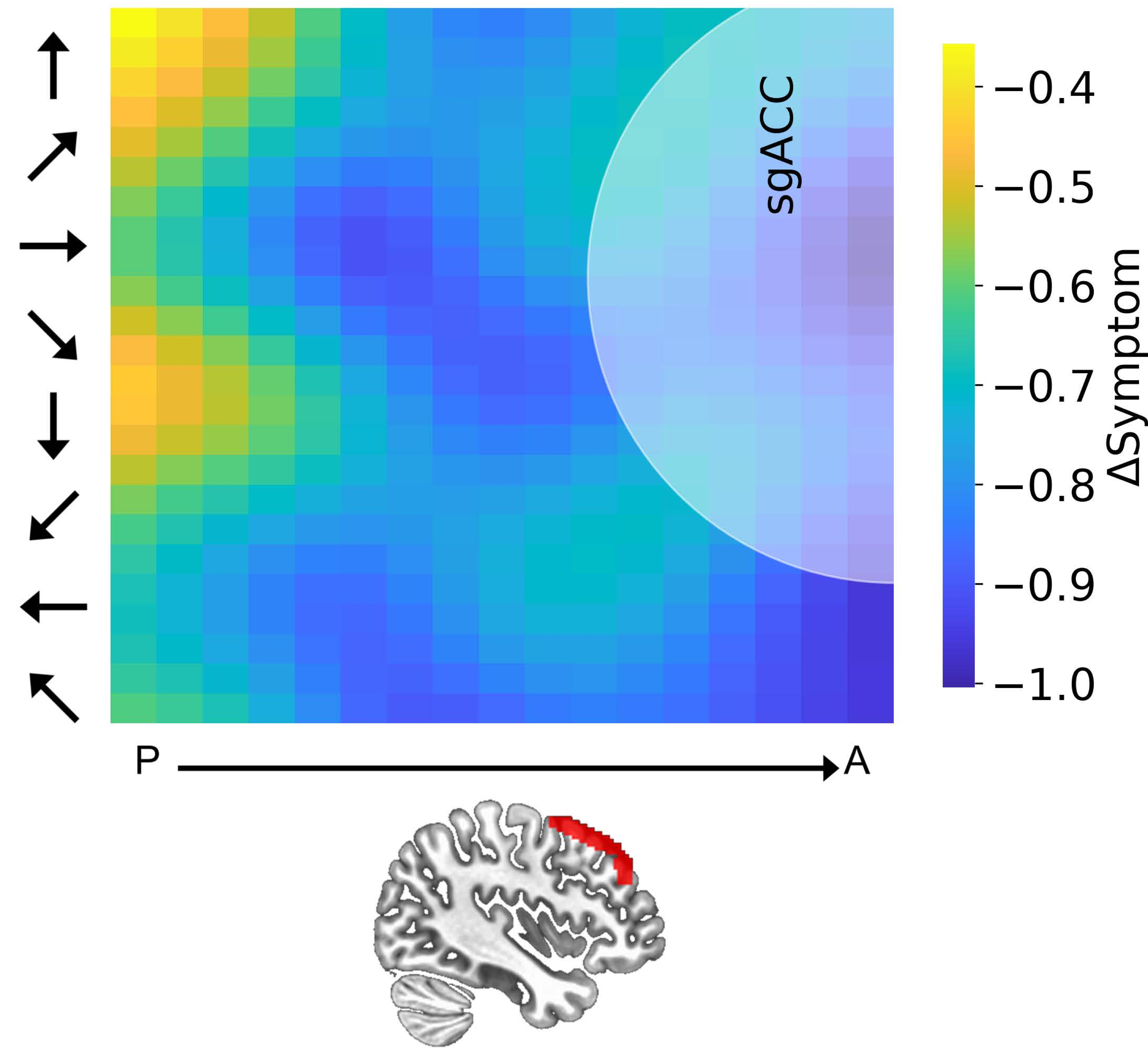

NDARVL144YVF_madrs_left_dlpfc

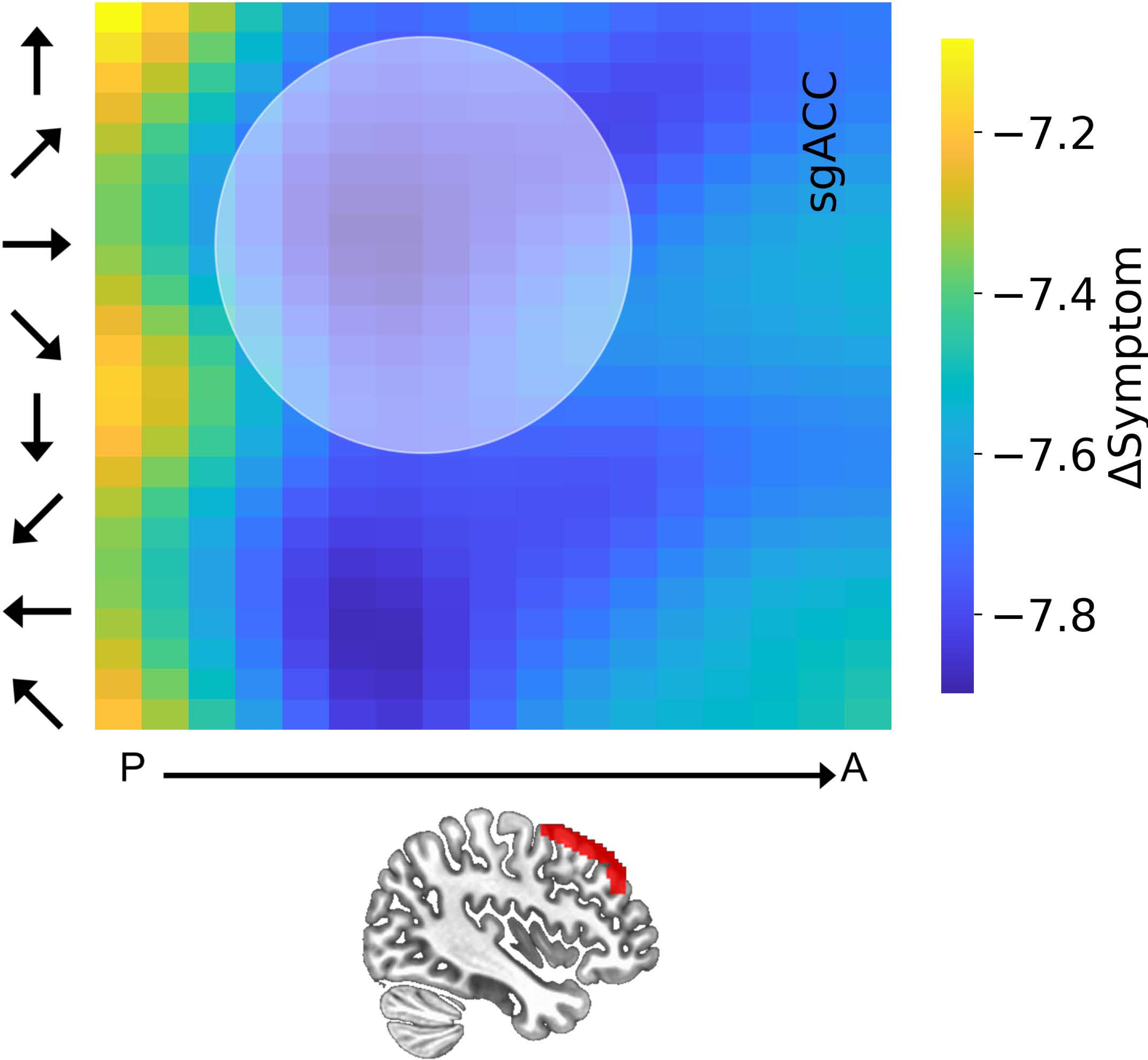

NDARWH523DTV_madrs_left_dlpfc

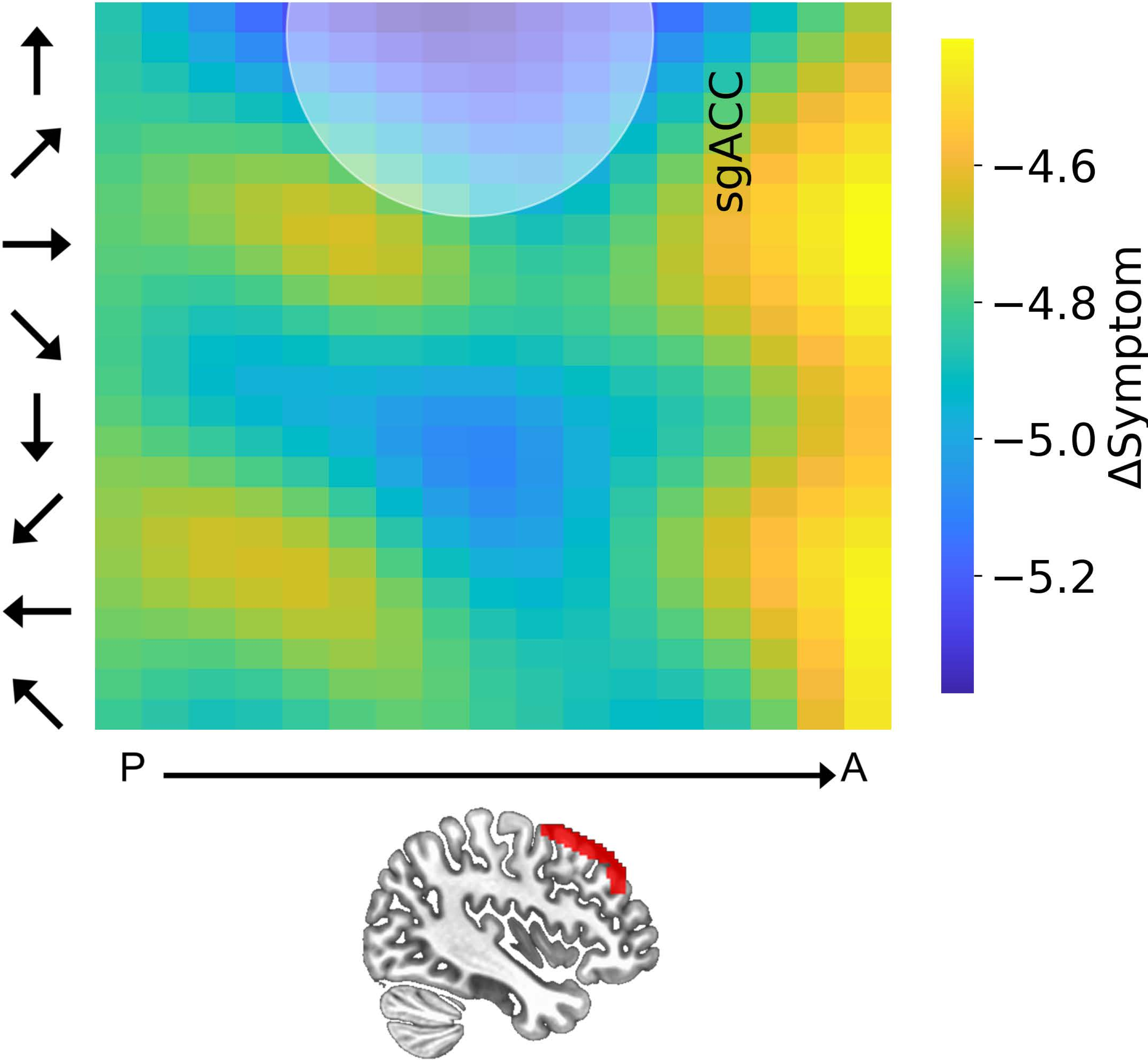

NDARWJ818VXA_madrs_left_dlpfc

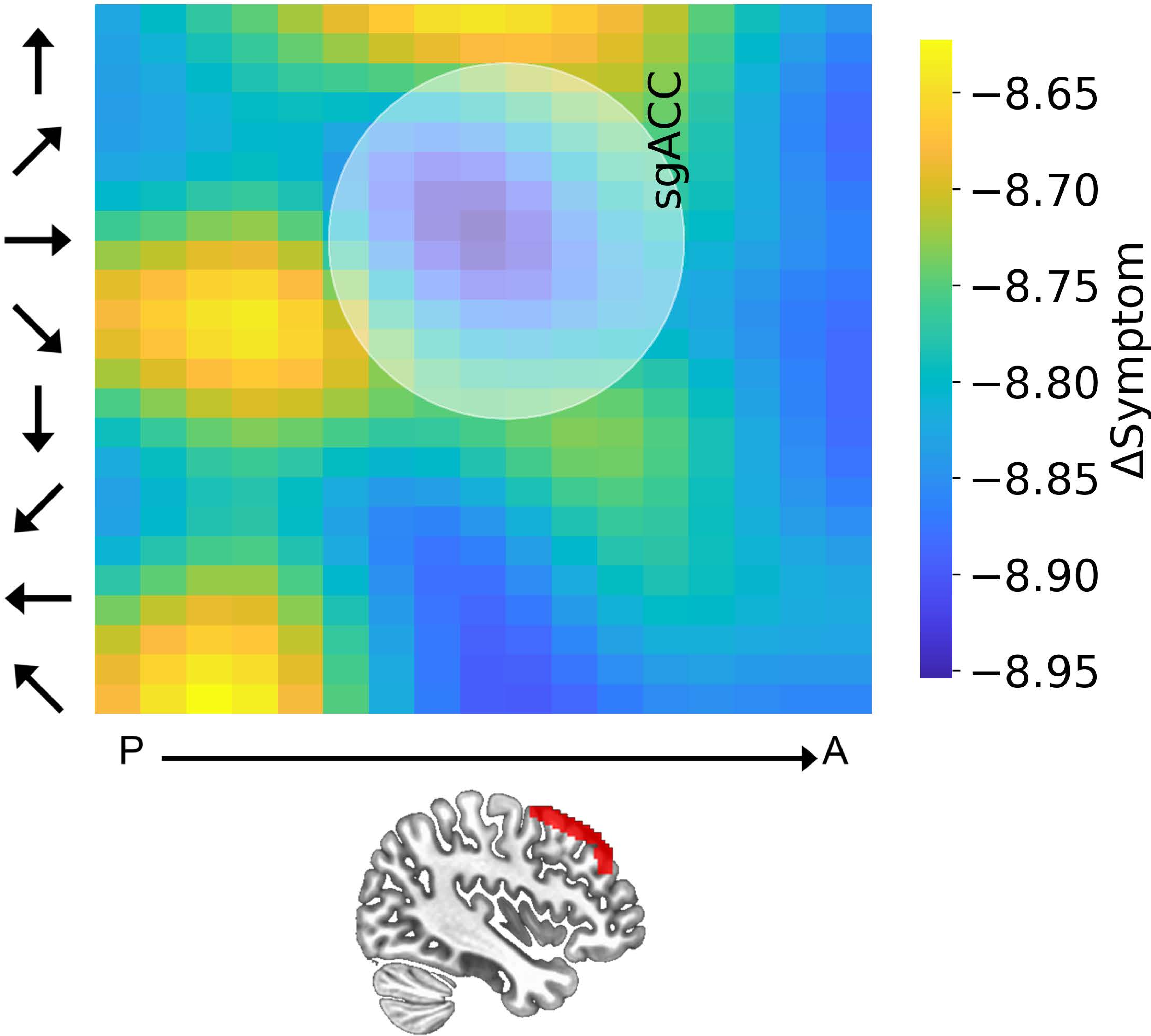

NDARWK081NTV_madrs_left_dlpfc

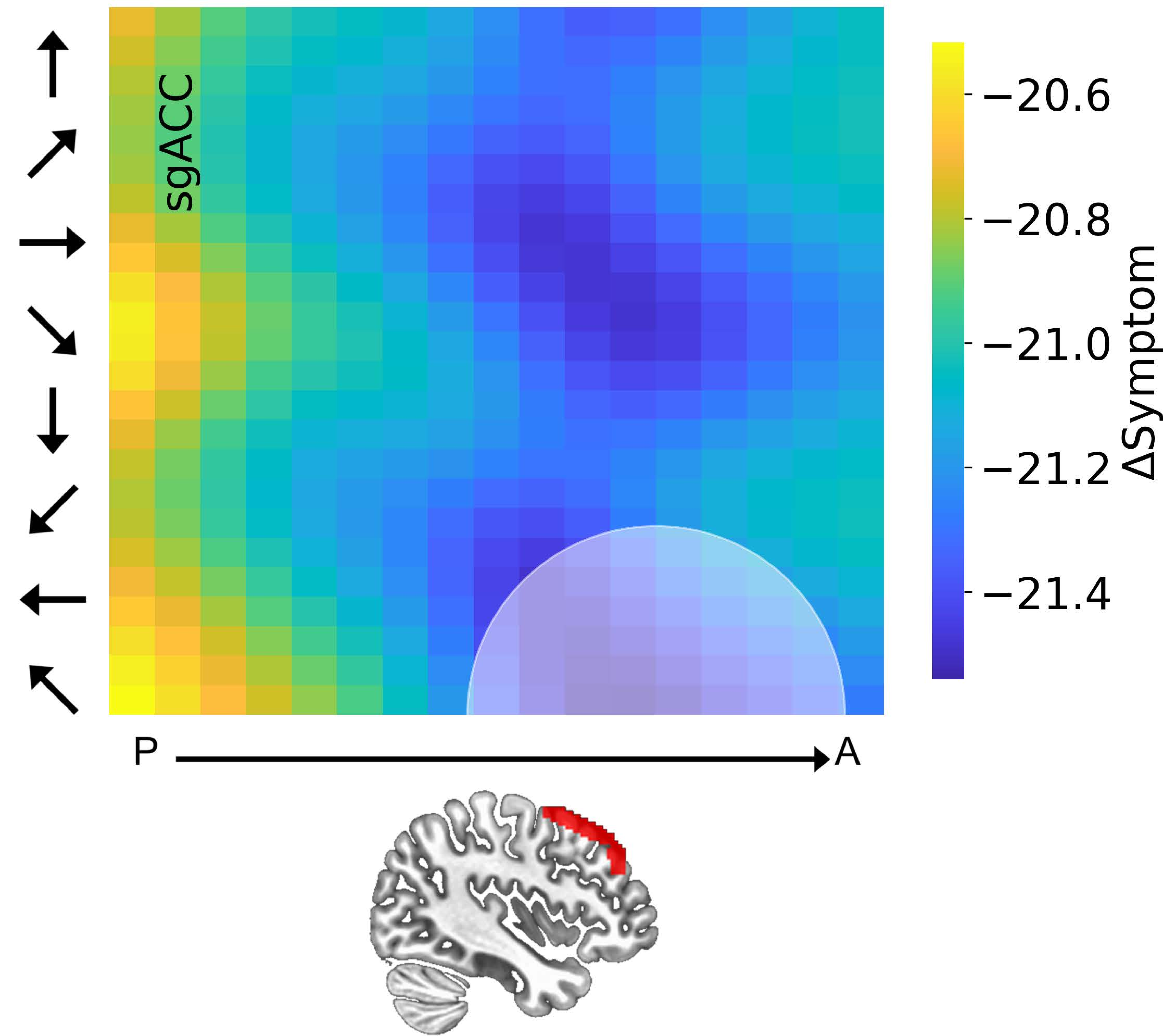

NDARWL277NXF_madrs_left_dlpfc

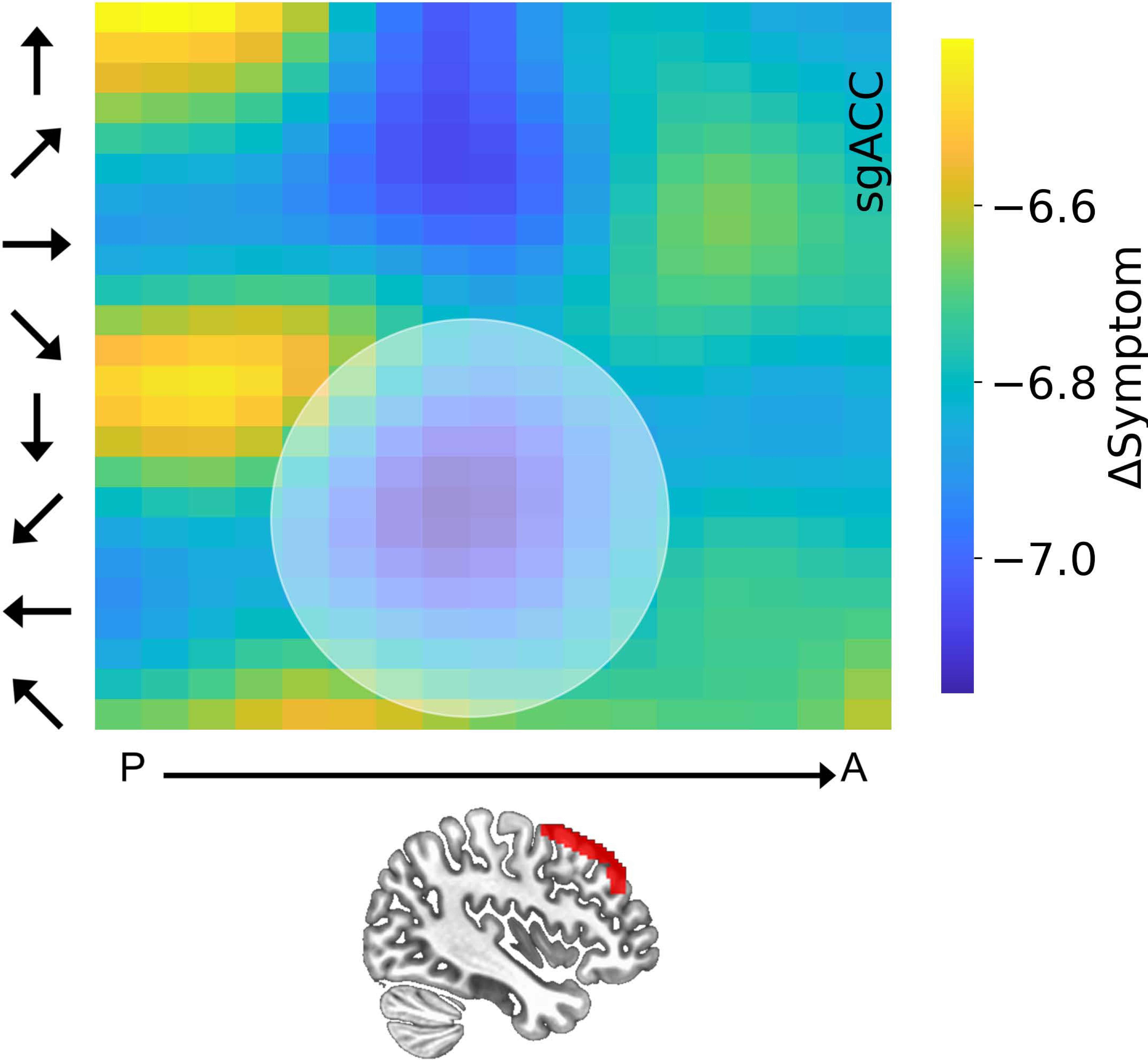

NDARWV016ZAT_madrs_left_dlpfc

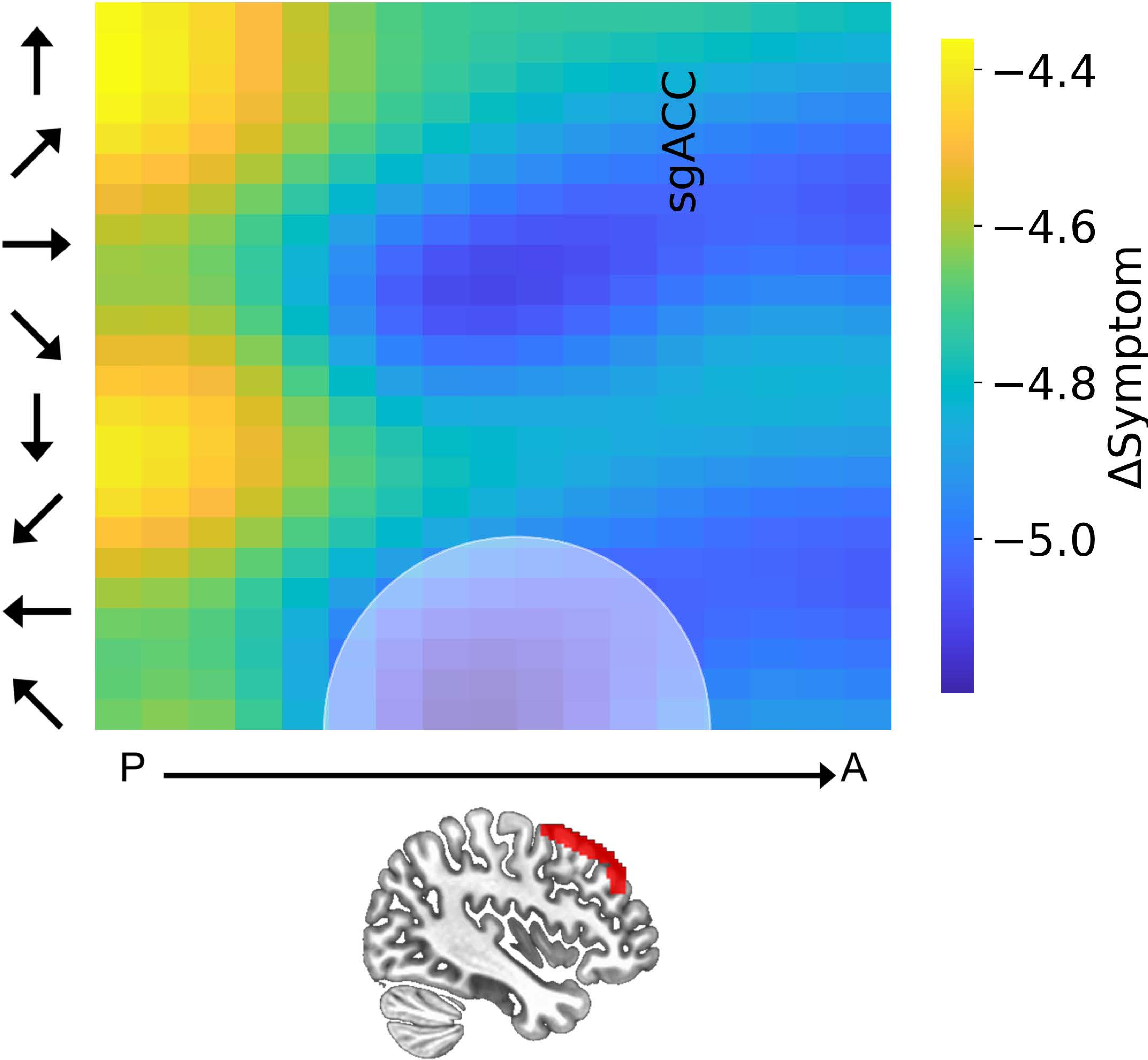

NDARXA148KPL_madrs_left_dlpfc

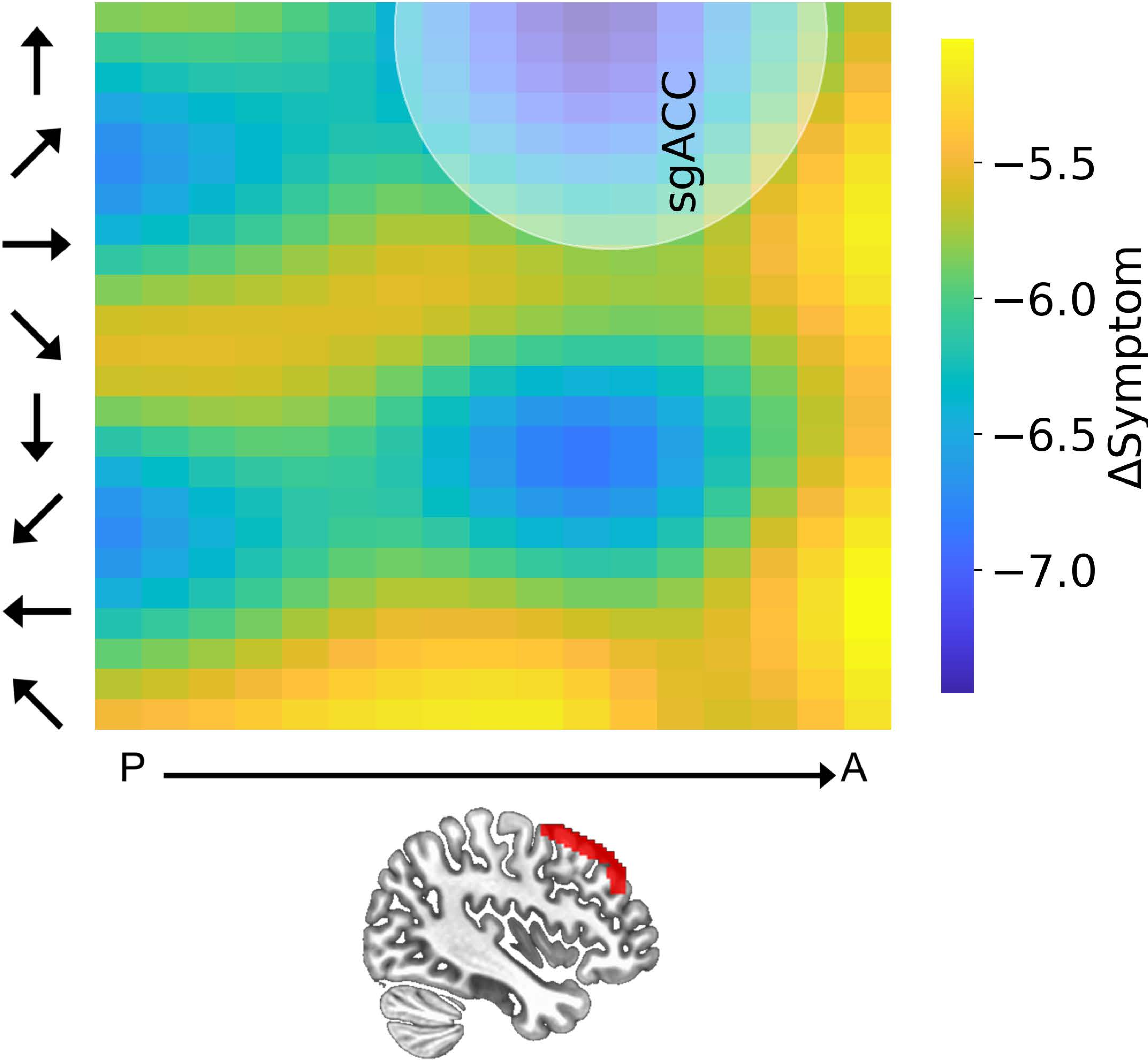

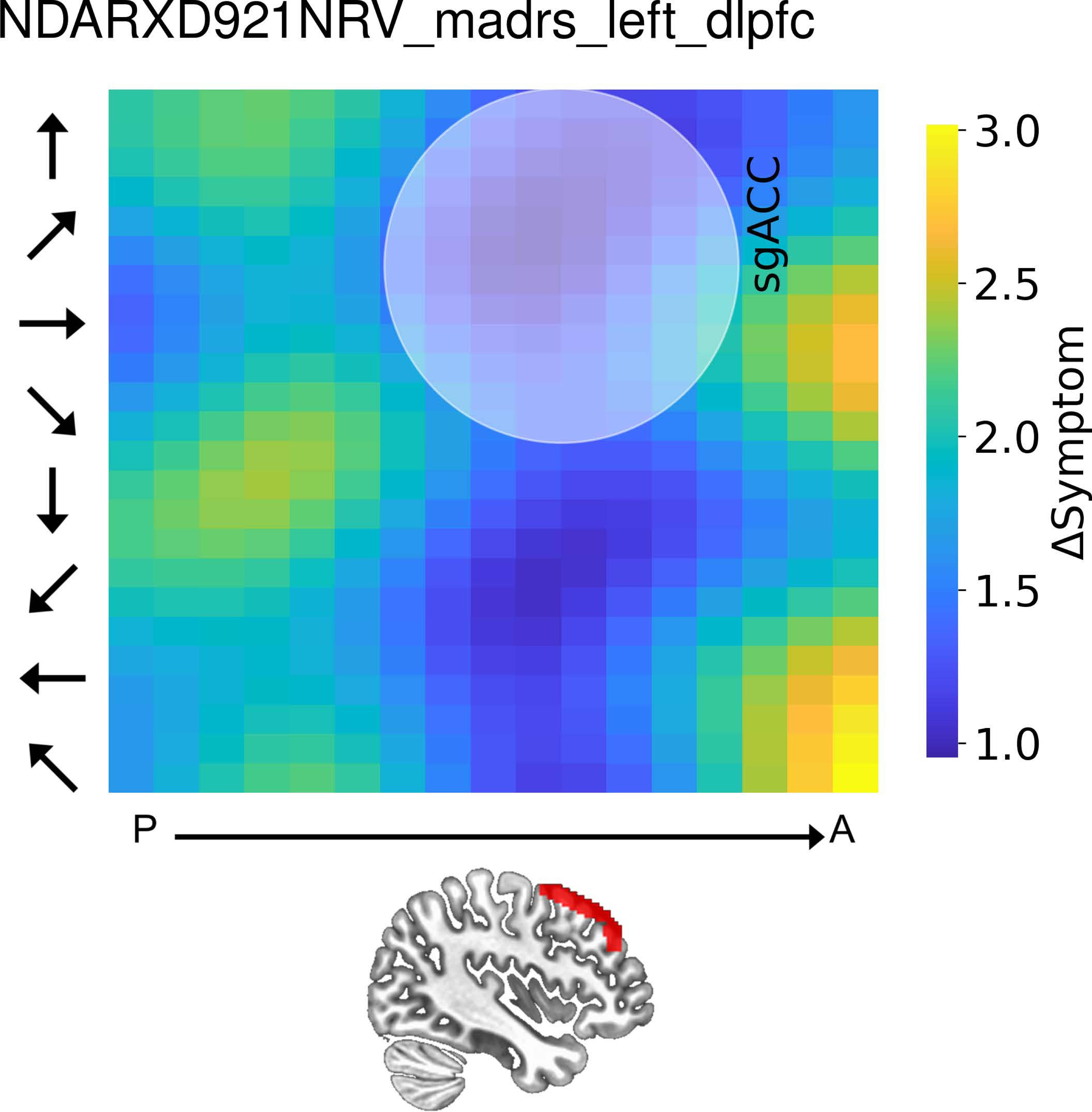

NDARXD921NRV_madrs_left_dlpfc
sgACC
3.0
2.5
2.0
1.5
1.0
ΔSymptom
P
A

NDARXU500EFT_madrs_left_dlpfc

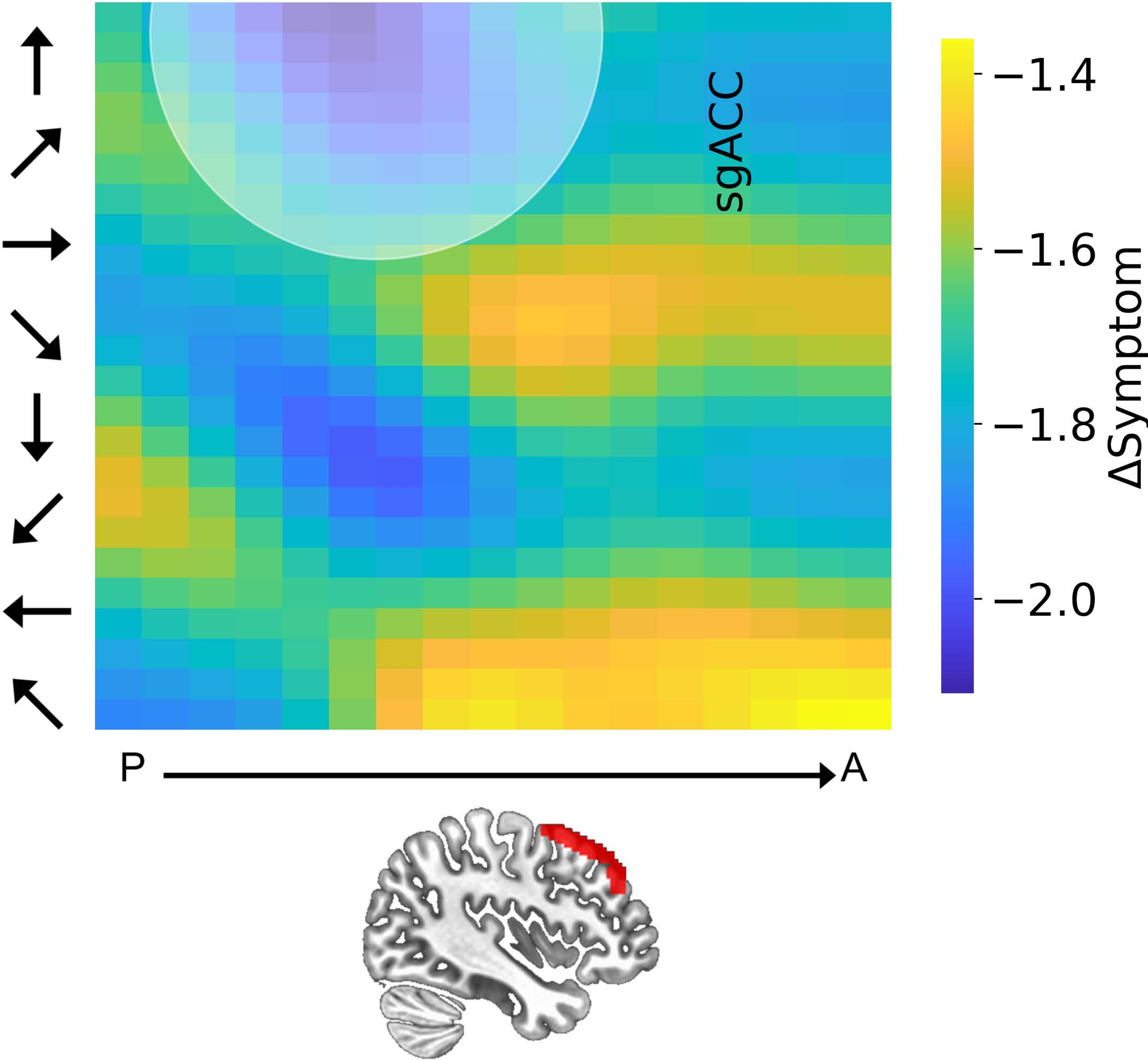

NDARYB181GTB_madrs_left_dlpfc

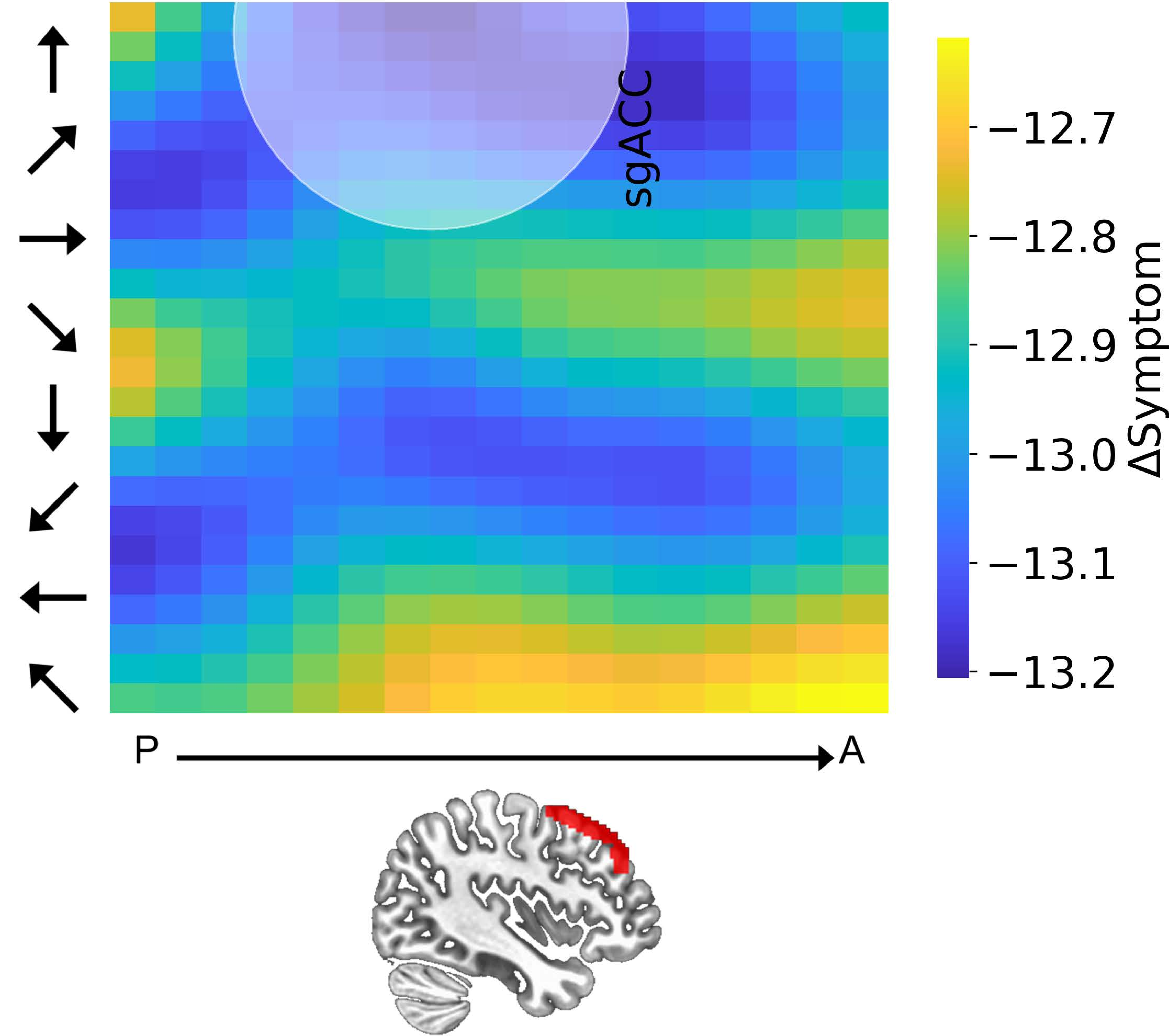

NDARYD169AYQ_madrs_left_dlpfc

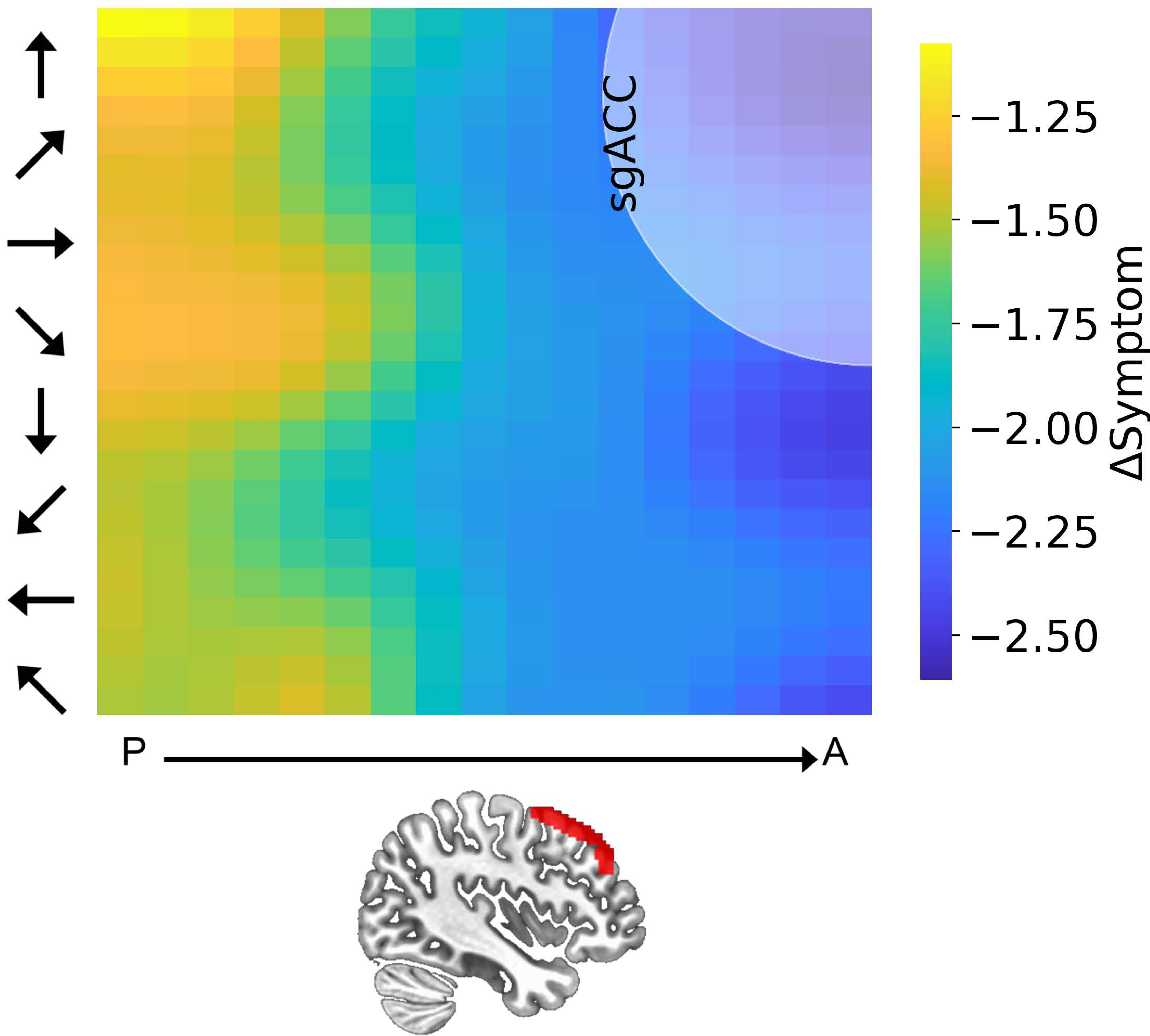

NDARYE405JJL_madrs_left_dlpfc

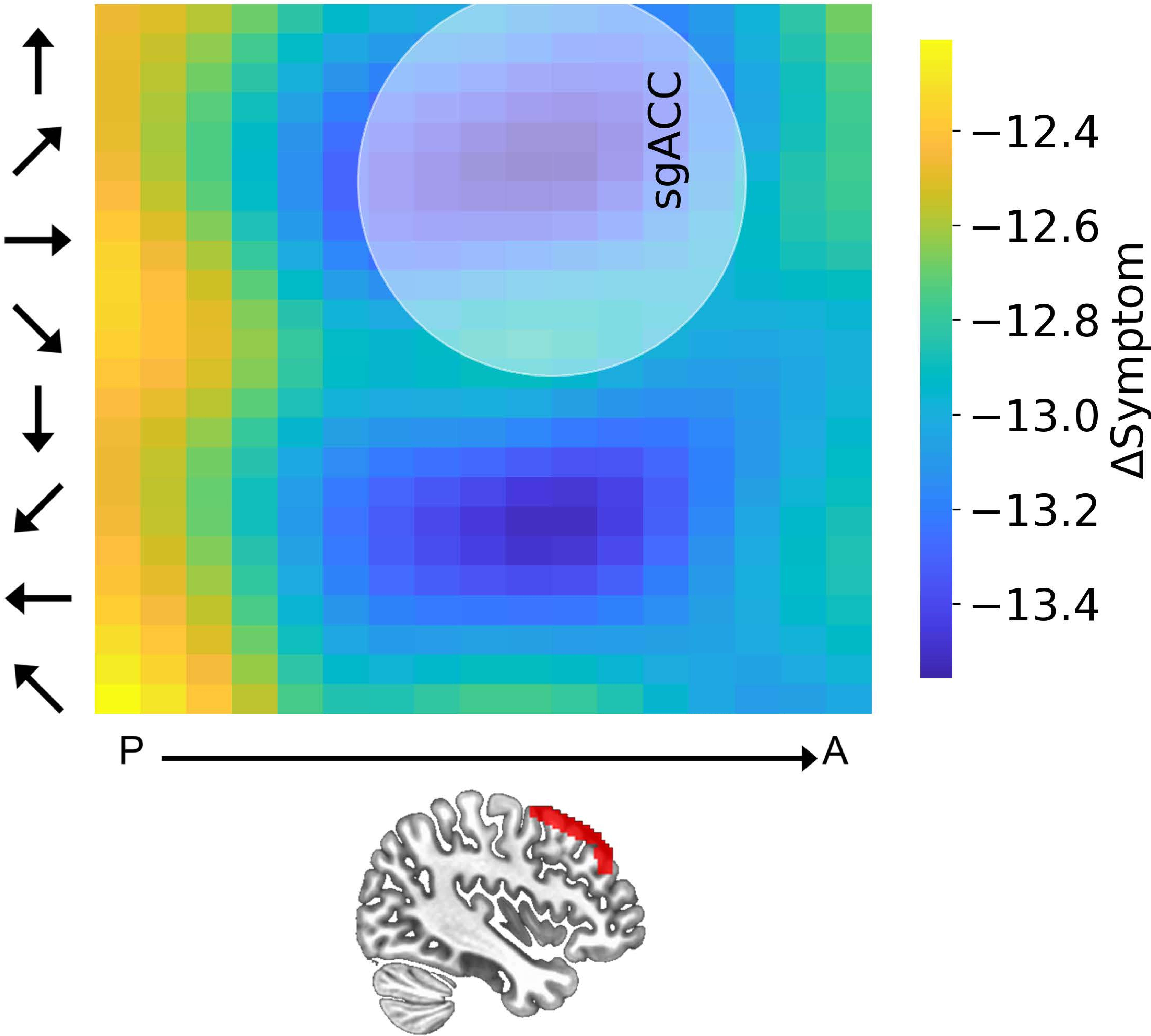

NDARYM648GK3_madrs_left_dlpfc

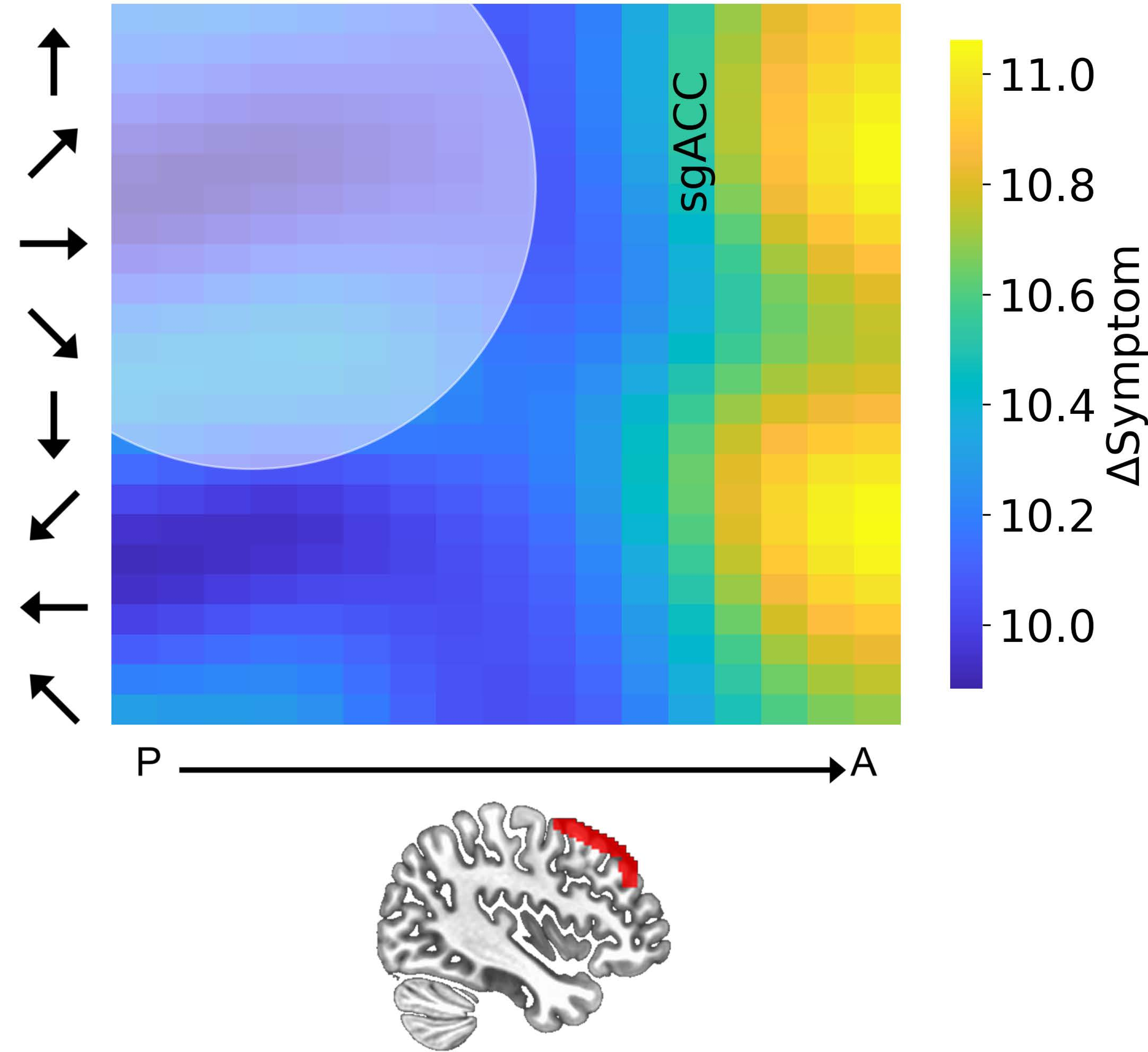

NDARZH200GZU_madrs_left_dlpfc

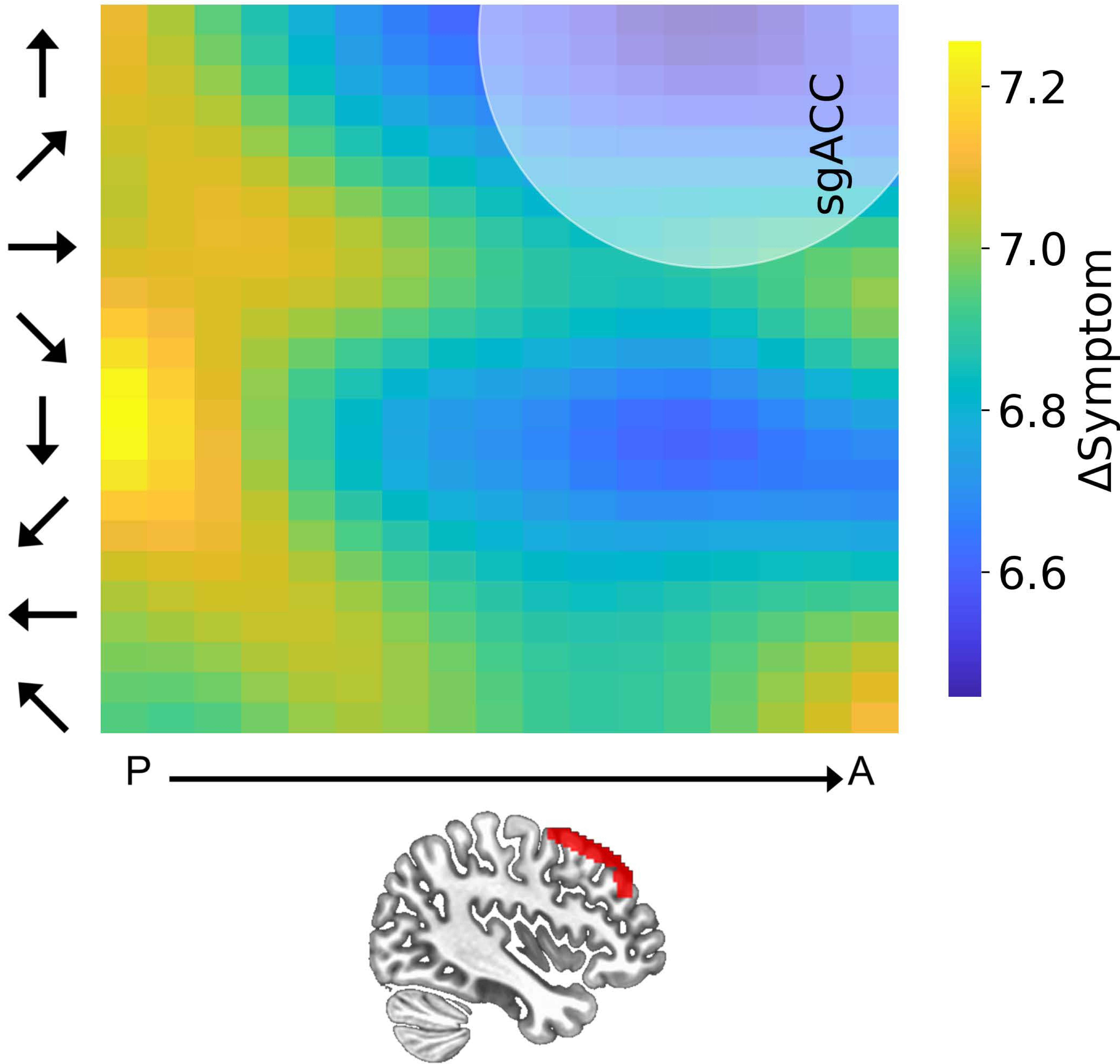

NDARBB471NAU_madrs_right_dlpfc

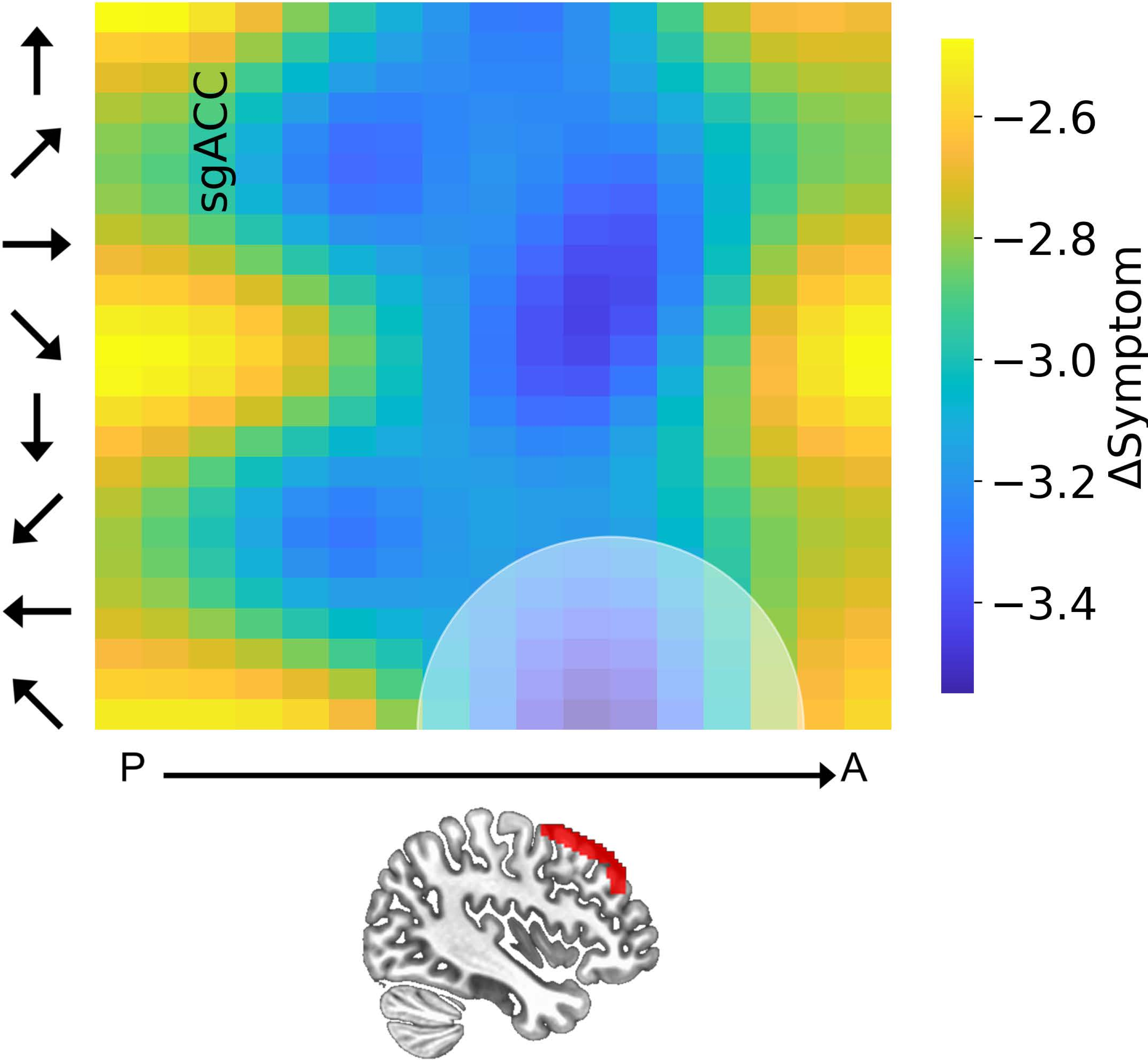

NDARBG497RE1_madrs_right_dlpfc

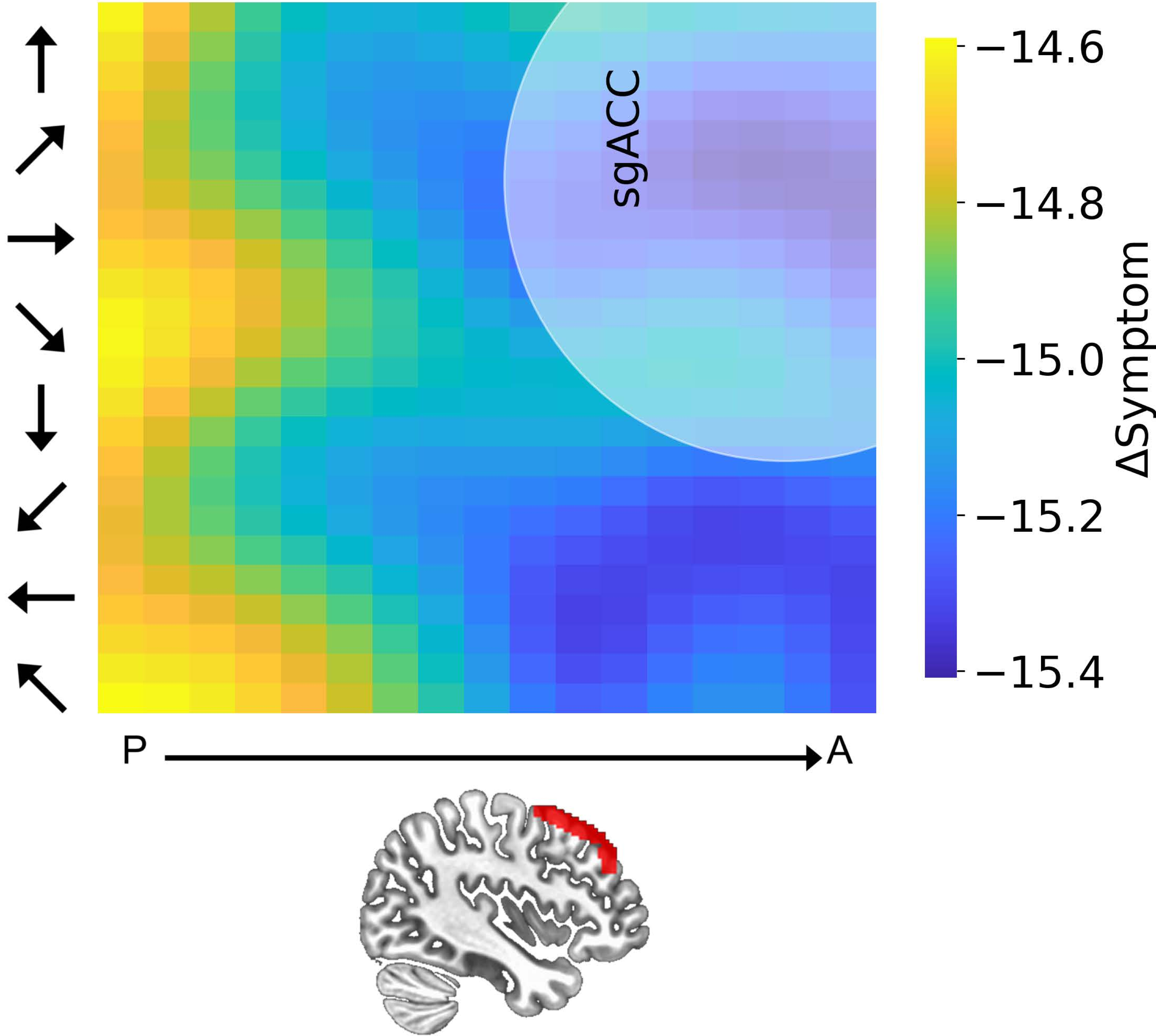

NDARBK027PDF_madrs_right_dlpfc

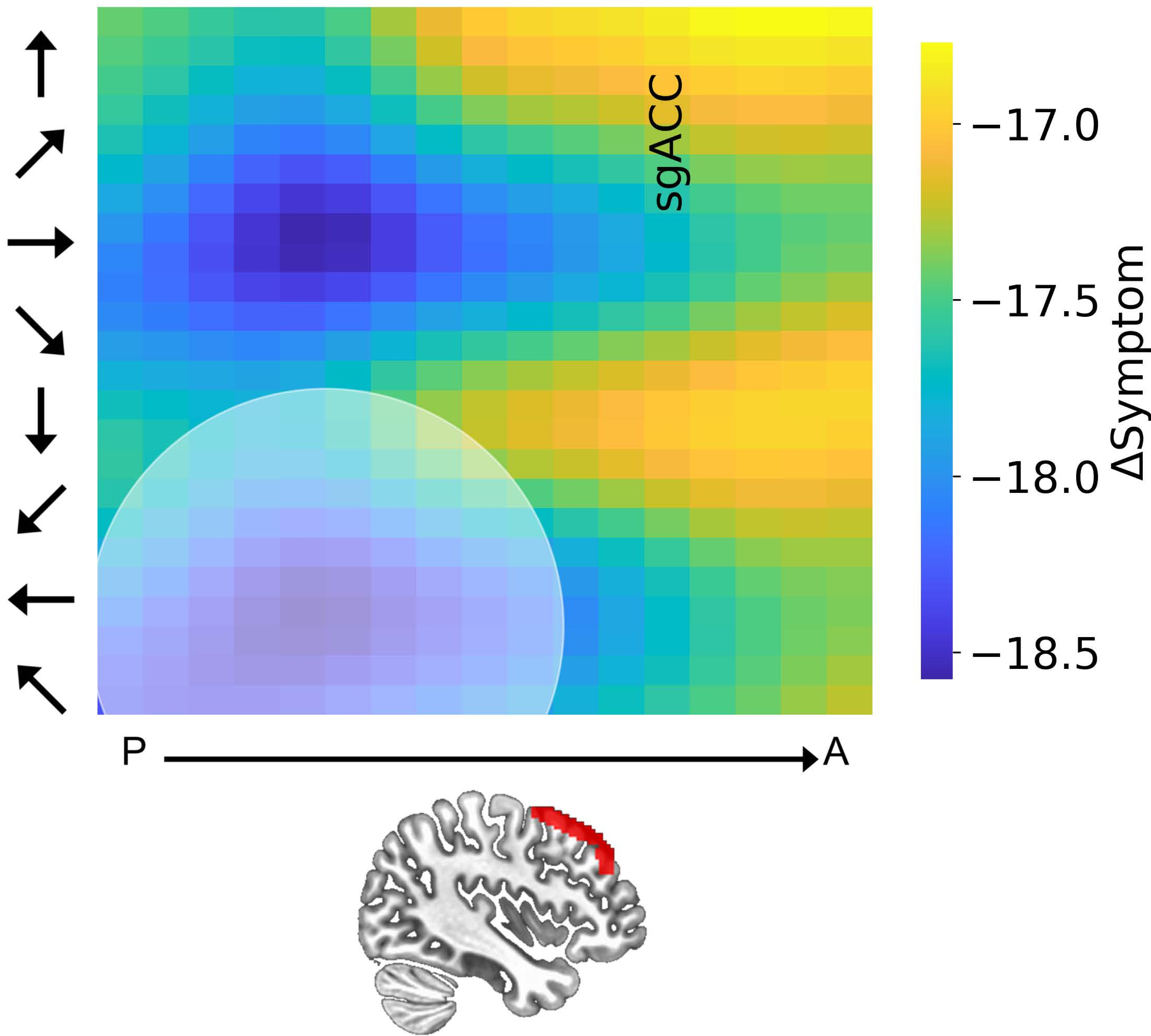

NDARBL814BJK_madrs_right_dlpfc

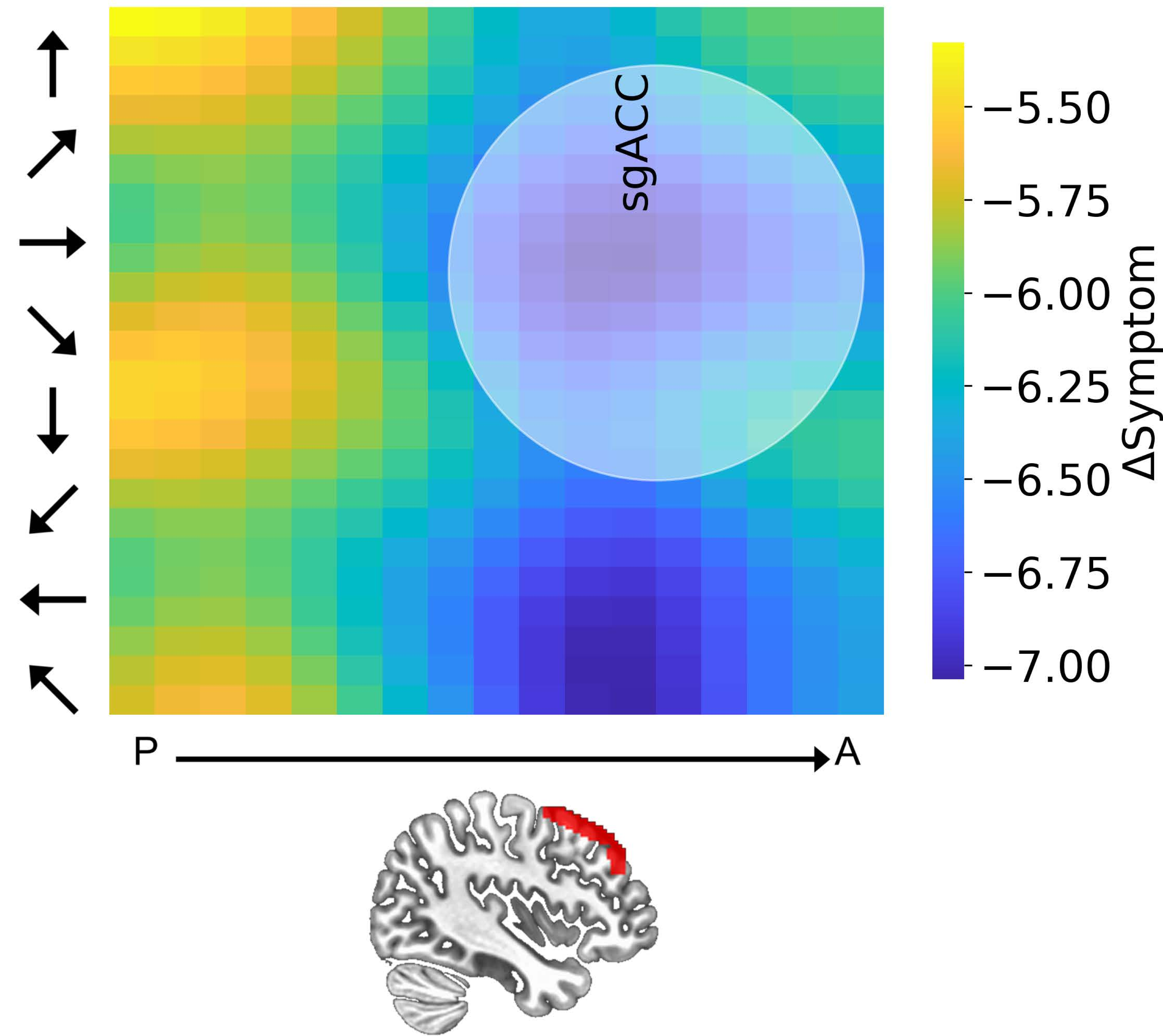

NDARBP353XD2_madrs_right_dlpfc

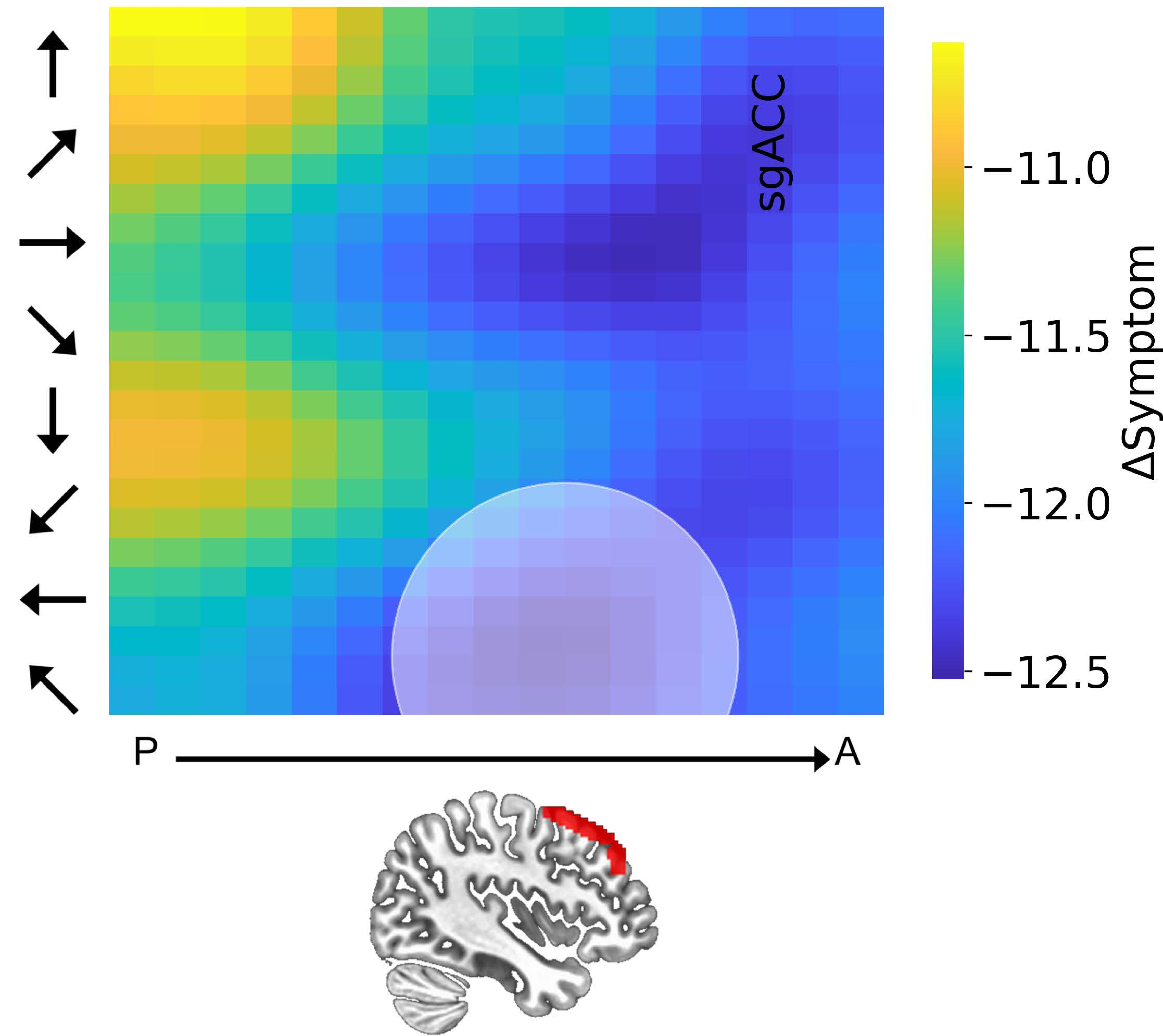

NDARBT583XH6_madrs_right_dlpfc

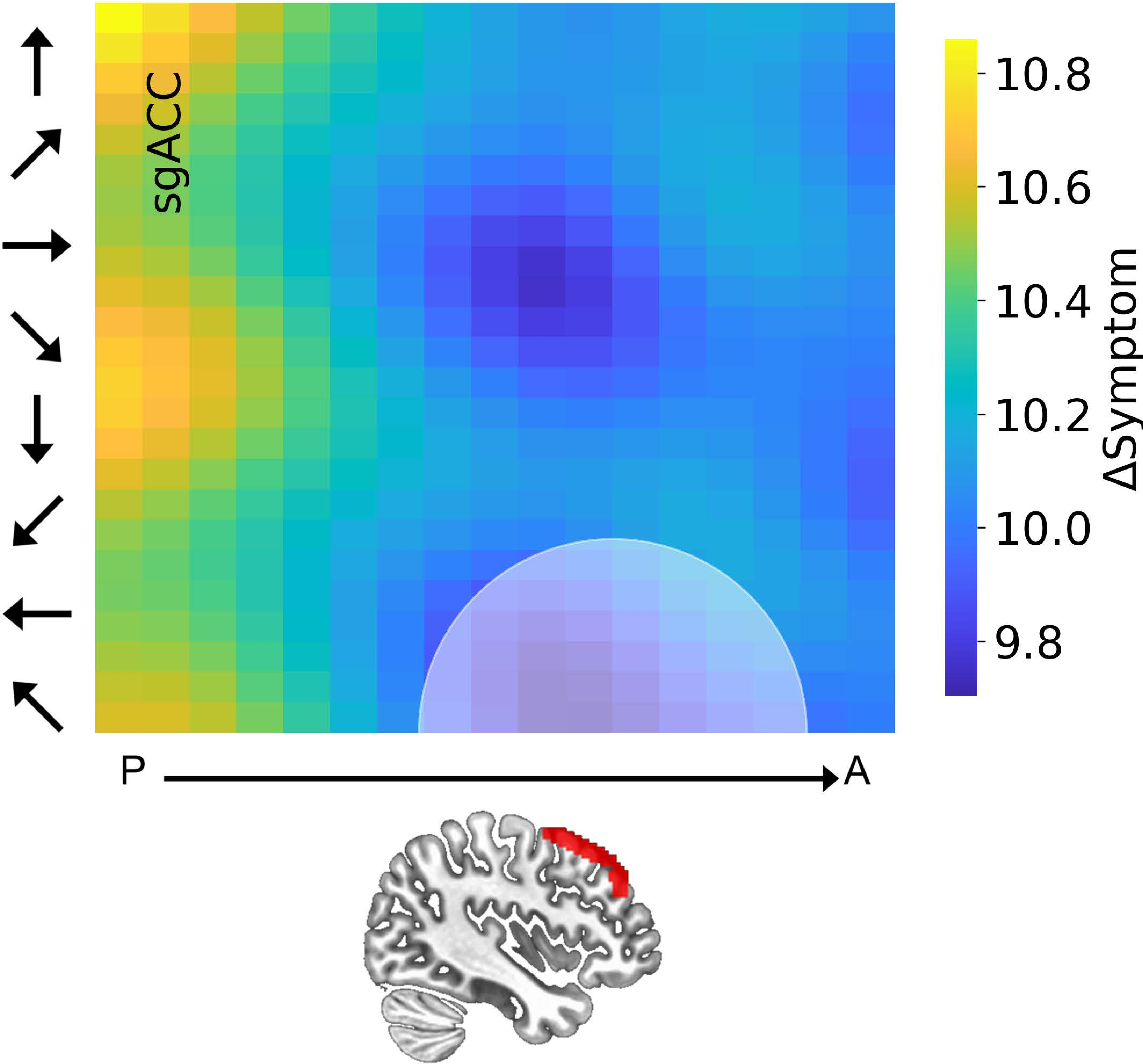

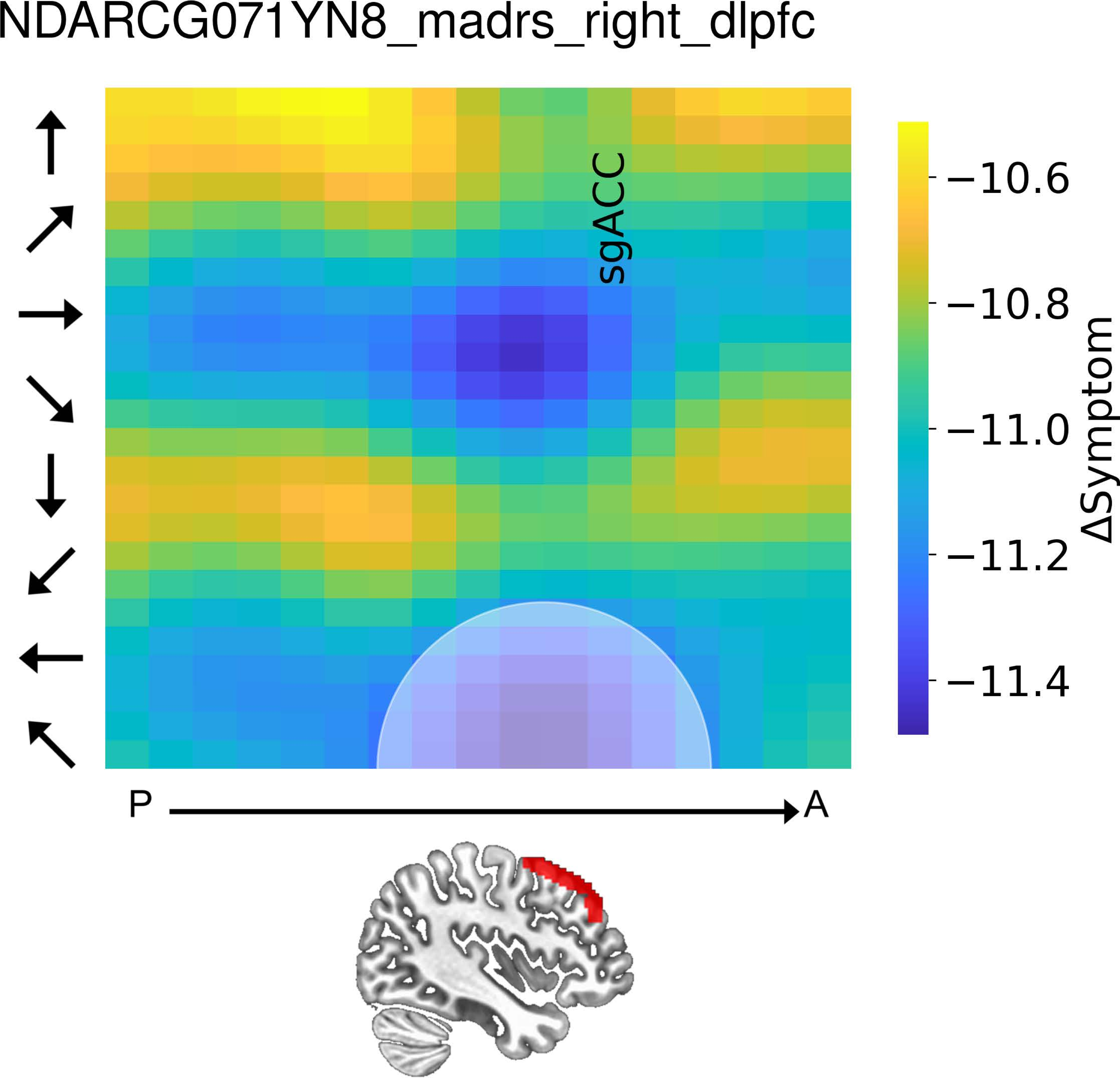
NDARCG071YN8_madrs_right_dlpfc
sgACC
P
A
−10.6
−10.8
−11.0
−11.2
−11.4
ΔSymptom

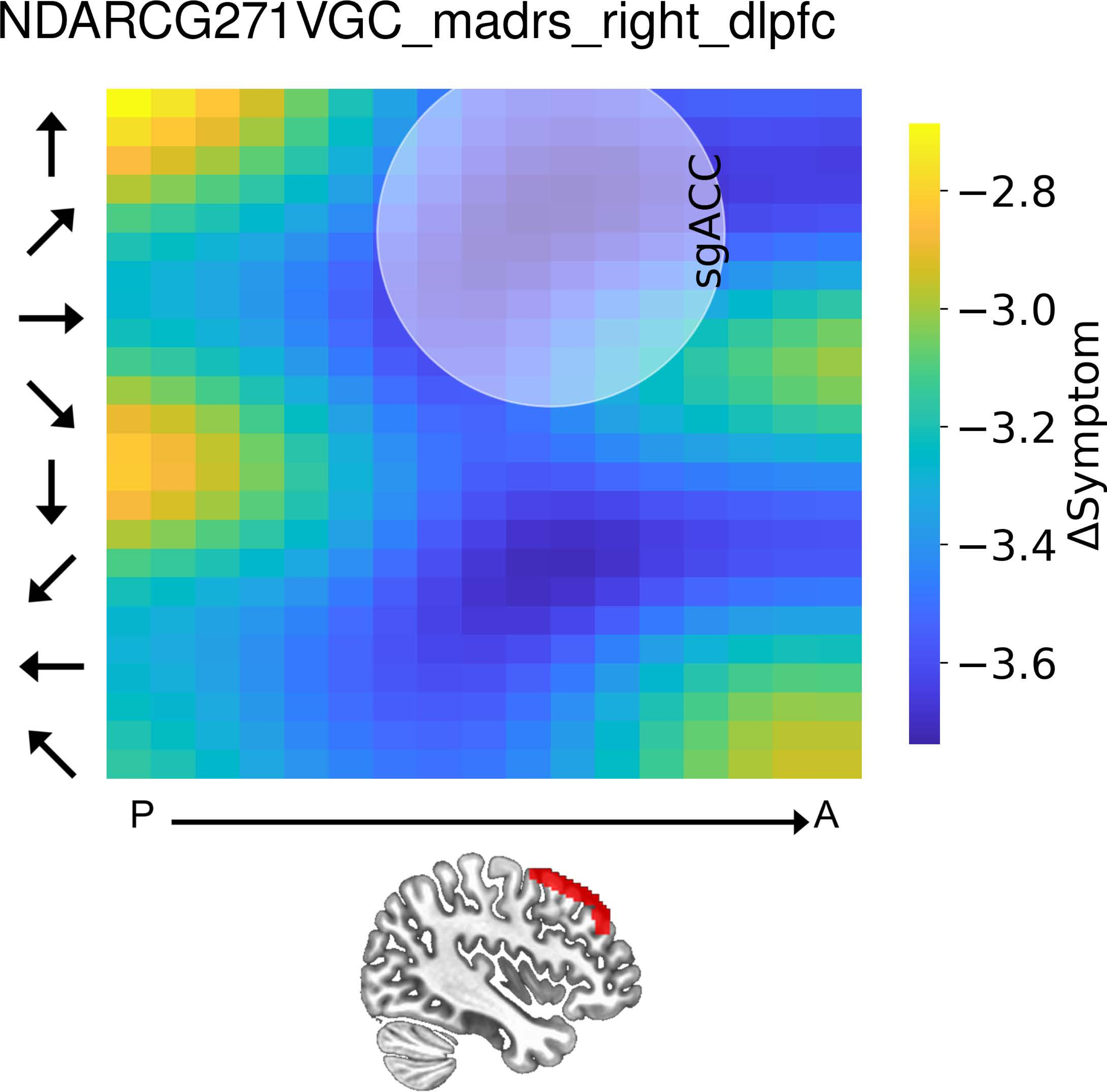
NDARCG271VGC_madrs_right_dlpfc
sgACC
−2.8
−3.0
−3.2
−3.4
−3.6
ΔSymptom
P
A

NDARCM769WZL_madrs_right_dlpfc

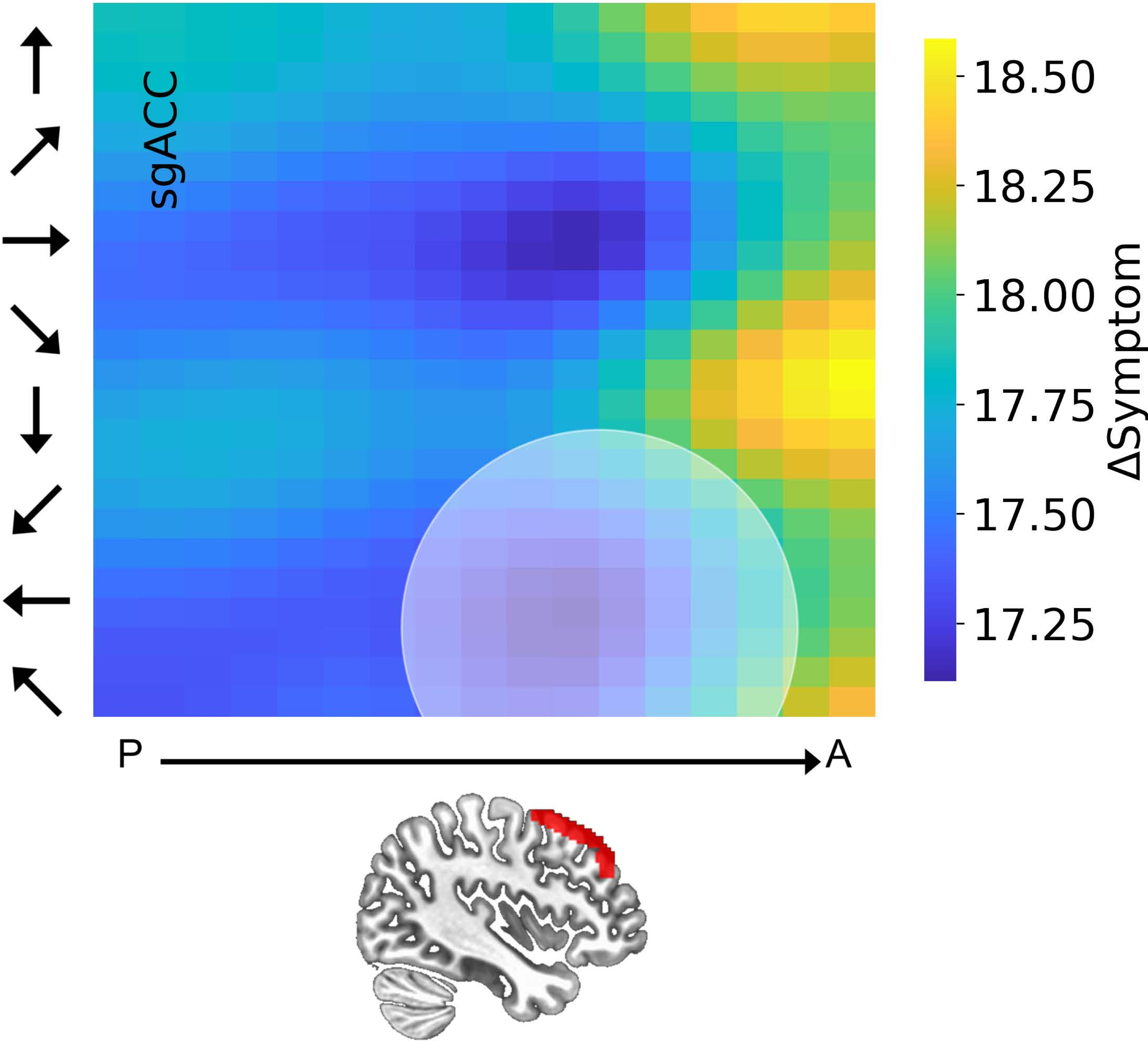

NDARDL291XT0_madrs_right_dlpfc

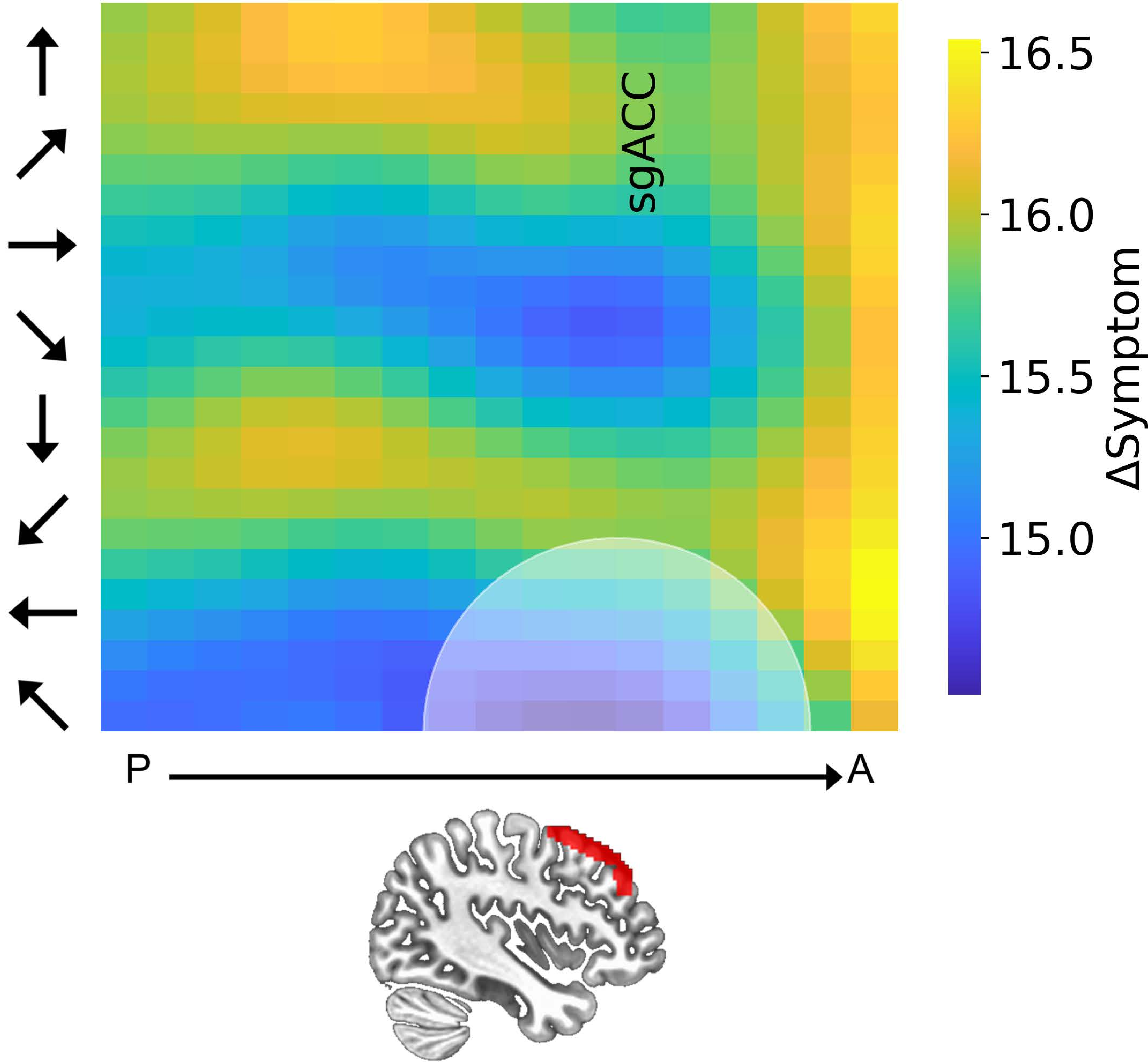

NDARDL667VMW_madrs_right_dlpfc

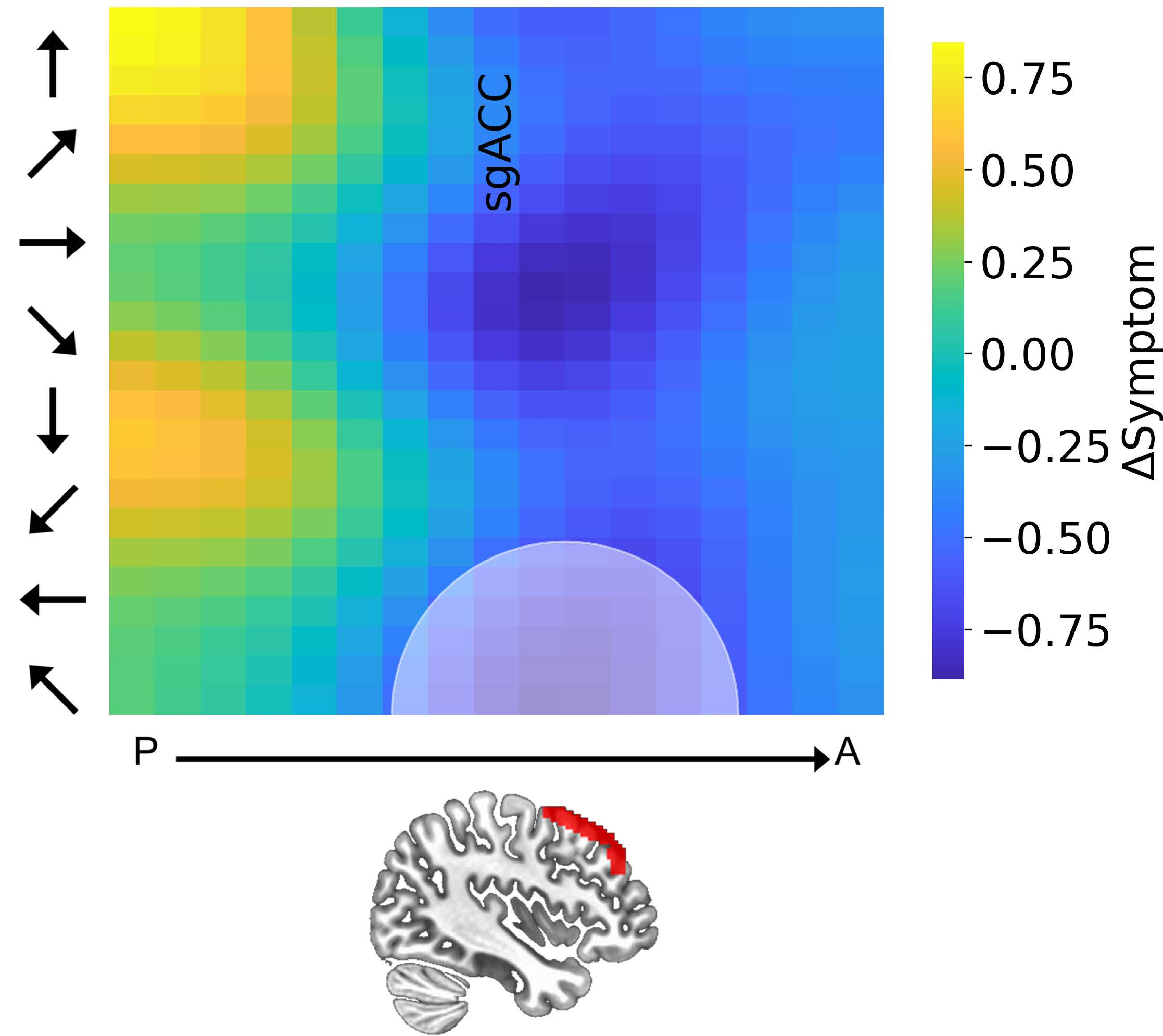

NDARDR017YX2_madrs_right_dlpfc

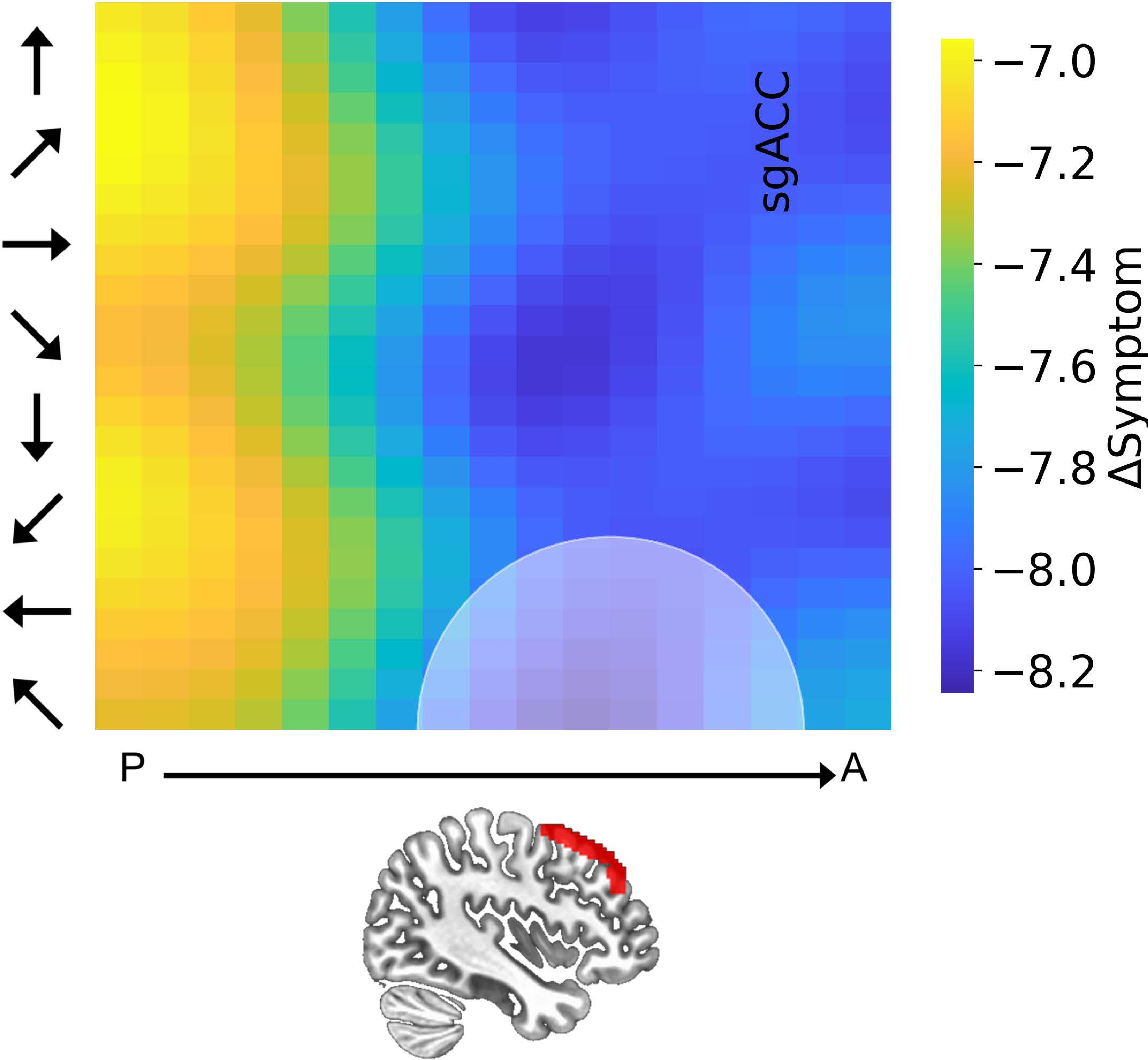

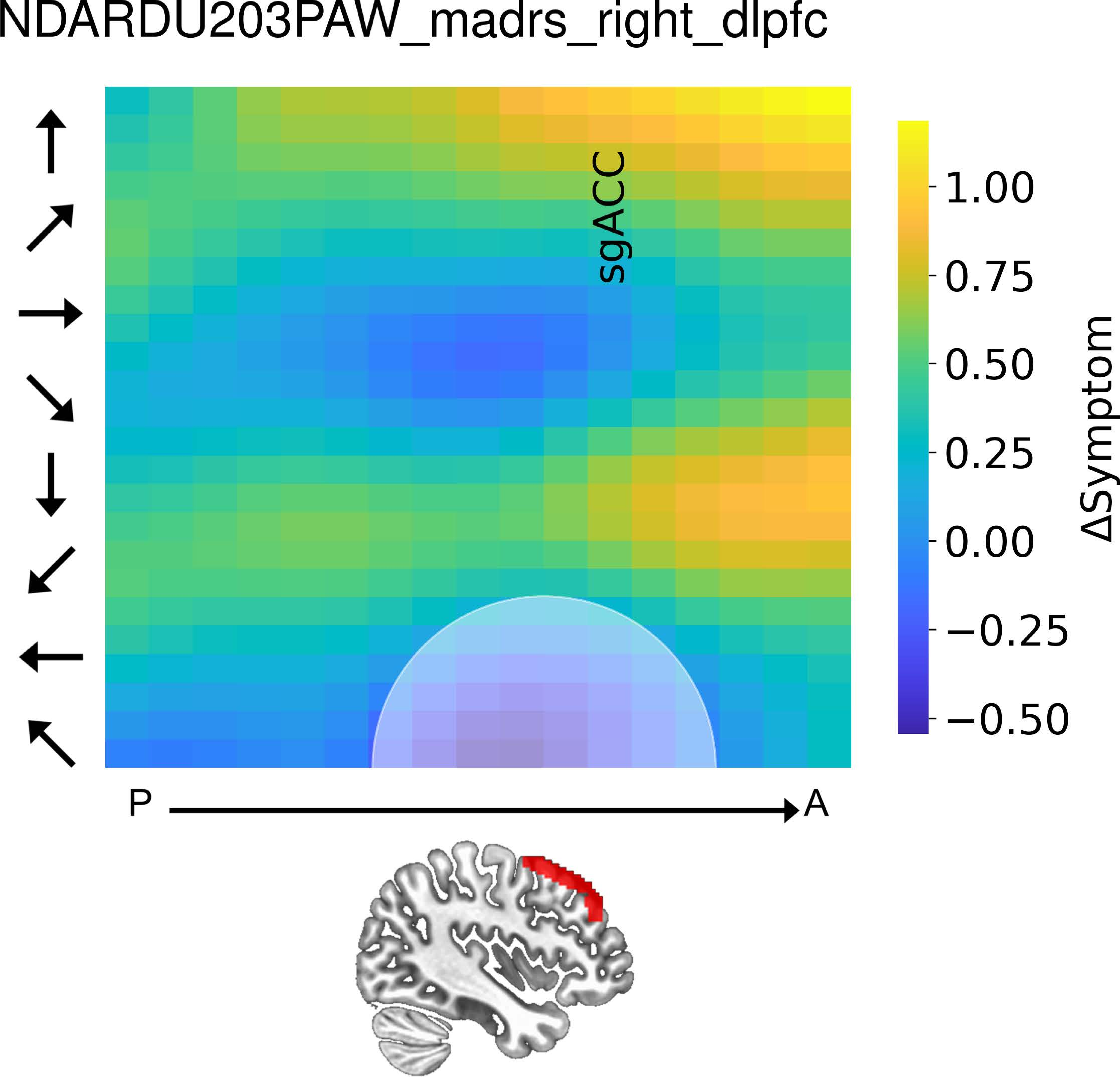

NDARDU203PAW_madrs_right_dlpfc
sgACC
1.00
0.75
0.50
0.25
0.00
−0.25
−0.50
ΔSymptom
P
A

NDARDU282NKX_madrs_right_dlpfc

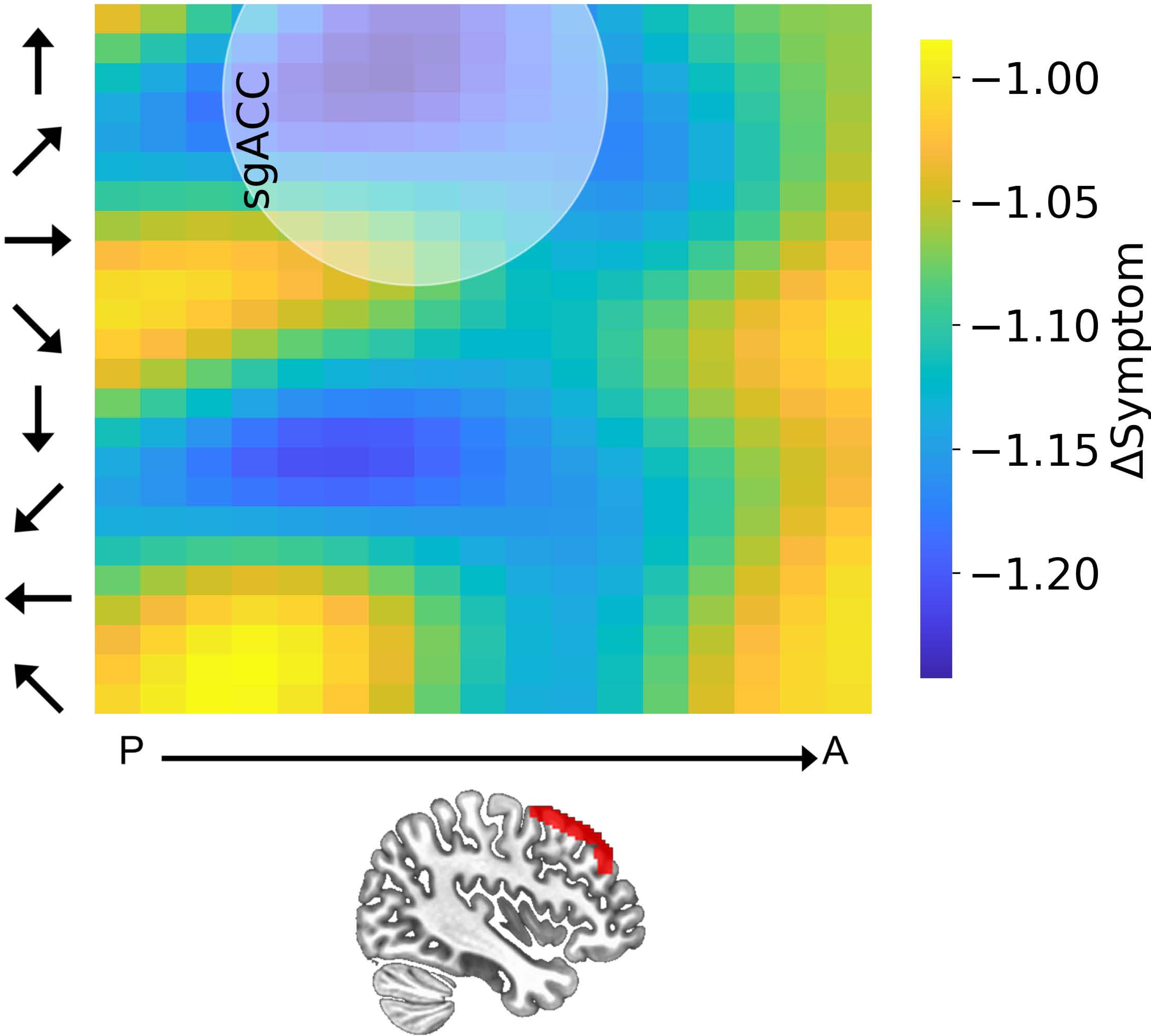

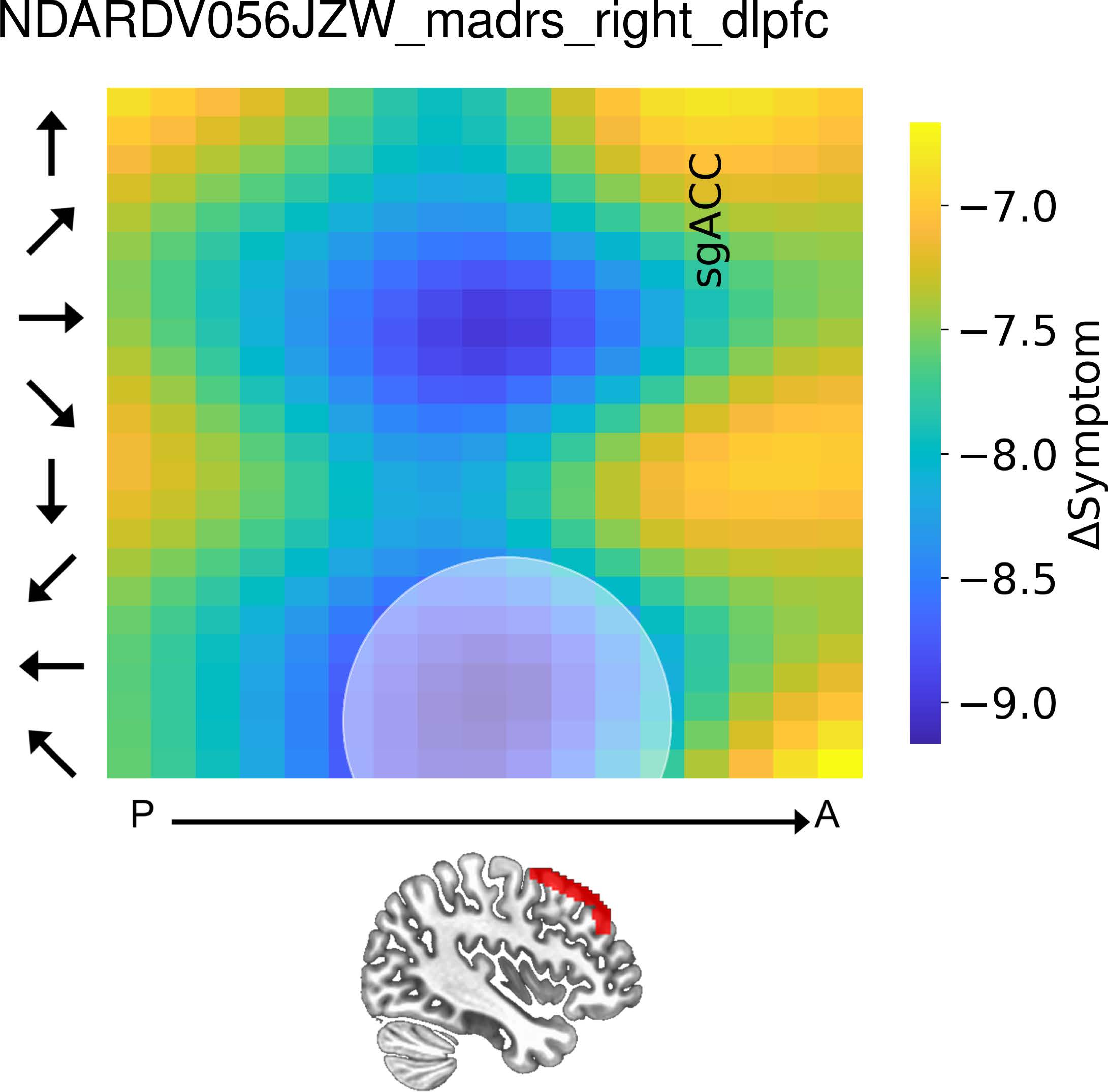
NDARDV056JZW_madrs_right_dlpfc
sgACC
−7.0
−7.5
−8.0
−8.5
−9.0
ΔSymptom
P
A

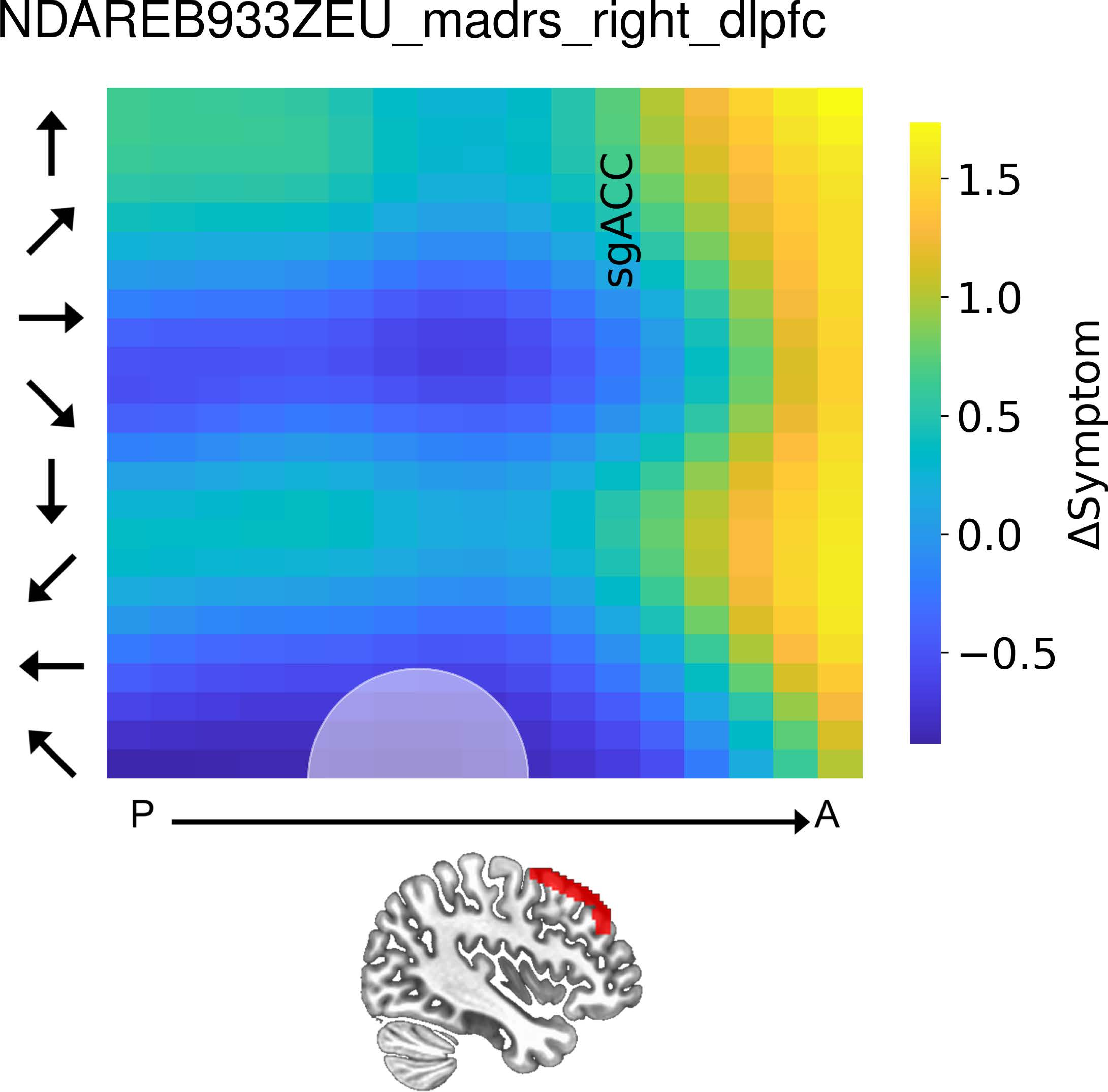
NDAREB933ZEU_madrs_right_dlpfc
sgACC
1.5
1.0
0.5
0.0
−0.5
ΔSymptom
P
A

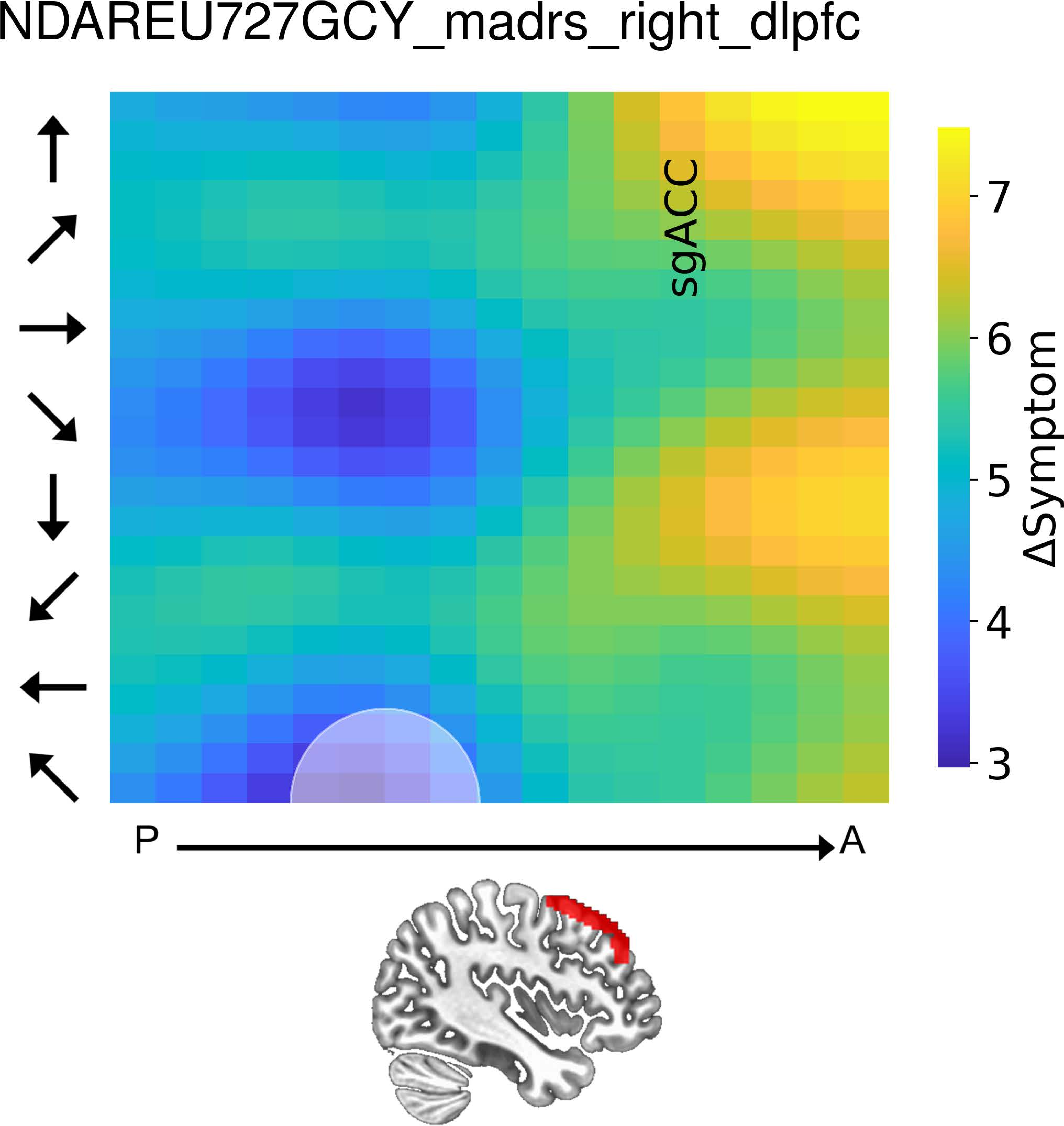

NDAREU727GCY_madrs_right_dlpfc
sgACC
P
A
ΔSymptom
7
6
5
4
3

NDAREW484UCJ_madrs_right_dlpfc

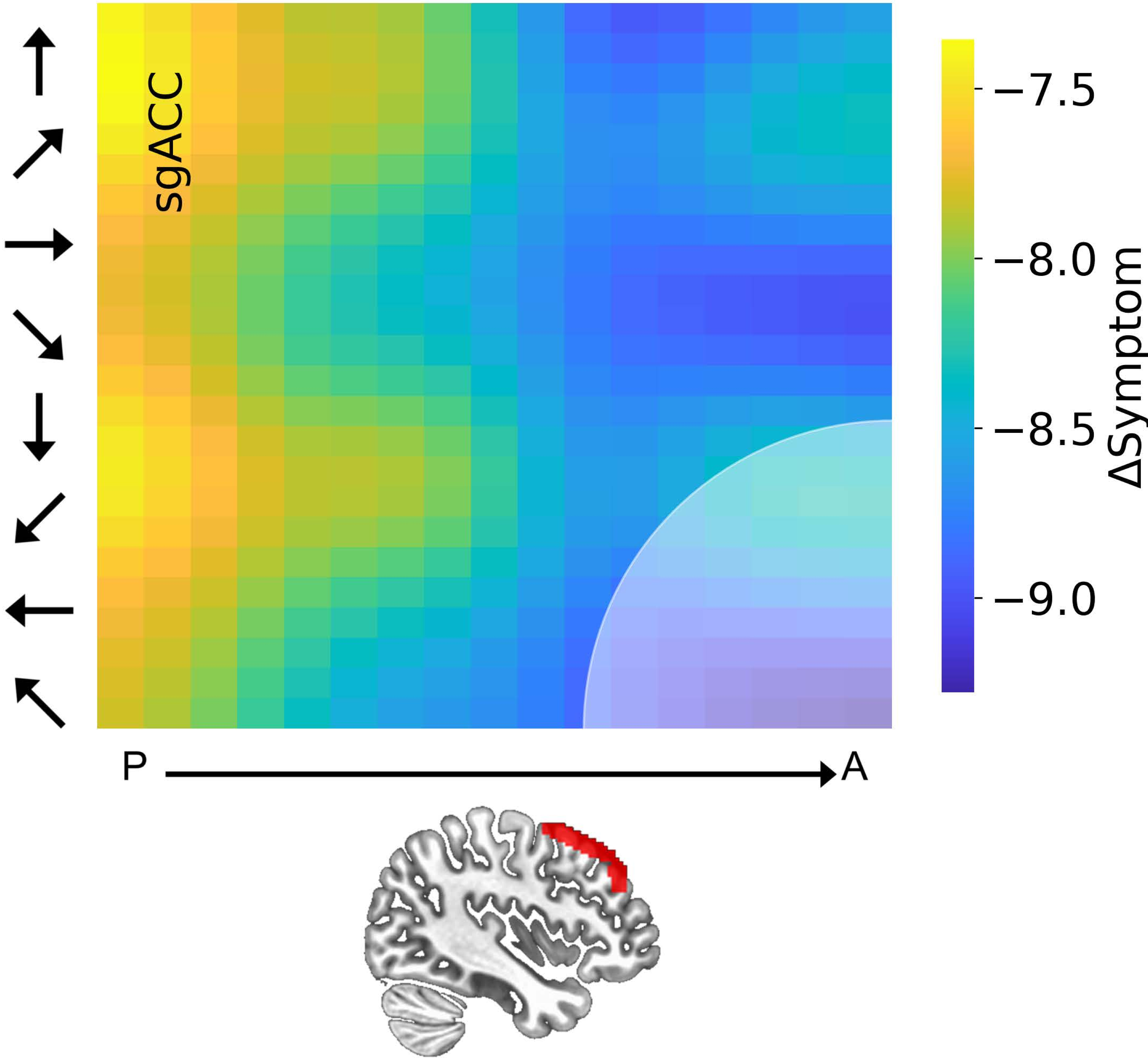

NDAREZ915JK5_madrs_right_dlpfc

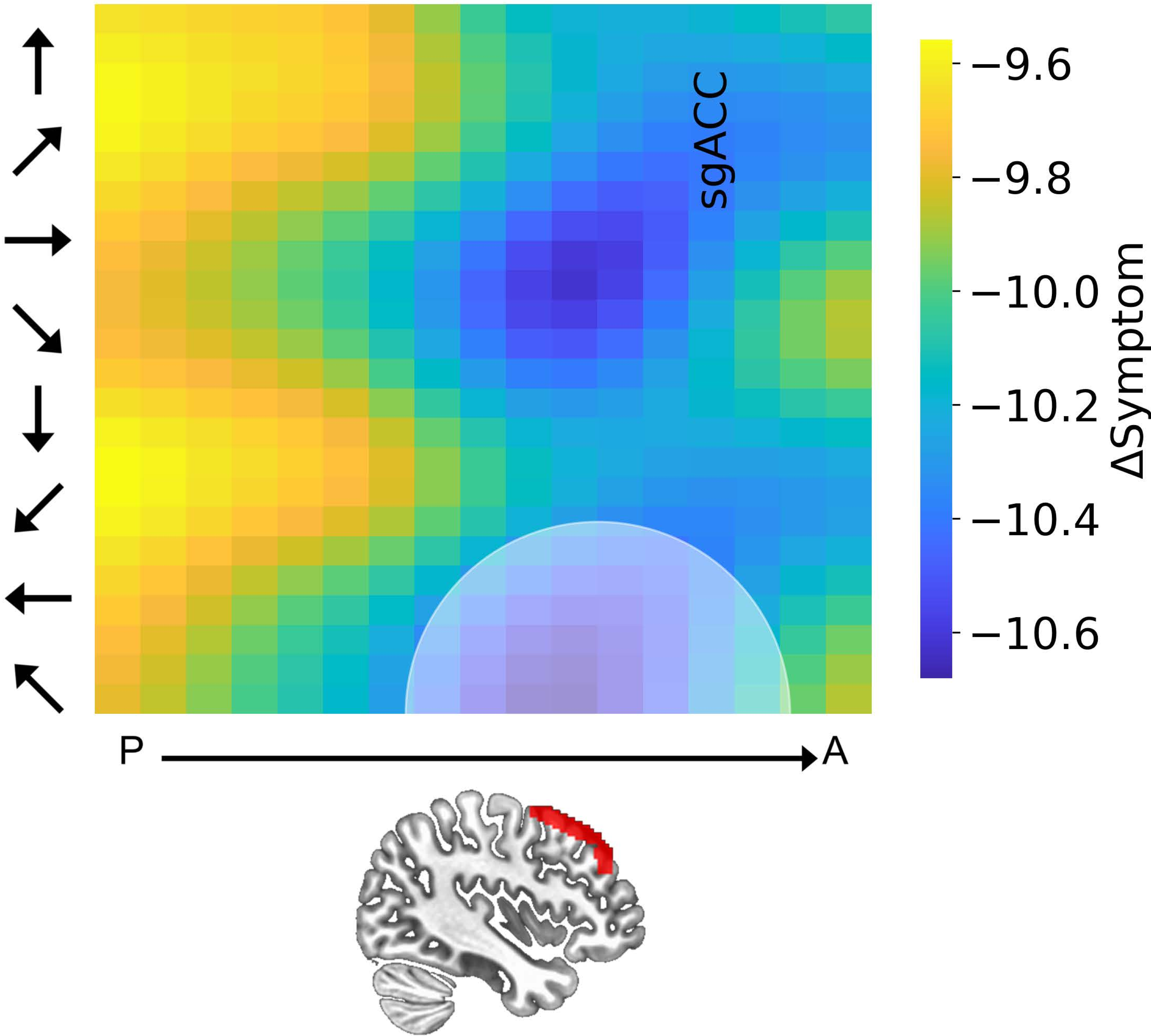

NDARFH525MFZ_madrs_right_dlpfc

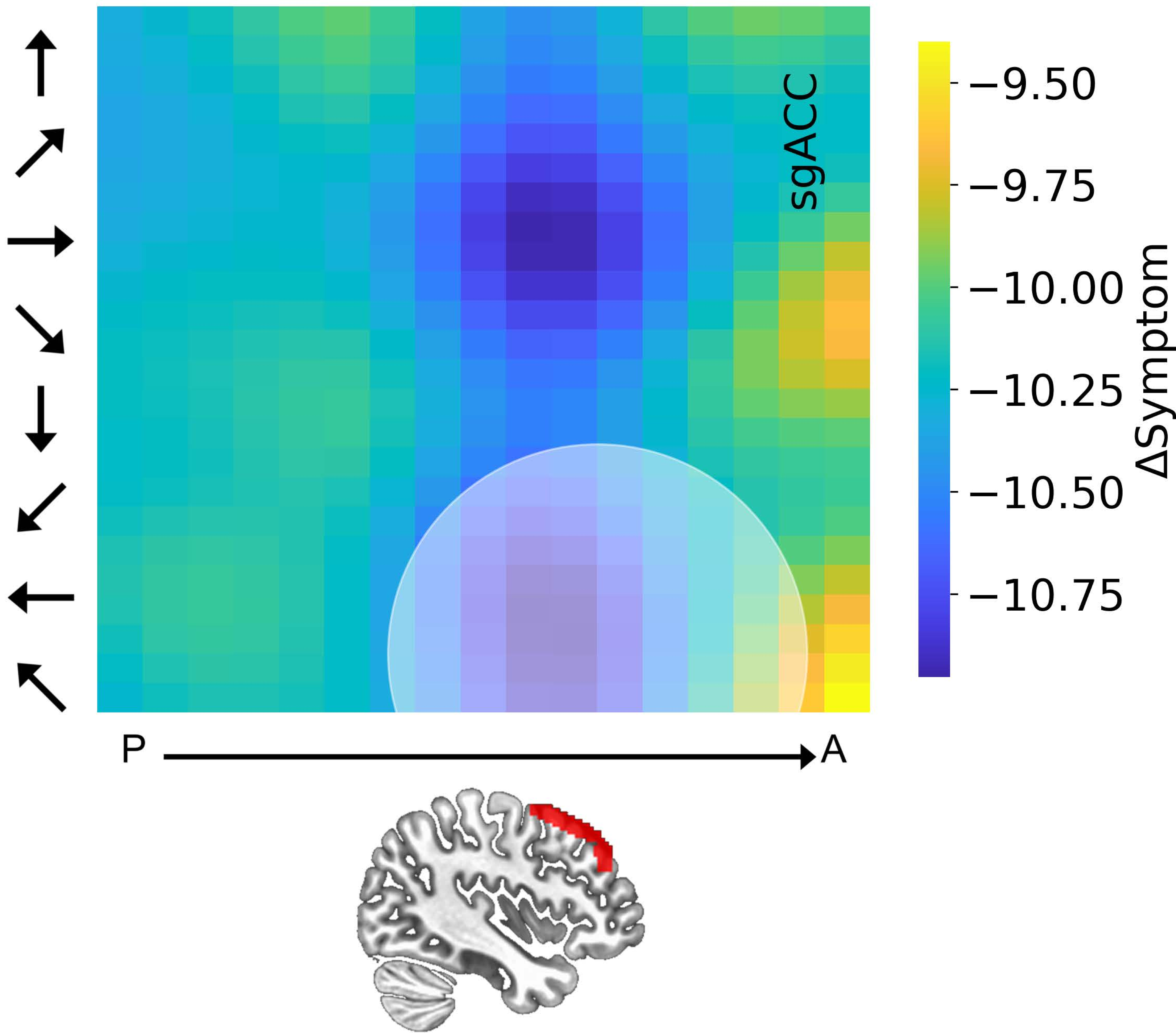

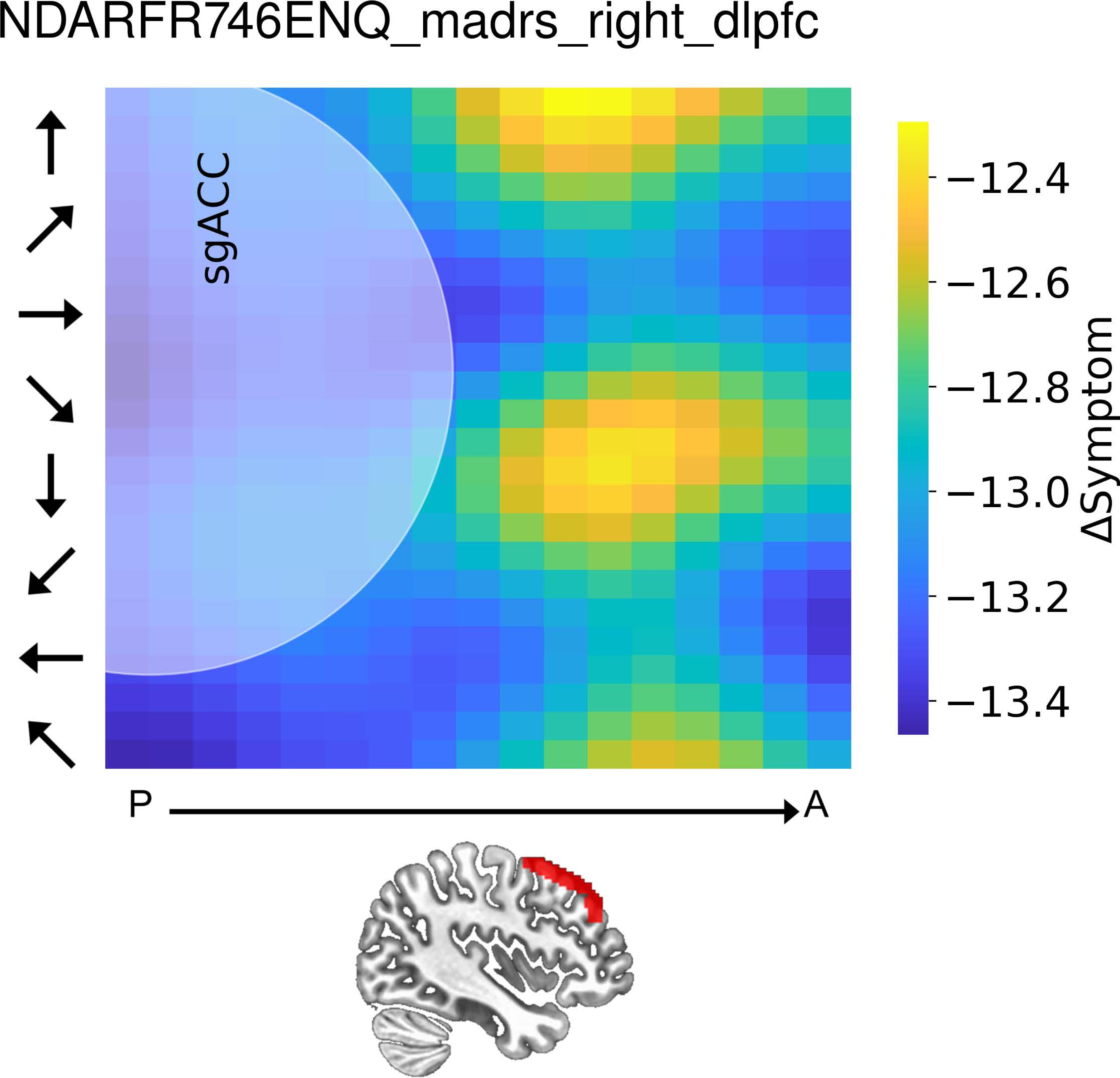
NDARFR746ENQ_madrs_right_dlpfc
sgACC
P
A
−12.4
−12.6
−12.8
−13.0
−13.2
−13.4
ΔSymptom

NDARFW096LMD_madrs_right_dlpfc

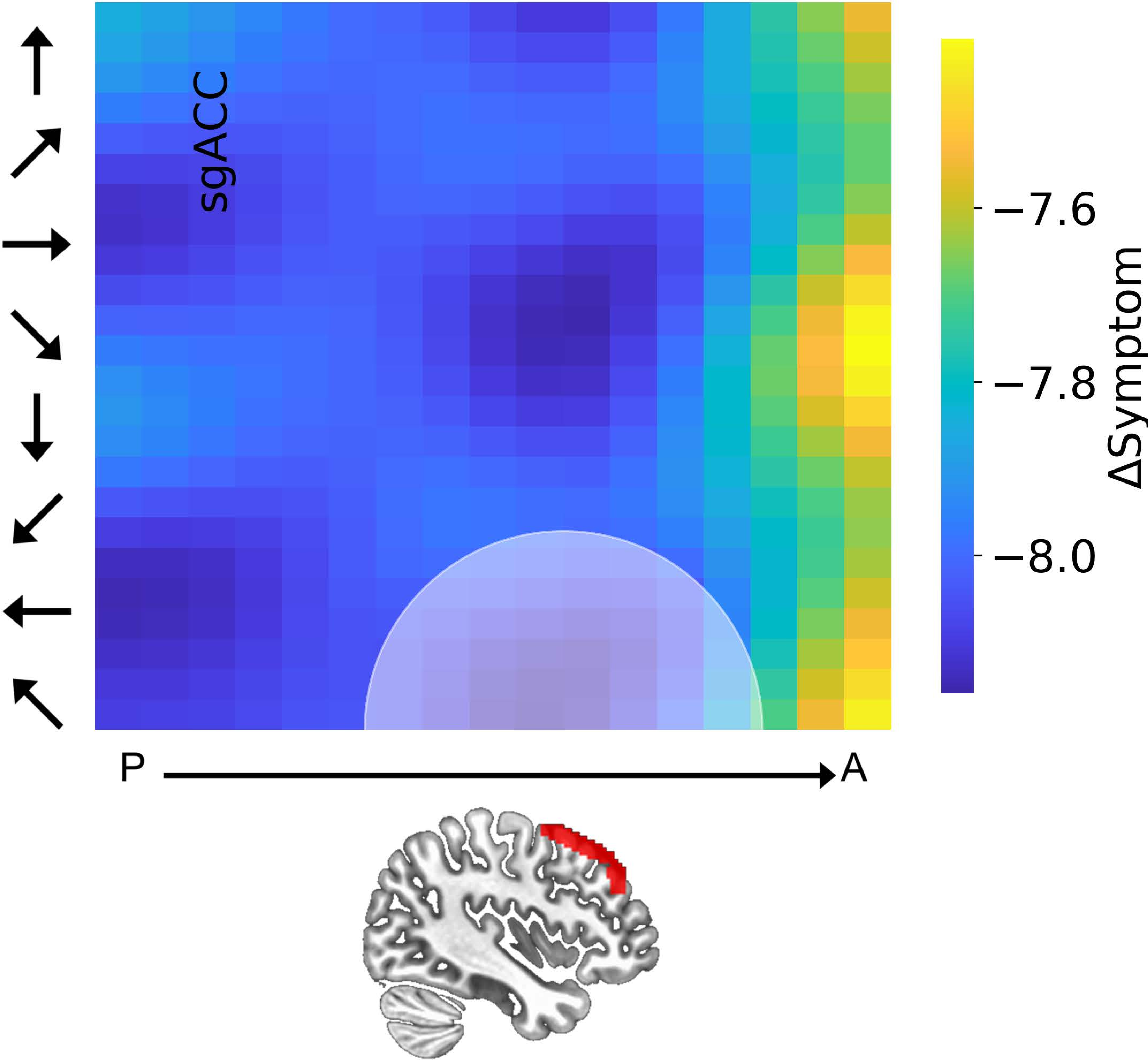

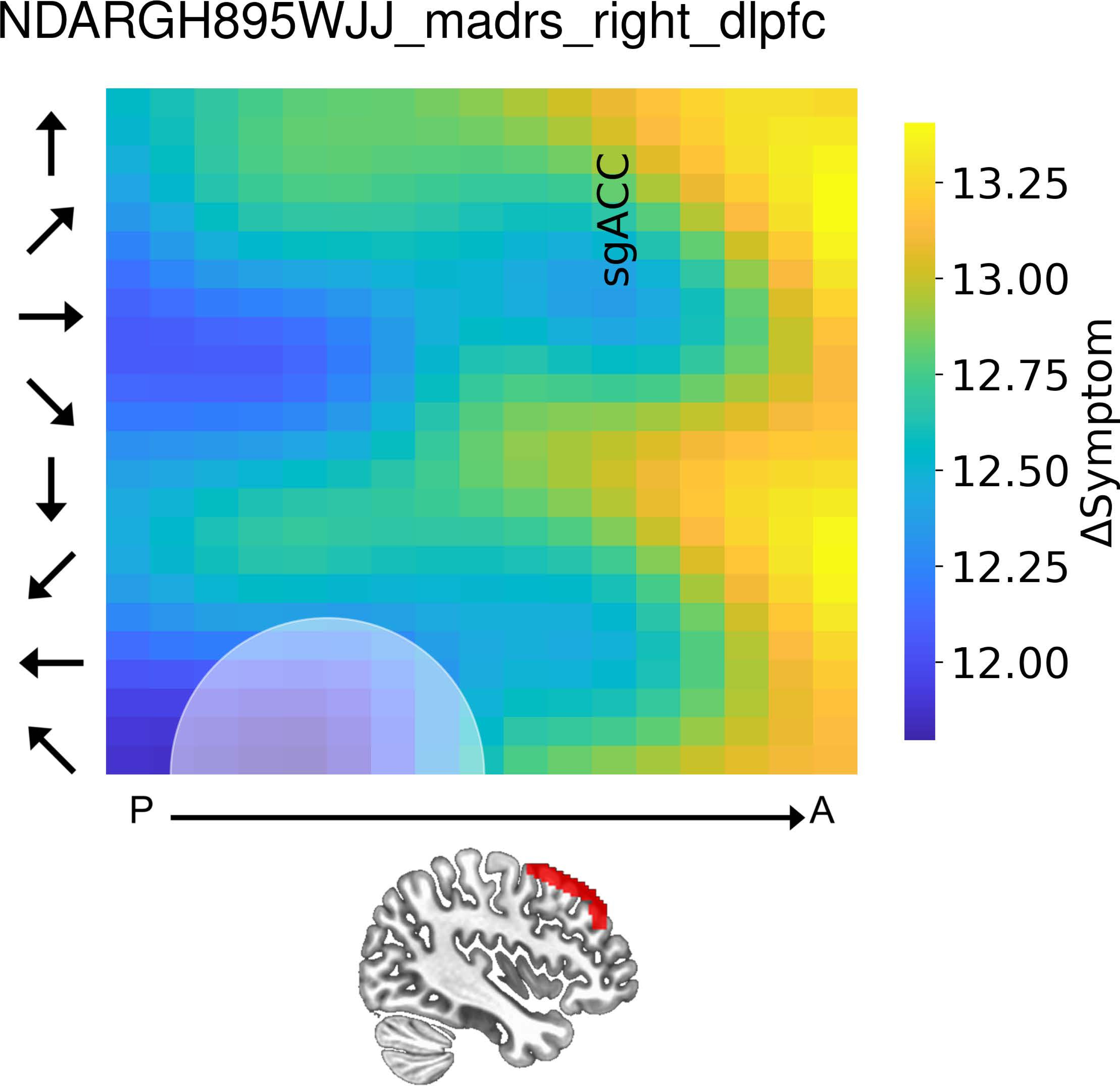
NDARGH895WJJ_madrs_right_dlpfc
sgACC
13.25
13.00
12.75
12.50
12.25
12.00
ΔSymptom
P
A

NDARGR637FTT_madrs_right_dlpfc

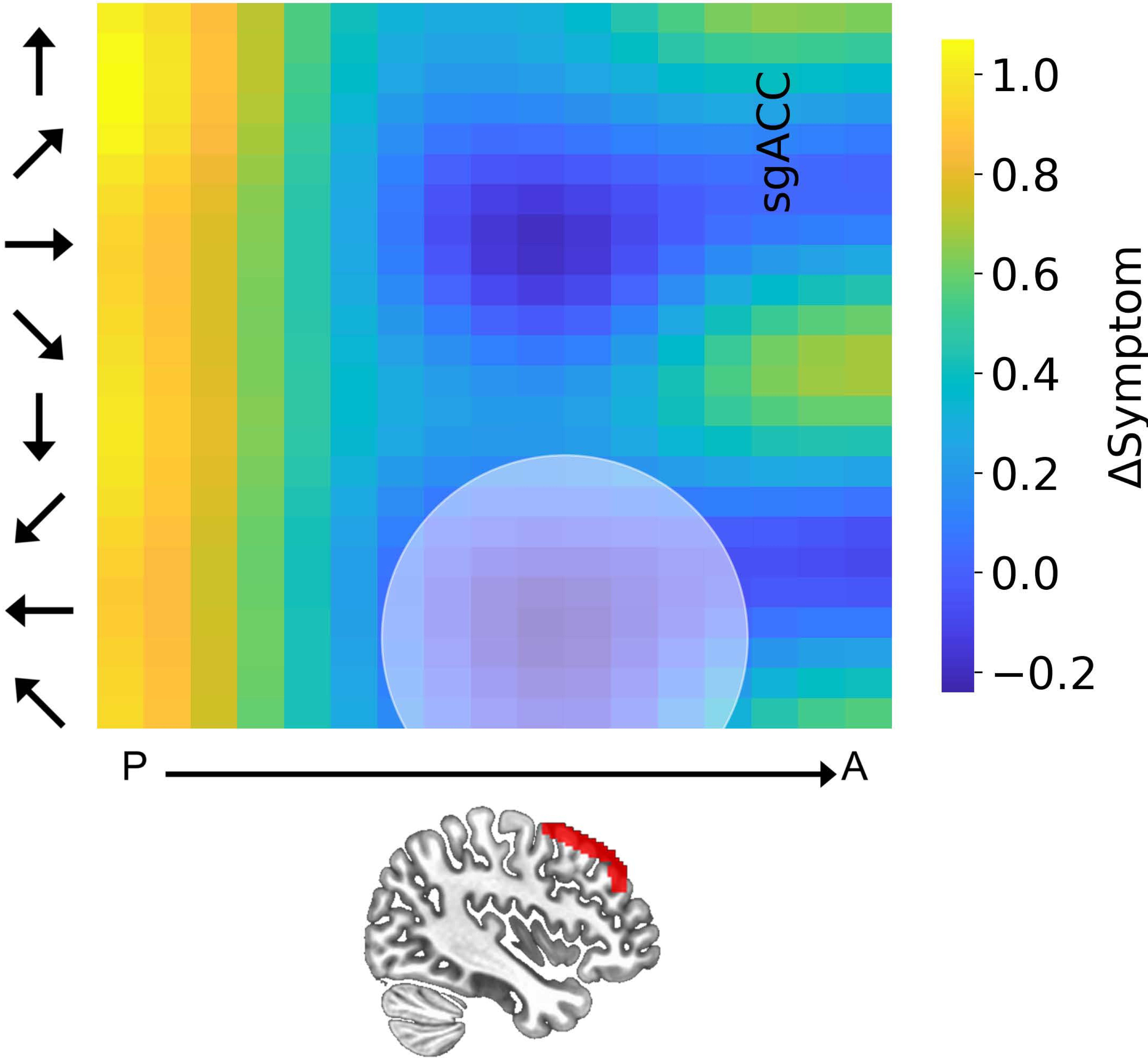

NDARGR782ALD_madrs_right_dlpfc

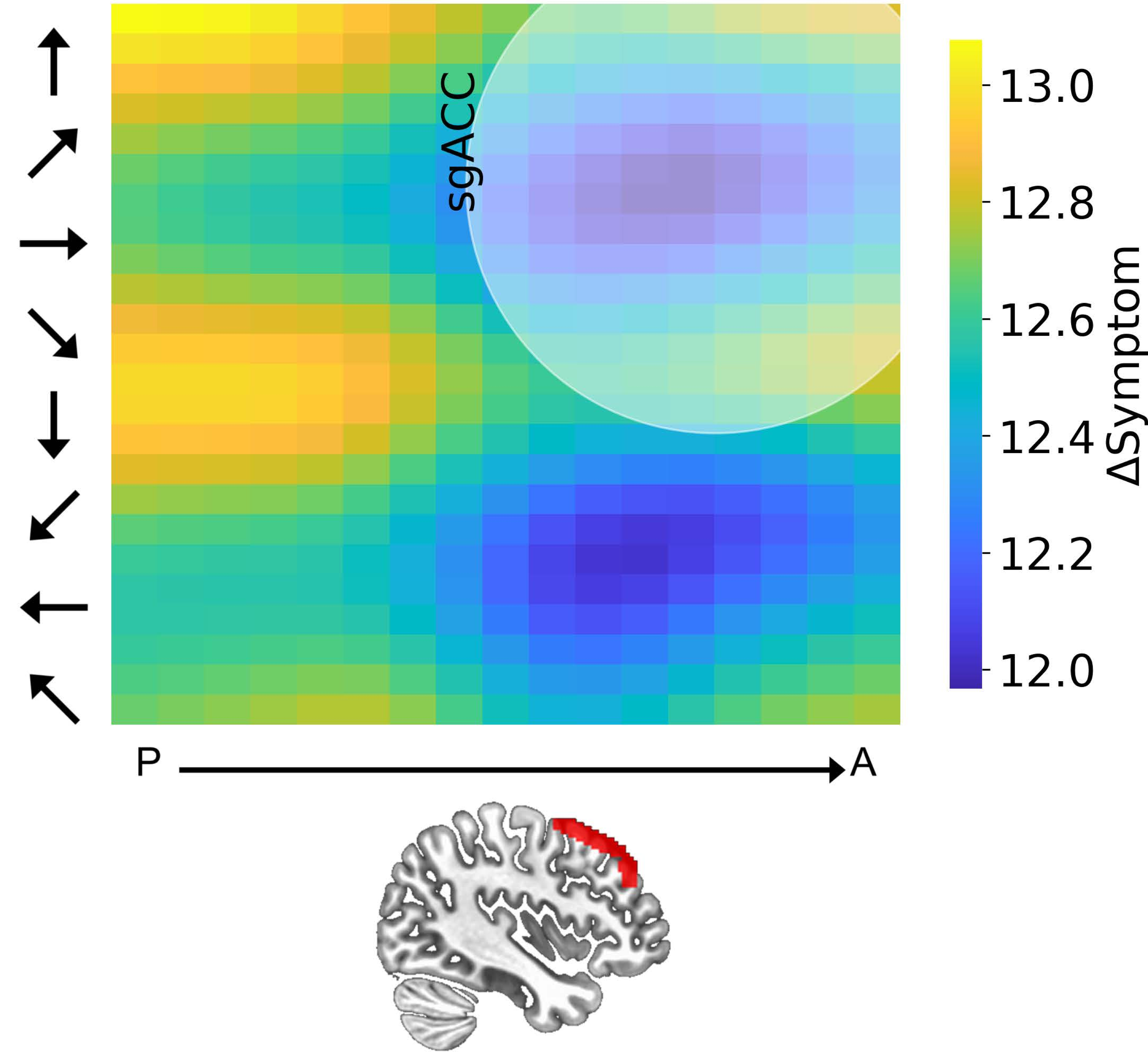

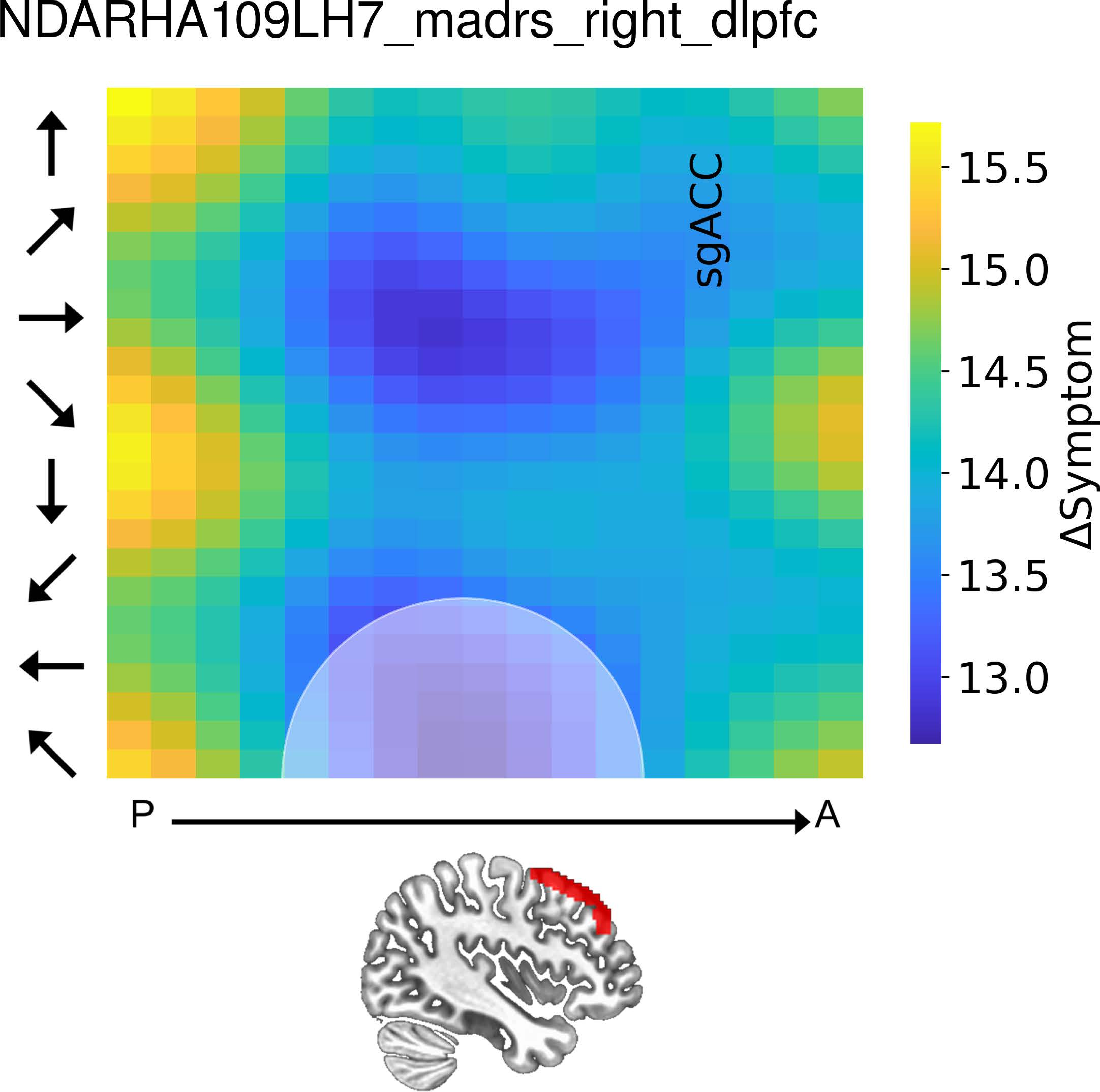
NDARHA109LH7_madrs_right_dlpfc
sgACC
15.5
15.0
14.5
14.0
13.5
13.0
ΔSymptom
P
A

NDARHR981AP1_madrs_right_dlpfc

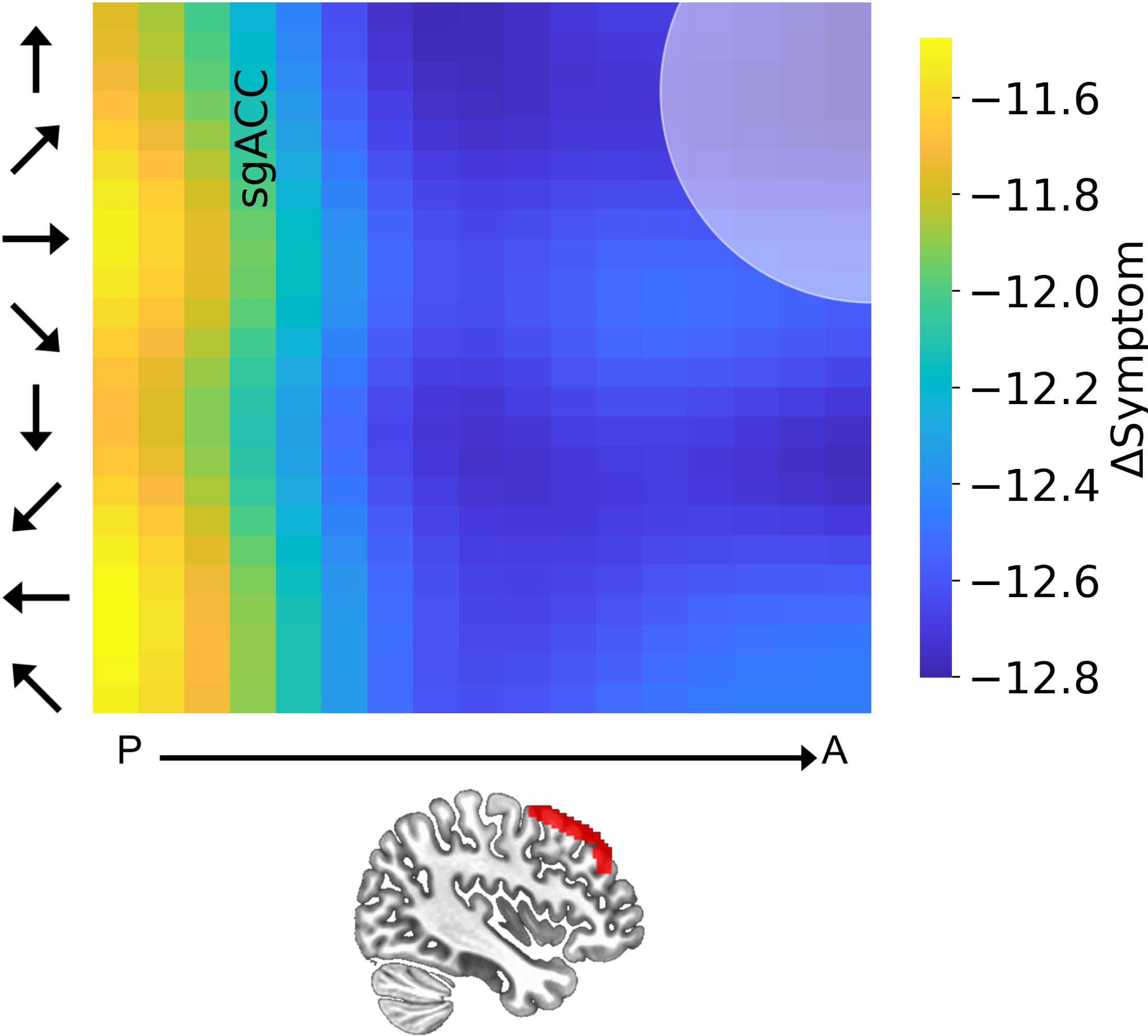

NDARHX487CT9_madrs_right_dlpfc

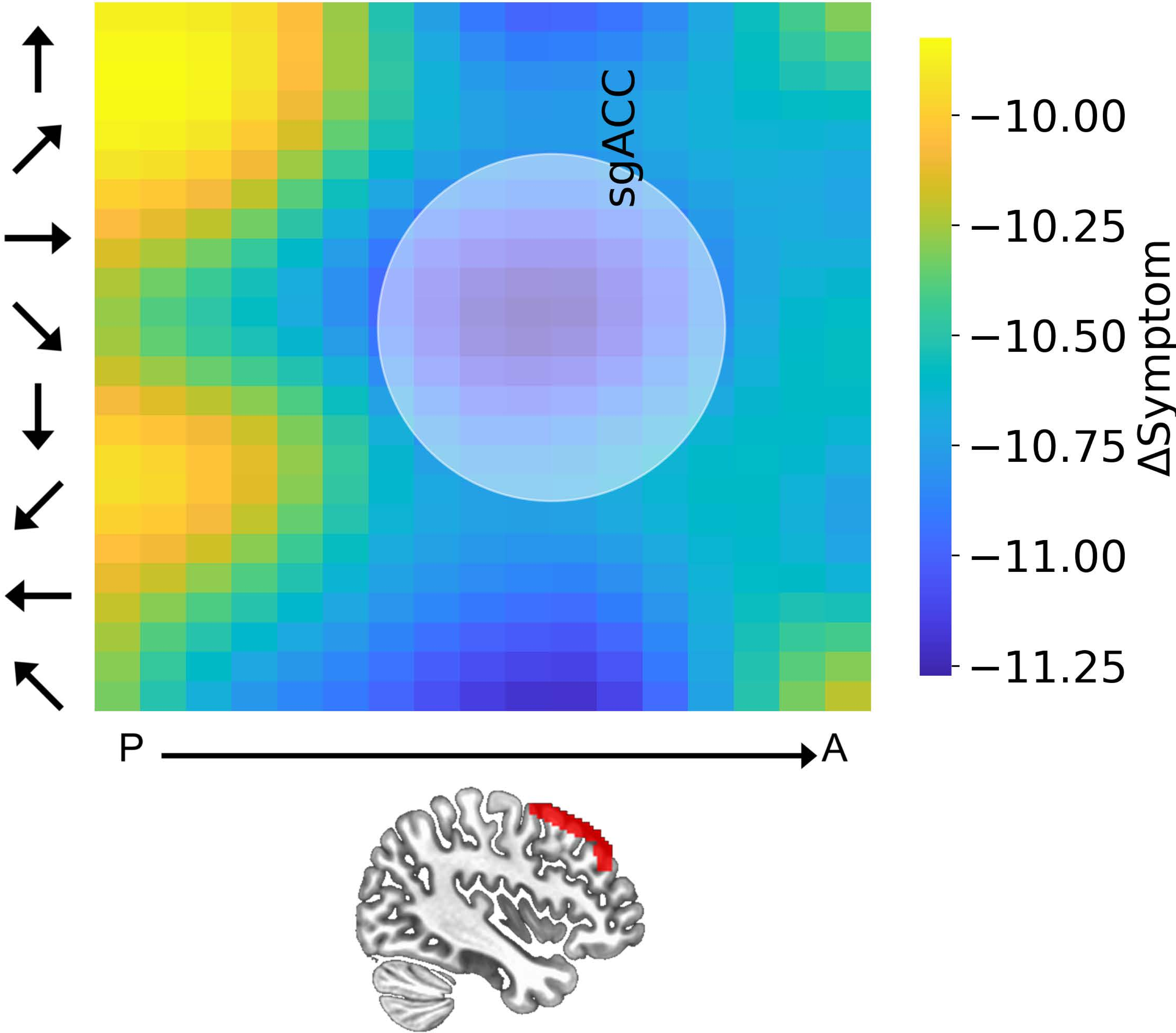

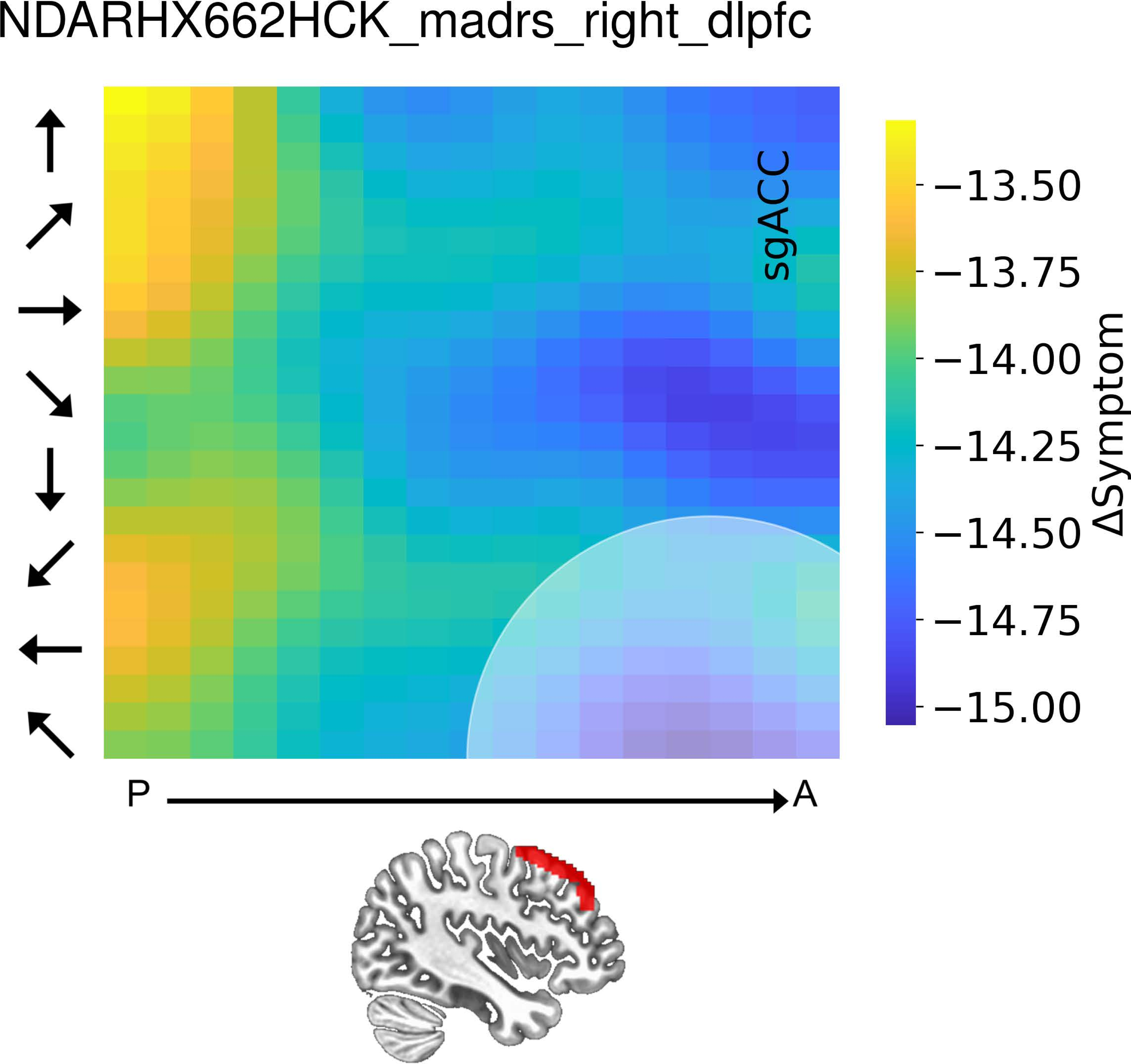

NDARHX662HCK_madrs_right_dlpfc
sgACC
P
A
−13.50
−13.75
−14.00
−14.25
−14.50
−14.75
−15.00
ΔSymptom

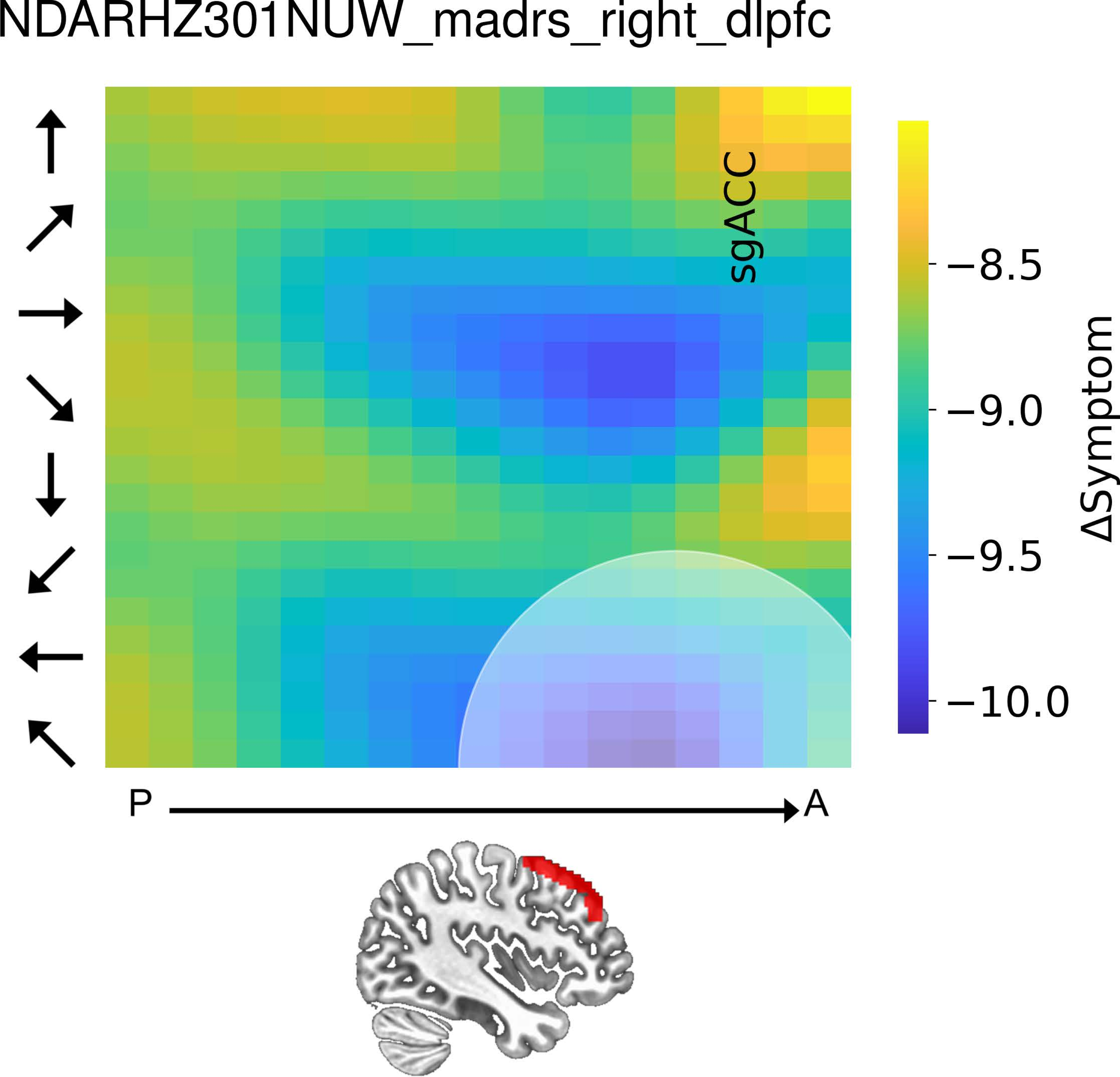
NDARHZ301NUW_madrs_right_dlpfc
sgACC
−8.5
−9.0
−9.5
−10.0
ΔSymptom
P
A

NDARHZ662EPK_madrs_right_dlpfc

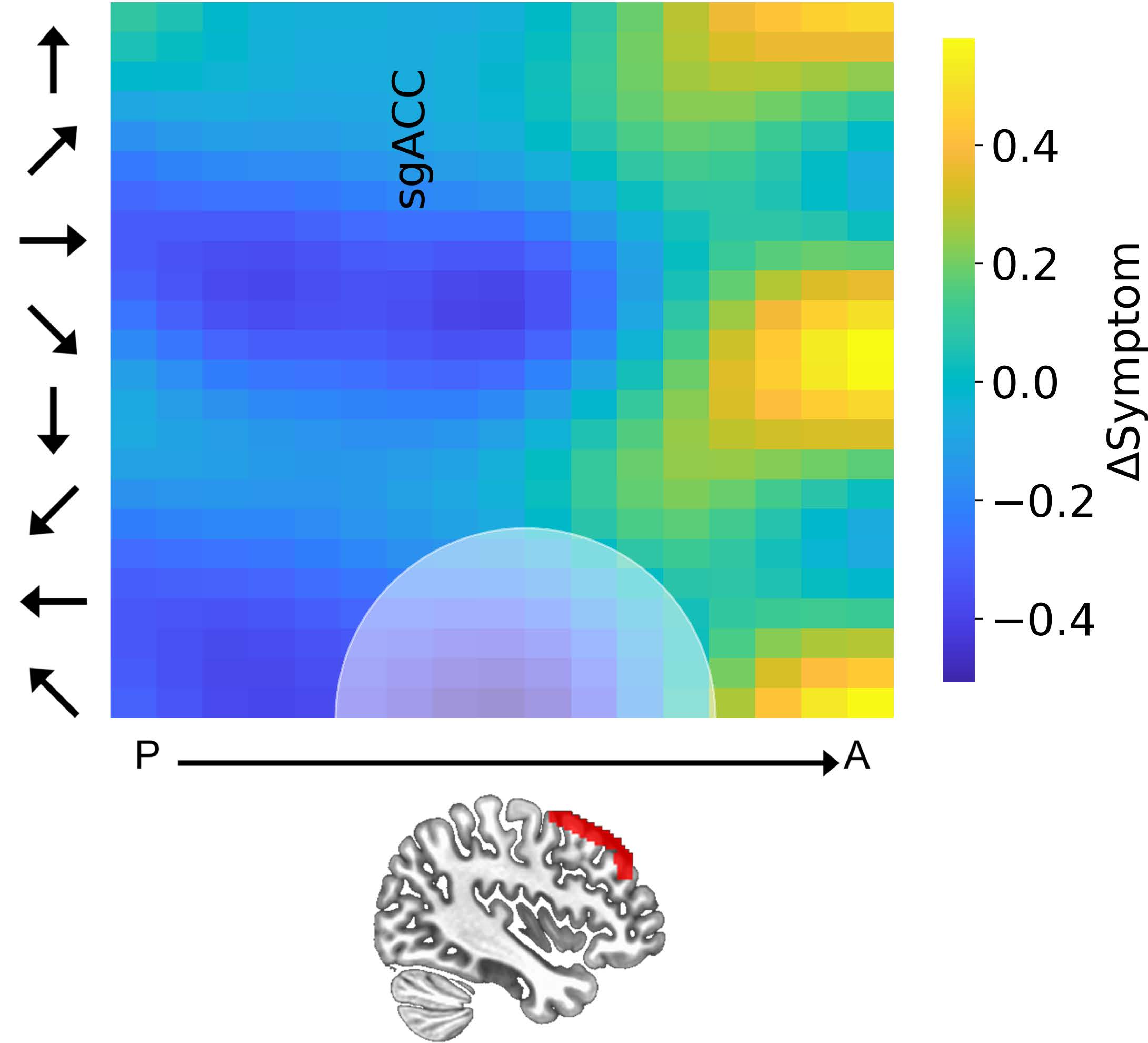

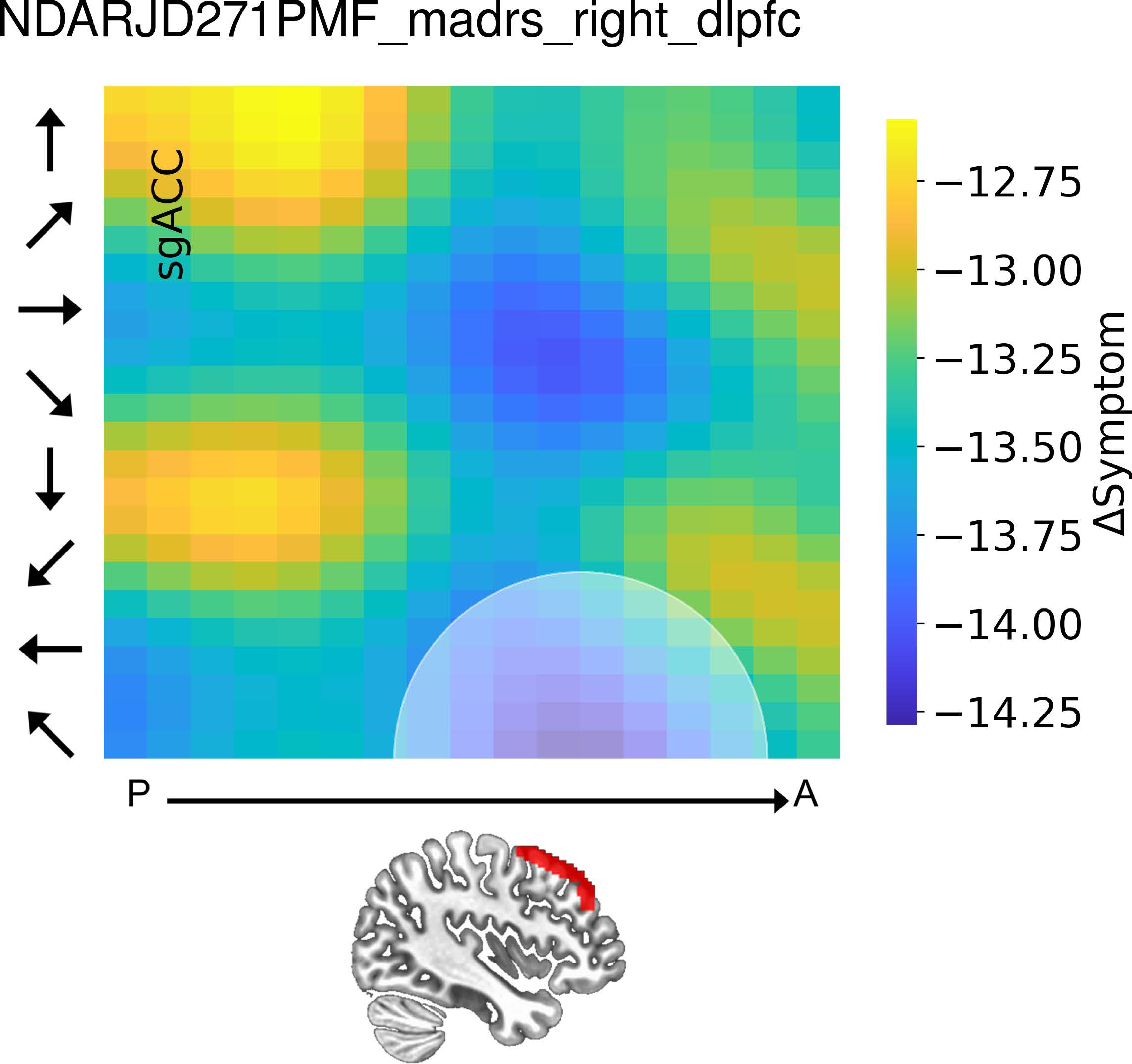
NDARJD271PMF_madrs_right_dlpfc
sgACC
P
A
−12.75
−13.00
−13.25
−13.50
−13.75
−14.00
−14.25
ΔSymptom

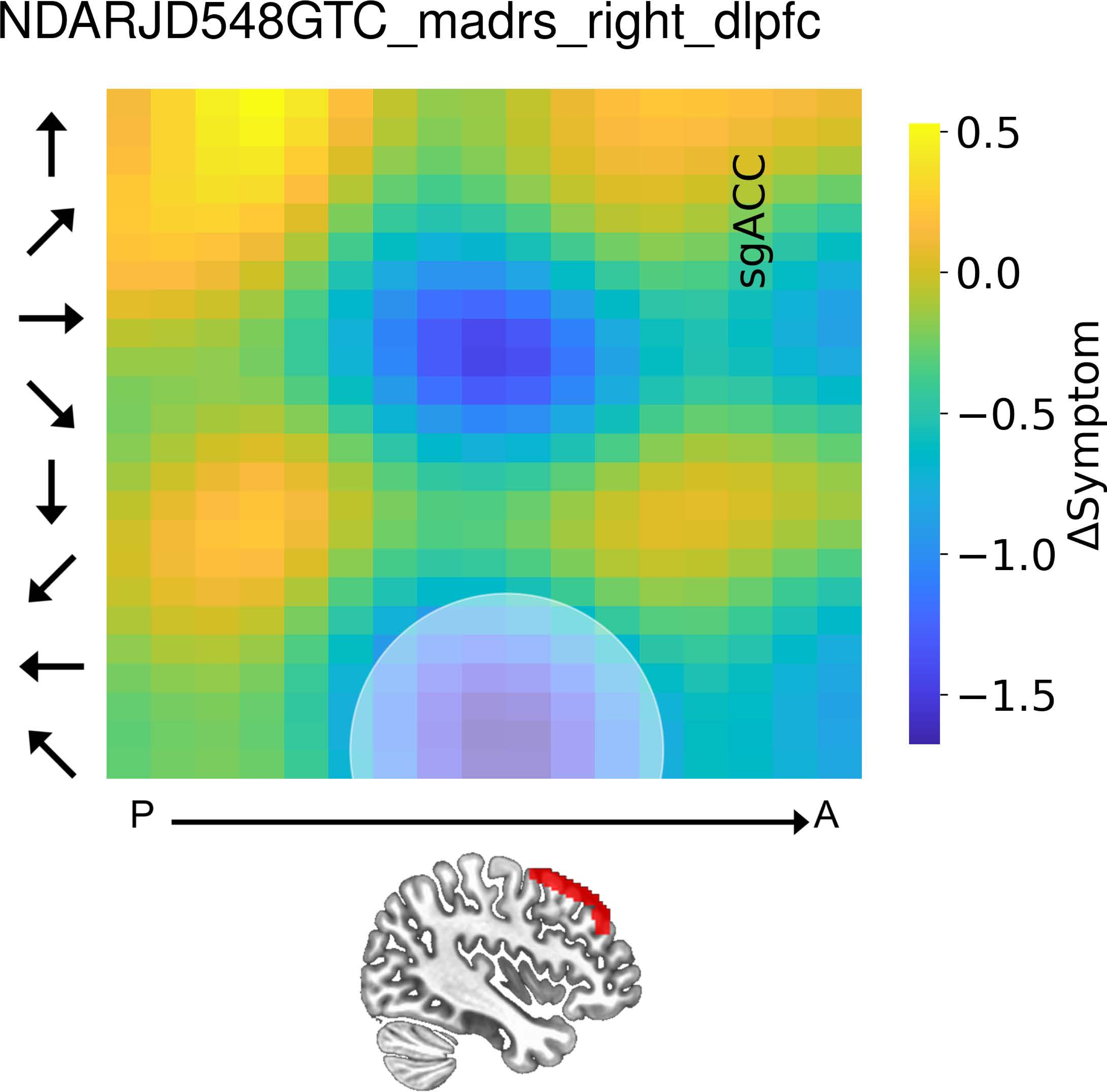
NDARJD548GTC_madrs_right_dlpfc
sgACC
0.5
0.0
−0.5
−1.0
−1.5
ΔSymptom
P
A

NDARJF993NV1_madrs_right_dlpfc

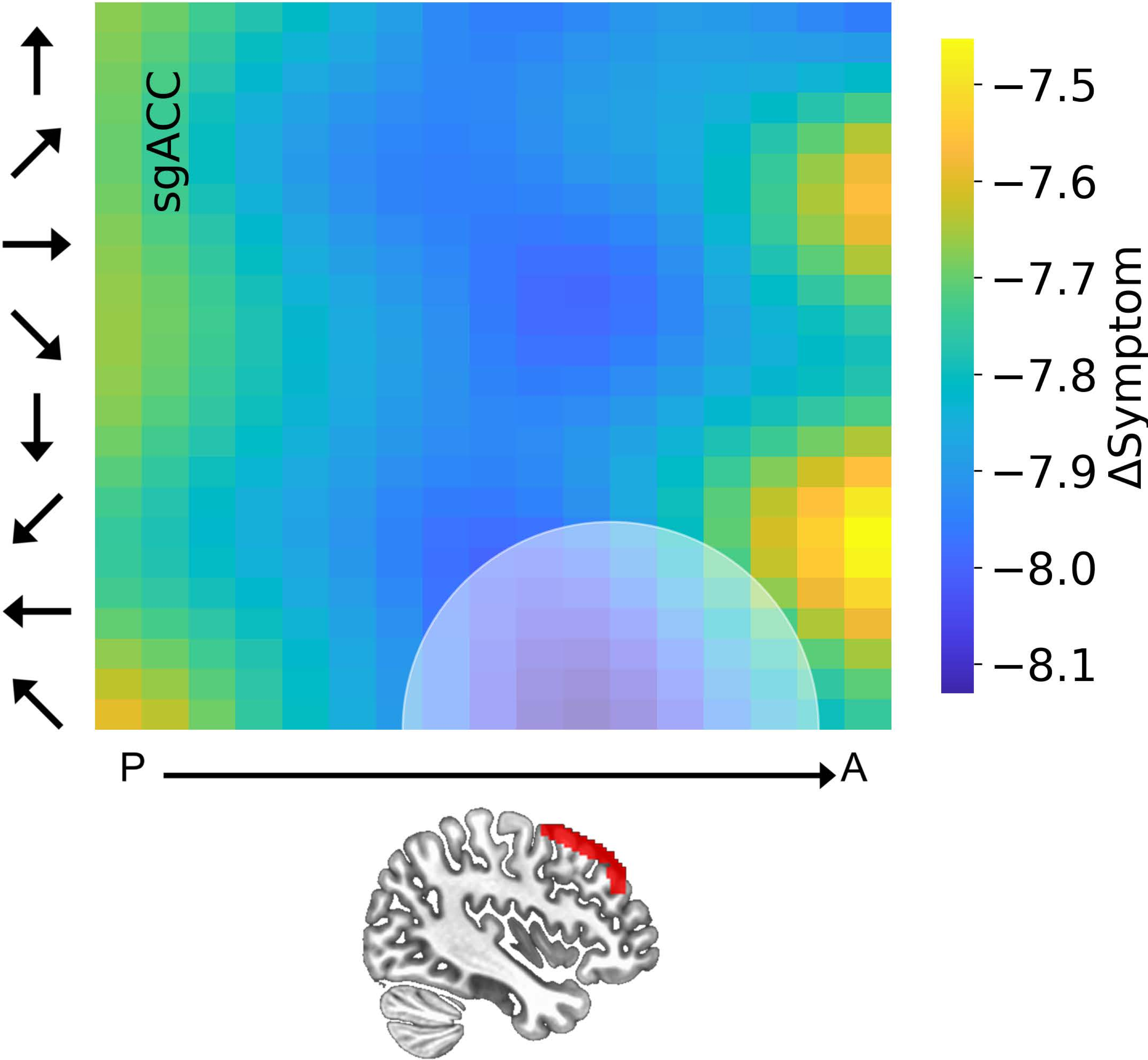

NDARJH406WC9_madrs_right_dlpfc

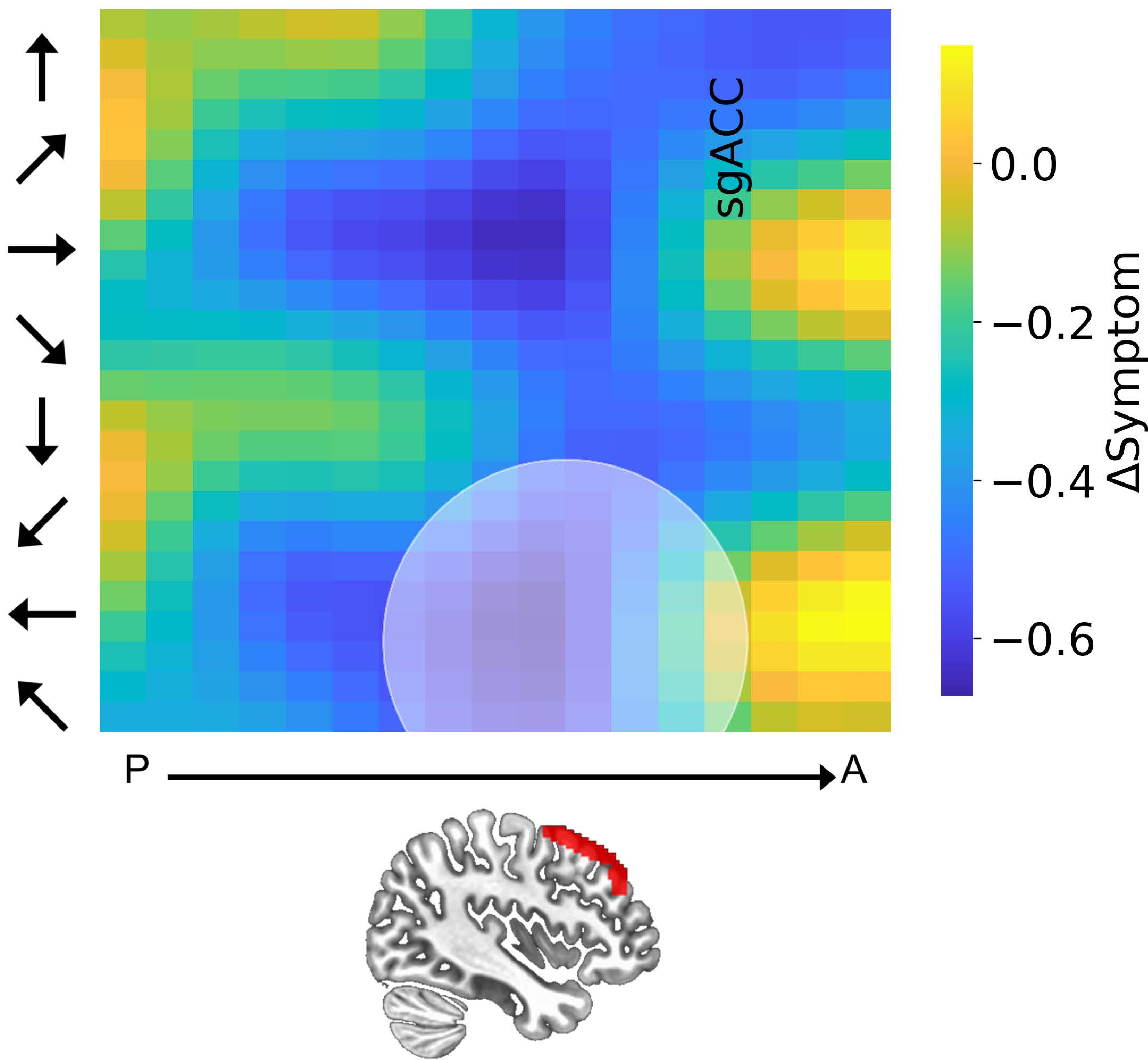

NDARJX575DVK_madrs_right_dlpfc

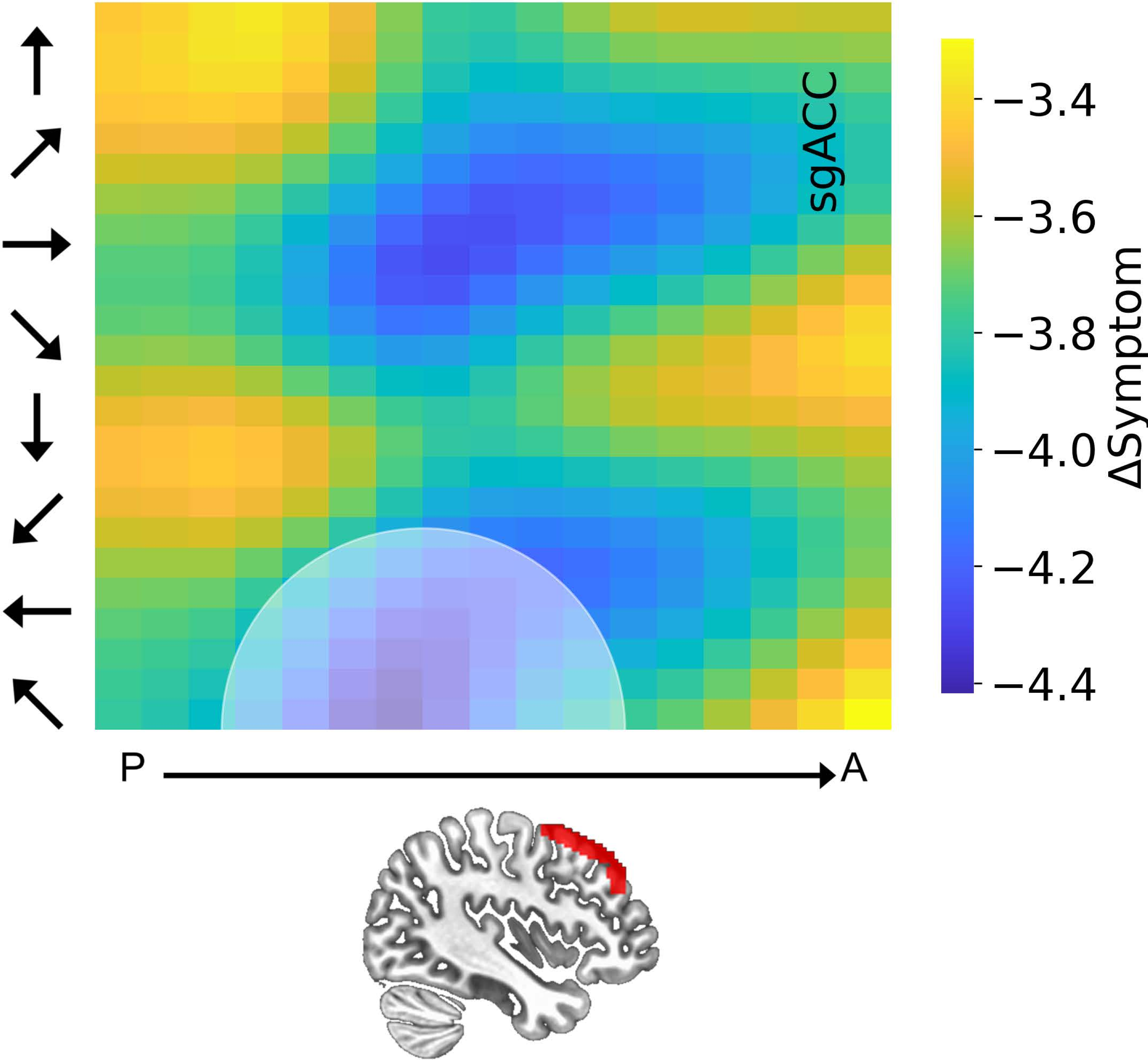

NDARJY042TLY_madrs_right_dlpfc

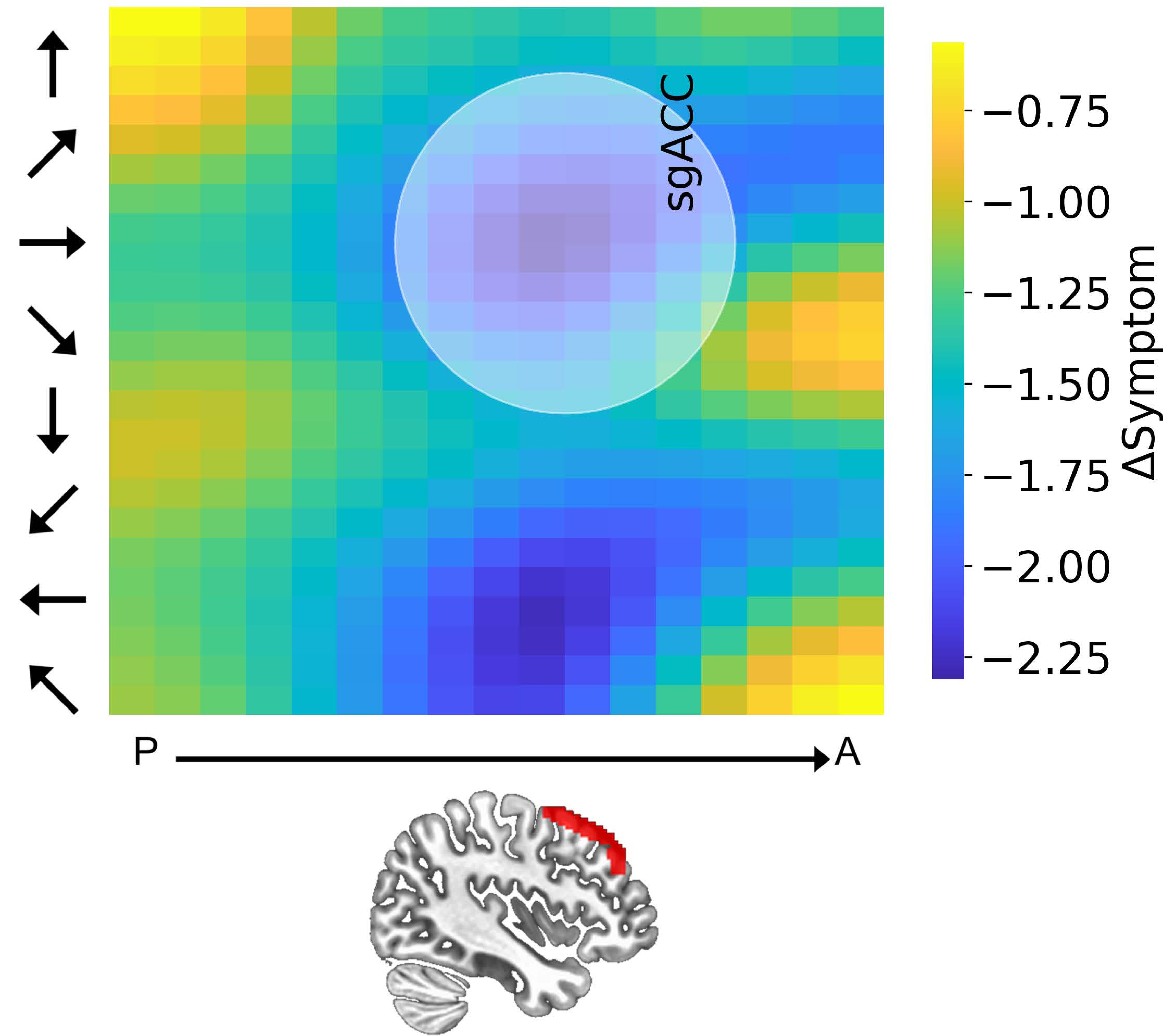

NDARKK212YTX_madrs_right_dlpfc

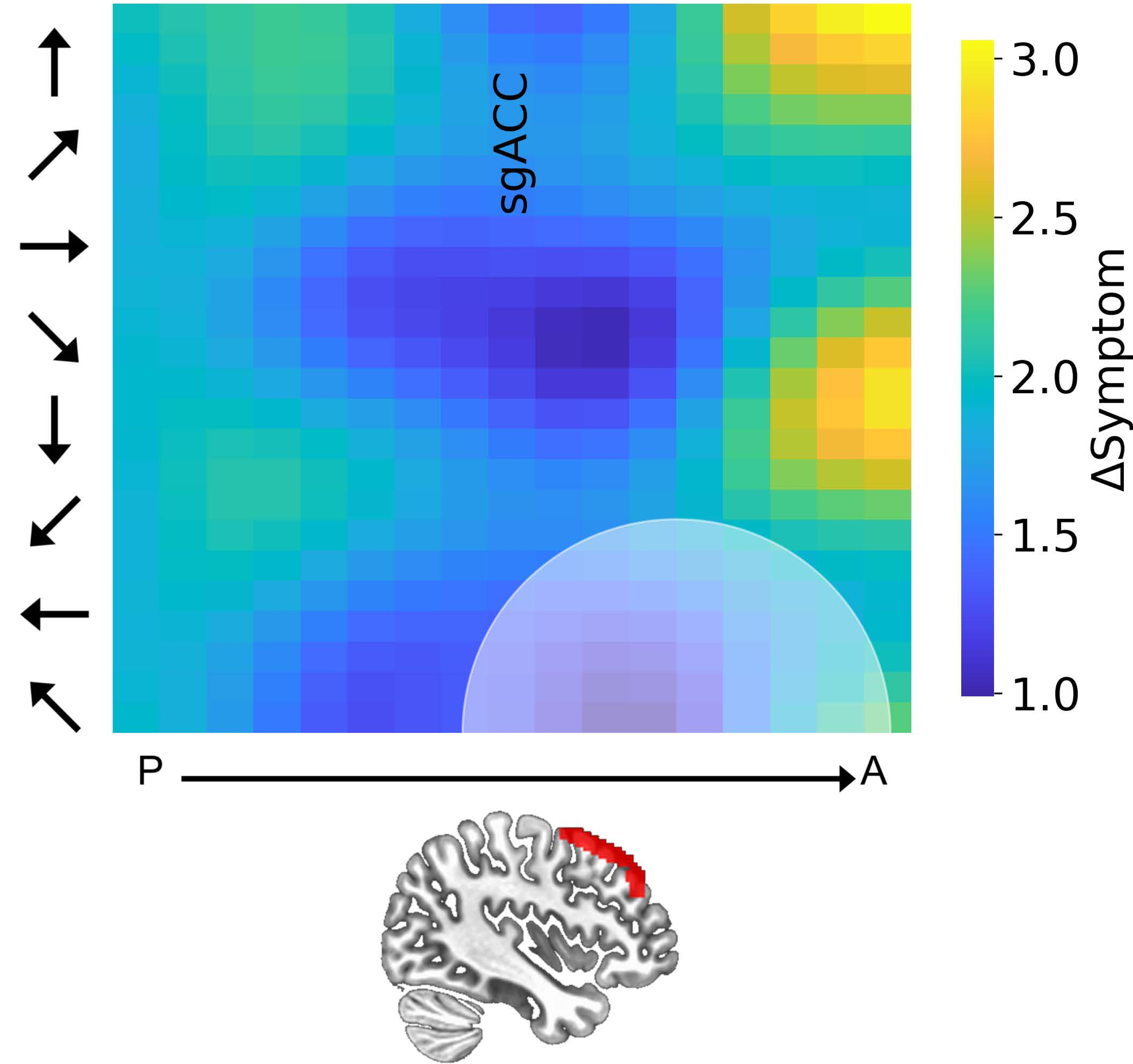

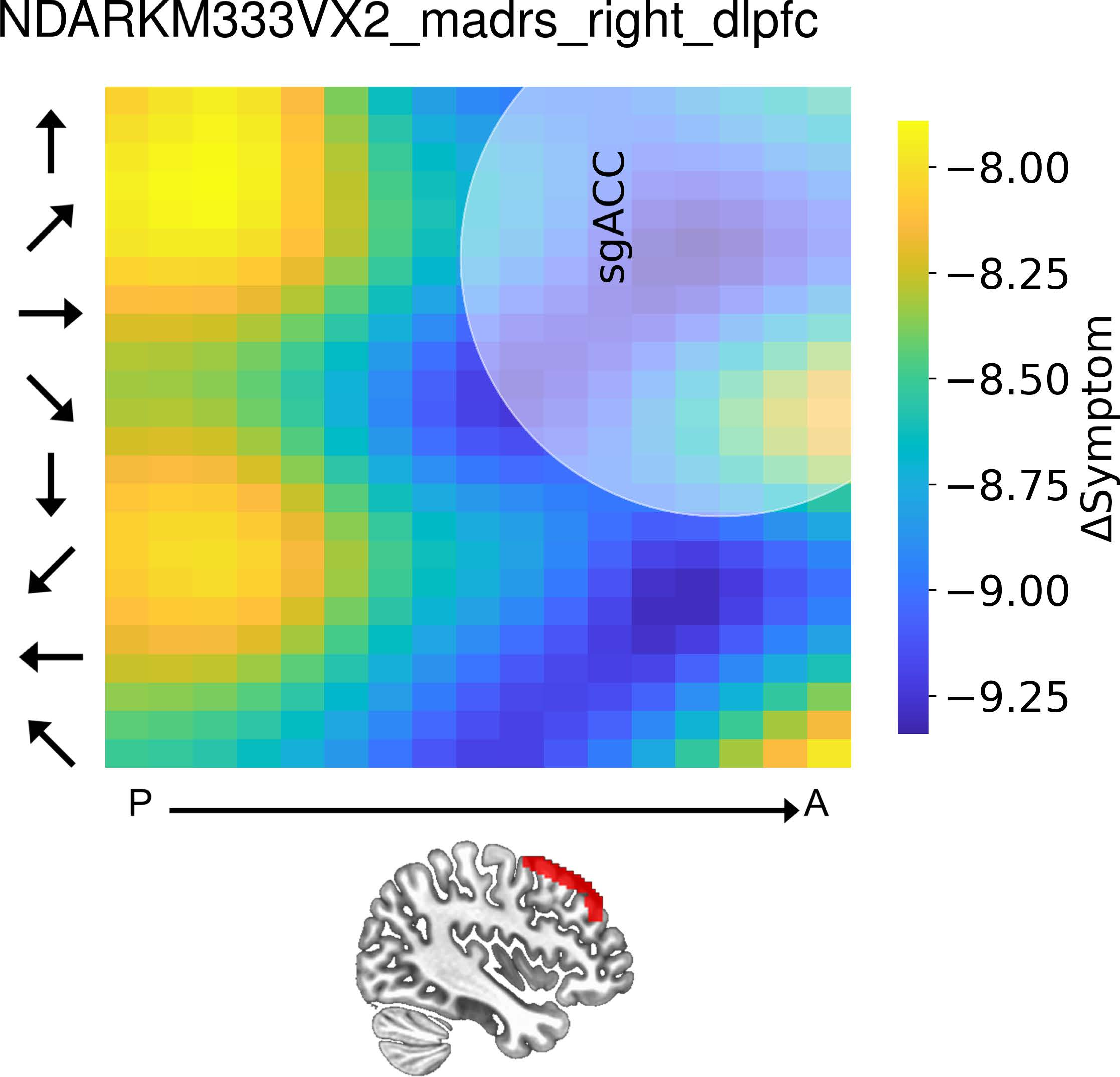
NDARKM333VX2_madrs_right_dlpfc
sgACC
−8.00
−8.25
−8.50
−8.75
−9.00
−9.25
ΔSymptom
P
A

NDARKT991DUC_madrs_right_dlpfc

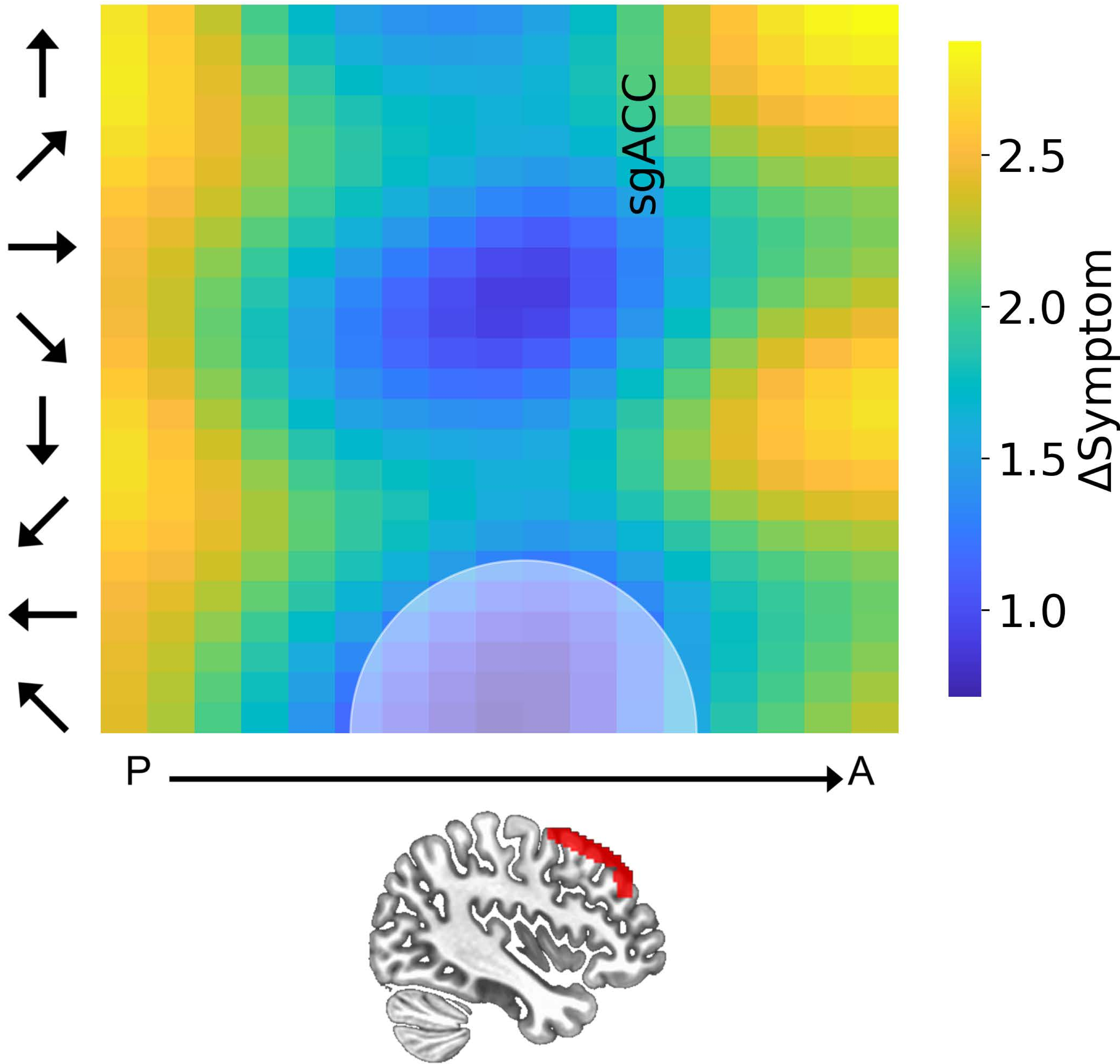

NDARKU015LTM_madrs_right_dlpfc

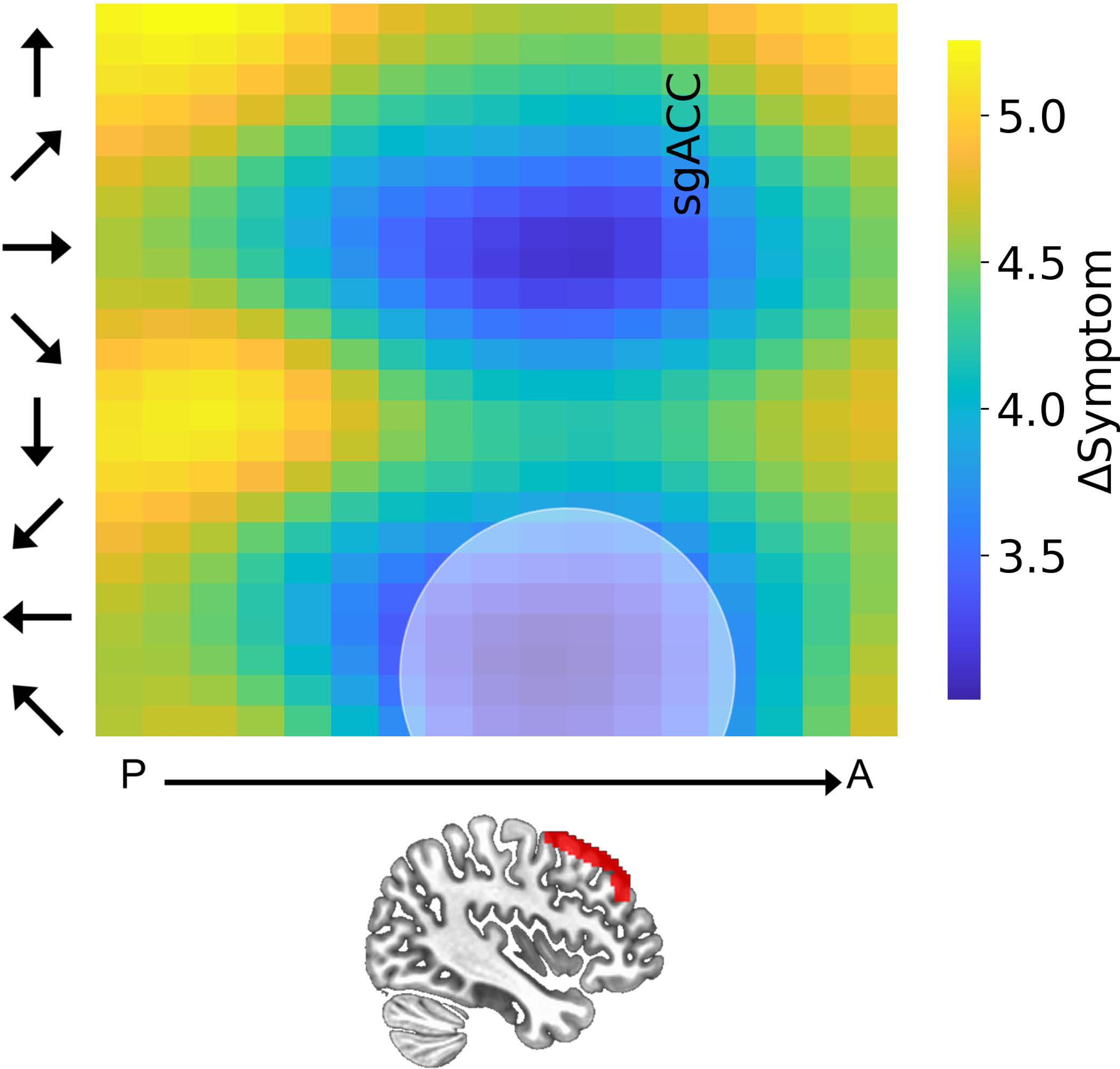

NDARKV126RTP_madrs_right_dlpfc

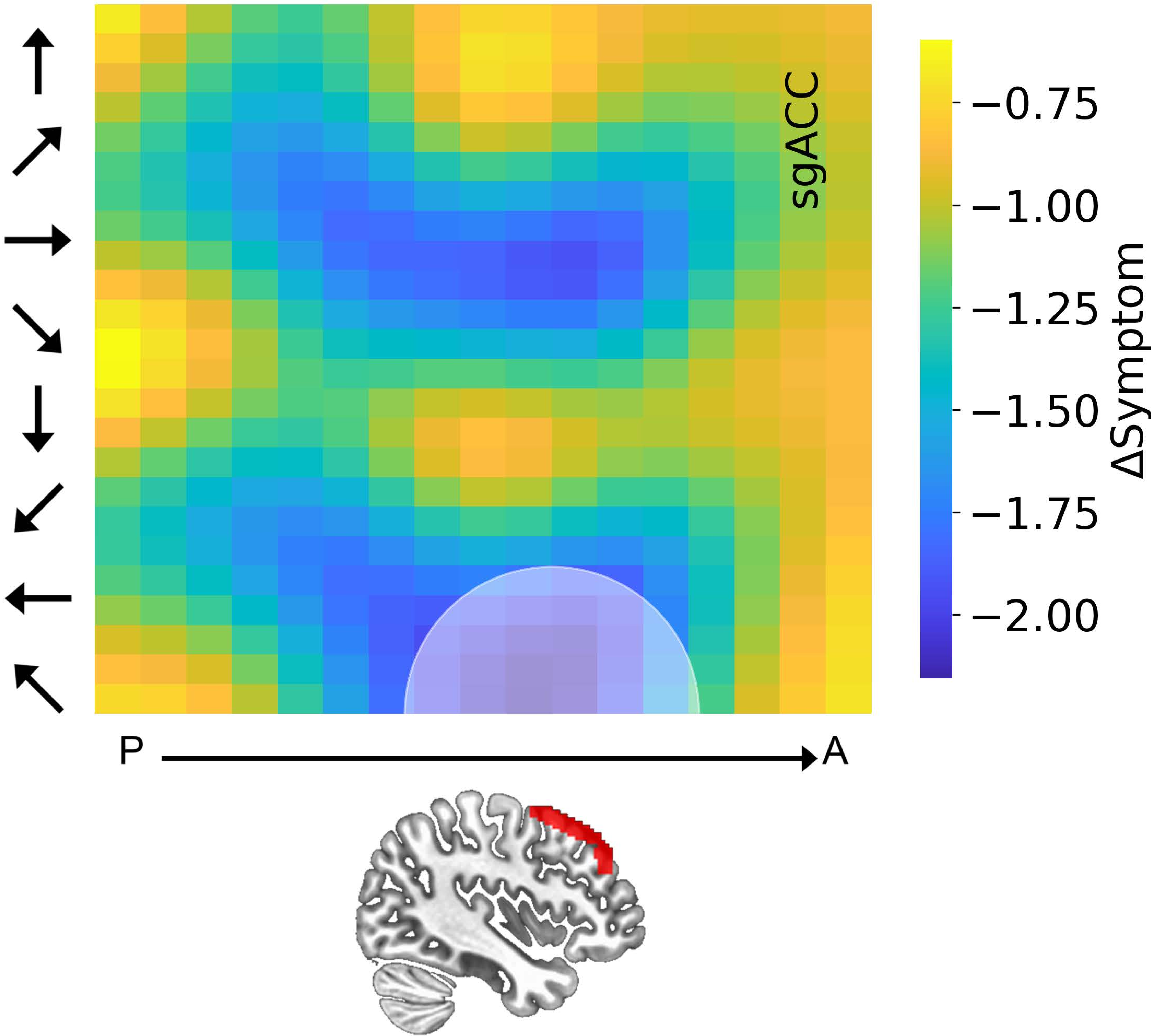

NDARLF826BAB_madrs_right_dlpfc

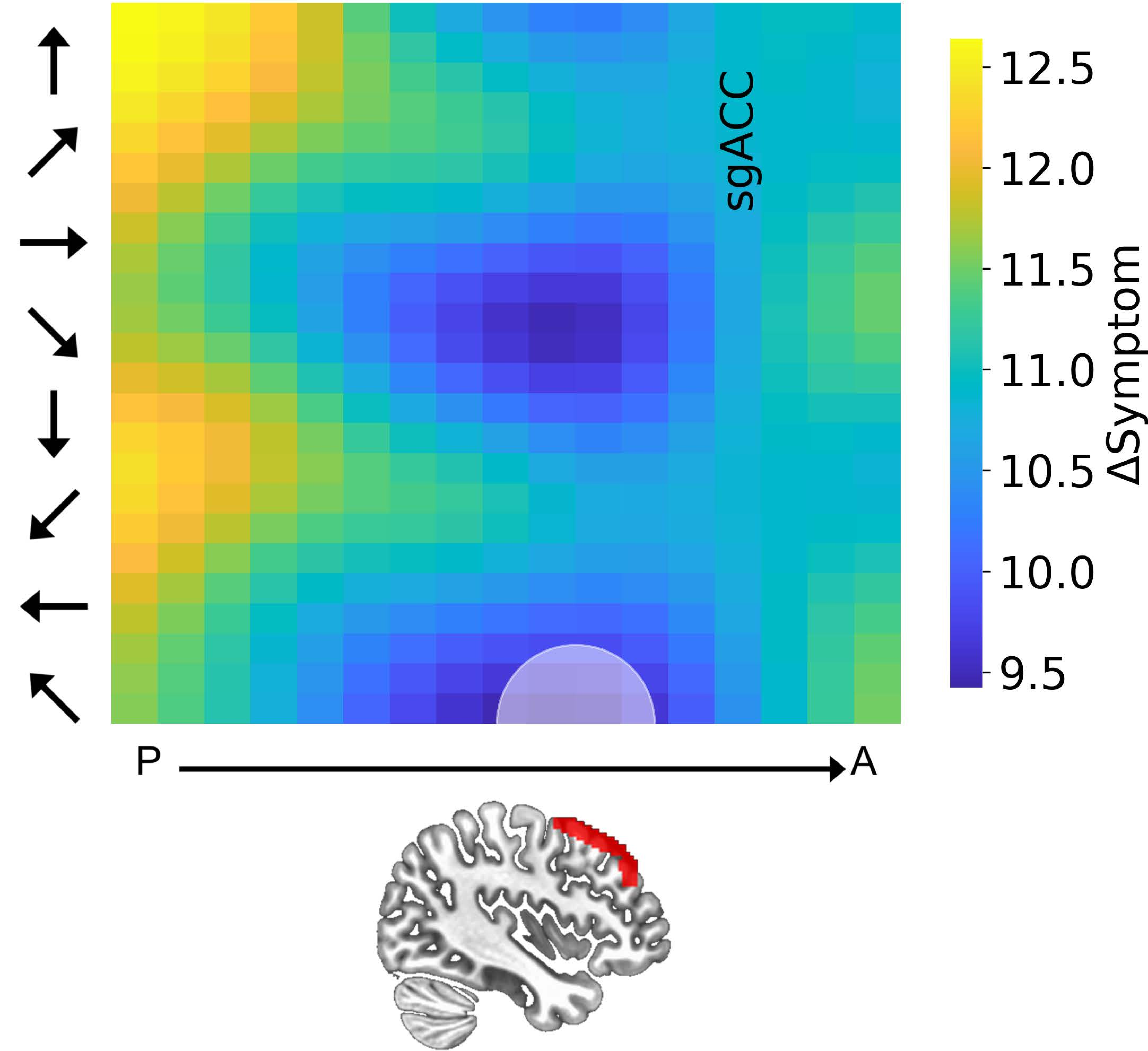

NDARLM890ELE_madrs_right_dlpfc

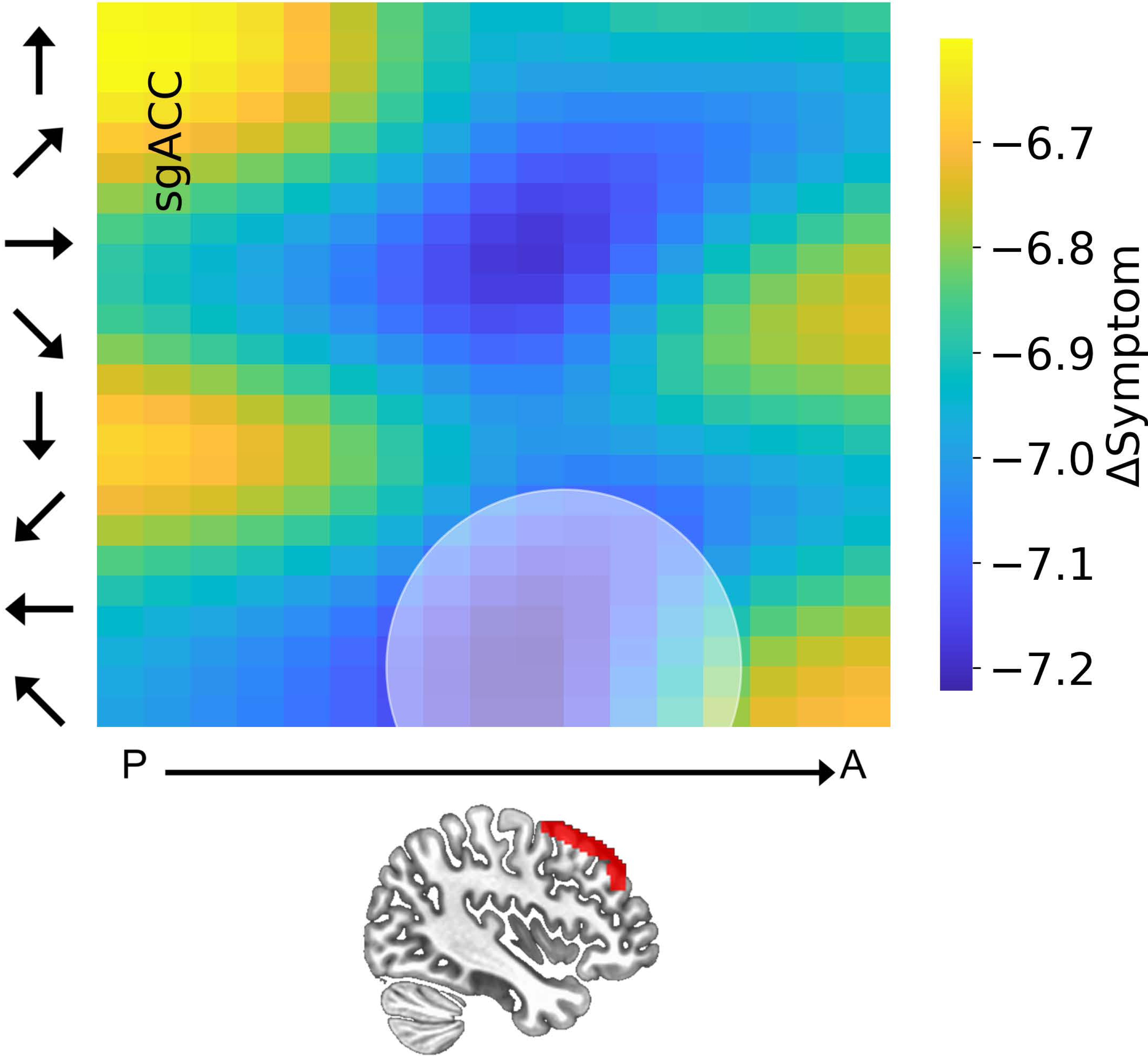

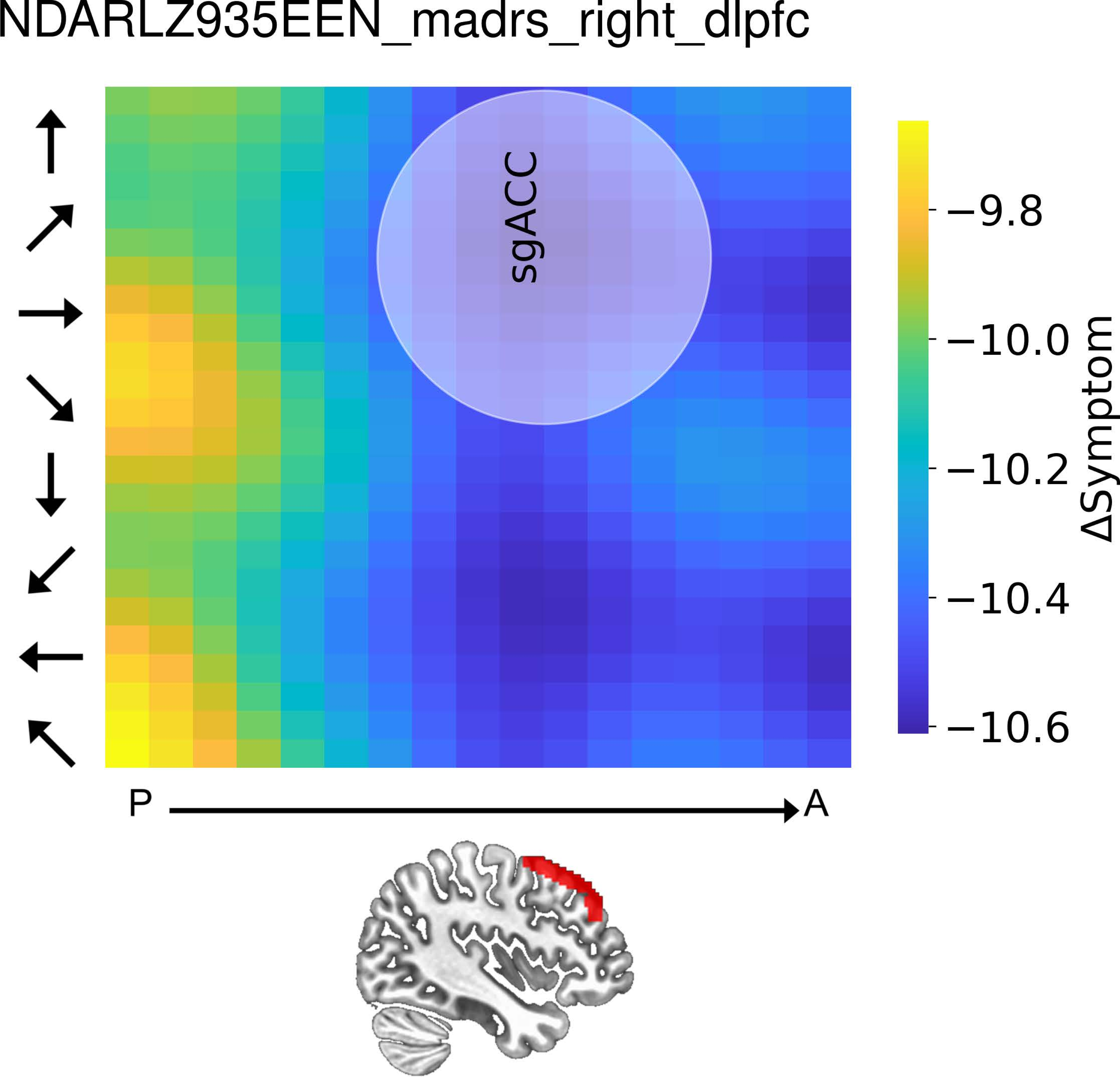

NDARLZ935EEN_madrs_right_dlpfc
sgACC
−9.8
−10.0
−10.2
−10.4
−10.6
ΔSymptom
P
A

NDARMA155ATG_madrs_right_dlpfc

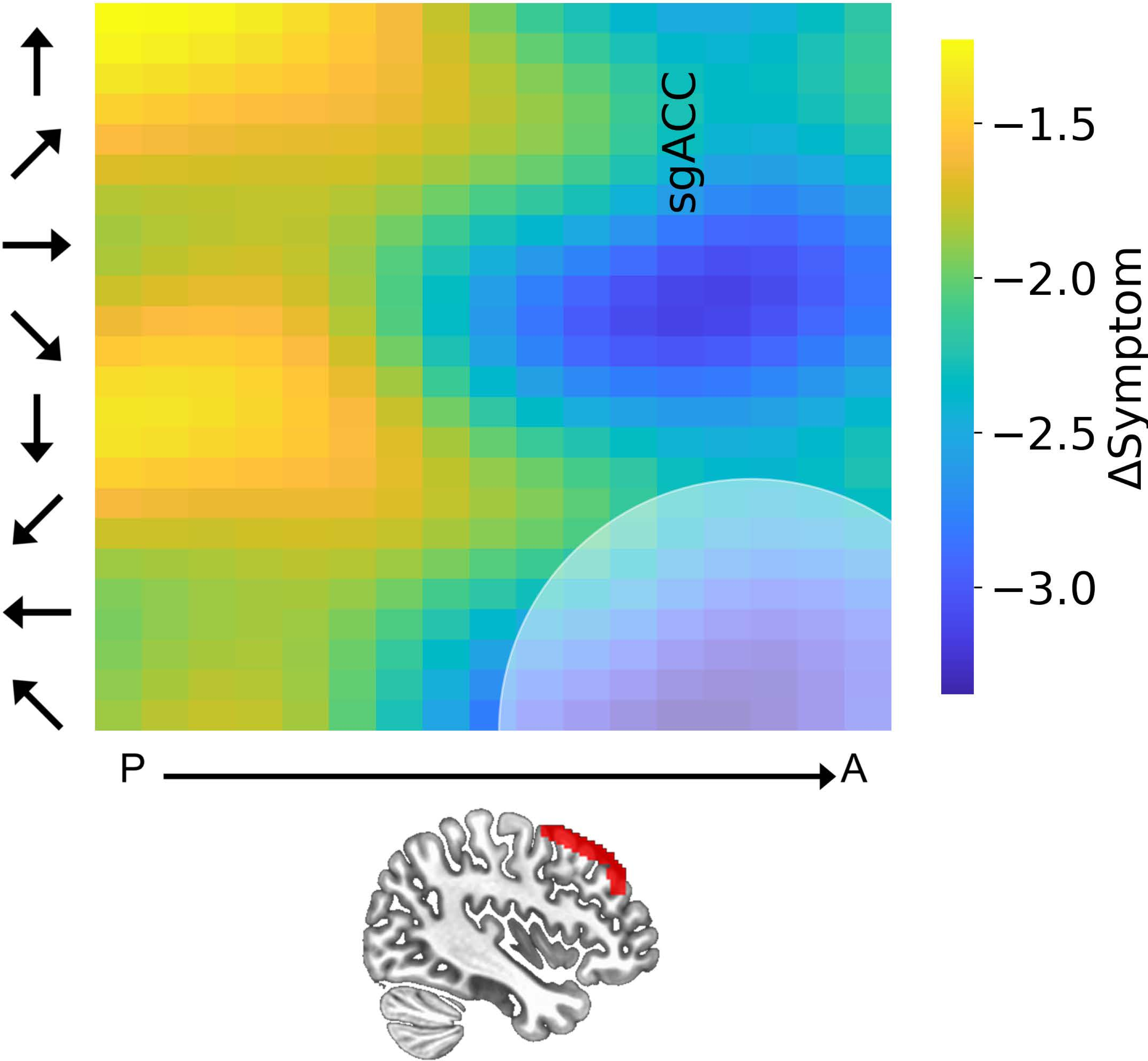

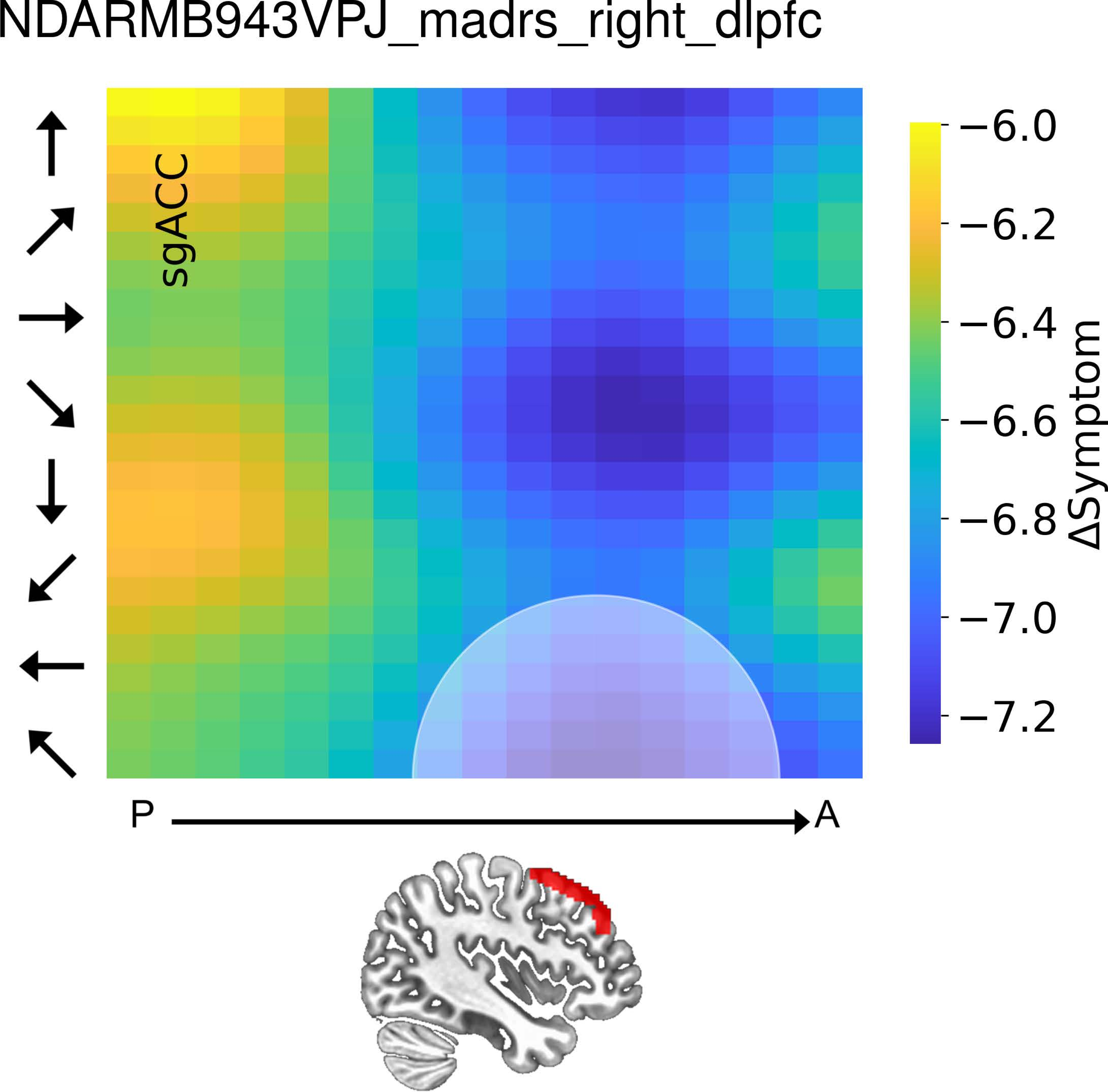

NDARMB943VPJ_madrs_right_dlpfc
sgACC
P
A
−6.0
−6.2
−6.4
−6.6
−6.8
−7.0
−7.2
ΔSymptom

NDARMD016CB8_madrs_right_dlpfc

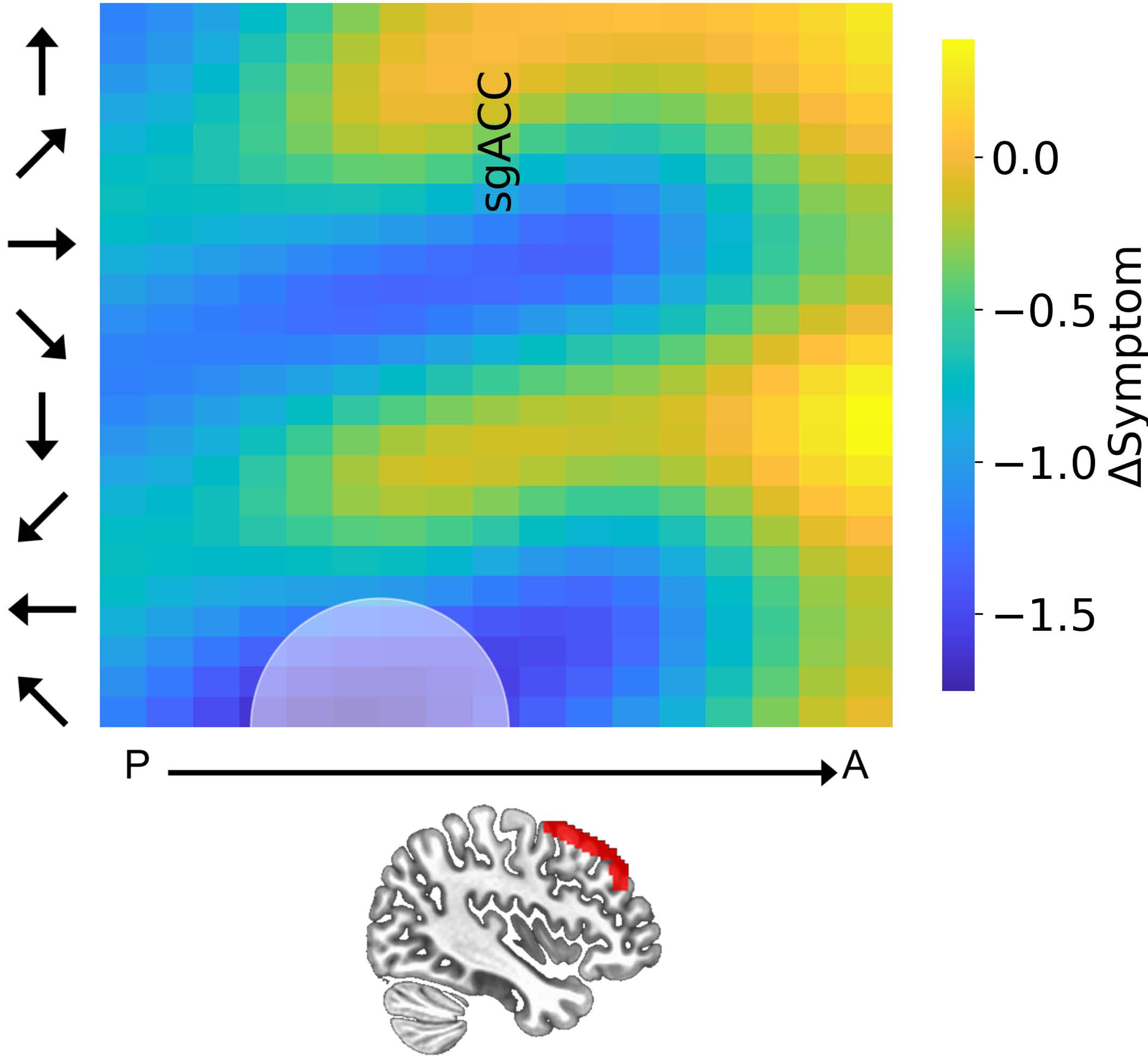

NDARMD601CTU_madrs_right_dlpfc

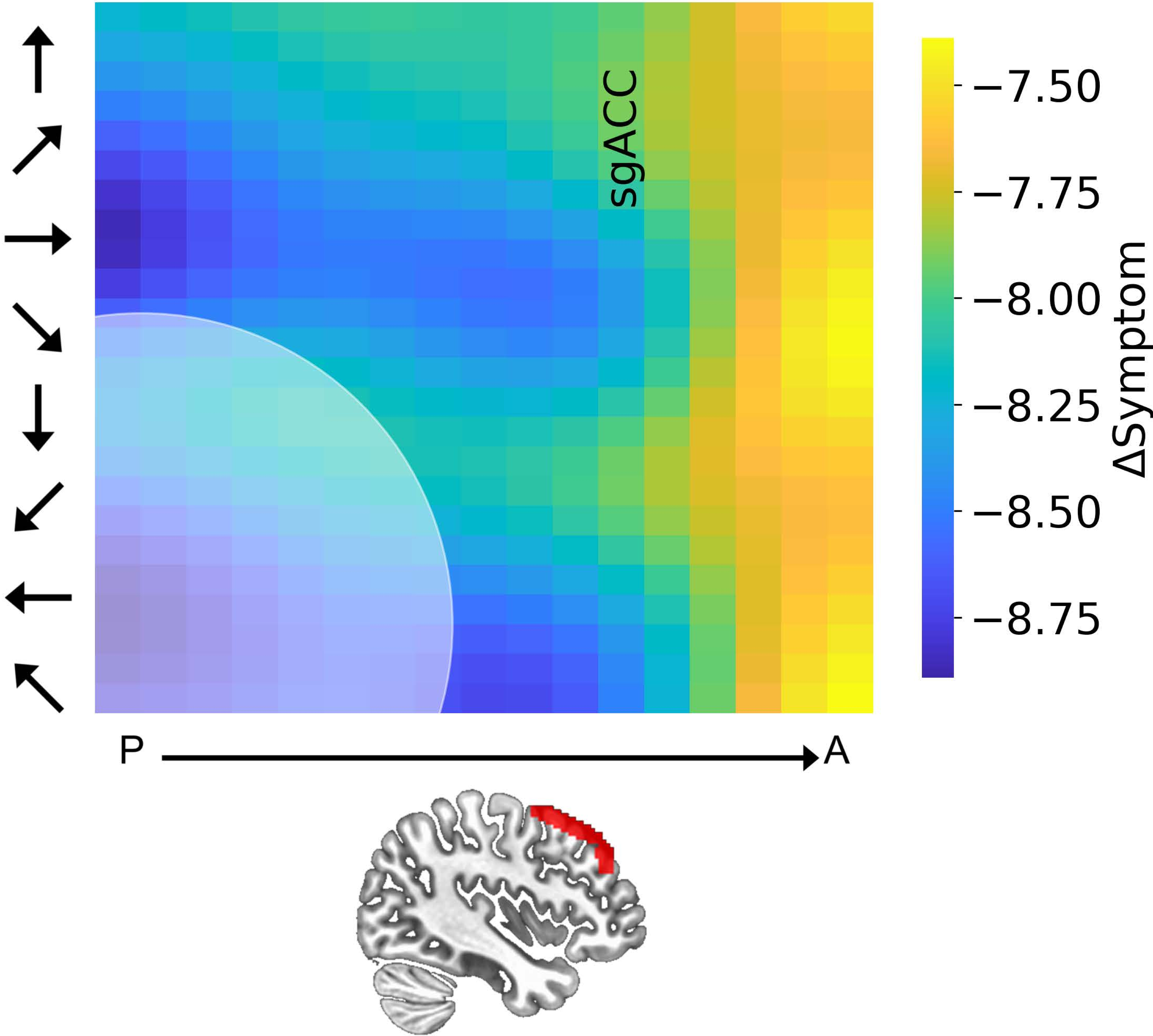

NDARME189AXP_madrs_right_dlpfc

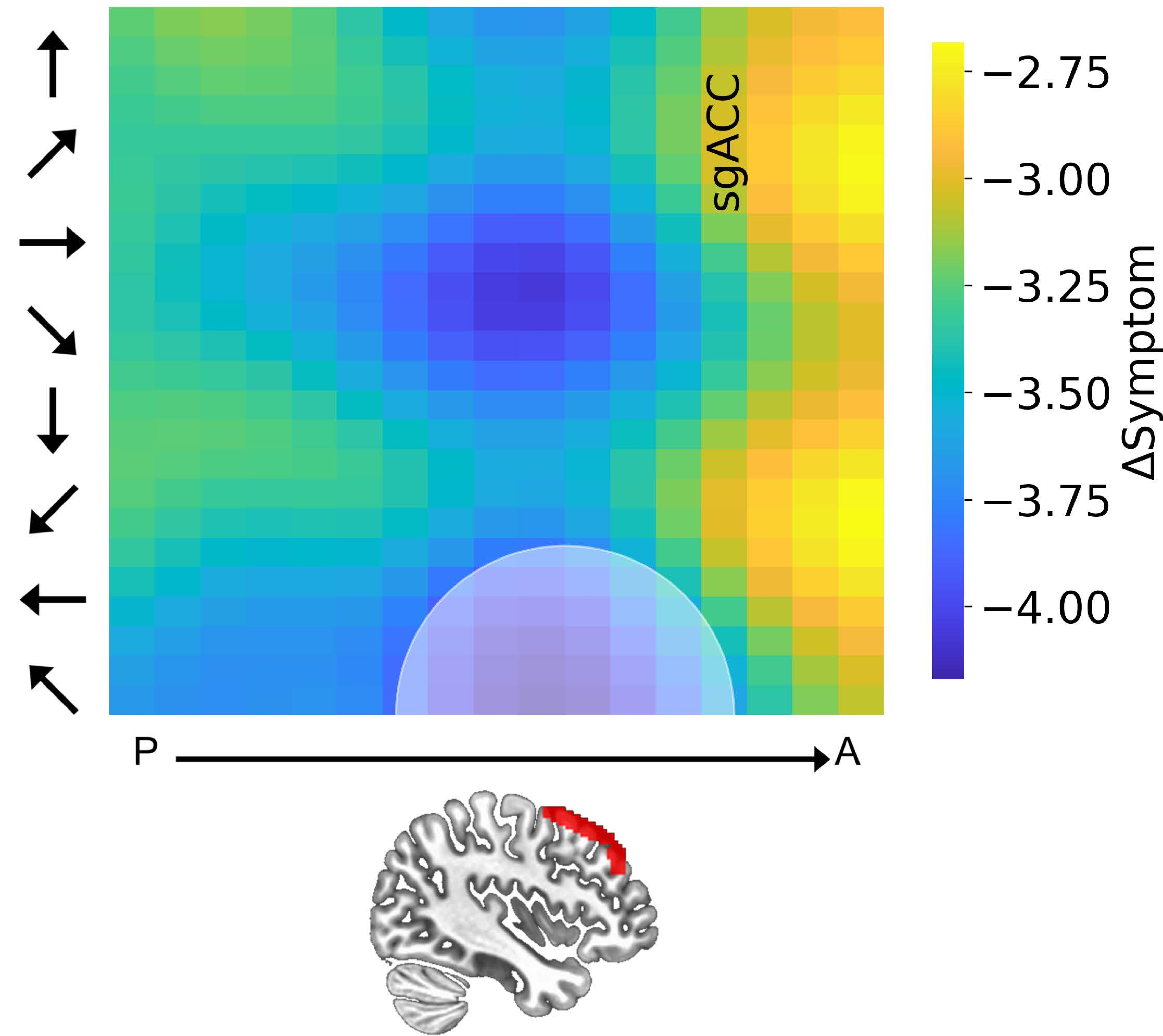

NDARME335LZJ_madrs_right_dlpfc

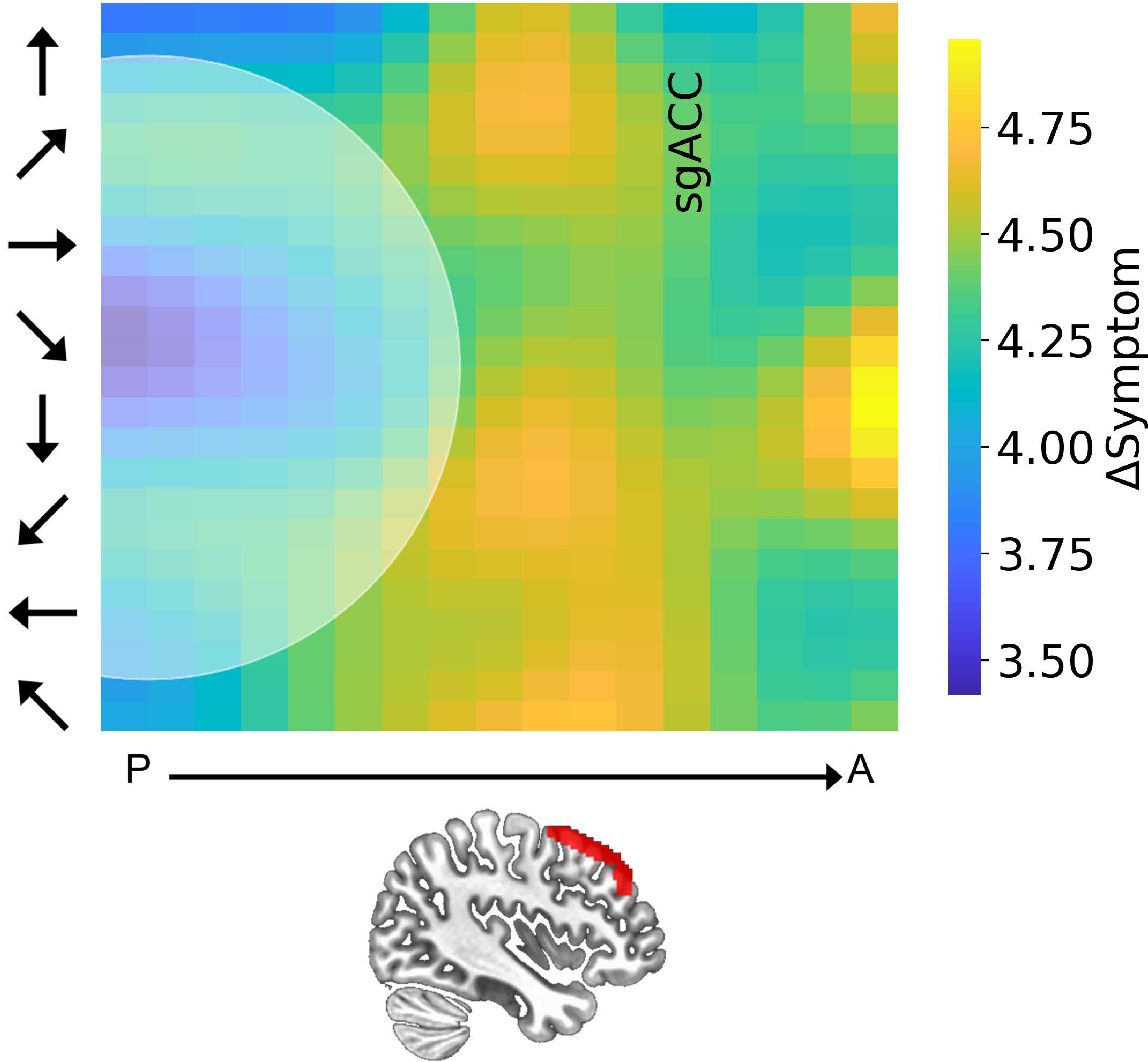

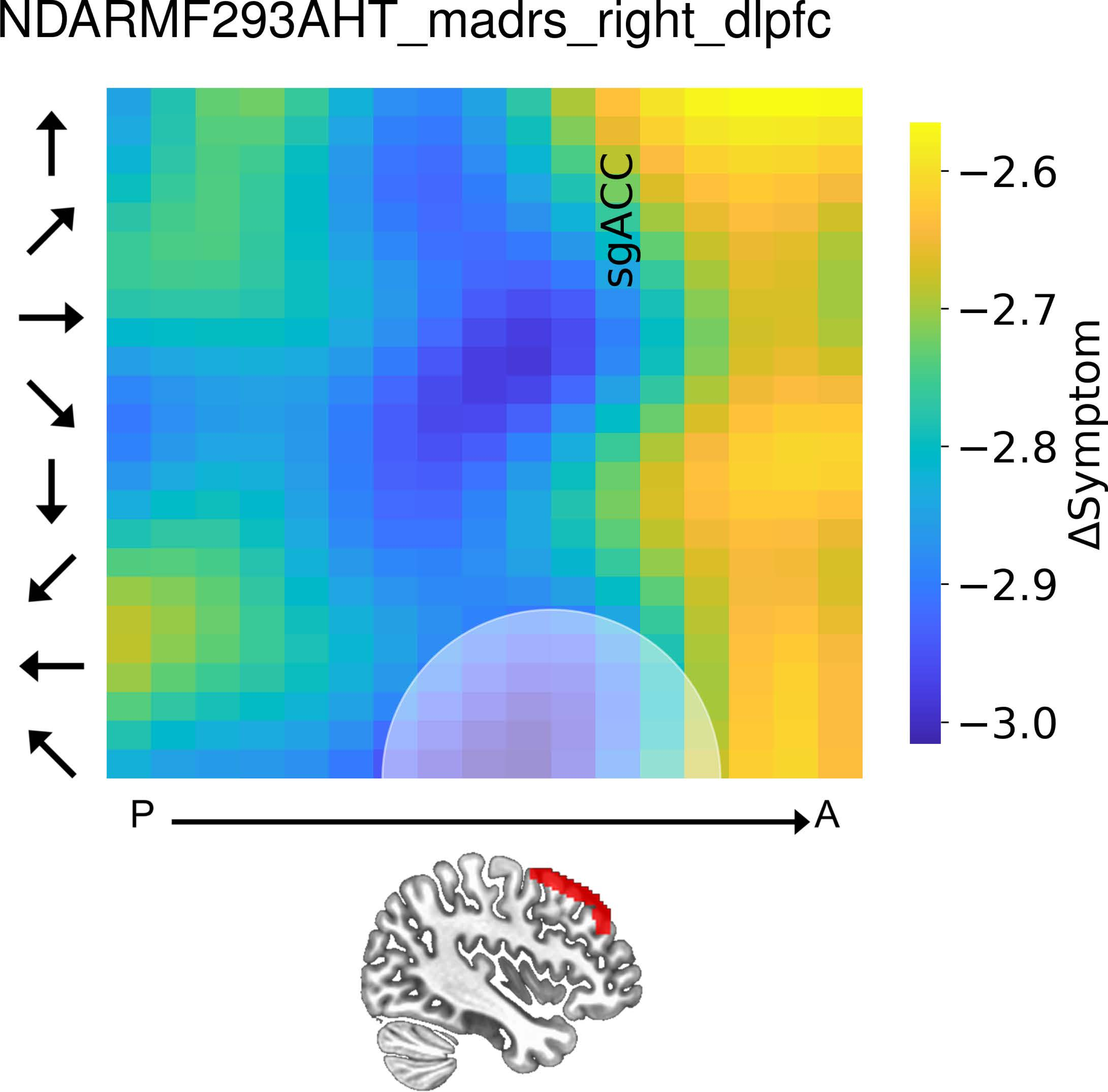

NDARMF293AHT_madrs_right_dlpfc
sgACC
P
A
−2.6
−2.7
−2.8
−2.9
−3.0
ΔSymptom

NDARMK972BFE_madrs_right_dlpfc

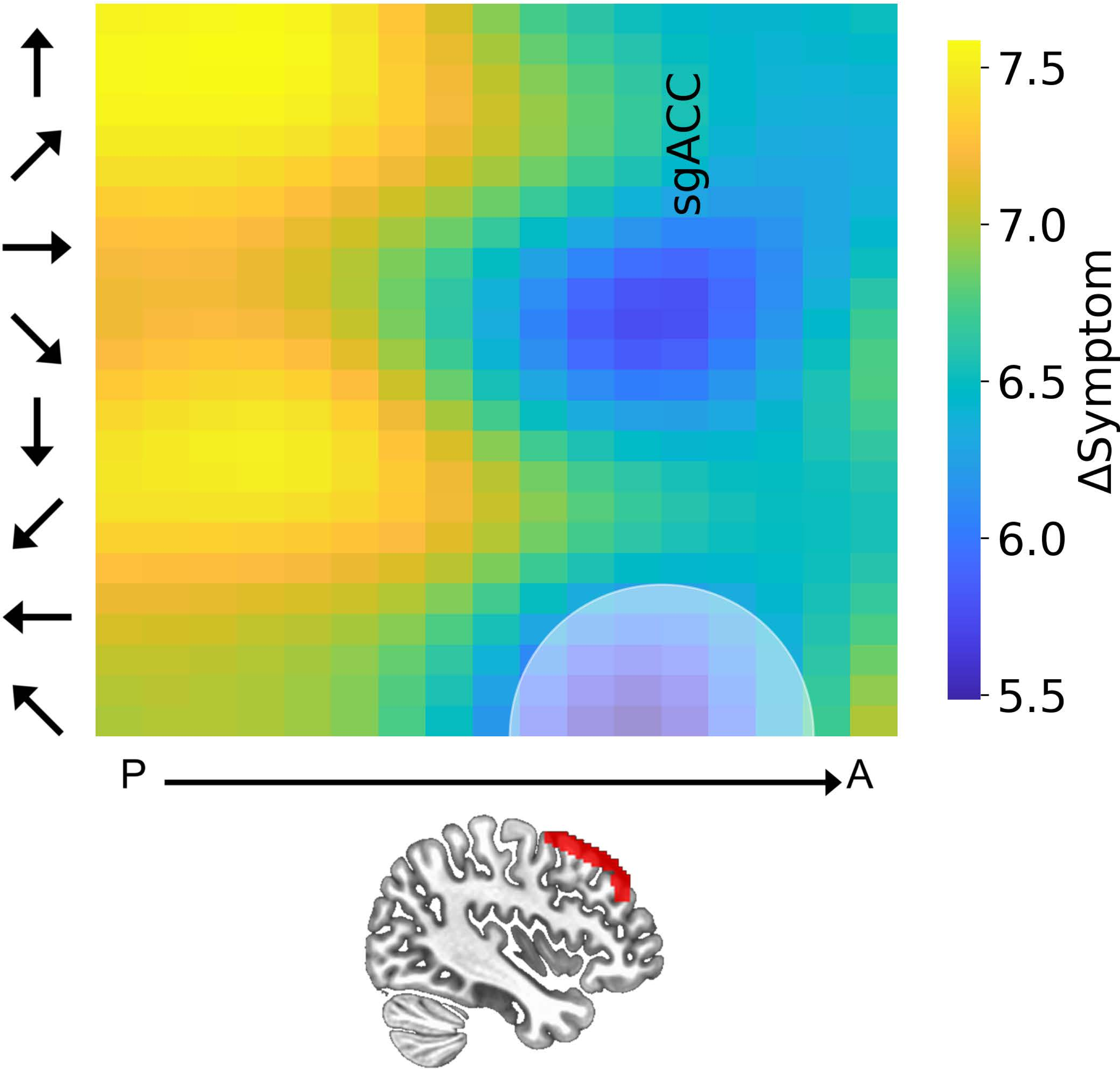

NDARNA955LXG_madrs_right_dlpfc

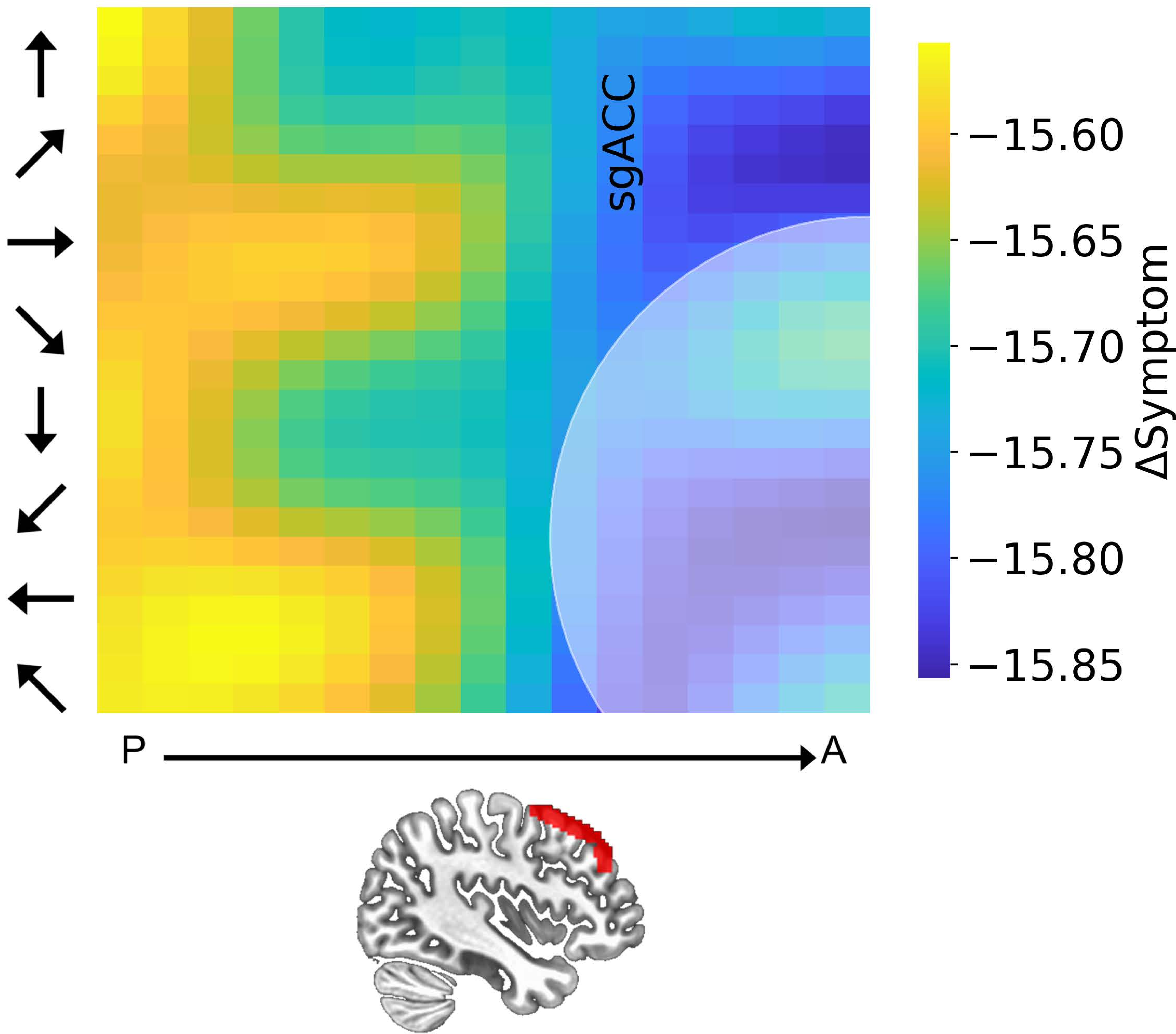

NDARNR764NAK_madrs_right_dlpfc

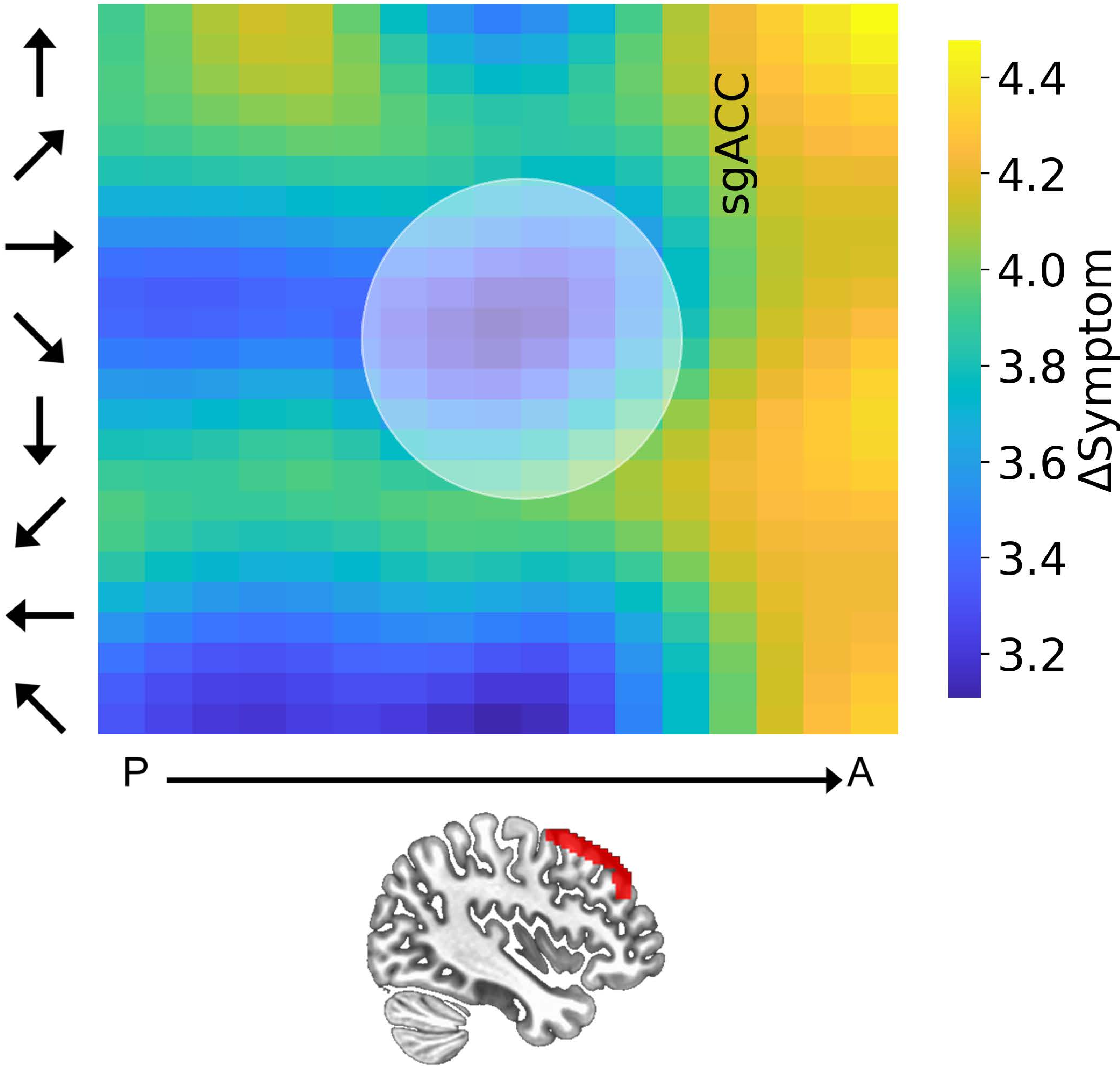

NDARNU180HL3_madrs_right_dlpfc

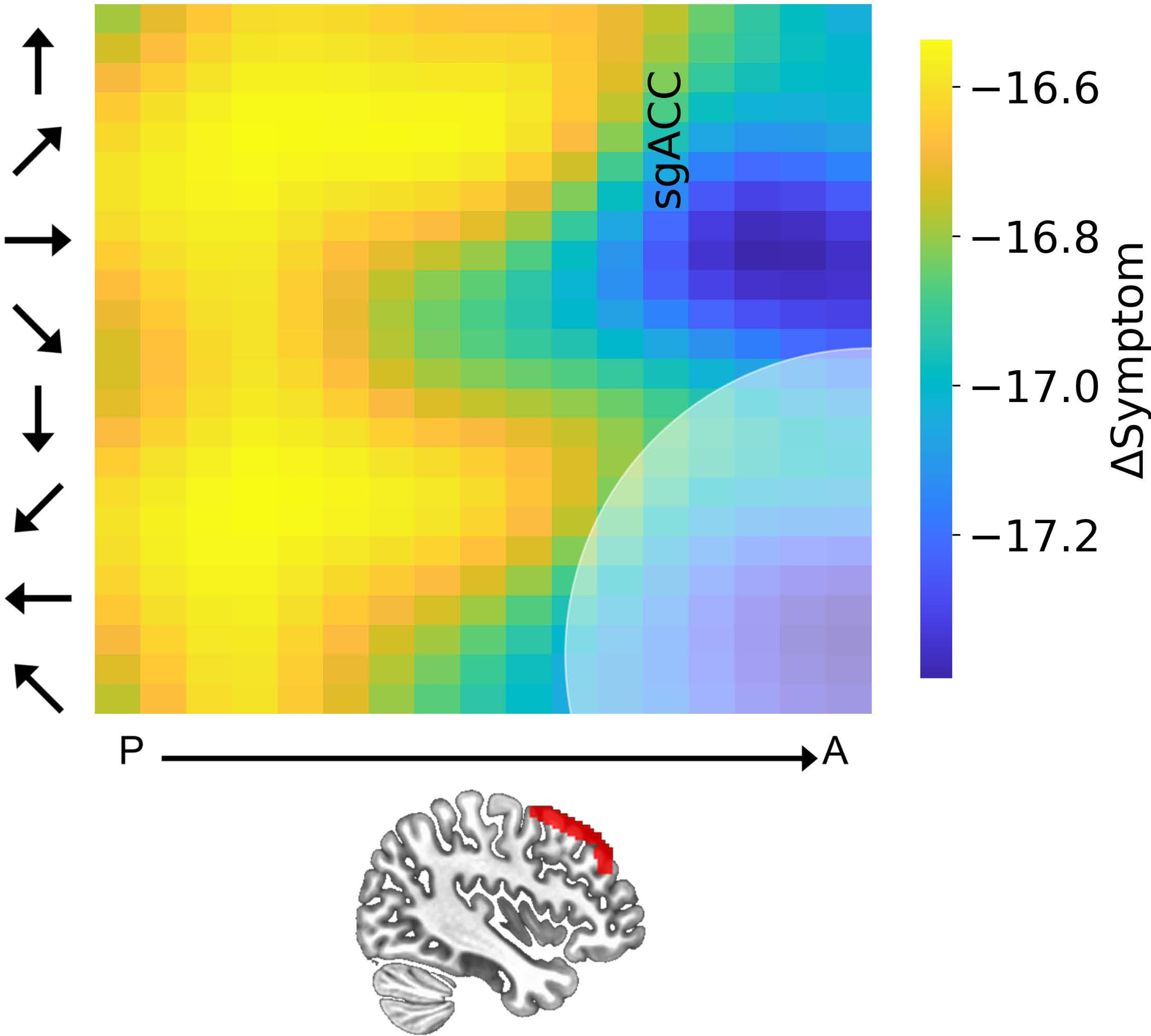

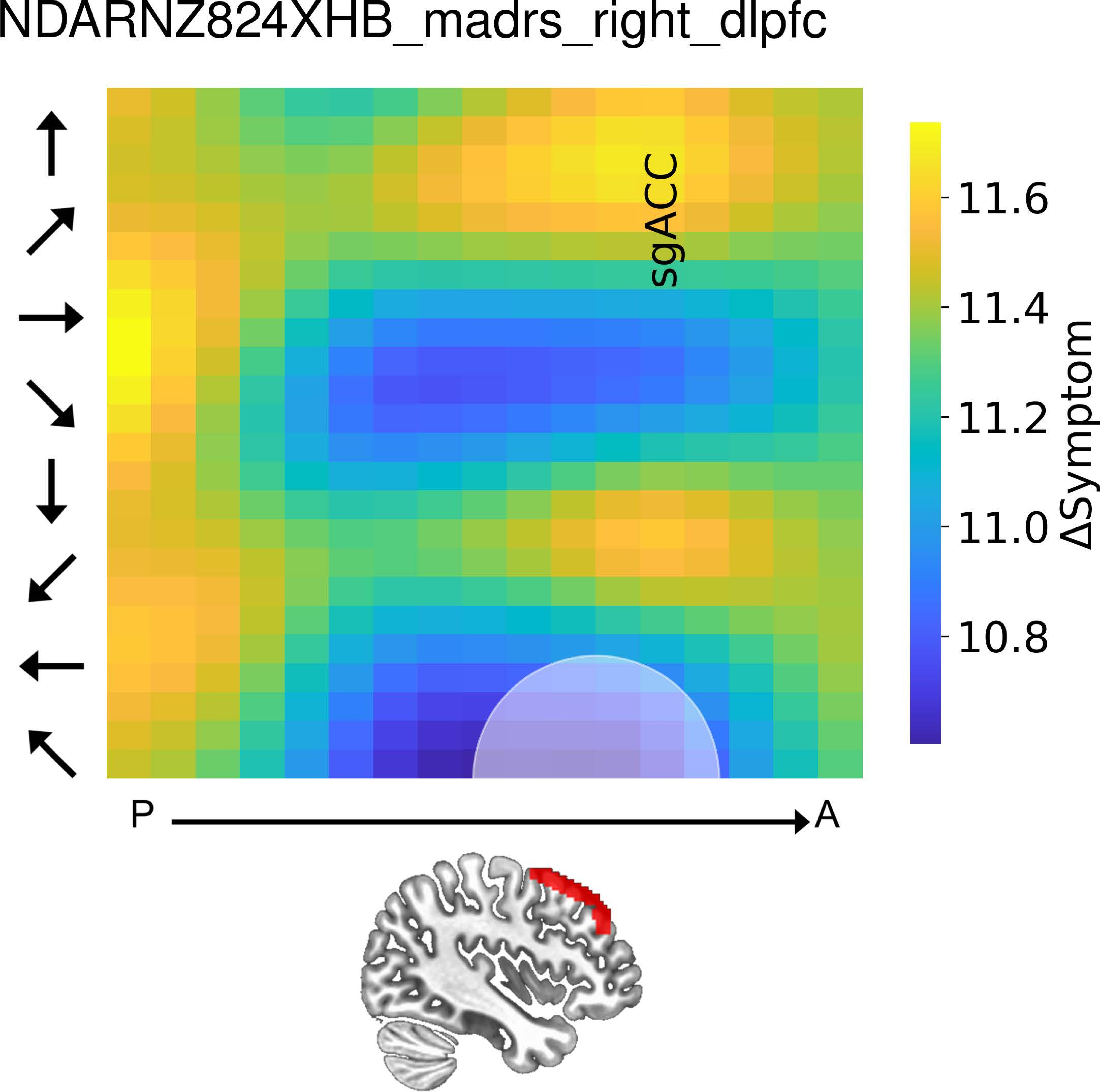
NDARNZ824XHB_madrs_right_dlpfc
sgACC
11.6
11.4
11.2
11.0
10.8
ΔSymptom
P
A

NDARPA762UGZ_madrs_right_dlpfc

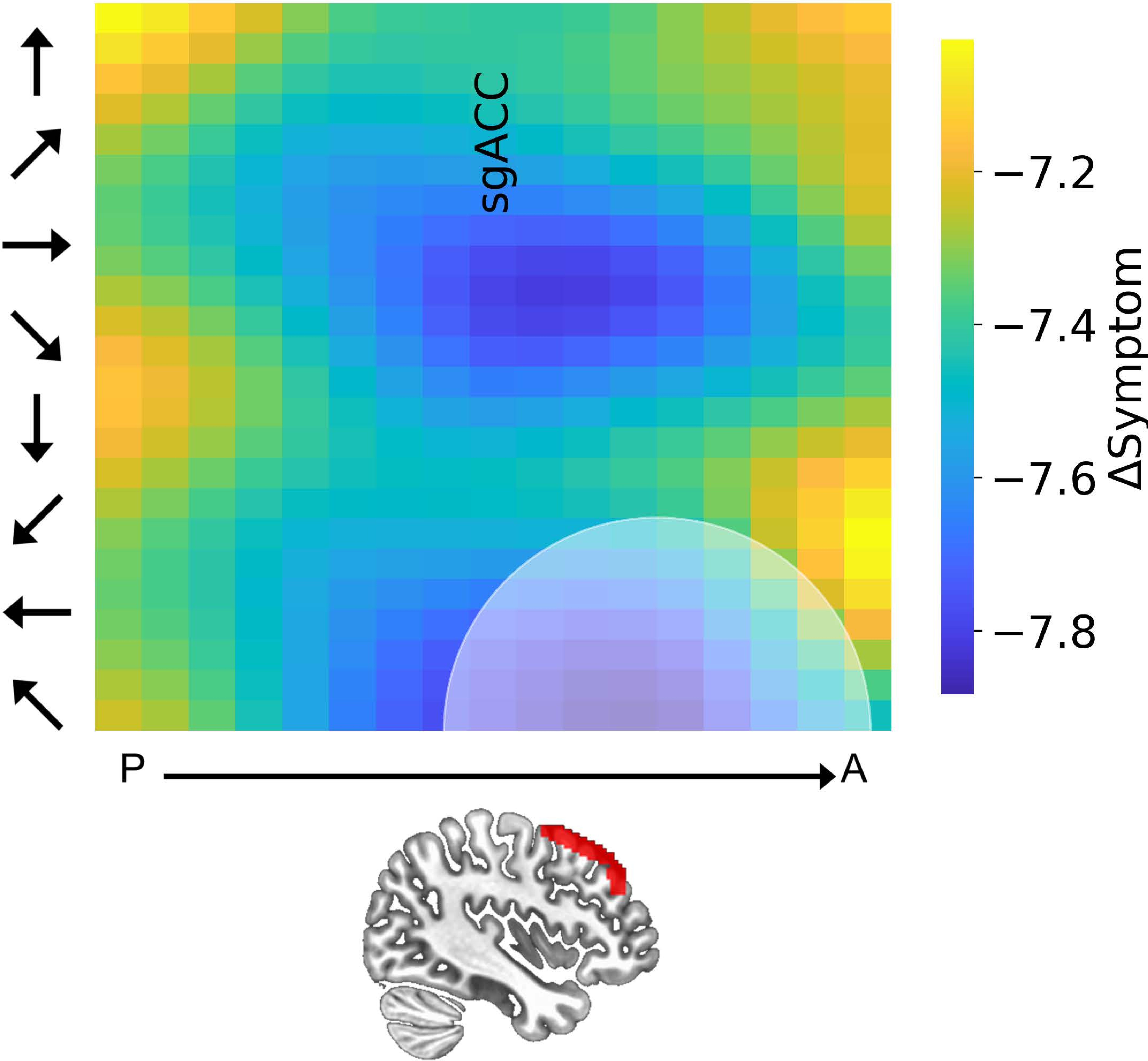

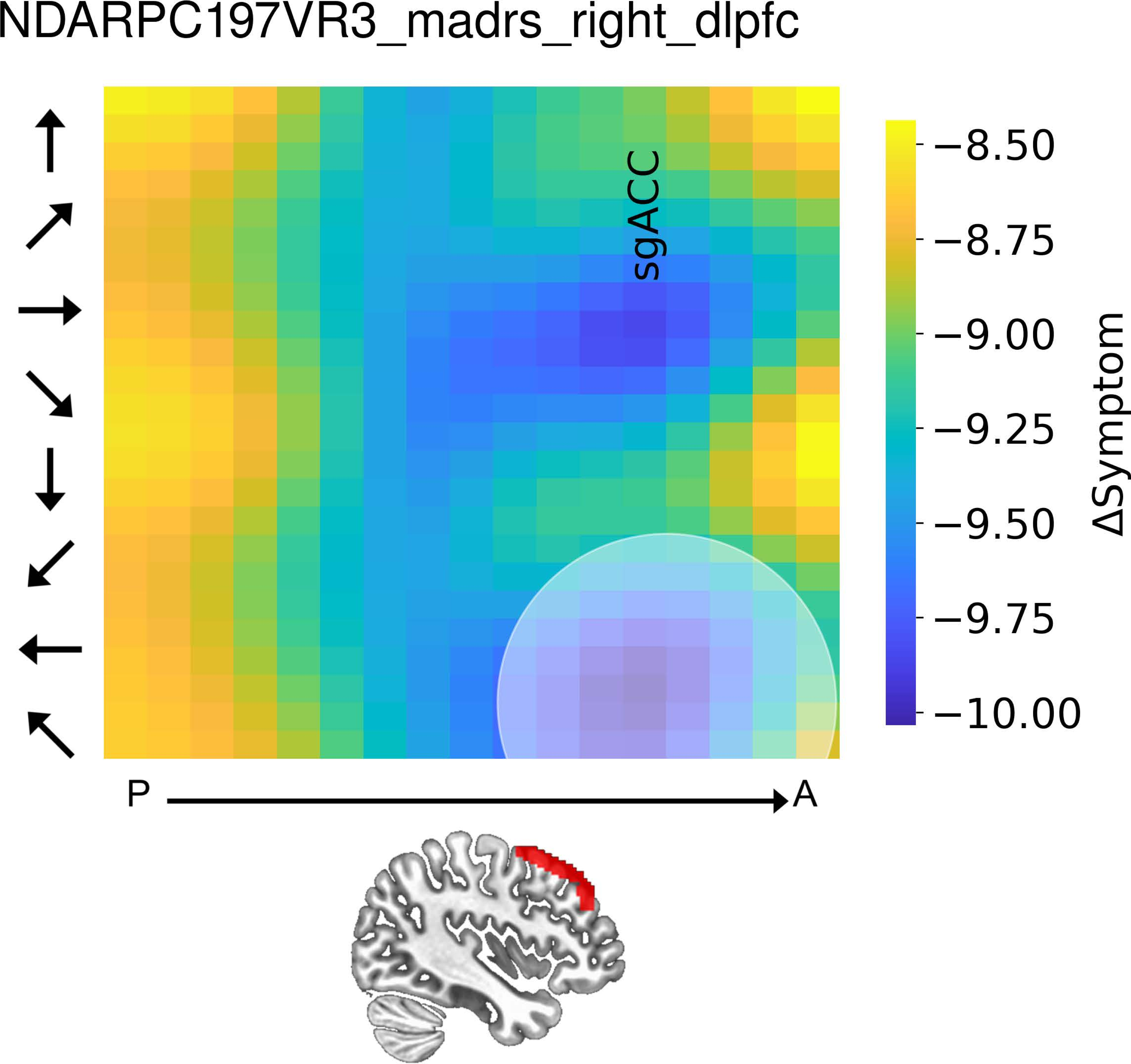

NDARPC197VR3_madrs_right_dlpfc
sgACC
−8.50
−8.75
−9.00
−9.25
−9.50
−9.75
−10.00
ΔSymptom
P
A

NDARPF499HVH_madrs_right_dlpfc

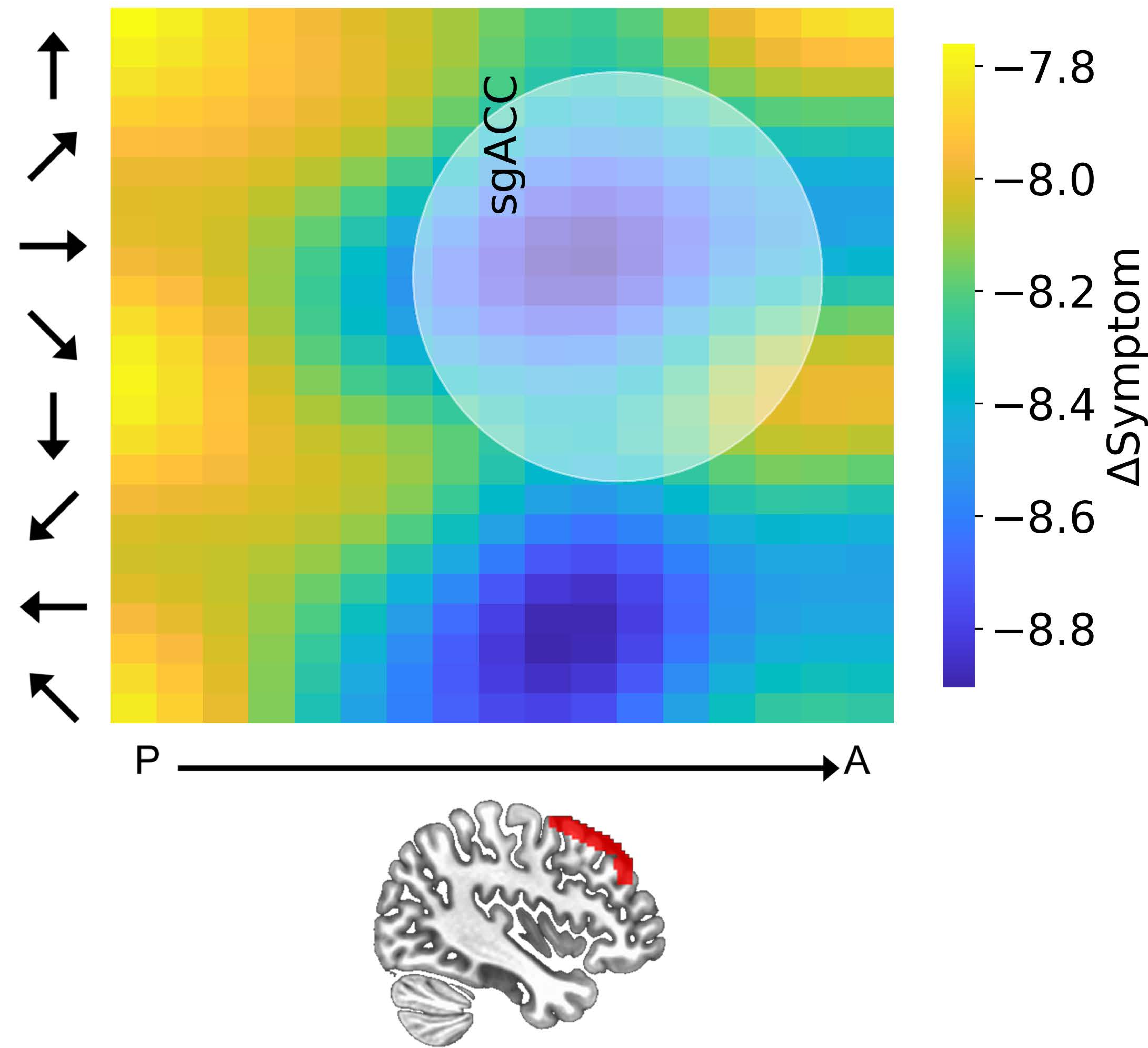

NDARPK811MVW_madrs_right_dlpfc

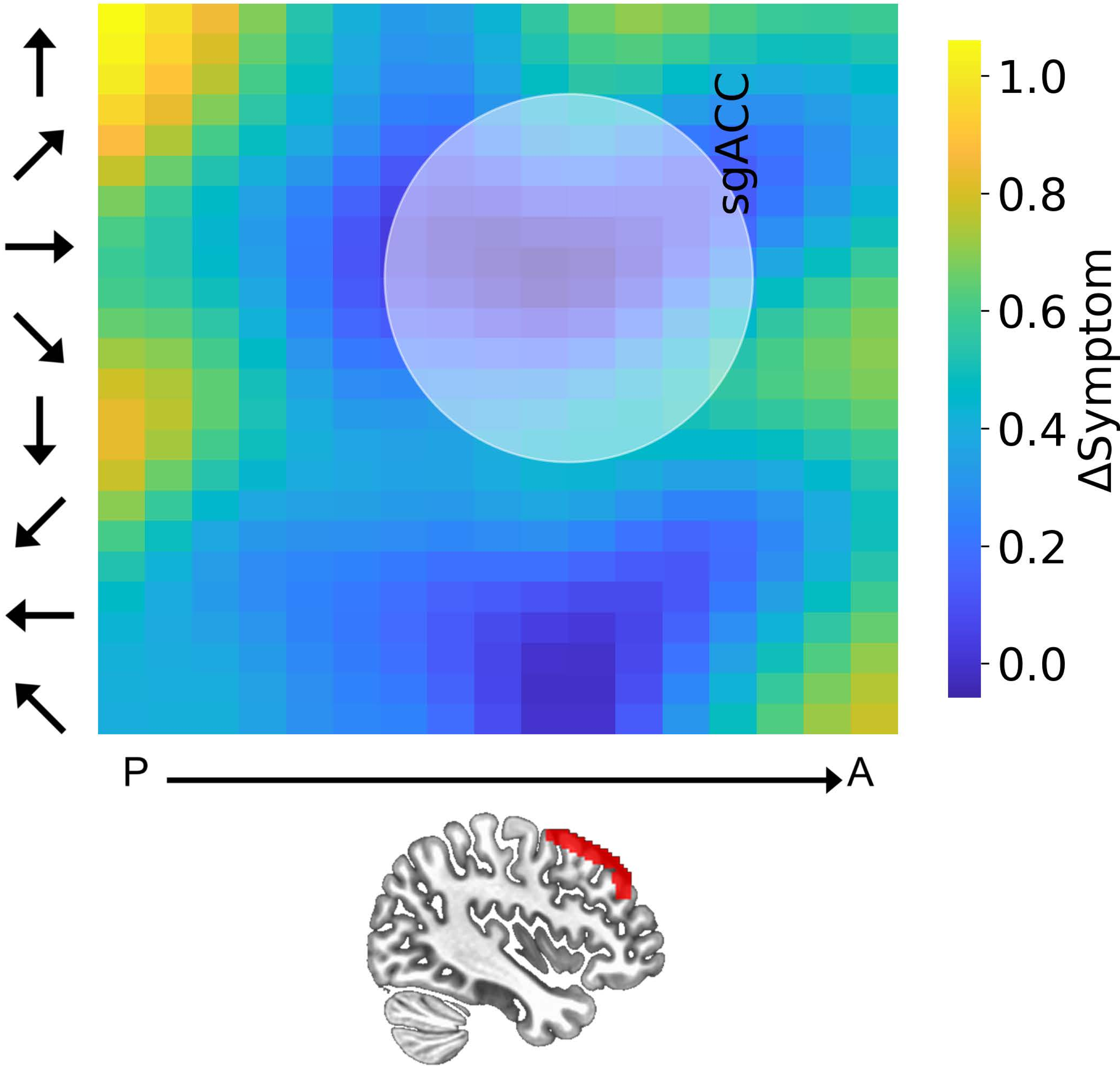

NDARPT040EGR_madrs_right_dlpfc

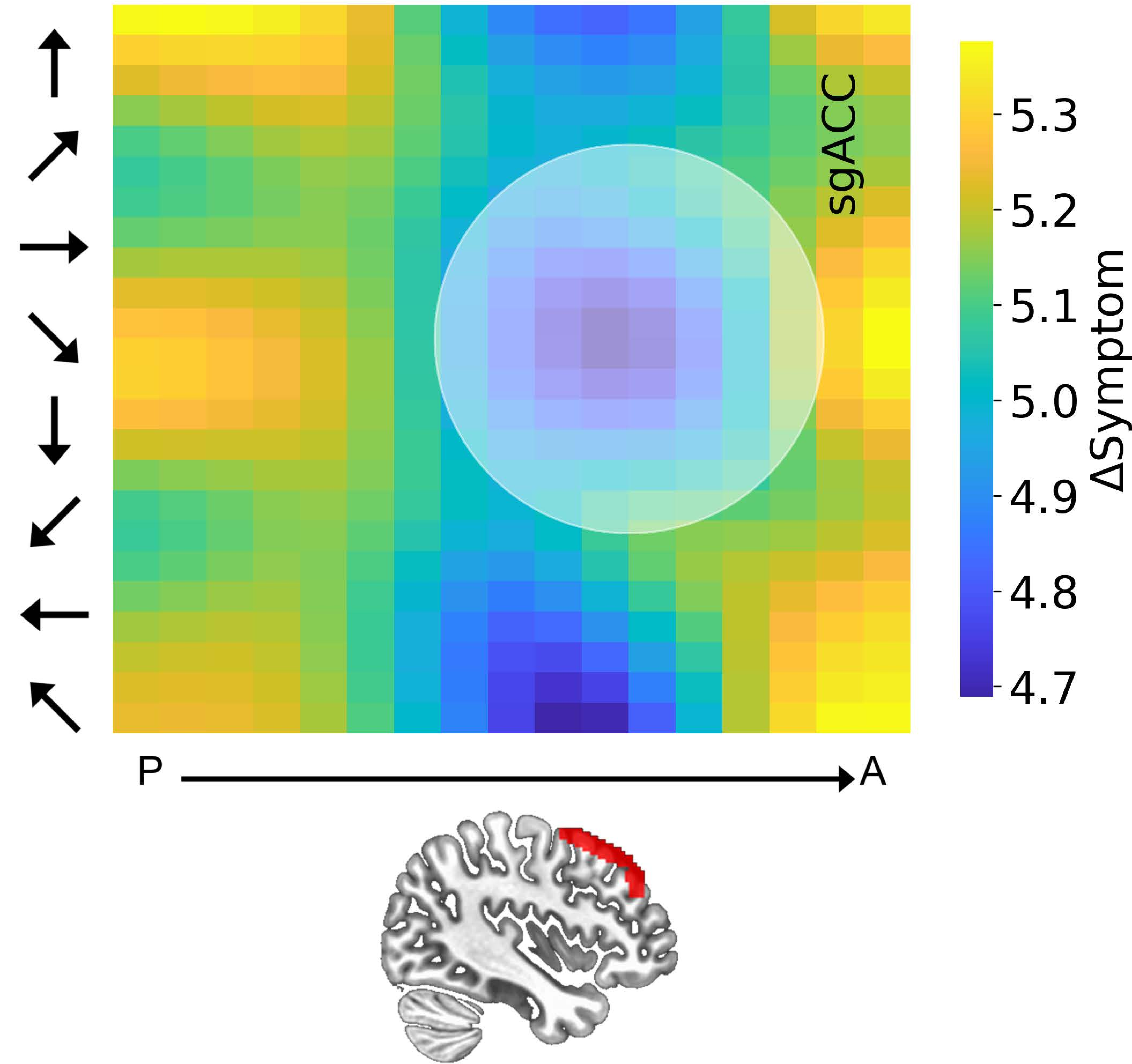

NDARPW706YNA_madrs_right_dlpfc

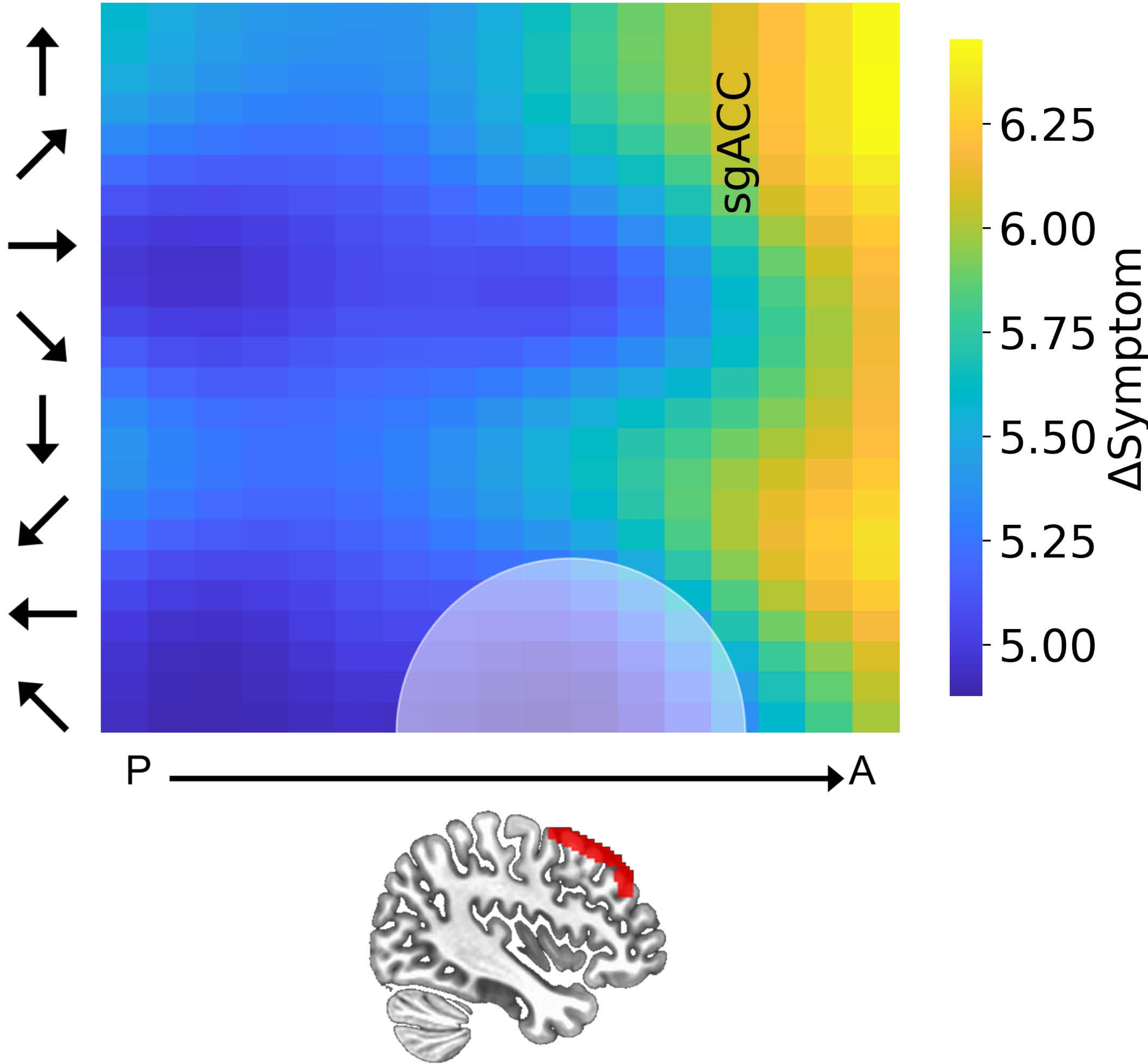

NDARPZ537YY0_madrs_right_dlpfc

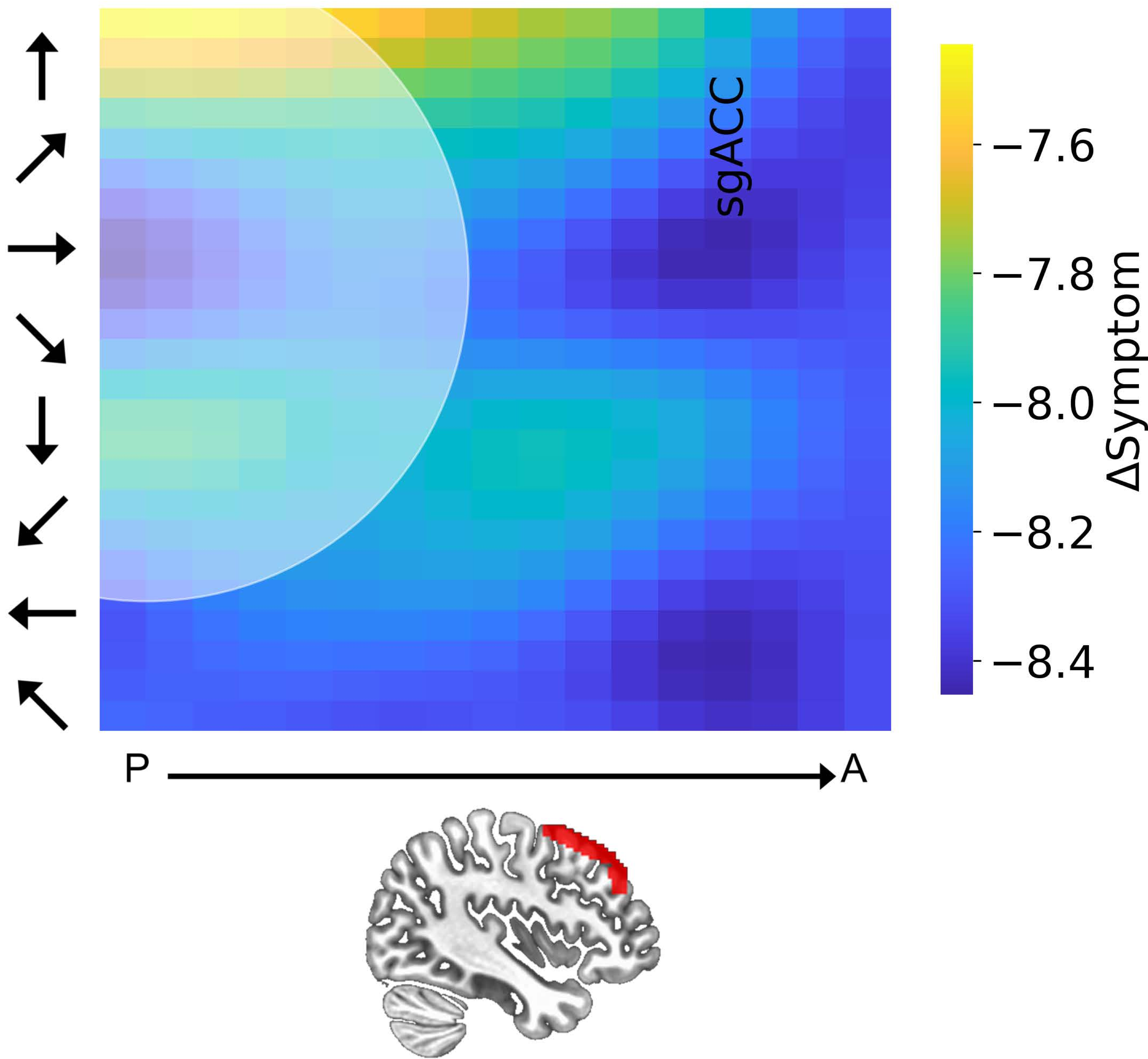

NDARRD555DDH_madrs_right_dlpfc

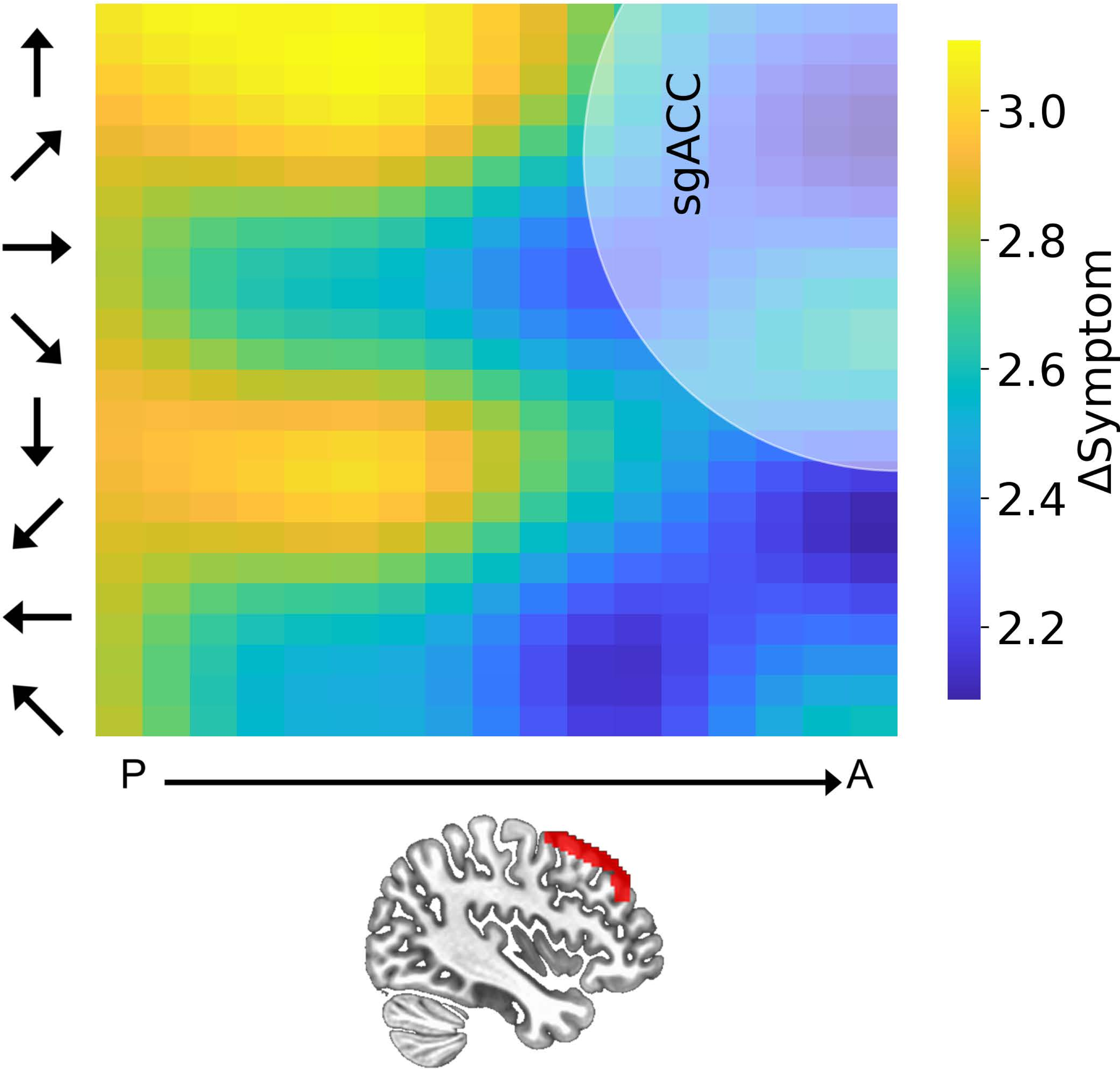

NDARRF879TA3_madrs_right_dlpfc

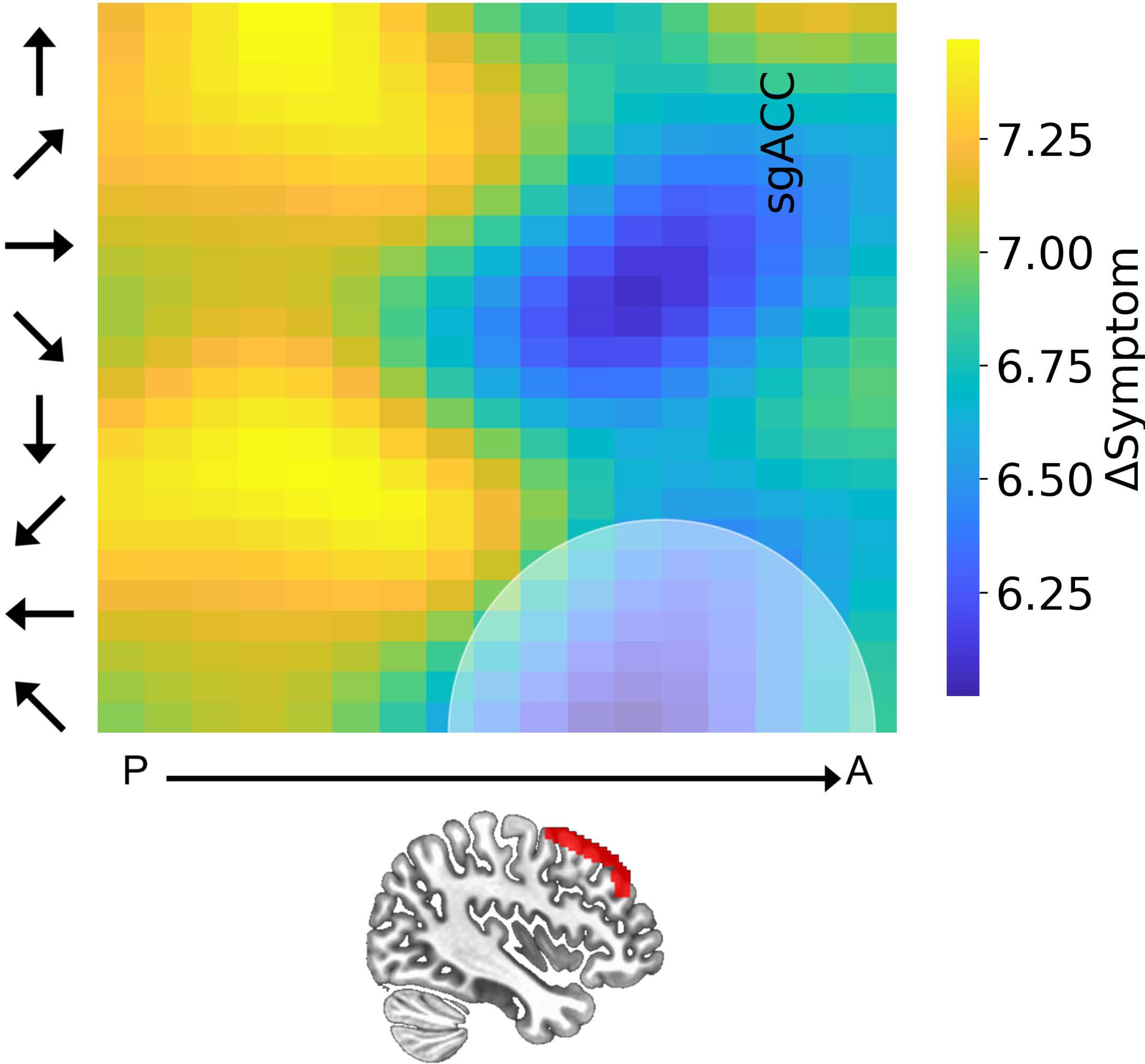

NDARRG943WGE_madrs_right_dlpfc

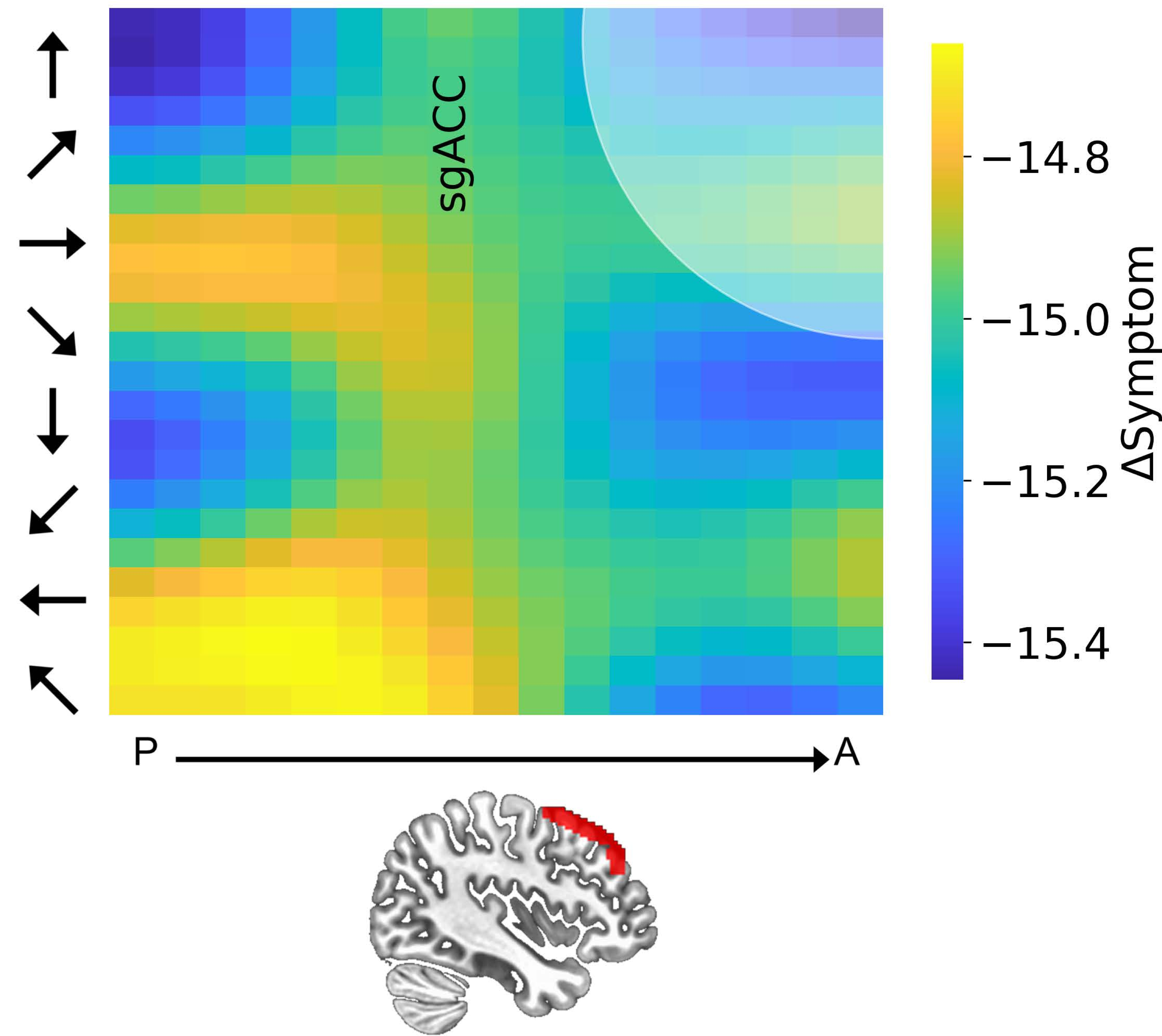

NDARRM328EP8_madrs_right_dlpfc

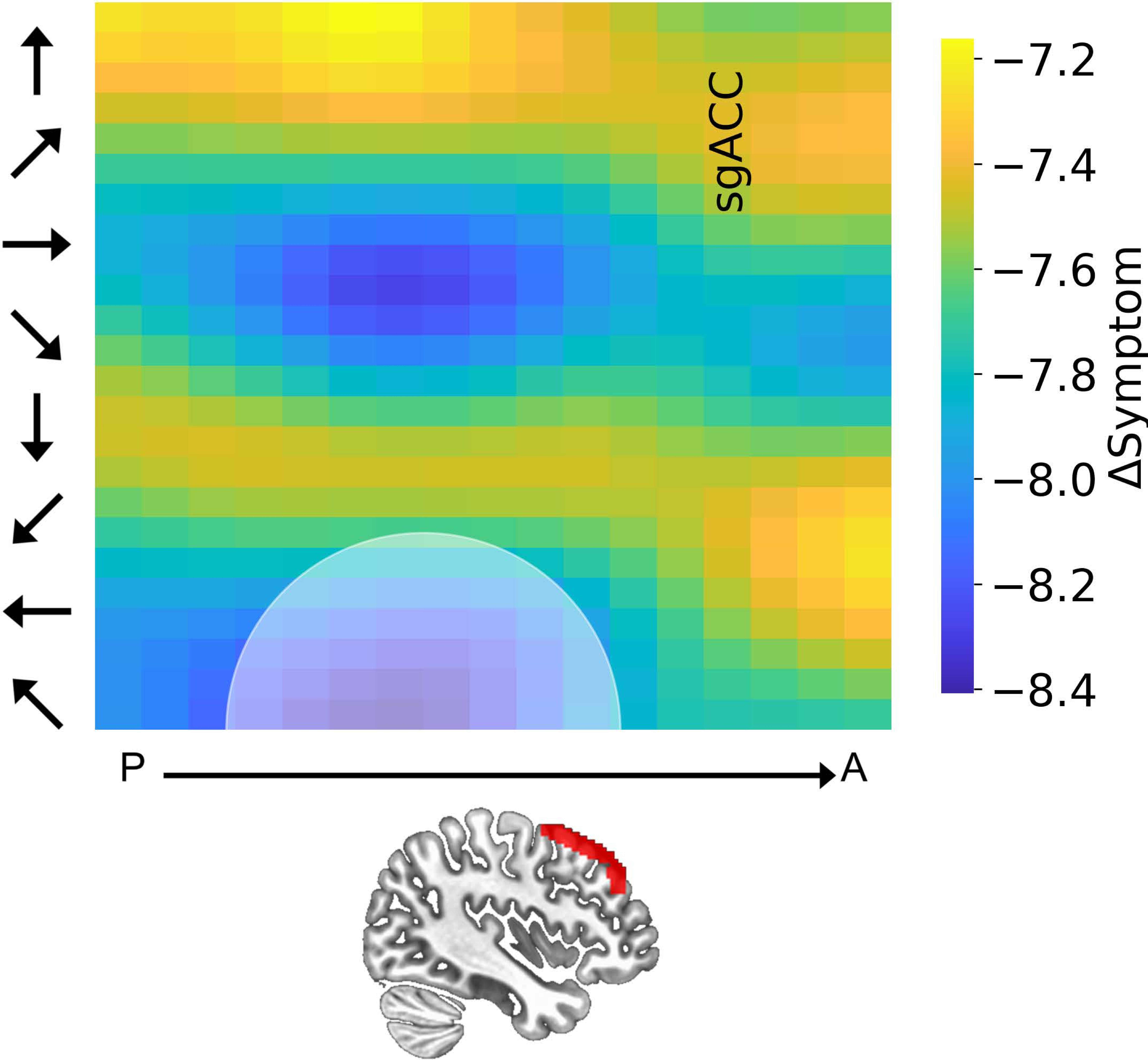

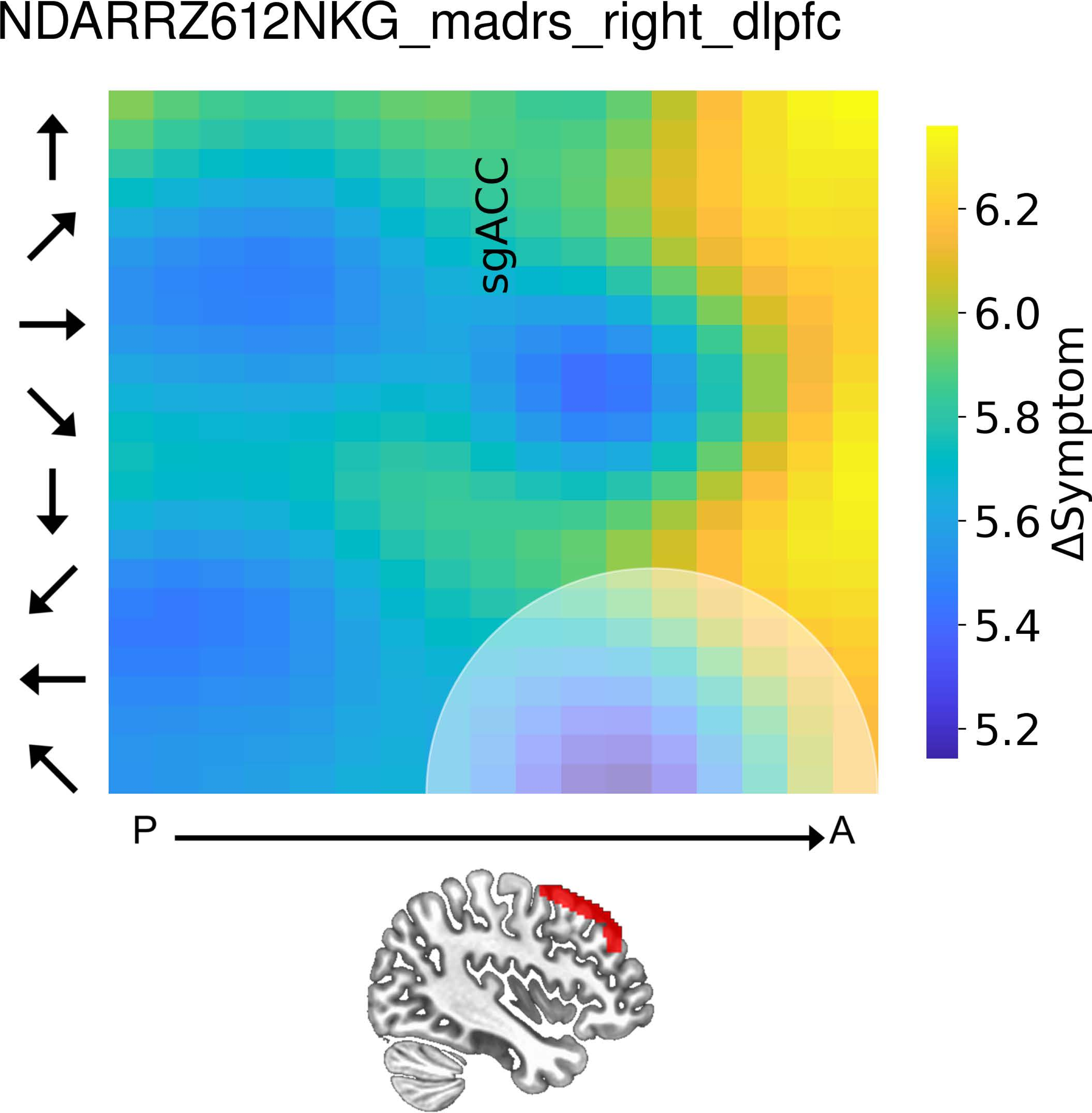

NDARRZ612NKG_madrs_right_dlpfc
sgACC
6.2
6.0
5.8
5.6
5.4
5.2
ΔSymptom
P
A

NDARTH509MNV_madrs_right_dlpfc

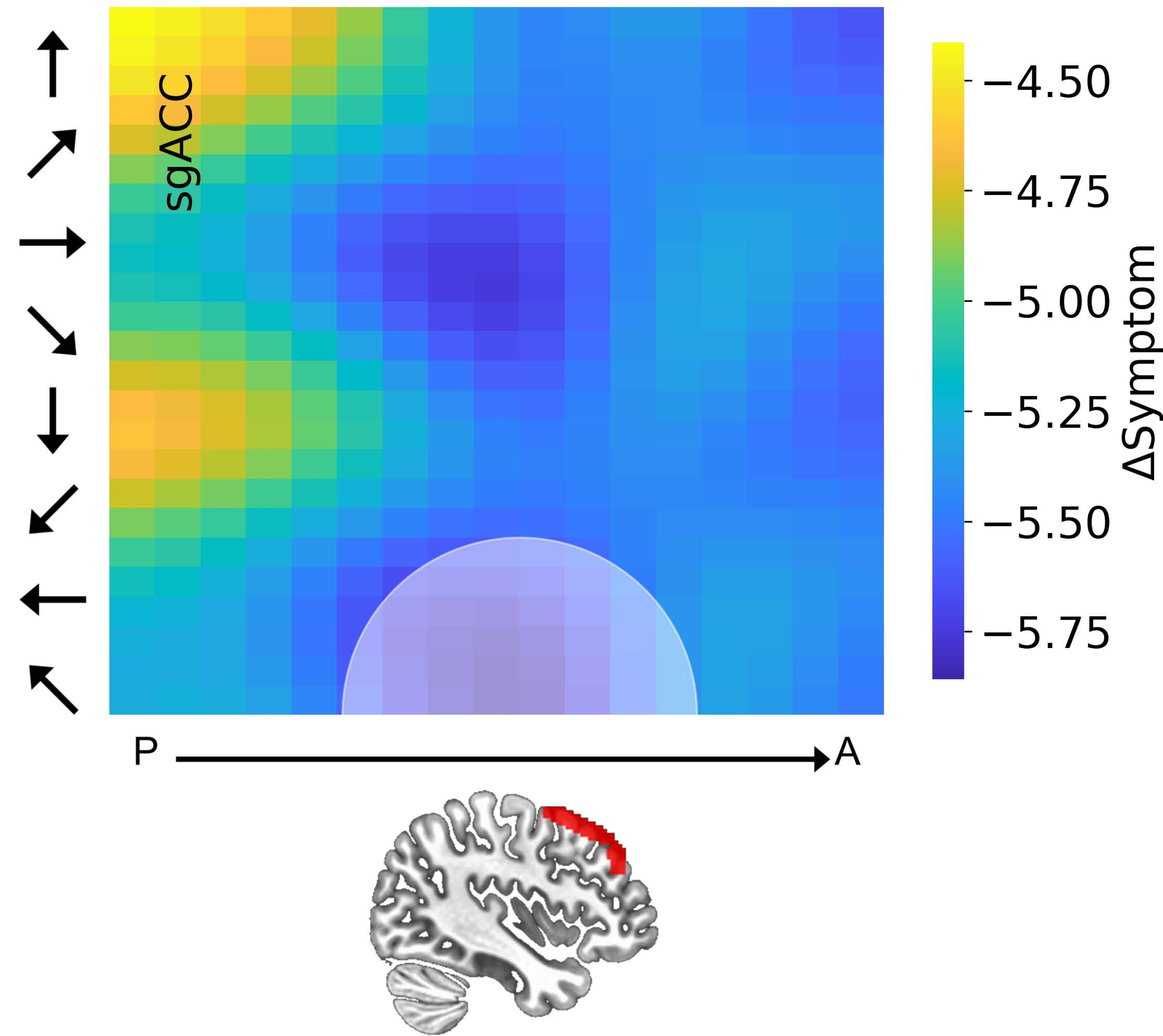

NDARTT796GJK_madrs_right_dlpfc

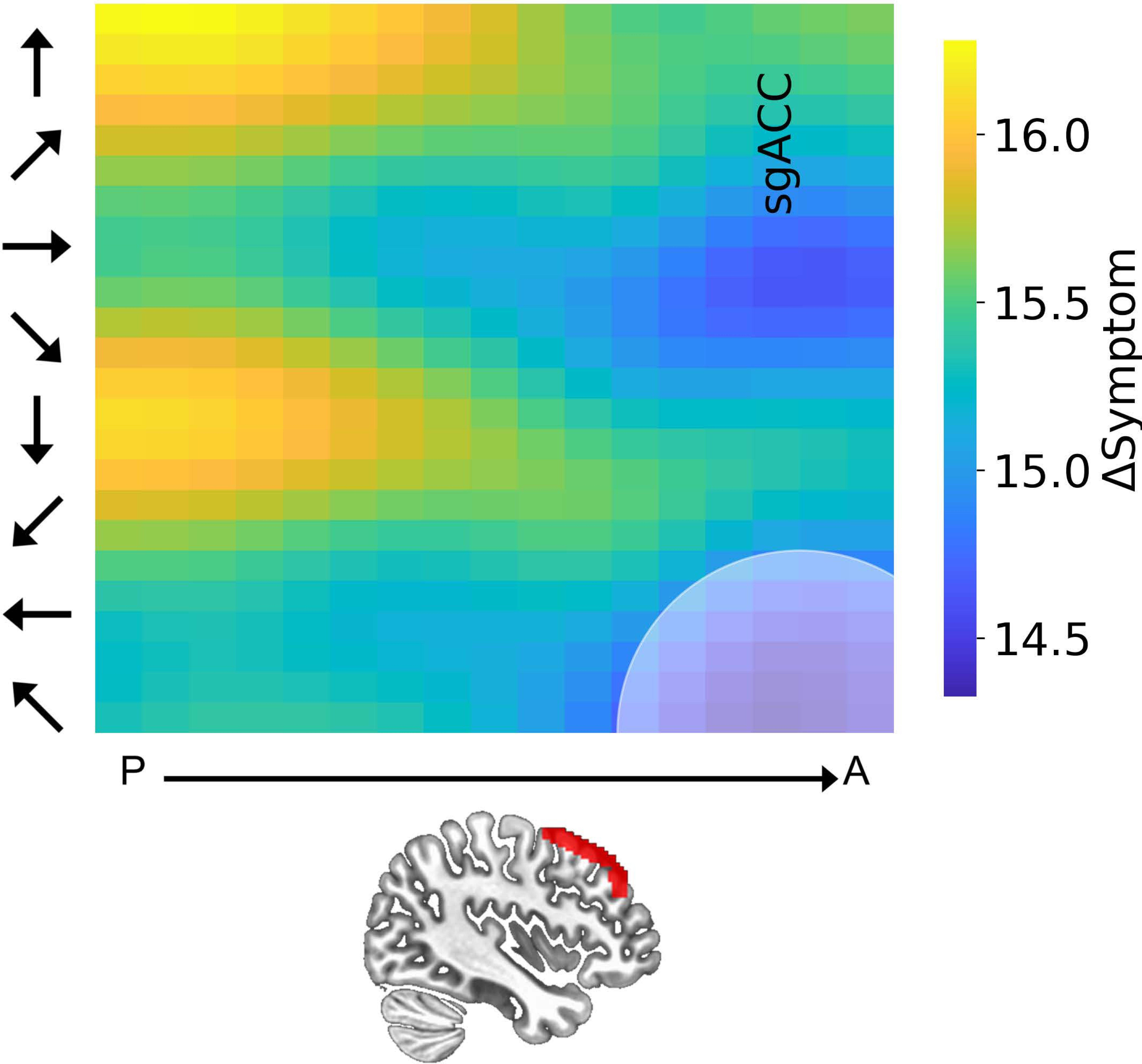

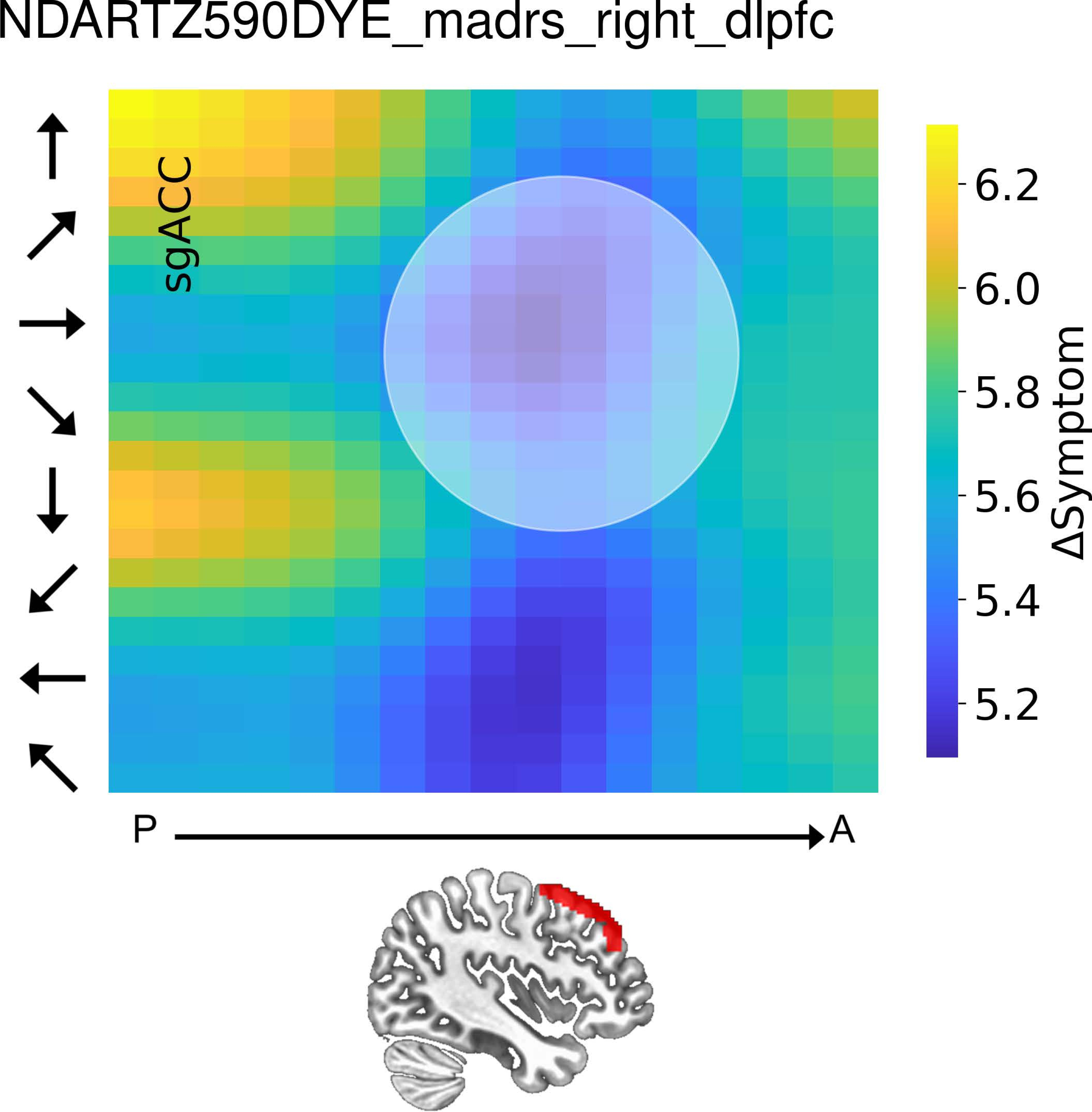

NDARTZ590DYE_madrs_right_dlpfc
sgACC
P
A
6.2
6.0
5.8
5.6
5.4
5.2
ΔSymptom

NDARUJ223ZPN_madrs_right_dlpfc

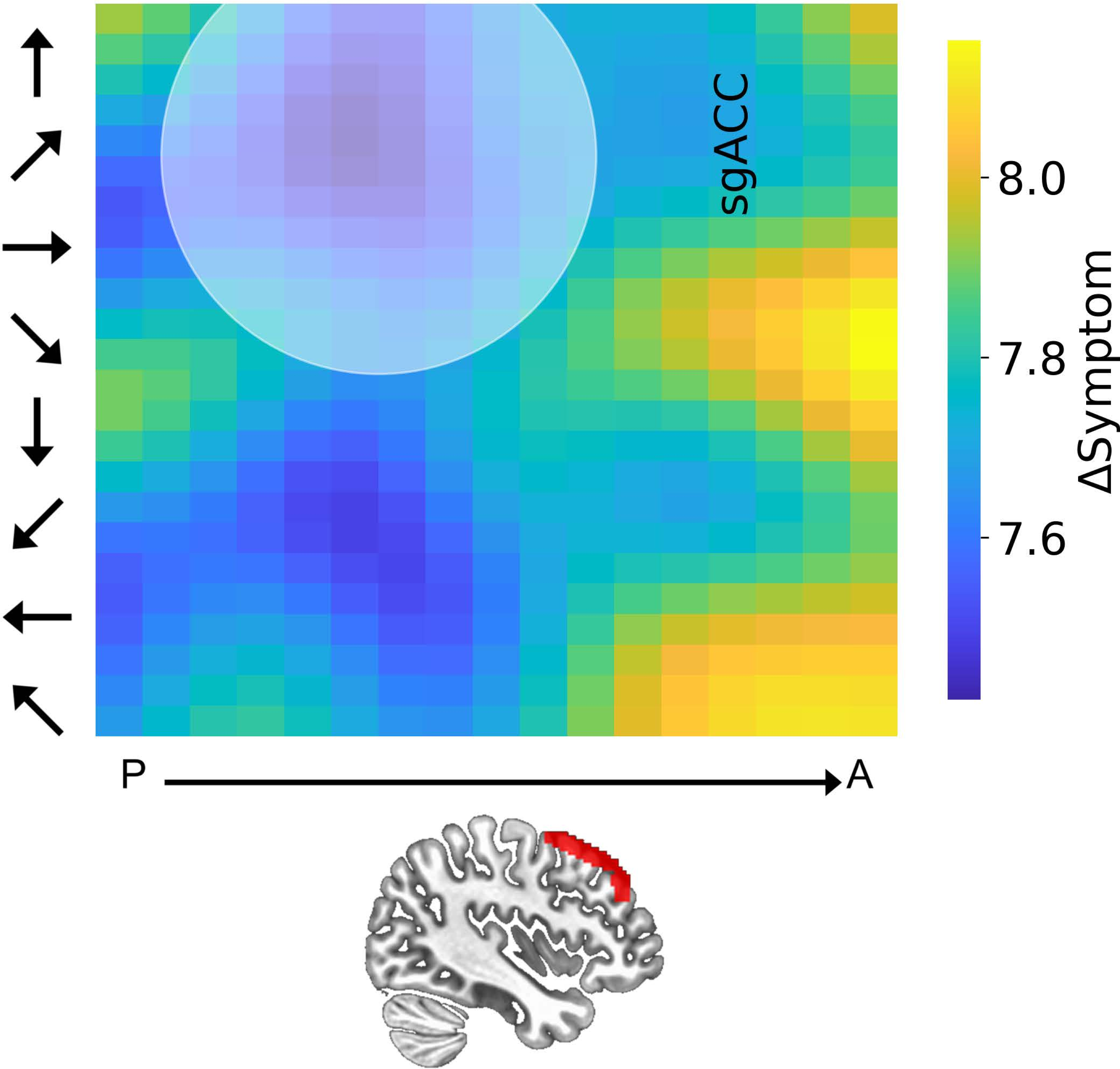

NDARUM818JC1_madrs_right_dlpfc

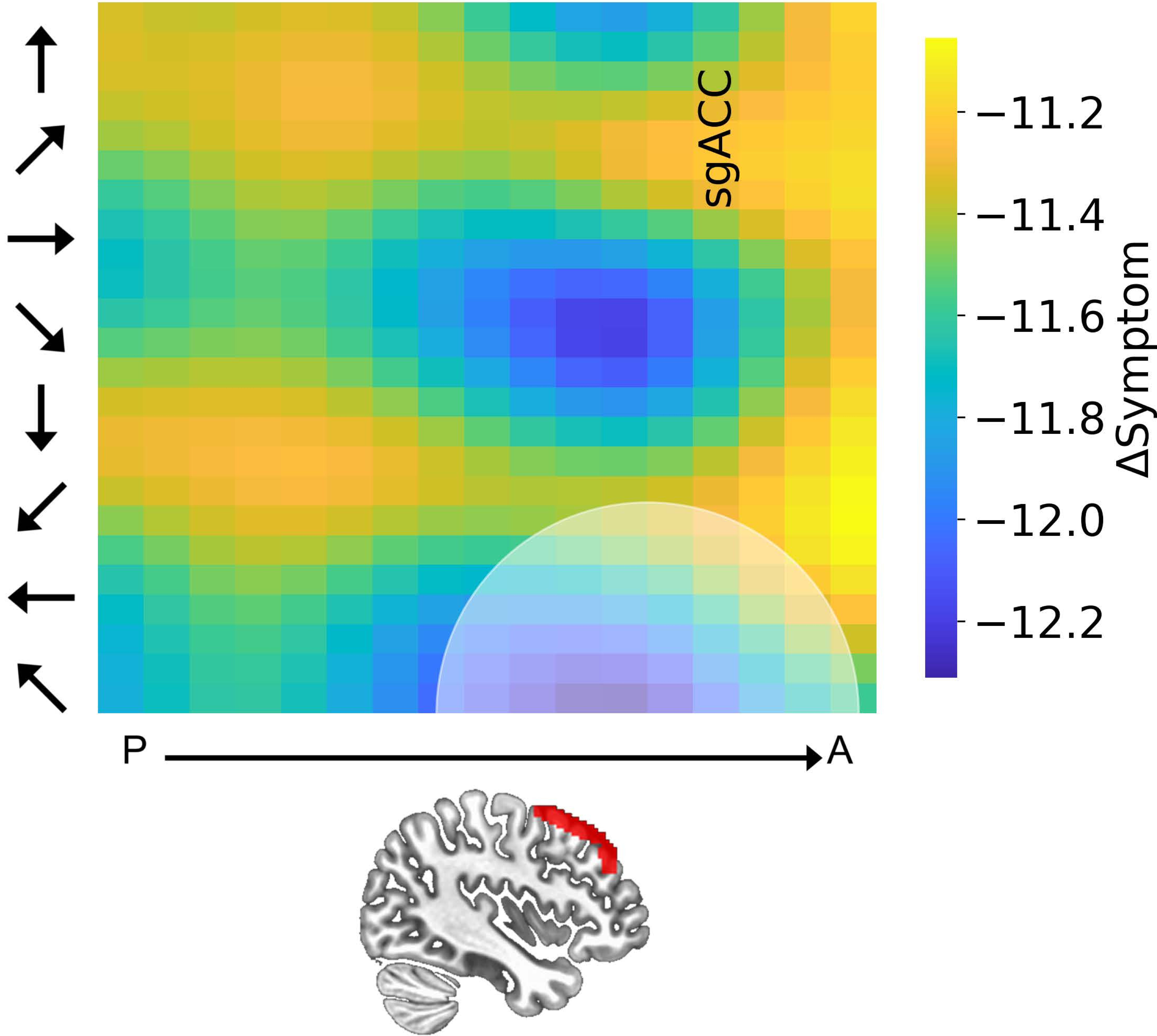

NDARUT852KUX_madrs_right_dlpfc

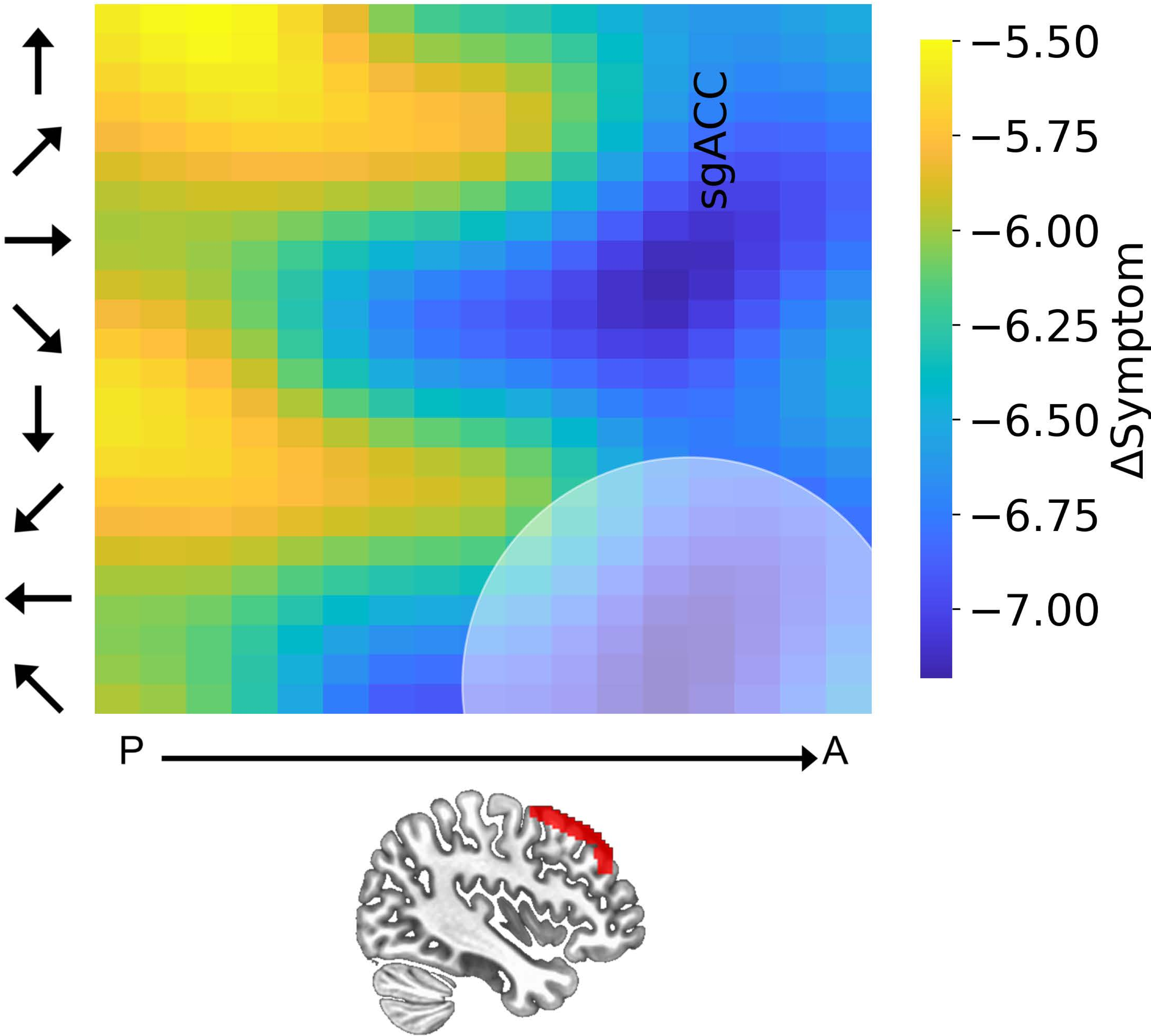

NDARVE642FAN_madrs_right_dlpfc

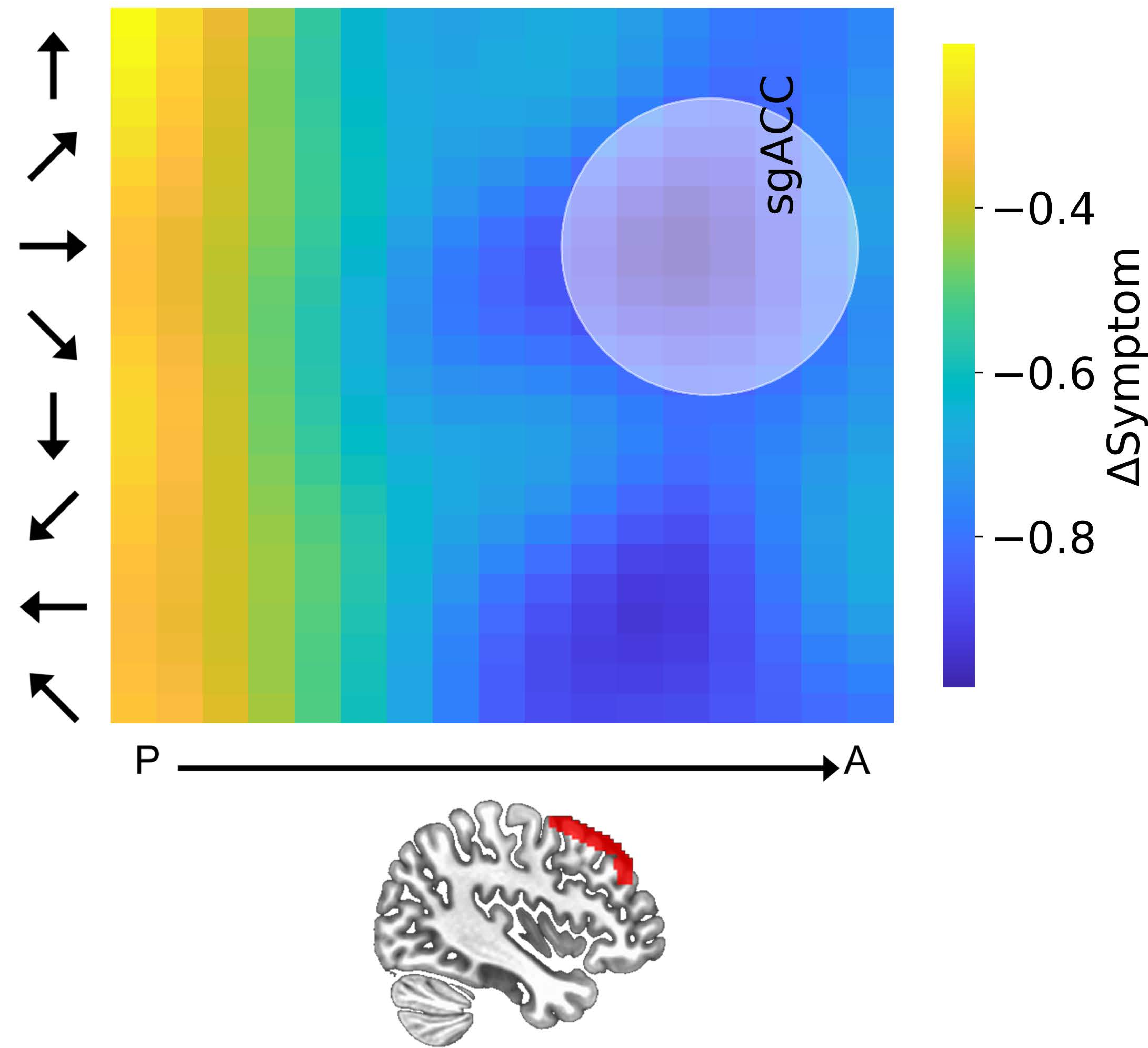

NDARVL144YVF_madrs_right_dlpfc

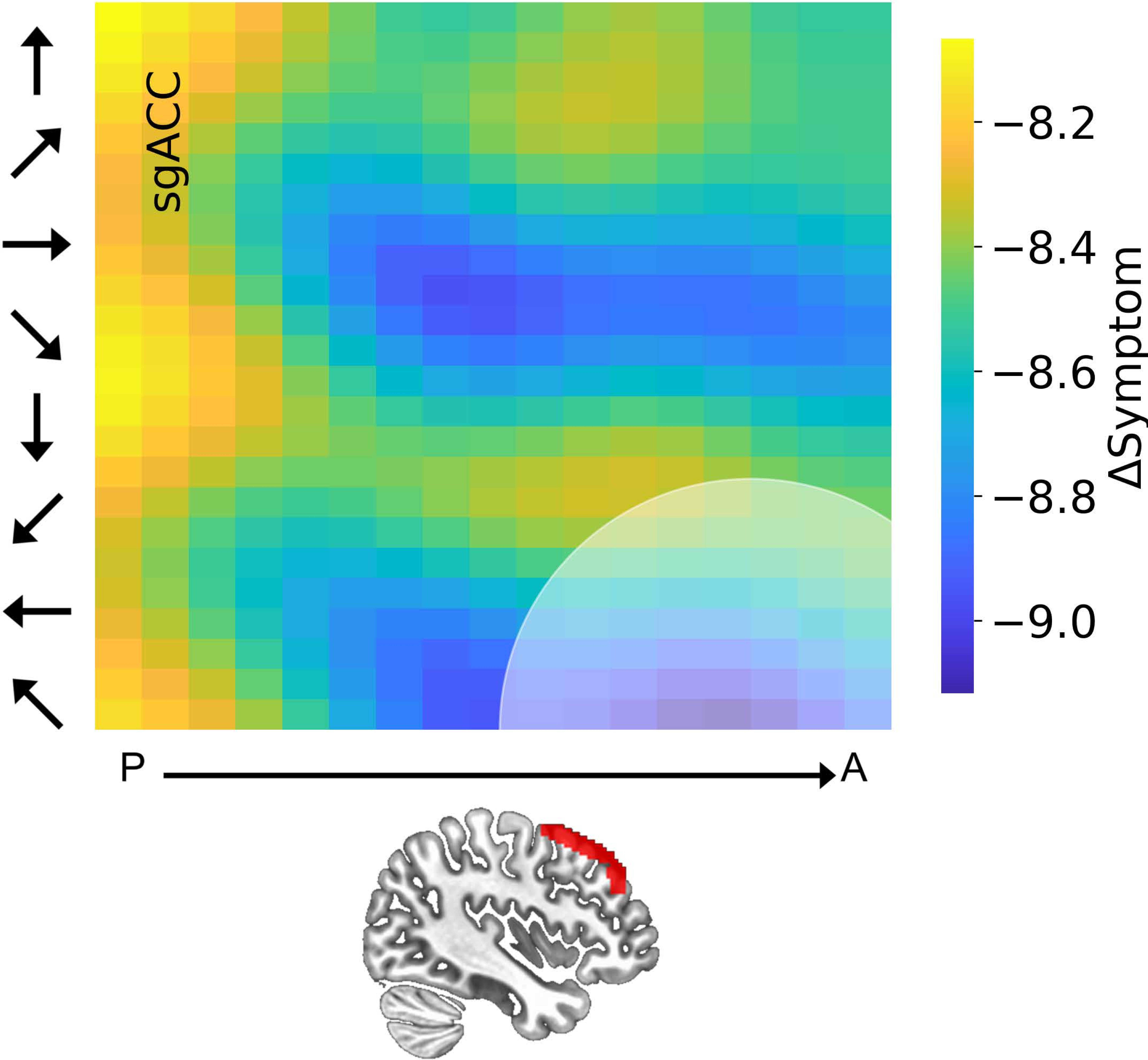

NDARWH523DTV_madrs_right_dlpfc

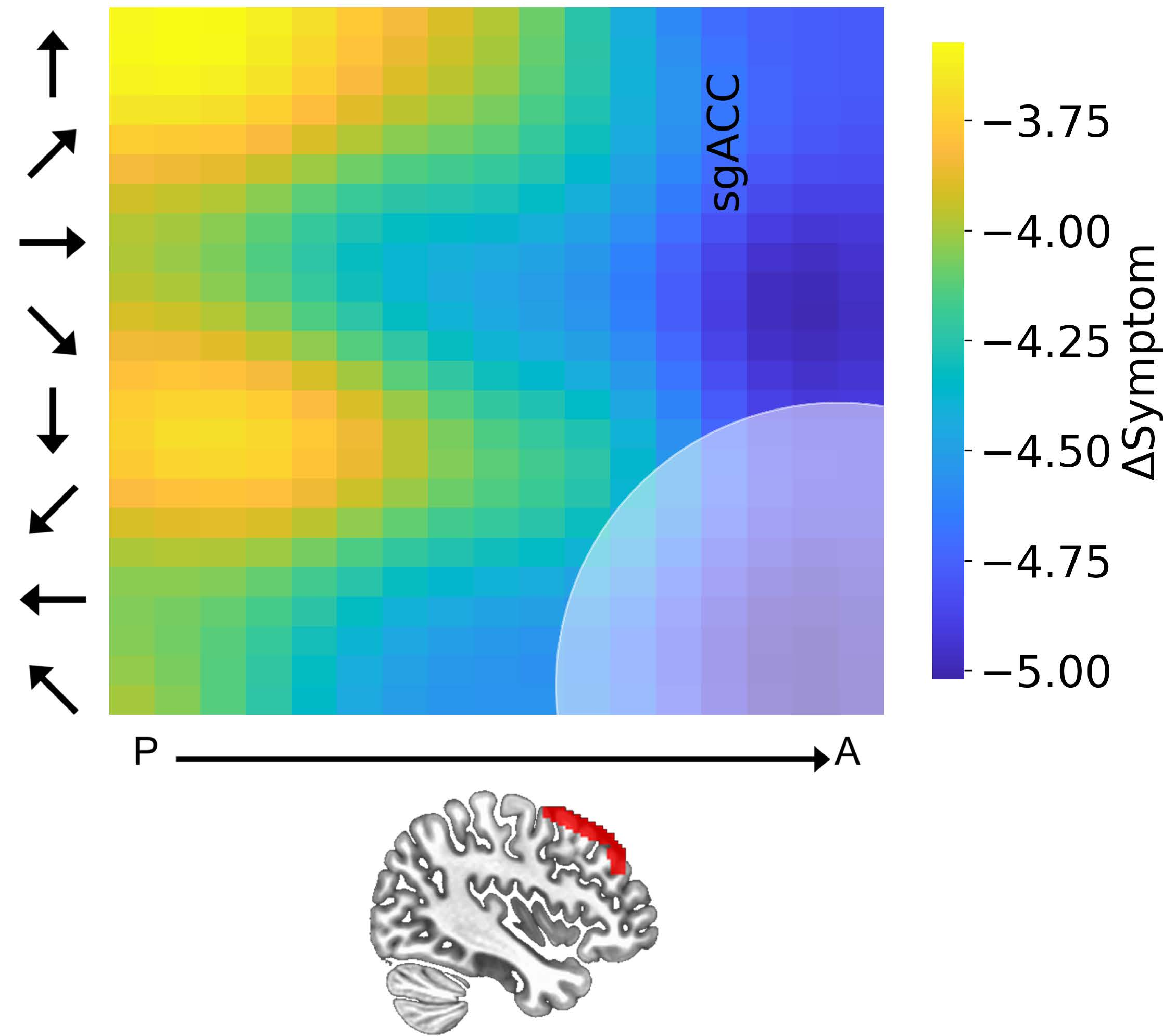

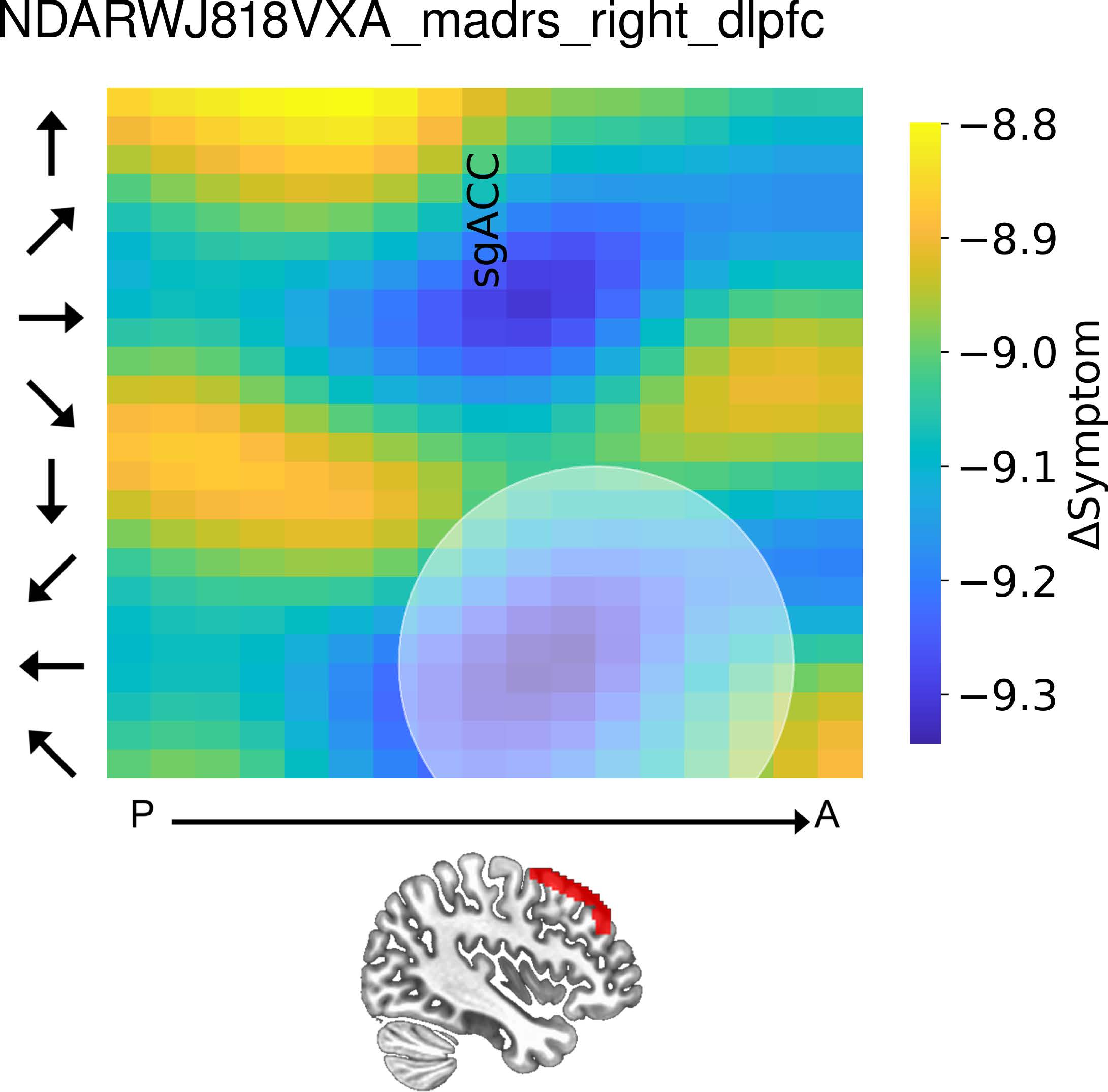
NDARWJ818VXA_madrs_right_dlpfc
sgACC
−8.8
−8.9
−9.0
−9.1
−9.2
−9.3
ΔSymptom
P
A

NDARWK081NTV_madrs_right_dlpfc

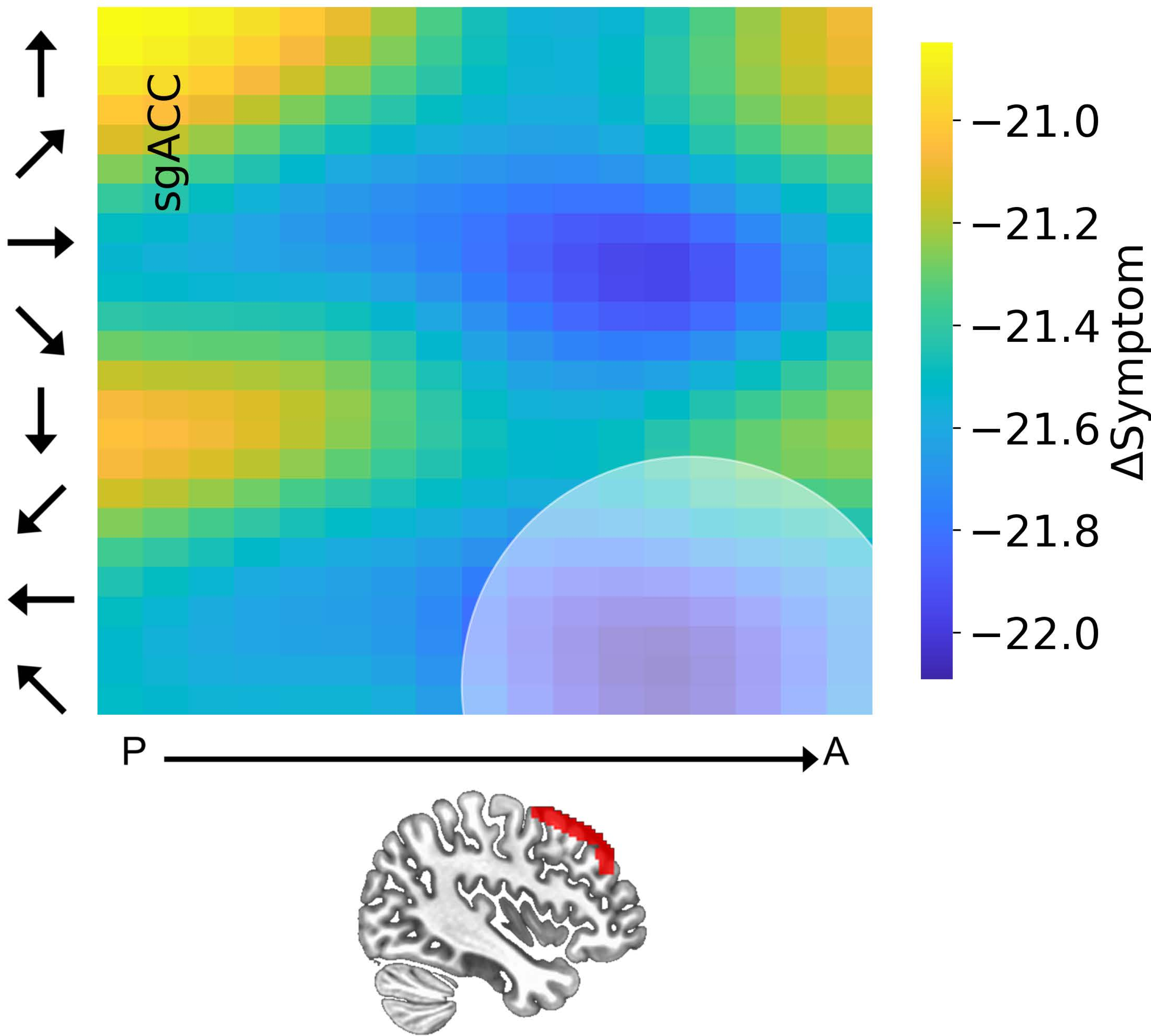

NDARWL277NXF_madrs_right_dlpfc

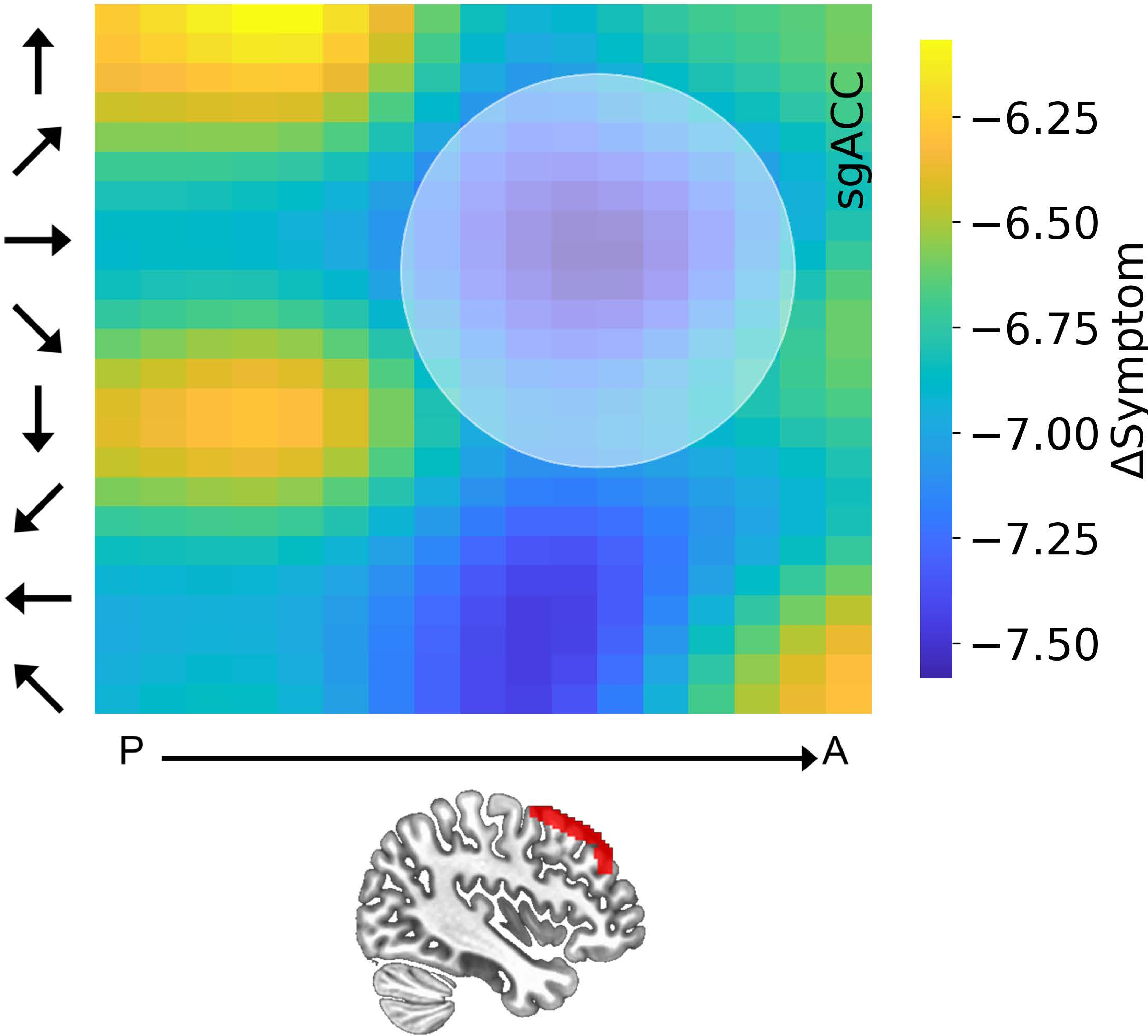

NDARWV016ZAT_madrs_right_dlpfc

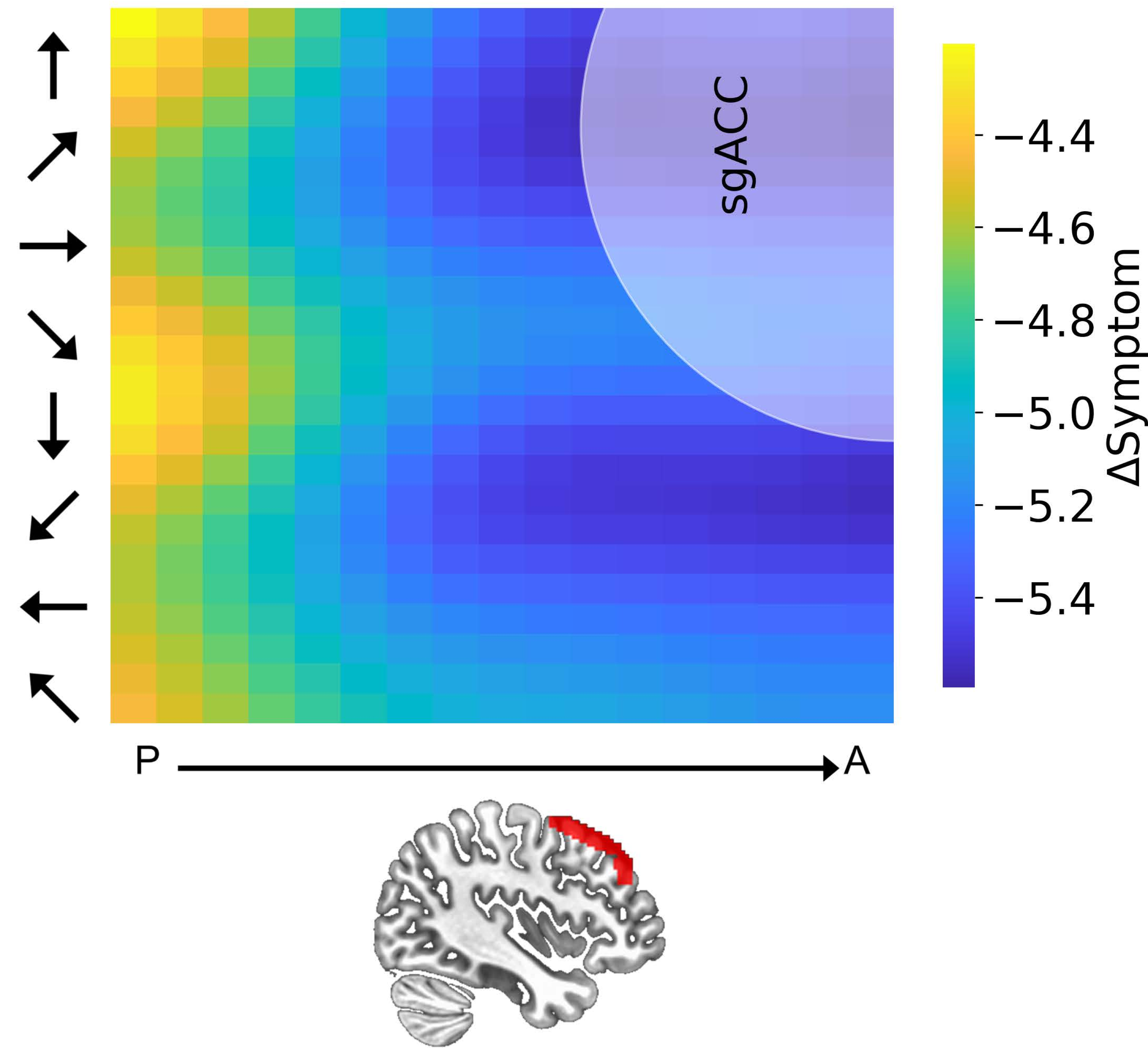

NDARXA148KPL_madrs_right_dlpfc

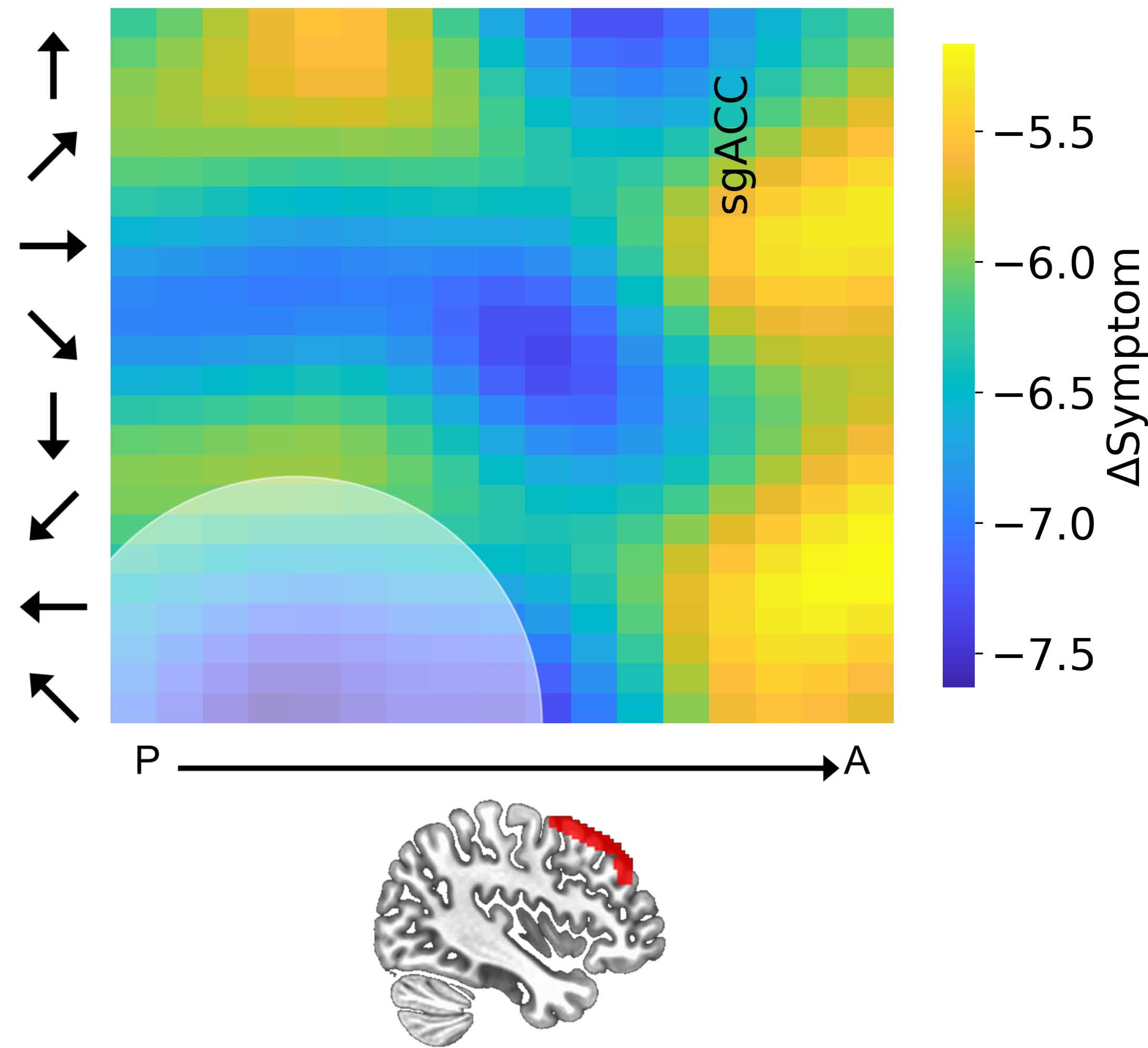

NDARXD921NRV_madrs_right_dlpfc

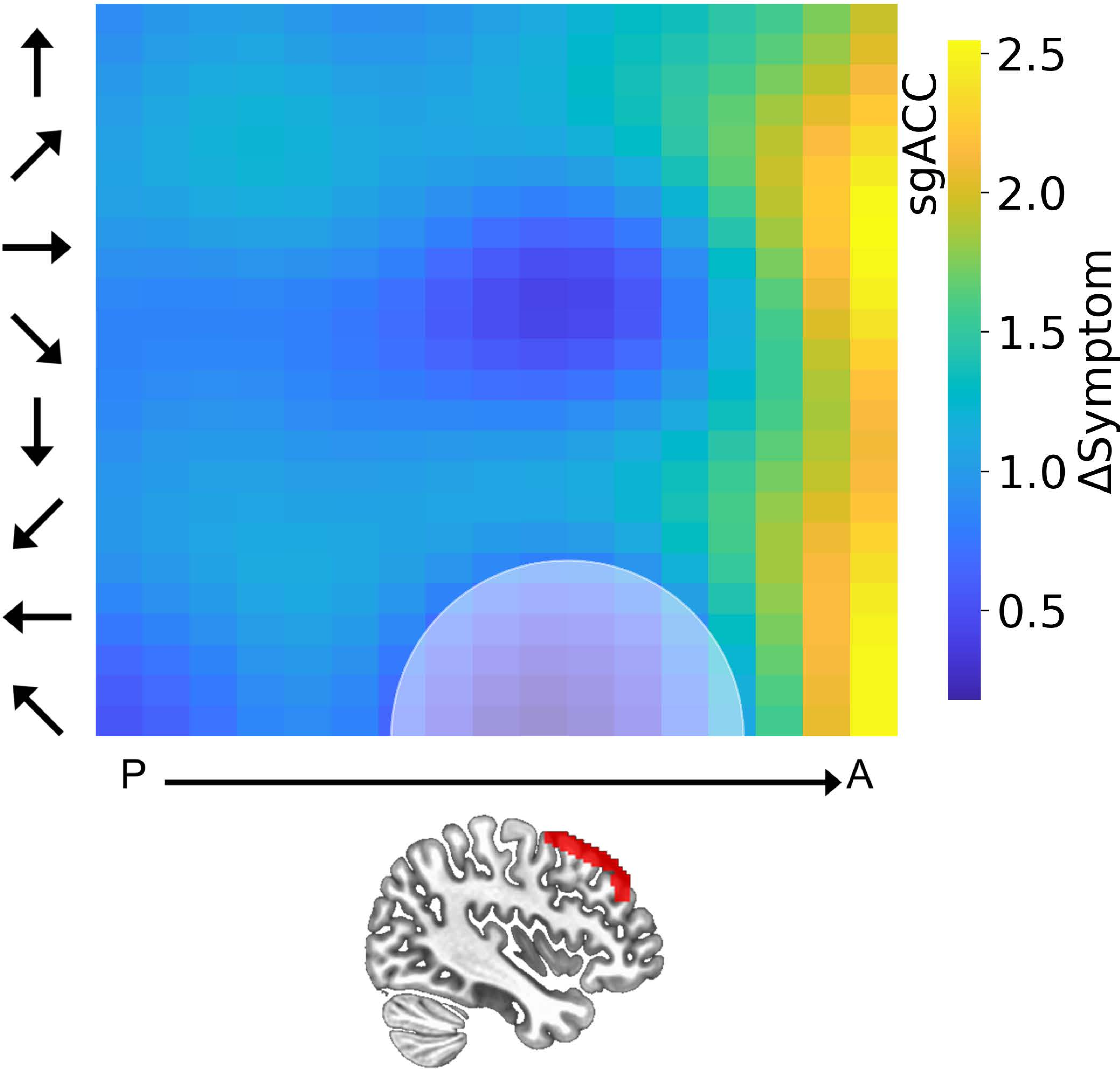

NDARXU500EFT_madrs_right_dlpfc

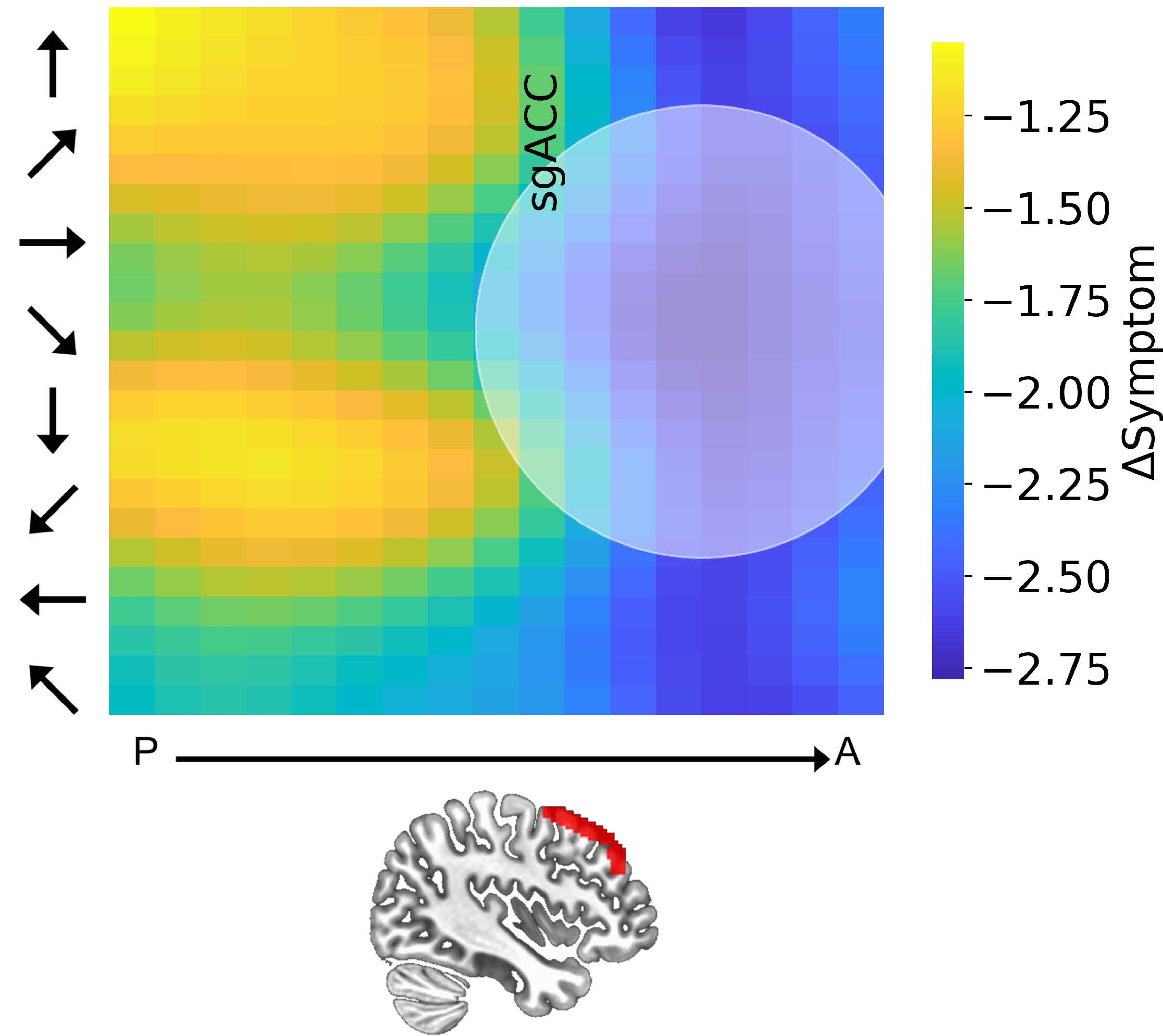

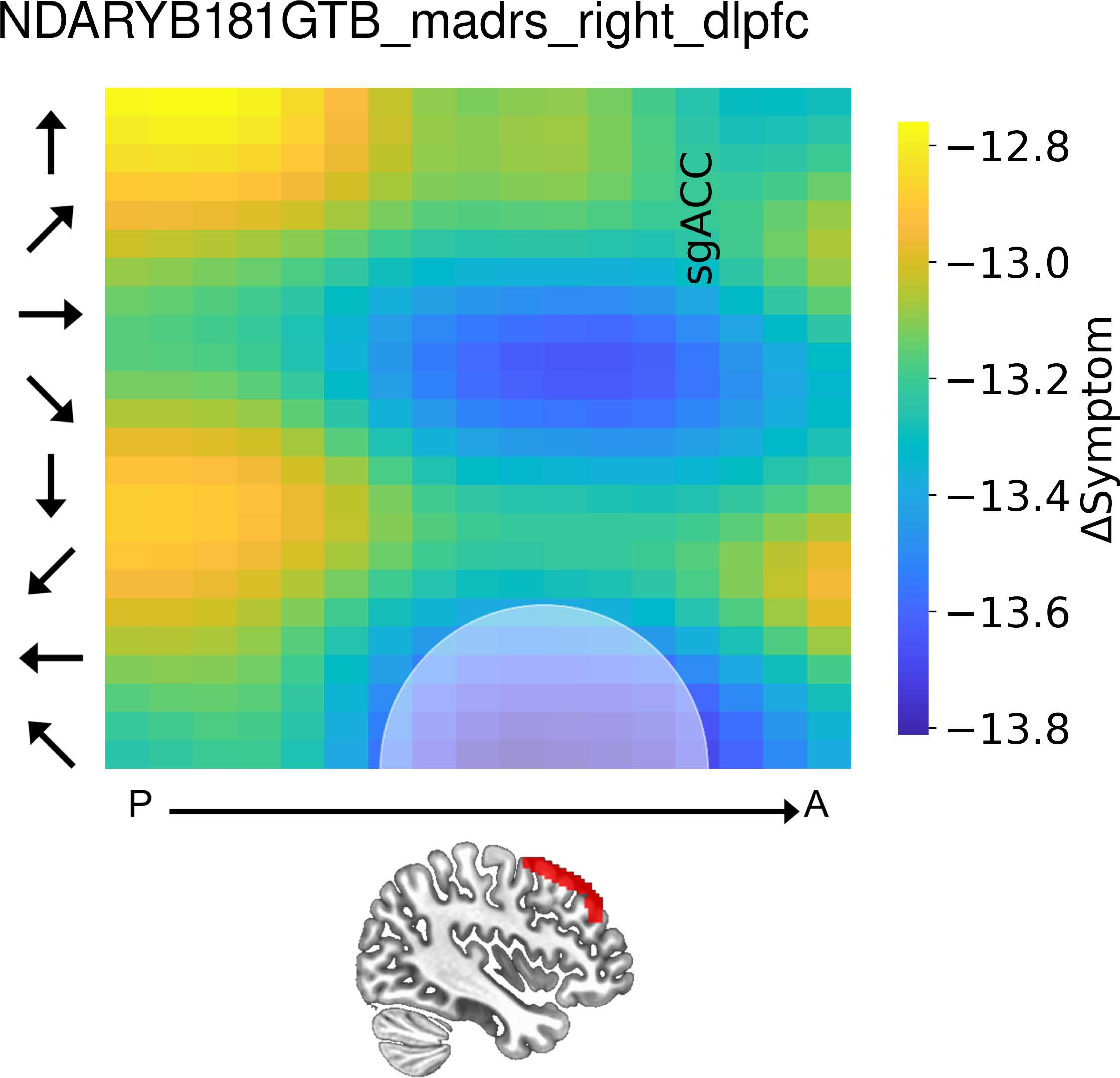

NDARYB181GTB_madrs_right_dlpfc
sgACC
−12.8
−13.0
−13.2
−13.4
−13.6
−13.8
ΔSymptom
P
A

NDARYD169AYQ_madrs_right_dlpfc

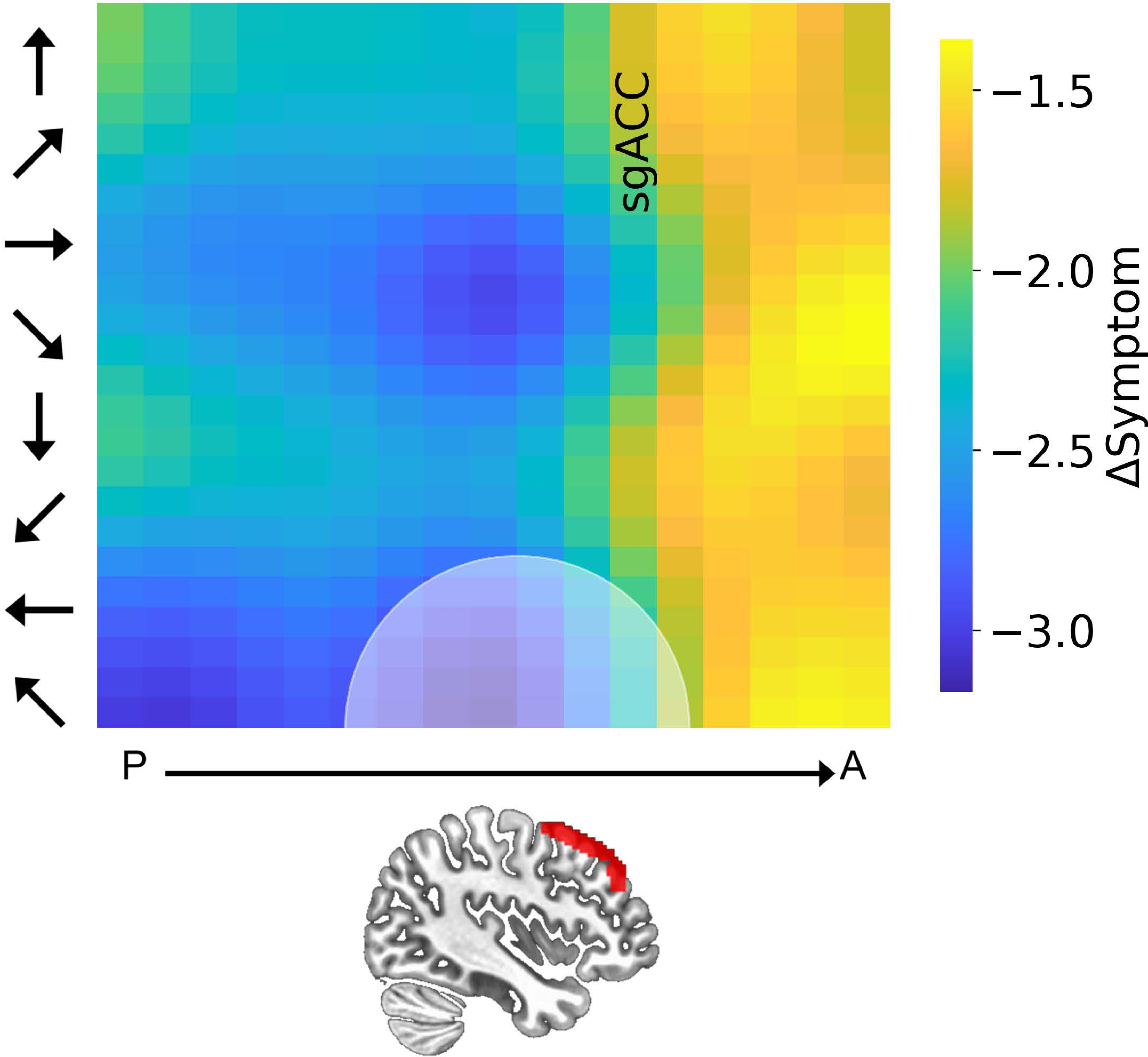

NDARYE405JJL_madrs_right_dlpfc

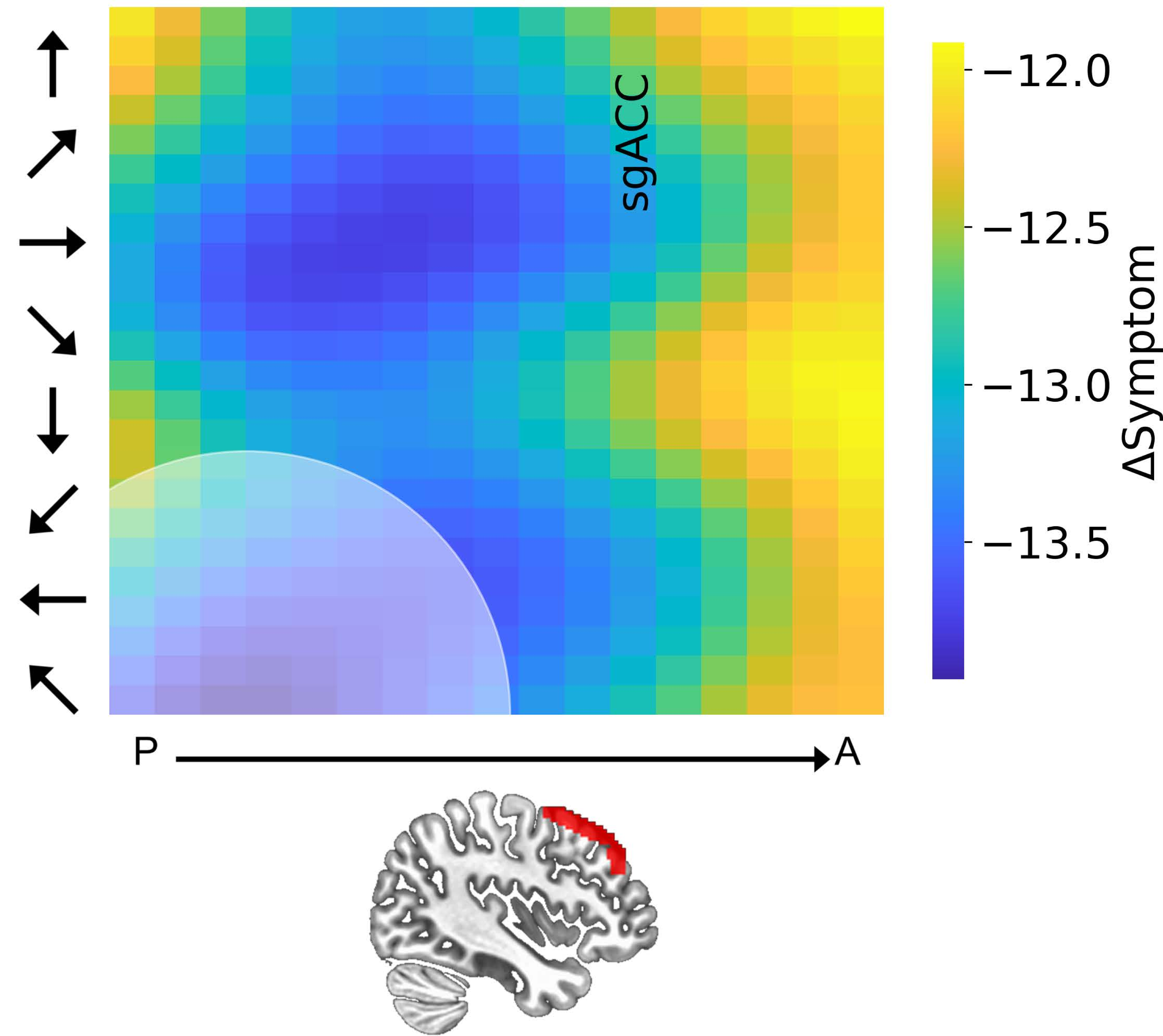

NDARYM648GK3_madrs_right_dlpfc

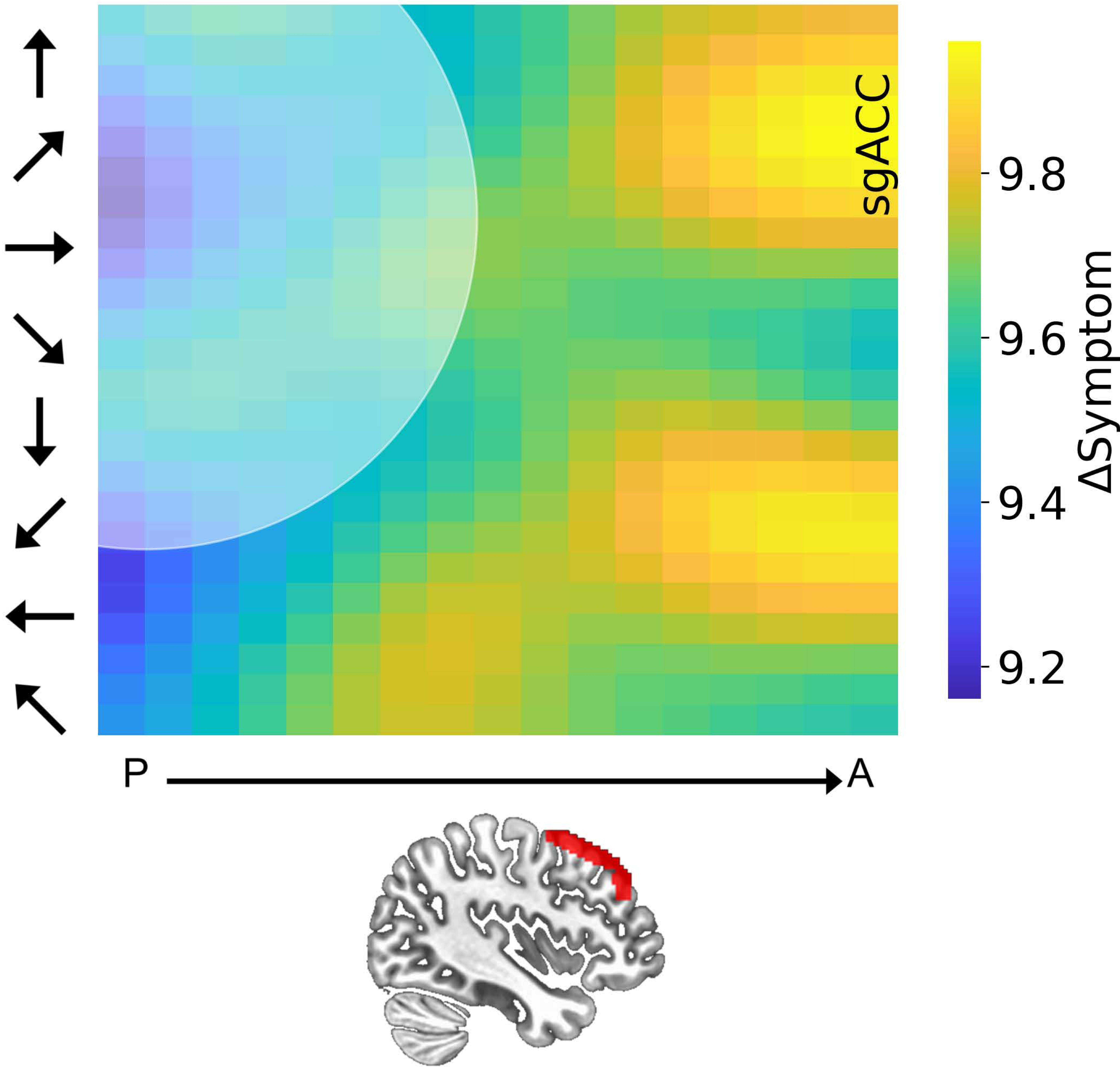

NDARZH200GZU_madrs_right_dlpfc

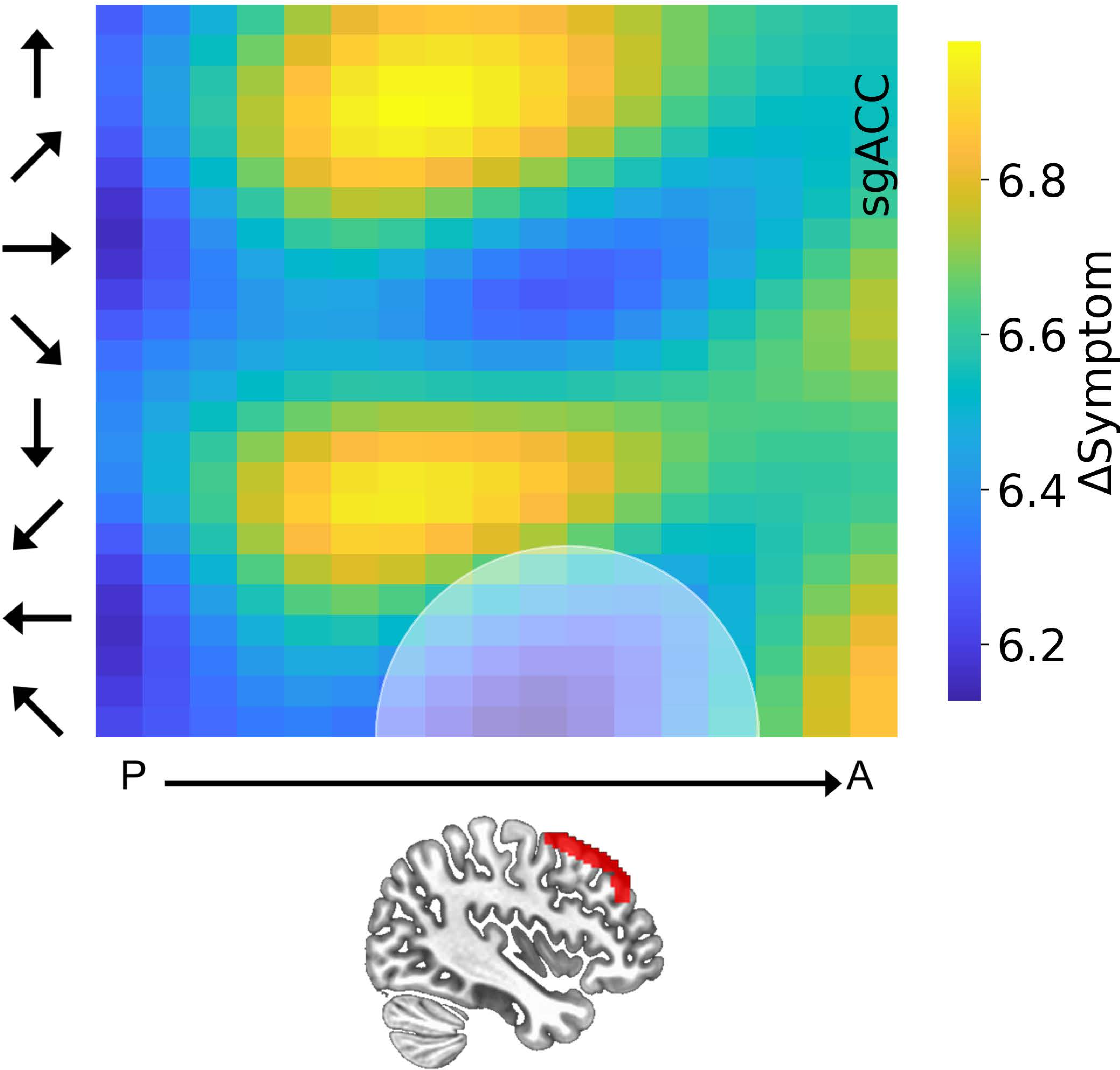

NDARBB471NAU_hamd_left_dlpfc

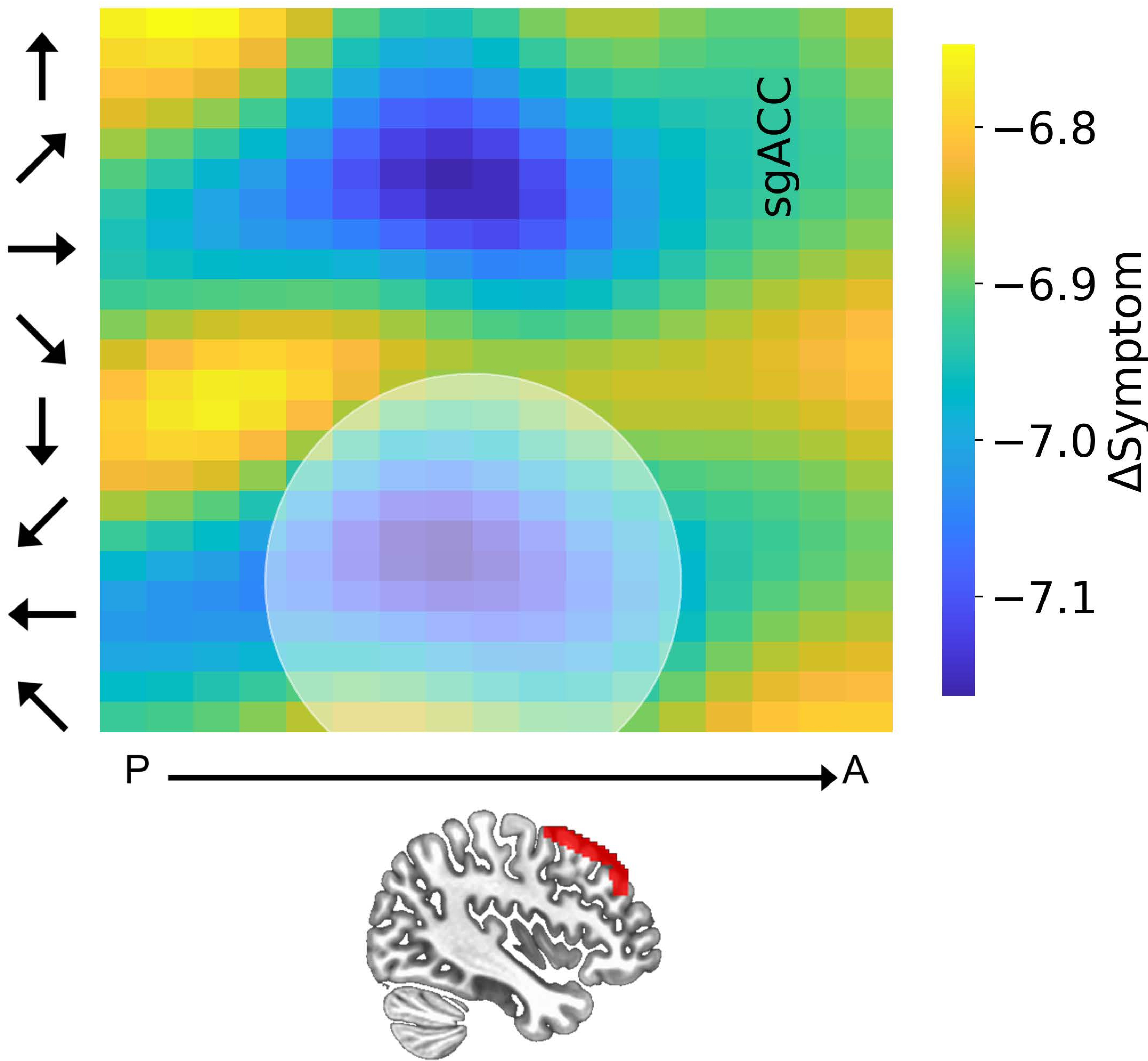

NDARBG497RE1_hamd_left_dlpfc

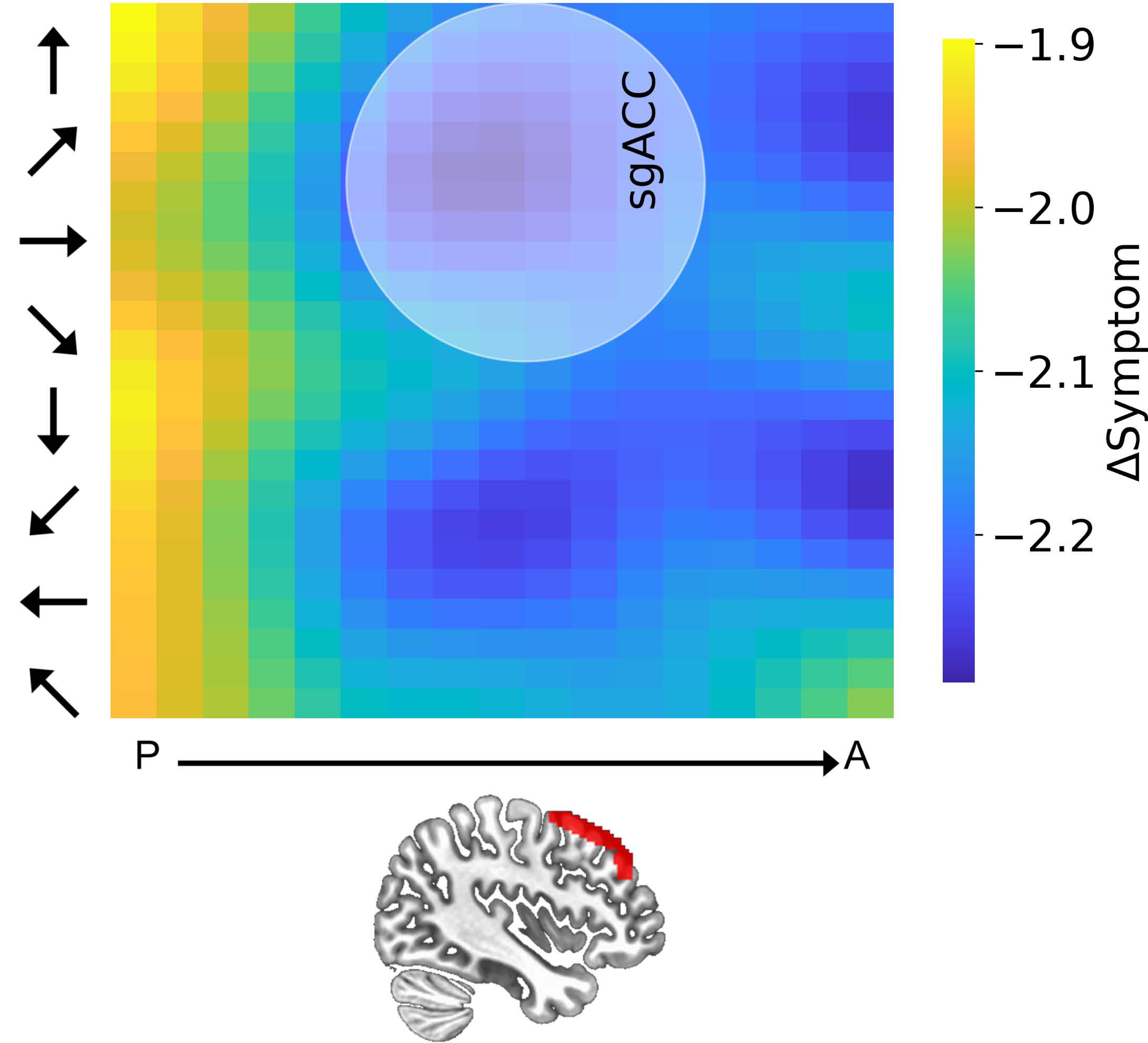

NDARBK027PDF_hamd_left_dlpfc

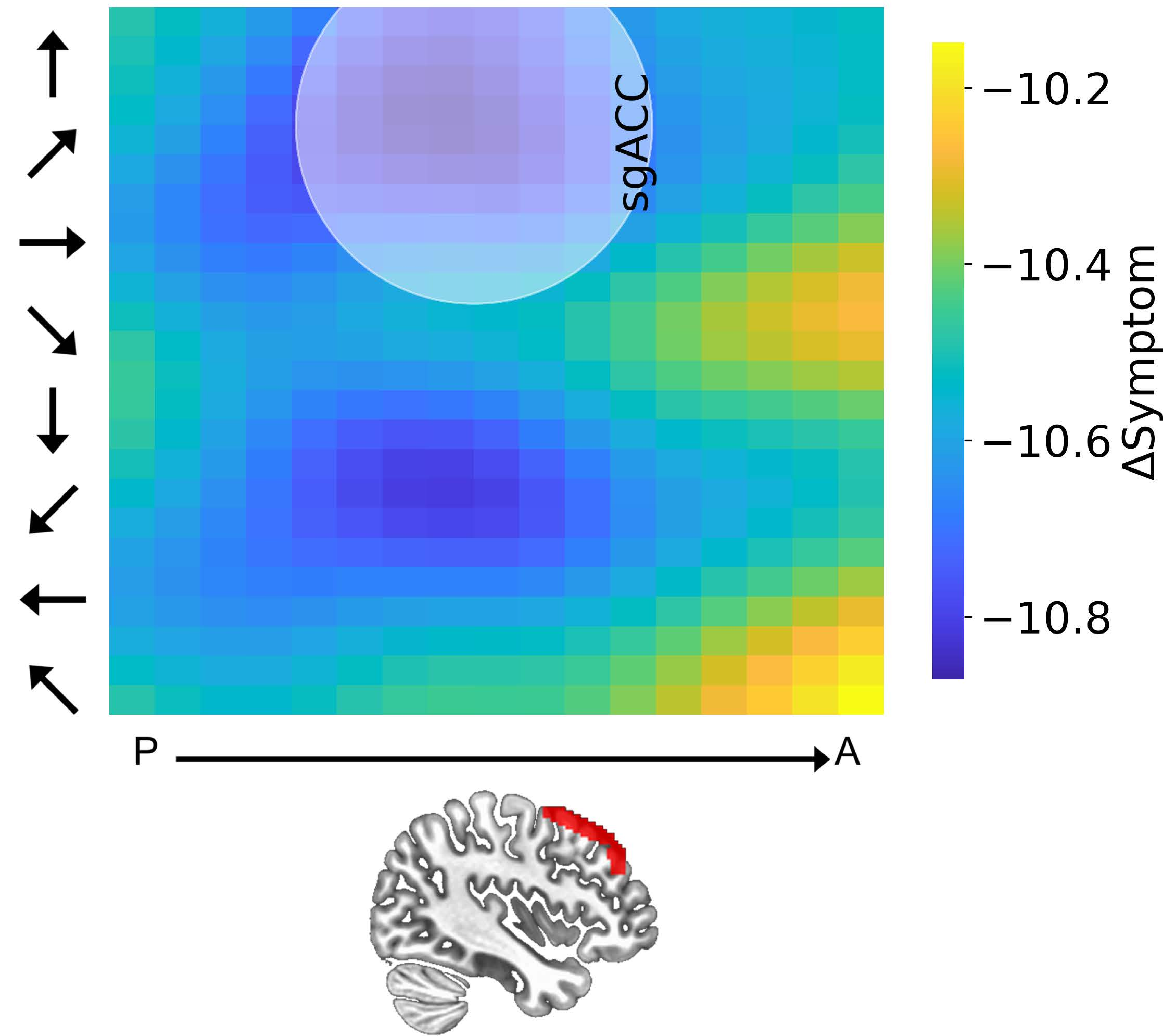

NDARBL814BJK_hamd_left_dlpfc

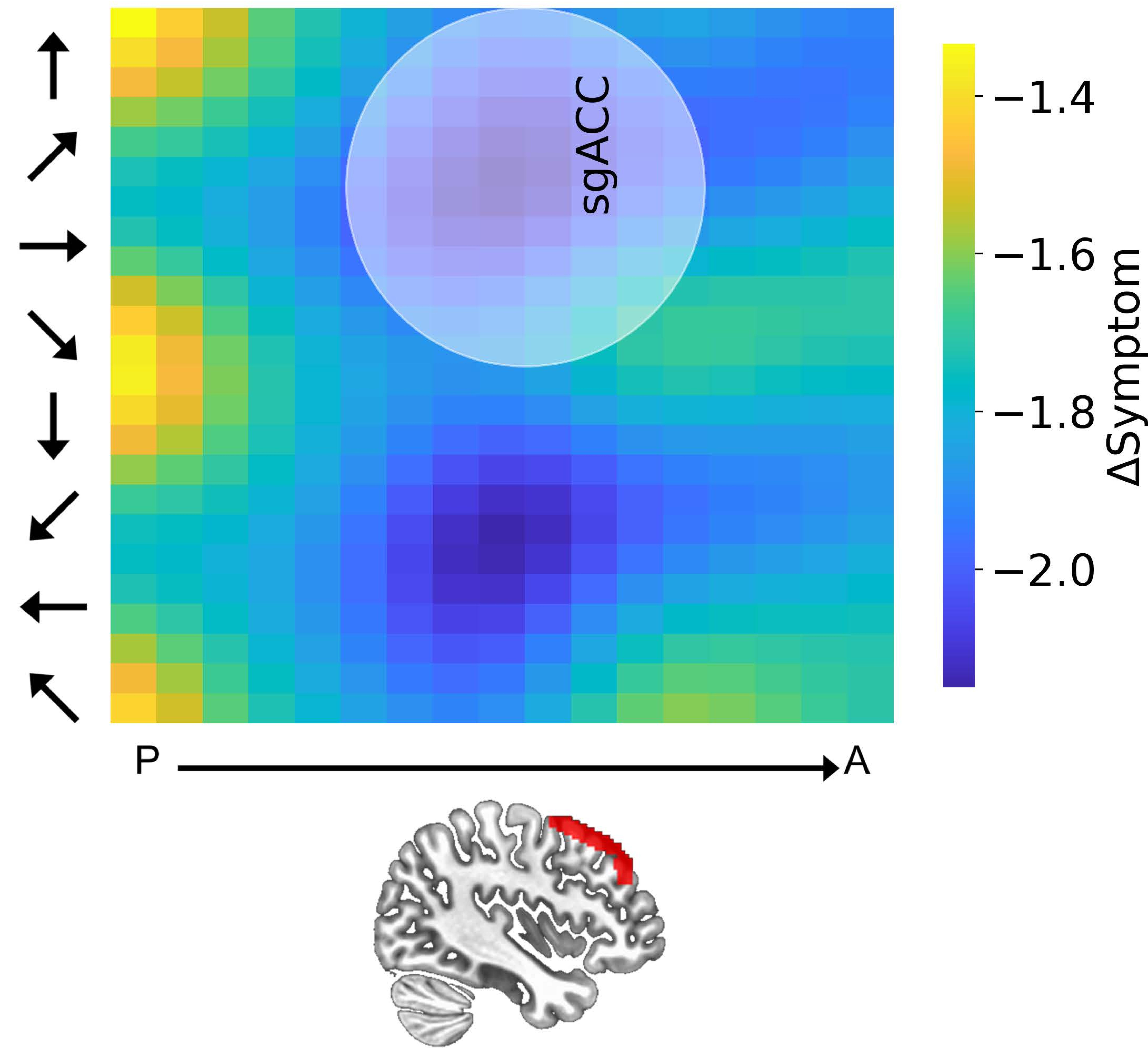

NDARBP353XD2_hamd_left_dlpfc

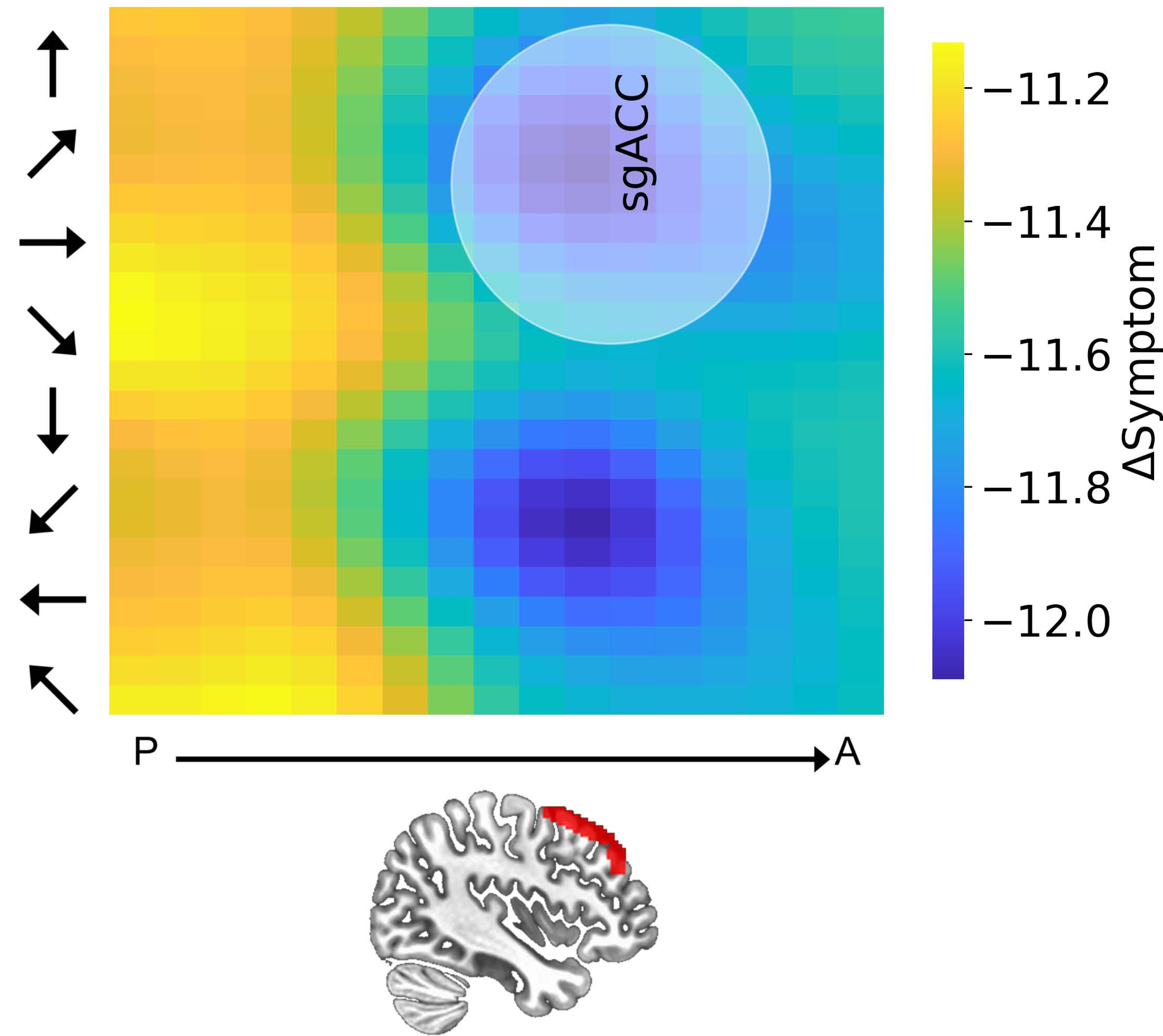

NDARBT583XH6_hamd_left_dlpfc

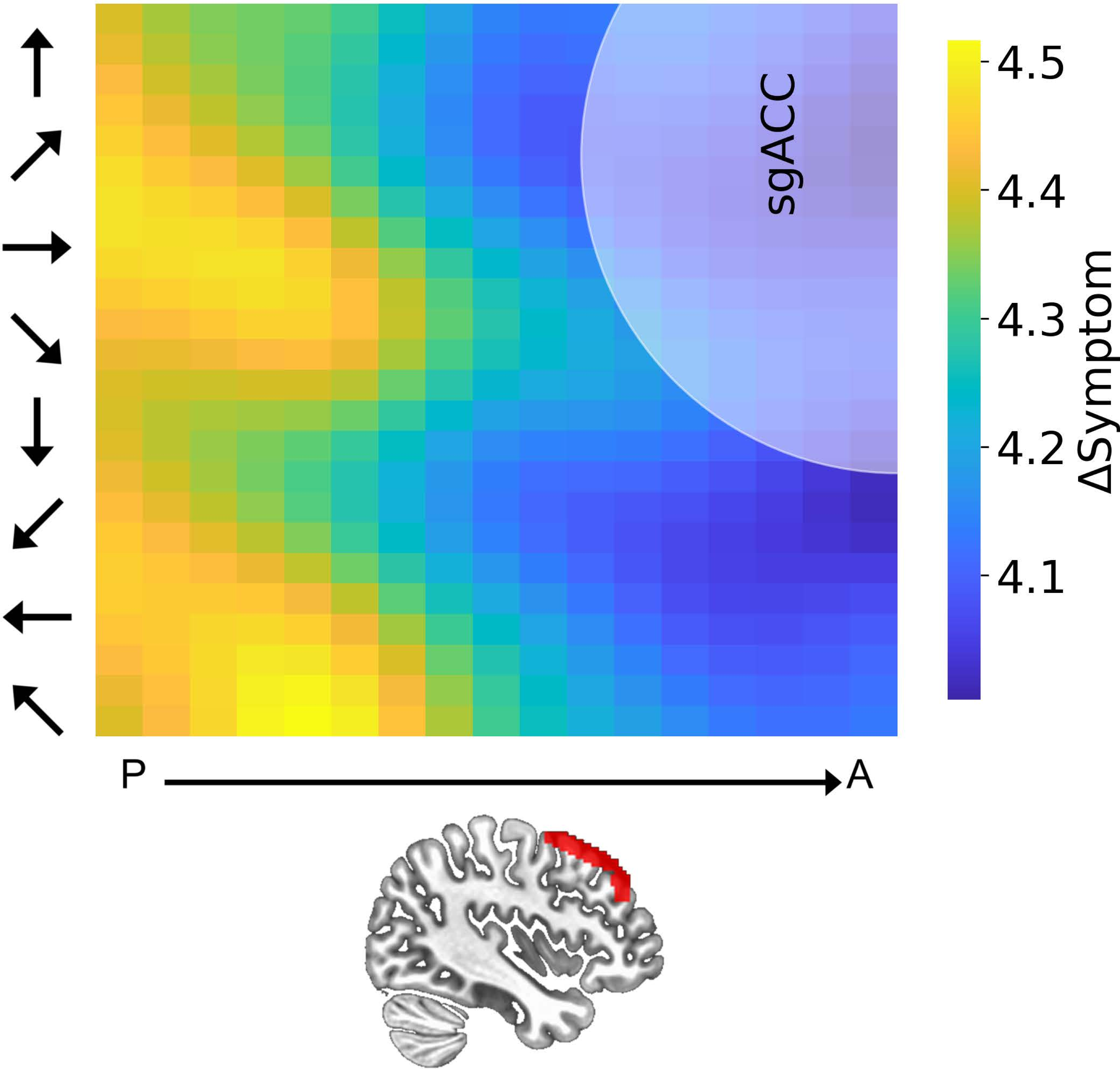

NDARCG071YN8_hamd_left_dlpfc

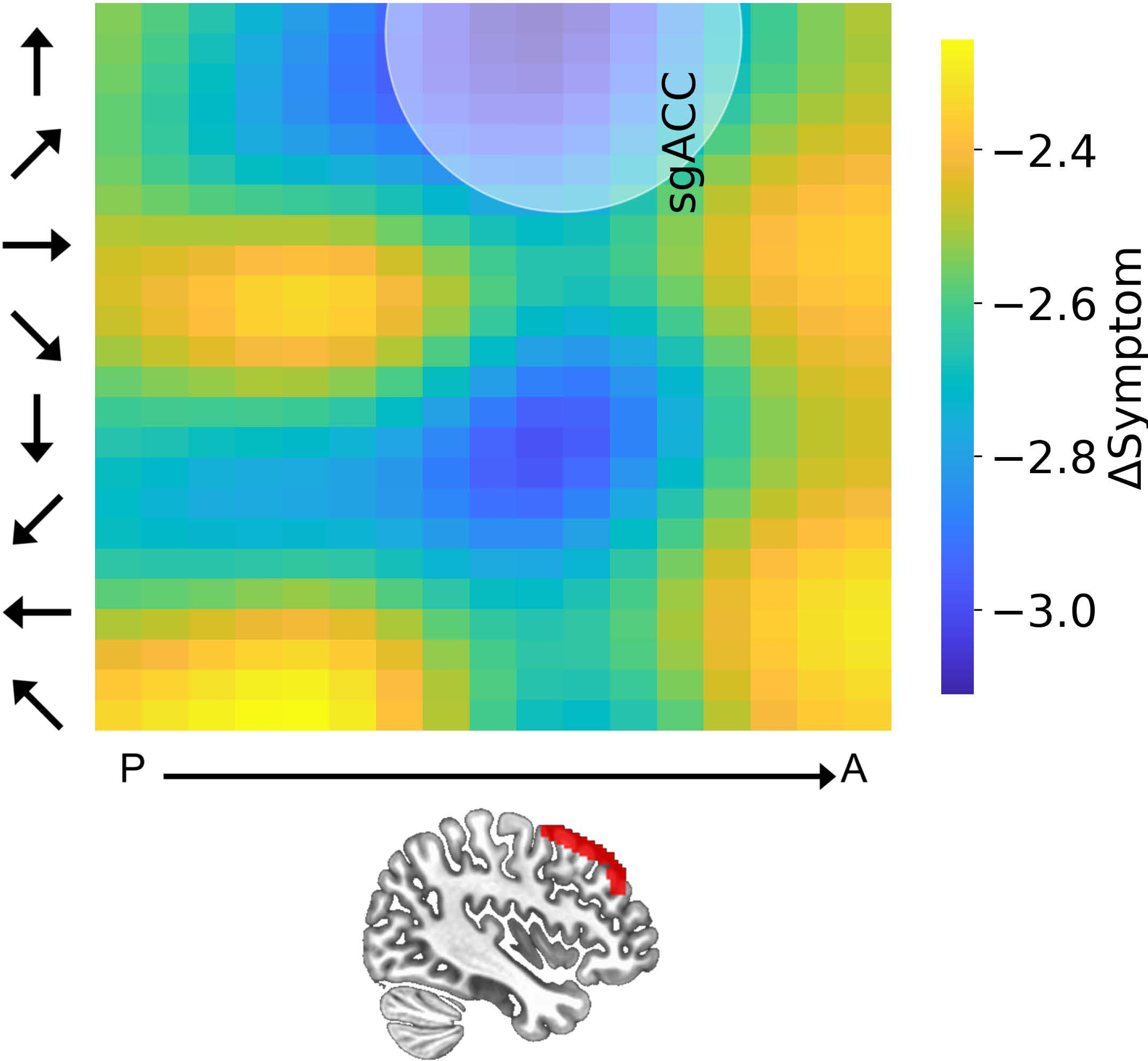

NDARCG271VGC_hamd_left_dlpfc

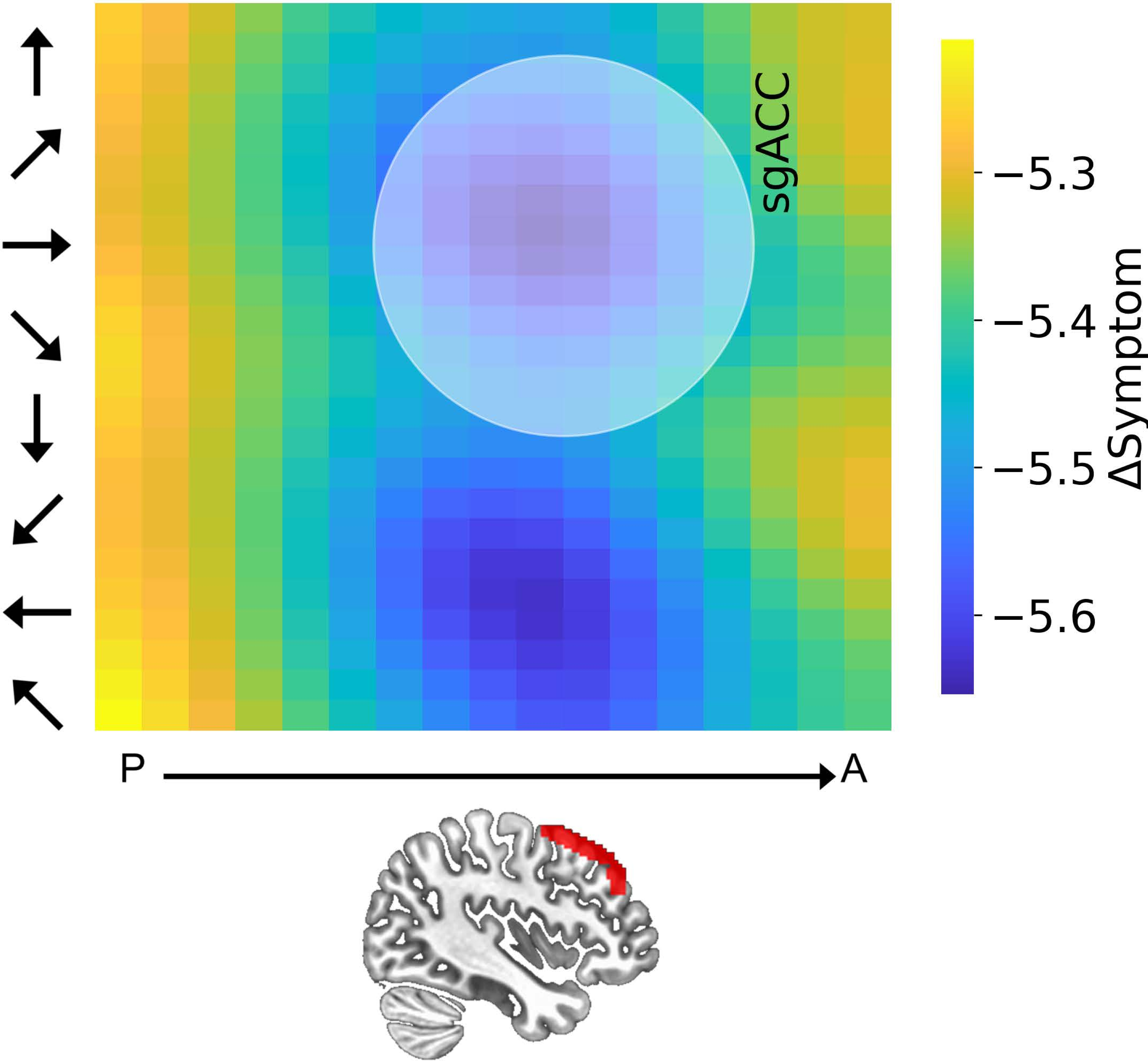

NDARCM769WZL_hamd_left_dlpfc

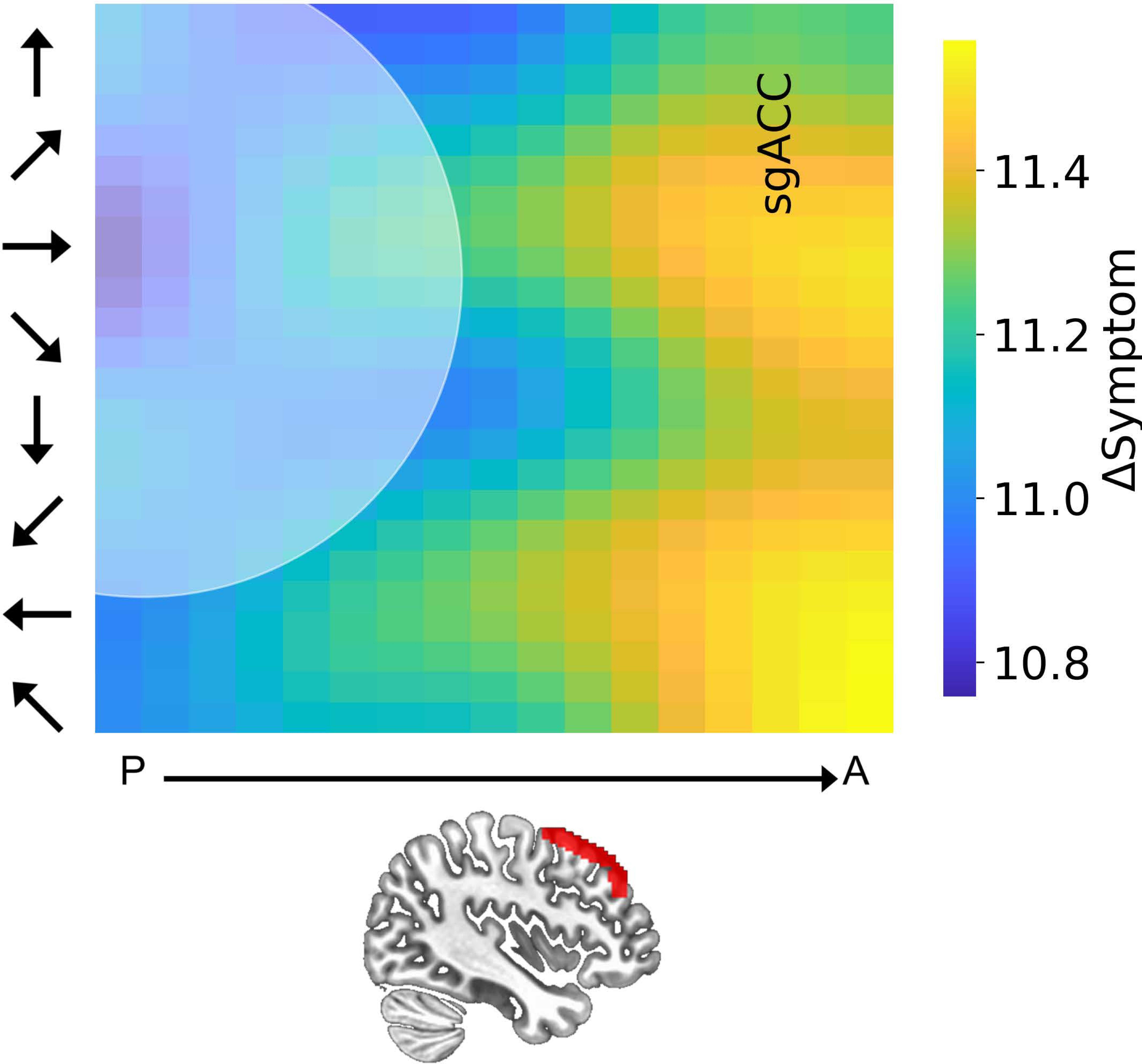

NDARDL291XT0_hamd_left_dlpfc

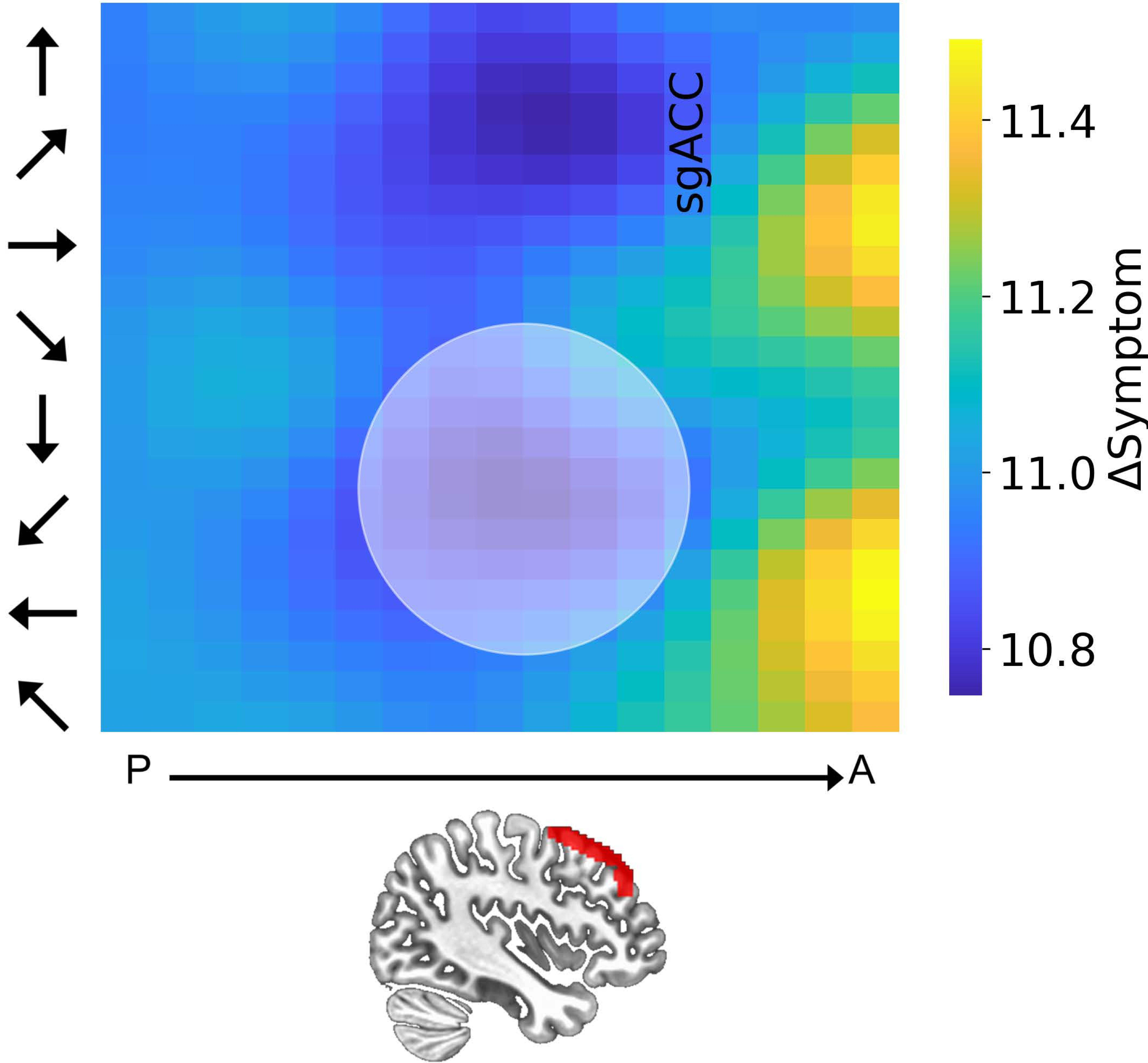

NDARDL667VMW_hamd_left_dlpfc

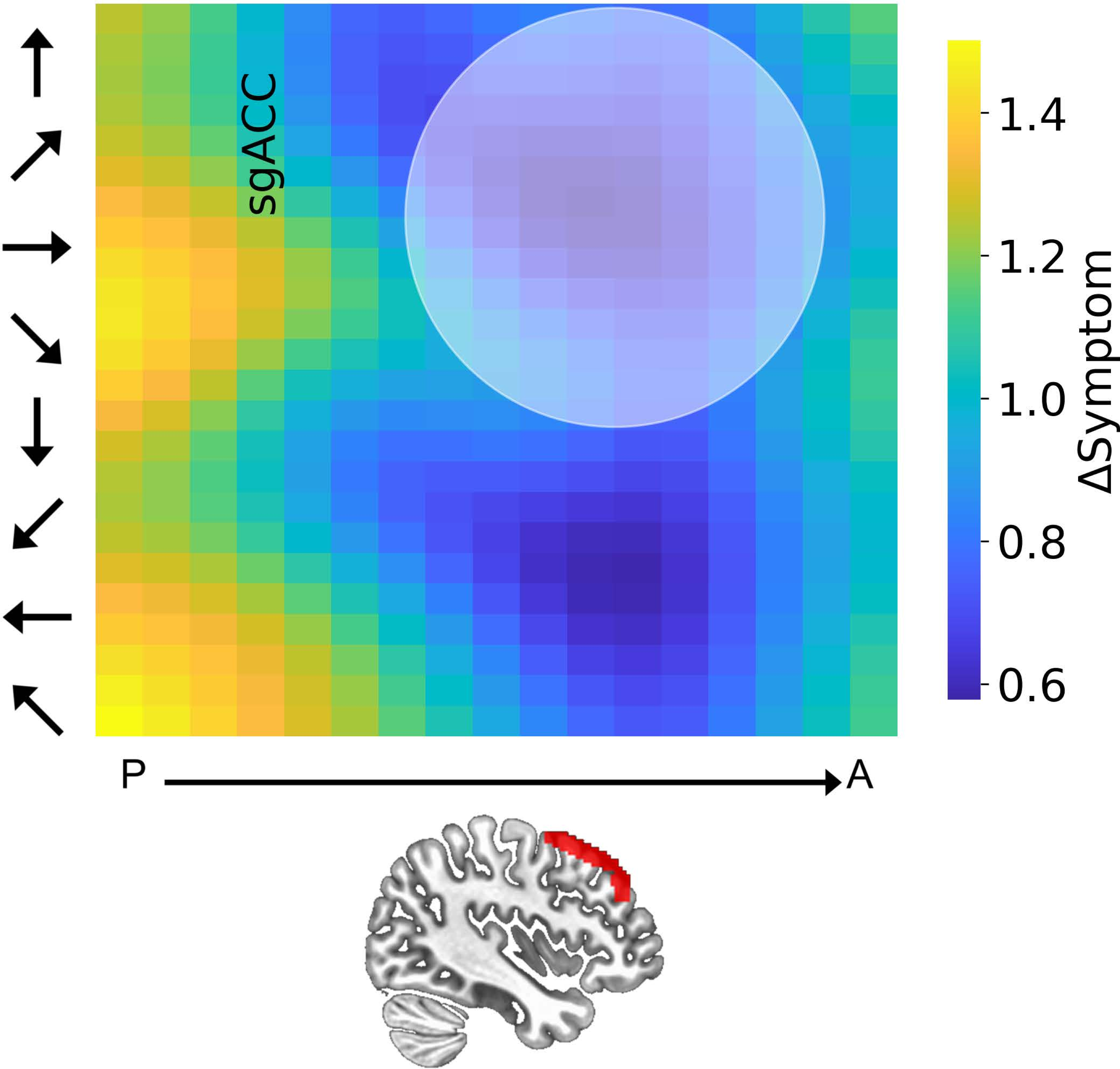

NDARDR017YX2_hamd_left_dlpfc

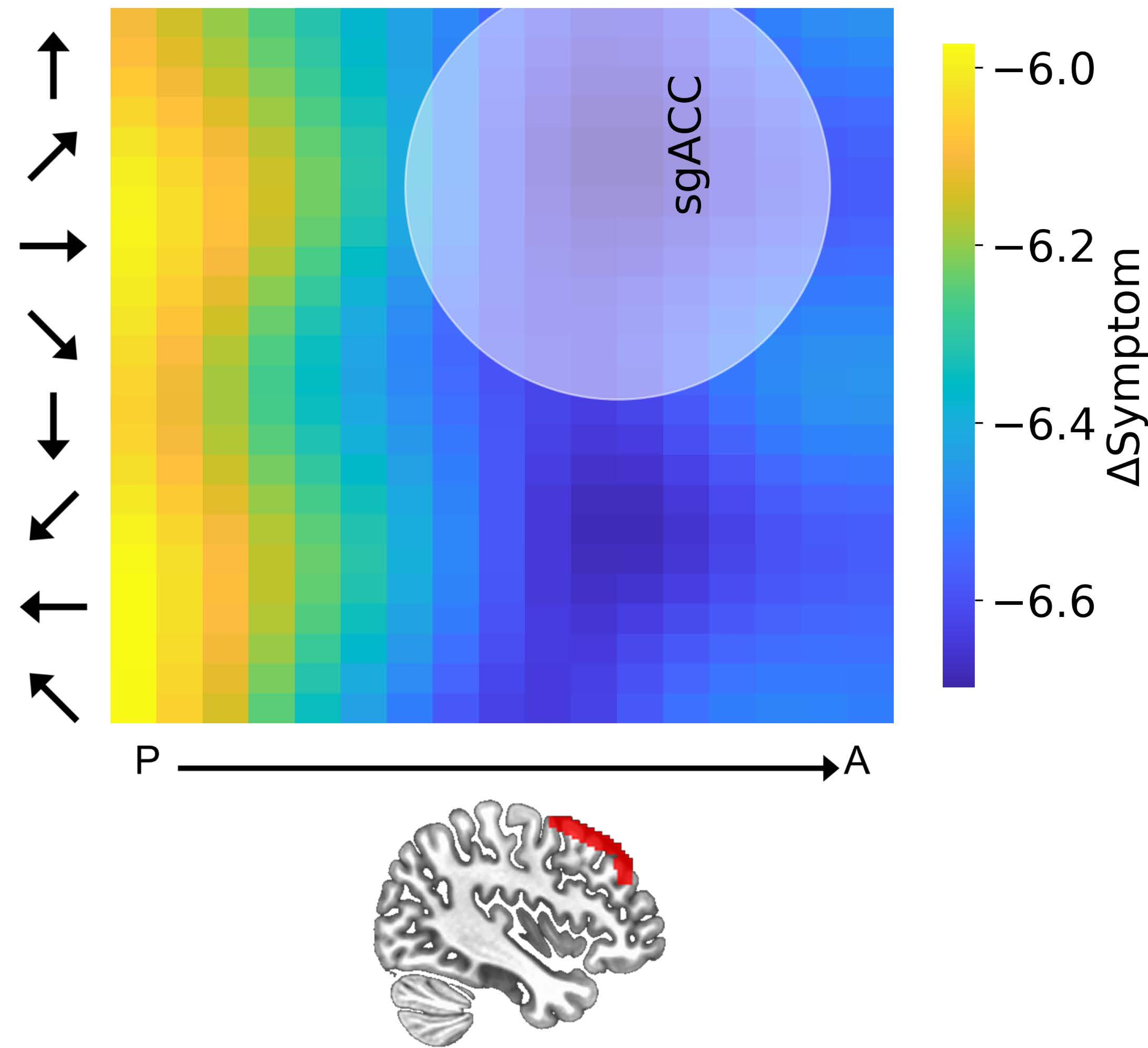

NDARDU203PAW_hamd_left_dlpfc

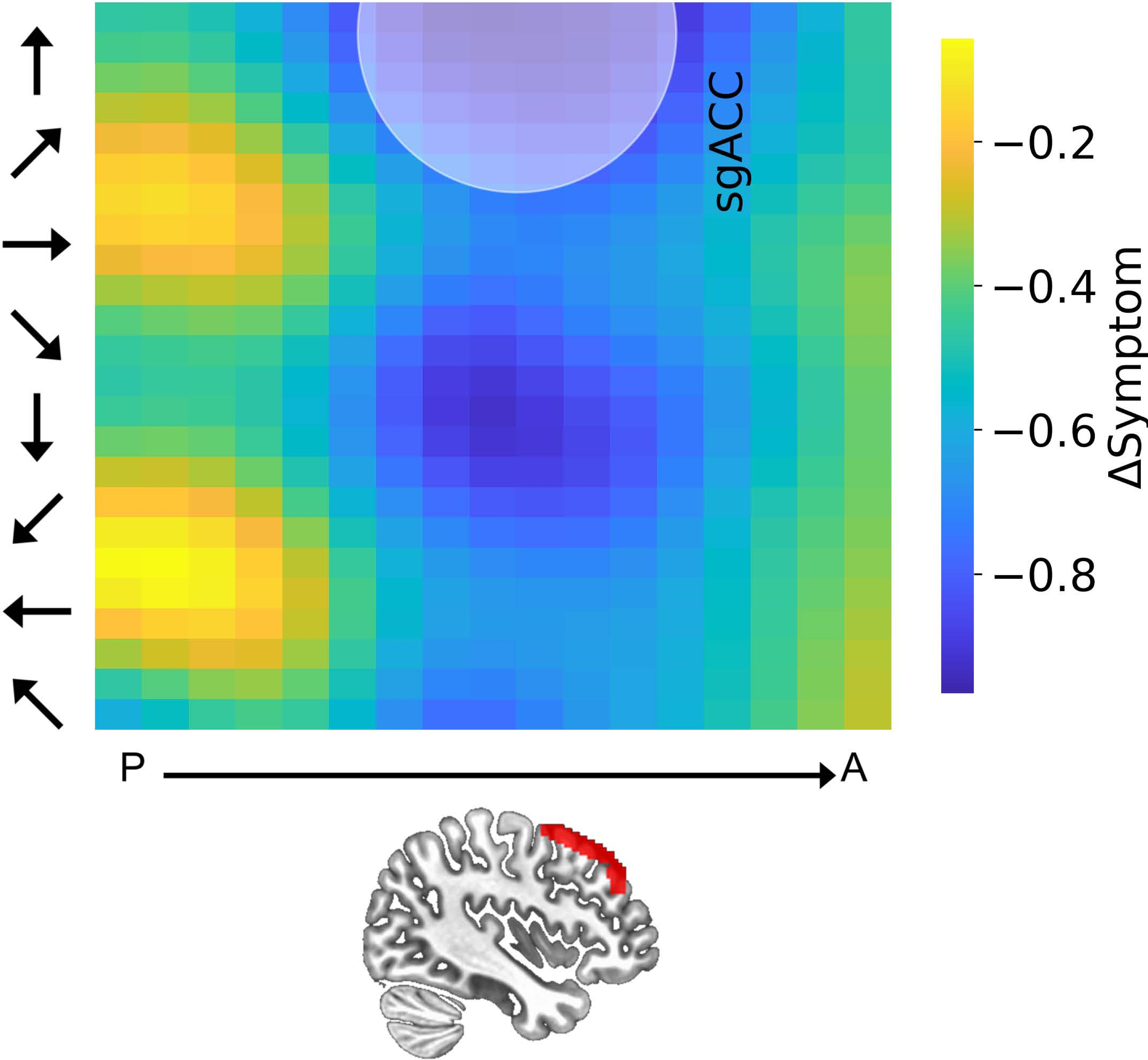

NDARDU282NKX_hamd_left_dlpfc

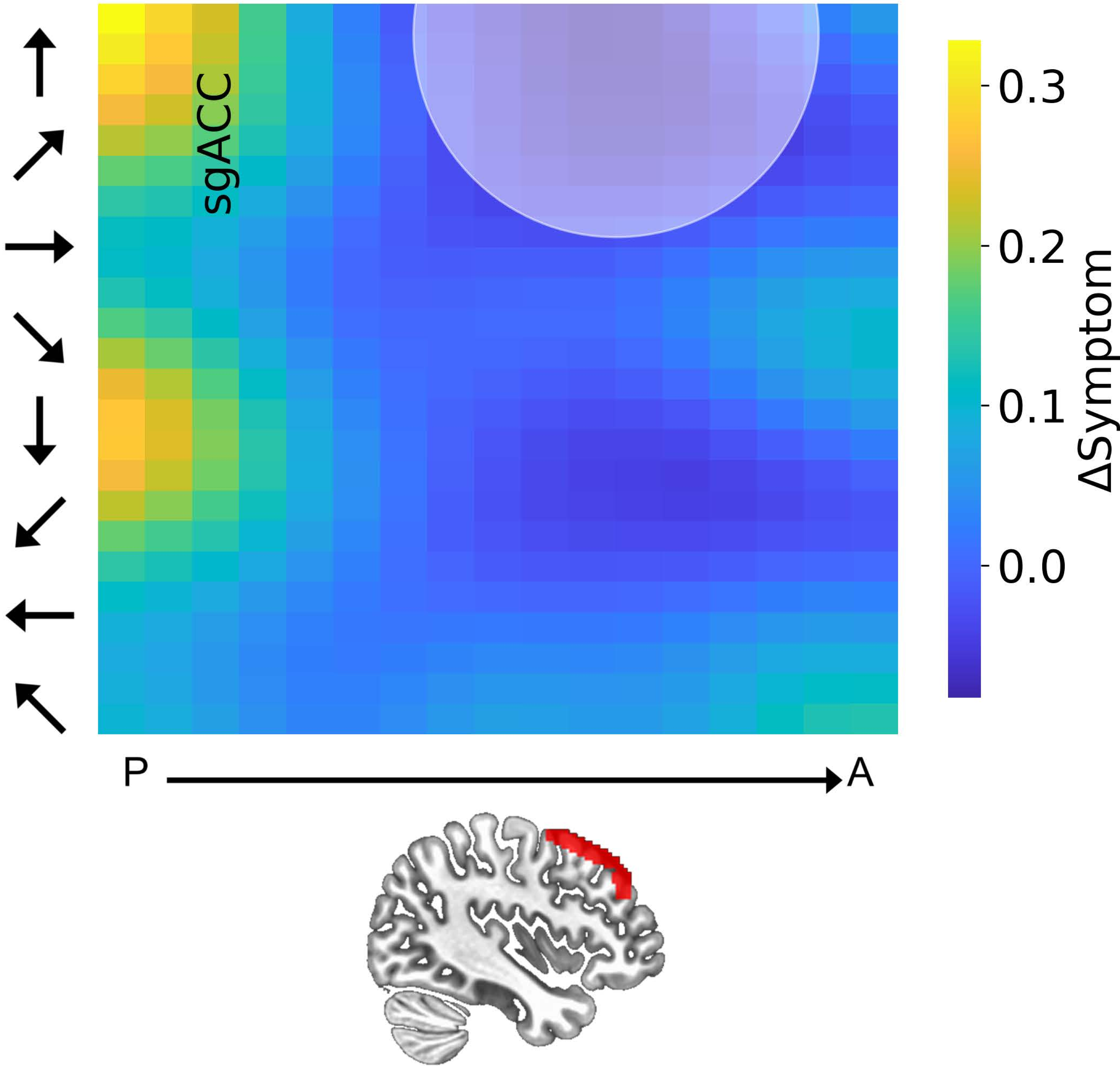

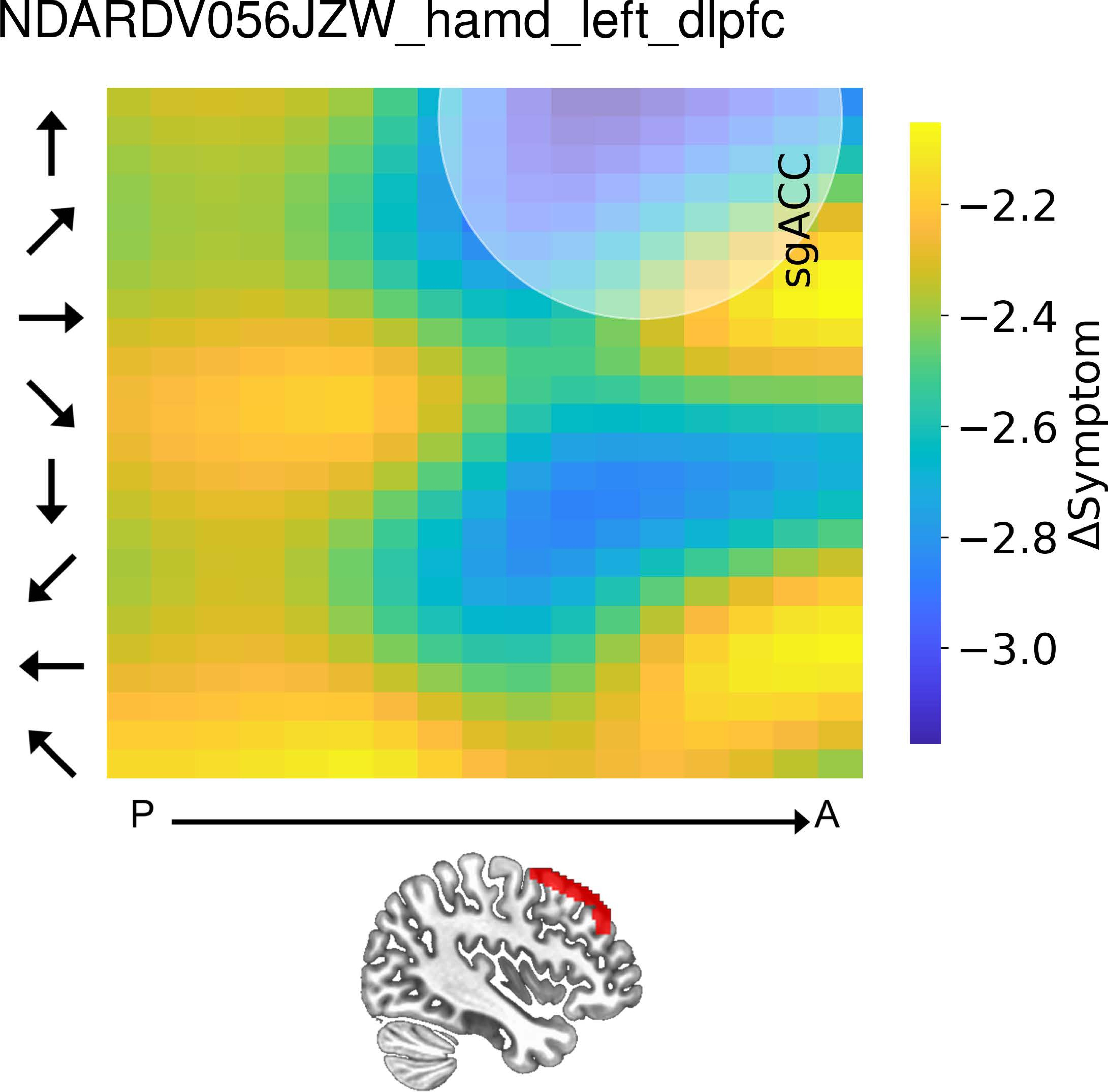
NDARDV056JZW_hamd_left_dlpfc
sgACC
−2.2
−2.4
−2.6
−2.8
−3.0
ΔSymptom
P
A

NDAREB933ZEU_hamd_left_dlpfc

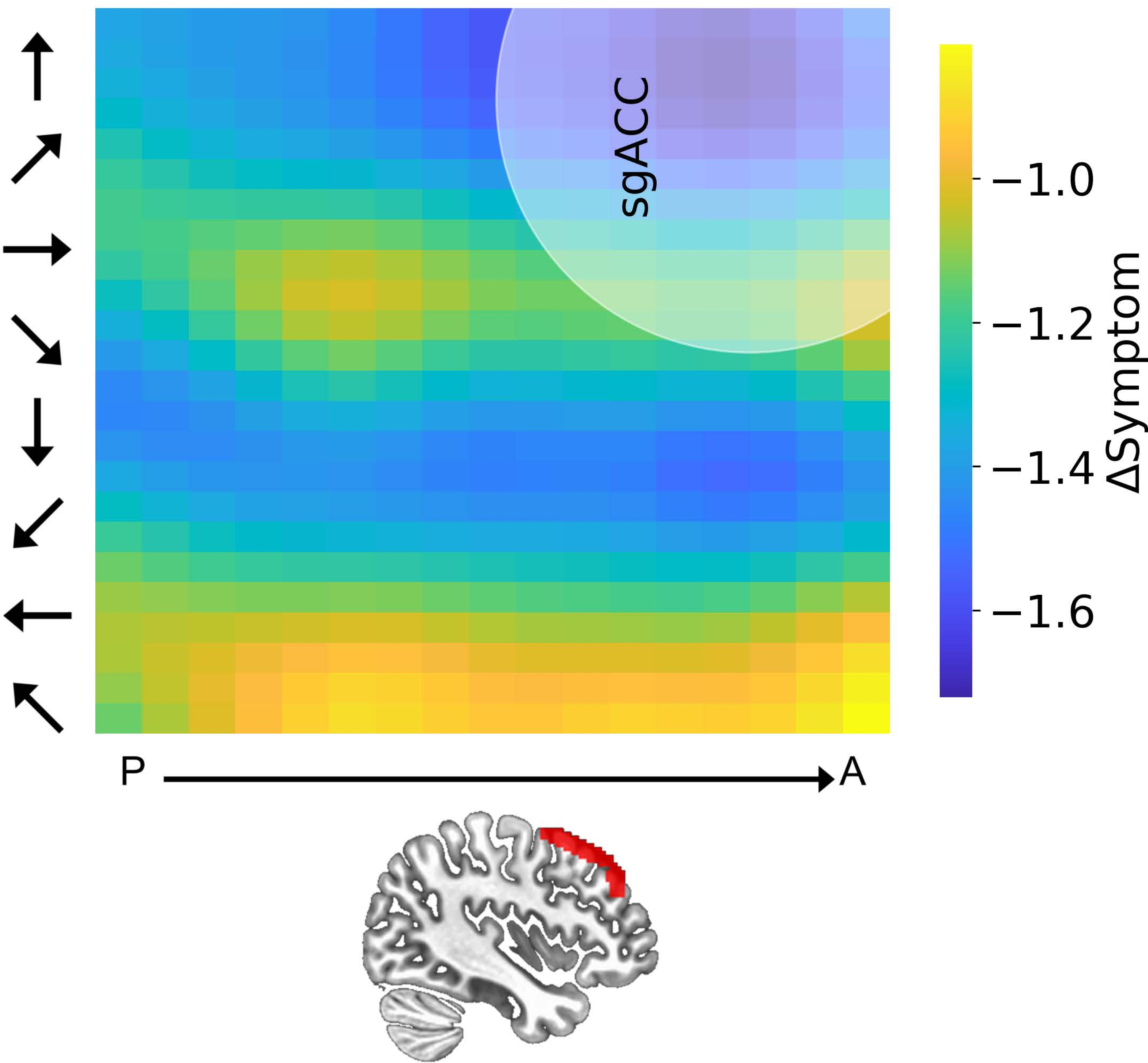

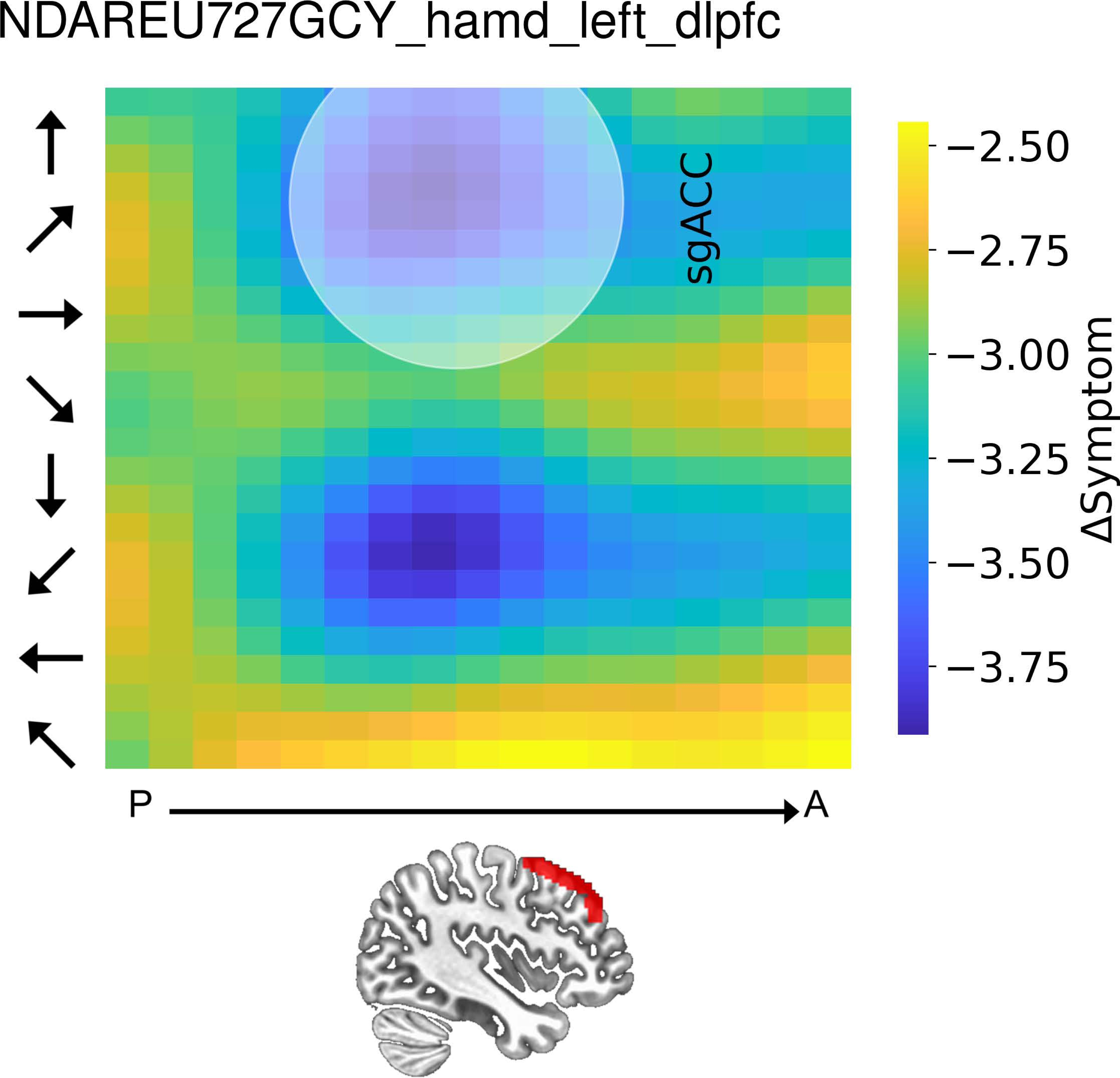
NDAREU727GCY_hamd_left_dlpfc
sgACC
−2.50
−2.75
−3.00
−3.25
−3.50
−3.75
ΔSymptom
P
A

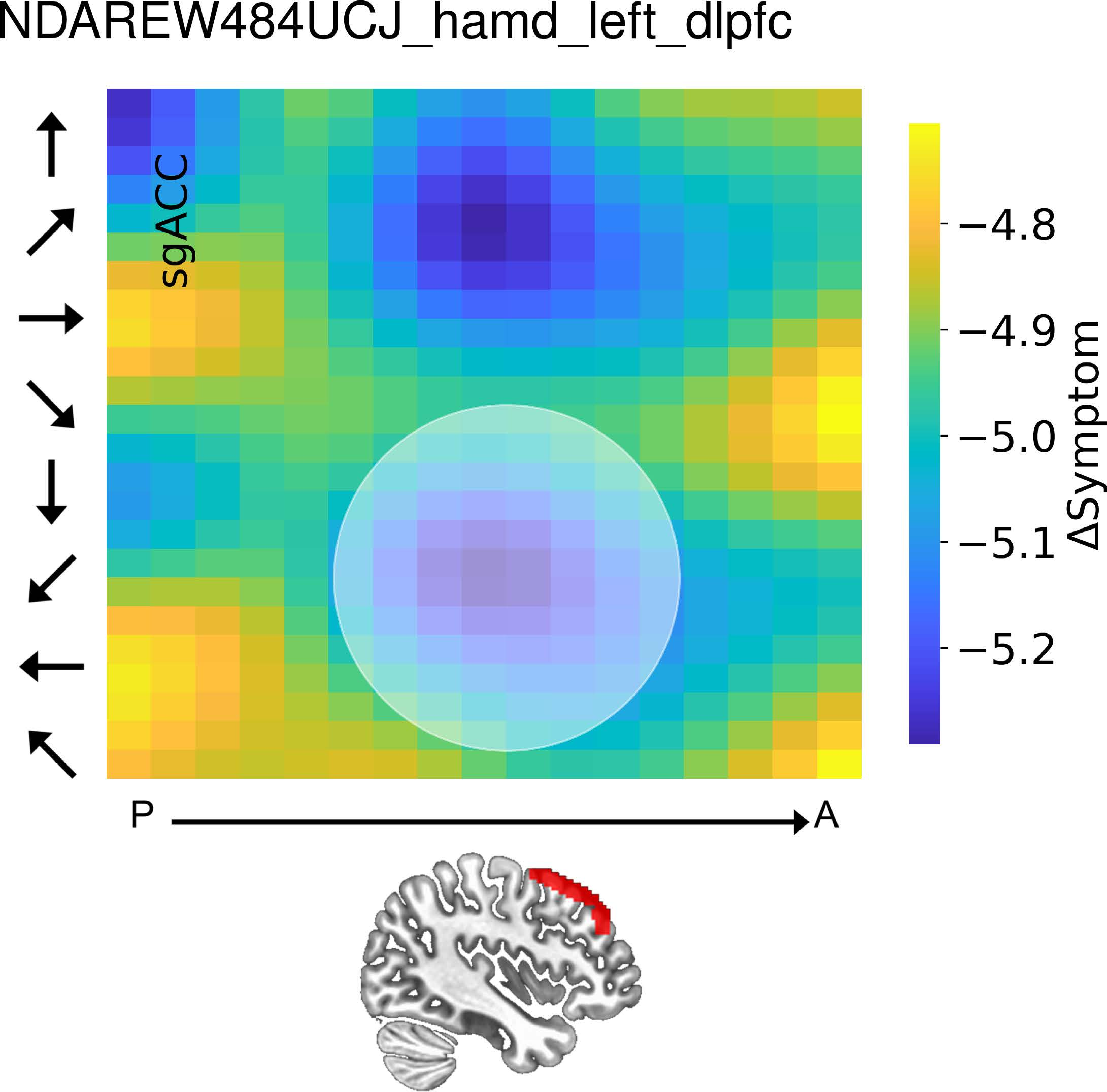
NDAREW484UCJ_hamd_left_dlpfc
sgACC
P
A
−4.8
−4.9
−5.0
−5.1
−5.2
ΔSymptom

NDAREZ915JK5_hamd_left_dlpfc

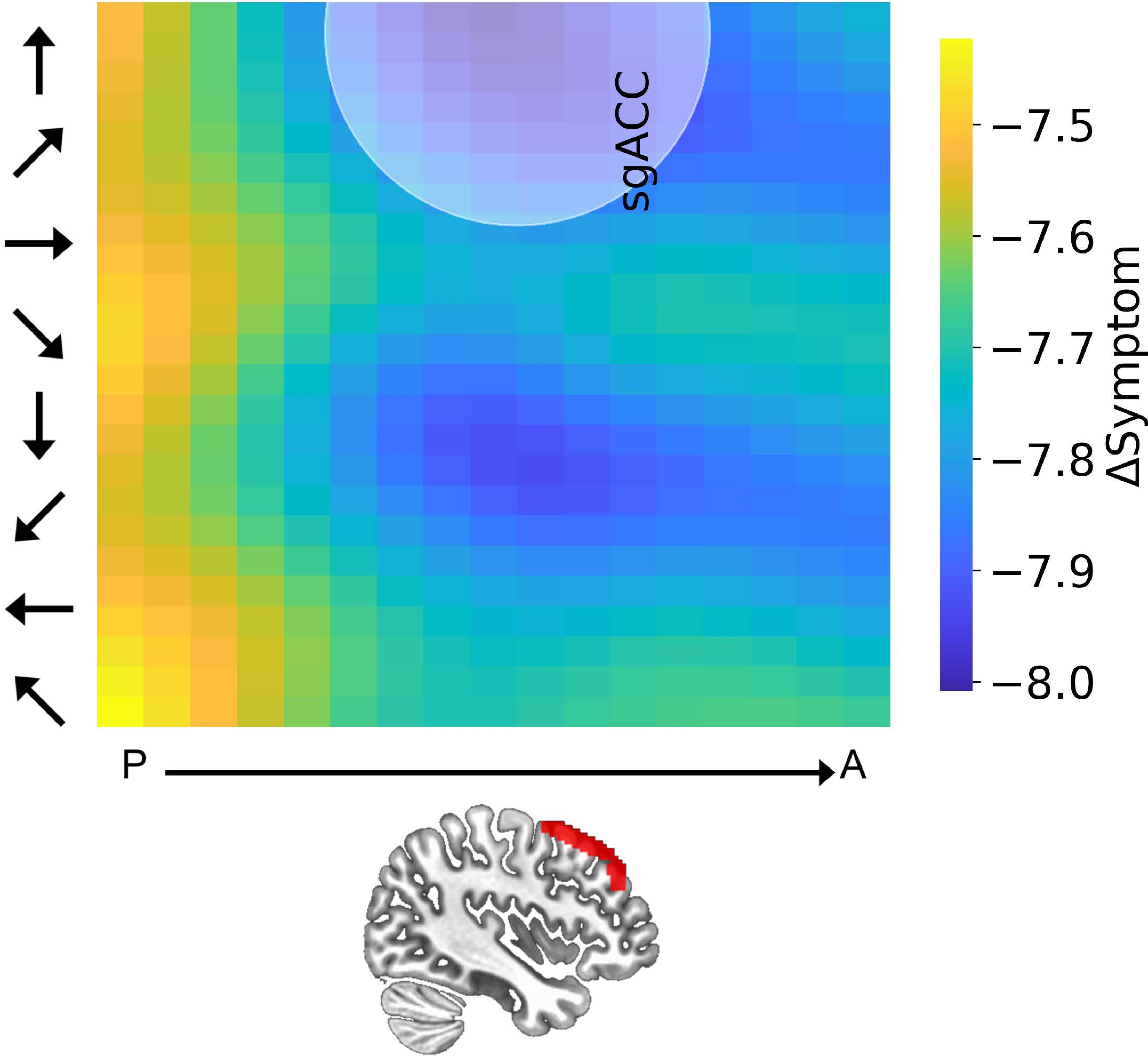

NDARFH525MFZ_hamd_left_dlpfc

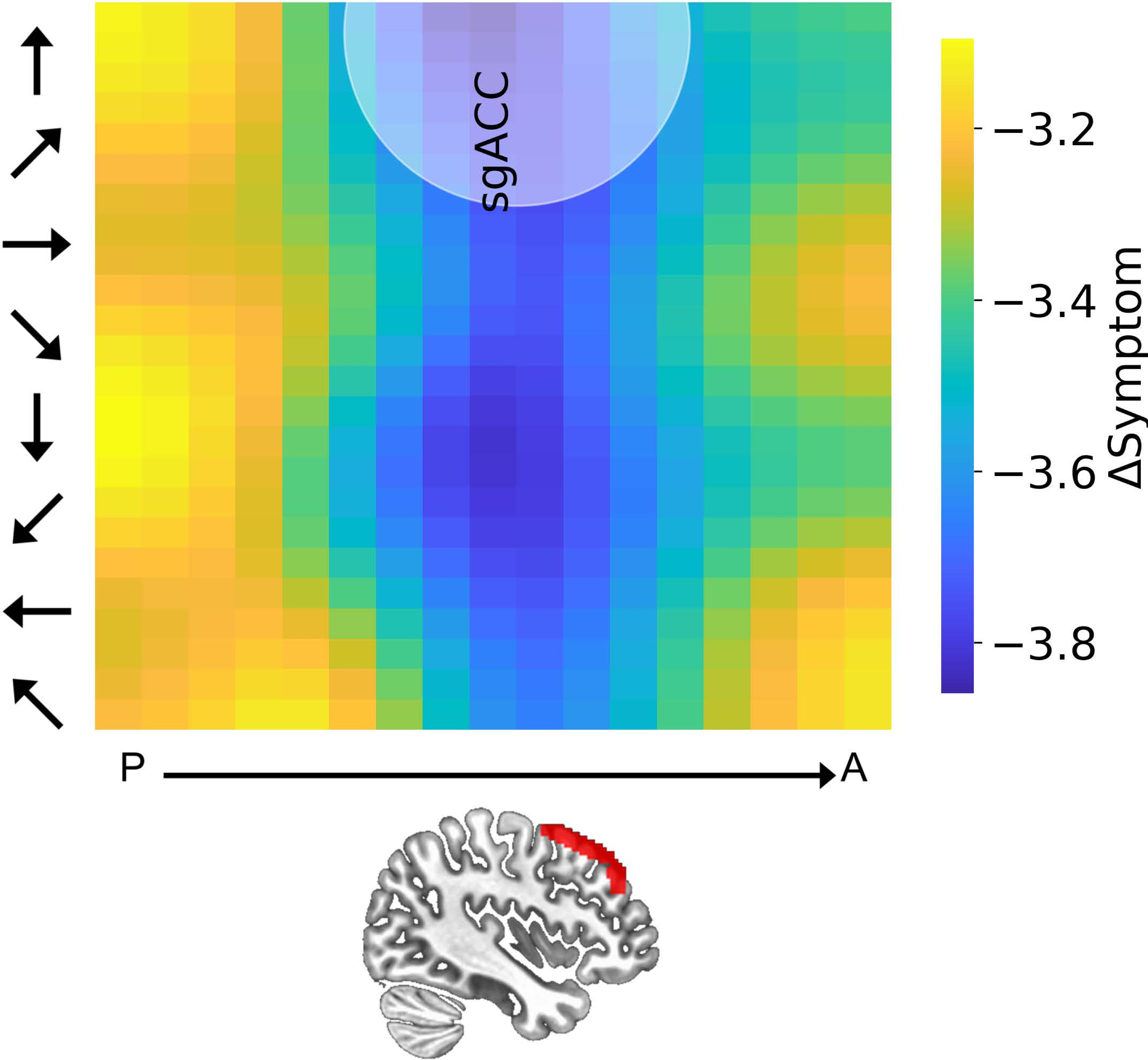

NDARFR746ENQ_hamd_left_dlpfc

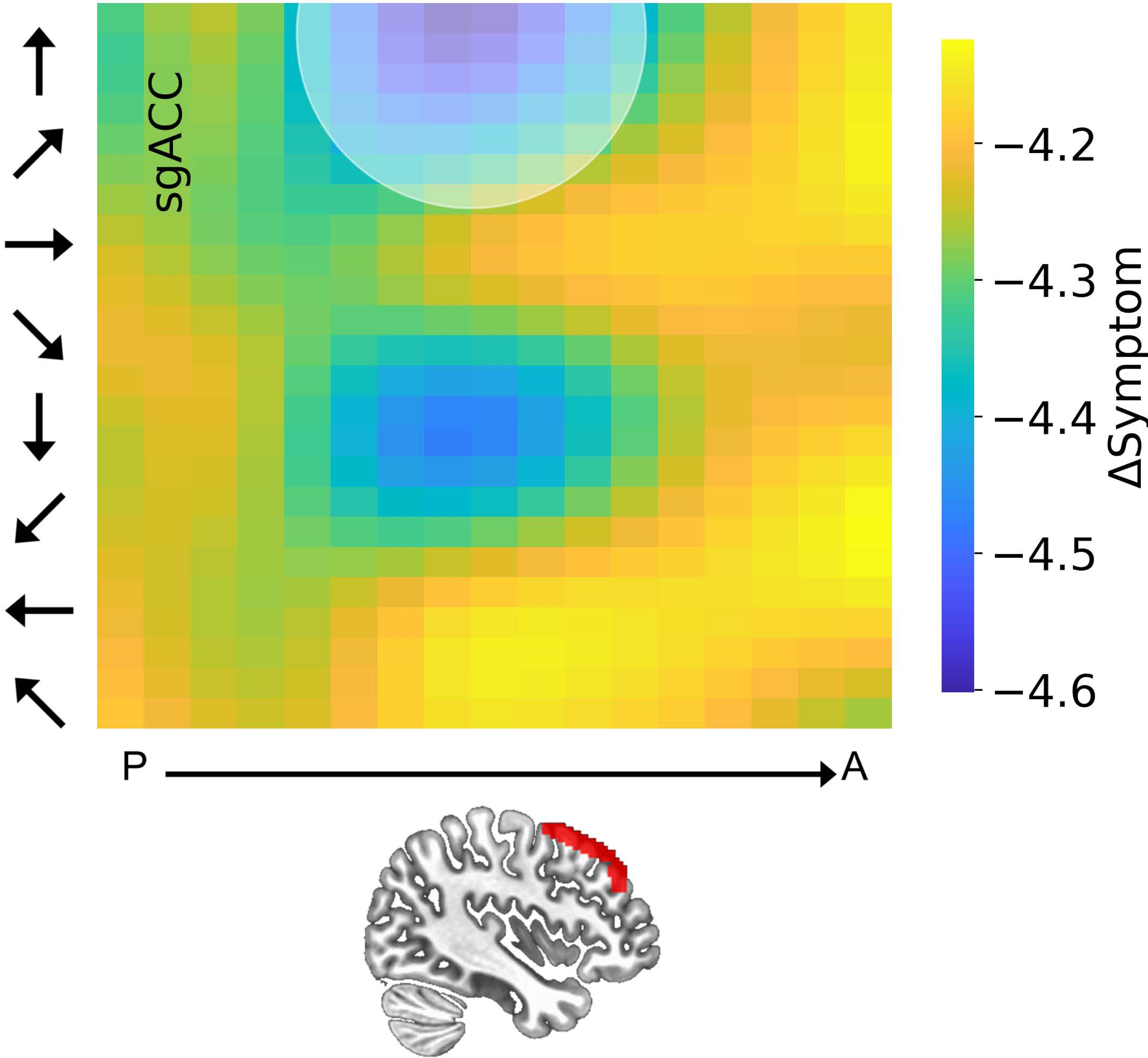

NDARFW096LMD_hamd_left_dlpfc

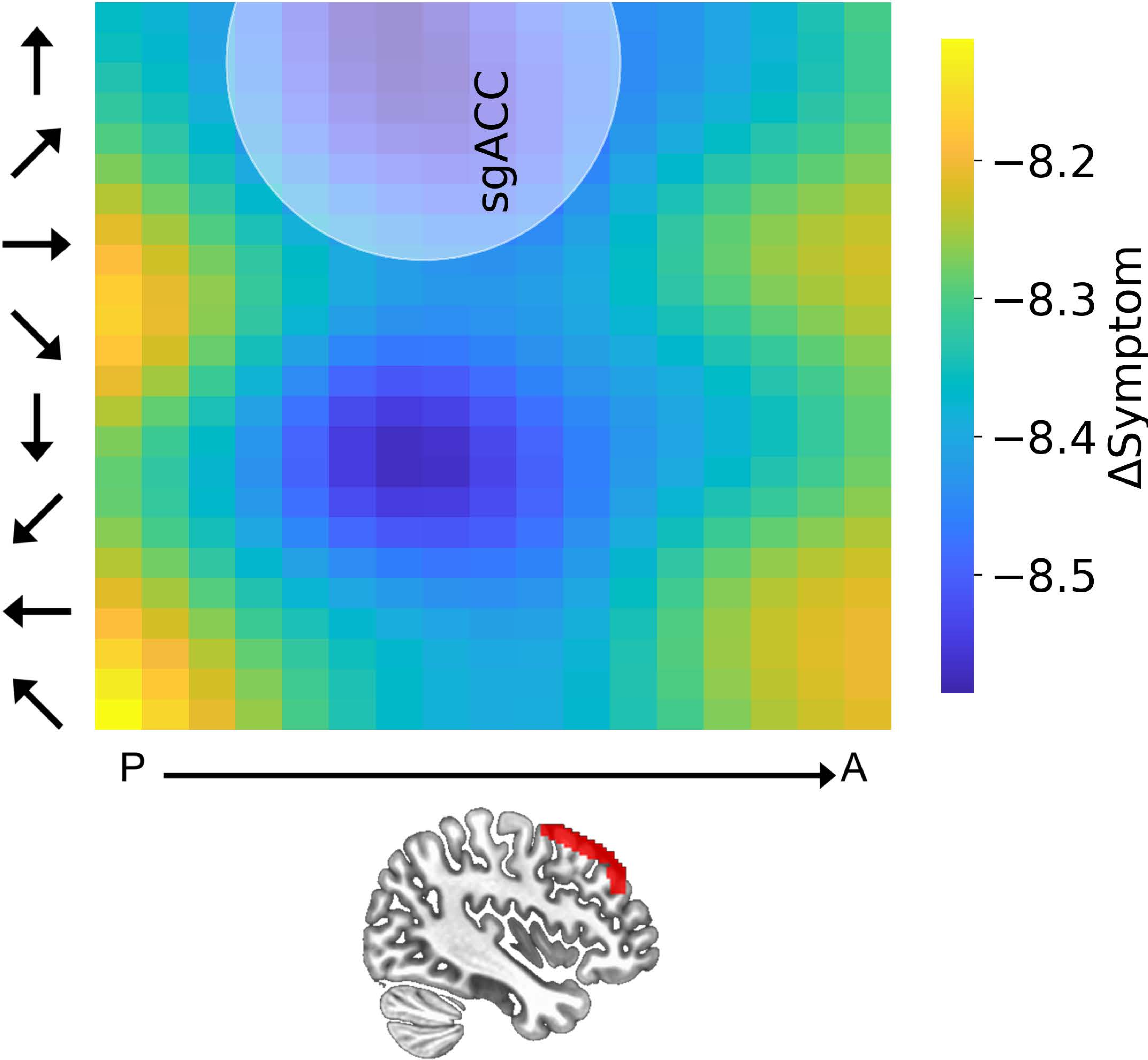

NDARGH895WJJ_hamd_left_dlpfc

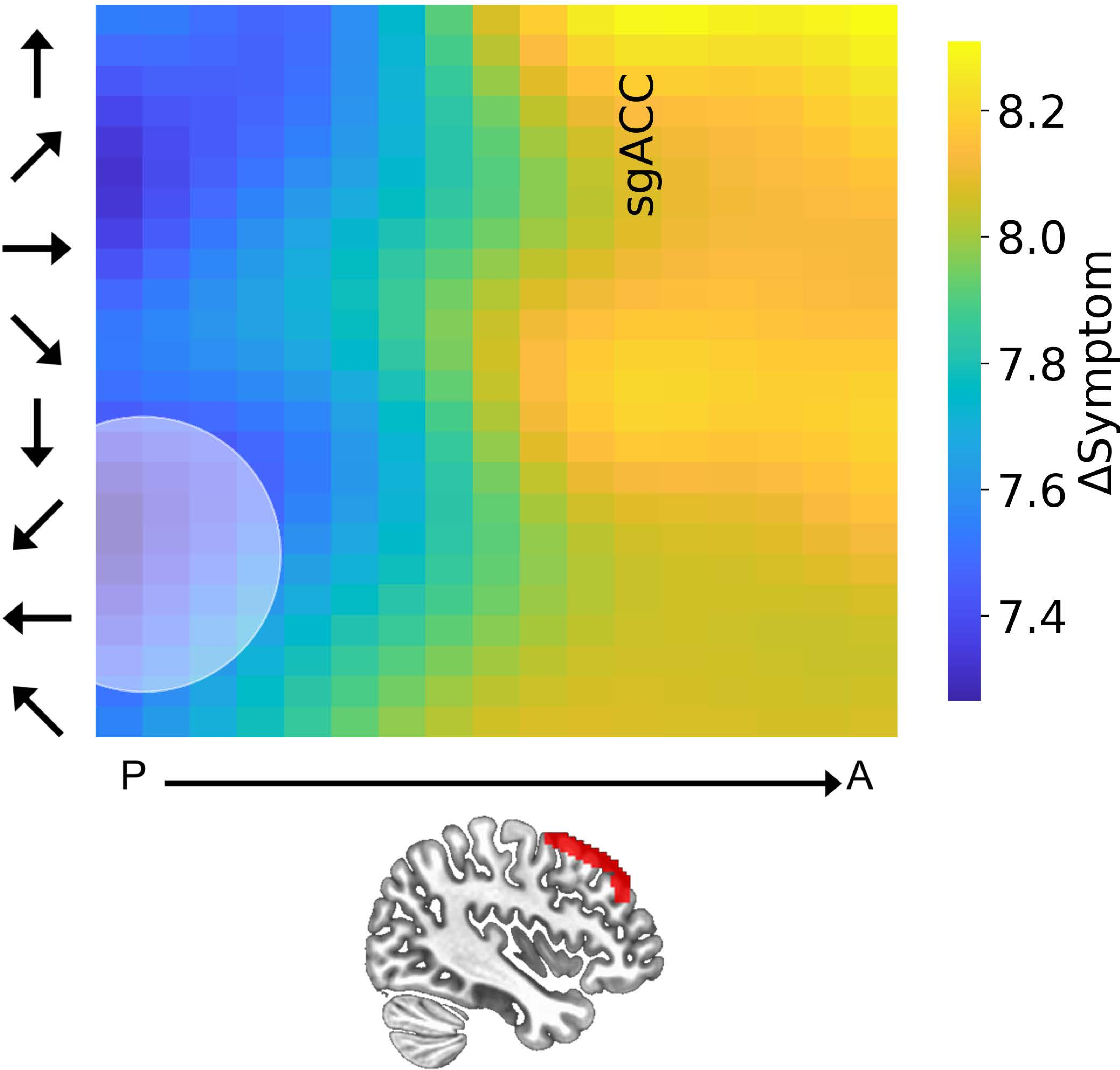

NDARGR637FTT_hamd_left_dlpfc

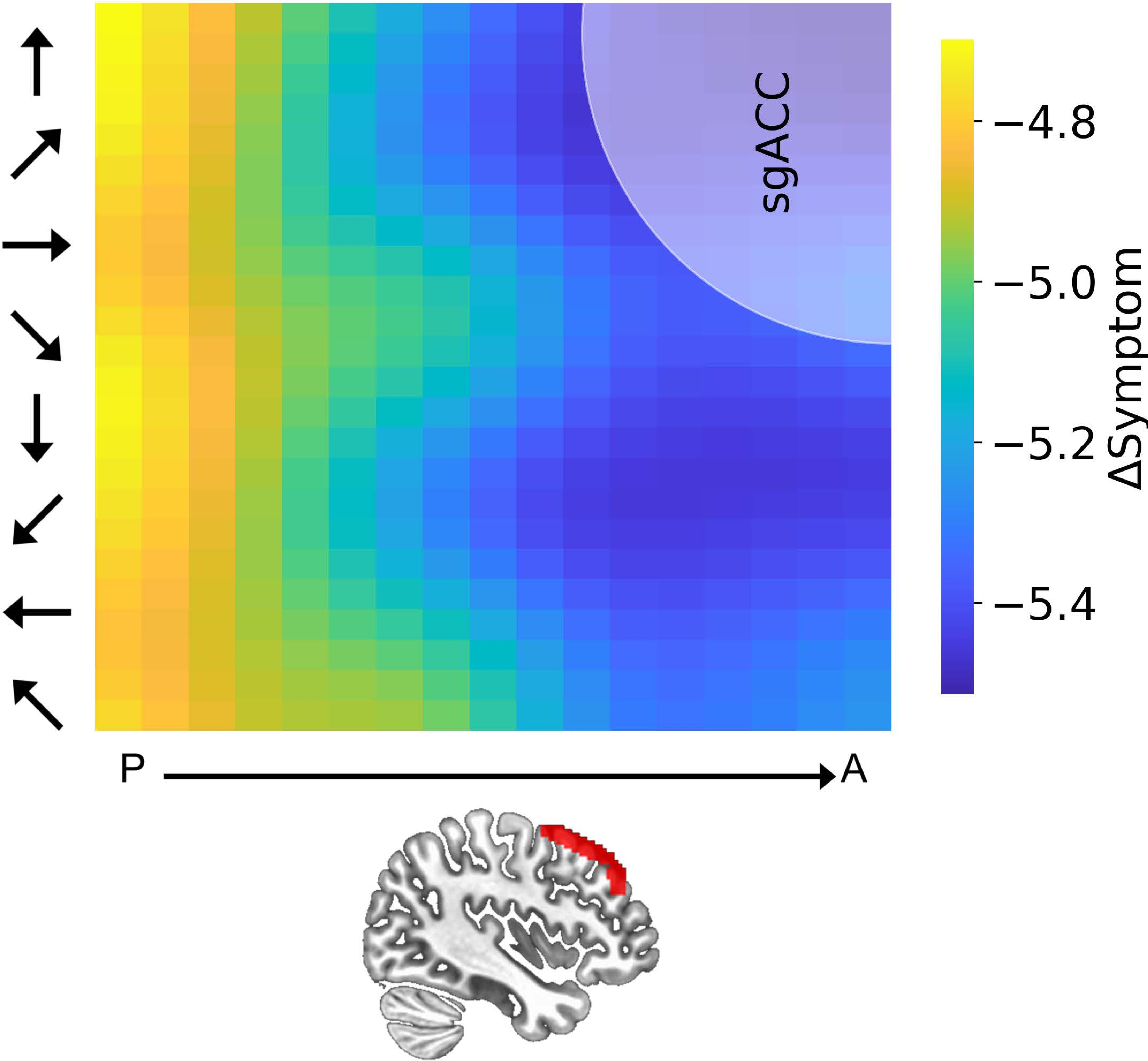

NDARGR782ALD_hamd_left_dlpfc

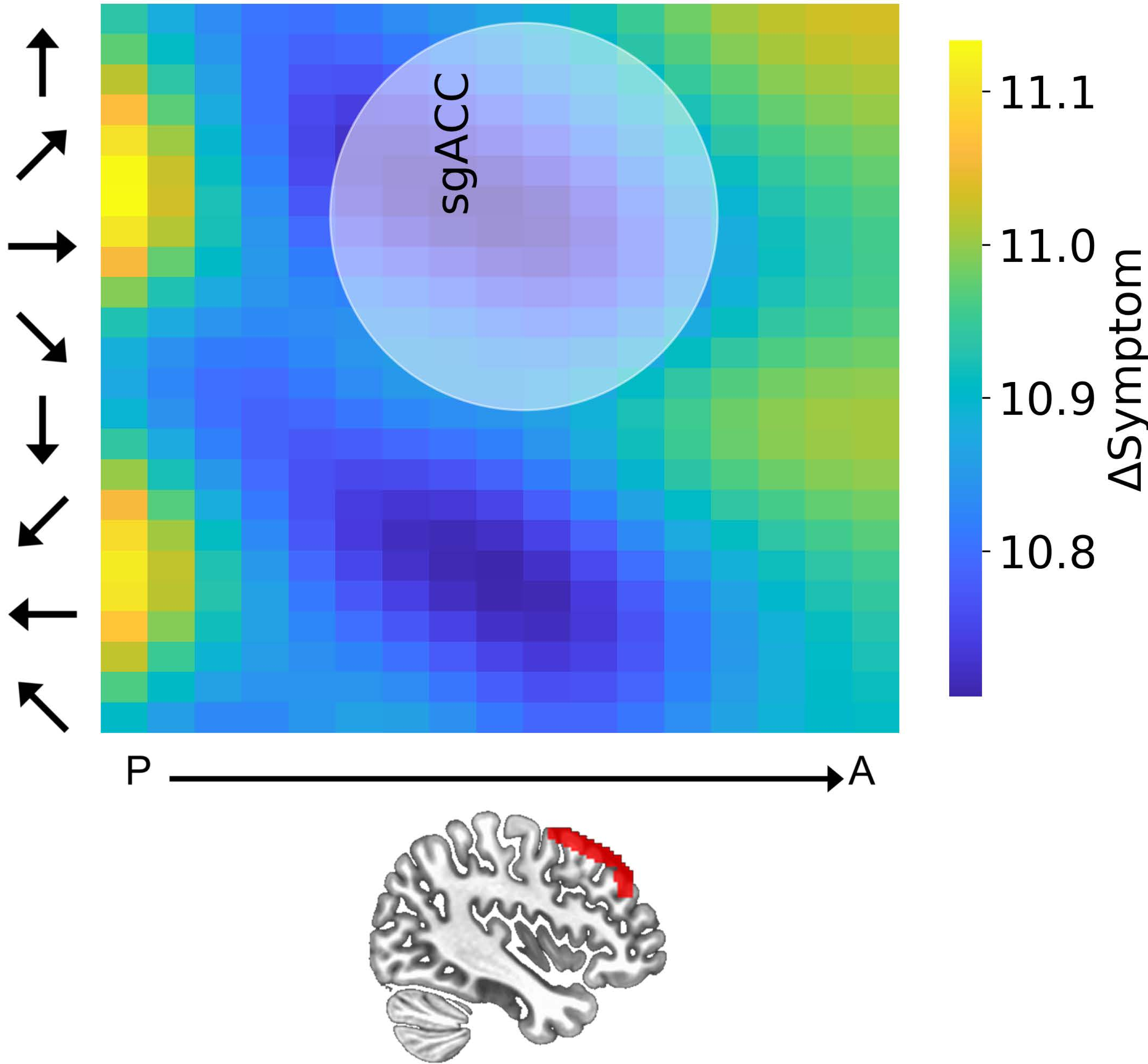

NDARHA109LH7_hamd_left_dlpfc

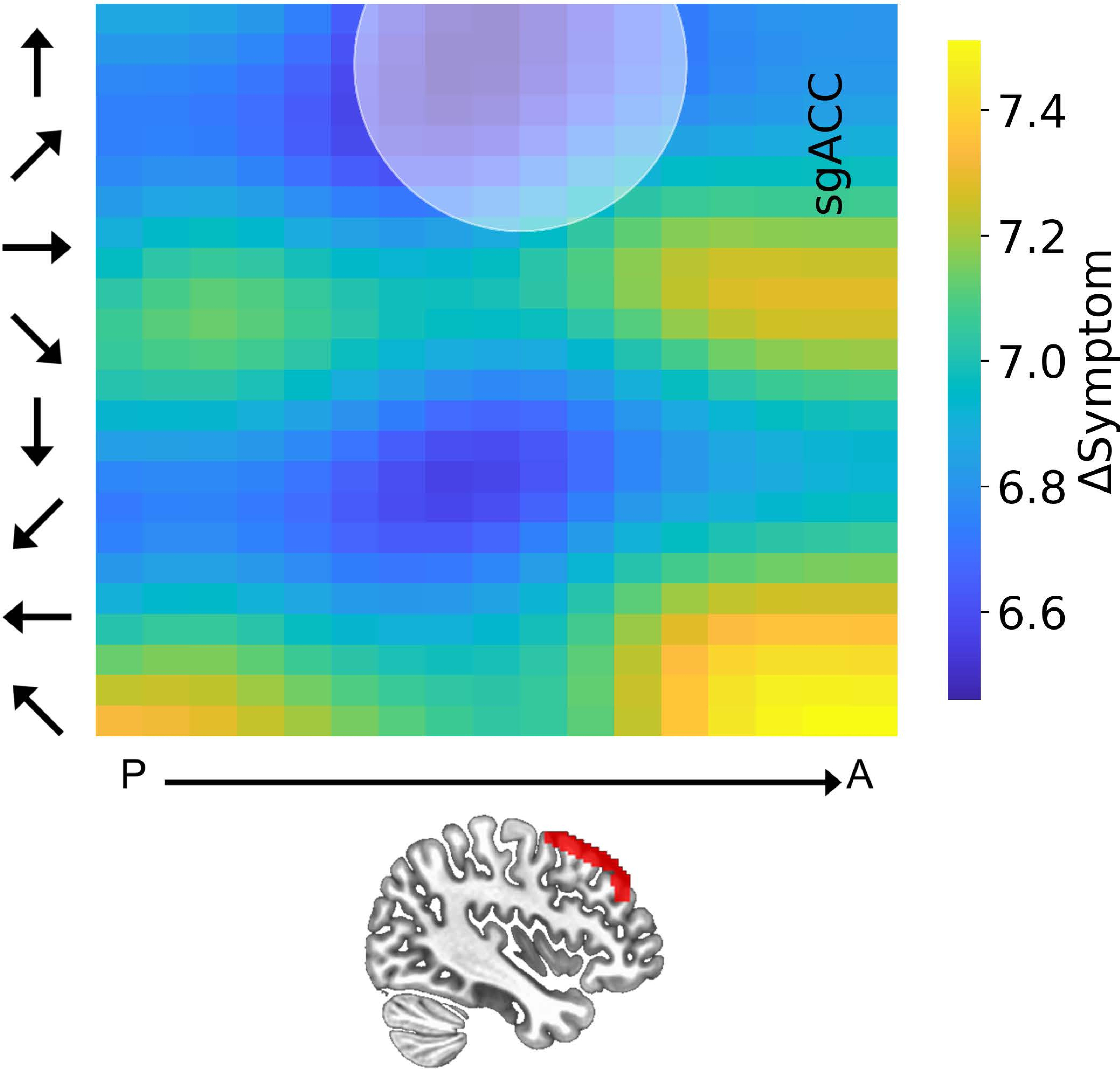

NDARHR981AP1_hamd_left_dlpfc

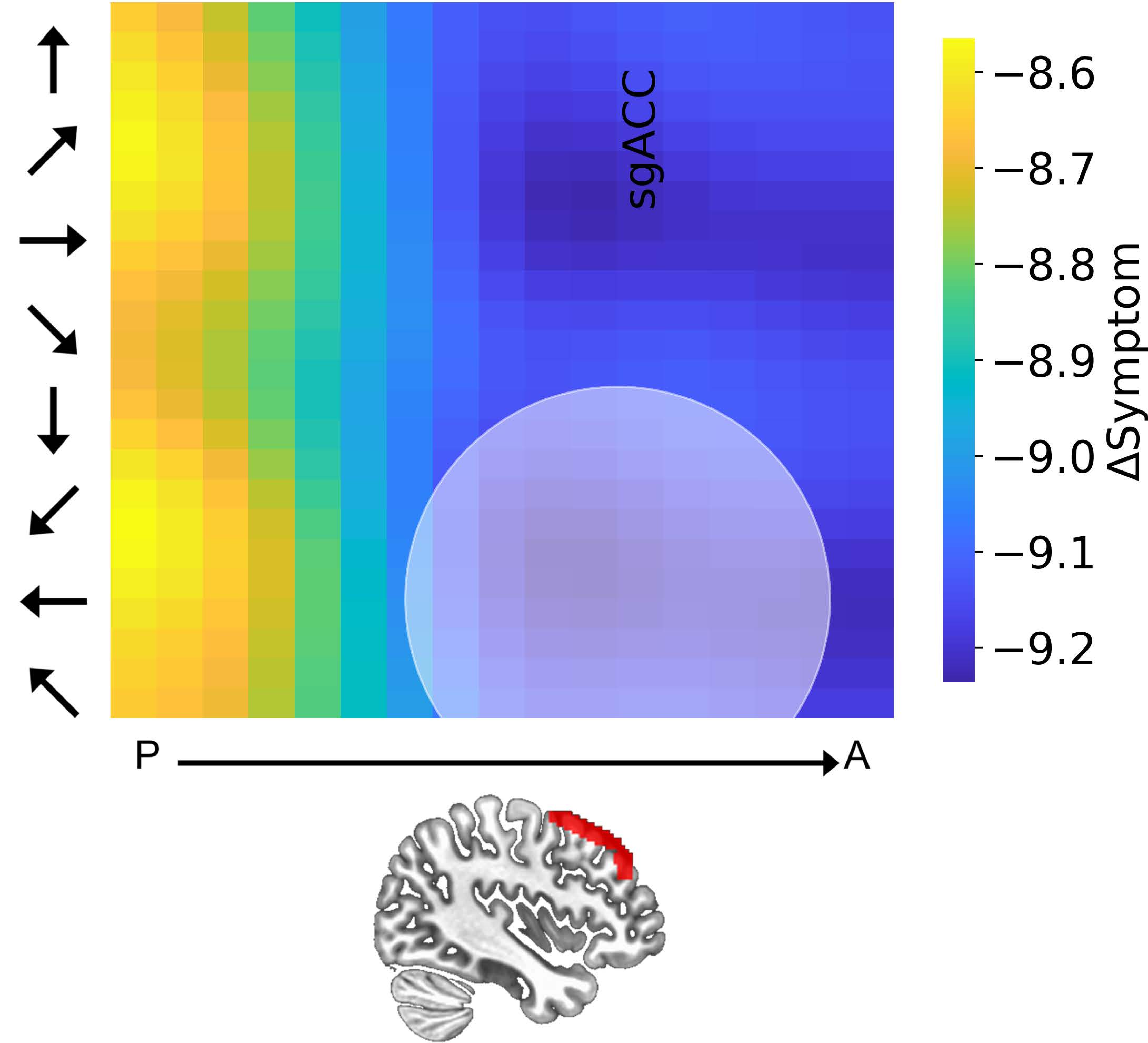

NDARHX487CT9_hamd_left_dlpfc

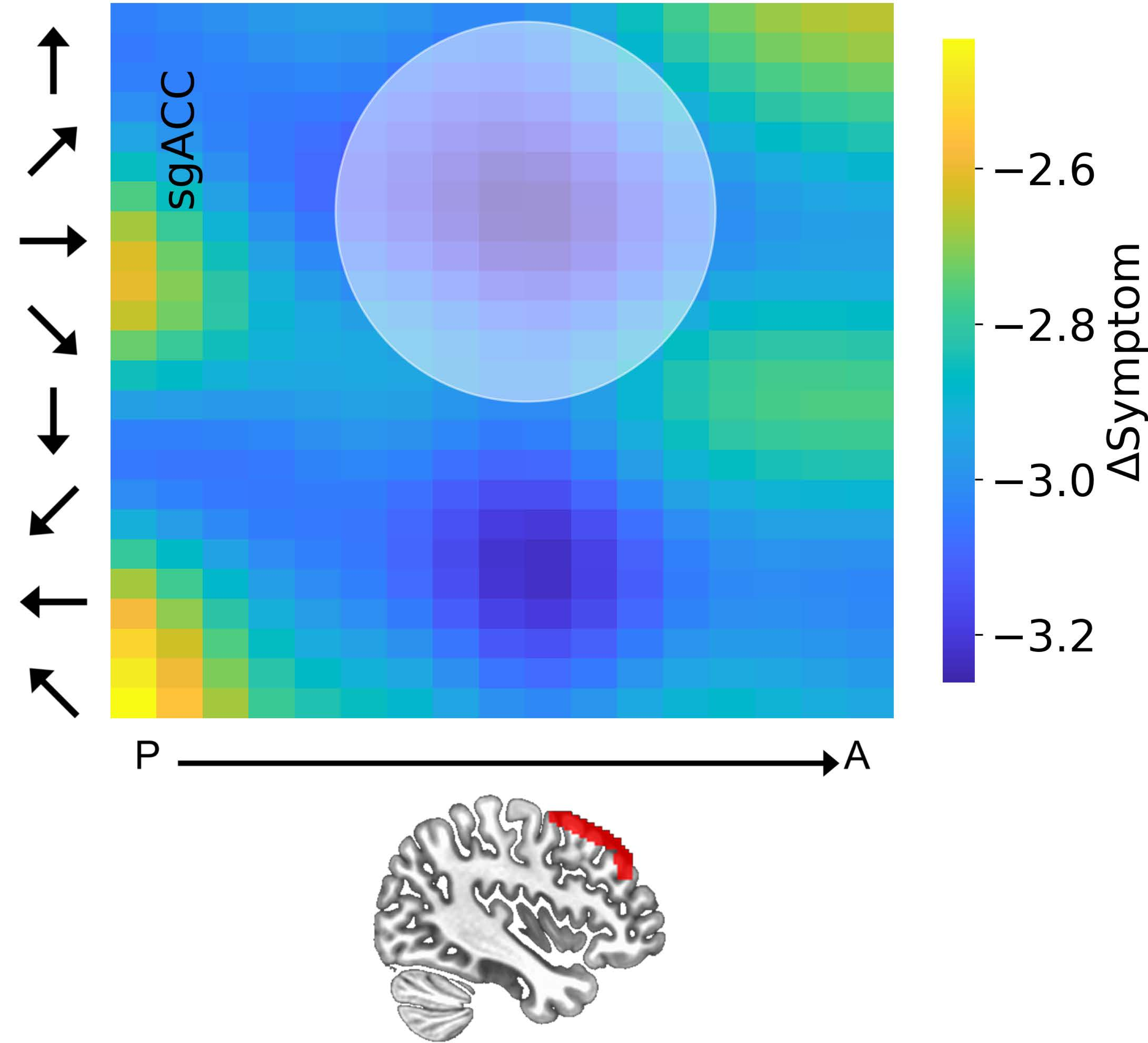

NDARHZ301NUW_hamd_left_dlpfc

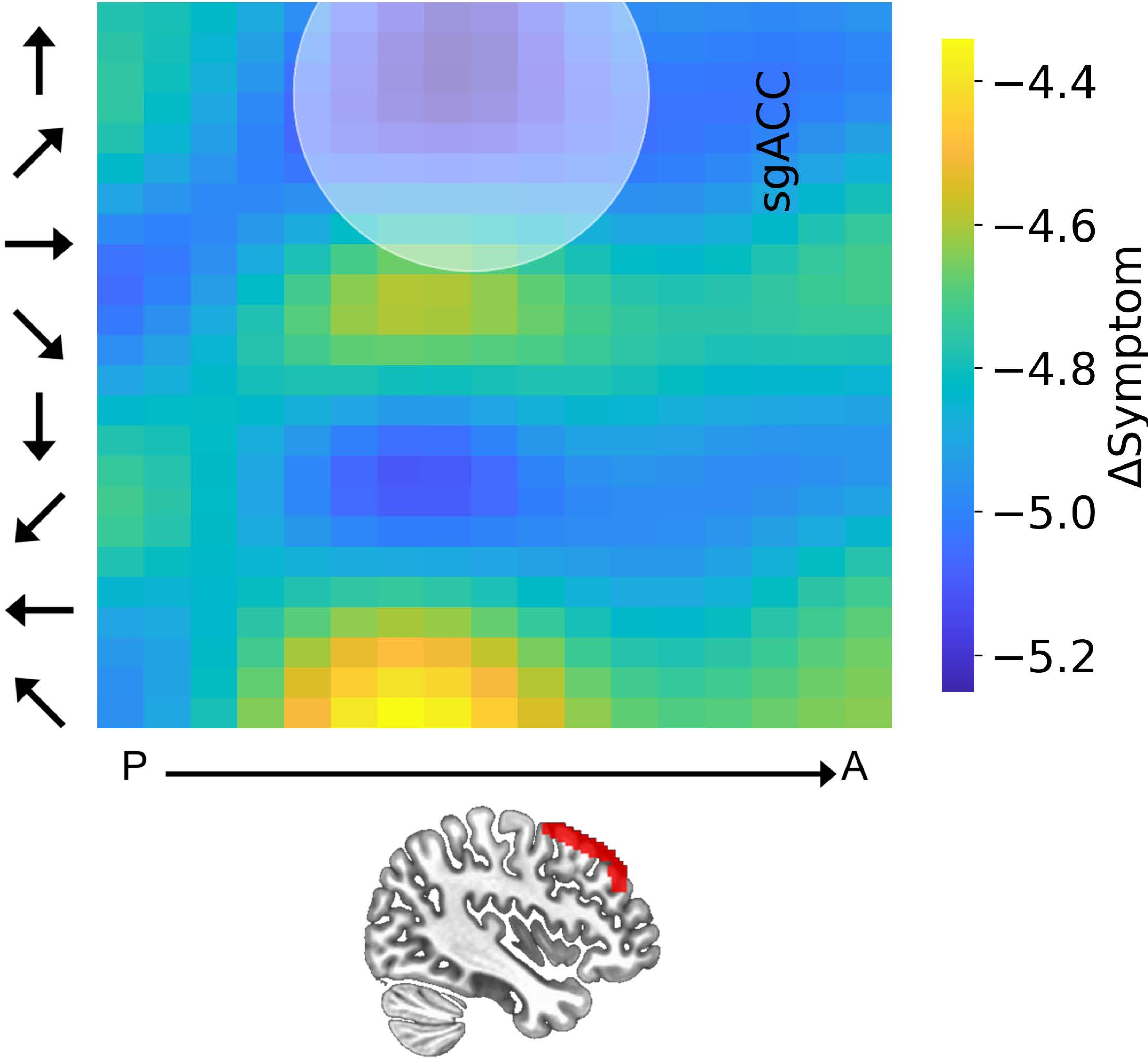

NDARHZ662EPK_hamd_left_dlpfc

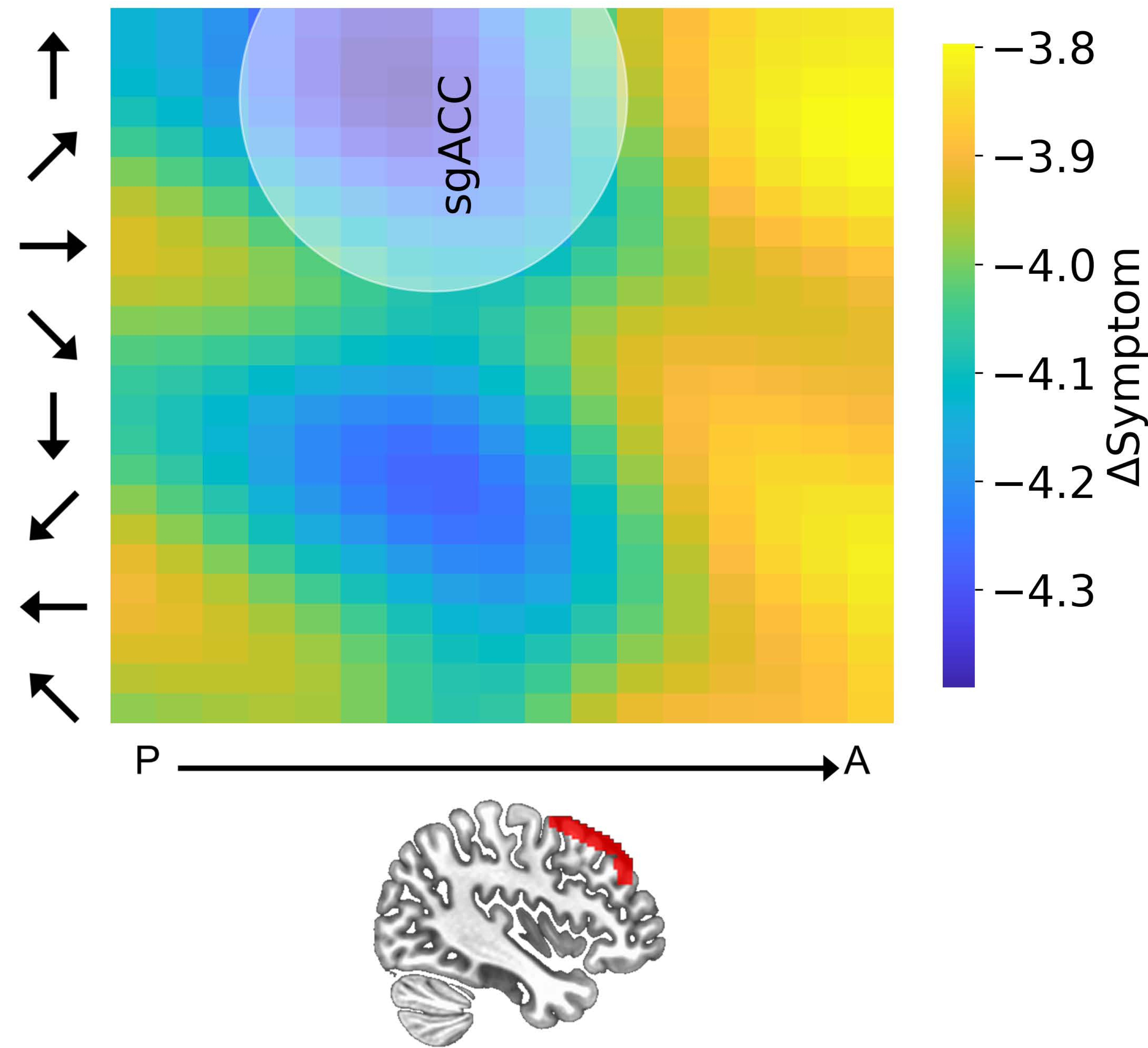

NDARJD271PMF_hamd_left_dlpfc

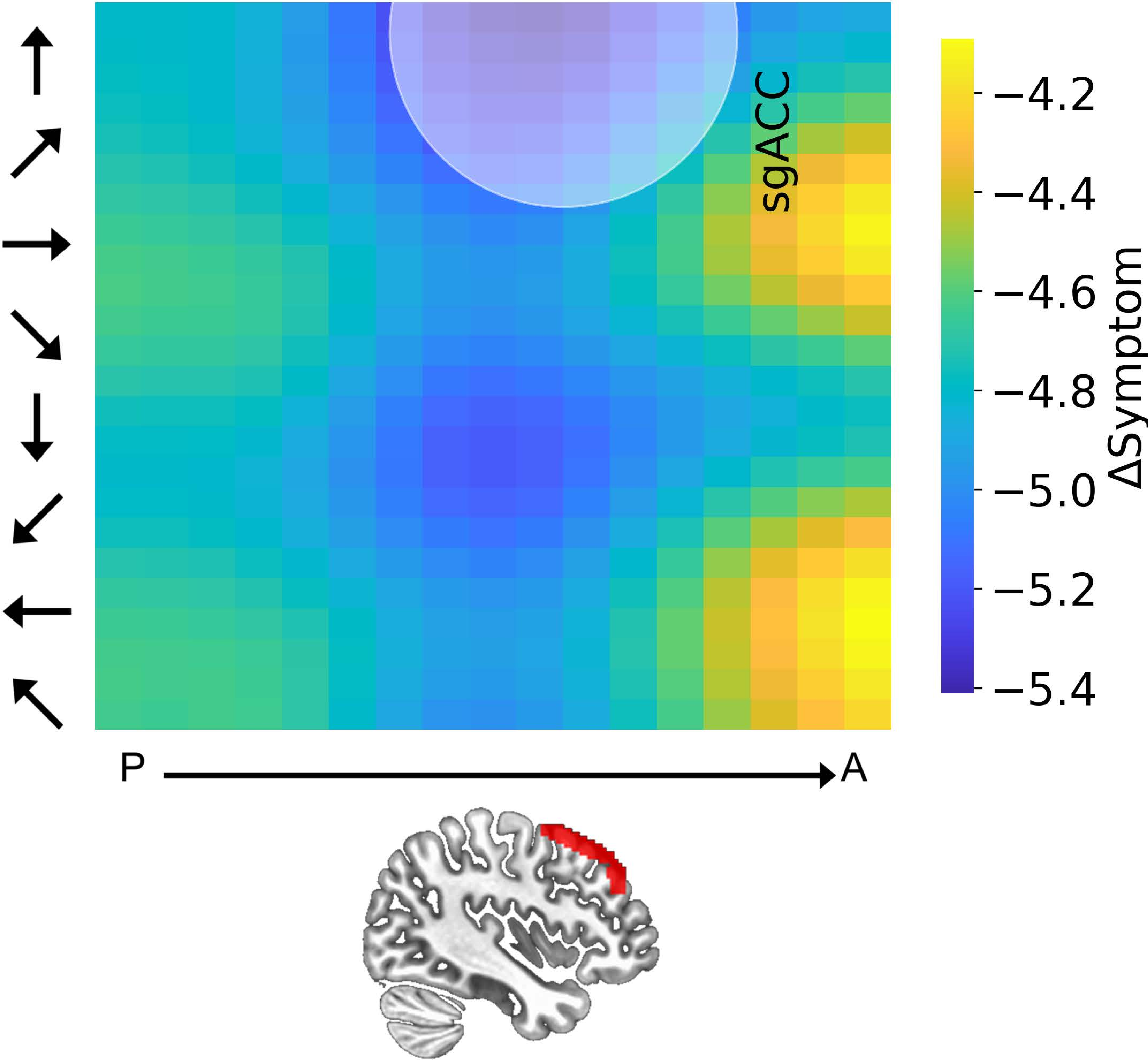

NDARJD548GTC_hamd_left_dlpfc

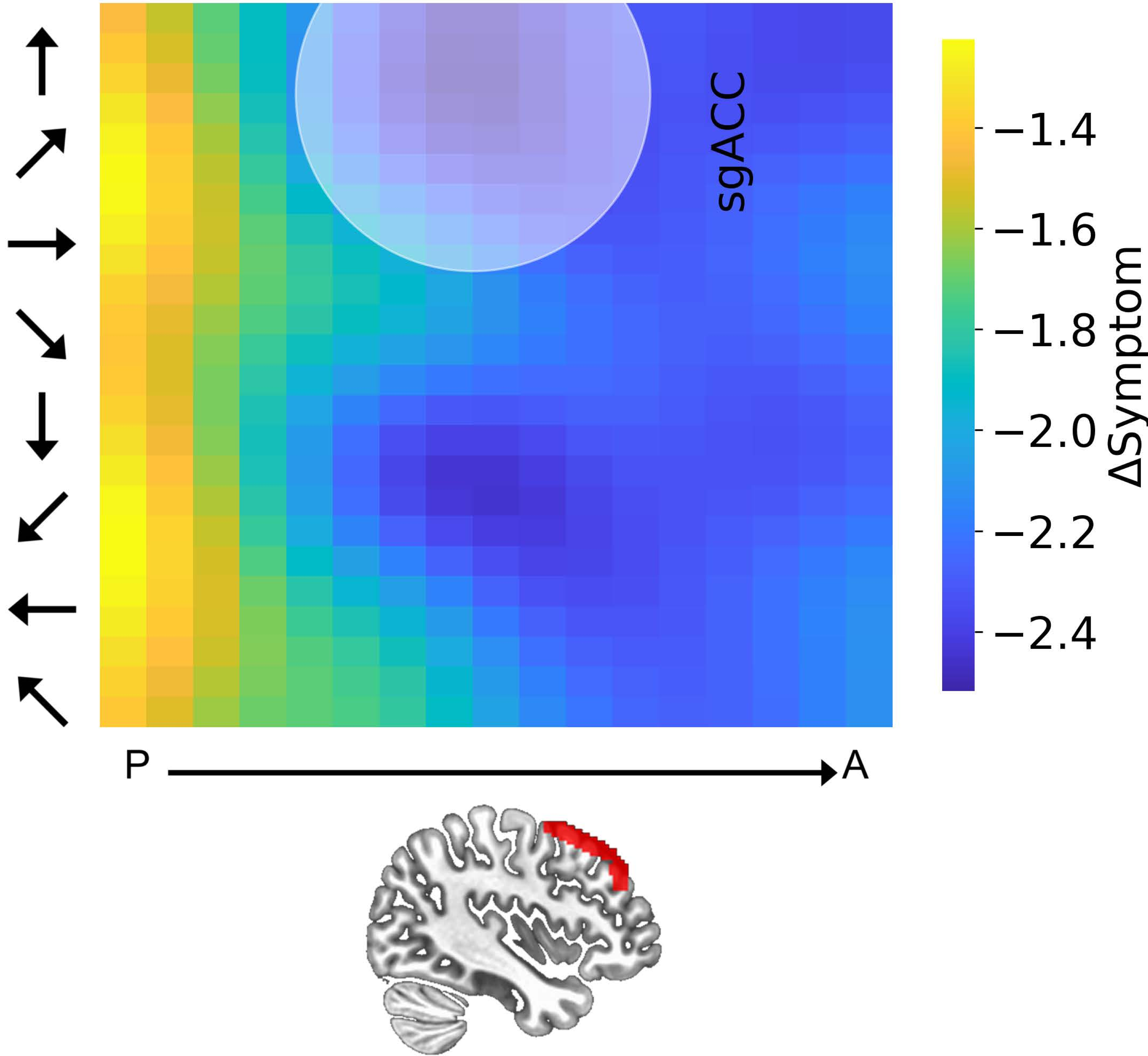

NDARJF993NV1_hamd_left_dlpfc

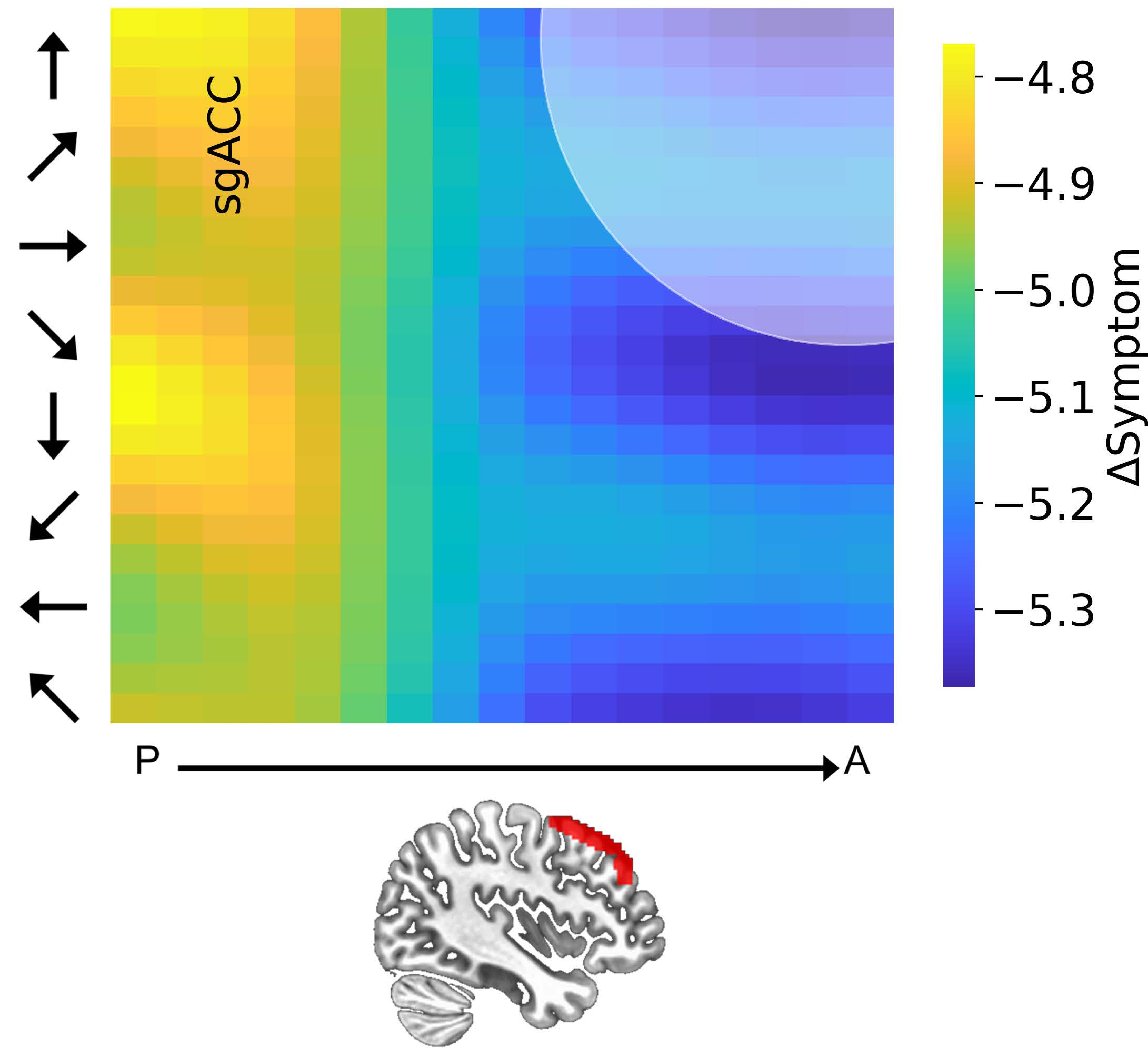

NDARJH406WC9_hamd_left_dlpfc

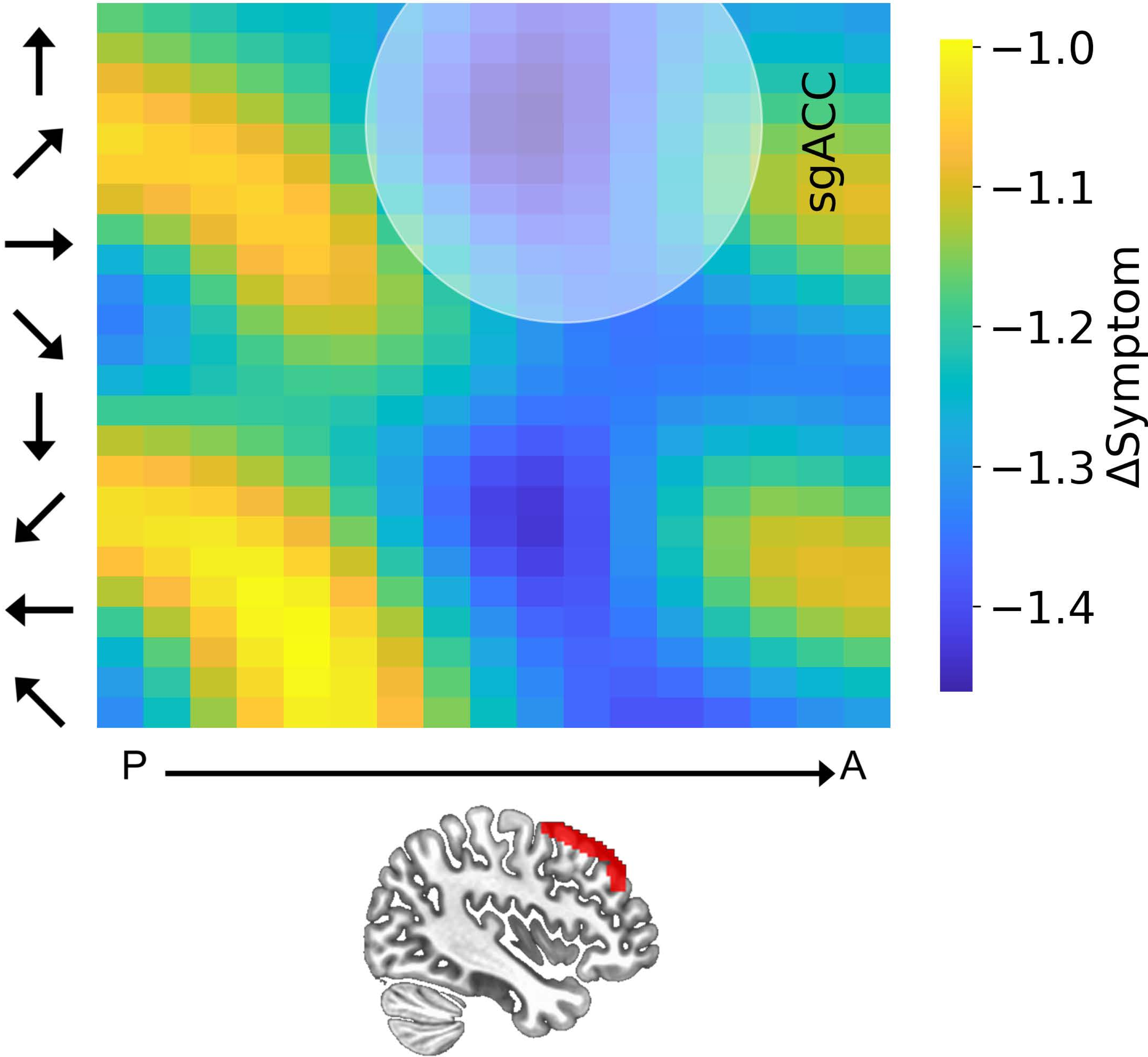

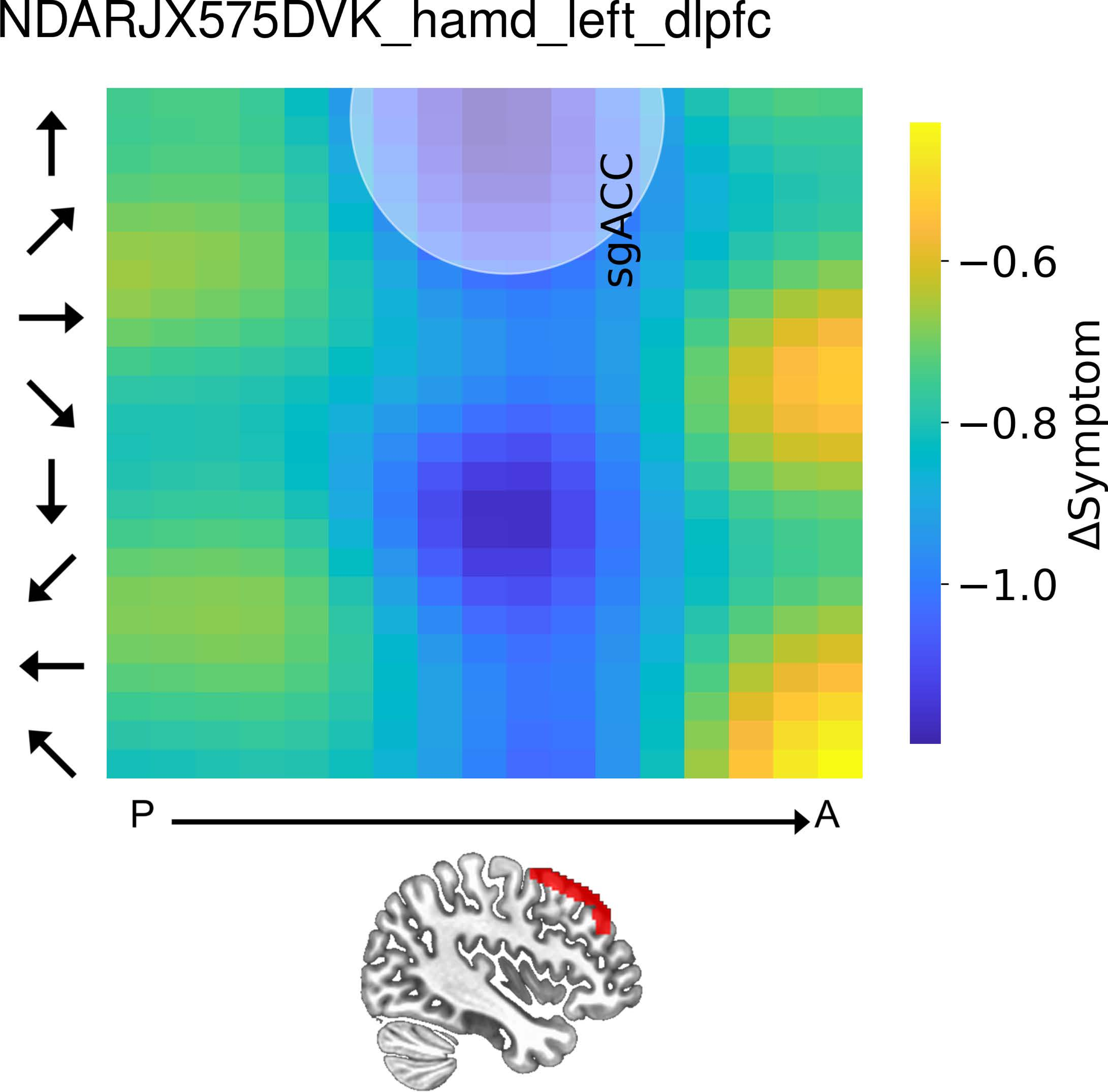
NDARJX575DVK_hamd_left_dlpfc
sgACC
P
A
−0.6
−0.8
−1.0
ΔSymptom

NDARJY042TLY_hamd_left_dlpfc

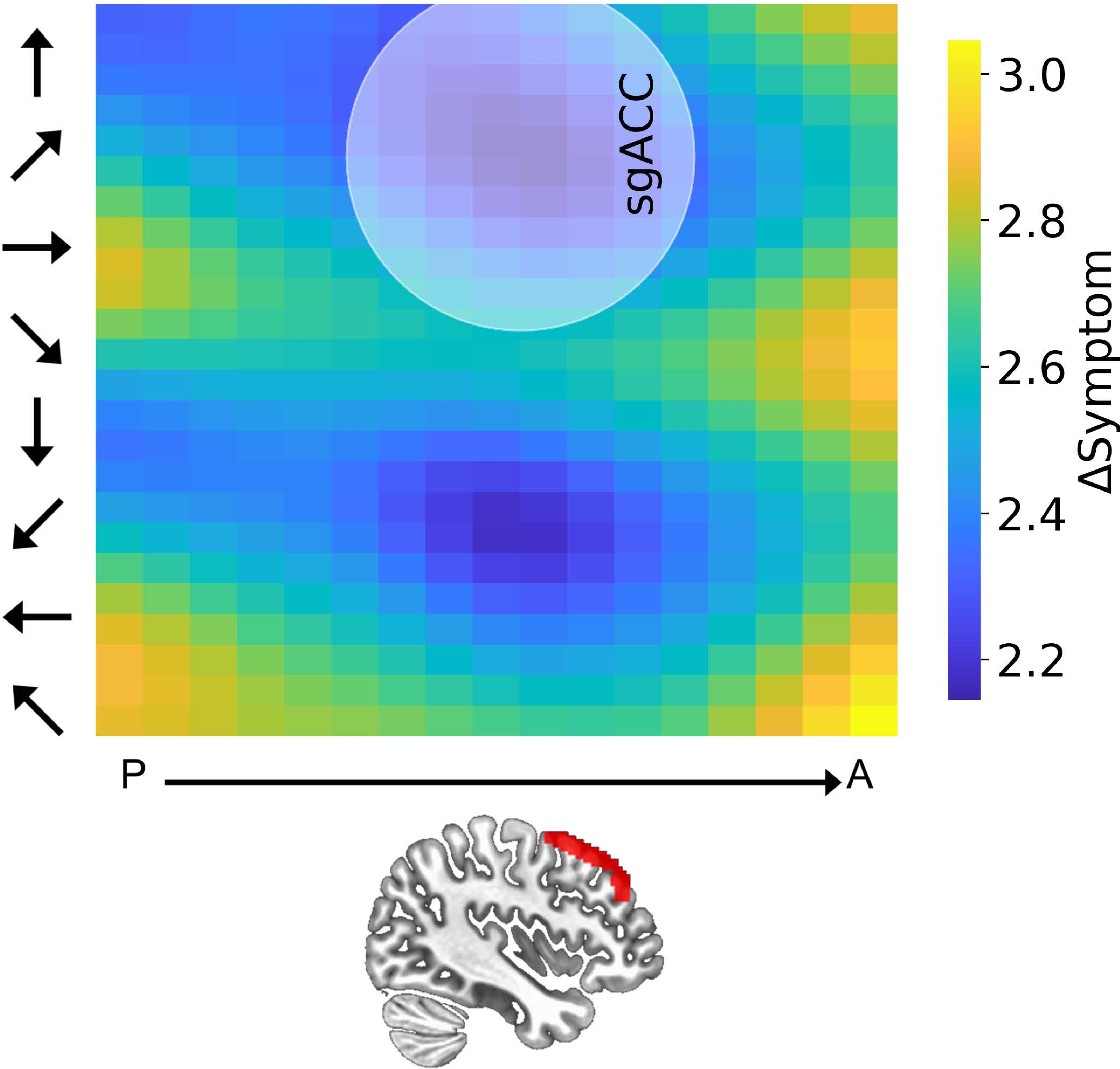

NDARKK212YTX_hamd_left_dlpfc

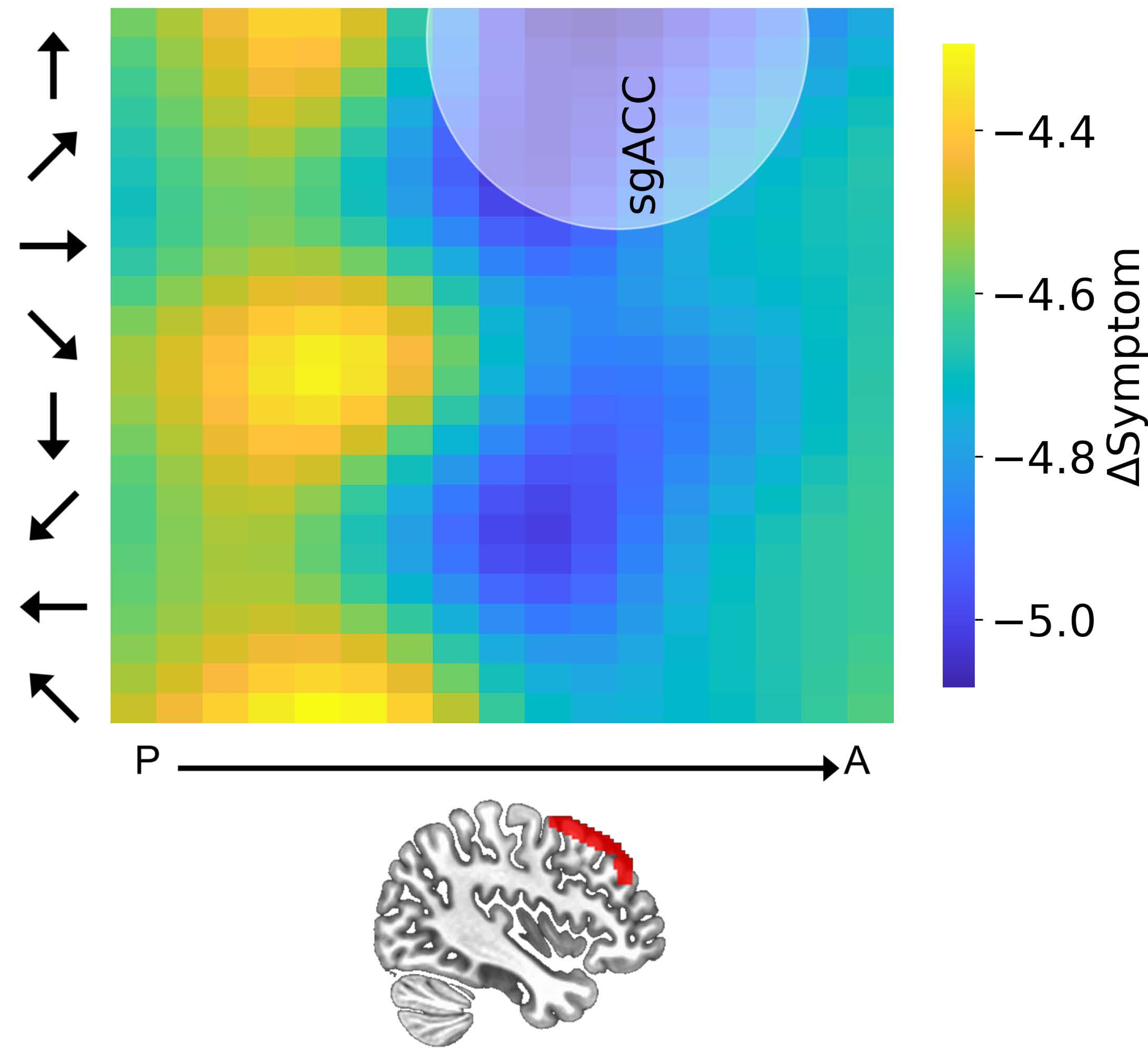

NDARKM333VX2_hamd_left_dlpfc

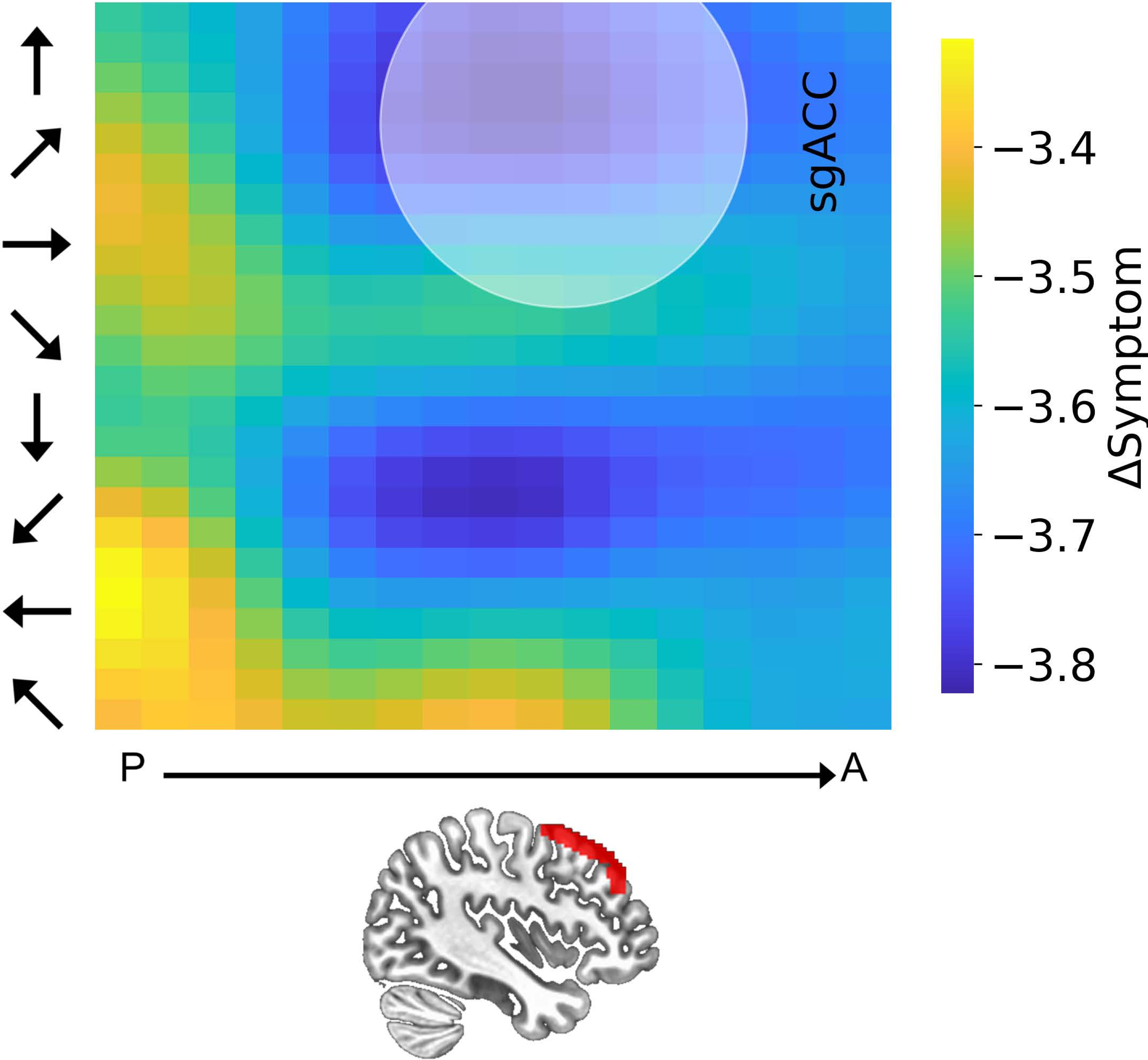

NDARKT991DUC_hamd_left_dlpfc

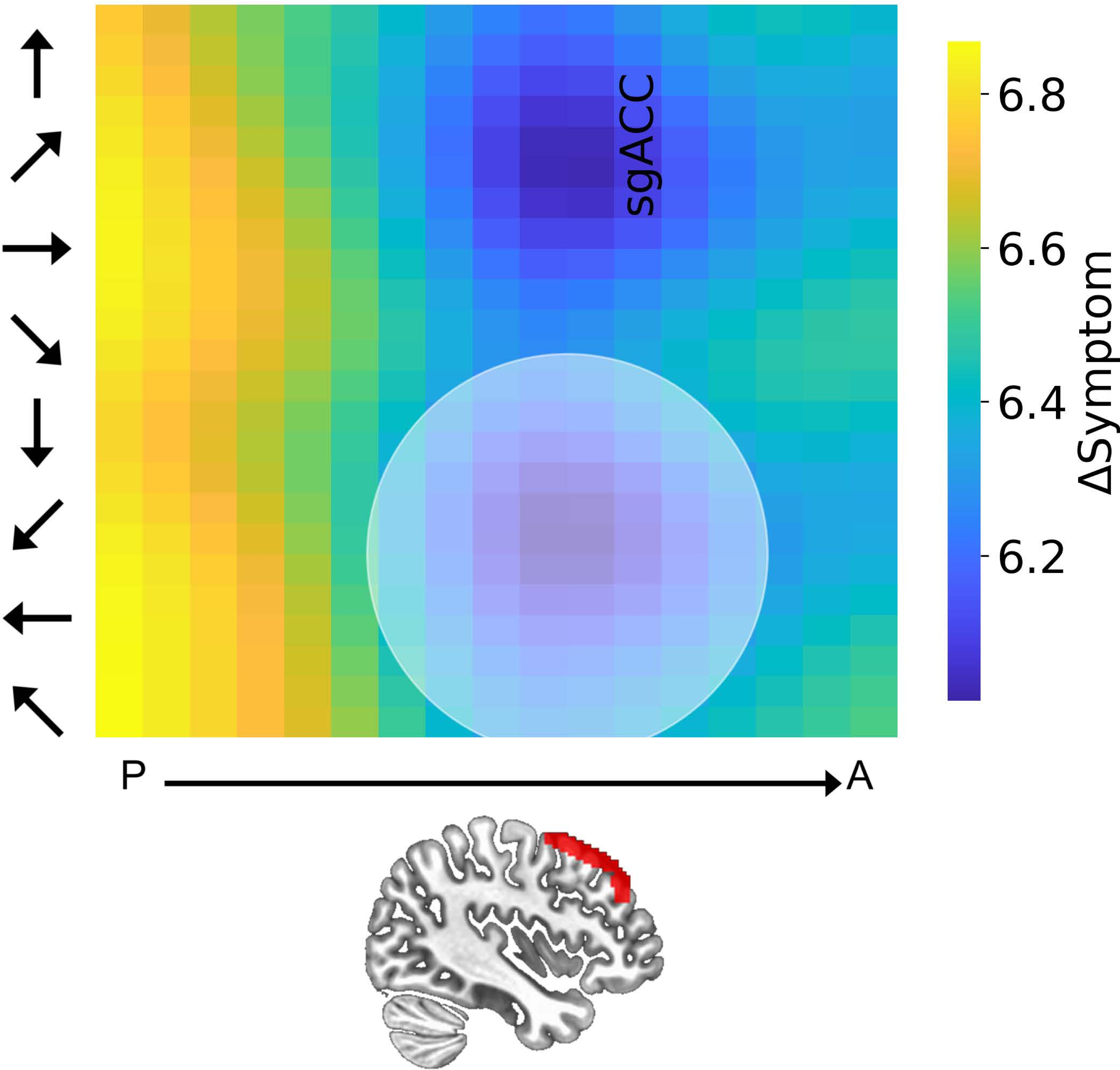

NDARKU015LTM_hamd_left_dlpfc

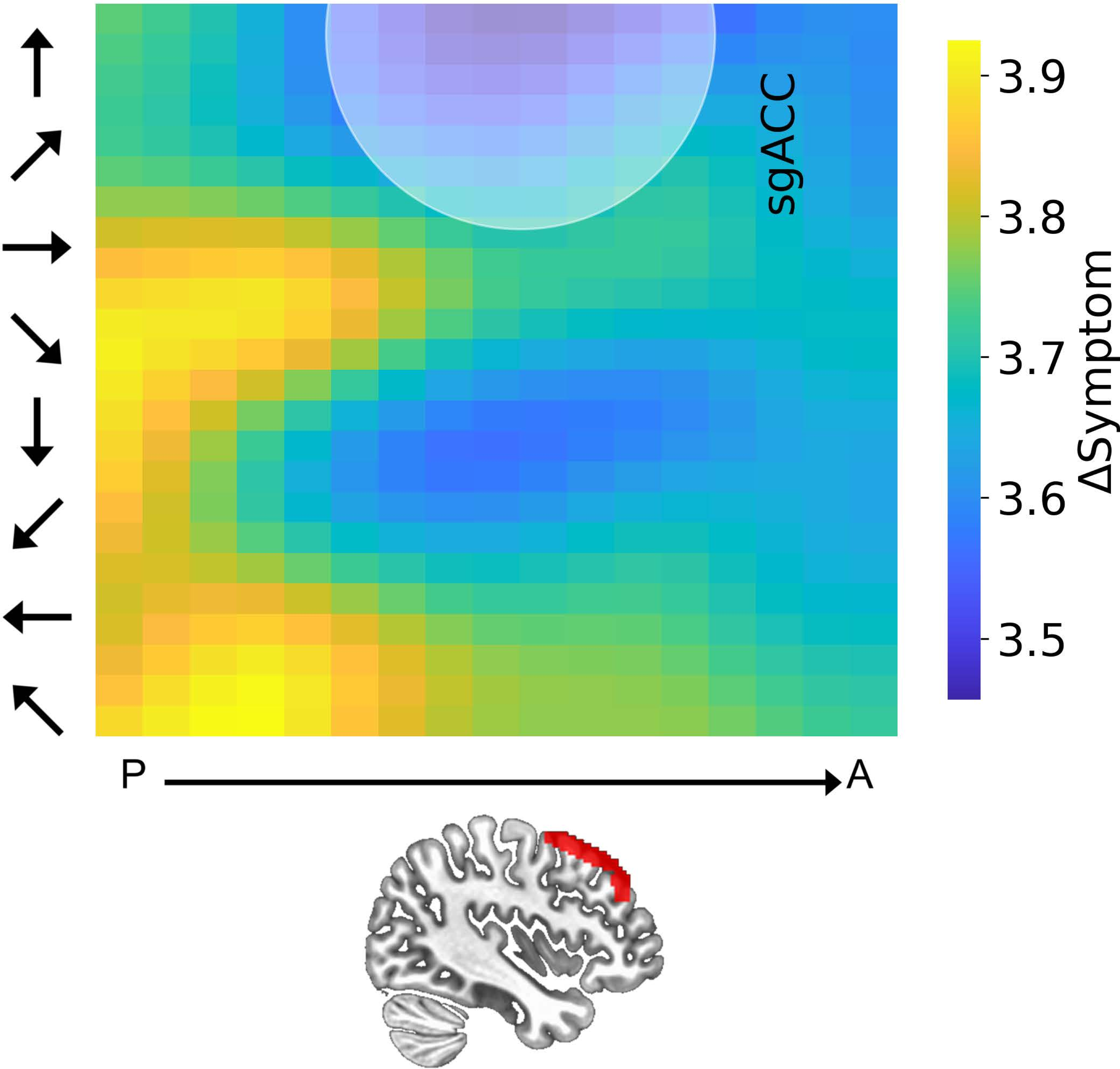

NDARKV126RTP_hamd_left_dlpfc

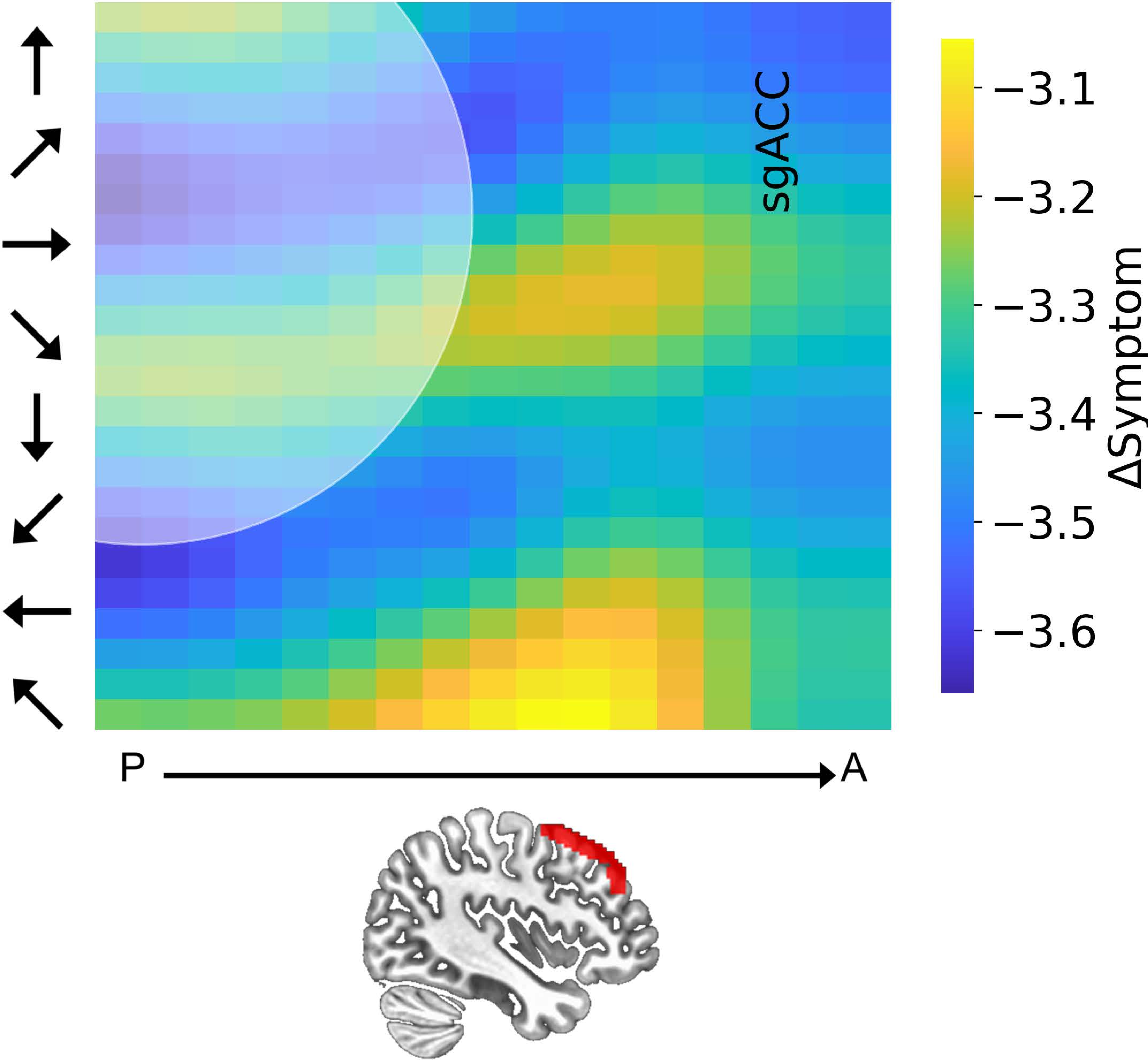

NDARLF826BAB_hamd_left_dlpfc

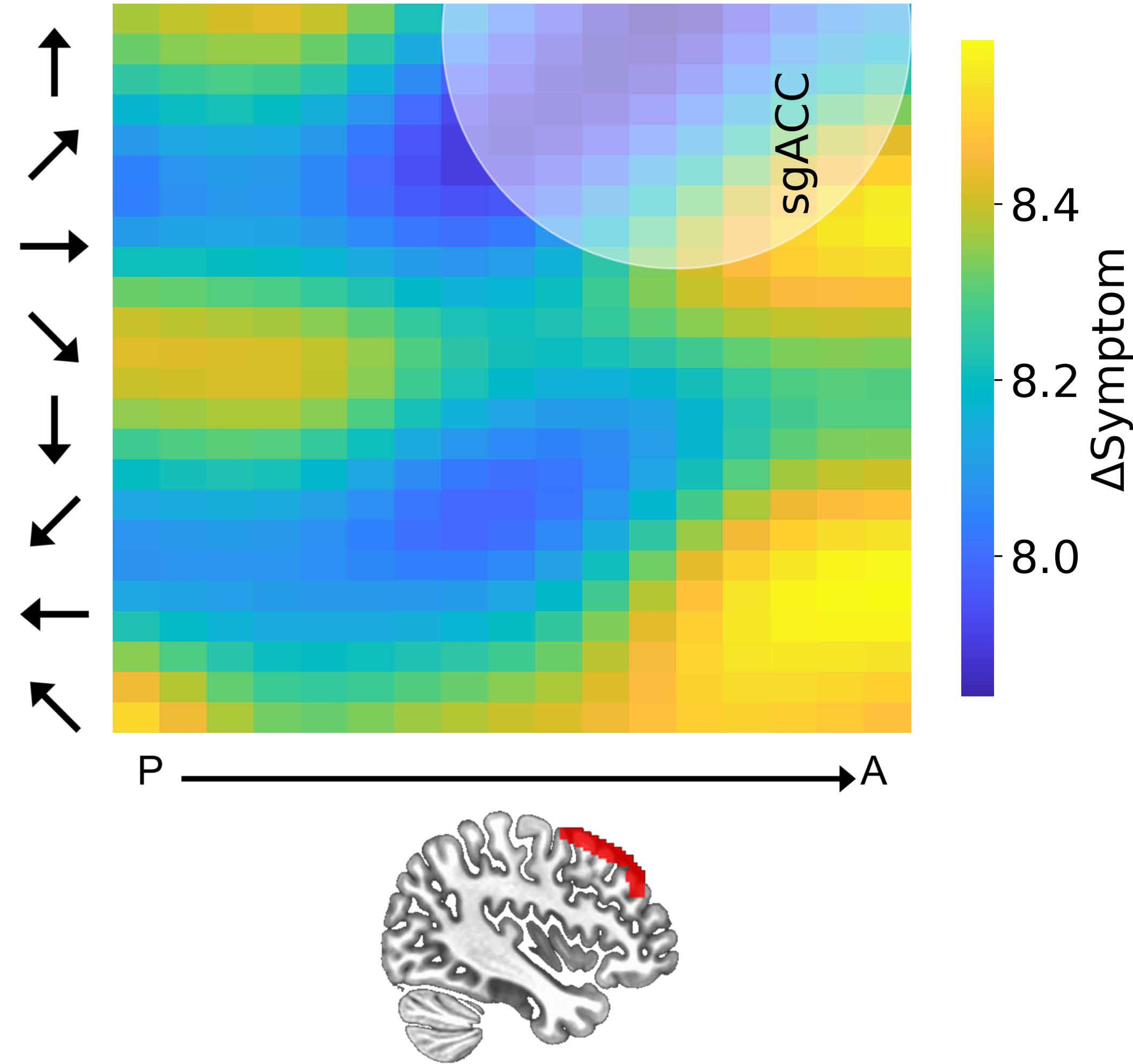

NDARLM890ELE_hamd_left_dlpfc

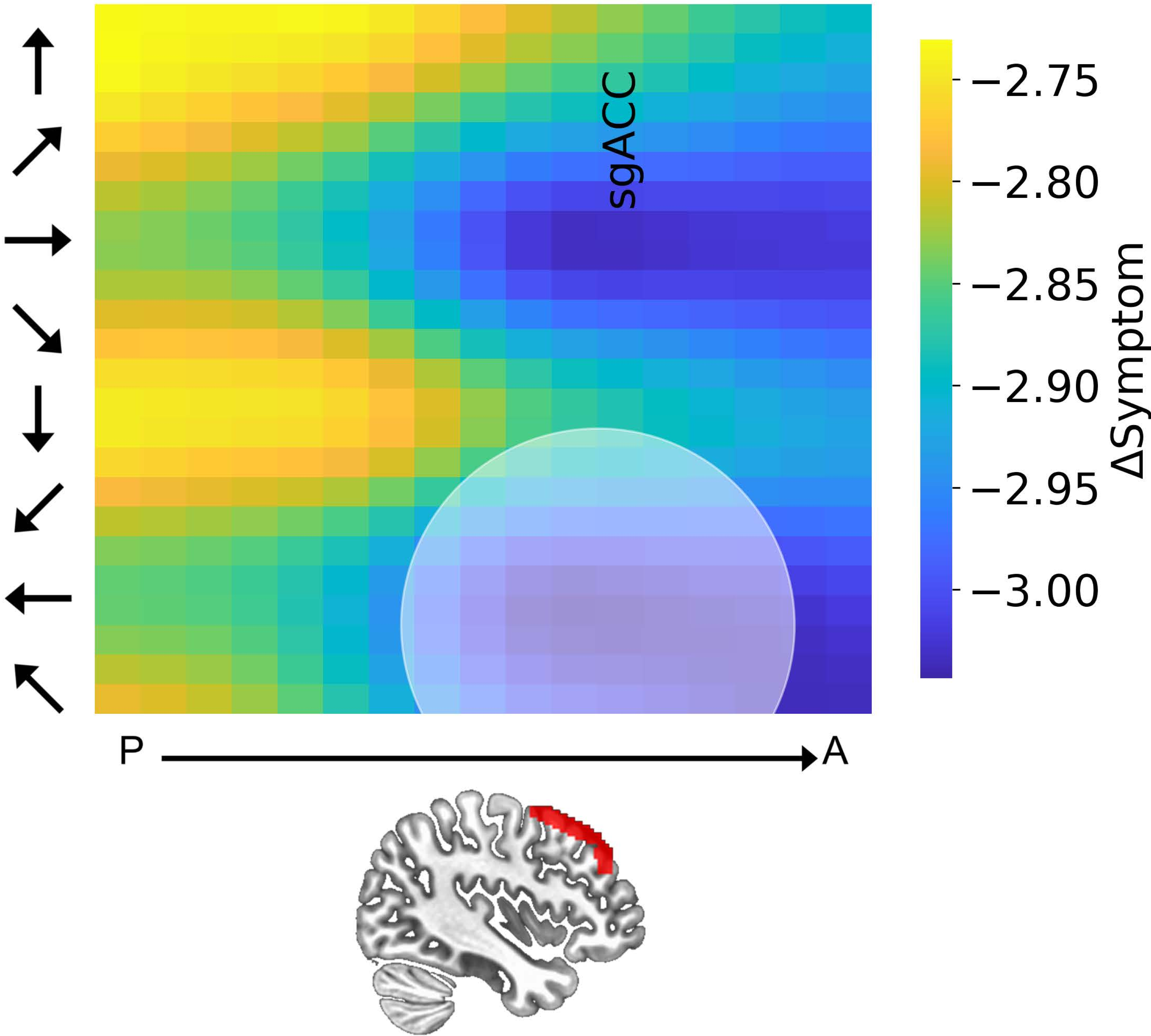

NDARLZ935EEN_hamd_left_dlpfc

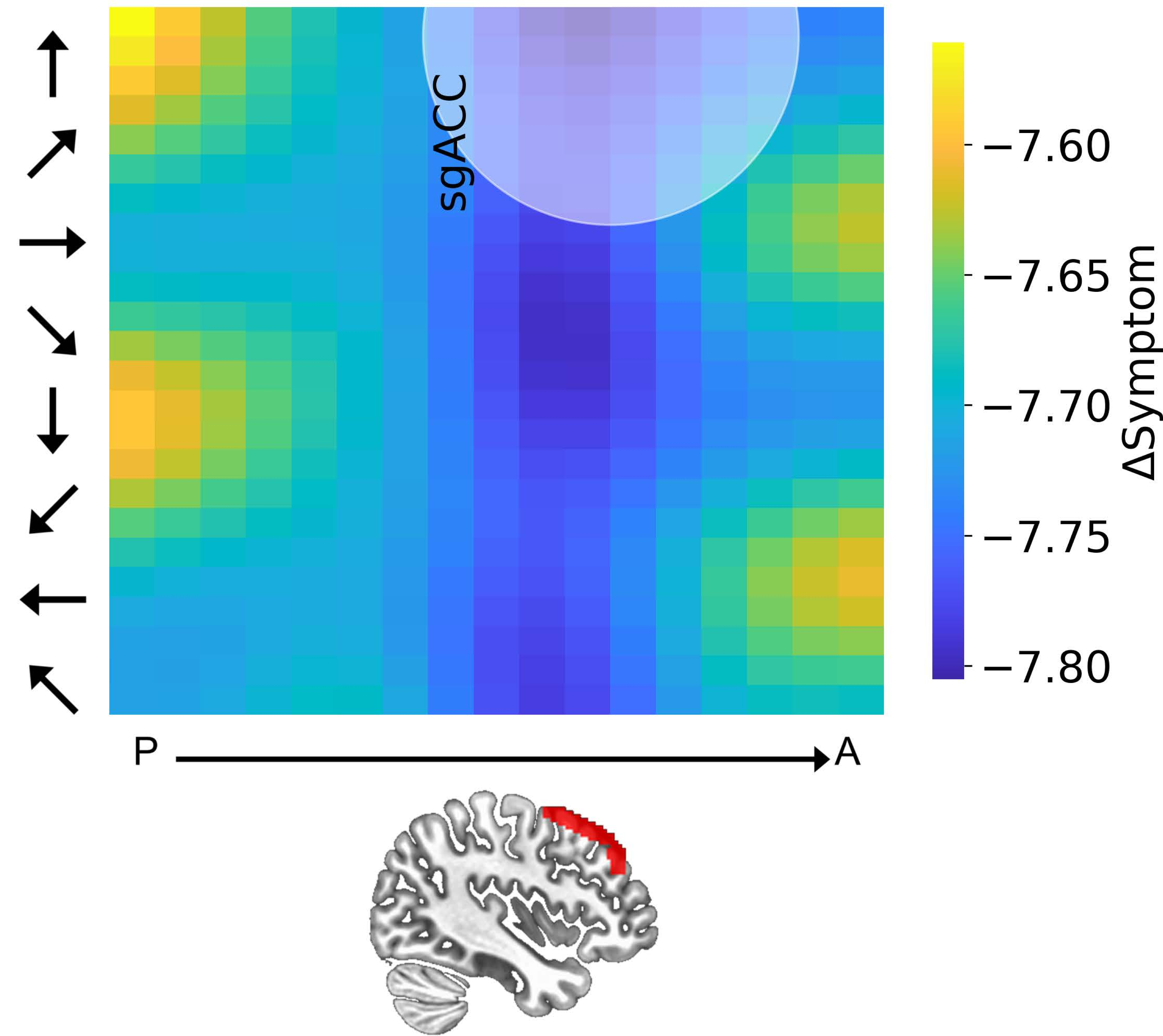

NDARMA155ATG_hamd_left_dlpfc

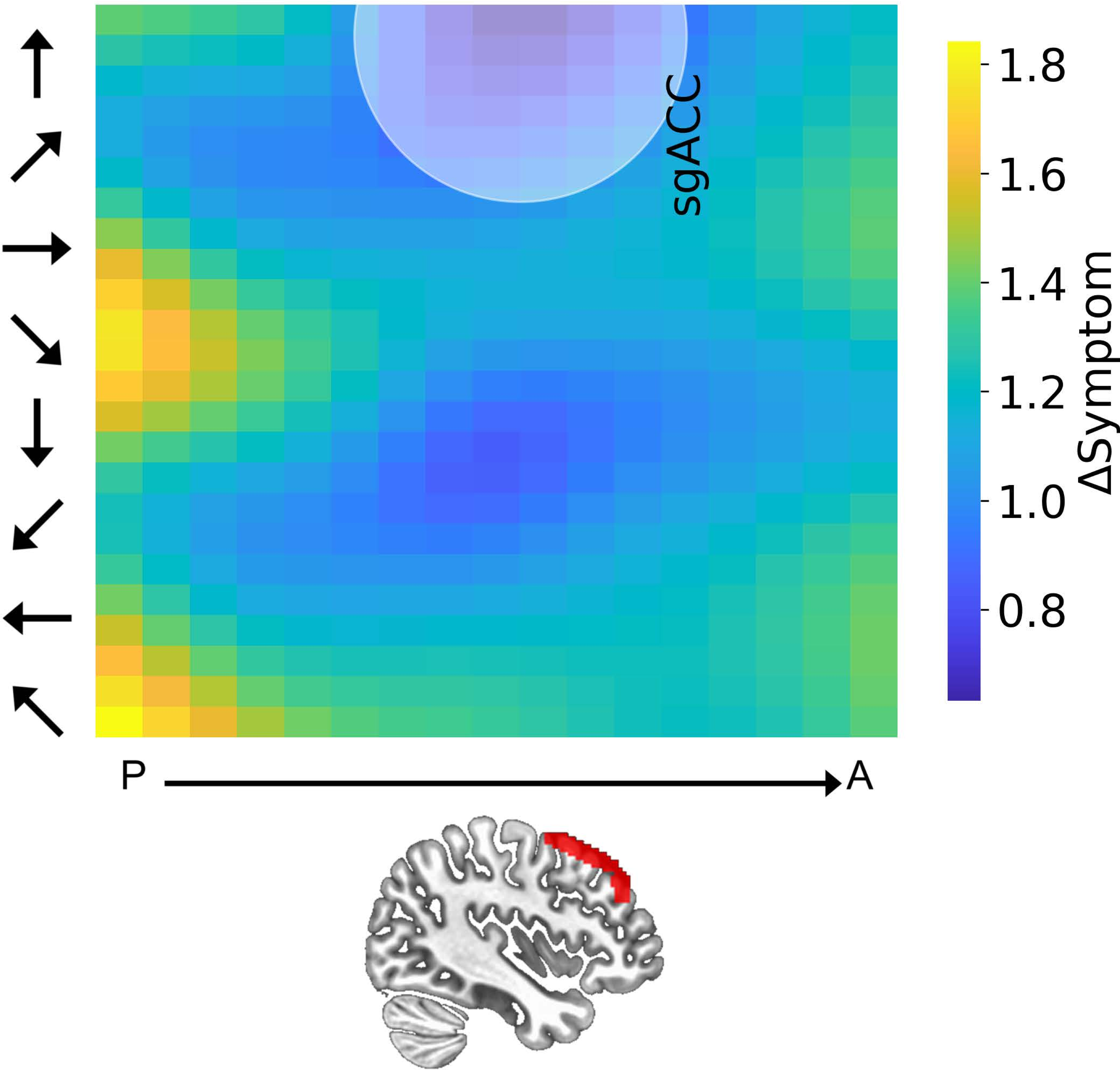

NDARMB943VPJ_hamd_left_dlpfc

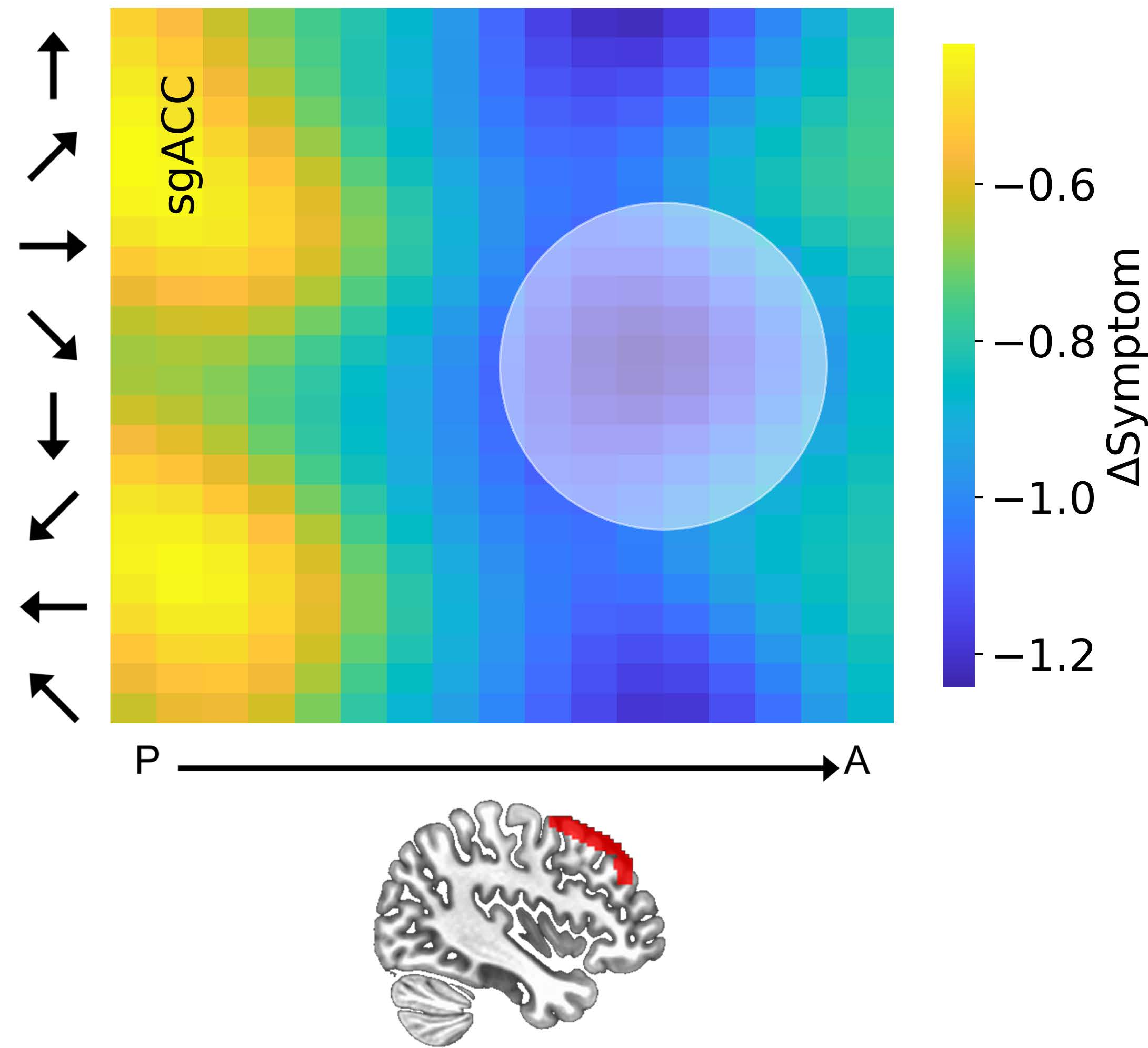

NDARMD016CB8_hamd_left_dlpfc

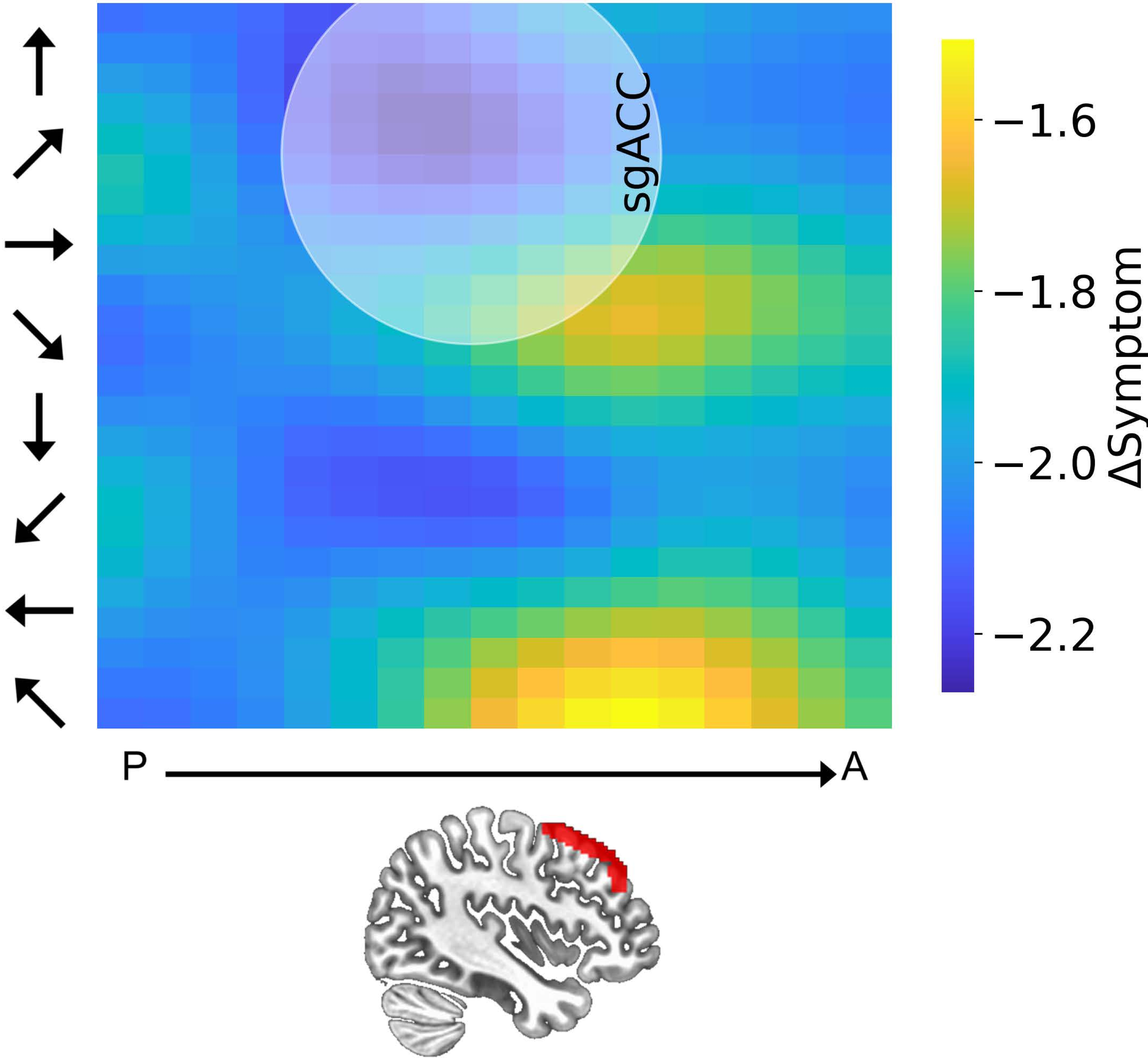

NDARMD601CTU_hamd_left_dlpfc

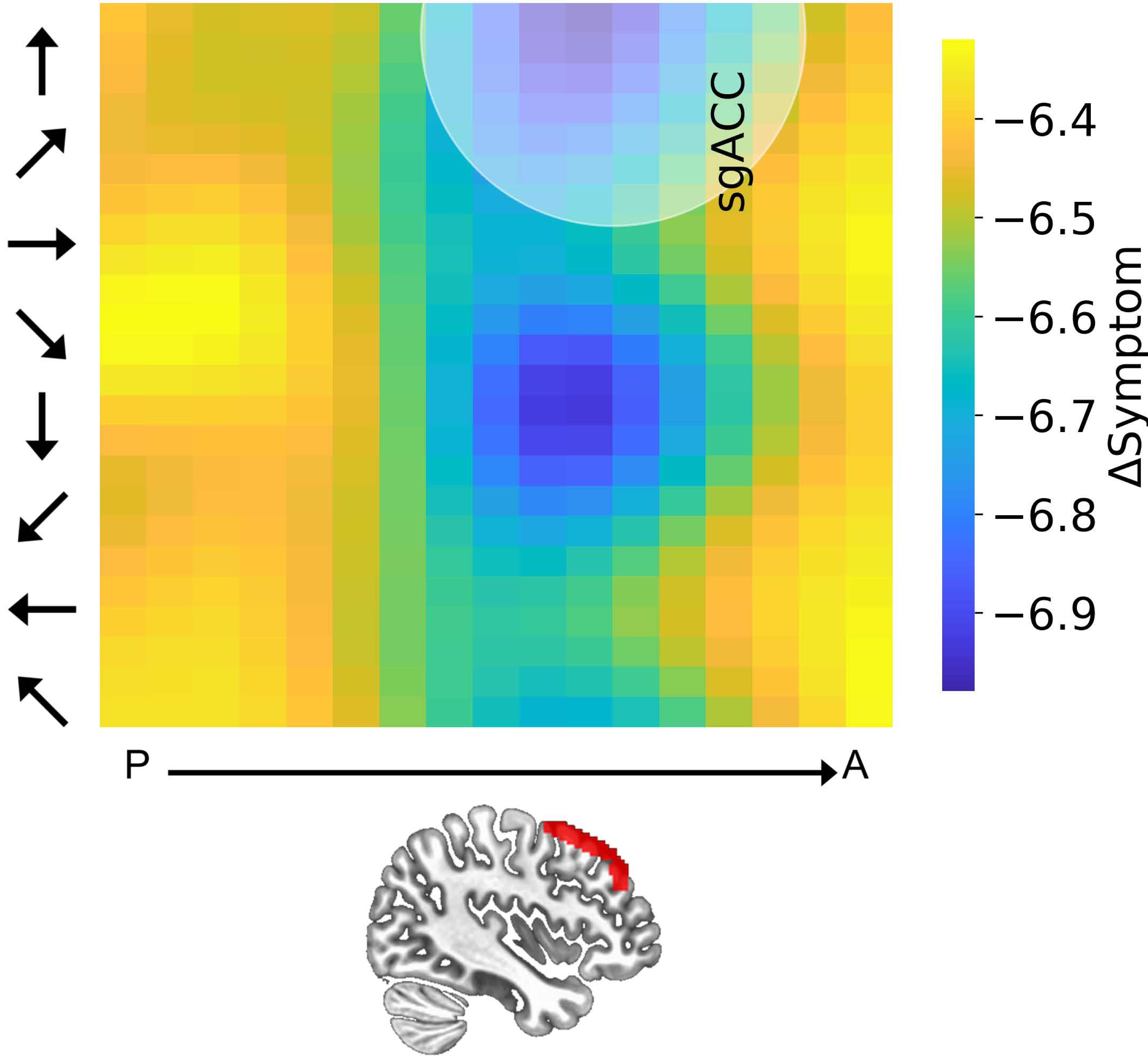

NDARME189AXP_hamd_left_dlpfc

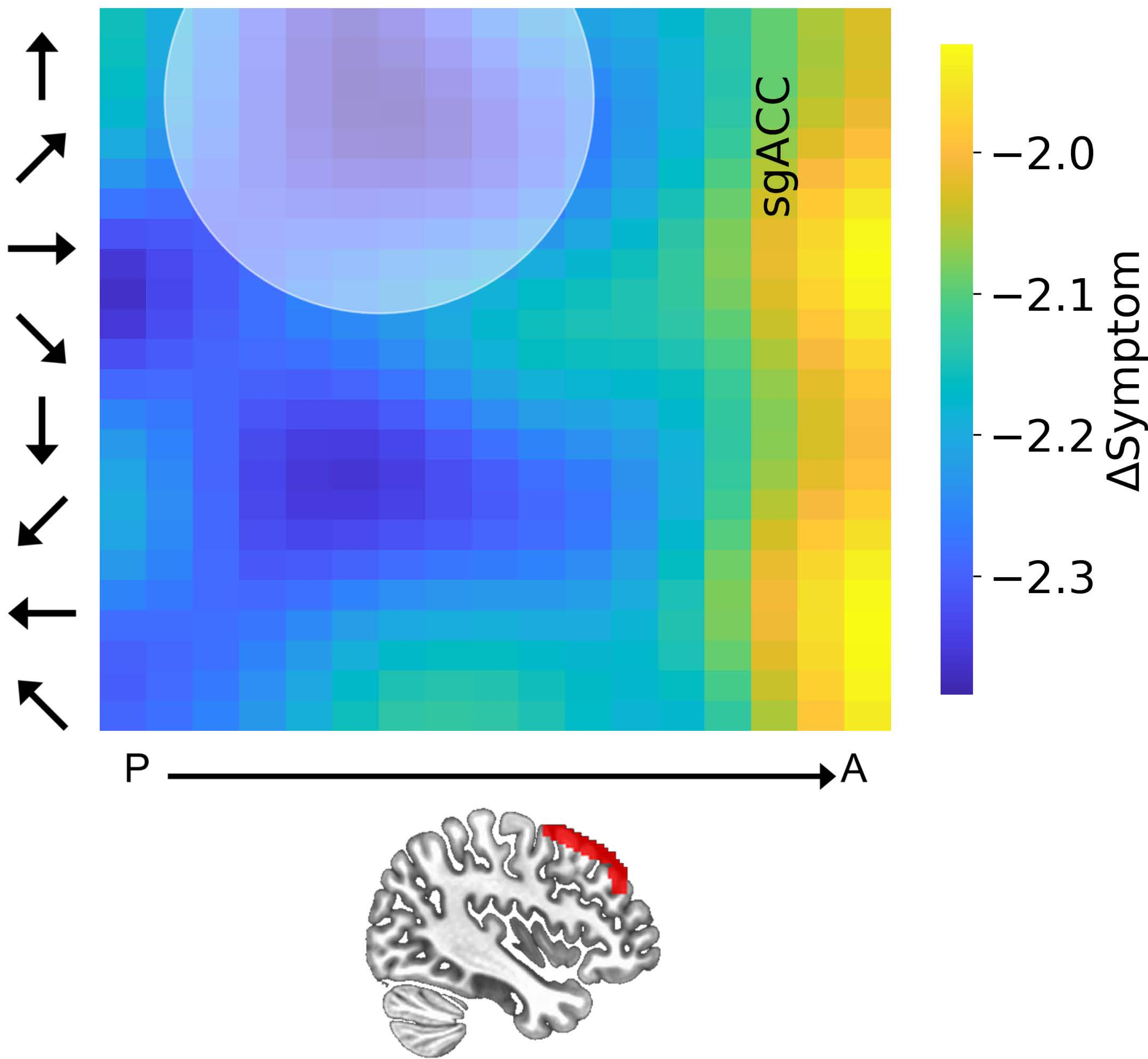

NDARME335LZJ_hamd_left_dlpfc

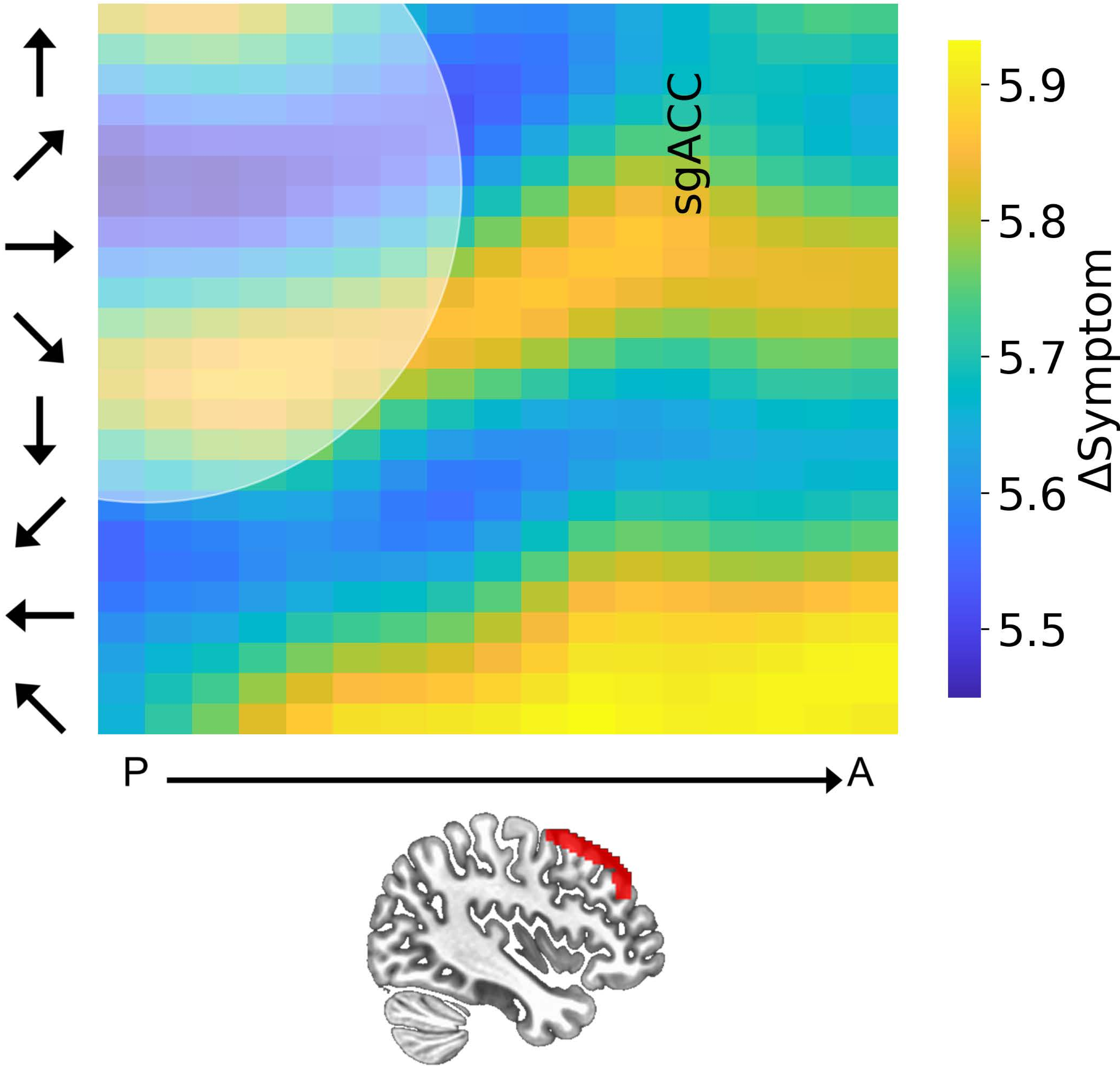

NDARMF293AHT_hamd_left_dlpfc

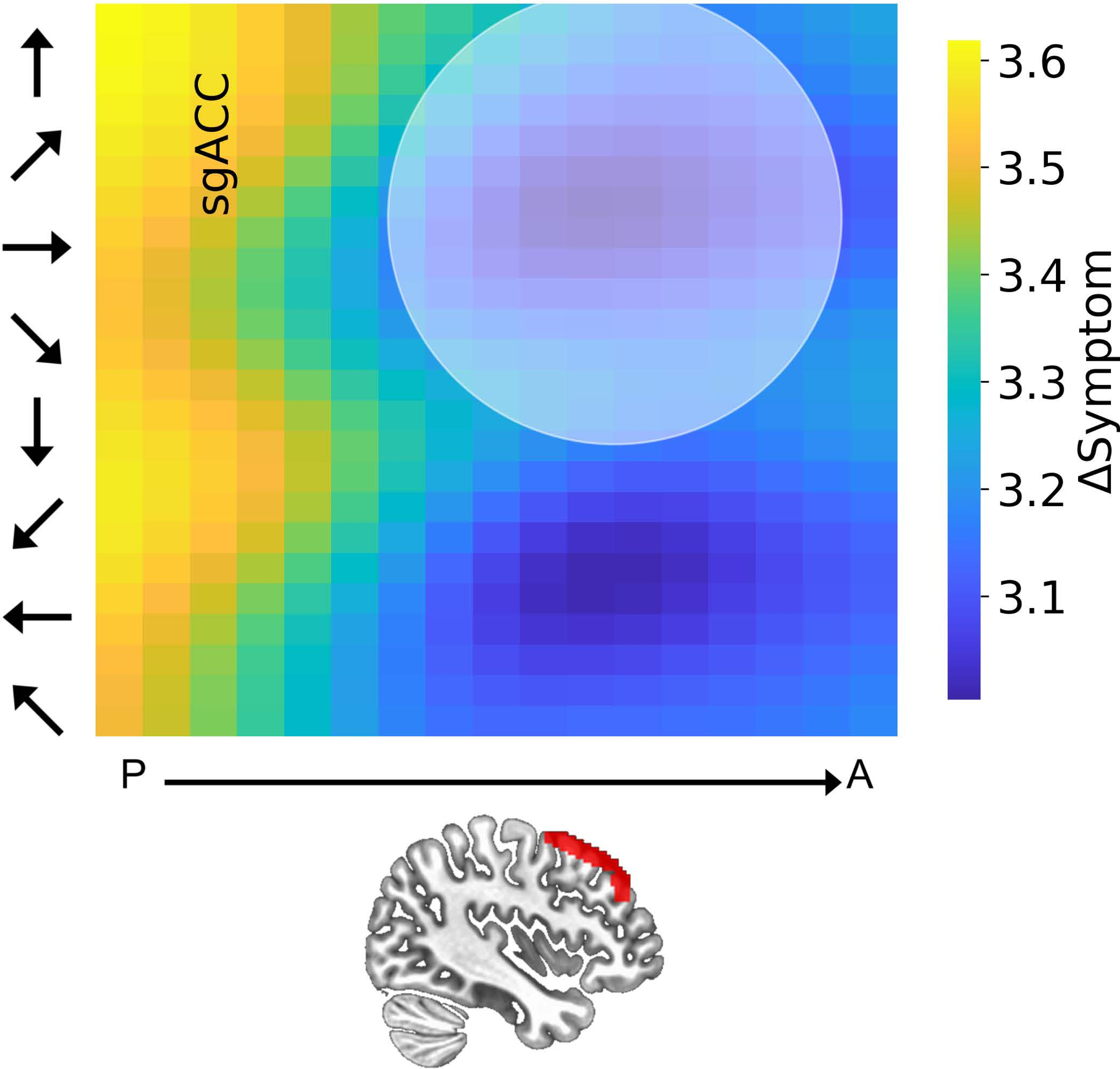

NDARMK972BFE_hamd_left_dlpfc

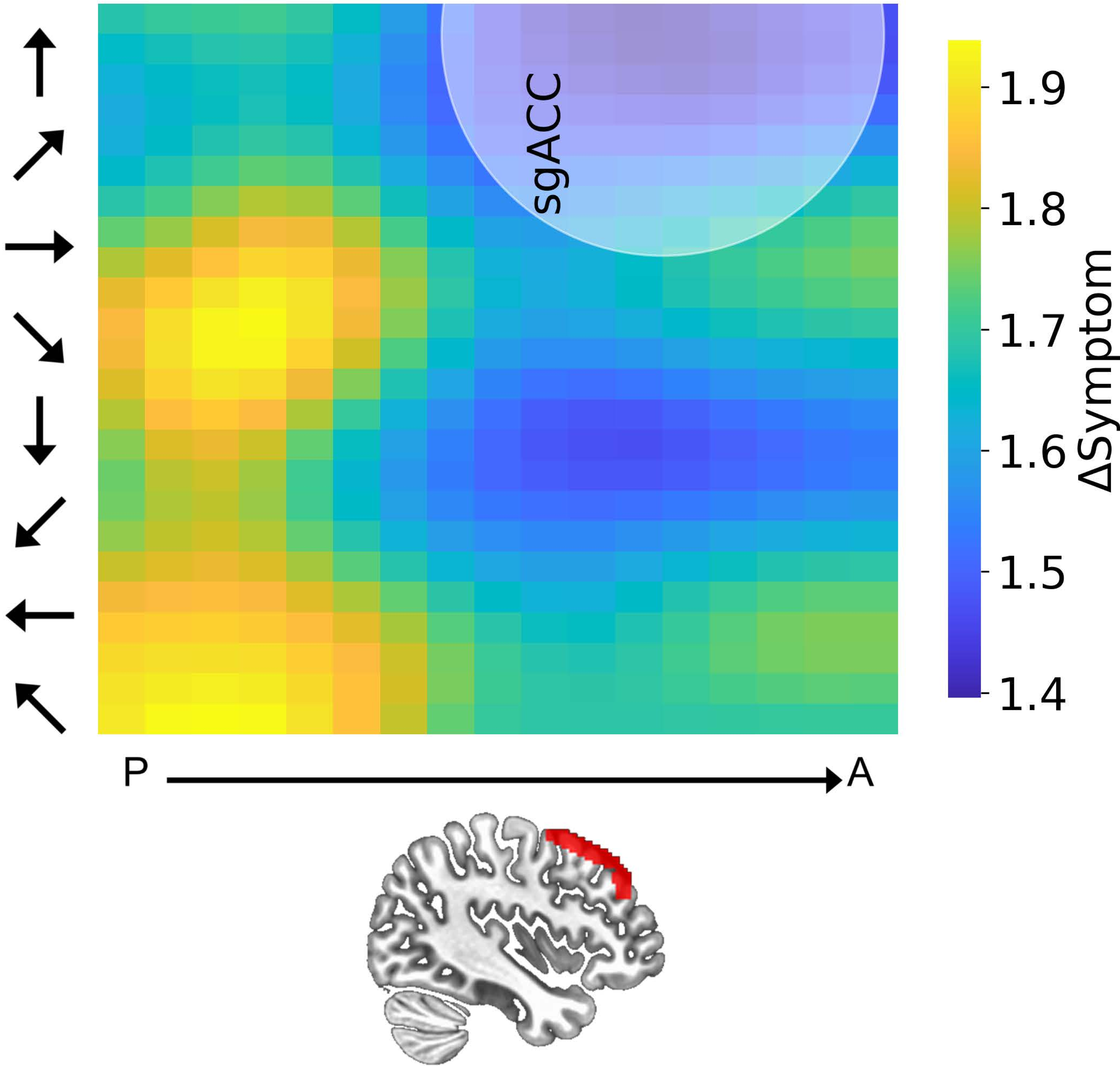

NDARNA955LXG_hamd_left_dlpfc

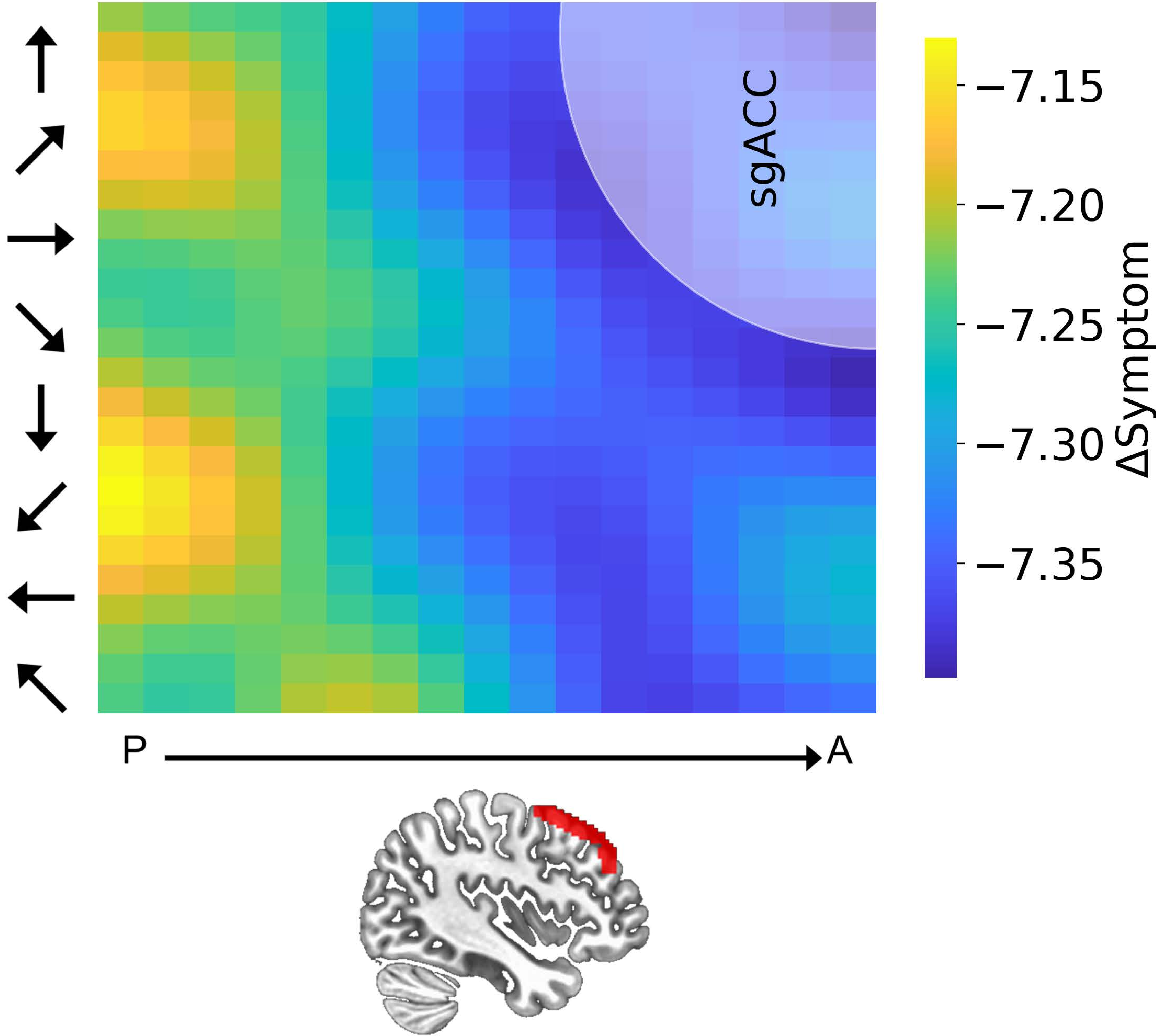

NDARNR764NAK_hamd_left_dlpfc

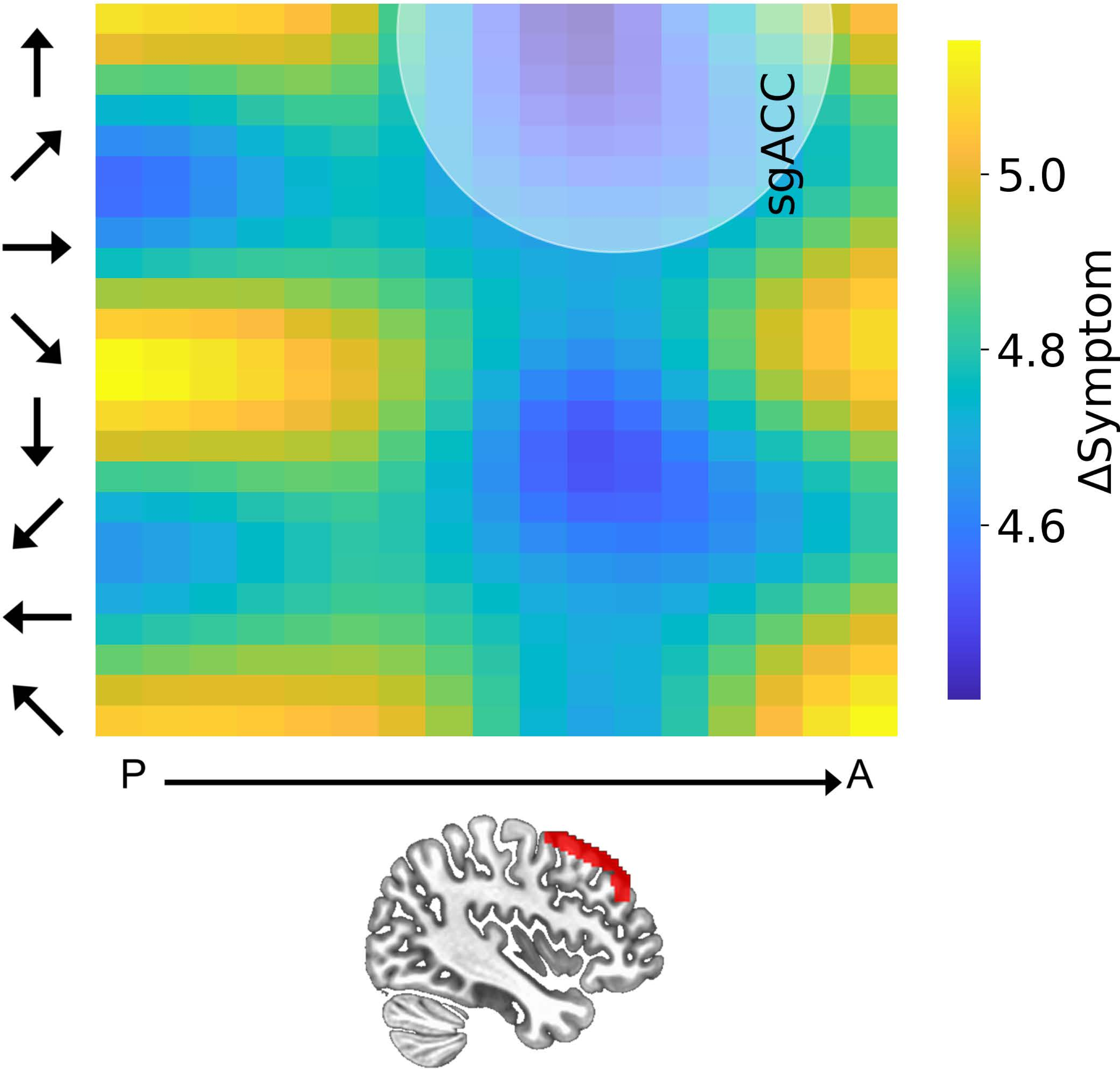

NDARNU180HL3_hamd_left_dlpfc

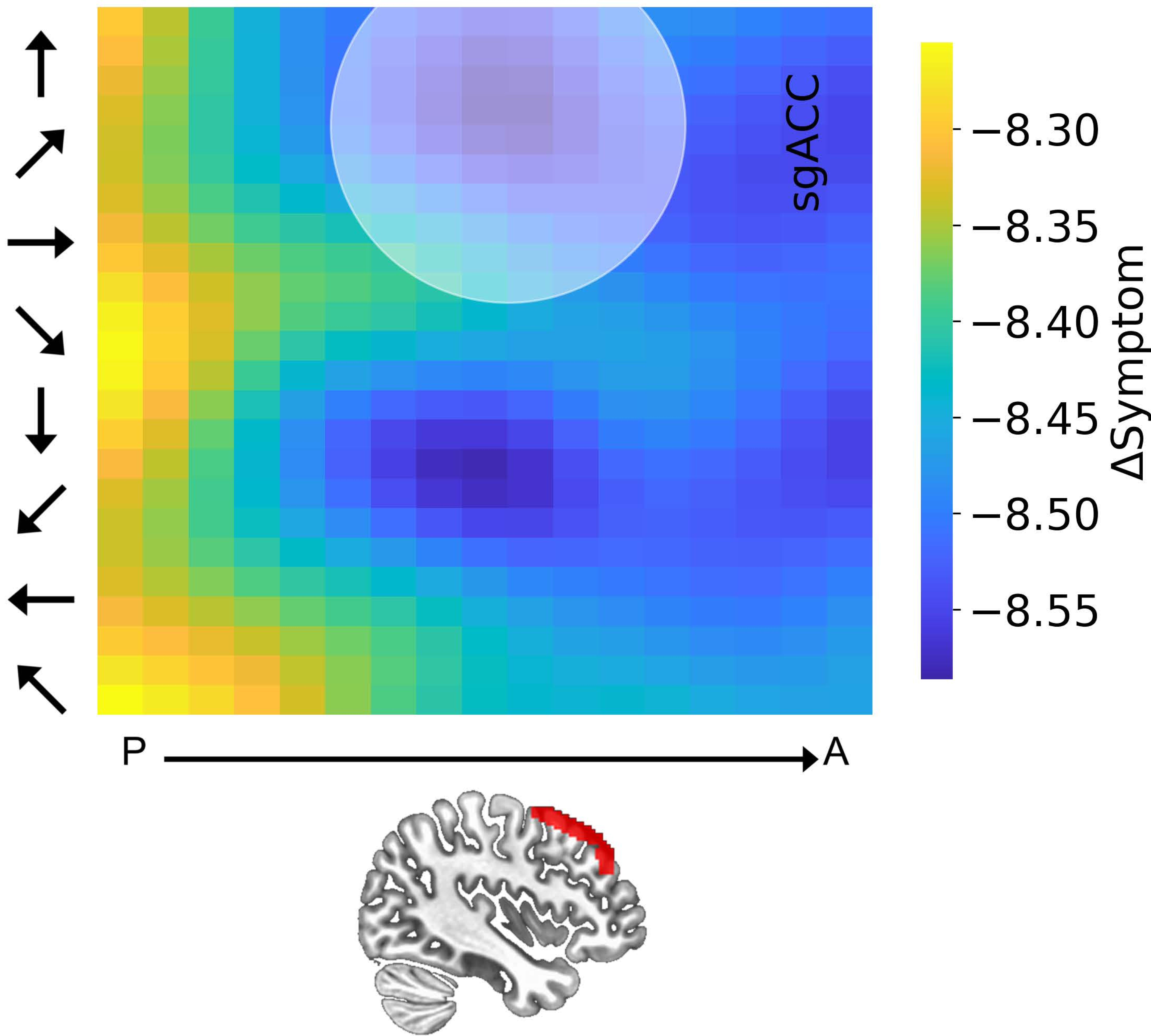

NDARNZ824XHB_hamd_left_dlpfc

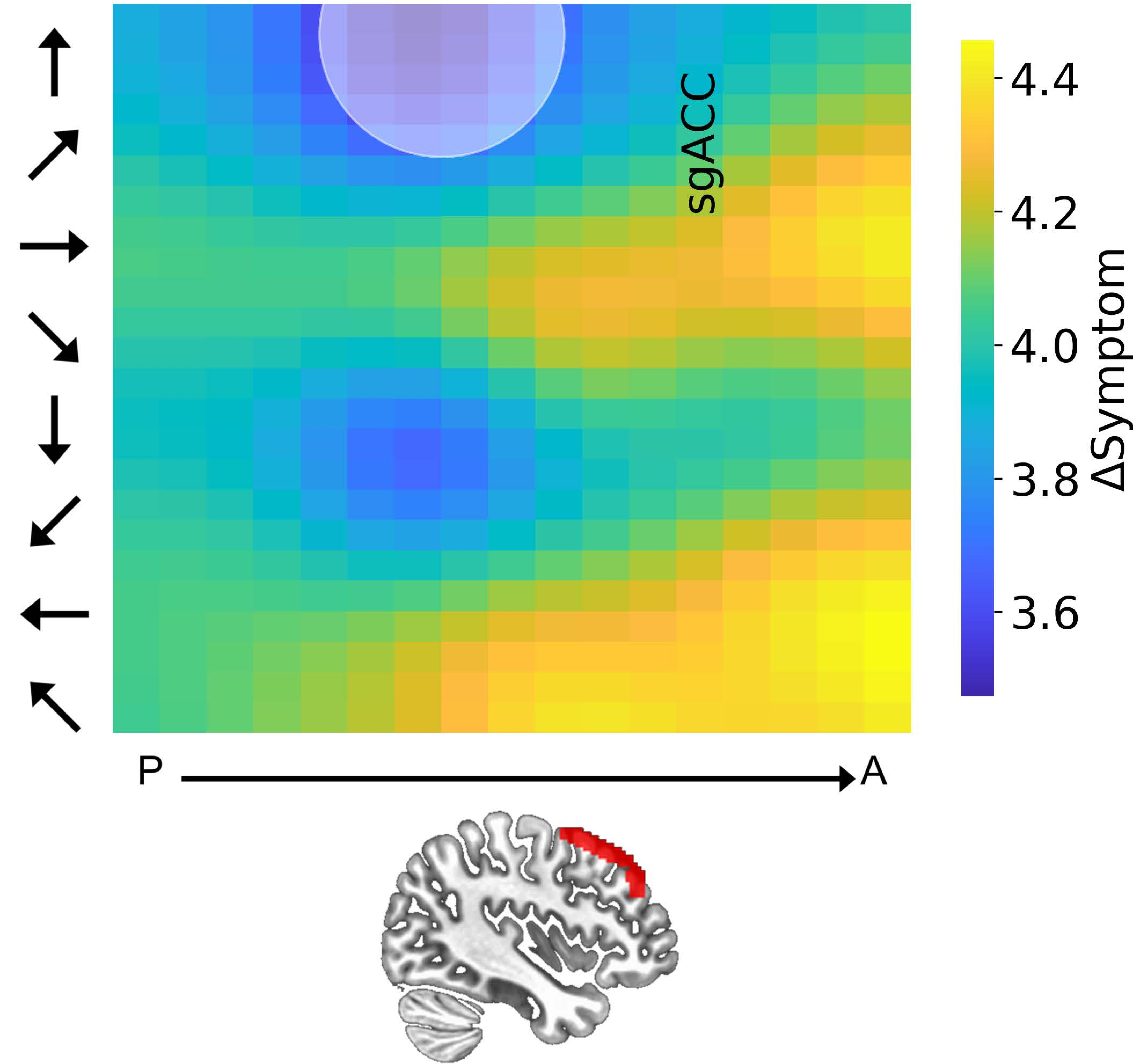

NDARPA762UGZ_hamd_left_dlpfc

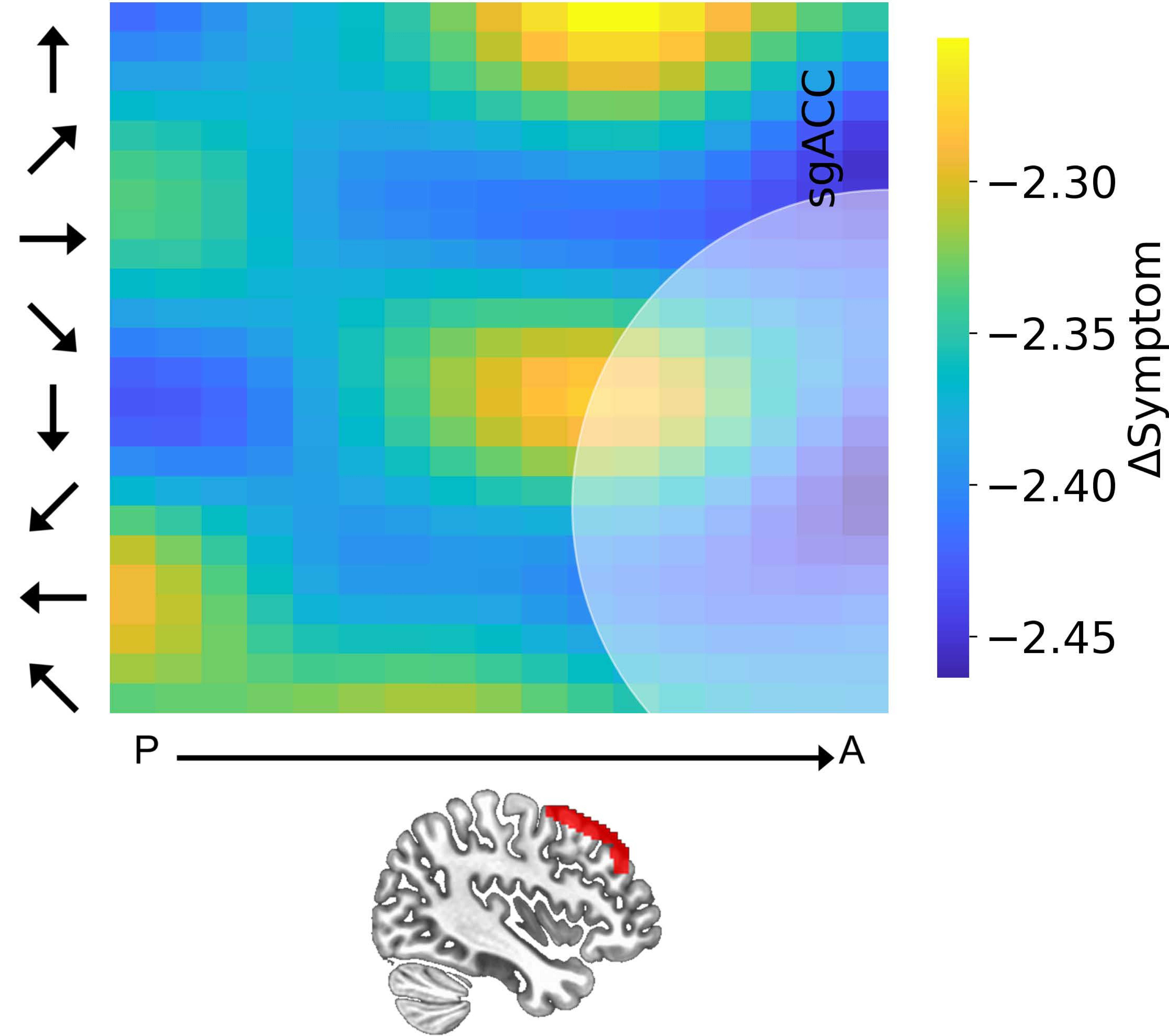

NDARPC197VR3_hamd_left_dlpfc

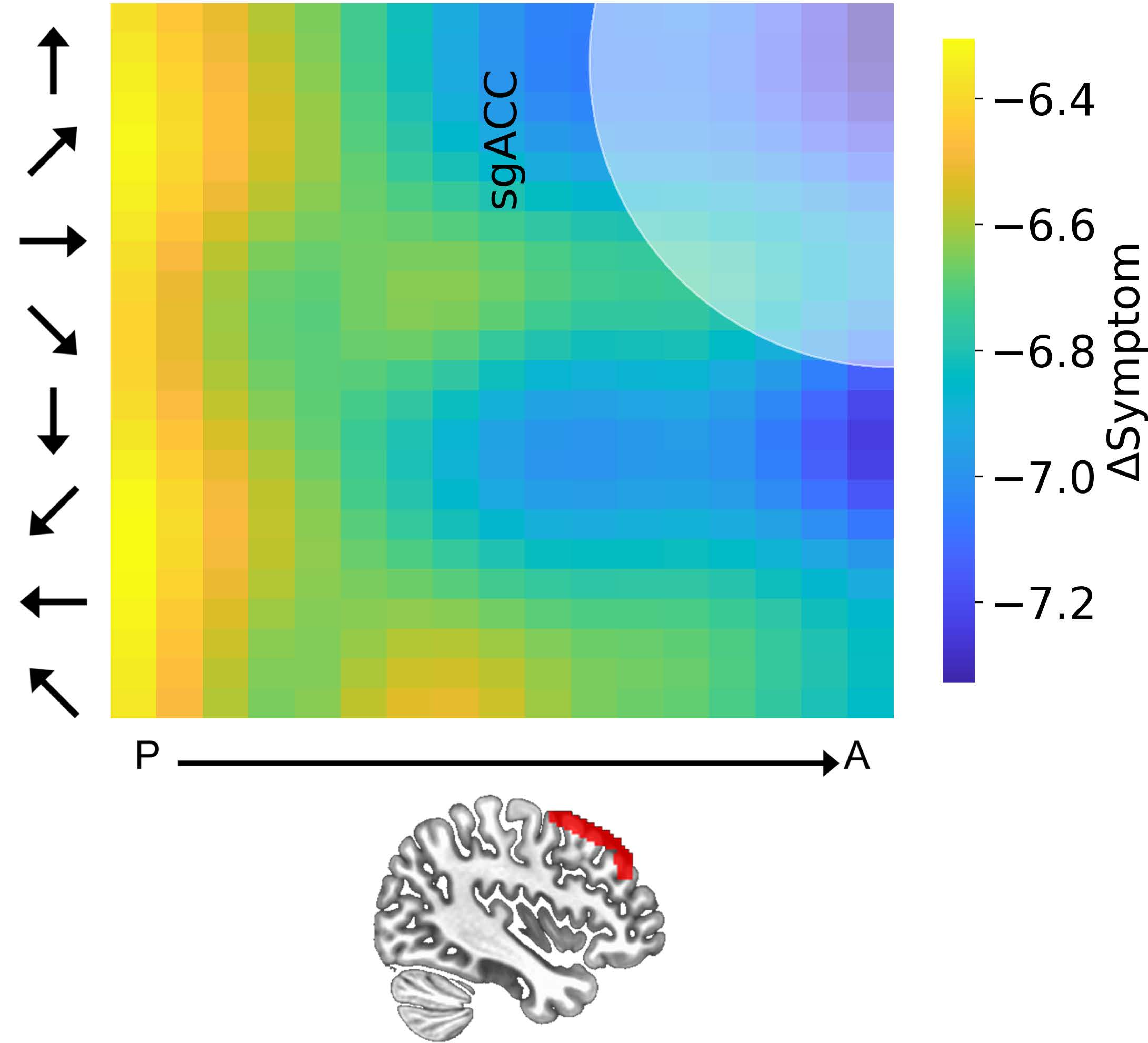

NDARPF499HVH_hamd_left_dlpfc

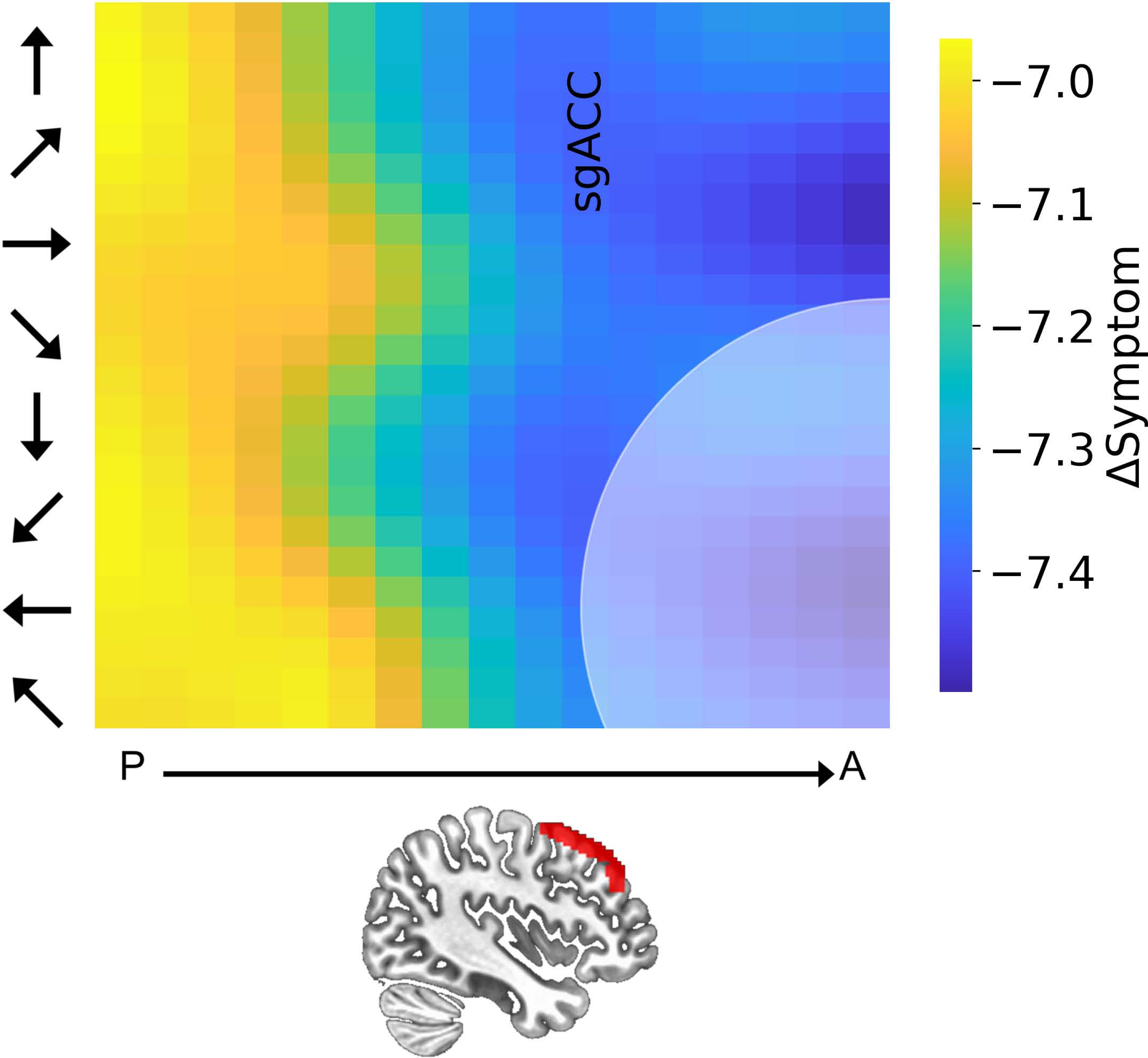

NDARPK811MVVW_hamd_left_dlpfc

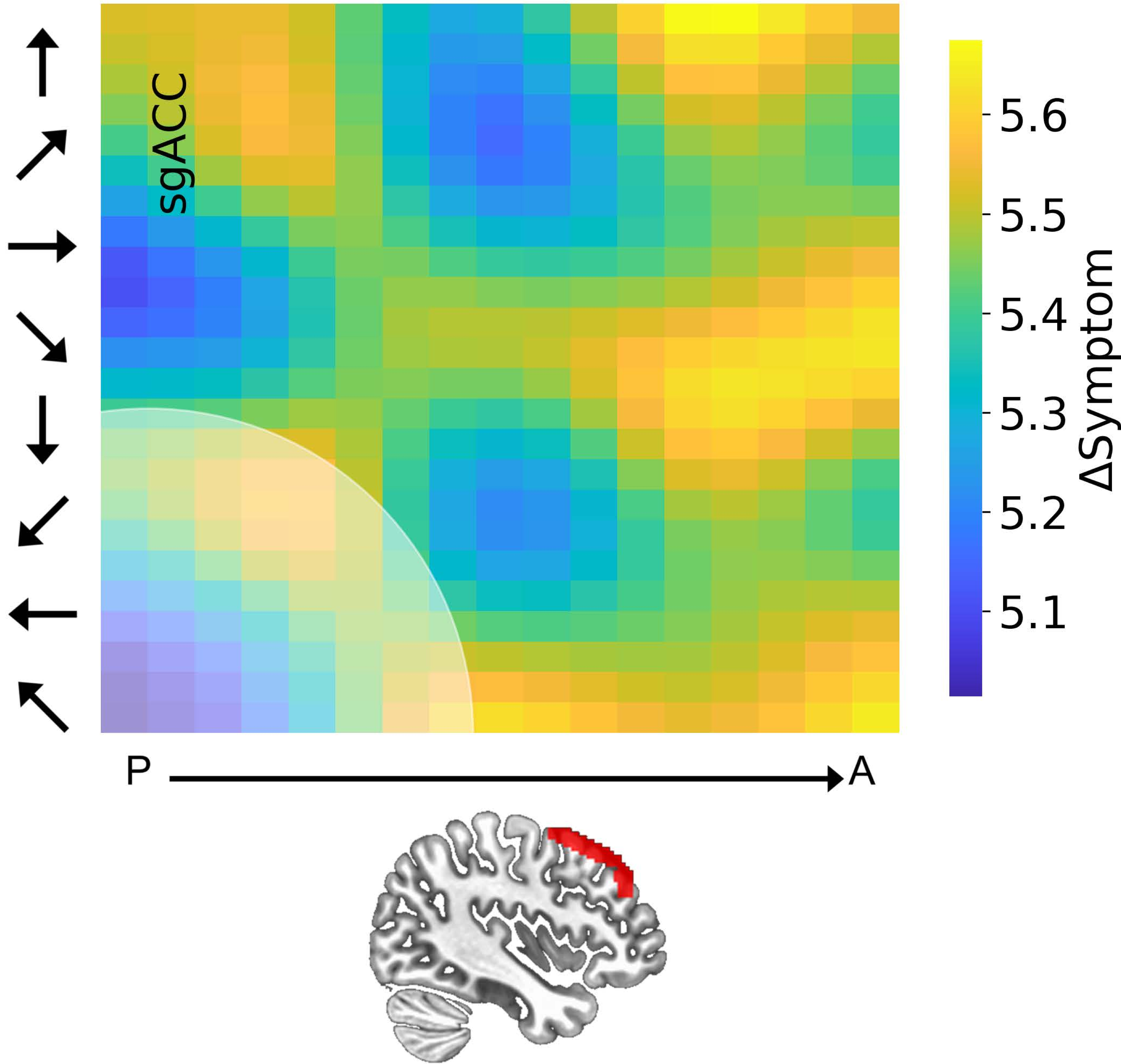

NDARPT040EGR_hamd_left_dlpfc

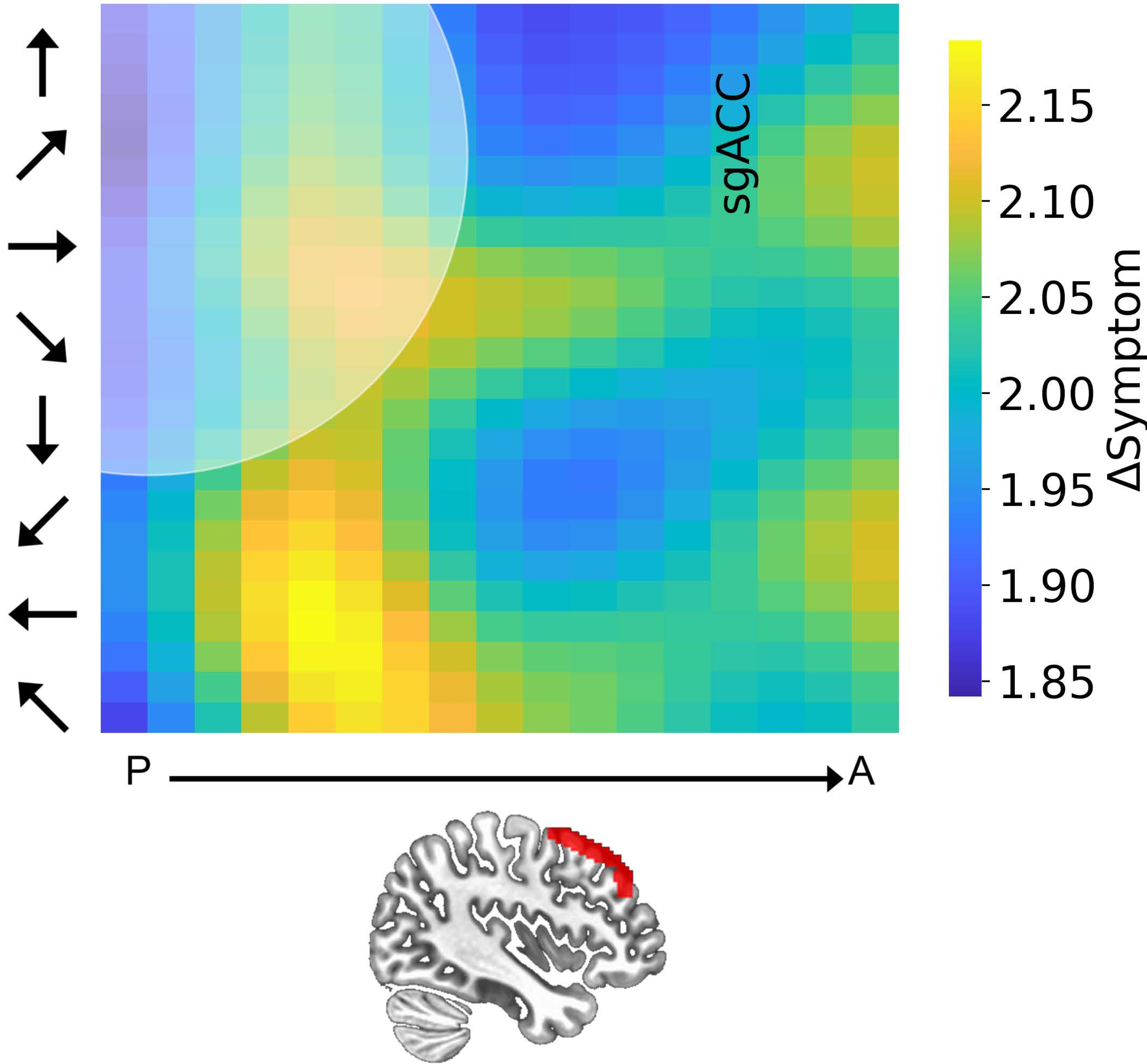

NDARPW706YNA_hamd_left_dlpfc

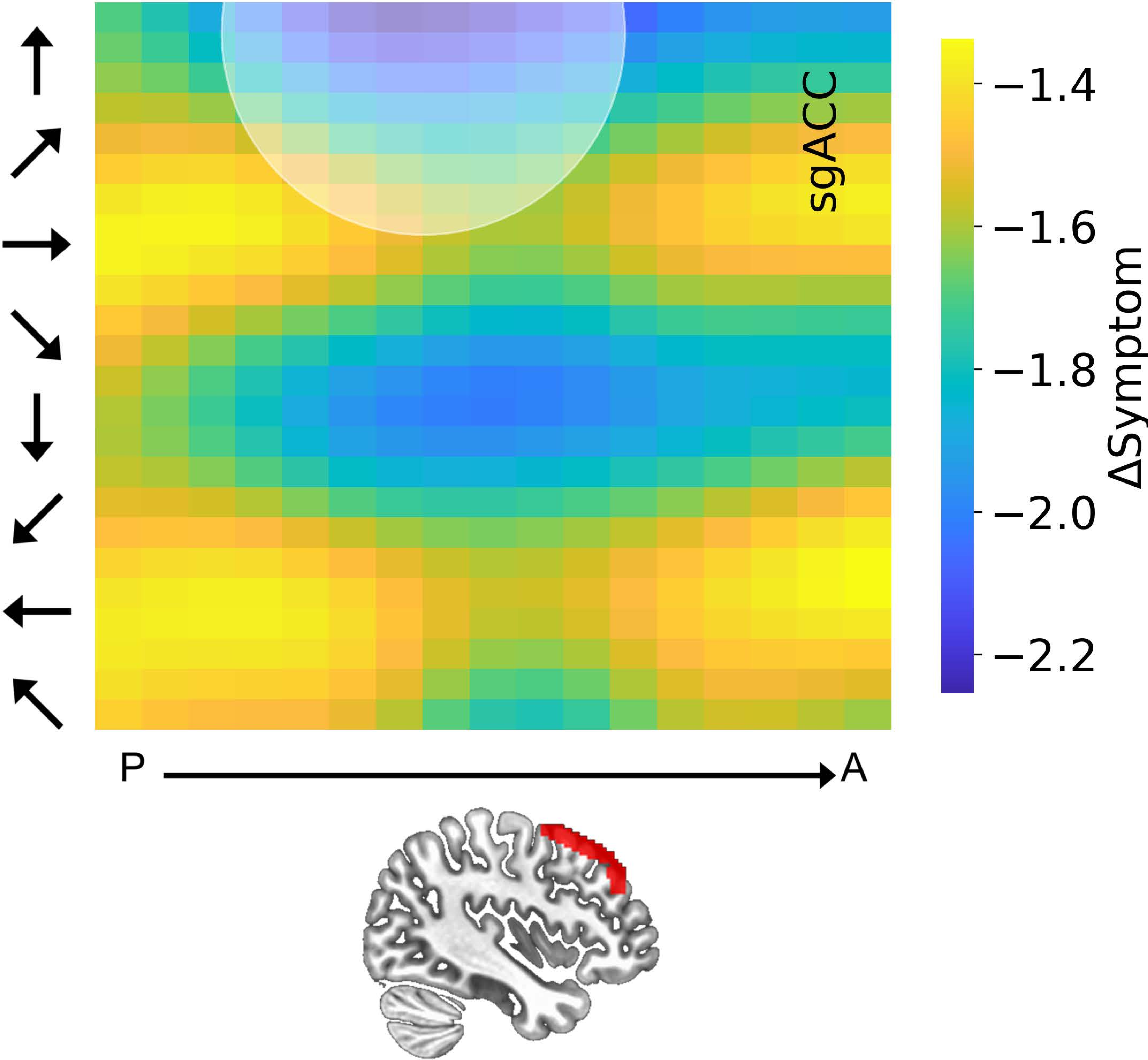

NDARPZ537YY0_hamd_left_dlpfc

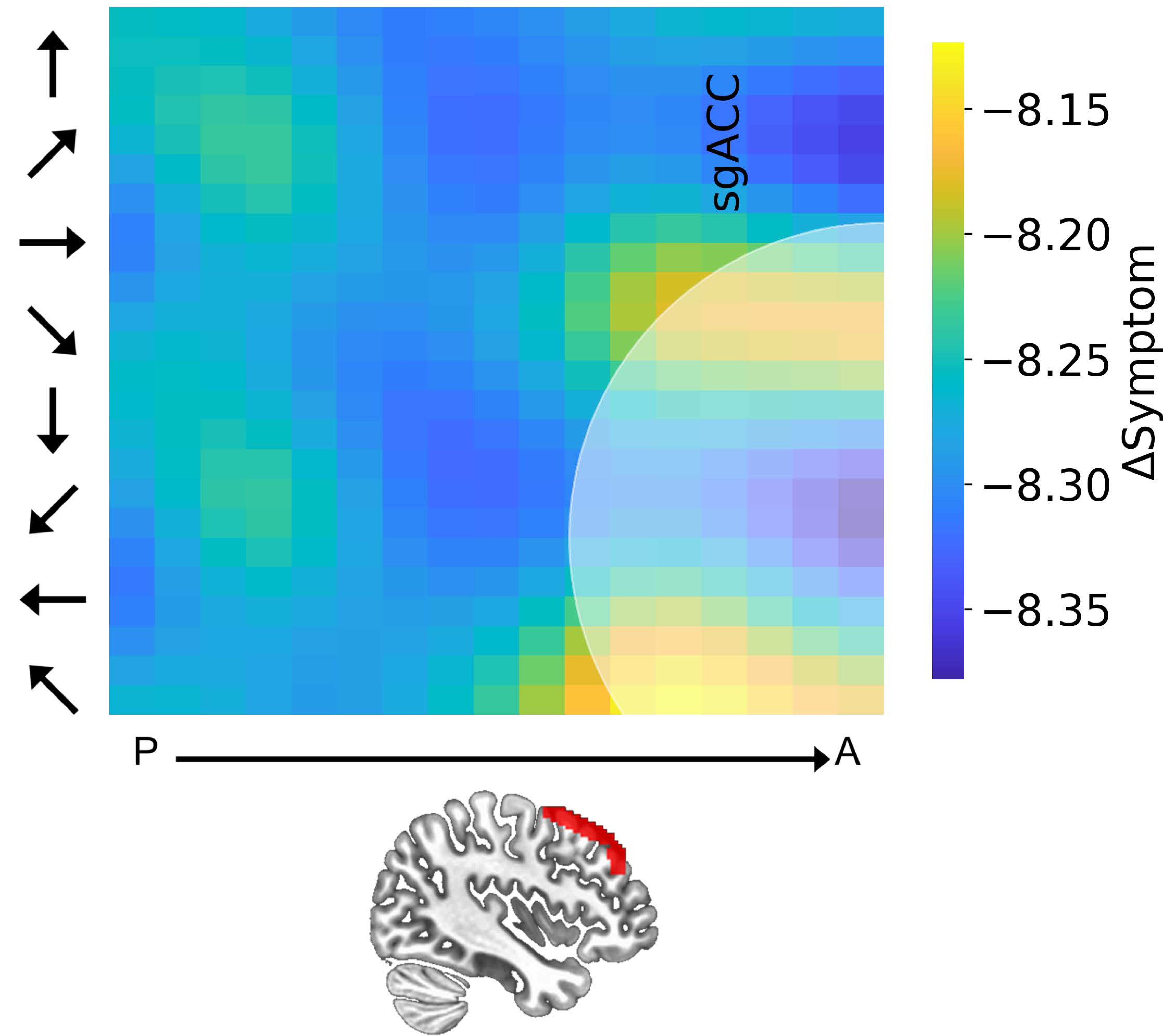

NDARRD555DDH_hamd_left_dlpfc

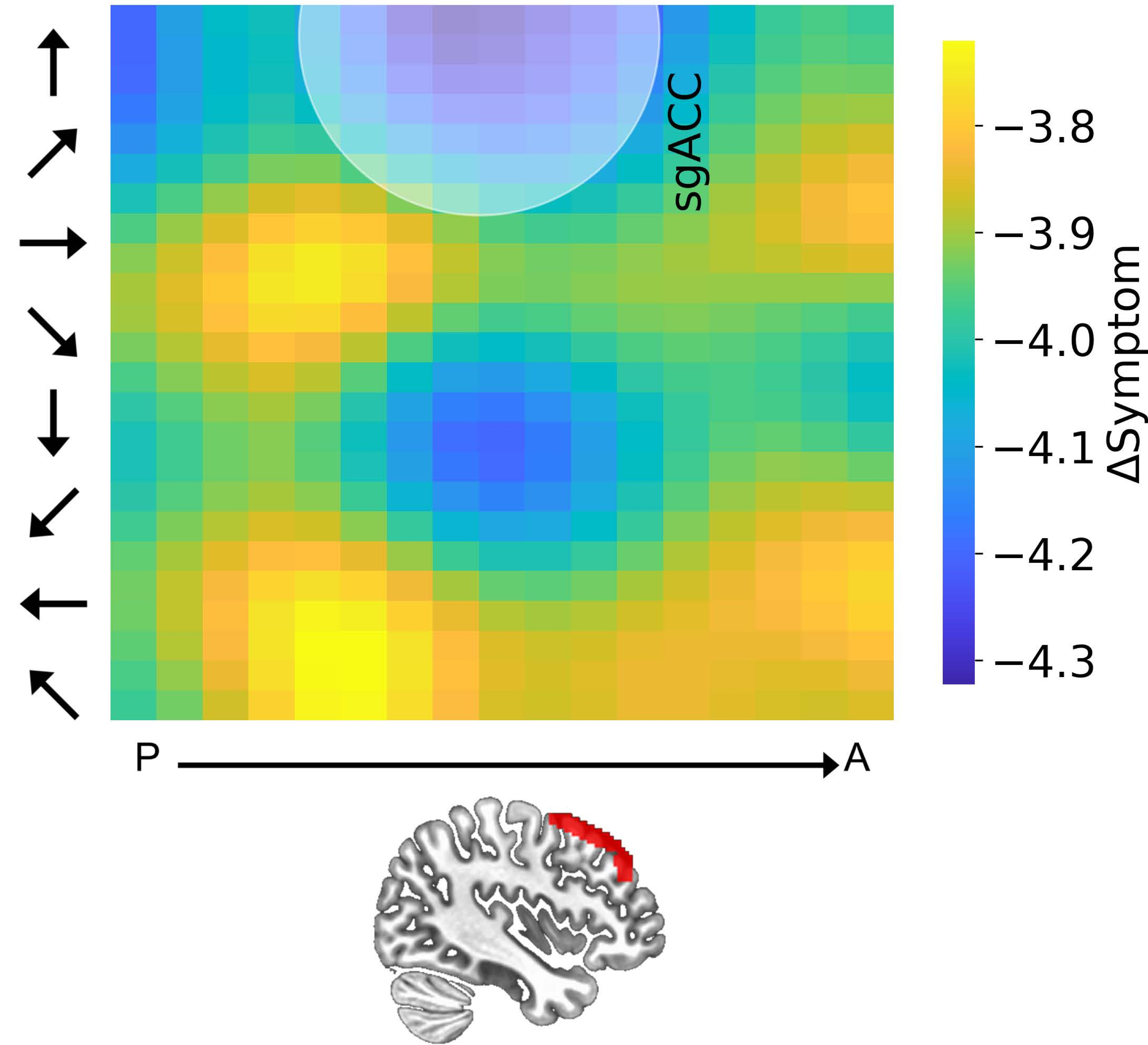

NDARRF879TA3_hamd_left_dlpfc

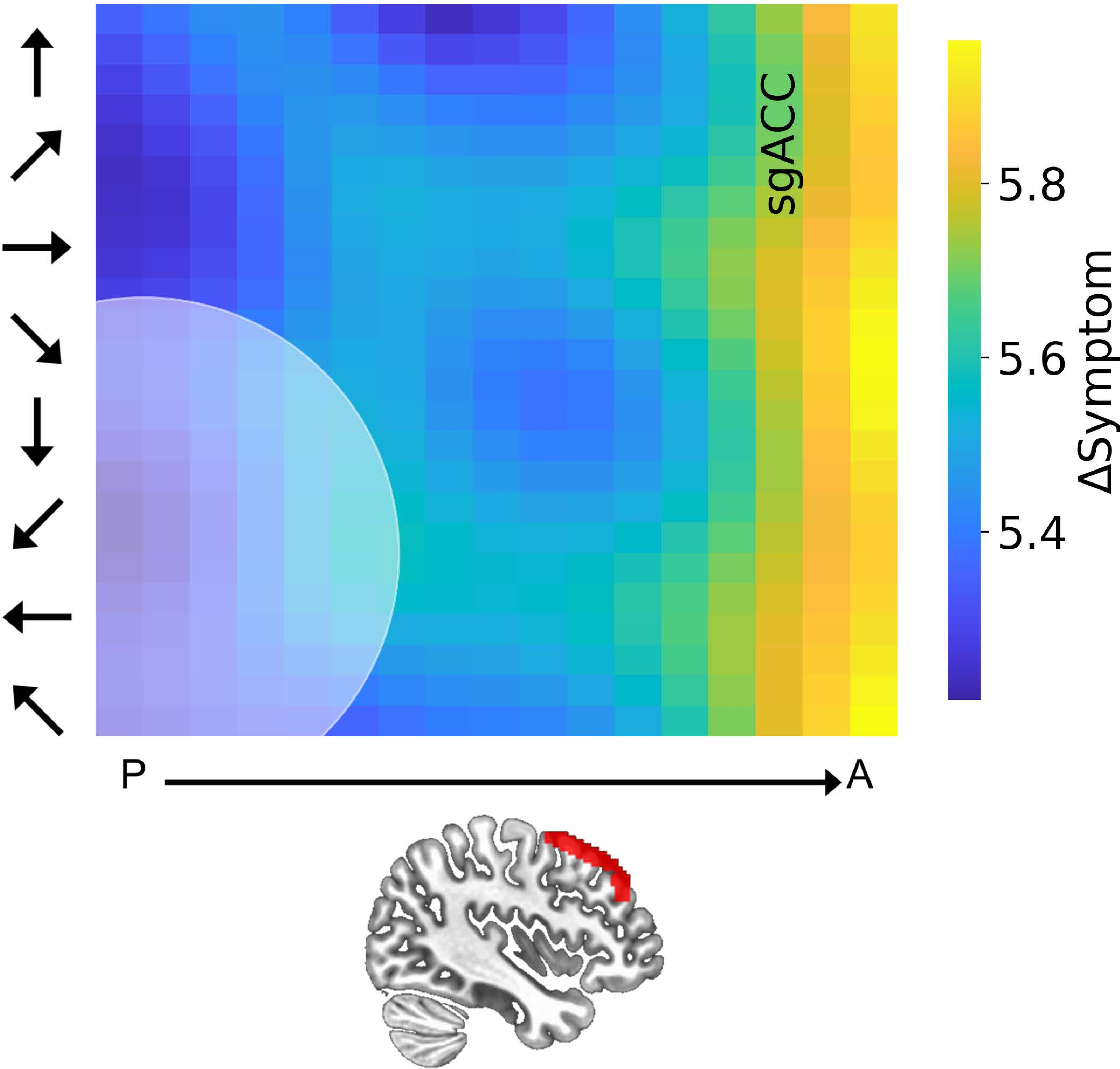

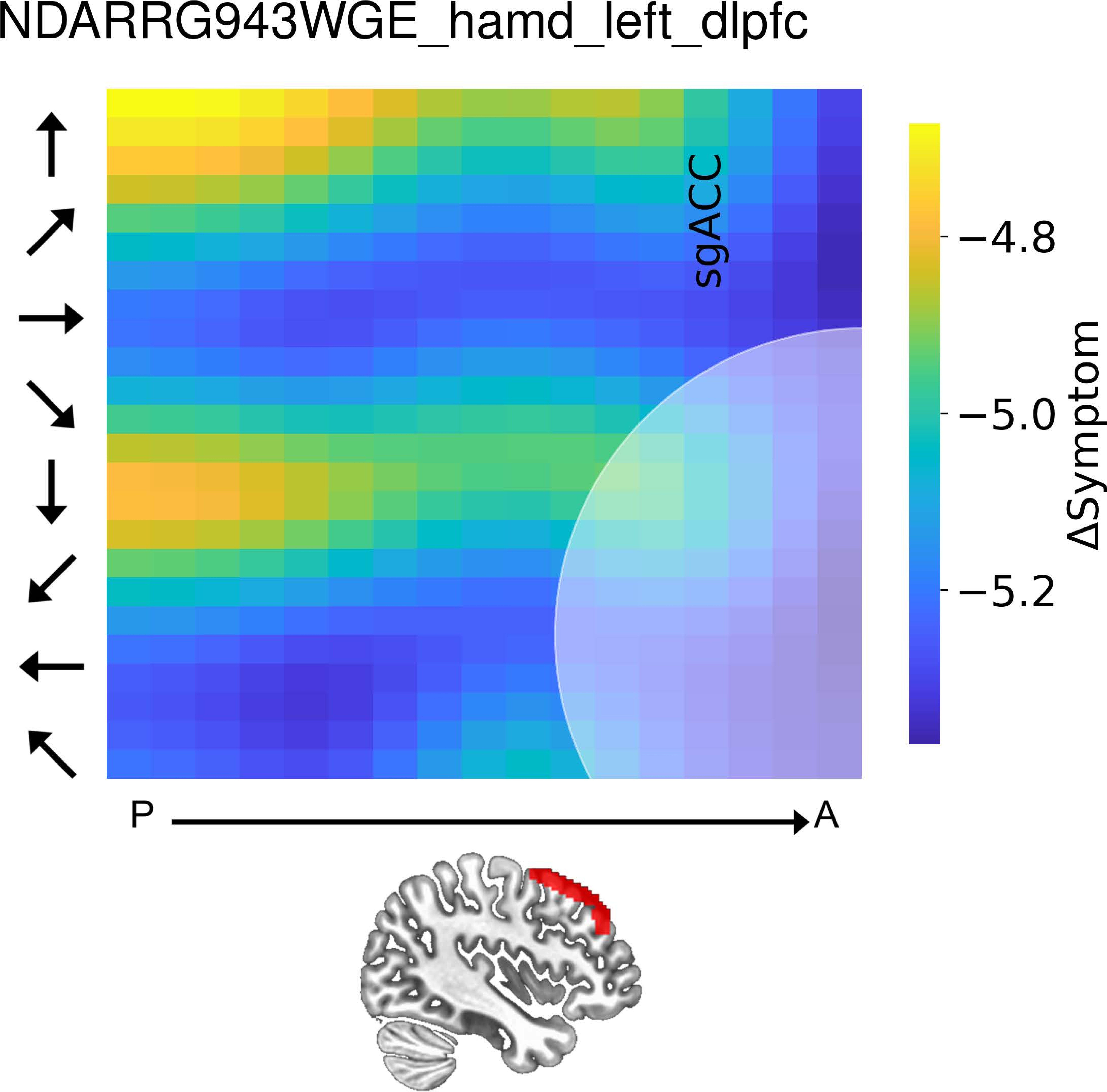
NDARRG943WGE_hamd_left_dlpfc
sgACC
−4.8
−5.0
−5.2
ΔSymptom
P
A

NDARRM328EP8_hamd_left_dlpfc

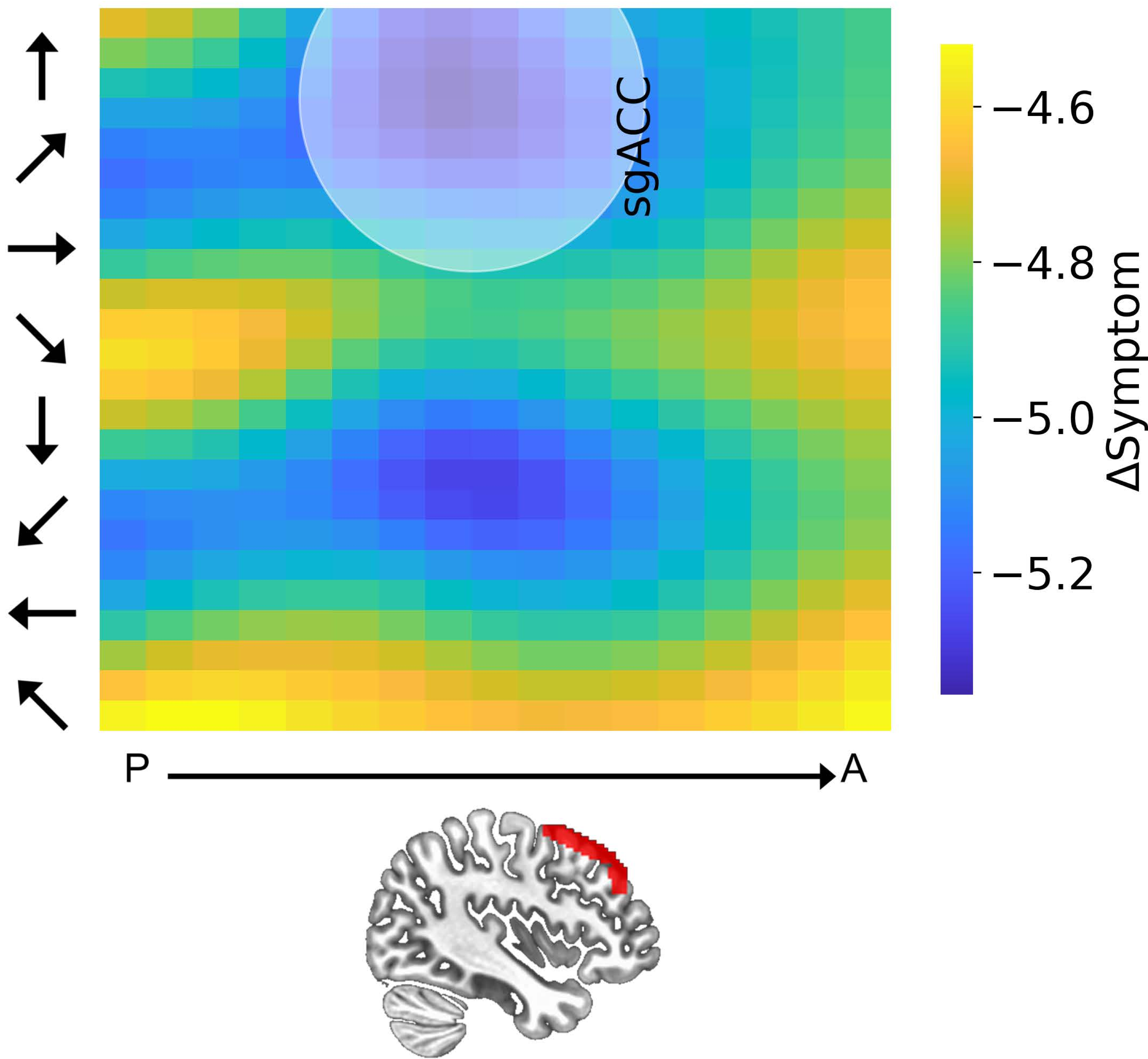

NDARRZ612NKG_hamd_left_dlpfc

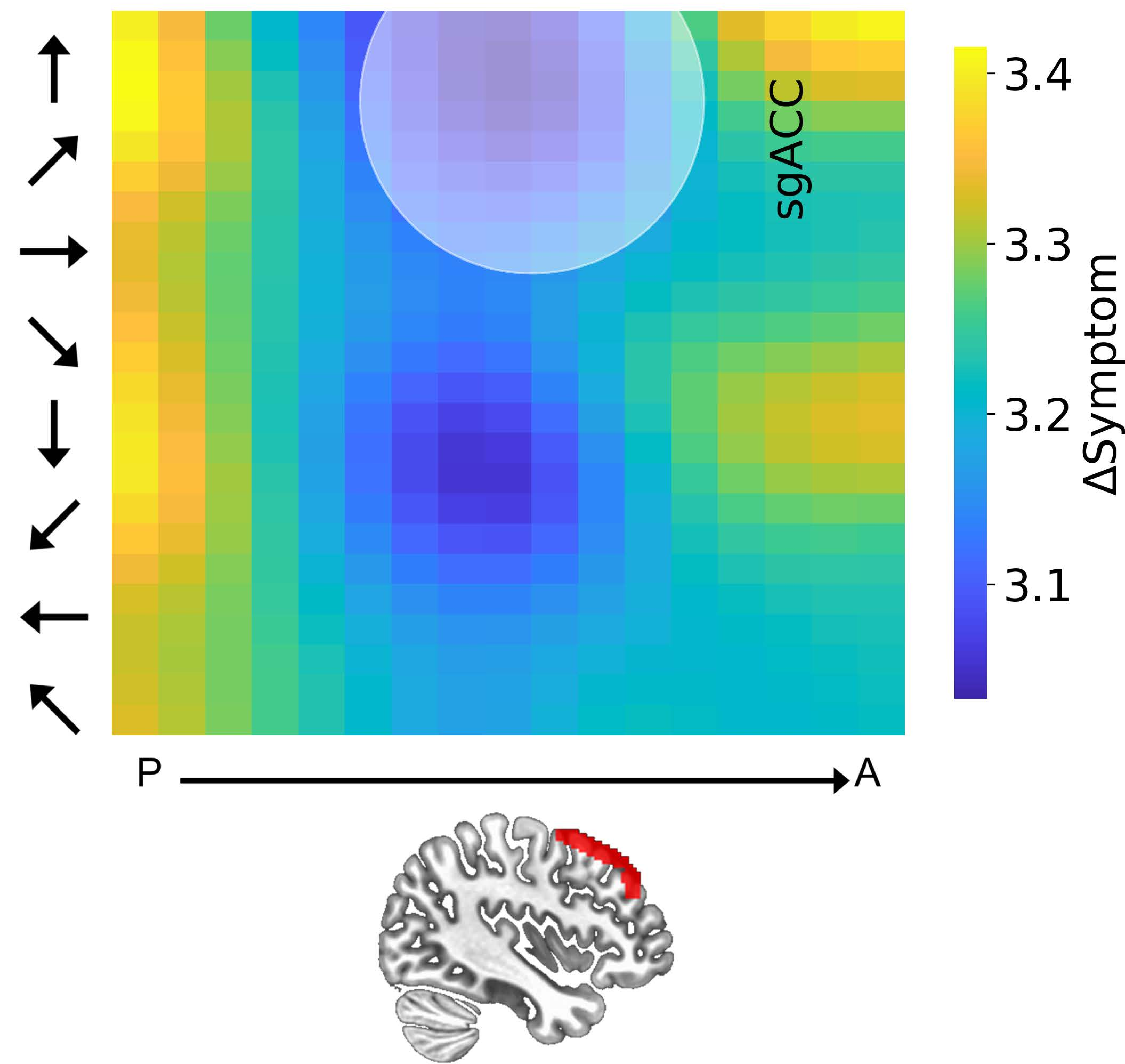

NDARTH509MNV_hamd_left_dlpfc

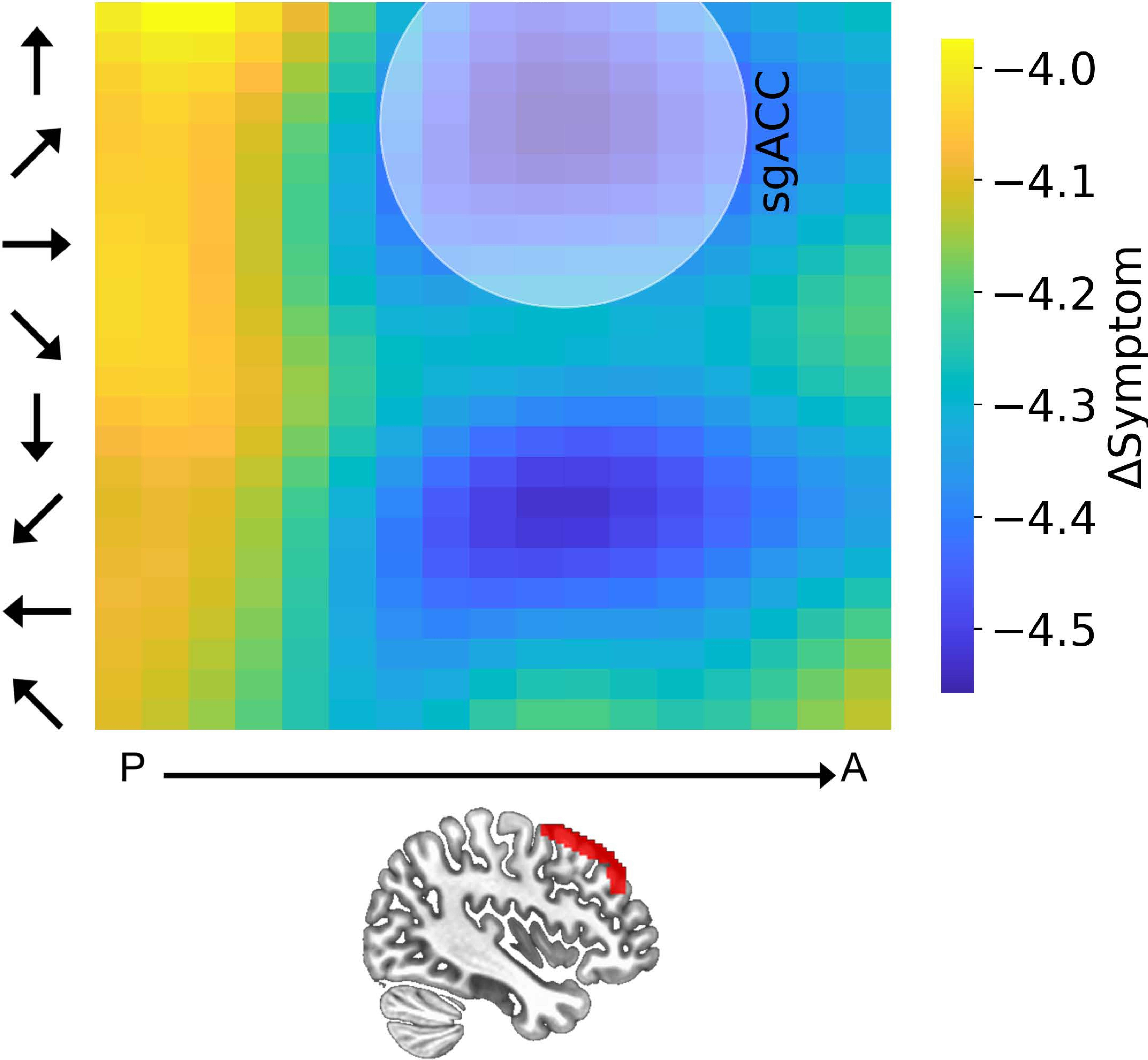

NDARTT796GJK_hamd_left_dlpfc

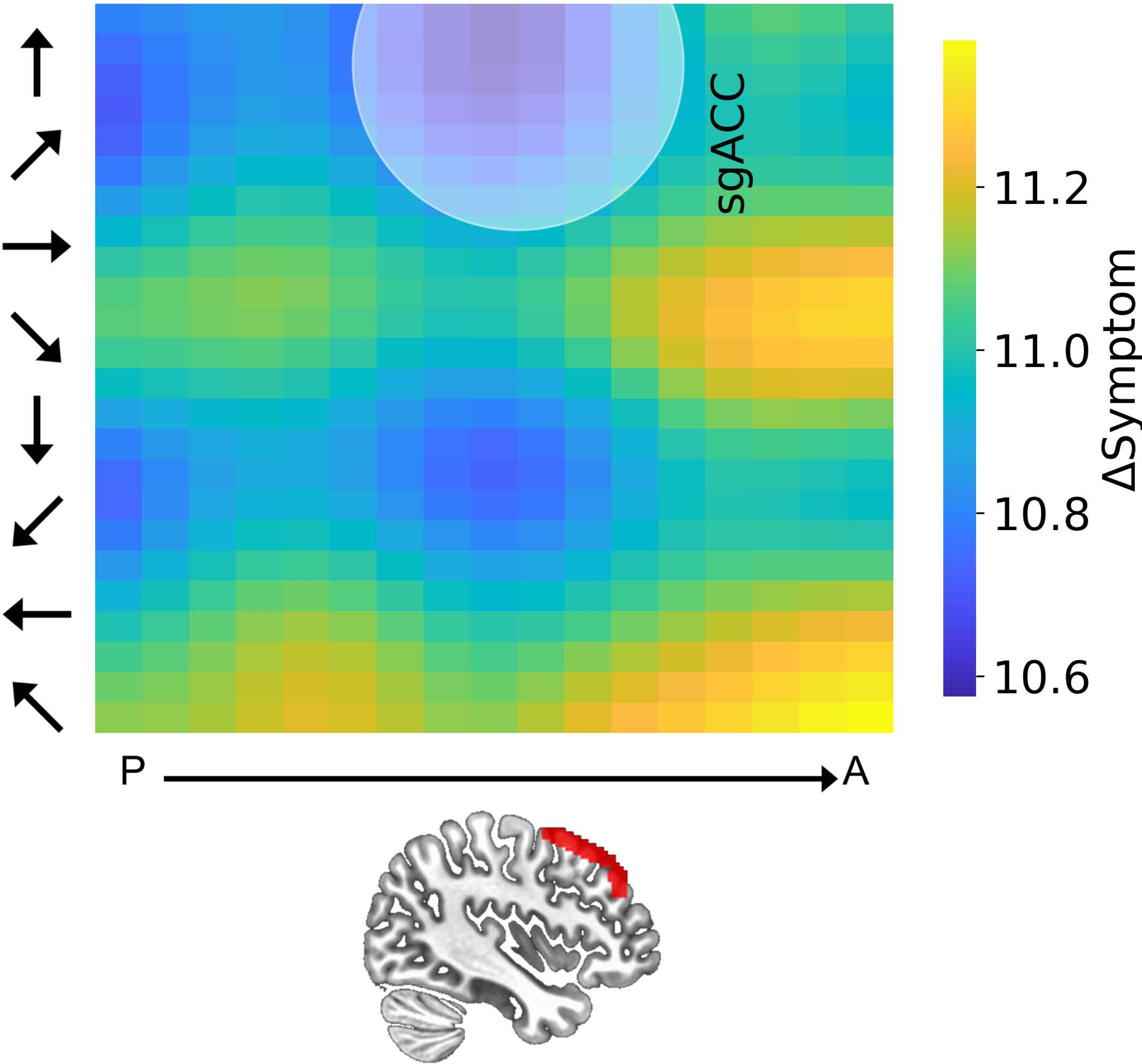

NDARTZ590DYE_hamd_left_dlpfc

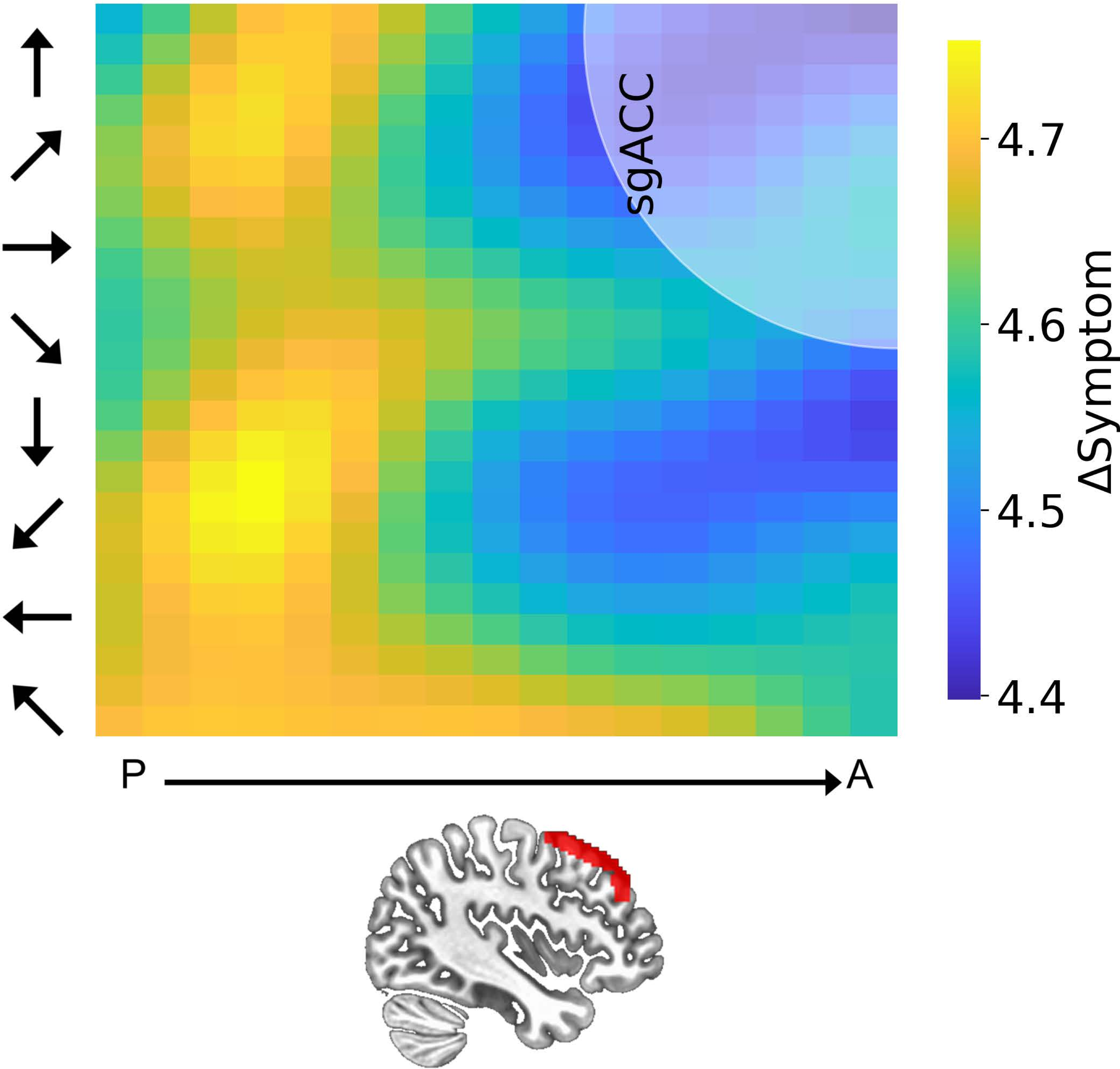

NDARUJ223ZPN_hamd_left_dlpfc

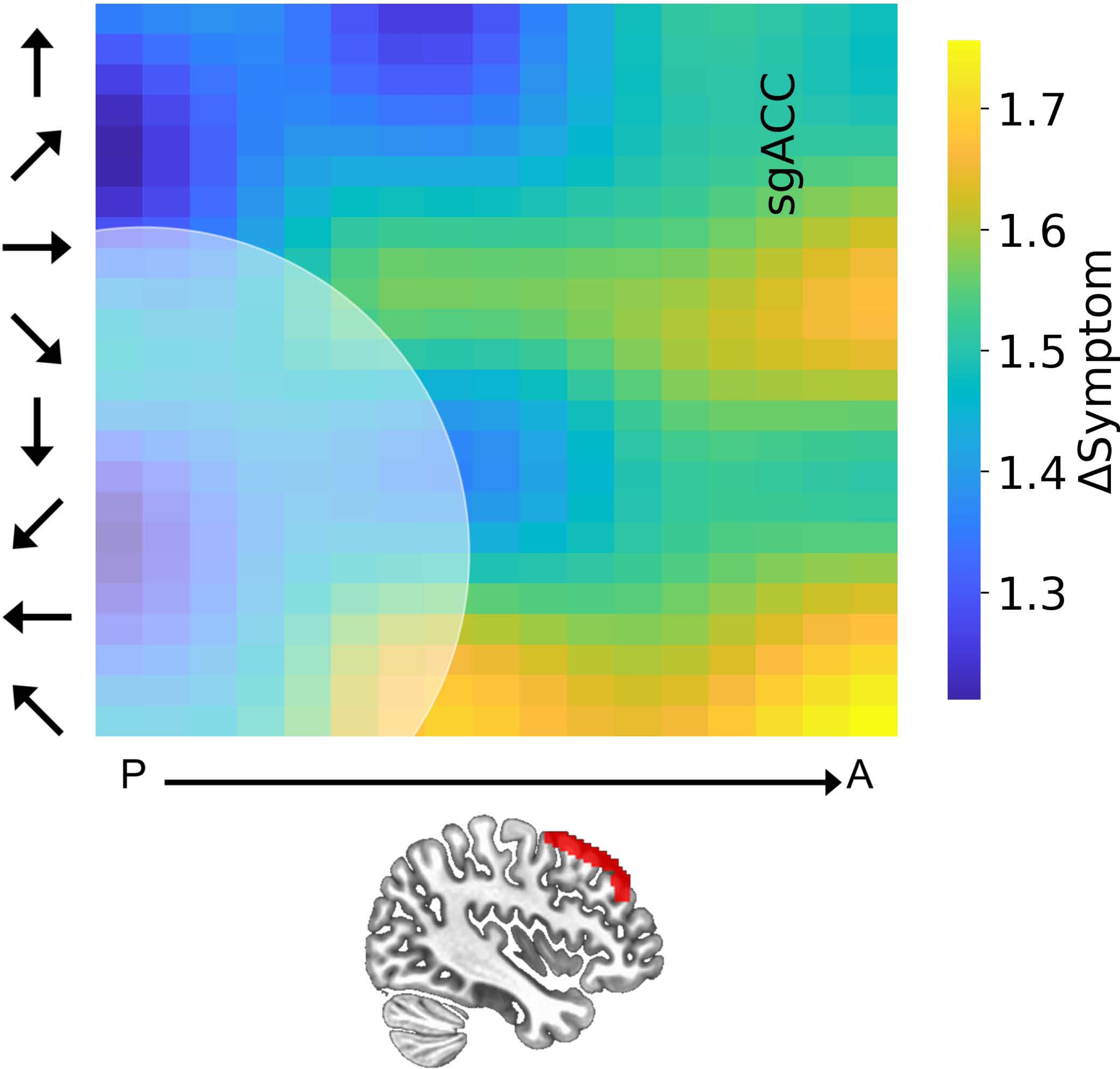

NDARUM818JC1_hamd_left_dlpfc

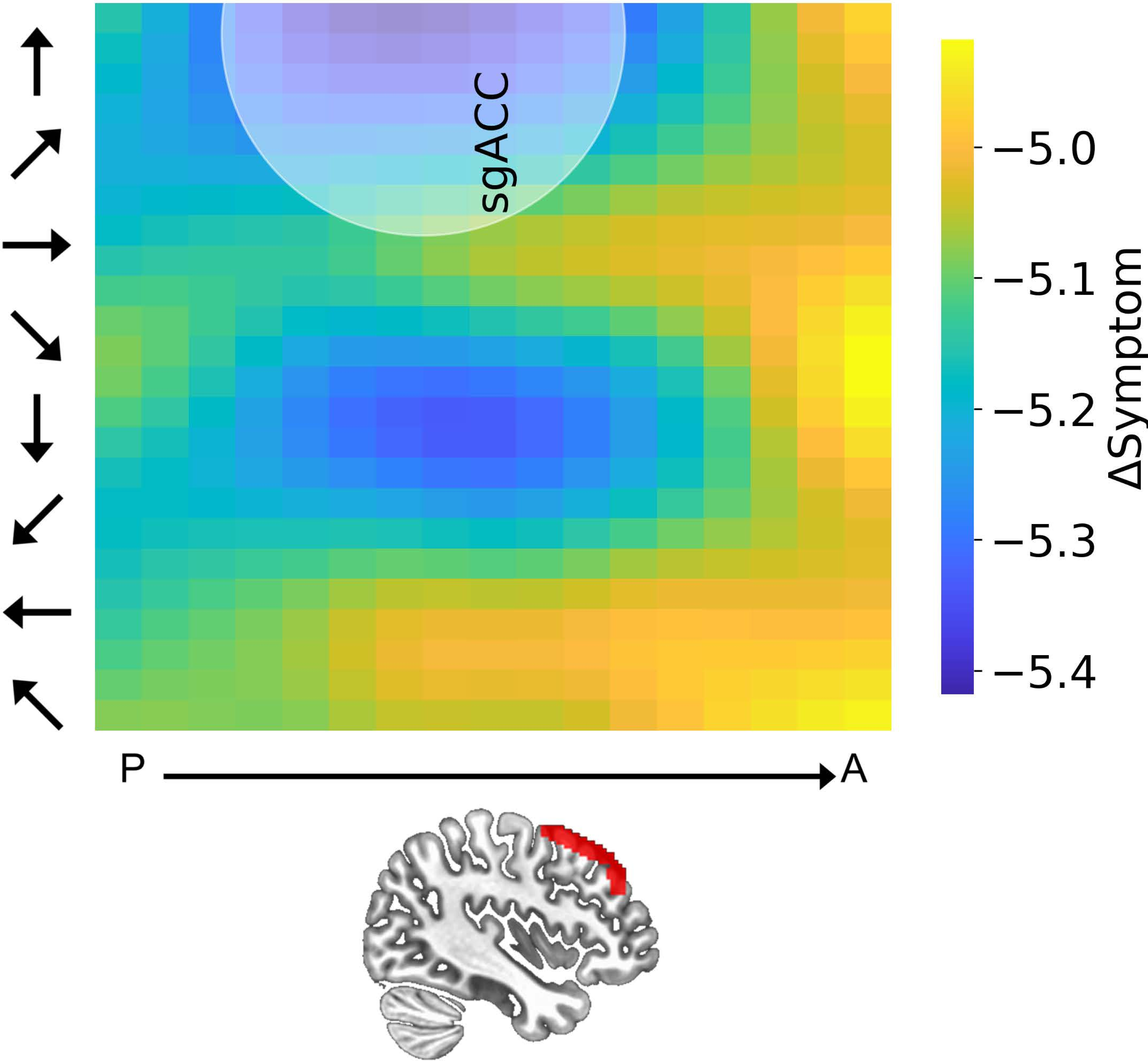

NDARUT852KUX_hamd_left_dlpfc

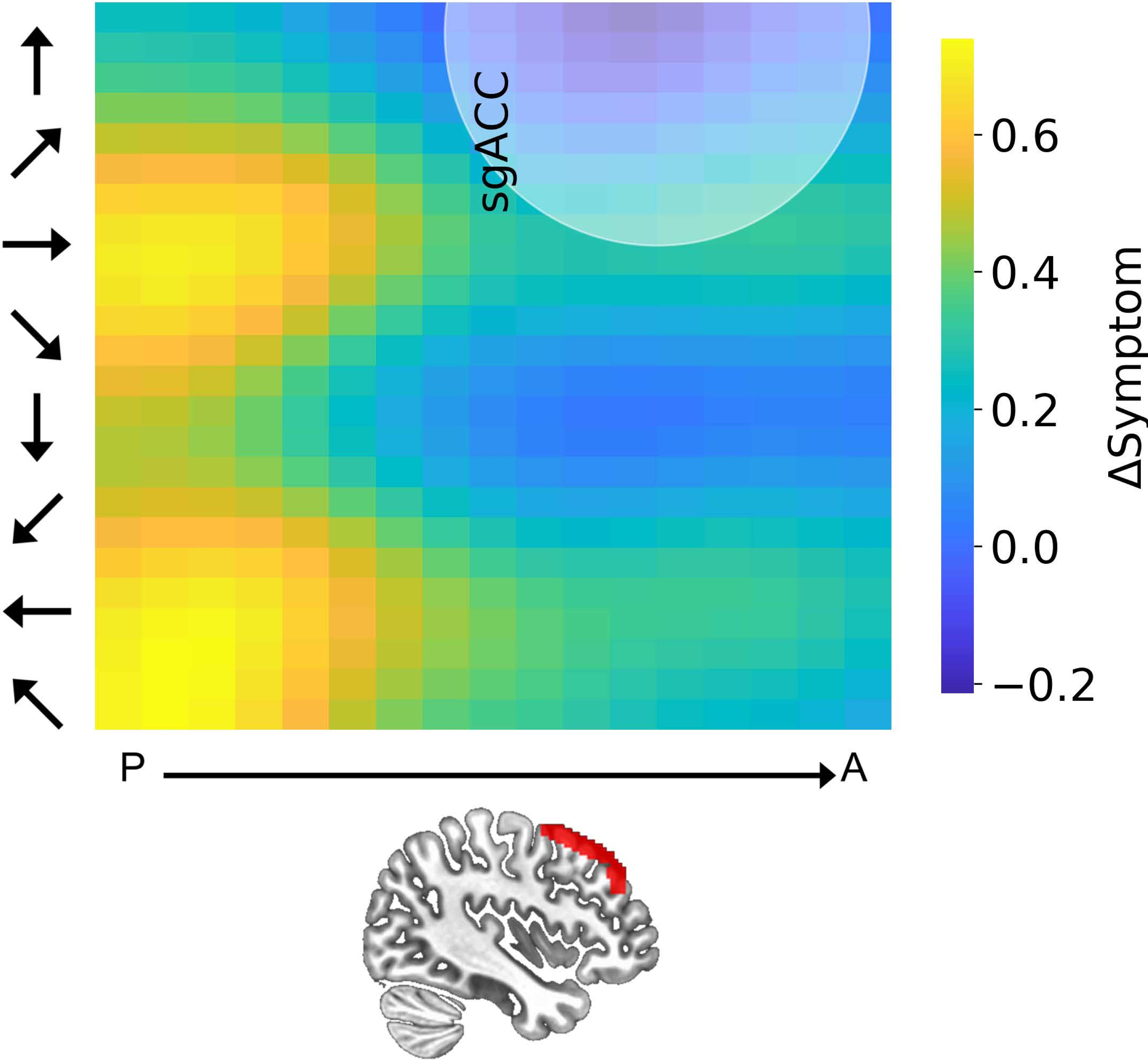

NDARVE642FAN_hamd_left_dlpfc

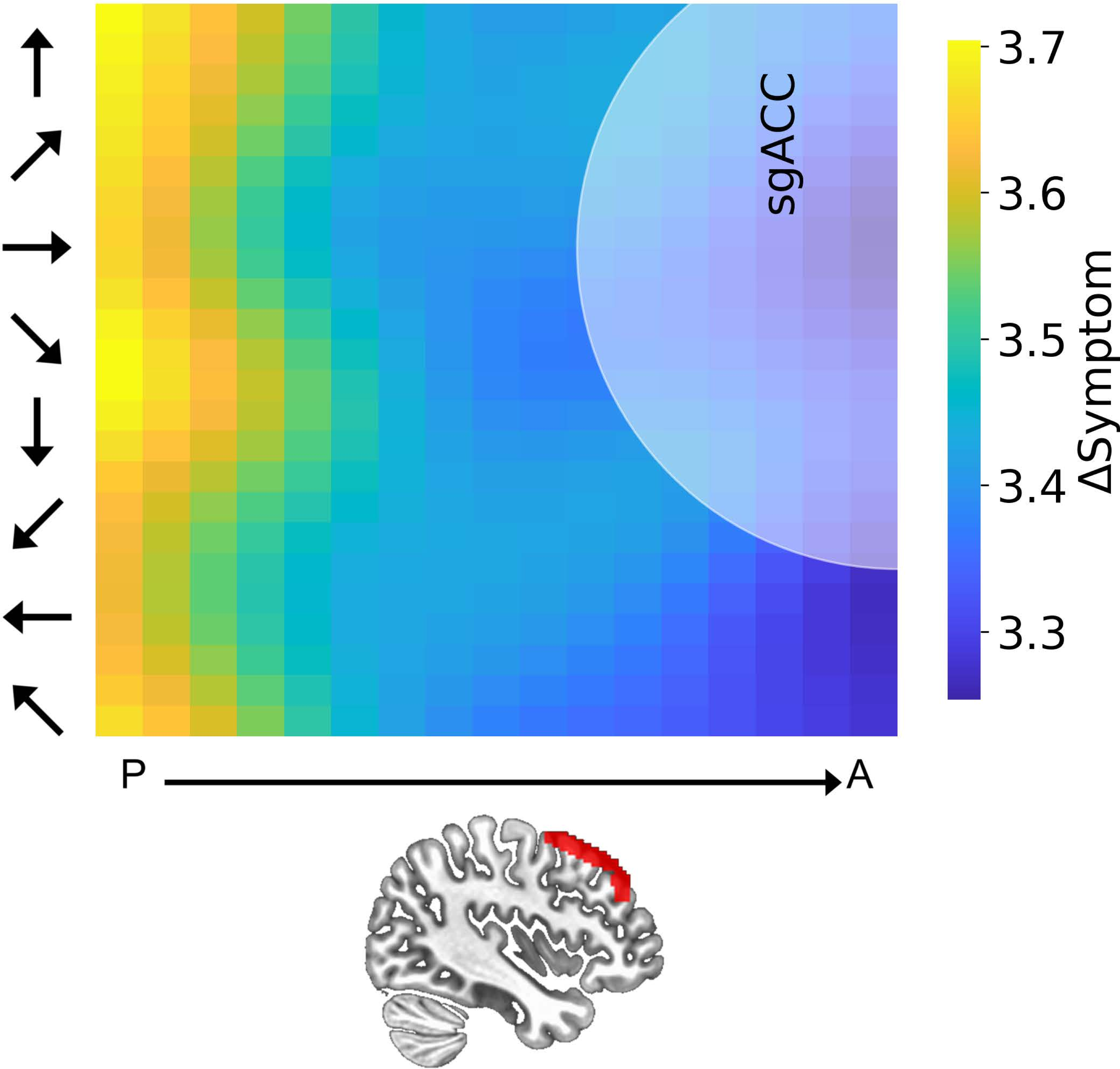

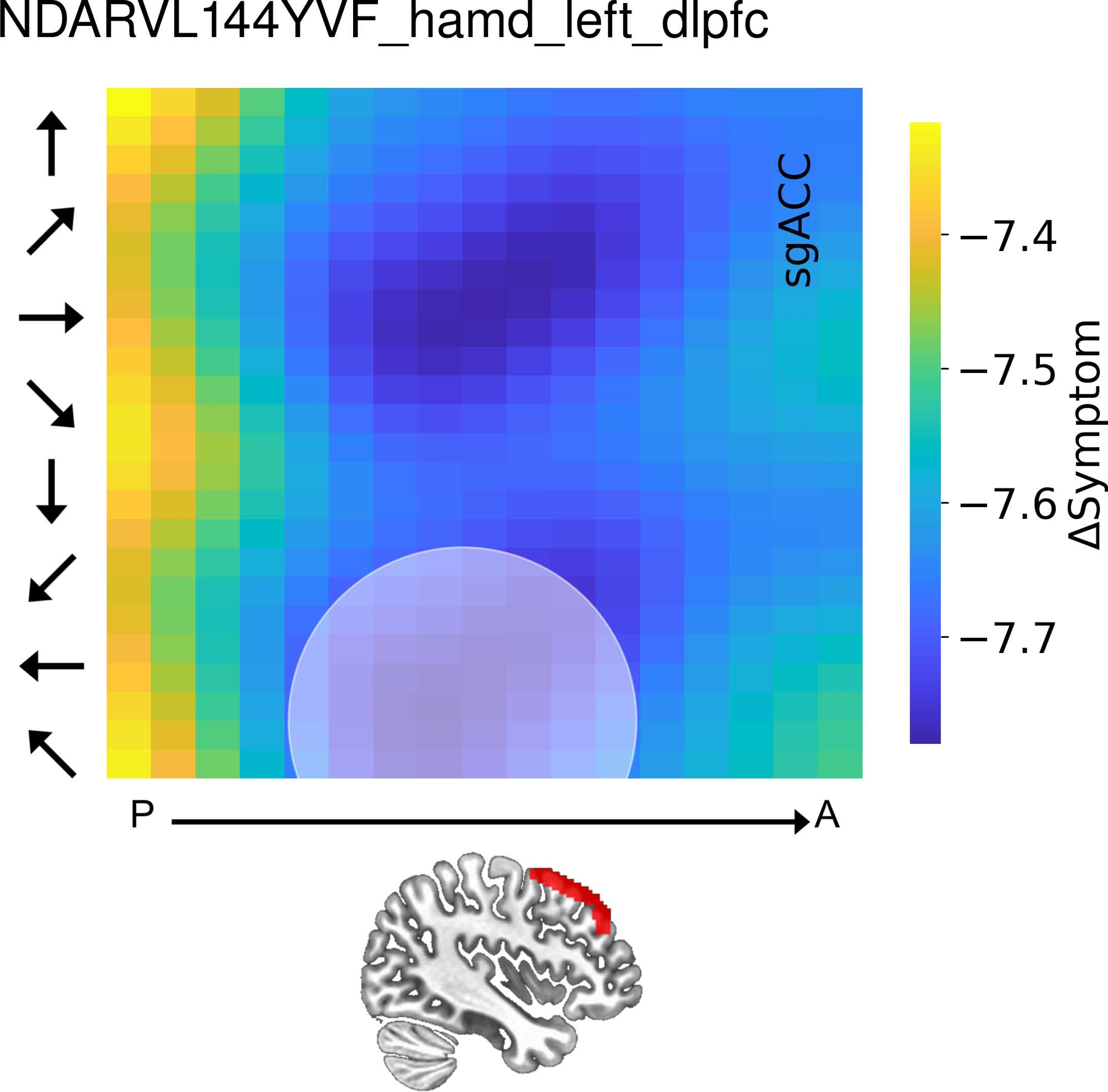

NDARVL144YVF_hamd_left_dlpfc
sgACC
−7.4
−7.5
−7.6
−7.7
ΔSymptom
P
A

NDARWH523DTV_hamd_left_dlpfc

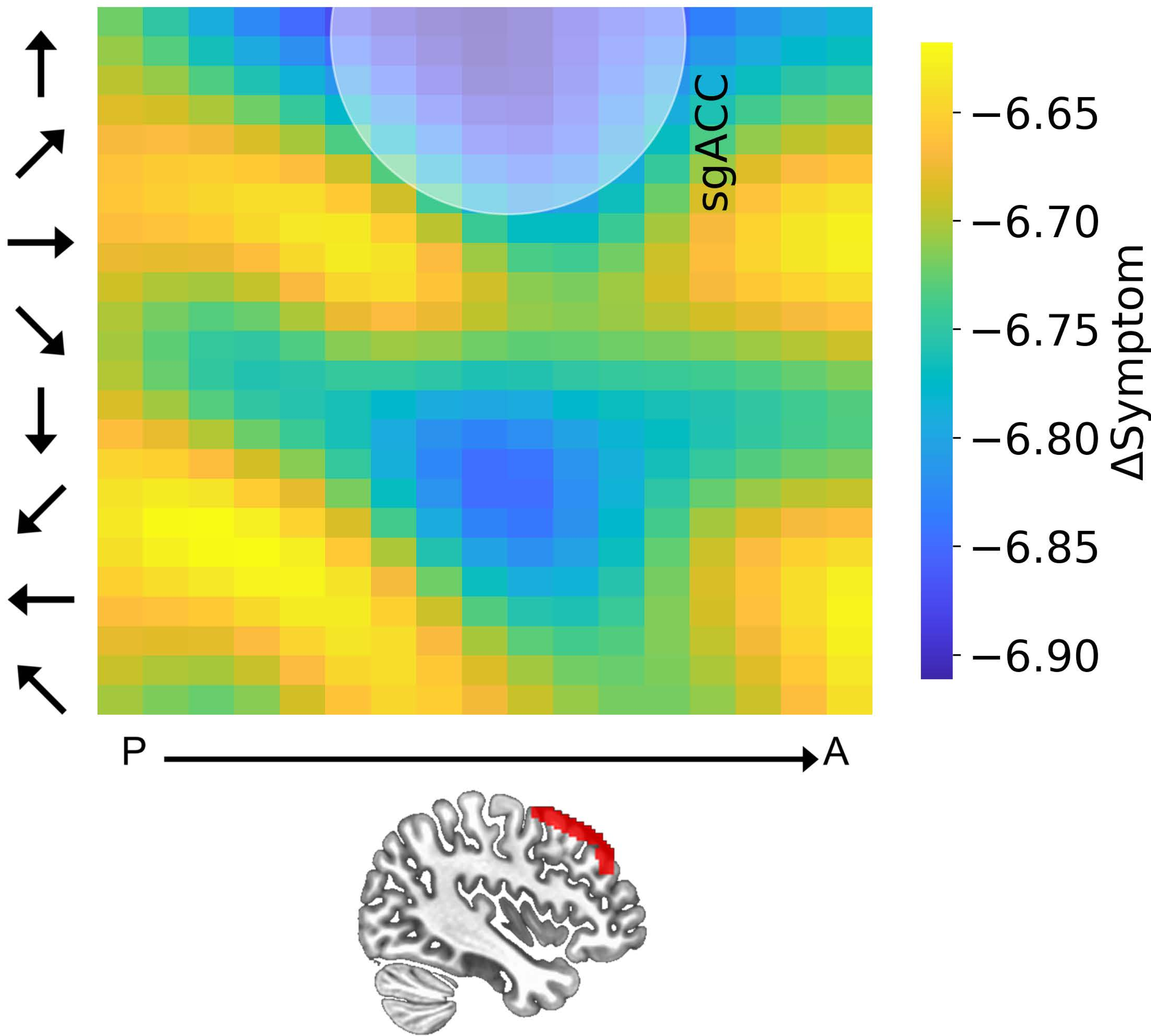

NDARWJ818VXA_hamd_left_dlpfc

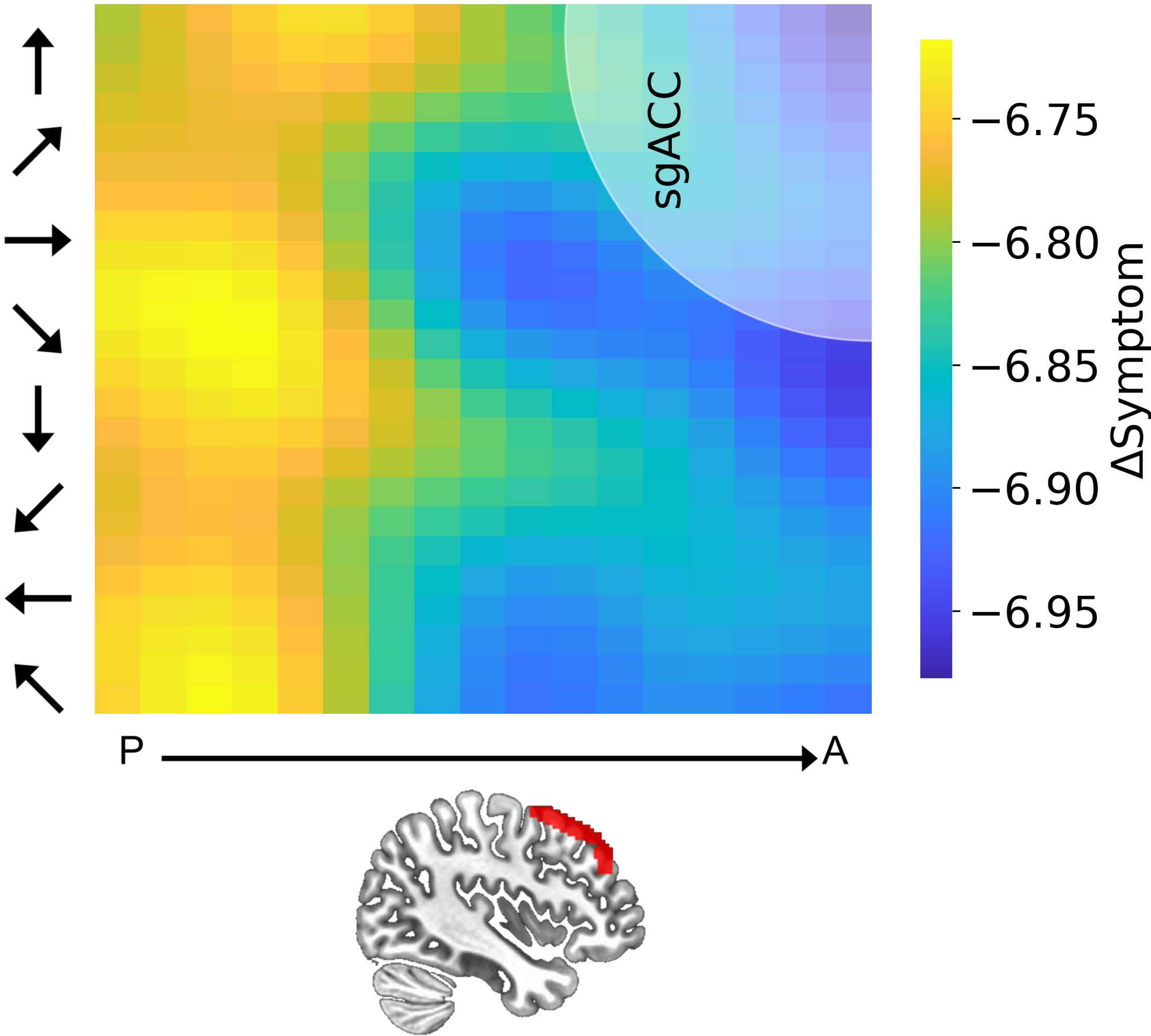

NDARWK081NTV_hamd_left_dlpfc

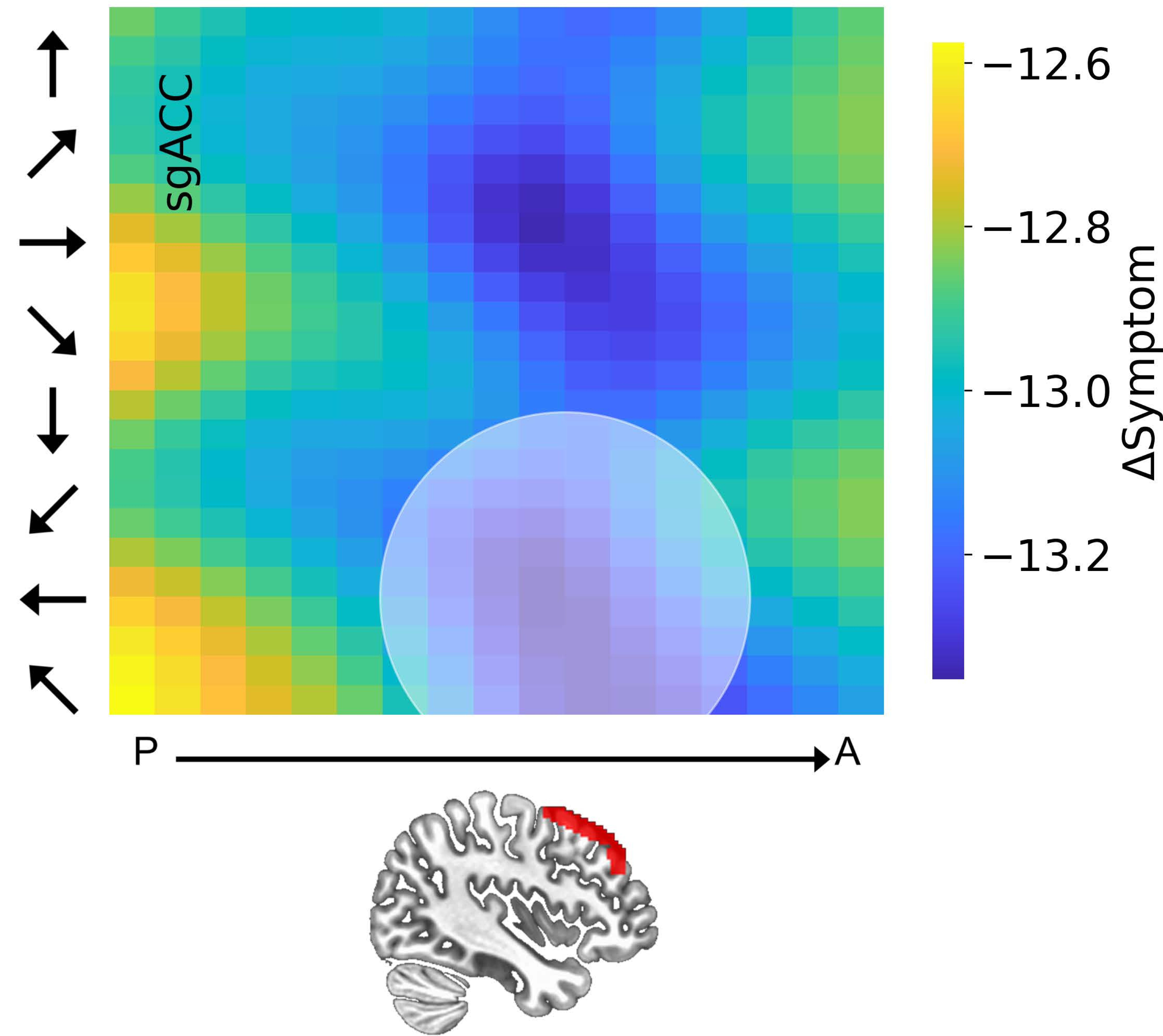

NDARWL277NXF_hamd_left_dlpfc

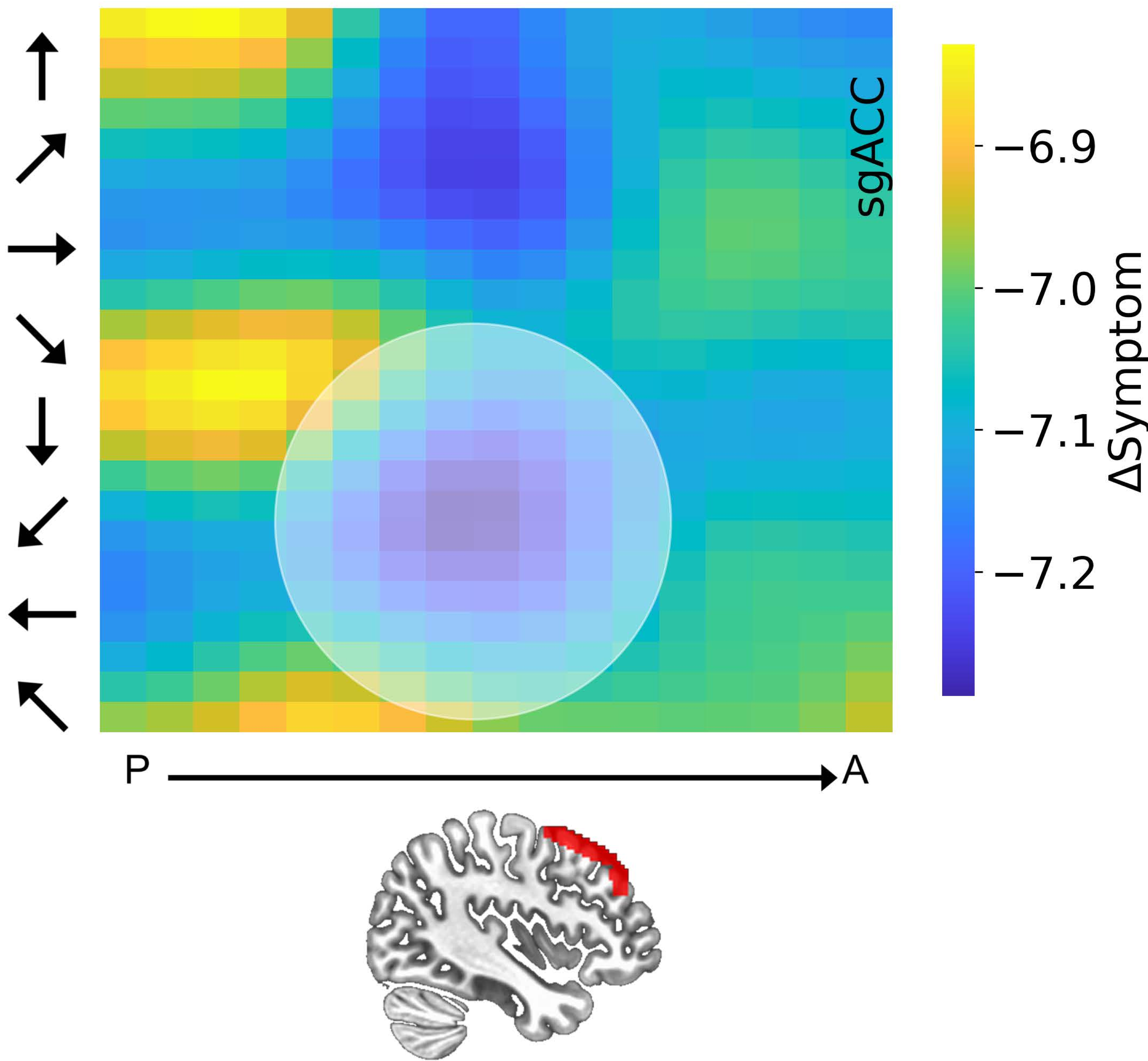

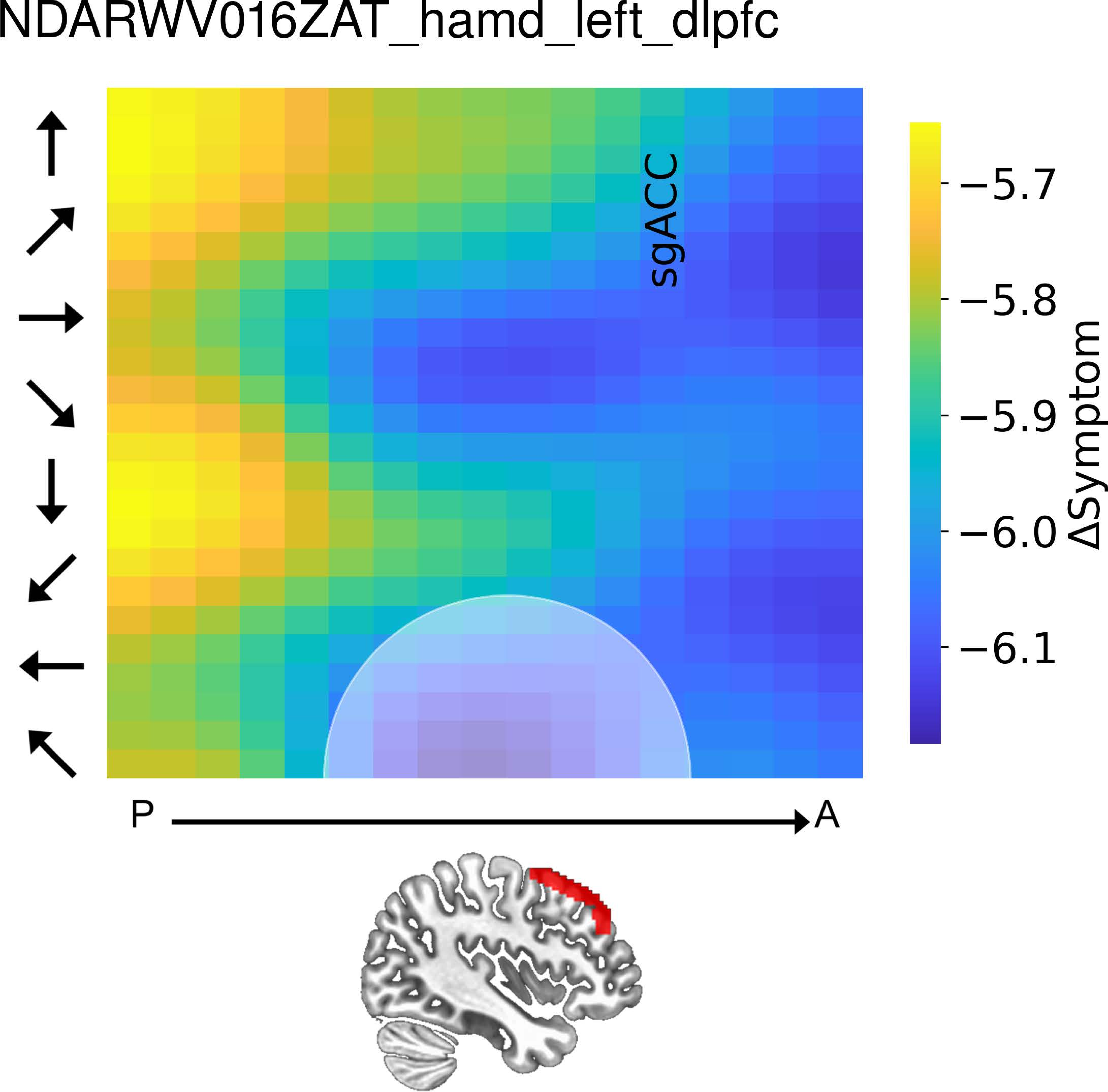
NDARWV016ZAT_hamd_left_dlpfc
sgACC
P
A
ΔSymptom
−5.7
−5.8
−5.9
−6.0
−6.1

NDARXA148KPL_hamd_left_dlpfc

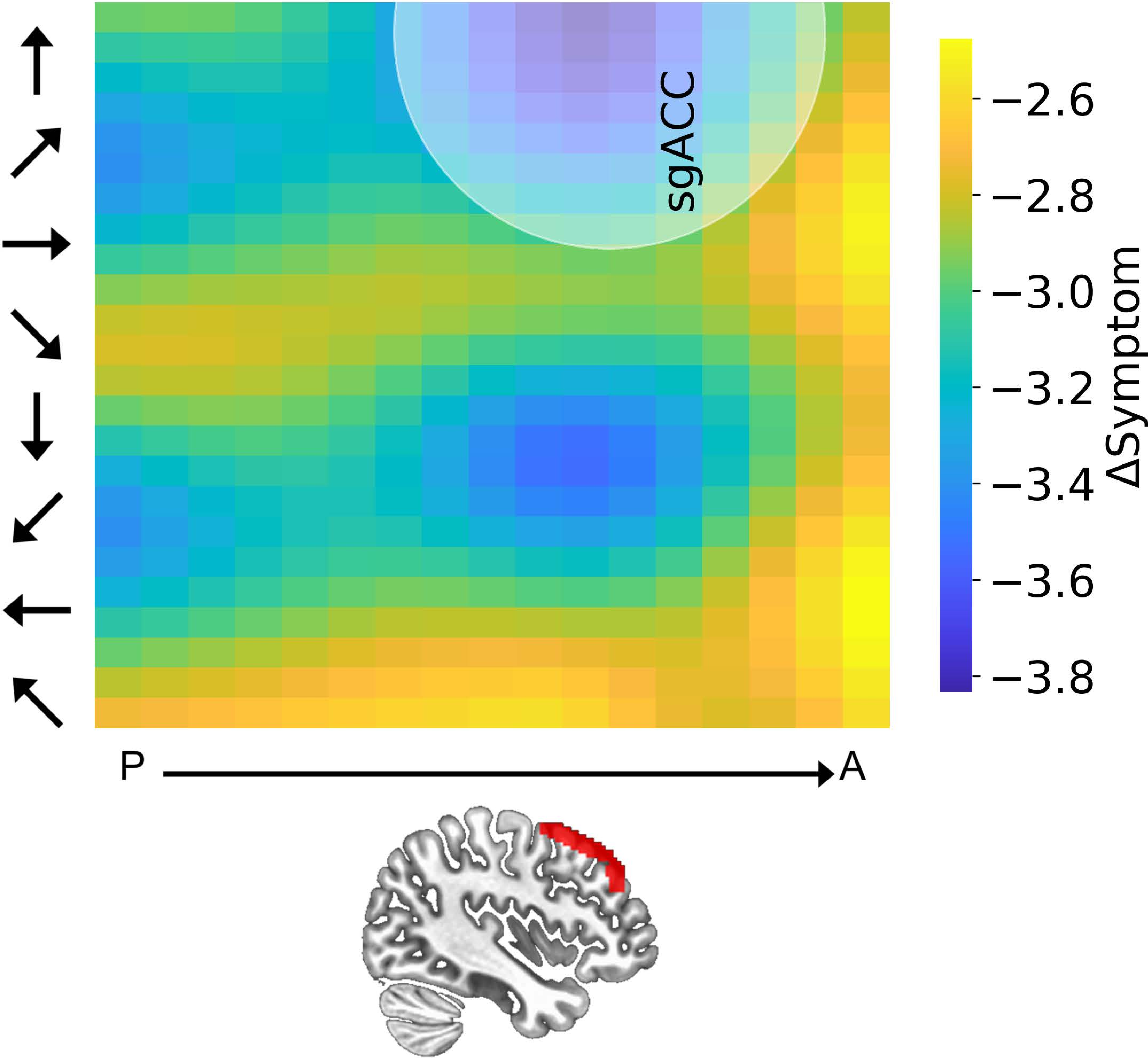

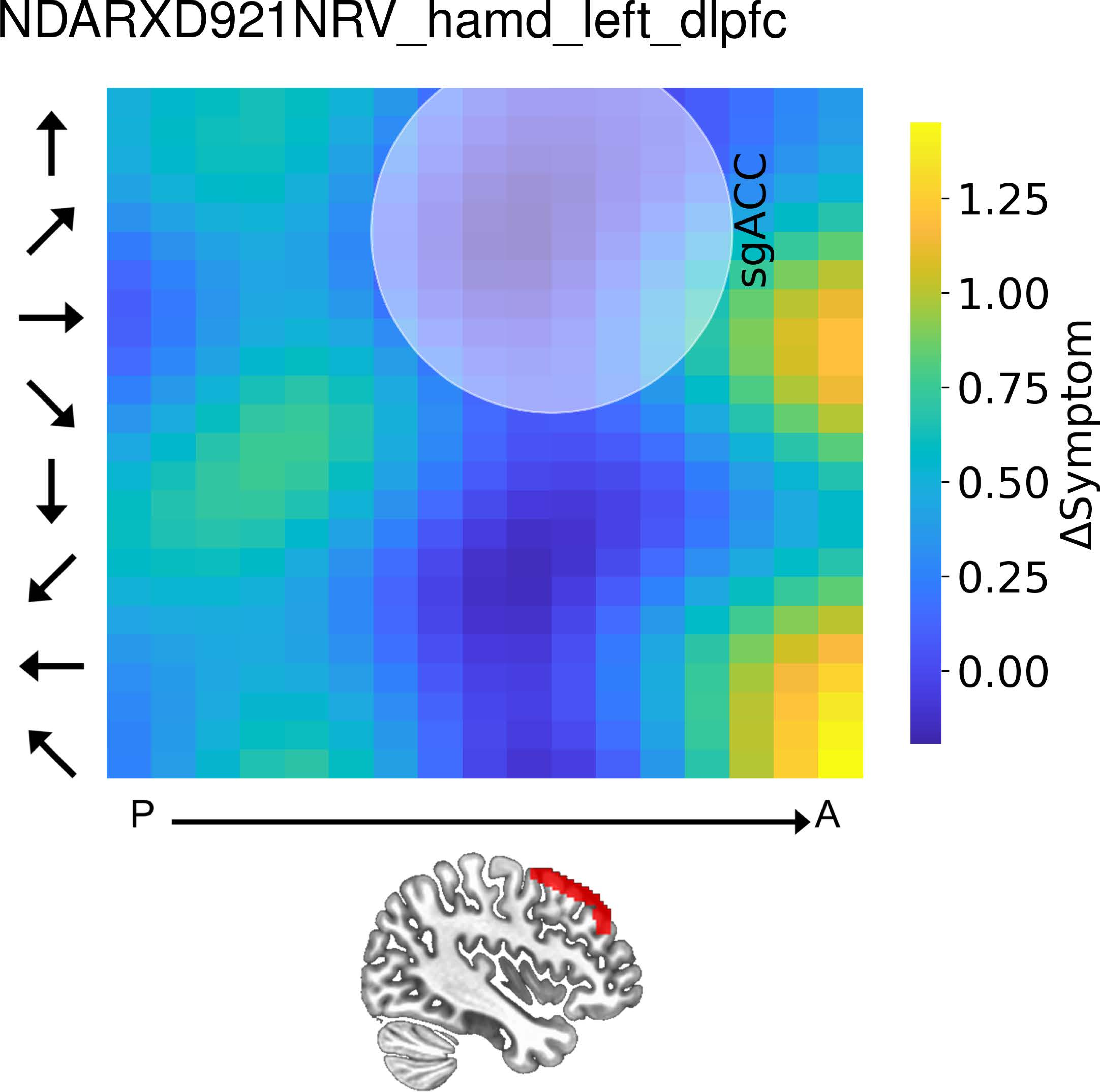
NDARXD921NRV_hamd_left_dlpfc
sgACC
1.25
1.00
0.75
0.50
0.25
0.00
ΔSymptom
P
A

NDARXU500EFT_hamd_left_dlpfc

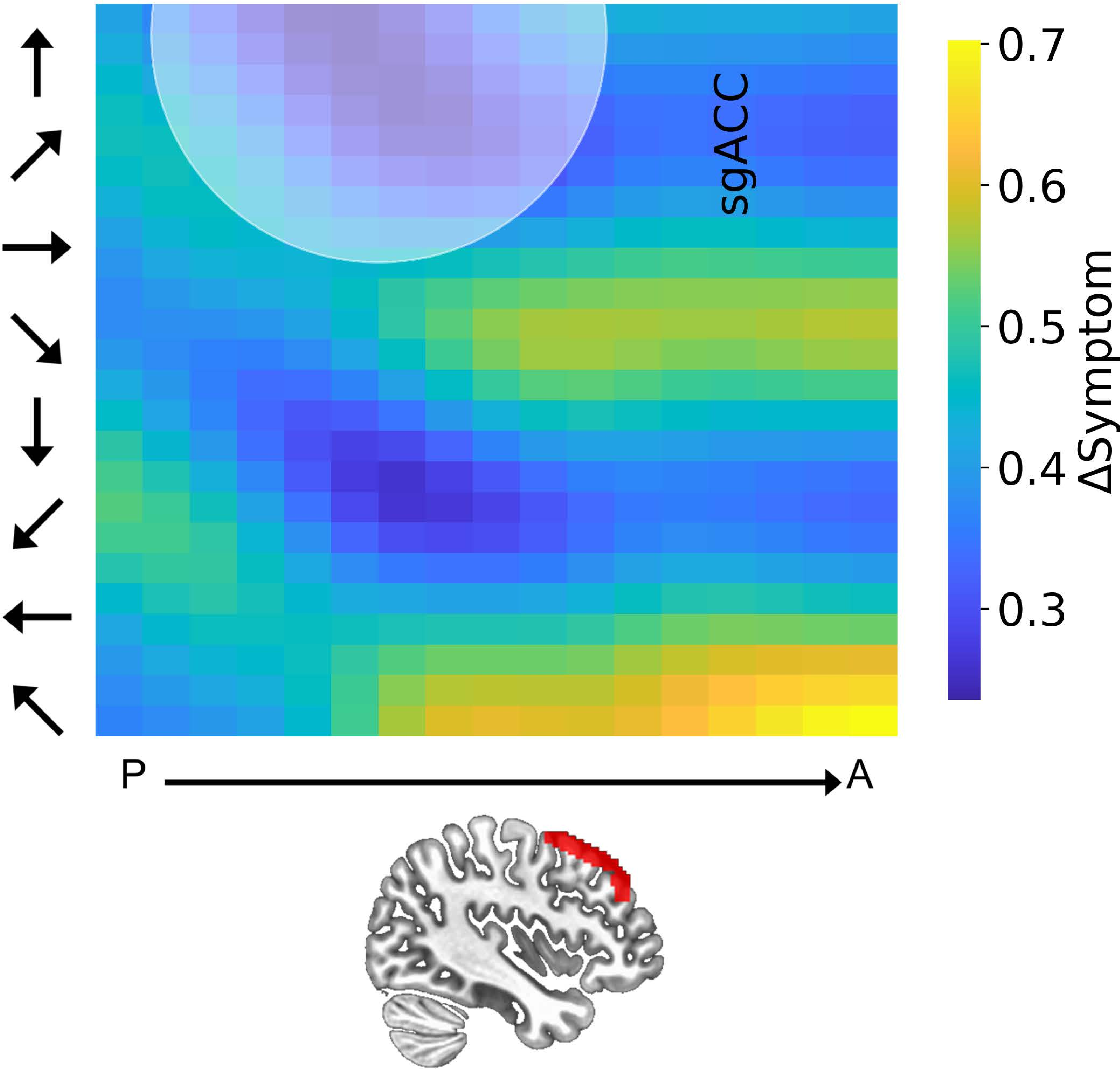

NDARYB181GTB_hamd_left_dlpfc

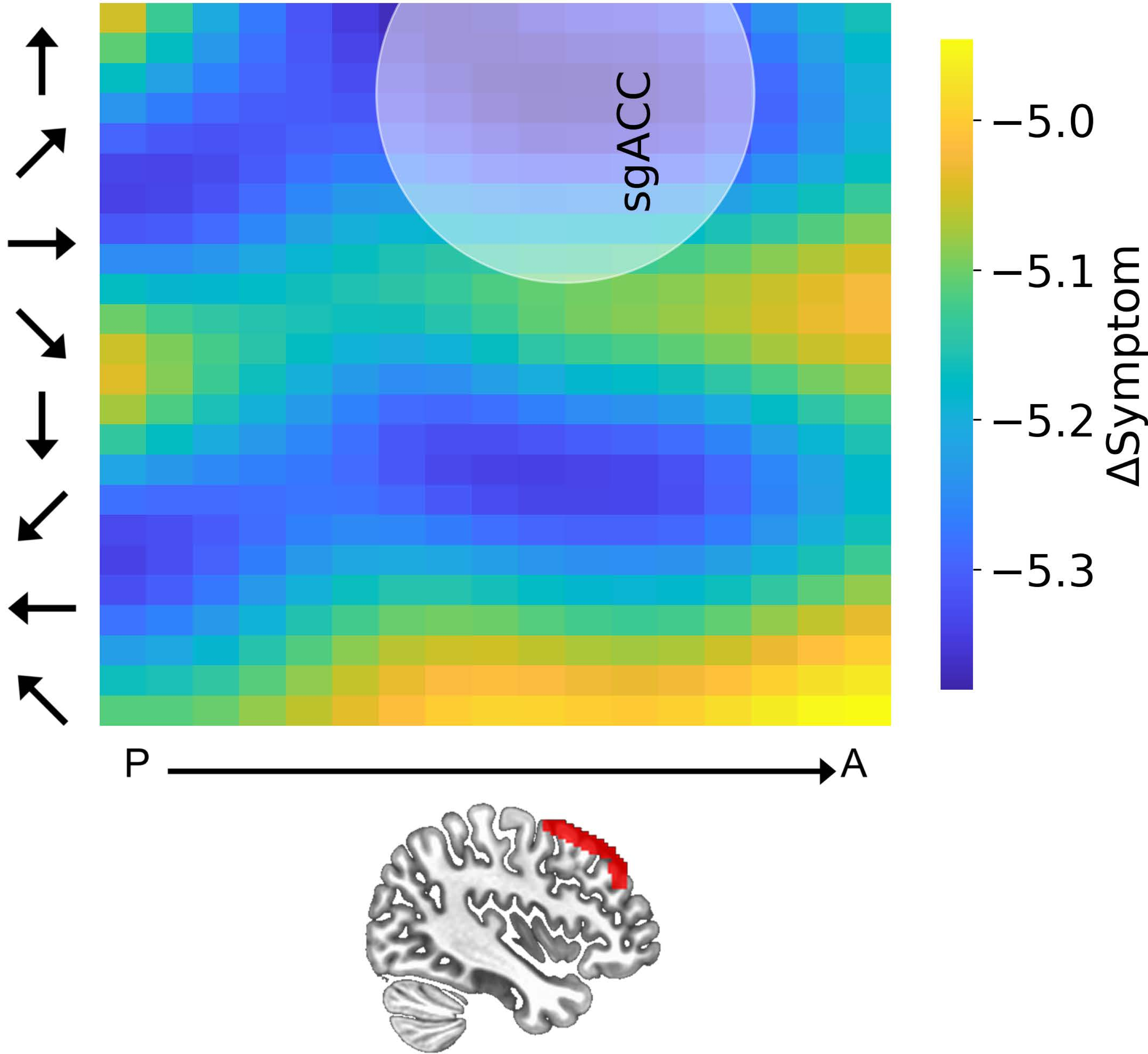

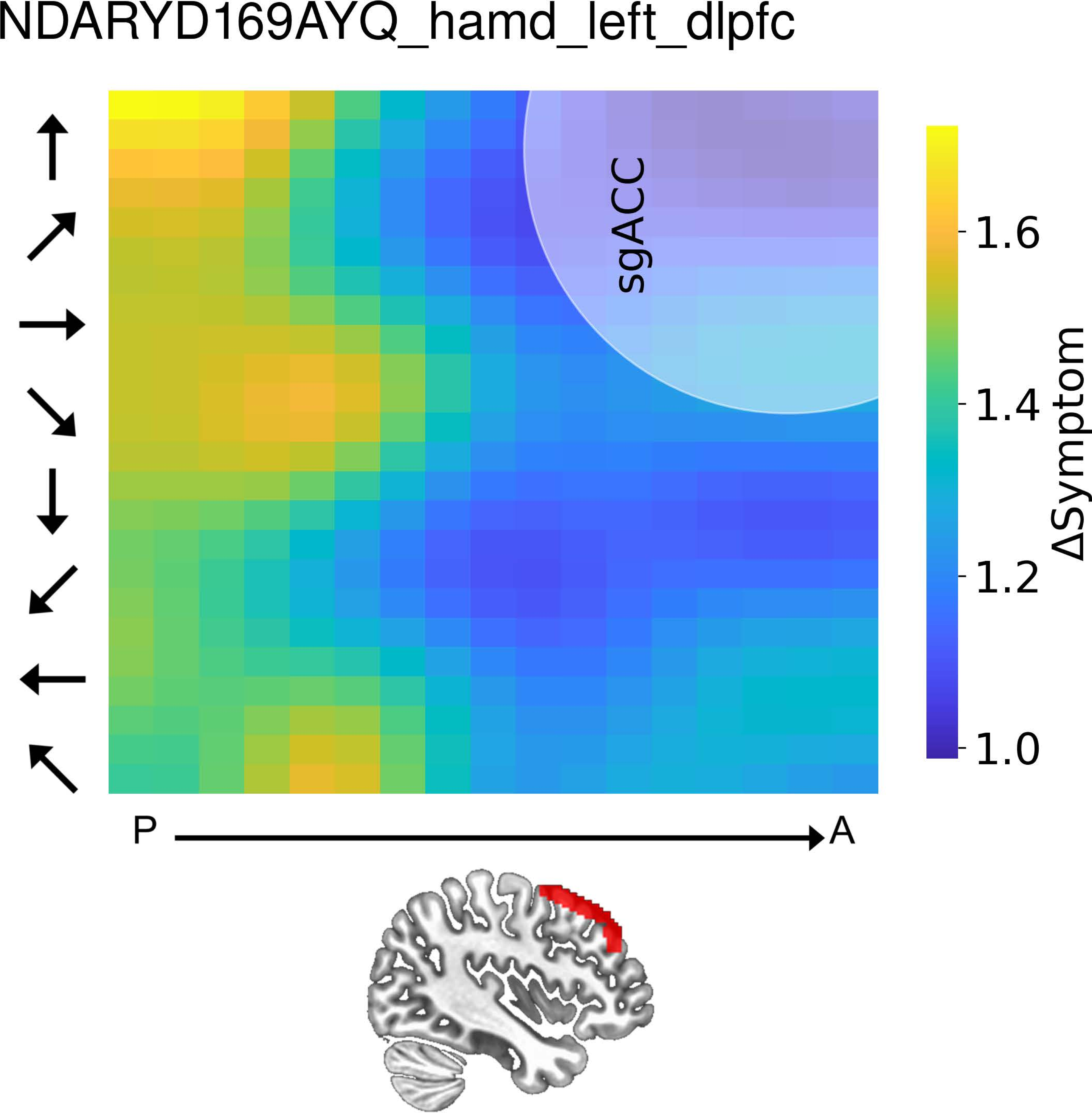

NDARYD169AYQ_hamd_left_dlpfc
sgACC
P
A
ΔSymptom
1.6
1.4
1.2
1.0

NDARYE405JJL_hamd_left_dlpfc

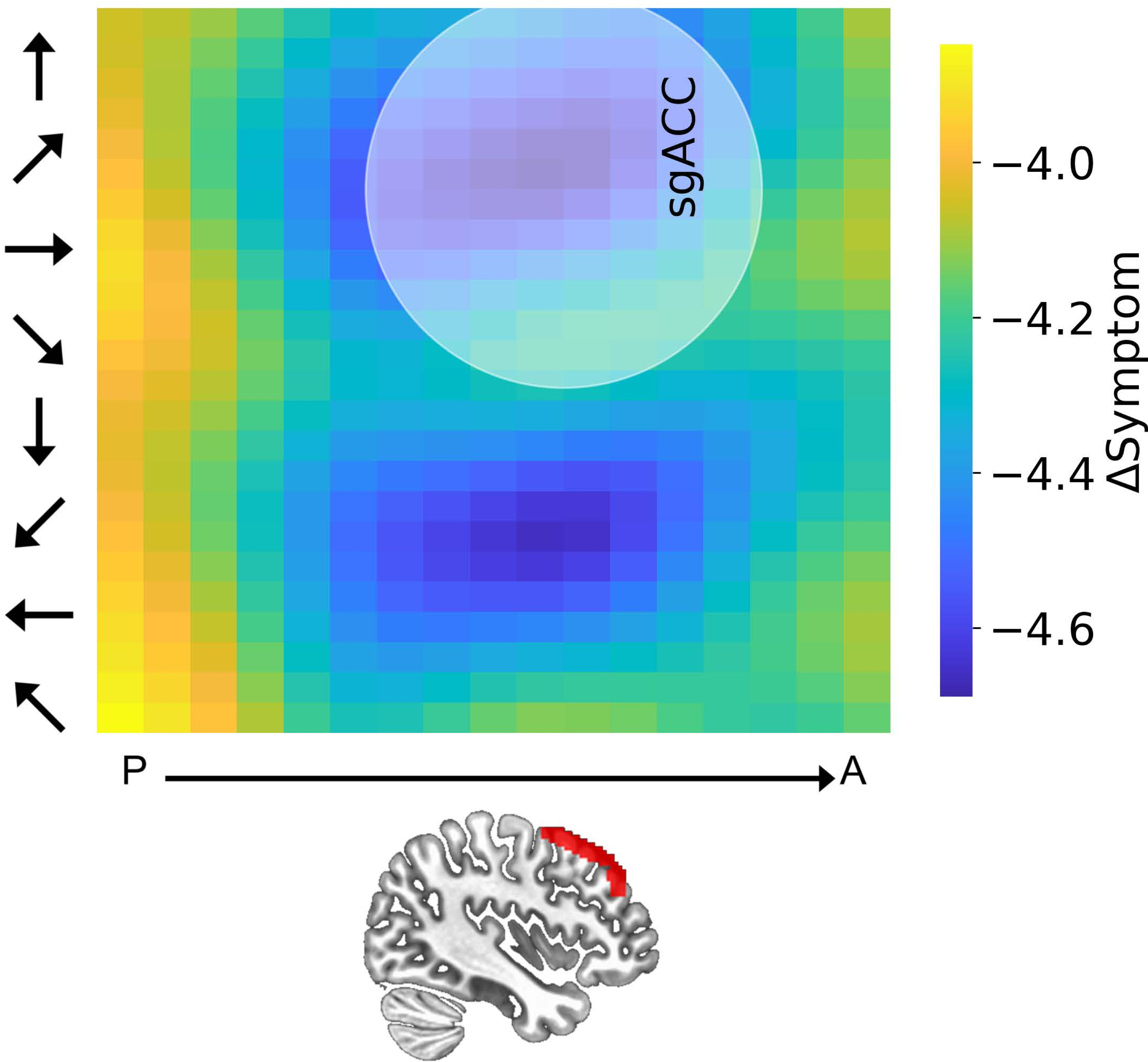

NDARYM648GK3_hamd_left_dlpfc

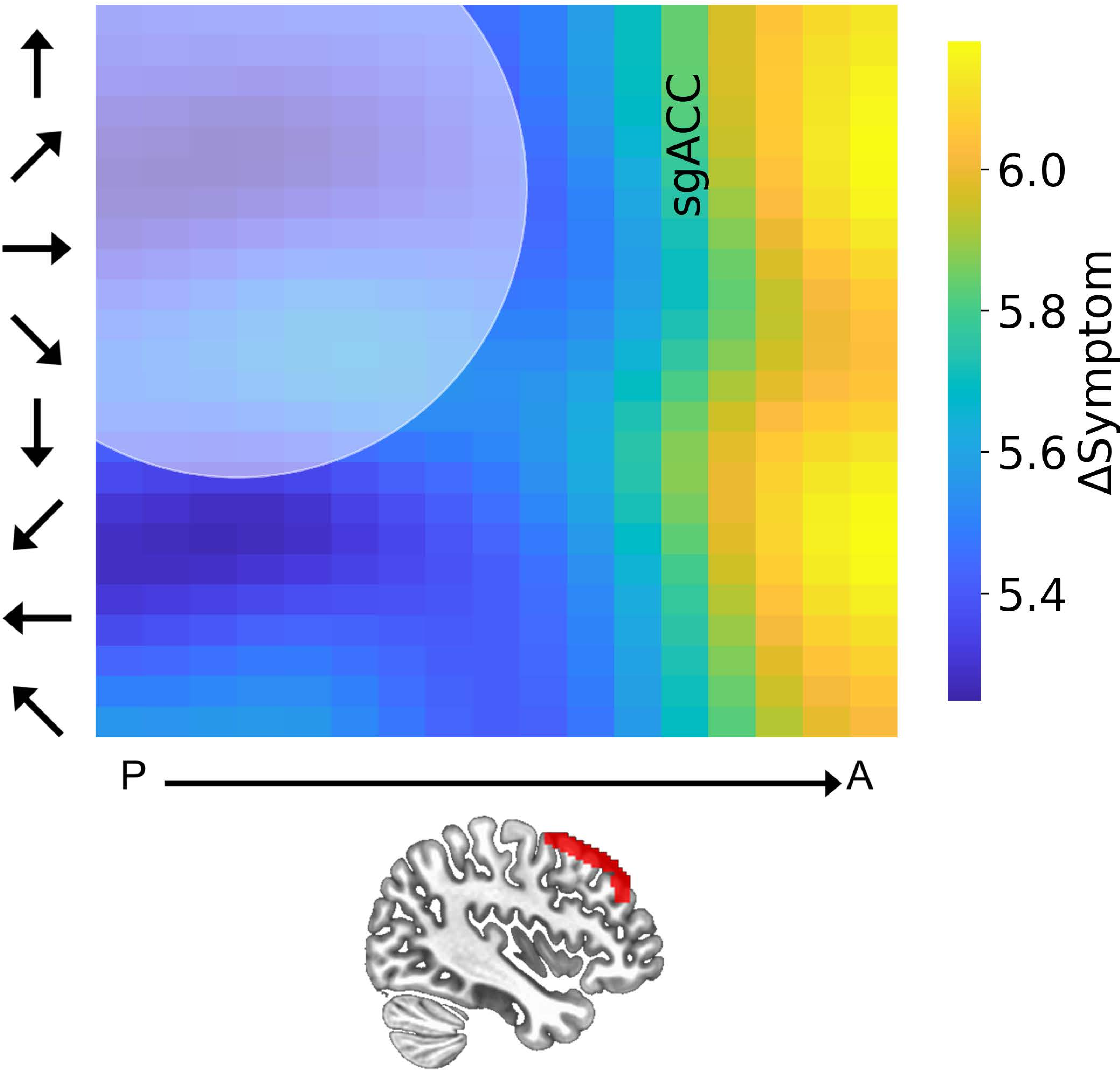

NDARZH200GZU_hamd_left_dlpfc

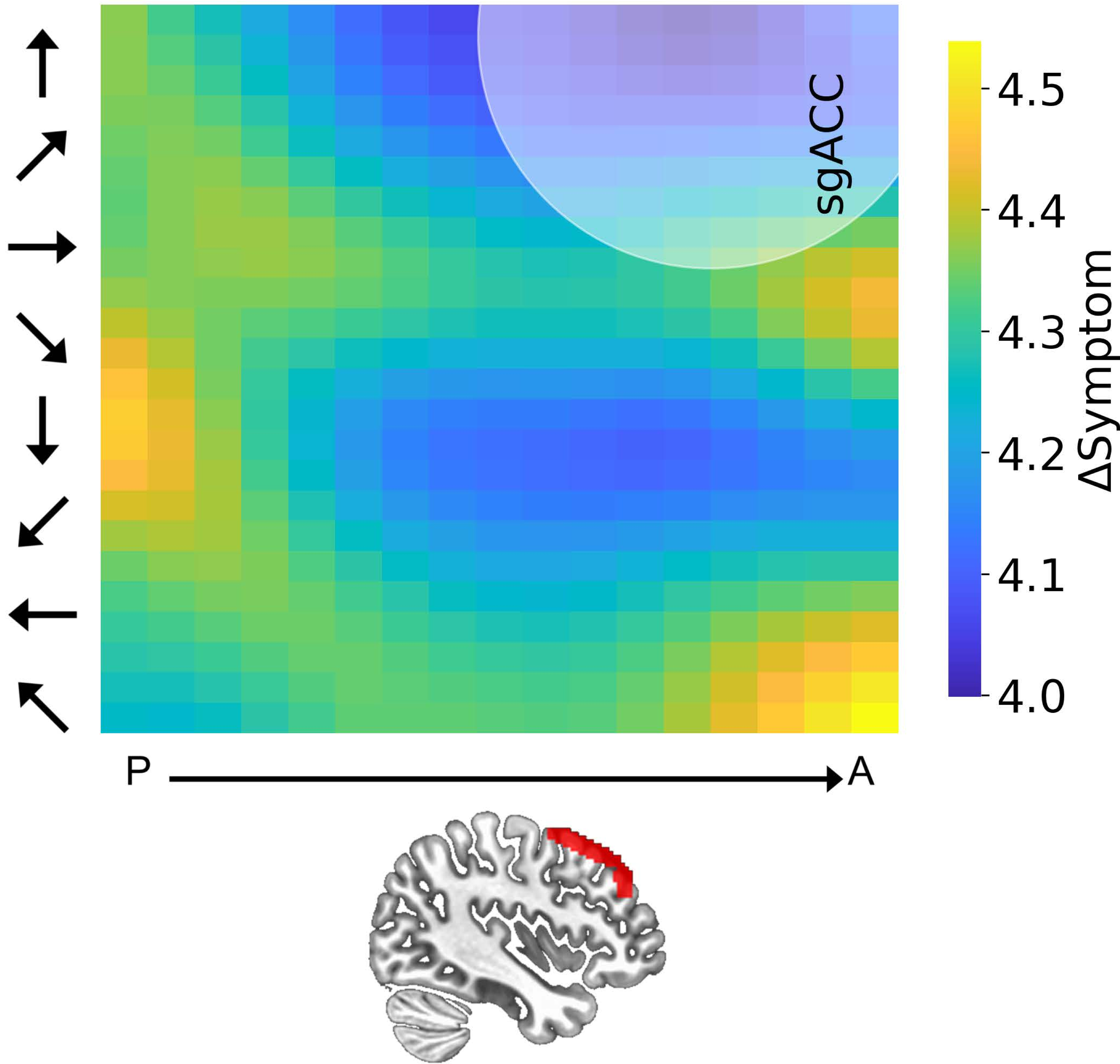

NDARBB471NAU_hamd_right_dlpfc

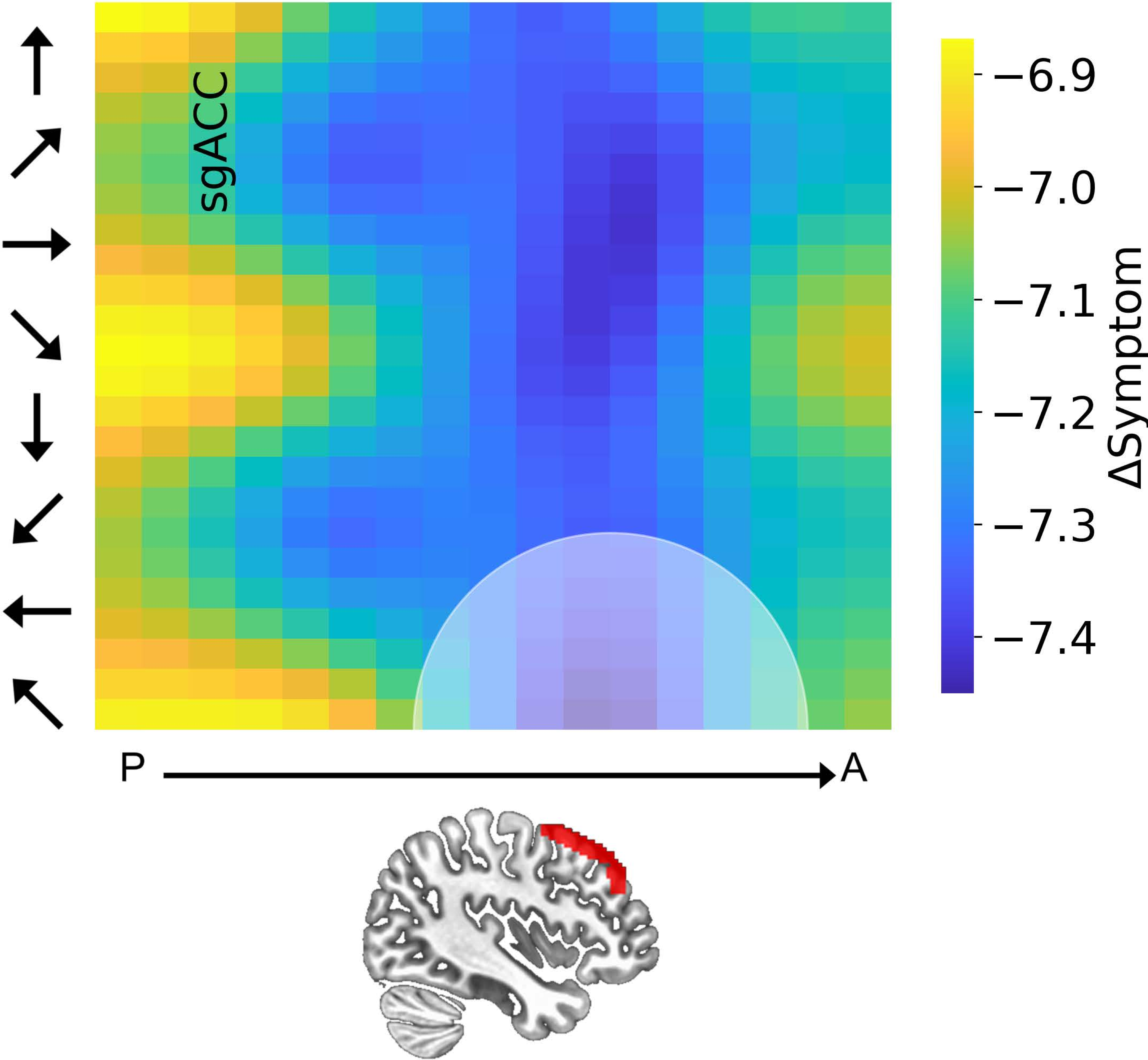

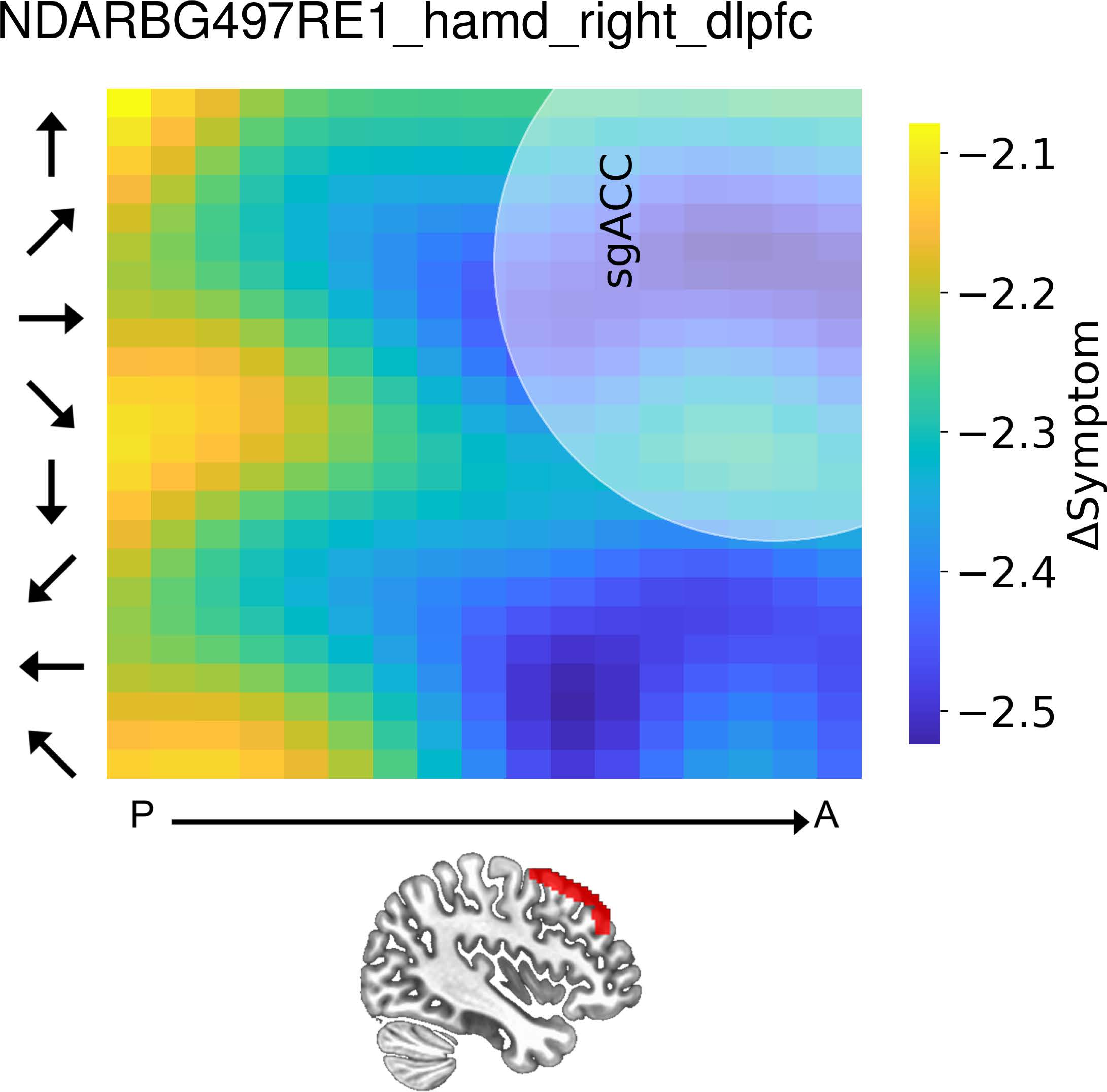

NDARBG497RE1_hamd_right_dlpfc
sgACC
P
A
−2.1
−2.2
−2.3
−2.4
−2.5
ΔSymptom

NDARBK027PDF_hamd_right_dlpfc

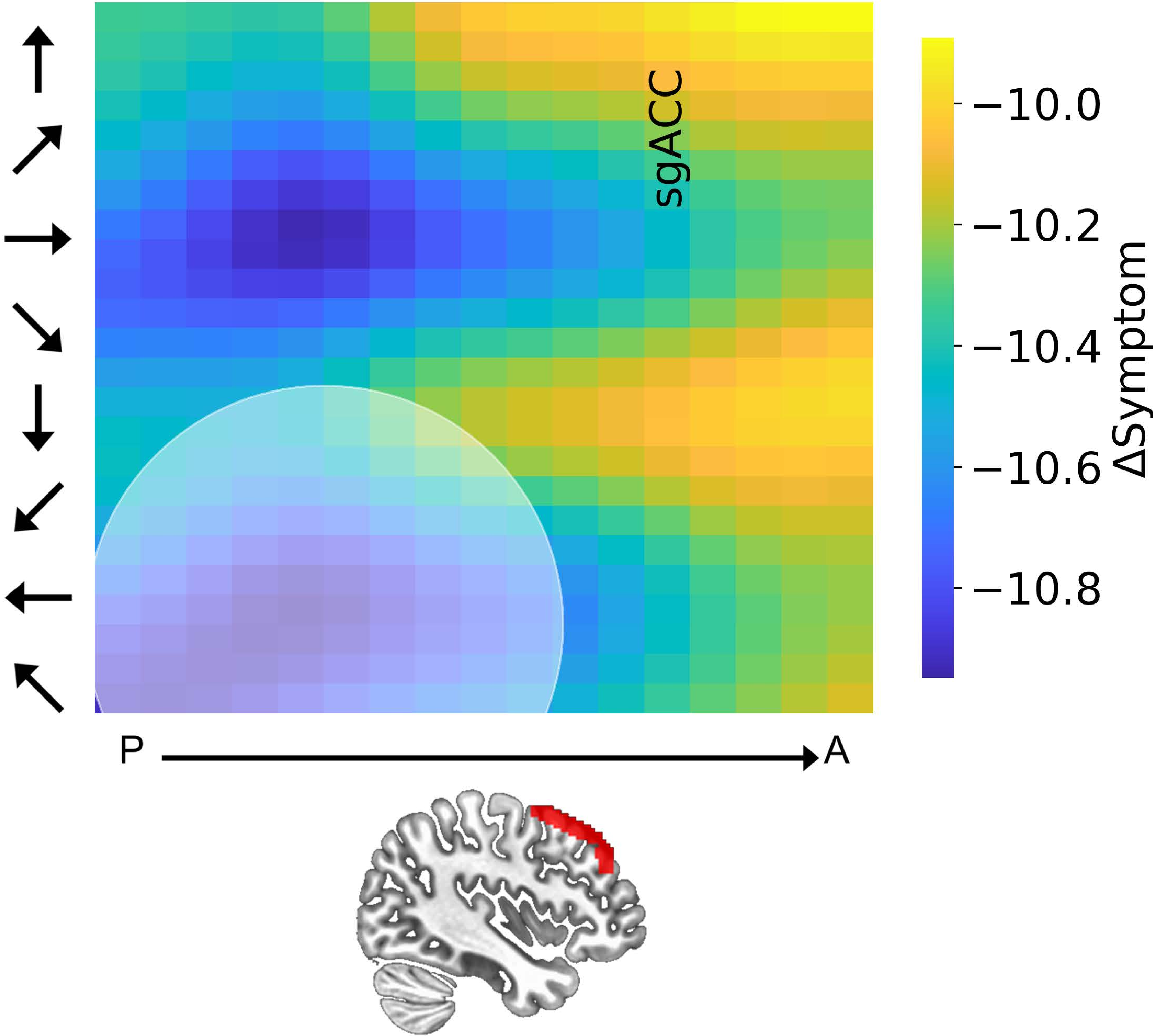

NDARBL814BJK_hamd_right_dlpfc

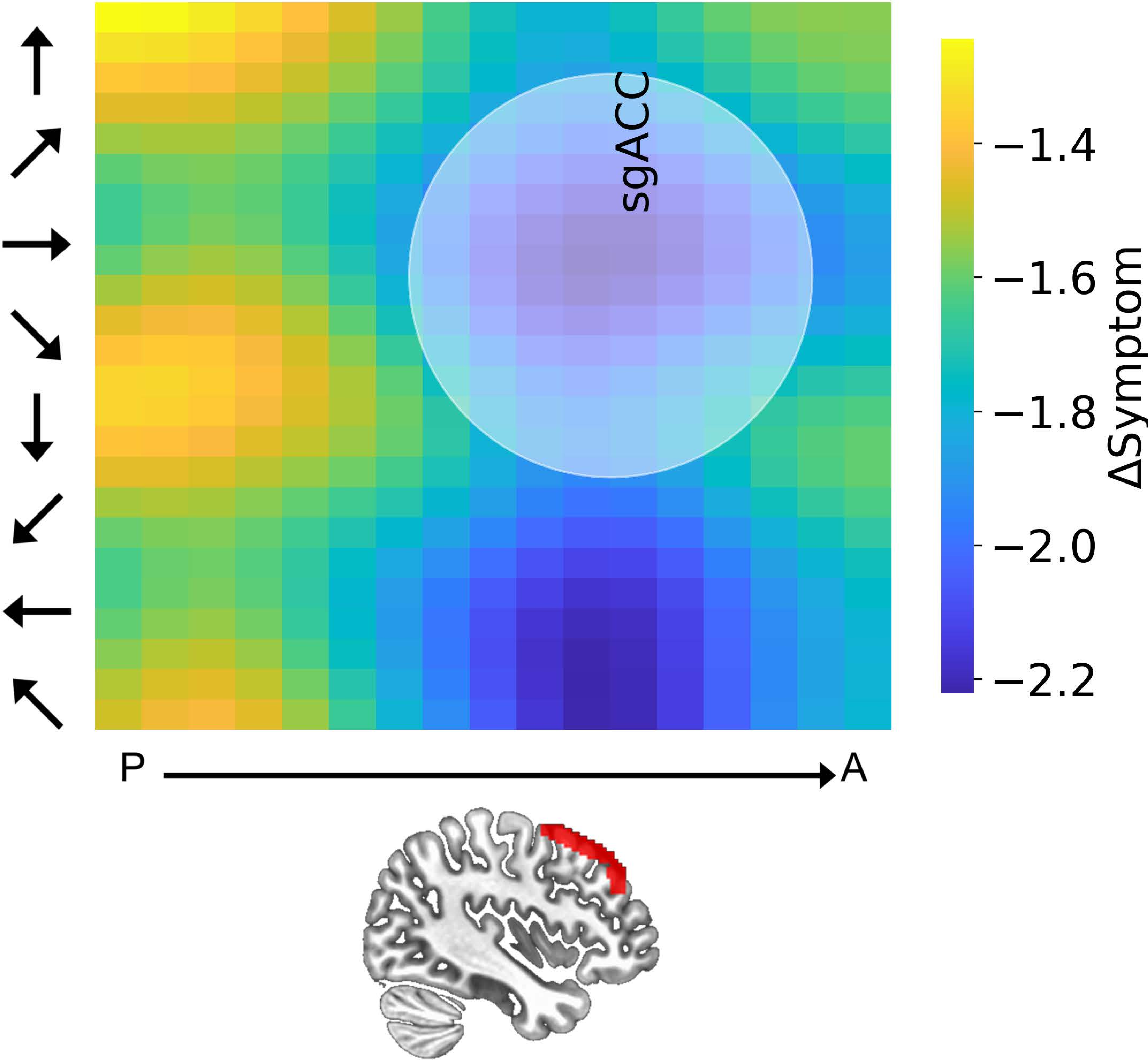

NDARBP353XD2_hamd_right_dlpfc

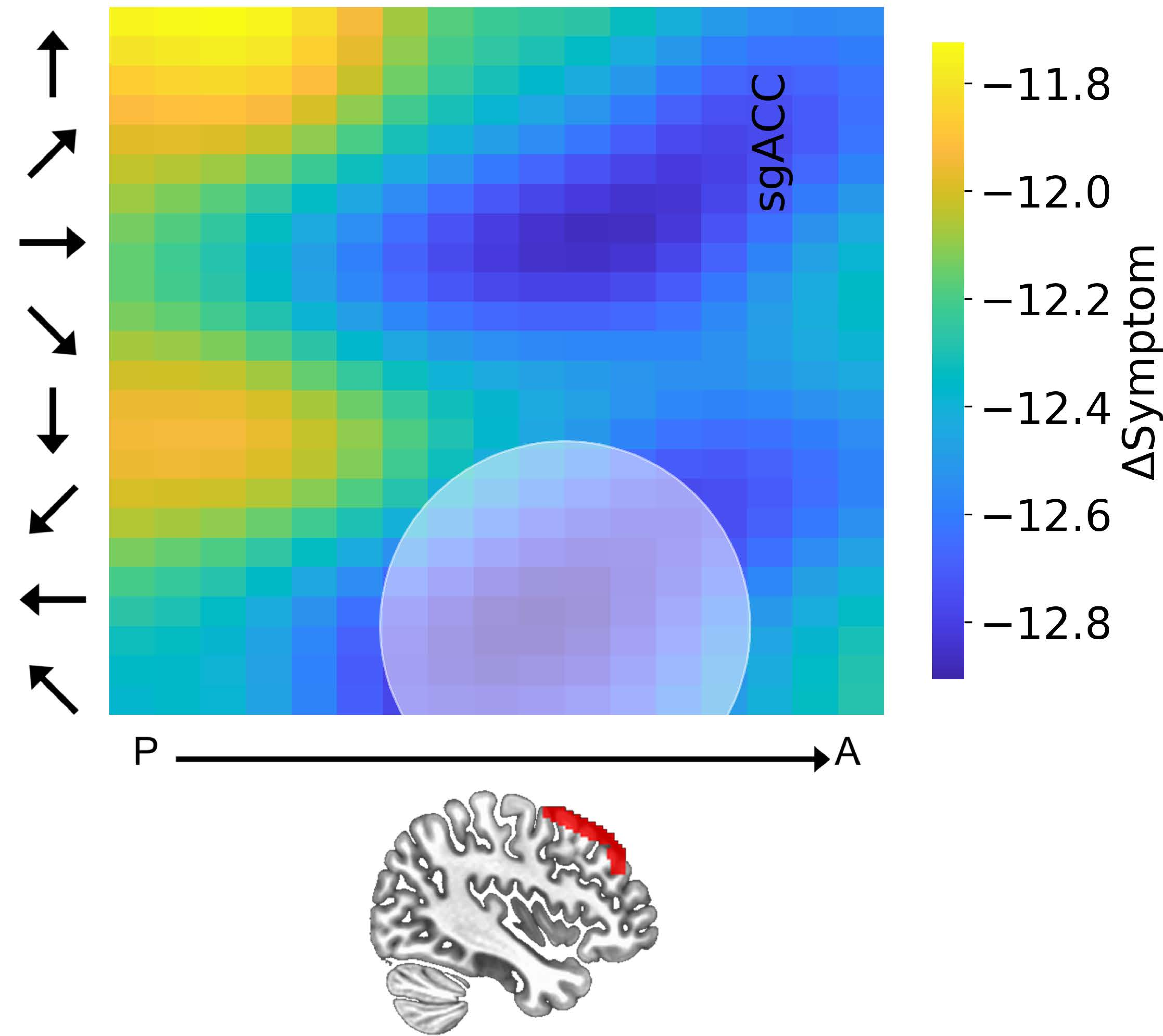

NDARBT583XH6_hamd_right_dlpfc

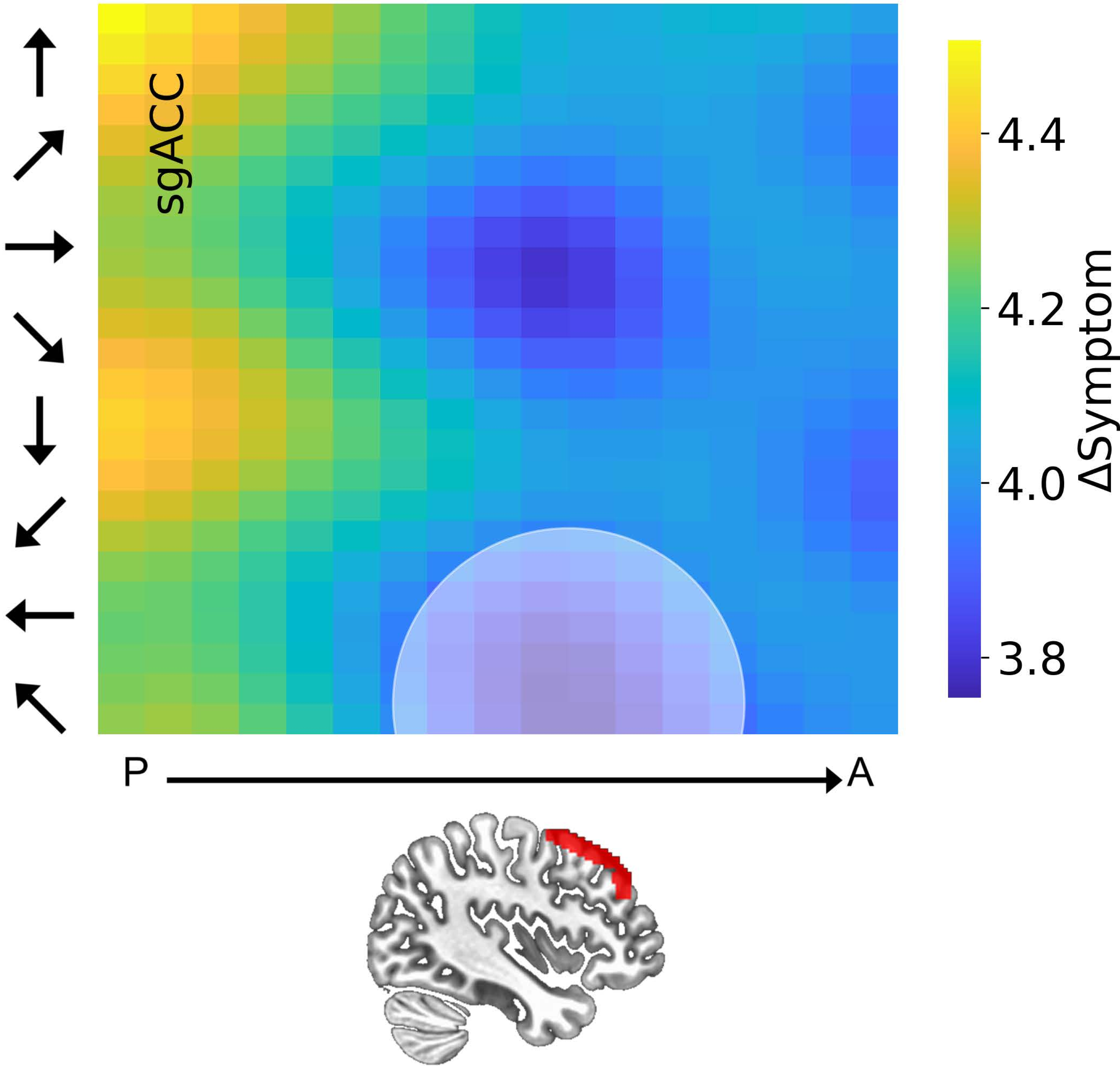

NDARCG071YN8_hamd_right_dlpfc

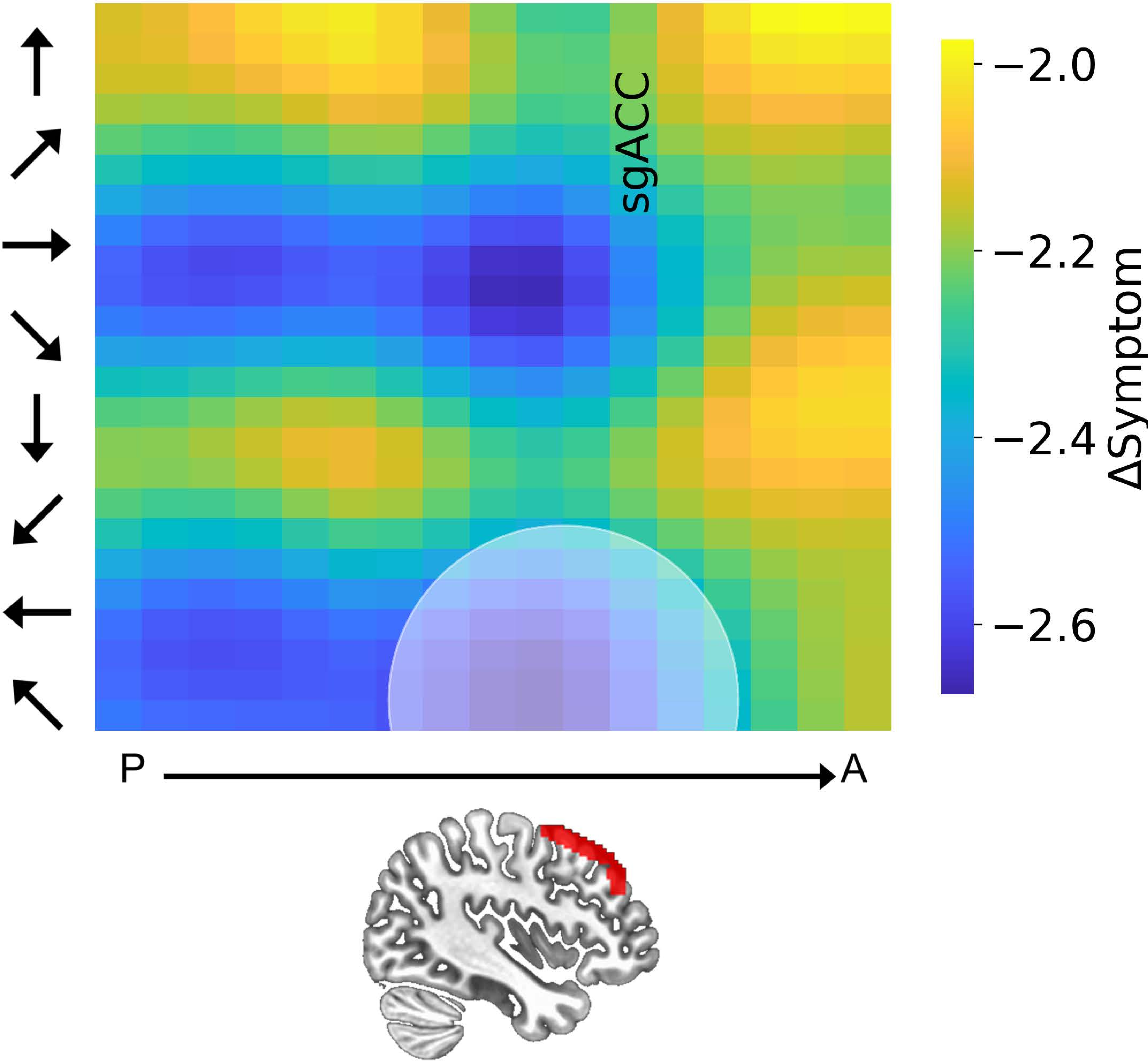

NDARCG271VGC_hamd_right_dlpfc

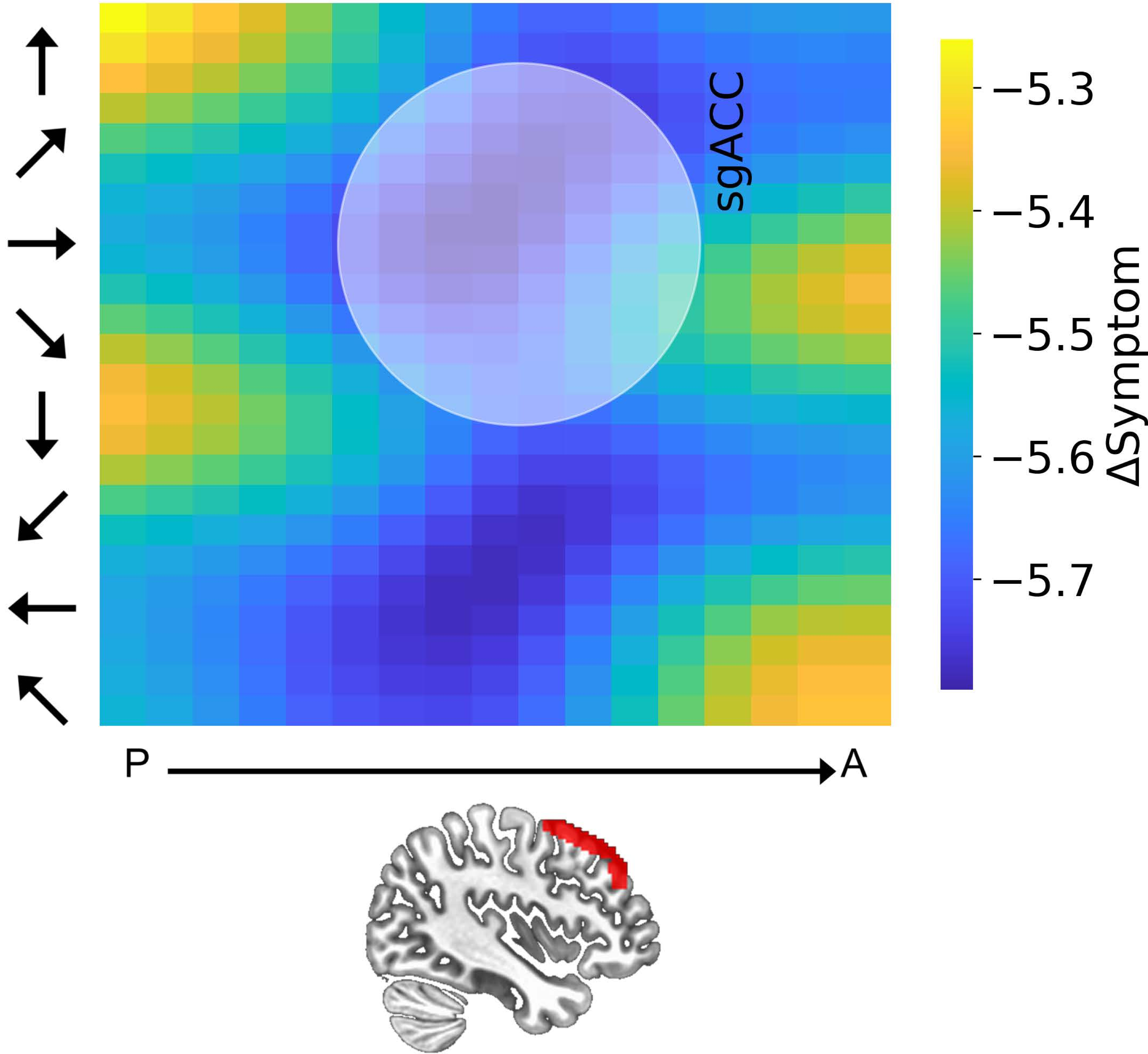

NDARCM769WZL_hamd_right_dlpfc

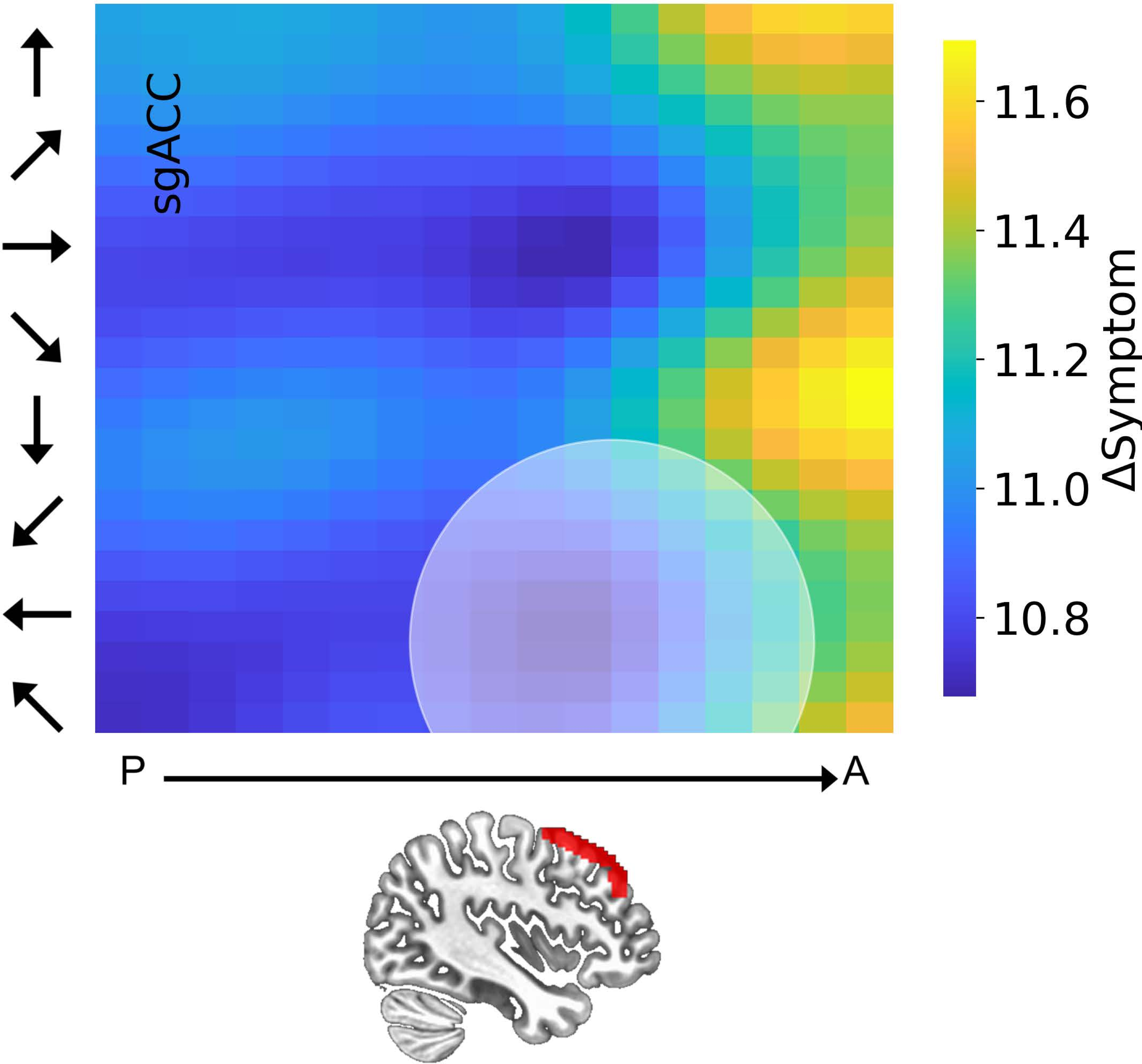

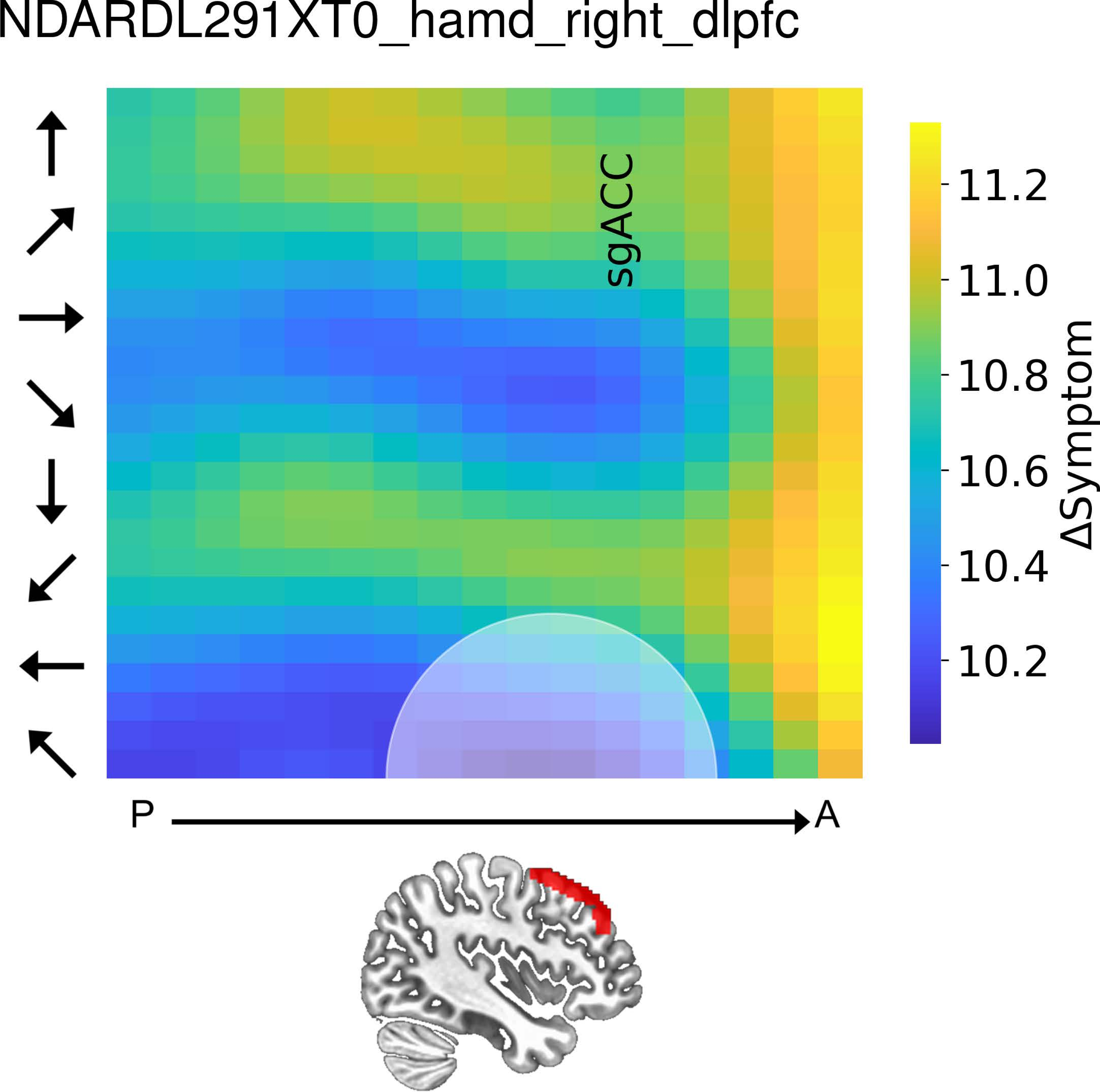
NDARDL291XT0_hamd_right_dlpfc
sgACC
P
A
11.2
11.0
10.8
10.6
10.4
10.2
ΔSymptom

NDARDL667VMW_hamd_right_dlpfc

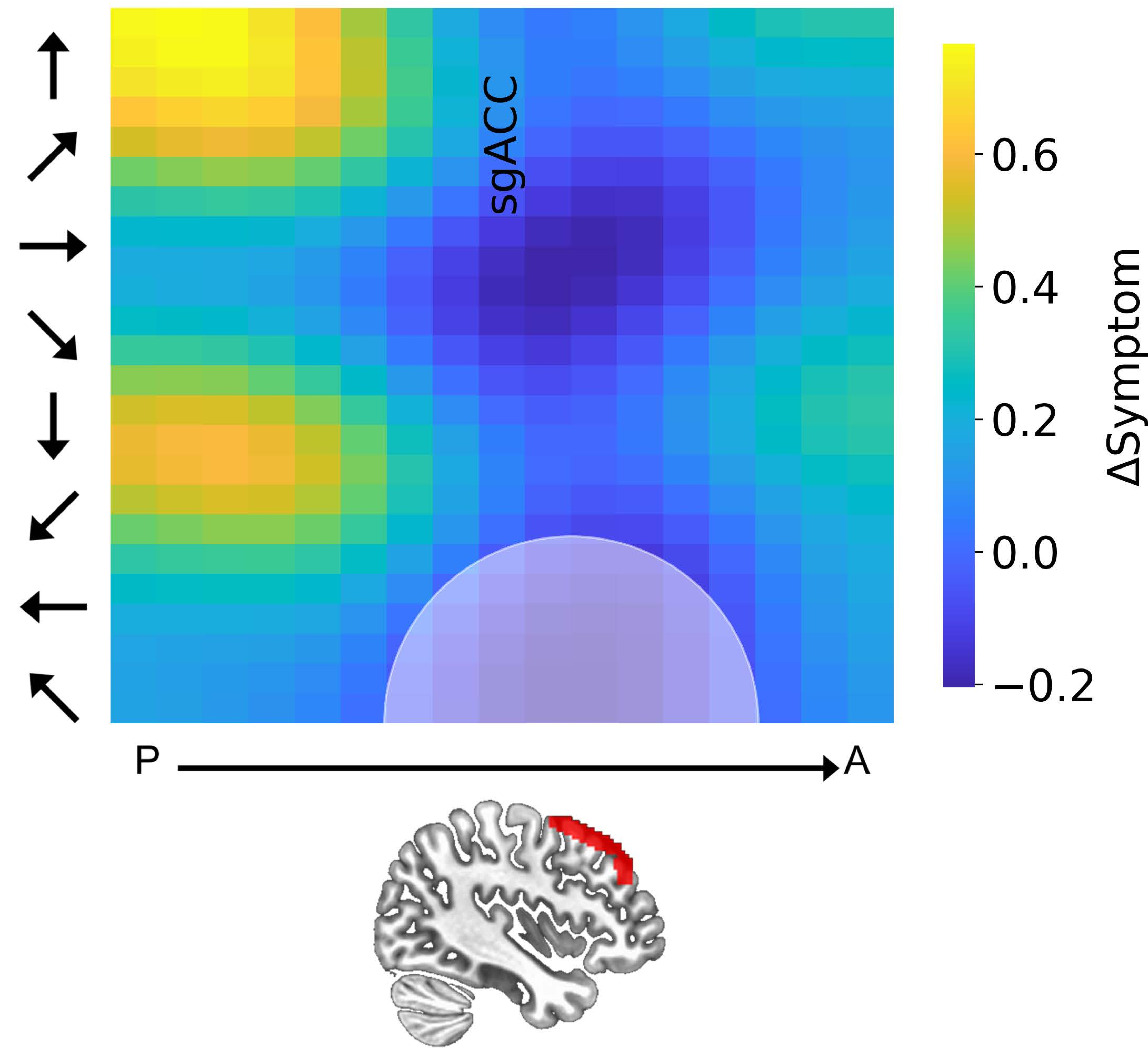

NDARDR017YX2_hamd_right_dlpfc

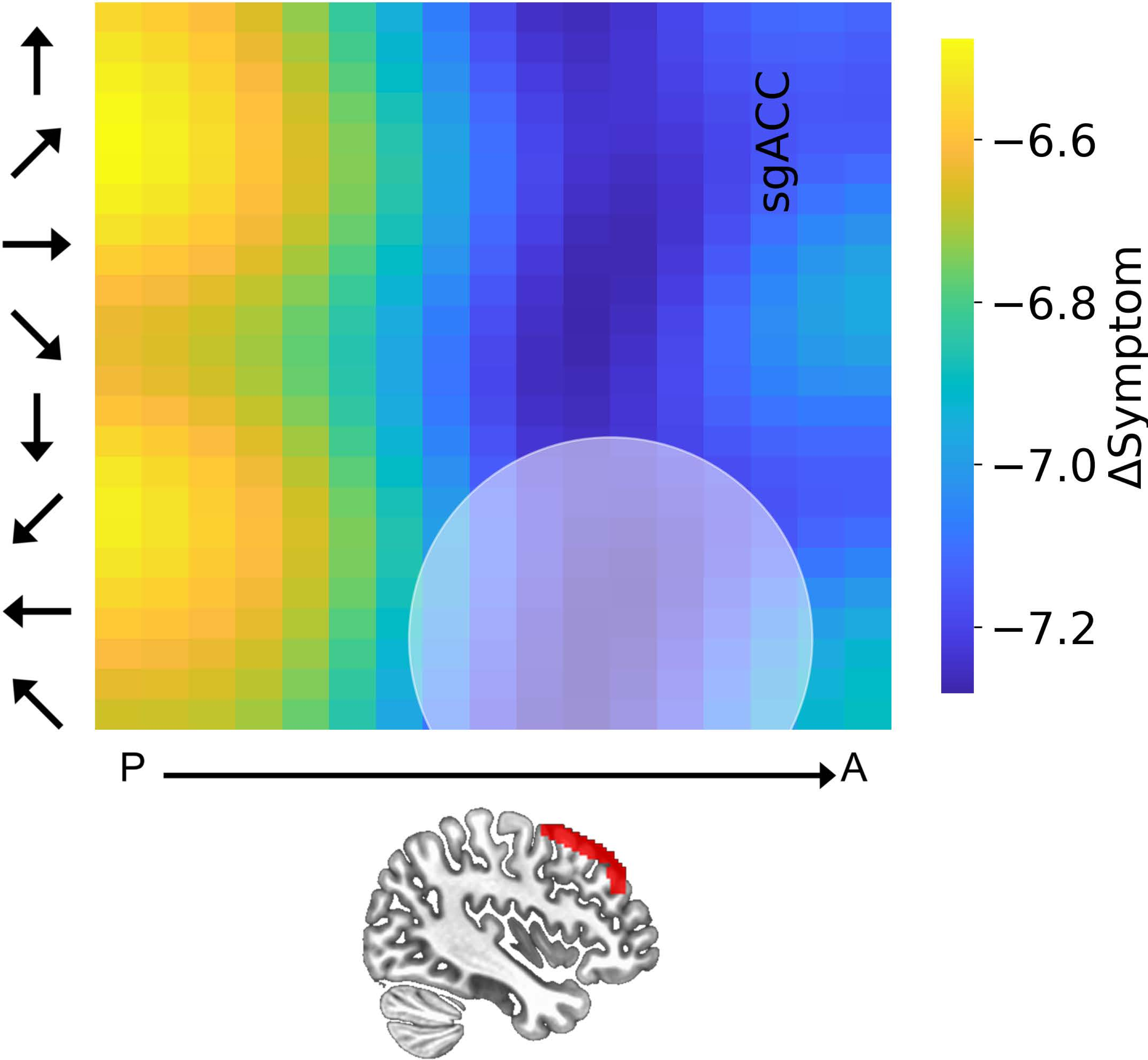

NDARDU203PAW_hamd_right_dlpfc

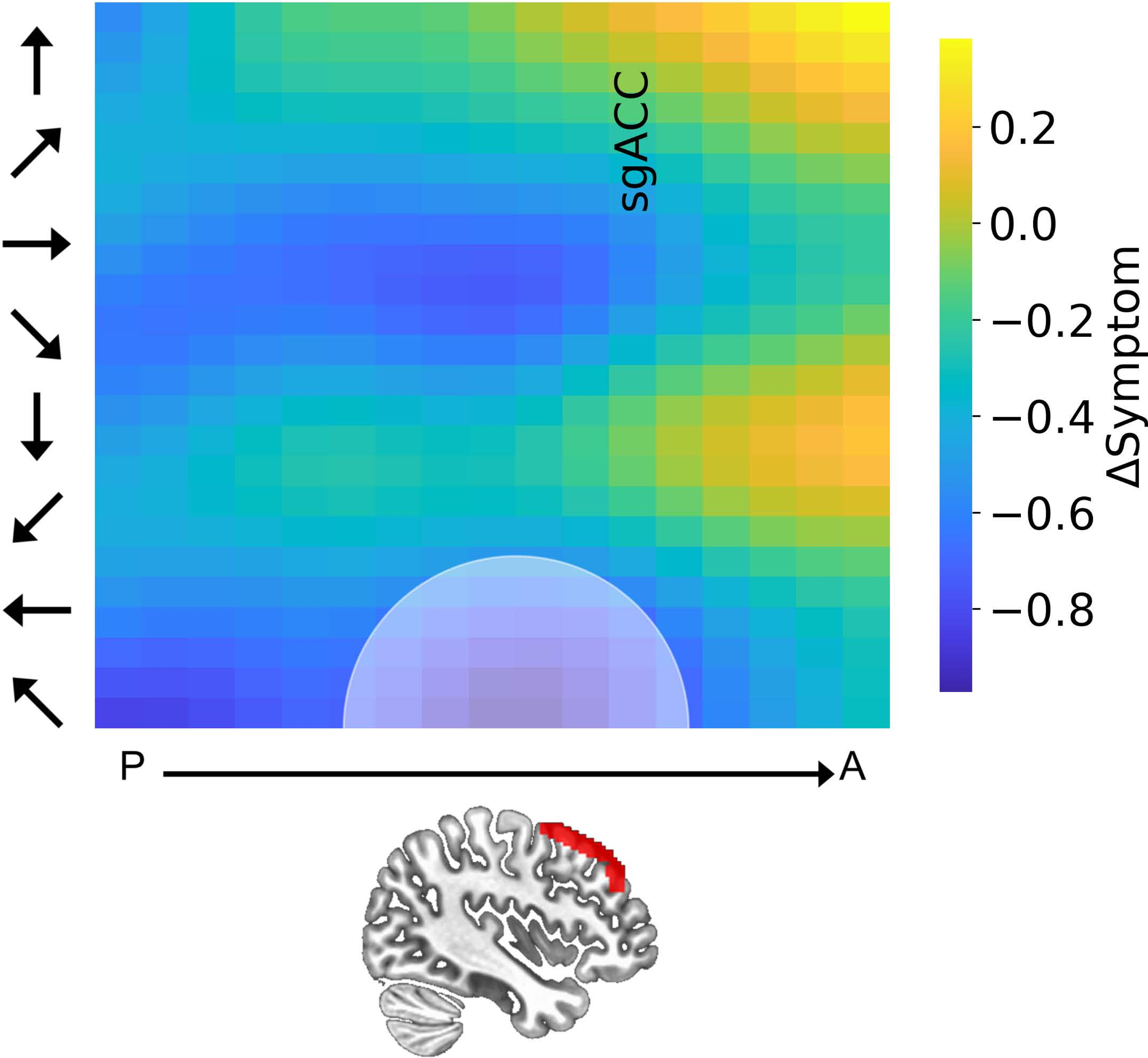

NDARDU282NKX_hamd_right_dlpfc

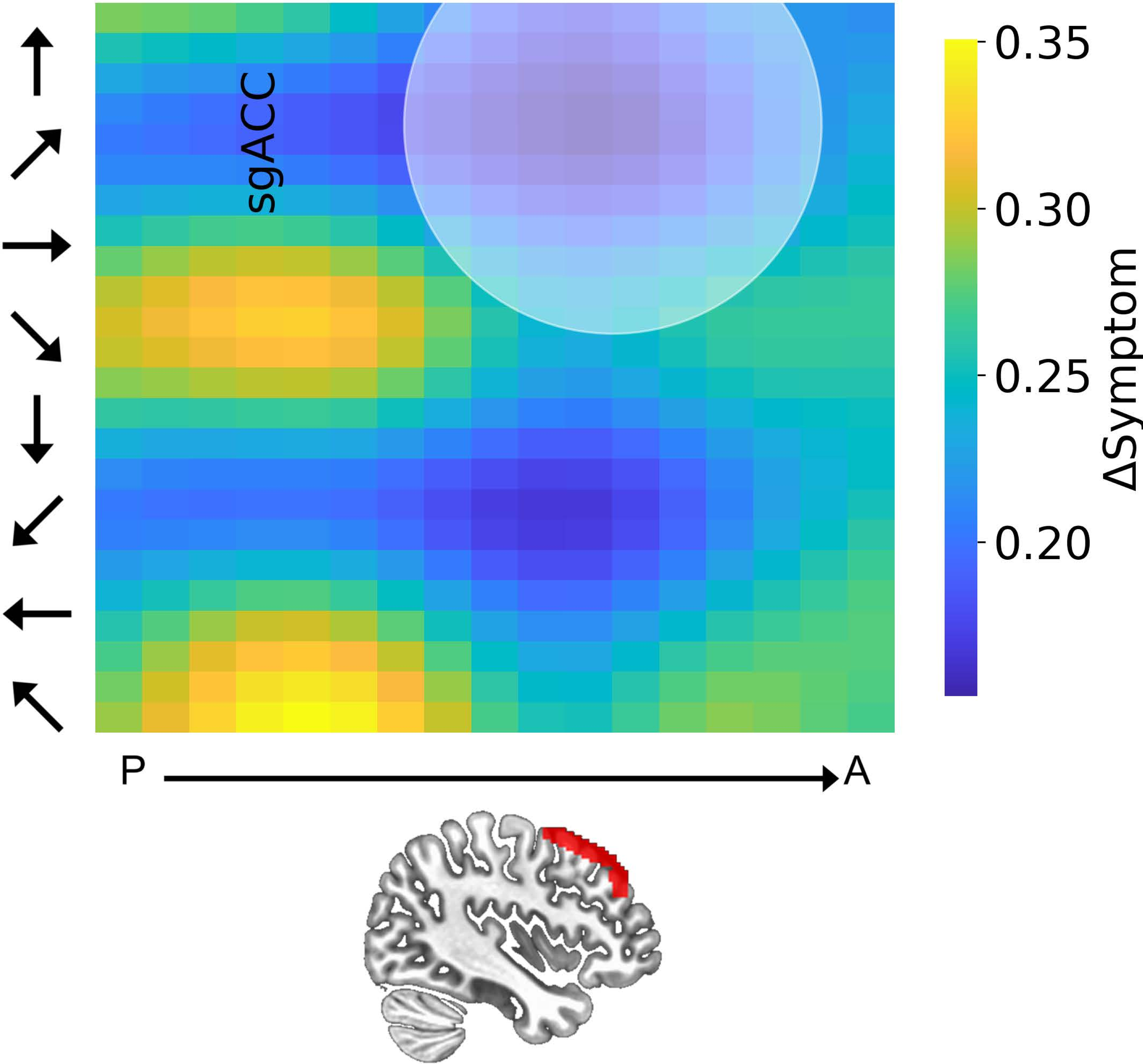

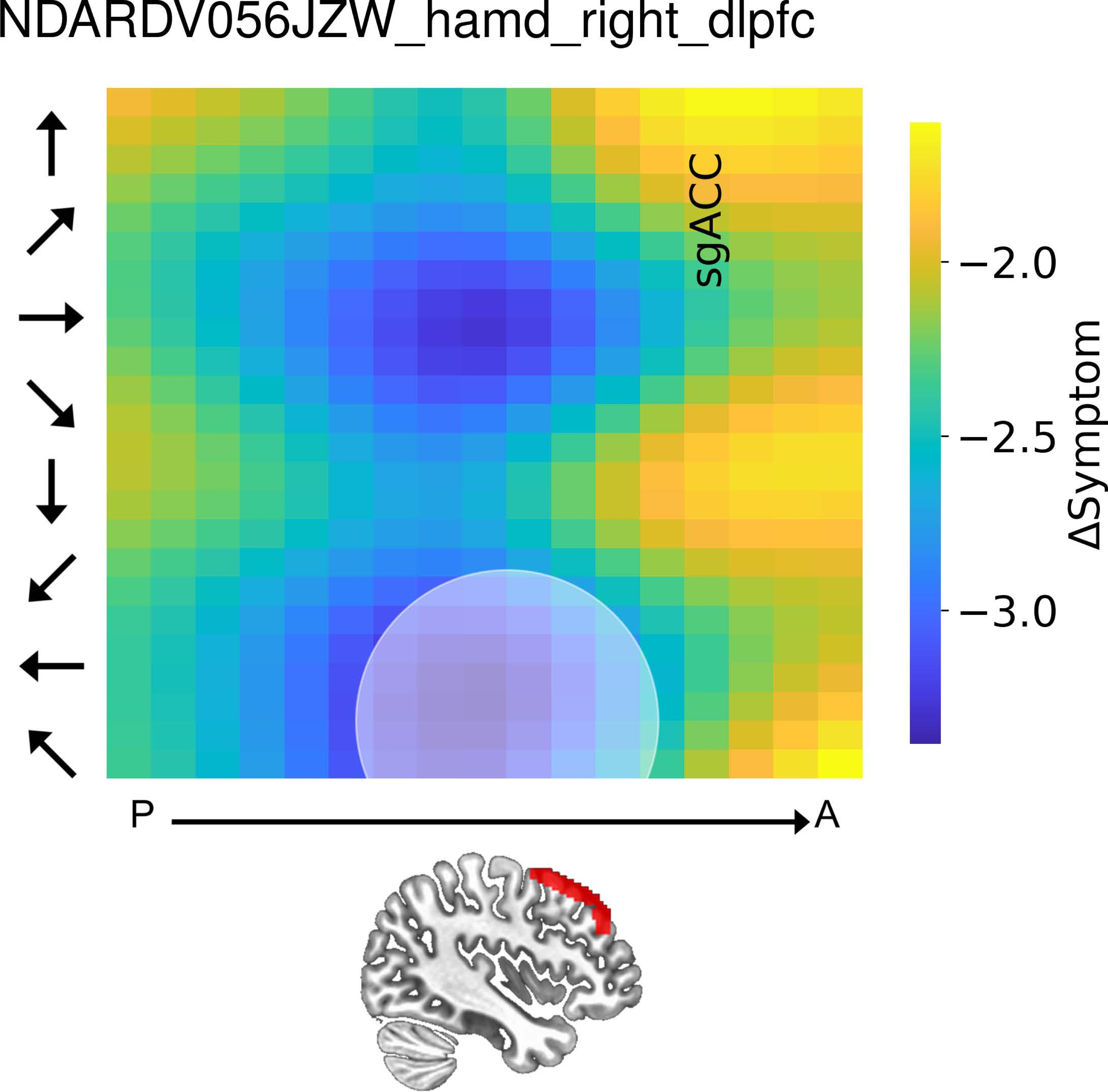
NDARDV056JZW_hamd_right_dlpfc
sgACC
−2.0
−2.5
−3.0
ΔSymptom
P
A

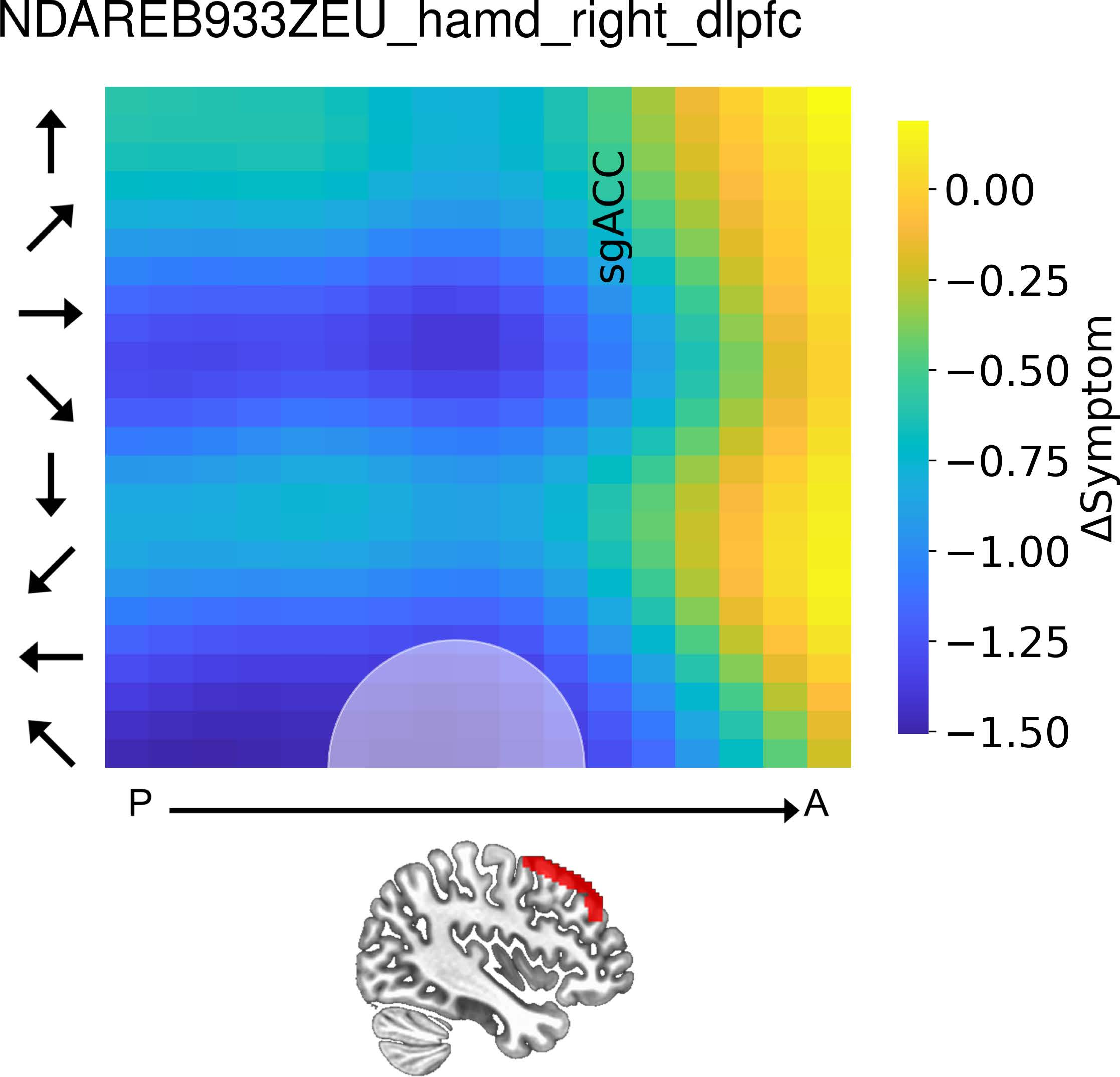
NDAREB933ZEU_hamd_right_dlpfc
sgACC
P
A
0.00
−0.25
−0.50
−0.75
−1.00
−1.25
−1.50
ΔSymptom

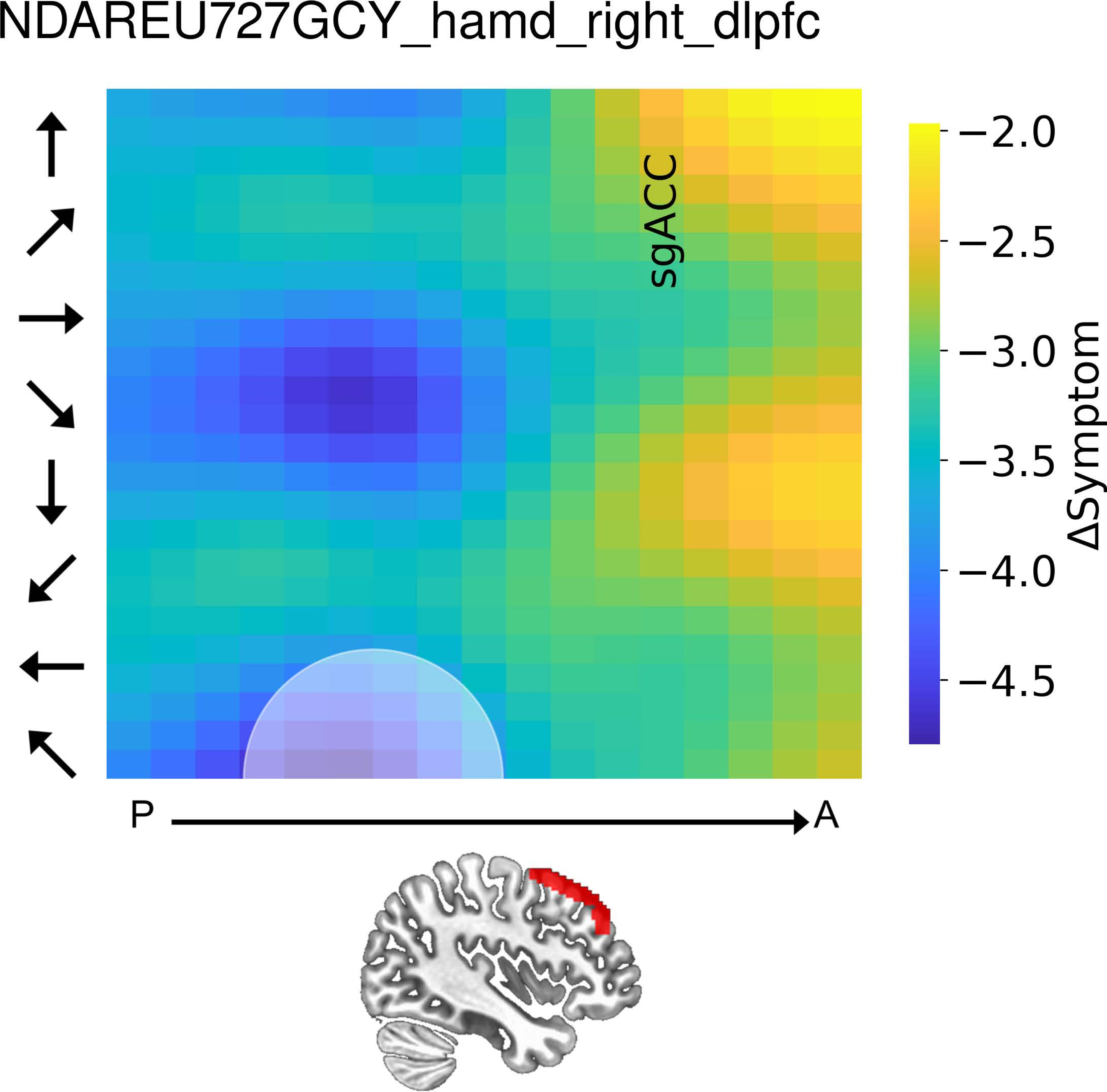
NDAREU727GCY_hamd_right_dlpfc
sgACC
−2.0
−2.5
−3.0
−3.5
−4.0
−4.5
ΔSymptom
P
A

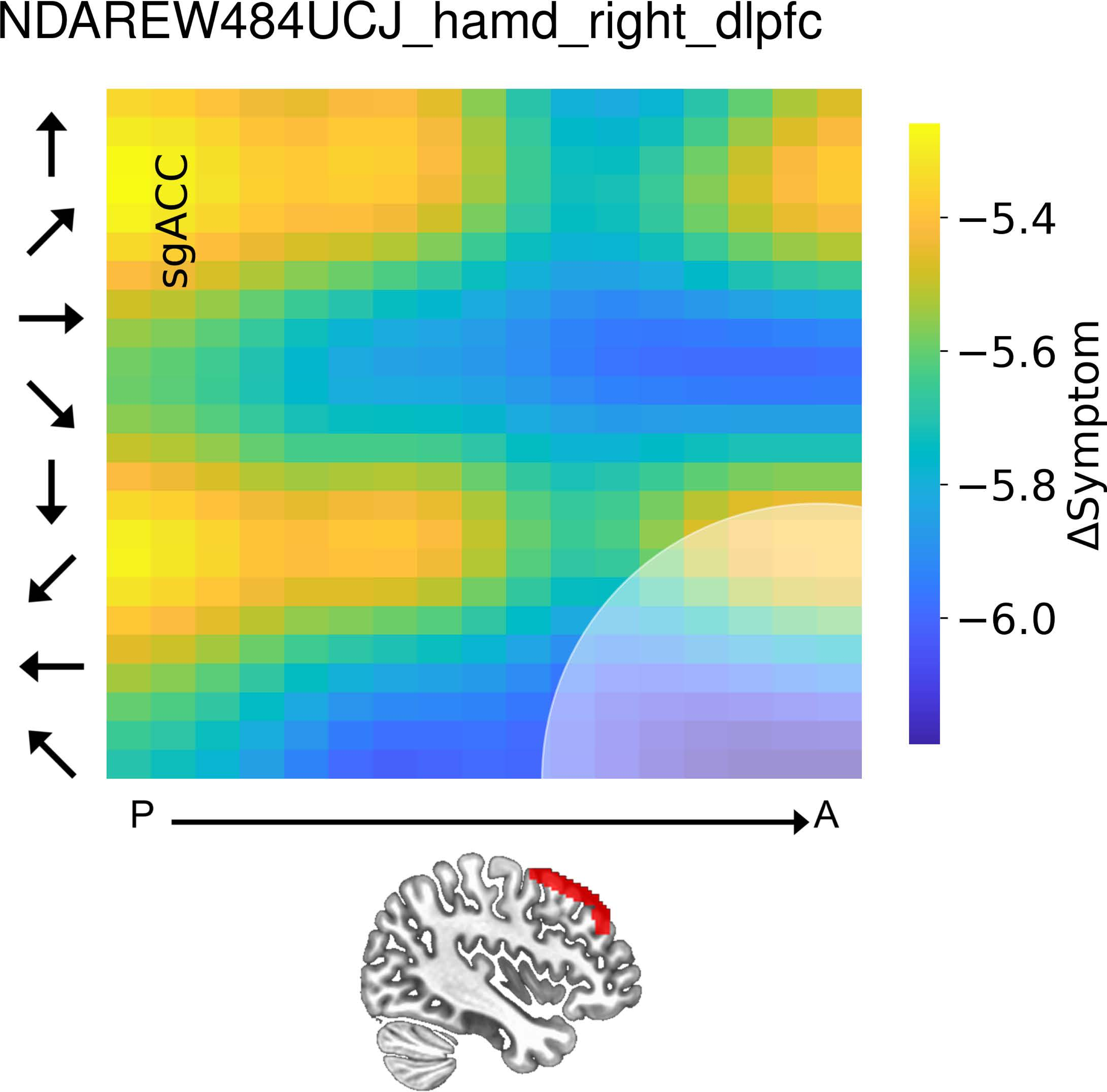

NDAREW484UCJ_hamd_right_dlpfc
sgACC
P
A
ΔSymptom
−5.4
−5.6
−5.8
−6.0

NDAREZ915JK5_hamd_right_dlpfc

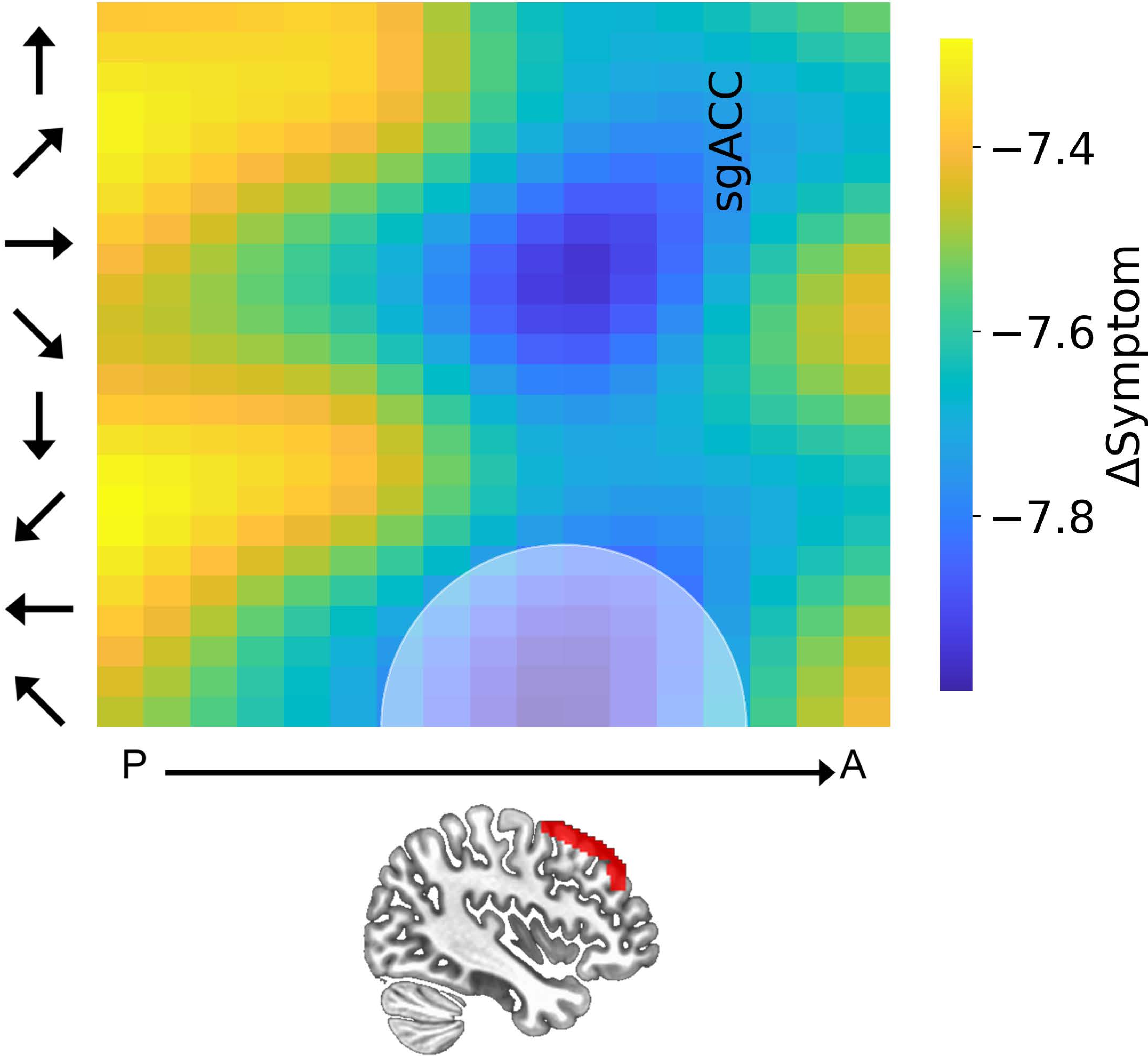

NDARFH525MFZ_hamd_right_dlpfc

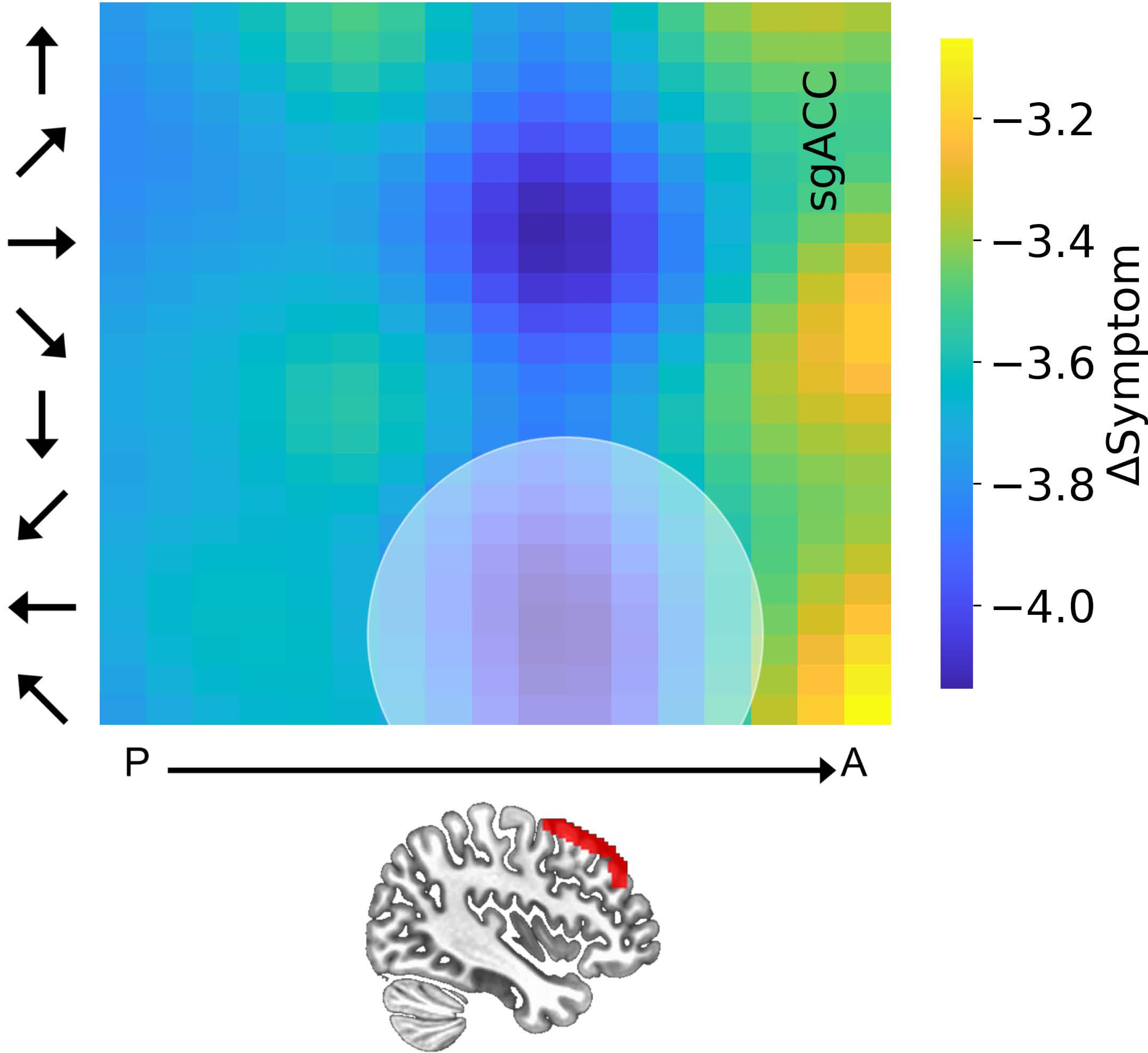

NDARFR746ENQ_hamd_right_dlpfc

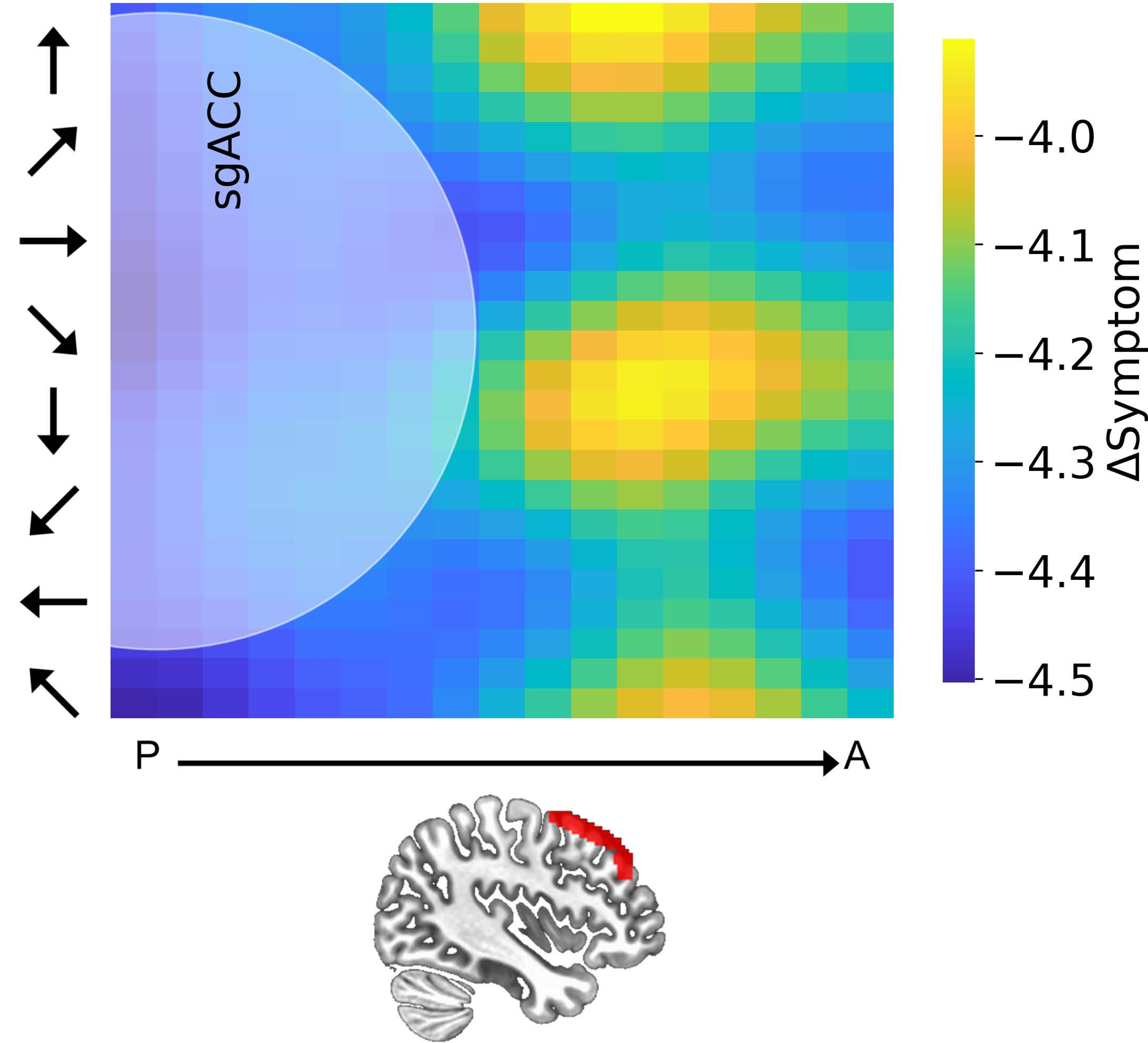

NDARFW096LMD_hamd_right_dlpfc

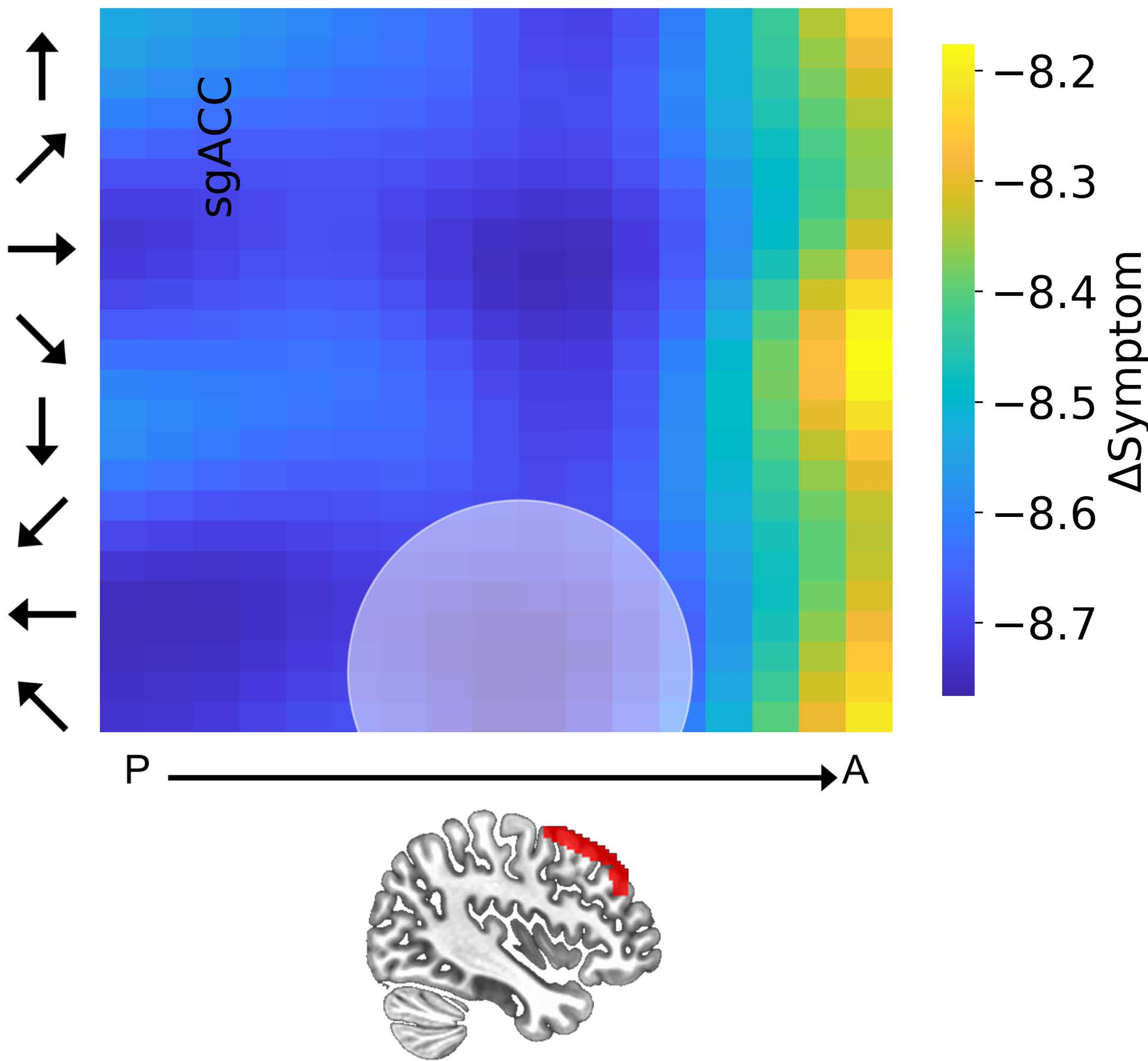

NDARGH895WJJ_hamd_right_dlpfc

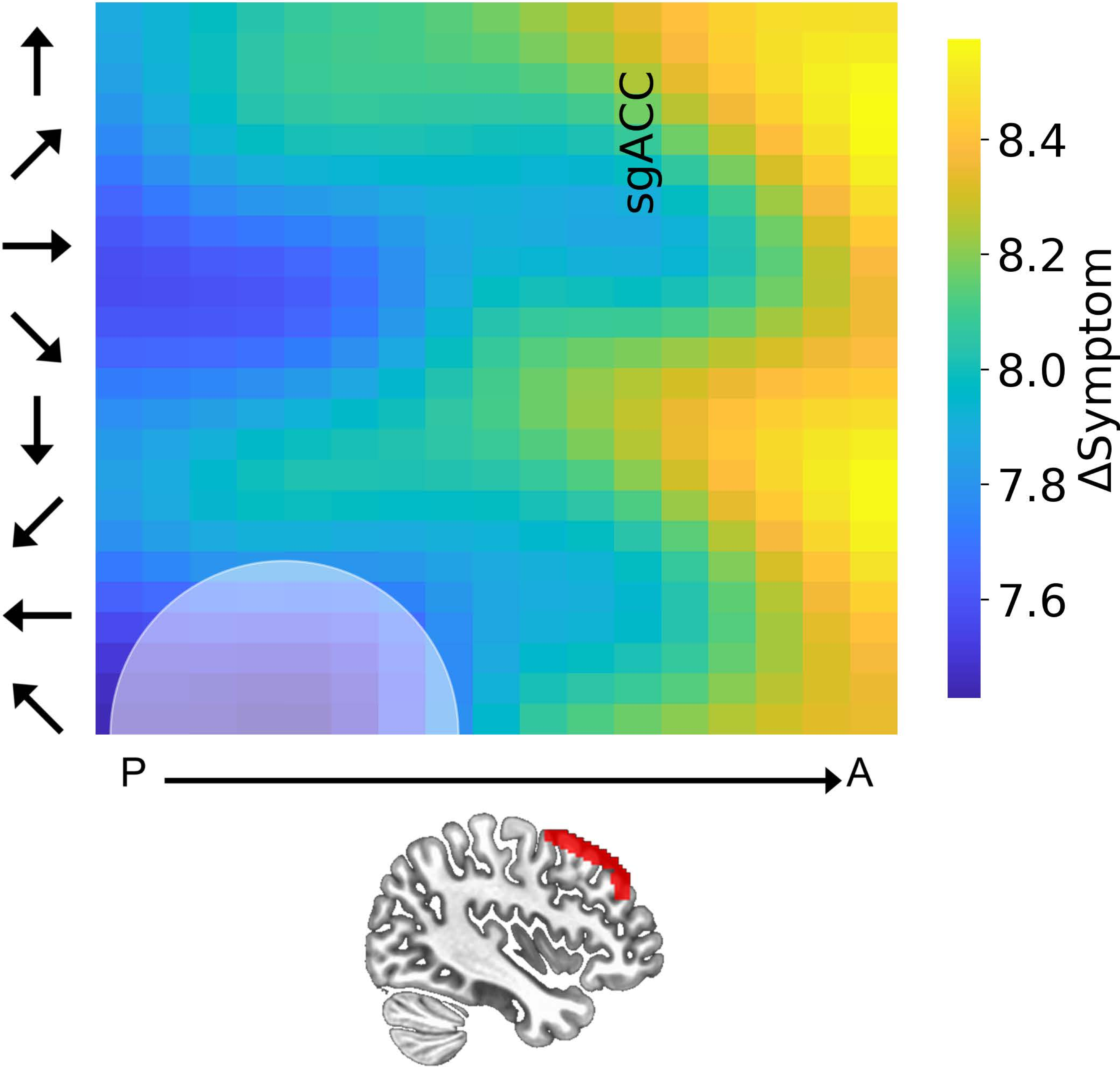

NDARGR637FTT_hamd_right_dlpfc

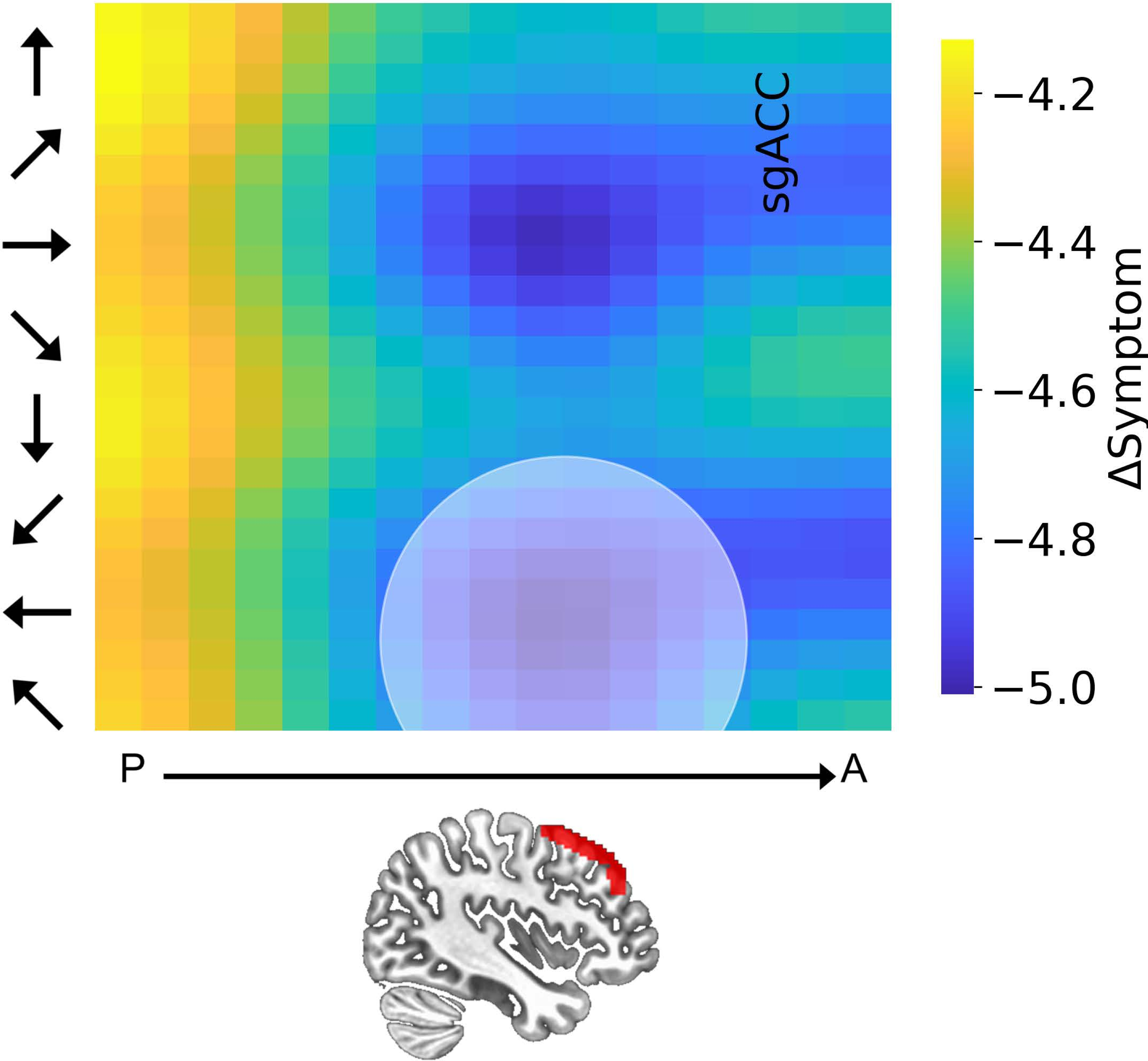

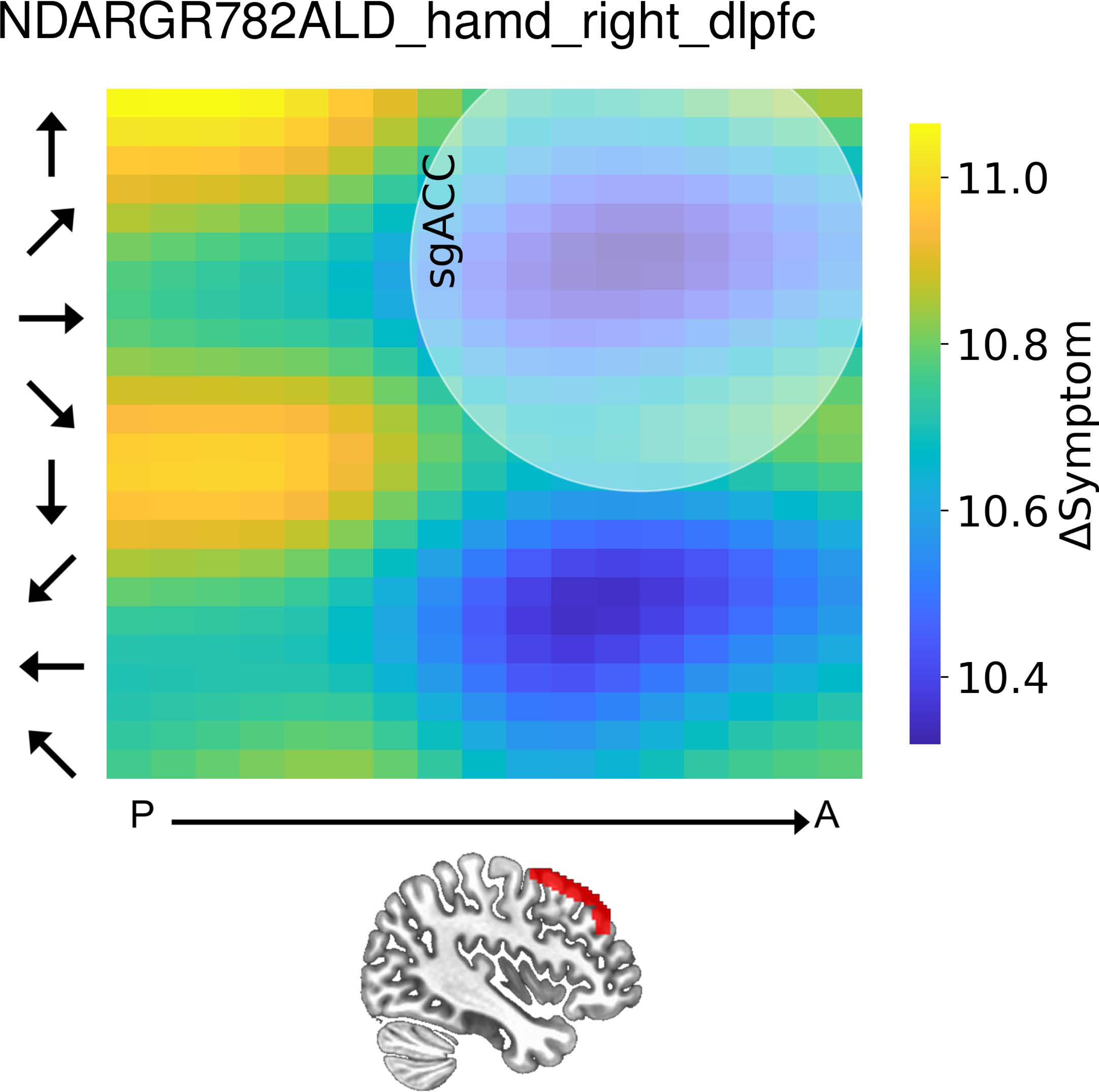

NDARGR782ALD_hamd_right_dlpfc
sgACC
11.0
10.8
10.6
10.4
ΔSymptom
P
A

NDARHA109LH7_hamd_right_dlpfc

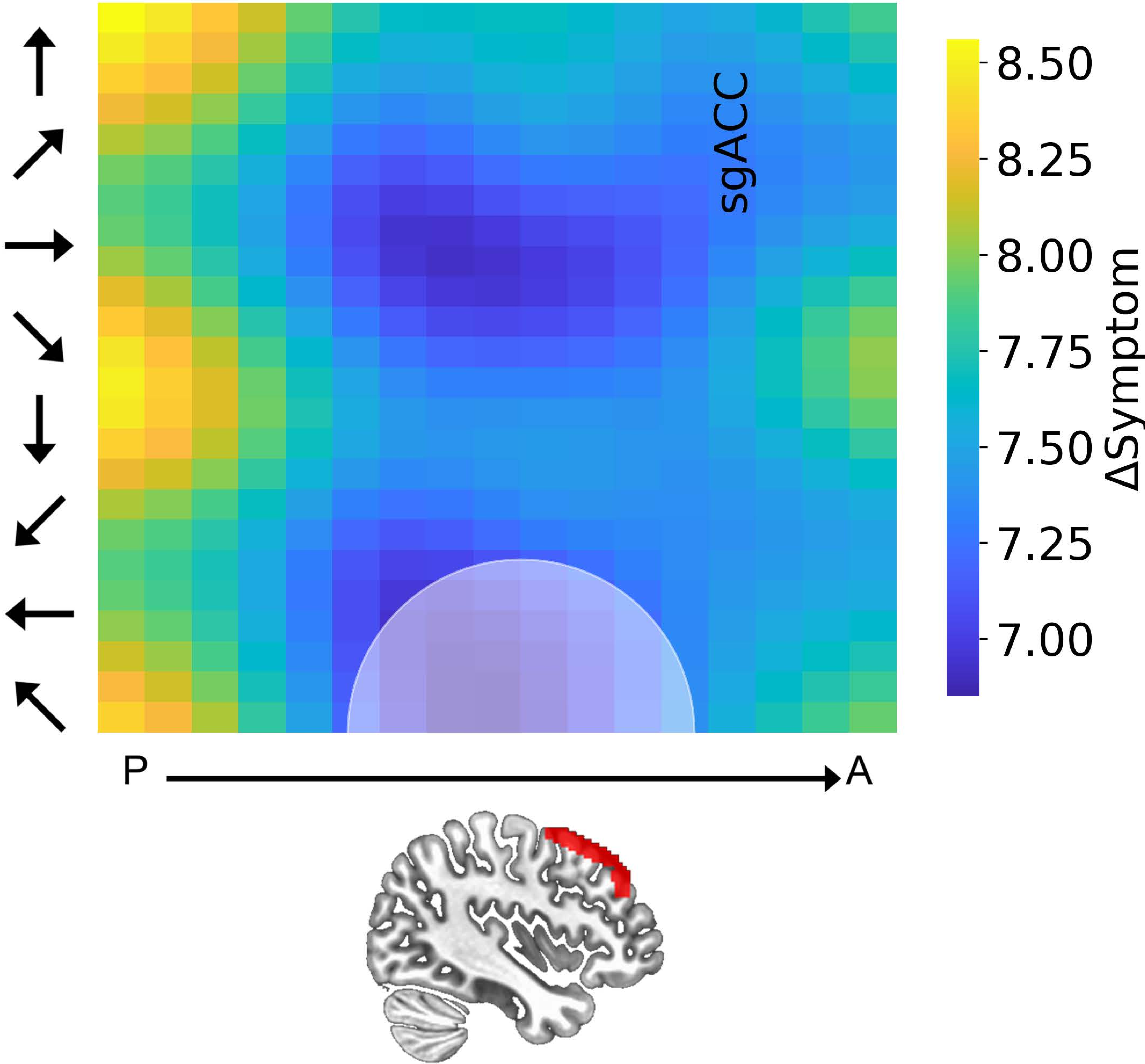

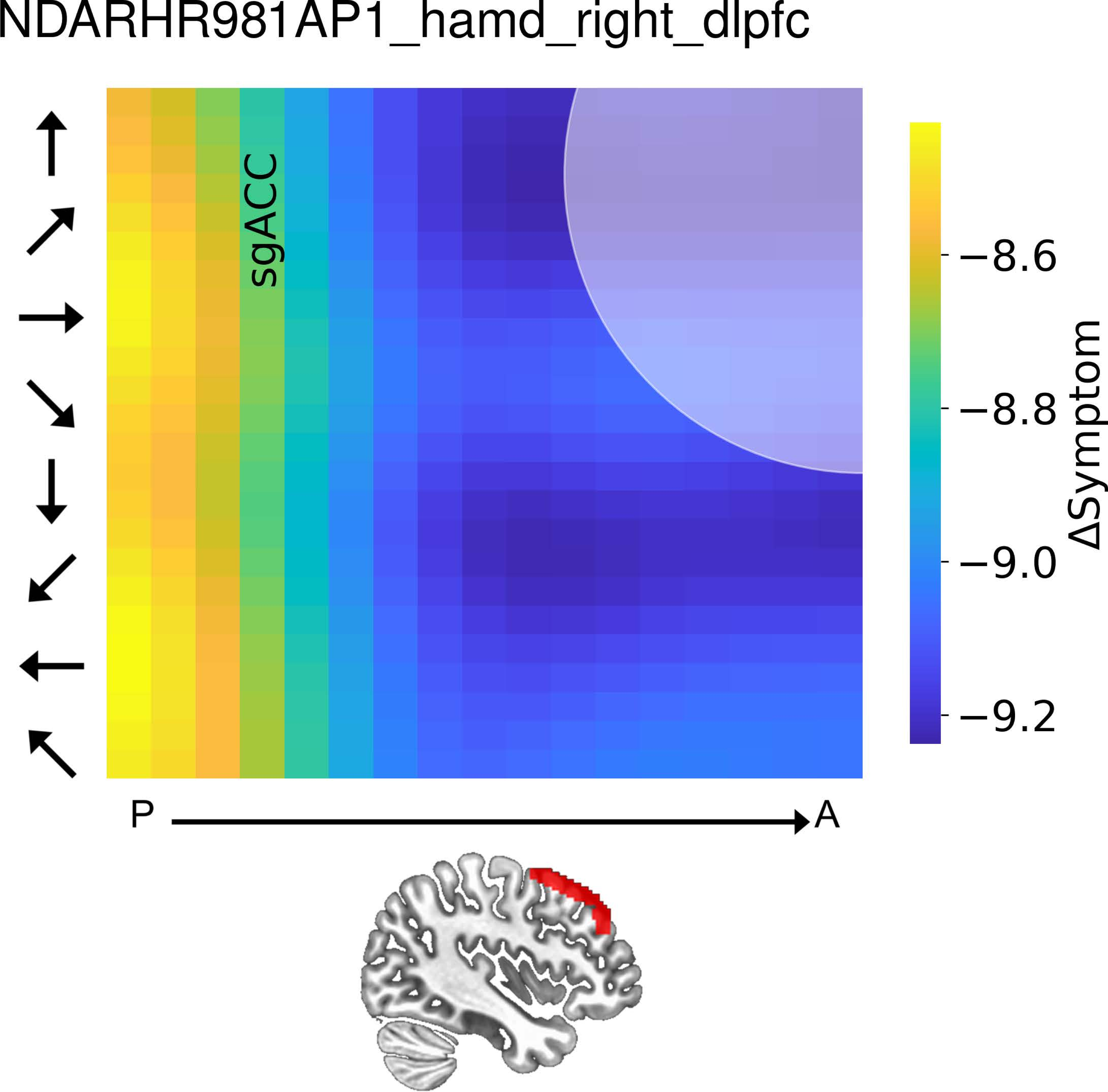
NDARHR981AP1_hamd_right_dlpfc
sgACC
P
A
−8.6
−8.8
−9.0
−9.2
ΔSymptom

NDARHX487CT9_hamd_right_dlpfc

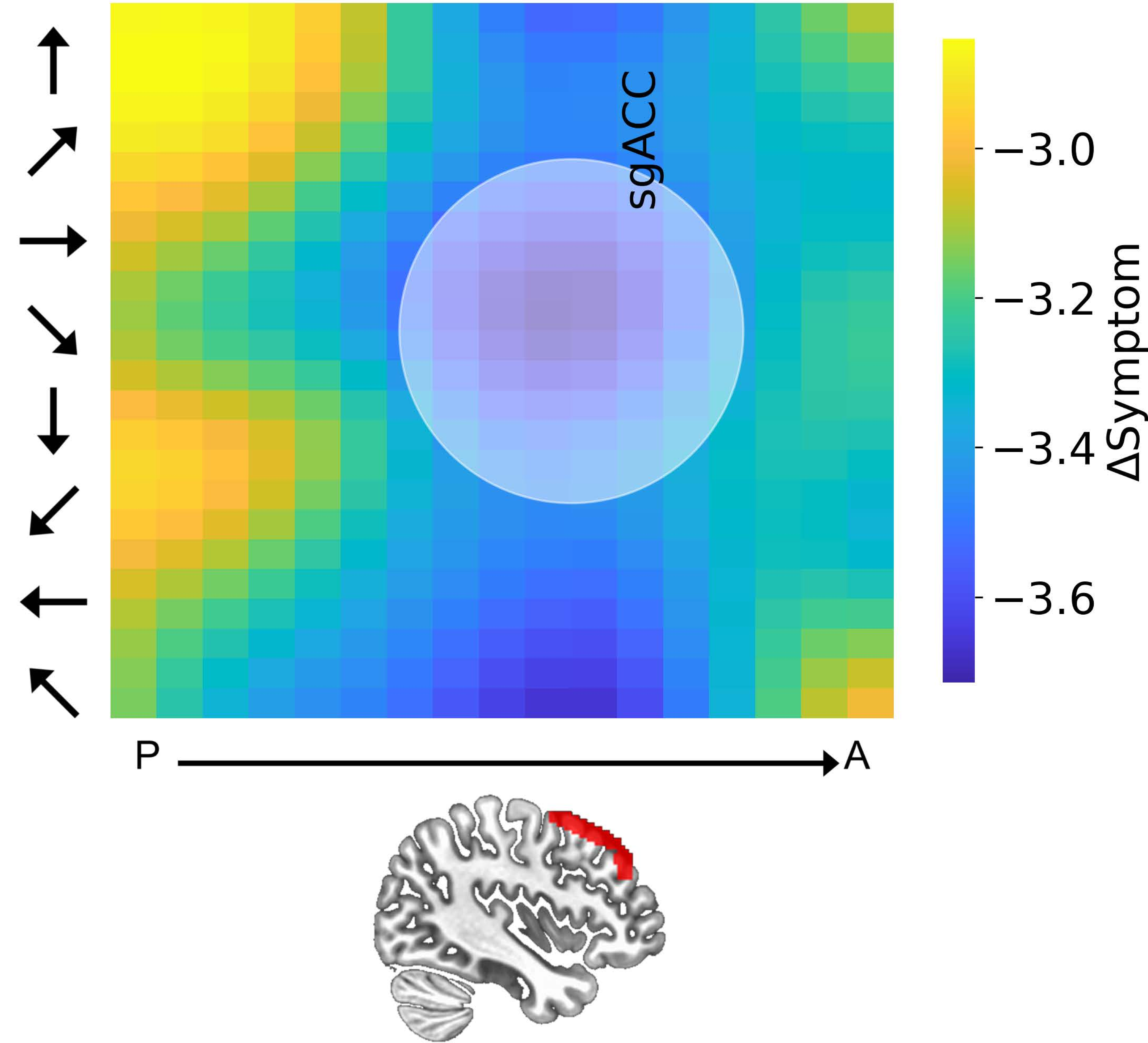

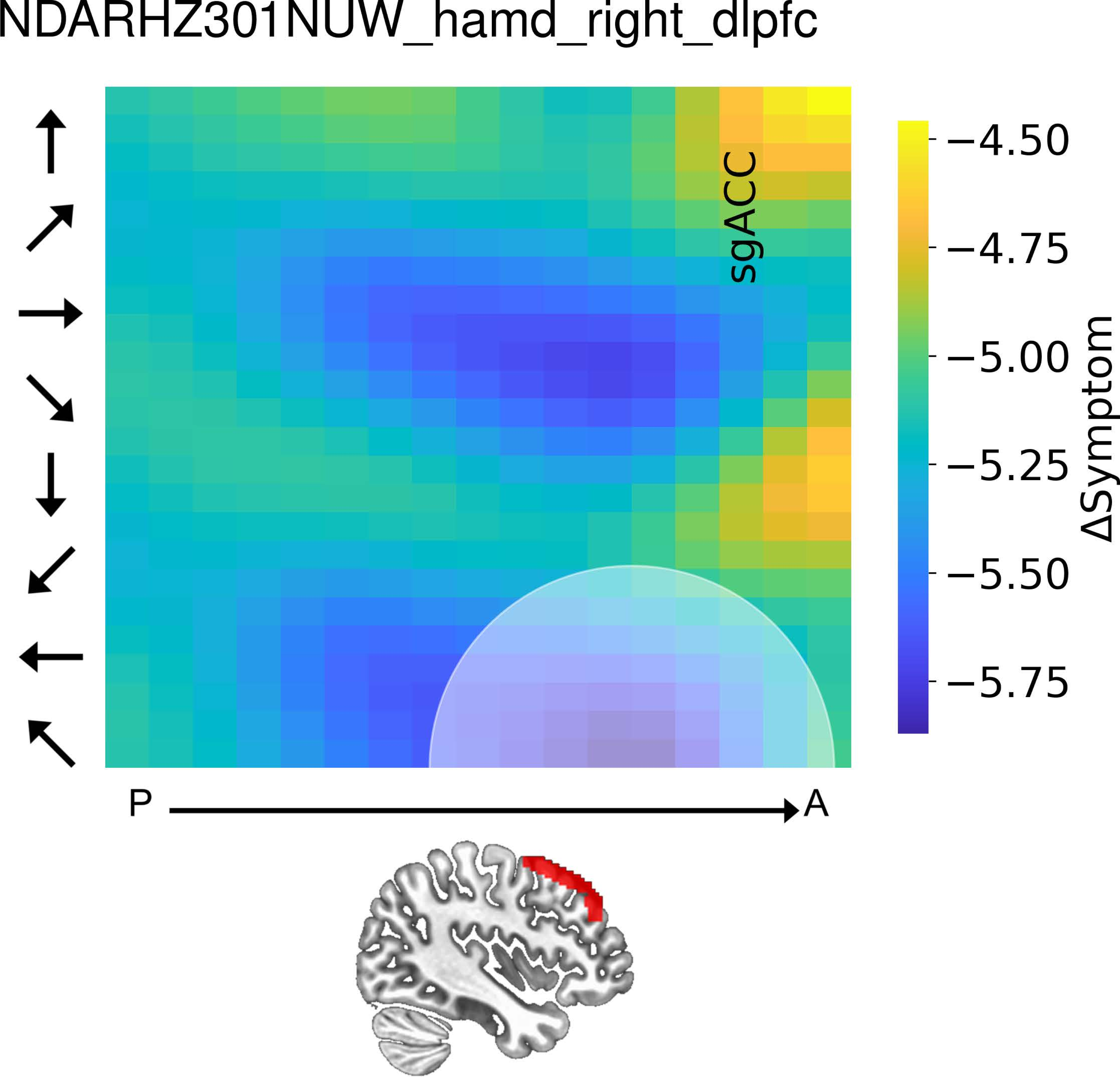
NDARHZ301NUW_hamd_right_dlpfc
sgACC
P
A
−4.50
−4.75
−5.00
−5.25
−5.50
−5.75
ΔSymptom

NDARHZ662EPK_hamd_right_dlpfc

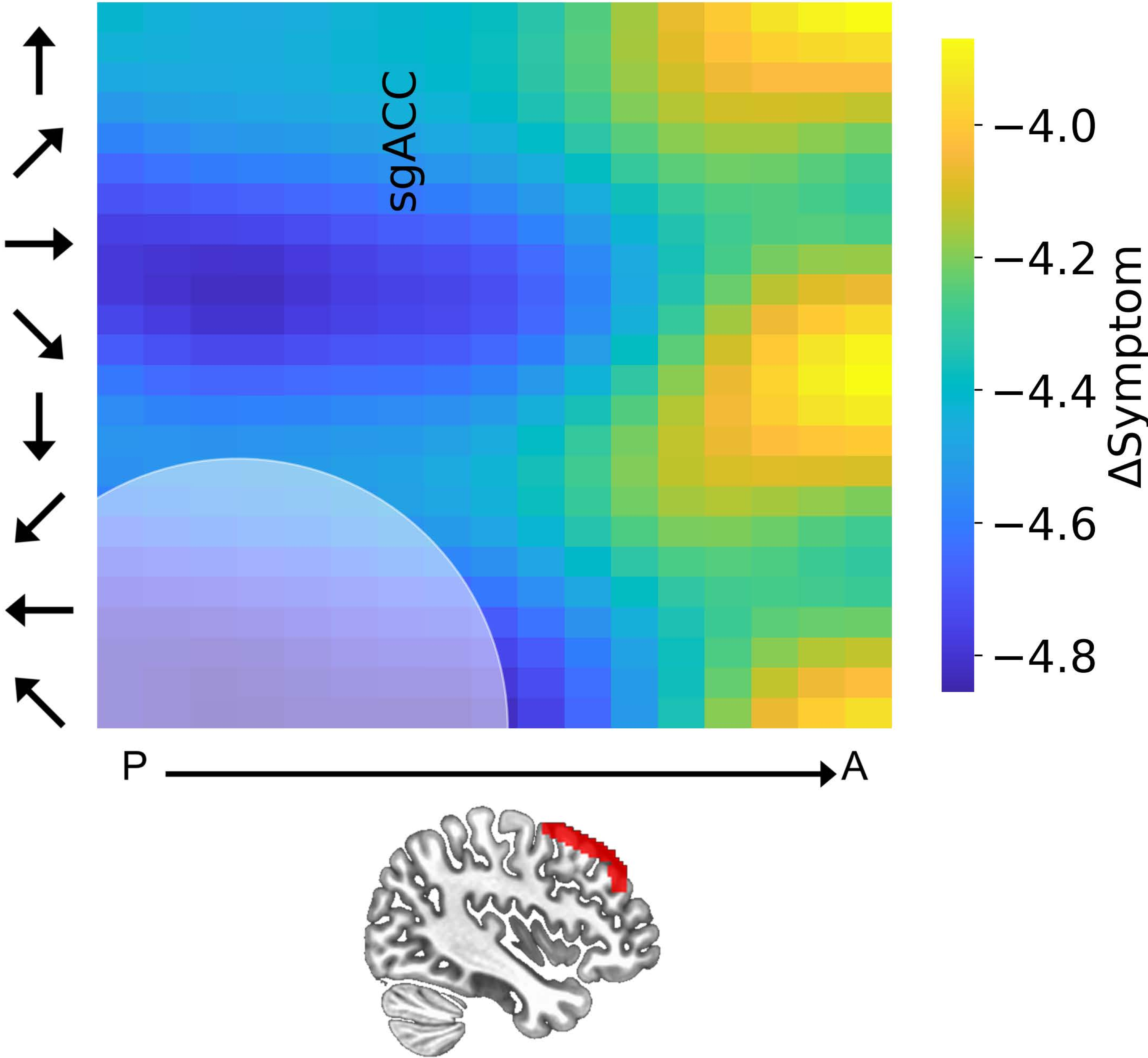

NDARJD271PMF_hamd_right_dlpfc

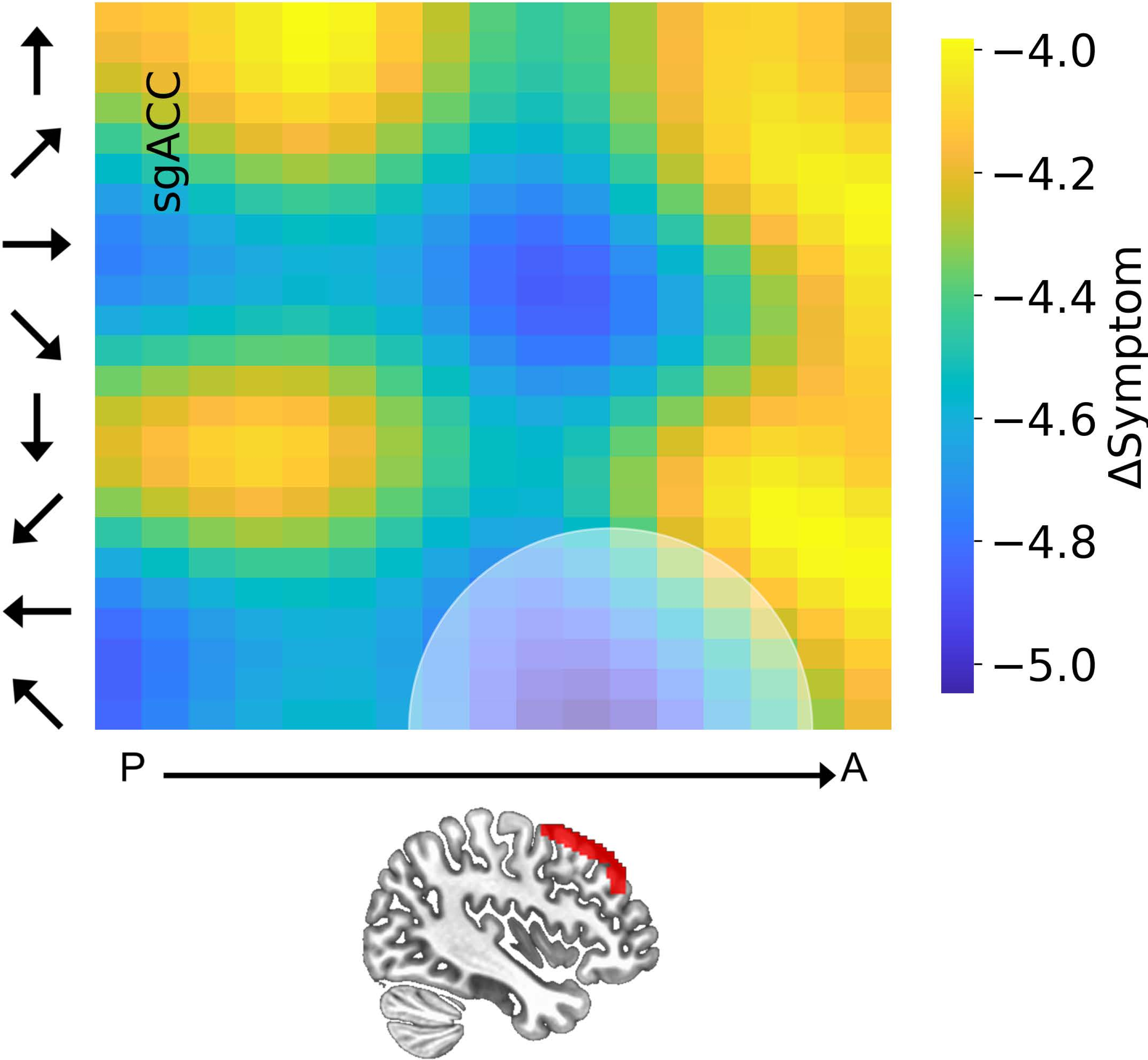

NDARJD548GTC_hamd_right_dlpfc

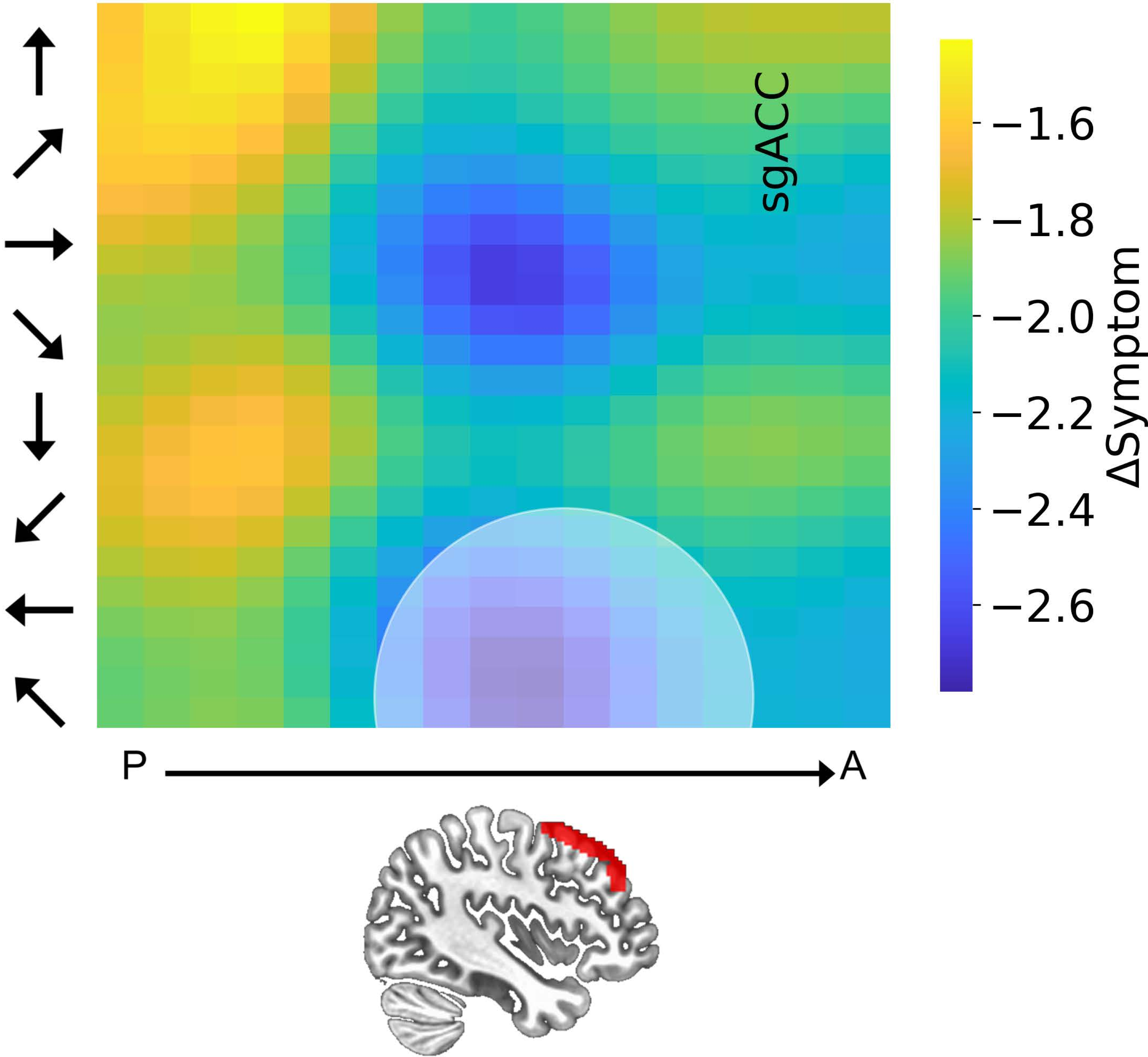

NDARJF993NV1_hamd_right_dlpfc

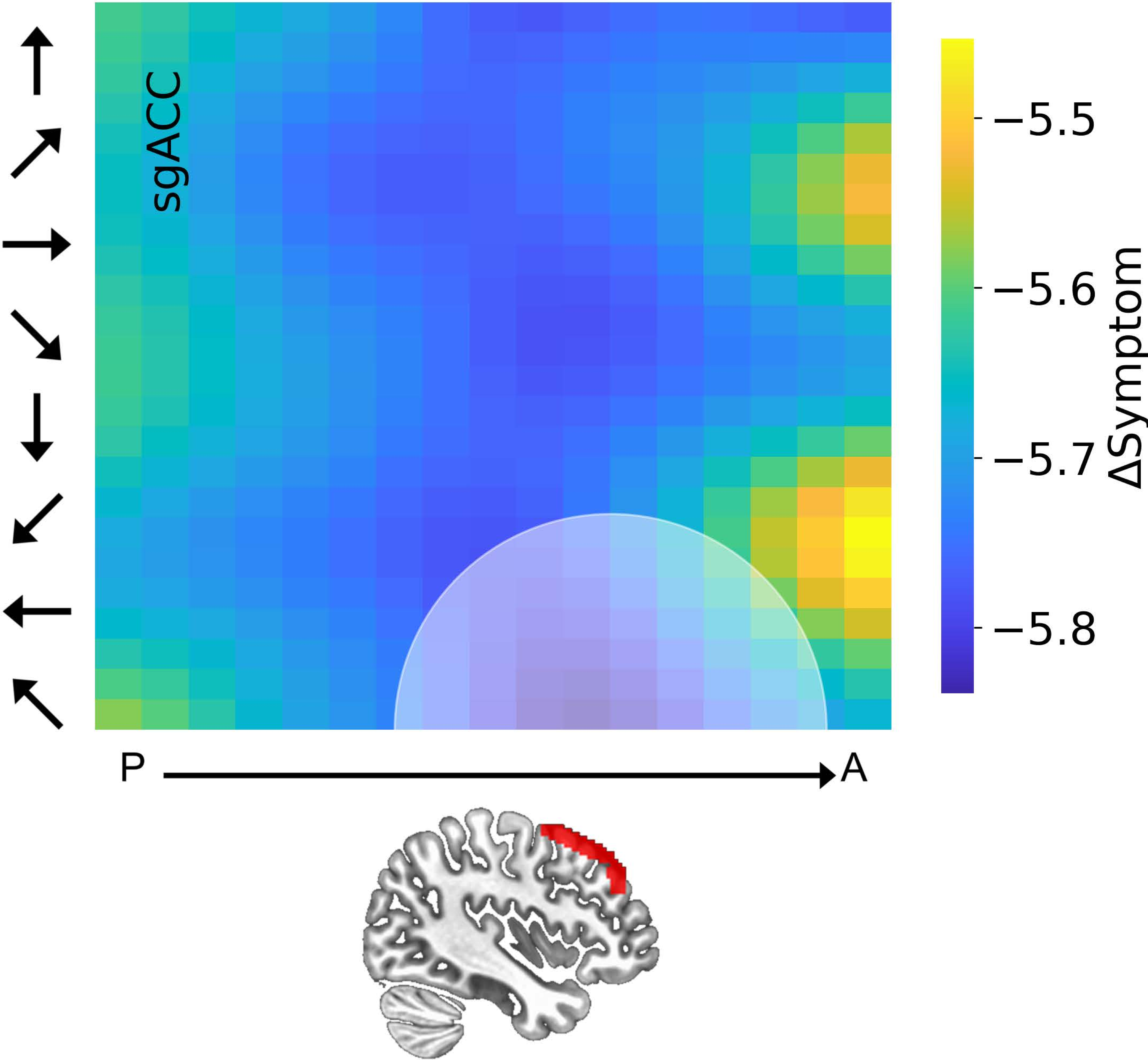

NDARJH406WC9_hamd_right_dlpfc

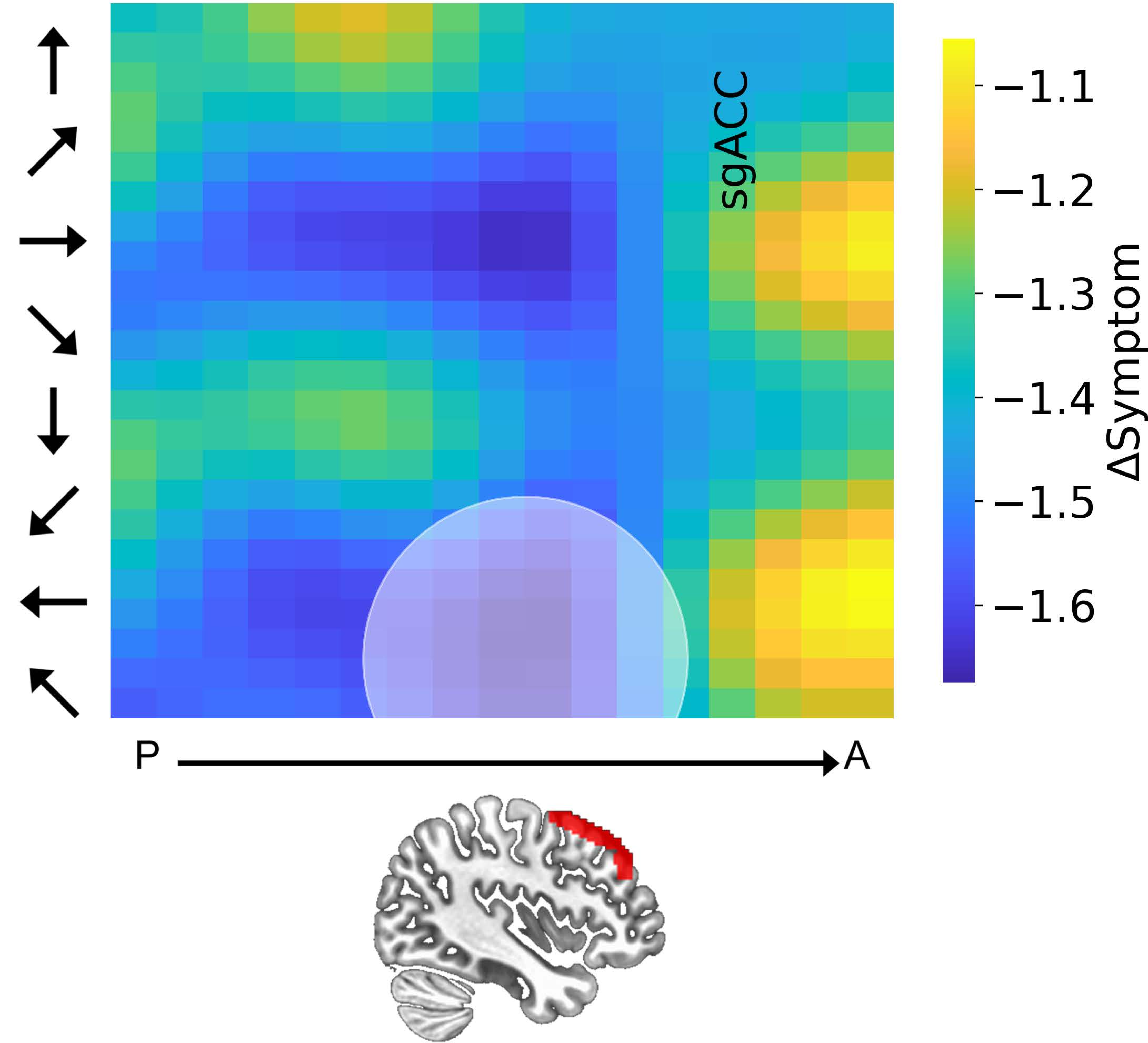

NDARJX575DVK_hamd_right_dlpfc

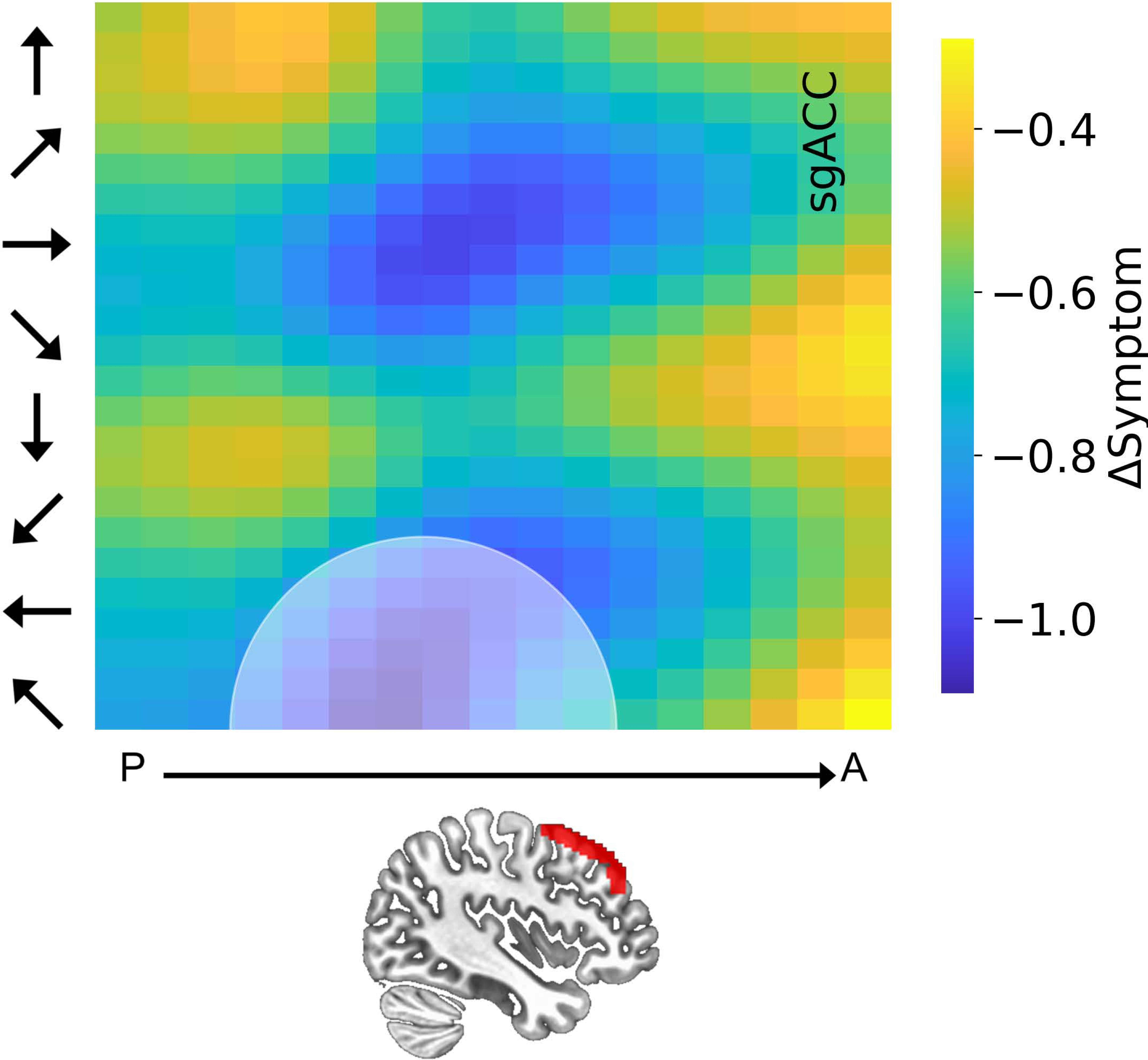

NDARJY042TLY_hamd_right_dlpfc

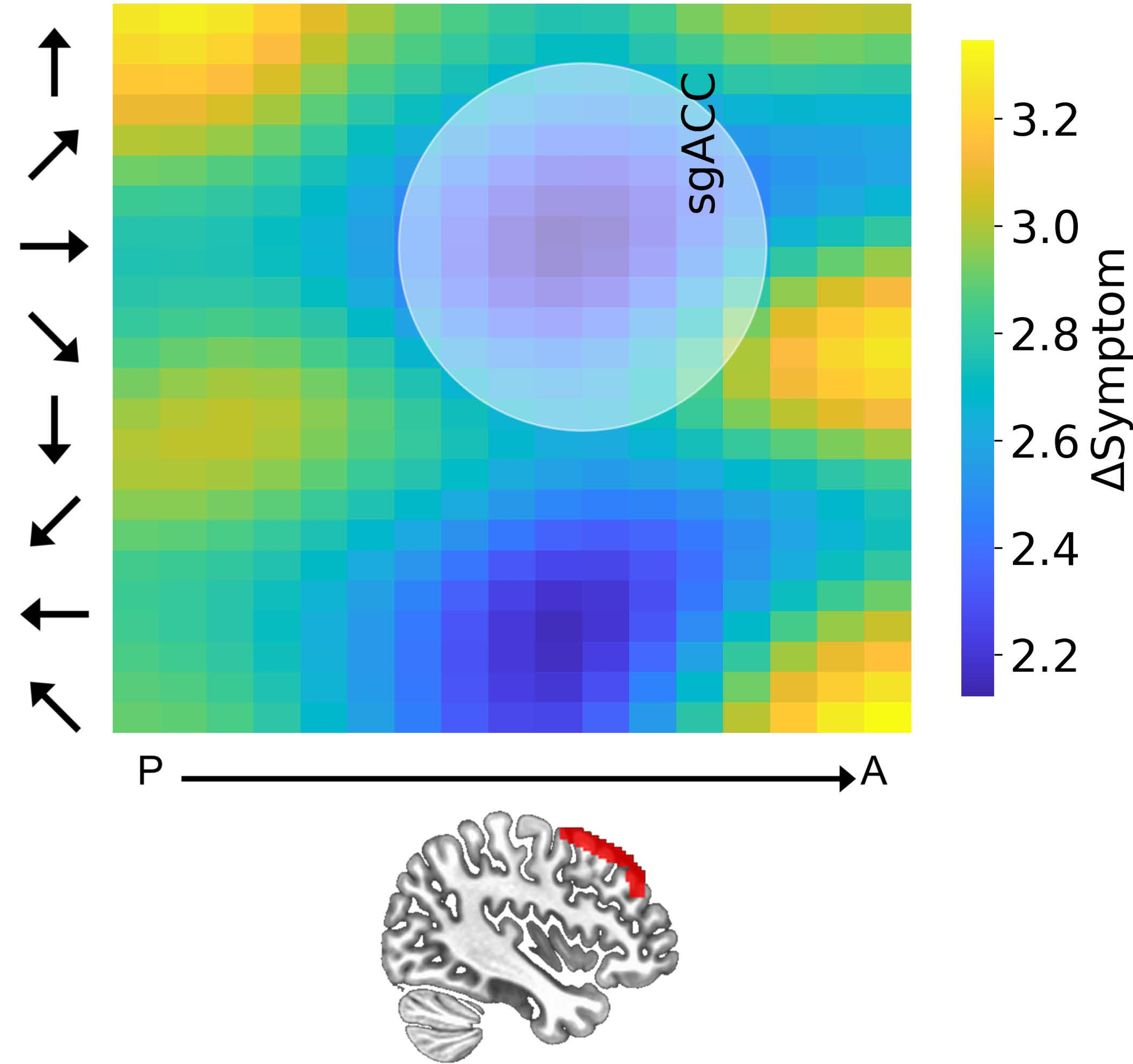

NDARKK212YTX_hamd_right_dlpfc

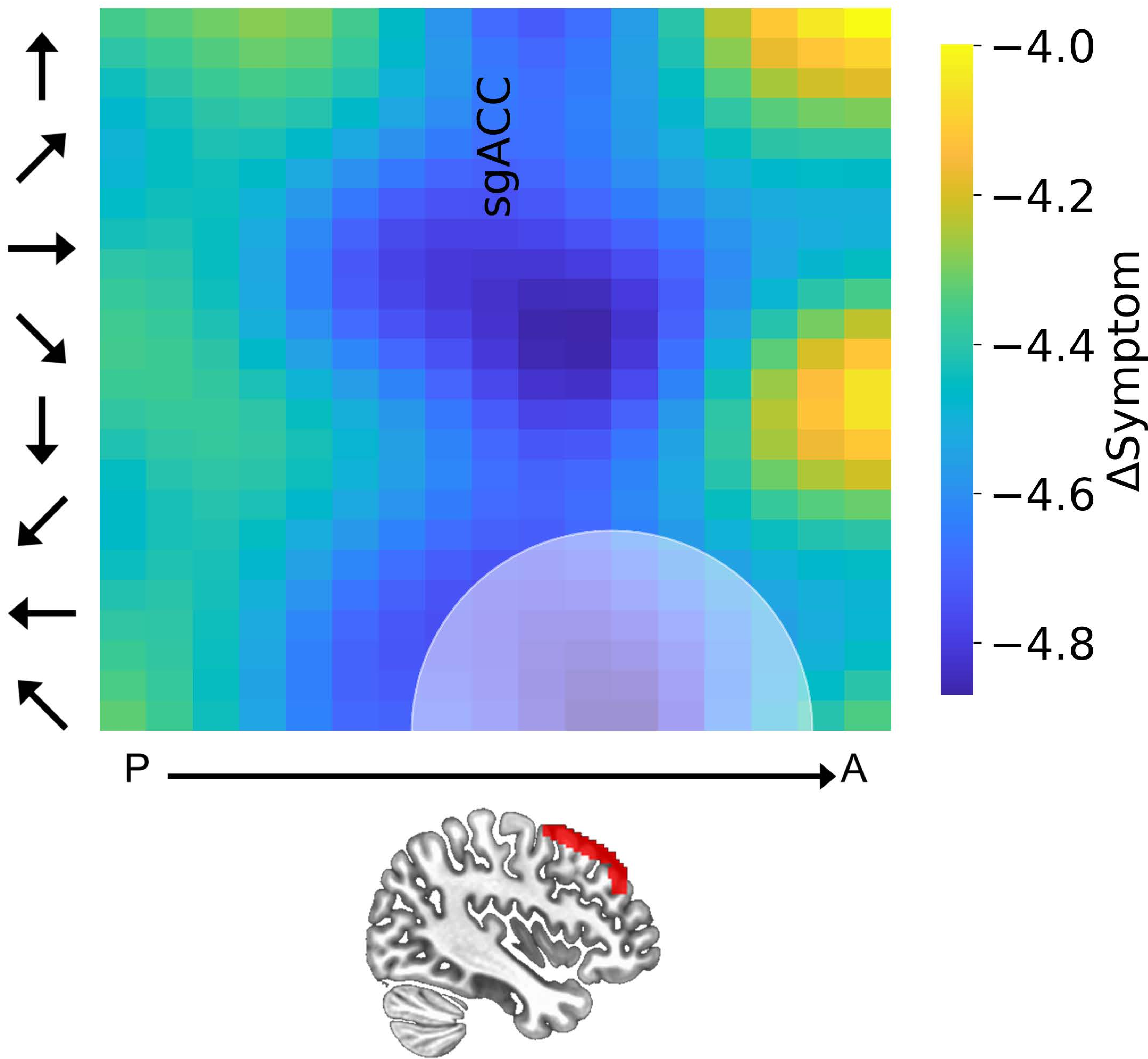

NDARKM333VX2_hamd_right_dlpfc

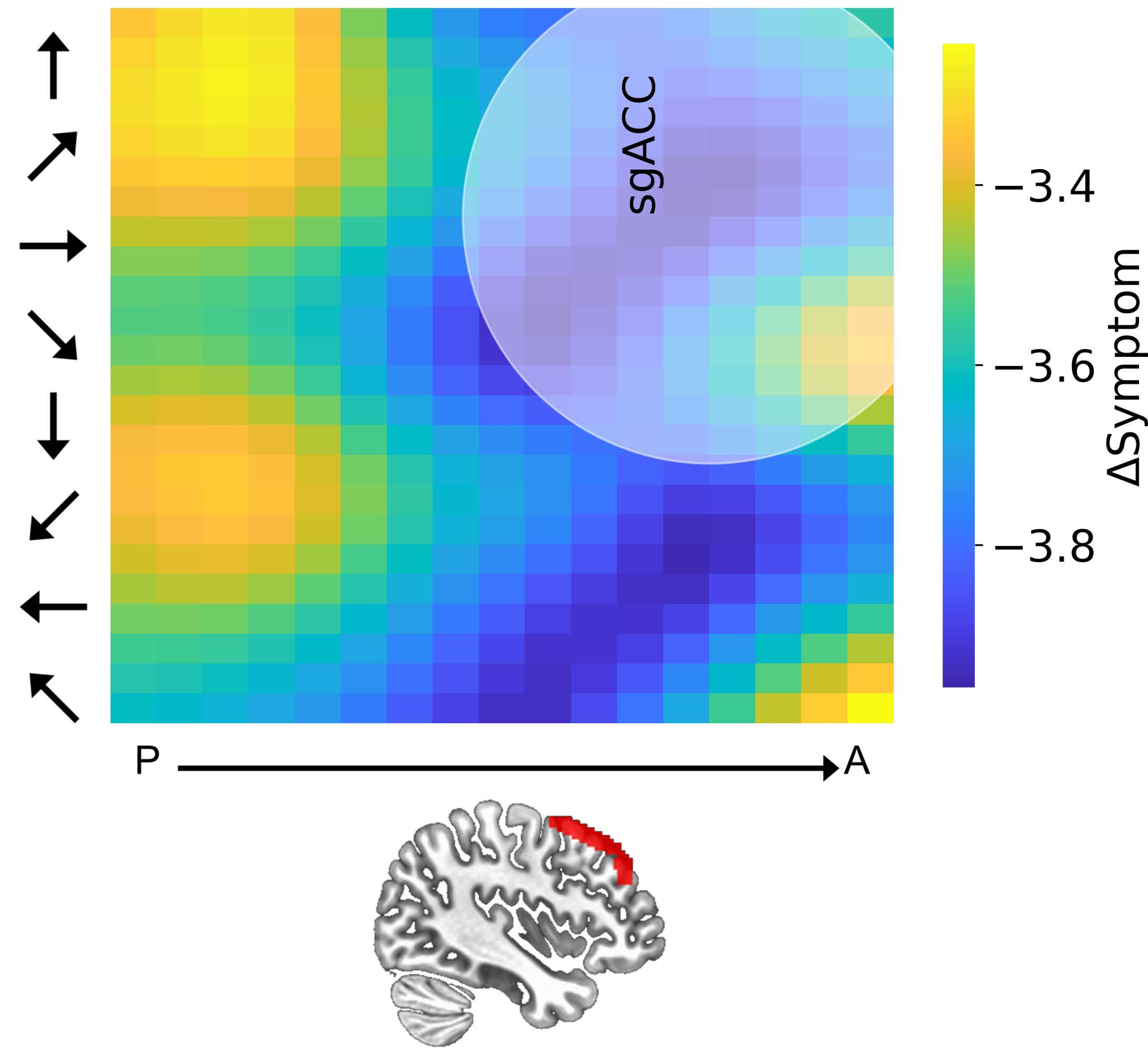

NDARKT991DUC_hamd_right_dlpfc

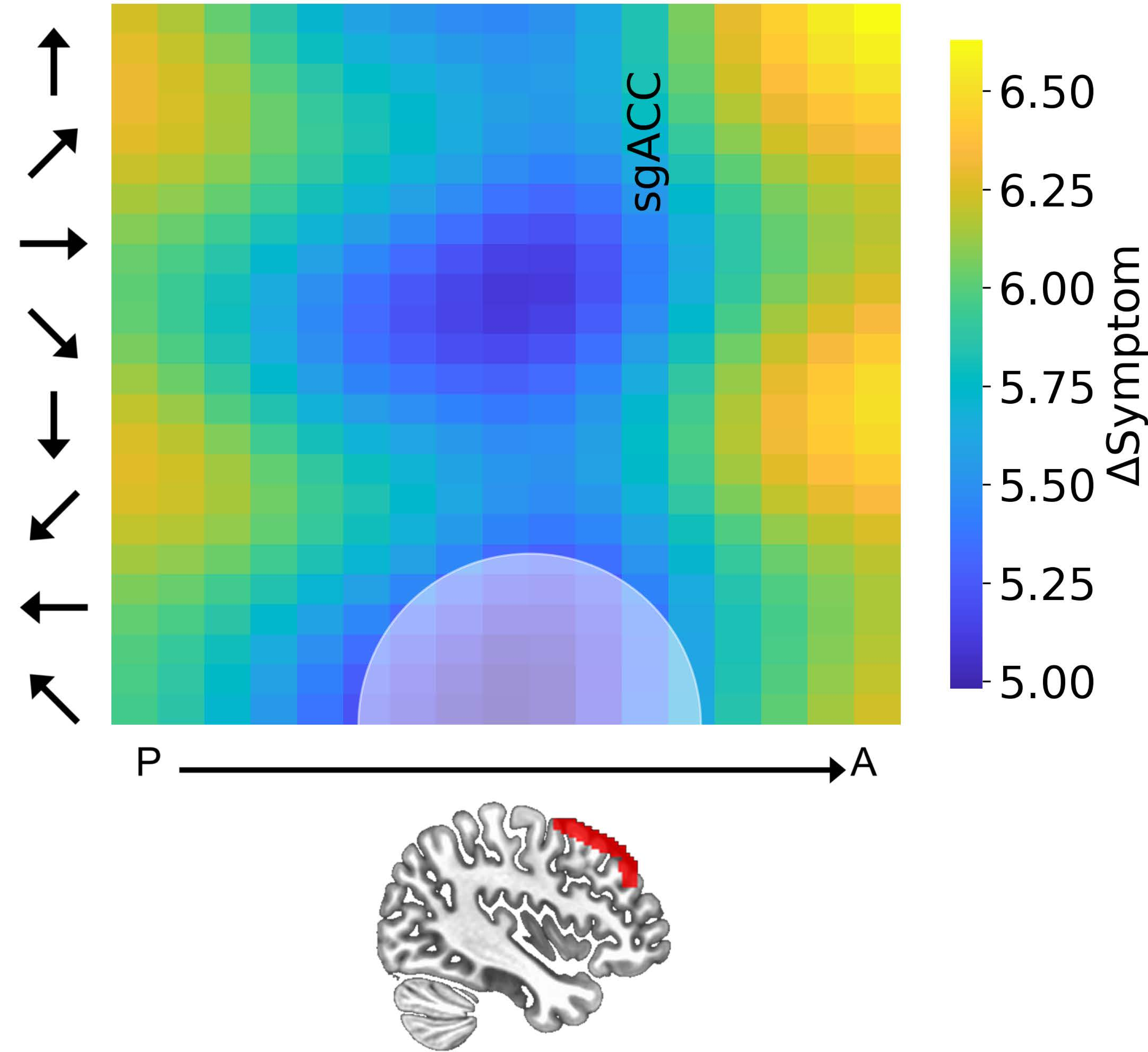

NDARKU015LTM_hamd_right_dlpfc

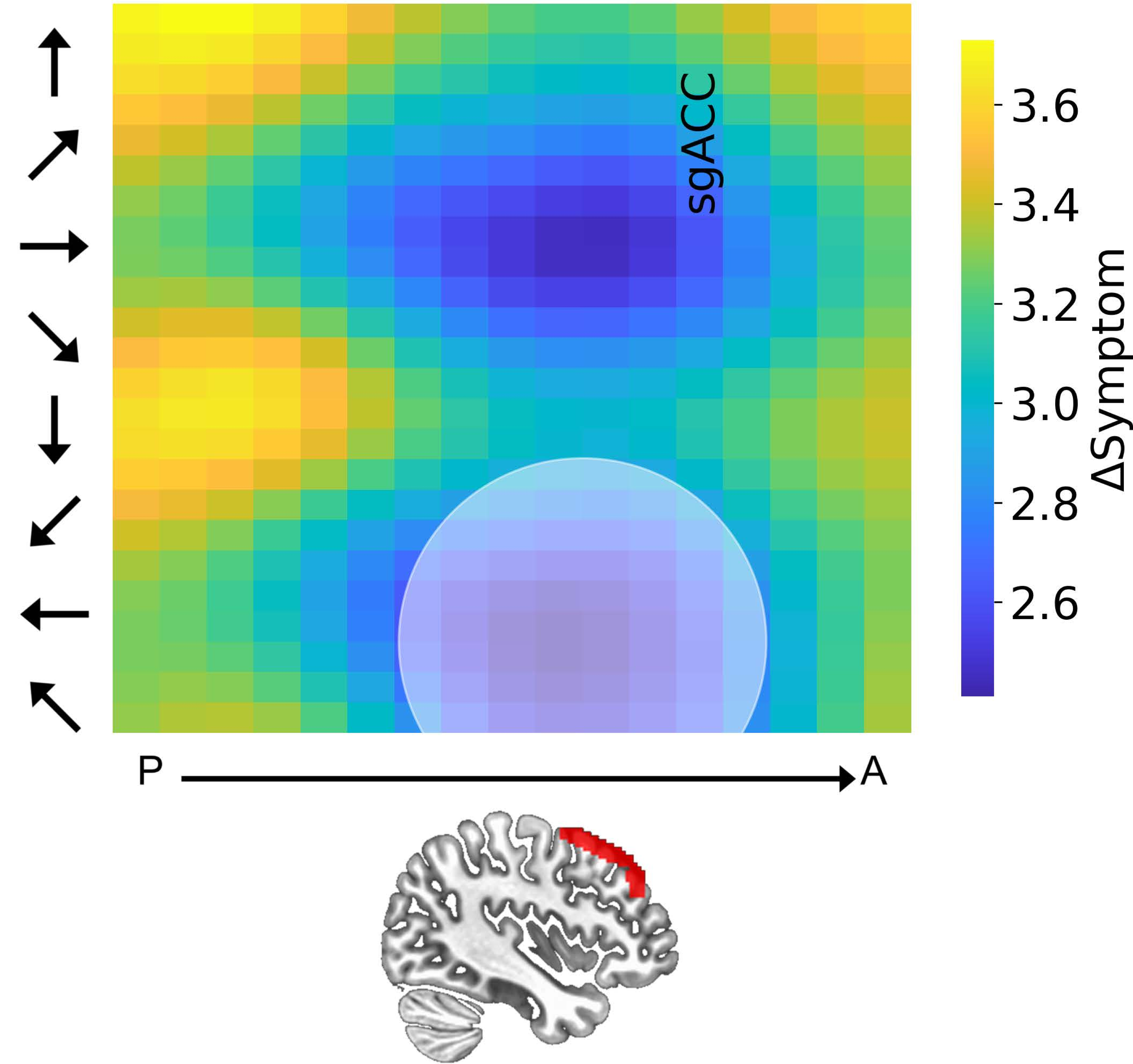

NDARKV126RTP_hamd_right_dlpfc

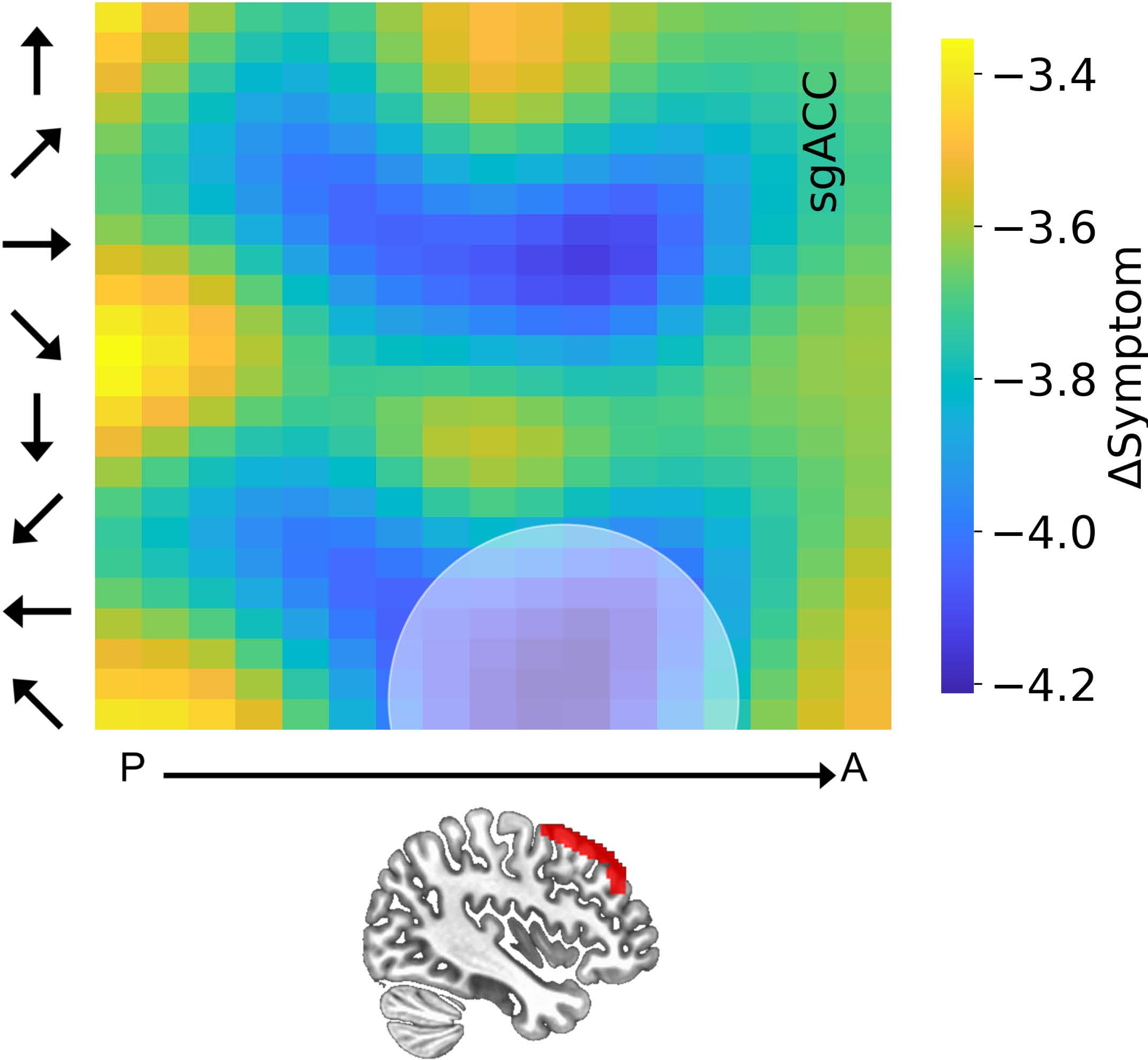

NDARLF826BAB_hamd_right_dlpfc

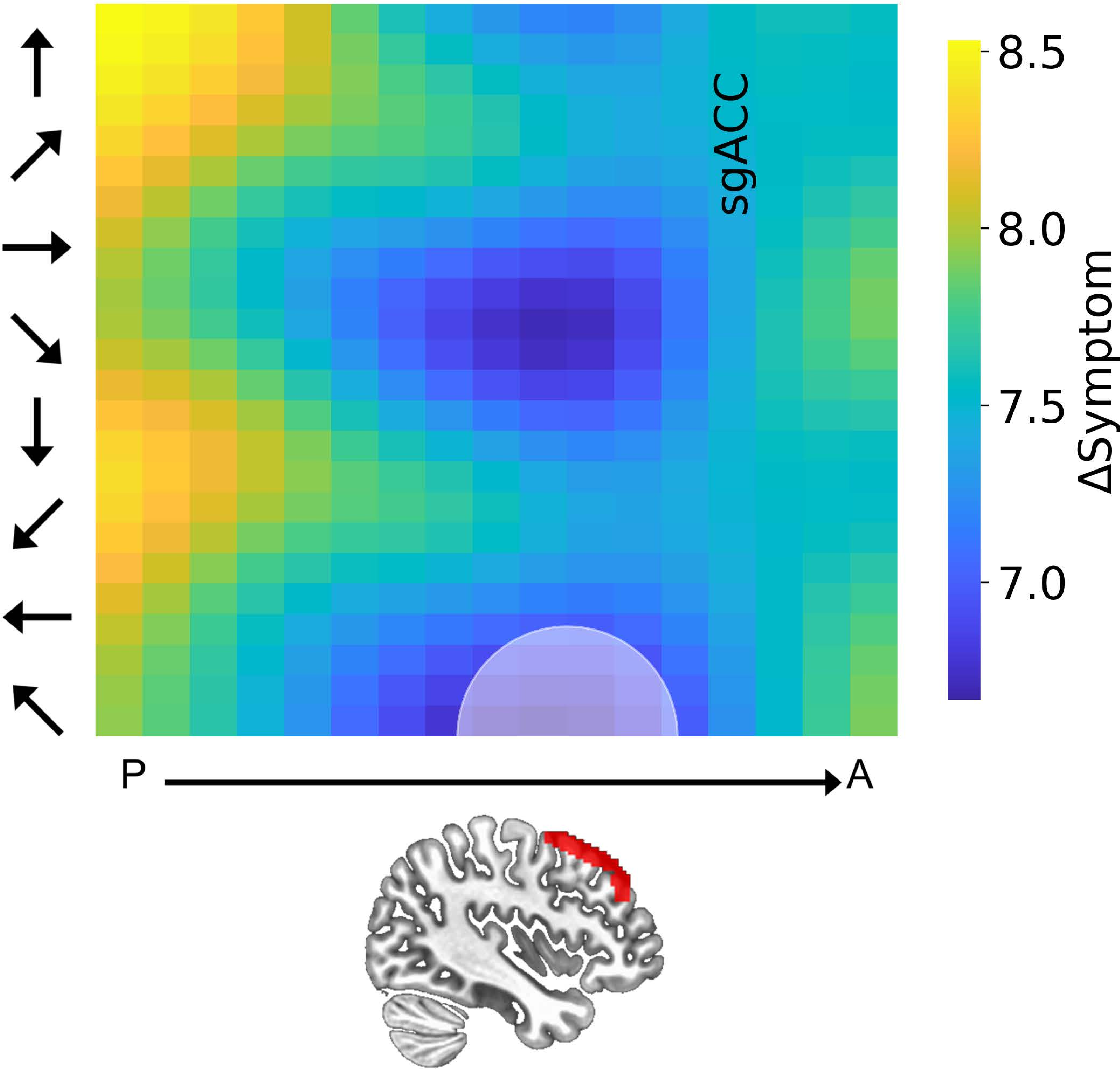

NDARLM890ELE_hamd_right_dlpfc

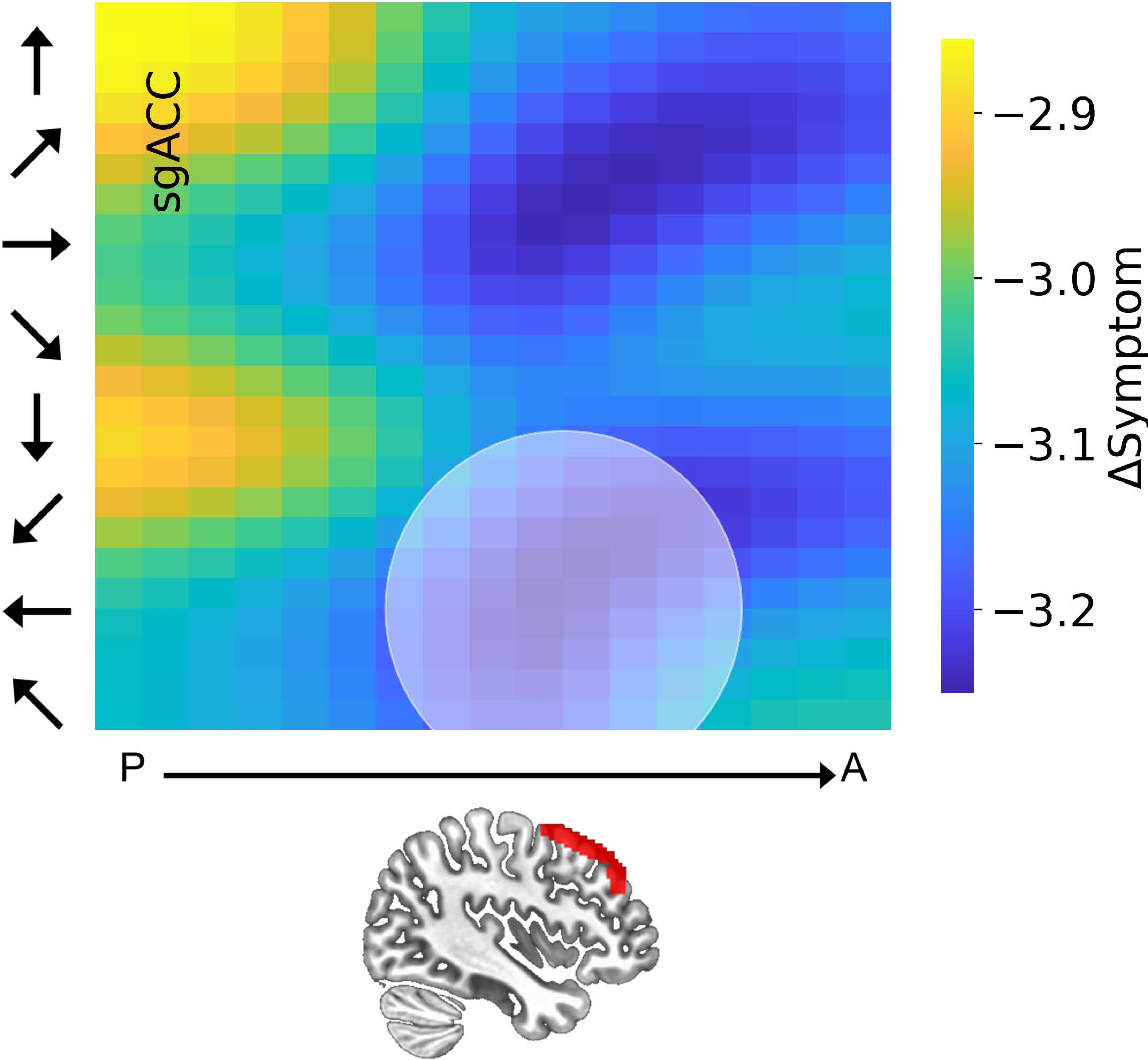

NDARLZ935EEN_hamd_right_dlpfc

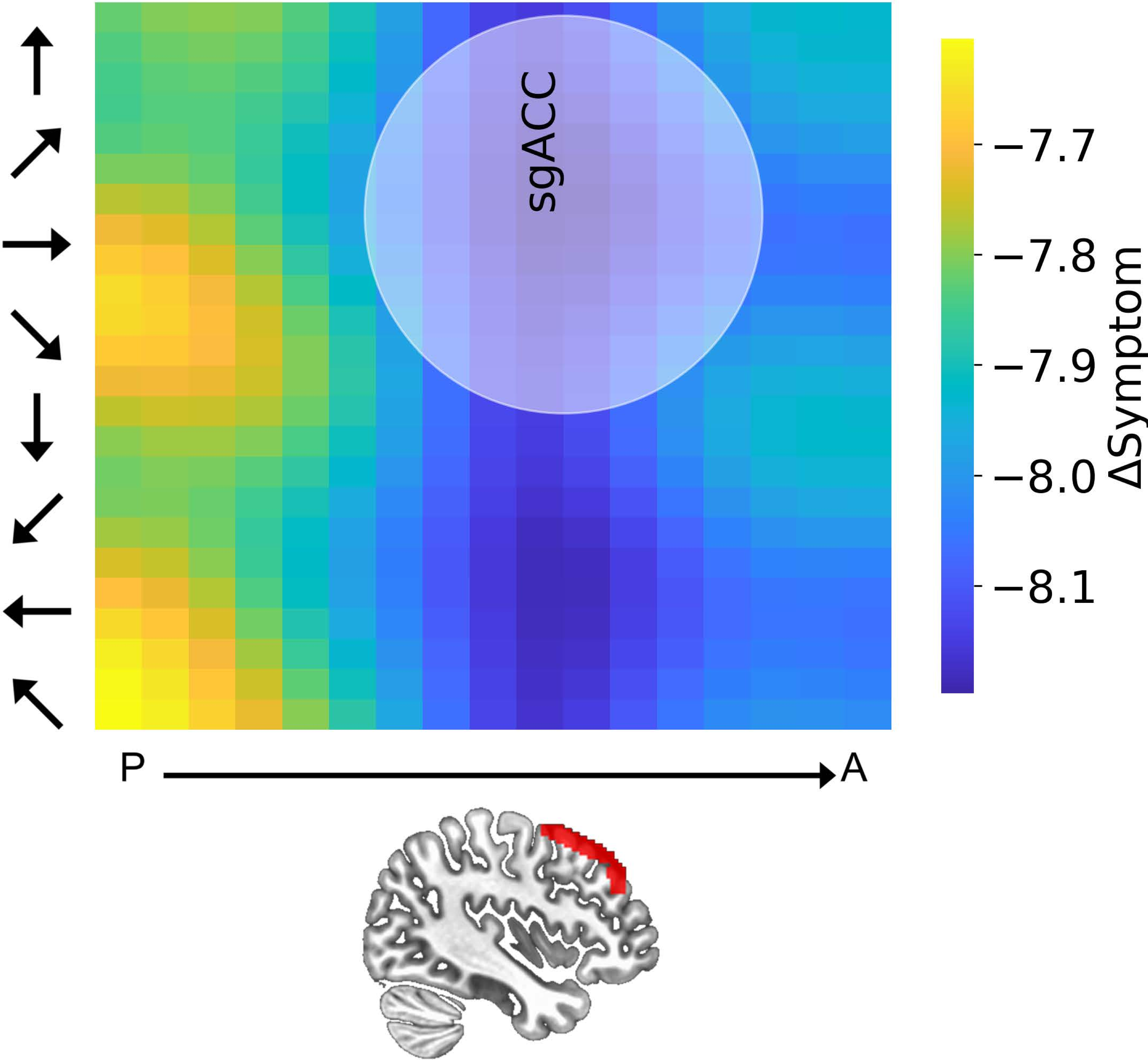

NDARMA155ATG_hamd_right_dlpfc

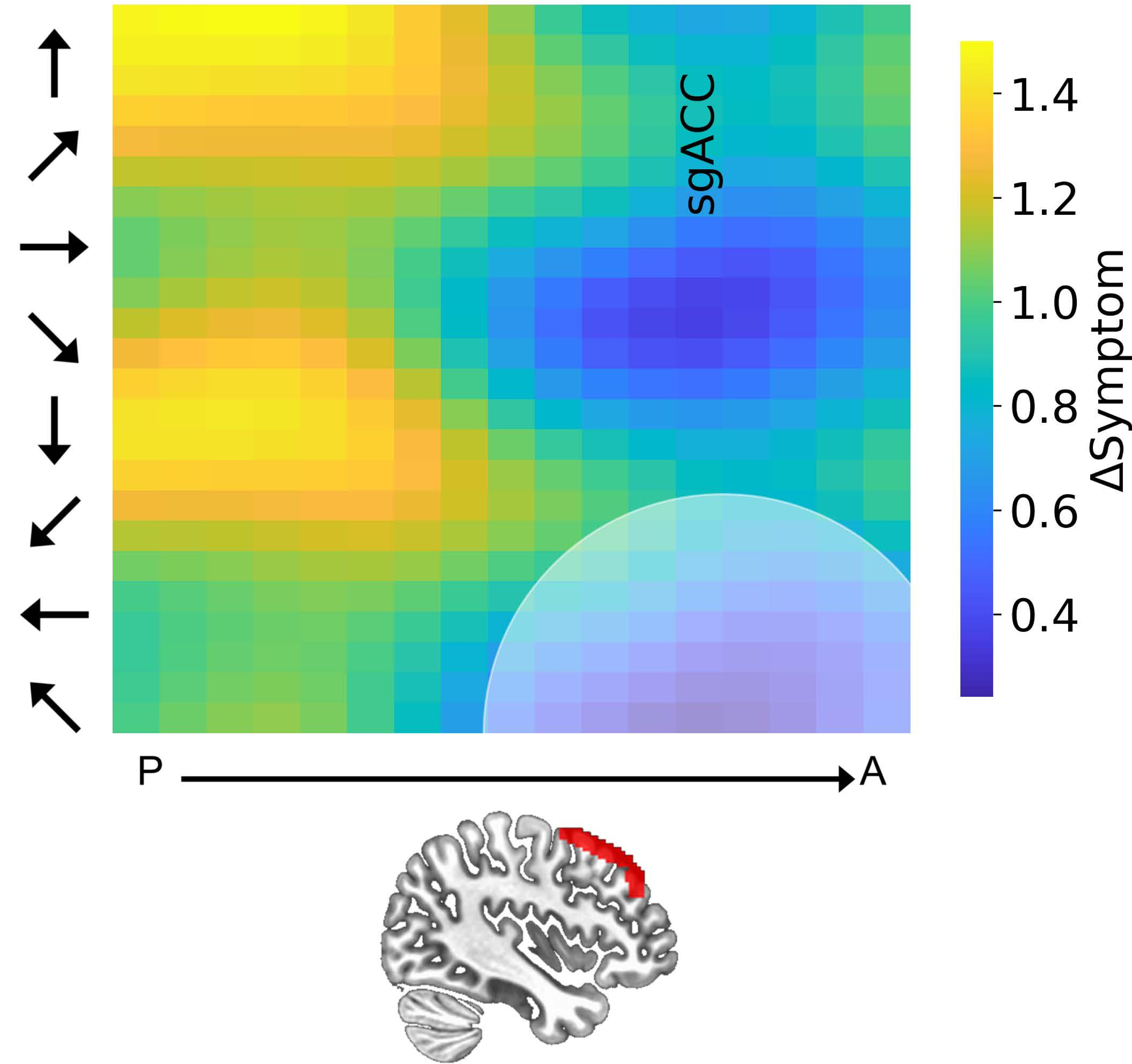

NDARMB943VPJ_hamd_right_dlpfc

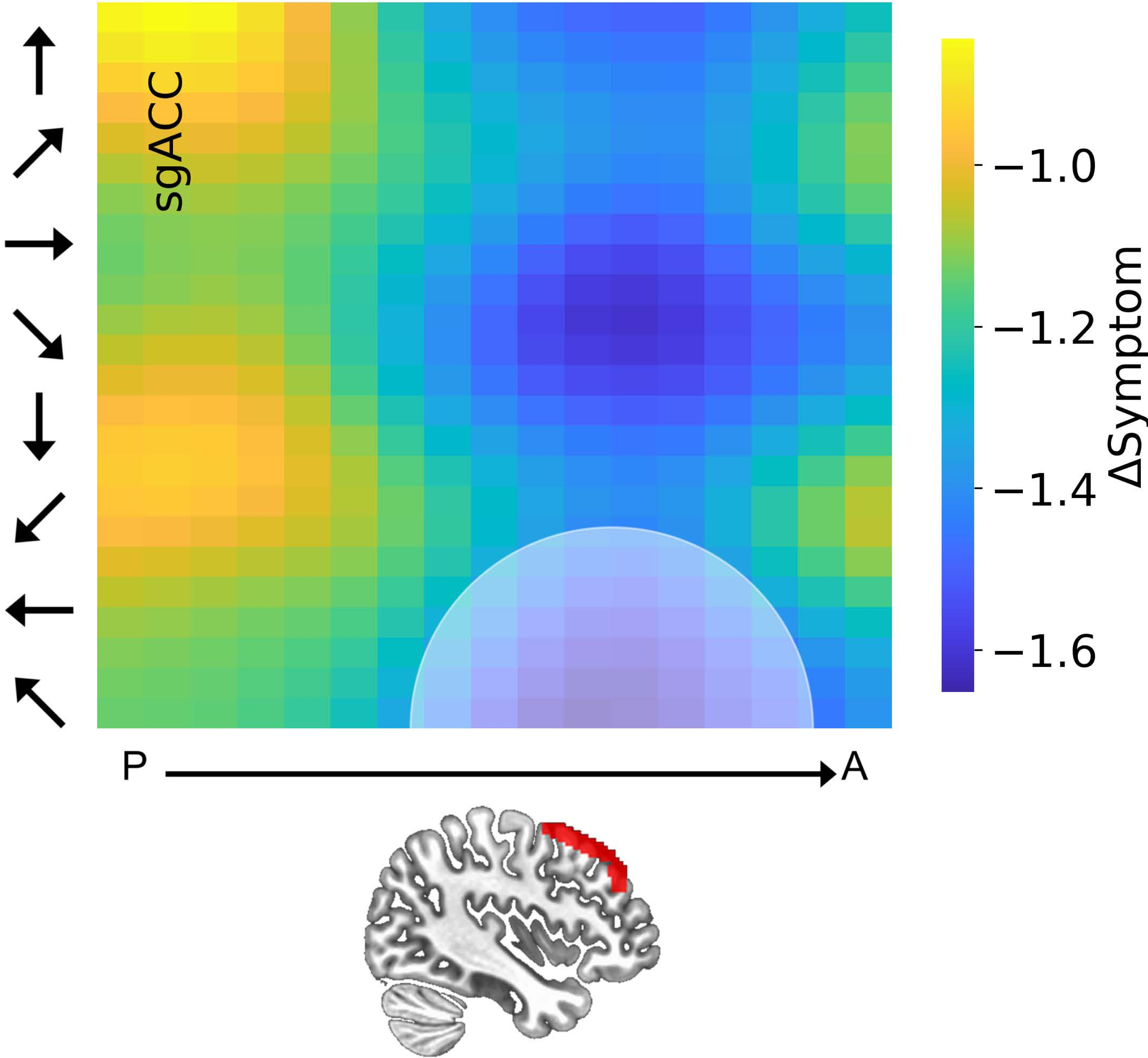

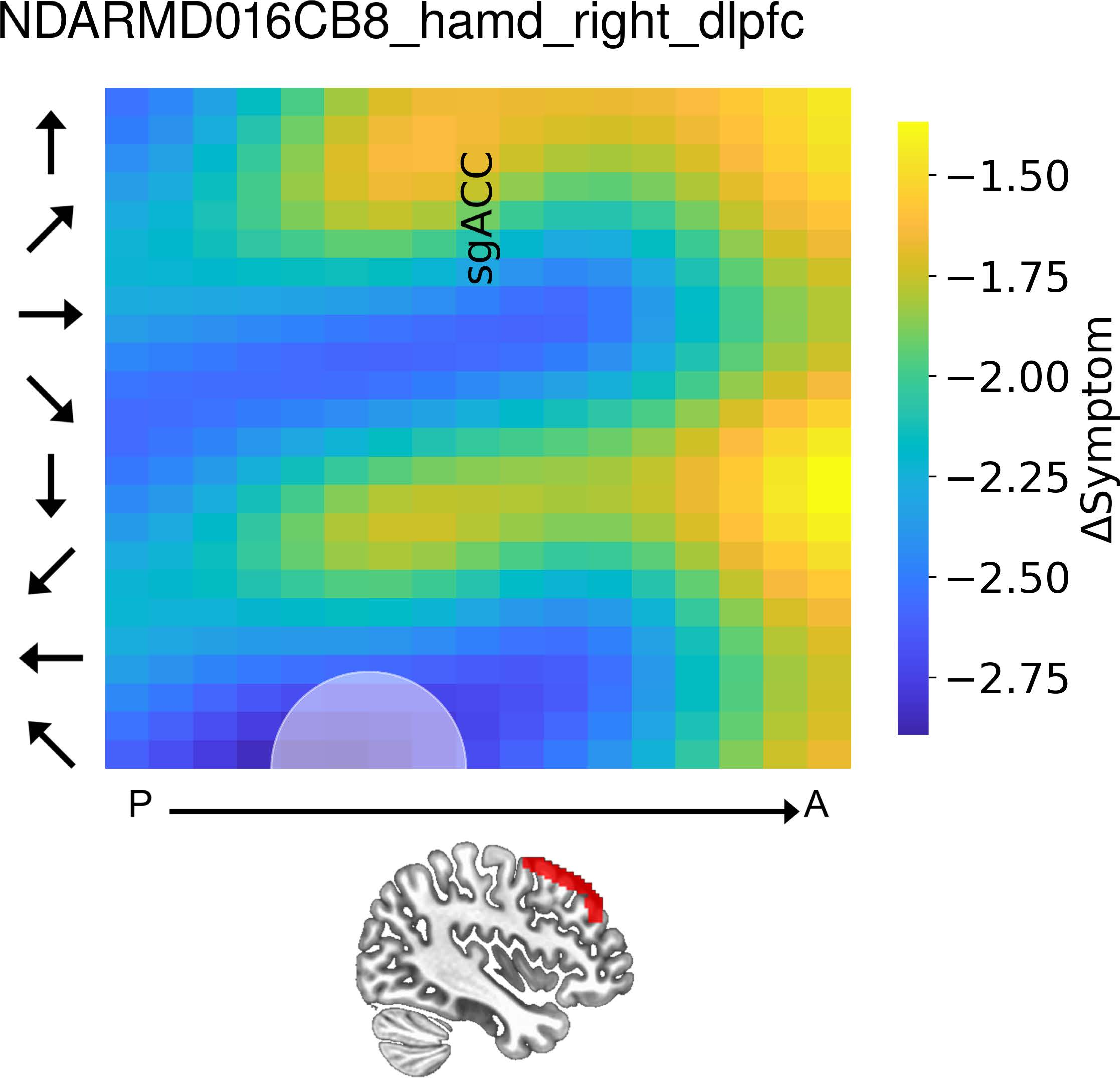
NDARMD016CB8_hamd_right_dlpfc
sgACC
P
A
−1.50
−1.75
−2.00
−2.25
−2.50
−2.75
ΔSymptom

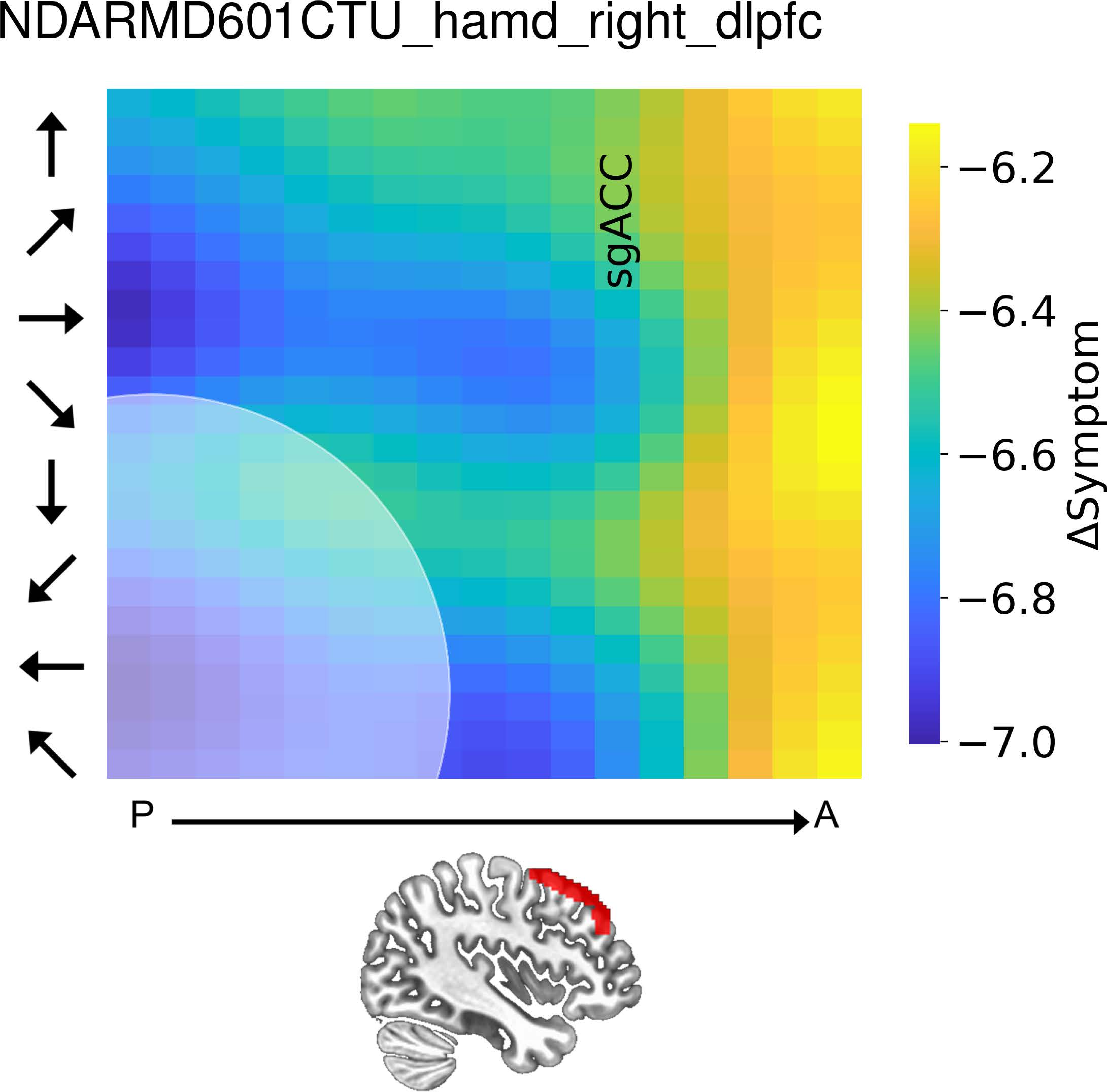
NDARMD601CTU_hamd_right_dlpfc
sgACC
−6.2
−6.4
−6.6
−6.8
−7.0
ΔSymptom
P
A

NDARME189AXP_hamd_right_dlpfc

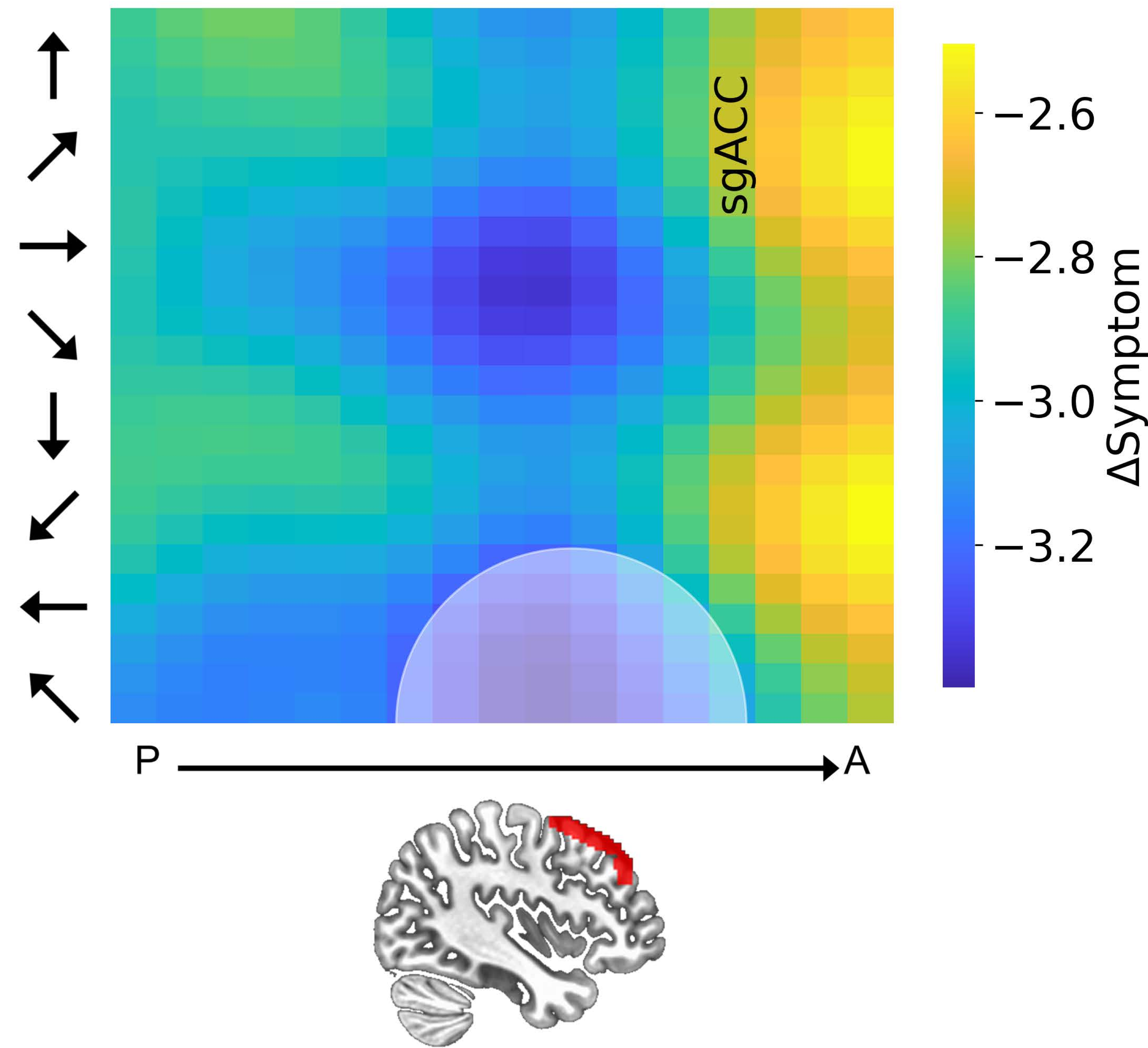

NDARME335LZJ_hamd_right_dlpfc

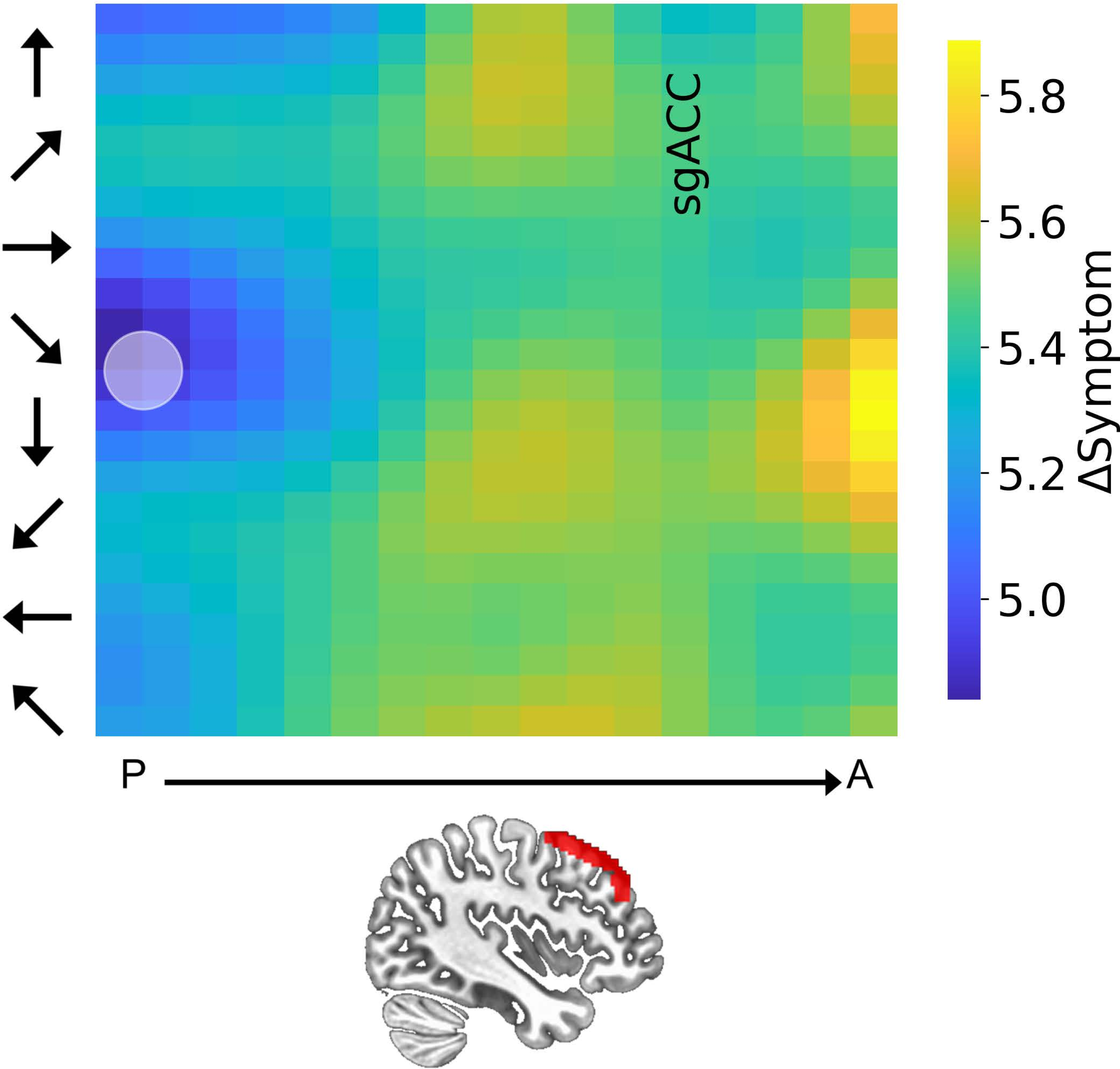

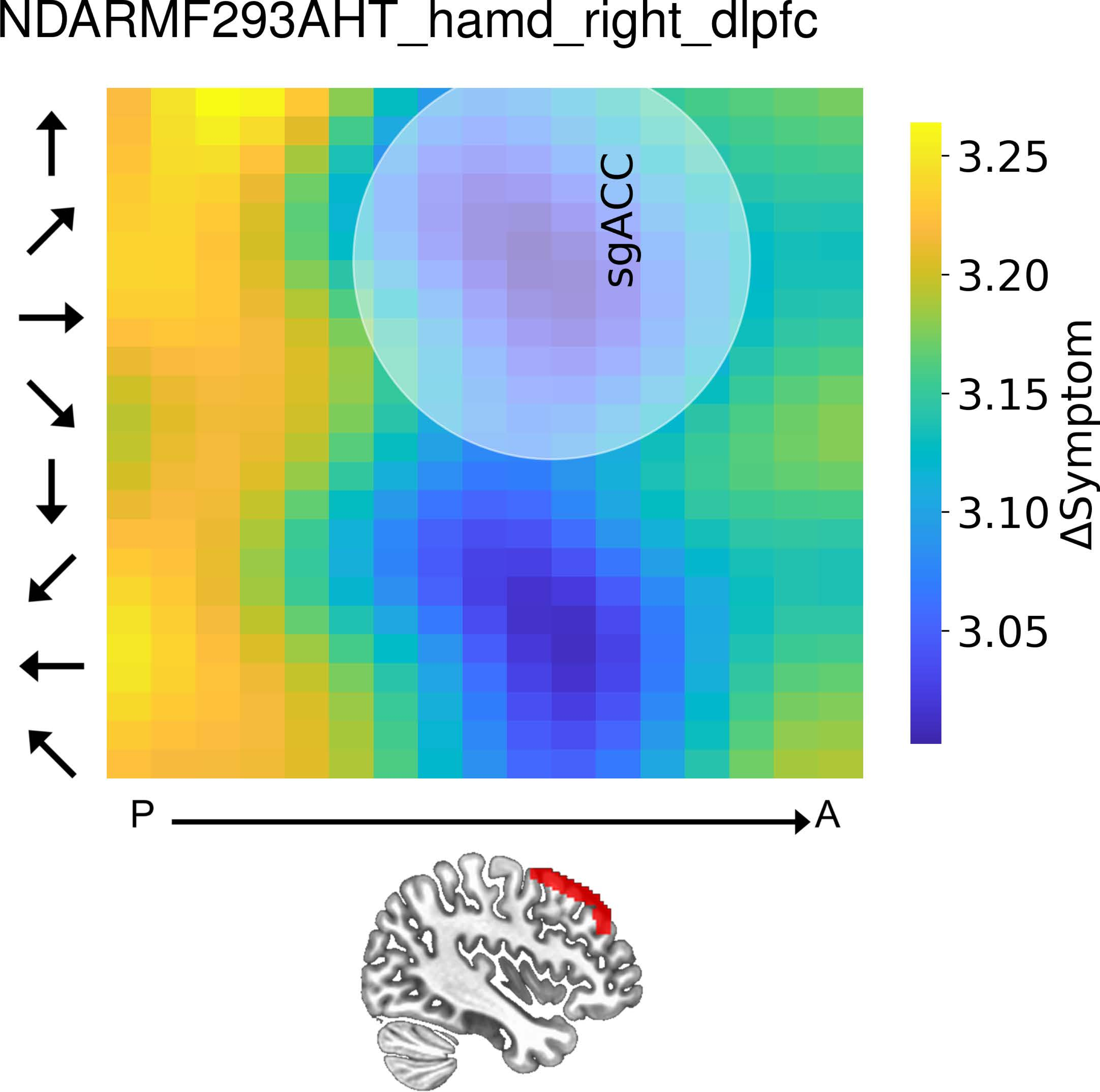
NDARMF293AHT_hamd_right_dlpfc
sgACC
3.25
3.20
3.15
3.10
3.05
ΔSymptom
P
A

NDARMK972BFE_hamd_right_dlpfc

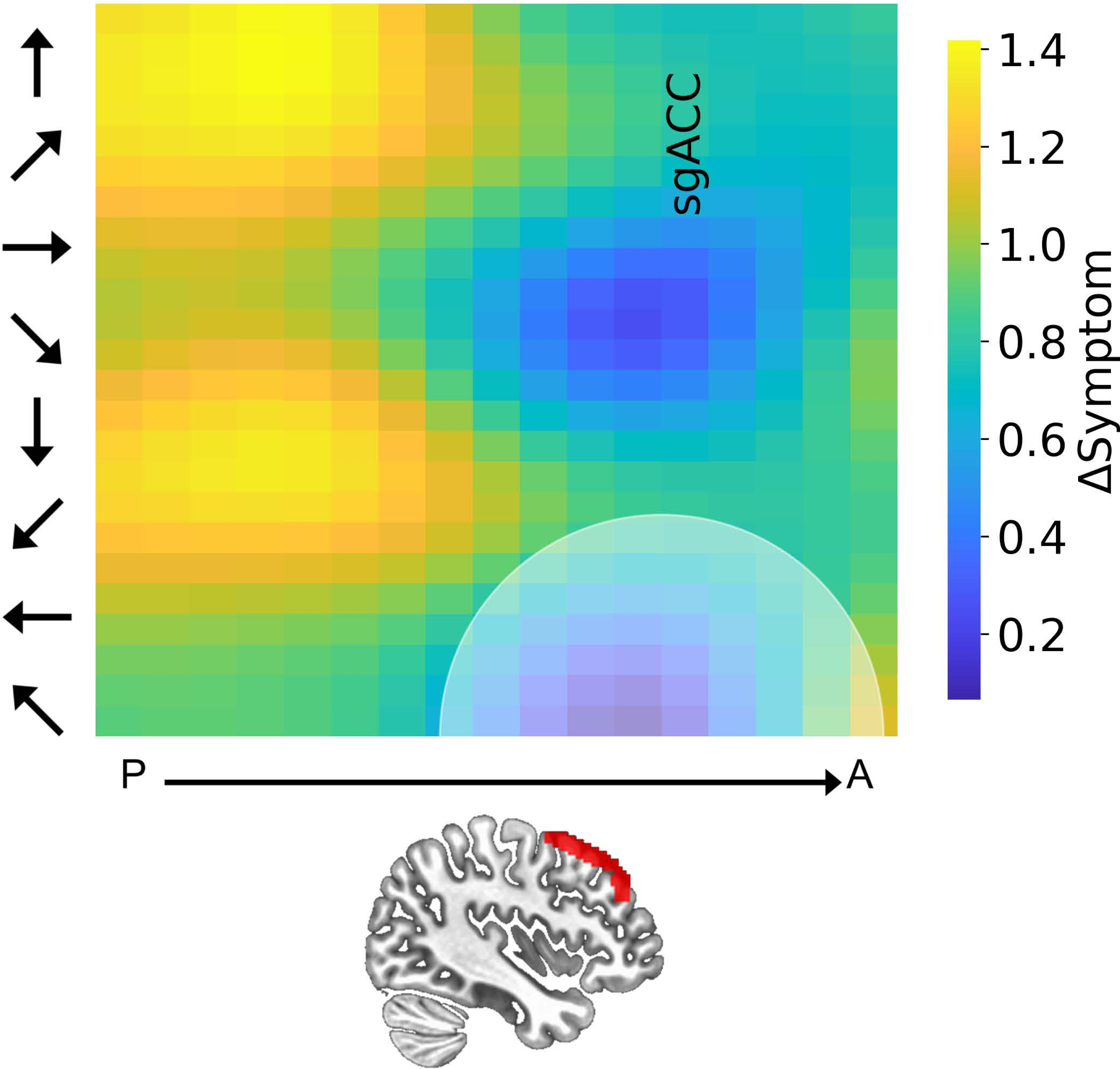

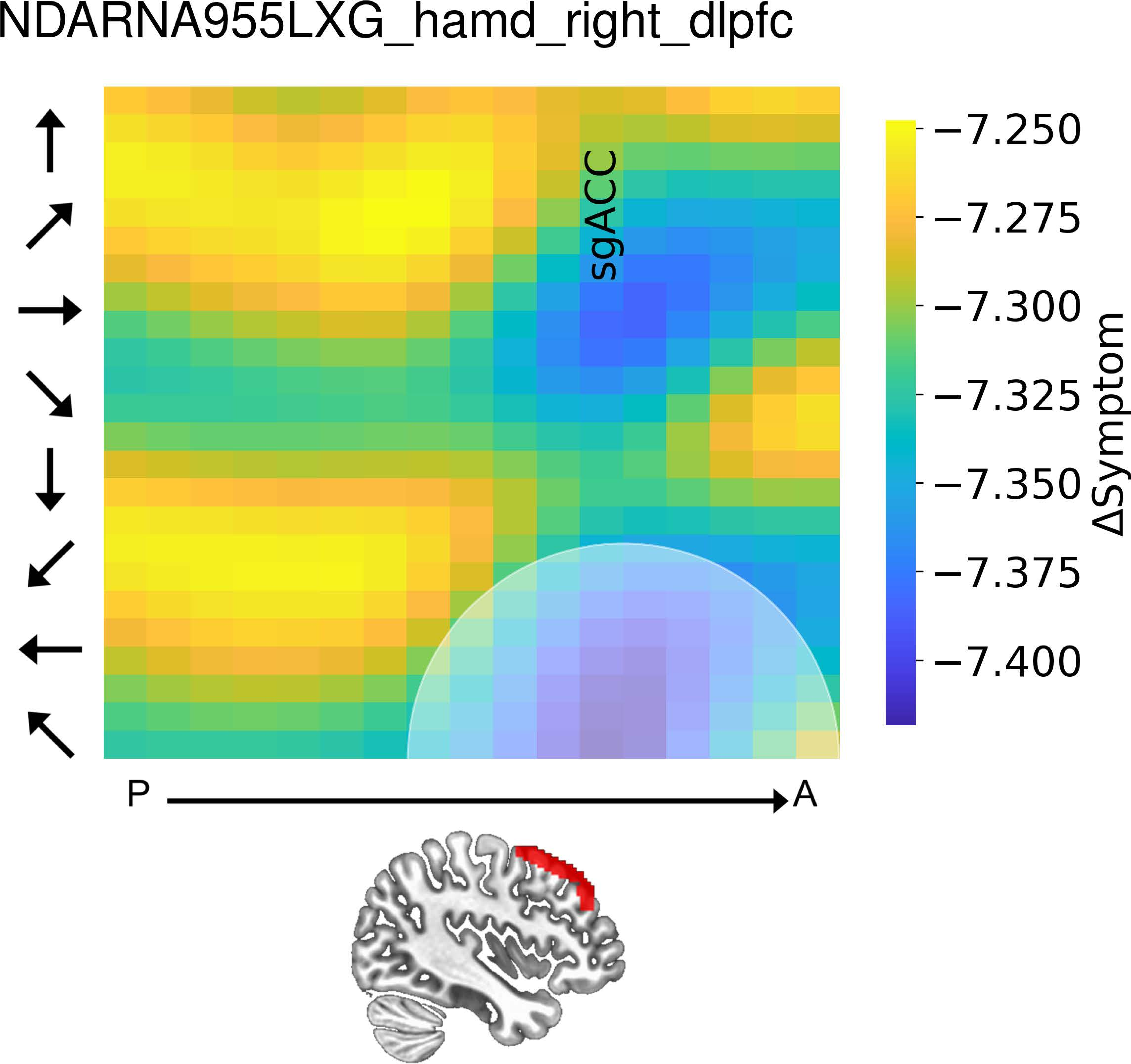

NDARNA955LXG_hamd_right_dlpfc
sgACC
−7.250
−7.275
−7.300
−7.325
−7.350
−7.375
−7.400
ΔSymptom
P
A

NDARNR764NAK_hamd_right_dlpfc

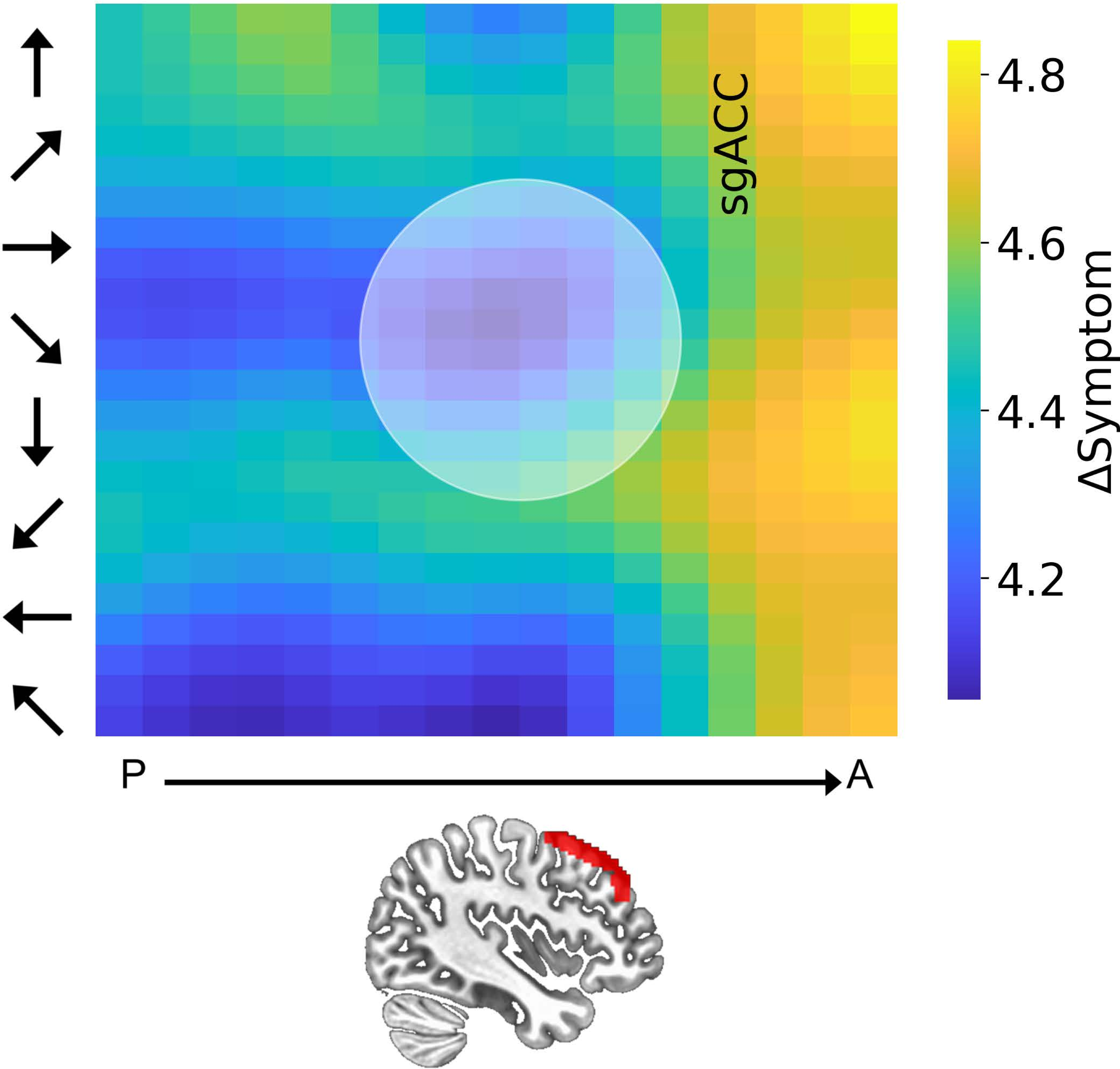

NDARNU180HL3_hamd_right_dlpfc

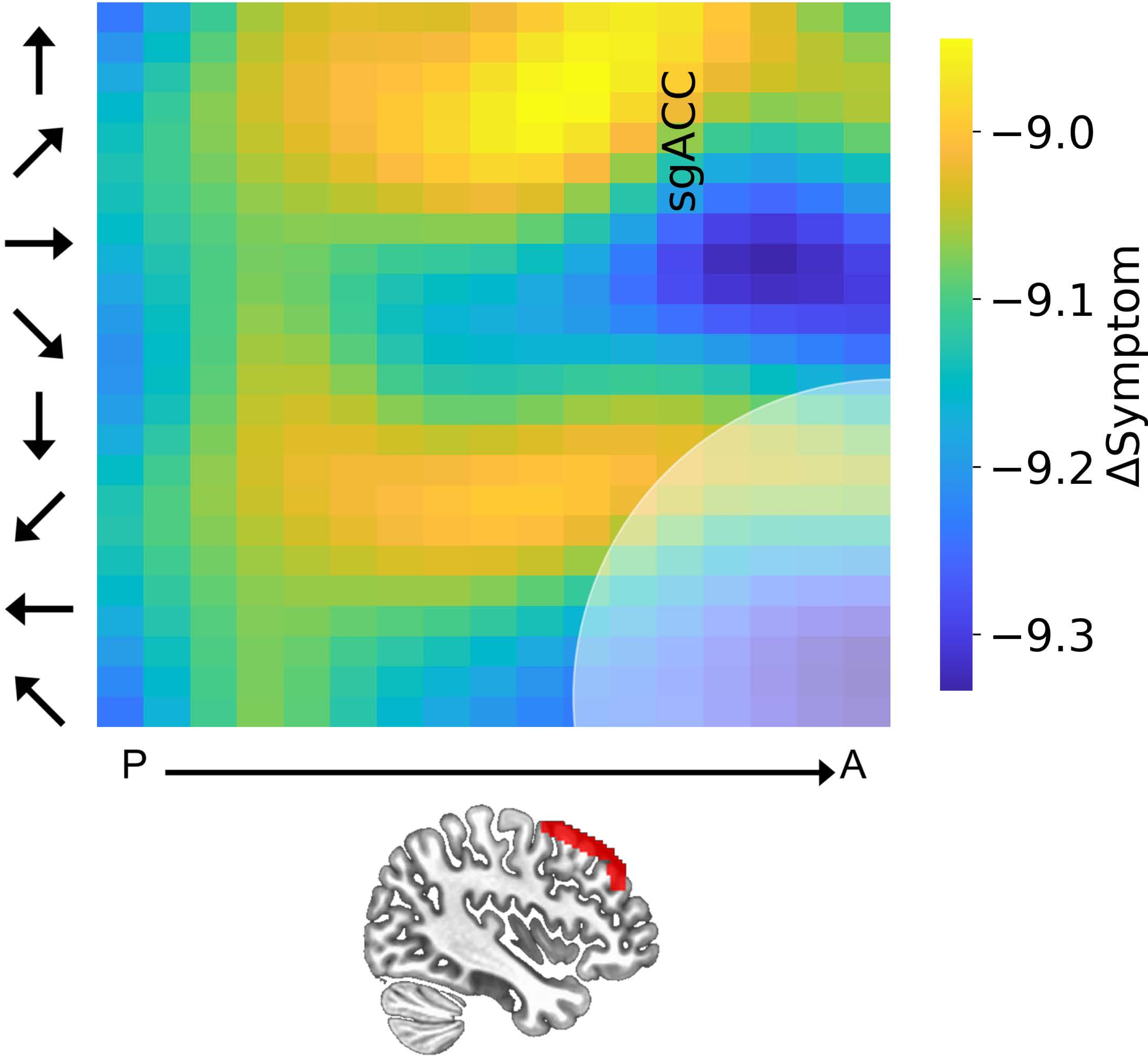

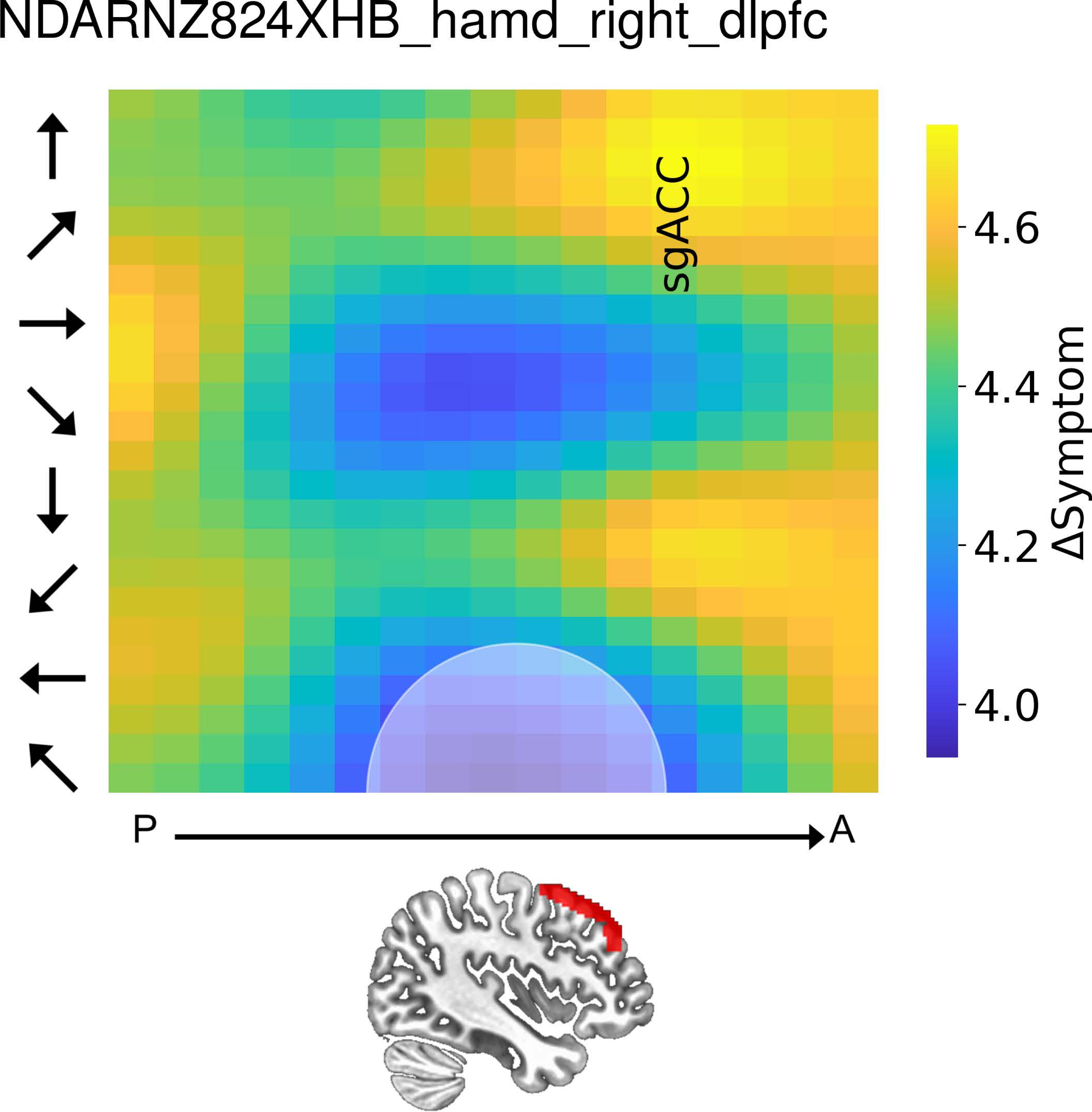
NDARNZ824XHB_hamd_right_dlpfc
sgACC
4.6
4.4
4.2
4.0
ΔSymptom
P
A

NDARPA762UGZ_hamd_right_dlpfc

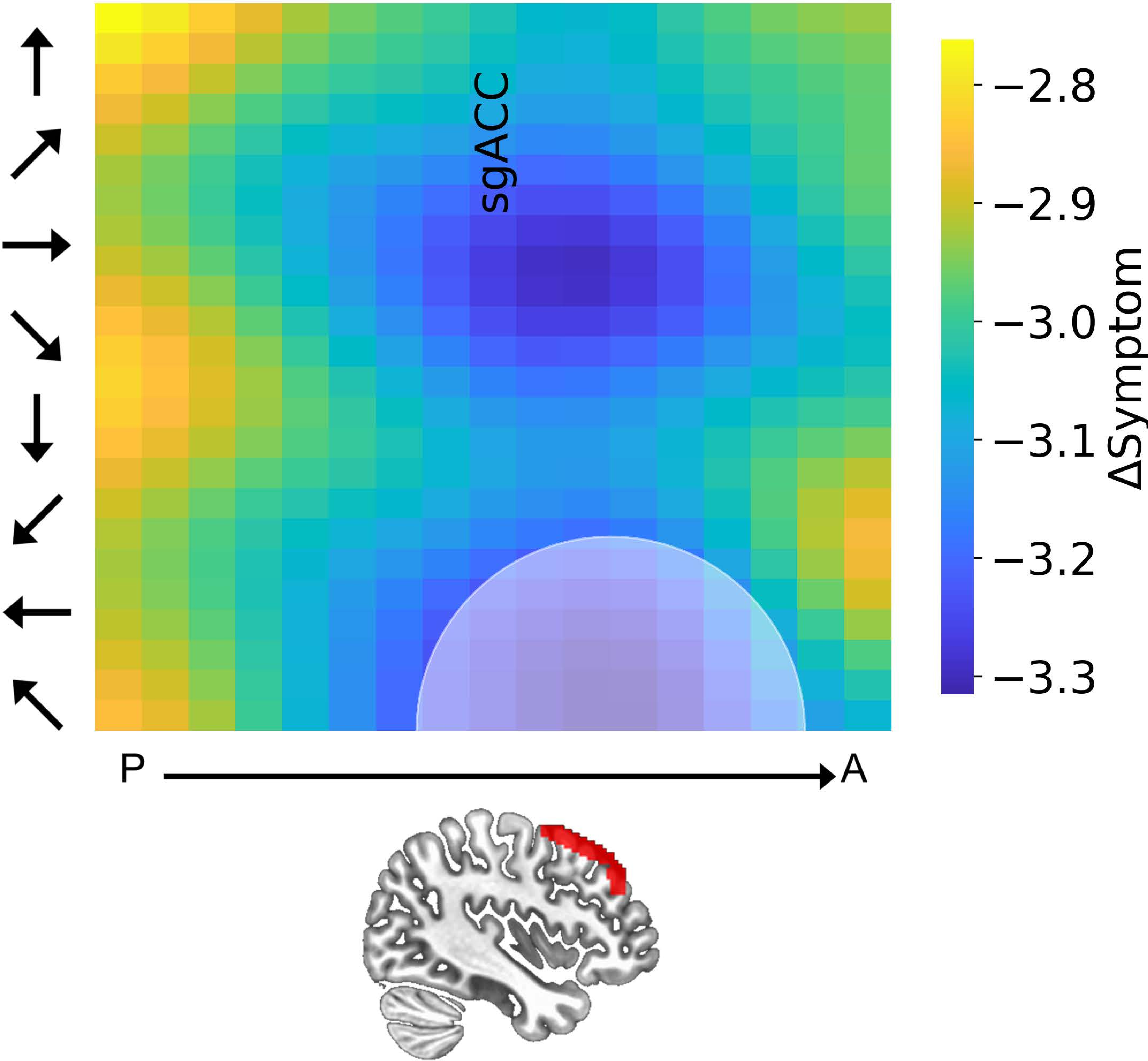

NDARPC197VR3_hamd_right_dlpfc

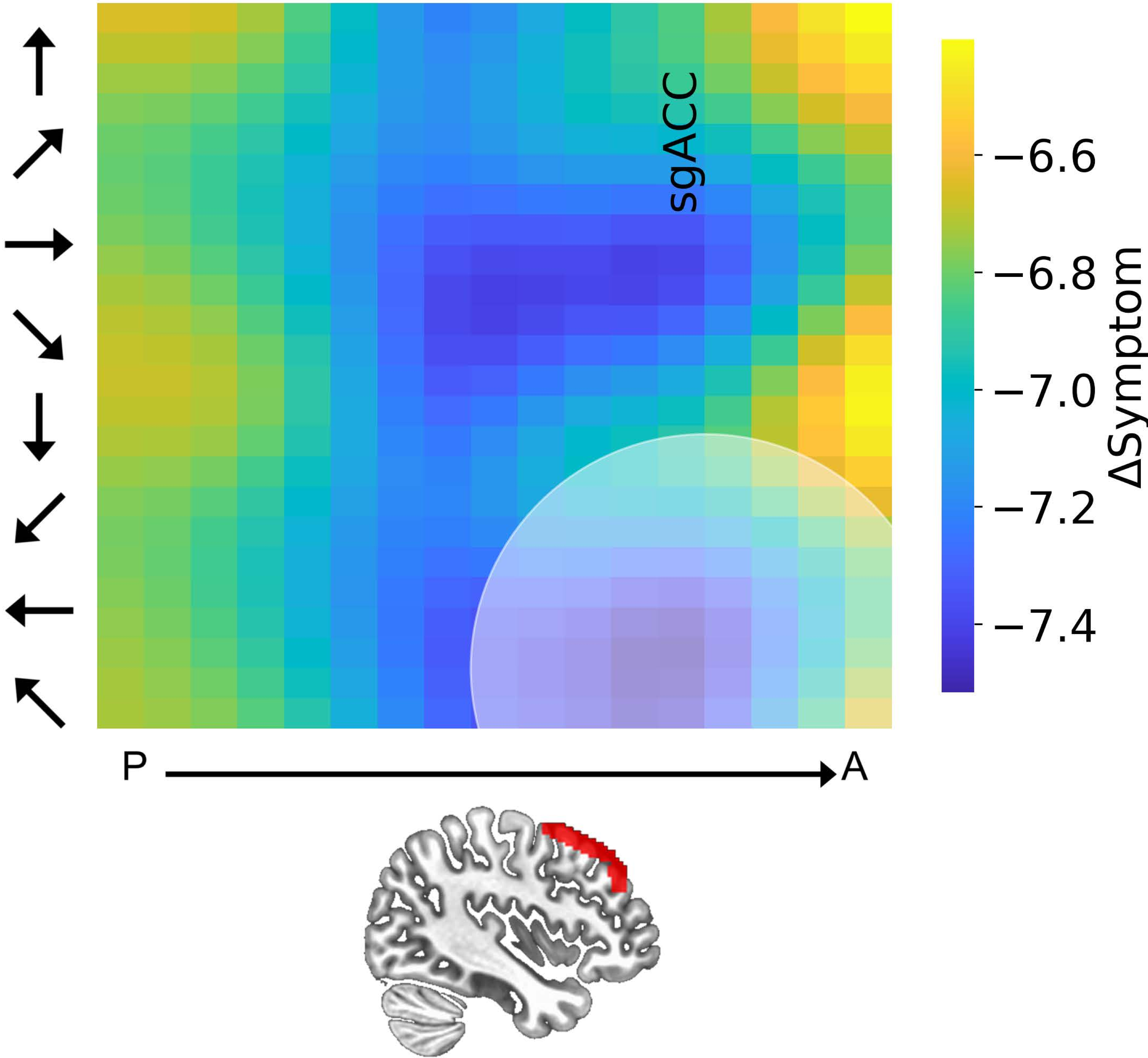

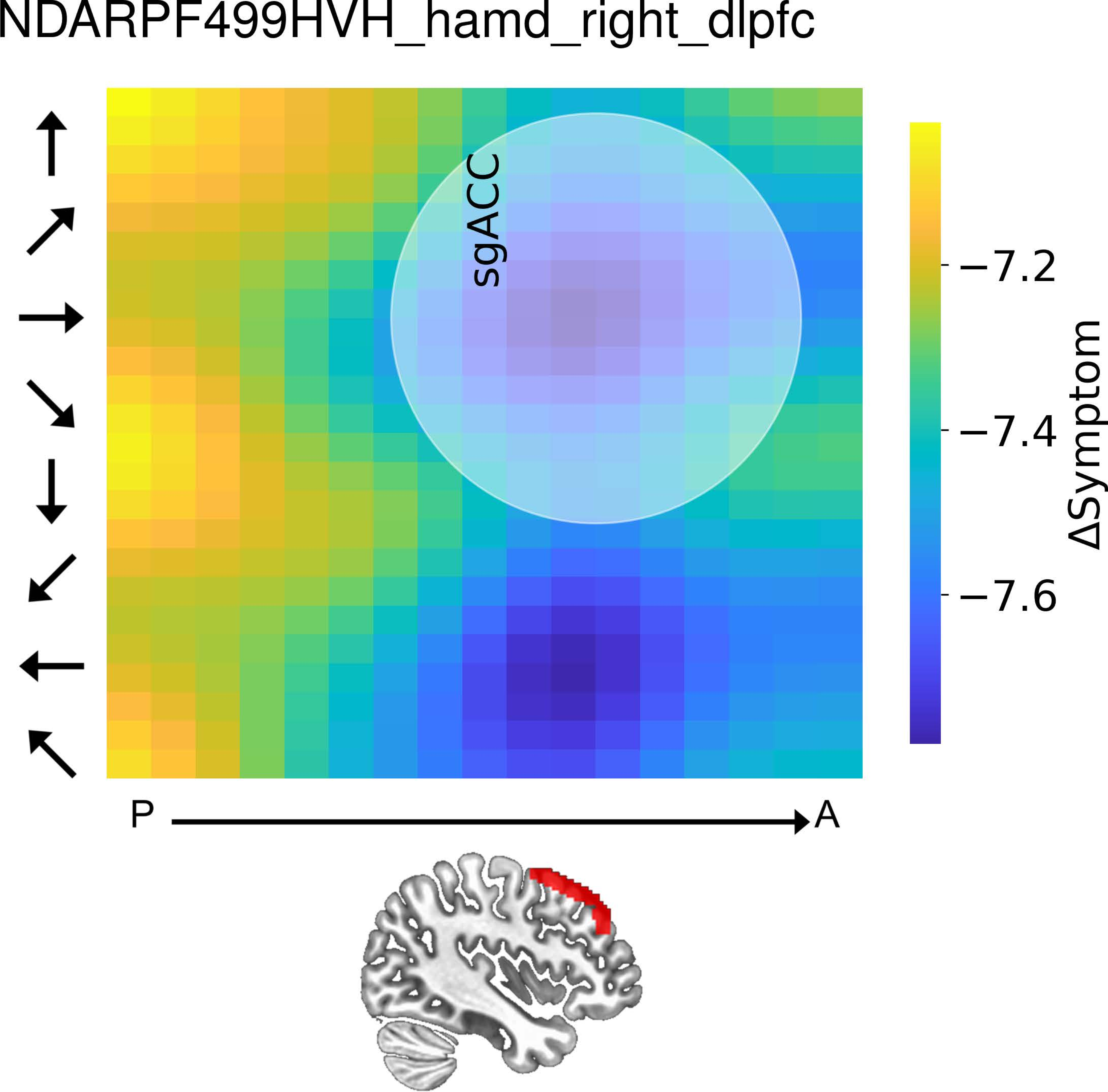
NDARPF499HVH_hamd_right_dlpfc
sgACC
−7.2
−7.4
−7.6
ΔSymptom
P
A

NDARPK811MVW_hamd_right_dlpfc

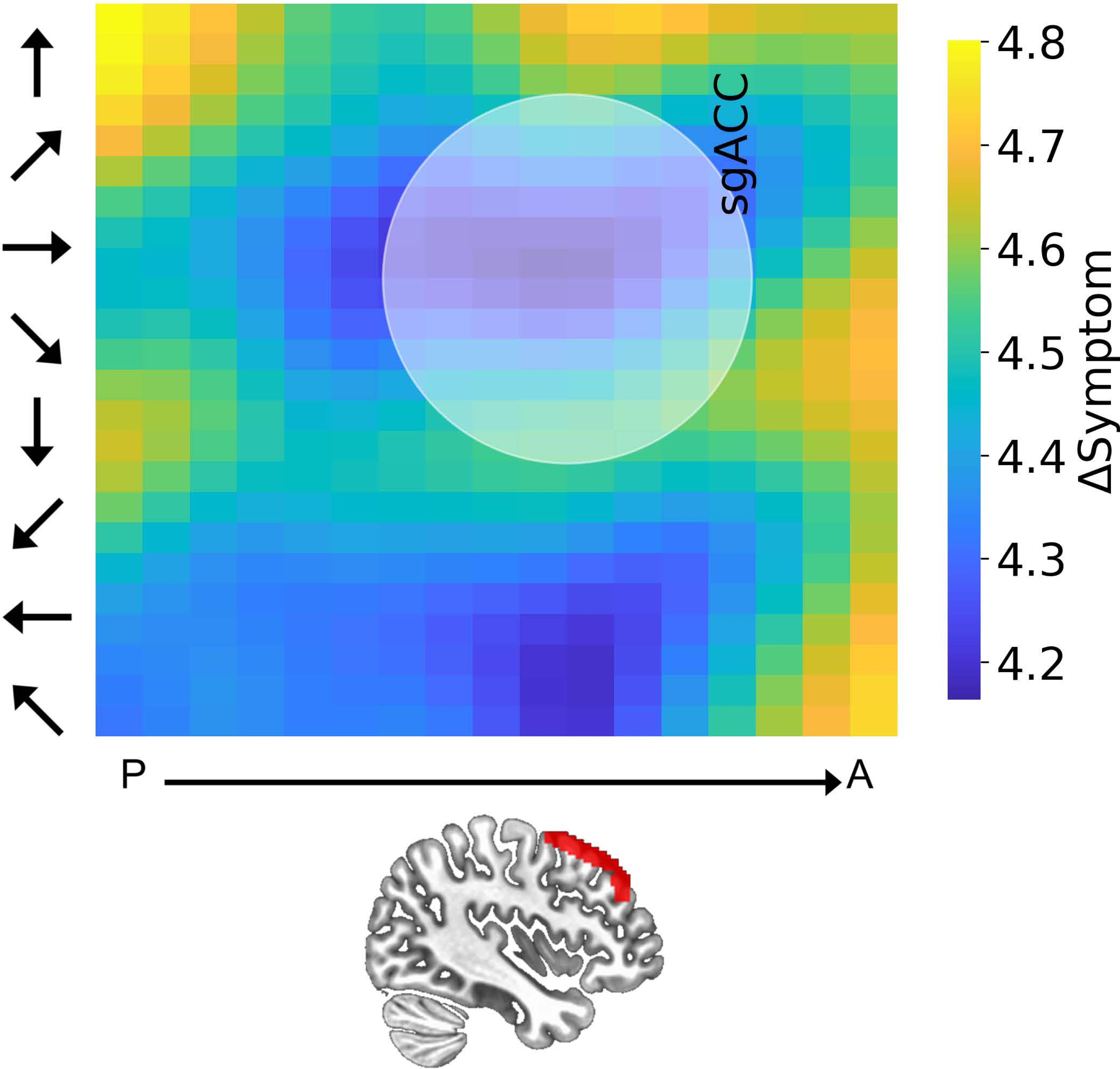

NDARPT040EGR_hamd_right_dlpfc

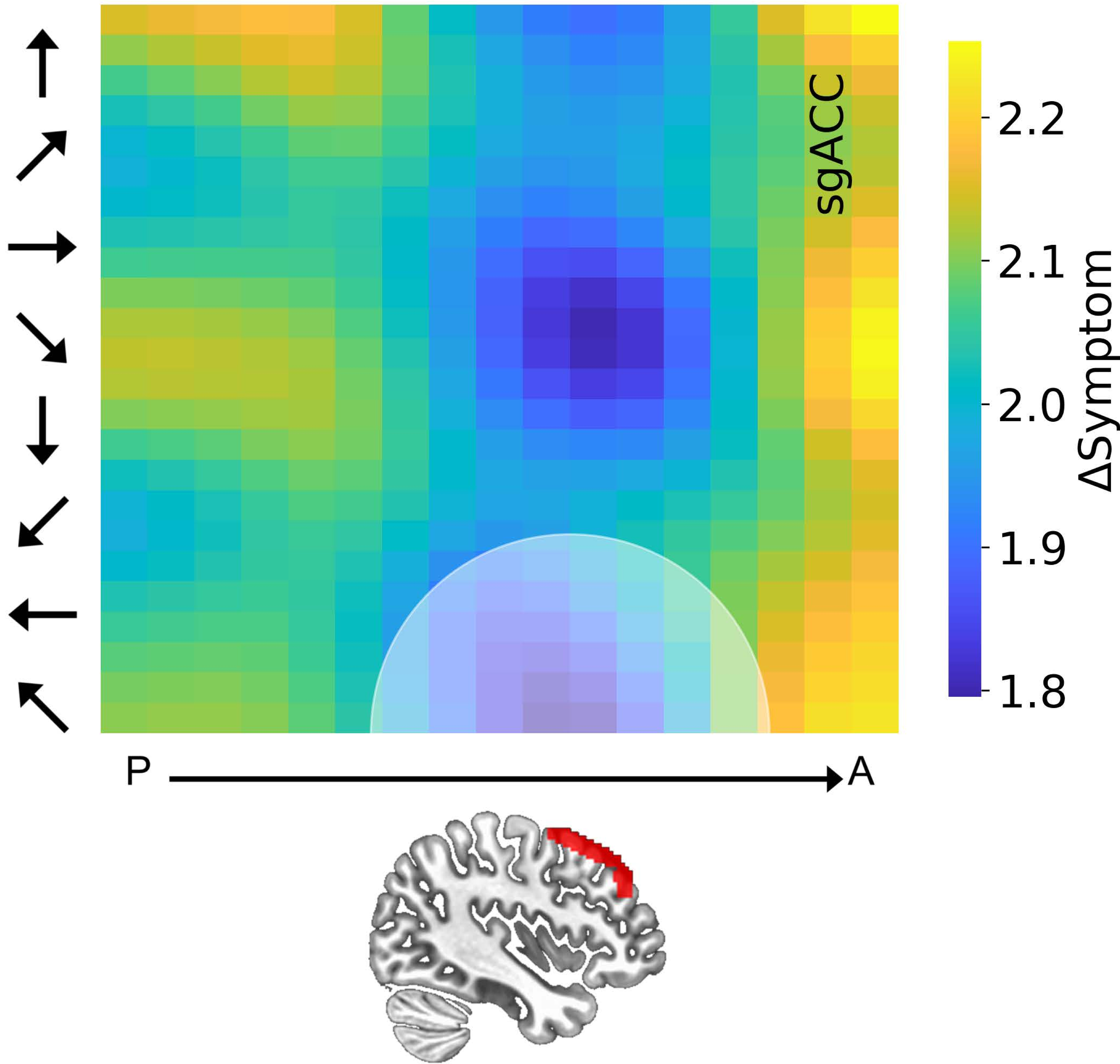

NDARPW706YNA_hamd_right_dlpfc

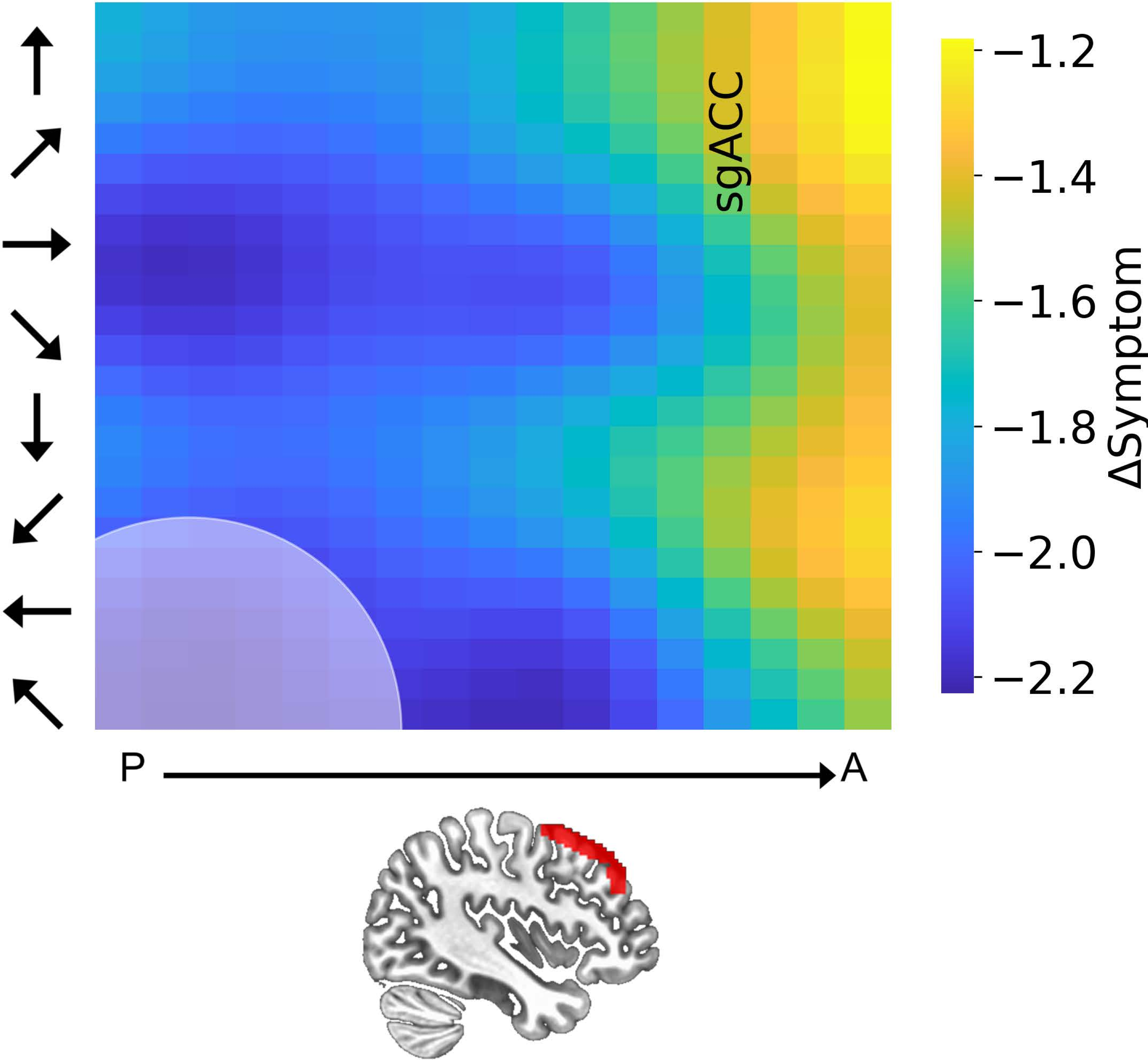

NDARPZ537YY0_hamd_right_dlpfc

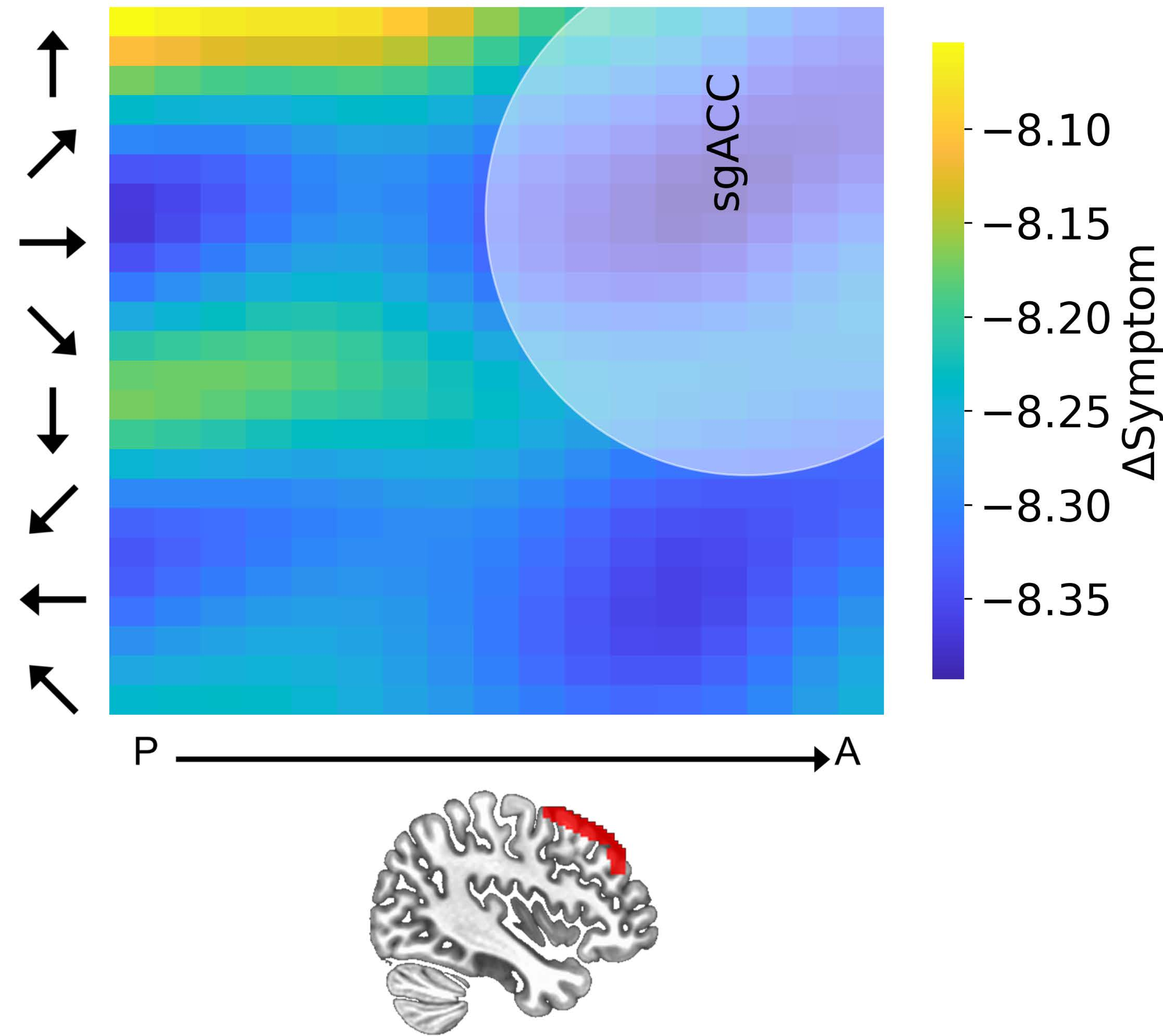

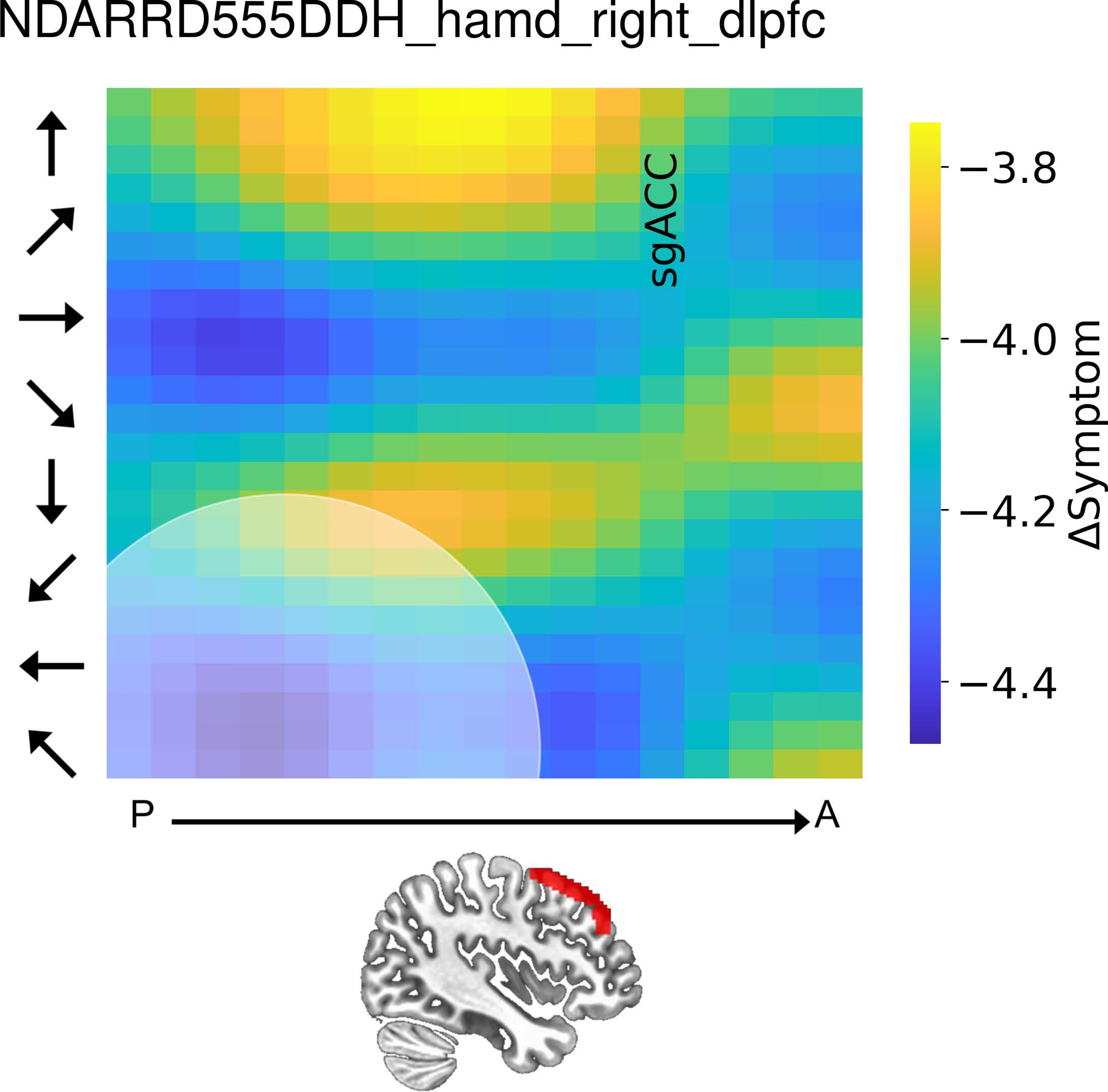
NDARRD555DDH_hamd_right_dlpfc
sgACC
P
A
−3.8
−4.0
−4.2
−4.4
ΔSymptom

NDARRF879TA3_hamd_right_dlpfc

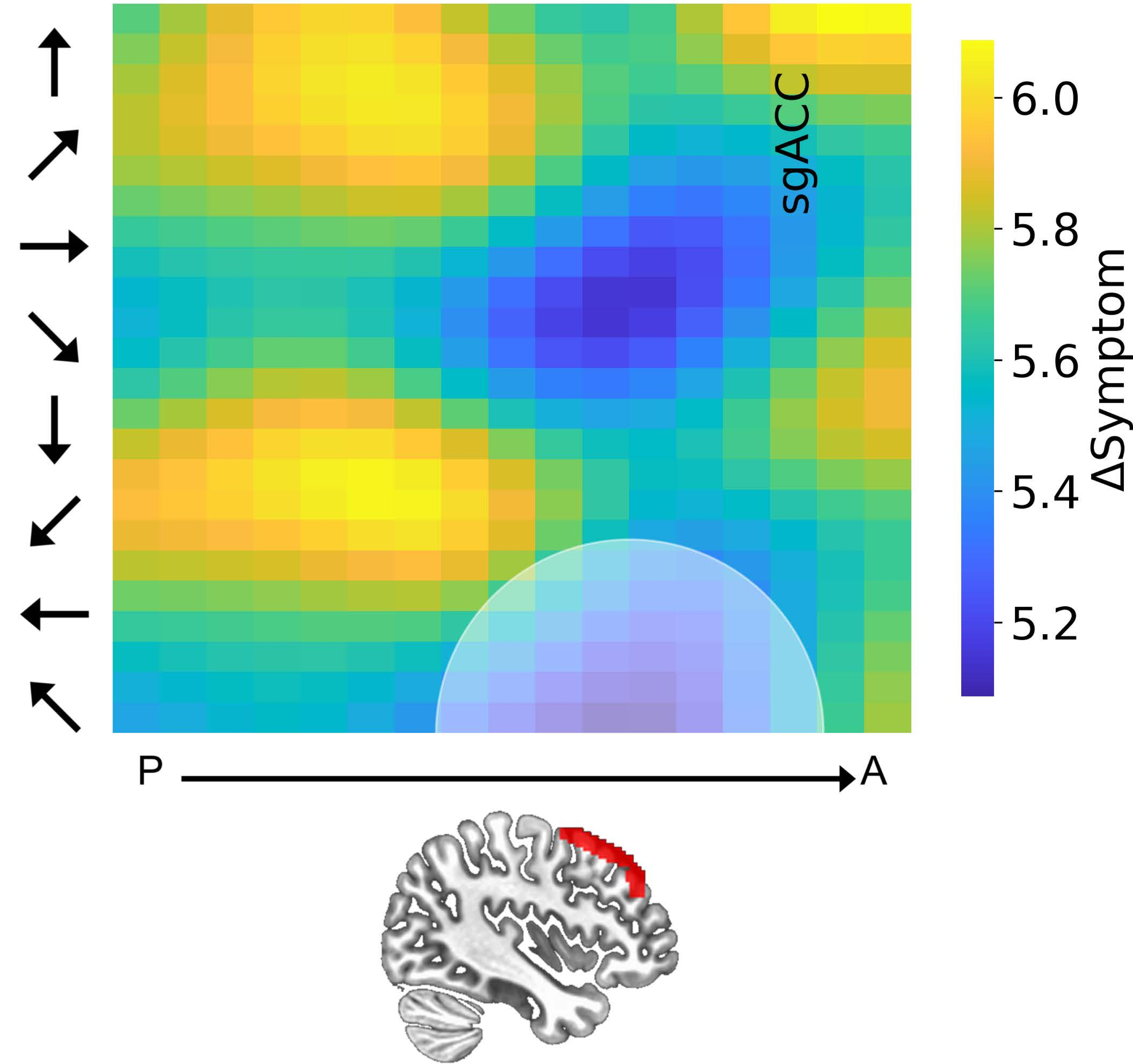

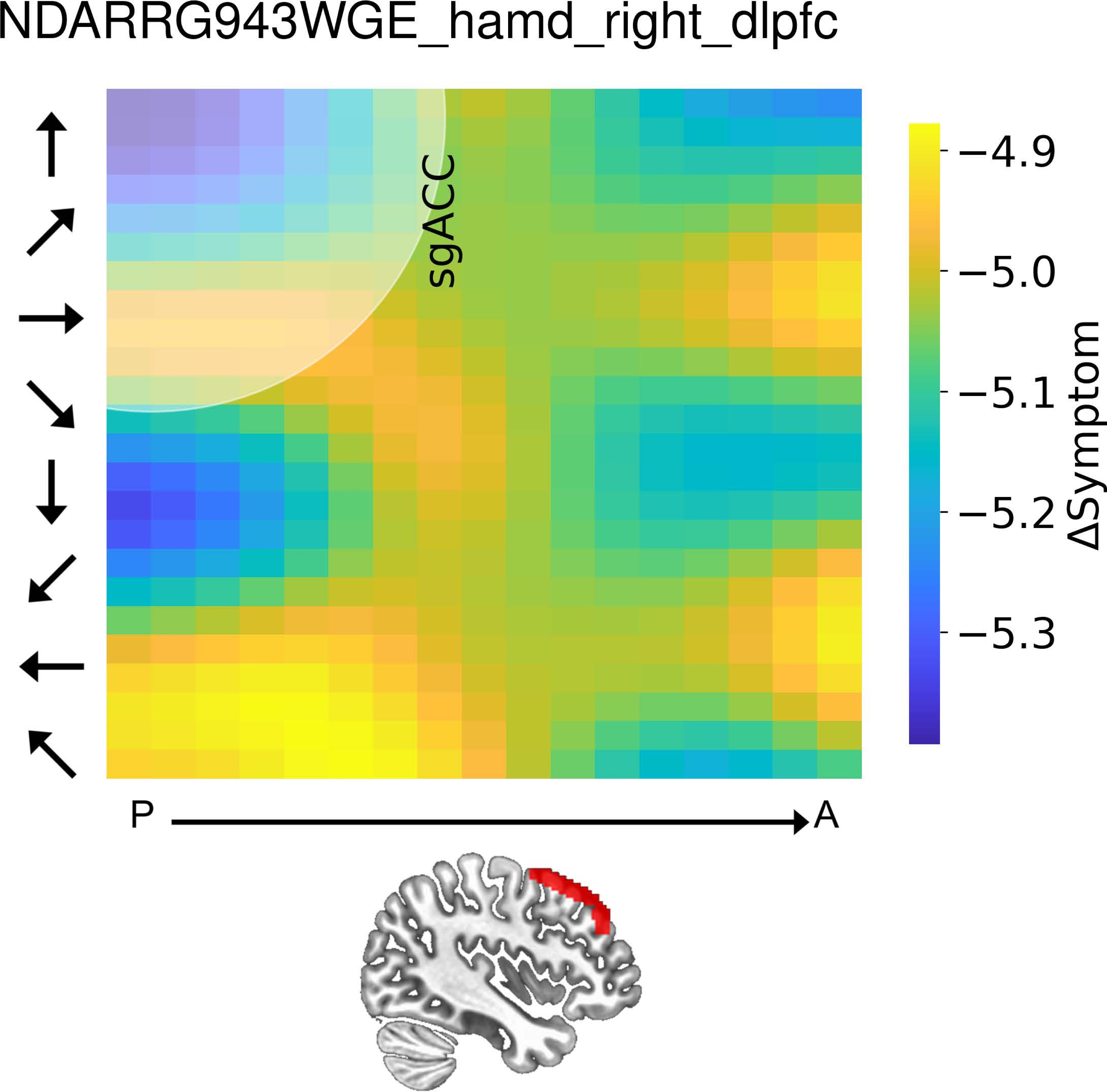
NDARRG943WGE_hamd_right_dlpfc
sgACC
−4.9
−5.0
−5.1
−5.2
−5.3
ΔSymptom
P
A

NDARRM328EP8_hamd_right_dlpfc

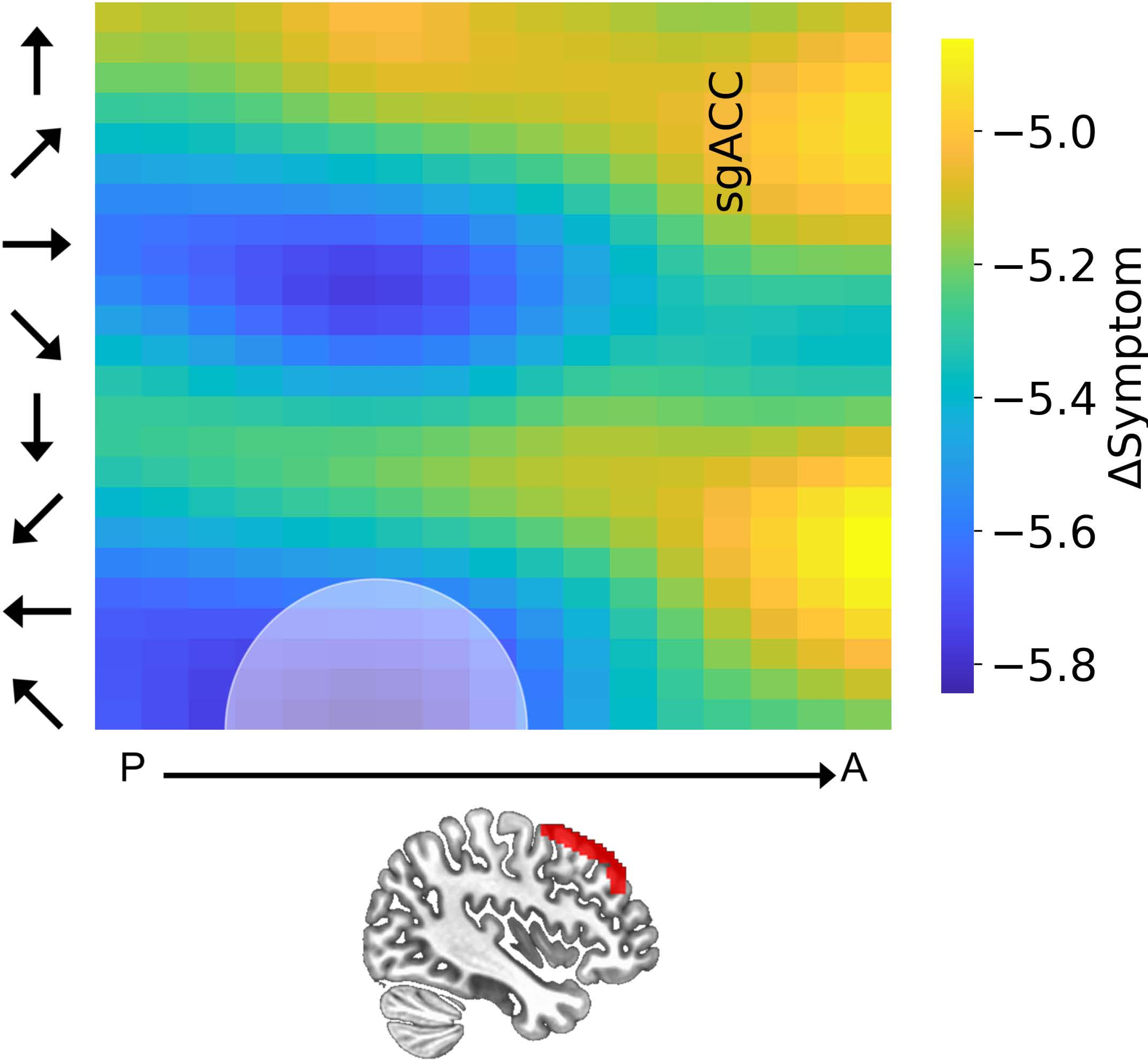

NDARRZ612NKG_hamd_right_dlpfc

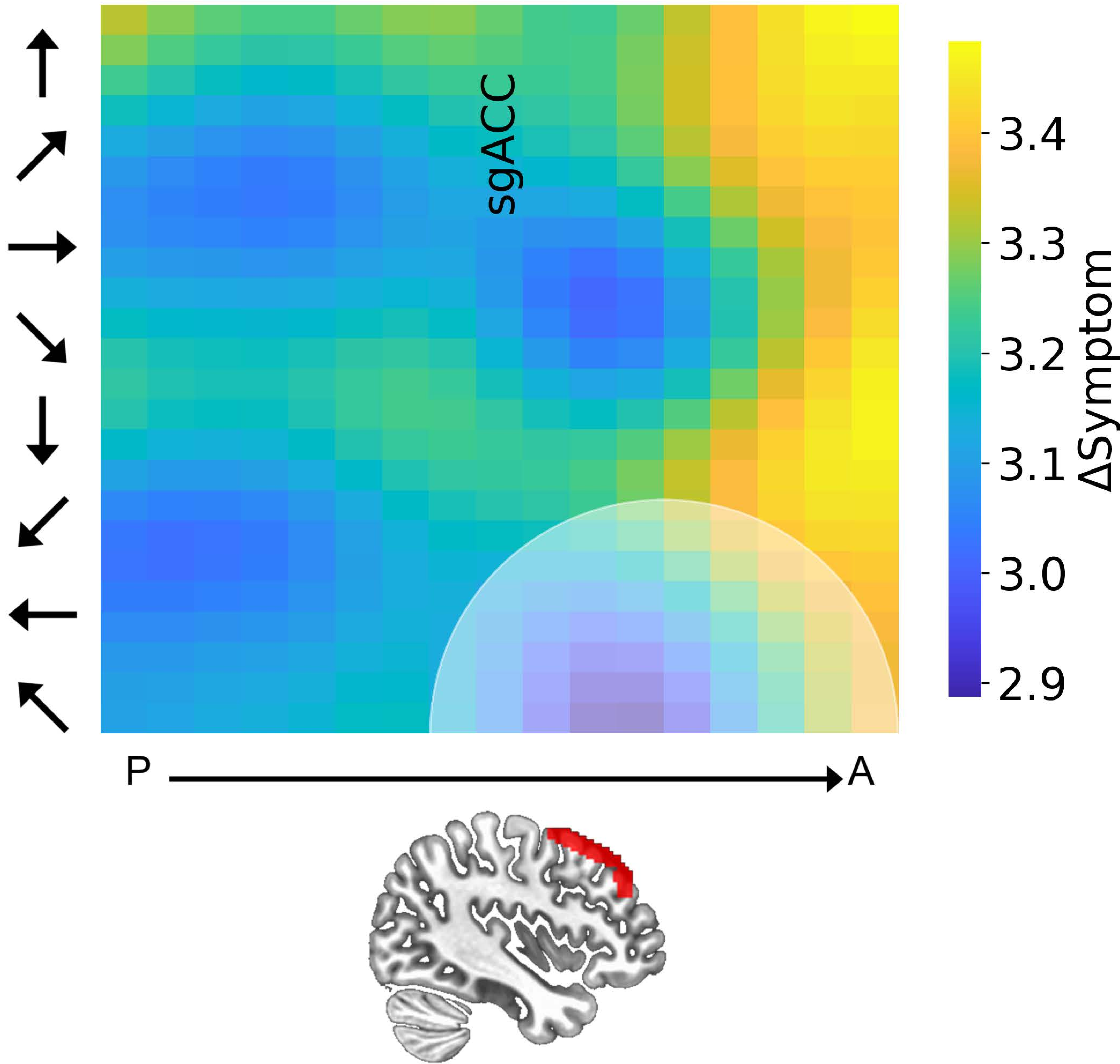

NDARTH509MNV_hamd_right_dlpfc

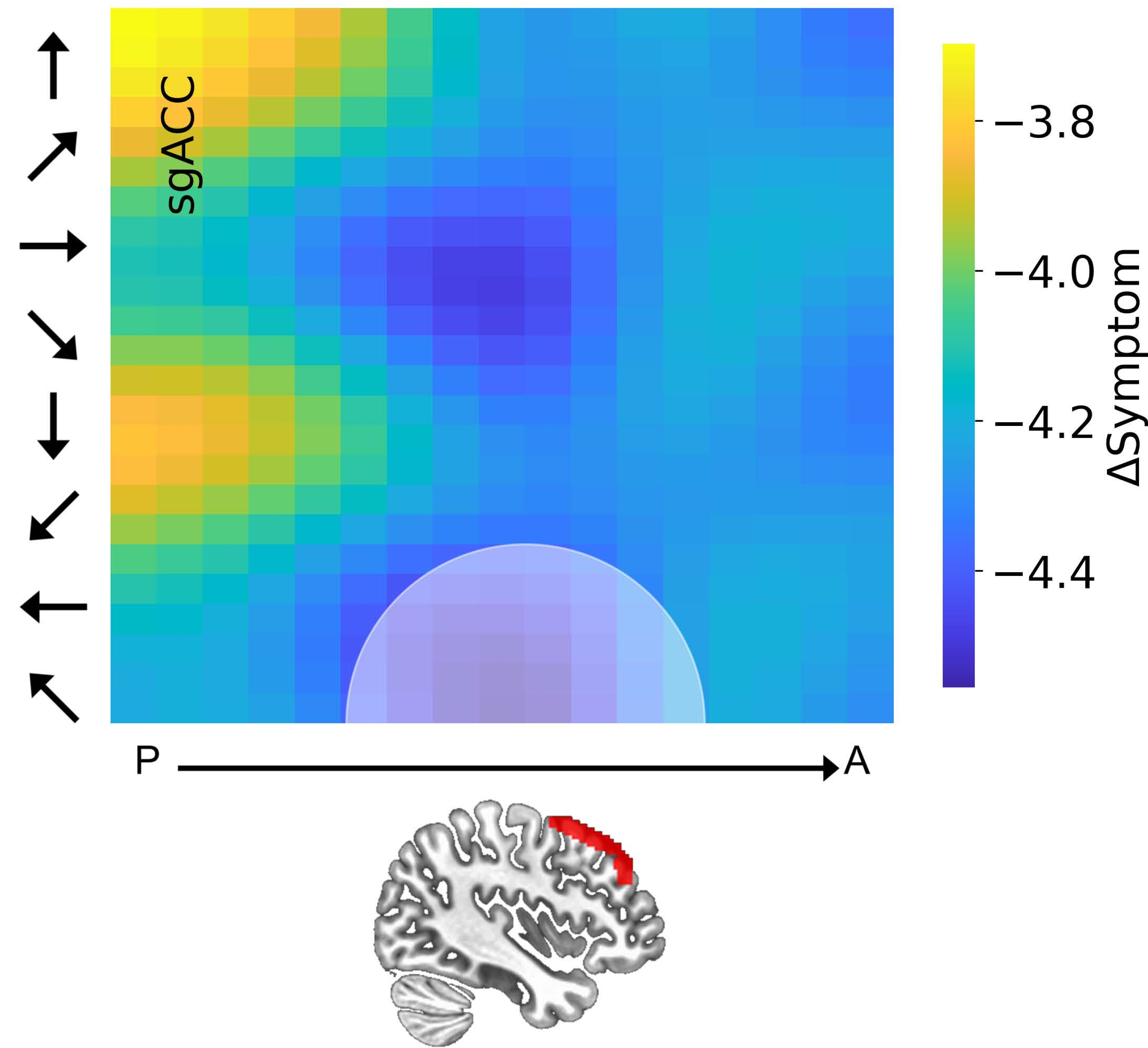

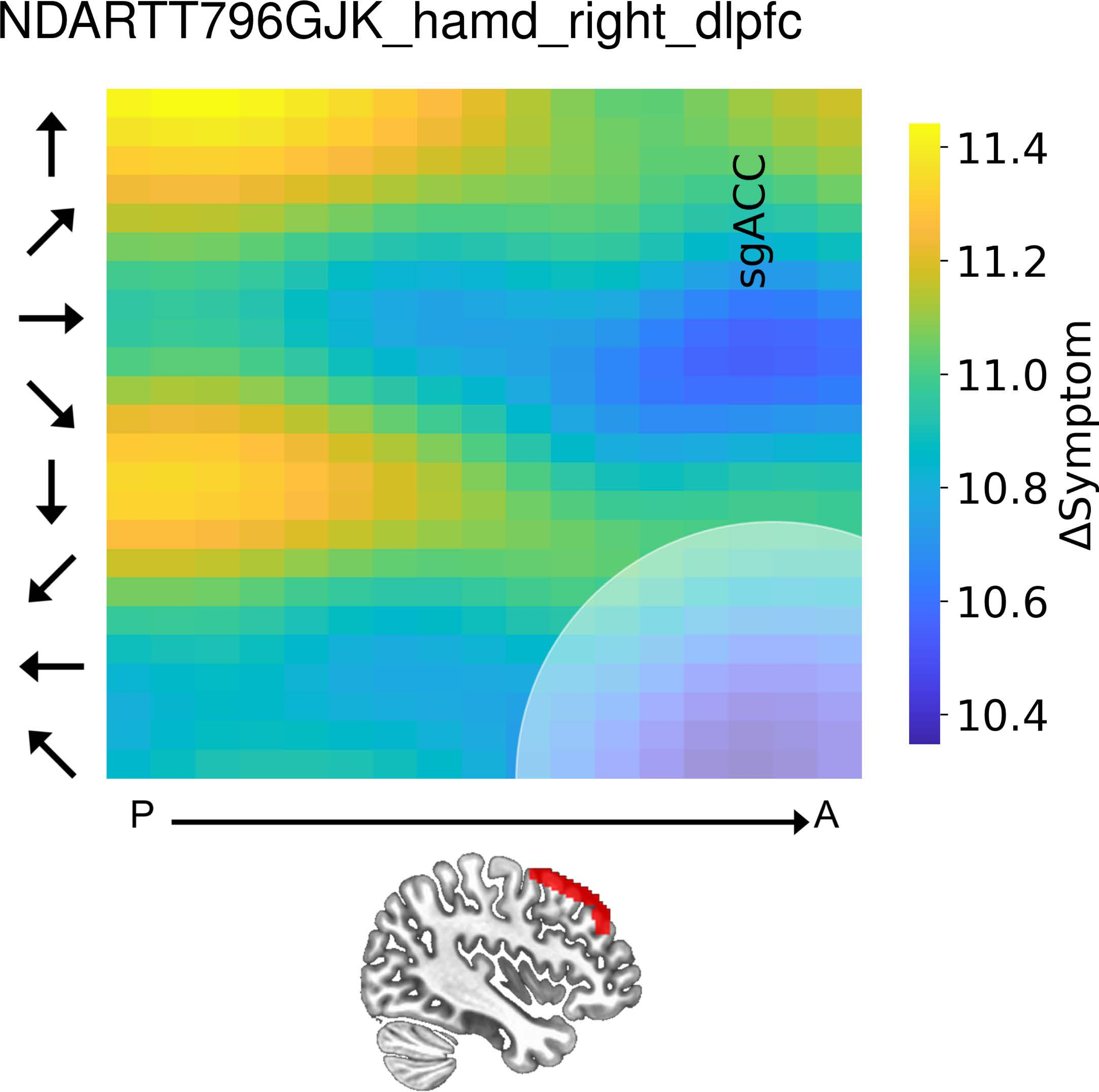

NDARTT796GJK_hamd_right_dlpfc
sgACC
P
A
ΔSymptom
11.4
11.2
11.0
10.8
10.6
10.4

NDARTZ590DYE_hamd_right_dlpfc

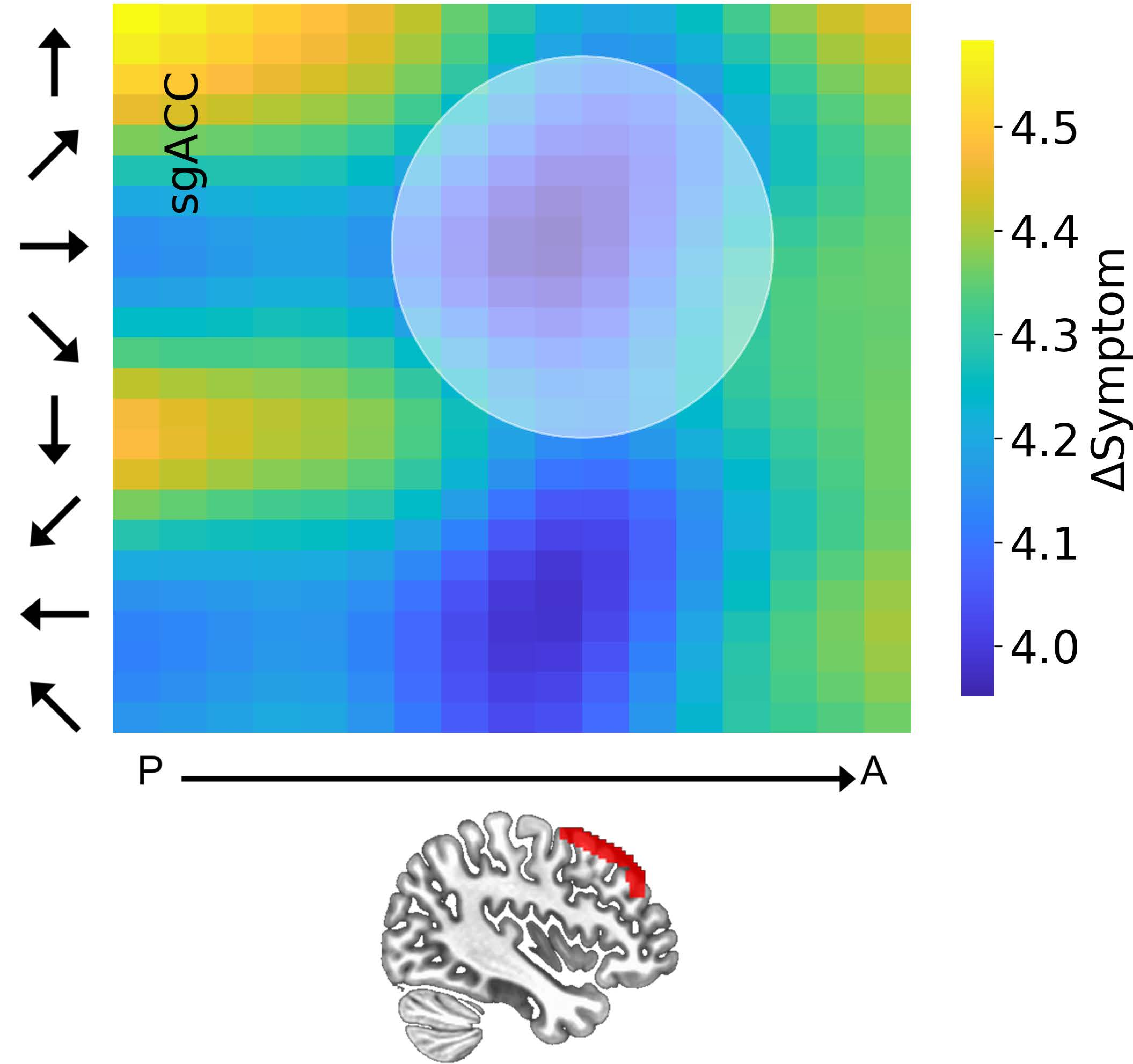

NDARUJ223ZPN_hamd_right_dlpfc

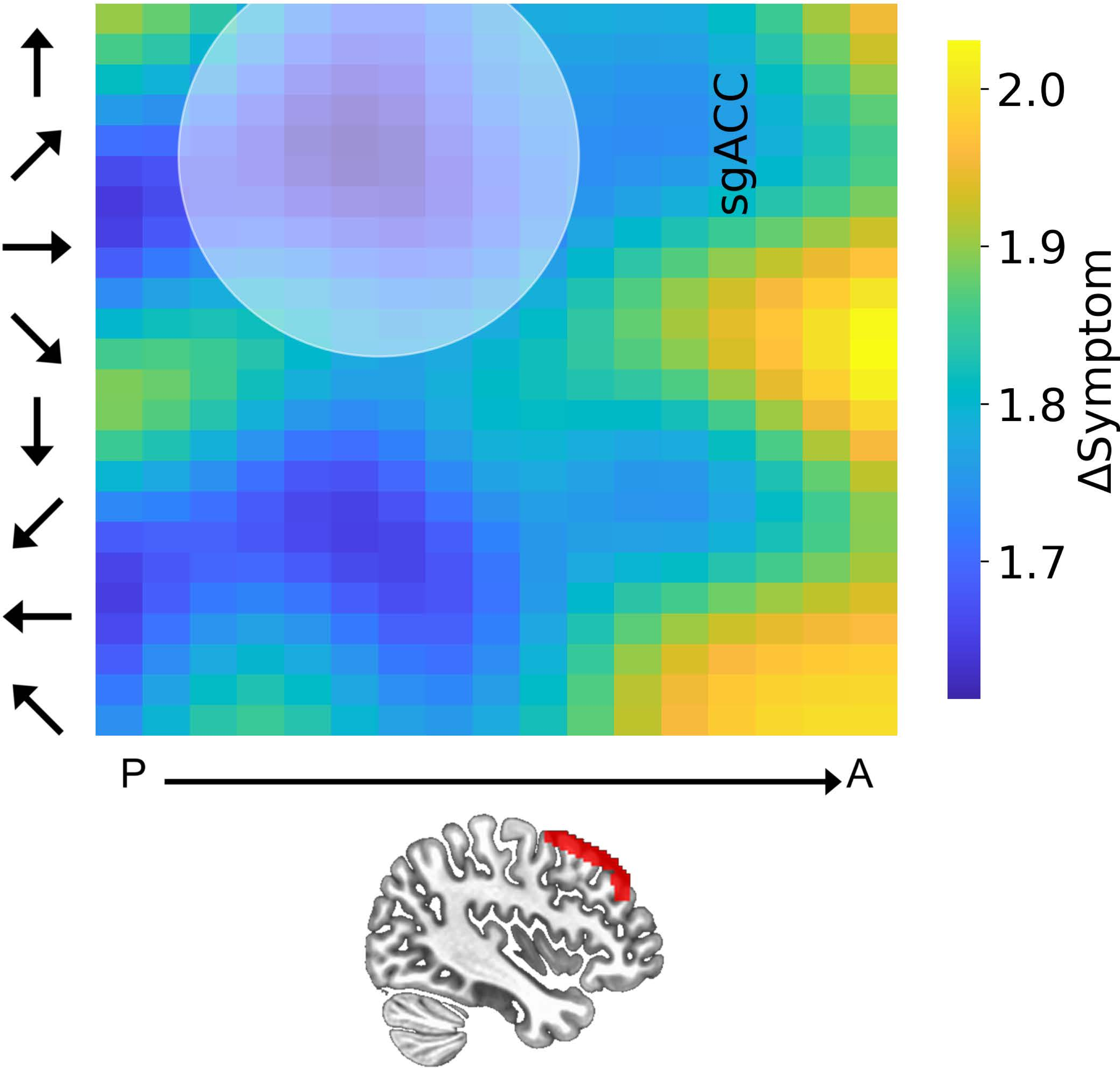

NDARUM818JC1_hamd_right_dlpfc

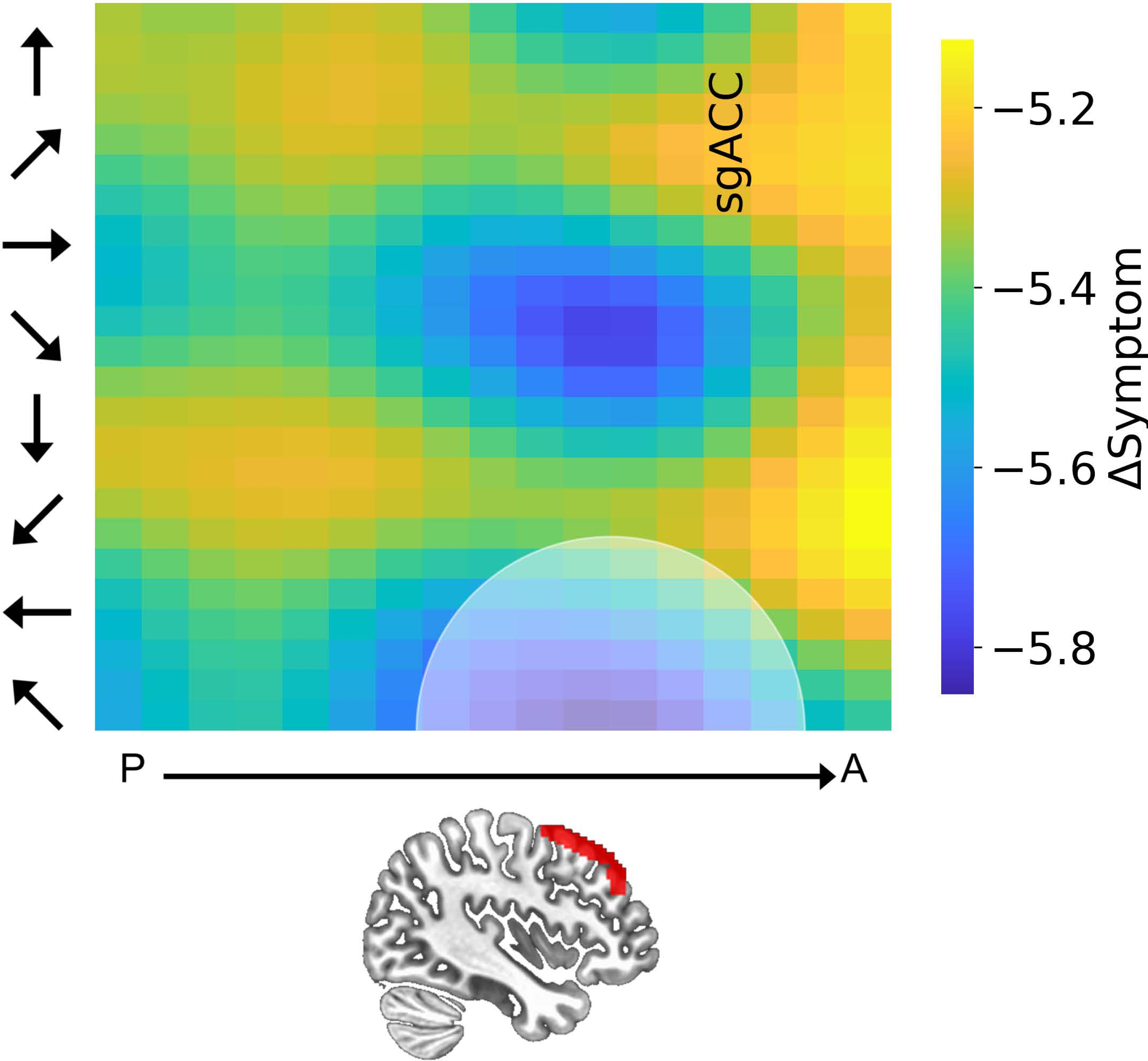

NDARUT852KUX_hamd_right_dlpfc

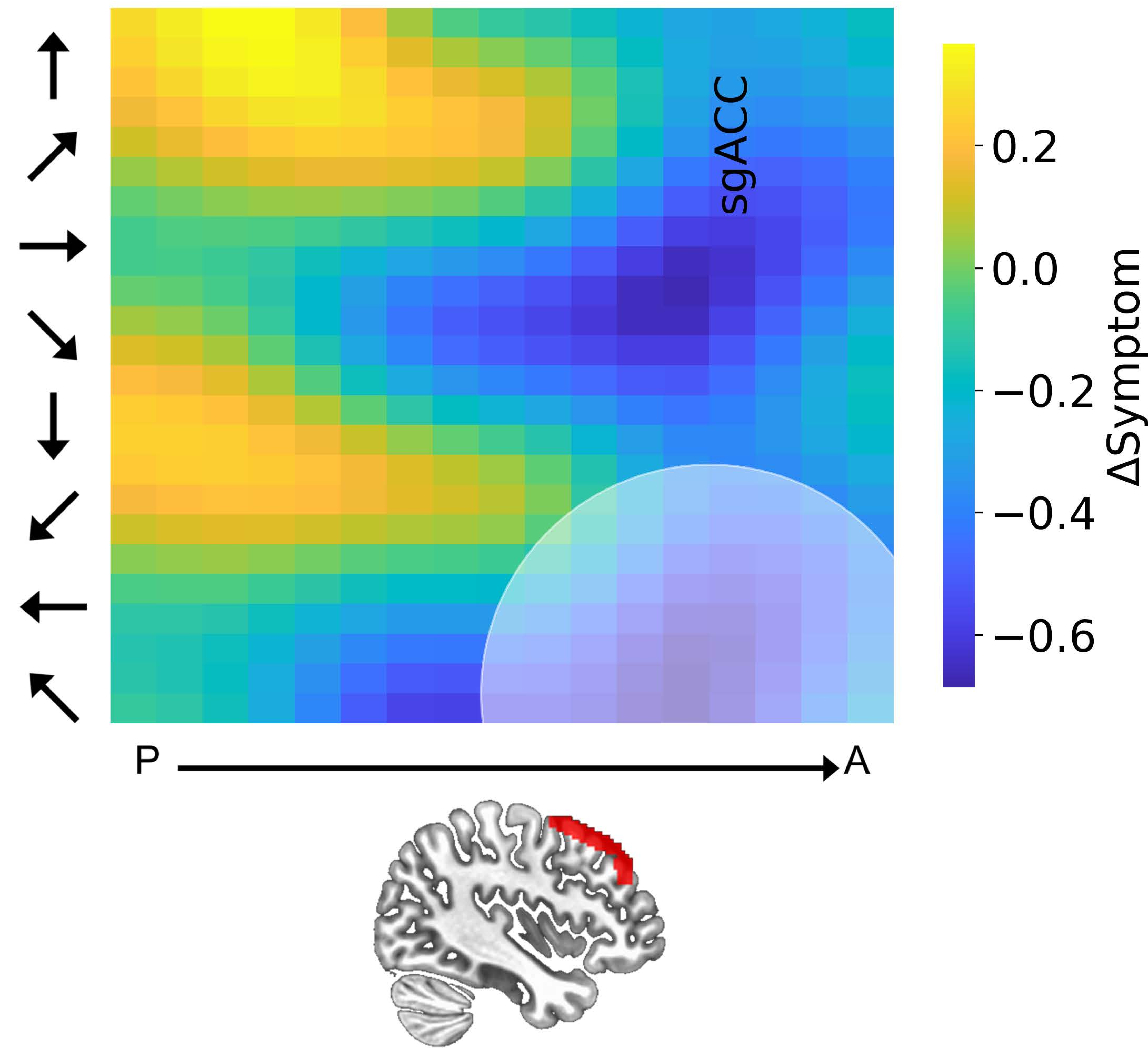

NDARVE642FAN_hamd_right_dlpfc

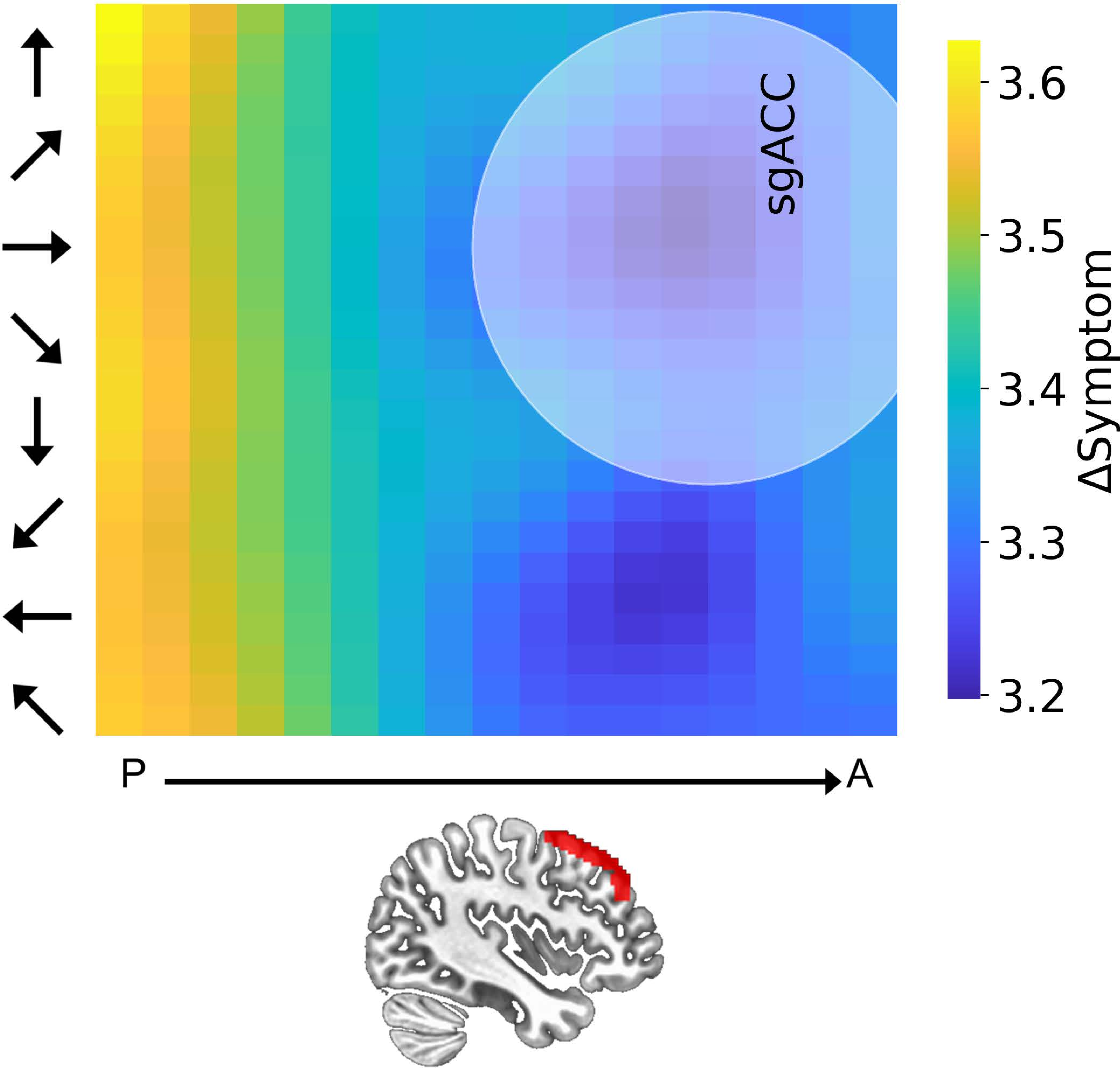

NDARVL144YVF_hamd_right_dlpfc

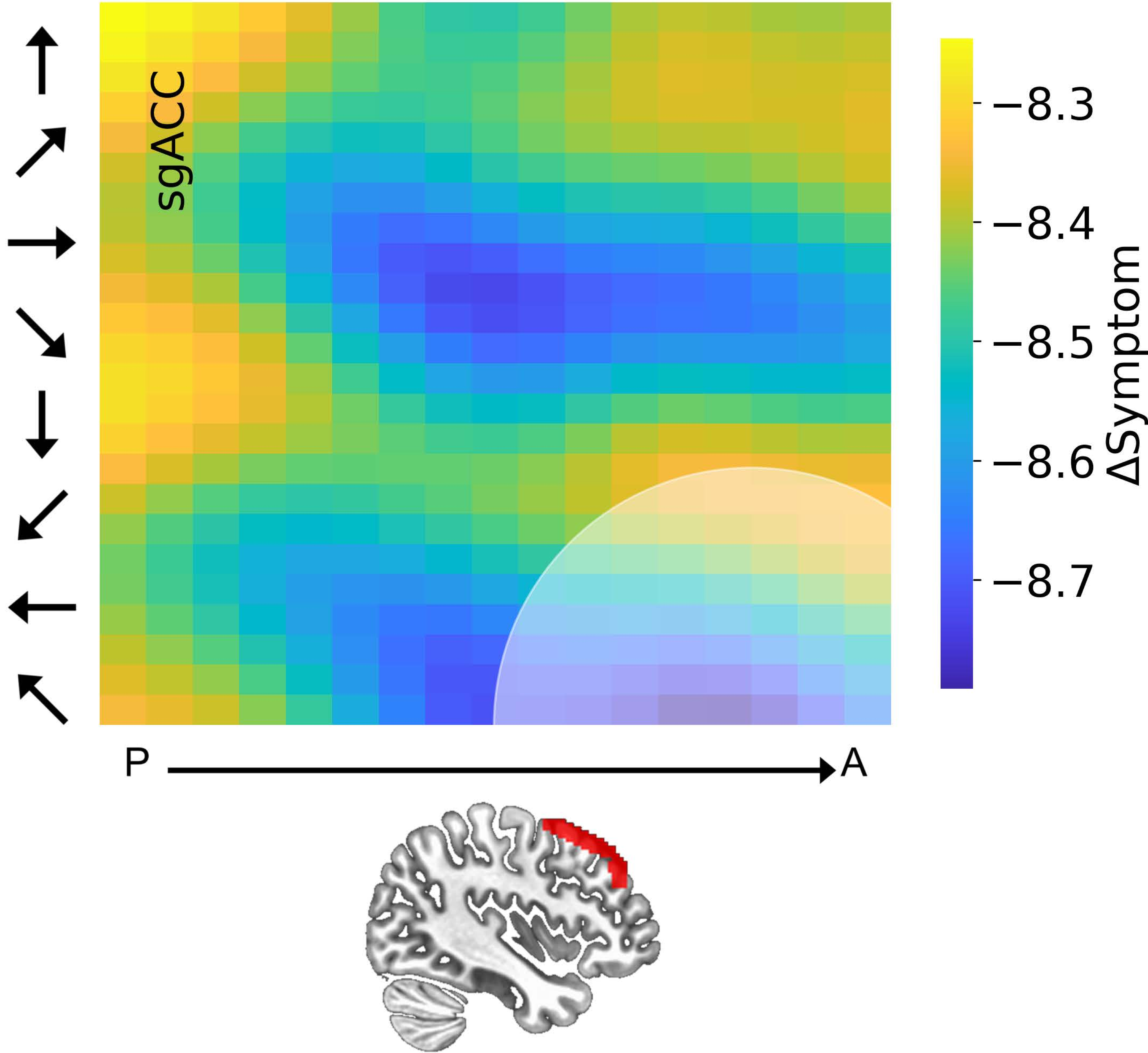

NDARWH523DTV_hamd_right_dlpfc

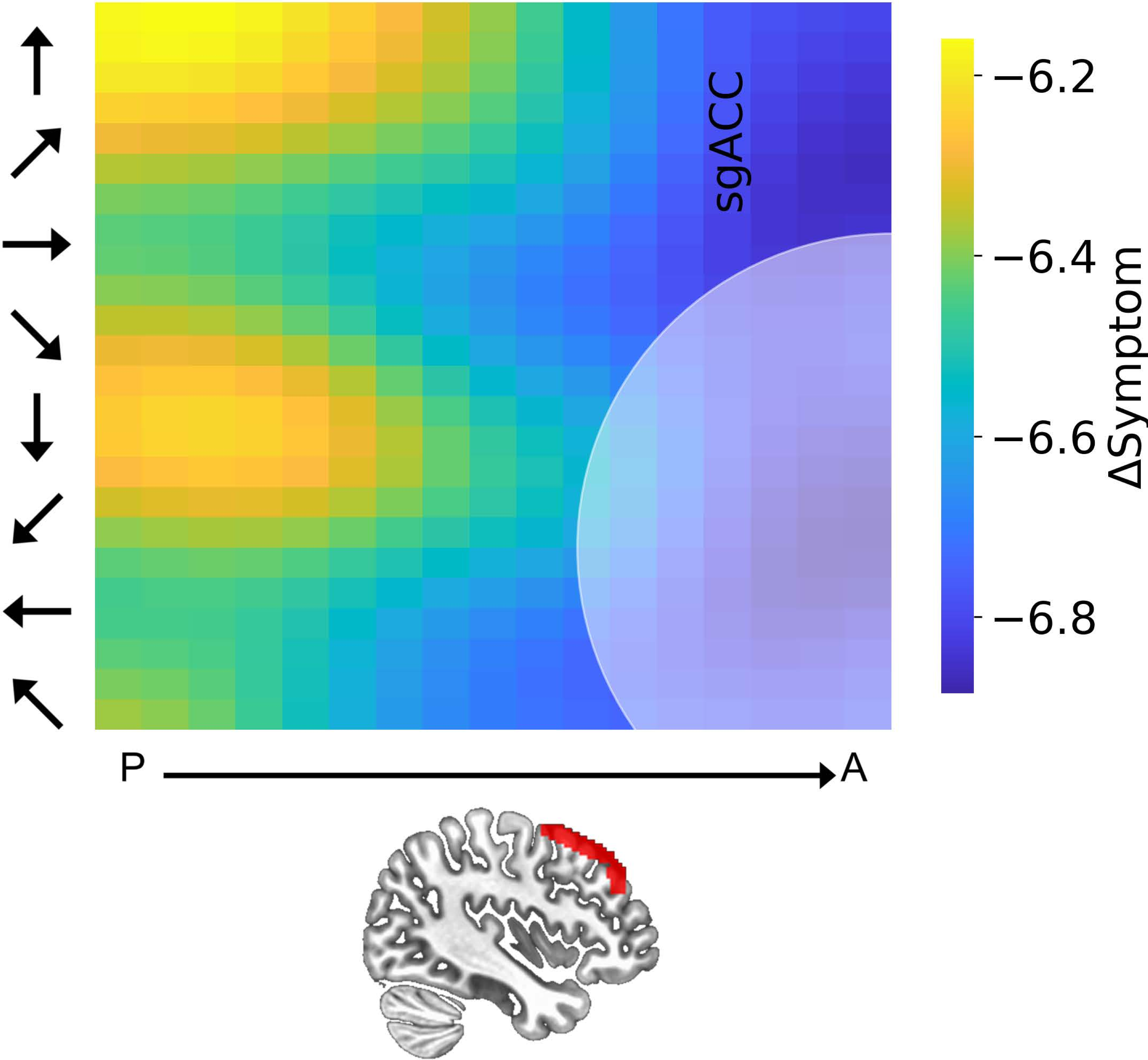

NDARWJ818VXA_hamd_right_dlpfc

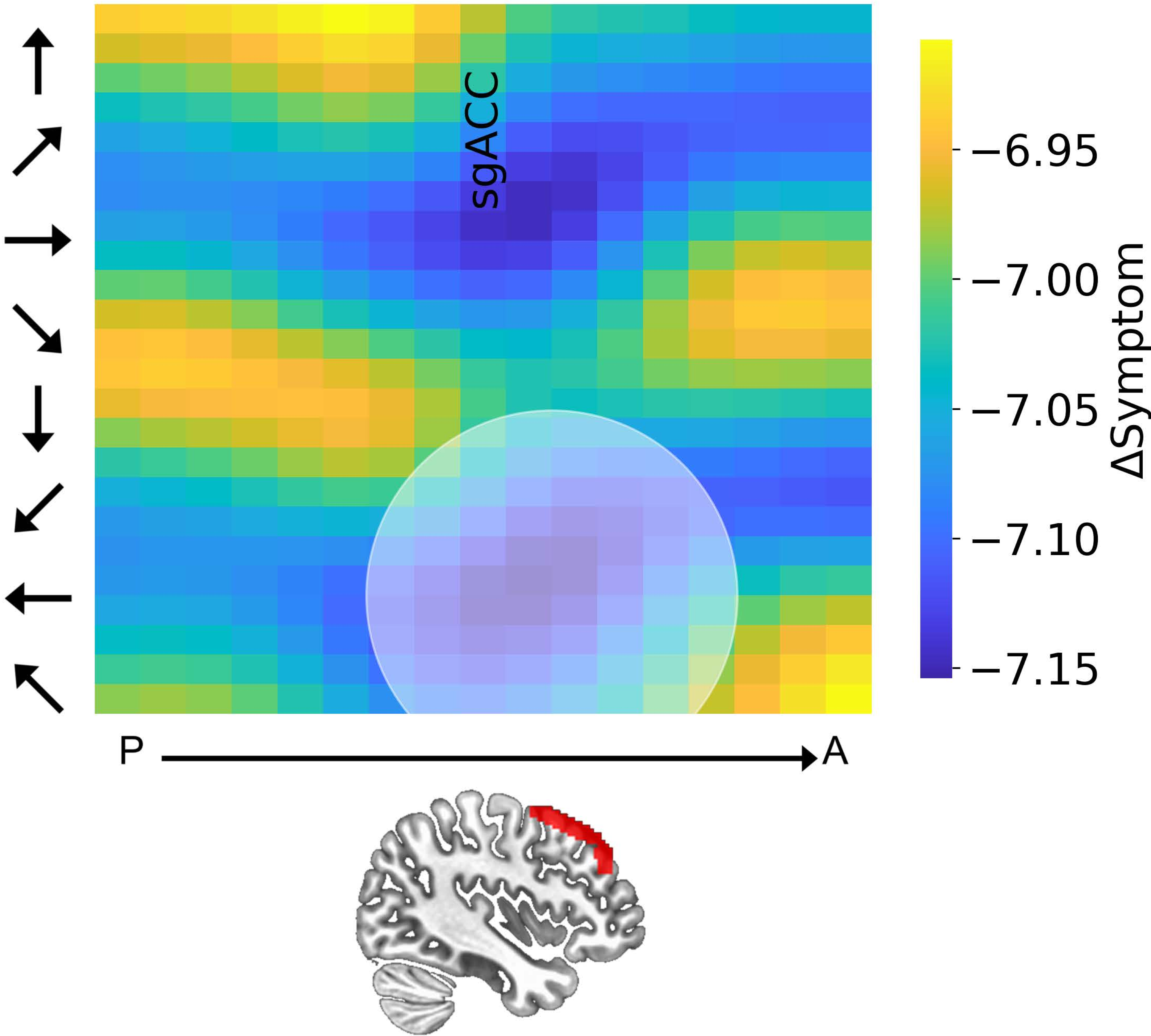

NDARWK081NTV_hamd_right_dlpfc

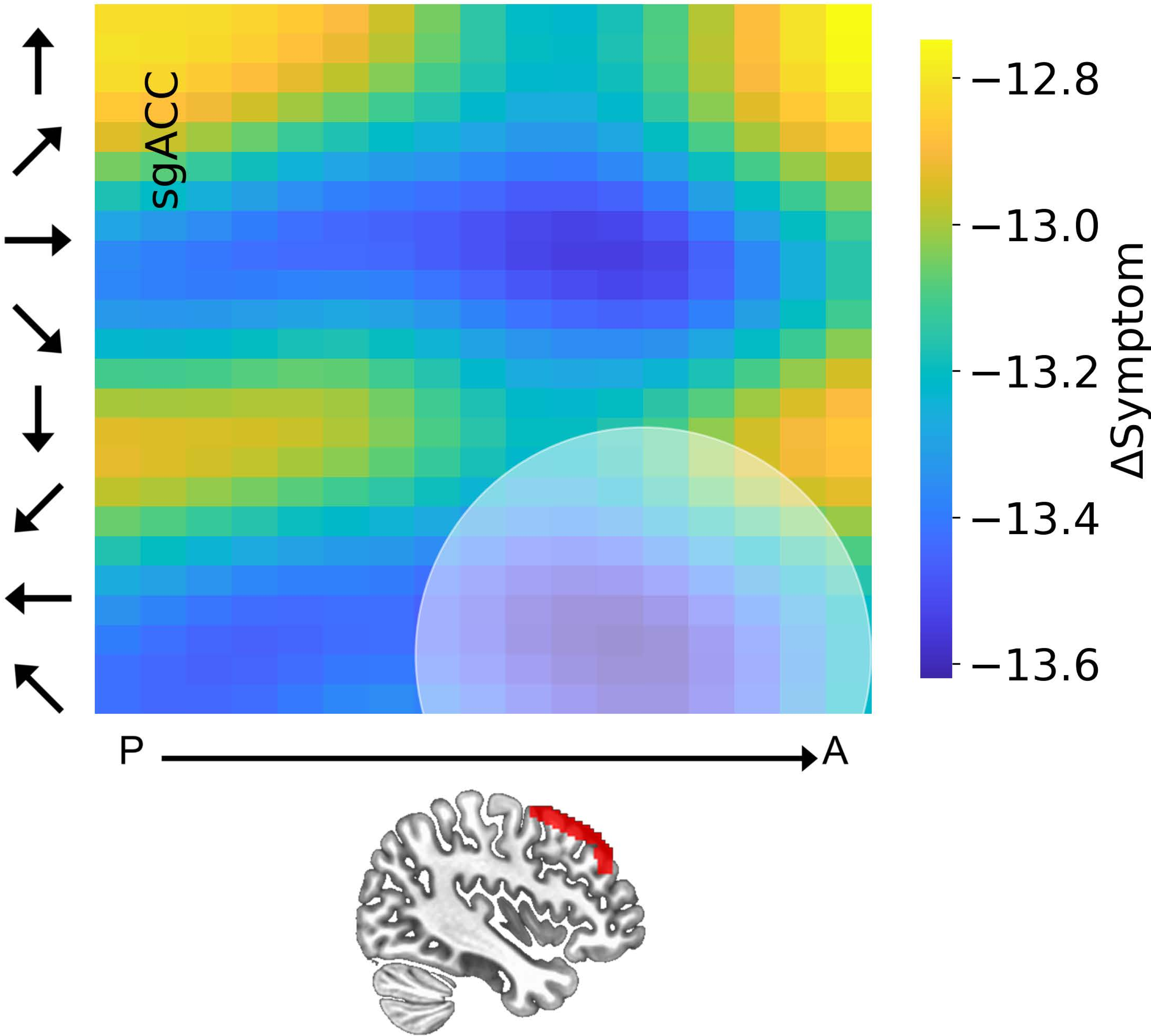

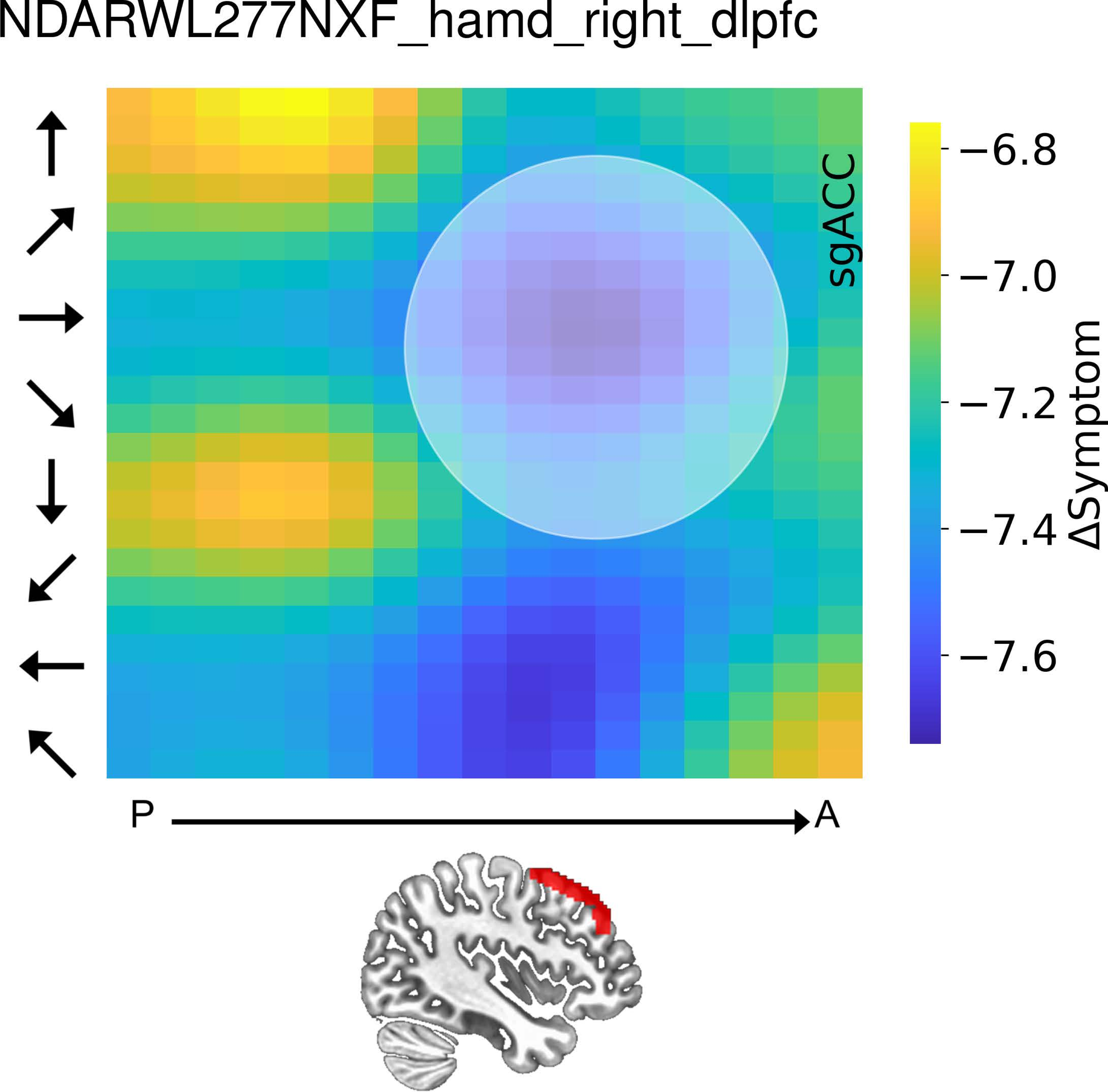
NDARWL277NXF_hamd_right_dlpfc
sgACC
−6.8
−7.0
−7.2
−7.4
−7.6
ΔSymptom
P
A

NDARWV016ZAT_hamd_right_dlpfc

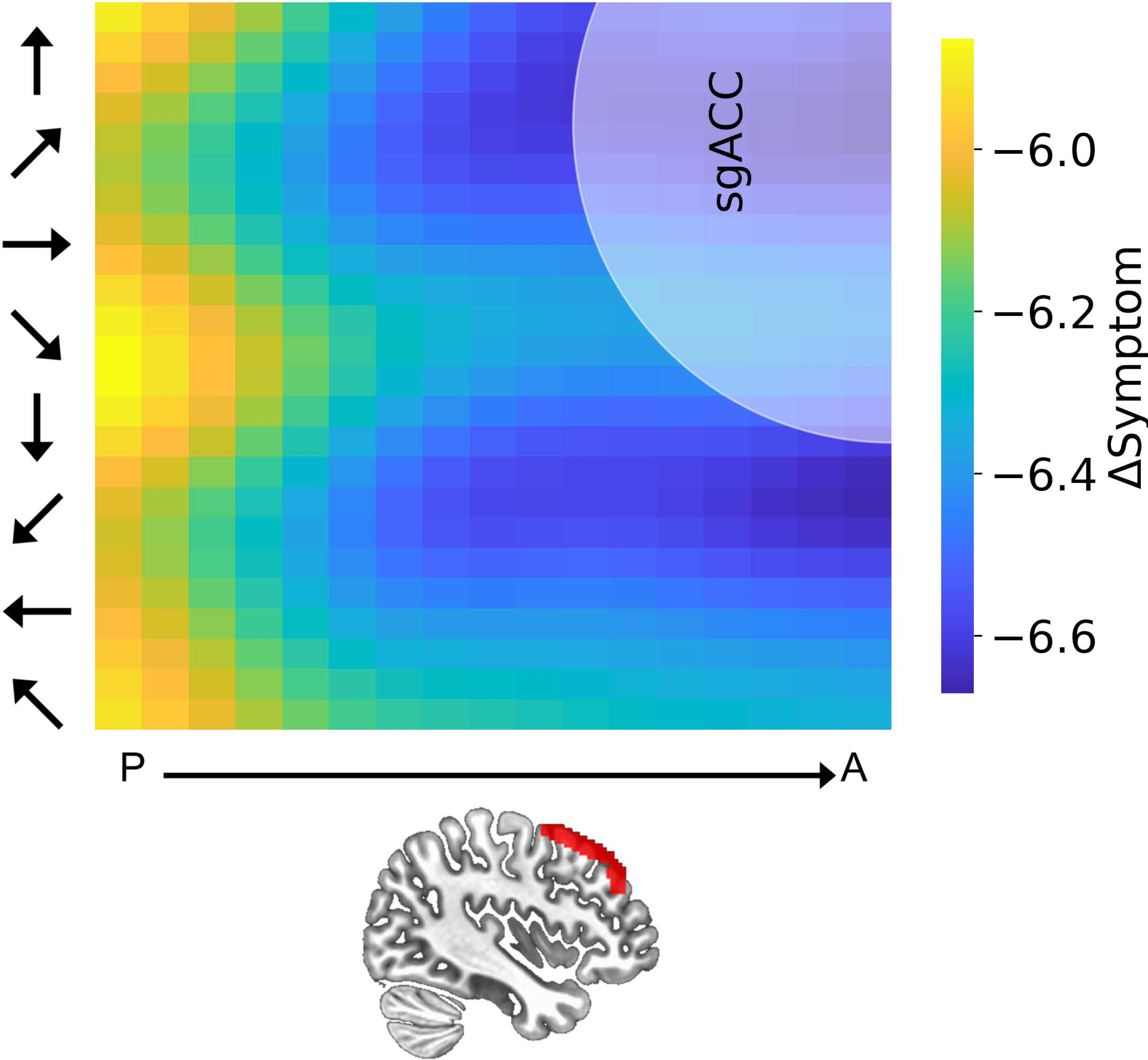

NDARXA148KPL_hamd_right_dlpfc

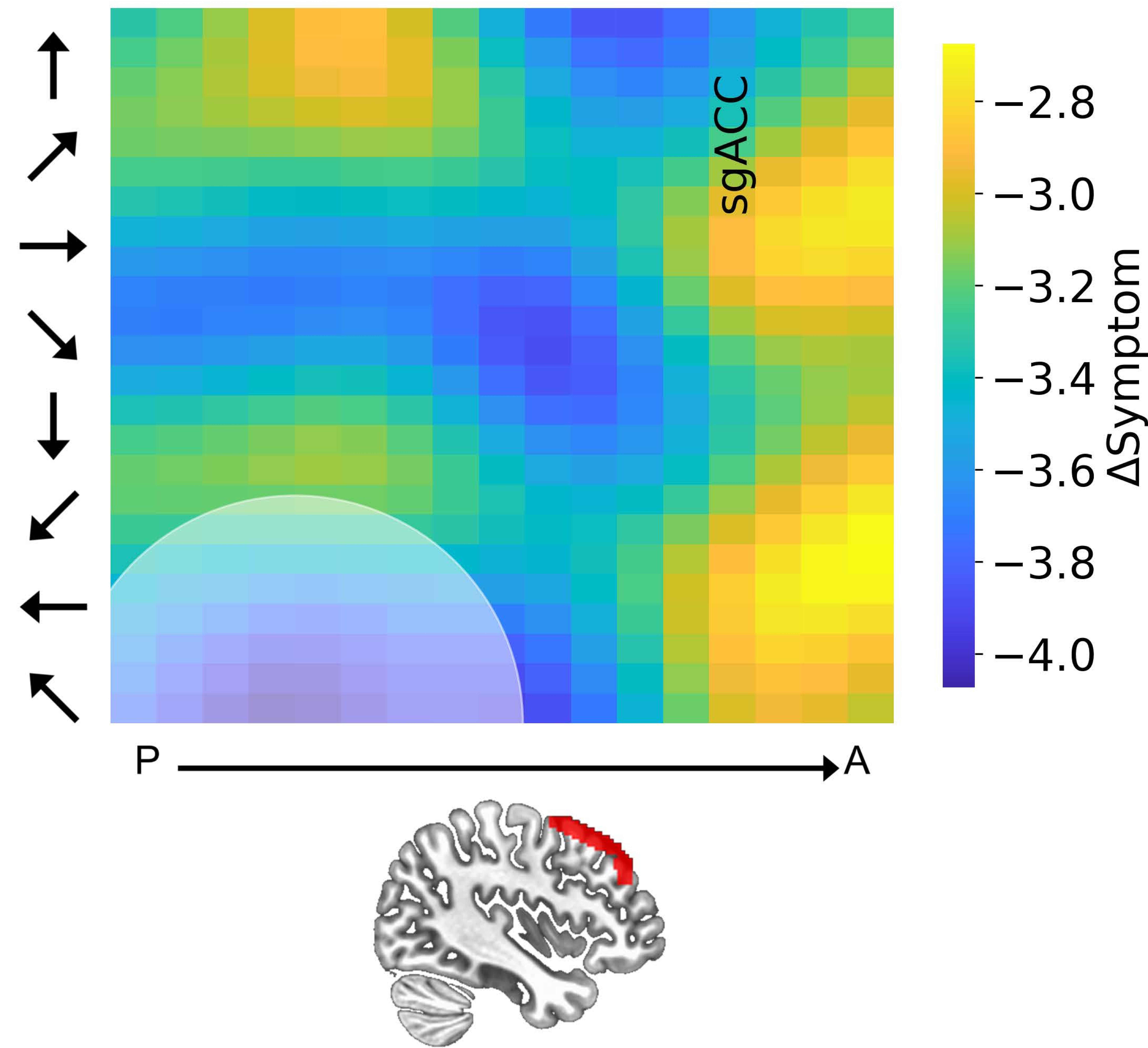

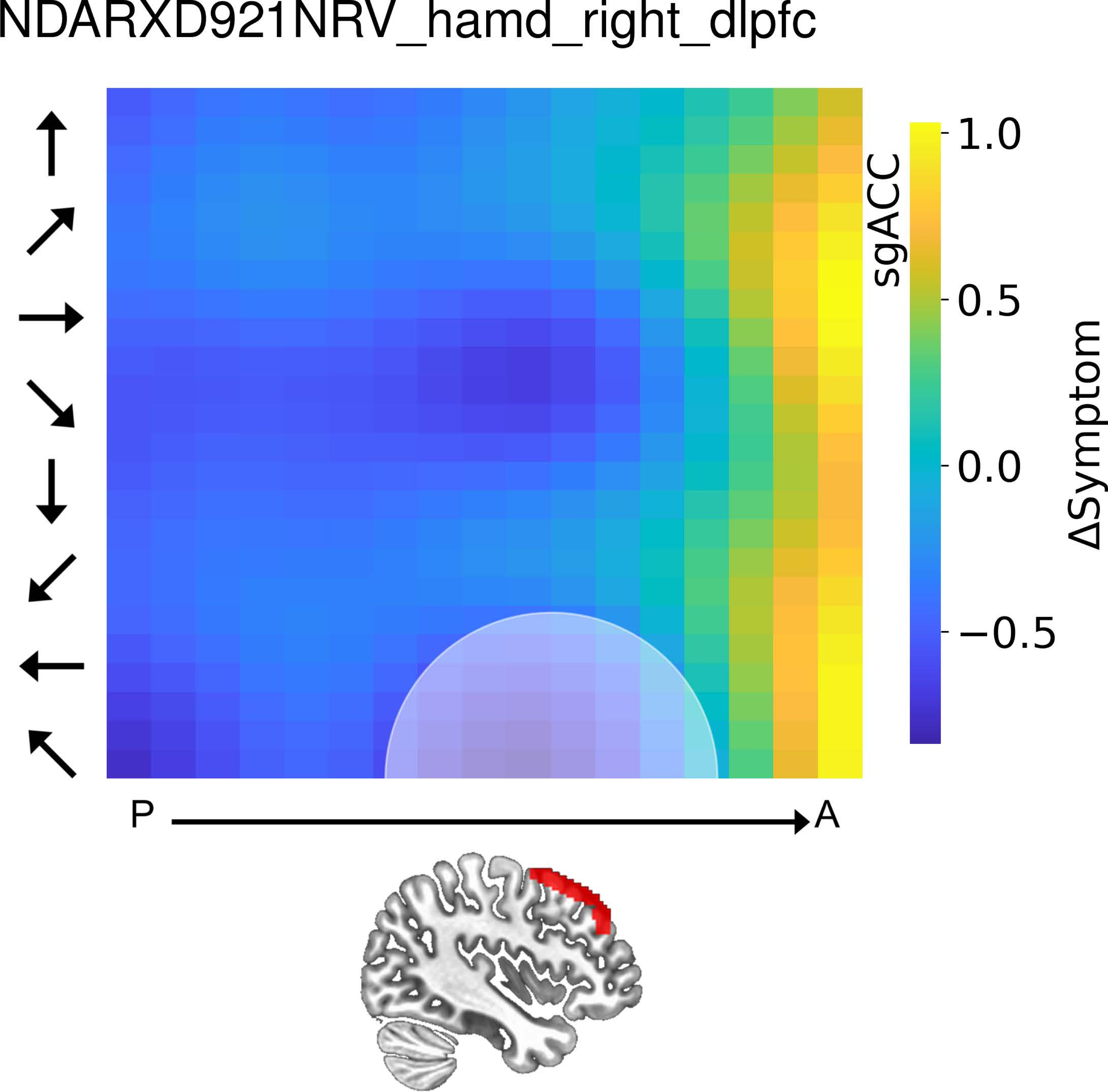
NDARXD921NRV_hamd_right_dlpfc
sgACC
ΔSymptom
1.0
0.5
0.0
−0.5
P
A

NDARXU500EFT_hamd_right_dlpfc

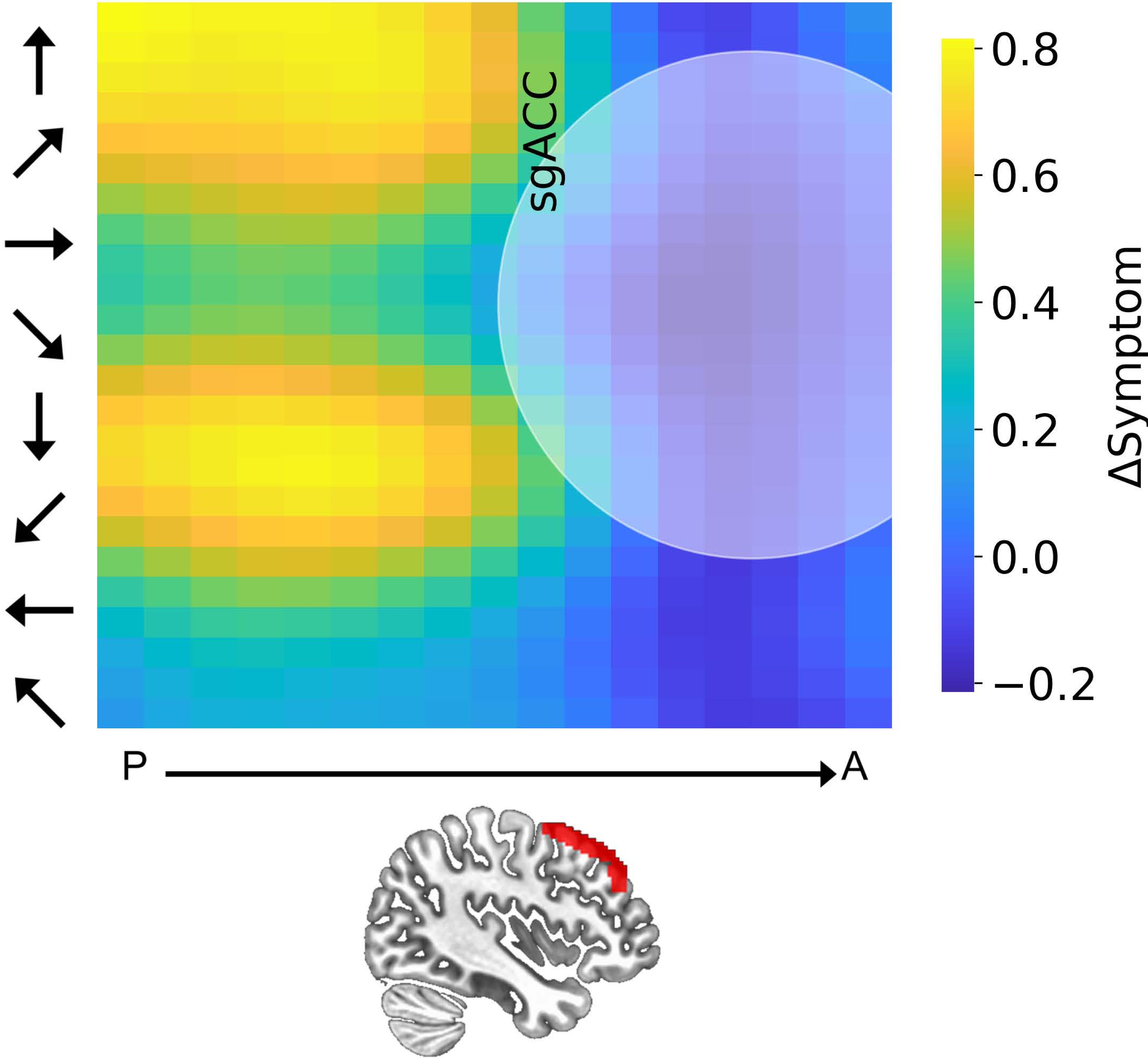

NDARYB181GTB_hamd_right_dlpfc

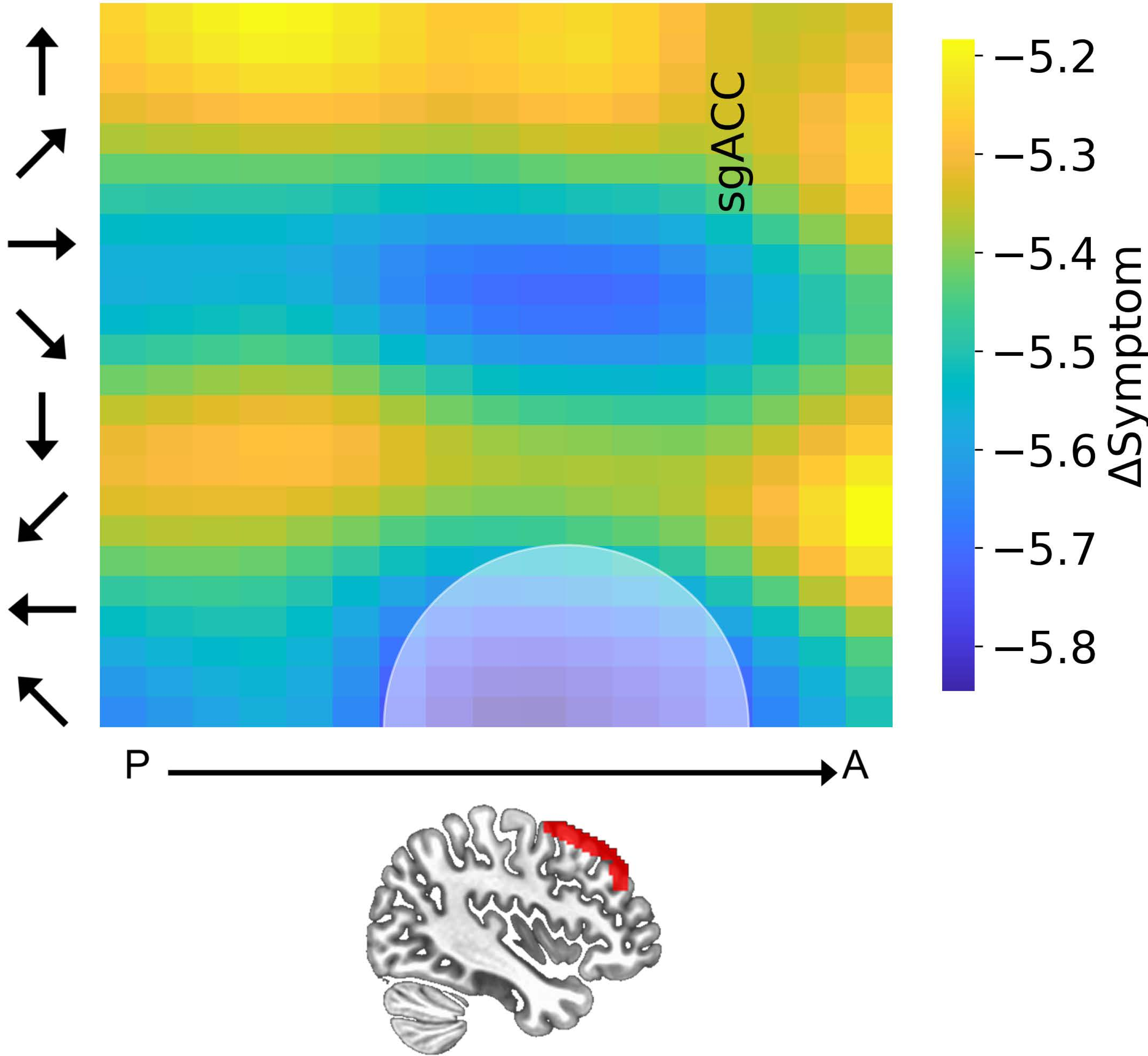

NDARYD169AYQ_hamd_right_dlpfc

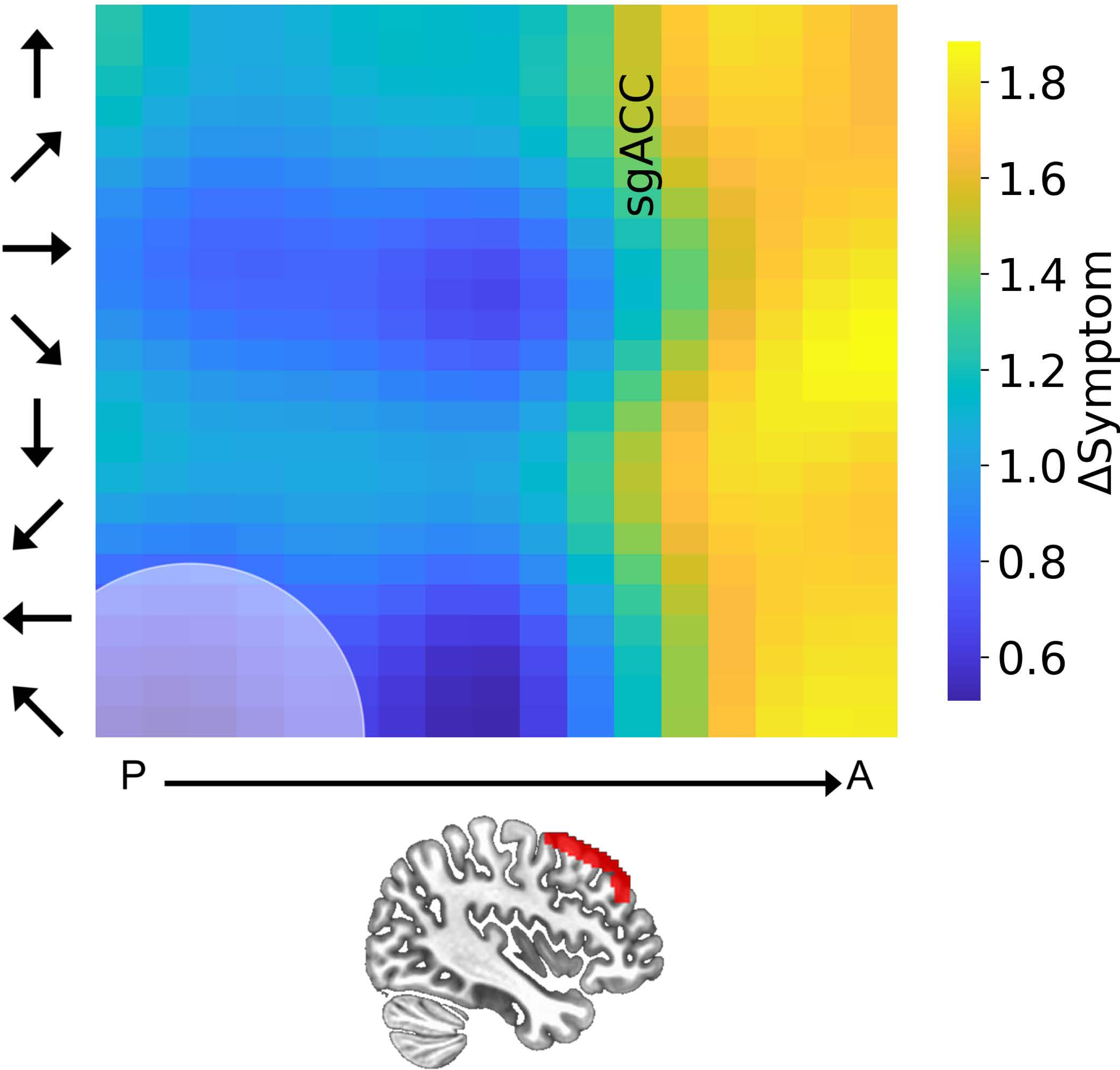

NDARYE405JJL_hamd_right_dlpfc

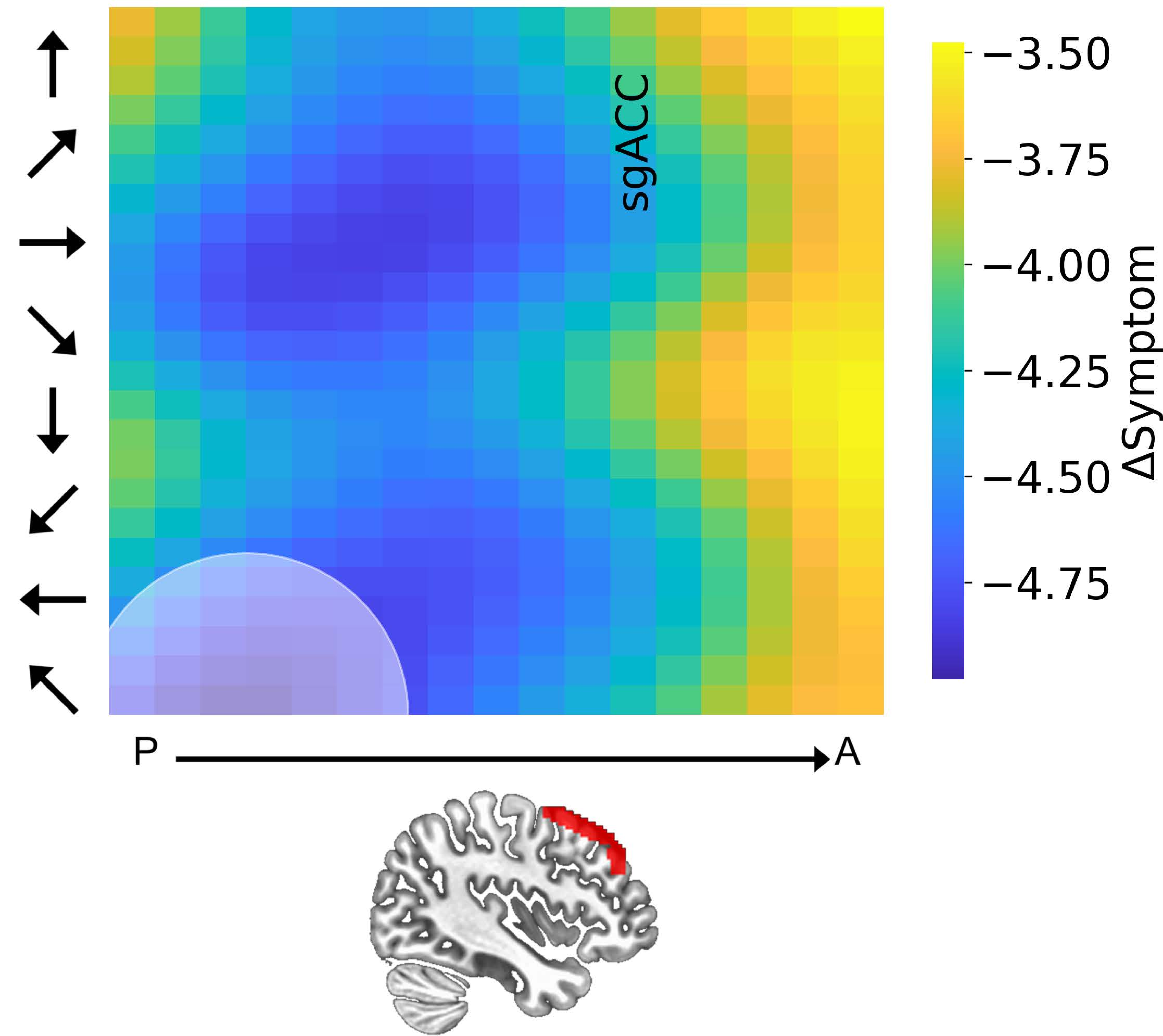

NDARYM648GK3_hamd_right_dlpfc

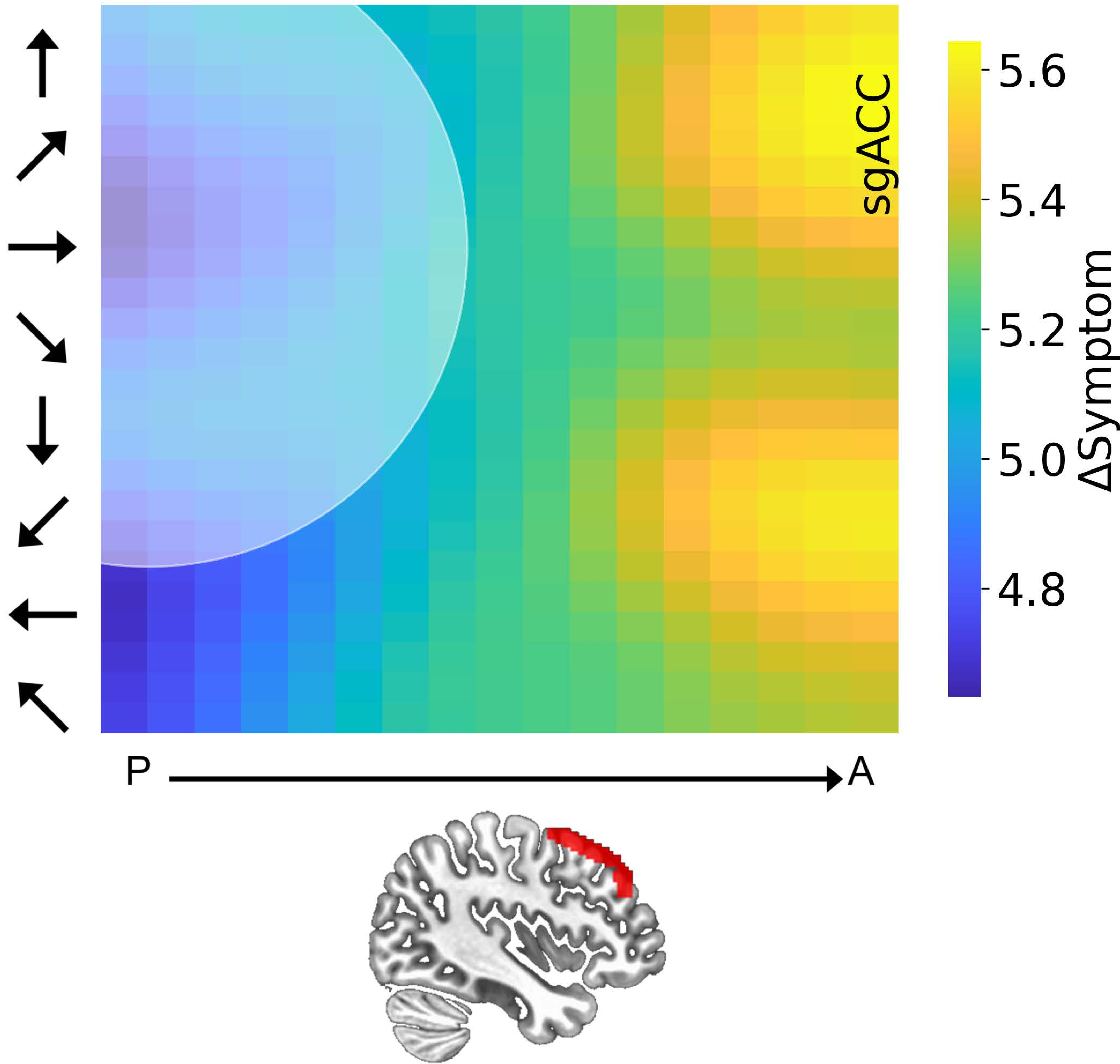

NDARZH200GZU_hamd_right_dlpfc

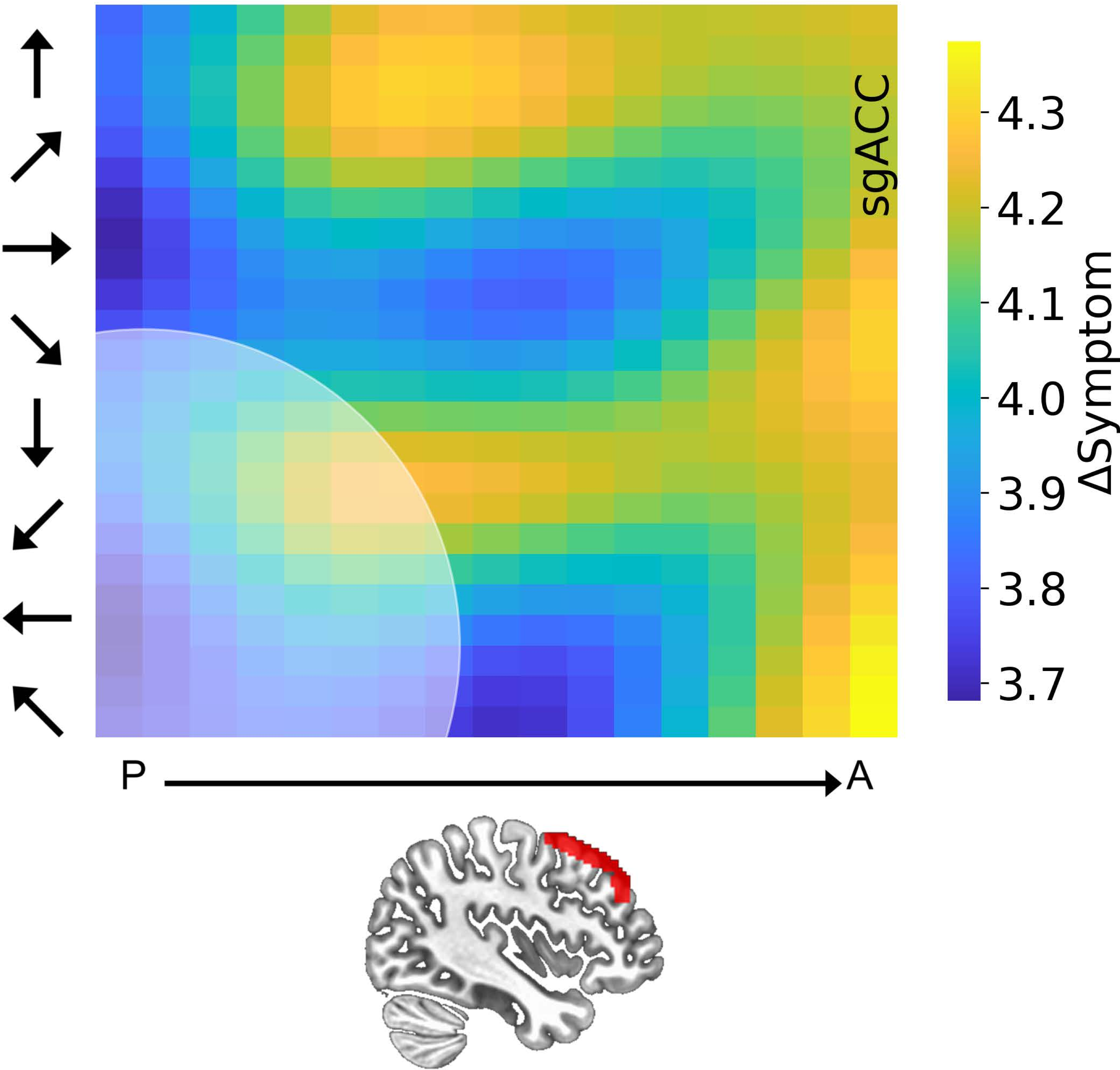

Healthy control subjects' heatmaps

NDARAC286ZE7_madrs_left_dlpfc

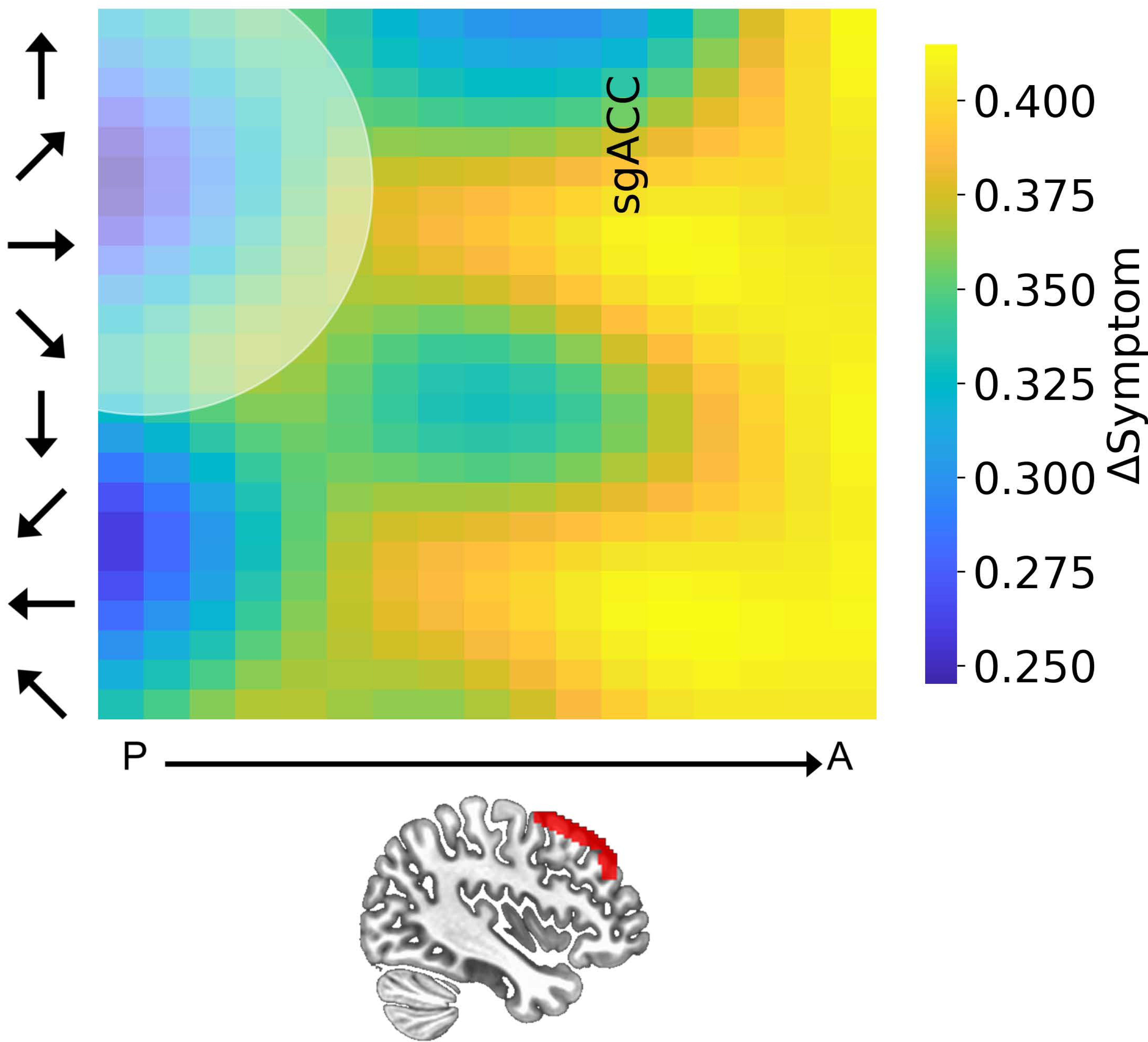

NDARAC789HEZ_madrs_left_dlpfc

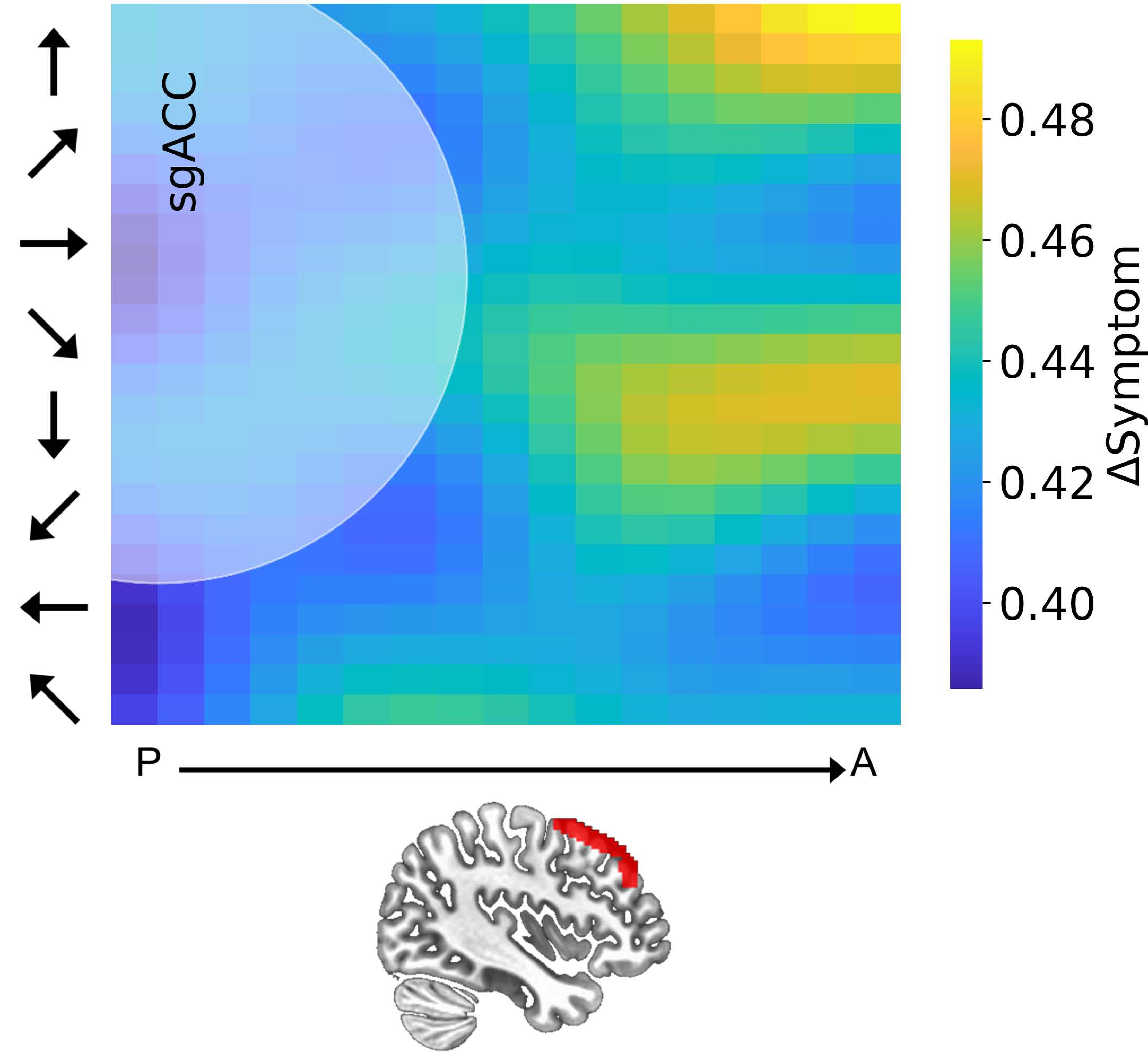

NDARBD224PBF_madrs_left_dlpfc

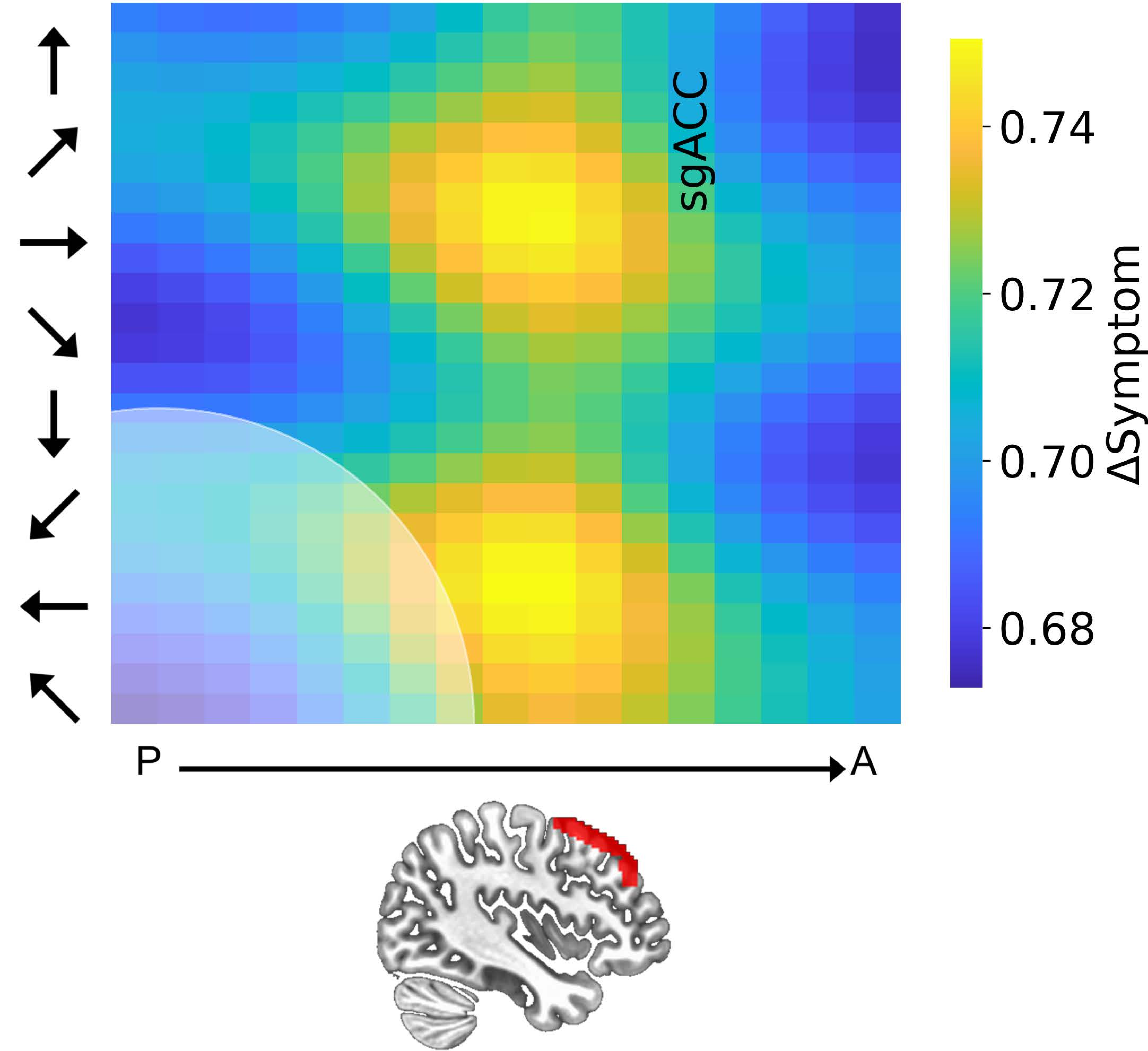

NDARBF292NPE_madrs_left_dlpfc

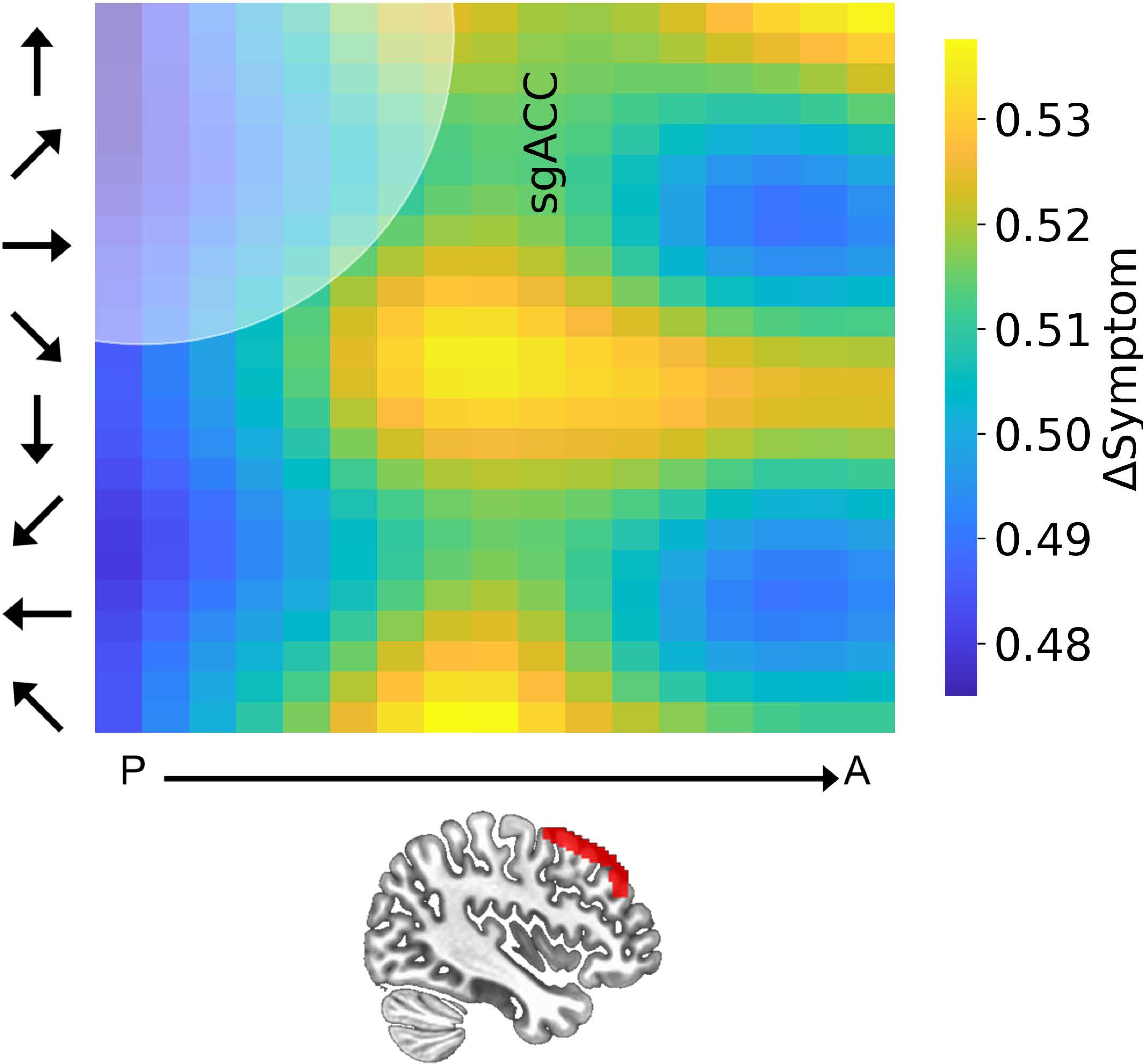

NDARCG535CPC_madrs_left_dlpfc

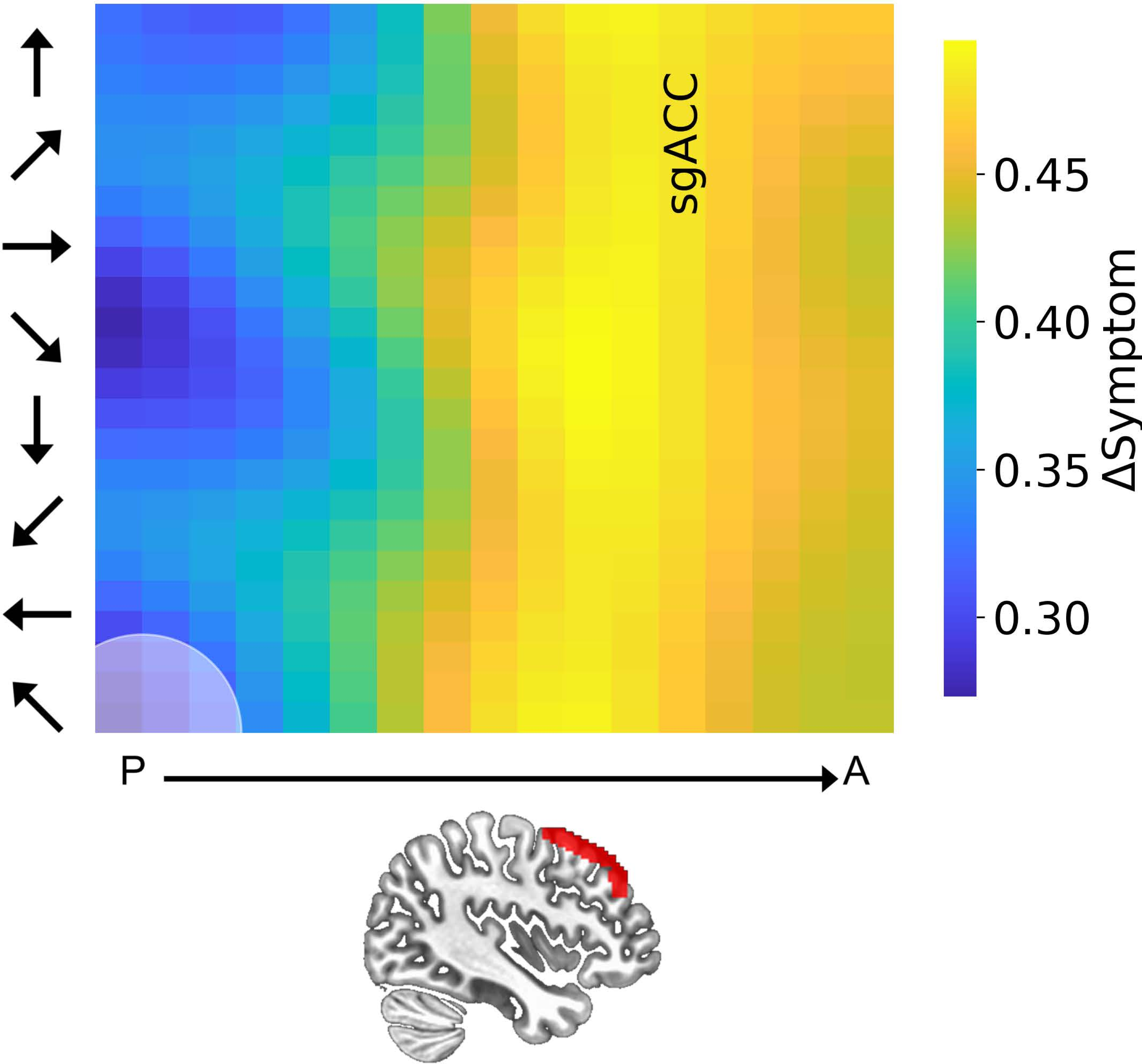

NDARCY406TU1_madrs_left_dlpfc

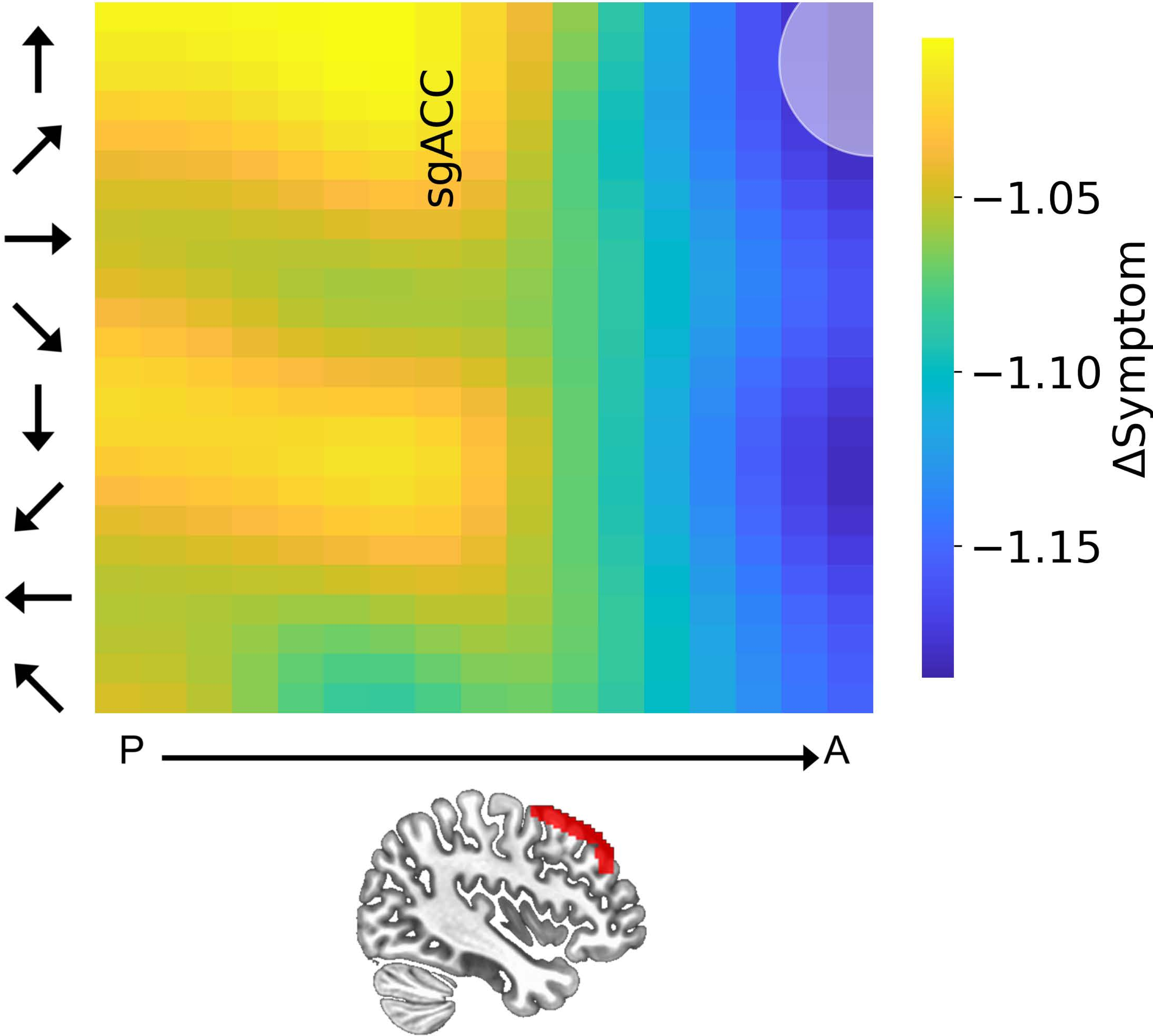

NDARDY771TXZ_madrs_left_dlpfc

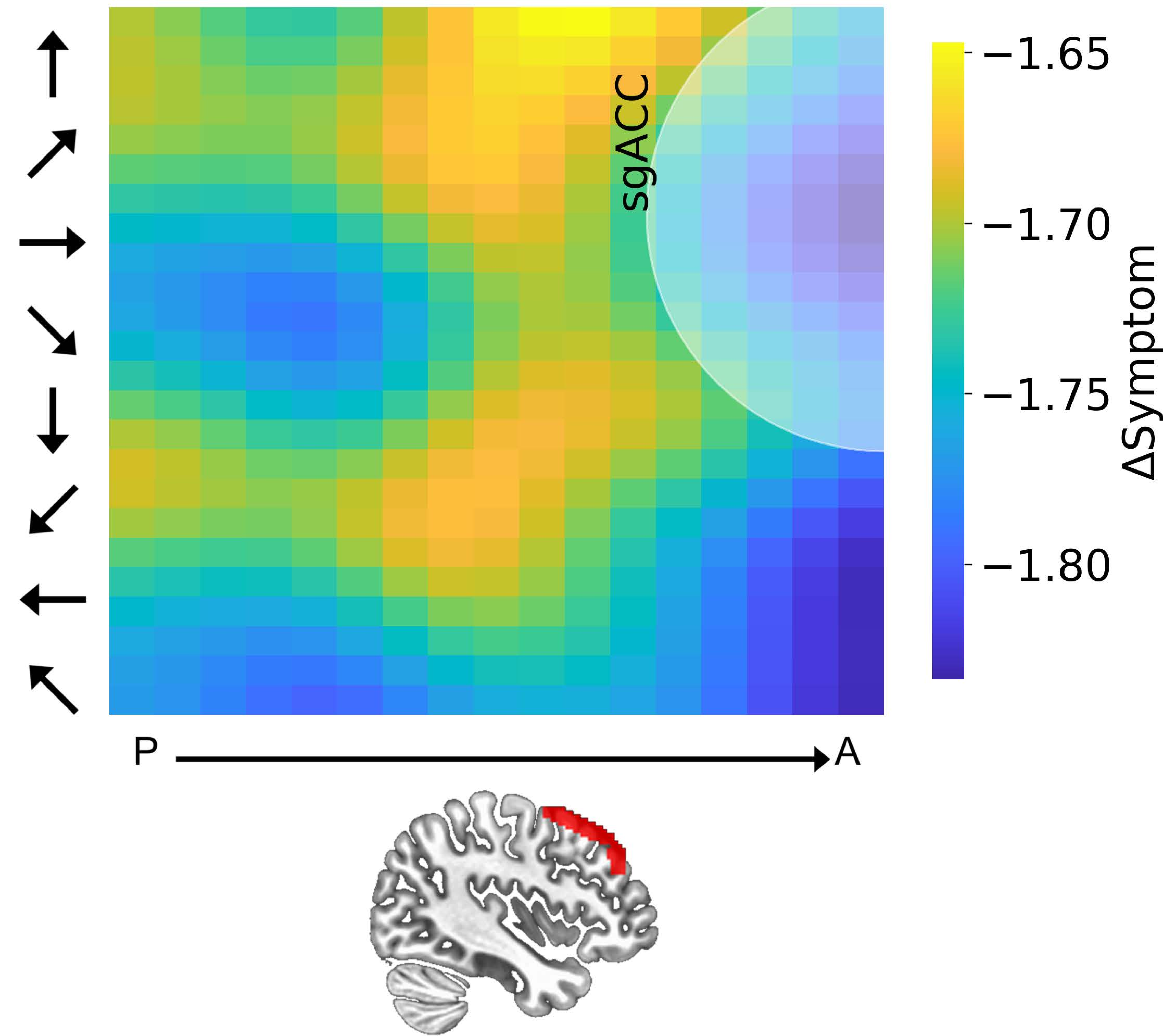

NDAREU790BVQ_madrs_left_dlpfc

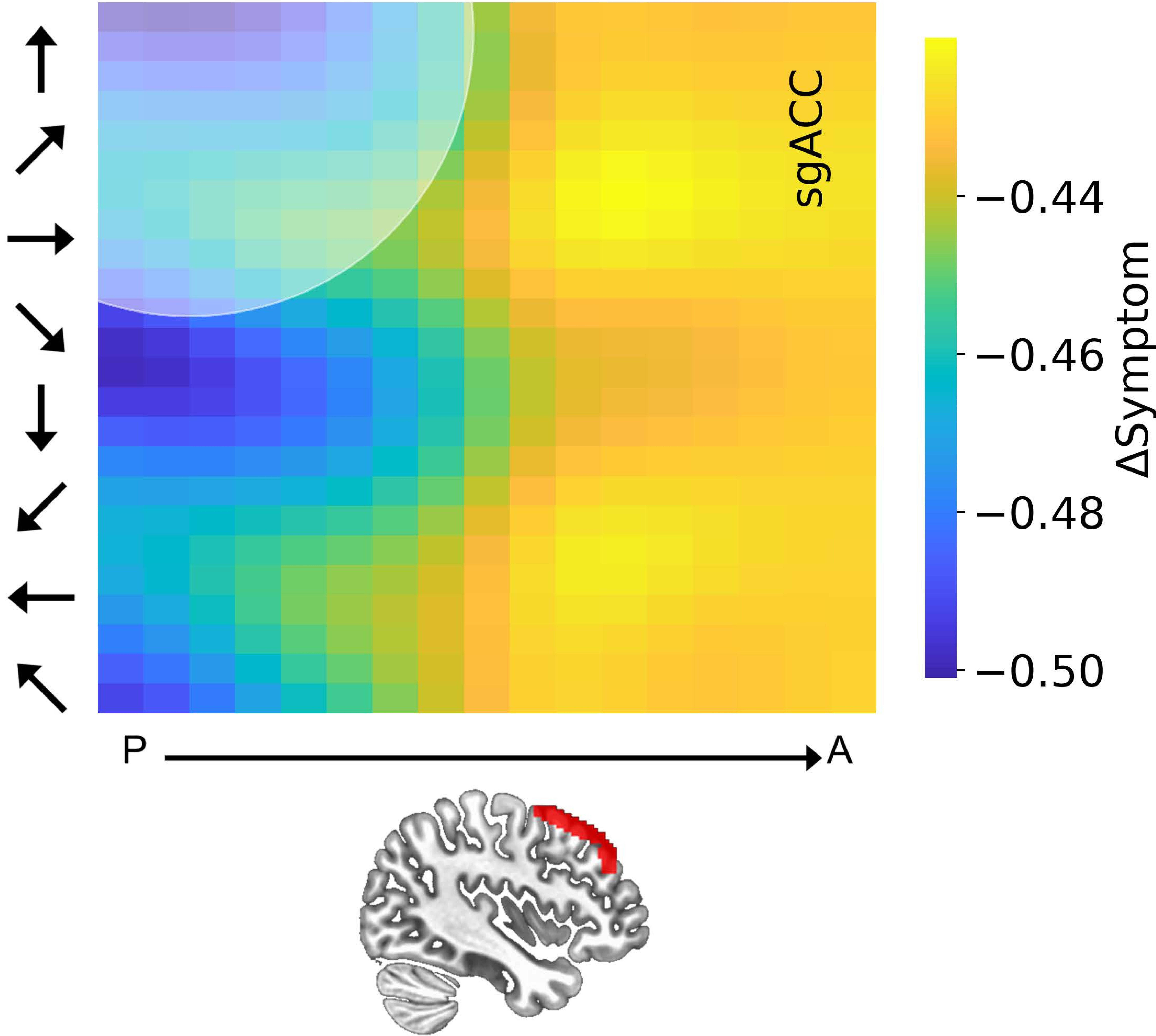

NDARGA844BF5_madrs_left_dlpfc

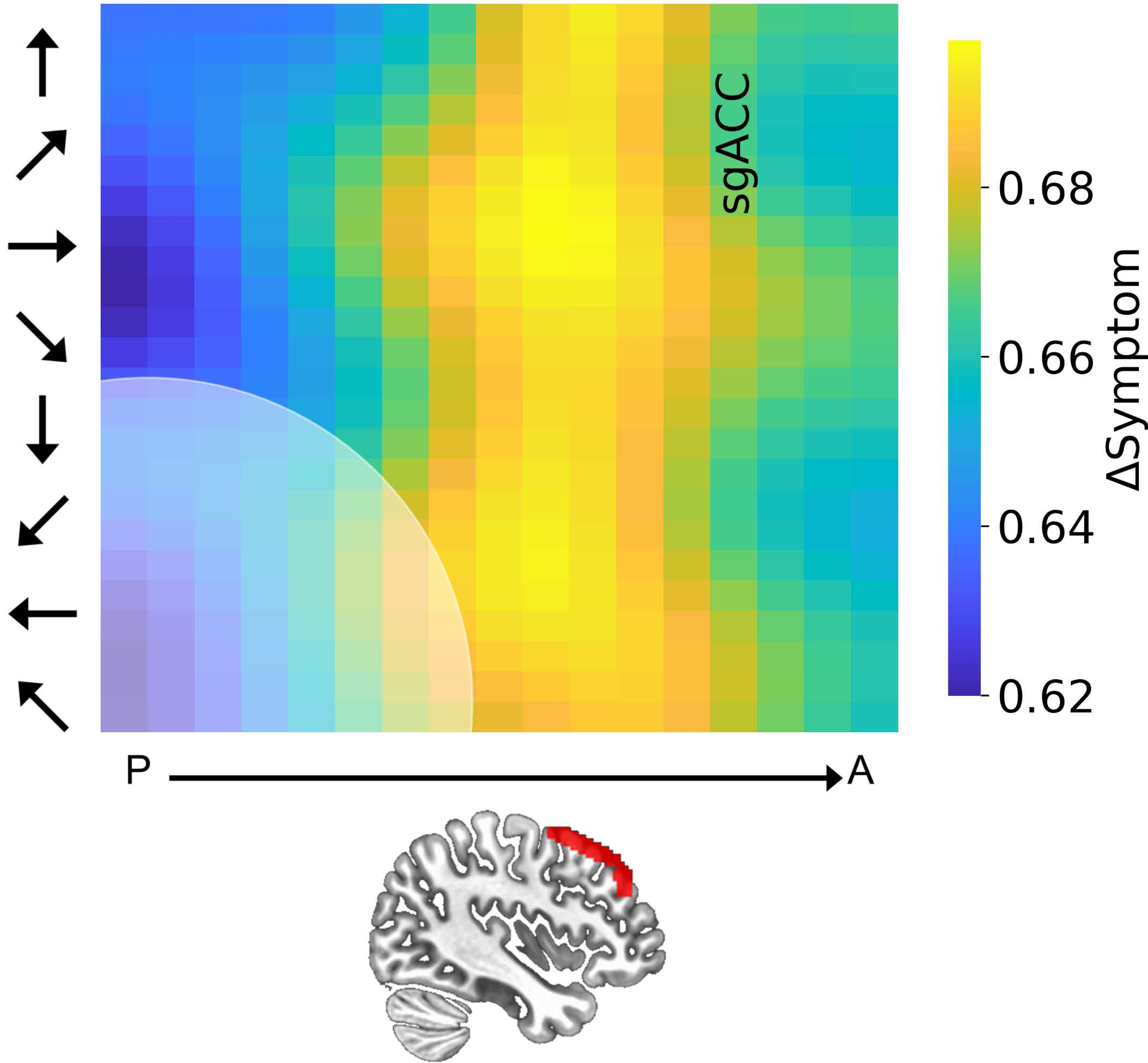

NDARGN596ENP_madrs_left_dlpfc

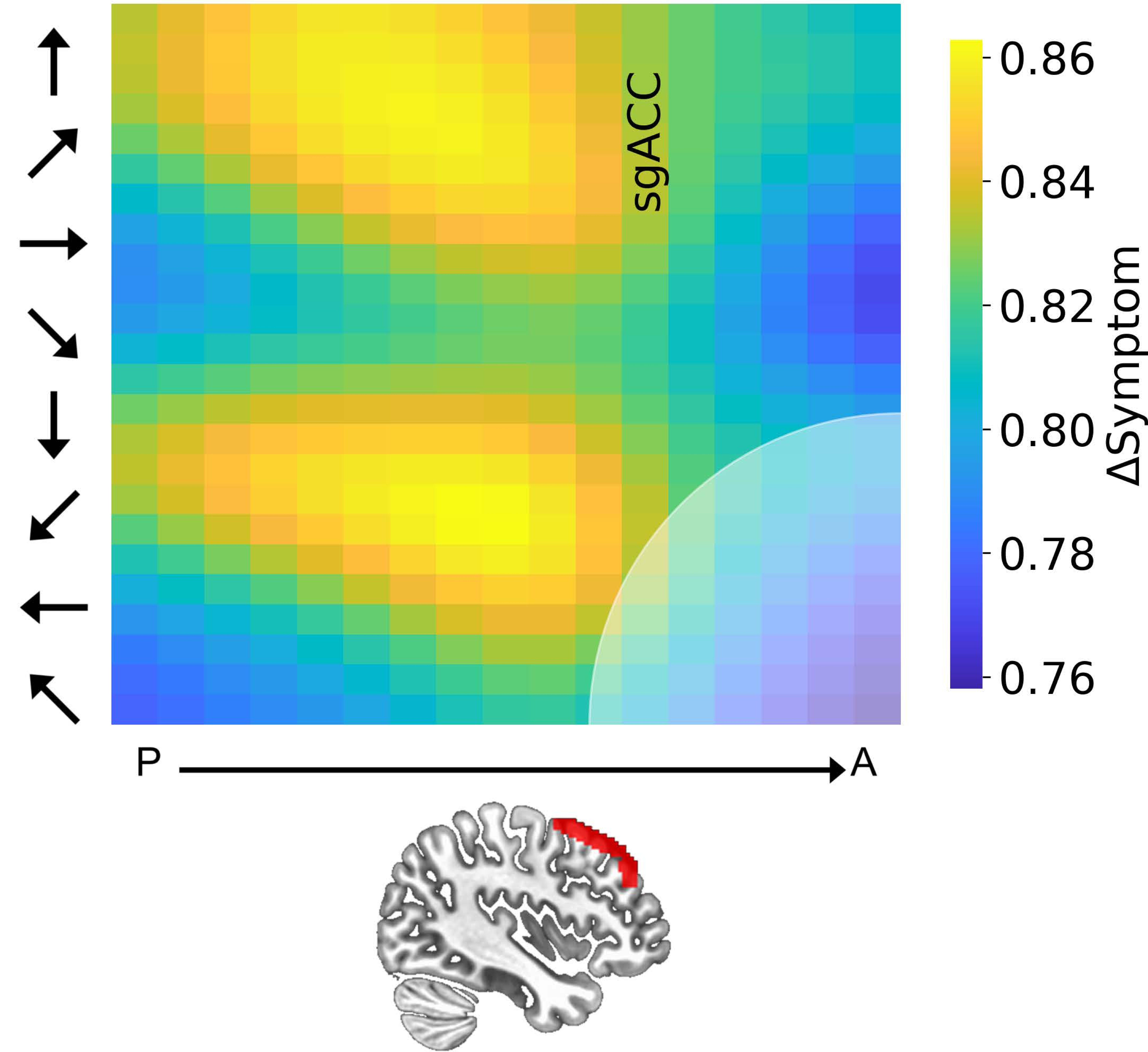

NDARHU677DFF_madrs_left_dlpfc

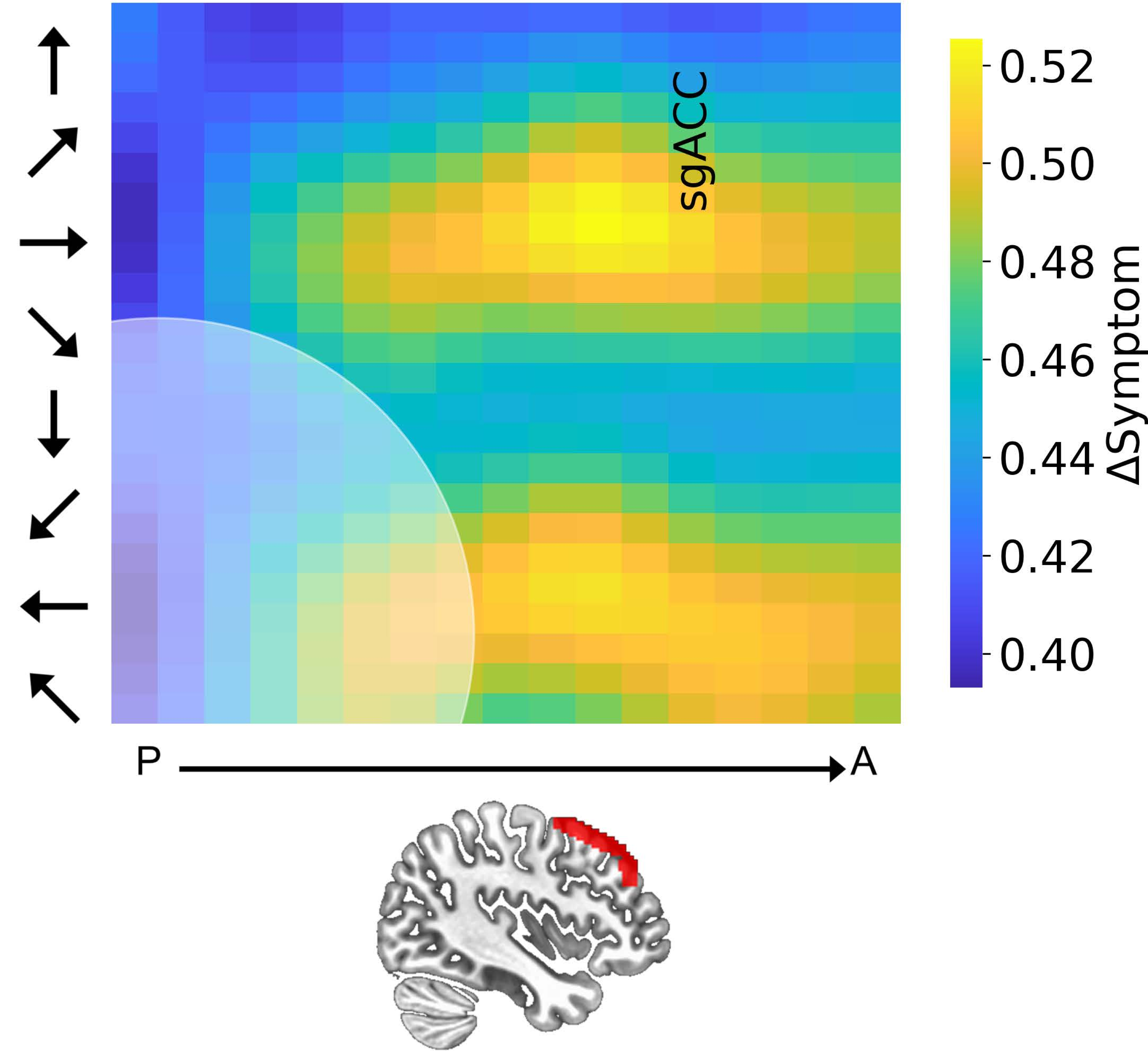

NDARJL083WMP_madrs_left_dlpfc

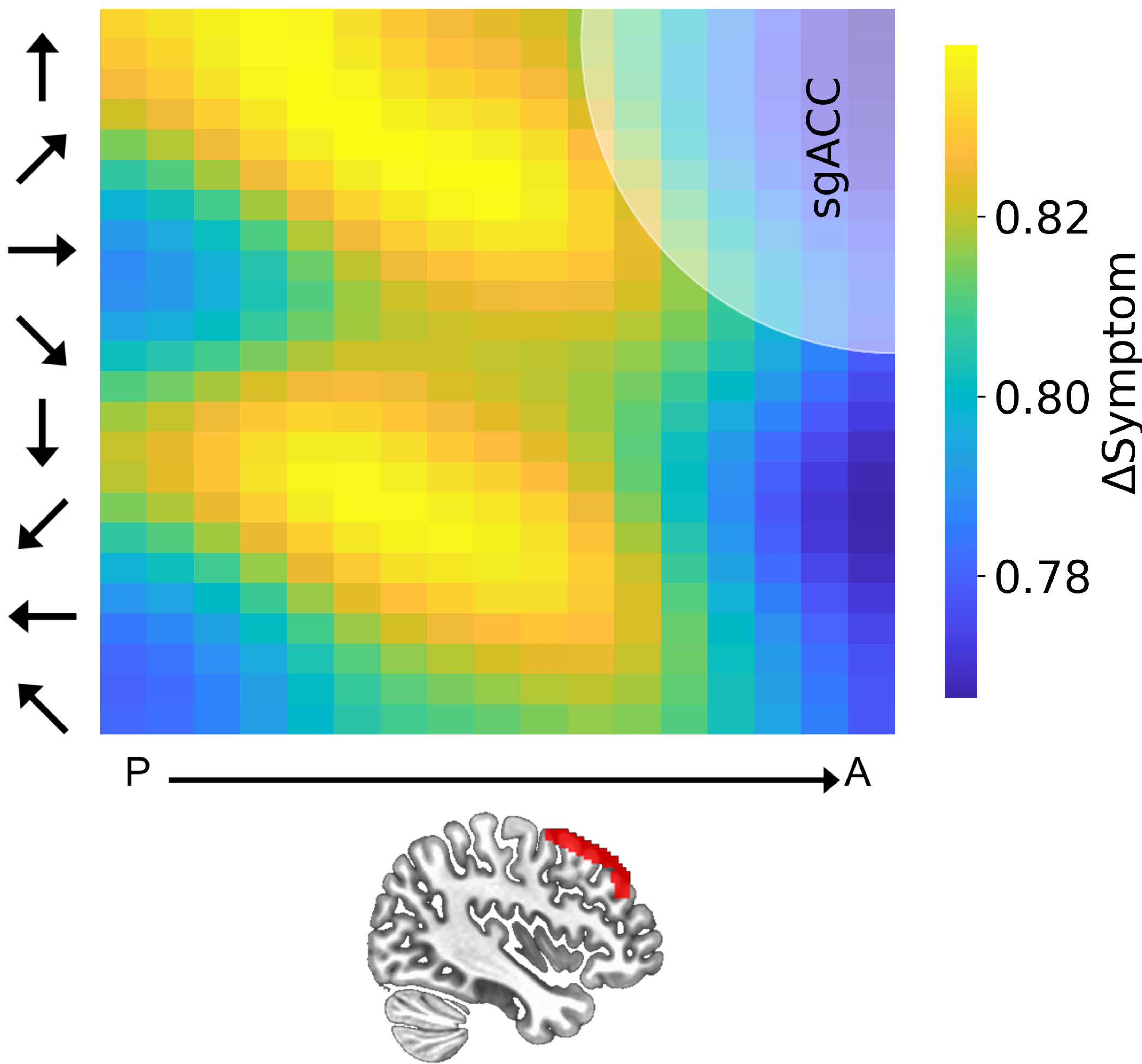

NDARKR291PWN_madrs_left_dlpfc

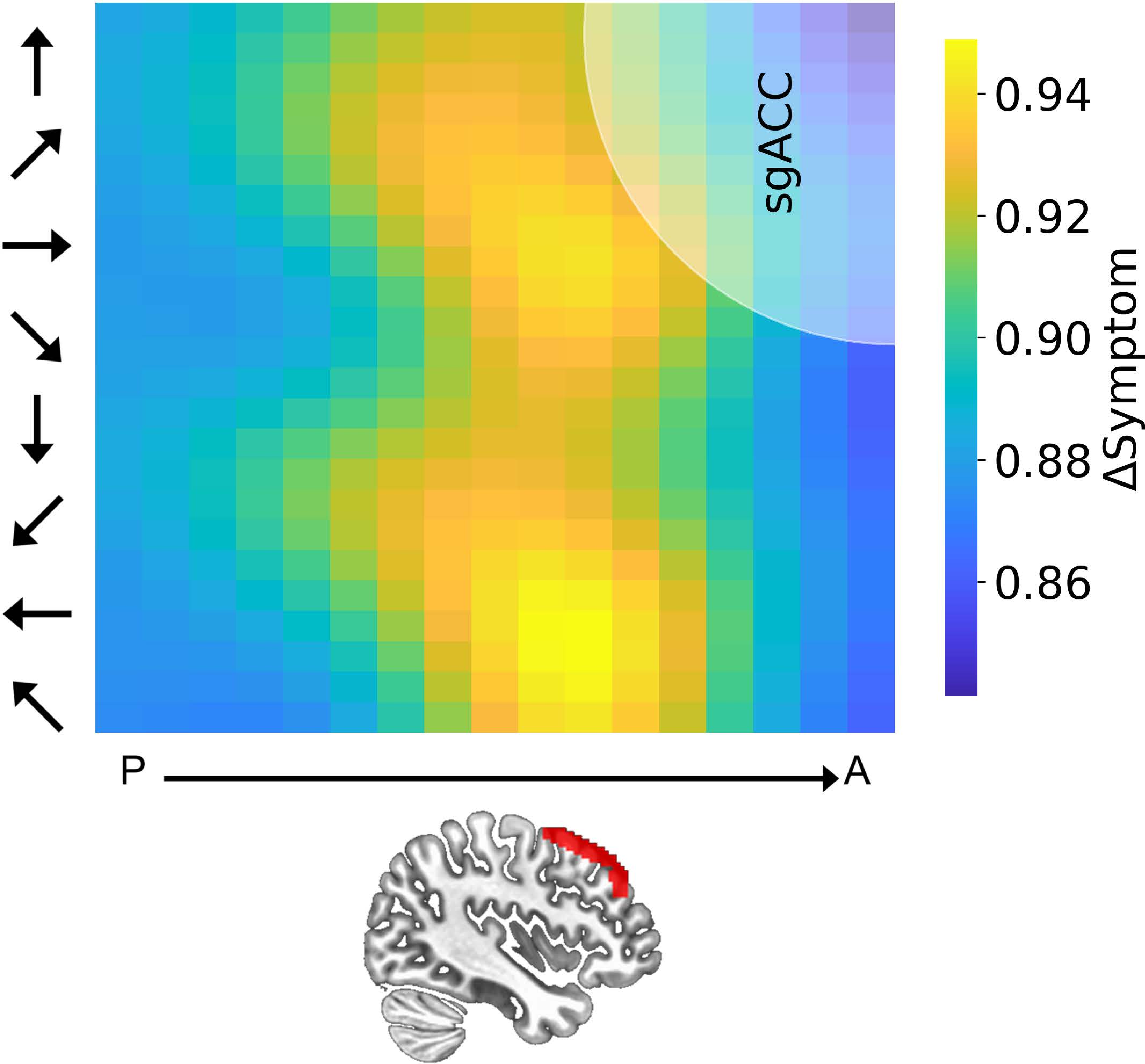

NDARLH479MBF_madrs_left_dlpfc

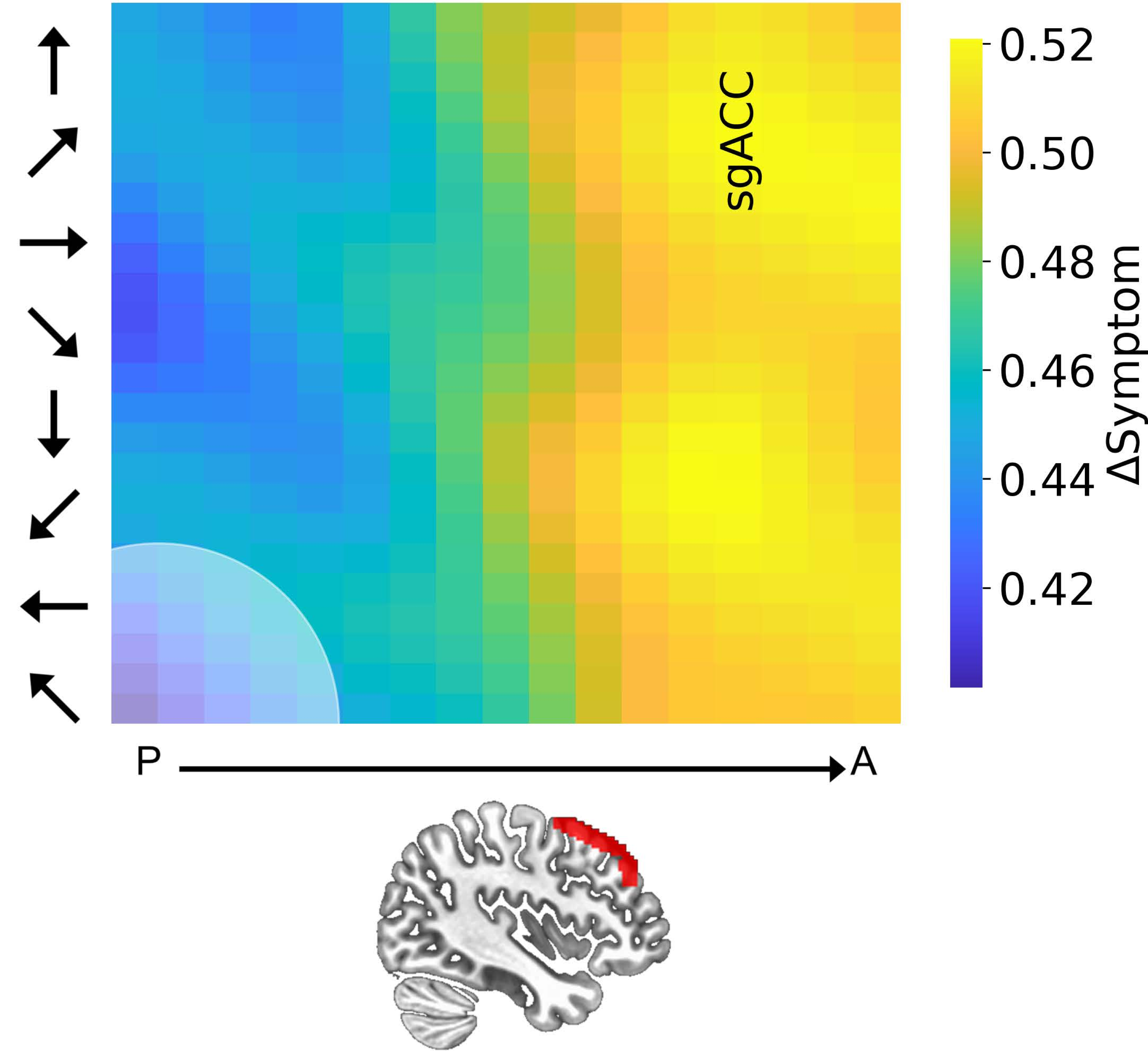

NDARME497RF8_madrs_left_dlpfc

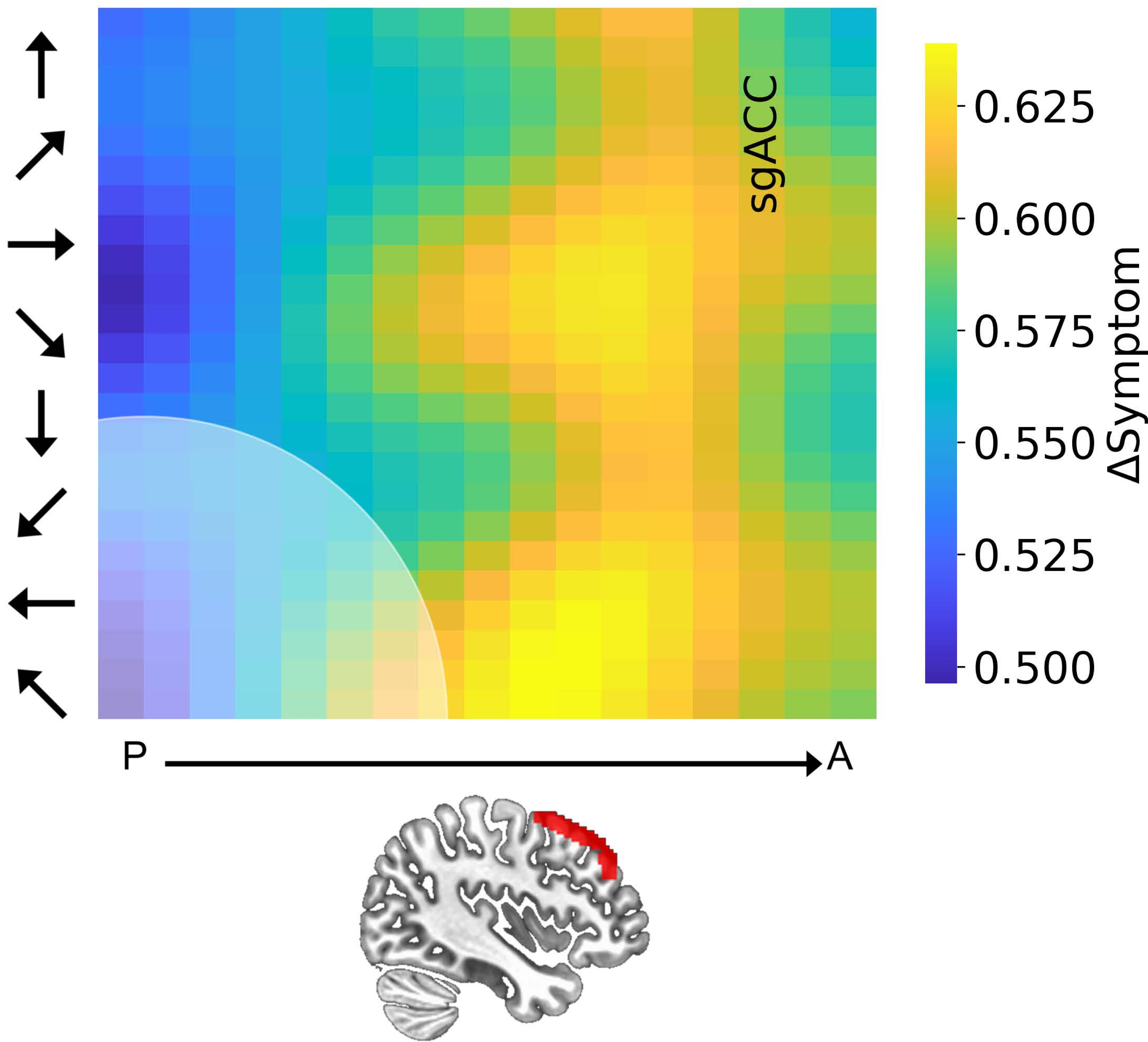

NDARNJ618KZJ_madrs_left_dlpfc

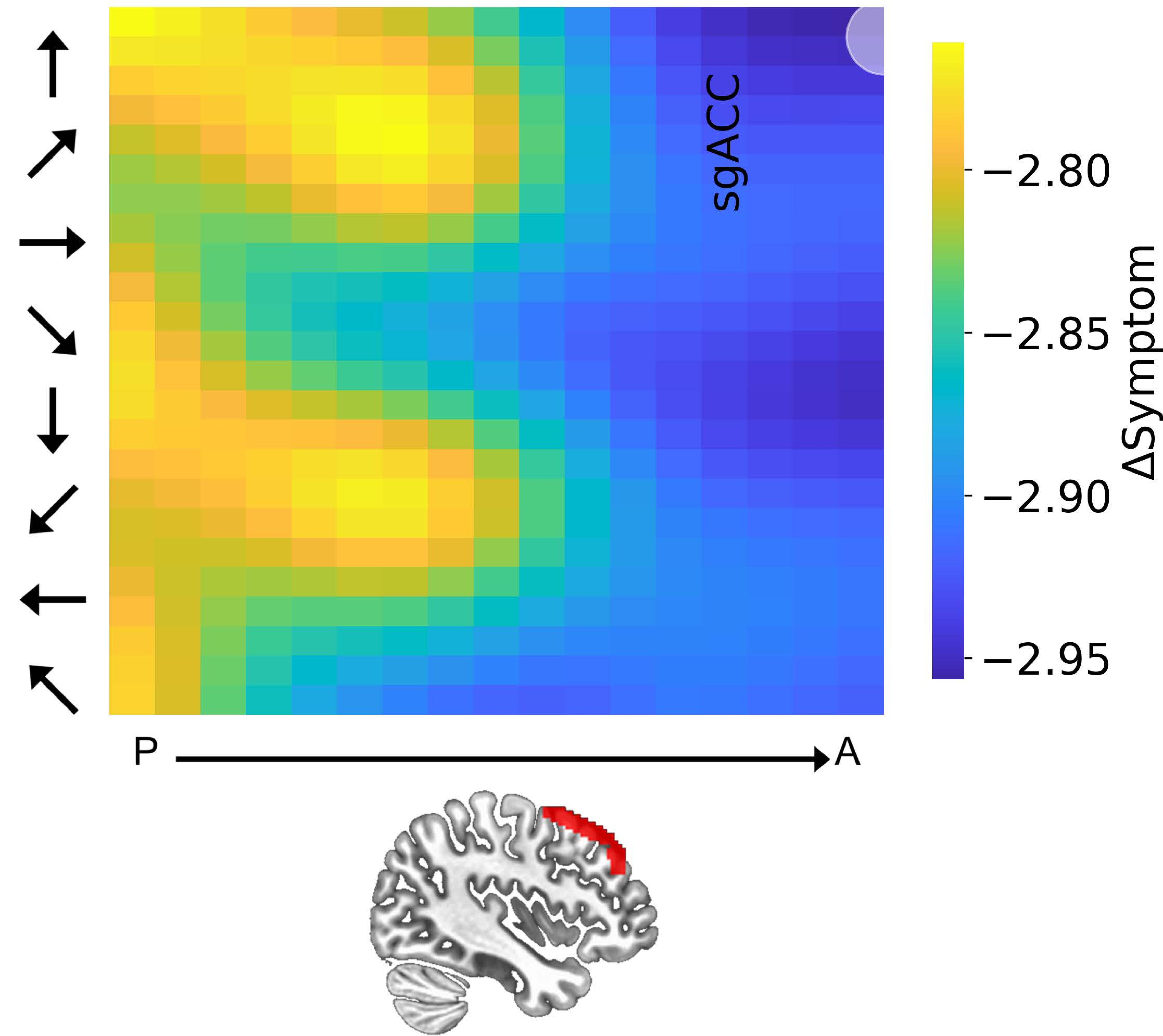

NDARNL715RTY_madrs_left_dlpfc

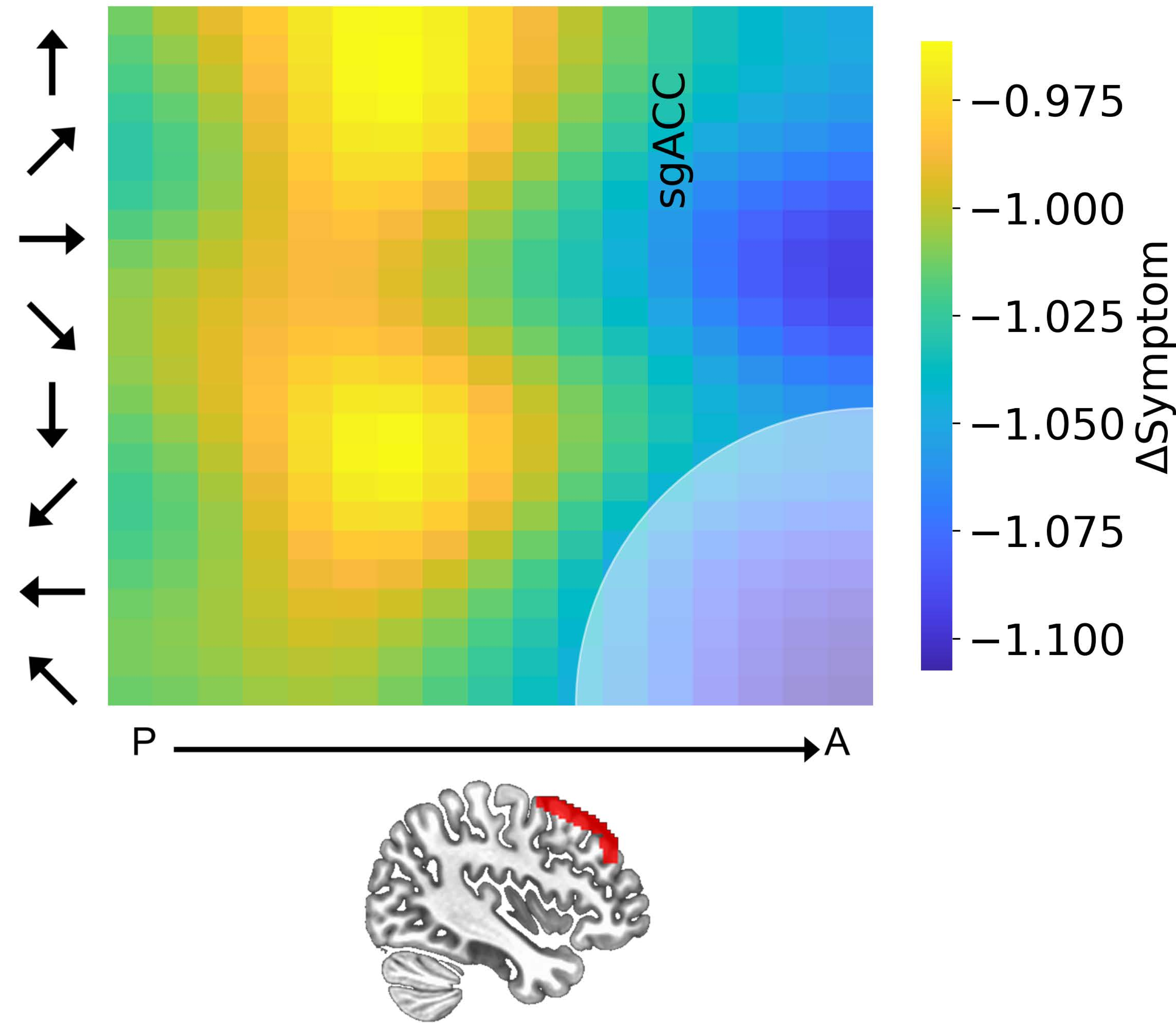

NDARPK532CVC_madrs_left_dlpfc

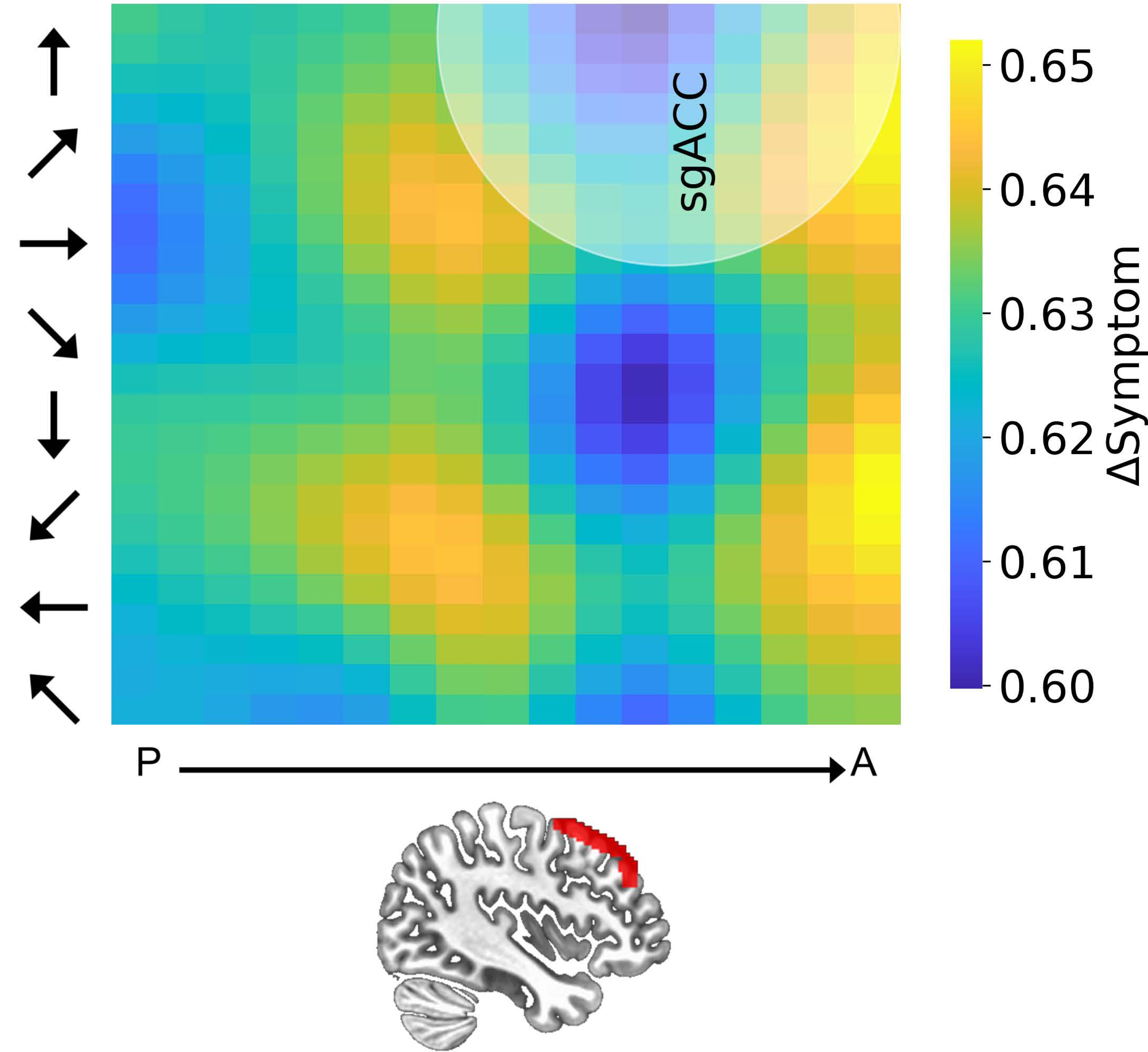

NDARWK858FA2_madrs_left_dlpfc

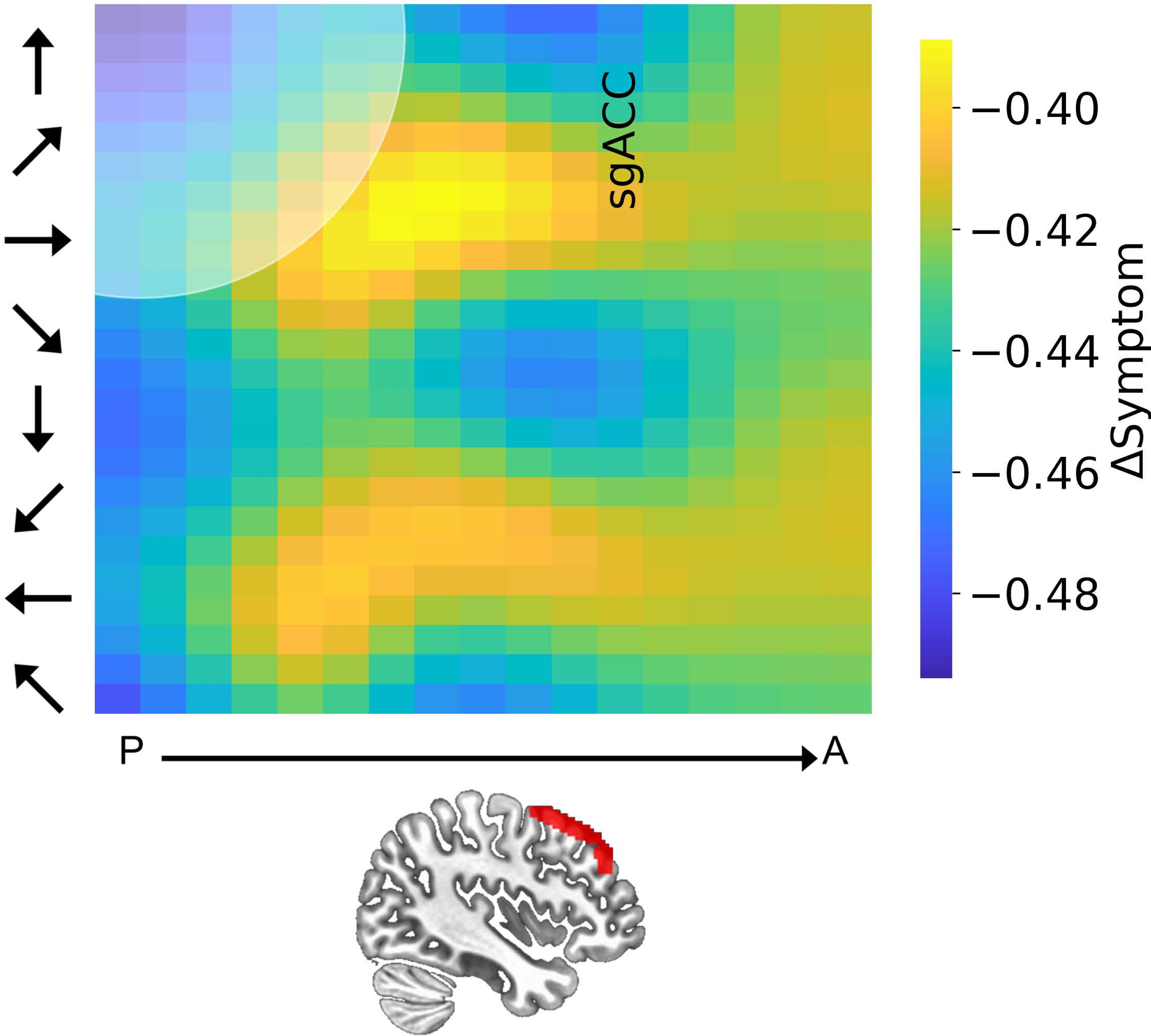

NDARWM071DRZ_madrs_left_dlpfc

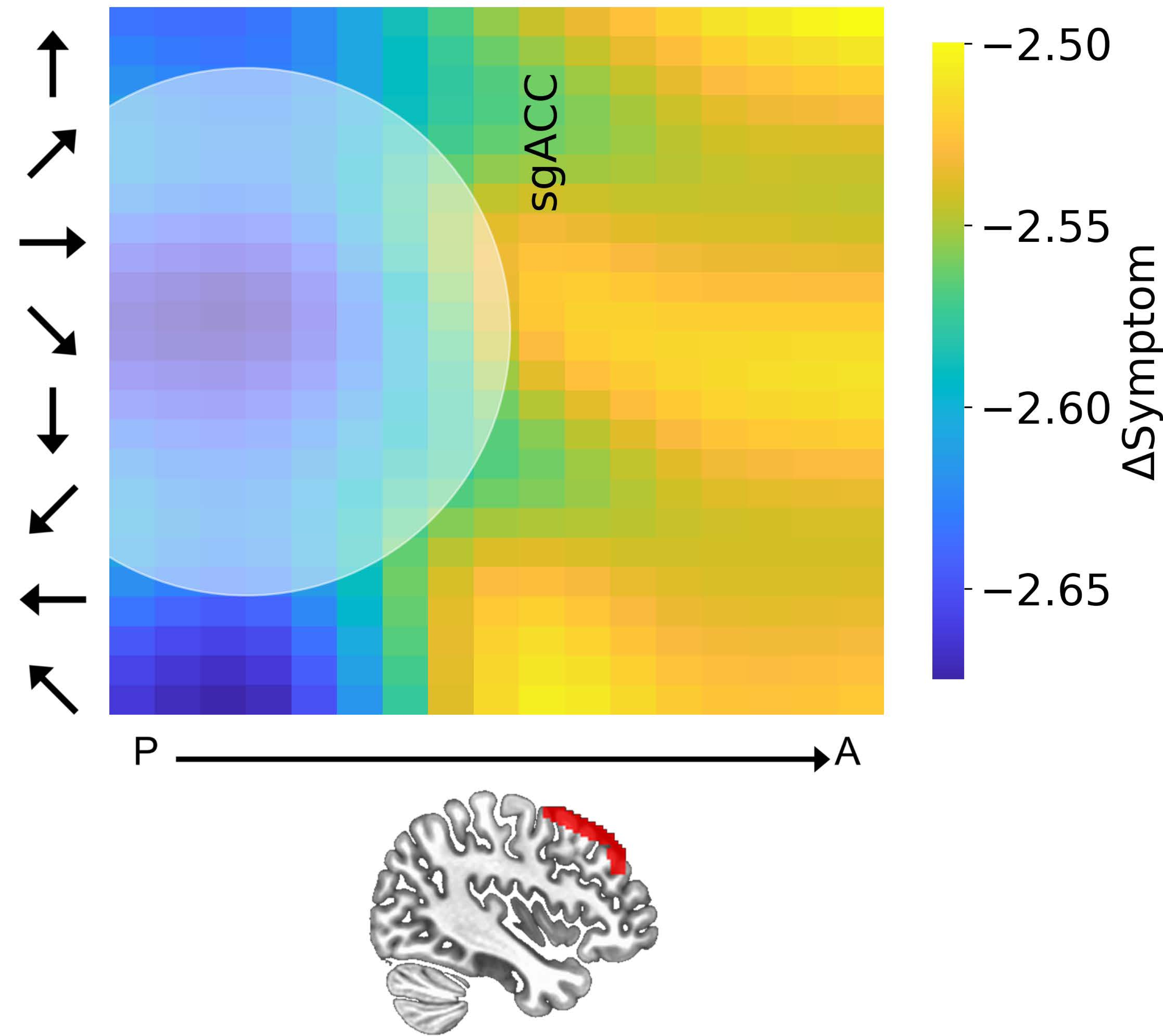

NDARWT226PU5_madrs_left_dlpfc

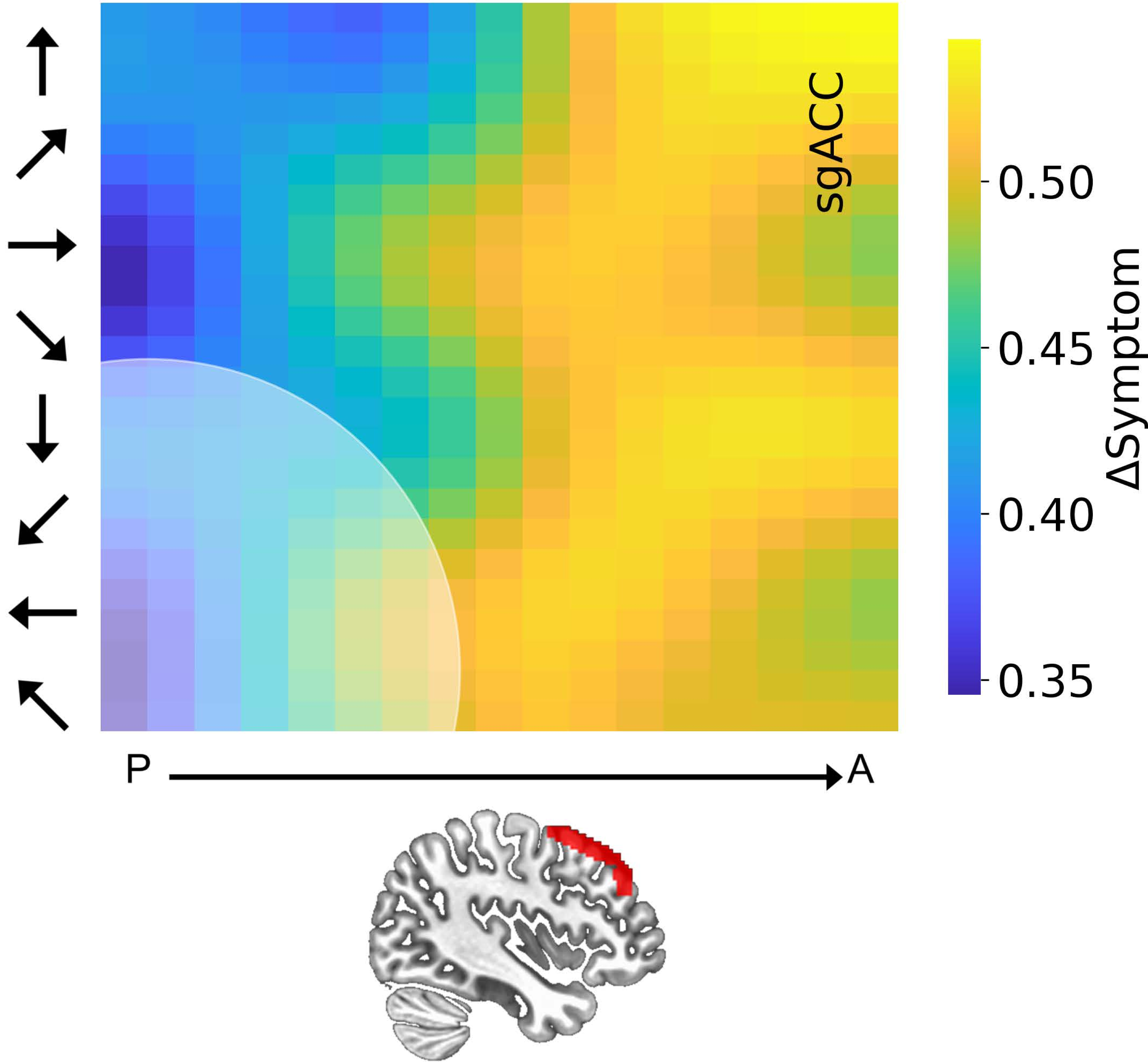

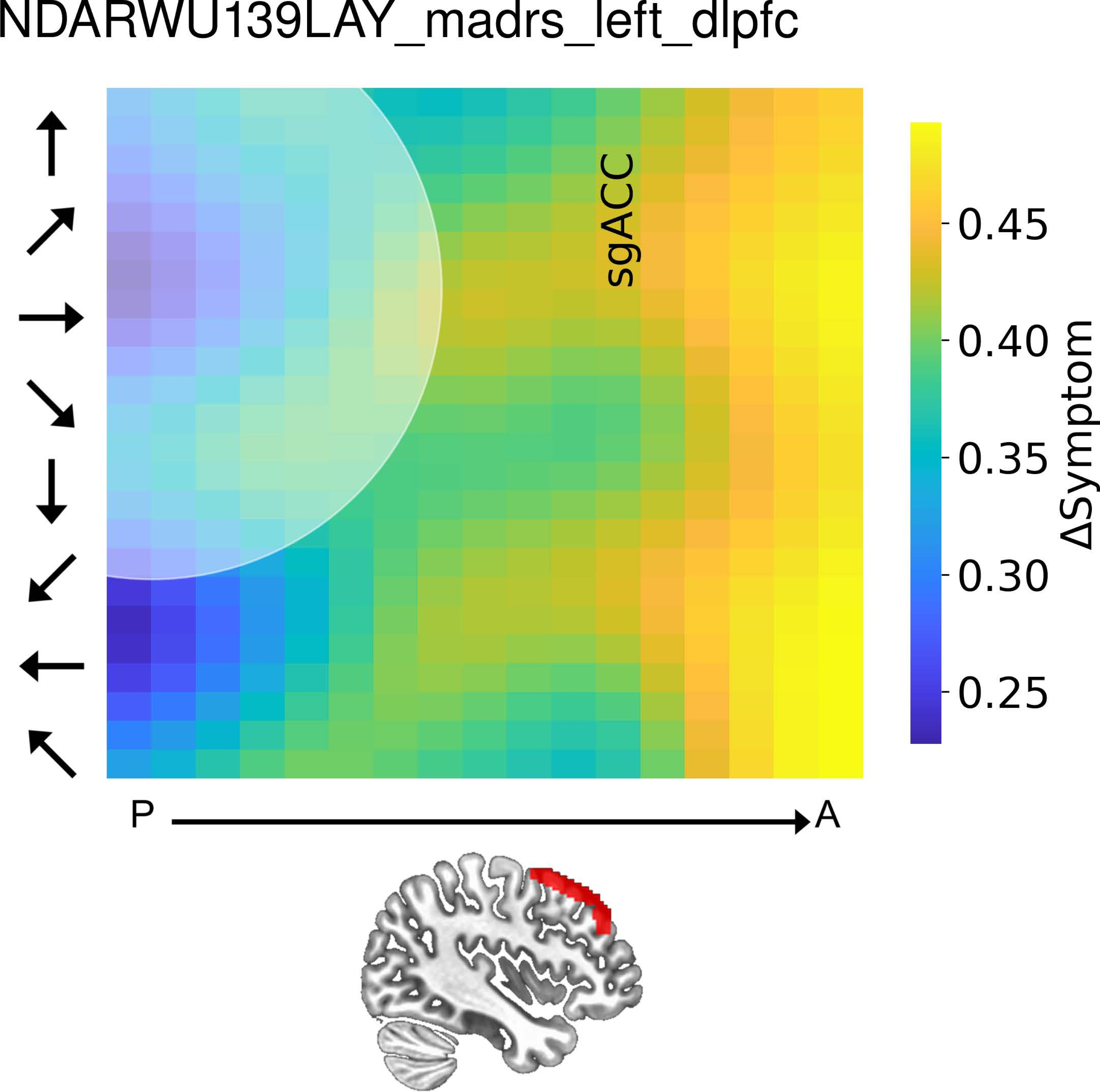

NDARWU139LAY_madrs_left_dlpfc
sgACC
0.45
0.40
0.35
0.30
0.25
ΔSymptom
P
A

NDARXH480BBY_madrs_left_dlpfc

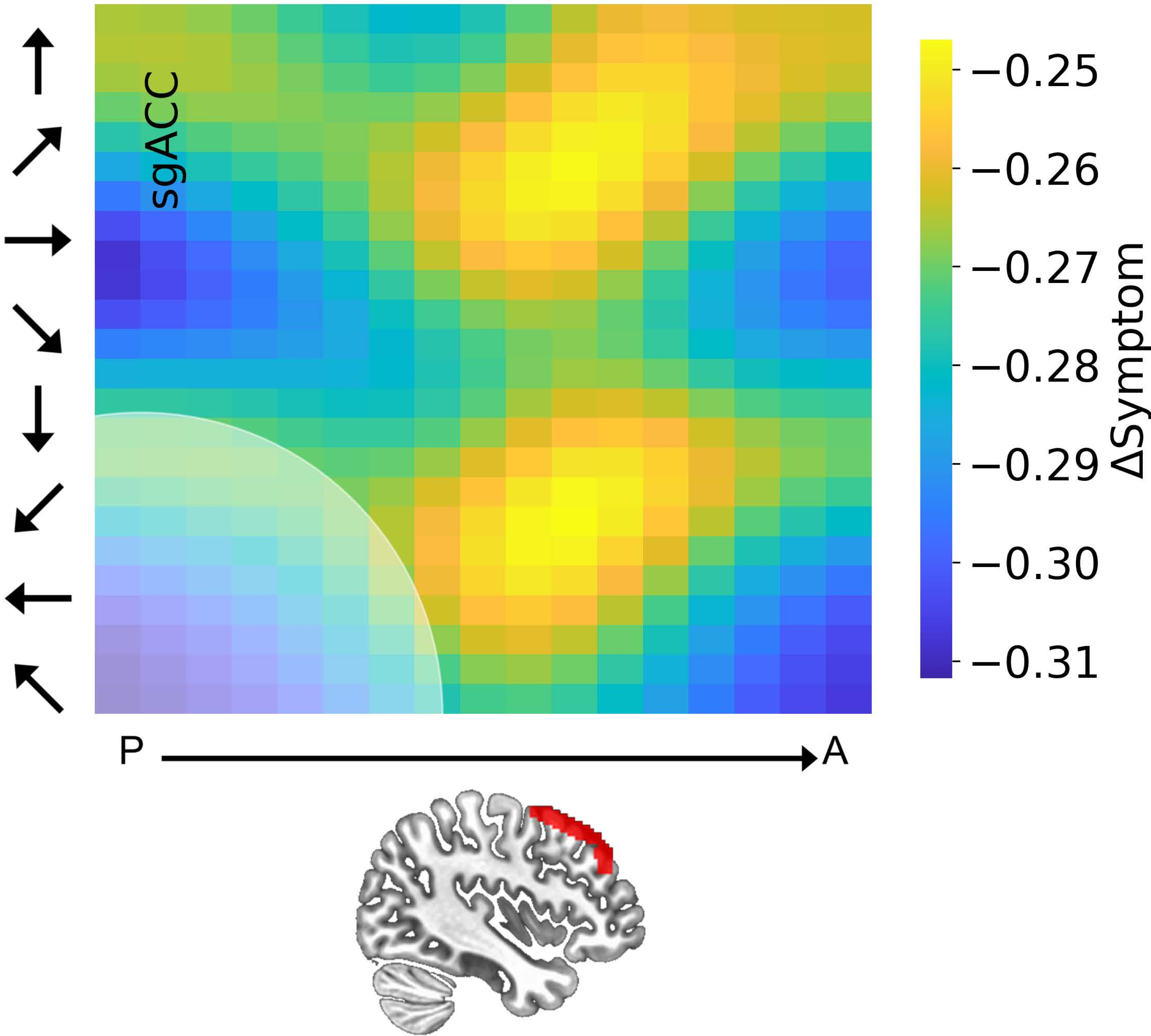

NDARXU558HYG_madrs_left_dlpfc

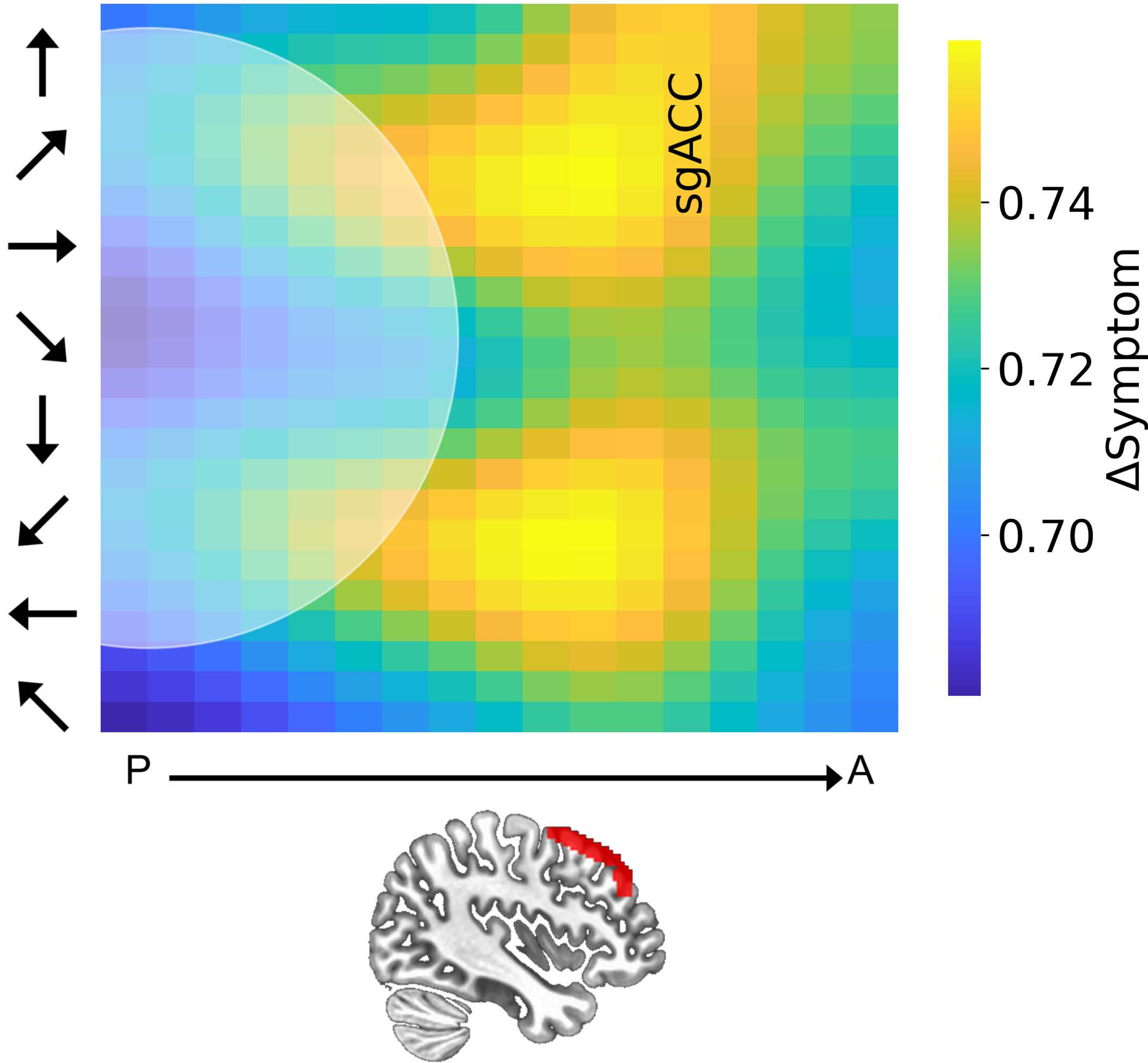

NDARZW884MZC_madrs_left_dlpfc

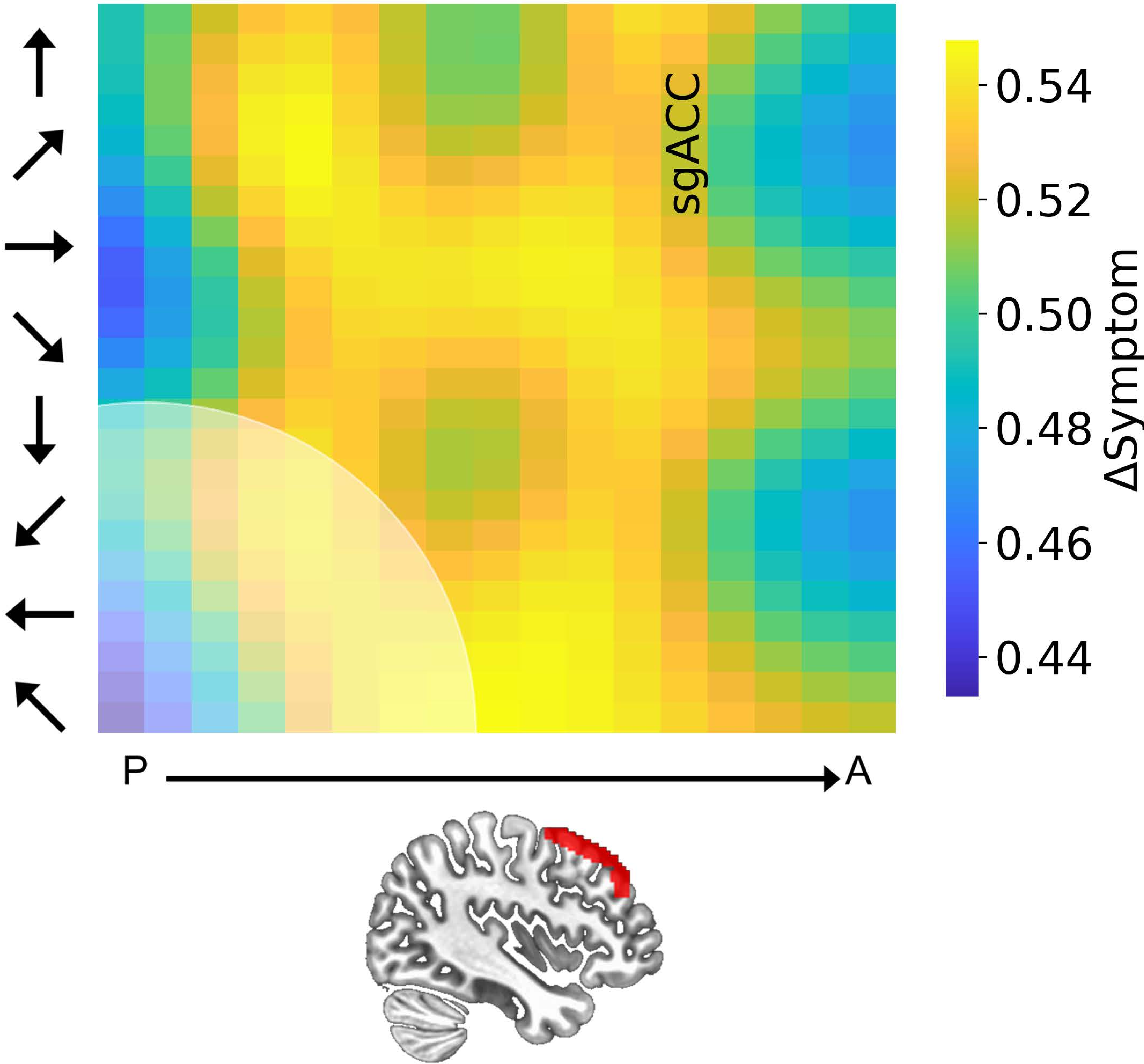

NDARAC286ZE7_madrs_right_dlpfc

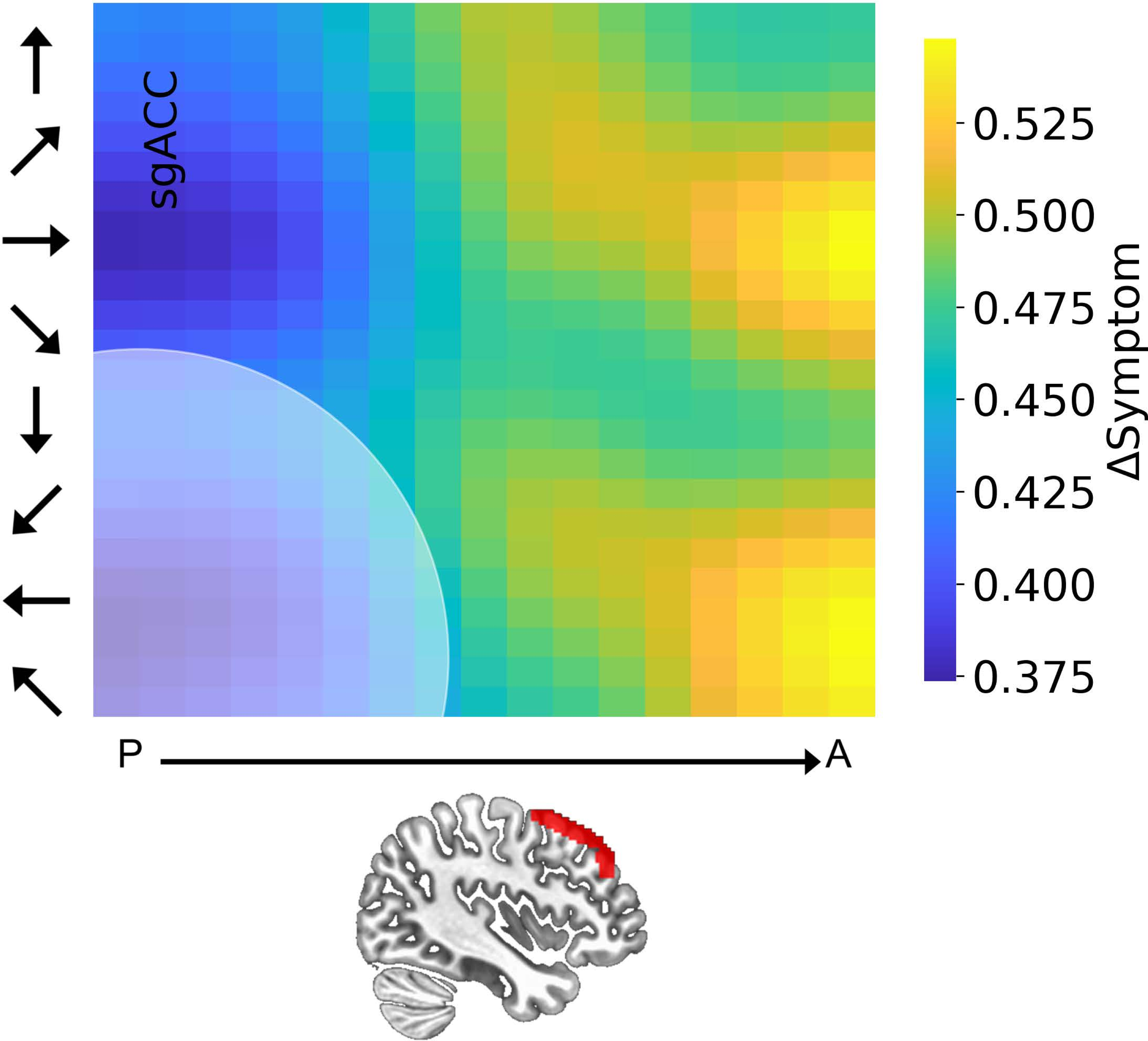

NDARAC789HEZ_madrs_right_dlpfc

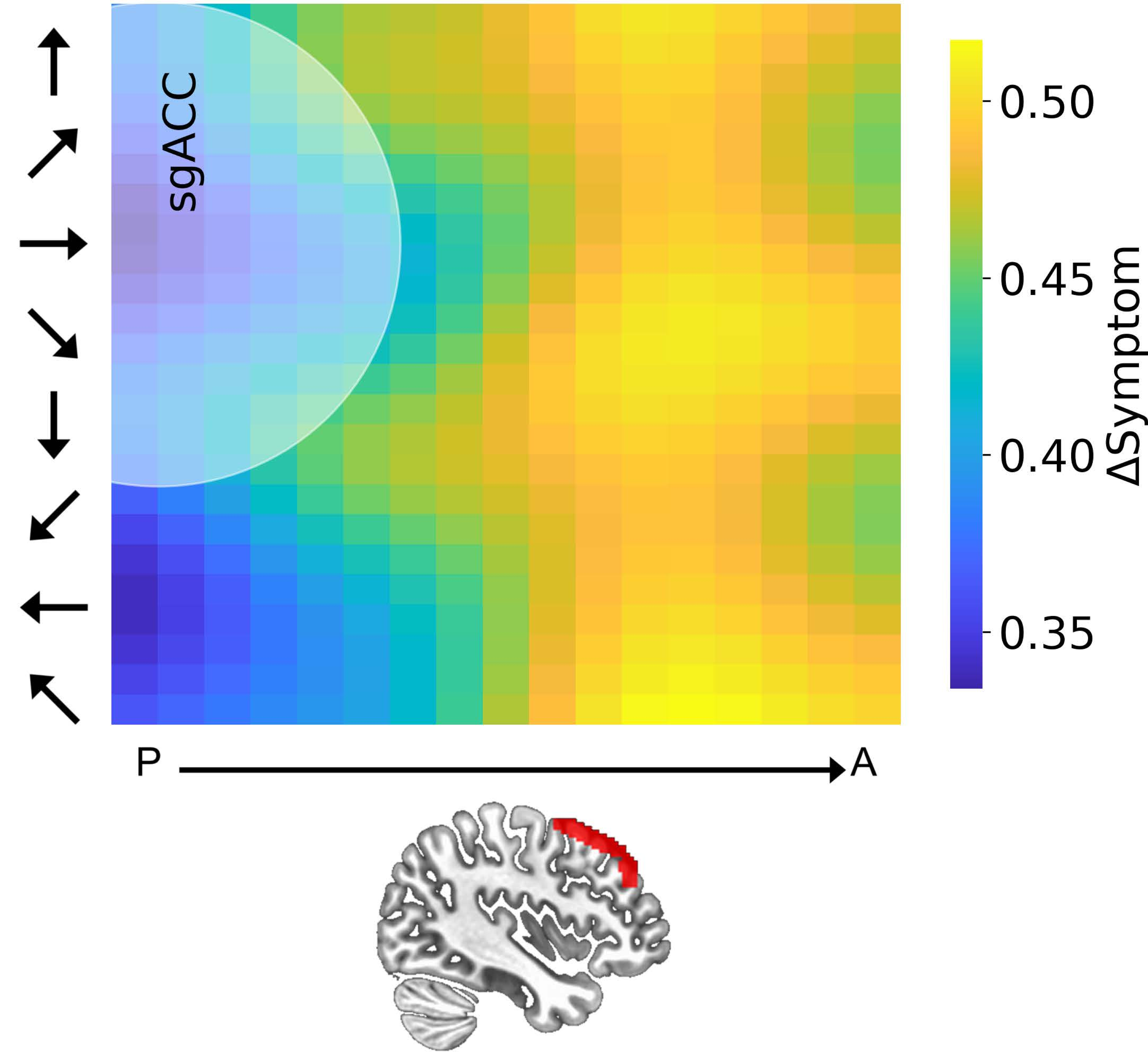

NDARBD224PBF_madrs_right_dlpfc

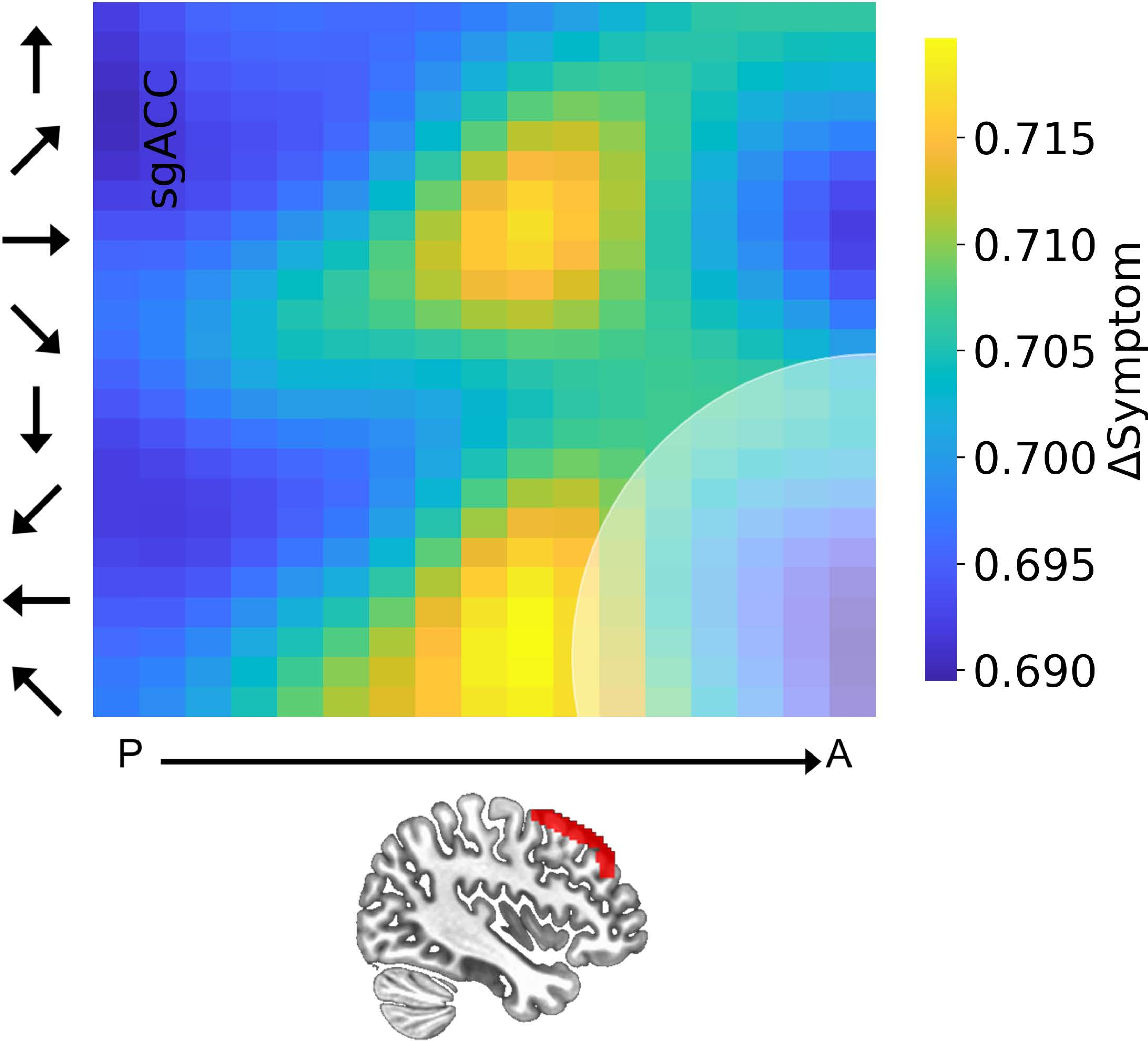

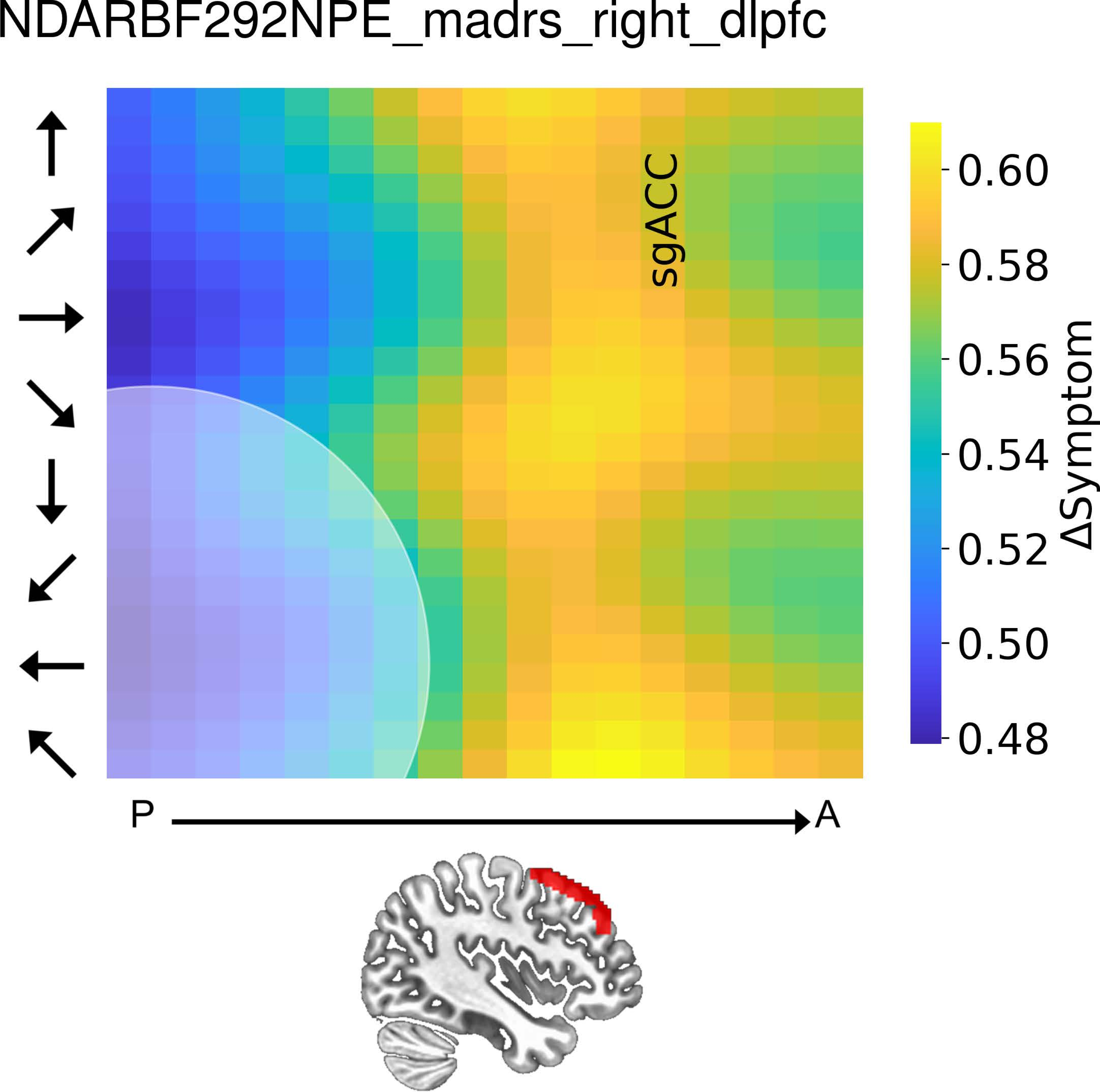
NDARBF292NPE_madrs_right_dlpfc
sgACC
P
A
ΔSymptom
0.60
0.58
0.56
0.54
0.52
0.50
0.48

NDARCG535CPC_madrs_right_dlpfc

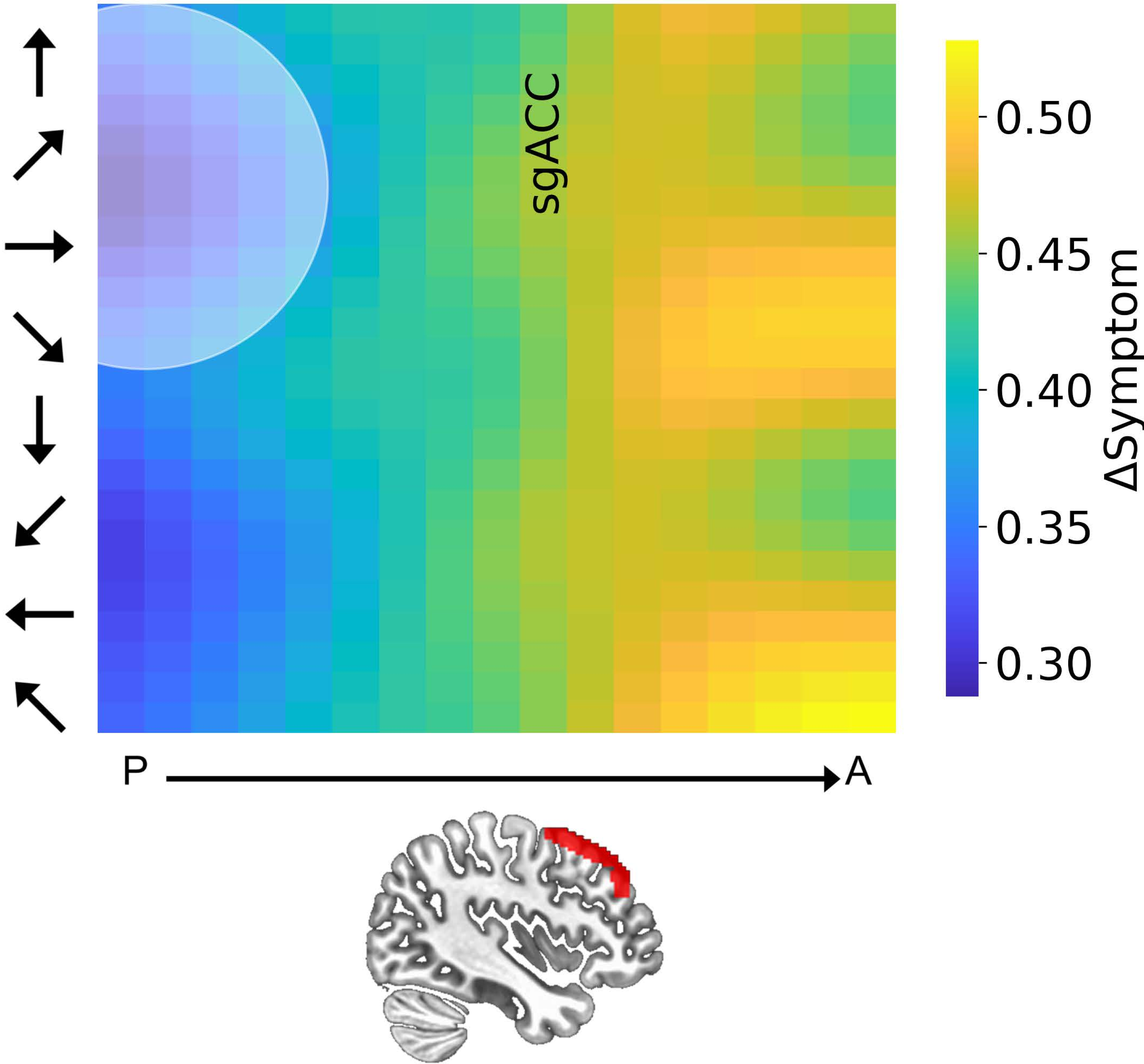

NDARCY406TU1_madrs_right_dlpfc

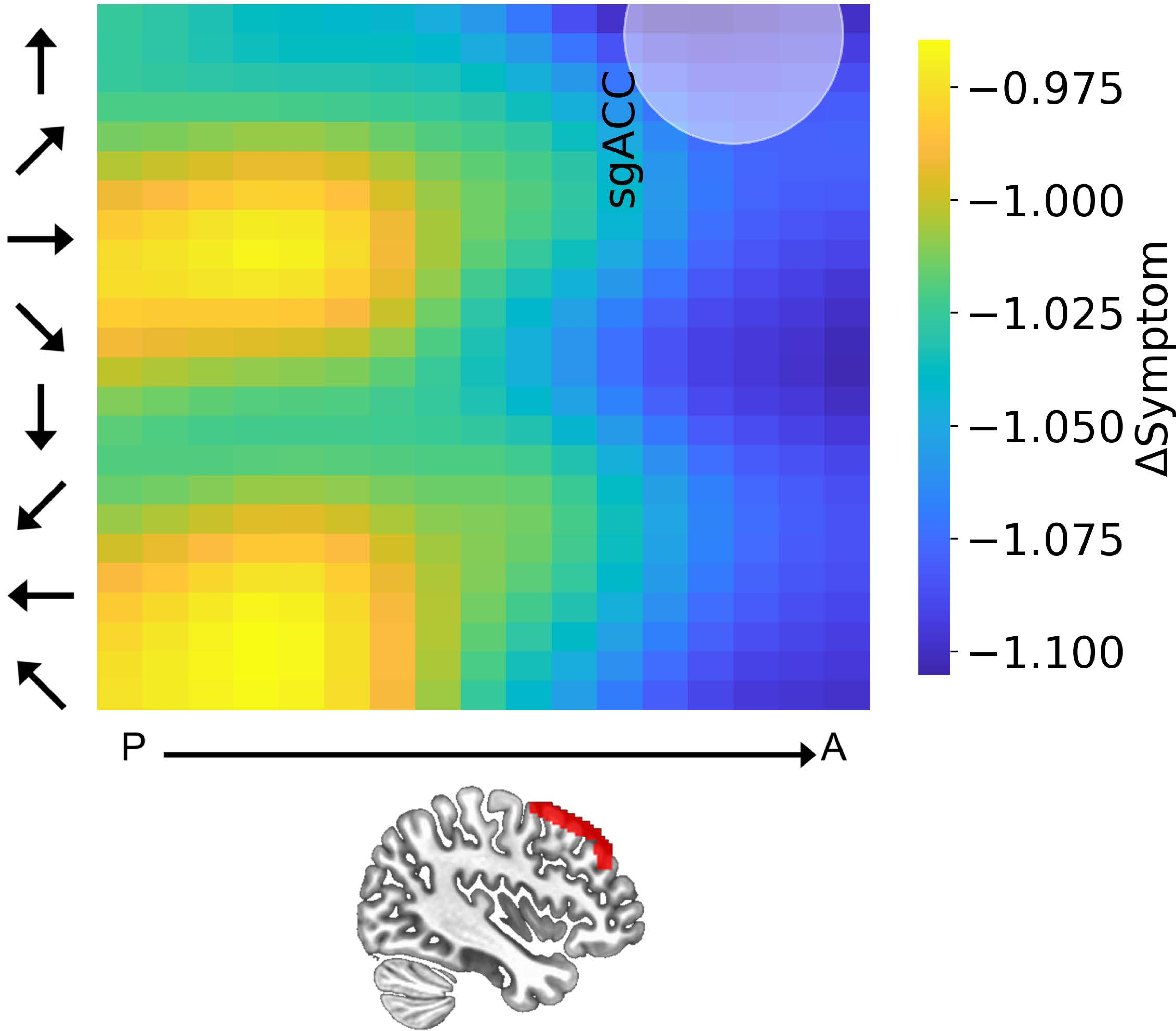

NDARDY771TXZ_madrs_right_dlpfc

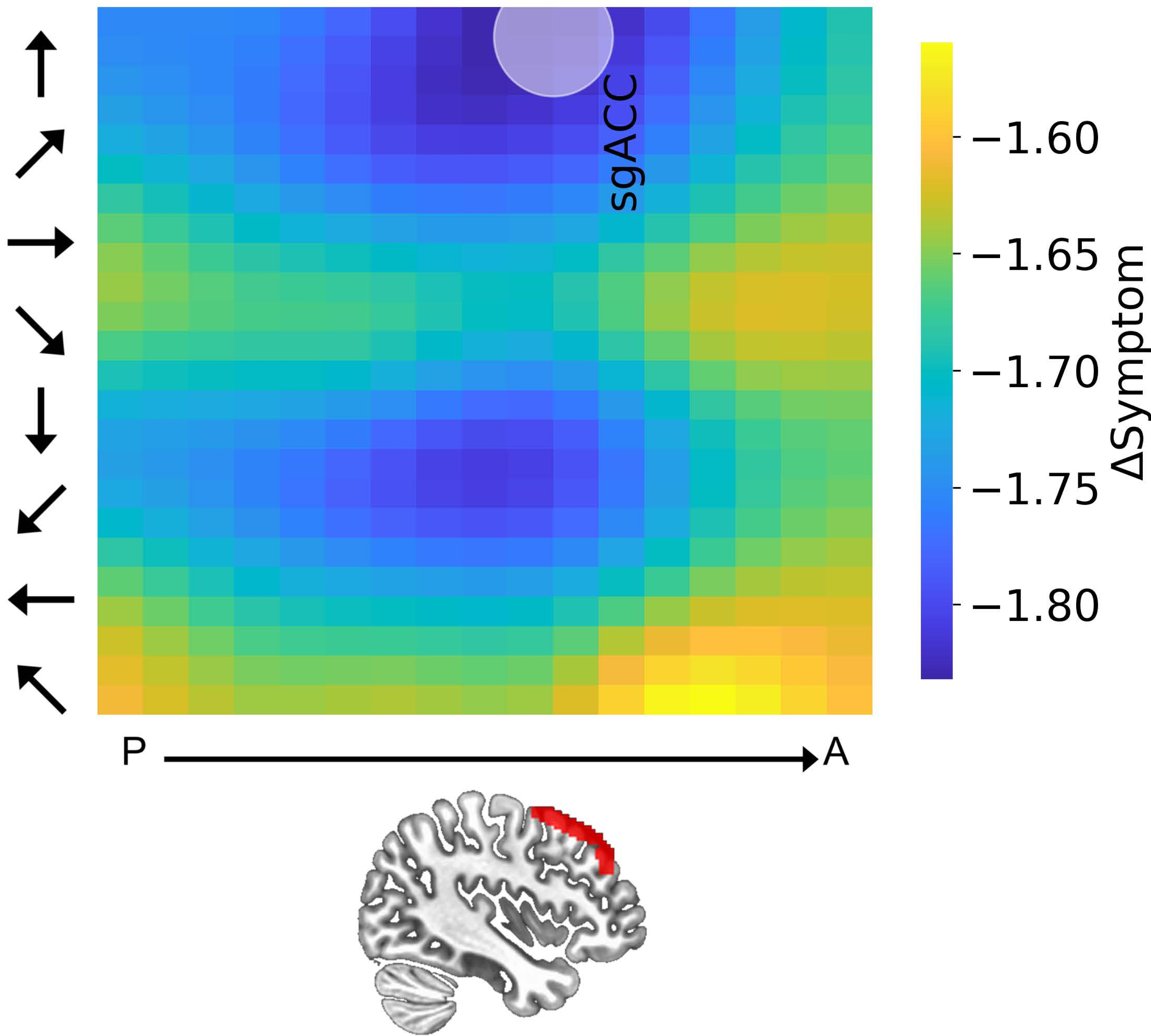

NDAREU790BVQ_madrs_right_dlpfc

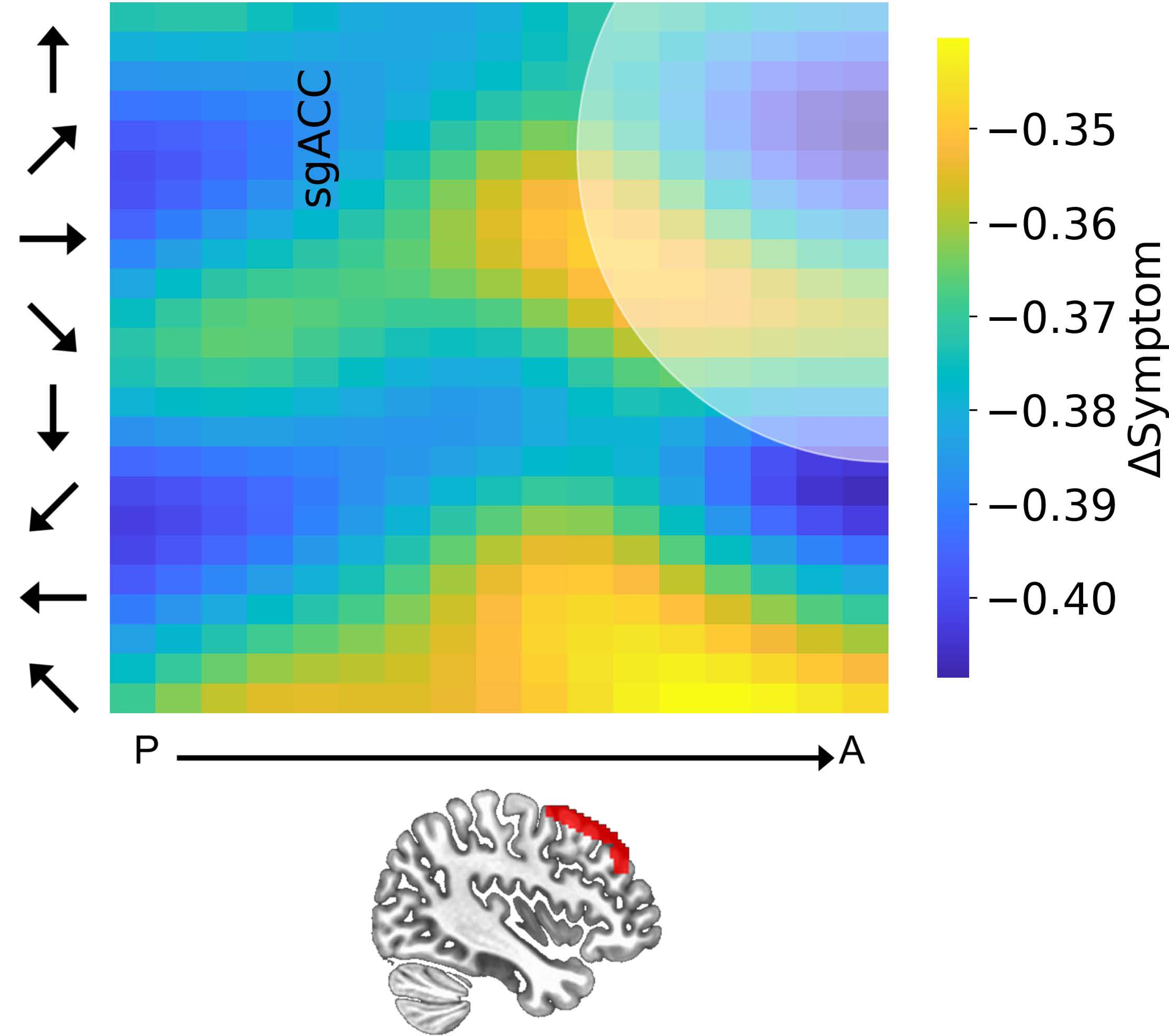

NDARGA844BF5_madrs_right_dlpfc

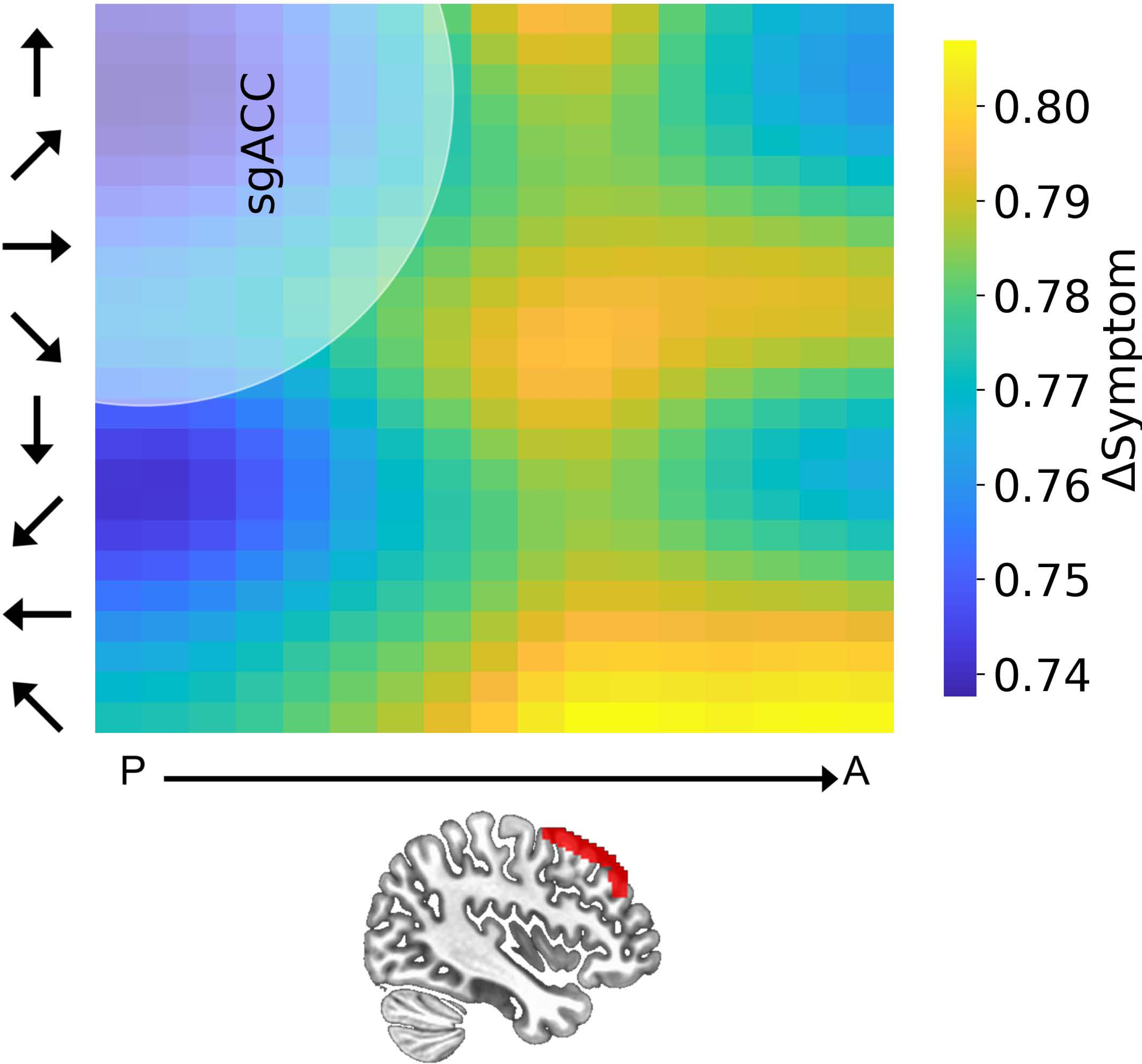

NDARGN596ENP_madrs_right_dlpfc

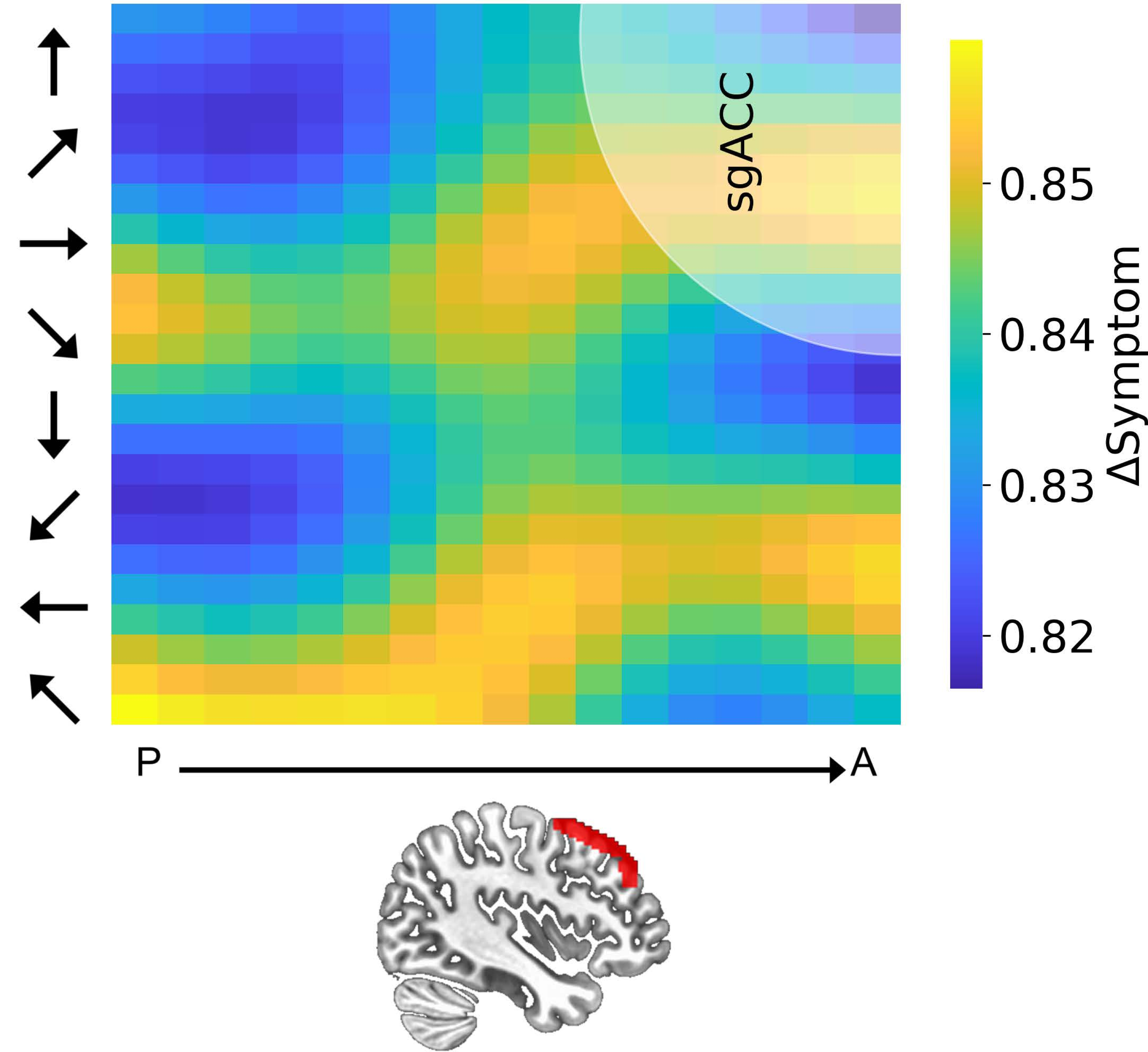

NDARHU677DFF_madrs_right_dlpfc

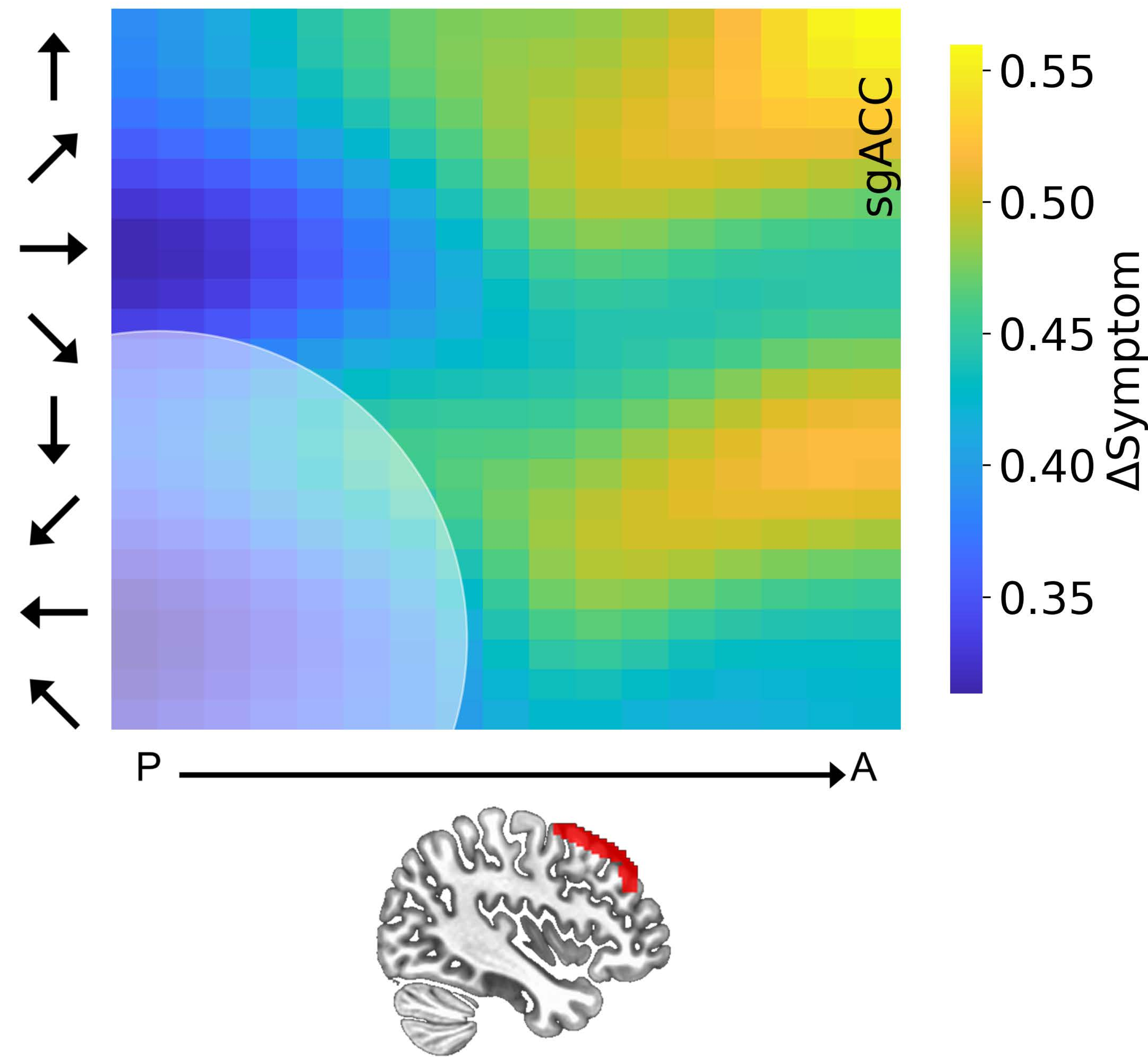

NDARJL083WMP_madrs_right_dlpfc

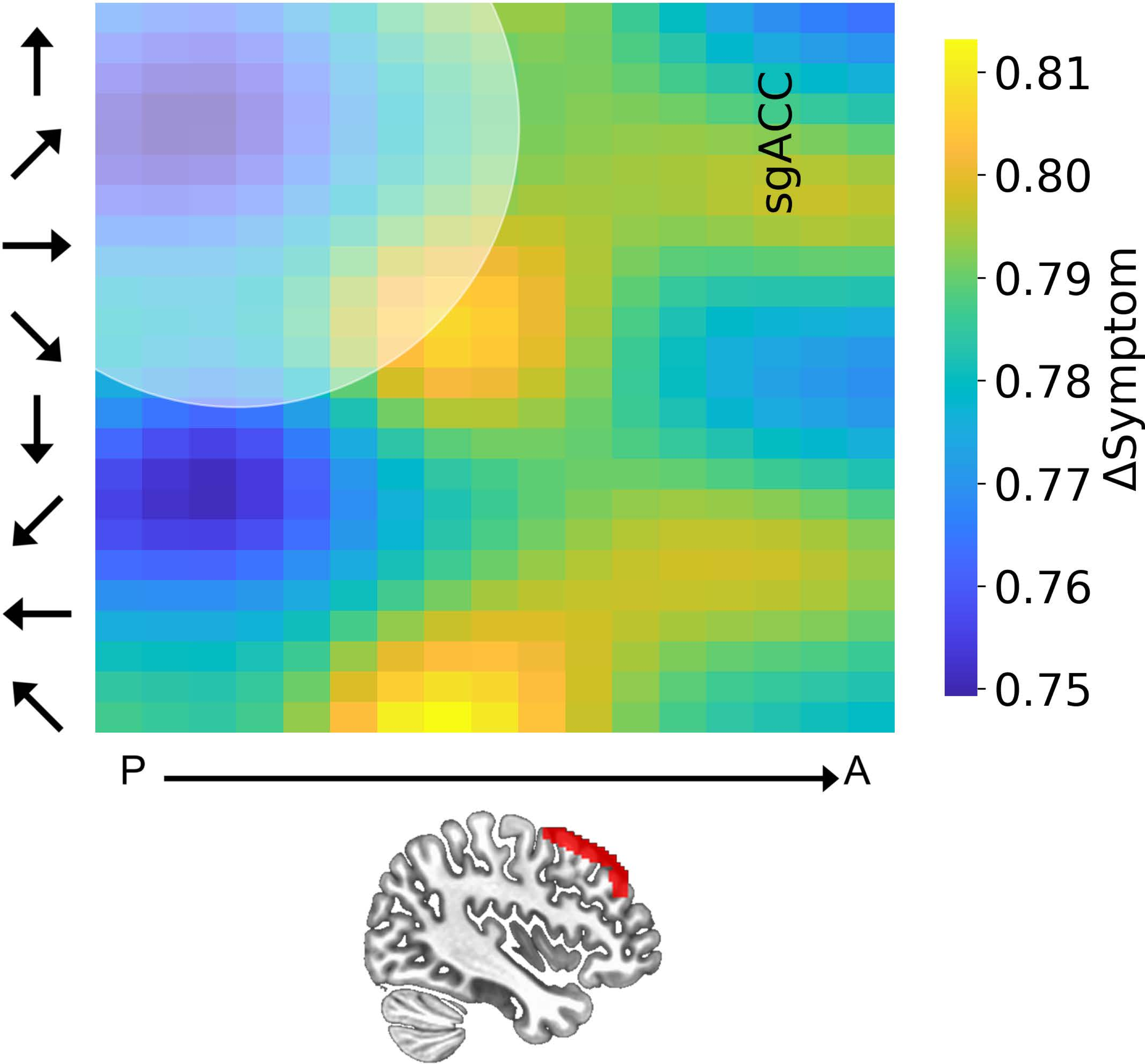

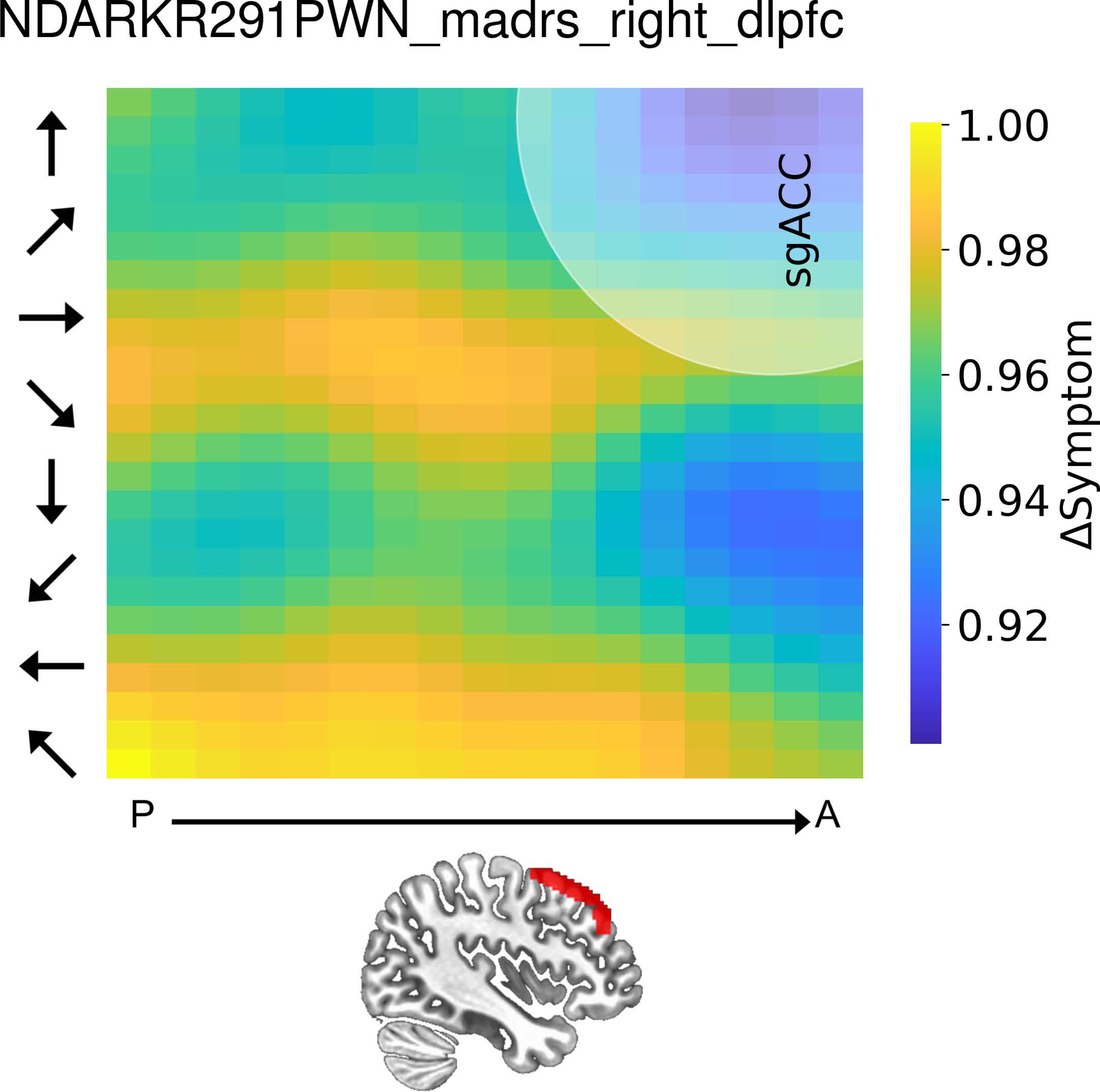
NDARKR291PWN_madrs_right_dlpfc
sgACC
1.00
0.98
0.96
0.94
0.92
ΔSymptom
P
A

NDARLH479MBF_madrs_right_dlpfc

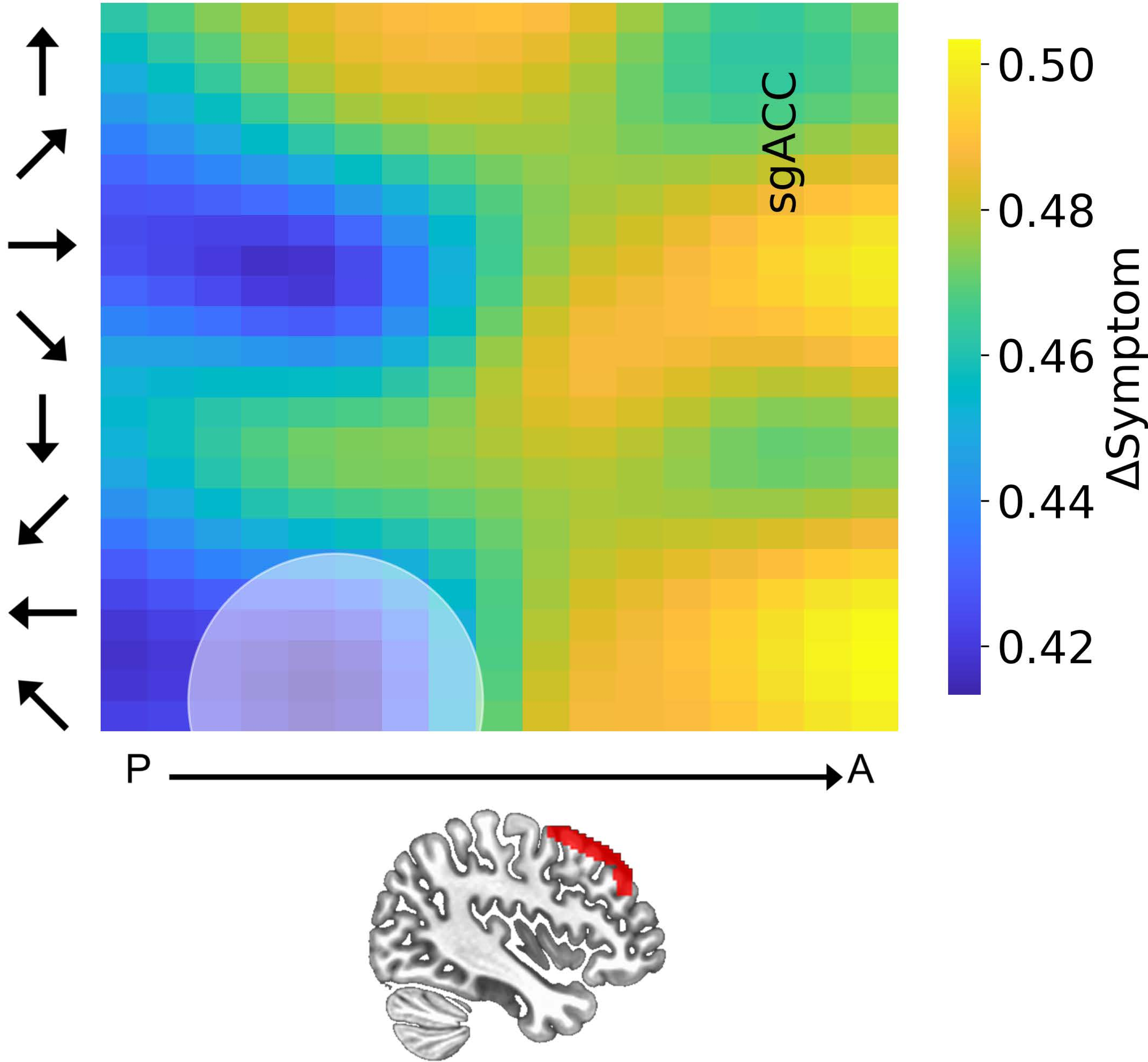

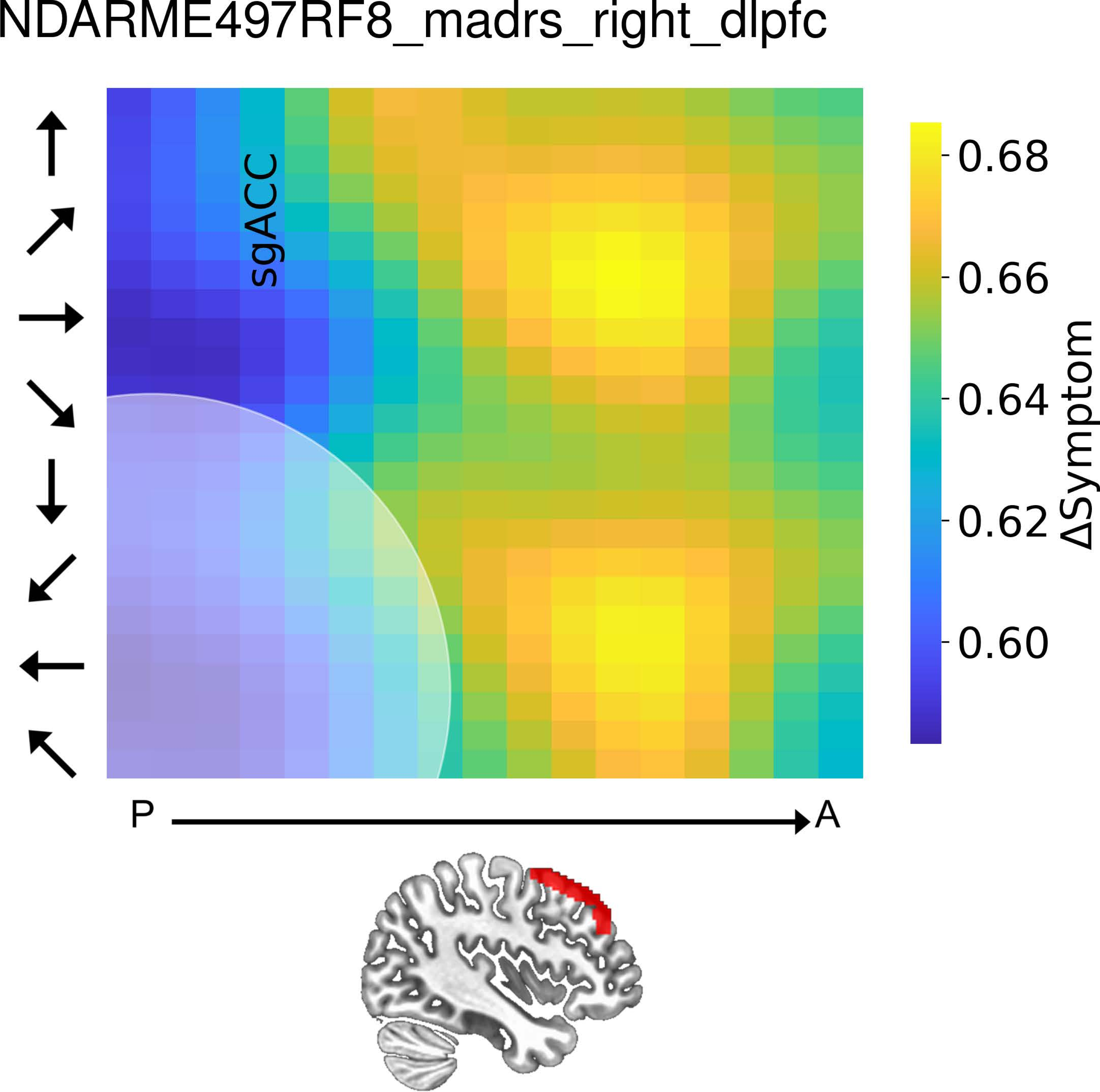

NDARME497RF8_madrs_right_dlpfc
sgACC
0.68
0.66
0.64
0.62
0.60
ΔSymptom
P
A

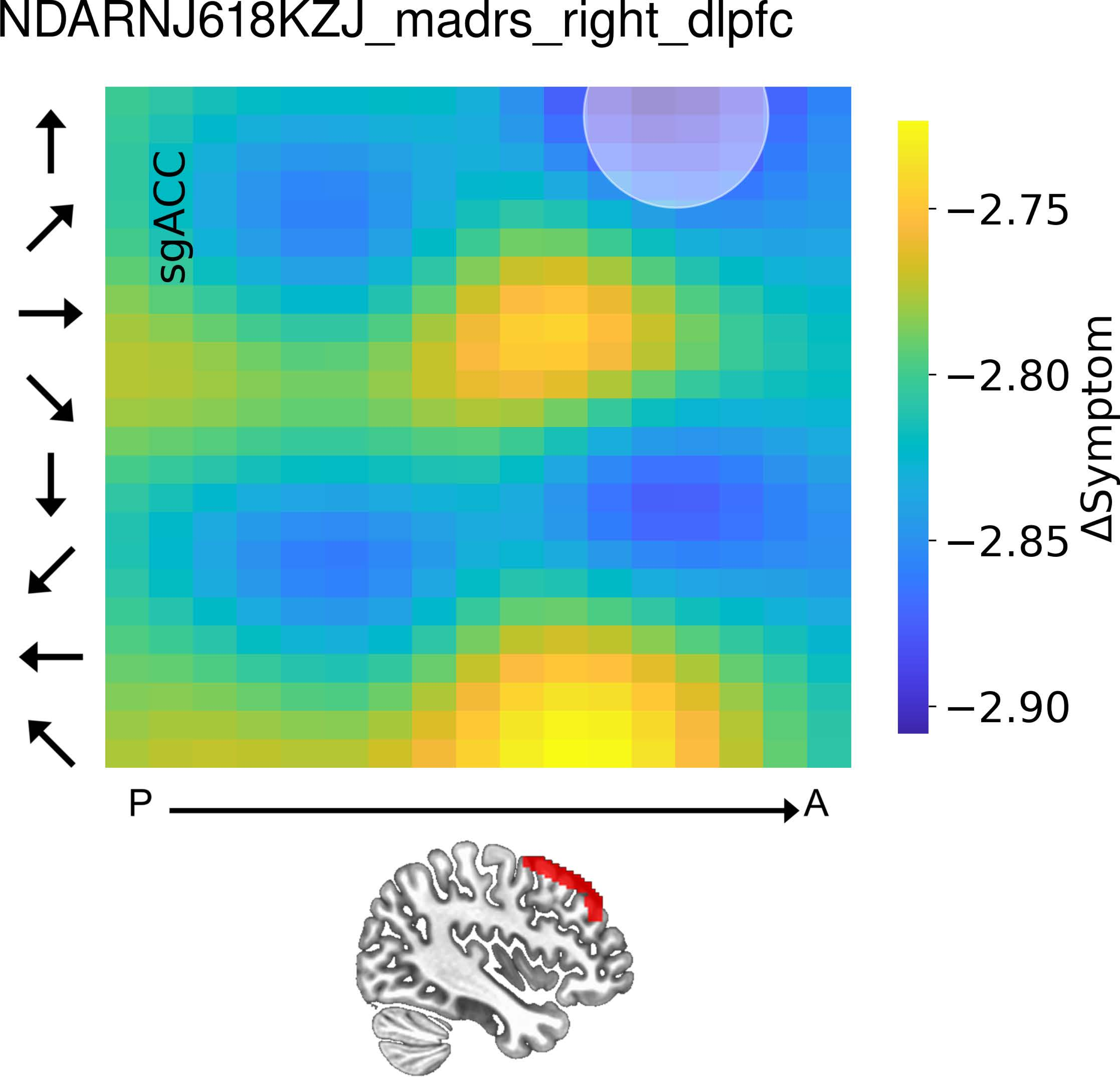

NDARNJ618KZJ_madrs_right_dlpfc
sgACC
−2.75
−2.80
−2.85
−2.90
ΔSymptom
P
A

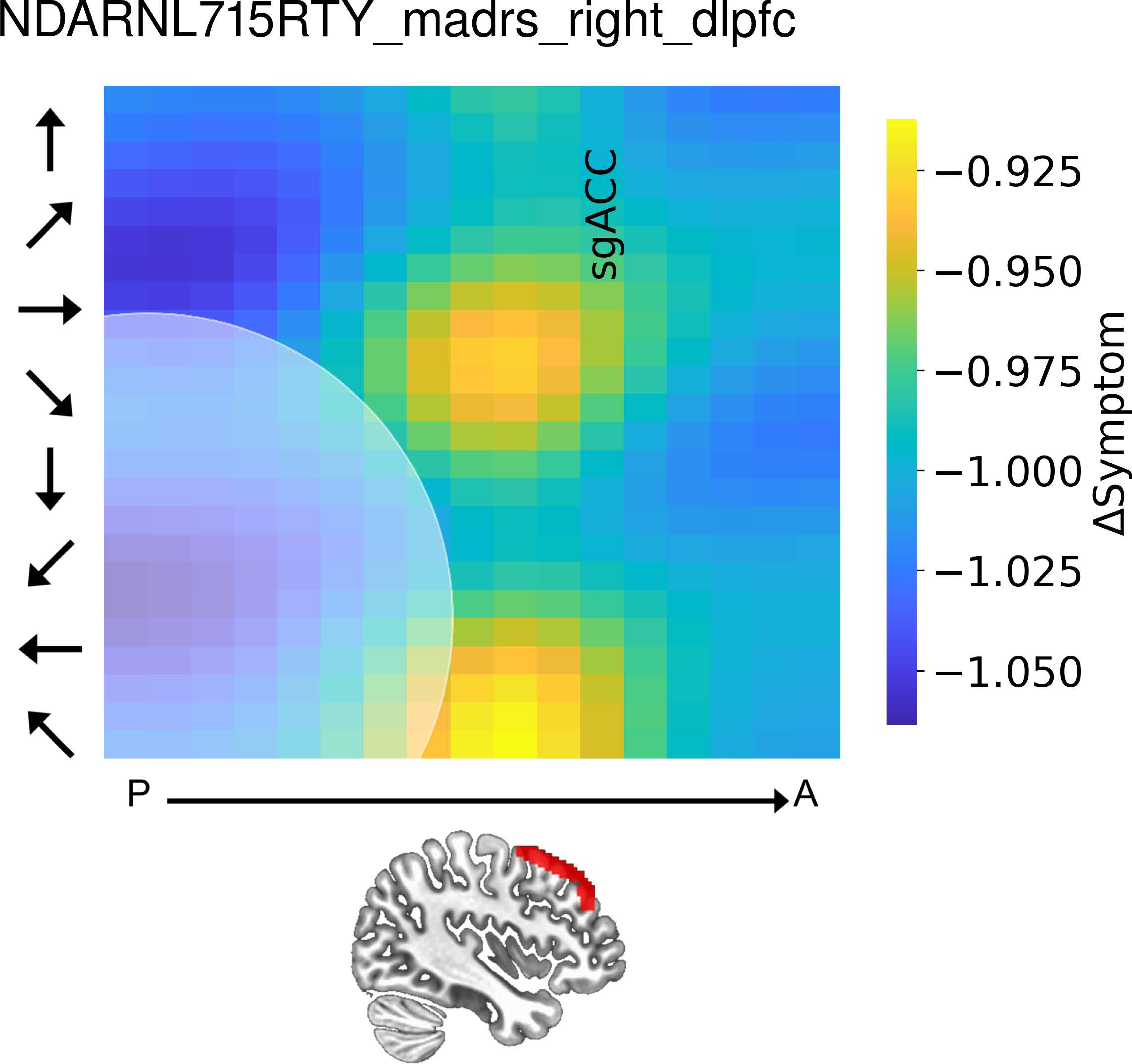

NDARNL715RTY_madrs_right_dlpfc
sgACC
−0.925
−0.950
−0.975
−1.000
−1.025
−1.050
ΔSymptom
P
A

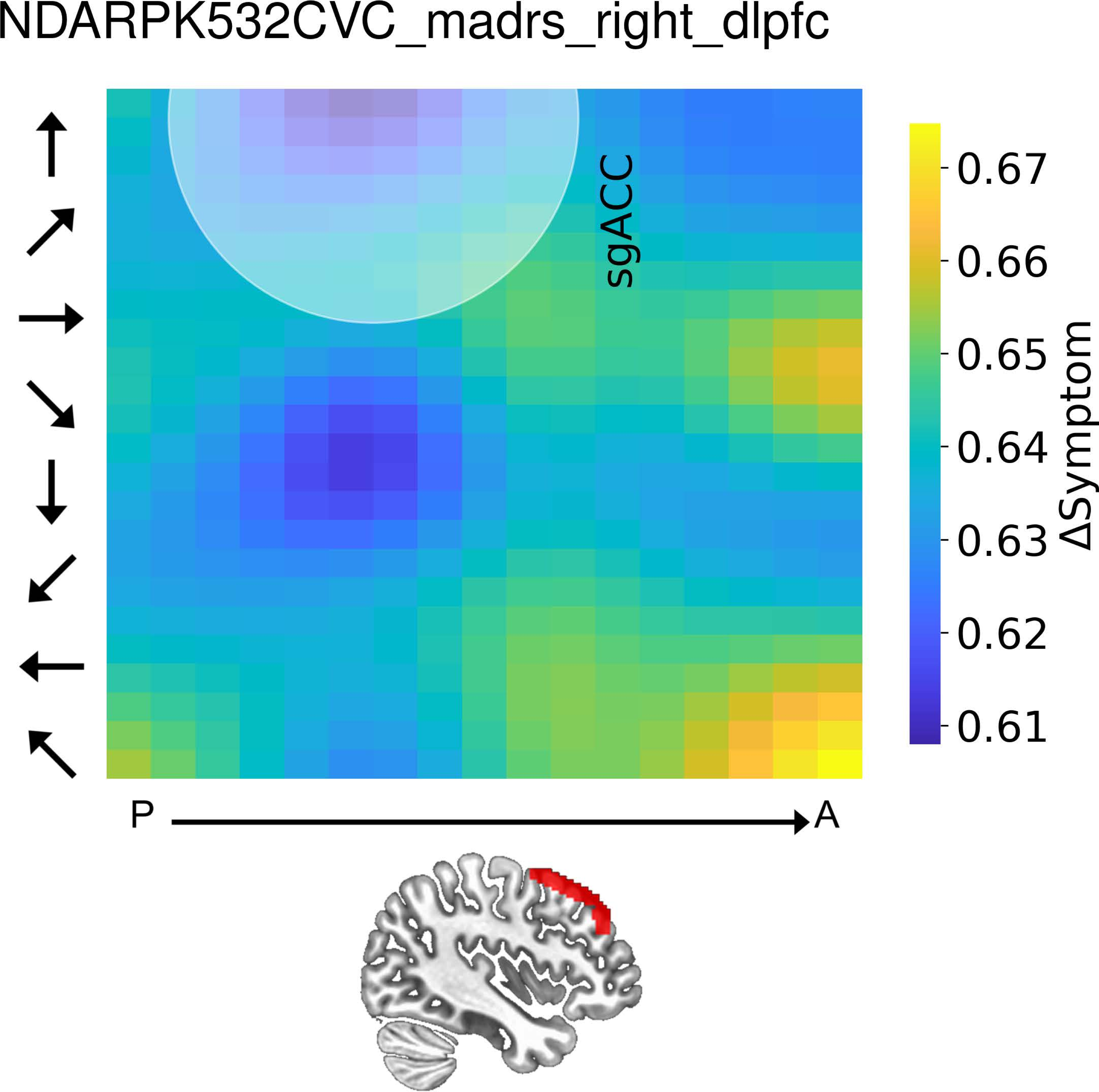

NDARPK532CVC_madrs_right_dlpfc
sgACC
0.67
0.66
0.65
0.64
0.63
0.62
0.61
ΔSymptom
P
A

NDARWK858FA2_madrs_right_dlpfc

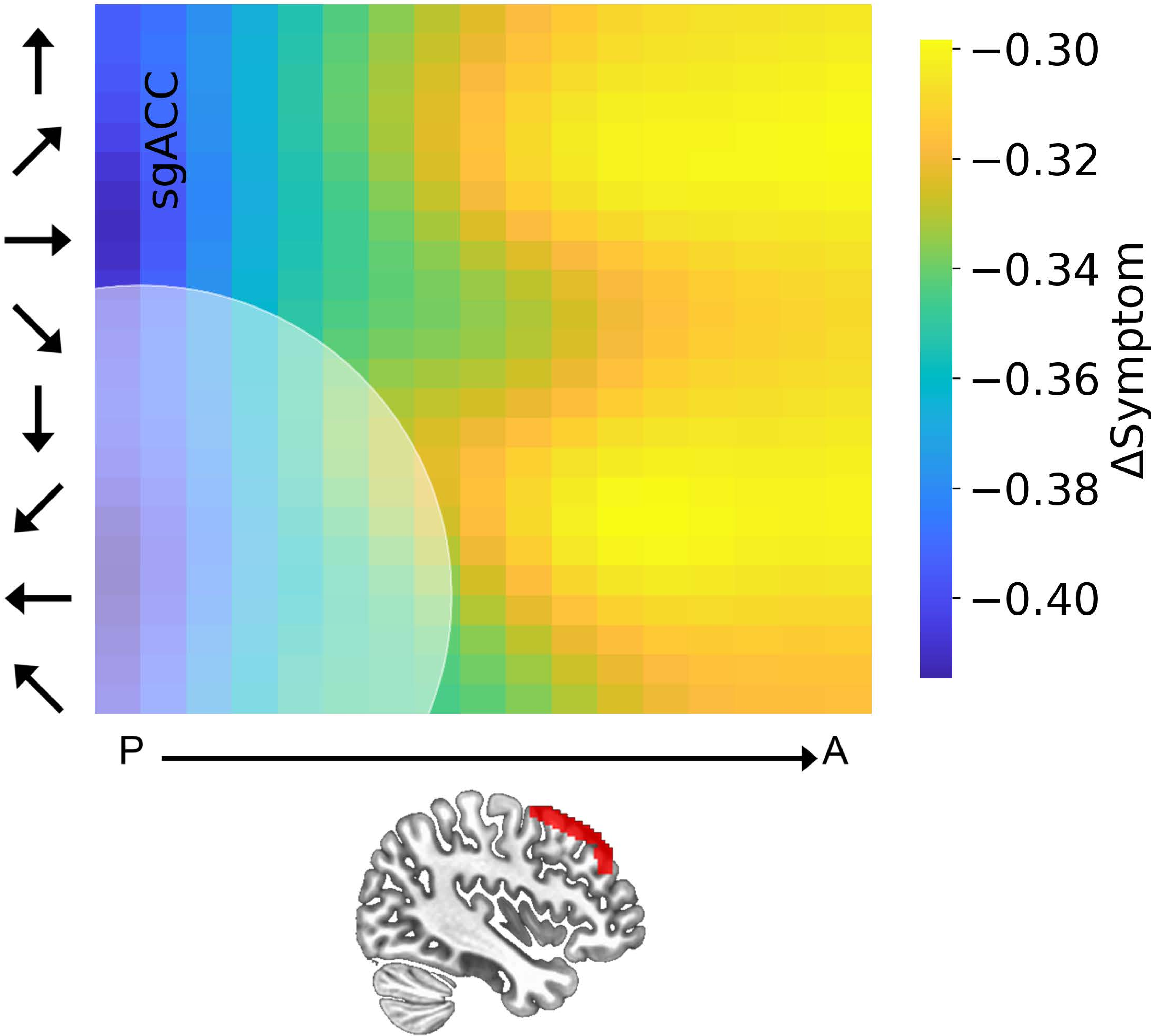

NDARWM071DRZ_madrs_right_dlpfc

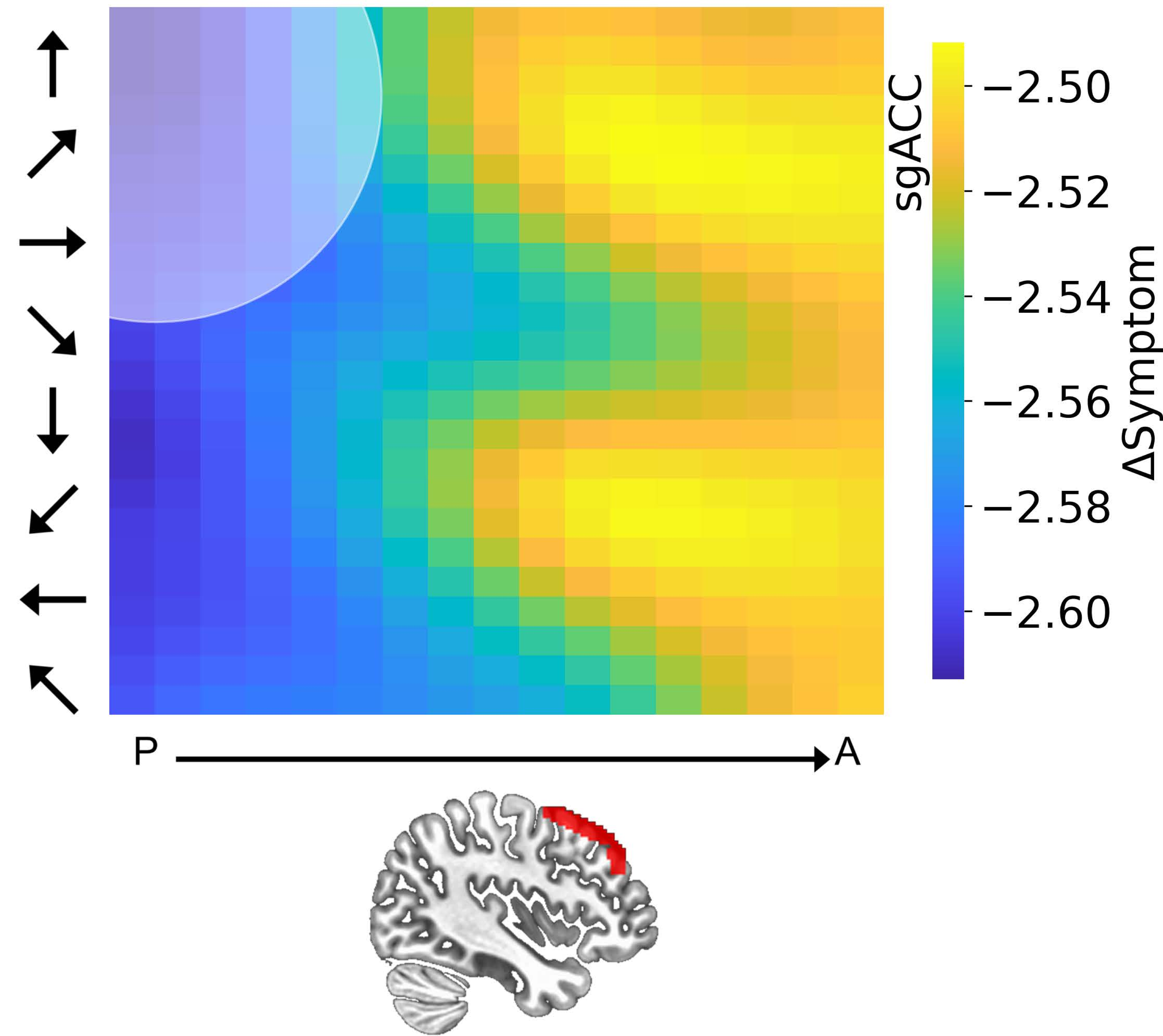

NDARWT226PU5_madrs_right_dlpfc

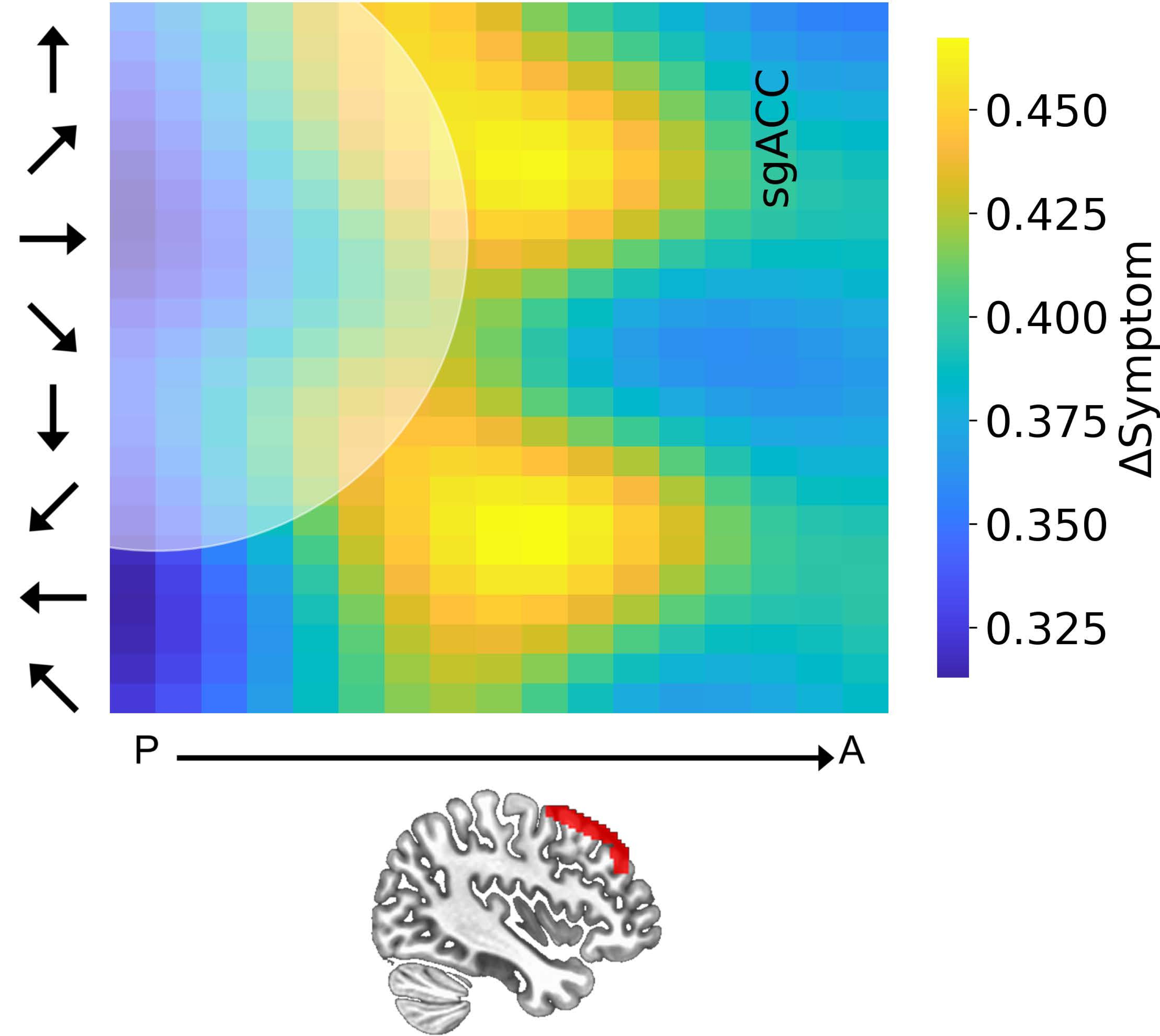

NDARWU139LAY_madrs_right_dlpfc

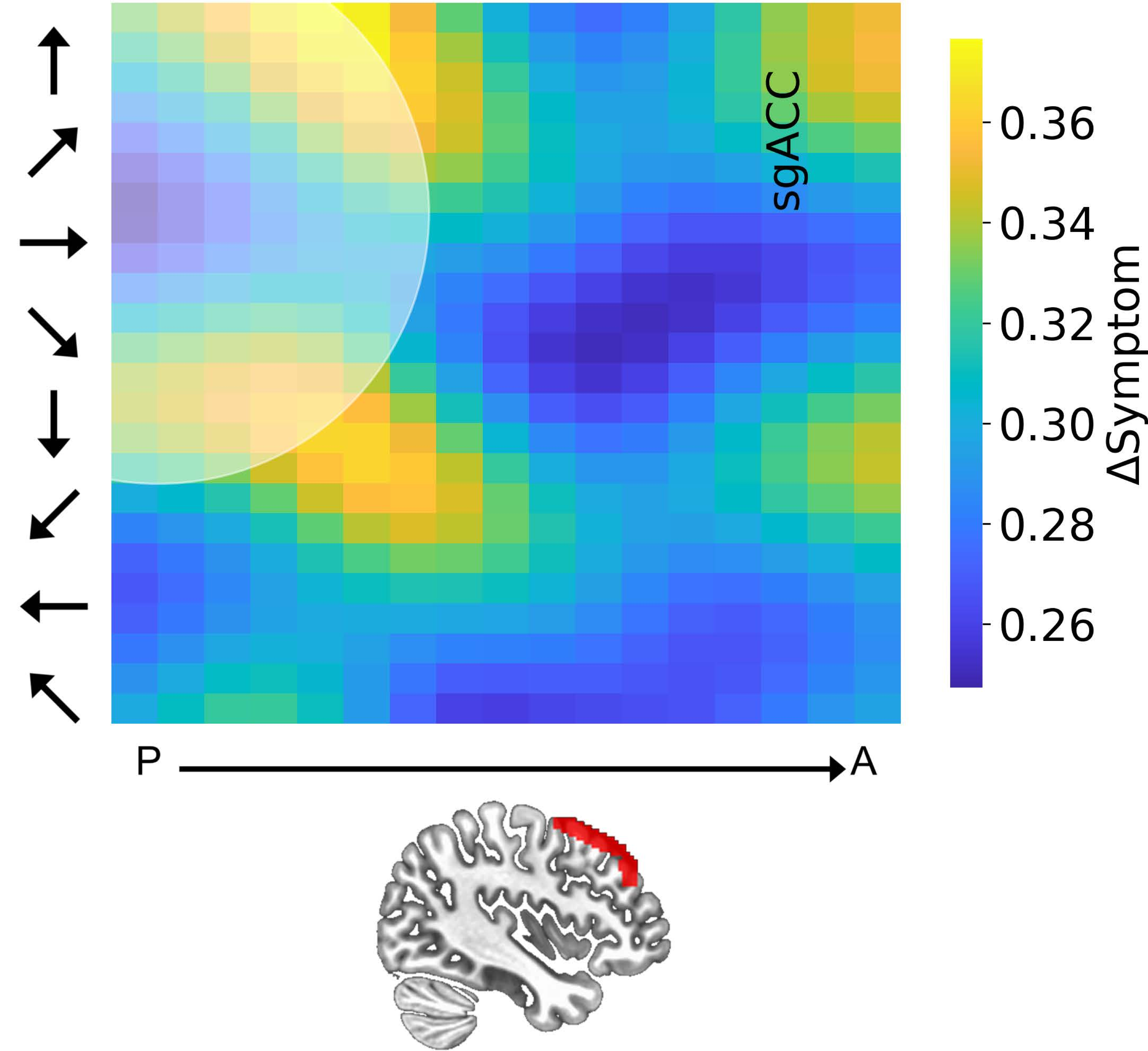

NDARXH480BBY_madrs_right_dlpfc

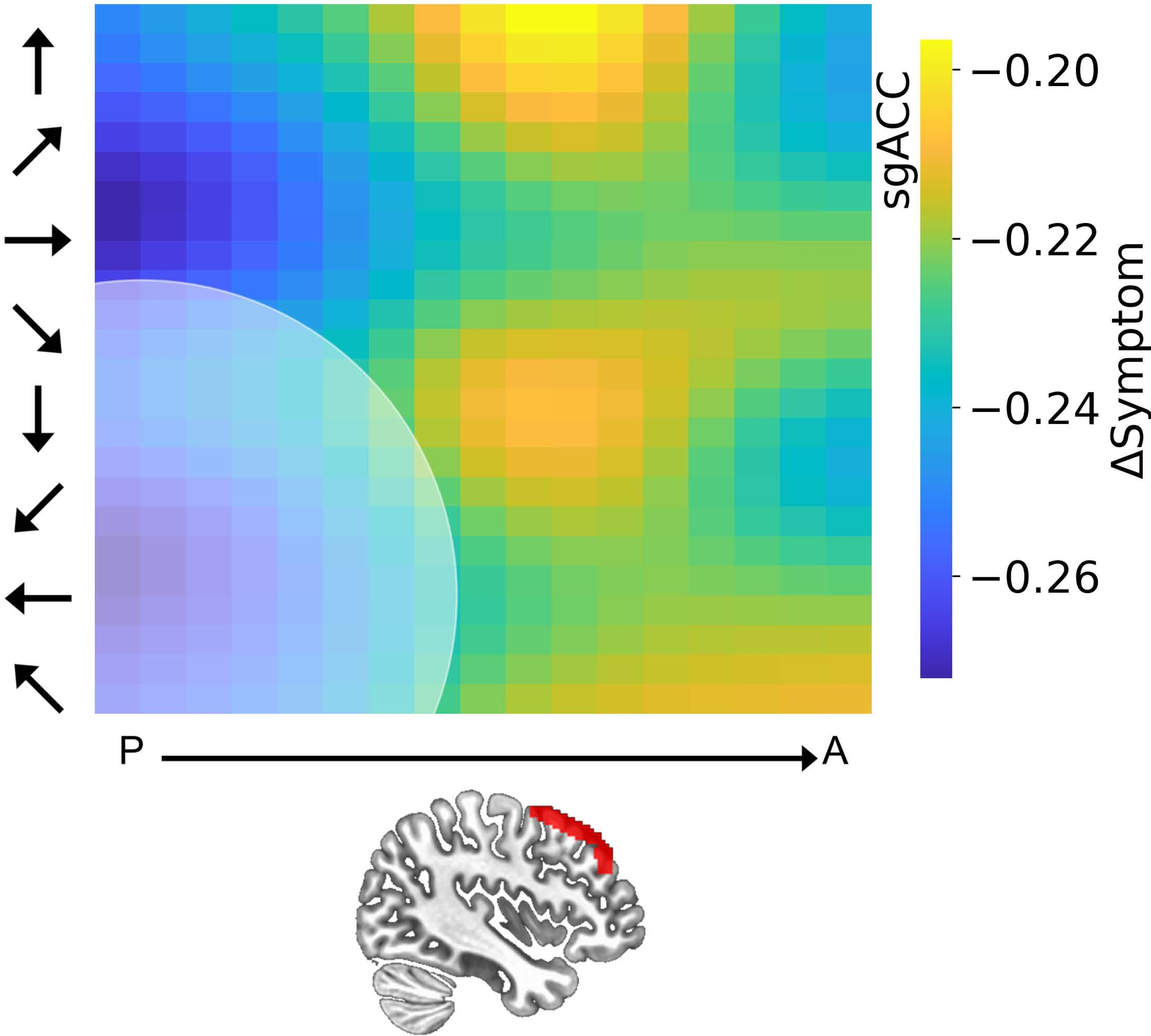

NDARXU558HYG_madrs_right_dlpfc

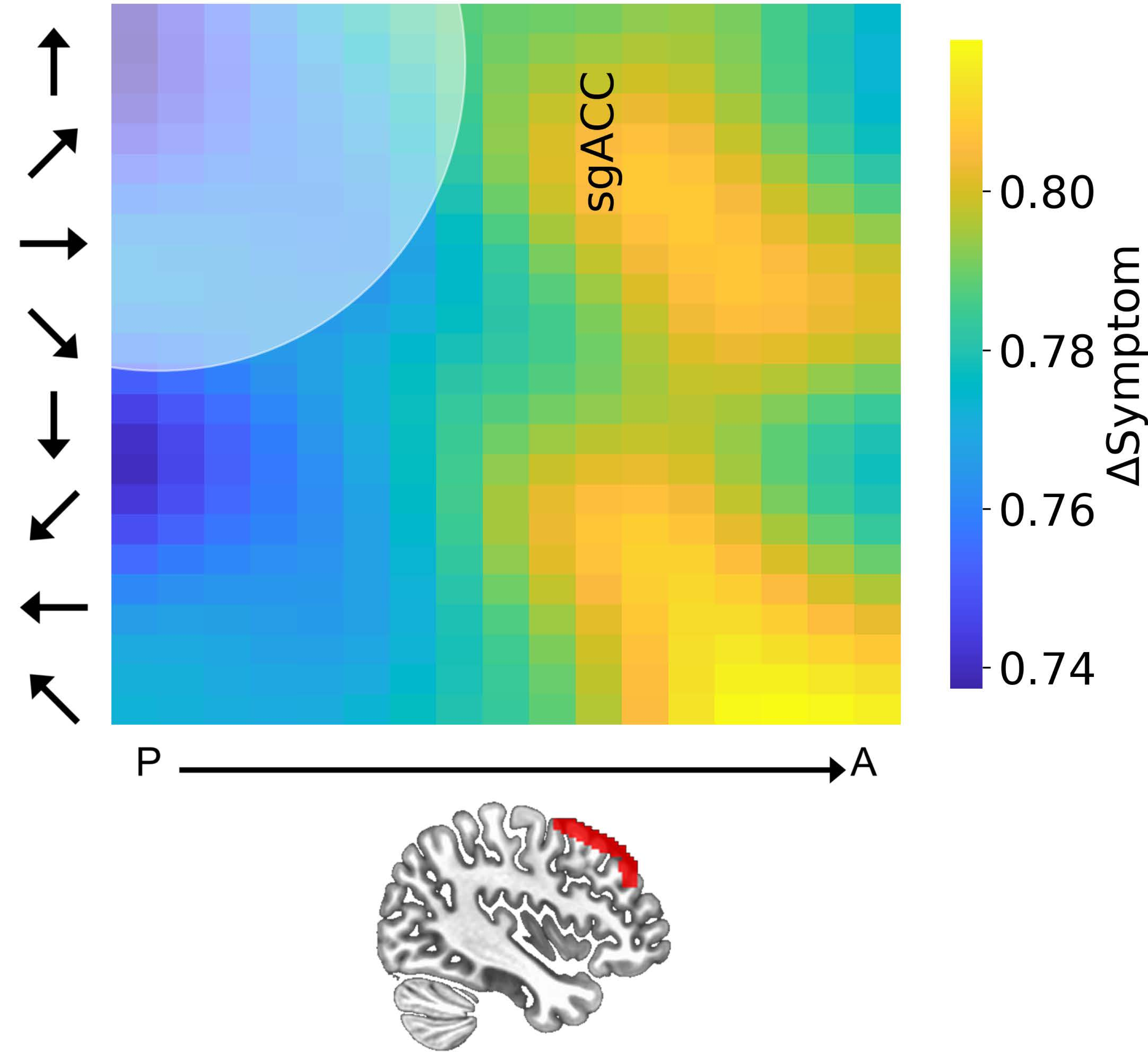

NDARZW884MZC_madrs_right_dlpfc

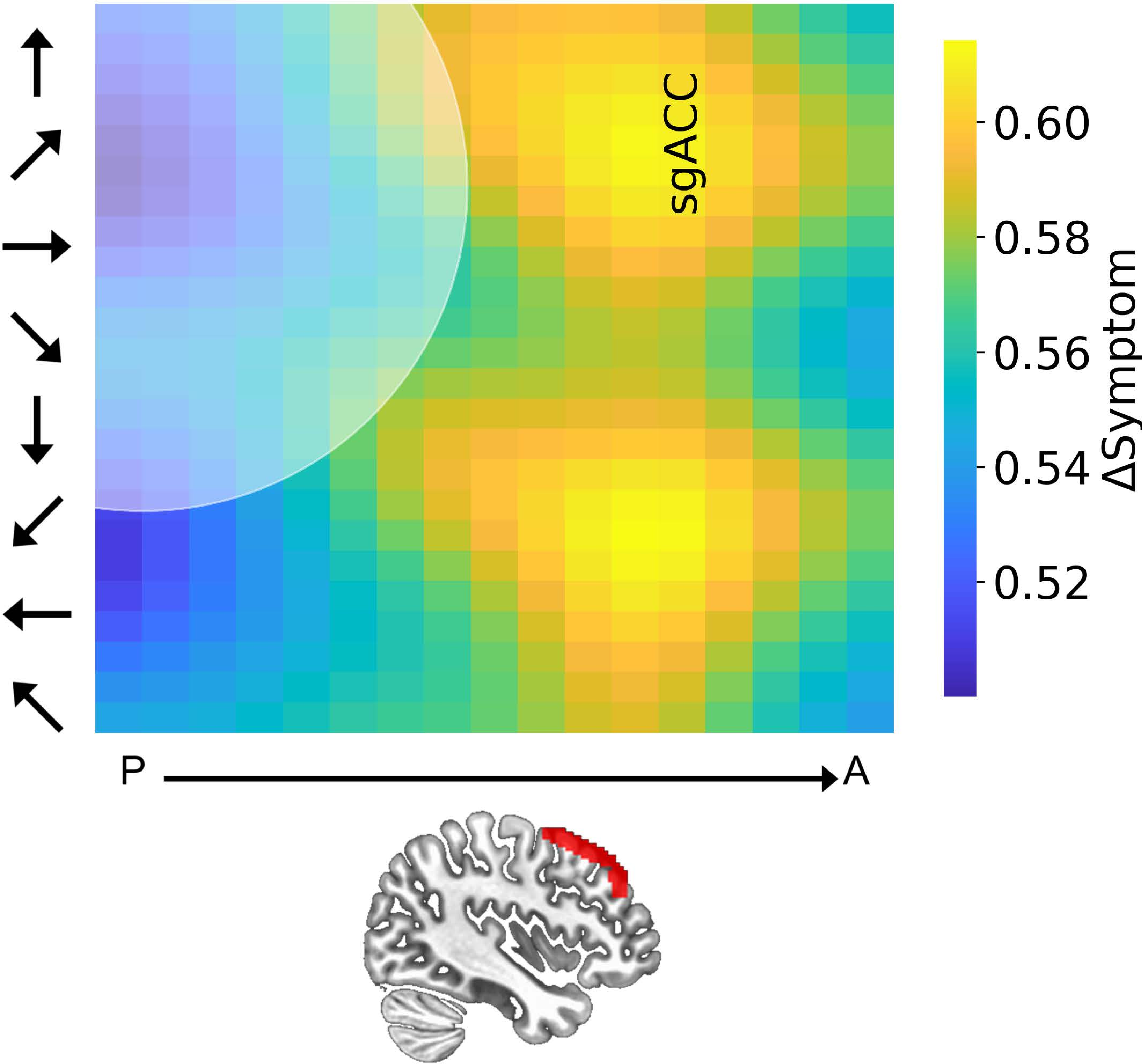

NDARAC286ZE7_hamd_left_dlpfc

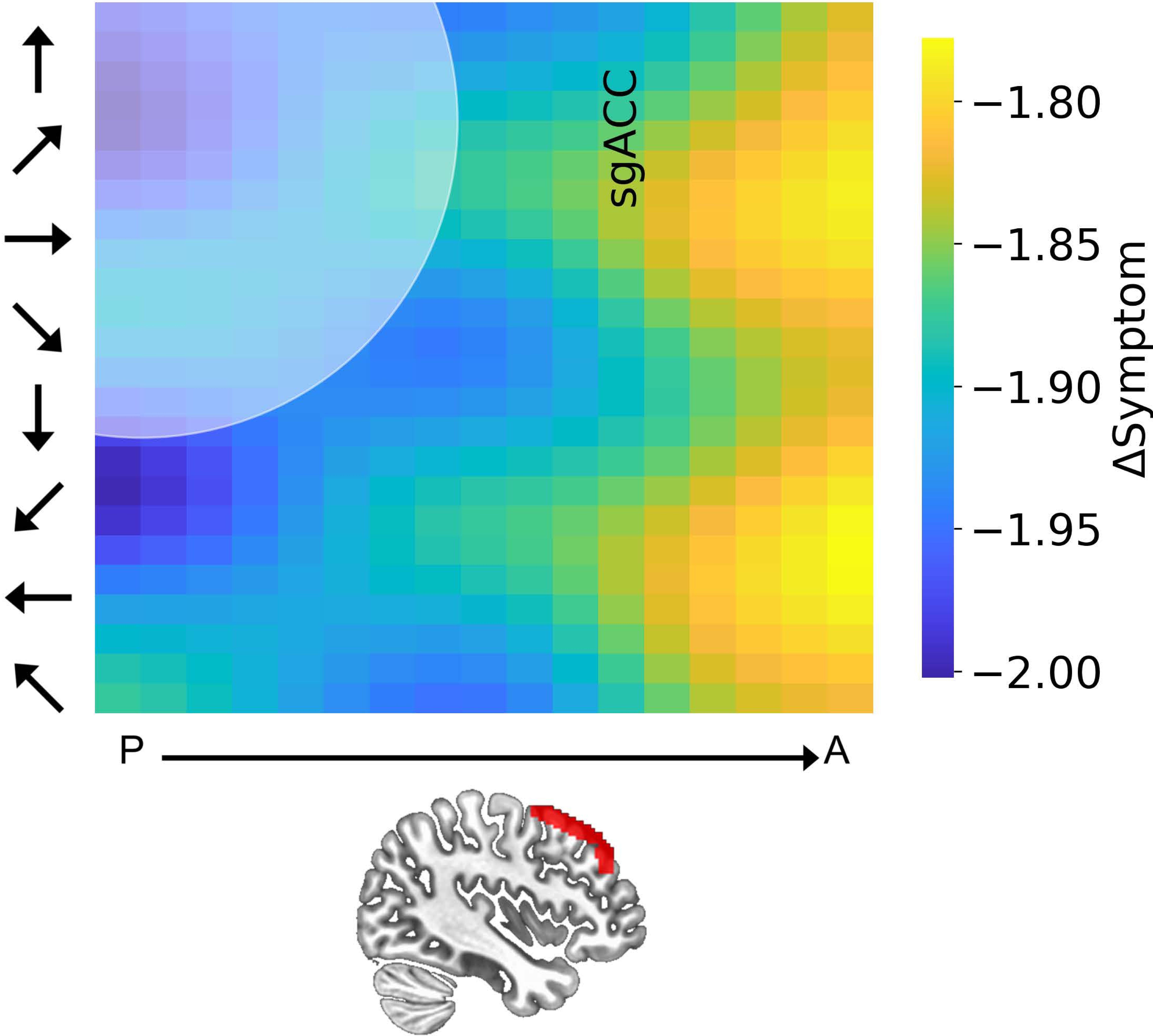

NDARAC789HEZ_hamd_left_dlpfc

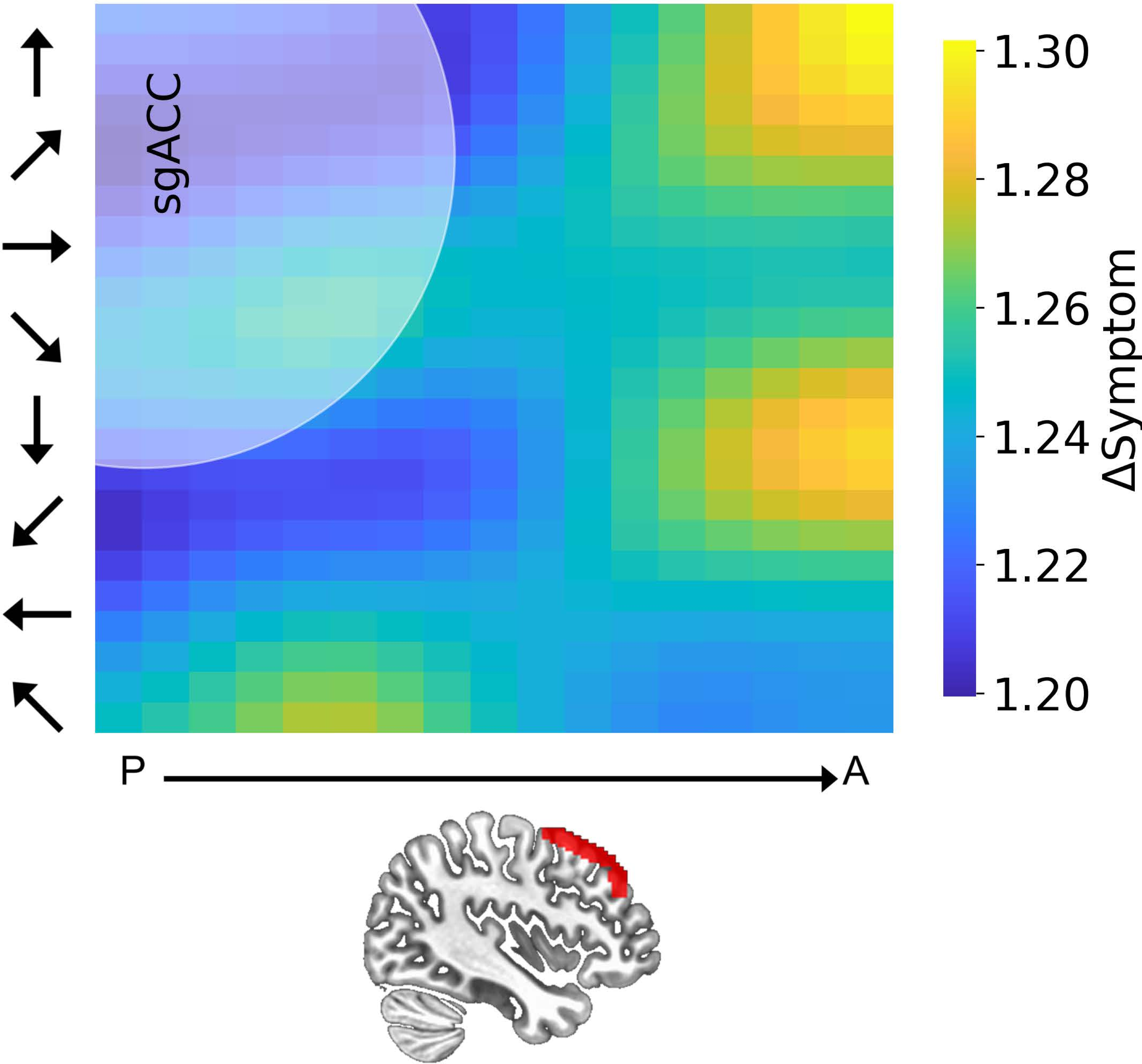

NDARBD224PBF_hamd_left_dlpfc

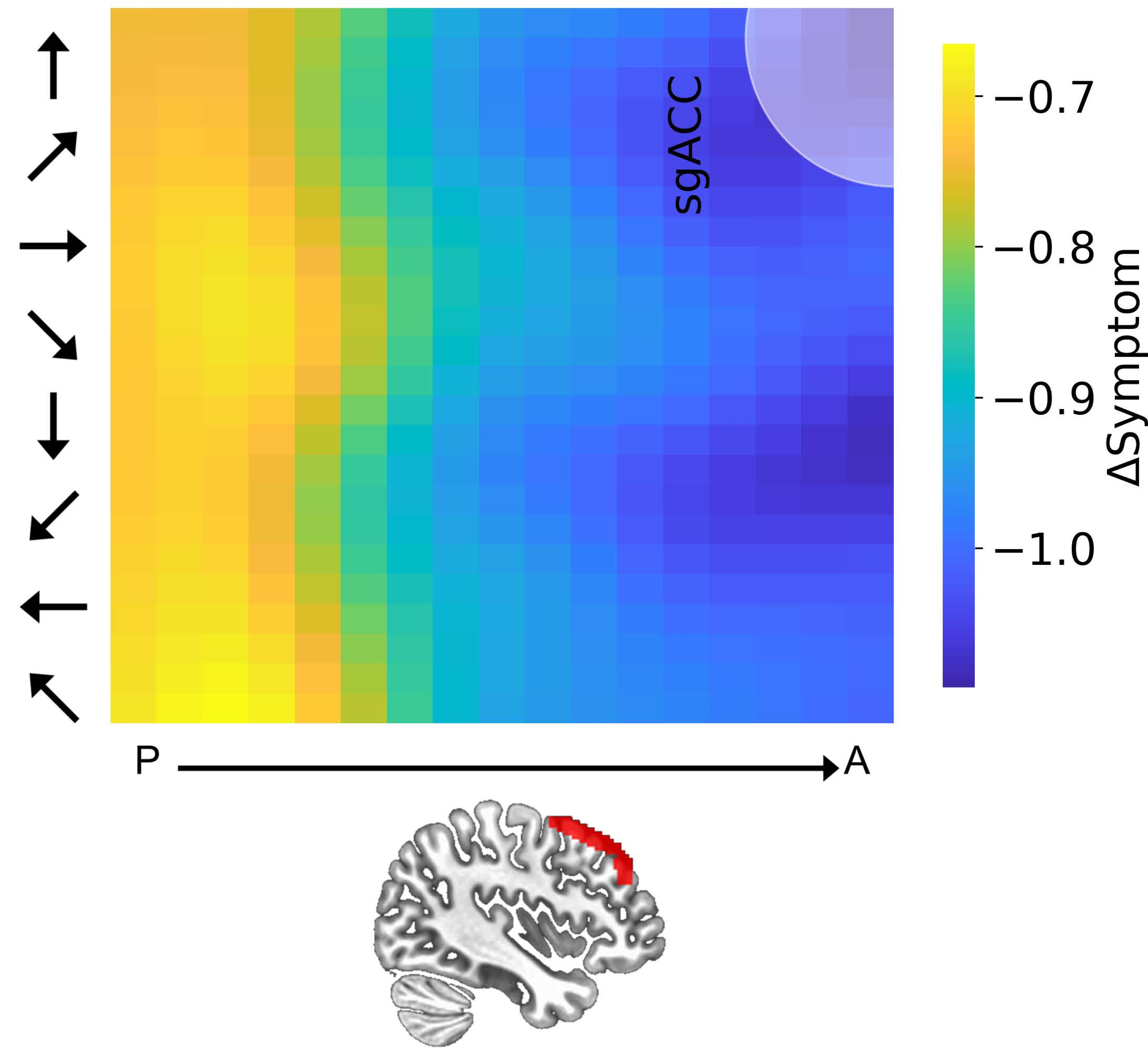

NDARBF292NPE_hamd_left_dlpfc

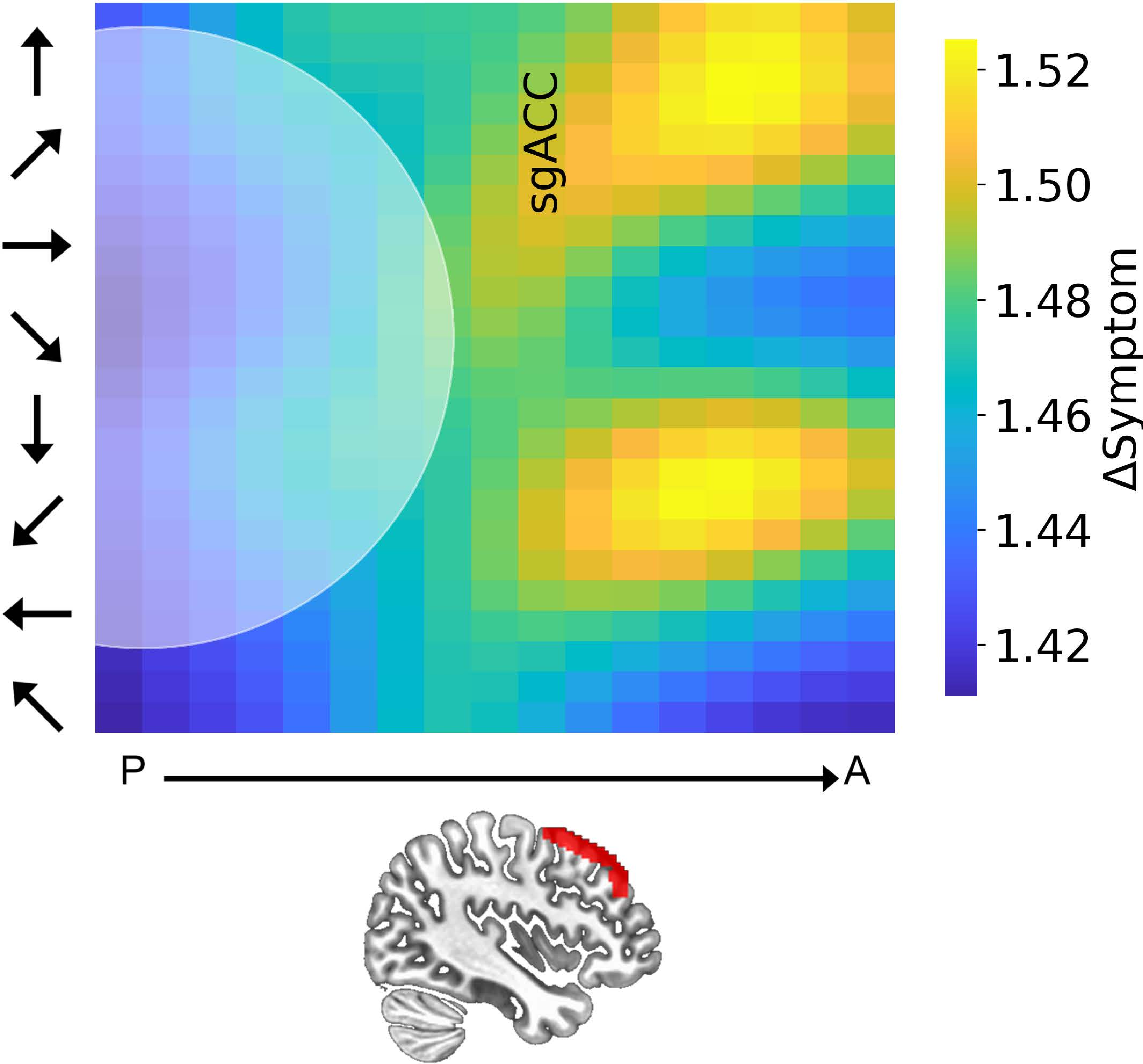

NDARCG535CPC_hamd_left_dlpfc

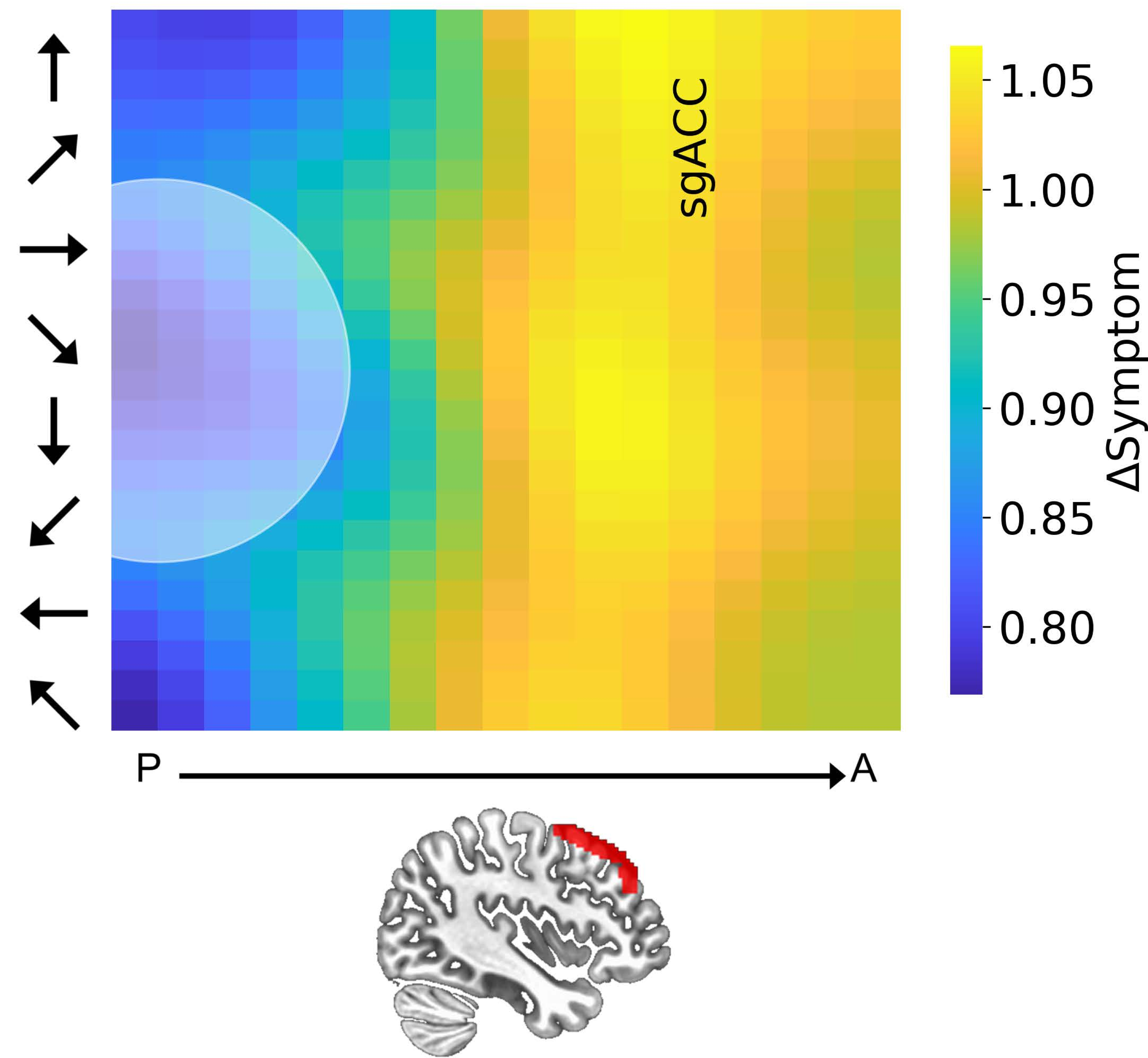

NDARCY406TU1_hamd_left_dlpfc

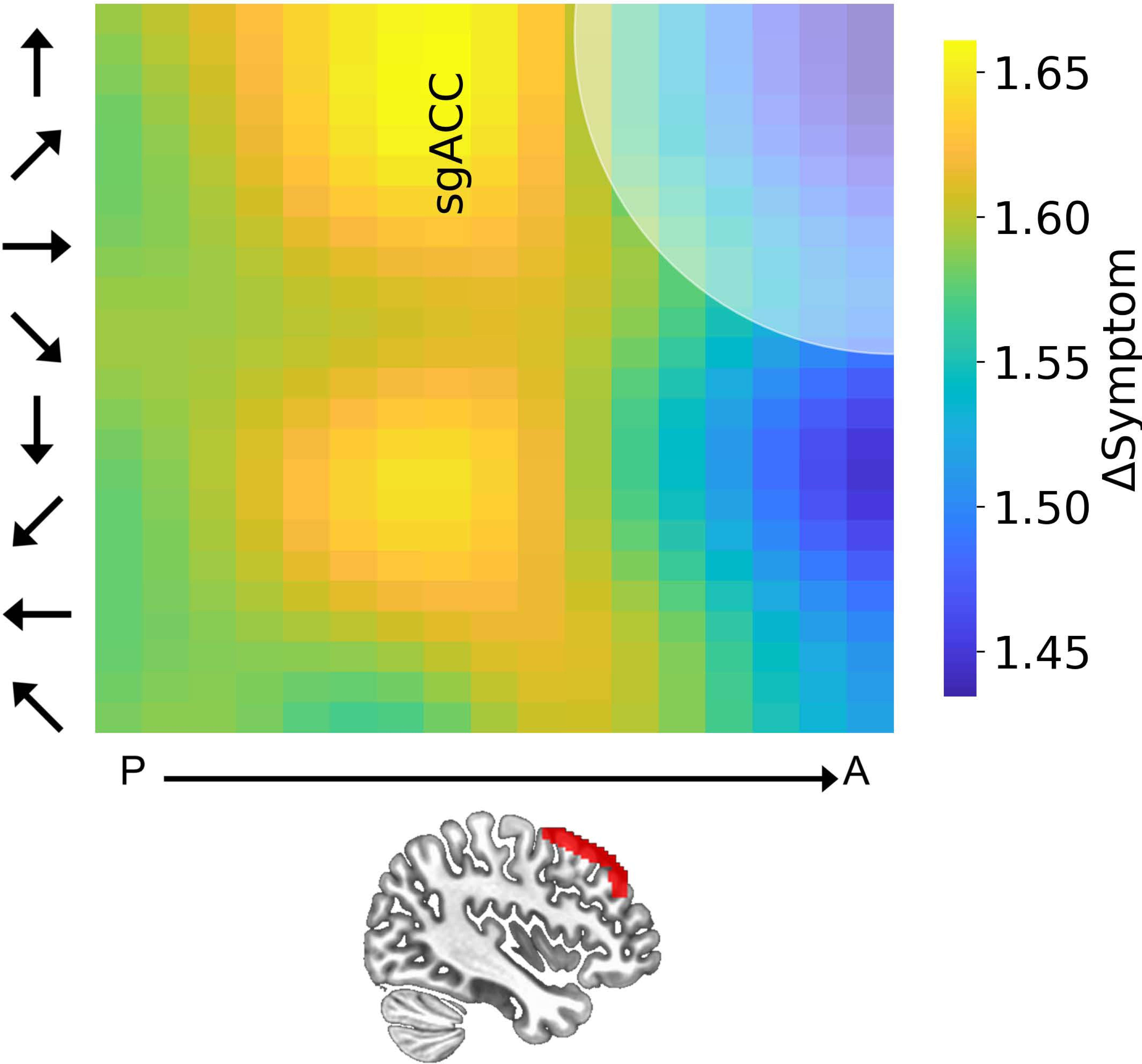

NDARDY771TXZ_hamd_left_dlpfc

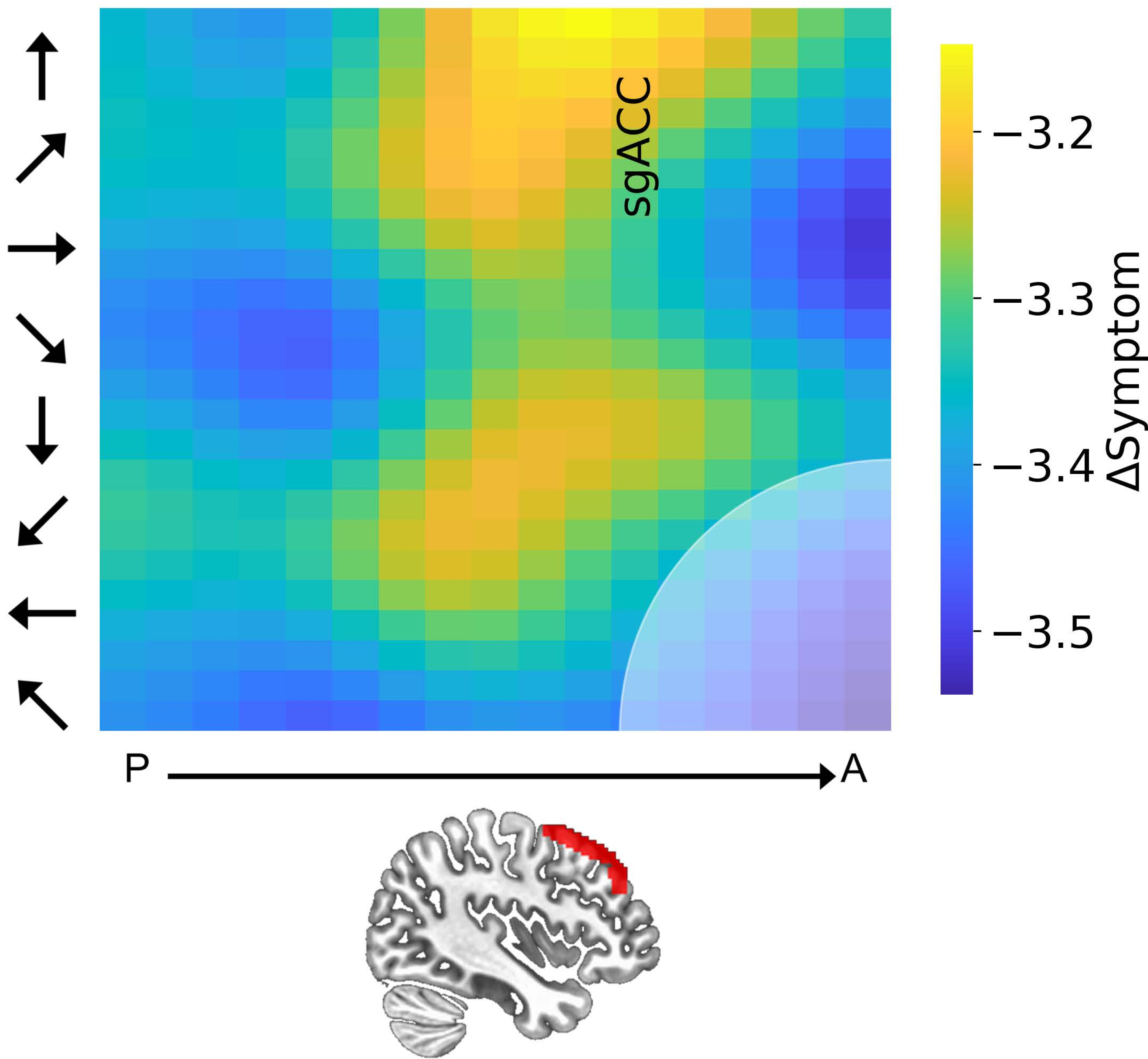

NDAREU790BVQ_hamd_left_dlpfc

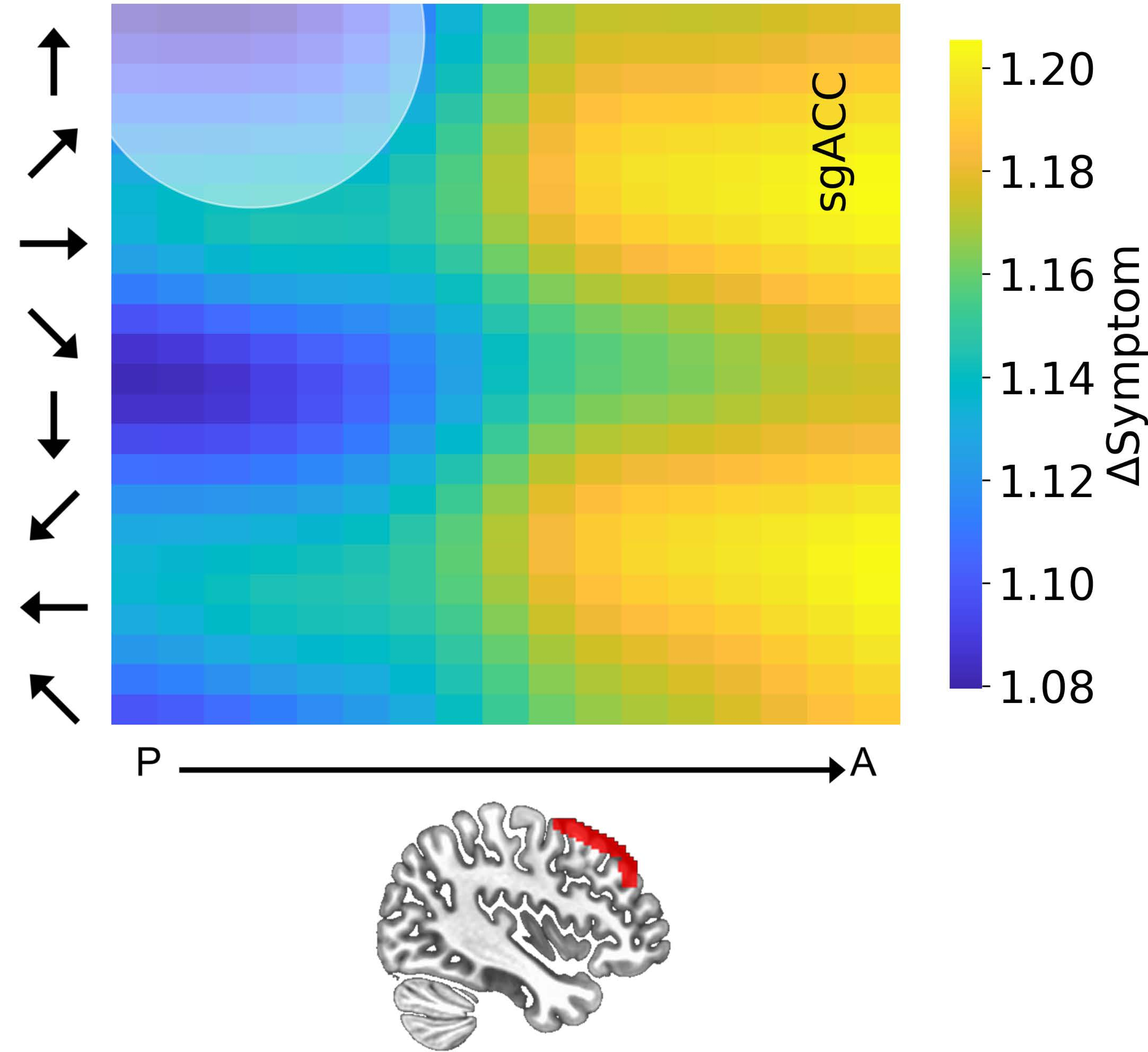

NDARGA844BF5_hamd_left_dlpfc

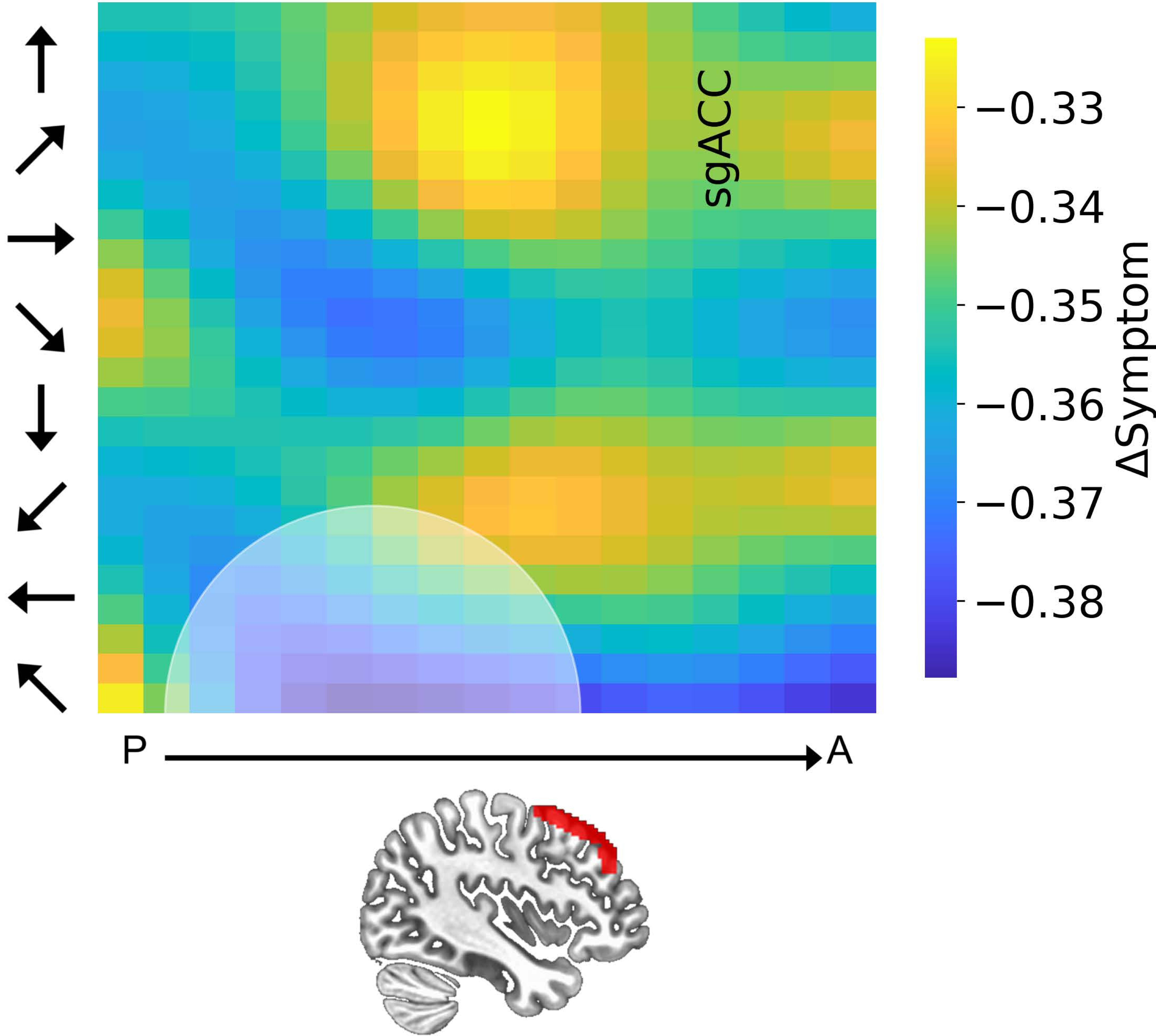

NDARGN596ENP_hamd_left_dlpfc

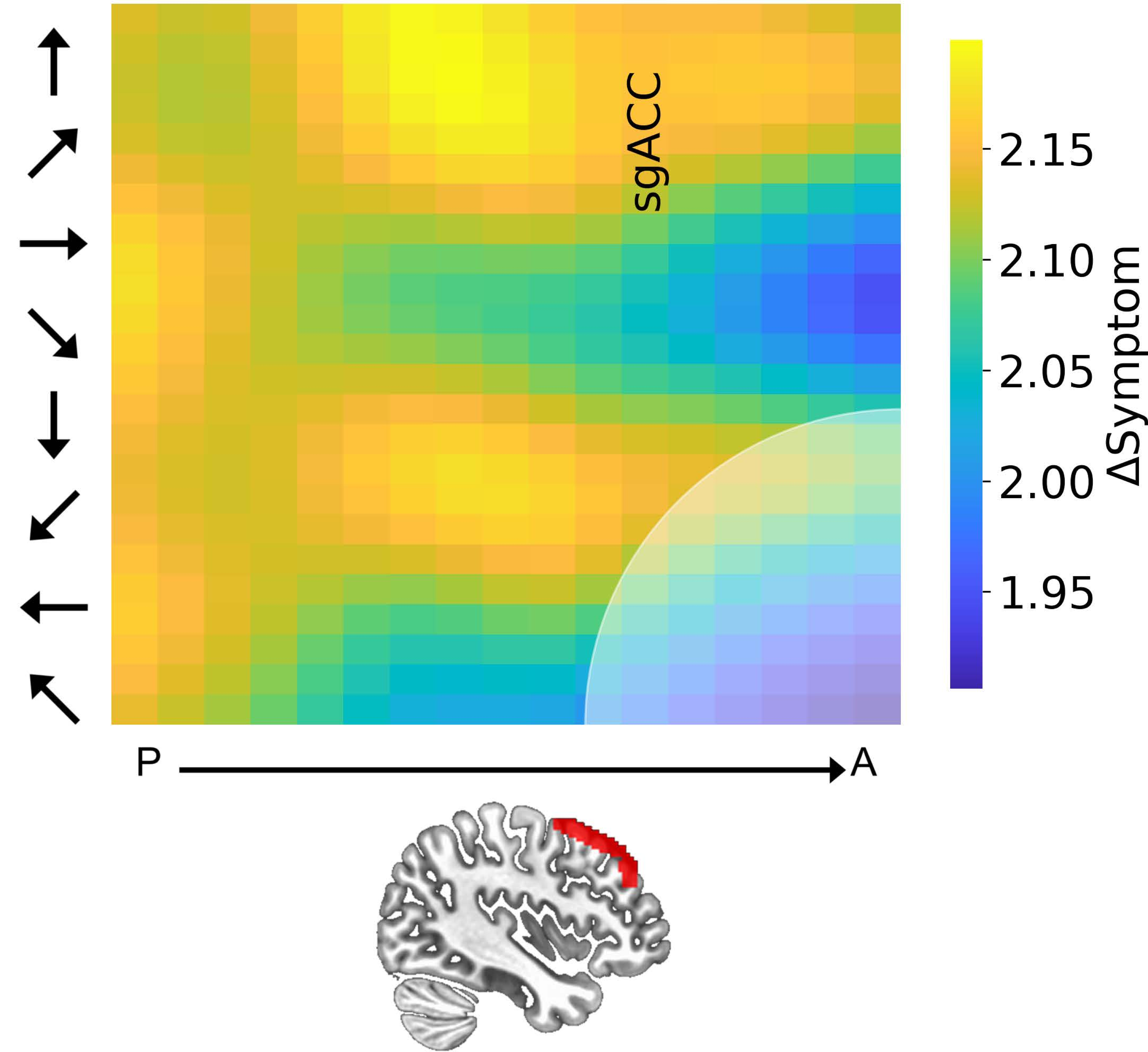

NDARHU677DFF_hamd_left_dlpfc

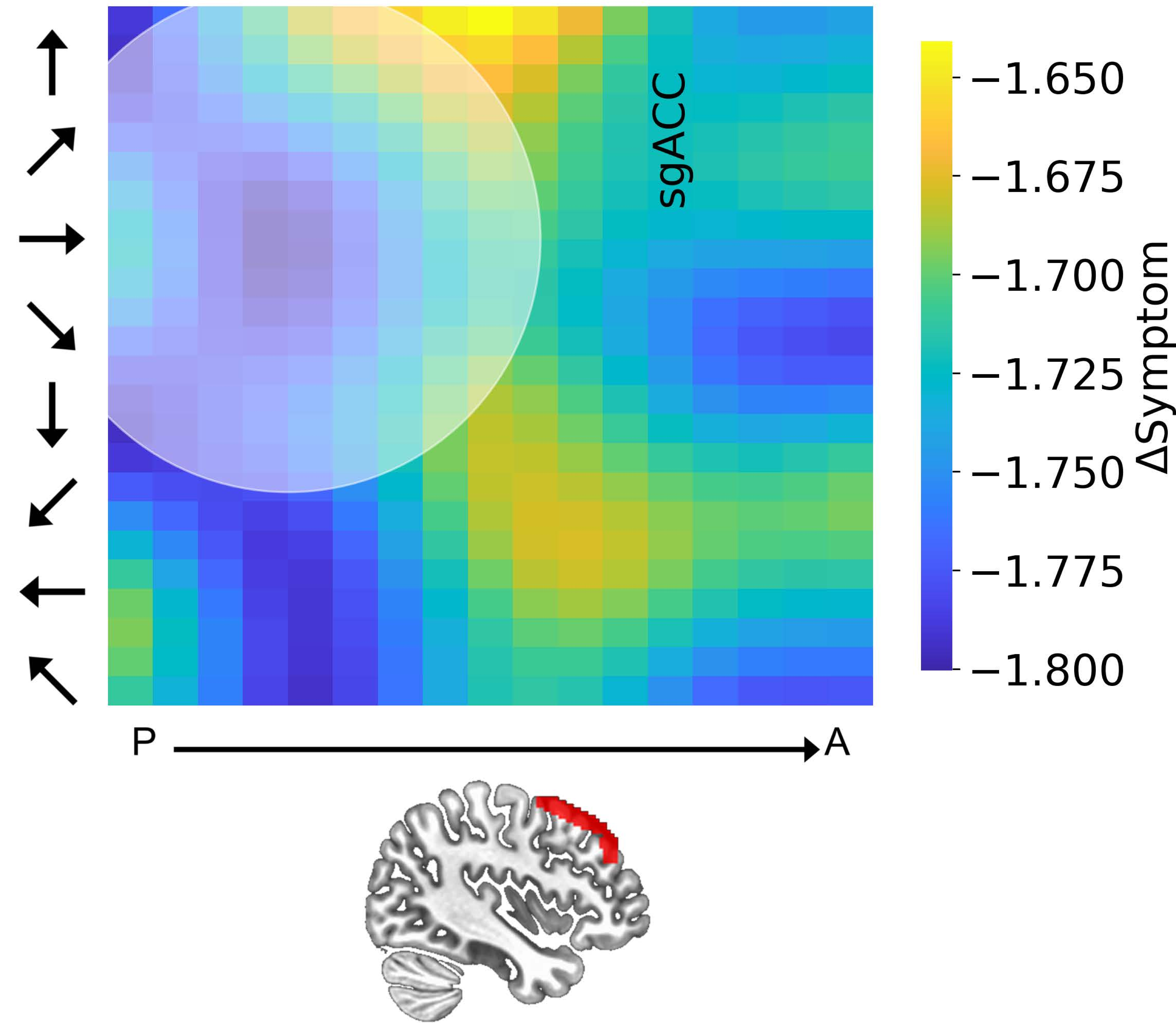

NDARJL083WMP_hamd_left_dlpfc

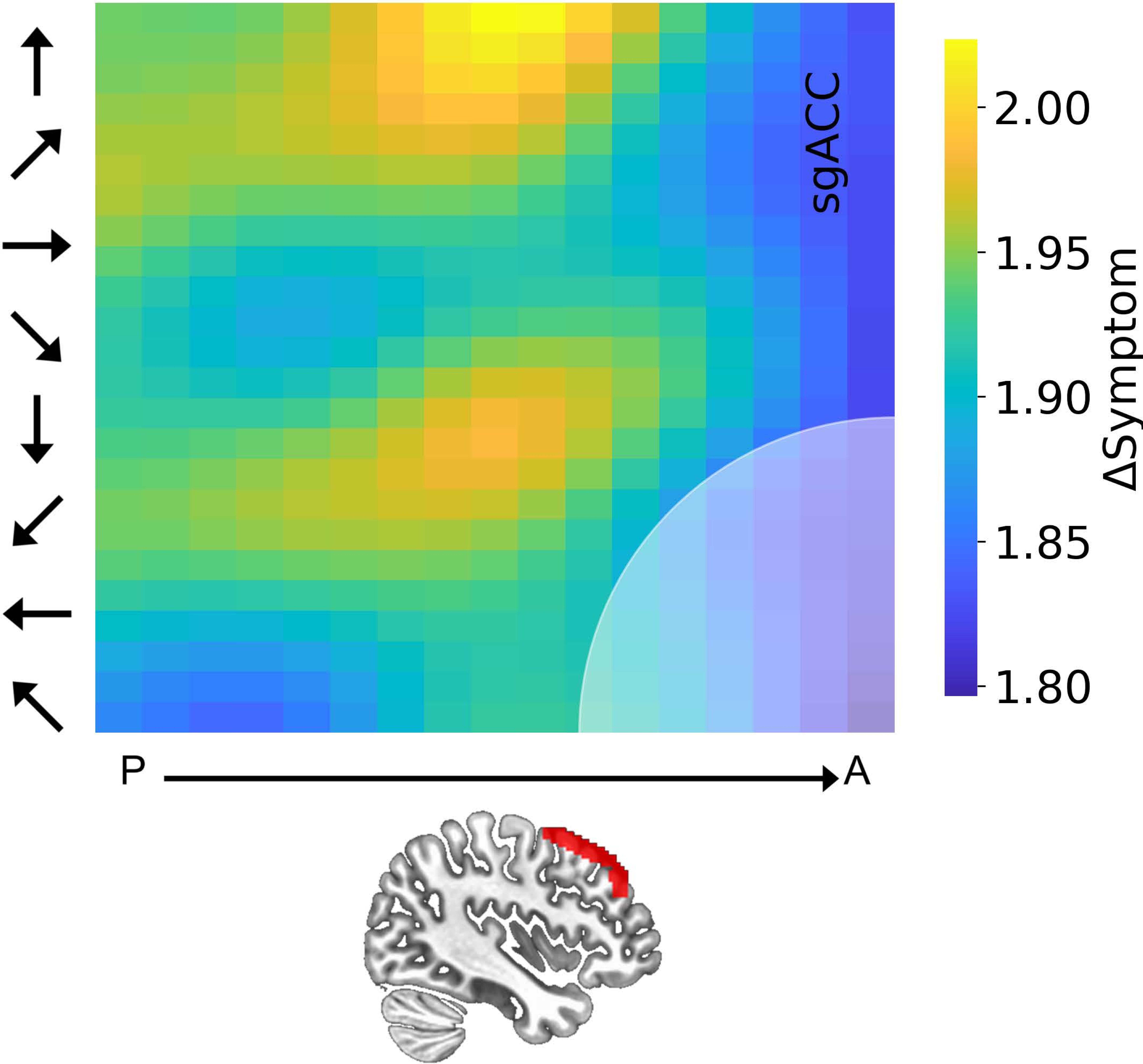

NDARKR291PWN_hamd_left_dlpfc

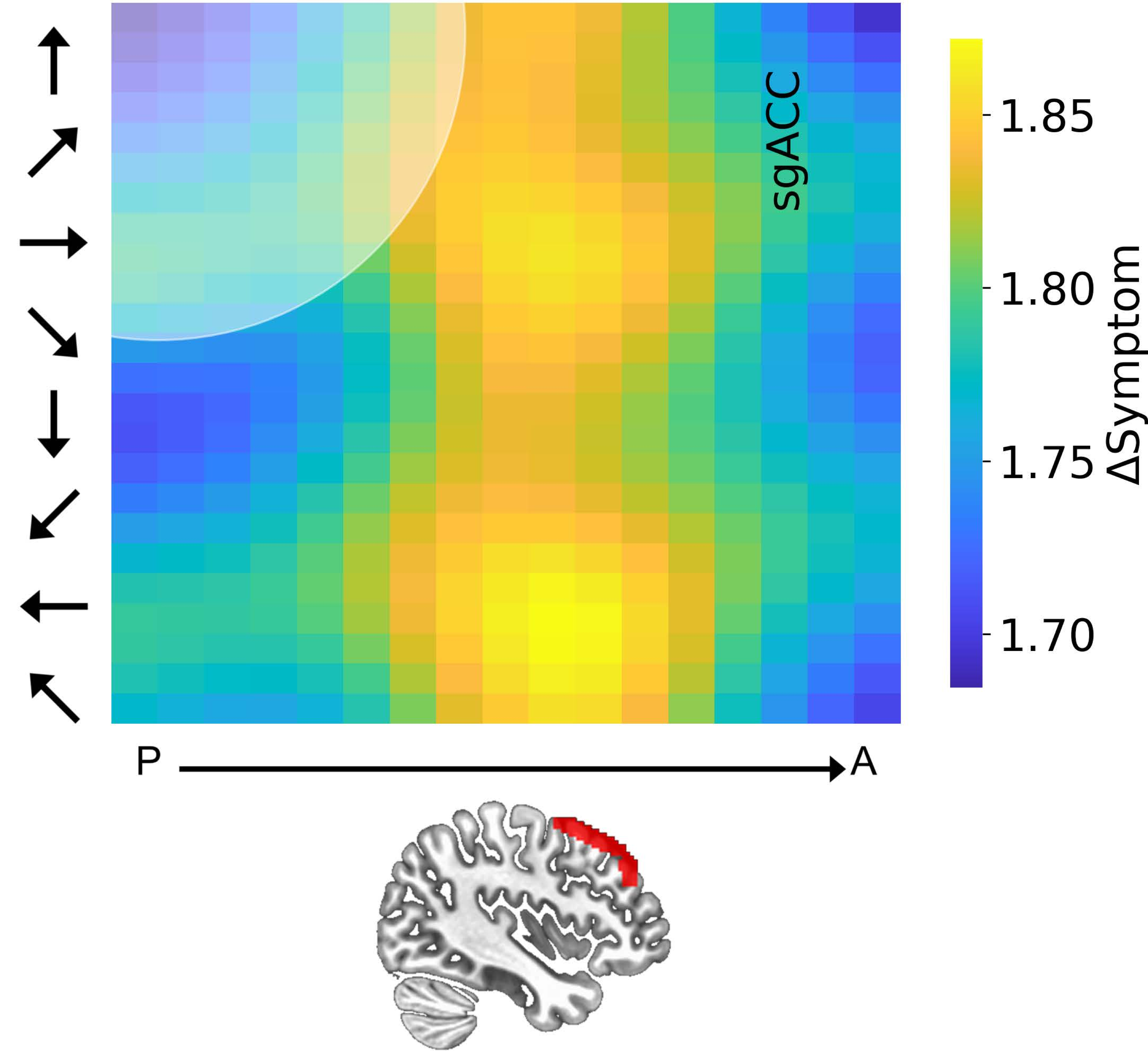

NDARLH479MBF_hamd_left_dlpfc

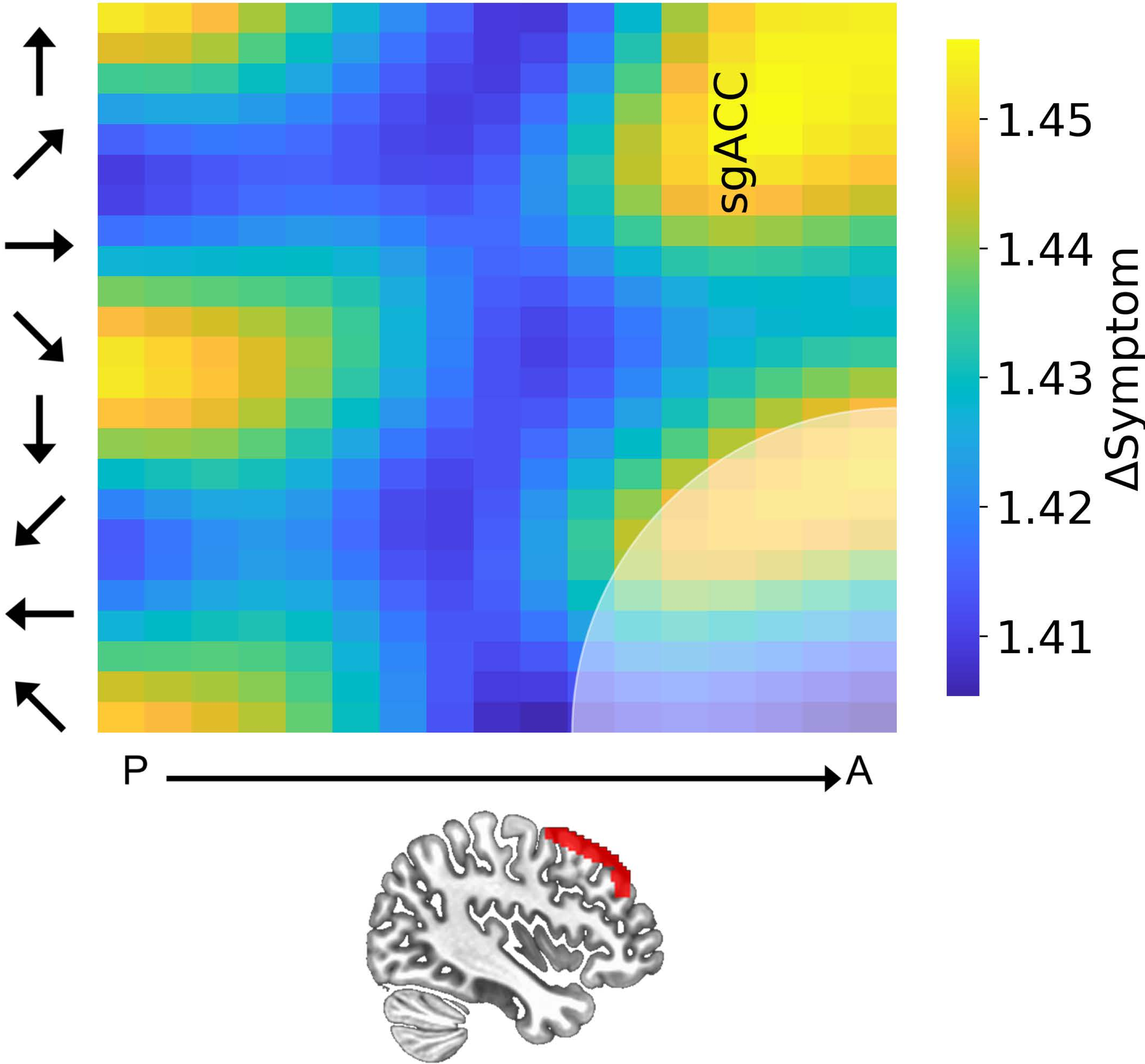

NDARME497RF8_hamd_left_dlpfc

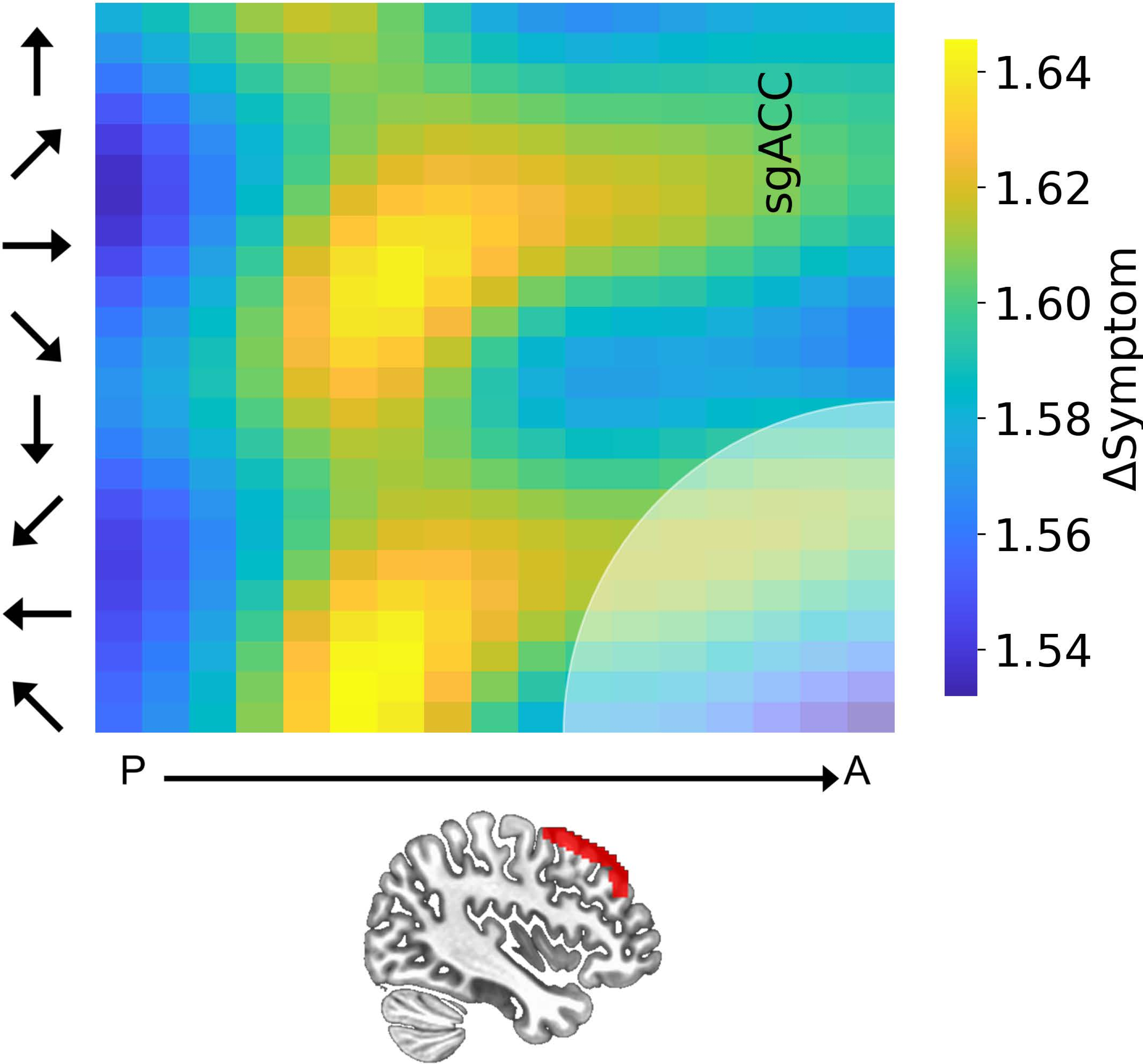

NDARNJ618KZJ_hamd_left_dlpfc

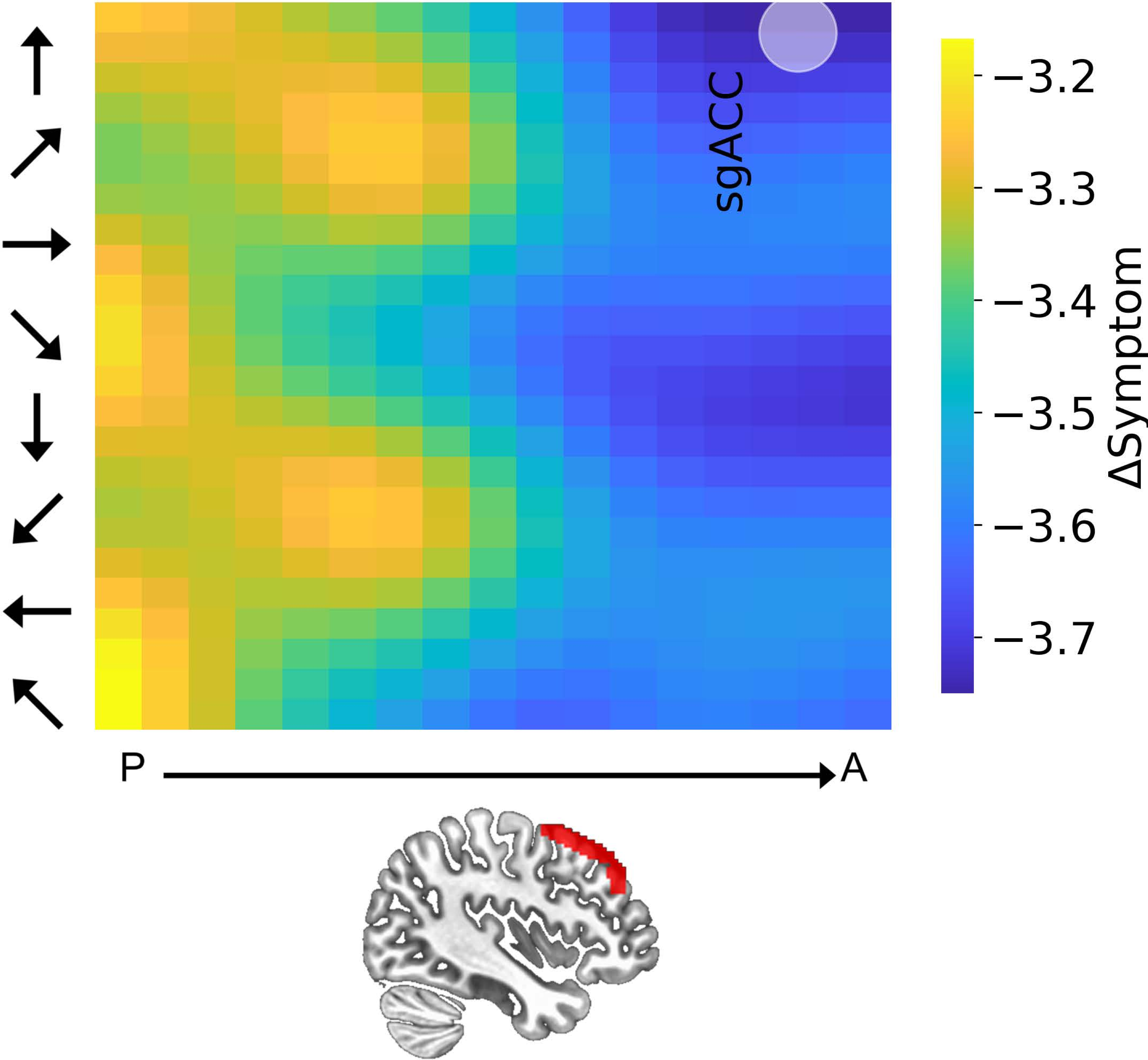

NDARNL715RTY_hamd_left_dlpfc

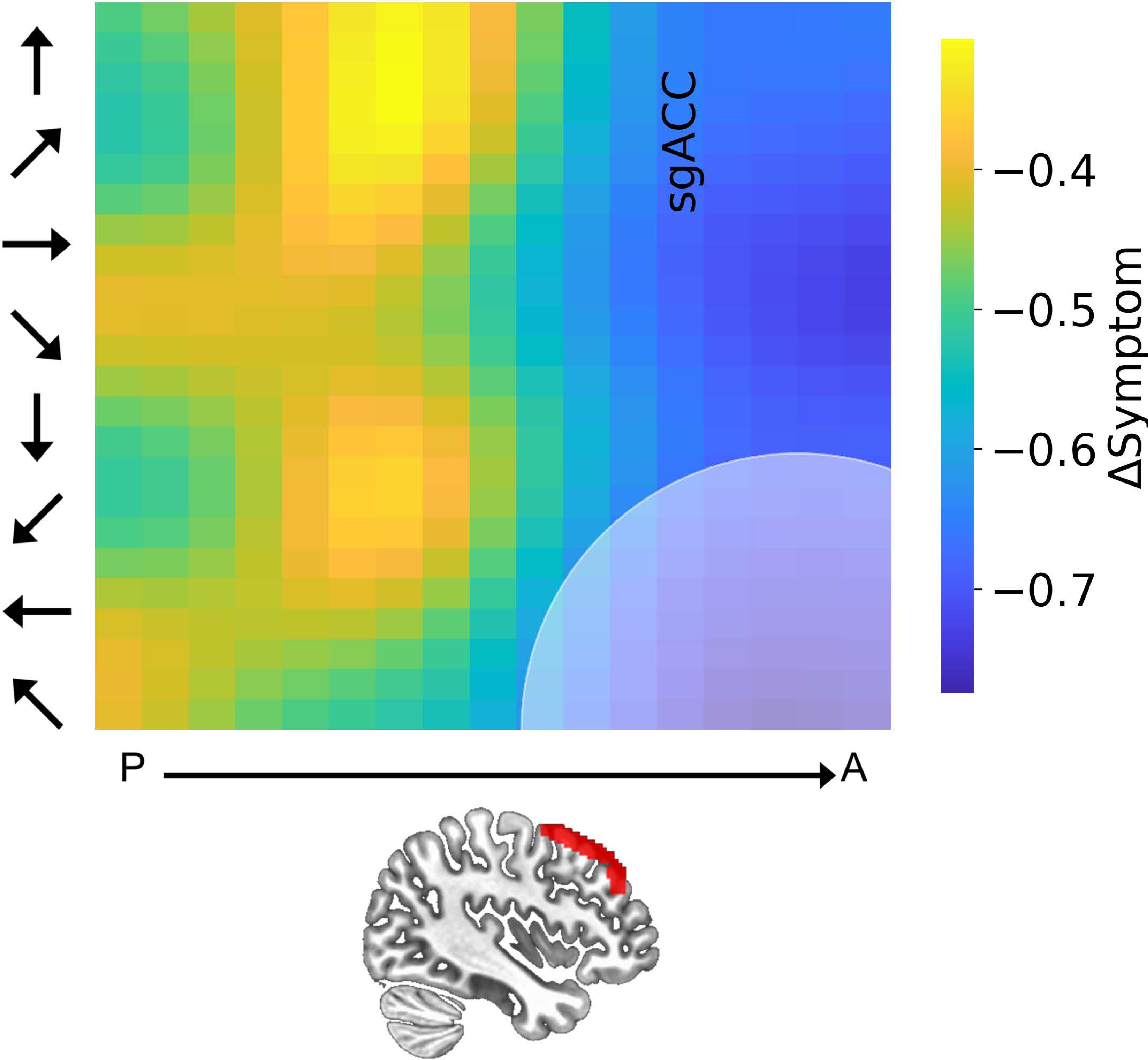

NDARPK532CVC_hamd_left_dlpfc

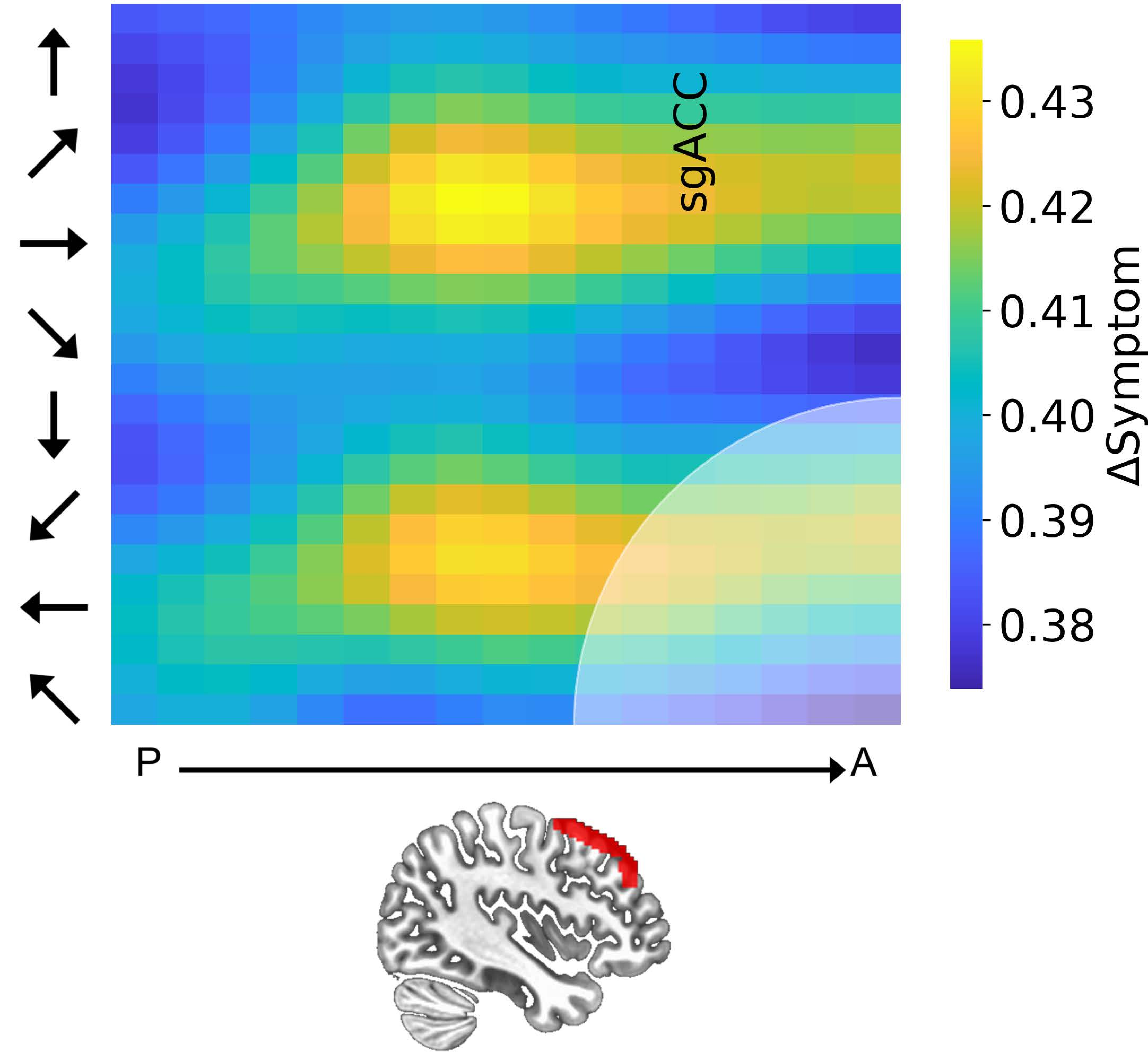

NDARWK858FA2_hamd_left_dlpfc

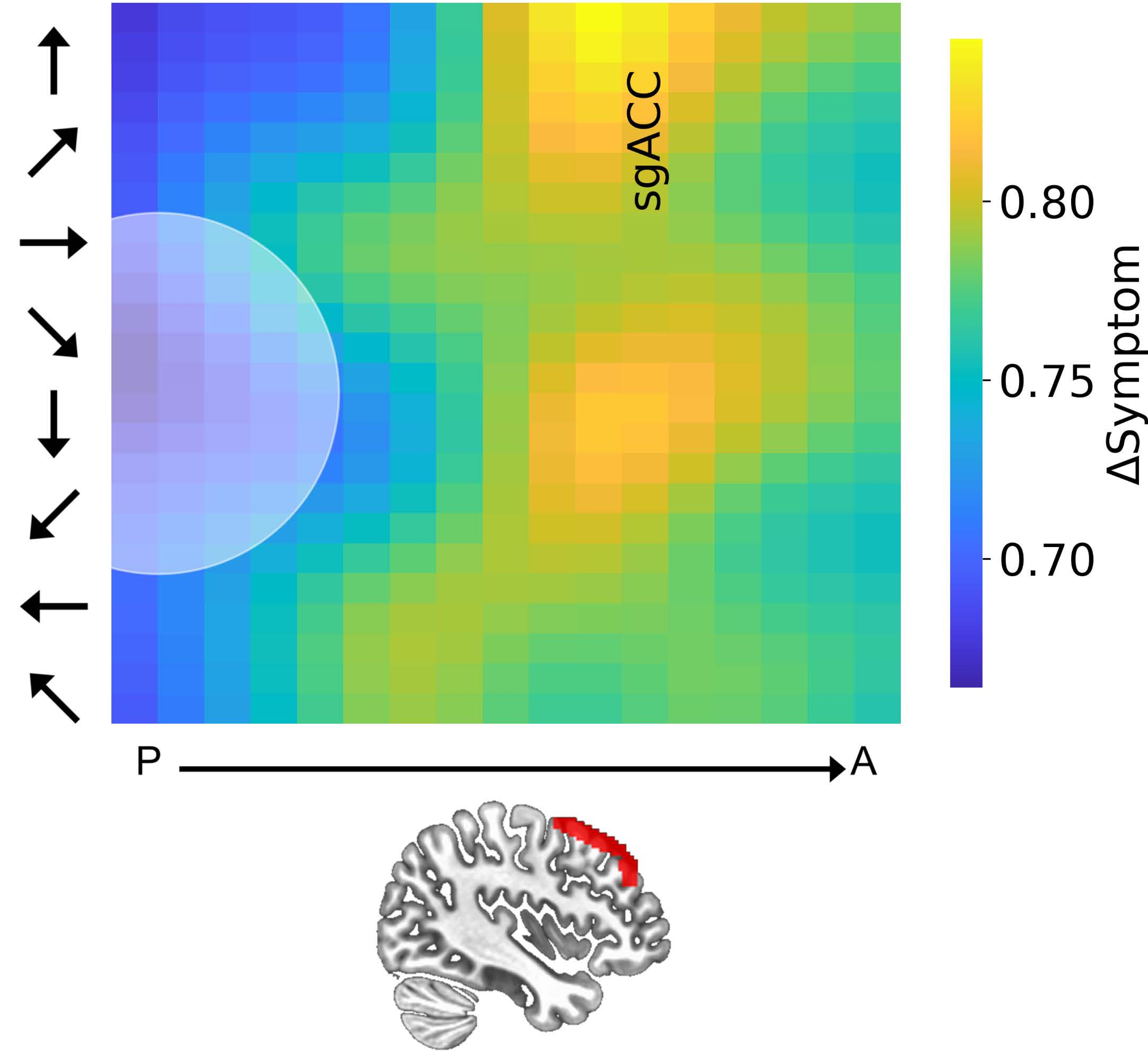

NDARWM071DRZ_hamd_left_dlpfc

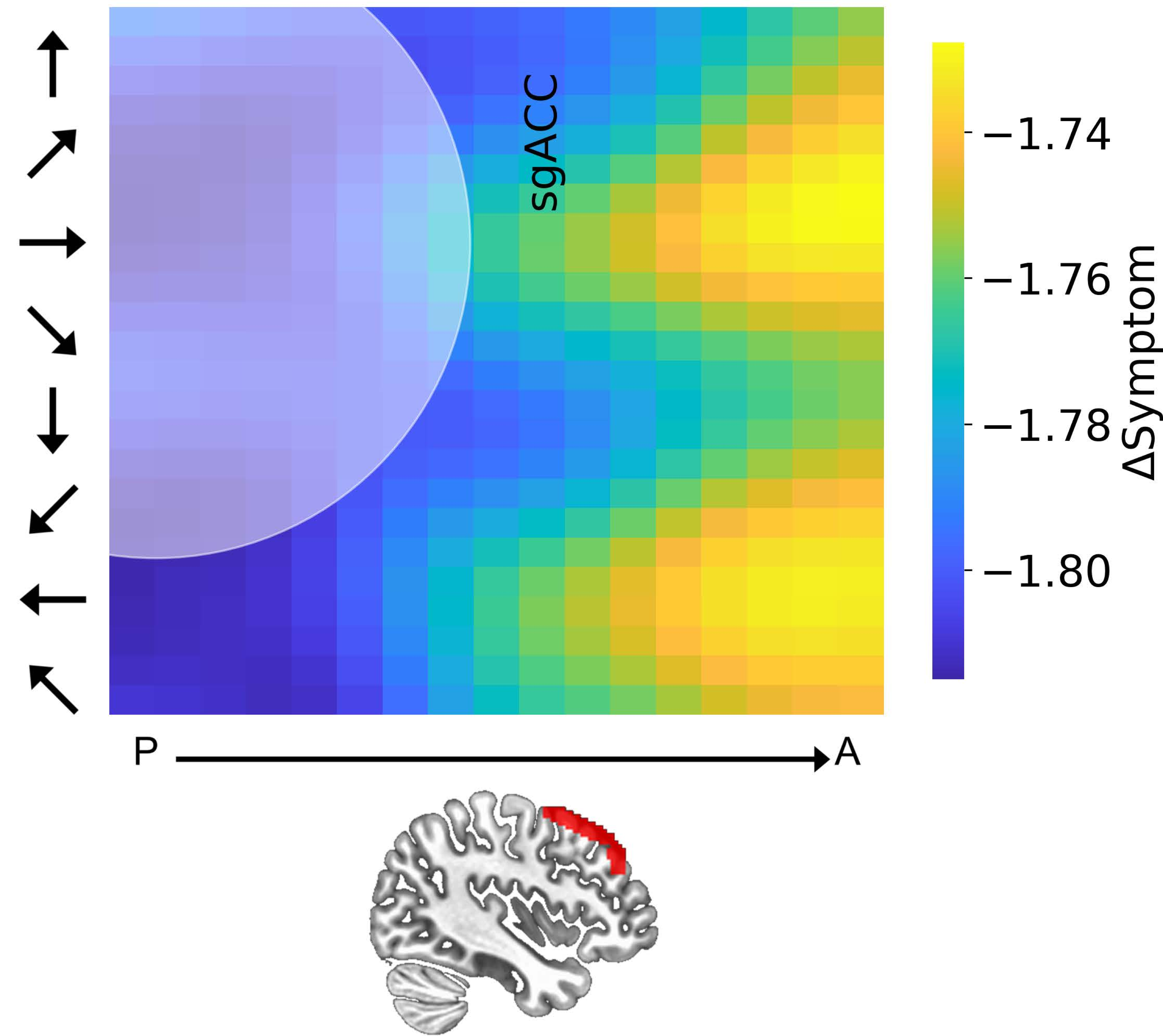

NDARWT226PU5_hamd_left_dlpfc

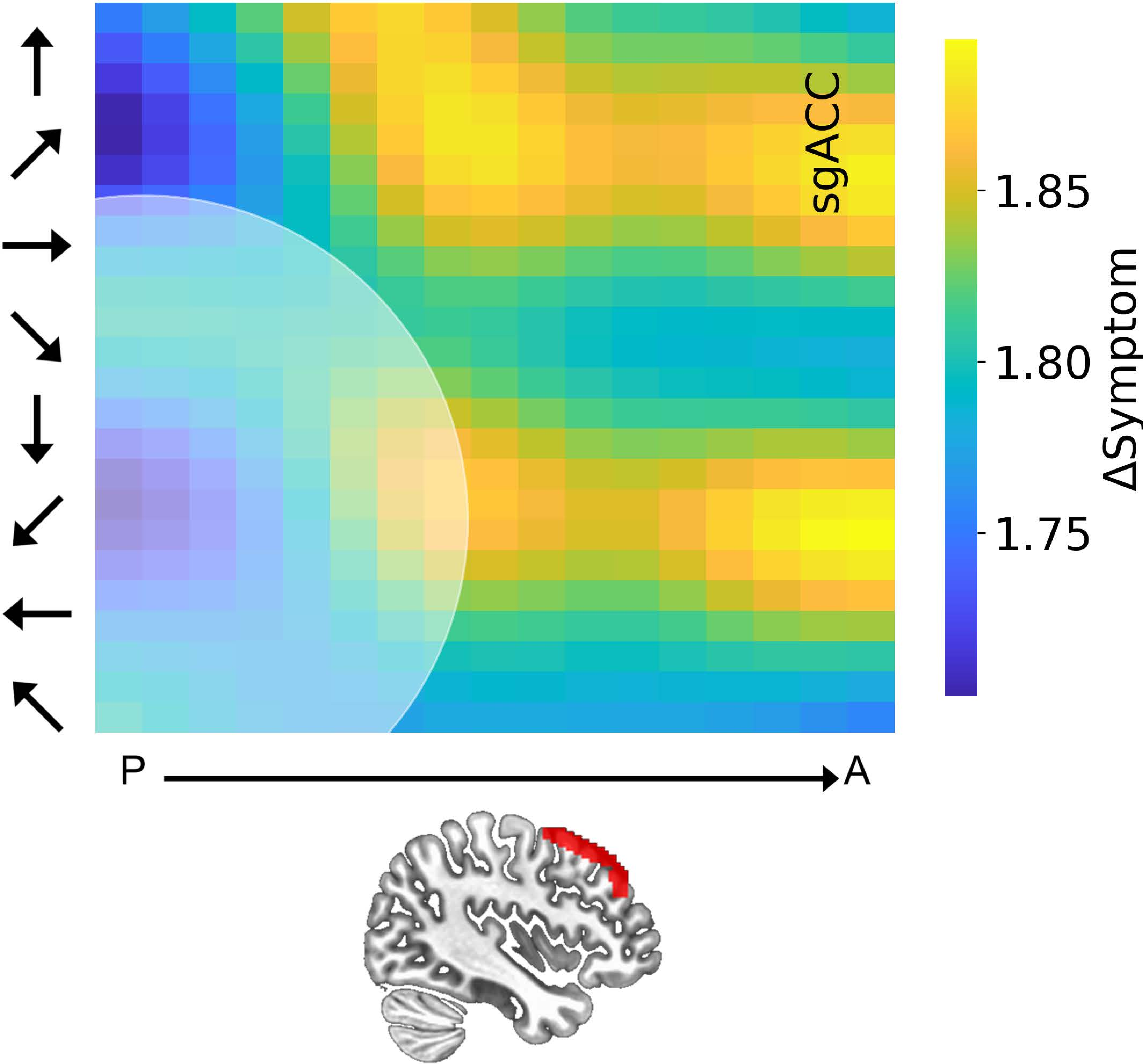

NDARWU139LAY_hamd_left_dlpfc

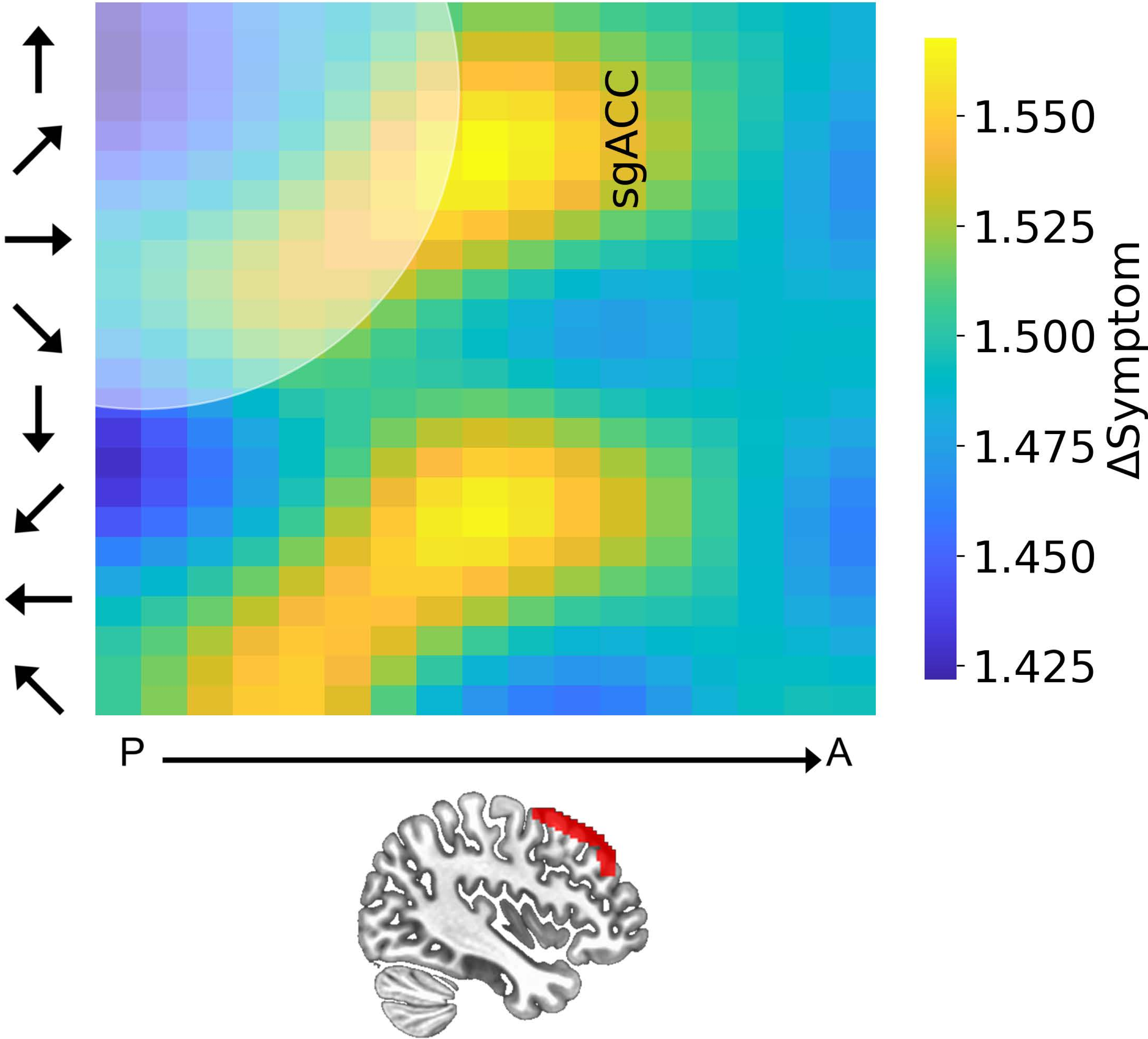

NDARXH480BBY_hamd_left_dlpfc

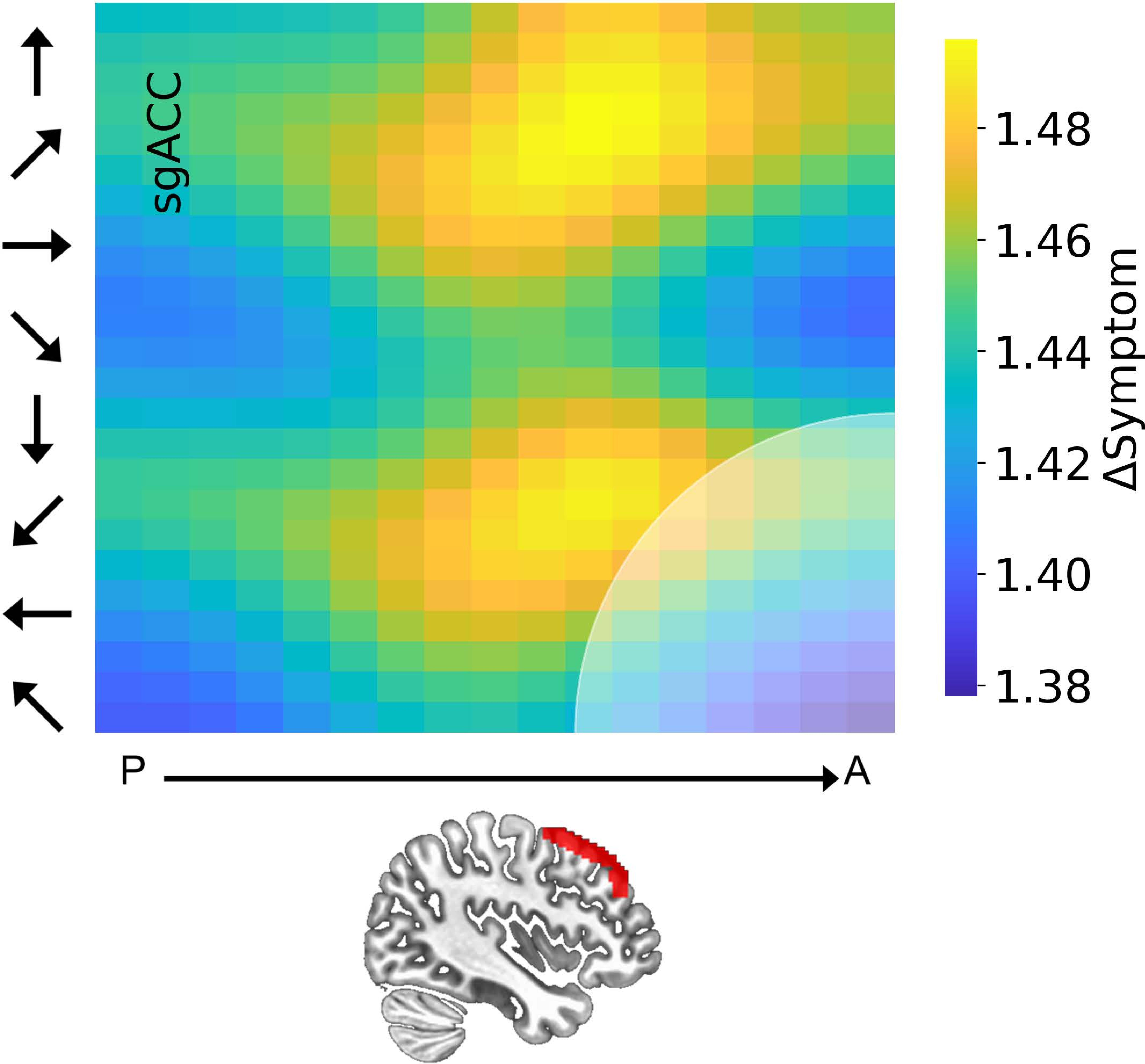

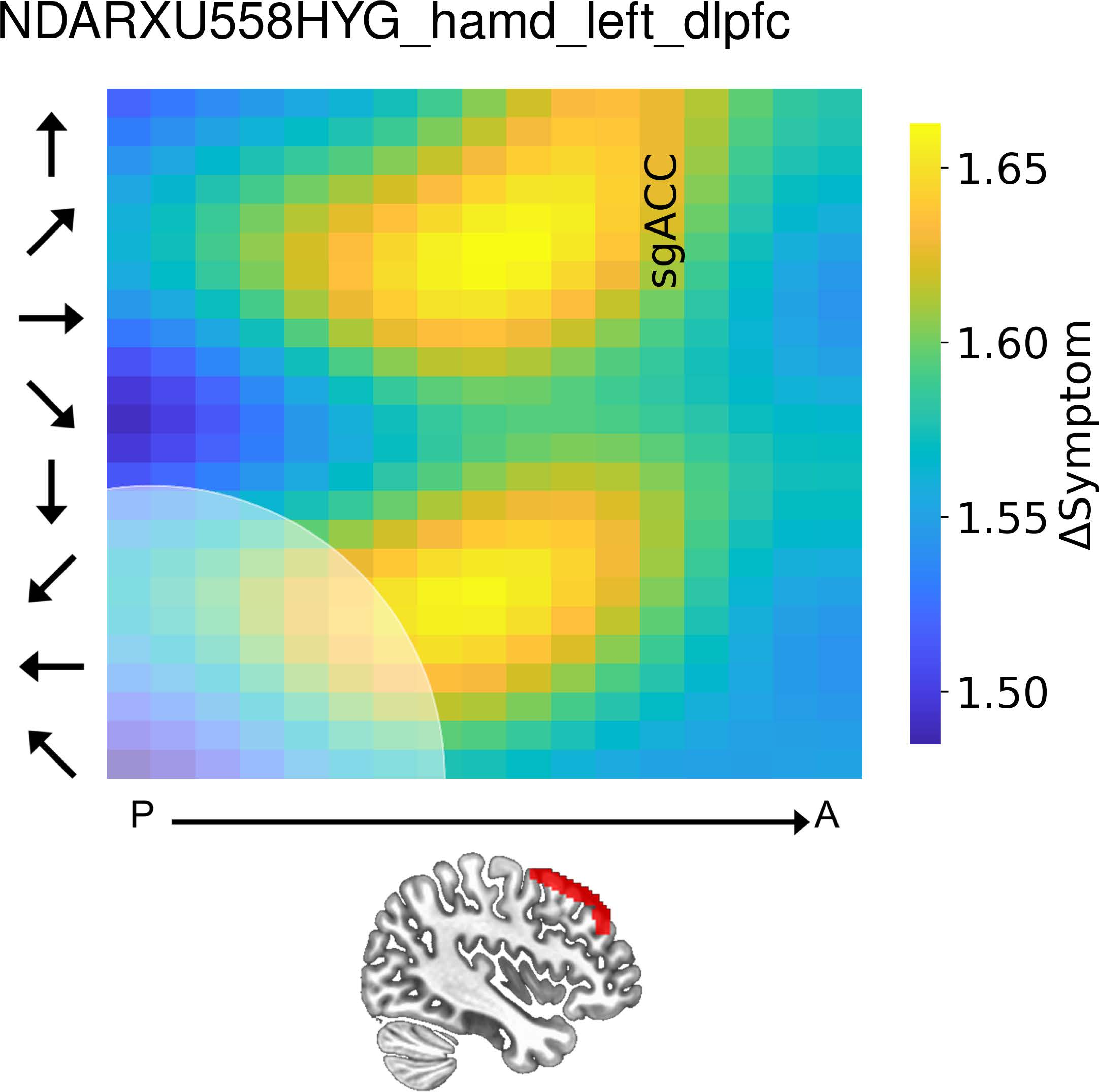

NDARXU558HYG_hamd_left_dlpfc
sgACC
1.65
1.60
1.55
1.50
ΔSymptom
P
A

NDARZW884MZC_hamd_left_dlpfc

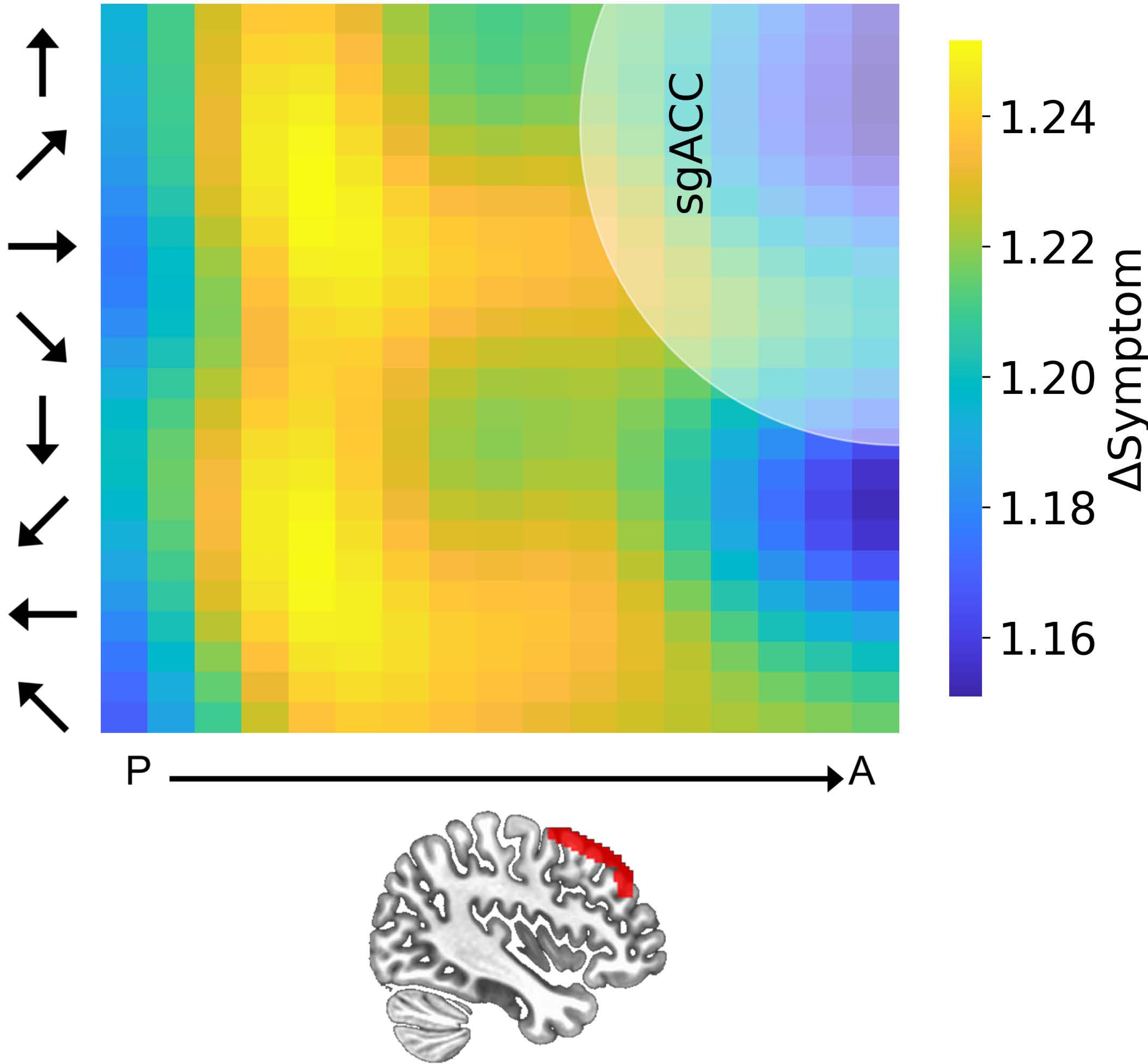

NDARAC286ZE7_hamd_right_dlpfc

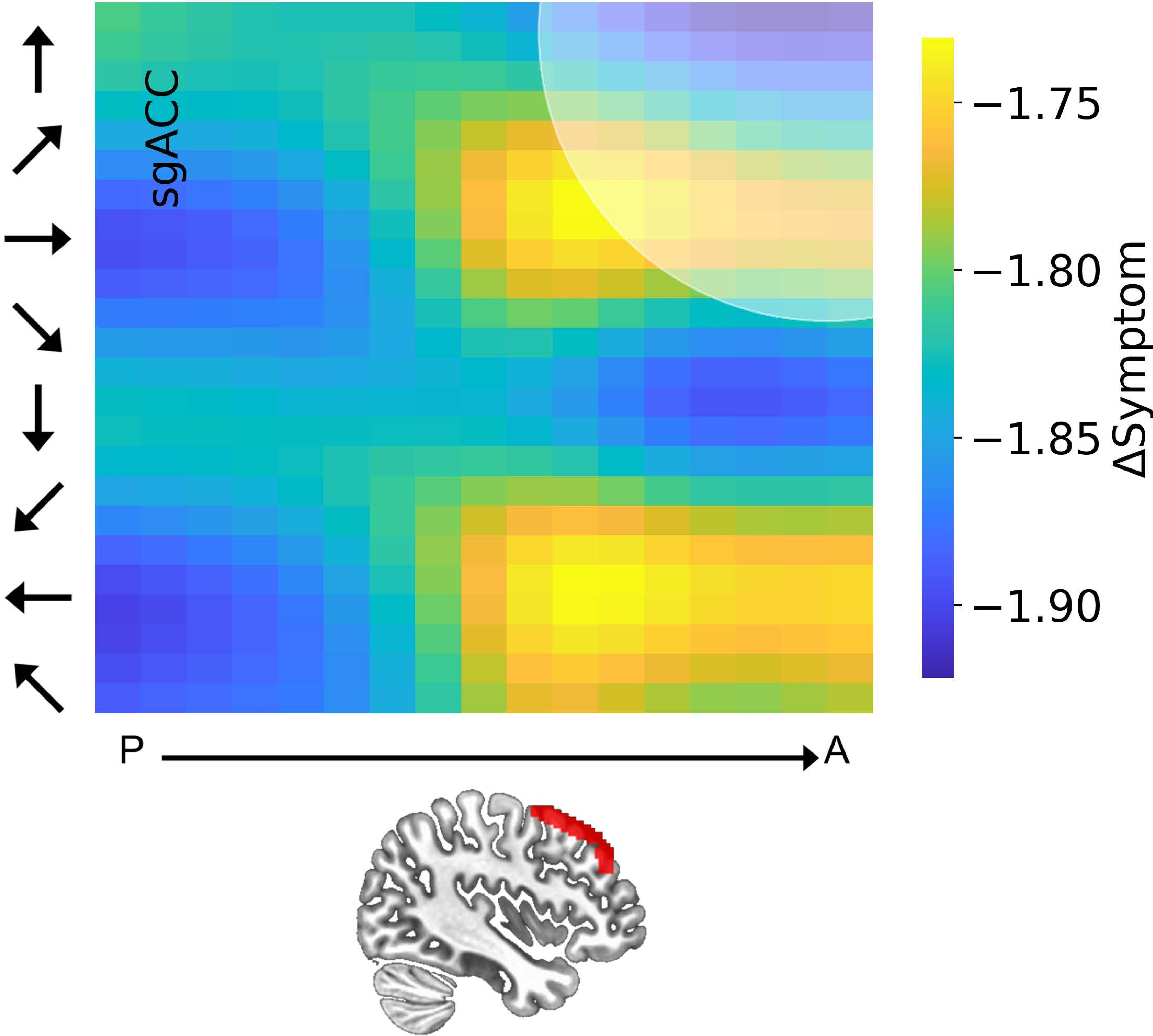

NDARAC789HEZ_hamd_right_dlpfc

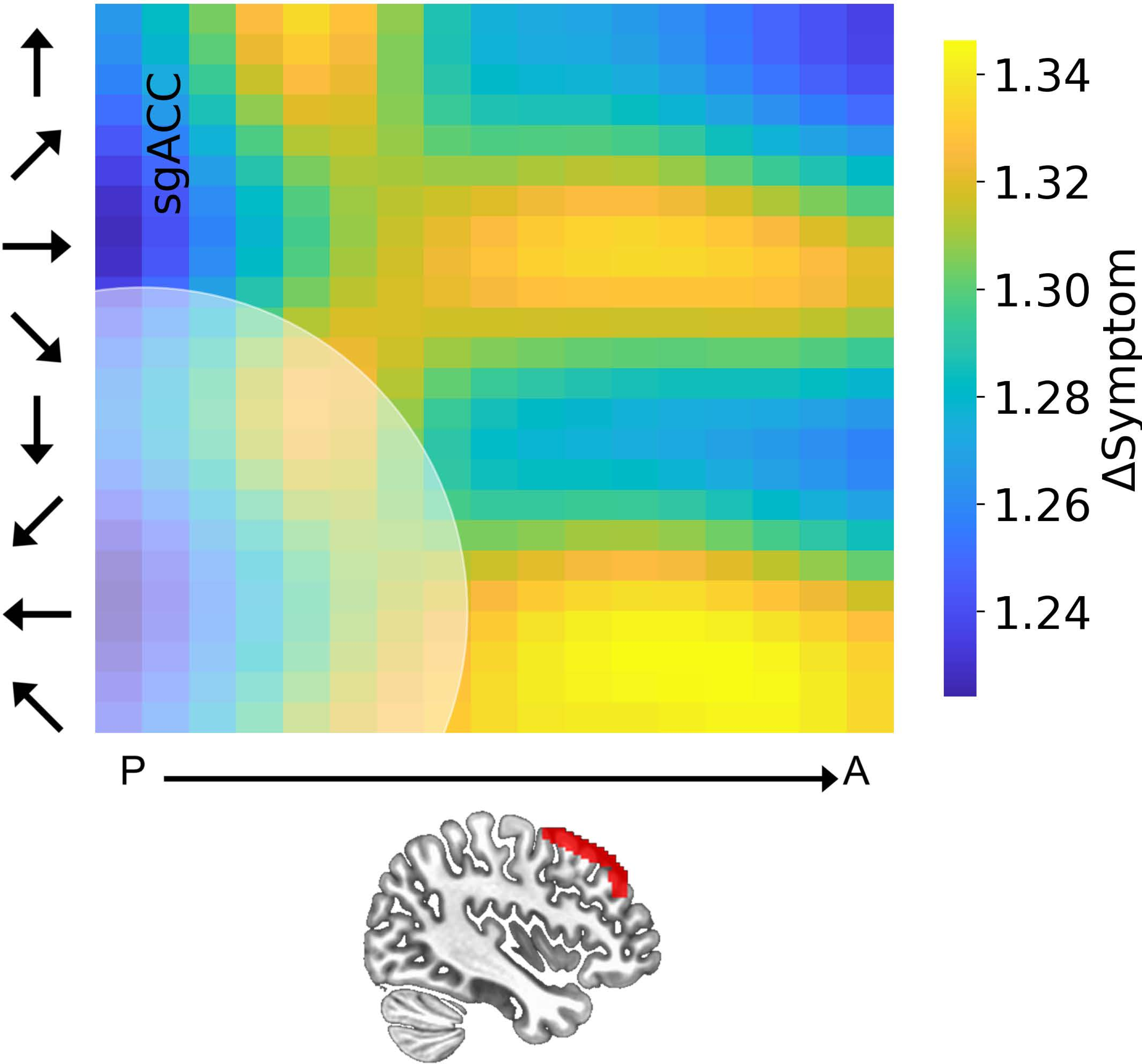

NDARBD224PBF_hamd_right_dlpfc

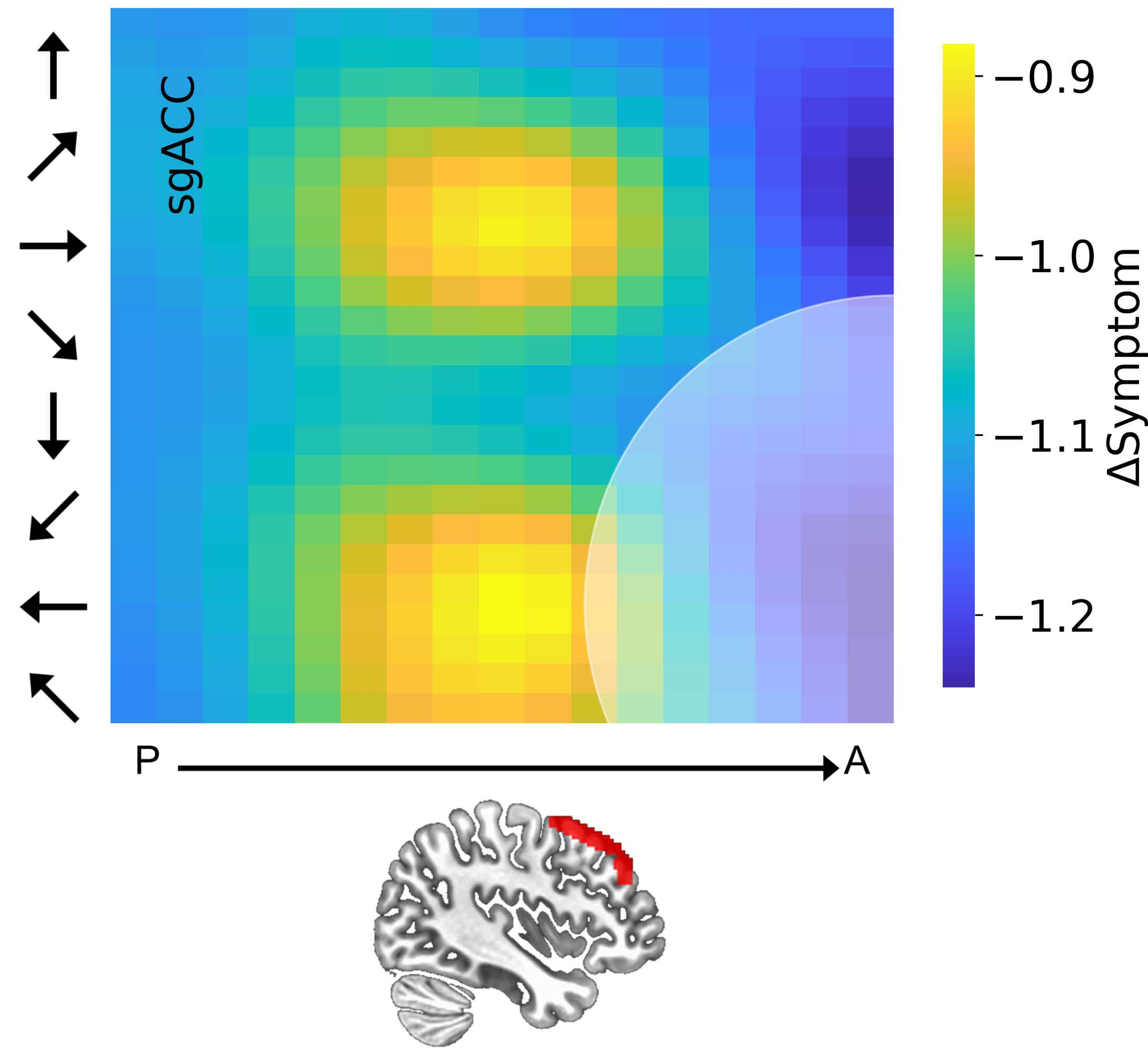

NDARBF292NPE_hamd_right_dlpfc

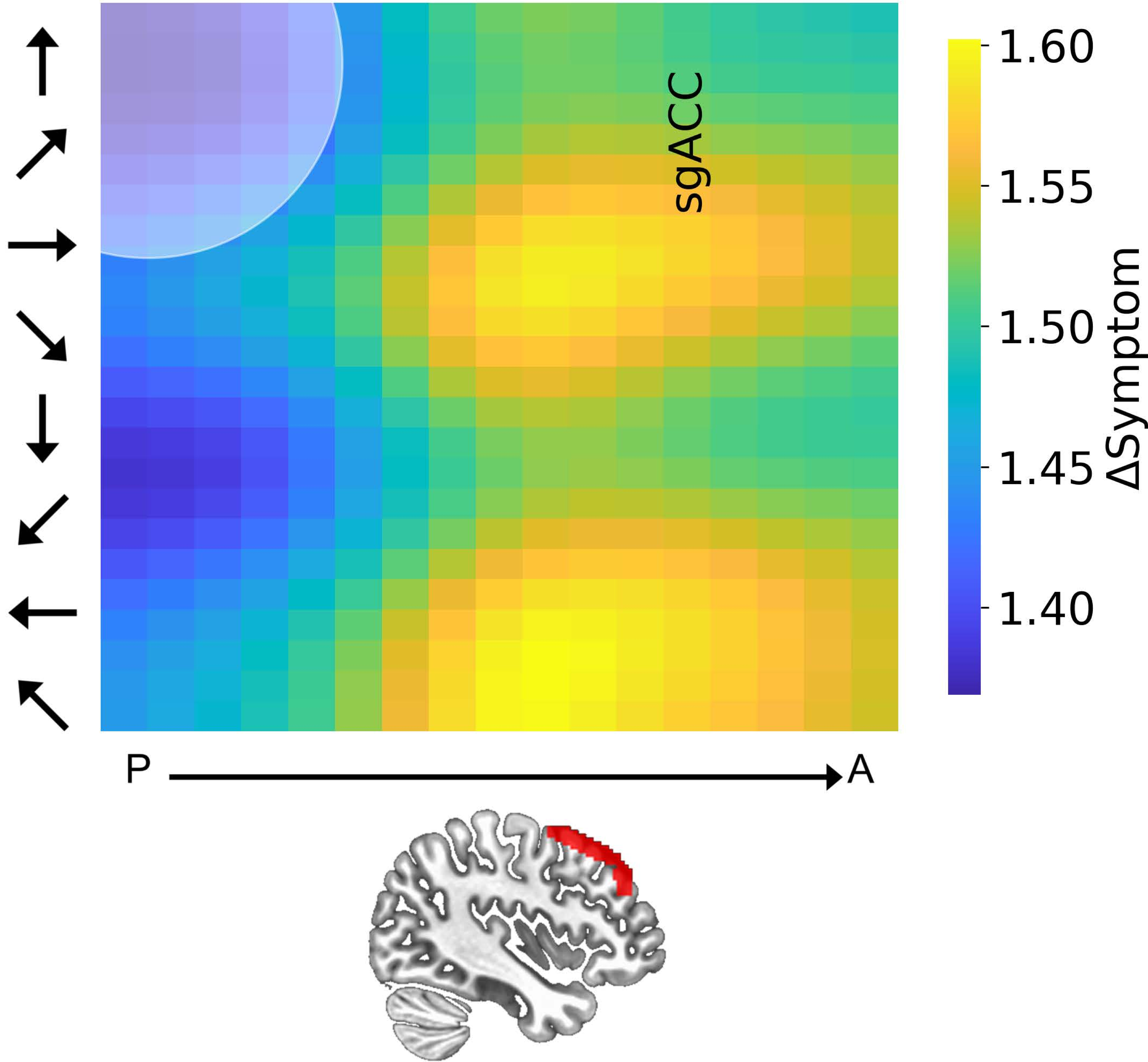

NDARCG535CPC_hamd_right_dlpfc

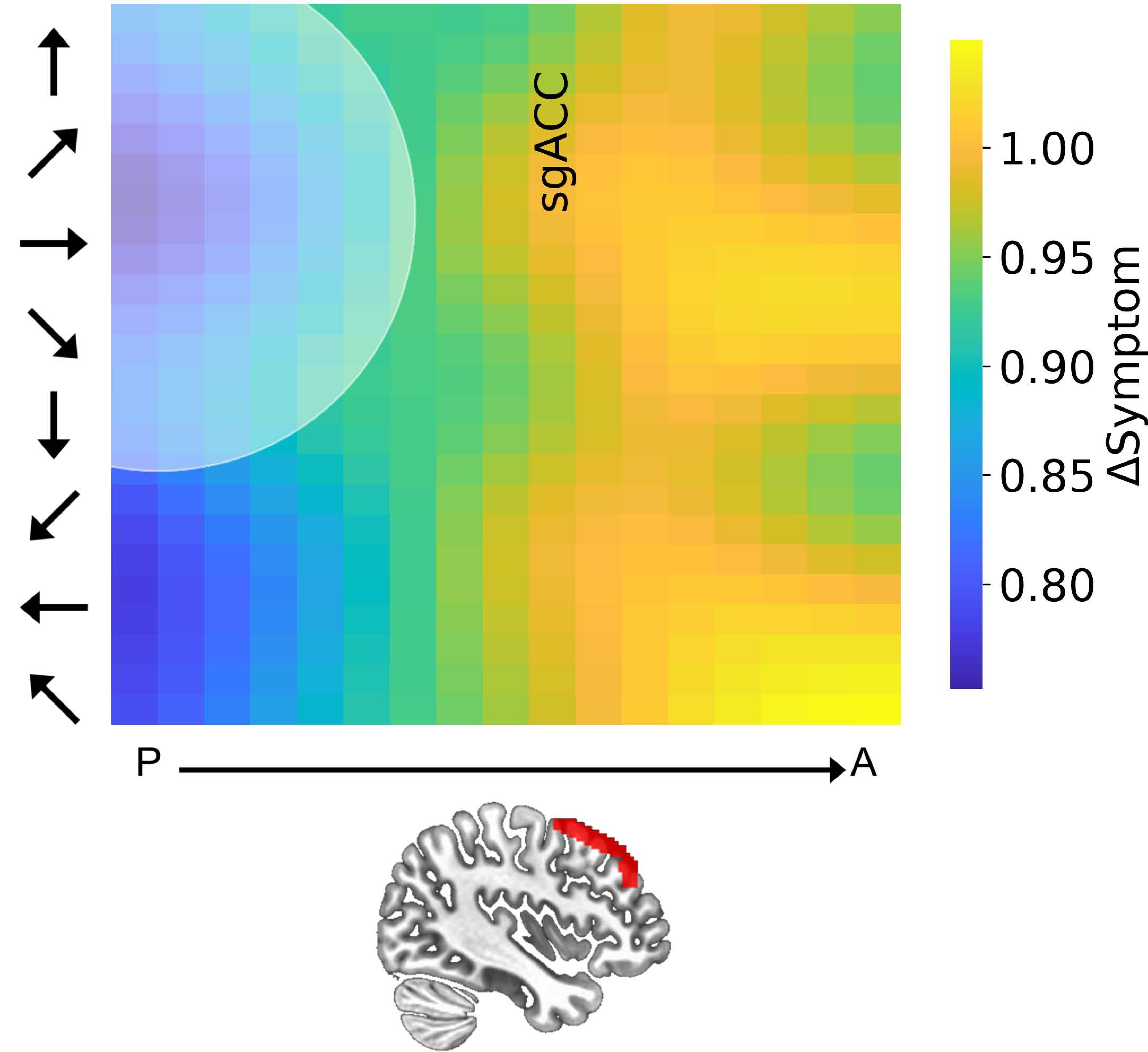

NDARCY406TU1_hamd_right_dlpfc

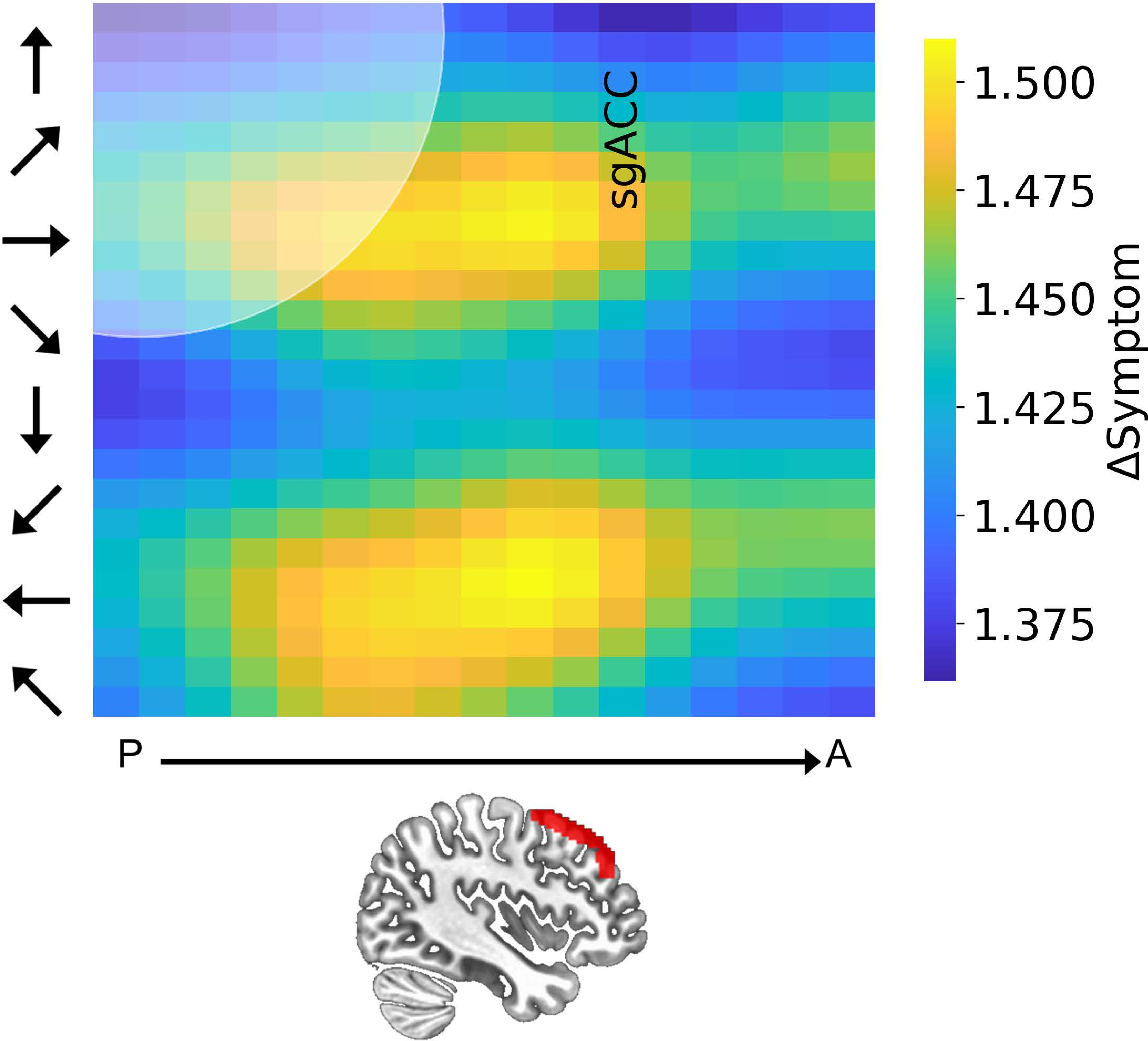

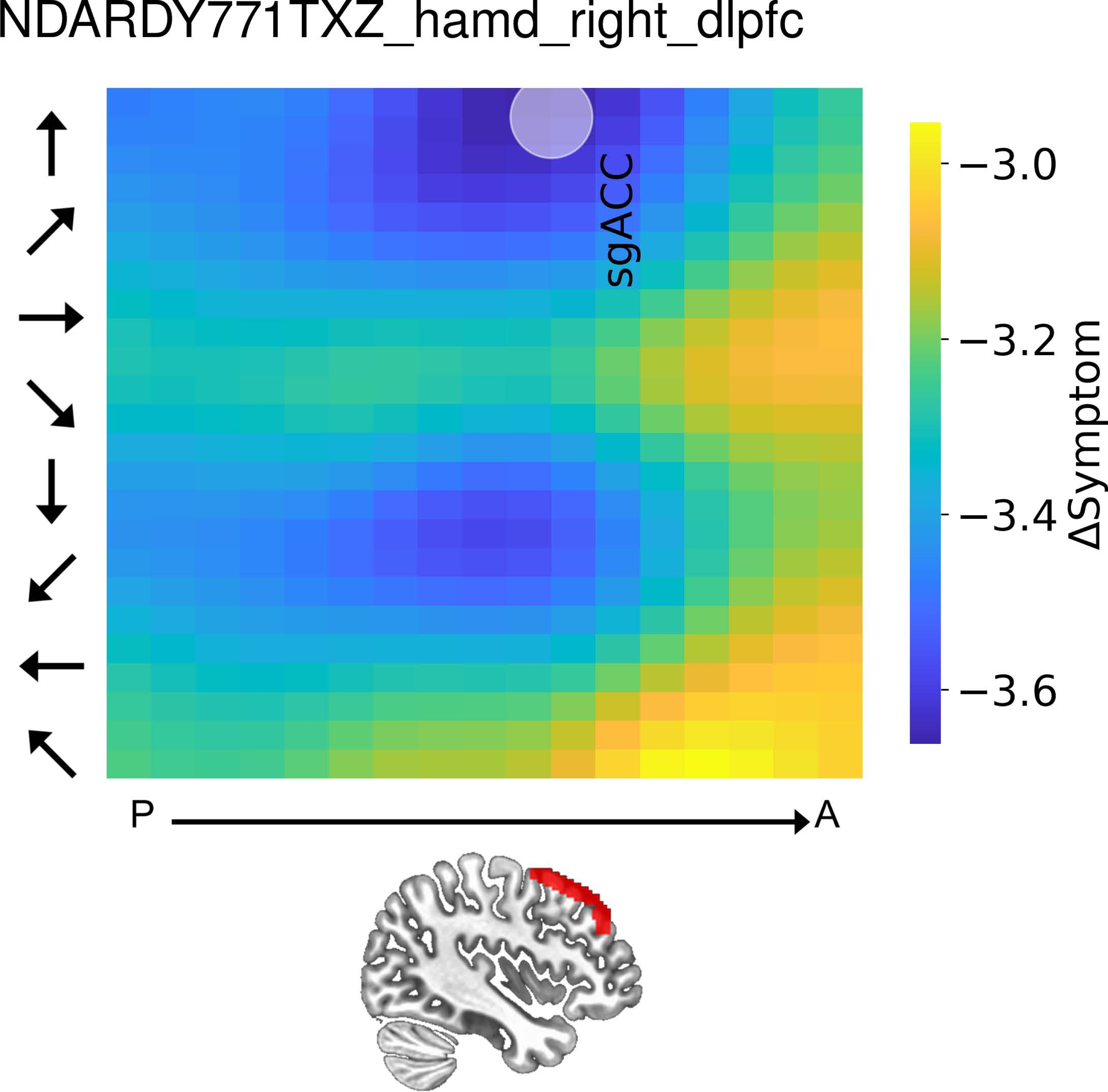

NDARDY771TXZ_hamd_right_dlpfc
sgACC
P
A
−3.0
−3.2
−3.4
−3.6
ΔSymptom

NDAREU790BVQ_hamd_right_dlpfc

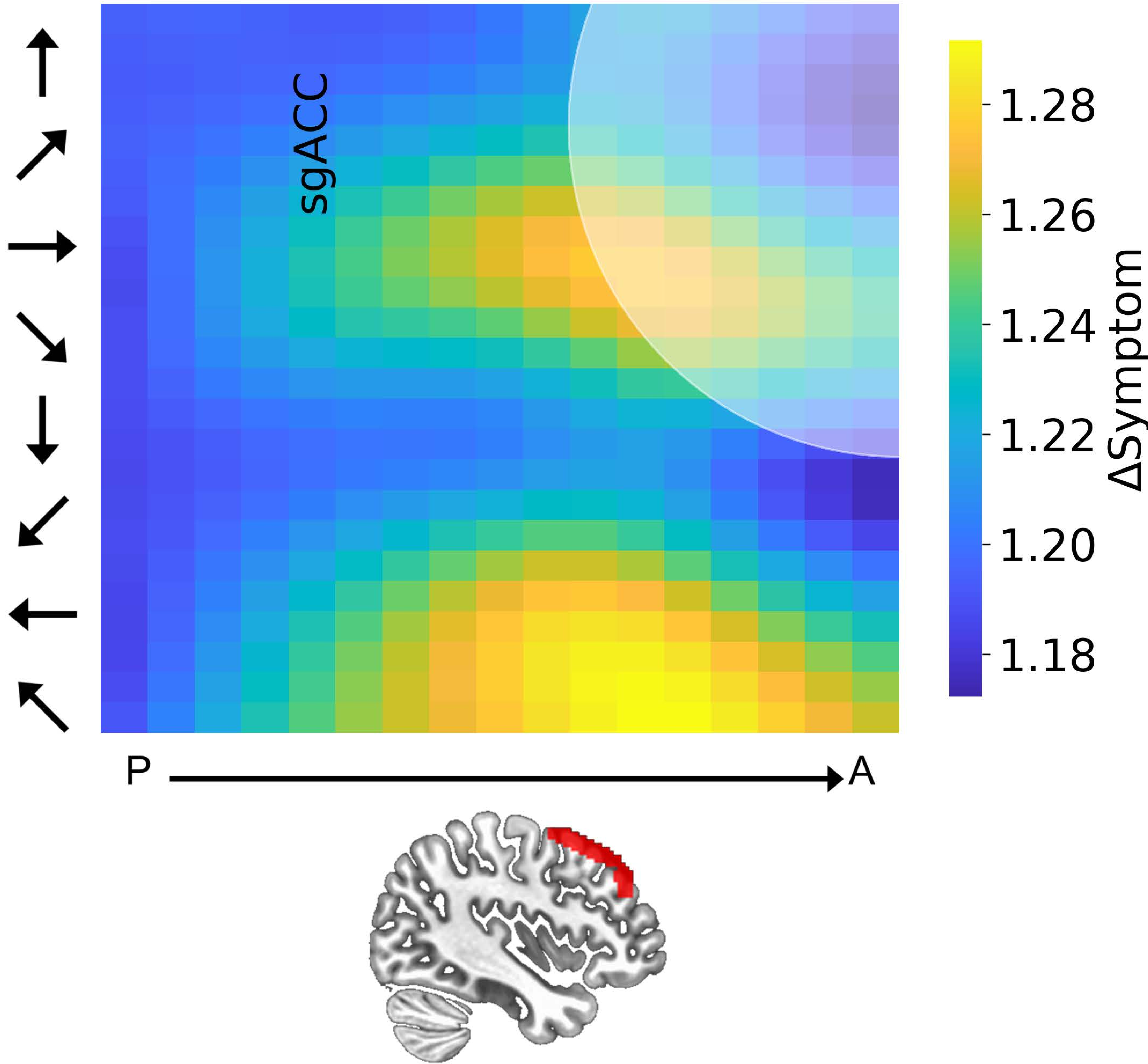

NDARGA844BF5_hamd_right_dlpfc

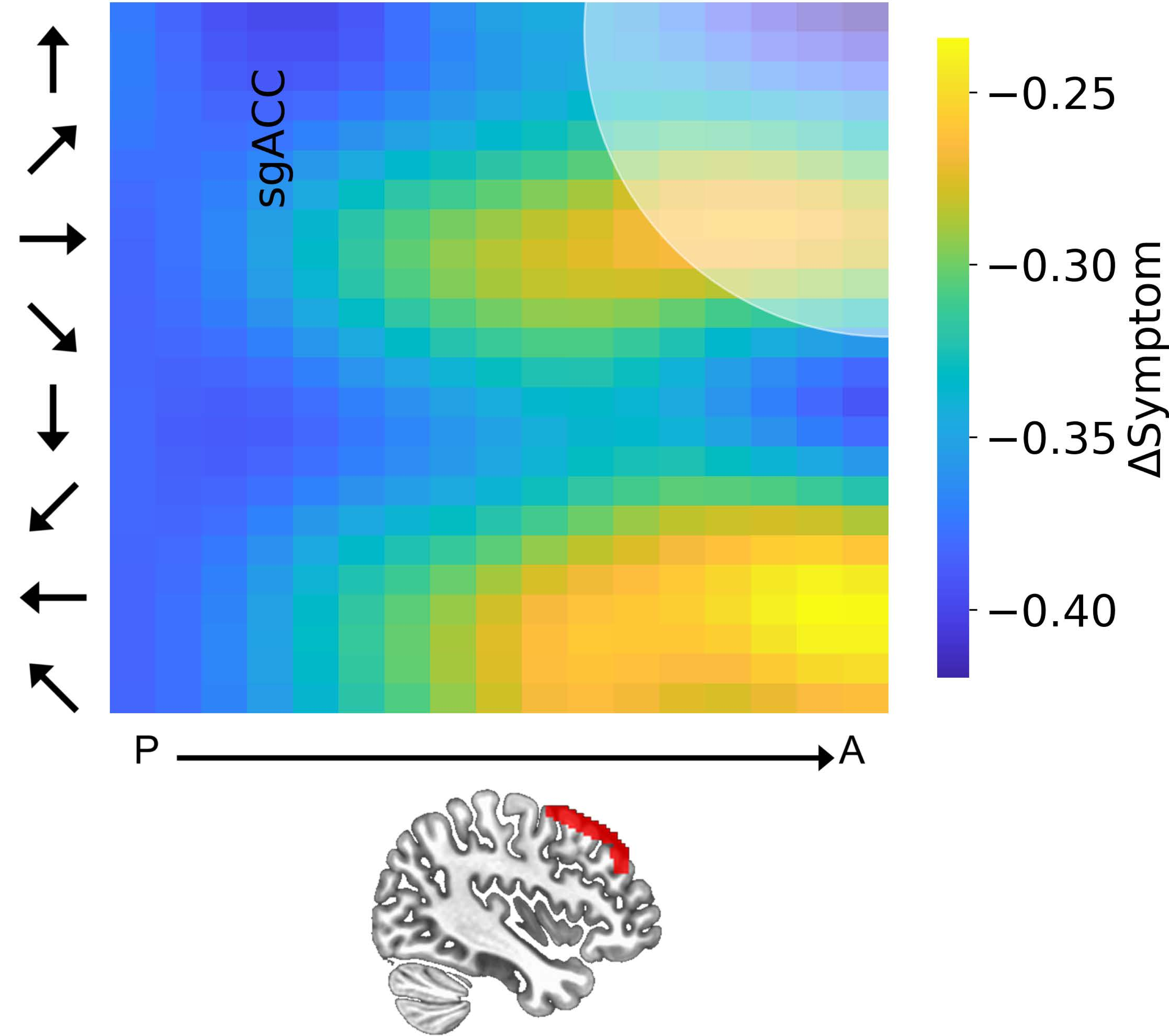

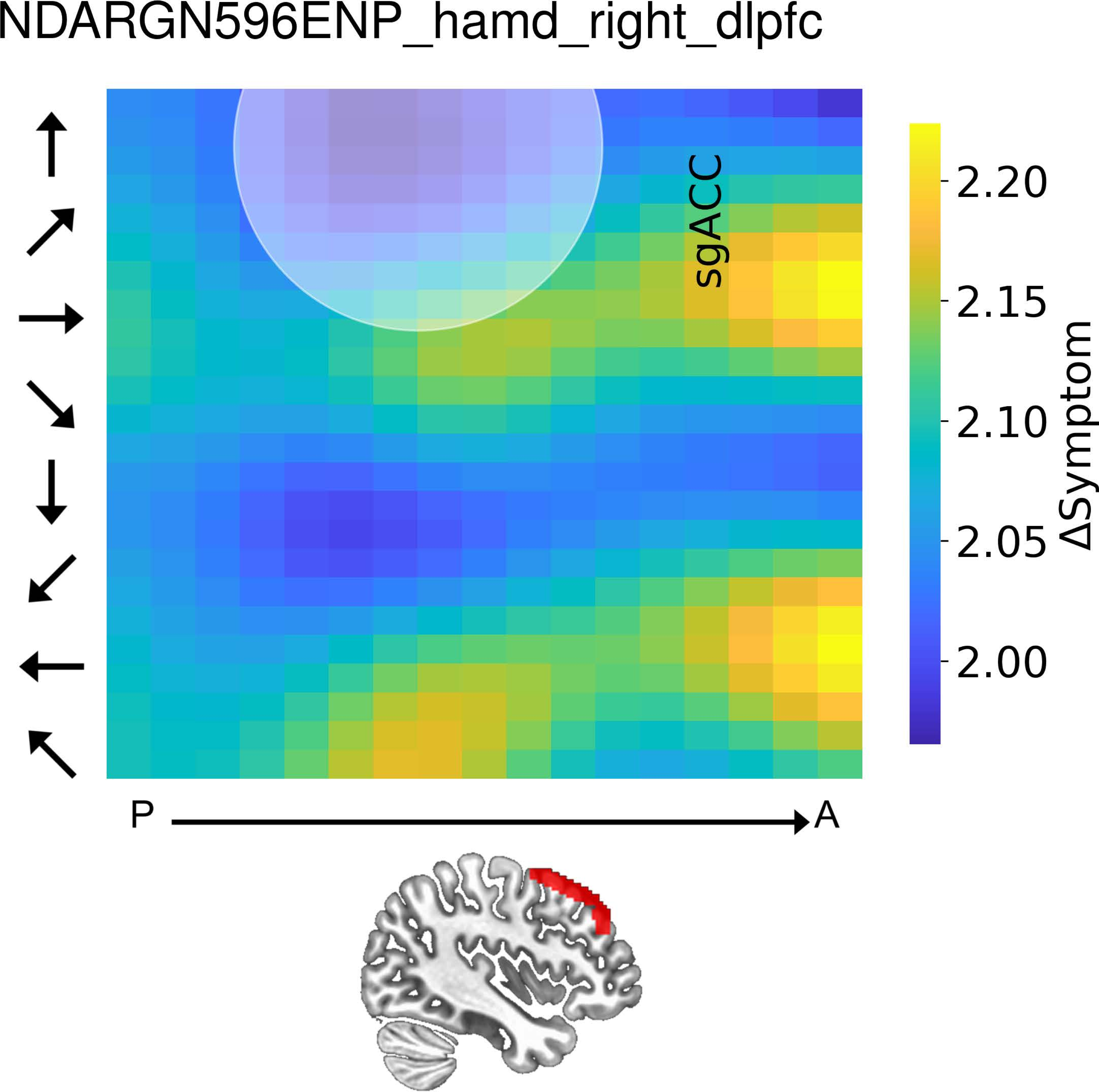
NDARGN596ENP_hamd_right_dlpfc
sgACC
2.20
2.15
2.10
2.05
2.00
ΔSymptom
P
A

NDARHU677DFF_hamd_right_dlpfc

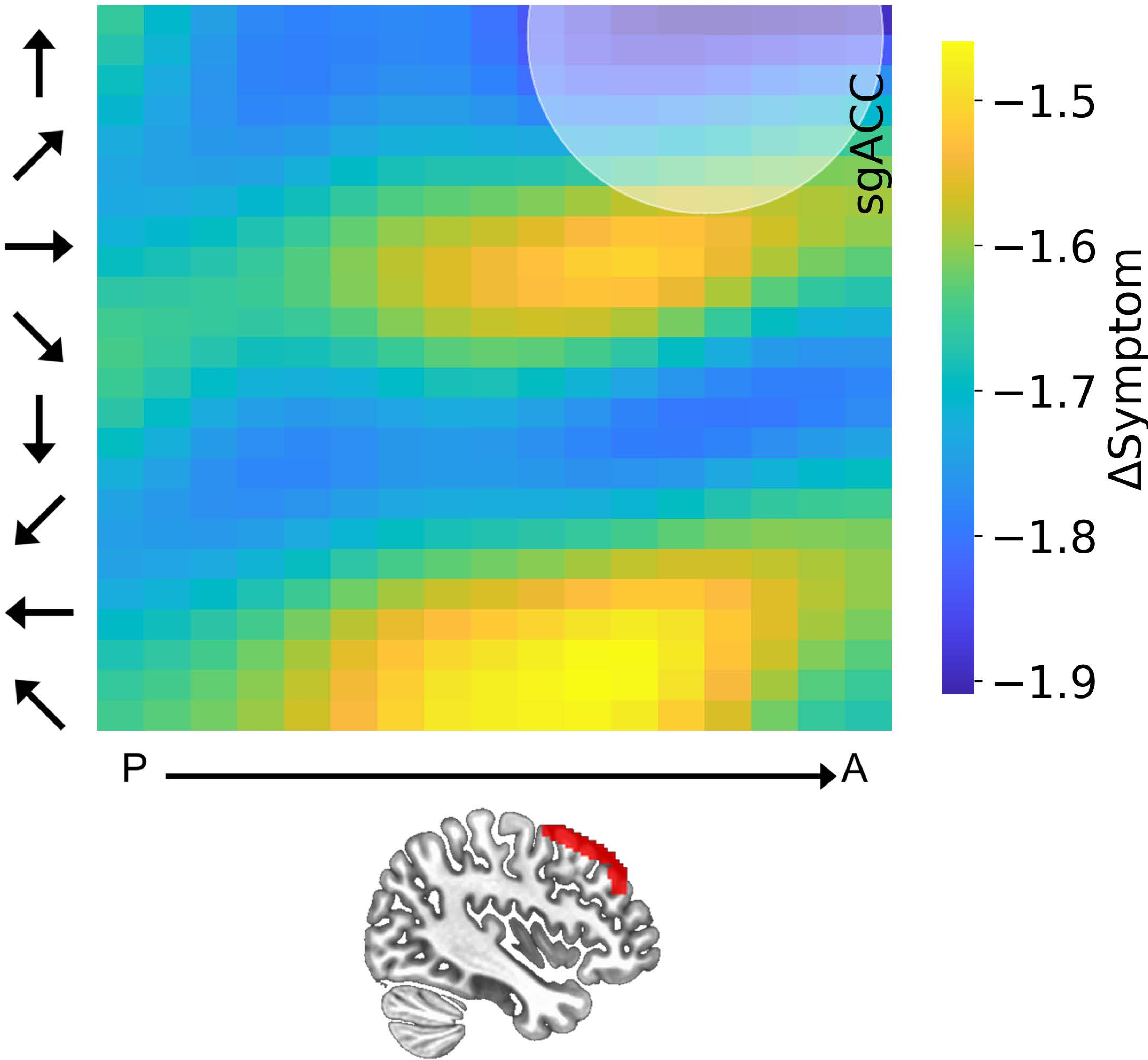

NDARJL083WMP_hamd_right_dlpfc

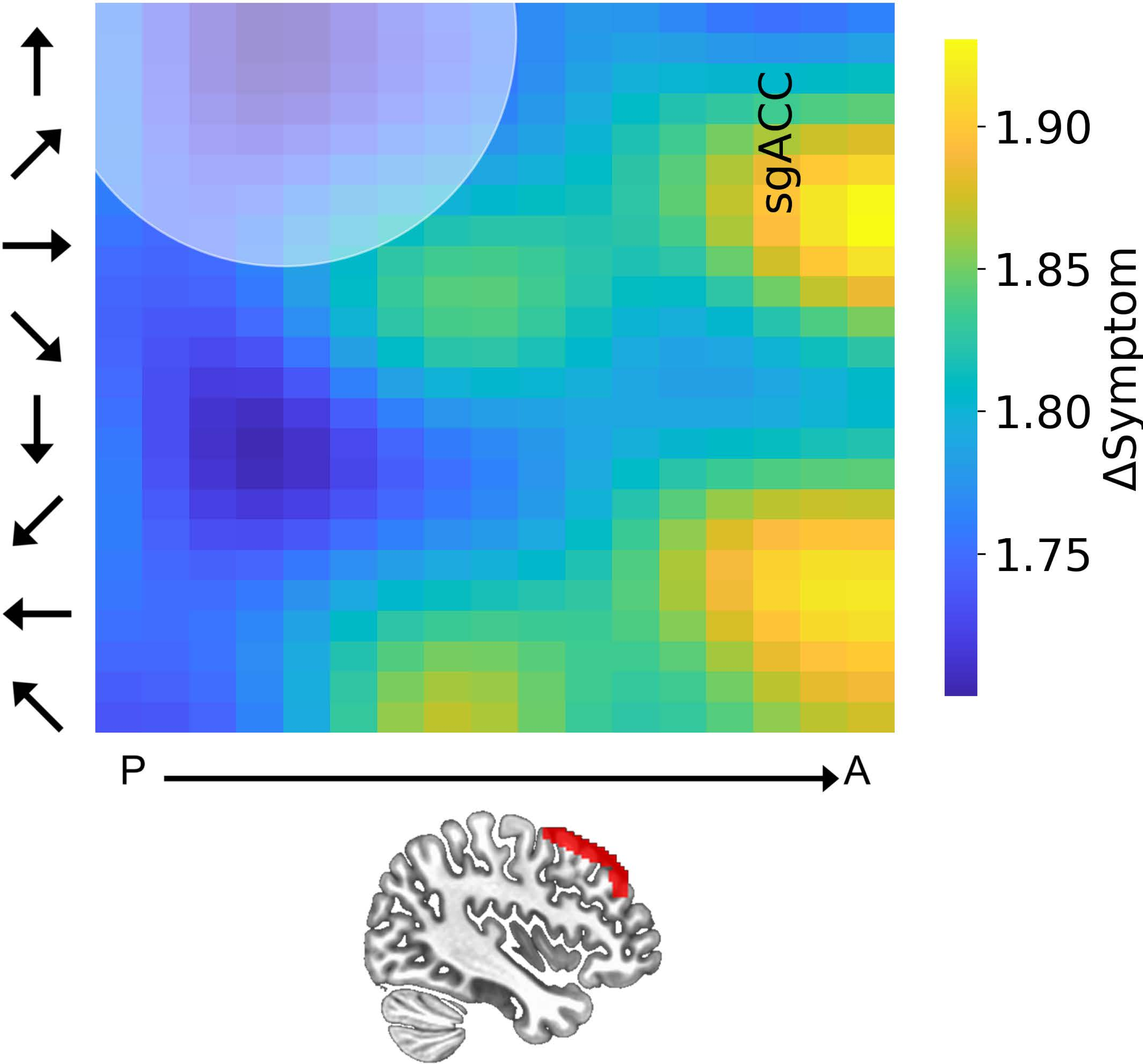

NDARKR291PWN_hamd_right_dlpfc

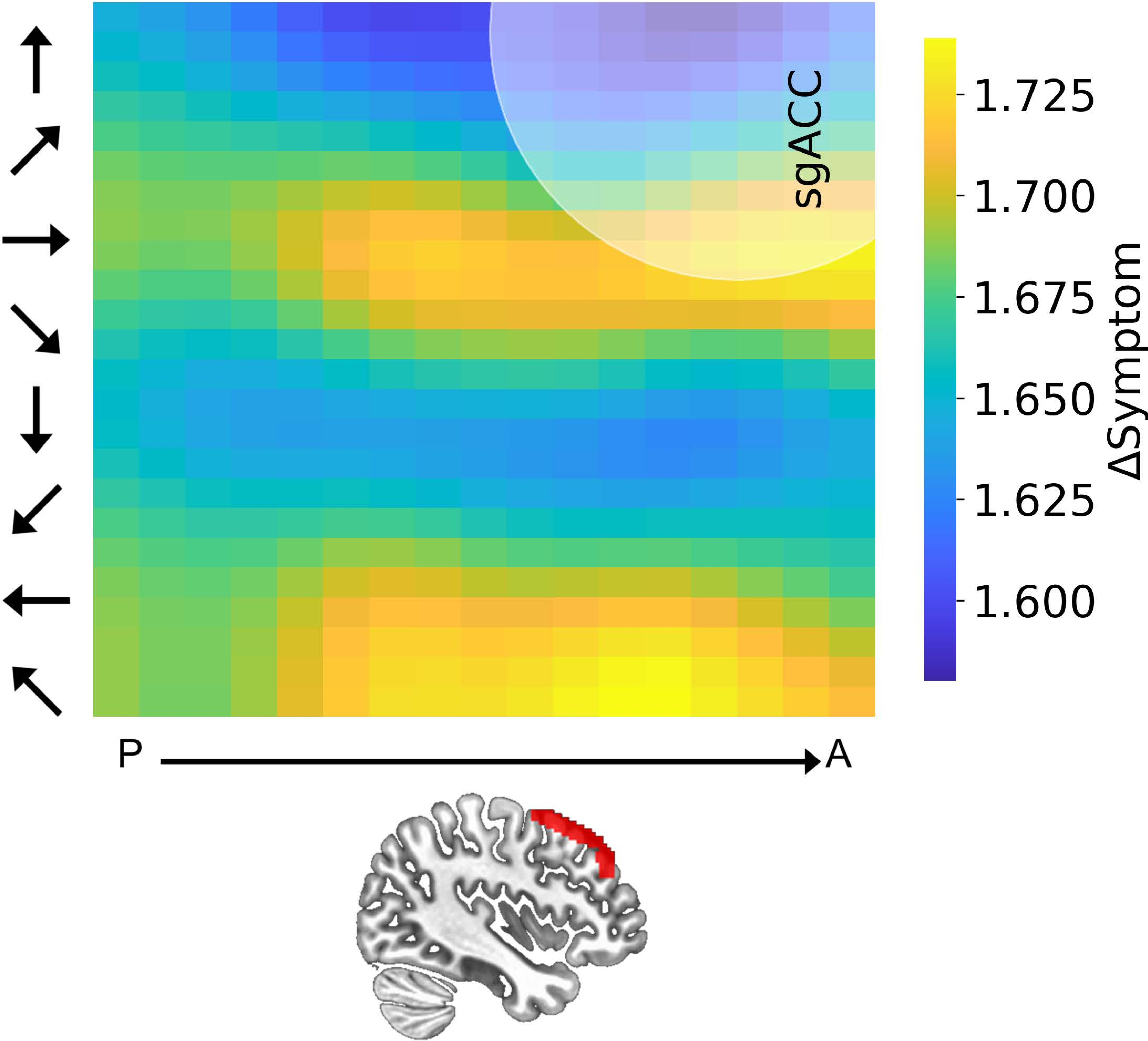

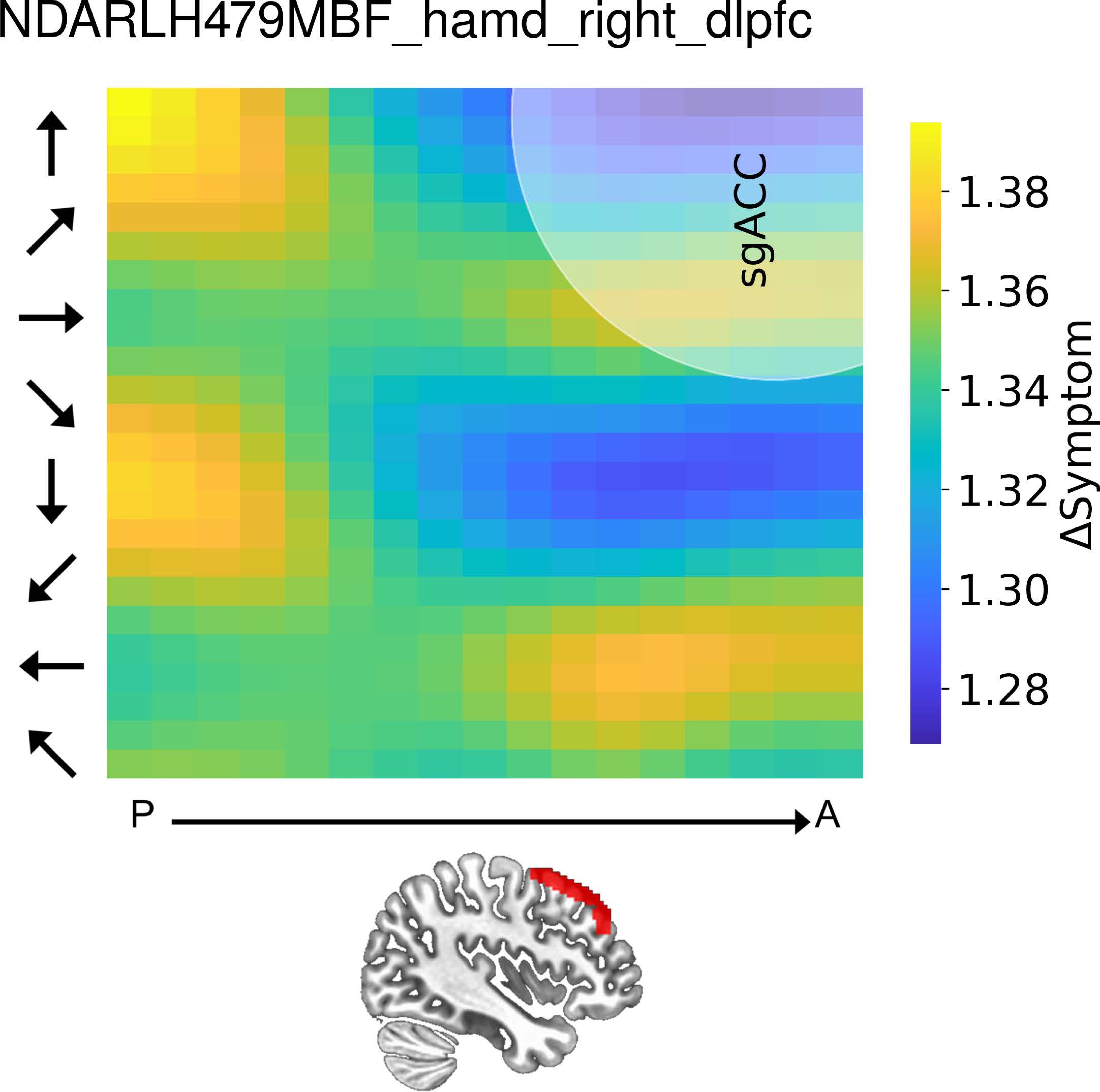
NDARLH479MBF_hamd_right_dlpfc
sgACC
1.38
1.36
1.34
1.32
1.30
1.28
ΔSymptom
P
A

NDARME497RF8_hamd_right_dlpfc

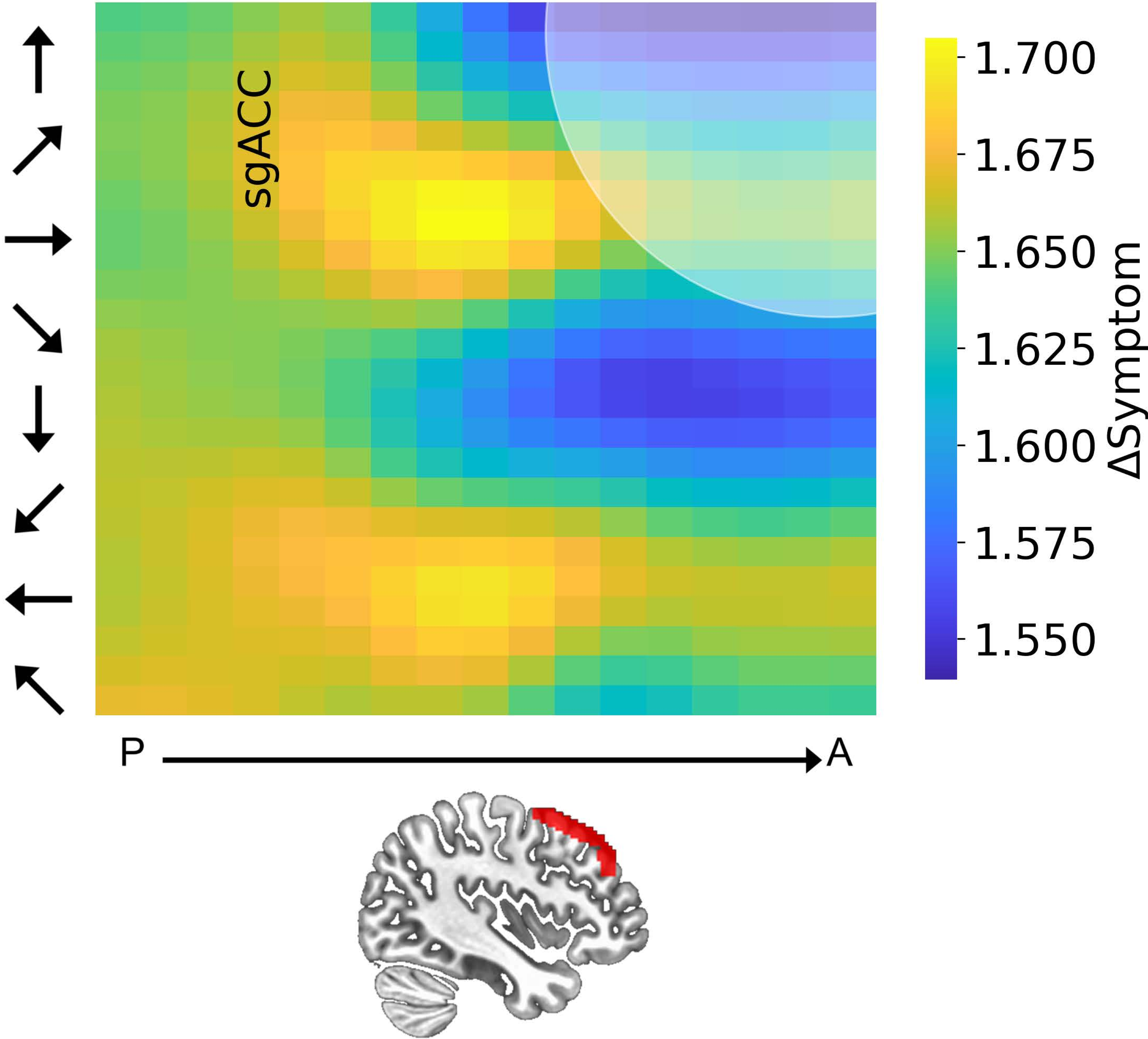

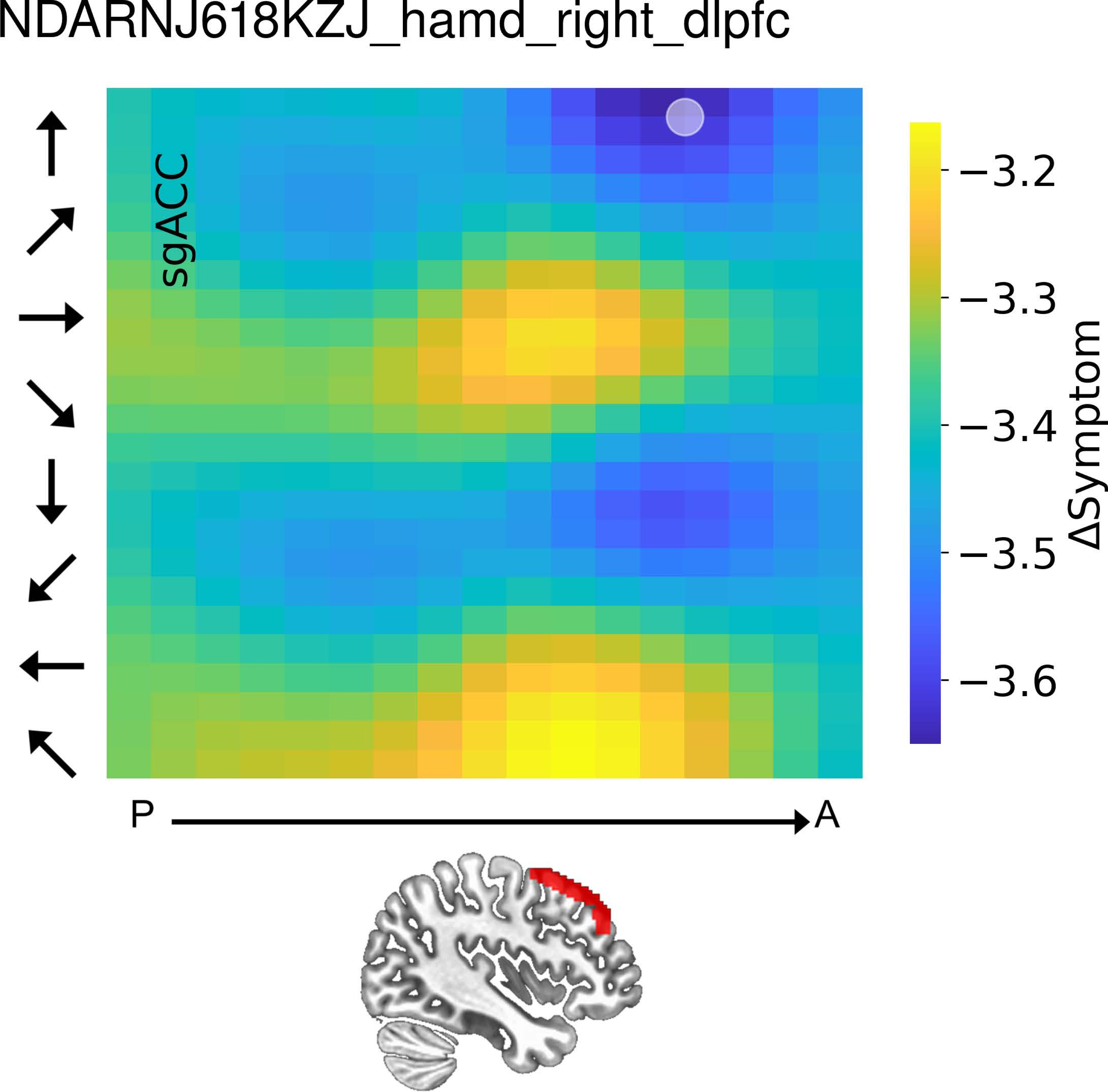

NDARNJ618KZJ_hamd_right_dlpfc
sgACC
P
A
−3.2
−3.3
−3.4
−3.5
−3.6
ΔSymptom

NDARNL715RTY_hamd_right_dlpfc

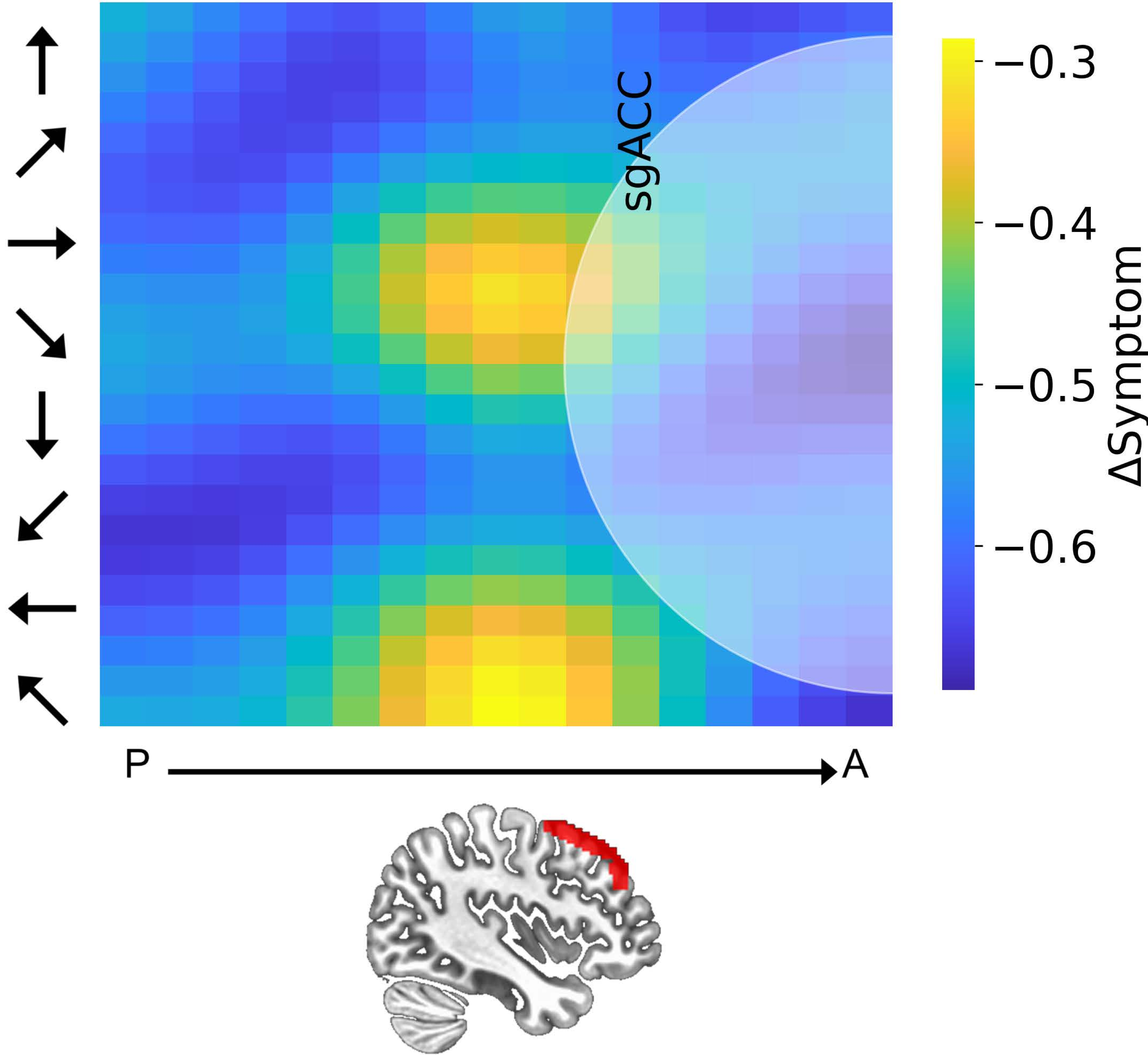

NDARPK532CVC_hamd_right_dlpfc

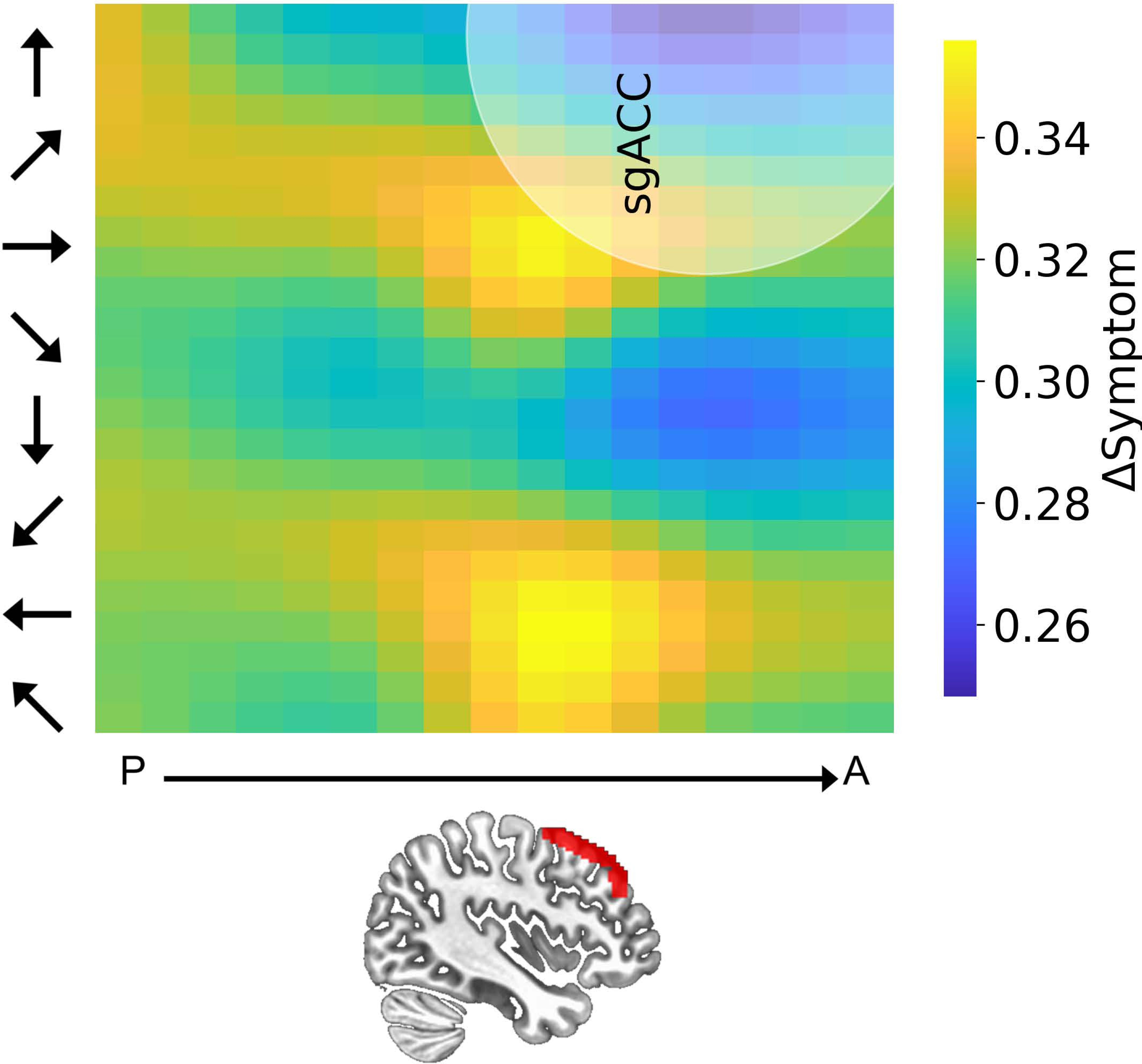

NDARWK858FA2_hamd_right_dlpfc

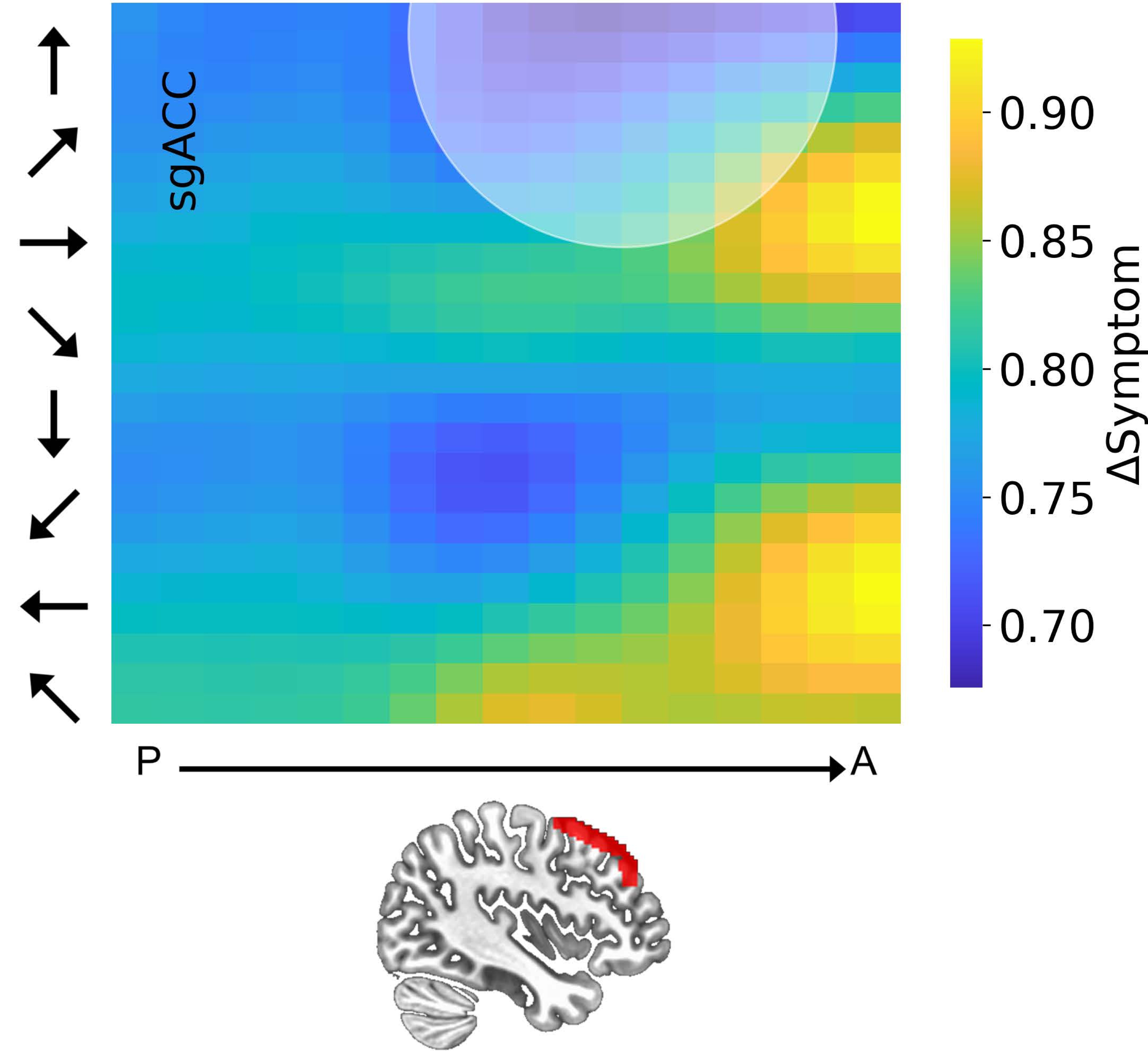

NDARWM071DRZ_hamd_right_dlpfc

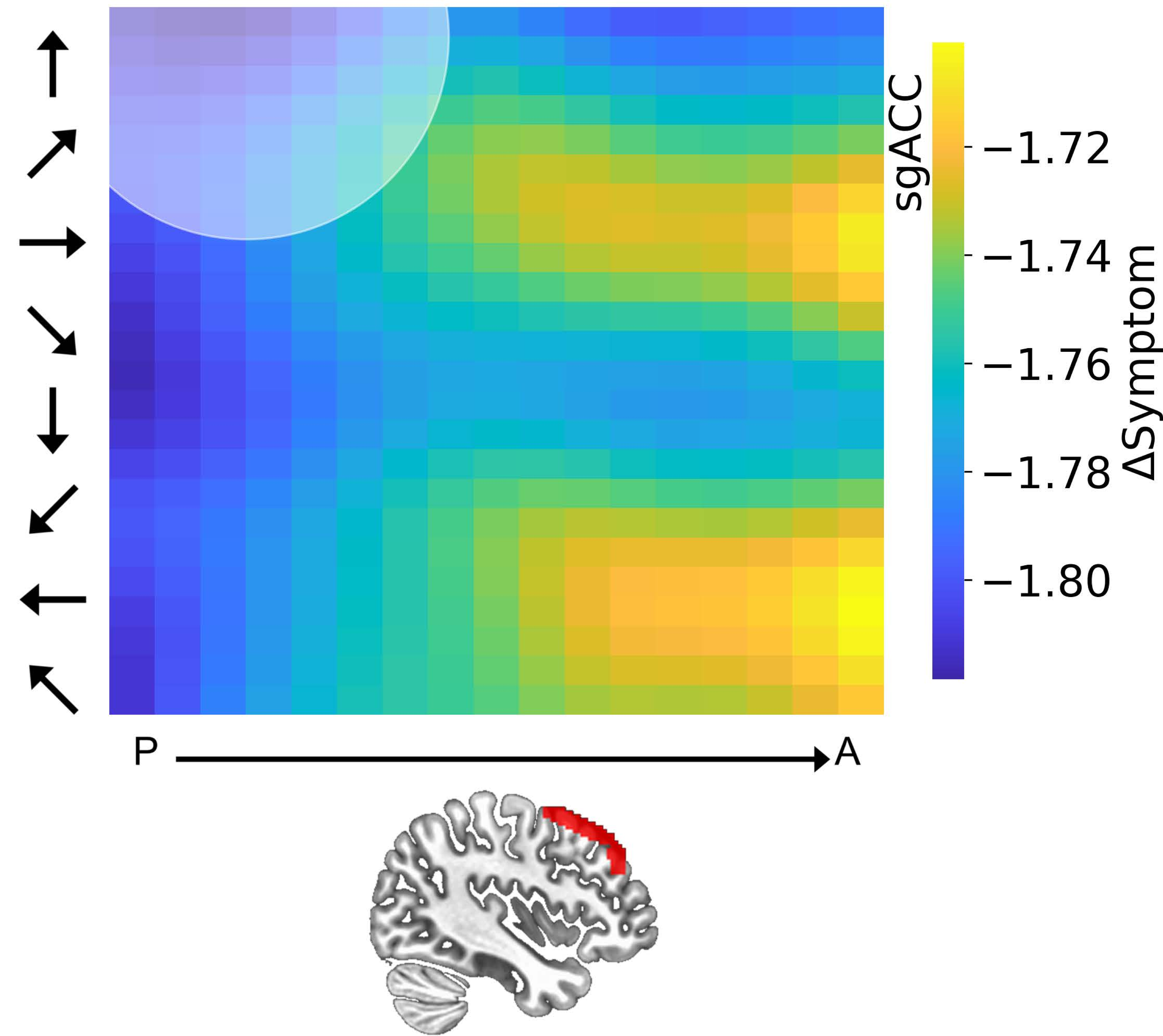

NDARWT226PU5_hamd_right_dlpfc

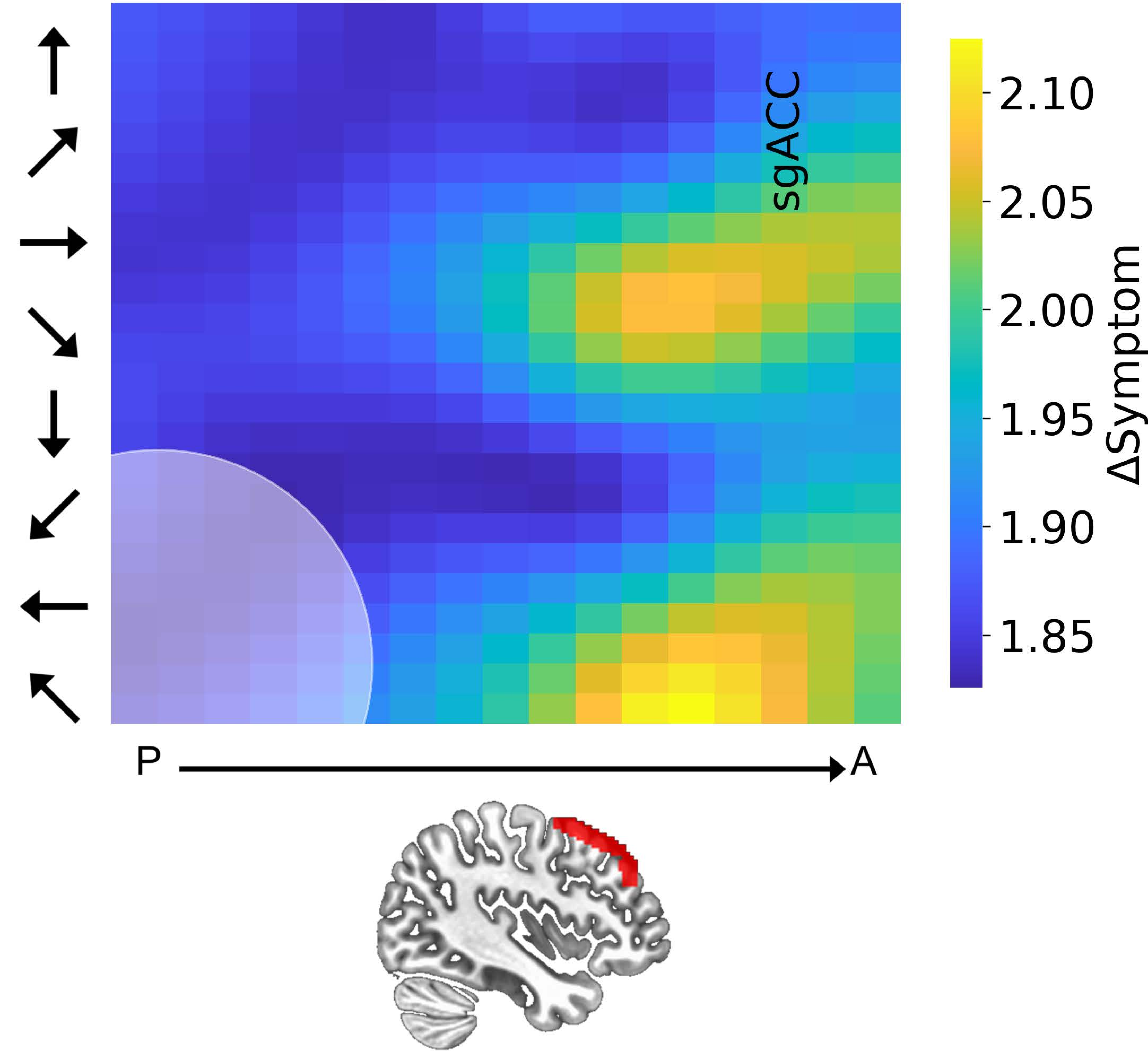

NDARWU139LAY_hamd_right_dlpfc

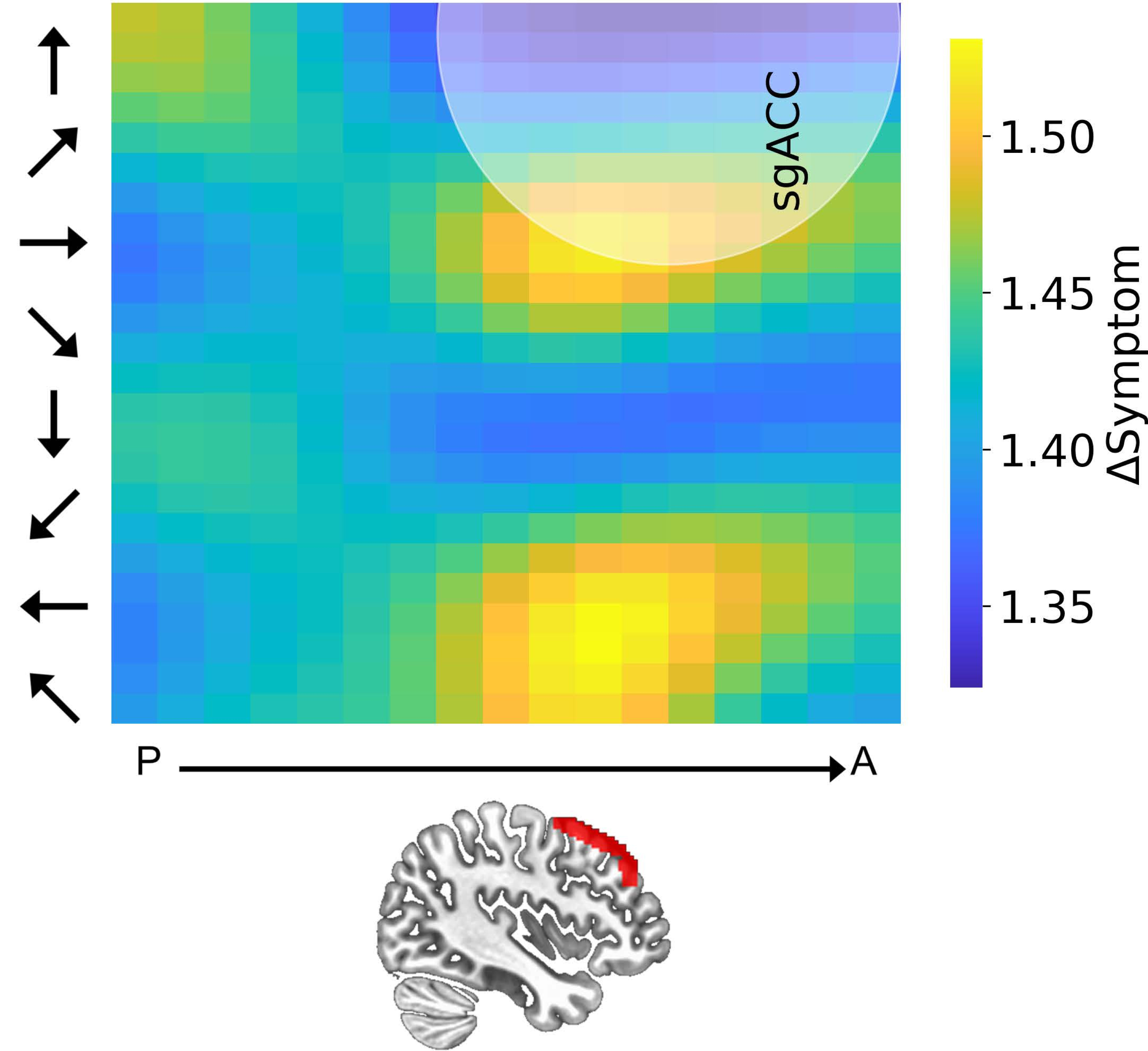

NDARXH480BBY_hamd_right_dlpfc

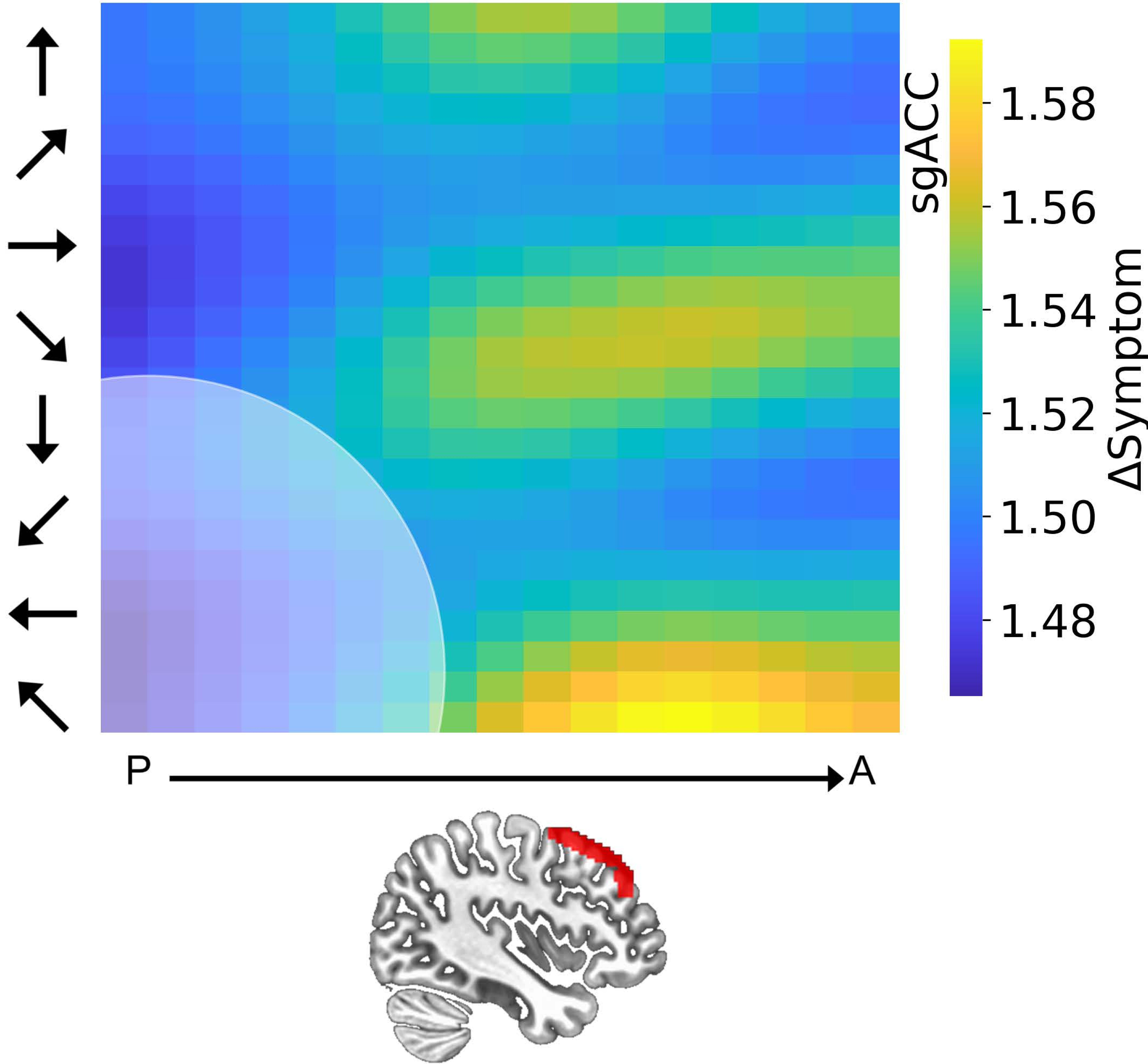

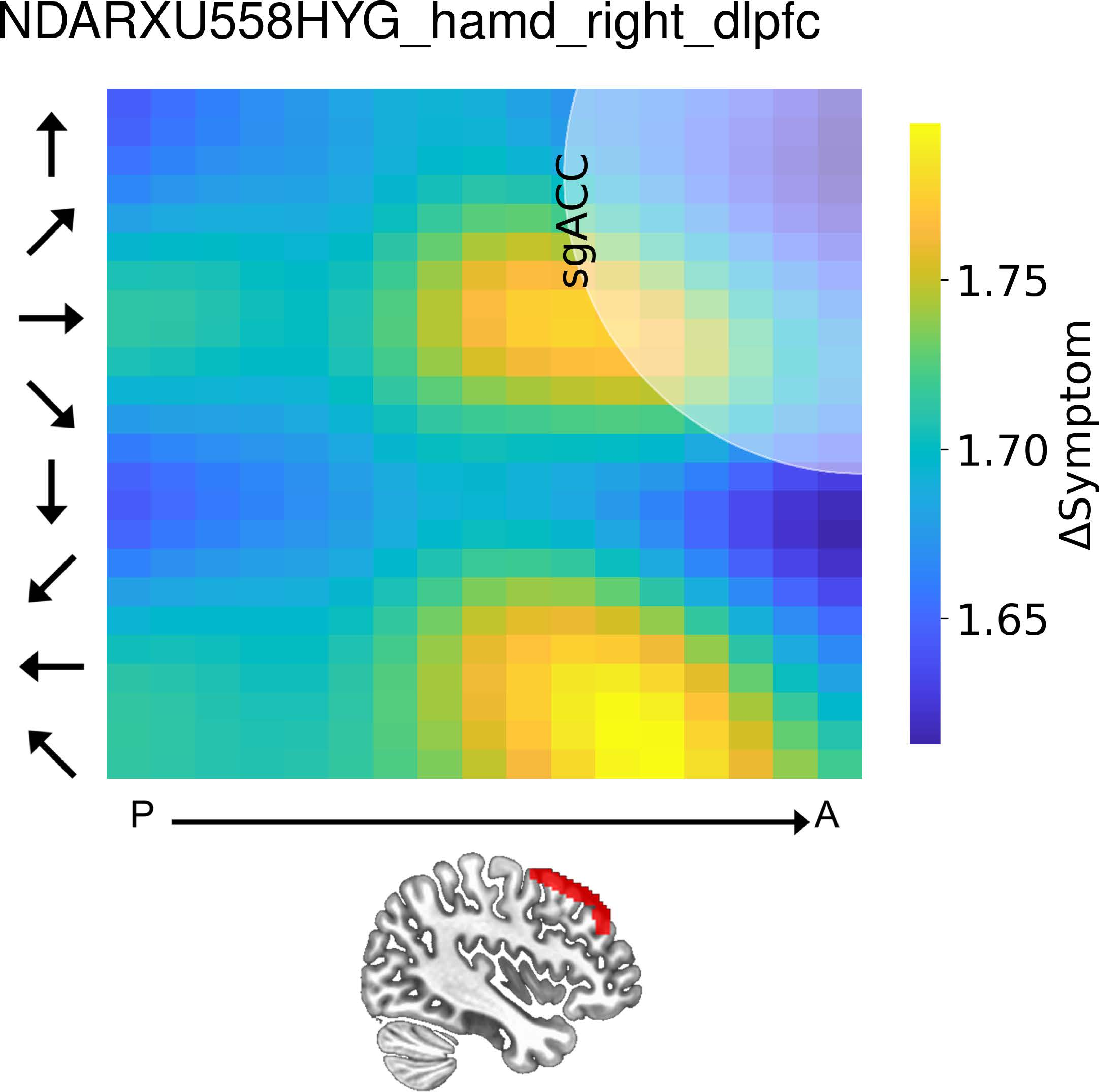
NDARXU558HYG_hamd_right_dlpfc
sgACC
1.75
1.70
1.65
ΔSymptom
P
A

NDARZW884MZC_hamd_right_dlpfc

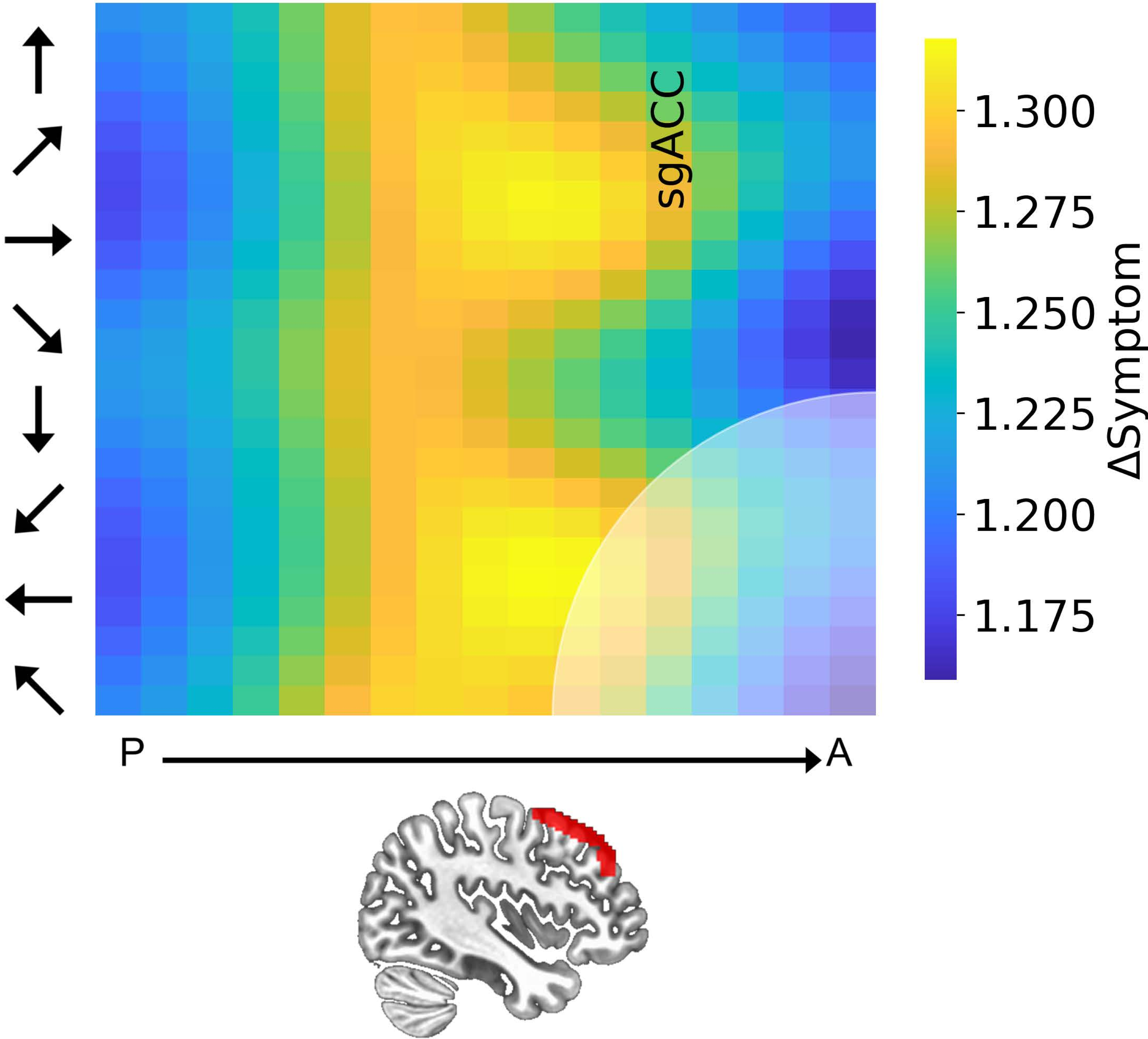

Supplement: Supplementary file 1 — supplemental material [file 41386_2021_1110_MOESM1_ESM.pdf]
